# Whole genome evaluation of horizontal transfers in the pathogenic fungus *Aspergillus fumigatus*

**DOI:** 10.1186/1471-2164-11-171

**Published:** 2010-03-12

**Authors:** Ludovic V Mallet, Jennifer Becq, Patrick Deschavanne

**Affiliations:** 1 Molécules thérapeutiques in silico (MTI), INSERM UMR-M 973, Université Paris Diderot - Paris 7, Bât Lamarck, 35 rue Hélène Brion, 75205, Paris Cedex 13, France; 2 DSIMB, INSERM UMR-S 665, Université Paris Diderot - Paris 7, INTS, 6 rue Alexandre Cabanel, 75739 Paris Cedex 15, France

## Abstract

**Background:**

Numerous cases of horizontal transfers (HTs) have been described for eukaryote genomes, but in contrast to prokaryote genomes, no whole genome evaluation of HTs has been carried out. This is mainly due to a lack of parametric methods specially designed to take the intrinsic heterogeneity of eukaryote genomes into account. We applied a simple and tested method based on local variations of genomic signatures to analyze the genome of the pathogenic fungus *Aspergillus fumigatus*.

**Results:**

We detected 189 atypical regions containing 214 genes, accounting for about 1 Mb of DNA sequences. However, the fraction of atypical DNA detected was smaller than the average amount detected in the same conditions in prokaryote genomes (3.1% vs 5.6%). It appeared that about one third of these regions contained no annotated genes, a proportion far greater than in prokaryote genomes. When analyzing the origin of these HTs by comparing their signatures to a home made database of species signatures, 3 groups of donor species emerged: bacteria (40%), fungi (25%), and viruses (22%). It is to be noticed that though inter-domain exchanges are confirmed, we only put in evidence very few exchanges between eukaryotic kingdoms.

**Conclusions:**

In conclusion, we demonstrated that HTs are not negligible in eukaryote genomes, bearing in mind that in our stringent conditions this amount is a floor value, though of a lesser extent than in prokaryote genomes. The biological mechanisms underlying those transfers remain to be elucidated as well as the biological functions of the transferred genes.

## Background

### Horizontal transfers in eukaryotes

Horizontal transfers (HTs) are a major force of evolution in prokaryotes [1-5]. The average amount of DNA transferred in prokaryote genomes varies from 0 to 17% according to different studies [4,6-8]. The transferred genes remaining in the genome either increase fitness or allow the colonization of new environments [2,3,9-11]. However, the extent of HT in eukaryotes is less known though they were proposed to play a role as important as for prokaryotes [12-19]. In fact, most of the documented cases concern insertions of viruses (especially retroviruses) into eukaryote genomes [20-23] and exchanges between symbiont, parasite [18,24] or organelle genomes [25,26] and their host genome. At last, as conjugation between distant species is unlikely by meiosis, a possibility of transfer between eukaryotes was evoked by gene introgression following hybridization between closely related species [27].

For the former examples, the biological mechanisms are understood, demonstrated or are hypotheses with strong support. However the mechanisms involved in DNA exchanges between distant species are mostly unknown, either between eukaryotes or to explain the numerous reports of HTs between prokaryotes and eukaryotes. Among the mechanisms acting in prokaryotes, transformation by free DNA is possible for eukaryotes but is less efficient than it is for prokaryotes [28]. Transduction was hypothesized but its efficiency differs as a function of species families from possible to unlikely by lack of vectors [29]. Also, alternative mechanisms were suggested, like phagocytosis or by means of bacterial type IV secretion systems that could promote the transfer of DNA from prokaryotes to eukaryotes [13,30]. Thus, while HT results are observed, the underlying mechanisms are yet to be discovered.

### Choice of a HT detecting method for *A. fumigatus*

The HT detection methods generally used in eukaryotes are based on gene homology. The determination method depends on the number of homologs of the target and of its phylogenetic distribution. In the case of the detection by Blast of only a few homologs for a gene of interest, an alignment analysis showing more homology with genes/proteins of distant species than to a closer one indicates a horizontal transfer event for this gene. A typical example of such detection is to find a close prokaryotic homolog to a eukaryotic gene [31-34]. A more reliable method can be used in the case of numerous homologs and their broad distribution in the evolution tree. In this latter case, a phylogenetic analysis is performed and incongruence in the phylogenetic tree leads to a similar conclusion [35,36]. In each of these cases, the study concerns only a peculiar gene or a small group of genes [37-42]. Indeed, due to the restrictions exposed above, a fair number of genes cannot be analyzed this way: ORFans of course but also genes with only one or a small number of homologs leading to an inconclusive situation. Moreover, due to the patchiness of eukaryote sequences in Genbank, it is difficult to assess horizontal transfer between eukaryotes species [43], while it is easier to assess transfers between prokaryote and eukaryote species [14]. However, many newly sequenced genomes were analyzed for horizontally transferred genes (HGT) and in some cases a large number of HGTs were detected by phylogenetic analyzes (for instance 587 genes - 5.6% of all genes - were found of bacterial origin in the diatom *P. tricornutum *[44]). Therefore, while a high number of genes could be found of alien origin, these studies, as discussed above, could not be qualified as whole genome studies.

In order to analyze whole genomes and to cope with the difficulties discussed previously, the so-called parametric methods were designed. They are based either on the whole set of genes of a species or on variations of the composition characteristics of the genomic sequence itself. Methods using gene information are based on differences in codon usage between highly expressed, lowly expressed and alien genes [45-48]. However, none of the methods based on codon usage can be applied to eukaryote genomes as gene regulation is different from prokaryotes and no tool has been designed to cope with this fact [47,48].

The other methods are based on the variations of base composition detected by different order Markov models along a genome: the so-called genomic signature [49,50]. This genomic signature was demonstrated to be species-specific and quite similar all along the genome [50-54]. This species-specificity was used to detect horizontally transferred DNA by analyzing a genome and searching for regions exhibiting a different signature than the majority of the genome [4,6-8,55-65]. These methods use only the information contained in the analyzed genome and when applied to the whole genome sequence they allow the detection of atypical regions containing no annotated genes [6,61].

Phylogenetic and parametric methods, while detecting common genes, diverge in certain cases. It was proposed that these two types of methods addressed different types of HGTs [66,67]. It was proposed that combining signature and gene based methods increased either specificity or sensitivity of HT detection [33,58,68].

In general, when compared to prokaryotic genomes, eukaryote genomes are larger and more complex due to the presence of non-coding sequences, low complexity regions, isochores and fragmented genes. Therefore, most of the parametric methods used for prokaryotes are either inefficient or not suitable to eukaryotic genomes. Likewise, methods based on variations of the G+C composition work poorly due to the intrinsic variations of base composition in eukaryote genomes [69]. For these reasons, no genome-wide study of horizontal transfers in an eukaryotic genome using parametric methods was published. However, some eukaryotic genomes present characteristics close to prokaryotic ones and allow attempting the use of parametric methods on them. For instance, it has been shown that variation of short oligonucleotide usage is moderate in some fungi genomes and that parametric methods based on this type of criterion could be applied to them [50,70,71]. Moreover, HTs seem to play an important role in the evolution of fungi [29,72-75]. Therefore, we chose to analyze the extent of horizontal transfers in the genome of *Aspergillus fumigatus *[76-78]. *A. fumigatus *is a pathogenic fungus causing a wide range of diseases including mycotoxicosis, systemic diseases and allergic reactions. The mortality rate is high in infected patients, especially in immuno-compromised ones. Here we propose to use a simple and tested method based on short oligonucleotide usage [6] to evaluate the amount of HTs in the genome of *Aspergillus fumigatus*.

We found that HTs in fungi are not negligible, accounting for 1 Mb, representing about 3% of the genome and that donor species belong mainly to 3 classes, bacteria, fungi and viruses.

## Methods

### Genome

The *Aspergillus fumigatus *Af293 genome (Genbank NC_007194 - NC_007201) [78] has a size of 29.4 Mb and is composed of 8 chromosomes. Its base composition is balanced: G+C% = 49.8%.

### HT detection method

We used a method based on the variations of tetranucleotide frequencies along a sequence. The method was already described and tested on prokaryotic genomes and the principles are recalled hereafter [6]. The specificities of eukaryotic genomes implied a pretreatment and in a first step, we removed from the genome all the centromeric and telomeric low complexity regions which exhibited an atypical signature and did not correspond to transferred DNA. The genome was subsequently analyzed by a 5 kb sliding window, with a step of 500 bp. The signature of each window was calculated and the Euclidian distance of every window signature to the whole genome signature was assessed. Then, the window signatures were clustered by a k-means algorithm and a partition based on the distance distributions per class and the average distances of the classes to the genome was performed. In a previous work with prokaryotic genomes, less than 8 classes (average 4 [6]) were required to take into account the intrinsic genome variation and the atypical signatures. Due to an increased intrinsic variation of base composition in eukaryote genomes, the number of classes was raised to 20 for the *A. fumigatus *genome. The partition separated the k-means classes into one group exhibiting rather homogeneous signatures whose distance to the whole genome signature was small (90% of the windows) and one group of heterogeneous classes with a large distance to the genome signature (10% of windows). Thus, we considered that the first group of classes represents the host genome and calculated the average signature of this host genome. The Euclidian distances of all the window signatures were recalculated with regards to this new host signature. Afterwards, taking into account only the windows of this putative host genome, we established a threshold equal to the 99% percentile of the Euclidian distance to the host genome. All the windows whose signature exhibited a distance above this threshold were considered atypical and potentially corresponding to foreign DNA. We chose a high threshold in order to favor specificity rather than sensitivity.

### Atypical region analysis

All genes included in the atypical regions were analyzed: we investigated their functions and compared them to Genbank by BlastP (E-value ≤ 10^-10 ^and coverage ≥ 80%) in order to identify the closest homologous sequences if any. For atypical regions containing no annotated coding sequences, a BlastX analysis (E-value ≤ 10^-1^) was done in order to identify remnants of coding sequences and a BlastN (E-value ≤ 10^-1^) to find homology at the DNA level.

### Phylogenetic analysis

Protein sequences from the Blast analysis were aligned by ClustalW [79]. The trees (neighbor joining algorithm [80]) were bootstrapped (1000 trees) and the consensus trees calculated with the Philip package [81]. Species trees were inferred by retaining only one homolog per species (the best strain or the best homolog, the less significant paralogs were discarded).

### Donor species

We have derived and updated Genstyle, a database of species signatures [82]. Our database contains about 65,000 signatures of species strains, organelles, viruses and plasmids. It was composed as following: for each entry, all non redundant sequences longer or equal to 1 kb were gathered from Genbank then concatenated for signature calculation. We calculated the signature of each atypical region and searched the database for the closest signatures in terms of Euclidian distance. As genomic signatures are species-specific [6,50,52-54,83-85], the species with the closest signatures could be considered as potential donors of the atypical regions only if the distances obtained were below the average threshold used for HT detection (241 AU) [6].

## Results

### Atypical regions

**Figure 1:**
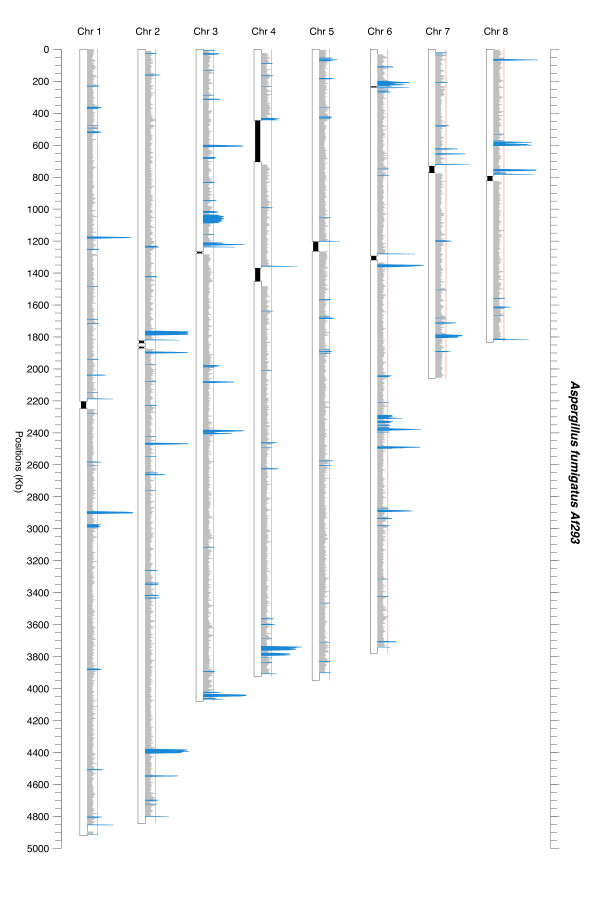
**Representation of the distance of each 5 kb window to the host genome for the 8 chromosomes of *A. fumigatus***. The red line indicates the threshold, all the windows above this line are considered as atypical. Black boxes represent the non-sequenced parts of the chromosomes [78].

In a first step, we checked that as already shown for other eukaryotes [71] all chromosomes of *A. fumigatus *presented a similar signature and intrinsic variability. The concatenated sequence of the 8 chromosomes was then used to establish the threshold. The study of the signature variations along the genome allowed for the distinguishing of 189 distinct atypical regions (Figure 1, Additional file 1). They represented 3.1% of the total genome (908 kb, Table 1). The average size of the atypical regions was 4.5 kb, ranging from 500 bp to 52.5 kb. In general, the atypical regions were spread along all chromosomes indicating no chromosome preference for foreign DNA insertions (Figure 1, Table 2).

**Table 1:** Number of genes and total size of atypical regions compared to the whole *A. fumigatus *genome.

	*A. f. genome*	Atypical regions	% of total
**# annotated genes**	9,631	214	2.2%

**Total size**	29.4 Mb	908 kb	3.1%

**Table 2:** Distribution of atypical regions per chromosome.

Chromosome #	1	2	3	4	5	6	7	8
**% of total genome**	16.7	16.5	13.9	13.4	13.4	12.9	7.0	6.2

**# regions/chromosome**	30	28	28	22	22	31	14	14

**% atypical/chromosome**	11.8	16.1	22	9.9	6.4	18.8	7.2	7.7

### HT distribution on chromosomes

Though all chromosomes contained atypical regions some seemed to exhibit a particular distribution like a sub-telomeric trend on chromosome 4 or an under-representation on the short arm of chromosome 2. We also denoted that in some cases, atypical regions were physically clustered as it can be seen at position 2.3 Mb of chromosome 6 (c6r14-c6r23, representing 53 kb of atypical sequences out of 107 kb of genomic DNA) (Figure 1, Table 2).

### Content of atypical regions

The 189 atypical regions detected can be divided into two groups: those containing annotated genes (134) and those with no coding features (55). A total of 214 annotated genes are encoded in the atypical regions. We checked by BlastP if new homologs were sequenced since the genome analysis [76,78]. Most of these genes exhibited homologous counterparts (Additional files 1 and 2) with the exception of ORFans. The ORFans can be divided in 2 classes: 16 genes from *A. fumigatus *have no homologs at all in GenBank and 5 have a homolog only in *N. fischieri *a very close neighbor of *A. fumigatus*.

The functions of 81 transferred genes are unknown. Considering the other 133 genes, a function is inferred for 39 of them and a putative one for the 94 others (Additional file 3). The majority of them (91; 68%) belong to central and intermediate metabolism. We detected few genes involved in virulence [78,86] among the horizontally transferred genes although this means of virulence spreading was already demonstrated for pathogenic fungi [16,72,87,88]. We detected a few genes proposed to play a role in pathogenicity: 1 lipase, 4 peptide transporters [89], 5 genes of gliotoxin synthesis involved in virulence [90,91] and two genes coding for allergenic proteins. Also, we observed a high number of mobile elements detected in the atypical regions. Alongside the 214 genes, we found 129 transposons belonging to 5 families: Copia, Gypsy, hAT, Line and DDE1. In some cases, these transposons are clustered in a single region (Additional file 1, see c2p24, c4p18 or c6p2 for instance). We checked the signatures of mobile elements and found that they exhibited a signature close to that of the host genome and so were not the cause of the detection of the region but more likely markers of the transfer events [92].

**Figure 2:**
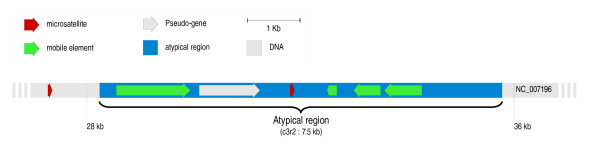
**Detail of the c3r2 region lacking annotated genes**.

Fifty-five atypical regions lacked annotated genes. Despite this, a BlastX and BlastN analyses allowed to propose the presence of gene relics in 24 (47%) of these regions (Table 3). Besides some rRNA genes (regions c4r5 and c4r6), supposedly not transferred but detected by the method, and transposons, we found pseudogenes of nuclear or mitochondrial origin and plasmid parts. Figure 2 shows an example of such a region containing both transposons and a pseudogene. The large numbers of transposons contained in these regions (Table 3) supports their status of horizontally transferred regions [92]. It is interesting to notice that 3 annotated genes and a pseudogene are of mitochondrial origin, indicating HTs between mitochondrial and nuclear genomes.

**Table 3:** Features detected by BlastX and/or BlastN in atypical regions lacking annotated genes.

Name	Features
**c1r1**	Short mitochondrial genome part
**c1r27**	Fragments of transposons, 1 DDE1 transposon
**c1r29**	Pseudogene
**c2r14**	Pseudogene, 1 gypsy transposon
**c2r17**	Fragments of transposons, 2 DDE1 transposons
**c2r27**	Pseudogene
**c3r2**	Pseudogene, 3 hAT transposons, 1 DDE1 tansposon
**c3r7**	Highly conservated transposon with in frame stops, 1 DDE1 transposon
**c3r8**	Pseudogene
**c3r9**	2 DDE1 transposons
**c3r12**	Numerous pseudogenes and fragments of transposons, 3 DDE1 transposons
**c3r14**	Pseudogene, 1 gypsy transposon
**c3r16**	Pseudogene
**c3r20**	Transposon-like element, 4 gypsy transposons, 1 LINE transposon
**c4r1**	Fragments of transposons, 1 DDE1 transposon
**c4r5**	28S rRNA, 4 LINE transposons
**c4r6**	18S rRNA
**c4r14**	Fragments of transposons, 2 DDE1 transposons
**c4r19**	Transposon-like element, 1 gypsy transposon, 3 LINE transposons
**c5r2**	1 LINE transposon
**c6r2**	Partial transposons and pseudogene, 5 gypsy transposons, 3 LINE transposons
**c6r3**	Plasmid part
**c6r14**	Partial transposons and pseudogene, 1 gypsy transposon, 2 LINE transposons
**c6r15**	1 LINE transposon
**c6r21**	Pseudogene, 2 TY1Copia transposons
**c6r22**	1 DDE1 tranposon
**c6r26**	5S rRNA
**c7r13**	Partial transposons and pseudogene, 2 gypsy transposons, 1 LINE transposon

Nomenclature of atypical regions is defined as follow: "c1" indicates the chromosome number and "r2" references the # of this region on the chromosome.

### Putative origin of the atypical regions

**Figure 3:**
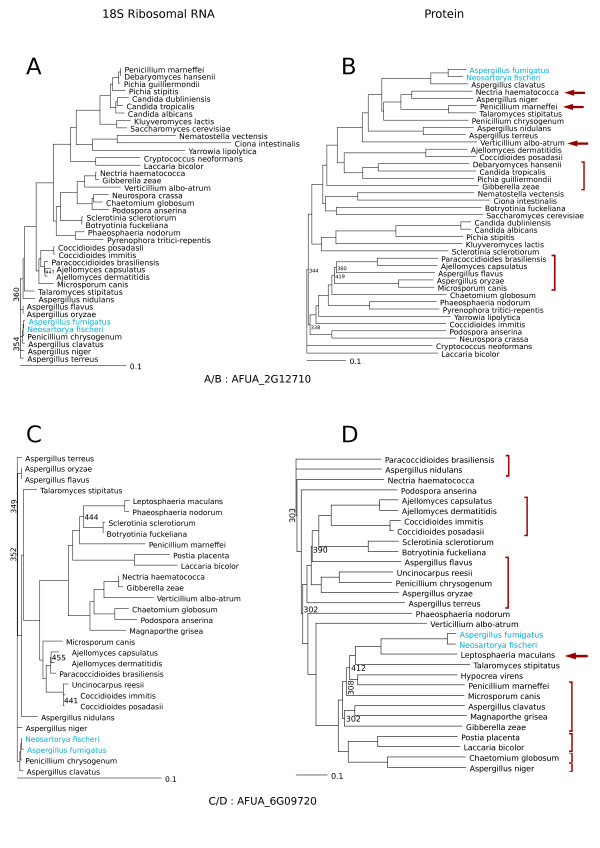
**Phylogenetic trees for gene/protein AFUA.2G12710 and AFUA.6G09720 and their respective SSU rRNA trees**. *A. fumigatus *and *N. fischieri *were highlighted in blue, main incongruencies between SSU rRNA tree and protein tree are indicated with red arrows or bars. Numbers at nodes correspond to the number of bootstrap trees out of 1000 supporting that node when this number is inferior to 500.

It is possible from the BlastP analysis to get an indication of the donor species except for the ORFans (Table 4, Additional file 2) or genes/proteins with few homologs. The majority of the homologs detected originated only from fungal species (56%). It is to be noted that 16 genes are specific of *A. fumigatus *(no homolog in other fungal species). All the other genes had homologs in at least one or the other *Aspergillus *sp. or *Neosartorya fischeri *(a very close relative of *A. fumigatus *[91]). This supports the view that most of the transfer events occurred before the *Aspergillus *speciation. For instance out of the 120 genes exhibiting homologs mainly in fungi, 18 (15% not taking into account the ORFans) had homologs only in *Aspergillus *sp. or in *N. fischeri*. However the patchiness of the *Aspergillus *species represented by the different genes suggests numerous rearrangements and gene losses in these species. Another point is that some genes had homologs only in *N. fischeri *(5, Table 4) confirming the very close relationship between *A. fumigatus *and *N. fischeri*. From the BlastP analysis, it can be noted that 19% exhibited homologs in other domains of life; for instance, 26 genes had homologs exclusively in prokaryotes out of the fungi homologs (Table 4). We also detected 19 homologs exclusively in other eukaryotic kingdoms (Table 4). From this analysis it is possible, not only to confirm the transferred status of the genes but also to propose in peculiar cases a source of these genes. The criterion for a confident result are a very high conservation (very low E-Value), a coverage over 90% and an alternation of fungi species with those from other domains or kingdoms. For instance, gene AFUA_7G06140 possibly originates from *Amoebozoa *species, gene AFUA_1G11310 from *Metazoa *species and genes AFUA_1G01660, AFUA_6G09600 and AFUA_6G09660 among others would be of Prokaryotic origin. Other genes exhibit a more complex perturbed evolutionary history like genes AFUA_1G05200 and AFUA_4G14130 originating from other Eukaryotic kingdoms and some exhibit a very complex history mixing Eukaryotic and Prokaryotic origins like genes AFUA_1G06810, AFUA_1G10110, AFUA_2G00720, AFUA_4G07710 or AFUA_5G10120 for instance. To confirm the transferred status and research an origin when the homologs were all from fungi origin or when the origin was more difficult to ascertain, phylogenetic trees were inferred (examples of phylogenetic trees are shown in Figure 3 and Additional file 4)). These phylogenetic protein trees exhibited large incongruencies as compared to their respective SSU rRNA trees. This confirmed a perturbed evolutionary history and supported the transferred status of these genes.

**Table 4:** Species of origin of BlastP hits for genes encoded in atypical regions.

Classes	ORFans	*A. fumigatus* + *N. fischeri*	*A. fumigatus* + *N. fischeri* + *A. clavatus*	Aspergillus species (+*N. fischeri*i)	fungi	fungi + plants	fungi + plants + procaryotes	fungi + procaryotes	fungi + animals	fungi + animals + procaryotes	fungi + eucaryotes	fungi + eucaryotes + procaryotes	Total
**Number**	16	5	5	8	102	3	12	26	5	12	11	9	214

**Figure 4:**
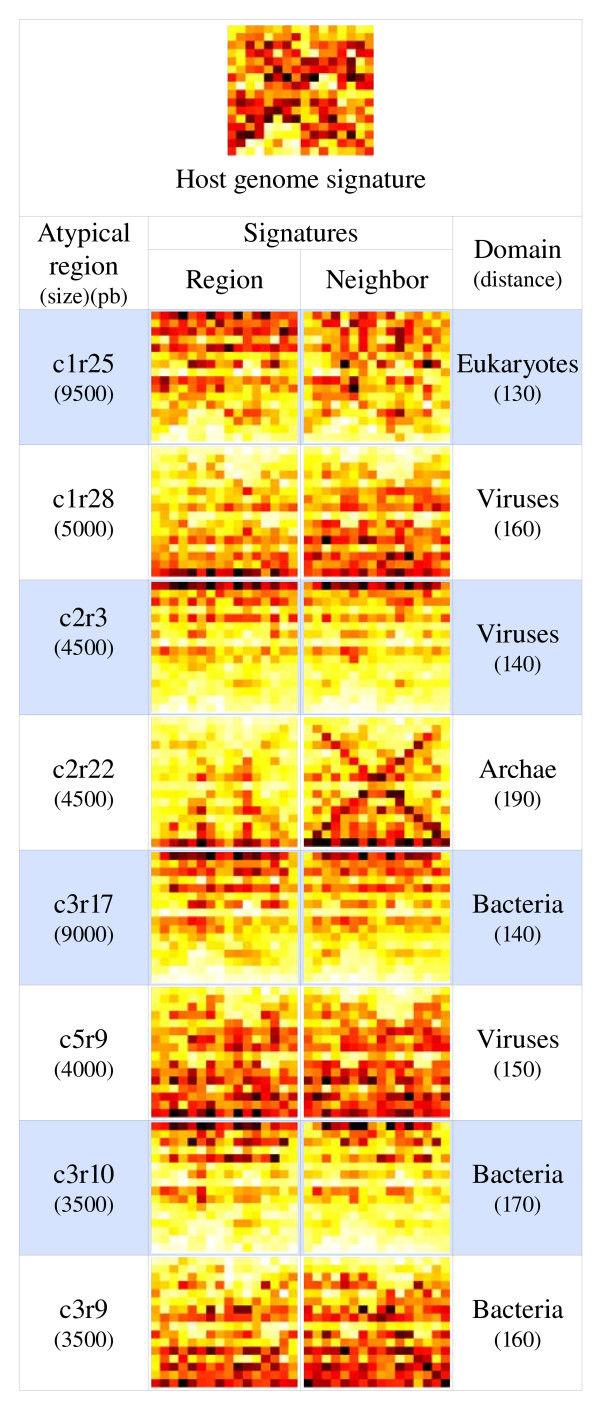
**Sample signatures of regions associated with the signature of their best neighbor (the distance between them is given in arbitrary units)**.

**Figure 5:**
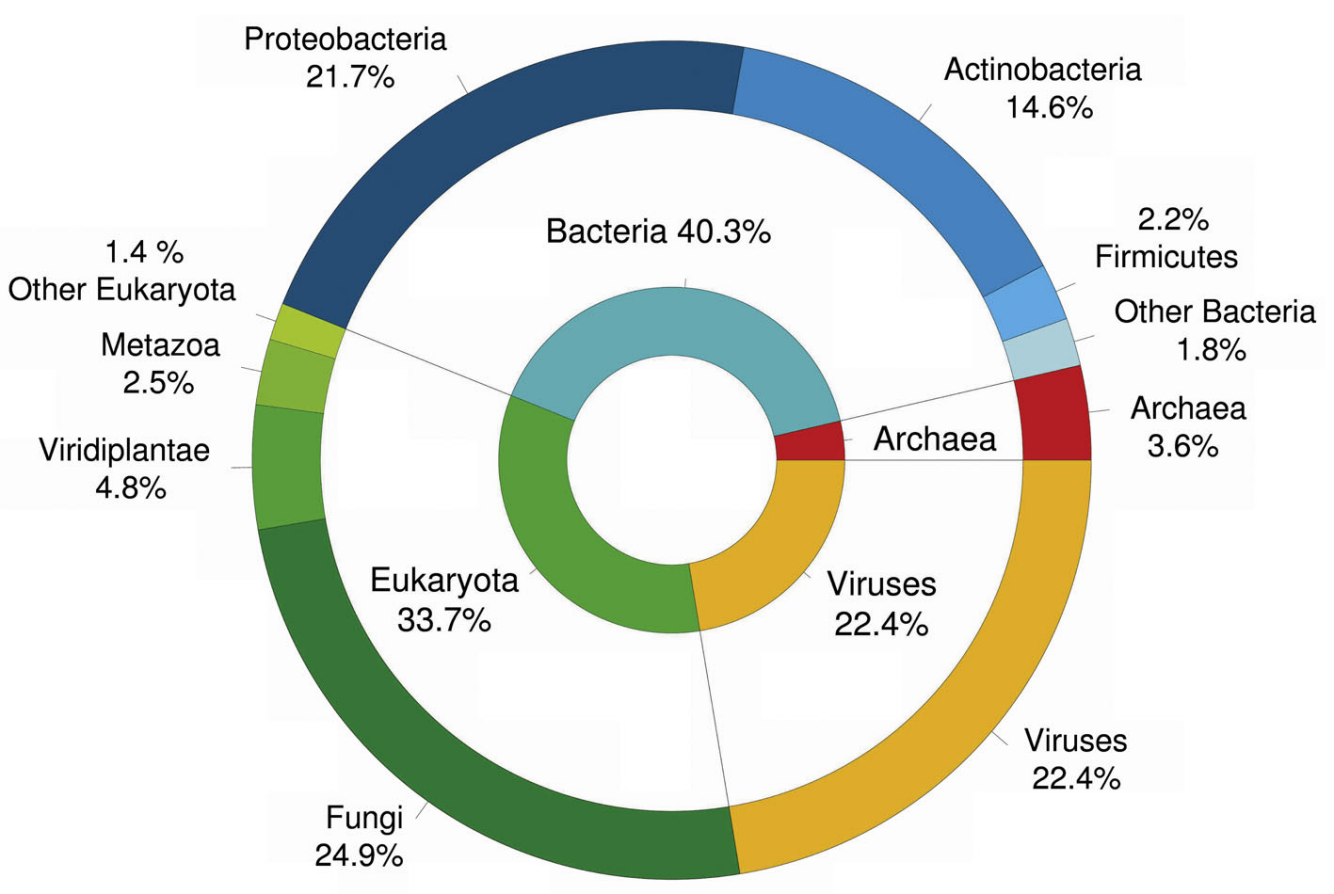
**Summary of the origin of atypical regions by domain and by family**.

It is difficult to assess the species of origin of the transferred genes from the Blast or the phylogenetic analyses due to the bias in homologous sequenced genes. Another way to propose a species of origin for an HT was to benefit from the species-specificity of the genomic signature. If the horizontally transferred regions have kept the characteristics of their species of origin, then by comparing their genomic signature to a homemade database of species signatures, we can obtain indications about their origin. We compared the signature of the 189 atypical regions to the database [53,82] and for 117 of them plausible donor species could be assigned (samples of region and of their closest neighbor signatures are shown in Figure 4). Due to possible miss-assignments caused either by the representativeness of the database or to the amelioration of the transferred sequences [93], only broad categories of donors are presented. Figure 5 presents the distribution of these donor species as a function of their origin. Three major groups of donors are identified: bacteria (40%), fungi (25%) and viruses (22%). Among the bacteria species two groups are over-represented: *Proteobacteria *and *Actinobacteria*. An important point is the very small number of exchanges between fungi and non-fungal eukaryotes detected either from the BlastP or the signature analyses.

## Discussion and Conclusion

As parametric methods were not used until now to detect horizontal transfers in eukaryote genomes, we used a method which requires only generic hypotheses: i.e. a signature quite homogeneous for the major part of the genome and a minority of regions exhibiting different signatures, these regions containing supposedly horizontally transferred DNA sequences. *A. fumigatus* is a genome of choice for this type of study, being an intermediate genome in terms of coding density (50% [78]) between high coding density prokaryotic genomes (often above 95%), or lower eukaryotes (P. tetraurelia ≈ 75%) and very low coding density of higher eukaryote genomes (Homo sapiens ≈ 1.5%). Moreover, the intrinsic variability of the *A. fumigatus* genome is quite low allowing the use of this type of parametric method (Figure 1).

The parameters used here are such that we favored specificity over sensitivity. In fact, the threshold of 99% percentile used in the definition of the host genome is very strict [6]. It was already shown that lowering the threshold level while increasing sensitivity decreases specificity such that the number of false positives increases [6,94]. Besides, the use of sliding windows does not allow the detection of short isolated genes and it is recommended to use it in combination with a gene-based method [33,58,68]. In our conditions, the quantity of HTs detected is probably under-estimated and could be considered, in the absence of a gold standard, as a minimum value. The Blast and phylogenetic analyzes confirmed the transferred status of the annotated genes embedded in the detected regions (Table 4, Additional file 2, Figure 3 and Additional file 4). These analyzes were possible only when the number of homologs was sufficient for such an analysis. Nevertheless, the agreement in all these methods supports the importance of horizontal transfers in A. fumigatus.

In our conditions, we were able to detect 189 regions, accounting for 3.1% of the genome exhibiting a signature different from that of the majority of the *A. fumigatus* genome (Table 1). The total amount of atypical DNA is consequent (almost 1 Mb) but with regards to the size of the genome it is under the average percentage detected in prokaryote genomes [6-8]. For instance, using the same method and in the same conditions, Dufraigne et al. detected an average of 5.6% of atypical regions for 22 prokaryote genomes as compared to the 3.1% detected here in *A. fumigatus*[6]. We also tested a lower threshold 97.5% percentile [6] to evaluate its effect on the quantity of atypical sequence detected. In this later case, the amount of atypical sequences of the genome accounted for 4.6%, so about a 50% increase as compared to the 99% percentile threshold but still lower than the amount detected in prokaryotic genomes. There are few direct comparison data for eukaryotes genomes as all the studies are based on Blast or phylogenetic studies and so concern only genes. For instance in the diatom *P. tricornutum*, 587 genes were considered of bacterial origin (about 6% of the total gene content but only about 2% of the genome sequence [44]), this is far more than the 214 annotated genes detected here in a genome of comparable size and coding density. Gene based methods do not take into account the whole transfer event which could contain intergenic regions or regions lacking annotated genes (relics of HT events) that could bring information on genome evolution as well as on transfer mechanisms.

Different causes could account, in the state of our knowledge, for the apparent lower amount of transfers in eukaryotes compared to prokaryotes. Either this is due to differences in the mechanisms responsible for HT in eukaryotes and prokaryotes making it biologically more difficult in eukaryotes and so decreasing its frequency. Either, if HTs occur at the same rate in both domains, foreign DNA is eliminated faster in eukaryote genomes. It must also be taken into account that considering gene exchange, the transferred genes must be selected and "ameliorated" to be expressed in a new eukaryotic environment. The high proportion of non-coding regions could be interpreted as an accelerated inactivation of useless genes, for instance because they originated from other domains of life and could not be expressed due to the differences in gene expression machinery. This phenomenon could account for the greater amount of detected regions lacking annotated genes that could be in the process of elimination as supported by the presence of pseudo-genes.

The putative HTs are spread among all the eight chromosomes exhibiting no positional bias (Figure 1, Table 2). The number of HTs per chromosome is proportional to the chromosome' size (Table 2). However, it seems that the average size of the transferred regions are a bit larger in*A. fumigatus* than the average in 22 prokaryotes species (4.5 kb vs 2.8 kb) [6]. Among the 189 atypical regions detected, six were larger than 20 kb and 35 (19%) exhibited the minimum detectable size of 500 bp.

Two detected regions (c4r5 and c4r6, Table 3, Additional file 1) are possibly false positives. Indeed, they contain rRNA and it was already shown that rRNA exhibits a specific signature [6,61]. One region (c4r6, 3 kb) contains quite exclusively rRNA (Table 3) while the other is an ambiguous case, it is larger (8 kb) than c4r6 and contains rRNA as well as two transposons and could be a remnant of a horizontal transfer event or a composite region with an HT event close to rRNA sequences (Table 3, Additional file 1).

For most of the genes included in the atypical regions, it was not possible to assign a function. Indeed, we were able to assign a putative function to 133 (62%) of the 214 atypical genes and 21 of them are ORFans. This fraction of HGTs with a function is comparable to recent publication where around 50% of the detected genes have no known function [68]. It is to be noted that 55 of the 189 atypical regions lack of annotated genes and apart from those containing rRNA (see above) they could be considered as remnants of HTs (see Table 3 for those containing pseudogenes or transposons) as the original gene content was presumably of no use for A. fumigatus. This proportion is far greater than for prokaryotic genomes, where only a few regions with no genes were detected [6,61]. Finally, the high number of transposons detected in atypical regions supports their horizontally transferred status [95].

The functions of transferred genes belonged mainly to the central and intermediate metabolism. Few genes seemed to be involved directly in pathogenicity, however, 5 genes (8 genes when using the 97.5% threshold, see above) out of 10 of the gliotoxin synthesis cluster, involved in virulence are detected as transferred. This result supports the hypothesis already proposed on the foreign origin of this cluster [91,96,97]. It is possible to propose a history of the evolution of this gene cluster. The original cluster was transferred in block to an ancestor of Aspergillus sp. on chromosome 6, then a duplication occurred giving birth to a second reduced cluster on chromosome 3 (7 genes) [91]. This small cluster was "ameliorated" (not detected) as it is often the case for duplicated genes. The original cluster also undergoes amelioration for some genes, as it appears that some genes cannot be detected in our conditions.

We obtained information of two different types on the origin of the transfers in A. fumigatus: one for genes only with the BlastP and the phylogenetic analyzes and another for whole HT regions with the signature analysis. These results are complementary and in rather good agreement if we take into account the fact that the first two analyzes are based on genes and the last on detected regions (including those with no annotated gene). The only discrepancy concerns the fact that we found no homologous genes in viruses (Table 4 and Figure 5). The BlastP analysis provided two striking facts. First, there are few horizontally transferred genes species-specific to *A. fumigatus* as we found only 16 genes (≈ 4% of the annotated transferred genes) with no homolog in other Aspergillus species nor in *N. fischeri*. Second, resulting from the previous statement, all the other genes exhibit homologous counterparts in other Aspergillus species or in *N. fischeri* indicating that these genes were transferred in a common ancestor of Aspergillus sp. and *N. fischeri* before the clade formation. This is why these genes belong to the Aspergillus core genome as defined by Fedorova et al. [76]. From the Blast analysis, we detected only 26 genes with only homologous counter-parts in fungi and prokaryotic genomes (Additional file 2), this number is in the lower bound of those reported for sequenced protist genomes by Keeling and Palmer (in "supplementary Table S1" [19]). Complementary information is provided by the search for the origin of the transferred regions as a whole. First of all, it is the only way to propose an origin for HT regions lacking annotated genes. Of course due to amelioration processes the species proposed could be different from the donor species. However, it was already shown that if we don't get the true species, we get information on the domain, the kingdom or the family as a function of the distance between the signature of the HT region and that of the proposed donors. For this reason, we only took into account broad categories of species to analyze the signature data (Figure 5). As already shown by different studies, the origin of HT regions is diverse and encompasses all domains of life (Figure 5) [12-14,16,24,29,72]. However, 3 groups of donor species are dominant here: bacteria, fungi and viruses (Figure 3). It was proposed that transduction was unlikely for HT in fungi due to a lack of knowledge about possible vectors [29]. Nevertheless, it appears that 22% of the donor species are viruses (Figure 5). A hypothesis to explain this fact would be that free viral DNA present in the environment [28] or in the intracellular compartment during phagocytosis [13,30] may be involved in transformation the same way as in prokaryotes.

Exchanges between eukaryotic species or between prokaryotes and eukaryotes are documented (see [29] for a review). However, while bacteria are represented by numerous donors belonging to Proteobacteria or Actinobacteria, archaea are seldom involved in HT in *A. fumigatus* (about 3% of the donor species and few homologs in Blast analysis, Additional file 2 and Figure 5). It is to be noted that if we proposed donor species from other domains of life, there are very few donor species from other eukaryotic kingdoms (only 9%, Figure 5) outside of the fungi kingdom (25%) whatever the method used (Table 4 and Figure 5) and the next eukaryotic group are plants (around 5%). This suggests that inter kingdom exchange of genetic material is more restricted than from the bacterial domain. However, due to the patchiness of the database for eukaryotic sequences, this result could change in the future when more sequences will be available for eukaryotic species. We also observed HT from organelle genomes as some mitochondrial fragments are embedded in atypical regions (Table 3, Additional files 1 and 3).

This work opens a field of study for evaluating the contribution of HTs to eukaryote genomes. The genomes concerned would be those presenting a low intrinsic variation, *i.e. *fungi, plants, lower eukaryotes, etc. with the exception of the highly intrinsically variable genomes of warm-blood vertebrates until appropriate methods are designed. At last, the biological mechanisms underlying those transfers remain to be elucidated as well as the biological role of the transferred genes.

## Abbreviations

HT: horizontal transfer; ME: mobile element; HGT: Horizontally transferred gene

## Authors' contributions

LM and JB carried out the experiments. PD designed the study and wrote the paper. LM and JB helped in the redaction of the paper. All the authors read and approved the final manuscript.

## Supplementary Material

Additional file 1 **Position and content of detected atypical regions**. Start and End = position of the region on the chromosome, Size in bp, ME = mobile element. Nomenclature of atypical regions is defined as follow: "c1" is indicating the chromosome number while "r2" made references to the # of this region on the chromosome. Click here for file

Additional file 2 **Origin of the homologous proteins from the Blast analysis**. For each annotated gene/protein the following information are given from left to right: Domain, Kingdom/class, species, Accession #, E-Value, and Coverage. Click here for file

Additional file 3 **Annotated function of the genes embedded in the atypical regions**. Annotated function of the genes embedded in the atypical regions. Click here for file

Additional file 4 **Phylogenetic trees**. Phylogenetic trees for genes/proteins AFUA.1G11310, AFUA.2G07440 and AFUA.2G17620 and their respective SSU rRNA trees. *A. fumigatus *and *N. fischieri *were highlighted in blue, main incongruencies between SSU rRNA tree and protein tree are indicated with red arrows or bars. Numbers at nodes correspond to the number of bootstrap trees out of 1000 supporting that node when this number is inferior to 500. Click here for file
