# Supplementary figures and images for: Whole genome evaluation of horizontal transfers in the pathogenic fungus Aspergillus fumigatus
Source: BMC Genomics. 2010 Mar 12;11:171. doi: 10.1186/1471-2164-11-171 (PMC2848249; doi:10.1186/1471-2164-11-171)

18S Ribosomal RNA

Protein

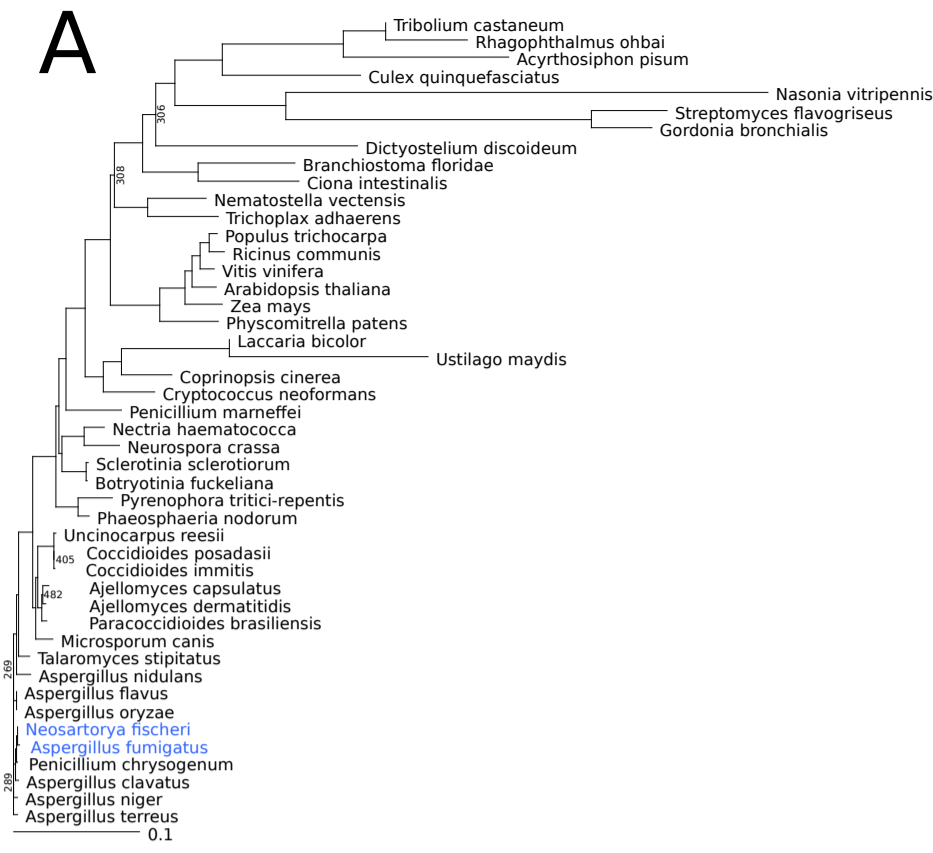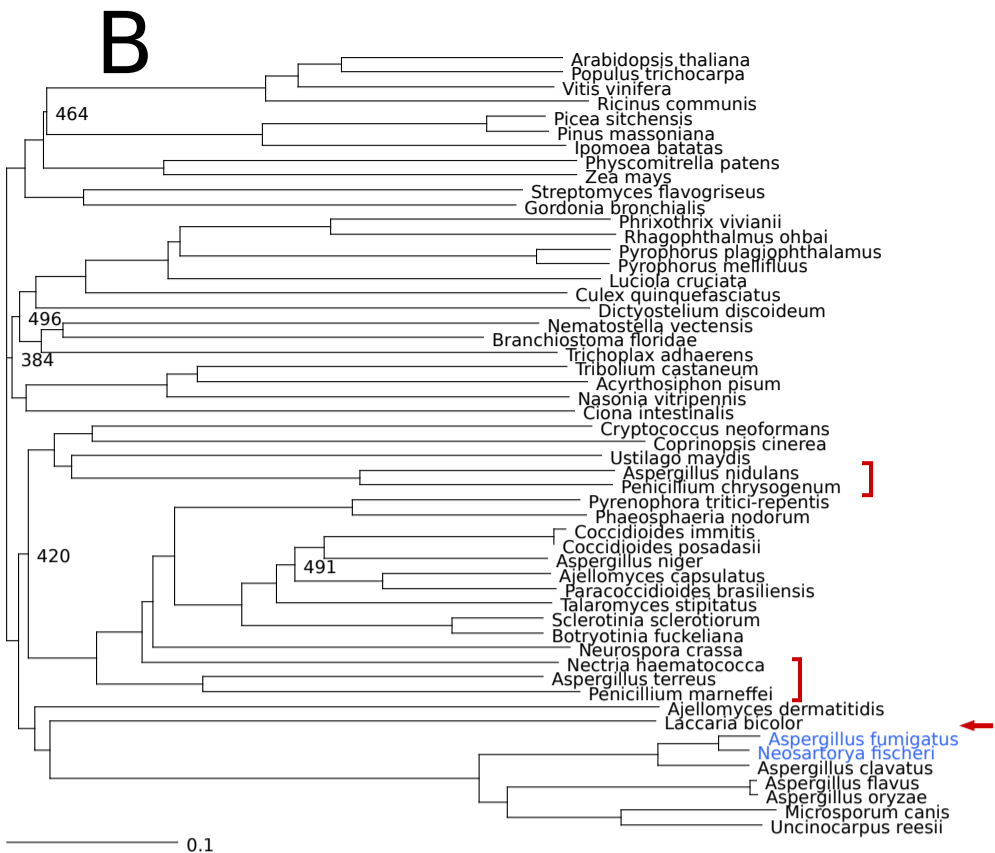

A/B : AFUA\_1G11310

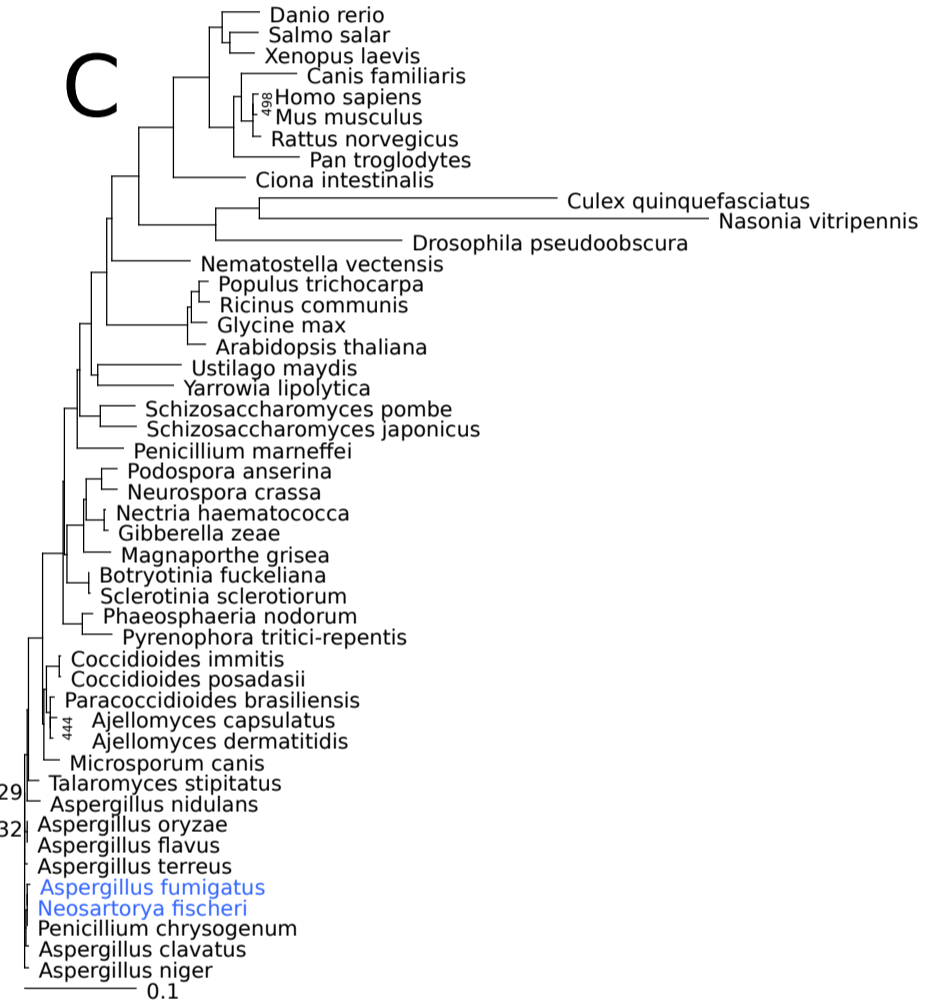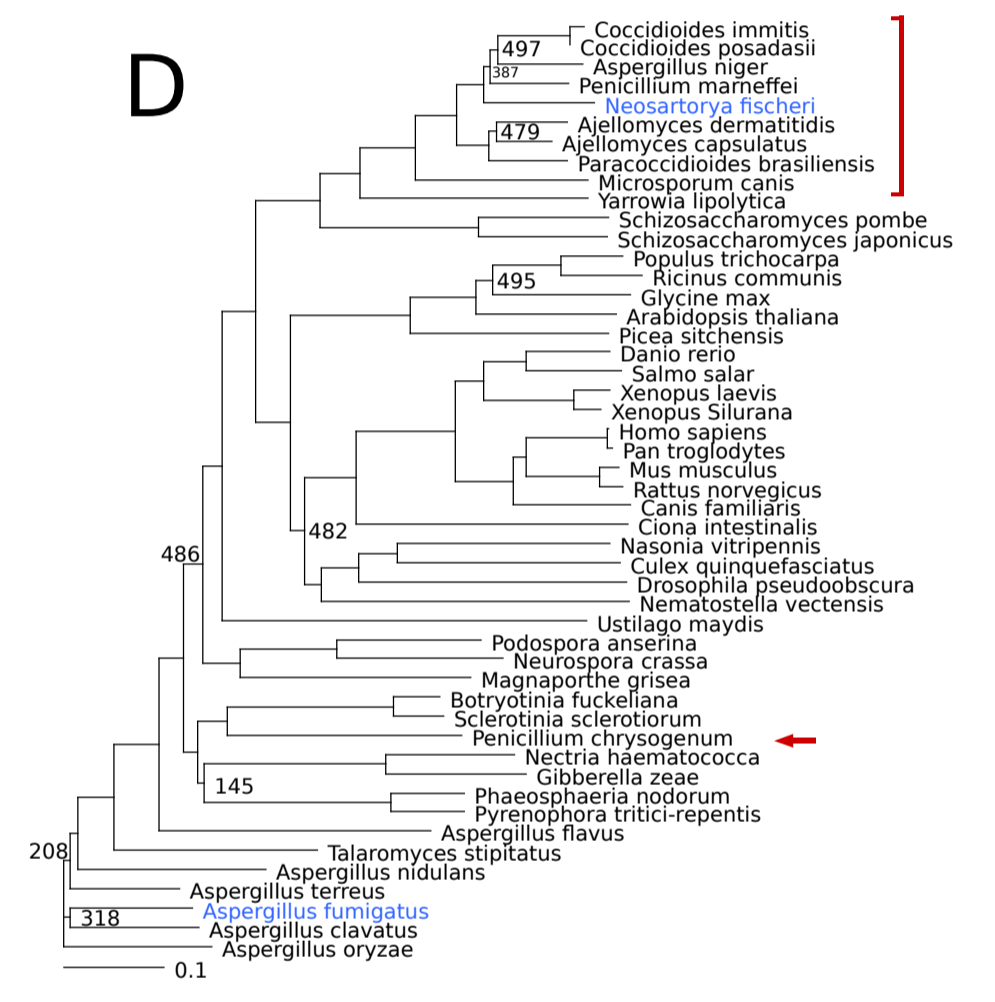

C/D : AFUA\_2G07440

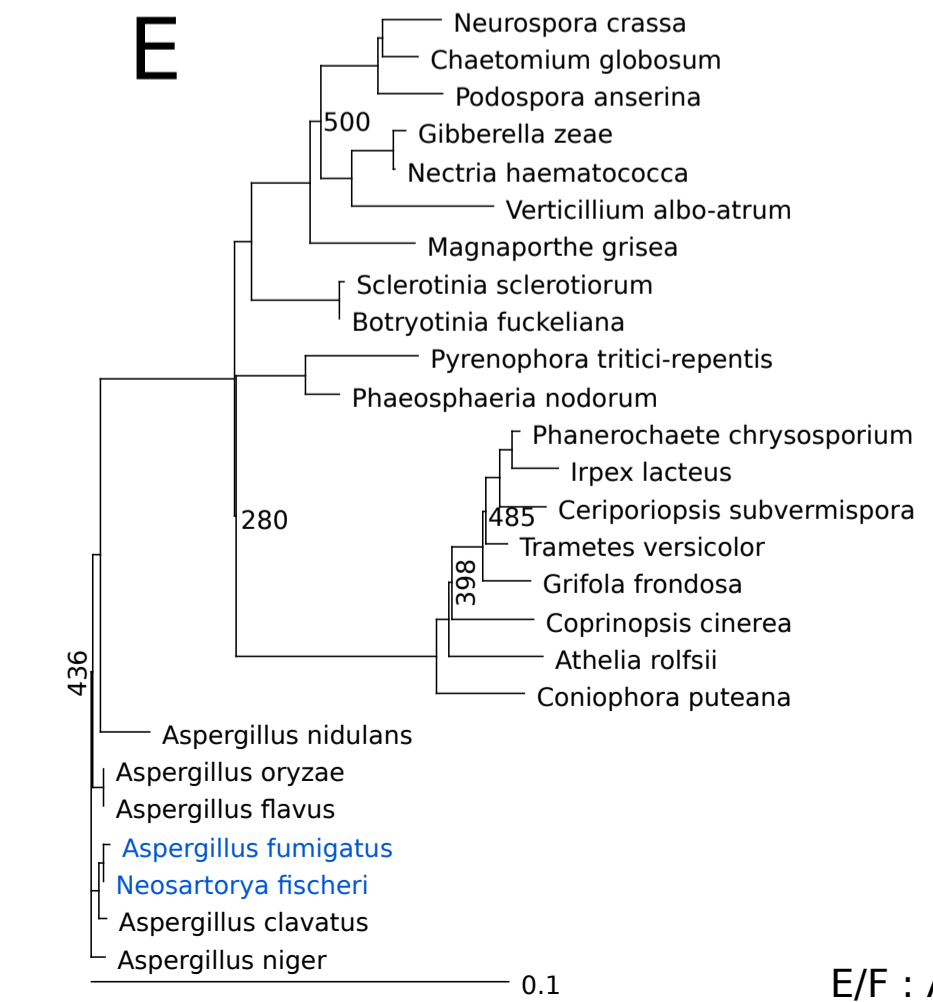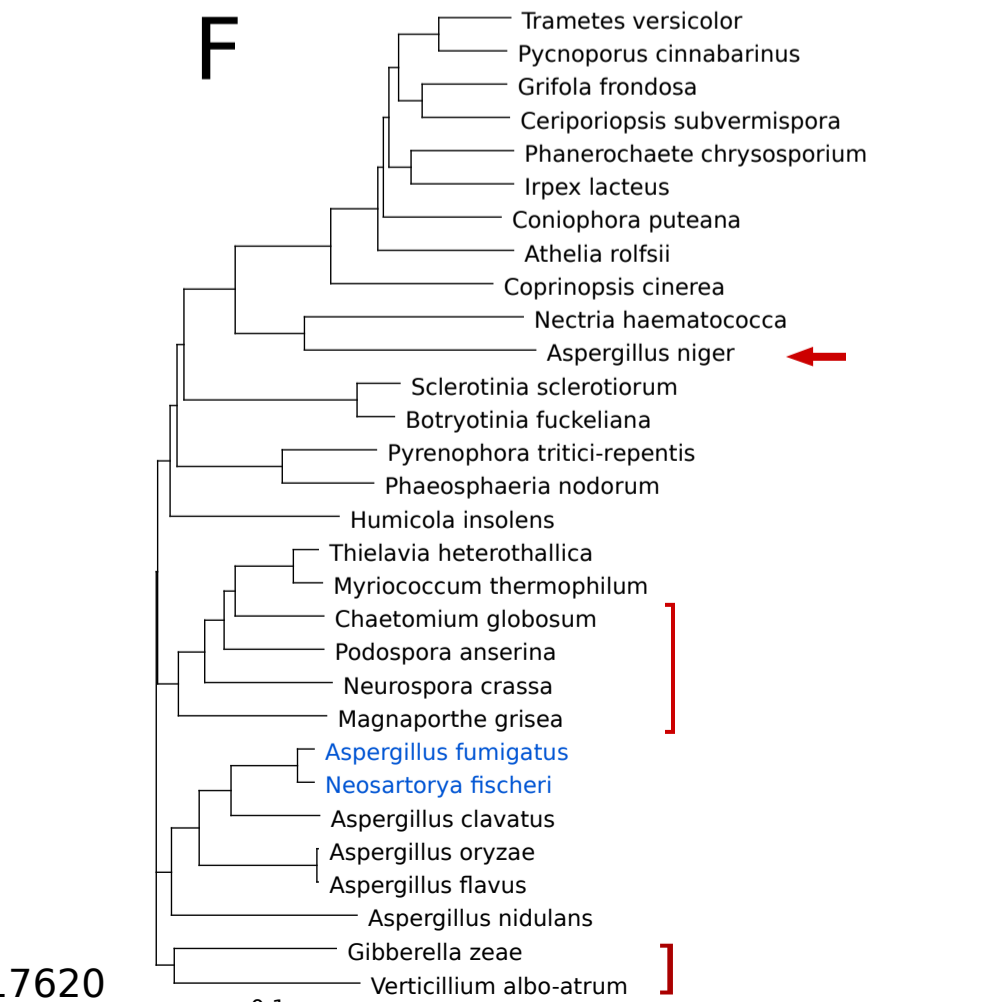

E/F : AFUA\_2G17620

Supplement: Additional file 1 — Position and content of detected atypical regions. Start and End = position of the region on the chromosome, Size in bp, ME = mobile element. Nomenclature of atypical regions is defined as follow: "c1" is indicating the chromosome number while "r2" made references to the # of this region on the chromosome. [file 1471-2164-11-171-S1.PDF]
