# Supplementary material for: Whole genome evaluation of horizontal transfers in the pathogenic fungus Aspergillus fumigatus
Source: BMC Genomics. 2010 Mar 12;11:171. doi: 10.1186/1471-2164-11-171 (PMC2848249; doi:10.1186/1471-2164-11-171)
Supplement: Additional file 2 — Origin of the homologous proteins from the Blast analysis. For each annotated gene/protein the following information are given from left to right: Domain, Kingdom/class, species, Accession #, E-Value, and Coverage. [file 1471-2164-11-171-S2.DOC]

**Additional file Table S1**:

Position and content of each atypical region detected (Start and End = position of the region on the chromosome, Size in bp, ME = mobile element). Nomenclature of atypical regions is defined as follow: “c1” is indicating the chromosome number while “r2” references the # of this region on the chromosome.

| Name | Start | End | size | # of genes | # of ME | Features and gene loci |
| --- | --- | --- | --- | --- | --- | --- |
| c1r1 | 231250 | 235250 | 4000 | 0 | 0 | Short mitochondrial genome part |
| c1r2 | 363750 | 369750 | 6000 | 0 | 0 |  |
| c1r3 | 370250 | 374250 | 4000 | 1 | 0 | AFUA1G01020 |
| c1r4 | 479750 | 480250 | 500 | 1 | 0 | AFUA1G01490 |
| c1r5 | 495250 | 496750 | 1500 | 1 | 0 | AFUA1G01570 |
| c1r6 | 517250 | 517750 | 500 | 1 | 0 | AFUA1G01660 |
| c1r7 | 518250 | 525250 | 7000 | 4 | 0 | AFUA1G01670, AFUA1G01680, AFUA1G01690, AFUA1G01700 |
| c1r8 | 1175250 | 1186250 | 11000 | 1 | 1 | AFUA1G04130, gypsy transposon |
| c1r9 | 1252750 | 1256250 | 3500 | 2 | 1 | AFUA1G04400, AFUA1G04410, DDE1 transposon |
| c1r10 | 1485750 | 1486750 | 1000 | 1 | 0 | AFUA1G05200 |
| c1r11 | 1693250 | 1693750 | 500 | 1 | 0 | AFUA1G05850 |
| c1r12 | 1694750 | 1695250 | 500 | 1 | 0 | AFUA1G05860 |
| c1r13 | 1718250 | 1719750 | 1500 | 1 | 0 | AFUA1G05960 |
| c1r14 | 1942250 | 1943750 | 1500 | 2 | 0 | AFUA1G06800, AFUA1G06810 |
| c1r15 | 2039250 | 2043750 | 4500 | 2 | 1 | AFUA1G07190, AFUA1G07195, DDE1 transposon |
| c1r16 | 2151250 | 2152250 | 1000 | 1 | 0 | AFUA1G07625 |
| c1r17 | 2188250 | 2191250 | 3000 | 1 | 1 | AFUA1G07750, 1 TY1Copia transposon |
| c1r18 | 2281250 | 2281750 | 500 | 0 | 0 |  |
| c1r19 | 2583750 | 2587250 | 3500 | 1 | 0 | AFUA1G10012 |
| c1r20 | 2607750 | 2608250 | 500 | 1 | 0 | AFUA1G10110 |
| c1r21 | 2893750 | 2908750 | 15000 | 1 | 4 | AFUA1G11060, 4 gypsy transposons |
| c1r22 | 2975750 | 2979750 | 4000 | 3 | 0 | AFUA1G11280, AFUA1G11290, AFUA1G11300 |
| c1r23 | 2980750 | 2981250 | 500 | 0 | 0 |  |
| c1r24 | 2982250 | 2984250 | 2000 | 1 | 0 | AFUA1G11310 |
| c1r25 | 2984750 | 2994250 | 9500 | 6 | 0 | AFUA1G11320, AFUA1G11330, AFUA1G11340, AFUA1G11350, AFUA1G11360, AFUA1G11370 |
| c1r26 | 3878250 | 3886250 | 8000 | 4 | 0 | AFUA1G14510, AFUA1G14520, AFUA1G14530, AFUA1G14540 |
| c1r27 | 4507750 | 4512250 | 4500 | 0 | 1 | Fragments of transposons, 1 DDE1 transposon |
| c1r28 | 4804250 | 4809250 | 5000 | 1 | 0 | AFUA1G17550 |
| c1r29 | 4853750 | 4856250 | 2500 | 0 | 0 | Pseudo-gene |
| c1r30 | 4914750 | 4915250 | 500 | 0 | 0 |  |
| c2r1 | 26750 | 27250 | 500 | 0 | 0 |  |
| c2r2 | 27750 | 29250 | 1500 | 1 | 0 | AFUA2G00160 |
| c2r3 | 161750 | 166250 | 4500 | 4 | 0 | AFUA2G00720, AFUA2G00730, AFUA2G00740, AFUA2G00750 |
| c2r4 | 1233750 | 1235750 | 2000 | 2 | 0 | AFUA2G04480, AFUA2G04490 |
| c2r5 | 1236250 | 1242250 | 6000 | 5 | 0 | AFUA2G04500, AFUA2G04510, AFUA2G04520, AFUA2G04530, AFUA2G04533 |
| c2r6 | 1423250 | 1427750 | 4500 | 1 | 0 | AFUA2G05150 |
| c2r7 | 1764750 | 1791750 | 27000 | 2 | 9 | AFUA2G06205, AFUA2G06220, 7 gyspy transposons, 2 TY1Copia transposons |
| c2r8 | 1819250 | 1822750 | 3500 | 1 | 0 | AFUA2G06330 |
| c2r9 | 1894750 | 1905250 | 10500 | 2 | 3 | AFUA2G07440, AFUA2G07450, 2 gypsy transposons, 1 DDE1 transposon |
| c2r10 | 1975750 | 1976250 | 500 | 1 | 0 | AFUA2G07710 |
| c2r11 | 2081750 | 2083250 | 1500 | 1 | 0 | AFUA2G08060 |
| c2r12 | 2230250 | 2232250 | 2000 | 1 | 0 | AFUA2G08670 |
| c2r13 | 2425250 | 2426250 | 1000 | 1 | 0 | AFUA2G09490 |
| c2r14 | 2465250 | 2476250 | 11000 | 0 | 1 | Pseudo-gene, 1 gypsy transposon |
| c2r15 | 2549750 | 2550750 | 1000 | 0 | 0 |  |
| c2r16 | 2651750 | 2655250 | 3500 | 2 | 1 | AFUA2G10360, AFUA2G10370, 1 gypsy transposon |
| c2r17 | 2661750 | 2666750 | 5000 | 0 | 2 | Fragments of transposons, 2 DDE1 transposons |
| c2r18 | 2761750 | 2762250 | 500 | 0 | 0 |  |
| c2r19 | 3262250 | 3264750 | 2500 | 3 | 0 | AFUA2G12700, AFUA2G12710, AFUA2G12720 |
| c2r20 | 3340750 | 3344250 | 3500 | 1 | 0 | AFUA2G13025 |
| c2r21 | 3350250 | 3353750 | 3500 | 1 | 0 | AFUA2G13060 |
| c2r22 | 3417750 | 3422250 | 4500 | 0 | 0 |  |
| c2r23 | 3433750 | 3437750 | 4000 | 3 | 0 | AFUA2G13295, AFUA2G13300, AFUA2G13310 |
| c2r24 | 4380750 | 4408250 | 27500 | 1 | 7 | AFUA2G16510, 5 gypsy transposons, 2 DDE1 transposons |
| c2r25 | 4546250 | 4552750 | 6500 | 2 | 0 | AFUA2G17000, AFUA2G17040 |
| c2r26 | 4699250 | 4703750 | 4500 | 2 | 0 | AFUA2G17610, AFUA2G17620 |
| c2r27 | 4724750 | 4726250 | 1500 | 0 | 0 | Pseudo-gene |
| c2r28 | 4800750 | 4803250 | 2500 | 1 | 0 | AFUA2G18070 |
| c3r1 | 10250 | 11750 | 1500 | 0 | 0 |  |
| c3r2 | 28250 | 35750 | 7500 | 0 | 4 | Pseudo-gene, 3 hAT transposons, 1 DDE1 tansposon |
| c3r3 | 136750 | 137750 | 1000 | 0 | 0 |  |
| c3r4 | 289250 | 290750 | 1500 | 0 | 0 |  |
| c3r5 | 312750 | 317750 | 5000 | 2 | 0 | AFUA3G01280, AFUA3G01290 |
| c3r6 | 601750 | 613750 | 12000 | 1 | 4 | AFUA3G02450, 4 gypsy transposons |
| c3r7 | 677750 | 682250 | 4500 | 0 | 1 | Highly conservated transposon with in frame stops, 1 DDE1 transposon |
| c3r8 | 683750 | 686750 | 3000 | 0 | 0 | Pseudo-gene |
| c3r9 | 834250 | 837750 | 3500 | 0 | 2 | 2 DDE1 transposons |
| c3r10 | 947750 | 951250 | 3500 | 2 | 0 | AFUA3G03540, AFUA3G03550 |
| c3r11 | 1015750 | 1025250 | 9500 | 0 | 0 |  |
| c3r12 | 1038250 | 1090750 | 52500 | 0 | 3 | Numerous pseudo-genes and fragments of transposons, 3 DDE1 transposons |
| c3r13 | 1161750 | 1162750 | 1000 | 1 | 0 | AFUA3G04100 |
| c3r14 | 1211750 | 1217750 | 6000 | 0 | 1 | Pseudo-gene, 1 gypsy transposon |
| c3r15 | 1219250 | 1229250 | 10000 | 1 | 2 | AFUA3G04300, 2 gypsy transposons |
| c3r16 | 1237750 | 1240750 | 3000 | 0 | 0 | Pseudo-gene |
| c3r17 | 1979250 | 1988250 | 9000 | 2 | 0 | AFUA3G07850, AFUA3G07860 |
| c3r18 | 1994250 | 1994750 | 500 | 0 | 0 |  |
| c3r19 | 2080250 | 2089250 | 9000 | 1 | 1 | AFUA3G08120, 1 gypsy transposon |
| c3r20 | 2384250 | 2400750 | 16500 | 0 | 5 | Transposon-like element, 4 gypsy transposons, 1 LINE transposon |
| c3r21 | 2403750 | 2410250 | 6500 | 2 | 3 | AFUA3G09450, AFUA3G09460, 3 TY1Copia transposons |
| c3r22 | 3117250 | 3118250 | 1000 | 1 | 0 | AFUA3G11840 |
| c3r23 | 3118750 | 3119250 | 500 | 1 | 0 | AFUA3G11850 |
| c3r24 | 3893750 | 3897250 | 3500 | 2 | 0 | AFUA3G14680, AFUA3G14690 |
| c3r25 | 4023750 | 4028750 | 5000 | 1 | 1 | AFUA3G15300, 1 DDE1 transposon |
| c3r26 | 4037250 | 4054750 | 17500 | 1 | 5 | AFUA3G15350, 4 gypsy transposons, 1 LINE transposon |
| c3r27 | 4063250 | 4065750 | 2500 | 0 | 0 |  |
| c3r28 | 4067250 | 4070250 | 3000 | 1 | 0 | AFUA3G15390 |
| c4r1 | 89750 | 90250 | 500 | 0 | 1 | Fragments of transposons, 1 DDE1 transposon |
| c4r2 | 91250 | 92750 | 1500 | 1 | 0 | AFUA4G00340 |
| c4r3 | 167250 | 169750 | 2500 | 2 | 0 | AFUA4G00610, AFUA4G00620 |
| c4r4 | 235250 | 235750 | 500 | 0 | 0 |  |
| c4r5 | 433750 | 441750 | 8000 | 0 | 4 | 28S rRNA, 4 LINE transposons |
| c4r6 | 442750 | 445750 | 3000 | 0 | 0 | 18S rRNA |
| c4r7 | 992750 | 993250 | 500 | 0 | 0 |  |
| c4r8 | 994750 | 995250 | 500 | 2 | 0 | AFUA4G03510, AFUA4G03520 |
| c4r9 | 1358250 | 1361750 | 3500 | 1 | 0 | AFUA4G04820 |
| c4r10 | 1639750 | 1641250 | 1500 | 2 | 0 | AFUA4G06410, AFUA4G06420 |
| c4r11 | 2013250 | 2014250 | 1000 | 1 | 0 | AFUA4G07710 |
| c4r12 | 2462250 | 2467250 | 5000 | 3 | 0 | AFUA4G09420, AFUA4G09440, AFUA4G09450 |
| c4r13 | 2492750 | 2493250 | 500 | 1 | 0 | AFUA4G09560 |
| c4r14 | 2624750 | 2629250 | 4500 | 0 | 2 | Fragments of transposons, 2 DDE1 transposons |
| c4r15 | 3564250 | 3567750 | 3500 | 1 | 0 | AFUA4G13630 |
| c4r16 | 3599250 | 3603750 | 4500 | 4 | 0 | AFUA4G13765, AFUA4G13770, AFUA4G13780, AFUA4G13800 |
| c4r17 | 3689750 | 3690250 | 500 | 1 | 0 | AFUA4G14130 |
| c4r18 | 3737750 | 3764250 | 26500 | 1 | 6 | AFUA4G14310, 6 gypsy transposons |
| c4r19 | 3779250 | 3796750 | 17500 | 0 | 4 | Transposon-like element, 1 gypsy transposon, 3 LINE transposons |
| c4r20 | 3809750 | 3810750 | 1000 | 1 | 0 | AFUA4G14410 |
| c4r21 | 3838250 | 3840250 | 2000 | 1 | 0 | AFUA4G14560 |
| c4r22 | 3909250 | 3910750 | 1500 | 0 | 0 |  |
| c5r1 | 56250 | 60250 | 4000 | 2 | 0 | AFUA5G00240, AFUA5G00250 |
| c5r2 | 66250 | 76250 | 10000 | 0 | 1 | 1 LINE transposon |
| c5r3 | 183750 | 188750 | 5000 | 2 | 1 | AFUA5G00650, AFUA5G00660, 1 DDE1 transposon |
| c5r4 | 368750 | 369250 | 500 | 0 | 0 |  |
| c5r5 | 424250 | 430250 | 6000 | 4 | 0 | AFUA5G01680, AFUA5G01690, AFUA5G01700, AFUA5G01710 |
| c5r6 | 436250 | 438250 | 2000 | 1 | 0 | AFUA5G01730 |
| c5r7 | 1056250 | 1057750 | 1500 | 1 | 0 | AFUA5G03960 |
| c5r8 | 1202250 | 1205250 | 3000 | 1 | 0 | AFUA5G04440 |
| c5r9 | 1567250 | 1571250 | 4000 | 0 | 0 |  |
| c5r10 | 1674250 | 1674750 | 500 | 1 | 0 | AFUA5G06800 |
| c5r11 | 1682250 | 1688750 | 6500 | 3 | 0 | AFUA5G06840, AFUA5G06850, AFUA5G06860 |
| c5r12 | 1879750 | 1880250 | 500 | 1 | 0 | AFUA5G07510 |
| c5r13 | 1892250 | 1896250 | 4000 | 2 | 0 | AFUA5G07570, AFUA5G07580 |
| c5r14 | 1903750 | 1905250 | 1500 | 1 | 0 | AFUA5G07620 |
| c5r15 | 2576750 | 2579750 | 3000 | 2 | 0 | AFUA5G09980, AFUA5G09990 |
| c5r16 | 2604750 | 2607250 | 2500 | 1 | 0 | AFUA5G10120 |
| c5r17 | 3467250 | 3467750 | 500 | 1 | 0 | AFUA5G13190 |
| c5r18 | 3715750 | 3716250 | 500 | 1 | 0 | AFUA5G14270 |
| c5r19 | 3832250 | 3832750 | 500 | 0 | 0 |  |
| c5r20 | 3834250 | 3835250 | 1000 | 1 | 0 | AFUA5G14840 |
| c5r21 | 3902750 | 3903750 | 1000 | 0 | 0 |  |
| c5r22 | 3904250 | 3904750 | 500 | 0 | 0 |  |
| c6r1 | 110750 | 115750 | 5000 | 2 | 1 | AFUA6G00440, AFUA6G00450 |
| c6r2 | 201750 | 229250 | 27500 | 0 | 8 | Fragments of transposons and pseudo-gene, 5 gypsy transposons, 3 LINE transposons |
| c6r3 | 232250 | 234250 | 2000 | 0 | 0 | Plasmid part |
| c6r4 | 241750 | 245250 | 3500 | 1 | 0 | AFUA6G01790 |
| c6r5 | 262750 | 263750 | 1000 | 1 | 0 | AFUA6G01860 |
| c6r6 | 267250 | 267750 | 500 | 1 | 0 | AFUA6G01900 |
| c6r7 | 269750 | 271250 | 1500 | 2 | 0 | AFUA6G01905, AFUA6G01910 |
| c6r8 | 750750 | 751750 | 1000 | 1 | 0 | AFUA6G03480 |
| c6r9 | 791750 | 793250 | 1500 | 1 | 0 | AFUA6G03630 |
| c6r10 | 1280750 | 1284250 | 3500 | 1 | 0 | AFUA6G05350 |
| c6r11 | 1349250 | 1365250 | 16000 | 1 | 4 | AFUA6G06350, 3 DDE1 transposons, 1 gypsy transposon |
| c6r12 | 2043750 | 2052250 | 8500 | 4 | 0 | AFUA6G08630, AFUA6G08640, AFUA6G08650, AFUA6G08660 |
| c6r13 | 2212250 | 2213250 | 1000 | 0 | 0 |  |
| c6r14 | 2291750 | 2304250 | 12500 | 0 | 3 | Fragments of transposons and pseudo-gene, 1 gypsy transposon, 2 LINE transposons |
| c6r15 | 2305250 | 2305750 | 500 | 0 | 1 | 1 LINE transposon |
| c6r16 | 2307250 | 2314750 | 7500 | 1 | 2 | AFUA6G09500, TY1Copia transposon, gypsy transposon |
| c6r17 | 2328250 | 2333250 | 5000 | 1 | 0 | AFUA6G09570 |
| c6r18 | 2335750 | 2339750 | 4000 | 4 | 0 | AFUA6G09580, AFUA6G09590, AFUA6G09600, AFUA6G09610 |
| c6r19 | 2352250 | 2357750 | 5500 | 2 | 0 | AFUA6G09650, AFUA6G09660 |
| c6r20 | 2368250 | 2371250 | 3000 | 3 | 0 | AFUA6G09720, AFUA6G09730, AFUA6G09740 |
| c6r21 | 2374250 | 2388250 | 14000 | 0 | 2 | Pseudo-gene, 2 TY1Copia transposons |
| c6r22 | 2396750 | 2398750 | 2000 | 0 | 1 | 1 DDE1 tranposon |
| c6r23 | 2487750 | 2501250 | 13500 | 2 | 2 | AFUA6G10100, AFUA6G10120, 1 gypsy transposon, 1 DDE1 transposon |
| c6r24 | 2873250 | 2874250 | 1000 | 1 | 0 | AFUA6G11580 |
| c6r25 | 2884750 | 2896750 | 12000 | 1 | 2 | AFUA6G11630, 2 gypsy transposons |
| c6r26 | 2934750 | 2940250 | 5500 | 0 | 0 | 5S rRNA |
| c6r27 | 2982250 | 2985250 | 3000 | 3 | 0 | AFUA6G11910, AFUA6G11920, AFUA6G11930 |
| c6r28 | 3316750 | 3317750 | 1000 | 1 | 0 | AFUA6G13120 |
| c6r29 | 3425750 | 3428250 | 2500 | 2 | 0 | AFUA6G13450, AFUA6G13460 |
| c6r30 | 3706750 | 3712250 | 5500 | 1 | 1 | AFUA6G14550, 1 DDE1 transposon |
| c6r31 | 3743750 | 3744250 | 500 | 0 | 0 |  |
| c7r1 | 23750 | 26250 | 2500 | 0 | 0 |  |
| c7r2 | 38750 | 39250 | 500 | 1 | 0 | AFUA7G00160 |
| c7r3 | 209250 | 211250 | 2000 | 1 | 0 | AFUA7G00805 |
| c7r4 | 479250 | 481750 | 2500 | 1 | 1 | AFUA7G01810, 1 gypsy transposon |
| c7r5 | 483750 | 484750 | 1000 | 1 | 0 | AFUA7G01820 |
| c7r6 | 623250 | 629250 | 6000 | 3 | 0 | AFUA7G02290, AFUA7G02300, AFUA7G02310 |
| c7r7 | 654250 | 660750 | 6500 | 1 | 0 | AFUA7G02390 |
| c7r8 | 721750 | 725250 | 3500 | 1 | 1 | AFUA7G02630, 1 gypsy transposon |
| c7r9 | 1198750 | 1206250 | 7500 | 4 | 0 | AFUA7G05080, AFUA7G05085, AFUA7G05090, AFUA7G05100 |
| c7r10 | 1508250 | 1508750 | 500 | 1 | 0 | AFUA7G06140 |
| c7r11 | 1683750 | 1684750 | 1000 | 1 | 0 | AFUA7G06900 |
| c7r12 | 1712250 | 1718250 | 6000 | 1 | 0 | AFUA7G07010 |
| c7r13 | 1787250 | 1808250 | 21000 | 0 | 3 | Fragments of transposons and pseudo-gene, 2 gypsy transposons, 1 LINE transposon |
| c7r14 | 1891750 | 1896250 | 4500 | 2 | 0 | AFUA7G08340, AFUA7G08350 |
| c8r1 | 64250 | 73250 | 9000 | 1 | 2 | AFUA8G00342, TY1Copia transposon, gypsy transposon |
| c8r2 | 533750 | 534250 | 500 | 2 | 0 | AFUA8G02050, AFUA8G02060 |
| c8r3 | 571250 | 571750 | 500 | 1 | 0 | AFUA8G02200 |
| c8r4 | 580250 | 589250 | 9000 | 2 | 1 | AFUA8G02230, AFUA8G02250, 1 LINE transposon |
| c8r5 | 591750 | 606750 | 15000 | 1 | 6 | AFUA8G02260, 2 TY1Copia transposons, 4 gypsy transposons |
| c8r6 | 752750 | 765250 | 12500 | 2 | 3 | AFUA8G02810, AFUA8G02820, 2 sypsy transposons, 1 DDE1 transposons |
| c8r7 | 774750 | 778750 | 4000 | 2 | 0 | AFUA8G02850, AFUA8G02860 |
| c8r8 | 782750 | 786250 | 3500 | 1 | 2 | AFUA8G02870, 2 gyspsy transposons |
| c8r9 | 1559750 | 1562250 | 2500 | 1 | 0 | AFUA8G06470 |
| c8r10 | 1613250 | 1619250 | 6000 | 0 | 0 |  |
| c8r11 | 1623250 | 1623750 | 500 | 0 | 0 |  |
| c8r12 | 1669750 | 1670250 | 500 | 1 | 0 | AFUA8G06820 |
| c8r13 | 1814250 | 1816750 | 2500 | 1 | 0 | AFUA8G07380 |
| c8r14 | 1817250 | 1821250 | 4000 | 1 | 0 | AFUA8G07390 |
