# Supplementary material for: Whole genome evaluation of horizontal transfers in the pathogenic fungus Aspergillus fumigatus
Source: BMC Genomics. 2010 Mar 12;11:171. doi: 10.1186/1471-2164-11-171 (PMC2848249; doi:10.1186/1471-2164-11-171)
Supplement: Additional file 3 — Annotated function of the genes embedded in the atypical regions. Annotated function of the genes embedded in the atypical regions. [file 1471-2164-11-171-S3.PDF]

Additional file Table S2: Origin of the homologous proteins from the Blast analysis.

Domain	Kingdom	Species	Accession #	E-value	Coverage
AFUA_1G01020					
Eukaryota	Fungi	Aspergillus fumigatus Af293	XP_749852.1	0.0	1525/1525
Eukaryota	Fungi	Aspergillus niger CBS 513.88	XP_001398523.1	0.0	1249/1525
Eukaryota	Fungi	Talaromyces stipitatus ATCC 10500	XP_002339929.1	1 E-159	1496/1525
Eukaryota	Fungi	Penicillium marneffei ATCC 18224	XP_002146565.1	1 E-155	1468/1525
Eukaryota	Fungi	Penicillium marneffei ATCC 18224	XP_002152624.1	1 E-137	1361/1525
Eukaryota	Fungi	Penicillium marneffei ATCC 18224	XP_002144654.1	1 E-127	1279/1525
Eukaryota	Fungi	Penicillium marneffei ATCC 18224	XP_002149045.1	1 E-124	1357/1525
Eukaryota	Fungi	Talaromyces stipitatus ATCC 10500	XP_002487679.1	1 E-116	1307/1525
Eukaryota	Fungi	Aspergillus oryzae RIB40	XP_001825695.1	1 E-115	1352/1525
Eukaryota	Fungi	Aspergillus nidulans FGSC A4	XP_661147.1	1 E-115	1265/1525
Eukaryota	Fungi	Aspergillus oryzae RIB40	XP_001824688.1	1 E-109	1405/1525
AFUA_1G01490					
Eukaryota	Fungi	Aspergillus fumigatus Af293	XP_749899.1	0.0	1954/1954
Eukaryota	Fungi	Neosartorya fischeri NRRL 181	XP_001265502.1	0.0	1954/1954
Eukaryota	Fungi	Aspergillus clavatus NRRL 1	XP_001269989.1	0.0	1956/1954
Eukaryota	Fungi	Aspergillus flavus NRRL3357	XP_002382577.1	0.0	1963/1954
Eukaryota	Fungi	Aspergillus terreus NIH2624	XP_001214103.1	0.0	1953/1954
Eukaryota	Fungi	Aspergillus nidulans FGSC A4	XP_657995.1	0.0	1961/1954
Eukaryota	Fungi	Aspergillus oryzae RIB40	XP_001822471.1	0.0	1752/1954
Eukaryota	Fungi	Aspergillus niger CBS 513.88	XP_001397257.1	0.0	1782/1954
Eukaryota	Fungi	Penicillium chrysogenum Wisconsin 54-1255	XP_002558820.1	0.0	1780/1954
Eukaryota	Fungi	Aspergillus flavus NRRL3357	XP_002375393.1	0.0	1930/1954
Eukaryota	Fungi	Aspergillus oryzae RIB40	XP_001727157.1	0.0	1733/1954
Eukaryota	Fungi	Sclerotinia sclerotiorum 1980 UF-70	XP_001590074.1	1 E-173	1841/1954
Eukaryota	Fungi	Botryotinia fuckeliana B05.10	XP_001551874.1	1 E-167	1755/1954
Eukaryota	Fungi	Pyrenophora tritici-repentis Pt-1C-BFP	XP_001932759.1	1 E-110	1858/1954
Eukaryota	Fungi	Phaeosphaeria nodorum SN15	XP_001796509.1	1 E-110	1845/1954
Eukaryota	Fungi	Aspergillus terreus NIH2624	XP_001216531.1	3 E-94	1961/1954
Eukaryota	Fungi	Aspergillus fumigatus Af293	XP_749311.1	4 E-91	1903/1954
Eukaryota	Fungi	Talaromyces stipitatus ATCC 10500	XP_002481333.1	5 E-91	1845/1954
Eukaryota	Fungi	Aspergillus fumigatus A1163	EDP53839.1	7 E-91	1903/1954
Eukaryota	Fungi	Neosartorya fischeri NRRL 181	XP_001265739.1	4 E-89	1810/1954
Eukaryota	Fungi	Penicillium marneffei ATCC 18224	XP_002147183.1	3 E-87	1844/1954
Eukaryota	Fungi	Aspergillus clavatus NRRL 1	XP_001273035.1	2 E-85	1814/1954
Eukaryota	Fungi	Podospora anserina DSM 980	XP_001910398.1	3 E-83	1878/1954
Eukaryota	Fungi	Aspergillus clavatus NRRL 1	XP_001276715.1	1 E-82	1928/1954
Eukaryota	Fungi	Gibberella zeae PH-1	XP_383329.1	6 E-81	1960/1954
Eukaryota	Fungi	Aspergillus niger CBS 513.88	XP_001390542.1	5 E-80	1829/1954
Eukaryota	Fungi	Penicillium chrysogenum Wisconsin 54-1255	XP_002568702.1	6 E-79	1862/1954
Eukaryota	Fungi	Aspergillus flavus NRRL3357	XP_002384326.1	4 E-72	1918/1954
Eukaryota	Fungi	Aspergillus terreus NIH2624	XP_001215198.1	1 E-69	1863/1954
Eukaryota	Fungi	Penicillium chrysogenum Wisconsin 54-1255	XP_002559842.1	4 E-62	1927/1954
Eukaryota	Fungi	Penicillium chrysogenum Wisconsin 54-1255	XP_002561500.1	5 E-61	1942/1954
Eukaryota	Fungi	Aspergillus oryzae RIB40	XP_001827095.1	3 E-60	1754/1954
Eukaryota	Fungi	Ustilago maydis 521	XP_761590.1	6 E-36	1809/1954
AFUA_1G01570					
Eukaryota	Fungi	Aspergillus fumigatus Af293	XP_749907.1	0.0	365/365

Eukaryota	Fungi	Neosartorya fischeri NRRL 181	XP_001265494.1	1 E-109	352/365
Eukaryota	Fungi	Aspergillus clavatus NRRL 1	XP_001269981.1	3 E-29	328/365

AFUA_1G01660

Eukaryota	Fungi	Aspergillus fumigatus Af293	XP_749916.1	0.0	676/676
Eukaryota	Fungi	Neosartorya fischeri NRRL 181	XP_001265483.1	0.0	654/676
Eukaryota	Fungi	Aspergillus flavus NRRL3357	XP_002374055.1	0.0	645/676
Eukaryota	Fungi	Aspergillus oryzae RIB40	XP_001820356.1	0.0	645/676
Eukaryota	Fungi	Aspergillus niger CBS 513.88	XP_001389086.1	0.0	646/676
Eukaryota	Fungi	Aspergillus kawachii	BAF98236.1	0.0	644/676
Eukaryota	Fungi	Aspergillus aculeatus	AAK16249.1	0.0	646/676
Eukaryota	Fungi	Penicillium chrysogenum Wisconsin 54-1255	XP_002556953.1	0.0	637/676
Eukaryota	Fungi	Aspergillus aculeatus	AAG13964.1	0.0	592/676
Eukaryota	Fungi	Pyrenophora tritici-repentis Pt-1C-BFP	XP_001936288.1	0.0	625/676
Eukaryota	Fungi	Phaeosphaeria nodorum SN15	XP_001800644.1	0.0	612/676
Eukaryota	Fungi	Sclerotinia sclerotiorum 1980 UF-70	XP_001596870.1	0.0	595/676
Eukaryota	Fungi	Botryotinia fuckeliana B05.10	XP_001555198.1	1 E-158	630/676
Eukaryota	Fungi	Aspergillus fumigatus Af293	XP_750362.1	1 E-114	634/676
Eukaryota	Fungi	Neosartorya fischeri NRRL 181	XP_001265056.1	1 E-114	634/676
Bacteria	Proteobacteria	Stigmatella aurantiaca DW4/3-1	ZP_01461681.1	1 E-109	617/676
Eukaryota	Fungi	Talaromyces stipitatus ATCC 10500	XP_002480428.1	1 E-107	598/676
Eukaryota	Fungi	Postia placenta Mad-698-R	XP_002472899.1	1 E-107	605/676
Eukaryota	Fungi	Penicillium marneffei ATCC 18224	XP_002144047.1	1 E-106	638/676
Eukaryota	Fungi	Aspergillus terreus NIH2624	XP_001212196.1	1 E-105	616/676
Eukaryota	Fungi	Aspergillus flavus NRRL3357	XP_002383141.1	1 E-105	604/676
Bacteria	Actinobacteria	Catenulispora acidiphila DSM 44928	YP_003115974.1	1 E-104	552/676
Eukaryota	Fungi	Sclerotinia sclerotiorum 1980 UF-70	XP_001585617.1	1 E-103	616/676
Eukaryota	Fungi	Aspergillus nidulans FGSC A4	XP_659810.1	2 E-96	610/676
Eukaryota	Fungi	Emericella nidulans	ABF50852.1	3 E-96	610/676
Eukaryota	Fungi	Aspergillus niger CBS 513.88	XP_001398938.1	5 E-96	555/676
Eukaryota	Fungi	Penicillium chrysogenum Wisconsin 54-1255	XP_002558612.1	1 E-94	559/676
Eukaryota	Fungi	Podospora anserina DSM 980	XP_001911063.1	2 E-91	636/676
Eukaryota	Fungi	Verticillium albo-atrum VaMs.102	EEY15197.1	3 E-89	604/676
Eukaryota	Fungi	Debaryomyces hansenii	CAG87565.2	8 E-86	618/676
Eukaryota	Fungi	Debaryomyces hansenii CBS767	XP_459370.1	3 E-85	618/676
Eukaryota	Fungi	Yarrowia lipolytica CLIB122	XP_503039.1	2 E-83	644/676
Eukaryota	Fungi	Pichia pastoris GS115	XP_002493420.1	3 E-81	594/676
Eukaryota	Fungi	Verticillium albo-atrum VaMs.102	EEY23294.1	1 E-75	634/676
Eukaryota	Fungi	Sclerotinia sclerotiorum 1980 UF-70	XP_001594733.1	3 E-36	611/676
Eukaryota	Fungi	Magnaporthe grisea 70-15	XP_359531.1	2 E-27	658/676

AFUA_1G01670

Eukaryota	Fungi	Aspergillus fumigatus Af293	XP_749917.1	0.0	466/466

AFUA_1G01680

Eukaryota	Fungi	Aspergillus fumigatus Af293	XP_749918.1	0.0	379/379
Eukaryota	Fungi	Neosartorya fischeri NRRL 181	XP_001265481.1	0.0	379/379
Eukaryota	Fungi	Aspergillus clavatus NRRL 1	XP_001269973.1	0.0	377/379
Eukaryota	Fungi	Aspergillus oryzae RIB40	XP_001817940.1	1 E-176	388/379
Eukaryota	Fungi	Aspergillus terreus NIH2624	XP_001214101.1	1 E-171	357/379
Eukaryota	Fungi	Aspergillus flavus NRRL3357	XP_002373103.1	1 E-168	377/379
Eukaryota	Fungi	Paracoccidioides brasiliensis Pb01;	EEH35025.1	1 E-163	377/379
Eukaryota	Fungi	Paracoccidioides brasiliensis Pb18;	EEH43225.1	1 E-162	386/379
Eukaryota	Fungi	Ajellomyces capsulatus NAm1	XP_001541205.1	1 E-162	375/379
Eukaryota	Fungi	Ajellomyces dermatitidis SLH14081	XP_002625913.1	1 E-161	375/379

Eukaryota	Fungi	Ajellomyces capsulatus G186AR	EEH06435.1	1 E-161	375/379
Eukaryota	Fungi	Penicillium marneffei ATCC 18224	XP_002152979.1	1 E-161	378/379
Eukaryota	Fungi	Aspergillus nidulans FGSC A4	XP_657989.1	1 E-161	377/379
Eukaryota	Fungi	Talaromyces stipitatus ATCC 10500	XP_002484561.1	1 E-159	378/379
Eukaryota	Fungi	Coccidioides immitis RS;	XP_001238891.1	1 E-157	383/379
Eukaryota	Fungi	Penicillium chrysogenum Wisconsin 54-1255	XP_002566145.1	1 E-155	373/379
Eukaryota	Fungi	Microsporum canis CBS 113480	EEQ34267.1	1 E-149	369/379
Eukaryota	Fungi	Uncinocarpus reesii 1704	XP_002585370.1	1 E-145	362/379
Eukaryota	Fungi	Sclerotinia sclerotiorum 1980 UF-70	XP_001586239.1	1 E-138	369/379
Eukaryota	Fungi	Botryotinia fuckeliana B05.10	XP_001553555.1	1 E-136	372/379
Eukaryota	Fungi	Podospora anserina DSM 980	XP_001912778.1	1 E-135	367/379
Eukaryota	Fungi	Paracoccidioides brasiliensis Pb03;	EEH16496.1	1 E-135	333/379
Eukaryota	Fungi	Pyrenophora tritici-repentis Pt-1C-BFP	XP_001933179.1	1 E-134	344/379
Eukaryota	Fungi	Gibberella zeae PH-1	XP_385872.1	1 E-131	349/379
Eukaryota	Fungi	Phaeosphaeria nodorum SN15	XP_001794209.1	1 E-129	345/379
Eukaryota	Fungi	Nectria haematococca mpVI 77-13-4	EEU40620.1	1 E-127	376/379
Eukaryota	Fungi	Aspergillus niger CBS 513.88	XP_001389078.1	1 E-124	374/379
Eukaryota	Fungi	Magnaporthe grisea 70-15	XP_365423.1	1 E-121	377/379
Eukaryota	Fungi	Chaetomium globosum CBS 148.51	XP_001220449.1	1 E-111	350/379
Eukaryota	Fungi	Penicillium marneffei ATCC 18224	XP_002147519.1	1 E-111	349/379
Eukaryota	Fungi	Aspergillus oryzae RIB40	XP_001825739.1	1 E-106	336/379
Eukaryota	Fungi	Aspergillus clavatus NRRL 1	XP_001273101.1	1 E-106	351/379
Eukaryota	Fungi	Talaromyces stipitatus ATCC 10500	XP_002477831.1	1 E-106	347/379
Eukaryota	Fungi	Phaeosphaeria nodorum SN15	XP_001799395.1	1 E-104	350/379
Eukaryota	Fungi	Microsporum canis CBS 113480	EEQ35615.1	1 E-102	352/379
Eukaryota	Fungi	Aspergillus niger CBS 513.88	XP_001393514.1	1 E-101	363/379
Eukaryota	Fungi	Pyrenophora tritici-repentis Pt-1C-BFP	XP_001942152.1	3 E-99	366/379
Eukaryota	Fungi	Talaromyces stipitatus ATCC 10500	XP_002487171.1	1 E-98	345/379
Eukaryota	Fungi	Uncinocarpus reesii 1704	XP_002544949.1	2 E-97	352/379
Eukaryota	Fungi	Aspergillus nidulans FGSC A4	XP_681145.1	6 E-94	333/379
Eukaryota	Fungi	Alternaria alternata	BAI44740.1	3 E-93	348/379
Eukaryota	Fungi	Alternaria alternata	BAI44762.1	7 E-93	348/379
Eukaryota	Fungi	Aspergillus niger CBS 513.88	XP_001402456.1	1 E-92	322/379
Eukaryota	Fungi	Penicillium chrysogenum Wisconsin 54-1255	XP_002568114.1	3 E-91	334/379
Eukaryota	Fungi	Ustilago maydis 521	XP_758930.1	2 E-84	370/379
Eukaryota	Fungi	Cochliobolus carbonum	Q9Y885.1	1 E-82	346/379
Eukaryota	Fungi	Pyrenophora tritici-repentis Pt-1C-BFP	XP_001942349.1	1 E-78	314/379
Eukaryota	Fungi	Aspergillus niger CBS 513.88	XP_001390686.1	7 E-74	351/379
Eukaryota	Fungi	Pyrenophora tritici-repentis Pt-1C-BFP	XP_001939429.1	5 E-71	352/379
Eukaryota	stramenopiles	Phaeodactylum tricornutum CCAP 1055/1	XP_002178511.1	3 E-69	341/379
Bacteria	Bacteroidetes	Prevotella melaninogenica ATCC 25845	ZP_04832724.1	4 E-69	333/379
Bacteria	Fibrobacteres	Fibrobacter succinogenes subsp. succinogenes	YP_003248743.1	7 E-68	339/379
Bacteria	Lentisphaerae	Lentisphaera araneosa HTCC2155	ZP_01874755.1	1 E-67	335/379
Eukaryota	stramenopiles	Thalassiosira pseudonana CCMP1335	XP_002288882.1	2 E-67	339/379
Bacteria	Bacteroidetes	Prevotella veroralis F0319	ZP_05857581.1	4 E-67	333/379
Bacteria	Bacteroidetes	Porphyromonas uenonis 60-3	ZP_04055684.1	8 E-67	339/379
Eukaryota	Diplomonadida	Giardia intestinalis ATCC 50581	EES98261.1	2 E-65	348/379
Bacteria	Bacteroidetes	Porphyromonas endodontalis ATCC 35406	ZP_04388910.1	4 E-65	339/379
Bacteria	Bacteroidetes	Bacteroides caccae ATCC 43185	ZP_01961779.1	6 E-65	339/379
Bacteria	Bacteroidetes	Bacteroides plebeius DSM 17135	ZP_03209576.1	1 E-64	339/379
Eukaryota	Diplomonadida	Giardia lamblia ATCC 50803	XP_001706262.1	1 E-64	348/379
Eukaryota	Diplomonadida	Spironucleus barkhanus	AAM94644.1	3 E-64	334/379
Bacteria	Firmicutes	Streptococcus suis SC84	YP_003024703.1	5 E-64	337/379
Bacteria	Bacteroidetes	Bacteroides fragilis 3_1_12	ZP_05283912.1	5 E-64	339/379
Bacteria	Bacteroidetes	Bacteroides fragilis YCH46	YP_101273.1	5 E-64	339/379
Bacteria	Firmicutes	Streptococcus suis 98HAH33	YP_001200327.1	1 E-63	337/379
Bacteria	Bacteroidetes	Bacteroides cellulosilyticus DSM 14838	ZP_03676796.1	2 E-63	339/379
Bacteria	Bacteroidetes	Bacteroides coprophilus DSM 18228	ZP_03643990.1	2 E-63	339/379

Bacteria	Firmicutes	Catonella morbi ATCC 51271	ZP_04449221.1	2 E-63	332/379
Bacteria	Bacteroidetes	Prevotella sp. oral taxon	ZP_05916766.1	5 E-63	338/379
Bacteria	Bacteroidetes	Bacteroides coprocola DSM 17136	ZP_03010683.1	1 E-62	339/379
Bacteria	Bacteroidetes	Bacteroides intestinalis DSM 17393	ZP_03012533.1	1 E-62	339/379
Bacteria	Bacteroidetes	Bacteroides eggerthii DSM 20697	ZP_03457286.1	2 E-62	339/379
Bacteria	Firmicutes	Lactobacillus casei BL23	YP_001988132.1	8 E-62	333/379
Bacteria	Firmicutes	Acidaminococcus sp. D21	ZP_03929000.1	8 E-62	332/379
Bacteria	Firmicutes	Lactobacillus casei ATCC 334	YP_807238.1	1 E-61	333/379
Bacteria	Bacteroidetes	Porphyromonas gingivalis ATCC 33277	YP_001929196.1	1 E-61	339/379
Bacteria	Firmicutes	Streptococcus uberis 0140J	YP_002562336.1	2 E-61	336/379
Bacteria	Bacteroidetes	Bacteroides stercoris ATCC 43183	ZP_02437461.1	2 E-61	339/379
Bacteria	Bacteroidetes	Porphyromonas gingivalis W83	NP_905469.1	4 E-61	339/379
Bacteria	Firmicutes	Clostridium butyricum 5521	ZP_02950845.1	4 E-61	331/379
Bacteria	Bacteroidetes	Parabacteroides distasonis ATCC 8503	YP_001303142.1	4 E-61	339/379
Bacteria	Bacteroidetes	Bacteroides finegoldii DSM 17565	ZP_05414201.1	5 E-61	339/379
Bacteria	Firmicutes	Dorea formicigenerans ATCC 27755	ZP_02233973.1	6 E-61	336/379
Bacteria	Bacteroidetes	Bacteroides vulgatus ATCC 8482	YP_001297611.1	9 E-61	339/379
Bacteria	Bacteroidetes	Bacteroides ovatus ATCC 8483	ZP_02064147.1	1 E-60	339/379
Bacteria	Firmicutes	Ruminococcus lactaris ATCC 29176	ZP_03167699.1	1 E-60	337/379
Bacteria	Firmicutes	Lactobacillus acidophilus NCFM	YP_194199.1	1 E-60	332/379
Bacteria	Bacteroidetes	Bacteroides dorei DSM 17855	ZP_03298834.1	1 E-60	339/379
Bacteria	Firmicutes	Weissella paramesenteroides ATCC 33313	ZP_04783930.1	1 E-60	336/379
Bacteria	Bacteroidetes	Bacteroides thetaiotaomicron VPI-5482	NP_812803.1	1 E-60	339/379
Bacteria	Bacteroidetes	Bacteroides uniformis ATCC 8492	ZP_02071674.1	2 E-60	339/379
Bacteria	Firmicutes	Ruminococcus gnavus ATCC 29149	ZP_02042588.1	2 E-60	336/379
Bacteria	Firmicutes	Clostridium sp. L2-50	ZP_02073659.1	3 E-60	332/379
Bacteria	Firmicutes	Clostridium botulinum E3 str.	YP_001920416.1	3 E-60	331/379
Bacteria	Firmicutes	Lactobacillus rhamnosus Lc 705	YP_003174718.1	3 E-60	339/379
Bacteria	Firmicutes	Lactobacillus rhamnosus GG	YP_003171774.1	4 E-60	339/379
Bacteria	Firmicutes	Lactobacillus rhamnosus HN001	ZP_03212470.1	4 E-60	339/379
Bacteria	Firmicutes	Dorea longicatena DSM 13814	ZP_01995906.1	4 E-60	336/379
Bacteria	Firmicutes	Coprococcus eutactus ATCC 27759	ZP_02207731.1	5 E-60	337/379
Bacteria	Firmicutes	Lactobacillus fermentum IFO 3956	YP_001843738.1	7 E-60	336/379
Bacteria	Firmicutes	Lactobacillus fermentum 28-3-CHN	ZP_05864040.1	8 E-60	332/379
Bacteria	Firmicutes	Clostridium botulinum E1 str.	ZP_04820786.1	9 E-60	331/379
Bacteria	Firmicutes	Clostridium kluyveri DSM 555	YP_001396981.1	9 E-60	331/379
Bacteria	Firmicutes	Streptococcus agalactiae 2603V/R	NP_688161.1	9 E-60	337/379
Bacteria	Firmicutes	Streptococcus equi subsp. equi	YP_002746615.1	1 E-59	336/379
Bacteria	Firmicutes	Lactobacillus fermentum ATCC 14931	ZP_03945176.1	1 E-59	336/379
Bacteria	Firmicutes	Clostridium hylemonae DSM 15053	ZP_03777639.1	1 E-59	336/379
Bacteria	Firmicutes	Eubacterium ventriosum ATCC 27560	ZP_02027110.1	1 E-59	332/379
Bacteria	Firmicutes	Streptococcus pneumoniae SP14-BS69	ZP_01828872.1	1 E-59	337/379
Bacteria	Firmicutes	Alkaliphilus oremlandii OhILAs	YP_001511965.1	2 E-59	330/379
Bacteria	Firmicutes	Lactobacillus rhamnosus LMS2-1	ZP_04441898.1	2 E-59	339/379
Bacteria	Firmicutes	Clostridium botulinum B str.	YP_001885286.1	2 E-59	331/379
Bacteria	Firmicutes	Streptococcus equi subsp. zooepidemicus	YP_002123508.1	2 E-59	336/379
Bacteria	Firmicutes	Lactobacillus fermentum ATCC 14931	ZP_03944438.1	2 E-59	332/379
Bacteria	Firmicutes	Eubacterium rectale ATCC 33656	YP_002938245.1	3 E-59	331/379
Bacteria	Firmicutes	Streptococcus pyogenes MGAS10750	YP_602299.1	3 E-59	336/379
Bacteria	Firmicutes	Lactobacillus fermentum IFO 3956	YP_001843163.1	3 E-59	332/379
Bacteria	Firmicutes	Streptococcus pyogenes MGAS315	NP_664430.1	4 E-59	336/379
Bacteria	Firmicutes	Streptococcus infantarius subsp. infantarius	ZP_02920272.1	4 E-59	336/379
Bacteria	Firmicutes	Streptococcus pyogenes MGAS10394	YP_060048.1	5 E-59	336/379
Bacteria	Bacteroidetes	Bacteroides pectinophilus ATCC 43243	ZP_03461448.1	5 E-59	331/379
Bacteria	Firmicutes	Streptococcus pyogenes MGAS9429	YP_596501.1	5 E-59	336/379
Bacteria	Firmicutes	Streptococcus pyogenes MGAS8232	NP_607111.1	5 E-59	336/379
Bacteria	Firmicutes	Streptococcus pneumoniae TIGR4	NP_345345.1	8 E-59	337/379
Bacteria	Firmicutes	Streptococcus mutans NN2025	BAH87872.1	1 E-58	336/379

Bacteria	Bacteroidetes	Parabacteroides merdae ATCC 43184	ZP_02032465.1	1 E-58	339/379
Bacteria	Firmicutes	Streptococcus mutans UA159	NP_721583.1	1 E-58	336/379
Bacteria	Firmicutes	Streptococcus pyogenes MGAS6180	YP_280161.1	1 E-58	336/379
Bacteria	Firmicutes	Streptococcus pyogenes str. Manfredo	YP_001128644.1	2 E-58	336/379
Bacteria	Firmicutes	Streptococcus pyogenes M1 GAS	NP_269106.1	2 E-58	336/379
Bacteria	Firmicutes	Streptococcus dysgalactiae subsp. equisimilis	YP_002996880.1	2 E-58	336/379
Bacteria	Firmicutes	Coprococcus comes ATCC 27758	ZP_03799418.1	3 E-58	336/379
Bacteria	Firmicutes	Streptococcus pneumoniae MLV-016	ZP_02721448.1	3 E-58	337/379
Bacteria	Firmicutes	Streptococcus pneumoniae SP3-BS71	ZP_01817854.1	3 E-58	337/379
Bacteria	Firmicutes	Clostridium beijerinckii NCIMB 8052	YP_001308183.1	4 E-58	331/379
Bacteria	Firmicutes	Clostridium nexile DSM 1787	ZP_03290999.1	5 E-58	331/379
Bacteria	Firmicutes	Enterococcus faecium 1,230,933	ZP_05657824.1	5 E-58	335/379
Bacteria	Firmicutes	Streptococcus pyogenes NZ131	YP_002285732.1	5 E-58	336/379
Bacteria	Firmicutes	Enterococcus faecium Com12	ZP_05675689.1	6 E-58	335/379
Bacteria	Bacteroidetes	Parabacteroides johnsonii DSM 18315	ZP_03476390.1	6 E-58	339/379
Bacteria	Firmicutes	Ruminococcus torques ATCC 27756	ZP_01968024.1	7 E-58	337/379
Bacteria	Firmicutes	Lactobacillus plantarum subsp. plantarum	ZP_04014758.1	7 E-58	330/379
Bacteria	Firmicutes	Clostridium perfringens D str.	ZP_02952506.1	7 E-58	330/379
Bacteria	Firmicutes	Clostridium perfringens str. 13	NP_562436.1	9 E-58	330/379
Bacteria	Firmicutes	Enterococcus faecium 1,141,733	ZP_05667194.1	9 E-58	335/379
Bacteria	Firmicutes	Enterococcus faecium TX1330	ZP_03981393.1	1 E-57	335/379
Bacteria	Firmicutes	Lactobacillus brevis subsp. gravesensis	ZP_03939444.1	1 E-57	336/379
Bacteria	Firmicutes	Streptococcus salivarius SK126	ZP_04062818.1	1 E-57	336/379
Bacteria	Firmicutes	Clostridium scindens ATCC 35704	ZP_02430945.1	2 E-57	336/379
Bacteria	Firmicutes	Veillonella dispar ATCC 17748	ZP_04599903.1	2 E-57	332/379
Bacteria	Firmicutes	Butyrivibrio crossotus DSM 2876	ZP_05792740.1	2 E-57	336/379
Bacteria	Firmicutes	Lactobacillus plantarum WCFS1	NP_785849.1	2 E-57	330/379
Bacteria	Firmicutes	Enterococcus gallinarum EG2	ZP_05649426.1	2 E-57	330/379
Bacteria	Firmicutes	Streptococcus thermophilus LMG 18311	YP_139111.1	2 E-57	336/379
Bacteria	Bacteroidetes	Prevotella tannerae ATCC 51259	ZP_05734541.1	3 E-57	338/379
Bacteria	Proteobacteria	Pasteurella multocida subsp. multocida	NP_245503.1	3 E-57	339/379
Bacteria	Firmicutes	Lactobacillus ultunensis DSM 16047	ZP_04011094.1	3 E-57	332/379
Bacteria	Firmicutes	Enterococcus casseliflavus EC30	ZP_05647547.1	4 E-57	331/379
Bacteria	Firmicutes	Ruminococcus sp. 5_1_39BFAA	ZP_04856911.1	4 E-57	337/379
Bacteria	Firmicutes	Streptococcus pneumoniae R6	NP_358352.1	5 E-57	337/379
Bacteria	Firmicutes	Veillonella parvula DSM 2008	ZP_03854909.1	5 E-57	332/379
Bacteria	Firmicutes	Selenomonas flueggei ATCC 43531	ZP_04659227.1	5 E-57	331/379
Eukaryota	Viridiplantae	Ostreococcus tauri	CAL51895.1	5 E-57	340/379
Bacteria	Firmicutes	Listeria innocua Clip11262	NP_470314.1	6 E-57	330/379
Bacteria	Firmicutes	Streptococcus sanguinis SK36	YP_001035179.1	7 E-57	332/379
Bacteria	Firmicutes	Lactococcus lactis subsp. lactis	NP_267444.1	7 E-57	337/379
Bacteria	Firmicutes	Eubacterium eligens ATCC 27750	YP_002931348.1	8 E-57	332/379
Bacteria	Proteobacteria	Pasteurella dagmatis ATCC 43325	ZP_05920652.1	1 E-56	331/379
Bacteria	Actinobacteria	Atopobium rimae ATCC 49626	ZP_03568909.1	1 E-56	334/379
Bacteria	Firmicutes	Listeria welshimeri serovar 6b	YP_849160.1	1 E-56	330/379
Bacteria	Firmicutes	Lactobacillus salivarius UCC118	YP_534967.1	1 E-56	339/379
Bacteria	Lentisphaerae	Victivallis vadensis ATCC BAA-548	ZP_01924407.1	1 E-56	330/379
Bacteria	Firmicutes	Listeria monocytogenes Finland 1988	ZP_03668999.1	1 E-56	330/379
Bacteria	Firmicutes	Ruminococcus obeum ATCC 29174	ZP_01963605.1	1 E-56	337/379
Bacteria	Firmicutes	Mitsuokella multacida DSM 20544	ZP_05893297.1	2 E-56	332/379
Bacteria	Firmicutes	Listeria monocytogenes FSL N3-165	ZP_05232611.1	2 E-56	330/379
Bacteria	Firmicutes	Listeria monocytogenes str. 1/2a	ZP_00233865.1	2 E-56	330/379
Bacteria	Firmicutes	Clostridium botulinum Bf	ZP_02618791.1	3 E-56	331/379
Bacteria	Firmicutes	Blautia hansenii DSM 20583	ZP_05855136.1	3 E-56	337/379
Bacteria	Firmicutes	Clostridium sp. SS2/1	ZP_02437662.1	3 E-56	332/379
Bacteria	Firmicutes	Lactobacillus brevis subsp. gravesensis	ZP_03939445.1	4 E-56	334/379
Bacteria	Firmicutes	Blautia hydrogenotrophica DSM 10507	ZP_03781908.1	4 E-56	332/379
Bacteria	Actinobacteria	Slackia heliotrinireducens DSM 20476	YP_003143671.1	4 E-56	339/379

Bacteria	Firmicutes	Lactobacillus hilgardii ATCC 8290	ZP_03954111.1	4 E-56	334/379
Bacteria	Firmicutes	Selenomonas sputigena ATCC 35185	ZP_05898178.1	5 E-56	331/379
Bacteria	Firmicutes	Listeria grayi DSM 20601	ZP_04443874.1	5 E-56	336/379
Bacteria	Firmicutes	Listeria monocytogenes str. 4b	YP_013600.1	6 E-56	330/379
Bacteria	Firmicutes	Listeria monocytogenes EGD-e	NP_464503.1	7 E-56	330/379
Bacteria	Firmicutes	Clostridium sporogenes ATCC 15579	ZP_02994489.1	8 E-56	331/379
Bacteria	Firmicutes	Listeria monocytogenes HCC23	YP_002350600.1	9 E-56	330/379
Bacteria	Firmicutes	Lactobacillus helveticus DPC 4571	YP_001577707.1	1 E-55	332/379
Bacteria	Firmicutes	Listeria monocytogenes FSL J1-208	ZP_05295485.1	1 E-55	330/379
Bacteria	Firmicutes	Lactococcus lactis subsp. cremoris	YP_809347.1	1 E-55	337/379
Bacteria	Firmicutes	Clostridium botulinum A2 str.	YP_002803268.1	1 E-55	331/379
Bacteria	Firmicutes	Lactobacillus helveticus CNRZ32	ABH11629.1	1 E-55	332/379
Bacteria	Firmicutes	Clostridium botulinum NCTC 2916	ZP_02612967.1	1 E-55	331/379
Bacteria	Firmicutes	Lactobacillus helveticus DSM 20075	ZP_05751677.1	2 E-55	332/379
Bacteria	Firmicutes	Eubacterium hallii DSM 3353	ZP_03717099.1	2 E-55	332/379
Bacteria	Firmicutes	Lactococcus lactis subsp. cremoris	YP_001032494.1	2 E-55	337/379
Bacteria	Firmicutes	Blautia hydrogenotrophica DSM 10507	ZP_03781116.1	2 E-55	332/379
Bacteria	Firmicutes	Streptococcus pyogenes MGAS2096	YP_600381.1	2 E-55	326/379
Bacteria	Firmicutes	Clostridium acetobutylicum ATCC 824	NP_348107.1	3 E-55	332/379
Bacteria	Firmicutes	Granulicatella elegans ATCC 700633	ZP_05852290.1	4 E-55	330/379
Bacteria	Firmicutes	Clostridium carboxidivorans P7	ZP_05395131.1	4 E-55	331/379
Bacteria	Firmicutes	Streptococcus gordonii str. Challis	YP_001450522.1	5 E-55	337/379
Bacteria	Firmicutes	Alkaliphilus metalliredigens QYMF	YP_001321121.1	5 E-55	331/379
Bacteria	Firmicutes	Clostridium botulinum Ba4 str.	YP_002861752.1	6 E-55	332/379
Bacteria	Proteobacteria	Haemophilus somnus 2336	YP_001785097.1	6 E-55	331/379
Bacteria	Proteobacteria	Haemophilus somnus 129PT	YP_718705.1	6 E-55	331/379
Eukaryota	Viridiplantae	Micromonas sp. RCC299	XP_002504832.1	7 E-55	335/379
Bacteria	Firmicutes	Clostridiales bacterium 1_7_47FAA	ZP_04667878.1	8 E-55	331/379
Bacteria	Proteobacteria	Pseudomonas fluorescens Pf-5	YP_259637.1	1 E-54	332/379
Bacteria	Proteobacteria	Mannheimia succiniciproducens MBEL55E	YP_088088.1	1 E-54	336/379
Bacteria	Proteobacteria	Pseudomonas syringae pv. syringae	YP_234240.1	2 E-54	332/379
Bacteria	Firmicutes	Clostridium sp. 7_2_43FAA	ZP_05131579.1	2 E-54	331/379
Bacteria	Firmicutes	Oenococcus oeni PSU-1	YP_810833.1	3 E-54	335/379
Bacteria	Firmicutes	Clostridium bolteae ATCC BAA-613	ZP_02087401.1	3 E-54	331/379
Bacteria	Proteobacteria	Pseudomonas syringae pv. phaseolicola	YP_273484.1	6 E-54	332/379
Bacteria	Proteobacteria	Pseudomonas syringae pv. tabaci	ZP_05641351.1	6 E-54	332/379
Bacteria	Firmicutes	Oenococcus oeni ATCC BAA-1163	ZP_01544150.1	6 E-54	335/379
Bacteria	Proteobacteria	Pseudomonas entomophila L48	YP_607723.1	1 E-53	331/379
Bacteria	Proteobacteria	Pseudomonas syringae pv. tomato	NP_791159.1	1 E-53	332/379
Eukaryota	Parabasalidea	Trichomonas vaginalis G3	XP_001324767.1	2 E-53	332/379
Bacteria	Proteobacteria	Pseudomonas syringae pv. tomato	ZP_03396470.1	2 E-53	332/379
Bacteria	Actinobacteria	Cryptobacterium curtum DSM 15641	YP_003151251.1	2 E-53	339/379
Bacteria	Firmicutes	Shuttleworthia satelles DSM 14600	ZP_04454450.1	2 E-53	336/379
Eukaryota	Parabasalidea	Trichomonas vaginalis G3	XP_001326576.1	3 E-53	332/379
Bacteria	Actinobacteria	Atopobium parvulum DSM 20469	YP_003180090.1	4 E-53	338/379
Bacteria	Proteobacteria	Pseudomonas putida GB-1	YP_001668652.1	6 E-53	331/379
Bacteria	Bacteroidetes	Alistipes putredinis DSM 17216	ZP_02425972.1	7 E-53	336/379
Bacteria	Firmicutes	Oribacterium sp. oral taxon	ZP_05795913.1	8 E-53	331/379
Bacteria	Proteobacteria	Pseudomonas putida W619	YP_001749279.1	8 E-53	331/379
Bacteria	Proteobacteria	Pseudomonas putida F1	YP_001267587.1	1 E-52	331/379
Bacteria	Firmicutes	Oribacterium sinus F0268	ZP_03992638.1	1 E-52	332/379
Bacteria	Proteobacteria	Pseudomonas putida KT2440	NP_745648.1	1 E-52	331/379
Bacteria	Firmicutes	Lactobacillus delbrueckii subsp. bulgaricus	YP_619170.1	2 E-52	331/379
Bacteria	Firmicutes	Listeria monocytogenes FSL J2-064	ZP_05276367.1	2 E-52	314/379
Bacteria	Firmicutes	Lactobacillus delbrueckii subsp. bulgaricus	YP_813186.1	2 E-52	331/379
Eukaryota	Viridiplantae	Micromonas pusilla CCMP1545	EEH55547.1	3 E-52	330/379
Bacteria	Firmicutes	Abiotrophia defectiva ATCC 49176	ZP_04452152.1	3 E-52	331/379

AFUA_1G01690

Eukaryota	Fungi	Aspergillus fumigatus Af293	XP_749919.1	0.0	512/512
Eukaryota	Fungi	Neosartorya fischeri NRRL 181	XP_001265480.1	0.0	510/512
Eukaryota	Fungi	Aspergillus clavatus NRRL 1	XP_001269972.1	0.0	490/512
Eukaryota	Fungi	Aspergillus flavus NRRL3357	XP_002380808.1	0.0	505/512
Eukaryota	Fungi	Aspergillus terreus NIH2624	XP_001212071.1	0.0	481/512
Eukaryota	Fungi	Aspergillus nidulans FGSC A4	XP_682653.1	0.0	499/512
Eukaryota	Fungi	Aspergillus niger CBS 513.88	XP_001390153.1	0.0	503/512
Eukaryota	Fungi	Aspergillus niger CBS 513.88	XP_001388497.1	0.0	469/512
Eukaryota	Fungi	Aspergillus terreus NIH2624	XP_001208515.1	1 E-169	446/512
Eukaryota	Fungi	Microsporum canis CBS 113480	EEQ30150.1	1 E-169	491/512
Eukaryota	Fungi	Aspergillus fumigatus Af293	XP_747767.1	1 E-169	487/512
Eukaryota	Fungi	Aspergillus niger CBS 513.88	XP_001394678.1	1 E-169	490/512
Eukaryota	Fungi	Neosartorya fischeri NRRL 181	XP_001257501.1	1 E-167	478/512
Eukaryota	Fungi	Aspergillus niger CBS 513.88	XP_001400199.1	1 E-164	481/512
Eukaryota	Fungi	Phaeosphaeria nodorum SN15	XP_001792771.1	1 E-163	503/512
Eukaryota	Fungi	Aspergillus oryzae RIB40	XP_001827121.1	1 E-162	478/512
Eukaryota	Fungi	Aspergillus flavus NRRL3357	XP_002384360.1	1 E-157	495/512
Eukaryota	Fungi	Pyrenophora tritici-repentis Pt-1C-BFP	XP_001938589.1	1 E-157	495/512
Eukaryota	Fungi	Ajellomyces dermatitidis SLH14081	XP_002622526.1	1 E-138	499/512
Eukaryota	Fungi	Penicillium marneffei ATCC 18224	XP_002148355.1	1 E-136	489/512
Eukaryota	Fungi	Neosartorya fischeri NRRL 181	XP_001263094.1	1 E-136	474/512
Eukaryota	Fungi	Coccidioides posadasii C735 delta	EER29659.1	1 E-136	495/512
Eukaryota	Fungi	Ajellomyces capsulatus G186AR	EEH04429.1	1 E-136	484/512
Eukaryota	Fungi	Coccidioides immitis RS;	XP_001244958.1	1 E-135	495/512
Eukaryota	Fungi	Penicillium chrysogenum Wisconsin 54-1255	XP_002563873.1	1 E-134	495/512
Eukaryota	Fungi	Magnaporthe grisea 70-15	XP_361347.2	1 E-133	547/512
Eukaryota	Fungi	Botryotinia fuckeliana B05.10	XP_001554811.1	1 E-133	494/512
Eukaryota	Fungi	Paracoccidioides brasiliensis Pb03;	EEH19741.1	1 E-132	485/512
Eukaryota	Fungi	Paracoccidioides brasiliensis Pb01;	EEH38916.1	1 E-131	472/512
Eukaryota	Fungi	Uncinocarpus reesii 1704	XP_002542118.1	1 E-130	495/512
Eukaryota	Fungi	Magnaporthe grisea 70-15	XP_369556.1	1 E-128	481/512
Eukaryota	Fungi	Fusarium heterosporum Sordariomycetes;	AAV66104.1	1 E-128	504/512
Eukaryota	Fungi	Magnaporthe grisea 70-15	XP_362943.2	1 E-127	466/512
Eukaryota	Fungi	Aspergillus oryzae RIB40	XP_001821592.1	1 E-127	495/512
Eukaryota	Fungi	Aspergillus flavus NRRL3357	XP_002379858.1	1 E-127	472/512
Eukaryota	Fungi	Sclerotinia sclerotiorum 1980 UF-70	XP_001587438.1	1 E-126	506/512
Eukaryota	Fungi	Sclerotinia sclerotiorum 1980 UF-70	XP_001585586.1	1 E-125	514/512
Eukaryota	Fungi	Magnaporthe grisea 70-15	XP_364111.1	1 E-124	507/512
Eukaryota	Fungi	Botryotinia fuckeliana B05.10	XP_001559255.1	1 E-123	484/512
Eukaryota	Fungi	Podospora anserina DSM 980	XP_001904540.1	1 E-123	475/512
Eukaryota	Fungi	Talaromyces stipitatus ATCC 10500	XP_002480829.1	1 E-119	468/512
Eukaryota	Fungi	Ajellomyces capsulatus NAm1	XP_001537012.1	1 E-117	447/512
Eukaryota	Fungi	Aspergillus clavatus NRRL 1	XP_001274959.1	1 E-116	488/512
Eukaryota	Fungi	Microsporum canis CBS 113480	EEQ31785.1	1 E-115	443/512
Eukaryota	Fungi	Aspergillus terreus NIH2624	XP_001211376.1	1 E-114	499/512
Eukaryota	Fungi	Aspergillus clavatus NRRL 1	XP_001274843.1	1 E-112	505/512
Eukaryota	Fungi	Penicillium marneffei ATCC 18224	XP_002149824.1	1 E-111	460/512
Eukaryota	Fungi	Talaromyces stipitatus ATCC 10500	XP_002482834.1	1 E-111	468/512
Eukaryota	Fungi	Penicillium marneffei ATCC 18224	XP_002150795.1	1 E-111	513/512
Eukaryota	Fungi	Talaromyces stipitatus ATCC 10500	XP_002483325.1	1 E-110	510/512
Eukaryota	Fungi	Aspergillus niger CBS 513.88	XP_001400739.1	1 E-110	500/512
Eukaryota	Fungi	Debaryomyces hansenii	Q9Y758.1	1 E-109	483/512
Eukaryota	Fungi	Neosartorya fischeri NRRL 181	XP_001262425.1	1 E-109	509/512
Eukaryota	Fungi	Pichia guilliermondii ATCC 6260	XP_001482092.1	1 E-109	483/512
Eukaryota	Fungi	Aspergillus fumigatus Af293	XP_746567.1	1 E-109	509/512
Eukaryota	Fungi	Aspergillus oryzae RIB40	XP_001825995.1	1 E-108	505/512

Eukaryota	Fungi	Aspergillus flavus NRRL3357	XP_002377641.1	1 E-108	505/512
Eukaryota	Fungi	Penicillium chrysogenum Wisconsin 54-1255	XP_002560027.1	1 E-107	465/512
Eukaryota	Fungi	Coccidioides posadasii C735 delta	EER24493.1	1 E-107	501/512
Eukaryota	Fungi	Phaeosphaeria nodorum SN15	XP_001799910.1	1 E-107	470/512
Eukaryota	Fungi	Candida maltosa	P24458.1	1 E-106	500/512
Eukaryota	Fungi	Yarrowia lipolytica	BAA31433.1	1 E-106	516/512
Eukaryota	Fungi	Yarrowia lipolytica CLIB122	XP_504406.1	1 E-106	516/512
Eukaryota	Fungi	Candida albicans SC5314	XP_718670.1	1 E-105	477/512
Eukaryota	Fungi	Aspergillus nidulans FGSC A4	XP_664735.1	1 E-105	447/512
Eukaryota	Fungi	Candida maltosa	CAA36197.1	1 E-105	500/512
Eukaryota	Fungi	Candida maltosa	P16496.3	1 E-105	500/512
Eukaryota	Fungi	Candida maltosa	AAA34320.1	1 E-105	503/512
Eukaryota	Fungi	Candida dubliniensis CD36	XP_002421126.1	1 E-104	509/512
Eukaryota	Fungi	Candida albicans SC5314	XP_717999.1	1 E-104	510/512
Eukaryota	Fungi	Lodderomyces elongisporus NRRL YB-4239	XP_001524144.1	1 E-104	481/512
Eukaryota	Fungi	Pichia stipitis CBS 6054	XP_001383817.1	1 E-104	481/512
Eukaryota	Fungi	Debaryomyces hansenii CBS767	XP_457792.1	1 E-103	506/512
Eukaryota	Fungi	Candida dubliniensis CD36	XP_002421627.1	1 E-103	477/512
Eukaryota	Fungi	Candida maltosa	CAA39366.1	1 E-103	479/512
Eukaryota	Fungi	Verticillium albo-atrum VaMs.102	EEY16917.1	1 E-103	458/512
Eukaryota	Fungi	Candida maltosa	Q12589.1	1 E-103	503/512
Eukaryota	Fungi	Candida tropicalis	AAO73952.1	1 E-102	505/512
Eukaryota	Fungi	Penicillium marneffei ATCC 18224	XP_002152088.1	1 E-102	489/512
Eukaryota	Fungi	Nectria haematococca mpVI 77-13-4	EEU46759.1	1 E-102	473/512
Eukaryota	Fungi	Penicillium chrysogenum Wisconsin 54-1255	XP_002565827.1	1 E-102	512/512
Eukaryota	Fungi	Candida tropicalis MYA-3404	XP_002548823.1	1 E-102	422/512
Eukaryota	Fungi	Candida tropicalis	AAO73961.1	1 E-102	441/512
Eukaryota	Fungi	Aspergillus flavus NRRL3357	XP_002377989.1	1 E-101	511/512
Eukaryota	Fungi	Aspergillus oryzae RIB40	XP_001826299.1	1 E-101	511/512
Eukaryota	Fungi	Candida tropicalis	AAO73960.1	1 E-101	440/512
Eukaryota	Fungi	Aspergillus flavus NRRL3357	XP_002378813.1	1 E-100	493/512
Eukaryota	Fungi	Aspergillus fumigatus A1163	EDP54484.1	1 E-100	507/512
Eukaryota	Fungi	Debaryomyces hansenii	Q9Y757.2	1 E-100	477/512
Eukaryota	Fungi	Candida maltosa	Q12588.1	1 E-100	462/512
Eukaryota	Fungi	Aspergillus fumigatus Af293	XP_755288.1	1 E-100	507/512
Eukaryota	Fungi	Yarrowia lipolytica CLIB122	XP_504857.1	1 E-100	506/512
Eukaryota	Fungi	Pichia stipitis CBS 6054	XP_001383710.2	1 E-100	497/512
Eukaryota	Fungi	Pichia guilliermondii ATCC 6260	XP_001485567.1	1 E-100	477/512
Eukaryota	Fungi	Lodderomyces elongisporus NRRL YB-4239	XP_001527524.1	1 E-100	474/512
Eukaryota	Fungi	Candida tropicalis MYA-3404	XP_002548428.1	1 E-100	502/512
Eukaryota	Fungi	Yarrowia lipolytica CLIB122	XP_500856.1	1 E-100	485/512
Eukaryota	Fungi	Nectria haematococca mpVI 77-13-4	EEU46967.1	1 E-100	457/512
Eukaryota	Fungi	Neosartorya fischeri NRRL 181	XP_001260447.1	1 E-100	507/512
Eukaryota	Fungi	Botryotinia fuckeliana B05.10	XP_001552987.1	1 E-100	435/512
Eukaryota	Fungi	Candida tropicalis	AAO73958.1	1 E-100	506/512
Eukaryota	Fungi	Aspergillus clavatus NRRL 1	XP_001267871.1	1 E-100	503/512
Eukaryota	Fungi	Candida dubliniensis CD36	XP_002422222.1	1 E-99	510/512
Eukaryota	Fungi	Pichia stipitis CBS 6054	XP_001383636.2	2 E-99	455/512
Eukaryota	Fungi	Candida tropicalis MYA-3404	XP_002546279.1	2 E-99	424/512
Eukaryota	Fungi	Yarrowia lipolytica CLIB122	XP_500560.1	2 E-99	502/512
Eukaryota	Fungi	Lodderomyces elongisporus NRRL YB-4239	XP_001525578.1	3 E-99	500/512
Eukaryota	Fungi	Candida tropicalis	AAO73959.1	4 E-99	471/512
Eukaryota	Fungi	Phaeosphaeria nodorum SN15	XP_001806478.1	5 E-99	498/512
Eukaryota	Fungi	Gibberella zeae PH-1	XP_388496.1	2 E-98	452/512
Eukaryota	Fungi	Yarrowia lipolytica CLIB122	XP_500402.1	3 E-98	524/512
Eukaryota	Fungi	Candida tropicalis MYA-3404	XP_002548818.1	4 E-98	450/512
Eukaryota	Fungi	Debaryomyces hansenii	CAG88381.2	9 E-98	503/512
Eukaryota	Fungi	Candida tropicalis	P30608.1	1 E-97	424/512

Eukaryota	Fungi	Debaryomyces hansenii CBS767	XP_460111.1	2 E-97	503/512
Eukaryota	Fungi	Candida maltosa	CAA39367.1	2 E-97	490/512
Eukaryota	Fungi	Lodderomyces elongisporus NRRL YB-4239	XP_001525381.1	2 E-97	487/512
Eukaryota	Fungi	Candida tropicalis	AAO73954.1	4 E-97	424/512
Eukaryota	Fungi	Yarrowia lipolytica CLIB122	XP_500097.1	4 E-97	518/512
Eukaryota	Fungi	Pyrenophora tritici-repentis Pt-1C-BFP	XP_001936677.1	5 E-97	503/512
Eukaryota	Fungi	Debaryomyces hansenii CBS767	XP_457727.1	6 E-97	486/512
Eukaryota	Fungi	Debaryomyces hansenii CBS767	XP_460112.1	7 E-97	503/512
Eukaryota	Fungi	Candida albicans SC5314	XP_710174.1	1 E-96	474/512
Eukaryota	Fungi	Candida maltosa	P16141.3	1 E-96	490/512
Eukaryota	Fungi	Debaryomyces hansenii	CAG85755.2	1 E-96	486/512
Eukaryota	Fungi	Sclerotinia sclerotiorum 1980 UF-70	XP_001593058.1	1 E-96	511/512
Eukaryota	Fungi	Aspergillus niger CBS 513.88	XP_001396435.1	1 E-96	511/512
Eukaryota	Fungi	Candida tropicalis	AAO73953.1	1 E-96	424/512
Eukaryota	Fungi	Candida albicans WO-1	EEQ43763.1	2 E-96	474/512
Eukaryota	Fungi	Candida tropicalis MYA-3404	XP_002546278.1	2 E-96	438/512
Eukaryota	Fungi	Candida albicans SC5314	XP_718669.1	3 E-96	508/512
Eukaryota	Fungi	Debaryomyces hansenii	CAG88382.2	3 E-96	476/512
Eukaryota	Fungi	Yarrowia lipolytica CLIB122	XP_501148.1	4 E-96	506/512
Eukaryota	Fungi	Podospora anserina DSM 980	XP_001910644.1	5 E-96	464/512
Eukaryota	Fungi	Candida tropicalis	AAA34354.1	6 E-96	438/512
Eukaryota	Fungi	Pichia guilliermondii ATCC 6260	EDK41572.2	7 E-96	504/512
Eukaryota	Fungi	Candida tropicalis	P10615.3	9 E-96	438/512
Eukaryota	Fungi	Penicillium chrysogenum Wisconsin 54-1255	XP_002568429.1	1 E-95	469/512
Eukaryota	Fungi	Microsporum canis CBS 113480	EEQ29918.1	1 E-95	443/512
Eukaryota	Fungi	Candida albicans	CAA75058.1	1 E-95	474/512
Eukaryota	Fungi	Candida albicans WO-1	EEQ43157.1	1 E-95	508/512
Eukaryota	Fungi	Uncinocarpus reesii 1704	XP_002540883.1	3 E-95	469/512
Eukaryota	Fungi	Pichia guilliermondii ATCC 6260	XP_001482650.1	4 E-95	504/512
Eukaryota	Fungi	Lodderomyces elongisporus NRRL YB-4239	XP_001525577.1	4 E-95	480/512
Eukaryota	Fungi	Microsporum canis CBS 113480	EEQ33486.1	5 E-95	472/512
Eukaryota	Fungi	Candida tropicalis	prf1515252A	1 E-94	438/512
Eukaryota	Fungi	Pichia stipitis CBS 6054	XP_001383506.2	1 E-94	448/512
Eukaryota	Fungi	Botryotinia fuckeliana B05.10	XP_001552081.1	1 E-94	506/512
Eukaryota	Fungi	Debaryomyces hansenii	CAG88380.2	2 E-94	476/512
Eukaryota	Fungi	Debaryomyces hansenii CBS767	XP_460110.1	2 E-94	476/512
Eukaryota	Fungi	Candida tropicalis	AAO73955.1	4 E-94	423/512
Eukaryota	Fungi	Candida tropicalis	AAB24479.1	4 E-94	427/512
Eukaryota	Fungi	Candida tropicalis	P30609.1	4 E-94	427/512
Eukaryota	Fungi	Candida dubliniensis CD36	XP_002421628.1	6 E-94	500/512
Eukaryota	Fungi	Aspergillus terreus NIH2624	XP_001213081.1	6 E-94	485/512
Eukaryota	Fungi	Candida maltosa	Q12586.1	6 E-94	472/512
Eukaryota	Fungi	Yarrowia lipolytica CLIB122	XP_500855.1	7 E-94	519/512
Eukaryota	Fungi	Pichia guilliermondii ATCC 6260	XP_001483276.1	8 E-94	472/512
Eukaryota	Fungi	Pichia guilliermondii ATCC 6260	EDK39907.2	1 E-93	472/512
Eukaryota	Fungi	Candida tropicalis	P30610.1	2 E-93	416/512
Eukaryota	Fungi	Candida tropicalis	AAO73956.1	2 E-93	497/512
Eukaryota	Fungi	Candida apicola	P43083.1	7 E-93	470/512
Eukaryota	Fungi	Chaetomium globosum CBS 148.51	XP_001225874.1	8 E-93	508/512
Eukaryota	Fungi	Nectria haematococca mpVI 77-13-4	EEU34995.1	3 E-92	474/512
Eukaryota	Fungi	Aspergillus terreus NIH2624	XP_001216161.1	6 E-92	481/512
Eukaryota	Fungi	Starmerella bombicola	ACD75400.1	8 E-92	504/512
Eukaryota	Fungi	Aspergillus flavus NRRL3357	XP_002372373.1	1 E-91	517/512
Eukaryota	Fungi	Clavispora lusitaniae ATCC 42720	XP_002616743.1	2 E-91	473/512
Eukaryota	Fungi	Candida apicola	Q12573.1	4 E-91	490/512
Eukaryota	Fungi	Botryotinia fuckeliana B05.10	XP_001550556.1	4 E-91	487/512
Eukaryota	Fungi	Podospora anserina DSM 980	XP_001908613.1	5 E-91	470/512
Eukaryota	Fungi	Coccidioides immitis RS;	XP_001246560.1	9 E-91	461/512

Eukaryota	Fungi	Alternaria solani	BAD83681.1	9 E-91	470/512
Eukaryota	Fungi	Neurospora crassa	CAC10088.1	1 E-90	464/512
Eukaryota	Fungi	Aspergillus oryzae RIB40	XP_001817314.1	1 E-90	517/512
Eukaryota	Fungi	Microsporum canis CBS 113480	EEQ34899.1	4 E-90	486/512
Eukaryota	Fungi	Neurospora crassa OR74A	XP_958030.2	3 E-89	513/512
Eukaryota	Fungi	Graphium sp. ATCC 58400	AAR99474.1	5 E-89	490/512
Eukaryota	Fungi	Pyrenophora tritici-repentis Pt-1C-BFP	XP_001941811.1	8 E-89	476/512
Eukaryota	Fungi	Yarrowia lipolytica CLIB122	XP_501667.1	8 E-89	516/512
Eukaryota	Fungi	Aspergillus nidulans FGSC A4	XP_663661.1	2 E-88	507/512
Eukaryota	Fungi	Yarrowia lipolytica CLIB122	XP_504311.1	2 E-88	438/512
Eukaryota	Fungi	Aspergillus terreus NIH2624	XP_001215856.1	3 E-88	513/512
Eukaryota	Fungi	Magnaporthe grisea 70-15	XP_365075.1	3 E-88	479/512
Eukaryota	Fungi	Neosartorya fischeri NRRL 181	XP_001262753.1	2 E-87	472/512
Eukaryota	Fungi	Penicillium marneffei ATCC 18224	XP_002148942.1	2 E-87	482/512
Eukaryota	Fungi	Pichia stipitis CBS 6054	XP_001386440.2	4 E-87	411/512
Eukaryota	Fungi	Ajellomyces dermatitidis ER-3	EEQ89262.1	1 E-86	466/512
Eukaryota	Fungi	Candida tropicalis	AAO73957.1	2 E-86	452/512
Eukaryota	Fungi	Yarrowia lipolytica CLIB122	XP_500273.1	2 E-86	448/512
Eukaryota	Fungi	Penicillium marneffei ATCC 18224	XP_002145942.1	3 E-86	474/512
Eukaryota	Fungi	Starmerella bombicola	ACD75398.1	4 E-86	518/512
Eukaryota	Fungi	Neurospora crassa OR74A	XP_964653.2	6 E-86	427/512
Eukaryota	Fungi	Clavispora lusitaniae ATCC 42720	XP_002616857.1	6 E-86	441/512
Eukaryota	Fungi	Sclerotinia sclerotiorum 1980 UF-70	XP_001589398.1	7 E-86	430/512
Eukaryota	Fungi	Aspergillus fumigatus Af293	XP_748328.2	7 E-86	495/512
Eukaryota	Fungi	Ajellomyces dermatitidis SLH14081	XP_002628451.1	1 E-85	466/512
Eukaryota	Fungi	Magnaporthe grisea 70-15	XP_364102.1	5 E-85	535/512
Eukaryota	Fungi	Chaetomium globosum CBS 148.51	XP_001220831.1	5 E-85	483/512
Eukaryota	Fungi	Candida maltosa	Q12587.1	8 E-85	481/512
Eukaryota	Fungi	Candida maltosa	Q12585.1	9 E-85	490/512
Eukaryota	Fungi	Talaromyces stipitatus ATCC 10500	XP_002487181.1	8 E-84	477/512
Eukaryota	Fungi	Penicillium chrysogenum Wisconsin 54-1255	XP_002563618.1	1 E-83	467/512
Eukaryota	Fungi	Chaetomium globosum CBS 148.51	XP_001222615.1	1 E-83	462/512
Eukaryota	Fungi	Magnaporthe grisea 70-15	XP_001522438.1	2 E-83	481/512
Eukaryota	Fungi	Aspergillus fumigatus A1163	EDP48064.1	3 E-83	482/512
Eukaryota	Fungi	Pyrenophora tritici-repentis Pt-1C-BFP	XP_001934574.1	1 E-82	458/512
Eukaryota	Fungi	Uncinocarpus reesii 1704	XP_002541427.1	2 E-82	510/512
Eukaryota	Fungi	Penicillium chrysogenum Wisconsin 54-1255	XP_002562328.1	5 E-82	470/512
Eukaryota	Fungi	Nectria haematococca mpVI 77-13-4	EEU48386.1	5 E-82	482/512
Eukaryota	Fungi	Verticillium albo-atrum VaMs.102	EEY18833.1	6 E-82	426/512
Eukaryota	Fungi	Gibberella zeae PH-1	XP_381460.1	8 E-82	455/512
Eukaryota	Fungi	Aspergillus nidulans FGSC A4	XP_661521.1	9 E-82	453/512
Eukaryota	Fungi	Aspergillus oryzae RIB40	XP_001827526.1	1 E-81	445/512
Eukaryota	Fungi	Talaromyces stipitatus ATCC 10500	XP_002479350.1	2 E-81	477/512
Eukaryota	Fungi	Penicillium marneffei ATCC 18224	XP_002147083.1	2 E-81	509/512
Eukaryota	Fungi	Sclerotinia sclerotiorum 1980 UF-70	XP_001586739.1	7 E-81	490/512
Eukaryota	Fungi	Aspergillus nidulans FGSC A4	tpeCBF76609.1	4 E-80	492/512
Eukaryota	Fungi	Lodderomyces elongisporus NRRL YB-4239	XP_001528842.1	6 E-80	515/512
Eukaryota	Fungi	Aspergillus terreus NIH2624	XP_001214985.1	9 E-80	440/512
Eukaryota	Fungi	Penicillium marneffei ATCC 18224	XP_002149832.1	9 E-80	477/512
Eukaryota	Fungi	Aspergillus nidulans FGSC A4	XP_662462.1	1 E-79	448/512
Eukaryota	Fungi	Magnaporthe grisea 70-15	XP_365851.1	1 E-79	455/512
Eukaryota	Fungi	Aspergillus flavus NRRL3357	XP_002380252.1	3 E-79	499/512
Eukaryota	Fungi	Candida albicans WO-1	EEQ46951.1	5 E-79	482/512
Eukaryota	Fungi	Starmerella bombicola	ACD75402.1	7 E-79	517/512
Eukaryota	Fungi	Talaromyces stipitatus ATCC 10500	XP_002487227.1	1 E-78	516/512
Eukaryota	Fungi	Paracoccidioides brasiliensis Pb18;	EEH47609.1	4 E-78	476/512
Eukaryota	Fungi	Candida albicans SC5314	XP_721410.1	5 E-78	482/512
Eukaryota	Fungi	Candida dubliniensis CD36	XP_002421356.1	6 E-78	482/512

Eukaryota	Fungi	Paracoccidioides brasiliensis Pb01;	EEH38216.1	1 E-77	473/512
Eukaryota	Fungi	Paracoccidioides brasiliensis Pb03;	EEH19393.1	2 E-77	413/512
Eukaryota	Fungi	Microsporum canis CBS 113480	EEQ35829.1	2 E-77	464/512
Eukaryota	Fungi	Aspergillus fumigatus Af293	XP_746816.1	7 E-76	500/512
Eukaryota	Fungi	Uncinocarpus reesii 1704	XP_002541530.1	1 E-75	480/512
Eukaryota	Fungi	Coccidioides posadasii C735 delta	EER23898.1	5 E-75	485/512
Eukaryota	Fungi	Coccidioides immitis RS;	XP_001247332.1	6 E-75	485/512
Eukaryota	Fungi	Botryotinia fuckeliana B05.10	XP_001554305.1	8 E-75	464/512
Eukaryota	Fungi	Ajellomyces dermatitidis ER-3	EEQ92528.1	4 E-74	429/512

AFUA_1G01700

Eukaryota	Fungi	Aspergillus fumigatus Af293	XP_749920.1	0.0	1044/1044
Eukaryota	Fungi	Neosartorya fischeri NRRL 181	XP_001265479.1	0.0	1038/1044
Eukaryota	Fungi	Aspergillus clavatus NRRL 1	XP_001269971.1	0.0	1050/1044

AFUA_1G04130

Eukaryota	Fungi	Aspergillus fumigatus Af293	XP_750162.2	1 E-178	307/307
Eukaryota	Fungi	Neosartorya fischeri NRRL 181	XP_001265249.1	1 E-170	308/307
Eukaryota	Fungi	Aspergillus flavus NRRL3357	XP_002374393.1	1 E-134	295/307
Eukaryota	Fungi	Aspergillus oryzae RIB40	XP_001820051.1	1 E-133	295/307
Eukaryota	Fungi	Sclerotinia sclerotiorum 1980 UF-70	XP_001584850.1	7 E-86	302/307
Eukaryota	Fungi	Talaromyces stipitatus ATCC 10500	XP_002483842.1	2 E-64	256/307
Bacteria	Cyanobacteria	Synechococcus sp. PCC 7335	ZP_05039833.1	5 E-46	261/307
Bacteria	Cyanobacteria	Synechococcus sp. PCC 7335	ZP_05039833.1	2 E-37	250/307
Bacteria	Actinobacteria	Streptomyces hygroscopicus ATCC 53653	ZP_05512470.1	6 E-46	300/307
Bacteria	Cyanobacteria	Cyanothece sp. CCY0110	ZP_01730985.1	3 E-40	256/307
Bacteria	Firmicutes	Clostridium acetobutylicum ATCC 824	NP_149314.1	2 E-23	247/307
Bacteria	Firmicutes	Clostridium acetobutylicum ATCC 824	NP_149314.1	5 E-22	264/307
Bacteria	Firmicutes	Clostridium acetobutylicum ATCC 824	NP_149314.1	6 E-20	294/307
Eukaryota	Fungi	Phaeosphaeria nodorum SN15	XP_001792205.1	8 E-23	258/307
Bacteria	Proteobacteria	Hahella chejuensis KCTC 2396	YP_434647.1	6 E-21	267/307

AFUA_1G04400

Eukaryota	Fungi	Aspergillus fumigatus Af293	XP_750188.1	1 E-52	101/101
Eukaryota	Fungi	Neosartorya fischeri NRRL 181	XP_001265226.1	2 E-52	101/101
Eukaryota	Fungi	Aspergillus terreus NIH2624	XP_001212952.1	2 E-48	101/101
Eukaryota	Fungi	Aspergillus flavus NRRL3357	XP_002374420.1	4 E-48	101/101
Eukaryota	Fungi	Aspergillus clavatus NRRL 1	XP_001269737.1	2 E-46	108/101
Eukaryota	Fungi	Aspergillus niger CBS 513.88	XP_001388760.1	4 E-40	101/101
Eukaryota	Fungi	Talaromyces stipitatus ATCC 10500	XP_002483999.1	2 E-36	101/101
Eukaryota	Fungi	Penicillium marneffei ATCC 18224	XP_002150264.1	3 E-36	101/101
Eukaryota	Fungi	Sclerotinia sclerotiorum 1980 UF-70	XP_001592801.1	3 E-32	97/101
Eukaryota	Fungi	Botryotinia fuckeliana B05.10	XP_001552054.1	9 E-32	97/101
Eukaryota	Fungi	Coccidioides immitis RS;	XP_001245530.1	8 E-31	97/101
Eukaryota	Fungi	Ajellomyces capsulatus G186AR	EEH05961.1	6 E-30	98/101
Eukaryota	Fungi	Ajellomyces capsulatus NAm1	XP_001535999.1	1 E-29	98/101
Eukaryota	Fungi	Aspergillus nidulans FGSC A4	tpeCBF89443.1	5 E-28	86/101
Eukaryota	Fungi	Paracoccidioides brasiliensis Pb03;	EEH16319.1	4 E-26	95/101
Eukaryota	Fungi	Phaeosphaeria nodorum SN15	XP_001796749.1	4 E-25	83/101
Eukaryota	Fungi	Magnaporthe grisea 70-15	XP_364853.1	7 E-24	90/101
Eukaryota	Fungi	Uncinocarpus reesii 1704	XP_002583614.1	1 E-23	97/101
Eukaryota	Fungi	Podospora anserina DSM 980	XP_001904833.1	3 E-21	81/101
Eukaryota	Fungi	Microsporum canis CBS 113480	EEQ29375.1	3 E-21	95/101
Eukaryota	Fungi	Gibberella zeae PH-1	XP_390688.1	2 E-20	92/101
Eukaryota	Fungi	Chaetomium globosum CBS 148.51	XP_001228070.1	1 E-19	87/101

Eukaryota	Fungi	Verticillium albo-atrum VaMs.102	EEY16750.1	3 E-19	81/101
Eukaryota	Fungi	Neurospora crassa OR74A	XP_959829.1	5 E-19	86/101
Eukaryota	Fungi	Pichia pastoris GS115	XP_002490033.1	2 E-13	83/101
Eukaryota	Fungi	Ajellomyces dermatitidis SLH14081	XP_002621605.1	5 E-13	97/101

AFUA_1G04410

Eukaryota	Fungi	Aspergillus fumigatus Af293	XP_750189.1	0.0	519/519
Eukaryota	Fungi	Neosartorya fischeri NRRL 181	XP_001265225.1	0.0	447/519
Eukaryota	Fungi	Aspergillus clavatus NRRL 1	XP_001269738.1	0.0	510/519
Eukaryota	Fungi	Aspergillus flavus NRRL3357	XP_002374421.1	0.0	510/519
Eukaryota	Fungi	Aspergillus oryzae RIB40	XP_001820028.1	0.0	510/519
Eukaryota	Fungi	Aspergillus nidulans FGSC A4	XP_658062.1	0.0	488/519
Eukaryota	Fungi	Aspergillus terreus NIH2624	XP_001212951.1	0.0	456/519
Eukaryota	Fungi	Aspergillus niger CBS 513.88	XP_001388761.1	1 E-170	474/519
Eukaryota	Fungi	Penicillium chrysogenum Wisconsin 54-1255	XP_002561202.1	1 E-152	491/519
Eukaryota	Fungi	Penicillium marneffei ATCC 18224	XP_002150263.1	1 E-149	475/519
Eukaryota	Fungi	Talaromyces stipitatus ATCC 10500	XP_002484000.1	1 E-148	485/519
Eukaryota	Fungi	Ajellomyces dermatitidis ER-3	EEQ92345.1	1 E-143	474/519
Eukaryota	Fungi	Paracoccidioides brasiliensis Pb03;	EEH16318.1	1 E-142	535/519
Eukaryota	Fungi	Paracoccidioides brasiliensis Pb18;	EEH43032.1	1 E-142	536/519
Eukaryota	Fungi	Paracoccidioides brasiliensis Pb01;	EEH35953.1	1 E-139	534/519
Eukaryota	Fungi	Coccidioides posadasii C735 delta	EER29232.1	1 E-138	511/519
Eukaryota	Fungi	Uncinocarpus reesii 1704	XP_002583615.1	1 E-134	530/519
Eukaryota	Fungi	Coccidioides immitis RS;	XP_001245529.1	1 E-134	511/519
Eukaryota	Fungi	Ajellomyces dermatitidis SLH14081	XP_002621607.1	1 E-131	435/519
Eukaryota	Fungi	Microsporum canis CBS 113480	EEQ29374.1	1 E-130	471/519
Eukaryota	Fungi	Podospora anserina DSM 980	XP_001903520.1	1 E-113	510/519
Eukaryota	Fungi	Sclerotinia sclerotiorum 1980 UF-70	XP_001593225.1	1 E-107	534/519
Eukaryota	Fungi	Nectria haematococca mpVI 77-13-4	EEU42326.1	1 E-104	438/519
Eukaryota	Fungi	Neurospora crassa OR74A	XP_963573.2	1 E-104	518/519
Eukaryota	Fungi	Botryotinia fuckeliana B05.10	XP_001559798.1	1 E-103	484/519
Eukaryota	Fungi	Gibberella zeae PH-1	XP_390925.1	1 E-100	496/519
Eukaryota	Fungi	Pyrenophora tritici-repentis Pt-1C-BFP	XP_001931775.1	6 E-87	504/519
Eukaryota	Fungi	Phaeosphaeria nodorum SN15	XP_001804540.1	4 E-85	539/519

AFUA_1G05200

Eukaryota	Fungi	Aspergillus fumigatus Af293	XP_750269.2	0.0	1051/1051
Eukaryota	Fungi	Neosartorya fischeri NRRL 181	XP_001265147.1	0.0	1067/1051
Eukaryota	Fungi	Aspergillus niger CBS 513.88	XP_001388870.1	0.0	1049/1051
Eukaryota	Fungi	Aspergillus clavatus NRRL 1	XP_001269642.1	0.0	1043/1051
Eukaryota	Fungi	Aspergillus terreus NIH2624	XP_001211766.1	0.0	1038/1051
Eukaryota	Fungi	Aspergillus oryzae RIB40	XP_001819951.1	0.0	1036/1051
Eukaryota	Fungi	Aspergillus nidulans FGSC A4	tpeCBF84105.1	0.0	1033/1051
Eukaryota	Fungi	Aspergillus nidulans FGSC A4	XP_660347.1	0.0	1051/1051
Eukaryota	Fungi	Penicillium marneffei ATCC 18224	XP_002150165.1	0.0	1061/1051
Eukaryota	Fungi	Talaromyces stipitatus ATCC 10500	XP_002484098.1	0.0	1051/1051
Eukaryota	Fungi	Coccidioides posadasii C735 delta	EER29315.1	0.0	1029/1051
Eukaryota	Fungi	Coccidioides immitis RS;	XP_001245432.1	0.0	1025/1051
Eukaryota	Fungi	Uncinocarpus reesii 1704	XP_002583711.1	0.0	1036/1051
Eukaryota	Fungi	Microsporum canis CBS 113480	EEQ33191.1	0.0	1083/1051
Eukaryota	Fungi	Ajellomyces dermatitidis SLH14081	XP_002620617.1	0.0	1062/1051
Eukaryota	Fungi	Ajellomyces dermatitidis ER-3	EEQ86346.1	0.0	1068/1051
Eukaryota	Fungi	Paracoccidioides brasiliensis Pb01;	EEH37894.1	0.0	1060/1051
Eukaryota	Fungi	Aspergillus flavus NRRL3357	XP_002374513.1	0.0	975/1051
Eukaryota	Fungi	Ajellomyces capsulatus G186AR	EEH11269.1	0.0	1048/1051
Eukaryota	Fungi	Ajellomyces capsulatus NAm1	XP_001541471.1	0.0	1040/1051

Eukaryota	Fungi	Paracoccidioides brasiliensis Pb03;	EEH19097.1	0.0	970/1051
Eukaryota	Fungi	Paracoccidioides brasiliensis Pb18;	EEH47932.1	0.0	972/1051
Eukaryota	Fungi	Pyrenophora tritici-repentis Pt-1C-BFP	XP_001937483.1	0.0	1091/1051
Eukaryota	Fungi	Sclerotinia sclerotiorum 1980 UF-70	XP_001586581.1	0.0	1033/1051
Eukaryota	Fungi	Nectria haematococca mpVI 77-13-4	EEU46595.1	0.0	991/1051
Eukaryota	Fungi	Gibberella zeae PH-1	XP_387413.1	0.0	1055/1051
Eukaryota	Fungi	Chaetomium globosum CBS 148.51	XP_001221846.1	0.0	951/1051
Eukaryota	Fungi	Magnaporthe grisea 70-15	XP_365972.1	0.0	1069/1051
Eukaryota	Fungi	Podospora anserina DSM 980	XP_001908348.1	0.0	1052/1051
Eukaryota	Fungi	Neurospora crassa OR74A	XP_956151.2	0.0	1005/1051
Eukaryota	Fungi	Phaeosphaeria nodorum SN15	XP_001790980.1	0.0	957/1051
Eukaryota	Fungi	Ustilago maydis	Q4P358.2	0.0	950/1051
Eukaryota	Fungi	Malassezia globosa CBS 7966	XP_001729235.1	0.0	972/1051
Eukaryota	Fungi	Yarrowia lipolytica CLIB122	XP_505601.1	0.0	935/1051
Eukaryota	Fungi	Schizosaccharomyces pombe	NP_596379.1	0.0	926/1051
Eukaryota	Fungi	Debaryomyces hansenii	B5RUP5.1	0.0	857/1051
Eukaryota	Fungi	Pichia stipitis CBS 6054	EAZ63150.2	1 E-175	851/1051
Eukaryota	Fungi	Pichia stipitis CBS 6054	XP_001387173.1	1 E-175	851/1051
Eukaryota	Fungi	Candida albicans WO-1	EEQ41971.1	1 E-165	843/1051
Eukaryota	Fungi	Candida albicans SC5314	XP_711631.1	1 E-164	844/1051
Eukaryota	Fungi	Candida albicans SC5314	XP_711563.1	1 E-164	844/1051
Eukaryota	Viridiplantae	Physcomitrella patens subsp. patens	XP_001752885.1	1 E-143	1004/1051
Eukaryota	Metazoa	Nasonia vitripennis	XP_001605611.1	1 E-137	913/1051
Eukaryota	Metazoa	Hydra magnipapillata	XP_002165634.1	1 E-135	844/1051
Eukaryota	Viridiplantae	Arabidopsis thaliana	NP_192881.1	1 E-132	866/1051
Eukaryota	Metazoa	Strongylocentrotus purpuratus	XP_798957.2	1 E-131	884/1051
Eukaryota	Fungi	Kluyveromyces lactis NRRL Y-1140	XP_452849.1	1 E-129	884/1051
Eukaryota	Viridiplantae	Ricinus communis	XP_002512475.1	1 E-126	975/1051
Eukaryota	Fungi	Ashbya gossypii ATCC 10895	NP_982882.1	1 E-126	875/1051
Eukaryota	Metazoa	Drosophila yakuba	XP_002095950.1	1 E-126	982/1051
Eukaryota	Metazoa	Drosophila sechellia	B4I3P3.2	1 E-125	993/1051
Eukaryota	Metazoa	Drosophila erecta	XP_001978826.1	1 E-124	988/1051
Eukaryota	Viridiplantae	Populus trichocarpa	XP_002330181.1	1 E-122	930/1051
Eukaryota	Metazoa	Drosophila sechellia	XP_002038299.1	1 E-121	983/1051
Eukaryota	Fungi	Saccharomyces cerevisiae YJM789	EDN64692.1	1 E-119	923/1051
Eukaryota	Fungi	Saccharomyces cerevisiae	NP_009635.1	1 E-119	923/1051
Eukaryota	Fungi	Saccharomyces cerevisiae JAY291	EEU06931.1	1 E-118	923/1051
Eukaryota	Fungi	Saccharomyces cerevisiae AWRI1631	EDZ73807.1	1 E-118	923/1051
Eukaryota	Choanoflagellida	Monosiga brevicollis MX1	XP_001747825.1	1 E-112	873/1051
Eukaryota	Fungi	Vanderwaltozyma polyspora DSM 70294	XP_001644885.1	1 E-105	919/1051
Eukaryota	Metazoa	Schistosoma mansoni	XP_002571593.1	1 E-104	854/1051
Eukaryota	Metazoa	Brugia malayi	XP_001897252.1	8 E-97	845/1051
Eukaryota	Alveolata	Theileria parva strain Muguga	XP_765762.1	5 E-66	916/1051
Eukaryota	Alveolata	Theileria annulata strain Ankara	XP_954490.1	7 E-64	913/1051

AFUA_1G05850

Eukaryota	Fungi	Aspergillus fumigatus Af293	XP_750335.1	0.0	463/463
Eukaryota	Fungi	Neosartorya fischeri NRRL 181	XP_001265083.1	0.0	449/463
Eukaryota	Fungi	Aspergillus clavatus NRRL 1	XP_001269579.1	0.0	449/463
Eukaryota	Fungi	Aspergillus niger CBS 513.88	XP_001398819.1	0.0	459/463
Eukaryota	Fungi	Aspergillus flavus NRRL3357	XP_002383244.1	0.0	448/463
Eukaryota	Fungi	Aspergillus oryzae RIB40	XP_001816828.1	0.0	464/463
Eukaryota	Fungi	Penicillium chrysogenum Wisconsin 54-1255	XP_002558685.1	0.0	445/463
Eukaryota	Fungi	Aspergillus terreus NIH2624	XP_001212223.1	0.0	429/463
Eukaryota	Fungi	Penicillium marneffei ATCC 18224	XP_002144086.1	0.0	446/463
Eukaryota	Fungi	Talaromyces stipitatus ATCC 10500	XP_002480468.1	0.0	416/463
Eukaryota	Fungi	Aspergillus nidulans FGSC A4	XP_661709.1	0.0	453/463

Eukaryota	Fungi	Uncinocarpus reesii 1704	XP_002542552.1	0.0	448/463
Eukaryota	Fungi	Coccidioides immitis RS;	XP_001244439.1	0.0	449/463
Eukaryota	Fungi	Coccidioides posadasii C735 delta	EER26291.1	0.0	449/463
Eukaryota	Fungi	Ajellomyces dermatitidis ER-3	EEQ83774.1	1 E-179	446/463
Eukaryota	Fungi	Ajellomyces dermatitidis SLH14081	XP_002625161.1	1 E-179	446/463
Eukaryota	Fungi	Ajellomyces capsulatus G186AR	EEH04986.1	1 E-177	450/463
Eukaryota	Fungi	Microsporum canis CBS 113480	EEQ28048.1	1 E-159	450/463
Eukaryota	Fungi	Nectria haematococca mpVI 77-13-4	EEU46379.1	1 E-134	455/463
Bacteria	Proteobacteria	Mesorhizobium opportunistum WSM2075	ZP_05813616.1	1 E-134	436/463
Bacteria	Proteobacteria	Methylobacterium nodulans ORS 2060	YP_002497344.1	1 E-134	432/463
Bacteria	Proteobacteria	Oceanicola granulosus HTCC2516	ZP_01156758.1	1 E-131	443/463
Bacteria	Proteobacteria	Caulobacter sp. K31	YP_001684479.1	1 E-130	436/463
Eukaryota	Fungi	Gibberella zeae PH-1	XP_387171.1	1 E-129	452/463
Bacteria	Proteobacteria	Sinorhizobium meliloti 1021	NP_436628.1	1 E-129	443/463
Bacteria	Proteobacteria	Methylocella silvestris BL2	YP_002363968.1	1 E-128	423/463
Eukaryota	Fungi	Neurospora crassa	CAB88606.2	1 E-127	458/463
Eukaryota	Fungi	Chaetomium globosum CBS 148.51	XP_001225180.1	1 E-126	459/463
Bacteria	Proteobacteria	Phenylobacterium zucineum HLK1	YP_002132063.1	1 E-126	451/463
Bacteria	Proteobacteria	Sinorhizobium medicae WSM419	YP_001312789.1	1 E-126	429/463
Bacteria	Proteobacteria	Roseobacter sp. GAI101	ZP_05102450.1	1 E-124	427/463
Bacteria	Proteobacteria	Rhizobium leguminosarum bv. trifolii	YP_002979228.1	1 E-124	436/463
Bacteria	Proteobacteria	Burkholderia xenovorans LB400	YP_556490.1	1 E-123	430/463
Bacteria	Proteobacteria	Burkholderia graminis C4D1M	ZP_02886413.1	1 E-123	429/463
Eukaryota	Fungi	Podospora anserina DSM 980	XP_001911610.1	1 E-122	463/463
Eukaryota	Fungi	Neurospora crassa OR74A	XP_961723.2	1 E-120	436/463
Bacteria	Proteobacteria	Burkholderia multivorans ATCC 17616	YP_001946782.1	1 E-120	428/463
Bacteria	Proteobacteria	Burkholderia multivorans ATCC 17616	YP_001579097.1	1 E-120	428/463
Bacteria	Proteobacteria	Burkholderia multivorans CGD1	ZP_03584585.1	1 E-120	428/463
Bacteria	Proteobacteria	Burkholderia multivorans CGD2M	ZP_03572521.1	1 E-119	428/463
Bacteria	Proteobacteria	Erythrobacter sp. SD-21	ZP_01865005.1	1 E-118	423/463
Bacteria	Proteobacteria	Chelativorans sp. BNC1	YP_676064.1	1 E-118	423/463
Bacteria	Proteobacteria	Burkholderia dolosa AUO158	ZP_04945057.1	1 E-117	428/463
Bacteria	Proteobacteria	Rhizobium sp. NGR234	YP_002822788.1	1 E-114	423/463
Bacteria	Proteobacteria	Pseudomonas stutzeri A1501	YP_001173158.1	1 E-114	436/463
Bacteria	Proteobacteria	Beijerinckia indica subsp. indica	YP_001832074.1	1 E-113	432/463
Bacteria	Proteobacteria	Burkholderia sp. H160	ZP_03265624.1	1 E-113	439/463
Bacteria	Proteobacteria	Azotobacter vinelandii DJ	YP_002799118.1	1 E-113	426/463
Bacteria	Proteobacteria	Pseudomonas aeruginosa PA7	YP_001348719.1	1 E-112	434/463
Bacteria	Proteobacteria	Pseudomonas putida F1	YP_001267783.1	1 E-112	434/463
Bacteria	Proteobacteria	Pseudomonas putida KT2440	NP_745374.1	1 E-111	434/463
Bacteria	Proteobacteria	Methylobacterium sp. 4-46	YP_001767083.1	1 E-110	429/463
Bacteria	Proteobacteria	Bradyrhizobium japonicum USDA 110	NP_771291.1	1 E-110	429/463
Bacteria	Proteobacteria	Thiomicrospira crunogena XCL-2	YP_391338.1	1 E-110	436/463
Bacteria	Proteobacteria	Nitrobacter hamburgensis X14	YP_577196.1	1 E-108	448/463
Bacteria	Proteobacteria	Nitrobacter sp. Nb-311A	ZP_01045915.1	1 E-108	429/463
Bacteria	Proteobacteria	Rhizobium etli CFN 42	YP_471812.1	1 E-106	427/463
Bacteria	Proteobacteria	Rhizobium etli CIAT 652	YP_001984940.1	1 E-105	426/463
Bacteria	Proteobacteria	Ralstonia eutropha H16	YP_841068.1	1 E-104	415/463
Bacteria	Proteobacteria	Cupriavidus taiwanensis	YP_002008008.1	1 E-104	413/463
Bacteria	Verrucomicrobia	bacterium Ellin514	ZP_03626782.1	1 E-103	444/463
Bacteria	Candidatus	Protochlamydia amoebophila UWE25	YP_007850.1	1 E-102	425/463
Bacteria	Proteobacteria	Gluconacetobacter diazotrophicus PAl 5	YP_001604060.1	1 E-101	418/463
Bacteria	Proteobacteria	Gluconacetobacter diazotrophicus PAl 5	YP_002277059.1	1 E-101	413/463
Bacteria	Cyanobacteria	Nodularia spumigena CCY9414	ZP_01632263.1	3 E-98	426/463
Bacteria	Proteobacteria	Variovorax paradoxus S110	YP_002943882.1	3 E-96	442/463
Bacteria	Cyanobacteria	Cyanothece sp. PCC 7425	YP_002483369.1	1 E-94	443/463
Bacteria	Thermus	Deinococcus deserti VCD115	YP_002787868.1	1 E-92	426/463
Bacteria	Proteobacteria	Myxococcus xanthus DK 1622	YP_629089.1	4 E-88	412/463

Bacteria	Proteobacteria	Polaromonas naphthalenivorans CJ2	YP_982415.1	4 E-87	413/463
Bacteria	Actinobacteria	Rhodococcus sp. DK17	ABP48126.1	5 E-87	431/463
Bacteria	Proteobacteria	Methylobacterium nodulans ORS 2060	YP_002497601.1	5 E-86	418/463
Bacteria	Proteobacteria	Herminiimonas arsenicoxydans	YP_001100777.1	3 E-85	437/463
Bacteria	Proteobacteria	Polaromonas sp. JS666	YP_549210.1	2 E-84	413/463
Bacteria	Proteobacteria	Methylobacterium sp. 4-46	YP_001769728.1	4 E-83	422/463
Bacteria	Proteobacteria	Legionella drancourtii LLAP12	ZP_05111678.1	6 E-83	421/463
Bacteria	Proteobacteria	Burkholderia thailandensis E264	YP_441180.1	1 E-81	402/463
Bacteria	Proteobacteria	Sorangium cellulosum 'So ce	YP_001616080.1	2 E-81	447/463
Bacteria	Proteobacteria	Burkholderia thailandensis TXDOH	ZP_02372811.1	2 E-81	399/463
Bacteria	Proteobacteria	Nitrosospira multiformis ATCC 25196	YP_411859.1	7 E-81	416/463
Bacteria	Proteobacteria	Aromatoleum aromaticum EbN1	YP_160714.1	1 E-80	416/463
Bacteria	Proteobacteria	Burkholderia thailandensis MSMB43	ZP_02462436.1	9 E-80	399/463
Bacteria	Proteobacteria	Burkholderia phymatum STM815	YP_001863090.1	1 E-79	387/463
Bacteria	Proteobacteria	Stigmatella aurantiaca DW4/3-1	ZP_01467082.1	2 E-79	388/463
Eukaryota	Fungi	Postia placenta Mad-698-R	XP_002475658.1	3 E-79	481/463
Bacteria	Proteobacteria	Burkholderia oklahomensis C6786	ZP_02361783.1	7 E-79	399/463
Bacteria	Proteobacteria	Burkholderia oklahomensis EO147	ZP_02354589.1	9 E-79	399/463
Bacteria	Actinobacteria	Mycobacterium vanbaalenii PYR-1	YP_952233.1	1 E-78	432/463
Bacteria	Actinobacteria	Mycobacterium gilvum PYR-GCK	YP_001136242.1	7 E-78	455/463
Bacteria	Bacteroidetes	Candidatus Amoebophilus asiaticus 5a2	YP_001958470.1	3 E-77	401/463
Bacteria	Firmicutes	Bacillus sp. SG-1	ZP_01860099.1	4 E-77	399/463
Bacteria	Actinobacteria	Mycobacterium marinum M	YP_001851757.1	2 E-75	419/463
Bacteria	Actinobacteria	Mycobacterium kansasii ATCC 12478	ZP_04750176.1	3 E-75	454/463
Bacteria	Proteobacteria	Ralstonia eutropha JMP134	YP_299386.1	5 E-75	387/463
Bacteria	Actinobacteria	Mycobacterium sp. JLS	YP_001069405.1	8 E-75	419/463
Bacteria	Actinobacteria	Mycobacterium sp. KMS	YP_937101.1	9 E-75	419/463
Bacteria	Actinobacteria	Mycobacterium sp. MCS	YP_638251.1	9 E-75	419/463
Bacteria	Actinobacteria	Mycobacterium tuberculosis CPHL_A;	ZP_05764481.1	1 E-73	452/463
Bacteria	Actinobacteria	Mycobacterium tuberculosis H37Rv;	NP_216546.1	1 E-73	452/463
Bacteria	Actinobacteria	Mycobacterium tuberculosis K85;	ZP_05772806.1	1 E-73	452/463
Bacteria	Actinobacteria	Nocardiopsis dassonvillei subsp. dassonvillei	ZP_04335503.1	1 E-73	422/463
Bacteria	Proteobacteria	Ralstonia pickettii 12J	YP_001892908.1	6 E-73	387/463
Bacteria	Actinobacteria	Arthrobacter sp. FB24	YP_831527.1	2 E-72	440/463
Bacteria	Actinobacteria	Micromonospora sp. ATCC 39149	ZP_04605534.1	5 E-72	396/463
Bacteria	Actinobacteria	Geodermatophilus obscurus DSM 43160	ZP_03891228.1	8 E-71	402/463
Bacteria	Actinobacteria	Mycobacterium tuberculosis H37Ra;	ZP_02552717.1	5 E-70	429/463
Bacteria	Actinobacteria	Salinispora arenicola CNS-205	YP_001538824.1	5 E-70	396/463
Bacteria	Proteobacteria	Bdellovibrio bacteriovorus HD100	NP_968620.1	6 E-70	401/463
Bacteria	Actinobacteria	Salinispora tropica CNB-440	YP_001160475.1	1 E-69	396/463
Bacteria	Firmicutes	Bacillus sp. NRRL B-14911	ZP_01170431.1	2 E-61	383/463
Bacteria	Actinobacteria	Mycobacterium tuberculosis str. Erdman	AAA73348.1	5 E-59	376/463
Bacteria	Actinobacteria	Thermomonospora curvata DSM 43183	ZP_04031947.1	2 E-56	418/463
Bacteria	Candidatus	Accumulibacter phosphatis clade	YP_003167437.1	4 E-55	388/463
Bacteria	Bacteroidetes	Flavobacteriales bacterium HTCC2170	ZP_01108269.1	1 E-21	377/463
Bacteria	Firmicutes	Clostridium carboxidivorans P7	ZP_05390174.1	1 E-19	372/463
Bacteria	Firmicutes	Clostridium carboxidivorans P7	ZP_05392410.1	2 E-15	372/463
Bacteria	Actinobacteria	Streptomyces sviceus ATCC 29083	ZP_05020182.1	2 E-11	371/463

AFUA_1G05860

Eukaryota	Fungi	Aspergillus fumigatus Af293	XP_750336.1	0.0	404/404
Eukaryota	Fungi	Neosartorya fischeri NRRL 181	XP_001265082.1	0.0	372/404
Eukaryota	Fungi	Aspergillus clavatus NRRL 1	XP_001269580.1	0.0	381/404
Eukaryota	Fungi	Aspergillus flavus NRRL3357	XP_002383242.1	1 E-169	394/404
Eukaryota	Fungi	Penicillium chrysogenum Wisconsin 54-1255	XP_002558686.1	1 E-165	366/404
Eukaryota	Fungi	Penicillium marneffei ATCC 18224	XP_002144087.1	1 E-155	403/404
Eukaryota	Fungi	Aspergillus oryzae RIB40	XP_001816829.1	1 E-152	367/404

Eukaryota	Fungi	Talaromyces stipitatus ATCC 10500	XP_002480467.1	1 E-149	399/404
Eukaryota	Fungi	Aspergillus nidulans FGSC A4	tpeCBF74698.1	1 E-138	367/404
Eukaryota	Fungi	Coccidioides posadasii C735 delta	EER26290.1	1 E-136	348/404
Eukaryota	Fungi	Uncinocarpus reesii 1704	XP_002542553.1	1 E-135	348/404
Eukaryota	Fungi	Coccidioides immitis RS;	XP_001244440.1	1 E-134	348/404
Eukaryota	Fungi	Paracoccidioides brasiliensis Pb01;	EEH39610.1	1 E-131	352/404
Eukaryota	Fungi	Paracoccidioides brasiliensis Pb18;	EEH44195.1	1 E-127	352/404
Eukaryota	Fungi	Ajellomyces capsulatus G186AR	EEH04987.1	1 E-124	351/404
Eukaryota	Fungi	Ajellomyces dermatitidis SLH14081	XP_002625160.1	1 E-123	345/404
Eukaryota	Fungi	Ajellomyces capsulatus H143	EER45063.1	1 E-123	351/404
Eukaryota	Fungi	Paracoccidioides brasiliensis Pb03;	EEH19825.1	1 E-123	379/404
Eukaryota	Fungi	Aspergillus nidulans FGSC A4	XP_661708.1	1 E-111	326/404
Eukaryota	Fungi	Microsporum canis CBS 113480	EEQ28047.1	1 E-104	350/404
Eukaryota	Fungi	Aspergillus niger CBS 513.88	XP_001398818.1	1 E-98	370/404
Eukaryota	Fungi	Nectria haematococca mpVI 77-13-4	EEU34155.1	3 E-93	343/404
Eukaryota	Fungi	Chaetomium globosum CBS 148.51	XP_001229858.1	1 E-89	341/404
Eukaryota	Fungi	Podospora anserina DSM 980	XP_001908245.1	7 E-85	368/404

AFUA_1G05960

Eukaryota	Fungi	Aspergillus fumigatus Af293	XP_750346.1	0.0	965/965
Eukaryota	Fungi	Neosartorya fischeri NRRL 181	XP_001265072.1	0.0	967/965
Eukaryota	Fungi	Aspergillus clavatus NRRL 1	XP_001269565.1	0.0	973/965
Eukaryota	Fungi	Aspergillus nidulans FGSC A4	XP_661804.1	0.0	943/965
Eukaryota	Fungi	Aspergillus oryzae RIB40	XP_001816944.1	0.0	951/965
Eukaryota	Fungi	Aspergillus nidulans FGSC A4	tpeCBF74498.1	0.0	943/965
Eukaryota	Fungi	Aspergillus terreus NIH2624	XP_001212208.1	0.0	942/965
Eukaryota	Fungi	Aspergillus niger CBS 513.88	XP_001398837.1	0.0	986/965
Eukaryota	Fungi	Penicillium chrysogenum Wisconsin 54-1255	XP_002558658.1	0.0	970/965
Eukaryota	Fungi	Penicillium marneffei ATCC 18224	XP_002144066.1	0.0	977/965
Eukaryota	Fungi	Talaromyces stipitatus ATCC 10500	XP_002480449.1	0.0	985/965
Eukaryota	Fungi	Ajellomyces dermatitidis SLH14081	XP_002625187.1	0.0	985/965
Eukaryota	Fungi	Aspergillus flavus NRRL3357	XP_002383124.1	0.0	822/965
Eukaryota	Fungi	Paracoccidioides brasiliensis Pb03;	EEH19850.1	0.0	987/965
Eukaryota	Fungi	Paracoccidioides brasiliensis Pb18;	EEH44221.1	0.0	987/965
Eukaryota	Fungi	Paracoccidioides brasiliensis Pb01;	EEH39639.1	0.0	988/965
Eukaryota	Fungi	Coccidioides immitis RS;	XP_001244414.1	0.0	960/965
Eukaryota	Fungi	Ajellomyces capsulatus G186AR	EEH04959.1	0.0	984/965
Eukaryota	Fungi	Coccidioides posadasii C735 delta	EER26310.1	0.0	963/965
Eukaryota	Fungi	Ajellomyces capsulatus H143	EER45038.1	0.0	919/965
Eukaryota	Fungi	Microsporum canis CBS 113480	EEQ27944.1	0.0	947/965
Eukaryota	Fungi	Pyrenophora tritici-repentis Pt-1C-BFP	XP_001934391.1	0.0	953/965
Eukaryota	Fungi	Phaeosphaeria nodorum SN15	XP_001795964.1	0.0	955/965
Eukaryota	Fungi	Botryotinia fuckeliana B05.10	XP_001552553.1	0.0	1042/965
Eukaryota	Fungi	Sclerotinia sclerotiorum 1980 UF-70	XP_001586609.1	0.0	1040/965
Eukaryota	Fungi	Podospora anserina DSM 980	XP_001911135.1	0.0	1009/965
Eukaryota	Fungi	Nectria haematococca mpVI 77-13-4	EEU42122.1	0.0	1029/965
Eukaryota	Fungi	Verticillium albo-atrum VaMs.102	EEY23304.1	0.0	1016/965
Eukaryota	Fungi	Gibberella zeae PH-1	XP_382255.1	0.0	1033/965
Eukaryota	Fungi	Chaetomium globosum CBS 148.51	XP_001224992.1	0.0	993/965
Eukaryota	Fungi	Magnaporthe grisea 70-15	XP_370255.2	0.0	977/965
Eukaryota	Fungi	Laccaria bicolor S238N-H82	XP_001881620.1	2 E-98	954/965
Eukaryota	Fungi	Coprinopsis cinerea okayama7#130	XP_001829902.1	2 E-97	1018/965
Eukaryota	Fungi	Saccharomyces cerevisiae RM11-1a	EDV12028.1	1 E-82	912/965
Eukaryota	Fungi	Saccharomyces cerevisiae AWRI1631	EDZ73803.1	2 E-82	912/965
Eukaryota	Fungi	Saccharomyces cerevisiae JAY291	EEU06939.1	2 E-82	912/965
Eukaryota	Fungi	Saccharomyces cerevisiae YJM789	EDN64687.1	2 E-82	912/965
Eukaryota	Fungi	Saccharomyces cerevisiae	NP_009630.2	6 E-81	912/965

Eukaryota	Fungi	Lachancea thermotolerans CBS 6340	XP_002552441.1	2 E-77	901/965
Eukaryota	Fungi	Zygosaccharomyces rouxii CBS 732	XP_002498533.1	3 E-77	934/965
Eukaryota	Fungi	Pichia pastoris GS115	XP_002493193.1	4 E-75	883/965
Eukaryota	Fungi	Candida glabrata CBS 138	XP_449429.1	5 E-72	920/965
Eukaryota	Fungi	Lodderomyces elongisporus NRRL YB-4239	XP_001526057.1	1 E-68	896/965
Eukaryota	Fungi	Kluyveromyces lactis NRRL Y-1140	XP_455613.1	5 E-67	898/965
Eukaryota	Fungi	Vanderwaltozyma polyspora DSM 70294	XP_001644520.1	1 E-64	883/965
Eukaryota	Fungi	Candida dubliniensis CD36	XP_002418923.1	4 E-63	851/965
Eukaryota	Fungi	Yarrowia lipolytica CLIB122	XP_501316.1	5 E-62	911/965
Eukaryota	Fungi	Schizosaccharomyces pombe	NP_588494.1	9 E-62	785/965
Eukaryota	Fungi	Debaryomyces hansenii	CAG88967.2	2 E-61	959/965
Eukaryota	Fungi	Debaryomyces hansenii CBS767	XP_460635.1	1 E-59	959/965
Eukaryota	Fungi	Clavispora lusitaniae ATCC 42720	XP_002614845.1	2 E-58	913/965

AFUA_1G06800

Eukaryota	Fungi	Aspergillus fumigatus Af293	XP_750429.1	0.0	404/404
Eukaryota	Fungi	Neosartorya fischeri NRRL 181	XP_001264985.1	0.0	411/404
Eukaryota	Fungi	Aspergillus clavatus NRRL 1	XP_001269478.1	1 E-180	413/404
Eukaryota	Fungi	Aspergillus oryzae RIB40	XP_001818997.1	1 E-159	405/404
Eukaryota	Fungi	Aspergillus nidulans FGSC A4	XP_662838.1	1 E-156	413/404
Eukaryota	Fungi	Penicillium chrysogenum Wisconsin 54-1255	XP_002558613.1	1 E-152	405/404
Eukaryota	Fungi	Penicillium marneffei ATCC 18224	XP_002144310.1	1 E-140	415/404
Eukaryota	Fungi	Talaromyces stipitatus ATCC 10500	XP_002341238.1	1 E-140	413/404
Eukaryota	Fungi	Aspergillus niger CBS 513.88	XP_001399035.1	1 E-129	422/404
Eukaryota	Fungi	Nectria haematococca mpVI 77-13-4	EEU40685.1	1 E-108	407/404
Eukaryota	Fungi	Gibberella zeae PH-1	XP_383607.1	1 E-108	405/404
Eukaryota	Fungi	Botryotinia fuckeliana B05.10	XP_001554462.1	1 E-105	405/404
Eukaryota	Fungi	Sclerotinia sclerotiorum 1980 UF-70	XP_001595623.1	1 E-104	406/404
Eukaryota	Fungi	Verticillium albo-atrum VaMs.102	EEY22372.1	3 E-99	395/404
Eukaryota	Fungi	Aspergillus niger CBS 513.88	XP_001389108.1	1 E-67	398/404
Eukaryota	Fungi	Fusarium oxysporum;	ACB12554.1	8 E-67	409/404
Bacteria	Proteobacteria	Rhodopseudomonas palustris CGA009	NP_950156.1	1 E-64	364/404
Bacteria	Proteobacteria	Rhodopseudomonas palustris TIE-1	YP_001994270.1	1 E-64	364/404
Bacteria	Proteobacteria	Rhodopseudomonas palustris HaA2	YP_488293.1	3 E-64	364/404
Bacteria	Proteobacteria	Rhodopseudomonas palustris BisB5	YP_571526.1	7 E-64	364/404
Eukaryota	Fungi	Gibberella moniliformis	AAG27129.1	8 E-63	407/404
Bacteria	Proteobacteria	Rhodopseudomonas palustris BisA53	YP_783807.1	8 E-60	368/404
Bacteria	Proteobacteria	Novosphingobium aromaticivorans DSM 12444	YP_497725.1	5 E-58	375/404
Bacteria	Proteobacteria	Bradyrhizobium japonicum USDA 110	NP_767635.1	2 E-56	355/404
Bacteria	Proteobacteria	Bordetella bronchiseptica RB50	NP_889327.1	2 E-54	372/404
Bacteria	Proteobacteria	gamma proteobacterium NOR5-3	ZP_05129112.1	3 E-53	360/404
Bacteria	Proteobacteria	Oceanicola batsensis HTCC2597	ZP_00997619.1	7 E-51	356/404
Eukaryota	Fungi	Pyrenophora tritici-repentis Pt-1C-BFP	XP_001936260.1	2 E-42	350/404
Bacteria	Actinobacteria	Streptomyces sp. AA4	ZP_05478990.1	2 E-40	358/404
Bacteria	Actinobacteria	Rhodococcus jostii RHA1	YP_707645.1	3 E-40	339/404
Bacteria	Proteobacteria	Burkholderia graminis C4D1M	ZP_02886144.1	6 E-39	356/404
Eukaryota	Fungi	Gibberella zeae PH-1	XP_384039.1	8 E-34	339/404
Bacteria	Proteobacteria	Bradyrhizobium sp. BTAi1	YP_001241044.1	3 E-33	378/404
Bacteria	Proteobacteria	Bordetella bronchiseptica RB50	NP_887106.1	2 E-28	358/404
Bacteria	Proteobacteria	Bordetella parapertussis 12822	NP_882898.1	3 E-28	358/404
Bacteria	Proteobacteria	Geobacter sp. FRC-32	YP_002535735.1	2 E-24	364/404
Bacteria	Firmicutes	Bacillus selenitireducens MLS10	ZP_02170477.1	6 E-23	366/404
Bacteria	Proteobacteria	Paracoccus denitrificans PD1222	YP_916987.1	1 E-22	359/404
Bacteria	Proteobacteria	Shewanella sediminis HAW-EB3	YP_001474086.1	2 E-22	325/404
Bacteria	Firmicutes	Thermoanaerobacter mathranii subsp. mathranii	ZP_05378404.1	4 E-22	325/404
Bacteria	Proteobacteria	Shewanella halifaxensis HAW-EB4	YP_001674468.1	5 E-22	330/404
Eukaryota	Fungi	Ajellomyces capsulatus H143	EER41842.1	6 E-22	375/404

Bacteria	Firmicutes	Clostridium carboxidivorans P7	ZP_05392405.1	1 E-21	359/404
Bacteria	Proteobacteria	Shewanella benthica KT99	ZP_02159320.1	4 E-21	325/404
Bacteria	Proteobacteria	Escherichia albertii TW07627	ZP_02900332.1	4 E-21	326/404
Bacteria	Firmicutes	Thermoanaerobacter ethanolicus CCSD1	ZP_05493601.1	4 E-21	368/404
Eukaryota	Fungi	Microsporum canis CBS 113480	EEQ30646.1	5 E-21	348/404
Bacteria	Proteobacteria	Shewanella piezotolerans WP3	YP_002311946.1	7 E-21	340/404
Bacteria	Proteobacteria	Escherichia coli 101-1	ZP_03071743.1	7 E-21	326/404
Bacteria	Firmicutes	Halothermothrix orenii H 168	YP_002507967.1	7 E-21	335/404
Bacteria	Proteobacteria	Escherichia coli BL21(DE3)	YP_003035180.1	8 E-21	326/404
Bacteria	Proteobacteria	Escherichia coli HS	YP_001459571.1	8 E-21	326/404
Bacteria	Proteobacteria	Shewanella loihica PV-4	YP_001094035.1	9 E-21	330/404
Bacteria	Proteobacteria	Desulfuromonas acetoxidans DSM 684	ZP_01312671.1	1 E-20	363/404
Bacteria	Proteobacteria	Escherichia coli IAI39	YP_002409145.1	1 E-20	326/404
Bacteria	Firmicutes	Clostridium kluyveri DSM 555	YP_001395788.1	1 E-20	348/404
Bacteria	Proteobacteria	Escherichia coli O127:H6 str.	YP_002330547.1	2 E-20	326/404
Bacteria	Proteobacteria	Escherichia coli E24377A	YP_001464122.1	2 E-20	326/404
Bacteria	Proteobacteria	Escherichia coli O157:H7 EDL933	NP_289354.1	2 E-20	326/404
Bacteria	Firmicutes	Clostridium kluyveri NBRC 12016	YP_002472586.1	2 E-20	348/404
Bacteria	Proteobacteria	Shigella dysenteriae Sd197	YP_404527.1	2 E-20	326/404
Bacteria	Proteobacteria	Escherichia coli	CAA33124.1	2 E-20	326/404
Bacteria	Proteobacteria	Escherichia coli SMS-3-5	YP_001744963.1	2 E-20	326/404
Bacteria	Proteobacteria	Shigella flexneri 2a str.	NP_838316.1	3 E-20	326/404
Bacteria	Proteobacteria	Shigella sonnei Ss046	YP_311790.1	4 E-20	326/404
Bacteria	Proteobacteria	Escherichia coli CFT073	NP_755244.1	5 E-20	326/404
Bacteria	Proteobacteria	Escherichia coli UTI89	YP_542153.1	6 E-20	326/404
Bacteria	Proteobacteria	Shigella flexneri 5 str.	YP_690260.1	8 E-20	326/404
Eukaryota	Fungi	Pyrenophora tritici-repentis Pt-1C-BFP	XP_001938016.1	1 E-19	353/404
Bacteria	Proteobacteria	Escherichia coli 536	YP_670670.1	1 E-19	326/404
Bacteria	Firmicutes	Alkaliphilus oremlandii OhILAs	YP_001512341.1	1 E-19	352/404
Bacteria	Proteobacteria	Ralstonia eutropha H16	YP_726053.1	2 E-19	327/404
Bacteria	Proteobacteria	Escherichia coli ED1a	YP_002399134.1	2 E-19	326/404
Eukaryota	Fungi	Leptosphaeria maculans	ABB55460.1	2 E-19	333/404
Bacteria	Proteobacteria	Ralstonia eutropha JMP134	YP_295632.1	2 E-19	327/404
Bacteria	Spirochaetes	Brachyspira hyodysenteriae WA1	YP_002721937.1	2 E-19	373/404
Bacteria	Proteobacteria	Cupriavidus taiwanensis	YP_002005495.1	3 E-19	327/404
Bacteria	Actinobacteria	Bifidobacterium longum subsp. infantis	YP_002323678.1	4 E-19	388/404
Bacteria	Firmicutes	Enterococcus faecium DO	ZP_00604016.1	4 E-19	327/404
Bacteria	Proteobacteria	Pseudovibrio sp. JE062	ZP_05087379.1	4 E-19	386/404
Bacteria	Exiguobacterium	Exiguobacterium sp. AT1b;	YP_002884534.1	4 E-19	327/404
Bacteria	Proteobacteria	Yersinia bercovieri ATCC 43970	ZP_04628345.1	5 E-19	347/404
Bacteria	Actinobacteria	Bifidobacterium bifidum NCIMB 41171	ZP_03647027.1	5 E-19	390/404
Bacteria	Firmicutes	Enterococcus faecium 1,230,933	ZP_05658529.1	5 E-19	327/404
Bacteria	Actinobacteria	Bifidobacterium longum subsp. infantis	ZP_03976522.1	6 E-19	388/404
Bacteria	Firmicutes	Abiotrophia defectiva ATCC 49176	ZP_04450774.1	6 E-19	391/404
Bacteria	Proteobacteria	Desulfovibrio vulgaris str. 'Miyazaki	YP_002435413.1	6 E-19	348/404
Bacteria	Actinobacteria	Bifidobacterium longum DJO10A	ZP_00121713.1	6 E-19	388/404
Bacteria	Actinobacteria	Bifidobacterium longum subsp. infantis	ZP_04664038.1	7 E-19	388/404
Bacteria	Firmicutes	Listeria monocytogenes FSL N3-165	ZP_05233217.1	8 E-19	341/404
Bacteria	Actinobacteria	Bifidobacterium longum NCC2705	NP_696730.1	1 E-18	388/404
Bacteria	Proteobacteria	Geobacter uraniireducens Rf4	YP_001232260.1	2 E-18	358/404
Bacteria	Firmicutes	Bacillus pseudomycoides DSM 12442	ZP_04153030.1	2 E-18	410/404
Bacteria	Firmicutes	Enterococcus faecium 1,231,501	ZP_05665437.1	2 E-18	327/404
Bacteria	Firmicutes	Bacillus mycoides Rock3-17;	ZP_04158738.1	2 E-18	410/404
Bacteria	Proteobacteria	Ralstonia solanacearum GMI1000	NP_522547.1	2 E-18	327/404
Bacteria	Proteobacteria	Erwinia tasmaniensis Et1/99	YP_001907520.1	2 E-18	392/404
Bacteria	Proteobacteria	Yersinia rohdei ATCC 43380	ZP_04613358.1	3 E-18	383/404
Bacteria	Firmicutes	Listeria monocytogenes EGD-e	NP_464696.1	3 E-18	341/404

AFUA_1G06810

Eukaryota	Fungi	Aspergillus fumigatus Af293	XP_750430.1	0.0	799/799
Eukaryota	Fungi	Neosartorya fischeri NRRL 181	XP_001264984.1	0.0	799/799
Eukaryota	Fungi	Aspergillus clavatus NRRL 1	XP_001269477.1	0.0	799/799
Eukaryota	Fungi	Aspergillus niger CBS 513.88	XP_001393703.1	0.0	802/799
Eukaryota	Fungi	Aspergillus oryzae RIB40	XP_001818998.1	0.0	799/799
Eukaryota	Fungi	Penicillium chrysogenum Wisconsin 54-1255	XP_002558749.1	0.0	797/799
Eukaryota	Fungi	Penicillium marneffei ATCC 18224	XP_002144311.1	0.0	792/799
Eukaryota	Fungi	Talaromyces stipitatus ATCC 10500	XP_002341237.1	0.0	794/799
Eukaryota	Fungi	Aspergillus nidulans FGSC A4	XP_661498.1	0.0	796/799
Eukaryota	Fungi	Aspergillus terreus NIH2624	XP_001212115.1	0.0	757/799
Eukaryota	Fungi	Coccidioides posadasii C735 delta	EER26196.1	0.0	798/799
Eukaryota	Fungi	Paracoccidioides brasiliensis Pb01;	EEH37425.1	0.0	798/799
Eukaryota	Fungi	Coccidioides immitis RS;	XP_001244575.1	0.0	794/799
Eukaryota	Fungi	Paracoccidioides brasiliensis Pb18;	EEH43317.1	0.0	774/799
Eukaryota	Fungi	Paracoccidioides brasiliensis Pb03;	EEH18689.1	0.0	780/799
Eukaryota	Fungi	Microsporum canis CBS 113480	EEQ27556.1	0.0	799/799
Eukaryota	Fungi	Ajellomyces dermatitidis ER-3	EEQ87874.1	0.0	809/799
Eukaryota	Fungi	Ajellomyces dermatitidis SLH14081	XP_002627881.1	0.0	809/799
Eukaryota	Fungi	Ajellomyces capsulatus G186AR	EEH08875.1	0.0	813/799
Eukaryota	Fungi	Uncinocarpus reesii 1704	XP_002542432.1	0.0	787/799
Eukaryota	Fungi	Botryotinia fuckeliana B05.10	XP_001557559.1	0.0	720/799
Eukaryota	Fungi	Podospora anserina DSM 980	XP_001912055.1	0.0	787/799
Eukaryota	Fungi	Magnaporthe grisea 70-15	XP_362853.1	0.0	782/799
Eukaryota	Fungi	Chaetomium globosum CBS 148.51	XP_001224733.1	0.0	764/799
Eukaryota	Fungi	Neurospora crassa OR74A	XP_961070.1	0.0	762/799
Eukaryota	Fungi	Nectria haematococca mpVI 77-13-4	EEU45097.1	0.0	767/799
Eukaryota	Fungi	Verticillium albo-atrum VaMs.102	EEY14563.1	0.0	815/799
Eukaryota	Fungi	Gibberella zeae PH-1	XP_390374.1	0.0	767/799
Eukaryota	Fungi	Sclerotinia sclerotiorum 1980 UF-70	XP_001588189.1	0.0	703/799
Eukaryota	Fungi	Schizosaccharomyces pombe	NP_596189.1	0.0	769/799
Eukaryota	Fungi	Schizosaccharomyces pombe	Q9P7D4.2	0.0	769/799
Eukaryota	Fungi	Candida albicans SC5314	XP_712172.1	0.0	772/799
Eukaryota	Fungi	Candida dubliniensis CD36	XP_002422112.1	0.0	772/799
Eukaryota	Fungi	Schizosaccharomyces japonicus yFS275	XP_002172879.1	0.0	752/799
Eukaryota	Fungi	Candida albicans WO-1	EEQ43656.1	0.0	772/799
Eukaryota	Fungi	Candida tropicalis MYA-3404	XP_002545861.1	0.0	770/799
Eukaryota	Fungi	Debaryomyces hansenii CBS767	XP_460250.1	0.0	772/799
Eukaryota	Fungi	Candida glabrata CBS 138	XP_446075.1	0.0	779/799
Eukaryota	Fungi	Kluyveromyces lactis NRRL Y-1140	XP_452349.1	0.0	753/799
Eukaryota	Fungi	Pichia stipitis CBS 6054	XP_001385589.2	0.0	787/799
Eukaryota	Fungi	Clavispora lusitaniae ATCC 42720	XP_002619575.1	0.0	784/799
Eukaryota	Fungi	Saccharomyces cerevisiae	NP_012335.1	0.0	779/799
Eukaryota	Fungi	Lachancea thermotolerans CBS 6340	XP_002554366.1	0.0	776/799
Eukaryota	Fungi	Saccharomyces cerevisiae RM11-1a	EDV12582.1	0.0	779/799
Eukaryota	Fungi	Lodderomyces elongisporus NRRL YB-4239	XP_001525239.1	0.0	775/799
Eukaryota	Fungi	Yarrowia lipolytica CLIB122	XP_503960.1	0.0	780/799
Eukaryota	Fungi	Saccharomyces cerevisiae YJM789	EDN63190.1	0.0	779/799
Eukaryota	Fungi	Pichia guilliermondii ATCC 6260	EDK37524.2	0.0	766/799
Eukaryota	Fungi	Pichia pastoris GS115	XP_002492885.1	0.0	781/799
Eukaryota	Fungi	Zygosaccharomyces rouxii CBS 732	XP_002496418.1	0.0	771/799
Eukaryota	Fungi	Pichia guilliermondii ATCC 6260	XP_001485951.1	0.0	766/799
Eukaryota	Fungi	Cryptococcus neoformans var. neoformans	XP_568476.1	0.0	782/799
Eukaryota	Fungi	Vanderwaltozyma polyspora DSM 70294	XP_001646409.1	0.0	758/799
Eukaryota	Fungi	Laccaria bicolor S238N-H82	XP_001873889.1	0.0	777/799
Eukaryota	Fungi	Ashbya gossypii ATCC 10895	NP_986176.1	0.0	764/799
Eukaryota	Fungi	Coprinopsis cinerea okayama7#130	XP_001828909.1	0.0	767/799

Eukaryota	Fungi	Cryptococcus neoformans var. neoformans	XP_570245.1	0.0	771/799
Eukaryota	Fungi	Ustilago maydis 521	XP_758838.1	0.0	770/799
Eukaryota	Fungi	Piromyces sp. E2	CAA76360.1	0.0	726/799
Eukaryota	Fungi	Schizosaccharomyces pombe	NP_594031.1	0.0	737/799
Eukaryota	Amoebozoa	Dictyostelium discoideum AX4	XP_641958.1	0.0	731/799
Eukaryota	Fungi	Laccaria bicolor S238N-H82	XP_001878558.1	0.0	731/799
Eukaryota	Fungi	Aspergillus flavus NRRL3357	XP_002374915.1	0.0	733/799
Eukaryota	Fungi	Aspergillus oryzae RIB40	XP_001819597.1	0.0	733/799
Eukaryota	Rhodophyta	Gracilaria gracilis	P49609.1	0.0	737/799
Eukaryota	Fungi	Coprinopsis cinerea okayama7#130	XP_001834931.1	0.0	731/799
Eukaryota	Fungi	Coccidioides posadasii;	ABH10644.1	0.0	733/799
Eukaryota	Fungi	Coccidioides immitis RS;	XP_001247958.1	0.0	733/799
Eukaryota	Fungi	Paracoccidioides brasiliensis Pb18;	EEH48819.1	0.0	751/799
Eukaryota	Fungi	Laccaria bicolor S238N-H82	XP_001879583.1	0.0	737/799
Eukaryota	Fungi	Aspergillus terreus	AAC61778.1	0.0	772/799
Eukaryota	Fungi	Neosartorya fischeri NRRL 181	XP_001258433.1	0.0	735/799
Eukaryota	Fungi	Malassezia globosa CBS 7966	XP_001728868.1	0.0	737/799
Eukaryota	Fungi	Aspergillus fumigatus Af293	XP_751171.1	0.0	735/799
Eukaryota	Fungi	Aspergillus niger CBS 513.88	XP_001393157.1	0.0	734/799
Eukaryota	Fungi	Ustilago maydis 521	XP_759046.1	0.0	735/799
Eukaryota	Fungi	Schizosaccharomyces japonicus yFS275	XP_002174726.1	0.0	732/799
Eukaryota	Fungi	Aspergillus fumigatus A1163	EDP55486.1	0.0	776/799
Eukaryota	Viridiplantae	Chlamydomonas reinhardtii	XP_001689702.1	0.0	745/799
Eukaryota	Fungi	Ajellomyces capsulatus G186AR	EEH09145.1	0.0	733/799
Eukaryota	Fungi	Paracoccidioides brasiliensis Pb03;	EEH22293.1	0.0	724/799
Eukaryota	Choanoflagellida	Monosiga brevicollis MX1	XP_001746450.1	0.0	736/799
Eukaryota	Fungi	Podospora anserina DSM 980	XP_001929771.1	0.0	733/799
Eukaryota	Metazoa	Apis mellifera	XP_391994.1	0.0	763/799
Eukaryota	Fungi	Ajellomyces dermatitidis ER-3	EEQ85091.1	0.0	733/799
Eukaryota	Metazoa	Nasonia vitripennis	XP_001607990.1	0.0	772/799
Eukaryota	Fungi	Sclerotinia sclerotiorum 1980 UF-70	XP_001587807.1	0.0	732/799
Eukaryota	Fungi	Zygosaccharomyces rouxii CBS 732	XP_002498940.1	0.0	730/799
Eukaryota	Fungi	Aspergillus nidulans FGSC A4	tpeCBF81741.1	0.0	770/799
Eukaryota	Fungi	Ajellomyces dermatitidis SLH14081	XP_002629078.1	0.0	730/799
Eukaryota	Fungi	Candida tropicalis MYA-3404	XP_002548754.1	0.0	735/799
Eukaryota	Fungi	Penicillium marneffei ATCC 18224	XP_002147388.1	0.0	781/799
Eukaryota	Fungi	Clavispora lusitaniae ATCC 42720	XP_002619843.1	0.0	773/799
Eukaryota	Fungi	Lachancea thermotolerans CBS 6340	XP_002552883.1	0.0	734/799
Eukaryota	Fungi	Aspergillus nidulans FGSC A4	XP_663129.1	0.0	763/799
Eukaryota	Fungi	Candida glabrata CBS 138	XP_445684.1	0.0	762/799
Eukaryota	Fungi	Aspergillus clavatus NRRL 1	XP_001268345.1	0.0	735/799
Eukaryota	Fungi	Penicillium chrysogenum Wisconsin 54-1255	XP_002564244.1	0.0	735/799
Eukaryota	Fungi	Neurospora crassa OR74A	XP_959787.1	0.0	778/799
Eukaryota	Metazoa	Antheraea yamamai	BAC65324.1	0.0	761/799
Eukaryota	Fungi	Talaromyces stipitatus ATCC 10500	XP_002481522.1	0.0	734/799
Eukaryota	Metazoa	Caenorhabditis briggsae AF16	XP_001666692.1	0.0	729/799
Eukaryota	Fungi	Vanderwaltozyma polyspora DSM 70294	XP_001644039.1	0.0	738/799
Eukaryota	Fungi	Candida albicans SC5314	XP_716225.1	0.0	735/799
Eukaryota	Fungi	Chaetomium globosum CBS 148.51	XP_001219634.1	0.0	762/799
Eukaryota	Fungi	Microsporum canis CBS 113480	EEQ30480.1	0.0	735/799
Eukaryota	Fungi	Magnaporthe grisea 70-15	XP_360978.1	0.0	763/799
Eukaryota	Fungi	Saccharomyces cerevisiae	NP_013407.1	0.0	730/799
Eukaryota	Fungi	Kluyveromyces lactis NRRL Y-1140	XP_452974.1	0.0	733/799
Eukaryota	Fungi	Pichia guilliermondii ATCC 6260	XP_001486725.1	0.0	734/799
Eukaryota	Fungi	Nectria haematococca mpVI 77-13-4	EEU35008.1	0.0	768/799
Eukaryota	Fungi	Candida albicans WO-1	EEQ43860.1	0.0	735/799
Eukaryota	Metazoa	Tetraodon nigroviridis	CAG04508.1	0.0	759/799
Eukaryota	Fungi	Aspergillus flavus NRRL3357	XP_002382144.1	0.0	735/799

Eukaryota	Fungi	Debaryomyces hansenii CBS767	XP_459431.1	0.0	736/799
Eukaryota	Fungi	Candida dubliniensis CD36	XP_002422320.1	0.0	735/799
Eukaryota	Fungi	Ashbya gossypii ATCC 10895	NP_984065.1	0.0	732/799
Eukaryota	Fungi	Lodderomyces elongisporus NRRL YB-4239	XP_001525166.1	0.0	736/799
Eukaryota	Metazoa	Caenorhabditis elegans	NP_741235.1	0.0	729/799
Eukaryota	Fungi	Saccharomyces cerevisiae	AAA34389.1	0.0	758/799
Eukaryota	Fungi	Pichia pastoris GS115	XP_002489444.1	0.0	734/799
Eukaryota	Metazoa	Danio rerio	NP_944590.1	0.0	733/799
Eukaryota	Fungi	Pichia stipitis CBS 6054	XP_001386080.1	0.0	737/799
Eukaryota	Metazoa	Pediculus humanus corporis	XP_002427220.1	0.0	734/799
Eukaryota	Metazoa	Tribolium castaneum	XP_974860.1	0.0	737/799
Eukaryota	Fungi	Gibberella zeae PH-1	XP_388129.1	0.0	764/799
Eukaryota	Metazoa	Trichoplax adhaerens	XP_002109178.1	0.0	736/799
Eukaryota	Fungi	Paracoccidioides brasiliensis Pb01;	EEH33999.1	0.0	689/799
Bacteria	Bacteroidetes	Dyadobacter fermentans DSM 18053	YP_003085622.1	0.0	740/799
Eukaryota	Fungi	Yarrowia lipolytica CLIB122	XP_502616.2	0.0	732/799
Eukaryota	Fungi	Ajellomyces capsulatus NAm1	XP_001539799.1	0.0	718/799
Eukaryota	Fungi	Uncinocarpus reesii 1704	XP_002584750.1	0.0	709/799
Eukaryota	Choanoflagellida	Monosiga brevicollis MX1	XP_001747281.1	0.0	735/799
Bacteria	Chlorobi	Chloroherpeton thalassium ATCC 35110	YP_001997349.1	0.0	741/799
Eukaryota	Metazoa	Xenopus laevis	AAH68910.1	0.0	733/799
Eukaryota	Metazoa	Aedes aegypti	XP_001664086.1	0.0	773/799
Eukaryota	Metazoa	Homo sapiens	CAG38805.1	0.0	733/799
Eukaryota	Metazoa	Drosophila ananassae	XP_001961809.1	0.0	737/799
Eukaryota	Metazoa	Drosophila mojavensis	XP_002003587.1	0.0	737/799
Eukaryota	Metazoa	Homo sapiens	AAH26196.1	0.0	733/799
Eukaryota	Metazoa	Drosophila erecta	XP_001974057.1	0.0	787/799
Eukaryota	Metazoa	Aedes aegypti	XP_001663037.1	0.0	773/799
Eukaryota	Fungi	Yarrowia lipolytica	AAT92542.1	0.0	732/799
Eukaryota	Metazoa	Xenopus laevis	NP_001086263.1	0.0	733/799
Eukaryota	Metazoa	Drosophila persimilis	XP_002018616.1	0.0	738/799
Eukaryota	Metazoa	Daphnia pulex	CAB72317.1	0.0	759/799
Eukaryota	Metazoa	Taeniopygia guttata	XP_002197422.1	0.0	733/799
Eukaryota	Metazoa	Drosophila pseudoobscura pseudoobscura	XP_001357493.2	0.0	738/799
Eukaryota	Metazoa	Drosophila melanogaster	NP_524708.1	0.0	769/799
Eukaryota	Metazoa	Anopheles gambiae str. PEST	XP_317642.4	0.0	736/799
Eukaryota	Metazoa	Ciona intestinalis	XP_002131821.1	0.0	771/799
Eukaryota	Metazoa	Drosophila simulans	XP_002080131.1	0.0	737/799
Eukaryota	Metazoa	Drosophila sechellia	XP_002042528.1	0.0	737/799
Eukaryota	Metazoa	Xenopus (Silurana) tropicalis	NP_001011445.1	0.0	733/799
Bacteria	Spirochaetes	Leptospira borgpetersenii serovar Hardjo-bovis	YP_799496.1	0.0	744/799
Bacteria	Bacteroidetes	Chitinophaga pinensis DSM 2588	YP_003121712.1	0.0	746/799
Eukaryota	Metazoa	Brugia malayi	XP_001892954.1	0.0	724/799
Eukaryota	Metazoa	Drosophila yakuba	XP_002090242.1	0.0	737/799
Eukaryota	Metazoa	Drosophila grimshawi	XP_001988617.1	0.0	737/799
Eukaryota	Metazoa	Drosophila virilis	XP_002052901.1	0.0	737/799
Eukaryota	Metazoa	Drosophila willistoni	XP_002069284.1	0.0	737/799
Eukaryota	Metazoa	Gallus gallus	CAG30932.1	0.0	733/799
Bacteria	Bacteroidetes	Spirosoma linguale DSM 74	ZP_04488412.1	0.0	738/799
Eukaryota	Metazoa	Gallus gallus	NP_989519.1	0.0	733/799
Bacteria	Spirochaetes	Leptospira interrogans serovar Copenhageni	YP_003633.1	0.0	744/799
Bacteria	Spirochaetes	Leptospira interrogans serovar Lai	NP_714871.1	0.0	744/799
Eukaryota	Metazoa	Equus caballus	XP_001502547.1	0.0	737/799
Eukaryota	Metazoa	Canis lupus familiaris	XP_849166.1	0.0	737/799
Eukaryota	Metazoa	Equus caballus	XP_001502551.1	0.0	736/799
Eukaryota	Fungi	Aspergillus niger CBS 513.88	XP_001400233.1	0.0	733/799
Eukaryota	Metazoa	Canis lupus familiaris	XP_858344.1	0.0	733/799
Eukaryota	Metazoa	Bos taurus	P20004.4	0.0	733/799

Eukaryota	Metazoa	Monodelphis domestica	XP_001378711.1	0.0	733/799
Eukaryota	Metazoa	Mus musculus	BAE29252.1	0.0	733/799
Eukaryota	Metazoa	Bos taurus	pdb1AMIA	0.0	733/799
Eukaryota	Metazoa	Bos taurus	pdb1ACOA	0.0	733/799
Eukaryota	Metazoa	Rattus norvegicus	NP_077374.2	0.0	733/799
Eukaryota	Metazoa	Canis lupus familiaris	XP_858265.1	0.0	730/799
Eukaryota	Metazoa	Mus musculus	NP_542364.1	0.0	733/799
Eukaryota	Metazoa	Mus musculus	BAE25770.1	0.0	733/799
Eukaryota	Metazoa	Bos taurus	pdb1NISA	0.0	733/799
Eukaryota	Metazoa	Bos taurus	AAI02643.1	0.0	733/799
Eukaryota	Metazoa	Mus musculus	AAH94462.1	0.0	733/799
Eukaryota	Metazoa	Bos taurus	NP_776402.1	0.0	733/799
Eukaryota	Viridiplantae	Micromonas pusilla CCMP1545	EEH52362.1	0.0	747/799
Eukaryota	Metazoa	Macaca mulatta	XP_001105023.1	0.0	733/799
Bacteria	Spirochaetes	Leptospira biflexa serovar Patoc	YP_001964972.1	0.0	744/799
Eukaryota	Metazoa	Sus scrofa	pdb5ACNA	0.0	733/799
Eukaryota	Metazoa	Homo sapiens	NP_001089.1	0.0	733/799
Eukaryota	Metazoa	Sus scrofa	NP_999119.1	0.0	733/799
Eukaryota	Metazoa	Sus scrofa	pdb1B0JA	0.0	733/799
Eukaryota	Metazoa	Homo sapiens	AAB38416.1	0.0	733/799
Bacteria	Bacteroidetes	Bacteroides sp. 3_2_5	ZP_04843591.1	0.0	727/799
Eukaryota	Metazoa	Sus scrofa	pdb1B0MA	0.0	733/799
Eukaryota	Metazoa	Homo sapiens	BAG37362.1	0.0	733/799
Bacteria	Bacteroidetes	Bacteroides fragilis YCH46	YP_101031.1	0.0	727/799
Bacteria	Bacteroidetes	Bacteroides fragilis	AAM10631.1	0.0	727/799
Bacteria	Bacteroidetes	Capnocytophaga gingivalis ATCC 33624	ZP_04057001.1	0.0	740/799
Eukaryota	Metazoa	Homo sapiens	AAD19351.2	0.0	733/799
Bacteria	Bacteroidetes	Capnocytophaga ochracea DSM 7271	YP_003141087.1	0.0	749/799
Bacteria	Bacteroidetes	Bacteroides fragilis NCTC 9343	YP_213145.1	0.0	727/799
Eukaryota	Viridiplantae	Micromonas sp. RCC299	XP_002507787.1	0.0	733/799
Eukaryota	Metazoa	Strongylocentrotus purpuratus	XP_001176556.1	0.0	732/799
Bacteria	Bacteroidetes	Parabacteroides merdae ATCC 43184	ZP_02032556.1	0.0	734/799
Eukaryota	Metazoa	Drosophila virilis	XP_002053432.1	0.0	785/799
Eukaryota	Metazoa	Rattus norvegicus	CAC11018.1	0.0	733/799
Bacteria	Bacteroidetes	Bacteroides plebeius DSM 17135	ZP_03208203.1	0.0	740/799
Eukaryota	Metazoa	Bos taurus	ABE02803.1	0.0	718/799
Eukaryota	Metazoa	Canis lupus familiaris	XP_858303.1	0.0	763/799
Bacteria	Bacteroidetes	Parabacteroides johnsonii DSM 18315	ZP_03476478.1	0.0	734/799
Eukaryota	Metazoa	Caenorhabditis elegans	NP_498738.2	0.0	675/799
Bacteria	Bacteroidetes	Robiginitalea biformata HTCC2501	YP_003195361.1	0.0	736/799
Eukaryota	Fungi	Aspergillus terreus NIH2624	XP_001212503.1	0.0	703/799
Bacteria	Bacteroidetes	Bacteroides fragilis 3_1_12	ZP_05283304.1	0.0	727/799
Eukaryota	Metazoa	Nematostella vectensis	XP_001640014.1	0.0	681/799
Eukaryota	Metazoa	Drosophila mojavensis	XP_002000243.1	0.0	785/799
Bacteria	Bacteroidetes	Bacteroides coprocola DSM 17136	ZP_03011366.1	0.0	741/799
Eukaryota	Metazoa	Drosophila pseudoobscura pseudoobscura	XP_001359921.1	0.0	736/799
Bacteria	Bacteroidetes	Bacteroides coprophilus DSM 18228	ZP_03642569.1	0.0	722/799
Bacteria	Bacteroidetes	Bacteroides uniformis ATCC 8492	ZP_02070255.1	0.0	734/799
Bacteria	Bacteroidetes	Bacteroides sp. 9_1_42FAA	ZP_04539225.1	0.0	734/799
Eukaryota	Metazoa	Drosophila persimilis	XP_002019737.1	0.0	736/799
Bacteria	Bacteroidetes	Bacteroides vulgatus ATCC 8482	YP_001299253.1	0.0	734/799
Bacteria	Bacteroidetes	Bacteroides sp. D4	ZP_04554667.1	0.0	734/799
Bacteria	Bacteroidetes	Bacteroides dorei DSM 17855	ZP_03301713.1	0.0	734/799
Bacteria	Bacteroidetes	Bacteroides eggerthii DSM 20697	ZP_03457527.1	0.0	725/799
Eukaryota	Metazoa	Drosophila melanogaster	NP_650042.2	0.0	735/799
Bacteria	Bacteroidetes	Bacteroides sp. 4_3_47FAA	ZP_05255467.1	0.0	734/799
Eukaryota	Fungi	Aspergillus oryzae RIB40	XP_001819143.1	0.0	690/799
Eukaryota	Metazoa	Macaca mulatta	XP_001104944.1	0.0	758/799

Eukaryota	Metazoa	Drosophila willistoni	XP_002073193.1	0.0	783/799
Eukaryota	Metazoa	Macaca mulatta	XP_001104650.1	0.0	685/799
Bacteria	Bacteroidetes	Parabacteroides sp. D13	ZP_05545488.1	0.0	735/799
Bacteria	Bacteroidetes	Bacteroides finegoldii DSM 17565	ZP_05415008.1	0.0	734/799
Bacteria	Bacteroidetes	Sphingobacterium spiritivorum ATCC 33861	ZP_04780977.1	0.0	738/799
Bacteria	Proteobacteria	Zymomonas mobilis subsp. mobilis	YP_162278.1	0.0	731/799
Bacteria	Proteobacteria	Zymomonas mobilis subsp. mobilis	ZP_04759175.1	0.0	731/799
Bacteria	Bacteroidetes	Bacteroides caccae ATCC 43185	ZP_01961523.1	0.0	727/799
Eukaryota	Metazoa	Drosophila erecta	XP_001980610.1	0.0	733/799
Bacteria	Bacteroidetes	Sphingobacterium spiritivorum ATCC 33300	ZP_03966826.1	0.0	738/799
Eukaryota	Metazoa	Homo sapiens	CAM25244.1	0.0	758/799
Bacteria	Bacteroidetes	Bacteroides sp. D2	ZP_05760464.1	0.0	727/799
Bacteria	Bacteroidetes	Capnocytophaga sputigena ATCC 33612	ZP_03390663.1	0.0	744/799
Bacteria	Bacteroidetes	Parabacteroides distasonis ATCC 8503	YP_001302462.1	0.0	735/799
Bacteria	Bacteroidetes	Bacteroides stercoris ATCC 43183	ZP_02436892.1	0.0	727/799
Bacteria	Bacteroidetes	Bacteroides cellulosilyticus DSM 14838	ZP_03676667.1	0.0	727/799
Eukaryota	Metazoa	Drosophila ananassae	XP_001954442.1	0.0	731/799

AFUA_1G07190

Eukaryota	Fungi	Aspergillus fumigatus Af293	XP_750466.2	1 E-127	219/219
Eukaryota	Fungi	Neosartorya fischeri NRRL 181	XP_001264949.1	1 E-117	219/219
Eukaryota	Fungi	Aspergillus clavatus NRRL 1	XP_001269444.1	3 E-50	226/219

AFUA_1G07195

Eukaryota	Fungi	Aspergillus fumigatus Af293	XP_750467.1	0.0	379/379
Eukaryota	Fungi	Neosartorya fischeri NRRL 181	XP_001264948.1	0.0	377/379
Eukaryota	Fungi	Aspergillus clavatus NRRL 1	XP_001269443.1	1 E-146	385/379
Eukaryota	Fungi	Aspergillus niger CBS 513.88	XP_001392052.1	1 E-123	371/379
Eukaryota	Fungi	Penicillium chrysogenum Wisconsin 54-1255	XP_002558336.1	1 E-110	352/379
Eukaryota	Fungi	Aspergillus nidulans FGSC A4	XP_657941.1	2 E-98	362/379
Eukaryota	Fungi	Talaromyces stipitatus ATCC 10500	XP_002479503.1	4 E-94	376/379
Eukaryota	Fungi	Penicillium marneffei ATCC 18224	XP_002143205.1	5 E-93	371/379
Eukaryota	Fungi	Nectria haematococca mpVI 77-13-4	EEU43106.1	1 E-84	365/379
Eukaryota	Fungi	Chaetomium globosum CBS 148.51	XP_001221672.1	4 E-83	364/379
Eukaryota	Fungi	Podospora anserina DSM 980	XP_001908472.1	6 E-80	370/379
Eukaryota	Fungi	Microsporum canis CBS 113480	EEQ35169.1	2 E-76	349/379
Eukaryota	Fungi	Aspergillus nidulans FGSC A4	XP_662794.1	1 E-71	399/379
Eukaryota	Fungi	Gibberella zeae PH-1	XP_385592.1	4 E-71	370/379
Eukaryota	Fungi	Nectria haematococca mpVI 77-13-4	EEU34611.1	1 E-69	344/379
Eukaryota	Fungi	Nectria haematococca mpVI 77-13-4	EEU36603.1	8 E-64	365/379
Eukaryota	Fungi	Claviceps fusiformis	ABV57818.1	3 E-61	360/379
Eukaryota	Fungi	Aspergillus fumigatus A1163	EDP55312.1	9 E-57	356/379
Eukaryota	Fungi	Talaromyces stipitatus ATCC 10500	XP_002479170.1	5 E-49	329/379
Eukaryota	Fungi	Aspergillus flavus NRRL3357	XP_002383783.1	5 E-46	362/379
Eukaryota	Fungi	Aspergillus oryzae RIB40	XP_001824813.1	3 E-40	318/379
Eukaryota	Fungi	Coprinopsis cinerea okayama7#130	XP_001838435.1	1 E-22	307/379

AFUA_1G07625

Eukaryota	Fungi	Aspergillus fumigatus Af293	XP_001481715.1	0.0	423/423
Eukaryota	Fungi	Neosartorya fischeri NRRL 181	XP_001264907.1	0.0	423/423
Eukaryota	Fungi	Aspergillus clavatus NRRL 1	XP_001269401.1	1 E-128	419/423
Eukaryota	Fungi	Penicillium chrysogenum Wisconsin 54-1255	XP_002557367.1	1 E-102	392/423
Eukaryota	Fungi	Aspergillus niger CBS 513.88	XP_001392101.1	1 E-102	409/423
Eukaryota	Fungi	Aspergillus terreus NIH2624	XP_001209516.1	1 E-100	391/423
Eukaryota	Fungi	Paracoccidioides brasiliensis Pb18;	EEH43262.1	4 E-81	443/423

Eukaryota	Fungi	Paracoccidioides brasiliensis Pb03;	EEH23159.1	7 E-80	443/423
Eukaryota	Fungi	Ajellomyces dermatitidis SLH14081	XP_002622853.1	4 E-79	458/423
Eukaryota	Fungi	Ajellomyces dermatitidis ER-3	EEQ90558.1	7 E-79	460/423
Eukaryota	Fungi	Paracoccidioides brasiliensis Pb01;	EEH38696.1	2 E-75	415/423
Eukaryota	Fungi	Penicillium marneffei ATCC 18224	XP_002149162.1	1 E-74	407/423
Eukaryota	Fungi	Microsporum canis CBS 113480	EEQ32476.1	3 E-72	417/423
Eukaryota	Fungi	Coccidioides posadasii C735 delta	EER29078.1	6 E-72	397/423
Eukaryota	Fungi	Ajellomyces capsulatus G186AR	EEH03825.1	2 E-71	464/423
Eukaryota	Fungi	Talaromyces stipitatus ATCC 10500	XP_002485017.1	8 E-71	423/423
Eukaryota	Fungi	Coccidioides immitis RS;	XP_001248769.1	1 E-70	397/423
Eukaryota	Fungi	Uncinocarpus reesii 1704	XP_002543674.1	2 E-69	395/423
Eukaryota	Fungi	Ajellomyces capsulatus H143	EER37244.1	3 E-66	346/423
Eukaryota	Fungi	Pyrenophora tritici-repentis Pt-1C-BFP	XP_001934004.1	7 E-51	400/423
Eukaryota	Fungi	Phaeosphaeria nodorum SN15	XP_001791894.1	1 E-46	342/423
Eukaryota	Fungi	Chaetomium globosum CBS 148.51	XP_001222799.1	1 E-45	415/423
Eukaryota	Fungi	Nectria haematococca mpVI 77-13-4	EEU35284.1	3 E-44	388/423
Eukaryota	Fungi	Gibberella zeae PH-1	XP_382966.1	4 E-43	390/423
Eukaryota	Fungi	Neurospora crassa	CAE81964.1	7 E-42	432/423
Eukaryota	Fungi	Verticillium albo-atrum VaMs.102	EEY15798.1	8 E-42	415/423
Eukaryota	Fungi	Magnaporthe grisea 70-15	XP_001522074.1	1 E-38	410/423
Eukaryota	Fungi	Podospora anserina DSM 980	XP_001906531.1	2 E-36	418/423
Eukaryota	Fungi	Magnaporthe grisea 70-15	XP_364180.2	5 E-32	341/423
Eukaryota	Fungi	Neurospora crassa OR74A	XP_965496.2	9 E-30	392/423

AFUA_1G07750

Eukaryota	Fungi	Aspergillus fumigatus Af293	XP_750521.1	0.0	377/377
Eukaryota	Fungi	Neosartorya fischeri NRRL 181	XP_001264894.1	0.0	377/377
Eukaryota	Fungi	Aspergillus clavatus NRRL 1	XP_001269388.1	1 E-176	377/377
Eukaryota	Fungi	Aspergillus niger CBS 513.88	XP_001392119.1	1 E-172	374/377
Eukaryota	Fungi	Aspergillus flavus NRRL3357	XP_002376290.1	1 E-171	376/377
Eukaryota	Fungi	Aspergillus oryzae RIB40	XP_001727956.1	1 E-170	376/377
Eukaryota	Fungi	Aspergillus terreus NIH2624	XP_001209528.1	1 E-164	376/377
Eukaryota	Fungi	Penicillium chrysogenum Wisconsin 54-1255	XP_002557354.1	1 E-155	369/377
Eukaryota	Fungi	Aspergillus nidulans FGSC A4	tpeCBF80856.1	1 E-153	375/377
Eukaryota	Fungi	Aspergillus nidulans FGSC A4	XP_662703.1	1 E-147	366/377
Eukaryota	Fungi	Talaromyces stipitatus ATCC 10500	XP_002485050.1	1 E-141	370/377
Eukaryota	Fungi	Penicillium marneffei ATCC 18224	XP_002149132.1	1 E-140	371/377
Eukaryota	Fungi	Ajellomyces capsulatus NAm1	XP_001544991.1	1 E-131	371/377
Eukaryota	Fungi	Ajellomyces dermatitidis SLH14081	XP_002622839.1	1 E-128	369/377
Eukaryota	Fungi	Paracoccidioides brasiliensis Pb01;	EEH38709.1	1 E-127	369/377
Eukaryota	Fungi	Ajellomyces dermatitidis ER-3	EEQ90545.1	1 E-127	369/377
Eukaryota	Fungi	Paracoccidioides brasiliensis Pb03;	EEH23172.1	1 E-127	369/377
Eukaryota	Fungi	Ajellomyces capsulatus G186AR	EEH02561.1	1 E-126	371/377
Eukaryota	Fungi	Uncinocarpus reesii 1704	XP_002543656.1	1 E-105	363/377
Eukaryota	Fungi	Coccidioides posadasii C735 delta	EER29062.1	1 E-100	366/377
Eukaryota	Fungi	Coccidioides immitis RS;	XP_001248791.1	1 E-100	366/377
Eukaryota	Fungi	Microsporum canis CBS 113480	EEQ32457.1	6 E-98	380/377
Eukaryota	Fungi	Phaeosphaeria nodorum SN15	XP_001792386.1	1 E-67	366/377
Eukaryota	Fungi	Pyrenophora tritici-repentis Pt-1C-BFP	XP_001934355.1	6 E-67	367/377
Eukaryota	Fungi	Sclerotinia sclerotiorum 1980 UF-70	XP_001595212.1	1 E-50	344/377
Eukaryota	Fungi	Verticillium albo-atrum VaMs.102	EEY15795.1	7 E-50	353/377
Eukaryota	Fungi	Gibberella zeae PH-1	XP_382967.1	3 E-48	369/377
Eukaryota	Fungi	Neurospora crassa OR74A	XP_965493.2	1 E-46	365/377
Eukaryota	Fungi	Neurospora crassa	CAE81961.1	1 E-46	365/377

AFUA_1G10012

Eukaryota	Fungi	Aspergillus fumigatus Af293	XP_001481719.1	0.0	1929/1929
Eukaryota	Fungi	Neosartorya fischeri NRRL 181	XP_001264767.1	0.0	1638/1929

AFUA_1G10110

Eukaryota	Fungi	Aspergillus fumigatus Af293	XP_752377.1	1 E-158	279/279
Eukaryota	Fungi	Neosartorya fischeri NRRL 181	XP_001264758.1	1 E-156	279/279
Eukaryota	Fungi	Aspergillus clavatus NRRL 1	XP_001269251.1	1 E-147	279/279
Eukaryota	Fungi	Aspergillus oryzae RIB40	XP_001825346.1	1 E-144	277/279
Eukaryota	Fungi	Ajellomyces dermatitidis ER-3	EEQ90370.1	1 E-131	279/279
Eukaryota	Fungi	Aspergillus niger CBS 513.88	XP_001392314.1	1 E-131	277/279
Eukaryota	Fungi	Ajellomyces dermatitidis SLH14081	XP_002623801.1	1 E-131	279/279
Eukaryota	Fungi	Ajellomyces capsulatus G186AR	EEH07962.1	1 E-130	279/279
Eukaryota	Fungi	Ajellomyces capsulatus NAm1	XP_001541273.1	1 E-129	279/279
Eukaryota	Fungi	Talaromyces stipitatus ATCC 10500	XP_002341450.1	1 E-129	279/279
Eukaryota	Fungi	Ajellomyces capsulatus H143	EER42929.1	1 E-125	297/279
Eukaryota	Fungi	Penicillium marneffei ATCC 18224	XP_002151588.1	1 E-119	279/279
Eukaryota	Fungi	Botryotinia fuckeliana B05.10	XP_001555089.1	1 E-114	277/279
Eukaryota	Fungi	Aspergillus nidulans FGSC A4	tpeCBF84626.1	1 E-113	279/279
Eukaryota	Fungi	Sclerotinia sclerotiorum 1980 UF-70	XP_001598469.1	1 E-113	277/279
Bacteria	Chloroflexi	Thermomicrobium roseum DSM 5159	YP_002523360.1	1 E-111	274/279
Bacteria	Firmicutes	Geobacillus sp. Y412MC10	YP_003245296.1	1 E-109	270/279
Eukaryota	Fungi	Neurospora crassa OR74A	XP_964780.1	1 E-109	275/279
Eukaryota	Fungi	Penicillium chrysogenum Wisconsin 54-1255	XP_002558911.1	1 E-109	279/279
Eukaryota	Fungi	Phaeosphaeria nodorum SN15	XP_001799812.1	1 E-109	271/279
Bacteria	Firmicutes	Alkaliphilus oremlandii OhILAs	YP_001513745.1	1 E-107	271/279
Eukaryota	Viridiplantae	Vitis vinifera	CAO48327.1	1 E-106	279/279
Eukaryota	Viridiplantae	Vitis vinifera	XP_002277083.1	1 E-106	279/279
Eukaryota	Viridiplantae	Populus trichocarpa	XP_002310260.1	1 E-104	279/279
Bacteria	Synergistetes	Anaerobaculum hydrogeniformans ATCC BAA	ZP_05798349.1	1 E-104	273/279
Bacteria	Firmicutes	Clostridium tetani E88	NP_782382.1	1 E-104	273/279
Eukaryota	Fungi	Aspergillus nidulans FGSC A4	XP_682201.1	1 E-104	250/279
Eukaryota	Viridiplantae	Solanum lycopersicum	BAF75725.1	1 E-103	279/279
Eukaryota	Viridiplantae	Musa acuminata	ABF70043.1	1 E-103	272/279
Eukaryota	Viridiplantae	Physcomitrella patens subsp. patens	XP_001753439.1	1 E-103	272/279
Eukaryota	Viridiplantae	Solanum lycopersicum	BAF75724.1	1 E-103	279/279
Bacteria	Firmicutes	Coprothermobacter proteolyticus DSM 5265	YP_002247840.1	1 E-102	272/279
Bacteria	Firmicutes	Clostridium botulinum E1 str.	ZP_04821056.1	1 E-101	270/279
Eukaryota	Fungi	Ustilago maydis 521	XP_758186.1	1 E-101	274/279
Bacteria	Firmicutes	Clostridium botulinum B str.	YP_001886225.1	1 E-101	270/279
Bacteria	Firmicutes	Clostridium botulinum E3 str.	YP_001920959.1	1 E-100	270/279
Eukaryota	Viridiplantae	Oryza sativa Japonica Group	NP_001057623.1	1 E-100	279/279
Eukaryota	Viridiplantae	Thellungiella halophila	ABB45848.1	1 E-100	272/279
Bacteria	Chloroflexi	Roseiflexus sp. RS-1	YP_001277094.1	1 E-100	272/279
Eukaryota	Viridiplantae	Oryza sativa Indica Group	EAZ00916.1	1 E-100	279/279
Bacteria	Firmicutes	Clostridium botulinum A2 str.	YP_002803958.1	1 E-99	270/279
Bacteria	Firmicutes	Clostridium sporogenes ATCC 15579	ZP_02996290.1	2 E-99	270/279
Bacteria	Firmicutes	Clostridium botulinum NCTC 2916	ZP_02614852.1	2 E-99	270/279
Bacteria	Firmicutes	Clostridium botulinum F str.	YP_001390878.1	3 E-99	270/279
Bacteria	Proteobacteria	Sagittula stellata E-37	ZP_01746881.1	3 E-99	272/279
Bacteria	Firmicutes	Clostridium botulinum A3 str.	YP_001786957.1	3 E-99	270/279
Eukaryota	Fungi	Podospora anserina DSM 980	XP_001907065.1	4 E-99	284/279
Bacteria	Firmicutes	Clostridium botulinum A str.	YP_001254045.1	6 E-99	270/279
Bacteria	Proteobacteria	Chelativorans sp. BNC1	YP_676251.1	7 E-99	267/279
Bacteria	Chloroflexi	Roseiflexus castenholzii DSM 13941	YP_001432496.1	6 E-98	272/279
Eukaryota	Viridiplantae	Sorghum bicolor;	XP_002438251.1	2 E-97	271/279
Archaea	Euryarchaeota	Haloarcula marismortui ATCC 43049	YP_138077.1	1 E-96	275/279
Eukaryota	Viridiplantae	Zea mays Spermatophyta;	ACN34091.1	1 E-96	271/279

Bacteria	Firmicutes	Natranaerobius thermophilus JW/NM-WN-LF	YP_001917054.1	3 E-96	272/279
Bacteria	Firmicutes	Clostridium sp. M62/1	ZP_03733757.1	8 E-96	271/279
Bacteria	Firmicutes	Clostridium novyi NT	YP_878718.1	3 E-95	271/279
Bacteria	Firmicutes	Clostridium botulinum C str.	ZP_02621117.1	3 E-95	271/279
Eukaryota	Viridiplantae	Arabidopsis thaliana	NP_001032163.1	3 E-95	272/279
Eukaryota	Viridiplantae	Arabidopsis thaliana	NP_569034.1	4 E-95	272/279
Bacteria	Actinobacteria	Saccharopolyspora erythraea NRRL 2338	YP_001107805.1	1 E-94	273/279
Bacteria	Proteobacteria	Providencia rettgeri DSM 1131	ZP_03638105.1	2 E-94	270/279
Eukaryota	Viridiplantae	Arabidopsis thaliana	BAF01733.1	6 E-94	246/279
Bacteria	Planctomycetes	Gemmata obscuriglobus UQM 2246	ZP_02731933.1	2 E-93	260/279
Bacteria	Proteobacteria	Yersinia aldovae ATCC 35236	ZP_04621320.1	2 E-93	275/279
Bacteria	Proteobacteria	Yersinia enterocolitica subsp. enterocolitica	YP_001004587.1	2 E-93	275/279
Bacteria	Proteobacteria	Pectobacterium wasabiae WPP163	YP_003260758.1	3 E-93	271/279
Bacteria	Proteobacteria	Bradyrhizobium sp. ORS278	YP_001206724.1	5 E-93	277/279
Bacteria	Proteobacteria	Burkholderia thailandensis Bt4	ZP_02386859.1	5 E-93	262/279
Bacteria	Proteobacteria	Pectobacterium carotovorum subsp. brasiliensis	ZP_03827342.1	5 E-93	276/279
Bacteria	Proteobacteria	Providencia alcalifaciens DSM 30120	ZP_03319452.1	7 E-93	270/279
Bacteria	Proteobacteria	Yersinia kristensenii ATCC 33638	ZP_04624578.1	7 E-93	275/279
Bacteria	Proteobacteria	Pectobacterium carotovorum subsp. carotovorur	YP_003016463.1	8 E-93	276/279
Bacteria	Proteobacteria	Pectobacterium atrosepticum SCRI1043	YP_049078.1	8 E-93	271/279
Bacteria	Proteobacteria	Photorhabdus luminescens subsp. laumondii	NP_928345.1	8 E-93	269/279
Bacteria	Proteobacteria	Dickeya dadantii Ech703	YP_002988625.1	1 E-92	270/279
Bacteria	Proteobacteria	Yersinia rohdei ATCC 43380	ZP_04612186.1	2 E-92	275/279
Bacteria	Proteobacteria	Providencia stuartii ATCC 25827	ZP_02961608.1	2 E-92	271/279
Bacteria	Synergistetes	Dethiosulfovibrio peptidovorans DSM 11002	ZP_04340527.1	3 E-92	269/279
Bacteria	Proteobacteria	Yersinia intermedia ATCC 29909	ZP_04637195.1	4 E-92	275/279
Bacteria	Proteobacteria	Yersinia pestis KIM	NP_667730.1	4 E-92	274/279
Bacteria	Proteobacteria	Yersinia pseudotuberculosis IP 31758	YP_001399209.1	4 E-92	274/279
Bacteria	Proteobacteria	Yersinia mollaretii ATCC 43969	ZP_04642071.1	6 E-92	275/279
Bacteria	Proteobacteria	Yersinia bercovieri ATCC 43970	ZP_04630147.1	1 E-91	275/279
Bacteria	Proteobacteria	Providencia rustigianii DSM 4541	ZP_05973455.1	2 E-91	270/279
Bacteria	Proteobacteria	Rhizobium etli GR56	ZP_03521541.1	2 E-91	264/279
Bacteria	Proteobacteria	Burkholderia thailandensis E264	YP_439080.1	2 E-91	273/279
Bacteria	Proteobacteria	Burkholderia thailandensis TXDOH	ZP_02370376.1	2 E-91	273/279
Bacteria	Proteobacteria	Yersinia frederiksenii ATCC 33641	ZP_04633173.1	3 E-91	275/279
Bacteria	Firmicutes	Eubacterium hallii DSM 3353	ZP_03717557.1	3 E-91	271/279
Bacteria	Proteobacteria	Bradyrhizobium sp. BTAi1	YP_001240745.1	3 E-91	277/279
Bacteria	Proteobacteria	Burkholderia pseudomallei K96243	YP_111492.1	6 E-91	270/279
Bacteria	Proteobacteria	Burkholderia pseudomallei 1710b	YP_335677.1	7 E-91	270/279
Bacteria	Proteobacteria	Mesorhizobium loti MAFF303099	NP_085906.1	9 E-91	275/279
Bacteria	Proteobacteria	Burkholderia thailandensis MSMB43	ZP_02466790.1	2 E-90	273/279
Bacteria	Proteobacteria	Burkholderia oklahomensis EO147	ZP_02359058.1	2 E-90	273/279
Bacteria	Proteobacteria	Rhizobium leguminosarum bv. viciae	YP_765482.1	3 E-90	267/279
Bacteria	Proteobacteria	Rhizobium sp. NGR234	NP_444011.1	3 E-90	273/279
Bacteria	Proteobacteria	Rhizobium etli CIAT 652	YP_001984854.1	3 E-90	271/279
Bacteria	Proteobacteria	Bordetella parapertussis 12822	NP_884088.1	5 E-90	276/279
Bacteria	Proteobacteria	Proteus mirabilis HI4320	YP_002149935.1	7 E-90	271/279
Bacteria	Proteobacteria	Proteus mirabilis ATCC 29906	ZP_03842407.1	1 E-89	271/279
Bacteria	Proteobacteria	Yersinia ruckeri ATCC 29473	ZP_04617601.1	1 E-89	270/279
Bacteria	Proteobacteria	Mesorhizobium opportunistum WSM2075	ZP_05813189.1	1 E-89	269/279
Bacteria	Proteobacteria	Rhodospirillum rubrum ATCC 11170	YP_427864.1	3 E-88	270/279
Bacteria	Proteobacteria	Bordetella pertussis Tohama I	NP_879977.1	3 E-88	276/279
Bacteria	Proteobacteria	Rhizobium leguminosarum bv. viciae	YP_765102.1	4 E-88	271/279
Bacteria	Proteobacteria	Bradyrhizobium japonicum USDA 110	NP_770021.1	8 E-88	272/279
Bacteria	Proteobacteria	Rhizobium leguminosarum bv. trifolii	YP_002973167.1	9 E-88	271/279
Bacteria	Proteobacteria	Bordetella bronchiseptica RB50	NP_889824.1	1 E-87	276/279
Bacteria	Proteobacteria	Mesorhizobium loti MAFF303099	NP_104505.1	1 E-84	260/279
Bacteria	Proteobacteria	Gluconacetobacter diazotrophicus PAl 5	YP_001600881.1	1 E-84	274/279

Eukaryota	Viridiplantae	Ricinus communis	XP_002521539.1	2 E-84	230/279
Bacteria	Proteobacteria	Gluconacetobacter diazotrophicus PAl 5	YP_002275798.1	4 E-84	274/279
Bacteria	Proteobacteria	Sinorhizobium meliloti 1021	NP_435974.1	3 E-82	271/279
Bacteria	Proteobacteria	Oceanicola batsensis HTCC2597	ZP_01000581.1	6 E-82	273/279
Bacteria	Planctomycetes	Rhodopirellula baltica SH 1	NP_869686.1	1 E-81	282/279
Bacteria	Actinobacteria	Beutenbergia cavernae DSM 12333	YP_002883800.1	6 E-78	271/279
Bacteria	Bacteroidetes	Dyadobacter fermentans DSM 18053	YP_003085944.1	7 E-78	276/279
Bacteria	Proteobacteria	Sinorhizobium medicae WSM419	YP_001314482.1	2 E-77	272/279
Bacteria	Proteobacteria	alpha proteobacterium BAL199	ZP_02189972.1	3 E-76	279/279
Bacteria	Bacteroidetes	Algoriphagus sp. PR1	ZP_01718630.1	1 E-75	277/279
Bacteria	Verrucomicrobia	bacterium Ellin514	ZP_03632276.1	4 E-75	277/279
Bacteria	Actinobacteria	Rhodococcus jostii RHA1	YP_707418.1	1 E-74	262/279
Bacteria	Bacteroidetes	Flavobacteriales bacterium HTCC2170	ZP_01107245.1	1 E-72	278/279
Bacteria	Verrucomicrobia	Verrucomicrobium spinosum DSM 4136	ZP_02927427.1	6 E-71	278/279
Bacteria	Verrucomicrobia	Chthoniobacter flavus Ellin428	ZP_03127927.1	5 E-69	274/279
Bacteria	Firmicutes	Clostridium asparagiforme DSM 15981	ZP_03756710.1	8 E-62	277/279
Bacteria	Firmicutes	Anaerotruncus colihominis DSM 17241	ZP_02441441.1	2 E-61	267/279
Bacteria	Firmicutes	Geobacillus sp. Y412MC10	YP_003245298.1	2 E-59	269/279
Bacteria	Firmicutes	Clostridium botulinum A3 str.	YP_001786955.1	9 E-59	268/279
Bacteria	Firmicutes	Clostridium botulinum Bf	ZP_02617123.1	6 E-58	268/279
Bacteria	Firmicutes	Clostridium botulinum A str.	YP_001254043.1	6 E-58	268/279
Bacteria	Firmicutes	Clostridium botulinum Ba4 str.	YP_002862421.1	8 E-58	268/279
Bacteria	Firmicutes	Clostridium botulinum F str.	YP_001390876.1	8 E-58	268/279
Bacteria	Firmicutes	Clostridium botulinum A str.	YP_001383878.1	8 E-58	268/279
Bacteria	Firmicutes	Clostridium sporogenes ATCC 15579	ZP_02996292.1	9 E-58	268/279
Bacteria	Firmicutes	Clostridium botulinum B1 str.	YP_001781166.1	1 E-57	268/279
Bacteria	Firmicutes	Clostridium botulinum A2 str.	YP_002803956.1	7 E-57	268/279
Bacteria	Firmicutes	Clostridium botulinum E1 str.	ZP_04823538.1	4 E-56	268/279
Bacteria	Firmicutes	Clostridium botulinum E3 str.	YP_001920957.1	7 E-56	268/279
Bacteria	Firmicutes	Clostridium novyi NT	YP_878720.1	2 E-55	265/279
Bacteria	Firmicutes	Clostridium botulinum C str.	ZP_02621119.1	6 E-54	265/279
Bacteria	Firmicutes	Clostridium botulinum B str.	YP_001886227.1	8 E-53	267/279
Bacteria	Firmicutes	Eubacterium hallii DSM 3353	ZP_03717559.1	4 E-50	267/279
Bacteria	Firmicutes	Clostridium hylemonae DSM 15053	ZP_03777549.1	4 E-47	275/279
Bacteria	Firmicutes	Clostridium hylemonae DSM 15053	ZP_03777547.1	2 E-45	275/279
Bacteria	Firmicutes	Clostridium tetani E88	NP_782385.1	9 E-45	269/279
Bacteria	Proteobacteria	Rhizobium sp. NGR234	NP_444014.1	1 E-39	267/279
Bacteria	Proteobacteria	Burkholderia phymatum STM815	YP_001863452.1	1 E-39	270/279
Bacteria	Proteobacteria	Mesorhizobium loti MAFF303099	NP_085903.1	1 E-39	265/279
Bacteria	Proteobacteria	Bradyrhizobium japonicum USDA 110	NP_768536.1	3 E-39	255/279
Bacteria	Firmicutes	Clostridium hylemonae DSM 15053	ZP_03777550.1	9 E-36	269/279
Bacteria	Proteobacteria	Rhodobacteraceae bacterium KLH11	ZP_05124866.1	3 E-35	276/279
Bacteria	Proteobacteria	Oceanicola granulosus HTCC2516	ZP_01156059.1	9 E-35	277/279
Bacteria	Firmicutes	Clostridium hylemonae DSM 15053	ZP_03777551.1	1 E-34	253/279
Bacteria	Proteobacteria	Oceanicola batsensis HTCC2597	ZP_01000573.1	3 E-25	243/279
Bacteria	Firmicutes	Clostridium hylemonae DSM 15053	ZP_03777546.1	3 E-23	241/279
Bacteria	Firmicutes	Clostridium hylemonae DSM 15053	ZP_03777545.1	6 E-20	263/279

AFUA_1G11060

Eukaryota	Fungi	Aspergillus fumigatus Af293	XP_752471.1	0.0	346/346
Eukaryota	Fungi	Neosartorya fischeri NRRL 181	XP_001264663.1	1 E-160	310/346
Eukaryota	Fungi	Aspergillus clavatus NRRL 1	XP_001269160.1	1 E-133	311/346
Eukaryota	Fungi	Aspergillus oryzae RIB40	XP_001825242.1	1 E-107	295/346
Eukaryota	Fungi	Aspergillus flavus NRRL3357	XP_002380409.1	1 E-105	289/346
Eukaryota	Fungi	Aspergillus niger CBS 513.88	XP_001392448.1	1 E-98	292/346
Eukaryota	Fungi	Penicillium marneffei ATCC 18224	XP_002151430.1	1 E-82	298/346
Eukaryota	Fungi	Talaromyces stipitatus ATCC 10500	XP_002341547.1	6 E-77	300/346

Eukaryota	Fungi	Aspergillus nidulans FGSC A4	XP_658777.1	2 E-74	350/346
Eukaryota	Fungi	Coccidioides posadasii C735 delta	EER27988.1	7 E-74	309/346
Eukaryota	Fungi	Coccidioides immitis RS;	XP_001243042.1	3 E-72	307/346
Eukaryota	Fungi	Paracoccidioides brasiliensis Pb01;	EEH41444.1	4 E-71	295/346
Eukaryota	Fungi	Paracoccidioides brasiliensis Pb03;	EEH17901.1	6 E-70	278/346
Eukaryota	Fungi	Ajellomyces dermatitidis SLH14081	XP_002629016.1	1 E-66	296/346
Eukaryota	Fungi	Ajellomyces dermatitidis ER-3	EEQ85152.1	1 E-66	296/346
Eukaryota	Fungi	Podospora anserina DSM 980	XP_001910410.1	4 E-46	302/346
Eukaryota	Fungi	Neurospora crassa	CAF06043.1	6 E-41	297/346

AFUA_1G11280

Eukaryota	Fungi	Aspergillus fumigatus Af293	XP_752493.1	0.0	357/357
Eukaryota	Fungi	Neosartorya fischeri NRRL 181	XP_001264637.1	0.0	355/357
Eukaryota	Fungi	Aspergillus clavatus NRRL 1	XP_001270453.1	0.0	354/357
Eukaryota	Fungi	Uncinocarpus reesii 1704	XP_002543444.1	2 E-67	315/357
Eukaryota	Fungi	Aspergillus oryzae RIB40	XP_001818761.1	5 E-67	315/357
Eukaryota	Fungi	Aspergillus flavus NRRL3357	XP_002380182.1	6 E-67	315/357
Eukaryota	Fungi	Pyrenophora tritici-repentis Pt-1C-BFP	XP_001940135.1	5 E-39	329/357
Eukaryota	Fungi	Phaeosphaeria nodorum SN15	XP_001801015.1	7 E-39	330/357
Eukaryota	Fungi	Microsporum canis CBS 113480	EEQ32502.1	1 E-38	329/357
Eukaryota	Fungi	Neurospora crassa OR74A	XP_957307.1	8 E-38	324/357
Eukaryota	Fungi	Nectria haematococca mpVI 77-13-4	EEU46560.1	3 E-37	325/357
Eukaryota	Fungi	Aspergillus flavus NRRL3357	XP_002381173.1	5 E-37	331/357
Eukaryota	Fungi	Aspergillus oryzae RIB40	XP_001824091.1	5 E-37	331/357
Eukaryota	Fungi	Magnaporthe grisea 70-15	XP_369972.1	3 E-35	320/357
Eukaryota	Fungi	Aspergillus niger CBS 513.88	XP_001401298.1	5 E-35	330/357
Eukaryota	Fungi	Aspergillus terreus NIH2624	XP_001211197.1	9 E-35	331/357
Eukaryota	Fungi	Aspergillus fumigatus A1163	EDP51984.1	2 E-34	335/357
Eukaryota	Fungi	Nectria haematococca mpVI 77-13-4	EEU39974.1	2 E-34	335/357
Eukaryota	Fungi	Gibberella zeae PH-1	XP_387479.1	3 E-34	300/357
Eukaryota	Fungi	Paracoccidioides brasiliensis Pb18;	EEH49576.1	3 E-34	331/357
Eukaryota	Fungi	Penicillium marneffei ATCC 18224	XP_002148285.1	8 E-34	332/357
Eukaryota	Fungi	Paracoccidioides brasiliensis Pb03;	EEH22783.1	1 E-33	331/357
Bacteria	Proteobacteria	Caulobacter crescentus CB15	NP_419019.1	1 E-33	289/357
Eukaryota	Fungi	Neosartorya fischeri NRRL 181	XP_001259450.1	2 E-33	335/357
Eukaryota	Fungi	Talaromyces stipitatus ATCC 10500	XP_002482550.1	3 E-33	334/357
Eukaryota	Fungi	Aspergillus fumigatus Af293	XP_753405.1	4 E-33	335/357
Eukaryota	Fungi	Ustilago maydis 521	XP_760526.1	5 E-33	323/357
Eukaryota	Fungi	Ajellomyces capsulatus G186AR	EEH10013.1	1 E-32	333/357
Eukaryota	Fungi	Aspergillus nidulans FGSC A4	XP_681222.1	1 E-32	306/357
Eukaryota	Fungi	Laccaria bicolor S238N-H82	XP_001877061.1	1 E-32	324/357
Eukaryota	Fungi	Chaetomium globosum CBS 148.51	XP_001221774.1	3 E-32	341/357
Eukaryota	Fungi	Ajellomyces capsulatus H143	EER38854.1	3 E-32	333/357
Bacteria	Bacteroidetes	Flavobacterium johnsoniae UW101	YP_001195180.1	4 E-32	302/357
Eukaryota	Fungi	Ajellomyces dermatitidis SLH14081	XP_002621200.1	4 E-32	336/357
Eukaryota	Fungi	Sclerotinia sclerotiorum 1980 UF-70	XP_001595389.1	6 E-32	335/357
Eukaryota	Fungi	Paracoccidioides brasiliensis Pb01;	EEH40455.1	1 E-31	298/357
Eukaryota	Fungi	Penicillium chrysogenum Wisconsin 54-1255	XP_002558179.1	1 E-31	303/357
Eukaryota	Fungi	Ajellomyces capsulatus NAm1	XP_001542405.1	1 E-31	309/357
Eukaryota	Fungi	Ajellomyces capsulatus H143	EER45337.1	2 E-31	337/357
Eukaryota	Fungi	Aspergillus clavatus NRRL 1	XP_001274537.1	2 E-31	308/357
Eukaryota	Fungi	Paracoccidioides brasiliensis Pb01;	EEH41533.1	2 E-31	328/357
Eukaryota	Fungi	Coprinopsis cinerea okayama7#130	XP_001840903.1	4 E-31	306/357
Eukaryota	Fungi	Paracoccidioides brasiliensis Pb03;	EEH17985.1	6 E-31	328/357
Bacteria	Proteobacteria	Halothiobacillus neapolitanus c2	ZP_03739799.1	1 E-30	307/357
Eukaryota	Viridiplantae	Oryza sativa Japonica Group	AAM92286.1	2 E-30	301/357
Eukaryota	Viridiplantae	Micromonas pusilla CCMP1545	EEH54541.1	2 E-30	298/357

Bacteria	Bacteroidetes	Kordia algicida OT-1	ZP_02163991.1	2 E-30	309/357
Eukaryota	Fungi	Aspergillus oryzae RIB40	XP_001816906.1	2 E-30	336/357
Eukaryota	Viridiplantae	Oryza sativa Japonica Group	AAO00709.1	3 E-30	301/357
Eukaryota	Fungi	Aspergillus nidulans FGSC A4	XP_661276.1	3 E-30	333/357
Eukaryota	Fungi	Ajellomyces dermatitidis ER-3	EEQ90020.1	3 E-30	312/357
Bacteria	Proteobacteria	Phenylobacterium zucineum HLK1	YP_002129160.1	4 E-30	290/357
Eukaryota	Viridiplantae	Oryza sativa Indica Group	EAY79254.1	4 E-30	303/357
Bacteria	Proteobacteria	Hirschia baltica ATCC 49814	YP_003060355.1	4 E-30	288/357
Eukaryota	Fungi	Phaeosphaeria nodorum SN15	XP_001805938.1	5 E-30	345/357
Eukaryota	Fungi	Magnaporthe grisea 70-15	XP_364516.1	8 E-30	370/357
Eukaryota	Fungi	Penicillium chrysogenum Wisconsin 54-1255	XP_002567813.1	8 E-30	332/357
Eukaryota	Fungi	Ajellomyces dermatitidis SLH14081	XP_002627660.1	8 E-30	328/357
Eukaryota	Fungi	Penicillium chrysogenum Wisconsin 54-1255	XP_002564582.1	9 E-30	330/357
Eukaryota	Fungi	Ajellomyces dermatitidis ER-3	EEQ88111.1	9 E-30	328/357
Eukaryota	stramenopiles	Thalassiosira pseudonana CCMP1335	XP_002286453.1	2 E-29	294/357
Bacteria	Proteobacteria	Leptothrix cholodnii SP-6	YP_001793373.1	2 E-29	321/357
Eukaryota	Fungi	Pyrenophora tritici-repentis Pt-1C-BFP	XP_001936794.1	3 E-29	318/357
Eukaryota	Fungi	Aspergillus nidulans FGSC A4	tpeCBF90299.1	3 E-29	330/357
Eukaryota	Fungi	Aspergillus nidulans FGSC A4	XP_657655.1	4 E-29	330/357
Eukaryota	Fungi	Aspergillus oryzae RIB40	XP_001827566.1	4 E-29	303/357
Bacteria	Bacteroidetes	Polaribacter sp. MED152	ZP_01051787.1	4 E-29	299/357
Bacteria	Proteobacteria	Erythrobacter sp. NAP1	ZP_01041307.1	6 E-29	291/357
Eukaryota	Viridiplantae	Oryza sativa Japonica Group	ABG66202.1	6 E-29	303/357
Eukaryota	Fungi	Coccidioides immitis RS;	XP_001243162.1	7 E-29	328/357
Eukaryota	Fungi	Coccidioides posadasii C735 delta	EER28065.1	7 E-29	329/357
Eukaryota	Viridiplantae	Oryza sativa Japonica Group	NP_001065102.1	7 E-29	301/357
Bacteria	Proteobacteria	Maricaulis maris MCS10	YP_756928.1	8 E-29	286/357
Eukaryota	Viridiplantae	Oryza sativa Japonica Group	AAO00707.2	8 E-29	303/357
Bacteria	Bacteroidetes	Chitinophaga pinensis DSM 2588	YP_003120701.1	1 E-28	311/357
Eukaryota	Viridiplantae	Sorghum bicolor;	XP_002467628.1	1 E-28	303/357
Bacteria	Bacteroidetes	Dokdonia donghaensis MED134	ZP_01048784.1	2 E-28	287/357
Bacteria	Proteobacteria	Pseudomonas putida W619	YP_001749855.1	2 E-28	309/357
Bacteria	Proteobacteria	Sphingopyxis alaskensis RB2256	YP_616090.1	2 E-28	295/357
Eukaryota	Fungi	Ajellomyces capsulatus G186AR	EEH07197.1	2 E-28	309/357
Eukaryota	Viridiplantae	Arabidopsis thaliana	CAB88075.1	2 E-28	299/357
Bacteria	Bacteroidetes	Flavobacterium psychrophilum JIP02/86	YP_001295934.1	3 E-28	294/357
Eukaryota	Fungi	Aspergillus terreus NIH2624	XP_001217720.1	3 E-28	296/357
Bacteria	Proteobacteria	Xanthomonas oryzae pv. oryzicola	ZP_02243829.1	4 E-28	288/357
Eukaryota	Fungi	Botryotinia fuckeliana B05.10	XP_001553508.1	4 E-28	335/357
Eukaryota	Fungi	Gibberella zeae PH-1	XP_383389.1	8 E-28	303/357
Bacteria	Proteobacteria	Plesiocystis pacifica SIR-1	ZP_01905970.1	1 E-27	293/357
Bacteria	Proteobacteria	Erythrobacter sp. SD-21	ZP_01864714.1	1 E-27	290/357
Eukaryota	Euglenozoa	Trypanosoma cruzi strain CL	XP_804402.1	2 E-27	313/357
Bacteria	Proteobacteria	Xanthomonas campestris pv. vesicatoria	YP_364593.1	3 E-27	288/357
Bacteria	Proteobacteria	Xanthomonas oryzae pv. oryzae	YP_452105.1	4 E-27	288/357
Eukaryota	Euglenozoa	Trypanosoma cruzi strain CL	XP_818816.1	4 E-27	313/357
Bacteria	Bacteroidetes	Microscilla marina ATCC 23134	ZP_01691038.1	4 E-27	312/357
Bacteria	Proteobacteria	Xanthomonas oryzae pv. oryzae	AAW76502.1	5 E-27	288/357
Bacteria	Chloroflexi	Dehalococcoides sp. VS	ZP_02205064.1	5 E-27	288/357
Bacteria	Proteobacteria	Pseudomonas aeruginosa PA7	YP_001345621.1	6 E-27	310/357
Bacteria	Proteobacteria	Xanthomonas axonopodis pv. citri	NP_643022.2	6 E-27	288/357
Bacteria	Proteobacteria	Xanthomonas axonopodis pv. citri	AAM37558.1	6 E-27	288/357
Bacteria	Bacteroidetes	Dyadobacter fermentans DSM 18053	YP_003087087.1	7 E-27	307/357
Eukaryota	Fungi	Aspergillus niger CBS 513.88	XP_001389200.1	7 E-27	332/357
Bacteria	Proteobacteria	Xanthomonas campestris pv. campestris	NP_637886.1	7 E-27	288/357
Eukaryota	Fungi	Nectria haematococca mpVI 77-13-4	EEU41744.1	9 E-27	329/357
Eukaryota	Viridiplantae	Micromonas sp. RCC299	XP_002502782.1	1 E-26	298/357
Bacteria	Proteobacteria	Pseudomonas aeruginosa UCBPP-PA14	YP_788305.1	1 E-26	309/357

Eukaryota	Fungi	Talaromyces stipitatus ATCC 10500	XP_002486355.1	1 E-26	302/357
Eukaryota	Viridiplantae	Ostreococcus lucimarinus CCE9901	XP_001420626.1	2 E-26	289/357
Bacteria	Cyanobacteria	Synechococcus sp. PCC 7335	ZP_05036376.1	2 E-26	310/357
Bacteria	Proteobacteria	Pseudomonas aeruginosa PAO1	NP_248837.1	3 E-26	310/357
Bacteria	Proteobacteria	Asticcacaulis excentricus CB 48	ZP_04772073.1	3 E-26	297/357
Bacteria	Proteobacteria	Stenotrophomonas maltophilia K279a	YP_001973126.1	3 E-26	288/357
Bacteria	Cyanobacteria	Nostoc punctiforme PCC 73102	YP_001865549.1	3 E-26	299/357
Bacteria	Proteobacteria	Oceanicaulis alexandrii HTCC2633	ZP_00957888.1	3 E-26	287/357
Eukaryota	Viridiplantae	Populus trichocarpa	XP_002325020.1	4 E-26	294/357
Bacteria	Proteobacteria	Pseudomonas aeruginosa PACS2	ZP_01363056.1	4 E-26	309/357
Eukaryota	Fungi	Laccaria bicolor S238N-H82	XP_001886260.1	4 E-26	288/357
Bacteria	Proteobacteria	Novosphingobium aromaticivorans DSM 12444	YP_496608.1	5 E-26	302/357
Bacteria	Actinobacteria	Mycobacterium smegmatis str. MC2	YP_888294.1	5 E-26	328/357
Bacteria	Bacteroidetes	Algoriphagus sp. PR1	ZP_01719716.1	6 E-26	293/357
Eukaryota	Metazoa	Branchiostoma floridae	XP_002611171.1	7 E-26	311/357
Eukaryota	Fungi	Aspergillus nidulans FGSC A4	XP_659994.1	7 E-26	341/357
Eukaryota	Viridiplantae	Vitis vinifera	CAO65454.1	8 E-26	292/357
Bacteria	Proteobacteria	Rhodobacterales bacterium HTCC2255	ZP_01446741.1	8 E-26	300/357
Eukaryota	Viridiplantae	Vitis vinifera	XP_002283713.1	1 E-25	292/357
Bacteria	Proteobacteria	Pseudomonas stutzeri A1501	YP_001173562.1	1 E-25	310/357
Bacteria	Proteobacteria	Erythrobacter litoralis HTCC2594	YP_459125.1	2 E-25	290/357
Bacteria	Bacteroidetes	Flavobacteriales bacterium HTCC2170	ZP_01106734.1	2 E-25	294/357
Bacteria	Bacteroidetes	Robiginitalea biformata HTCC2501	YP_003195926.1	2 E-25	306/357
Eukaryota	Viridiplantae	Populus trichocarpa	ABK95132.1	2 E-25	303/357
Bacteria	Proteobacteria	Stenotrophomonas maltophilia R551-3	YP_002029217.1	2 E-25	288/357
Bacteria	Actinobacteria	Frankia alni ACN14a	YP_715504.1	2 E-25	292/357
Eukaryota	stramenopiles	Phaeodactylum tricornutum CCAP 1055/1	XP_002176333.1	3 E-25	310/357
Bacteria	Proteobacteria	Stenotrophomonas sp. SKA14	ZP_05134618.1	3 E-25	288/357
Bacteria	Proteobacteria	Mesorhizobium opportunistum WSM2075	ZP_05810888.1	4 E-25	315/357
Eukaryota	Fungi	Cryptococcus neoformans var. neoformans	XP_571513.1	4 E-25	294/357
Eukaryota	Viridiplantae	Ostreococcus tauri	CAL57265.1	4 E-25	311/357
Bacteria	Proteobacteria	Congregibacter litoralis KT71	ZP_01104703.1	5 E-25	300/357
Eukaryota	Fungi	Cryptococcus neoformans var. neoformans	XP_774654.1	5 E-25	294/357
Eukaryota	Metazoa	Danio rerio	XP_001341121.2	6 E-25	313/357
Bacteria	Proteobacteria	Methylobacterium populi BJ001	YP_001926990.1	6 E-25	322/357
Eukaryota	Metazoa	Danio rerio	CAN88419.1	6 E-25	313/357
Eukaryota	Metazoa	Danio rerio	NP_001096105.1	7 E-25	315/357
Bacteria	Bacteroidetes	Chryseobacterium gleum ATCC 35910	ZP_03850961.1	7 E-25	294/357
Eukaryota	Fungi	Rhodotorula glutinis	ACR66220.1	7 E-25	301/357
Eukaryota	Viridiplantae	Physcomitrella patens subsp. patens	XP_001774706.1	8 E-25	293/357
Bacteria	Bacteroidetes	Flavobacteriaceae bacterium 3519-10	YP_003096948.1	8 E-25	294/357
Bacteria	Proteobacteria	Burkholderia sp. H160	ZP_03265246.1	8 E-25	305/357
Eukaryota	Viridiplantae	Glycine max	ACU21218.1	1 E-24	314/357
Eukaryota	Viridiplantae	Populus trichocarpa	XP_002304292.1	1 E-24	303/357
Eukaryota	Fungi	Podospora anserina DSM 980	XP_001908396.1	2 E-24	322/357
Bacteria	Proteobacteria	Burkholderia graminis C4D1M	ZP_02886980.1	3 E-24	305/357
Bacteria	Cyanobacteria	Planktothrix rubescens NIVA-CYA 98	CAQ48273.1	3 E-24	300/357
Bacteria	Cyanobacteria	Synechococcus sp. JA-3-3Ab	YP_474174.1	3 E-24	301/357
Bacteria	Proteobacteria	Pseudomonas fluorescens Pf-5	YP_257862.1	4 E-24	304/357
Eukaryota	Fungi	Penicillium marneffei ATCC 18224	XP_002146999.1	5 E-24	311/357
Bacteria	Proteobacteria	Pseudoalteromonas tunicata D2	ZP_01132259.1	5 E-24	286/357
Eukaryota	Viridiplantae	Vitis vinifera	XP_002283708.1	6 E-24	298/357
Bacteria	Proteobacteria	Variovorax paradoxus S110	YP_002946715.1	7 E-24	329/357
Eukaryota	Viridiplantae	Physcomitrella patens subsp. patens	XP_001761719.1	7 E-24	298/357
Eukaryota	Fungi	Rhodotorula glutinis	AAU12179.1	9 E-24	301/357
Bacteria	Proteobacteria	Pseudomonas syringae pv. syringae	YP_233770.1	1 E-23	304/357
Eukaryota	Viridiplantae	Micromonas pusilla CCMP1545	EEH57230.1	1 E-23	290/357
Bacteria	Proteobacteria	gamma proteobacterium NOR5-3	ZP_05125992.1	1 E-23	306/357

Bacteria	Proteobacteria	Pseudomonas fluorescens Pf0-1	YP_346405.1	1 E-23	304/357
Bacteria	Bacteroidetes	Flavobacteria bacterium MS024-3C	ZP_03701586.1	1 E-23	299/357
Bacteria	Proteobacteria	Pseudomonas syringae pv. phaseolicola	YP_276740.1	2 E-23	304/357
Bacteria	Proteobacteria	Marinomonas sp. MED121	ZP_01077887.1	2 E-23	298/357
Eukaryota	Viridiplantae	Glycine max	ACU18453.1	2 E-23	292/357
Eukaryota	Viridiplantae	Zea mays;	ACN26498.1	2 E-23	292/357
Eukaryota	Fungi	Coprinopsis cinerea okayama7#130	XP_001835619.1	3 E-23	303/357
Bacteria	Proteobacteria	Mariprofundus ferrooxydans PV-1	ZP_01453379.1	3 E-23	314/357
Eukaryota	Metazoa	Branchiostoma floridae	XP_002607556.1	3 E-23	304/357
Bacteria	Proteobacteria	Pseudomonas syringae pv. tabaci	ZP_05636357.1	4 E-23	304/357
Bacteria	Proteobacteria	Pseudomonas syringae pv. tomato	NP_790605.1	5 E-23	304/357
Bacteria	Proteobacteria	Thalassiobium sp. R2A62	ZP_05341107.1	7 E-23	302/357
Eukaryota	Fungi	Sclerotinia sclerotiorum 1980 UF-70	XP_001594697.1	8 E-23	337/357
Bacteria	Proteobacteria	Burkholderia phymatum STM815	YP_001861244.1	9 E-23	307/357
Bacteria	Proteobacteria	Pseudomonas stutzeri A1501	YP_001173385.1	1 E-22	302/357
Eukaryota	Viridiplantae	Oryza sativa Indica Group	EAY72839.1	2 E-22	297/357
Bacteria	Proteobacteria	Pseudomonas syringae pv. tomato	ZP_03397489.1	2 E-22	309/357
Eukaryota	Viridiplantae	Oryza sativa Japonica Group	NP_001042255.1	2 E-22	297/357
Eukaryota	Viridiplantae	Chlamydomonas reinhardtii	XP_001698645.1	2 E-22	293/357
Eukaryota	Viridiplantae	Ricinus communis	XP_002514229.1	2 E-22	319/357
Bacteria	Bacteroidetes	Pedobacter sp. BAL39	ZP_01886433.1	3 E-22	298/357
Bacteria	Proteobacteria	Halothiobacillus neapolitanus c2	ZP_03740448.1	3 E-22	298/357
Eukaryota	Fungi	Laccaria bicolor S238N-H82	XP_001876151.1	3 E-22	298/357
Eukaryota	Fungi	Talaromyces stipitatus ATCC 10500	XP_002482477.1	5 E-22	332/357
Eukaryota	Viridiplantae	Zea mays;	NP_001131262.1	6 E-22	297/357
Eukaryota	Fungi	Aspergillus oryzae RIB40	XP_001818583.1	6 E-22	299/357
Bacteria	Proteobacteria	Pseudomonas syringae pv. syringae	YP_235932.1	6 E-22	328/357
Bacteria	Proteobacteria	Pseudomonas syringae pv. oryzae	ZP_04586035.1	6 E-22	304/357
Bacteria	Proteobacteria	Hyphomonas neptunium ATCC 15444	YP_760937.1	7 E-22	286/357
Bacteria	Bacteroidetes	Pedobacter heparinus DSM 2366	YP_003090763.1	8 E-22	288/357
Eukaryota	Fungi	Magnaporthe grisea 70-15	XP_366019.1	8 E-22	320/357
Eukaryota	Viridiplantae	Capsella rubella	CAB96198.1	1 E-21	303/357
Eukaryota	Fungi	Nectria haematococca mpVI 77-13-4	EEU41585.1	1 E-21	315/357
Bacteria	Cyanobacteria	Synechococcus sp. JA-2-3B'a(2-13)	YP_477041.1	1 E-21	305/357
Bacteria	Proteobacteria	Rhizobium leguminosarum bv. viciae	YP_771645.1	1 E-21	302/357
Bacteria	Proteobacteria	Pseudomonas mendocina ymp	YP_001186859.1	2 E-21	306/357
Eukaryota	Fungi	Magnaporthe grisea 70-15	XP_361338.2	2 E-21	296/357
Eukaryota	Metazoa	Ciona intestinalis	XP_002127067.1	3 E-21	324/357
Eukaryota	Viridiplantae	Arabidopsis thaliana	NP_190233.2	3 E-21	297/357
Bacteria	Bacteroidetes	Flavobacteria bacterium BBFL7	ZP_01202453.1	3 E-21	310/357
Eukaryota	Metazoa	Branchiostoma floridae	XP_002588861.1	3 E-21	309/357
Eukaryota	Fungi	Sclerotinia sclerotiorum 1980 UF-70	XP_001588037.1	5 E-21	321/357
Bacteria	Proteobacteria	Burkholderia phytofirmans PsJN	YP_001888580.1	5 E-21	305/357
Bacteria	Proteobacteria	Pseudomonas stutzeri A1501	YP_001174054.1	5 E-21	321/357

AFUA_1G11290

Eukaryota	Fungi	Aspergillus fumigatus Af293	XP_752494.1	0.0	534/534
Eukaryota	Fungi	Neosartorya fischeri NRRL 181	XP_001264636.1	0.0	526/534
Eukaryota	Fungi	Aspergillus clavatus NRRL 1	XP_001270454.1	0.0	512/534
Eukaryota	Fungi	Aspergillus terreus NIH2624	XP_001210601.1	1 E-178	455/534
Eukaryota	Fungi	Neosartorya fischeri NRRL 181	XP_001259256.1	1 E-178	487/534
Eukaryota	Fungi	Aspergillus fumigatus Af293	XP_753234.1	1 E-169	486/534
Eukaryota	Fungi	Aspergillus fumigatus A1163	EDP52156.1	1 E-167	486/534
Eukaryota	Fungi	Microsporum canis CBS 113480	EEQ27480.1	4 E-25	434/534

AFUA_1G11300

Eukaryota	Fungi	Aspergillus fumigatus Af293	XP_752495.1	1 E-161	280/280
Eukaryota	Fungi	Neosartorya fischeri NRRL 181	XP_001264635.1	1 E-139	260/280
Eukaryota	Fungi	Aspergillus clavatus NRRL 1	XP_001270455.1	1 E-118	256/280
Eukaryota	Fungi	Uncinocarpus reesii 1704	XP_002543446.1	6 E-54	237/280
Eukaryota	Fungi	Aspergillus flavus NRRL3357	XP_002380175.1	4 E-53	242/280
Eukaryota	Fungi	Aspergillus oryzae RIB40	XP_001818754.1	5 E-52	242/280
Eukaryota	Fungi	Uncinocarpus reesii 1704	XP_002541738.1	8 E-47	234/280
Eukaryota	Fungi	Aspergillus niger CBS 513.88	XP_001391626.1	2 E-45	268/280
Eukaryota	Fungi	Chaetomium globosum CBS 148.51	XP_001223306.1	3 E-38	247/280
Eukaryota	Fungi	Aspergillus fumigatus A1163	EDP54092.1	5 E-11	231/280
Eukaryota	Fungi	Aspergillus fumigatus Af293	XP_749570.1	6 E-11	231/280

AFUA_1G11310

Eukaryota	Fungi	Aspergillus fumigatus Af293	XP_752496.2	0.0	655/655
Eukaryota	Fungi	Neosartorya fischeri NRRL 181	XP_001264634.1	0.0	567/655
Eukaryota	Fungi	Aspergillus clavatus NRRL 1	XP_001270457.1	0.0	567/655
Eukaryota	Fungi	Aspergillus flavus NRRL3357	XP_002380178.1	0.0	555/655
Eukaryota	Fungi	Microsporum canis CBS 113480	EEQ27481.1	0.0	557/655
Eukaryota	Fungi	Aspergillus oryzae RIB40	XP_001818757.1	0.0	555/655
Eukaryota	Fungi	Uncinocarpus reesii 1704	XP_002543447.1	0.0	552/655
Eukaryota	Fungi	Ustilago maydis 521	XP_757318.1	4 E-43	536/655
Eukaryota	Fungi	Pyrenophora tritici-repentis Pt-1C-BFP	XP_001932297.1	9 E-42	574/655
Eukaryota	Fungi	Aspergillus nidulans FGSC A4	XP_682485.1	1 E-40	555/655
Eukaryota	Fungi	Penicillium chrysogenum Wisconsin 54-1255	XP_002569052.1	7 E-40	560/655
Eukaryota	Fungi	Aspergillus flavus NRRL3357	XP_002382394.1	2 E-39	529/655
Eukaryota	Fungi	Cryptococcus neoformans var. neoformans	XP_568637.1	1 E-38	551/655
Eukaryota	Fungi	Penicillium chrysogenum Wisconsin 54-1255	XP_002565927.1	8 E-38	559/655
Eukaryota	Fungi	Aspergillus niger CBS 513.88	XP_001397791.1	8 E-38	564/655
Eukaryota	Fungi	Aspergillus terreus NIH2624	XP_001209307.1	8 E-38	533/655
Eukaryota	Metazoa	Luciola cruciata	BAE80729.1	7 E-37	528/655
Eukaryota	Fungi	Nectria haematococca mpVI 77-13-4	EEU43201.1	1 E-36	542/655
Eukaryota	Fungi	Aspergillus terreus NIH2624	XP_001211888.1	2 E-36	536/655
Eukaryota	Fungi	Sclerotinia sclerotiorum 1980 UF-70	XP_001586224.1	1 E-35	538/655
Eukaryota	Fungi	Aspergillus flavus NRRL3357	XP_002377908.1	2 E-35	546/655
Eukaryota	Fungi	Aspergillus oryzae RIB40	XP_001826226.1	3 E-35	546/655
Eukaryota	Fungi	Ajellomyces capsulatus G186AR	EEH04398.1	7 E-35	542/655
Eukaryota	Fungi	Penicillium chrysogenum Wisconsin 54-1255	XP_002569172.1	8 E-35	529/655
Eukaryota	Fungi	Botryotinia fuckeliana B05.10	XP_001556516.1	9 E-35	534/655
Eukaryota	Fungi	Aspergillus fumigatus A1163	EDP54539.1	1 E-34	549/655
Eukaryota	Metazoa	Branchiostoma floridae	XP_002612162.1	1 E-34	533/655
Eukaryota	Fungi	Aspergillus fumigatus Af293	XP_755349.1	1 E-34	549/655
Eukaryota	Fungi	Penicillium marneffei ATCC 18224	XP_002146968.1	1 E-34	531/655
Eukaryota	Fungi	Pyrenophora tritici-repentis Pt-1C-BFP	XP_001939087.1	2 E-34	542/655
Eukaryota	Metazoa	Nasonia vitripennis	XP_001604694.1	3 E-34	541/655
Eukaryota	Amoebozoa	Dictyostelium discoideum	Q54P79.2	4 E-34	538/655
Eukaryota	Fungi	Talaromyces stipitatus ATCC 10500	XP_002478646.1	5 E-34	549/655
Eukaryota	Metazoa	Tribolium castaneum	XP_966640.1	5 E-34	565/655
Eukaryota	Amoebozoa	Dictyostelium discoideum AX4	XP_638379.1	5 E-34	538/655
Eukaryota	Fungi	Coccidioides immitis RS;	XP_001240389.1	8 E-34	549/655
Eukaryota	Fungi	Neosartorya fischeri NRRL 181	XP_001260500.1	9 E-34	549/655
Eukaryota	Fungi	Aspergillus terreus NIH2624	XP_001213629.1	9 E-34	542/655
Eukaryota	Fungi	Coccidioides posadasii C735 delta	EER25935.1	1 E-33	549/655
Eukaryota	Fungi	Aspergillus clavatus NRRL 1	XP_001275317.1	2 E-33	564/655
Eukaryota	Fungi	Penicillium marneffei ATCC 18224	XP_002146352.1	3 E-33	548/655
Eukaryota	Fungi	Paracoccidioides brasiliensis Pb18;	EEH46276.1	3 E-33	546/655
Eukaryota	Viridiplantae	Ipomoea batatas	BAG82851.1	4 E-33	538/655
Eukaryota	Fungi	Paracoccidioides brasiliensis Pb03;	EEH19618.1	7 E-33	546/655

Eukaryota	Metazoa	Tribolium castaneum	XP_966892.1	8 E-33	546/655
Eukaryota	Fungi	Ajellomyces capsulatus NAm1	XP_001538766.1	9 E-33	525/655
Eukaryota	Fungi	Aspergillus nidulans FGSC A4	XP_663594.1	1 E-32	559/655
Eukaryota	Fungi	Neurospora crassa OR74A	XP_964221.1	2 E-32	560/655
Eukaryota	Fungi	Ajellomyces capsulatus H143	EER41905.1	2 E-32	527/655
Eukaryota	Amoebozoa	Dictyostelium discoideum AX4	XP_638381.1	7 E-32	534/655
Eukaryota	Viridiplantae	Arabidopsis thaliana	NP_173472.1	1 E-31	536/655
Eukaryota	Viridiplantae	Physcomitrella patens subsp. patens	XP_001767771.1	1 E-31	531/655
Eukaryota	Viridiplantae	Arabidopsis thaliana	AAP03016.1	2 E-31	536/655
Eukaryota	Amoebozoa	Dictyostelium discoideum AX4	XP_638380.1	3 E-31	536/655
Eukaryota	Viridiplantae	Zea mays;	ACN32032.1	3 E-31	532/655
Eukaryota	Fungi	Nectria haematococca mpVI 77-13-4	EEU37978.1	4 E-31	548/655
Eukaryota	Viridiplantae	Zea mays;	NP_001150314.1	4 E-31	532/655
Eukaryota	Fungi	Coprinopsis cinerea okayama7#130	XP_001839229.1	4 E-31	584/655
Eukaryota	Fungi	Ustilago maydis 521	XP_762300.1	9 E-31	539/655
Eukaryota	Viridiplantae	Arabidopsis thaliana	AAF79612.1	3 E-30	535/655
Eukaryota	Fungi	Aspergillus oryzae RIB40	XP_001823173.1	2 E-30	556/655
Bacteria	Actinobacteria	Gordonia bronchialis DSM 43247	ZP_03885784.1	2 E-30	535/655
Eukaryota	Fungi	Aspergillus flavus NRRL3357	XP_002380165.1	6 E-30	545/655
Eukaryota	Fungi	Ajellomyces dermatitidis SLH14081	XP_002628017.1	1 E-29	548/655
Eukaryota	Fungi	Aspergillus nidulans FGSC A4	tpeCBF89565.1	1 E-29	539/655

AFUA_1G11320

Eukaryota	Fungi	Aspergillus fumigatus Af293	XP_752497.1	0.0	450/450
Eukaryota	Fungi	Neosartorya fischeri NRRL 181	XP_001264633.1	0.0	449/450
Eukaryota	Fungi	Aspergillus clavatus NRRL 1	XP_001270458.1	0.0	439/450
Eukaryota	Fungi	Aspergillus oryzae RIB40	XP_001818759.1	1 E-138	418/450
Eukaryota	Fungi	Aspergillus flavus NRRL3357	XP_002380180.1	1 E-138	418/450
Eukaryota	Fungi	Microsporum canis CBS 113480	EEQ27479.1	1 E-135	425/450
Eukaryota	Fungi	Yarrowia lipolytica CLIB122	XP_501221.1	2 E-98	432/450
Eukaryota	Fungi	Pyrenophora tritici-repentis Pt-1C-BFP	XP_001937465.1	6 E-93	467/450
Eukaryota	Fungi	Aspergillus niger CBS 513.88	XP_001401729.1	1 E-92	435/450
Eukaryota	Fungi	Talaromyces stipitatus ATCC 10500	XP_002483705.1	1 E-91	455/450
Eukaryota	Fungi	Penicillium chrysogenum Wisconsin 54-1255	XP_002562133.1	6 E-91	442/450
Eukaryota	Fungi	Ajellomyces capsulatus G186AR	EEH02549.1	1 E-90	456/450
Eukaryota	Fungi	Penicillium marneffei ATCC 18224	XP_002150497.1	5 E-90	456/450
Eukaryota	Fungi	Ajellomyces capsulatus H143	EER45252.1	5 E-90	456/450
Eukaryota	Fungi	Laccaria bicolor S238N-H82	XP_001887866.1	5 E-90	457/450
Eukaryota	Fungi	Coccidioides posadasii C735 delta	EER22943.1	6 E-89	457/450
Eukaryota	Fungi	Neosartorya fischeri NRRL 181	XP_001267145.1	8 E-89	441/450
Eukaryota	Fungi	Uncinocarpus reesii 1704	XP_002542966.1	1 E-88	457/450
Eukaryota	Fungi	Coccidioides immitis RS;	XP_001241139.1	2 E-88	457/450
Eukaryota	Fungi	Aspergillus fumigatus Af293	XP_751948.1	2 E-88	441/450
Eukaryota	Fungi	Paracoccidioides brasiliensis Pb18;	EEH47016.1	3 E-88	465/450
Eukaryota	Fungi	Ajellomyces dermatitidis ER-3	EEQ91359.1	3 E-88	456/450
Eukaryota	Fungi	Ajellomyces dermatitidis SLH14081	XP_002629287.1	3 E-88	456/450
Eukaryota	Fungi	Microsporum canis CBS 113480	EEQ29831.1	5 E-88	467/450
Eukaryota	Fungi	Aspergillus clavatus NRRL 1	XP_001271765.1	1 E-87	440/450
Eukaryota	Fungi	Phaeosphaeria nodorum SN15	XP_001791592.1	1 E-87	445/450
Eukaryota	Fungi	Aspergillus flavus NRRL3357	XP_002376962.1	5 E-86	458/450
Eukaryota	Fungi	Aspergillus oryzae RIB40	XP_001821200.1	3 E-85	450/450
Eukaryota	Fungi	Aspergillus nidulans FGSC A4	XP_659277.1	7 E-85	462/450
Eukaryota	Fungi	Podospora anserina DSM 980	XP_001912551.1	2 E-83	449/450
Bacteria	Proteobacteria	Pseudomonas stutzeri A1501	YP_001173004.1	3 E-83	435/450
Bacteria	Candidatus	Candidatus Pelagibacter ubique HTCC1062	YP_266557.1	4 E-83	442/450
Eukaryota	Fungi	Nectria haematococca mpVI 77-13-4	EEU43927.1	4 E-83	466/450
Bacteria	Proteobacteria	Methylobacterium nodulans ORS 2060	YP_002496560.1	5 E-83	436/450

Bacteria	Candidatus	Candidatus Pelagibacter sp. HTCC7211	ZP_05070047.1	8 E-83	447/450
Bacteria	Candidatus	Candidatus Pelagibacter ubique HTCC1002	ZP_01265090.1	8 E-83	442/450
Bacteria	Actinobacteria	Saccharopolyspora erythraea NRRL 2338	YP_001107656.1	1 E-82	385/450
Bacteria	Proteobacteria	Granulibacter bethesdensis CGDNIH1	YP_745241.1	1 E-82	437/450
Eukaryota	Fungi	Neurospora crassa OR74A	XP_963941.1	2 E-82	453/450
Bacteria	Proteobacteria	Methylobacterium sp. 4-46	YP_001768562.1	4 E-82	436/450
Bacteria	Proteobacteria	Providencia rustigianii DSM 4541	ZP_05972351.1	1 E-81	429/450
Bacteria	Proteobacteria	Burkholderia glumae BGR1	YP_002908575.1	3 E-81	435/450
Bacteria	Proteobacteria	Pseudoalteromonas tunicata D2	ZP_01131880.1	3 E-81	435/450
Eukaryota	Fungi	Ajellomyces capsulatus NAm1	XP_001538589.1	3 E-81	425/450
Eukaryota	Viridiplantae	Zea mays;	ACG46388.1	5 E-81	437/450
Eukaryota	Fungi	Coprinopsis cinerea okayama7#130	XP_001829726.1	6 E-81	414/450
Bacteria	Proteobacteria	Magnetospirillum magnetotacticum MS-1	ZP_00055006.1	7 E-81	437/450
Eukaryota	Viridiplantae	Sorghum bicolor;	XP_002464917.1	1 E-80	444/450
Bacteria	Proteobacteria	Gluconobacter oxydans 621H	YP_192164.1	1 E-80	448/450
Bacteria	Proteobacteria	Idiomarina baltica OS145	ZP_01043927.1	1 E-80	434/450
Bacteria	Proteobacteria	Plesiocystis pacifica SIR-1	ZP_01912169.1	2 E-80	431/450
Bacteria	Proteobacteria	Psychromonas ingrahamii 37	YP_943109.1	2 E-80	441/450
Eukaryota	Viridiplantae	Zea mays;	NP_001140252.1	2 E-80	437/450
Bacteria	Proteobacteria	Xanthomonas campestris	AAK14074.1	2 E-80	438/450
Bacteria	Proteobacteria	Bradyrhizobium sp. BTAi1	YP_001239492.1	2 E-80	431/450
Bacteria	Proteobacteria	Nitrobacter hamburgensis X14	YP_576765.1	2 E-80	434/450
Bacteria	Proteobacteria	Nitratiruptor sp. SB155-2	YP_001356650.1	3 E-80	444/450
Eukaryota	Fungi	Chaetomium globosum CBS 148.51	XP_001220995.1	3 E-80	450/450
Bacteria	Proteobacteria	Burkholderia sp. 383	YP_372333.1	3 E-80	436/450
Eukaryota	Viridiplantae	Vitis vinifera	CAN79559.1	4 E-80	435/450
Eukaryota	Viridiplantae	Vitis vinifera	XP_002266968.1	4 E-80	436/450
Eukaryota	Fungi	Malassezia globosa CBS 7966	XP_001730934.1	4 E-80	436/450
Bacteria	Proteobacteria	Magnetospirillum magneticum AMB-1	YP_420582.1	6 E-80	437/450
Bacteria	Proteobacteria	Hyphomicrobium denitrificans ATCC 51888	ZP_05375526.1	7 E-80	437/450
Bacteria	Proteobacteria	Nitrobacter sp. Nb-311A	ZP_01046383.1	8 E-80	434/450
Eukaryota	Viridiplantae	Arabidopsis thaliana	NP_173657.1	9 E-80	440/450
Bacteria	Proteobacteria	Reinekea blandensis MED297	ZP_01113705.1	9 E-80	441/450
Bacteria	Proteobacteria	Sulfurimonas denitrificans DSM 1251	YP_393782.1	1 E-79	435/450
Eukaryota	Viridiplantae	Oryza sativa Indica Group	EAY79548.1	1 E-79	445/450
Eukaryota	Viridiplantae	Oryza sativa Japonica Group	NP_001065411.1	1 E-79	445/450
Eukaryota	Viridiplantae	Morinda citrifolia	CAA75092.1	2 E-79	433/450
Eukaryota	Viridiplantae	Populus trichocarpa	XP_002307072.1	2 E-79	455/450
Eukaryota	Viridiplantae	Morinda citrifolia	CAA75386.1	3 E-79	438/450
Bacteria	Proteobacteria	Sulfurovum sp. NBC37-1	YP_001357783.1	3 E-79	442/450
Bacteria	Proteobacteria	Azorhizobium caulinodans ORS 571	YP_001525248.1	3 E-79	437/450
Bacteria	Proteobacteria	Hyphomonas neptunium ATCC 15444	YP_759437.1	3 E-79	435/450
Eukaryota	Fungi	Gibberella zeae PH-1	XP_381662.1	3 E-79	447/450
Eukaryota	Viridiplantae	Populus trichocarpa	XP_002326680.1	4 E-79	436/450
Eukaryota	Viridiplantae	Arabidopsis thaliana	NP_195708.1	4 E-79	451/450
Eukaryota	Fungi	Botryotinia fuckeliana B05.10	XP_001561221.1	4 E-79	452/450
Bacteria	Proteobacteria	Pseudomonas fluorescens Pf0-1	YP_349718.1	5 E-79	432/450
Bacteria	Proteobacteria	Oligotropha carboxidovorans OM5	YP_002289500.1	5 E-79	431/450
Bacteria	Proteobacteria	Xanthomonas campestris pv. campestris	AAM40232.1	5 E-79	438/450
Bacteria	Proteobacteria	Xanthomonas campestris pv. campestris	YP_001904837.1	5 E-79	438/450
Bacteria	Proteobacteria	Xanthomonas campestris pv. campestris	NP_636308.2	5 E-79	438/450
Bacteria	Proteobacteria	Stenotrophomonas maltophilia K279a	YP_001970817.1	6 E-79	436/450
Eukaryota	Viridiplantae	Sorghum bicolor;	XP_002444840.1	7 E-79	433/450
Bacteria	Proteobacteria	Campylobacterales bacterium GD 1	ZP_05072844.1	7 E-79	434/450
Bacteria	Proteobacteria	Legionella pneumophila str. Lens	YP_127340.1	7 E-79	431/450
Bacteria	Proteobacteria	Stenotrophomonas maltophilia R551-3	YP_002027172.1	8 E-79	436/450
Bacteria	Proteobacteria	Bradyrhizobium sp. ORS278	YP_001206651.1	9 E-79	431/450
Eukaryota	Fungi	Paracoccidioides brasiliensis Pb01;	EEH41674.1	9 E-79	433/450

Bacteria	Proteobacteria	Stigmatella aurantiaca	AAK49032.1	1 E-78	427/450
Eukaryota	Viridiplantae	Physcomitrella patens subsp. patens	XP_001759257.1	1 E-78	451/450
Eukaryota	Fungi	Paracoccidioides brasiliensis Pb03;	EEH18115.1	1 E-78	435/450
Eukaryota	Viridiplantae	Ricinus communis	XP_002531676.1	1 E-78	433/450
Bacteria	Proteobacteria	Legionella pneumophila subsp. pneumophila	YP_096043.1	1 E-78	431/450
Bacteria	Proteobacteria	Nitrobacter winogradskyi Nb-255	YP_317838.1	1 E-78	434/450
Bacteria	Proteobacteria	Marinomonas sp. MED121	ZP_01078756.1	1 E-78	433/450
Bacteria	Proteobacteria	Stenotrophomonas sp. SKA14	ZP_05133652.1	1 E-78	436/450
Bacteria	Proteobacteria	Idiomarina loihiensis L2TR	YP_155708.1	1 E-78	434/450
Eukaryota	Viridiplantae	Arabidopsis thaliana	AAA32784.1	1 E-78	451/450
Bacteria	Proteobacteria	Zymomonas mobilis subsp. mobilis	ZP_04759434.1	2 E-78	436/450
Bacteria	Proteobacteria	Methylocella silvestris BL2	YP_002360468.1	2 E-78	438/450
Bacteria	Proteobacteria	Parvularcula bermudensis HTCC2503	ZP_01016138.1	2 E-78	434/450
Bacteria	Proteobacteria	Maricaulis maris MCS10	YP_756205.1	2 E-78	431/450
Bacteria	Proteobacteria	Helicobacter canadensis MIT 98-5491	ZP_03656387.1	2 E-78	439/450
Bacteria	Proteobacteria	Legionella pneumophila str. Paris	YP_124323.1	2 E-78	431/450
Bacteria	Proteobacteria	Pseudomonas mendocina ymp	YP_001187632.1	2 E-78	435/450
Bacteria	Proteobacteria	Zymomonas mobilis subsp. mobilis	YP_161922.1	3 E-78	436/450
Bacteria	Proteobacteria	Kangiella koreensis DSM 16069	YP_003147299.1	3 E-78	436/450
Bacteria	Proteobacteria	Stigmatella aurantiaca DW4/3-1	ZP_01461537.1	3 E-78	427/450
Bacteria	Proteobacteria	Sorangium cellulosum 'So ce	YP_001614325.1	3 E-78	450/450
Eukaryota	Viridiplantae	Populus trichocarpa	XP_002307364.1	3 E-78	464/450
Bacteria	Proteobacteria	Xanthomonas oryzae pv. oryzicola	ZP_02242070.1	4 E-78	440/450
Eukaryota	Viridiplantae	Sorghum bicolor;	XP_002467762.1	4 E-78	429/450
Bacteria	Proteobacteria	Phenylobacterium zucineum HLK1	YP_002130826.1	4 E-78	435/450
Eukaryota	Viridiplantae	Petroselinum crispum;	AAB69320.1	4 E-78	436/450
Bacteria	Actinobacteria	Tropheryma whipplei str. Twist	NP_787343.1	5 E-78	447/450
Eukaryota	Viridiplantae	Fagus sylvatica	ABA54865.1	6 E-78	433/450
Eukaryota	Viridiplantae	Vitis vinifera	XP_002285754.1	6 E-78	433/450
Bacteria	Actinobacteria	Tropheryma whipplei TW08/27	NP_789484.1	6 E-78	447/450
Bacteria	Proteobacteria	Pseudomonas aeruginosa PA7	YP_001347682.1	6 E-78	444/450
Bacteria	Proteobacteria	Pseudomonas fluorescens SBW25	YP_002871361.1	6 E-78	432/450
Eukaryota	Viridiplantae	Arabidopsis thaliana	NP_195077.1	6 E-78	446/450
Bacteria	Proteobacteria	Neptuniibacter caesariensis	ZP_01167610.1	7 E-78	439/450
Eukaryota	Viridiplantae	Ricinus communis	XP_002531225.1	8 E-78	437/450
Eukaryota	Viridiplantae	Morinda citrifolia	CAA75093.1	8 E-78	432/450
Bacteria	Proteobacteria	Helicobacter bilis ATCC 43879	ZP_04581980.1	9 E-78	434/450
Bacteria	Proteobacteria	Pseudomonas aeruginosa UCBPP-PA14	YP_790330.1	9 E-78	435/450
Bacteria	Proteobacteria	Pseudoalteromonas atlantica T6c	YP_661282.1	1 E-77	441/450
Bacteria	Proteobacteria	Pseudomonas fluorescens Pf-5	YP_261346.1	1 E-77	432/450
Bacteria	Proteobacteria	Pseudomonas aeruginosa PAO1	NP_251533.1	1 E-77	435/450
Bacteria	Proteobacteria	Pseudomonas syringae pv. tomato	NP_791597.1	1 E-77	432/450
Bacteria	Proteobacteria	Sphingomonas sp. SKA58	ZP_01302190.1	1 E-77	431/450
Eukaryota	Viridiplantae	Ricinus communis	XP_002513951.1	1 E-77	442/450
Bacteria	Proteobacteria	Rhodopseudomonas palustris BisA53	YP_780915.1	1 E-77	434/450
Eukaryota	Viridiplantae	Oryza sativa Japonica Group	NP_001062082.1	1 E-77	433/450
Bacteria	Proteobacteria	Xanthomonas axonopodis pv. citri	NP_641347.1	1 E-77	440/450
Bacteria	Proteobacteria	Pseudomonas syringae pv. tabaci	ZP_05636725.1	1 E-77	432/450
Bacteria	Proteobacteria	Rhodospirillum centenum SW	YP_002297191.1	1 E-77	440/450
Bacteria	Proteobacteria	Rhodopseudomonas palustris HaA2	YP_486990.1	2 E-77	434/450
Bacteria	Proteobacteria	Pseudomonas syringae pv. phaseolicola	YP_275779.1	2 E-77	432/450
Bacteria	Proteobacteria	Desulfuromonas acetoxidans DSM 684	ZP_01313607.1	2 E-77	430/450
Eukaryota	Fungi	Magnaporthe grisea 70-15	XP_362841.1	2 E-77	453/450
Bacteria	Proteobacteria	alpha proteobacterium BAL199	ZP_02188268.1	2 E-77	436/450
Bacteria	Proteobacteria	Acetobacter pasteurianus IFO 3283-01	YP_003188419.1	2 E-77	435/450
Bacteria	Proteobacteria	Rhodopseudomonas palustris BisB5	YP_569194.1	2 E-77	434/450
Bacteria	Proteobacteria	Xanthomonas campestris pv. vesicatoria	YP_362759.1	2 E-77	436/450
Bacteria	Bacteroidetes	Salinibacter ruber DSM 13855	YP_446587.1	3 E-77	436/450

Eukaryota	Fungi	Sclerotinia sclerotiorum 1980 UF-70	XP_001594289.1	3 E-77	452/450
Bacteria	Proteobacteria	Magnetococcus sp. MC-1	YP_865327.1	3 E-77	437/450
Bacteria	Proteobacteria	Legionella drancourtii LLAP12	ZP_05108554.1	4 E-77	426/450
Eukaryota	Viridiplantae	Arabidopsis thaliana	AAA32785.1	4 E-77	446/450
Eukaryota	Viridiplantae	Solanum tuberosum	P37822.1	4 E-77	462/450
Bacteria	Proteobacteria	Pseudomonas syringae pv. tomato	ZP_03396836.1	5 E-77	432/450
Bacteria	Proteobacteria	Pseudomonas syringae pv. syringae	YP_236692.1	5 E-77	432/450
Eukaryota	Viridiplantae	Solanum lycopersicum	P37216.1	6 E-77	432/450
Bacteria	Proteobacteria	Helicobacter pullorum MIT 98-5489	ZP_04809871.1	7 E-77	435/450
Eukaryota	Viridiplantae	Arabidopsis thaliana	AAM65197.1	7 E-77	446/450
Eukaryota	Viridiplantae	Physcomitrella patens subsp. patens	XP_001778993.1	8 E-77	437/450
Eukaryota	Viridiplantae	Nicotiana tabacum	P27608.1	9 E-77	433/450
Bacteria	Proteobacteria	Rhodospirillum rubrum ATCC 11170	YP_425774.1	1 E-76	431/450
Bacteria	Lentisphaerae	Lentisphaera araneosa HTCC2155	ZP_01876010.1	1 E-76	433/450
Eukaryota	Viridiplantae	Vitis vinifera	ACM45080.1	1 E-76	449/450
Eukaryota	Viridiplantae	Ostreococcus lucimarinus CCE9901	XP_001418517.1	1 E-76	433/450
Bacteria	Proteobacteria	Rhodopseudomonas palustris BisB18	YP_531953.1	1 E-76	434/450
Bacteria	Proteobacteria	Gluconacetobacter diazotrophicus PAl 5	YP_001602459.1	1 E-76	435/450
Bacteria	Proteobacteria	Methylobacterium radiotolerans JCM 2831	YP_001755361.1	2 E-76	433/450
Eukaryota	Viridiplantae	Oryza sativa Japonica Group	NP_001060314.1	2 E-76	449/450
Eukaryota	Viridiplantae	Solanum tuberosum	P21357.2	2 E-76	432/450
Bacteria	Proteobacteria	Myxococcus xanthus DK 1622	YP_631829.1	2 E-76	427/450
Eukaryota	Viridiplantae	Solanum tuberosum	BAC23040.1	2 E-76	432/450
Bacteria	Proteobacteria	Bradyrhizobium japonicum USDA 110	NP_770418.1	2 E-76	434/450
Bacteria	Proteobacteria	Alteromonas macleodii ATCC 27126	ZP_04713560.1	3 E-76	442/450
Bacteria	Actinobacteria	Streptomyces pristinaespiralis ATCC 25486	ZP_05012026.1	3 E-76	425/450
Bacteria	Proteobacteria	Marinomonas sp. MWYL1	YP_001341448.1	3 E-76	432/450
Bacteria	Proteobacteria	Nautilia profundicola AmH	YP_002607804.1	3 E-76	436/450
Eukaryota	Viridiplantae	Solanum lycopersicum	P37215.1	4 E-76	462/450
Eukaryota	Viridiplantae	Physcomitrella patens subsp. patens	XP_001784090.1	4 E-76	435/450
Bacteria	Proteobacteria	Pseudoalteromonas haloplanktis TAC125	YP_342041.1	5 E-76	433/450
Bacteria	Proteobacteria	Rhodopseudomonas palustris CGA009	NP_947329.1	5 E-76	434/450
Eukaryota	Viridiplantae	Oryza sativa Japonica Group	AAR06362.1	5 E-76	430/450
Bacteria	Proteobacteria	Pseudomonas putida GB-1	YP_001667684.1	6 E-76	432/450
Bacteria	Actinobacteria	Streptomyces coelicolor A3(2)	NP_627424.1	6 E-76	449/450
Bacteria	Proteobacteria	Arcobacter butzleri RM4018	YP_001489169.1	7 E-76	442/450
Bacteria	Proteobacteria	Pseudomonas putida F1	YP_001269157.1	7 E-76	432/450
Bacteria	Proteobacteria	Desulfobacterium autotrophicum HRM2	YP_002602234.1	7 E-76	440/450
Bacteria	Proteobacteria	Hirschia baltica ATCC 49814	YP_003060230.1	7 E-76	435/450
Bacteria	Proteobacteria	Roseobacter sp. CCS2	ZP_01752012.1	8 E-76	433/450
Bacteria	Actinobacteria	Thermomonospora curvata DSM 43183	ZP_04033507.1	8 E-76	432/450
Bacteria	Proteobacteria	Roseobacter sp. AzwK-3b	ZP_01903374.1	9 E-76	430/450
Bacteria	Proteobacteria	Pseudomonas entomophila L48	YP_607242.1	1 E-75	432/450
Bacteria	Actinobacteria	Acidimicrobium ferrooxidans DSM 10331	YP_003109413.1	1 E-75	432/450
Bacteria	Proteobacteria	Pseudomonas putida KT2440	NP_744021.1	1 E-75	432/450
Bacteria	Actinobacteria	Streptomyces hygroscopicus ATCC 53653	ZP_05518527.1	1 E-75	425/450
Bacteria	Actinobacteria	Streptomyces griseus subsp. griseus	YP_001826898.1	1 E-75	425/450
Bacteria	Proteobacteria	Citreicella sp. SE45	ZP_05780472.1	1 E-75	433/450
Bacteria	Actinobacteria	marine actinobacterium PHSC20C1	ZP_01130237.1	1 E-75	426/450
Eukaryota	Viridiplantae	Sorghum bicolor;	XP_002463199.1	2 E-75	433/450
Bacteria	Proteobacteria	Yersinia rohdei ATCC 43380	ZP_04614461.1	2 E-75	433/450
Bacteria	Proteobacteria	Alteromonadales bacterium TW-7	ZP_01613299.1	2 E-75	433/450
Bacteria	Proteobacteria	Alteromonas macleodii 'Deep ecotype'	YP_002126493.1	2 E-75	442/450
Bacteria	Proteobacteria	Pseudomonas putida W619	YP_001748349.1	2 E-75	432/450
Eukaryota	Viridiplantae	Oryza sativa Japonica Group	AAG60192.1	2 E-75	469/450
Bacteria	Proteobacteria	Stigmatella aurantiaca Sg a15	AAG31131.1	2 E-75	435/450
Eukaryota	Viridiplantae	Physcomitrella patens subsp. patens	XP_001759726.1	2 E-75	437/450
Bacteria	Proteobacteria	Brevundimonas sp. BAL3	ZP_05031804.1	3 E-75	435/450

Eukaryota	Viridiplantae	Populus trichocarpa	XP_002301067.1	3 E-75	433/450
Bacteria	Actinobacteria	Acidothermus cellulolyticus 11B	YP_872743.1	3 E-75	428/450
Bacteria	Actinobacteria	Streptomyces clavuligerus ATCC 27064	ZP_05008203.1	3 E-75	425/450
Bacteria	Actinobacteria	Streptomyces lividans TK24	ZP_05525594.1	3 E-75	449/450
Bacteria	Proteobacteria	Rhodobacterales bacterium HTCC2083	ZP_05076157.1	3 E-75	430/450
Bacteria	Proteobacteria	Xanthobacter autotrophicus Py2	YP_001418113.1	4 E-75	434/450
Bacteria	Actinobacteria	Streptomyces roseosporus NRRL 15998	ZP_04692543.1	4 E-75	425/450
Eukaryota	Viridiplantae	Zea mays;	NP_001150866.1	6 E-75	433/450
Bacteria	Proteobacteria	Rhodobacterales bacterium Y4I	ZP_05077365.1	7 E-75	430/450
Bacteria	Proteobacteria	Helicobacter winghamensis ATCC BAA-430	ZP_04582510.1	7 E-75	435/450
Eukaryota	Viridiplantae	Physcomitrella patens subsp. patens	XP_001751707.1	7 E-75	432/450
Eukaryota	Viridiplantae	Oryza sativa Japonica Group	Q75W16.1	8 E-75	451/450
Bacteria	Actinobacteria	Streptomyces ghanaensis ATCC 14672	ZP_04688733.1	9 E-75	425/450
Bacteria	Proteobacteria	Roseobacter sp. SK209-2-6	ZP_01753583.1	9 E-75	433/450
Bacteria	Proteobacteria	Helicobacter cinaedi CCUG 18818	ZP_03657528.1	1 E-74	441/450
Bacteria	Proteobacteria	Rhizobium sp. NGR234	YP_002822429.1	1 E-74	436/450
Eukaryota	Viridiplantae	Oryza sativa Japonica Group	NP_001050271.1	1 E-74	429/450
Bacteria	Proteobacteria	Brucella microti CCM 4915	YP_003106944.1	1 E-74	436/450
Bacteria	Proteobacteria	Magnetospirillum gryphiswaldense MSR-1	CAM76230.1	1 E-74	444/450
Bacteria	Proteobacteria	Acidiphilium cryptum JF-5	YP_001235102.1	1 E-74	447/450
Eukaryota	Viridiplantae	Oryza sativa Japonica Group	BAD14924.1	1 E-74	429/450
Bacteria	Proteobacteria	Oceanicola granulosus HTCC2516	ZP_01158478.1	1 E-74	433/450
Bacteria	Actinobacteria	Streptomyces sp. SPB78	ZP_05486364.1	1 E-74	427/450
Bacteria	Proteobacteria	Roseovarius nubinhibens ISM	ZP_00960724.1	1 E-74	433/450
Eukaryota	Viridiplantae	Picea sitchensis	ABK24266.1	1 E-74	446/450
Bacteria	Actinobacteria	Saccharopolyspora erythraea NRRL 2338	YP_001106309.1	1 E-74	417/450
Bacteria	Actinobacteria	Leifsonia xyli subsp. xyli	YP_062452.1	2 E-74	425/450
Bacteria	Proteobacteria	Oceanibulbus indolifex HEL-45	ZP_02153723.1	2 E-74	430/450
Bacteria	Proteobacteria	Bartonella grahamii as4aup	YP_002971720.1	2 E-74	433/450
Bacteria	Proteobacteria	Brucella suis bv. 3	ZP_05166133.1	2 E-74	431/450
Bacteria	Actinobacteria	Actinomyces odontolyticus ATCC 17982	ZP_02044823.1	2 E-74	409/450
Bacteria	Proteobacteria	Sulfitobacter sp. NAS-14.1	ZP_00962386.1	2 E-74	433/450
Bacteria	Proteobacteria	Sulfitobacter sp. EE-36	ZP_00954906.1	2 E-74	433/450
Bacteria	Proteobacteria	Xanthomonas oryzae pv. oryzae	YP_449921.1	3 E-74	430/450
Bacteria	Actinobacteria	Streptomyces flavogriseus ATCC 33331	ZP_05805741.1	3 E-74	425/450
Bacteria	Proteobacteria	Bartonella tribocorum CIP 105476	YP_001609328.1	3 E-74	433/450
Bacteria	Proteobacteria	Roseobacter sp. GAI101	ZP_05098940.1	3 E-74	433/450
Bacteria	Actinobacteria	Saccharopolyspora erythraea NRRL 2338	YP_001105077.1	3 E-74	424/450
Bacteria	Actinobacteria	Streptomyces sp.	AAB88859.1	3 E-74	425/450
Bacteria	Proteobacteria	Parvibaculum lavamentivorans DS-1	YP_001412374.1	4 E-74	437/450
Eukaryota	Fungi	Aspergillus terreus NIH2624	XP_001214464.1	4 E-74	495/450

AFUA_1G11330

Eukaryota	Fungi	Aspergillus fumigatus Af293	XP_752498.1	0.0	442/442
Eukaryota	Fungi	Neosartorya fischeri NRRL 181	XP_001264632.1	0.0	442/442
Eukaryota	Fungi	Aspergillus clavatus NRRL 1	XP_001270459.1	0.0	442/442
Eukaryota	Fungi	Aspergillus oryzae RIB40	XP_001821621.1	1 E-159	396/442
Eukaryota	Fungi	Aspergillus flavus NRRL3357	XP_002379823.1	1 E-158	396/442
Eukaryota	Fungi	Aspergillus fumigatus Af293	XP_752266.1	8 E-92	386/442
Eukaryota	Fungi	Neosartorya fischeri NRRL 181	XP_001264866.1	1 E-91	386/442
Eukaryota	Fungi	Aspergillus oryzae RIB40	XP_001818531.1	2 E-89	384/442
Eukaryota	Fungi	Aspergillus flavus NRRL3357	XP_002373774.1	4 E-89	384/442
Eukaryota	Fungi	Aspergillus clavatus NRRL 1	XP_001269357.1	2 E-85	386/442
Eukaryota	Fungi	Aspergillus flavus NRRL3357	XP_002384442.1	8 E-73	425/442
Eukaryota	Fungi	Aspergillus flavus NRRL3357	XP_002384445.1	2 E-67	370/442
Eukaryota	Fungi	Aspergillus oryzae RIB40	XP_001827202.1	7 E-60	439/442

AFUA_1G11340

Eukaryota	Fungi	Aspergillus fumigatus Af293	XP_752499.1	0.0	516/516
Eukaryota	Fungi	Aspergillus fumigatus	CAE47860.1	0.0	513/516
Eukaryota	Fungi	Neosartorya fischeri NRRL 181	XP_001264631.1	0.0	479/516
Eukaryota	Fungi	Aspergillus clavatus NRRL 1	XP_001270460.1	1 E-176	421/516

AFUA_1G11350

Eukaryota	Fungi	Aspergillus fumigatus Af293	XP_752500.1	0.0	533/533
Eukaryota	Fungi	Neosartorya fischeri NRRL 181	XP_001264630.1	0.0	518/533
Eukaryota	Fungi	Aspergillus terreus NIH2624	XP_001210600.1	0.0	511/533
Eukaryota	Fungi	Aspergillus clavatus NRRL 1	XP_001270461.1	0.0	524/533
Eukaryota	Fungi	Talaromyces stipitatus ATCC 10500	XP_002478115.1	0.0	500/533
Eukaryota	Fungi	Nectria haematococca mpVI 77-13-4	EEU37078.1	1 E-173	504/533
Eukaryota	Fungi	Aspergillus niger CBS 513.88	XP_001402464.1	1 E-151	516/533
Eukaryota	Fungi	Aspergillus nidulans FGSC A4	XP_660229.1	1 E-147	511/533
Eukaryota	Fungi	Aspergillus terreus NIH2624	XP_001210224.1	1 E-146	525/533
Eukaryota	Fungi	Gibberella zeae PH-1	XP_383948.1	1 E-138	518/533
Eukaryota	Fungi	Nectria haematococca mpVI 77-13-4	EEU36152.1	1 E-135	499/533
Eukaryota	Fungi	Aspergillus niger CBS 513.88	XP_001392983.1	1 E-126	508/533
Eukaryota	Fungi	Aspergillus terreus NIH2624	XP_001216846.1	1 E-126	497/533
Eukaryota	Fungi	Neurospora crassa OR74A	XP_960454.1	1 E-124	526/533
Eukaryota	Fungi	Lachancea thermotolerans CBS 6340	XP_002552678.1	1 E-124	500/533
Eukaryota	Fungi	Aspergillus fumigatus Af293	XP_753646.1	1 E-122	504/533
Eukaryota	Fungi	Aspergillus nidulans FGSC A4	XP_682639.1	1 E-121	511/533
Eukaryota	Fungi	Aspergillus fumigatus A1163	EDP51737.1	1 E-121	504/533
Eukaryota	Fungi	Saccharomyces cerevisiae AWRI1631	EDZ69042.1	1 E-121	509/533
Eukaryota	Fungi	Saccharomyces cerevisiae	NP_015023.1	1 E-120	509/533
Eukaryota	Fungi	Saccharomyces cerevisiae YJM789	EDN63696.1	1 E-120	505/533
Eukaryota	Fungi	Saccharomyces cerevisiae RM11-1a	EDV10935.1	1 E-120	509/533
Eukaryota	Fungi	Neosartorya fischeri NRRL 181	XP_001259697.1	1 E-120	480/533
Eukaryota	Fungi	Penicillium marneffei ATCC 18224	XP_002153625.1	1 E-119	501/533
Eukaryota	Fungi	Talaromyces stipitatus ATCC 10500	XP_002487796.1	1 E-118	485/533
Eukaryota	Fungi	Kluyveromyces lactis NRRL Y-1140	XP_451545.1	1 E-116	490/533
Eukaryota	Fungi	Botryotinia fuckeliana B05.10	XP_001555163.1	1 E-115	516/533
Eukaryota	Fungi	Schizosaccharomyces pombe	NP_595059.1	1 E-113	495/533
Eukaryota	Fungi	Nectria haematococca mpVI 77-13-4	EEU43663.1	1 E-112	493/533
Eukaryota	Fungi	Sclerotinia sclerotiorum 1980 UF-70	XP_001596838.1	1 E-112	476/533
Eukaryota	Fungi	Aspergillus clavatus NRRL 1	XP_001274302.1	1 E-112	498/533
Eukaryota	Fungi	Aspergillus flavus NRRL3357	XP_002385025.1	1 E-111	500/533
Eukaryota	Fungi	Aspergillus oryzae RIB40	XP_001827013.1	1 E-111	500/533
Eukaryota	Fungi	Aspergillus niger CBS 513.88	XP_001392936.1	1 E-104	501/533
Eukaryota	Fungi	Aspergillus terreus NIH2624	XP_001210221.1	1 E-103	497/533
Eukaryota	Fungi	Exophiala dermatitidis	AAL68895.1	1 E-101	473/533
Eukaryota	Fungi	Magnaporthe grisea 70-15	XP_361014.2	6 E-97	492/533
Eukaryota	Fungi	Pichia stipitis CBS 6054	XP_001382535.2	5 E-92	474/533
Eukaryota	Fungi	Pichia guilliermondii ATCC 6260	EDK35987.2	2 E-91	491/533
Eukaryota	Fungi	Pichia guilliermondii ATCC 6260	XP_001486708.1	4 E-91	491/533
Eukaryota	Fungi	Penicillium marneffei ATCC 18224	XP_002149113.1	6 E-91	500/533
Eukaryota	Fungi	Neosartorya fischeri NRRL 181	XP_001265534.1	1 E-90	496/533
Eukaryota	Fungi	Aspergillus fumigatus	AAM27212.1	2 E-90	504/533
Eukaryota	Fungi	Candida dubliniensis CD36	XP_002417647.1	2 E-90	511/533
Eukaryota	Fungi	Lodderomyces elongisporus NRRL YB-4239	XP_001525634.1	2 E-90	504/533
Eukaryota	Fungi	Candida albicans SC5314	XP_714012.1	5 E-90	507/533
Eukaryota	Fungi	Aspergillus oryzae RIB40	XP_001827601.1	1 E-89	499/533
Eukaryota	Fungi	Pichia guilliermondii ATCC 6260	EDK41708.2	4 E-89	512/533
Eukaryota	Fungi	Pichia guilliermondii ATCC 6260	XP_001482043.1	3 E-88	512/533

Eukaryota	Fungi	Debaryomyces hansenii	CAG85888.2	4 E-88	491/533
Eukaryota	Fungi	Uncinocarpus reesii 1704	XP_002540763.1	7 E-88	486/533
Eukaryota	Fungi	Debaryomyces hansenii CBS767	XP_457843.1	9 E-87	491/533
Eukaryota	Fungi	Pichia guilliermondii ATCC 6260	EDK41060.2	1 E-86	489/533
Eukaryota	Fungi	Aspergillus flavus NRRL3357	XP_002384829.1	1 E-86	483/533
Eukaryota	Fungi	Candida tropicalis MYA-3404	XP_002548981.1	2 E-85	470/533
Eukaryota	Fungi	Talaromyces stipitatus ATCC 10500	XP_002483259.1	5 E-85	485/533
Eukaryota	Fungi	Pichia stipitis CBS 6054	XP_001383480.2	2 E-84	476/533
Eukaryota	Fungi	Debaryomyces hansenii	CAG87131.2	7 E-84	490/533
Eukaryota	Fungi	Debaryomyces hansenii CBS767	XP_458970.1	2 E-83	490/533
Eukaryota	Fungi	Pichia guilliermondii ATCC 6260	XP_001483203.1	4 E-82	489/533
Eukaryota	Fungi	Pichia stipitis CBS 6054	XP_001382958.2	3 E-81	461/533
Eukaryota	Fungi	Clavispora lusitaniae ATCC 42720	XP_002616155.1	4 E-81	473/533
Eukaryota	Fungi	Candida tropicalis MYA-3404	XP_002548289.1	5 E-80	445/533
Eukaryota	Fungi	Ustilago maydis 521	XP_758129.1	7 E-78	511/533
Eukaryota	Fungi	Lachancea kluyveri	AAO32555.1	8 E-77	511/533
Eukaryota	Fungi	Vanderwaltozyma polyspora DSM 70294	XP_001645631.1	1 E-76	476/533
Eukaryota	Fungi	Saccharomyces bayanus	AAO32364.1	5 E-76	503/533
Eukaryota	Fungi	Aspergillus fumigatus Af293	XP_748670.1	7 E-76	466/533
Eukaryota	Fungi	Saccharomyces cerevisiae	NP_013591.1	2 E-75	522/533
Eukaryota	Fungi	Candida glabrata CBS 138	XP_445072.1	4 E-75	497/533
Eukaryota	Fungi	Pichia guilliermondii ATCC 6260	EDK38098.2	1 E-74	468/533
Eukaryota	Fungi	Aspergillus fumigatus A1163	EDP53300.1	2 E-74	466/533
Eukaryota	Fungi	Naumovia castellii	AAO32476.1	4 E-74	503/533
Eukaryota	Fungi	Saccharomyces cerevisiae	AAA34449.1	2 E-73	516/533
Eukaryota	Fungi	Naumovia castellii	AAO32477.1	2 E-73	507/533
Eukaryota	Fungi	Saccharomyces cerevisiae AWRI1631	EDZ69948.1	8 E-73	515/533
Eukaryota	Fungi	Pichia guilliermondii ATCC 6260	XP_001486525.1	1 E-72	468/533
Eukaryota	Fungi	Saccharomyces cerevisiae	NP_014006.1	1 E-72	515/533
Eukaryota	Fungi	Saccharomyces cerevisiae RM11-1a	EDV11770.1	1 E-72	515/533
Eukaryota	Fungi	Candida albicans SC5314	XP_720131.1	1 E-71	469/533
Eukaryota	Fungi	Aspergillus clavatus NRRL 1	XP_001273645.1	1 E-71	470/533
Eukaryota	Fungi	Neosartorya fischeri NRRL 181	XP_001267457.1	9 E-71	510/533
Eukaryota	Fungi	Cryptococcus neoformans var. neoformans	XP_567402.1	7 E-70	460/533
Eukaryota	Fungi	Candida albicans WO-1	EEQ44361.1	1 E-69	469/533
Eukaryota	Fungi	Aspergillus fumigatus Af293	XP_746322.1	8 E-69	474/533
Eukaryota	Fungi	Lachancea thermotolerans CBS 6340	XP_002552391.1	1 E-68	438/533
Eukaryota	Fungi	Candida dubliniensis CD36	XP_002419235.1	1 E-68	468/533
Eukaryota	Fungi	Candida glabrata CBS 138	XP_449477.1	8 E-67	466/533
Eukaryota	Fungi	Cryptococcus neoformans var. neoformans	XP_775625.1	1 E-65	467/533
Eukaryota	Fungi	Cryptococcus neoformans var. neoformans	XP_570319.1	7 E-65	467/533
Eukaryota	Fungi	Neurospora crassa OR74A	XP_959082.1	7 E-65	512/533
Eukaryota	Fungi	Cryptococcus neoformans var. neoformans	XP_775631.1	2 E-62	455/533
Eukaryota	Fungi	Cryptococcus neoformans var. neoformans	XP_570327.1	2 E-62	455/533
Eukaryota	Fungi	Gibberella zeae PH-1	XP_386547.1	9 E-60	464/533
Eukaryota	Fungi	Talaromyces stipitatus ATCC 10500	XP_002341319.1	4 E-57	429/533
Eukaryota	Fungi	Botryotinia fuckeliana B05.10	XP_001559476.1	7 E-57	470/533
Eukaryota	Fungi	Nectria haematococca mpVI 77-13-4	EEU46240.1	5 E-55	450/533
Eukaryota	Fungi	Penicillium chrysogenum Wisconsin 54-1255	XP_002556929.1	5 E-55	501/533
Eukaryota	Fungi	Aspergillus nidulans FGSC A4	XP_659328.1	2 E-54	532/533
Eukaryota	Fungi	Uncinocarpus reesii 1704	XP_002541519.1	4 E-54	484/533
Eukaryota	Fungi	Candida glabrata CBS 138	XP_448880.1	4 E-54	460/533
Eukaryota	Fungi	Aspergillus terreus NIH2624	XP_001214439.1	4 E-51	437/533
Eukaryota	Fungi	Aspergillus oryzae RIB40	XP_001820441.1	3 E-50	487/533
Eukaryota	Fungi	Aspergillus flavus NRRL3357	XP_002373967.1	2 E-49	502/533
Eukaryota	Fungi	Pyrenophora tritici-repentis Pt-1C-BFP	XP_001940385.1	2 E-48	483/533
Eukaryota	Fungi	Paracoccidioides brasiliensis Pb03;	EEH18443.1	1 E-47	491/533
Eukaryota	Fungi	Paracoccidioides brasiliensis Pb18;	EEH47381.1	1 E-47	491/533

Eukaryota	Fungi	Microsporum canis CBS 113480	EEQ30871.1	1 E-47	463/533
Eukaryota	Fungi	Magnaporthe grisea 70-15	XP_367441.2	5 E-46	513/533
Eukaryota	Fungi	Aspergillus oryzae RIB40	XP_001821119.1	9 E-46	523/533
Eukaryota	Fungi	Coccidioides immitis RS;	XP_001239533.1	2 E-45	482/533
Eukaryota	Fungi	Ajellomyces capsulatus H143	EER38591.1	4 E-45	490/533
Eukaryota	Fungi	Ajellomyces capsulatus G186AR	EEH06915.1	5 E-45	490/533
Eukaryota	Fungi	Nectria haematococca mpVI 77-13-4	EEU42839.1	1 E-44	506/533
Eukaryota	Fungi	Coccidioides posadasii C735 delta	EER24974.1	1 E-44	482/533
Eukaryota	Fungi	Aspergillus niger CBS 513.88	XP_001400212.1	1 E-44	482/533
Eukaryota	Fungi	Gibberella zeae PH-1	XP_384602.1	3 E-44	517/533
Eukaryota	Fungi	Aspergillus flavus NRRL3357	XP_002376869.1	4 E-44	431/533
Eukaryota	Fungi	Nectria haematococca mpVI 77-13-4	EEU33970.1	9 E-44	499/533
Eukaryota	Fungi	Gibberella zeae PH-1	XP_383623.1	2 E-42	461/533
Eukaryota	Fungi	Ajellomyces dermatitidis SLH14081	XP_002623125.1	1 E-41	477/533
Eukaryota	Fungi	Gibberella zeae PH-1	XP_387726.1	2 E-41	514/533
Eukaryota	Fungi	Penicillium chrysogenum Wisconsin 54-1255	XP_002564887.1	1 E-40	476/533
Eukaryota	Fungi	Gibberella zeae PH-1	XP_380769.1	5 E-40	499/533
Eukaryota	Fungi	Verticillium albo-atrum VaMs.102	EEY22242.1	8 E-40	470/533
Eukaryota	Fungi	Nectria haematococca mpVI 77-13-4	EEU36676.1	4 E-39	503/533
Eukaryota	Fungi	Talaromyces stipitatus ATCC 10500	XP_002481295.1	4 E-39	497/533
Eukaryota	Fungi	Neosartorya fischeri NRRL 181	XP_001266667.1	5 E-39	510/533
Eukaryota	Fungi	Nectria haematococca mpVI 77-13-4	EEU40000.1	9 E-39	460/533
Eukaryota	Fungi	Botryotinia fuckeliana B05.10	XP_001551980.1	2 E-38	453/533
Eukaryota	Fungi	Podospora anserina DSM 980	XP_001906806.1	2 E-37	435/533
Eukaryota	Fungi	Botryotinia fuckeliana B05.10	XP_001548647.1	3 E-37	446/533
Eukaryota	Fungi	Aspergillus nidulans FGSC A4	XP_659972.1	9 E-37	477/533
Eukaryota	Fungi	Aspergillus terreus NIH2624	XP_001211337.1	9 E-37	500/533
Eukaryota	Fungi	Nectria haematococca mpVI 77-13-4	EEU48552.1	1 E-36	459/533
Eukaryota	Fungi	Penicillium chrysogenum Wisconsin 54-1255	XP_002557091.1	2 E-36	503/533
Eukaryota	Fungi	Nectria haematococca mpVI 77-13-4	EEU37756.1	5 E-36	451/533
Eukaryota	Fungi	Neurospora crassa OR74A	XP_957495.1	4 E-35	502/533
Eukaryota	Fungi	Podospora anserina DSM 980	XP_001910400.1	2 E-34	466/533
Eukaryota	Fungi	Penicillium marneffei ATCC 18224	XP_002148542.1	5 E-34	500/533
Eukaryota	Fungi	Nectria haematococca mpVI 77-13-4	EEU38162.1	6 E-34	486/533
Eukaryota	Fungi	Nectria haematococca mpVI 77-13-4	EEU40179.1	1 E-33	500/533
Eukaryota	Fungi	Ajellomyces capsulatus NAm1	XP_001541453.1	4 E-33	482/533
Eukaryota	Fungi	Talaromyces stipitatus ATCC 10500	XP_002482880.1	5 E-33	484/533
Eukaryota	Fungi	Ajellomyces capsulatus G186AR	EEH03164.1	7 E-33	487/533
Eukaryota	Fungi	Aspergillus flavus NRRL3357	XP_002384929.1	8 E-33	427/533
Eukaryota	Fungi	Magnaporthe grisea 70-15	XP_363491.1	2 E-32	506/533
Eukaryota	Fungi	Microsporum canis CBS 113480	EEQ35525.1	2 E-32	519/533
Eukaryota	Fungi	Yarrowia lipolytica CLIB122	XP_502147.1	3 E-32	536/533
Eukaryota	Fungi	Coccidioides immitis RS;	XP_001246174.1	5 E-32	507/533
Eukaryota	Fungi	Coccidioides posadasii C735 delta	EER25636.1	5 E-32	482/533
Eukaryota	Fungi	Aspergillus terreus NIH2624	XP_001216077.1	6 E-32	506/533
Eukaryota	Fungi	Microsporum canis CBS 113480	EEQ33861.1	9 E-32	498/533
Eukaryota	Fungi	Aspergillus niger CBS 513.88	XP_001398507.1	1 E-31	512/533
Eukaryota	Fungi	Gibberella zeae PH-1	XP_387808.1	2 E-31	484/533
Eukaryota	Fungi	Laccaria bicolor S238N-H82	XP_001881683.1	2 E-31	442/533
Eukaryota	Fungi	Verticillium albo-atrum VaMs.102	EEY20201.1	4 E-31	527/533
Eukaryota	Fungi	Microsporum canis CBS 113480	EEQ31041.1	9 E-31	498/533
Eukaryota	Fungi	Laccaria bicolor S238N-H82	XP_001881684.1	1 E-30	432/533
Eukaryota	Fungi	Magnaporthe grisea 70-15	XP_370297.2	1 E-30	459/533
Bacteria	Actinobacteria	Tsukamurella paurometabola DSM 20162	ZP_04026638.1	4 E-29	454/533
Eukaryota	Fungi	Ajellomyces dermatitidis ER-3	EEQ90932.1	1 E-28	429/533
Eukaryota	Fungi	Nectria haematococca mpVI 77-13-4	EEU40274.1	1 E-28	449/533
Eukaryota	Fungi	Coprinopsis cinerea okayama7#130	XP_001836758.1	1 E-28	457/533
Eukaryota	Fungi	Sclerotinia sclerotiorum 1980 UF-70	XP_001591332.1	2 E-28	468/533

Eukaryota	Fungi	Coccidioides posadasii C735 delta	EER25571.1	6 E-28	489/533
Eukaryota	Fungi	Paracoccidioides brasiliensis Pb03;	EEH16916.1	1 E-27	529/533
Eukaryota	Fungi	Nectria haematococca mpVI 77-13-4	EEU37827.1	2 E-27	451/533
Eukaryota	Fungi	Pichia pastoris GS115	XP_002492334.1	3 E-27	498/533
Eukaryota	Fungi	Ajellomyces dermatitidis ER-3	EEQ91398.1	5 E-27	497/533
Eukaryota	Fungi	Pyrenophora tritici-repentis Pt-1C-BFP	XP_001939435.1	5 E-27	444/533
Eukaryota	Fungi	Sclerotinia sclerotiorum 1980 UF-70	XP_001594493.1	6 E-27	505/533
Eukaryota	Fungi	Coccidioides immitis RS;	XP_001246094.1	1 E-26	489/533
Eukaryota	Fungi	Uncinocarpus reesii 1704	XP_002583219.1	4 E-26	430/533
Eukaryota	Fungi	Armillaria mellea	ABU50337.1	6 E-26	450/533
Eukaryota	Fungi	Aspergillus nidulans FGSC A4	XP_658205.1	8 E-26	444/533
Eukaryota	Fungi	Ajellomyces dermatitidis SLH14081	XP_002624326.1	9 E-26	489/533
Eukaryota	Fungi	Aspergillus niger CBS 513.88	XP_001389277.1	1 E-25	484/533
Bacteria	Actinobacteria	Streptomyces lavendulae	ABI22121.1	3 E-25	430/533
Eukaryota	Fungi	Malassezia globosa CBS 7966	XP_001728673.1	6 E-25	484/533
Eukaryota	Fungi	Paracoccidioides brasiliensis Pb18;	EEH50438.1	1 E-24	529/533
Eukaryota	Fungi	Podospora anserina DSM 980	XP_001904009.1	2 E-24	508/533
Eukaryota	Fungi	Verticillium albo-atrum VaMs.102	EEY15558.1	2 E-24	461/533
Bacteria	Actinobacteria	Salinispora arenicola CNS-205	YP_001535185.1	3 E-24	434/533
Eukaryota	Fungi	Cryptococcus neoformans var. neoformans	XP_569134.1	3 E-24	493/533
Eukaryota	Fungi	Cryptococcus neoformans var. neoformans	XP_569053.1	3 E-24	494/533
Eukaryota	Fungi	Magnaporthe grisea 70-15	XP_369226.2	9 E-24	427/533
Eukaryota	Fungi	Sclerotinia sclerotiorum 1980 UF-70	XP_001593435.1	1 E-23	439/533
Eukaryota	Fungi	Laccaria bicolor S238N-H82	XP_001882204.1	1 E-22	493/533
Eukaryota	Fungi	Armillaria mellea	ABU50338.1	7 E-22	466/533
Bacteria	Actinobacteria	Frankia sp. EAN1pec	YP_001507652.1	7 E-22	457/533
Eukaryota	Fungi	Aspergillus oryzae RIB40	XP_001822515.1	3 E-21	488/533
Eukaryota	Fungi	Coccidioides posadasii C735 delta	EER26843.1	3 E-21	432/533
Eukaryota	Fungi	Uncinocarpus reesii 1704	XP_002583165.1	1 E-20	461/533
Eukaryota	Fungi	Aspergillus nidulans FGSC A4	XP_658749.1	1 E-20	486/533

AFUA_1G11360

Eukaryota	Fungi	Aspergillus fumigatus Af293	XP_752501.1	0.0	352/352
Eukaryota	Fungi	Neosartorya fischeri NRRL 181	XP_001264629.1	0.0	352/352
Eukaryota	Fungi	Aspergillus clavatus NRRL 1	XP_001270462.1	1 E-178	352/352
Eukaryota	Fungi	Nectria haematococca mpVI 77-13-4	EEU36984.1	1 E-113	334/352
Eukaryota	Fungi	Aspergillus terreus NIH2624	XP_001210223.1	1 E-102	331/352
Eukaryota	Fungi	Talaromyces stipitatus ATCC 10500	XP_002488804.1	1 E-83	331/352
Eukaryota	Fungi	Phaeosphaeria nodorum SN15	XP_001791109.1	4 E-77	335/352
Eukaryota	Fungi	Botryotinia fuckeliana B05.10	XP_001554946.1	2 E-75	330/352
Eukaryota	Fungi	Sclerotinia sclerotiorum 1980 UF-70	XP_001585790.1	1 E-74	333/352
Eukaryota	Fungi	Aspergillus oryzae RIB40	XP_001817435.1	2 E-69	335/352
Eukaryota	Fungi	Aspergillus flavus NRRL3357	XP_002372509.1	1 E-68	335/352
Eukaryota	Fungi	Verticillium albo-atrum VaMs.102	EEY20659.1	2 E-67	331/352
Eukaryota	Fungi	Sporidiobolus salmonicolor	Q9UUN9.3	1 E-66	336/352
Eukaryota	Fungi	Ajellomyces capsulatus G186AR	EEH09765.1	2 E-61	332/352
Eukaryota	Fungi	Nectria haematococca mpVI 77-13-4	EEU35768.1	5 E-61	335/352
Eukaryota	Fungi	Verticillium albo-atrum VaMs.102	EEY18091.1	5 E-61	332/352
Eukaryota	Fungi	Ajellomyces capsulatus NAm1	XP_001542158.1	9 E-61	332/352
Eukaryota	Fungi	Ajellomyces dermatitidis SLH14081	XP_002623543.1	2 E-60	332/352
Eukaryota	Fungi	Ajellomyces dermatitidis ER-3	EEQ89775.1	2 E-60	332/352
Eukaryota	Fungi	Paracoccidioides brasiliensis Pb01;	EEH34334.1	1 E-59	336/352
Eukaryota	Fungi	Paracoccidioides brasiliensis Pb18;	EEH50652.1	2 E-59	336/352
Eukaryota	Fungi	Paracoccidioides brasiliensis Pb03;	EEH15905.1	3 E-59	336/352
Eukaryota	Fungi	Talaromyces stipitatus ATCC 10500	XP_002340745.1	2 E-57	325/352
Eukaryota	Fungi	Nectria haematococca mpVI 77-13-4	EEU41260.1	8 E-57	337/352
Eukaryota	Fungi	Nectria haematococca mpVI 77-13-4	EEU37671.1	2 E-56	339/352

Eukaryota	Fungi	Nectria haematococca mpVI 77-13-4	EEU33936.1	1 E-54	334/352
Eukaryota	Fungi	Aspergillus niger CBS 513.88	XP_001396107.1	2 E-54	343/352
Eukaryota	Fungi	Verticillium albo-atrum VaMs.102	EEY16690.1	3 E-54	318/352
Eukaryota	Fungi	Penicillium marneffei ATCC 18224	XP_002149790.1	5 E-54	314/352
Eukaryota	Fungi	Aspergillus flavus NRRL3357	XP_002374547.1	1 E-53	334/352
Eukaryota	Fungi	Penicillium marneffei ATCC 18224	XP_002143358.1	1 E-53	330/352
Eukaryota	Fungi	Talaromyces stipitatus ATCC 10500	XP_002478111.1	3 E-53	311/352
Eukaryota	Fungi	Nectria haematococca mpVI 77-13-4	EEU38867.1	7 E-53	329/352
Eukaryota	Fungi	Nectria haematococca mpVI 77-13-4	EEU37848.1	6 E-52	343/352
Eukaryota	Fungi	Talaromyces stipitatus ATCC 10500	XP_002339998.1	1 E-51	314/352
Eukaryota	Fungi	Aspergillus niger CBS 513.88	XP_001401316.1	3 E-51	343/352
Eukaryota	Fungi	Pyrenophora tritici-repentis Pt-1C-BFP	XP_001938518.1	3 E-51	335/352
Eukaryota	Fungi	Talaromyces stipitatus ATCC 10500	XP_002477931.1	5 E-51	314/352
Eukaryota	Fungi	Gibberella zeae PH-1	XP_391393.1	3 E-49	349/352
Eukaryota	Fungi	Phaeosphaeria nodorum SN15	XP_001798510.1	9 E-48	318/352
Eukaryota	Fungi	Aspergillus nidulans FGSC A4	XP_681852.1	2 E-46	282/352
Eukaryota	Fungi	Chaetomium globosum CBS 148.51	XP_001219508.1	2 E-45	283/352
Eukaryota	Fungi	Coccidioides immitis RS;	XP_001239747.1	5 E-45	325/352
Eukaryota	Fungi	Aspergillus clavatus NRRL 1	XP_001276060.1	3 E-44	318/352
Eukaryota	Fungi	Coccidioides posadasii C735 delta	EER25144.1	6 E-44	324/352
Eukaryota	Fungi	Gibberella zeae PH-1	XP_391655.1	9 E-43	332/352
Eukaryota	Fungi	Nectria haematococca mpVI 77-13-4	EEU37073.1	1 E-42	309/352
Eukaryota	Fungi	Aspergillus clavatus NRRL 1	XP_001273520.1	5 E-40	330/352
Eukaryota	Fungi	Nectria haematococca mpVI 77-13-4	EEU49027.1	4 E-39	329/352
Eukaryota	Fungi	Postia placenta Mad-698-R	XP_002474850.1	2 E-37	282/352
Eukaryota	Fungi	Talaromyces stipitatus ATCC 10500	XP_002483675.1	4 E-37	322/352
Eukaryota	Fungi	Postia placenta Mad-698-R	XP_002477356.1	6 E-37	282/352
Eukaryota	Fungi	Postia placenta Mad-698-R	XP_002474862.1	6 E-37	282/352
Eukaryota	Fungi	Neurospora crassa OR74A	XP_957420.1	8 E-36	341/352
Eukaryota	Fungi	Chaetomium globosum CBS 148.51	XP_001224584.1	1 E-34	338/352
Eukaryota	Fungi	Laccaria bicolor S238N-H82	XP_001884534.1	1 E-32	286/352
Eukaryota	Fungi	Postia placenta Mad-698-R	XP_002468807.1	3 E-32	329/352
Eukaryota	Fungi	Cryptococcus neoformans var. neoformans	XP_570923.1	2 E-31	331/352
Eukaryota	Fungi	Neurospora crassa OR74A	XP_964953.1	2 E-31	333/352
Eukaryota	Fungi	Coprinopsis cinerea okayama7#130	XP_001828233.1	4 E-31	288/352
Eukaryota	Fungi	Aspergillus oryzae RIB40	XP_001827418.1	4 E-31	324/352
Eukaryota	Fungi	Cryptococcus neoformans var. neoformans	XP_570921.1	1 E-30	337/352
Eukaryota	Fungi	Postia placenta Mad-698-R	XP_002477108.1	1 E-30	289/352
Eukaryota	Fungi	Aspergillus flavus NRRL3357	XP_002384616.1	1 E-30	289/352
Eukaryota	Fungi	Vanderwaltozyma polyspora DSM 70294	XP_001644505.1	2 E-29	328/352
Eukaryota	Fungi	Cryptococcus neoformans var. neoformans	XP_571198.1	3 E-29	285/352
Eukaryota	Fungi	Nectria haematococca mpVI 77-13-4	EEU36813.1	8 E-29	330/352
Eukaryota	Fungi	Candida tropicalis MYA-3404	XP_002547481.1	8 E-29	331/352
Eukaryota	Fungi	Ustilago maydis 521	XP_762521.1	9 E-29	346/352
Eukaryota	Fungi	Coprinopsis cinerea okayama7#130	XP_001837919.1	9 E-29	287/352
Eukaryota	Fungi	Aspergillus flavus NRRL3357	XP_002376456.1	1 E-28	285/352
Eukaryota	Fungi	Aspergillus oryzae RIB40	XP_001820731.1	3 E-28	285/352
Eukaryota	Fungi	Coprinopsis cinerea okayama7#130	XP_001837931.1	9 E-28	313/352
Eukaryota	Fungi	Aspergillus oryzae RIB40	XP_001824809.1	1 E-27	350/352
Eukaryota	Fungi	Moniliophthora perniciosa FA553	XP_002394510.1	2 E-27	306/352
Eukaryota	Fungi	Clavispora lusitaniae ATCC 42720	XP_002619074.1	4 E-27	305/352
Eukaryota	Fungi	Candida dubliniensis CD36	XP_002420567.1	5 E-27	331/352
Eukaryota	Fungi	Penicillium marneffei ATCC 18224	XP_002150492.1	1 E-26	290/352
Eukaryota	Fungi	Candida albicans WO-1	EEQ46322.1	2 E-26	327/352
Eukaryota	Fungi	Candida albicans SC5314	XP_720744.1	5 E-26	327/352
Eukaryota	Fungi	Candida tropicalis MYA-3404	XP_002547582.1	4 E-25	327/352
Eukaryota	Fungi	Clavispora lusitaniae ATCC 42720	XP_002617934.1	6 E-25	301/352
Eukaryota	Fungi	Clavispora lusitaniae ATCC 42720	XP_002617933.1	7 E-25	298/352

Eukaryota	Fungi	Lodderomyces elongisporus NRRL YB-4239	XP_001527072.1	8 E-25	334/352
Eukaryota	Fungi	Pichia guilliermondii ATCC 6260	XP_001484542.1	1 E-24	323/352
Eukaryota	Fungi	Candida albicans SC5314	XP_710382.1	2 E-24	339/352
Eukaryota	Fungi	Penicillium chrysogenum Wisconsin 54-1255	XP_002560438.1	2 E-24	320/352
Eukaryota	Fungi	Pichia guilliermondii ATCC 6260	EDK39148.2	2 E-24	318/352
Eukaryota	Fungi	Lodderomyces elongisporus NRRL YB-4239	XP_001527073.1	3 E-24	334/352
Eukaryota	Fungi	Candida dubliniensis CD36	XP_002418481.1	3 E-24	308/352
Eukaryota	Fungi	Pichia guilliermondii ATCC 6260	EDK41616.2	3 E-24	323/352
Eukaryota	Fungi	Pichia guilliermondii ATCC 6260	XP_001485517.1	3 E-24	318/352
Eukaryota	Fungi	Penicillium marneffei ATCC 18224	XP_002151938.1	4 E-24	324/352
Eukaryota	Fungi	Yarrowia lipolytica CLIB122	XP_502592.1	4 E-24	321/352
Eukaryota	Fungi	Candida albicans WO-1	EEQ47208.1	4 E-24	339/352
Eukaryota	Fungi	Candida tropicalis MYA-3404	XP_002547600.1	5 E-24	331/352
Eukaryota	Fungi	Saccharomyces cerevisiae JAY291	EEU06136.1	9 E-24	328/352
Eukaryota	Fungi	Pichia guilliermondii ATCC 6260	XP_001481951.1	9 E-24	325/352
Eukaryota	Fungi	Saccharomyces cerevisiae YJM789	EDN60856.1	9 E-24	328/352
Eukaryota	Fungi	Vanderwaltozyma polyspora DSM 70294	XP_001643691.1	9 E-24	329/352
Eukaryota	Fungi	Pichia pastoris GS115	XP_002493757.1	1 E-23	294/352
Eukaryota	Fungi	Aspergillus terreus NIH2624	XP_001213641.1	1 E-23	324/352
Eukaryota	Fungi	Neosartorya fischeri NRRL 181	XP_001260510.1	1 E-23	308/352
Eukaryota	Fungi	Pichia guilliermondii ATCC 6260	XP_001483204.1	1 E-23	285/352
Eukaryota	Fungi	Pichia stipitis CBS 6054	XP_001382959.2	2 E-23	308/352
Eukaryota	Fungi	Pichia stipitis CBS 6054	XP_001387655.2	2 E-23	307/352
Eukaryota	Fungi	Clavispora lusitaniae ATCC 42720	XP_002617329.1	2 E-23	319/352
Eukaryota	Fungi	Aspergillus fumigatus Af293	XP_755361.1	3 E-23	333/352
Eukaryota	Viridiplantae	Clitoria ternatea	BAF49294.1	3 E-23	323/352
Eukaryota	Fungi	Saccharomyces cerevisiae EC1118	CAY79381.1	3 E-23	327/352
Eukaryota	Fungi	Aspergillus oryzae RIB40	XP_001818640.1	3 E-23	328/352
Eukaryota	Fungi	Saccharomyces cerevisiae	NP_010830.1	3 E-23	328/352
Eukaryota	Fungi	Gibberella zeae PH-1	XP_381652.1	6 E-23	317/352
Eukaryota	Fungi	Pichia guilliermondii ATCC 6260	EDK39163.2	1 E-22	309/352
Eukaryota	Fungi	Pichia guilliermondii ATCC 6260	XP_001485532.1	1 E-22	309/352
Eukaryota	Fungi	Saccharomyces cerevisiae AWRI1631	EDZ69533.1	1 E-22	315/352
Eukaryota	Fungi	Pichia guilliermondii ATCC 6260	EDK40454.2	1 E-22	319/352
Eukaryota	Fungi	Candida tropicalis MYA-3404	XP_002549662.1	1 E-22	310/352
Eukaryota	Fungi	Pichia pastoris GS115	XP_002492673.1	1 E-22	290/352
Eukaryota	Fungi	Pichia guilliermondii ATCC 6260	EDK40794.2	2 E-22	310/352
Eukaryota	Fungi	Kluyveromyces lactis NRRL Y-1140	XP_453553.1	2 E-22	342/352
Eukaryota	Fungi	Clavispora lusitaniae ATCC 42720	XP_002614976.1	2 E-22	290/352
Eukaryota	Fungi	Pichia guilliermondii ATCC 6260	XP_001482937.1	3 E-22	310/352
Eukaryota	Fungi	Malassezia globosa CBS 7966	XP_001732224.1	3 E-22	327/352
Eukaryota	Fungi	Pichia stipitis CBS 6054	XP_001384998.1	3 E-22	323/352
Eukaryota	Fungi	Aspergillus flavus NRRL3357	XP_002380047.1	3 E-22	328/352
Eukaryota	Fungi	Aspergillus nidulans FGSC A4	XP_663581.1	3 E-22	324/352
Eukaryota	Fungi	Candida albicans WO-1	EEQ42302.1	3 E-22	310/352
Eukaryota	Fungi	Saccharomyces cerevisiae YJM789	EDN63727.1	4 E-22	315/352
Eukaryota	Fungi	Clavispora lusitaniae ATCC 42720	XP_002617822.1	4 E-22	305/352
Eukaryota	Fungi	Candida albicans SC5314	XP_723467.1	4 E-22	288/352
Eukaryota	Fungi	Saccharomyces cerevisiae JAY291	EEU07090.1	5 E-22	315/352
Eukaryota	Fungi	Magnaporthe grisea 70-15	XP_001407421.1	6 E-22	284/352
Eukaryota	Fungi	Saccharomyces cerevisiae	NP_014490.1	6 E-22	315/352
Eukaryota	Fungi	Pichia guilliermondii ATCC 6260	XP_001483823.1	7 E-22	319/352
Eukaryota	Viridiplantae	Glycine max	AAD54273.1	9 E-22	324/352
Eukaryota	Fungi	Candida tropicalis MYA-3404	XP_002549837.1	1 E-21	309/352
Eukaryota	Fungi	Ustilago maydis 521	XP_758319.1	1 E-21	364/352
Eukaryota	Fungi	Debaryomyces hansenii	CAG86665.2	1 E-21	323/352
Eukaryota	Fungi	Kluyveromyces lactis NRRL Y-1140	XP_453552.1	1 E-21	296/352
Eukaryota	Fungi	Debaryomyces hansenii CBS767	XP_458533.1	2 E-21	323/352

Eukaryota	Fungi	Nectria haematococca mpVI 77-13-4	EEU38888.1	2 E-21	324/352
Eukaryota	Fungi	Aspergillus flavus NRRL3357	XP_002377895.1	3 E-21	324/352
Eukaryota	Fungi	Pichia pastoris GS115	XP_002492675.1	4 E-21	290/352
Eukaryota	Fungi	Aspergillus niger CBS 513.88	XP_001397780.1	4 E-21	324/352
Eukaryota	Viridiplantae	Brassica rapa subsp. campestris	ACP20258.1	4 E-21	316/352
Eukaryota	Fungi	Penicillium chrysogenum Wisconsin 54-1255	XP_002565949.1	4 E-21	308/352
Eukaryota	Viridiplantae	Glycine max	ACU21070.1	5 E-21	307/352
Eukaryota	Fungi	Zygosaccharomyces rouxii CBS 732	XP_002497423.1	7 E-21	329/352
Eukaryota	Fungi	Phaeosphaeria nodorum SN15	XP_001798314.1	8 E-21	290/352
Eukaryota	Fungi	Ajellomyces capsulatus H143	EER45124.1	8 E-21	330/352
Eukaryota	Fungi	Pichia stipitis CBS 6054	XP_001385392.1	1 E-20	311/352
Eukaryota	Fungi	Aspergillus clavatus NRRL 1	XP_001275327.1	1 E-20	324/352
Eukaryota	Fungi	Zygosaccharomyces rouxii CBS 732	XP_002499370.1	1 E-20	306/352
Eukaryota	Fungi	Botryotinia fuckeliana B05.10	XP_001549714.1	1 E-20	304/352
Eukaryota	Fungi	Candida tropicalis MYA-3404	XP_002546077.1	1 E-20	295/352
Eukaryota	Fungi	Zygosaccharomyces rouxii CBS 732	XP_002498021.1	1 E-20	306/352

AFUA_1G11370

Eukaryota	Fungi	Aspergillus fumigatus Af293	XP_752502.2	0.0	612/612
Eukaryota	Fungi	Neosartorya fischeri NRRL 181	XP_001264628.1	0.0	595/612
Eukaryota	Fungi	Aspergillus clavatus NRRL 1	XP_001270463.1	0.0	614/612
Eukaryota	Fungi	Aspergillus flavus NRRL3357	XP_002373928.1	1 E-154	597/612
Eukaryota	Fungi	Aspergillus niger CBS 513.88	XP_001398576.1	1 E-149	573/612
Eukaryota	Fungi	Aspergillus oryzae RIB40	XP_001820476.1	1 E-149	587/612
Eukaryota	Fungi	Sclerotinia sclerotiorum 1980 UF-70	XP_001587168.1	1 E-146	576/612
Eukaryota	Fungi	Magnaporthe grisea 70-15	XP_359722.1	1 E-144	617/612
Eukaryota	Fungi	Aspergillus oryzae RIB40	XP_001817967.1	1 E-144	573/612
Eukaryota	Fungi	Pyrenophora tritici-repentis Pt-1C-BFP	XP_001937272.1	1 E-141	575/612
Eukaryota	Fungi	Aspergillus nidulans FGSC A4	XP_680638.1	1 E-140	585/612
Eukaryota	Fungi	Penicillium chrysogenum Wisconsin 54-1255	XP_002565328.1	1 E-140	596/612
Eukaryota	Fungi	Aspergillus nidulans FGSC A4	tpeCBF78527.1	1 E-140	590/612
Eukaryota	Fungi	Podospora anserina DSM 980	XP_001910399.1	1 E-139	577/612
Eukaryota	Fungi	Aspergillus flavus NRRL3357	XP_002373140.1	1 E-138	560/612
Eukaryota	Fungi	Aspergillus clavatus NRRL 1	XP_001275639.1	1 E-137	585/612
Eukaryota	Fungi	Botryotinia fuckeliana B05.10	XP_001559633.1	1 E-136	581/612
Eukaryota	Fungi	Microsporum canis CBS 113480	EEQ35647.1	1 E-135	577/612
Eukaryota	Fungi	Penicillium marneffei ATCC 18224	XP_002143250.1	1 E-134	576/612
Eukaryota	Fungi	Podospora anserina DSM 980	XP_001904483.1	1 E-132	615/612
Eukaryota	Fungi	Phaeosphaeria nodorum SN15	XP_001806098.1	1 E-132	581/612
Eukaryota	Fungi	Aspergillus clavatus NRRL 1	XP_001273087.1	1 E-131	577/612
Eukaryota	Fungi	Nectria haematococca mpVI 77-13-4	EEU35073.1	1 E-131	591/612
Eukaryota	Fungi	Aspergillus oryzae RIB40	XP_001727440.1	1 E-131	582/612
Eukaryota	Fungi	Gibberella zeae PH-1	XP_391404.1	1 E-130	591/612
Eukaryota	Fungi	Aspergillus niger CBS 513.88	XP_001396848.1	1 E-130	541/612
Eukaryota	Fungi	Neosartorya fischeri NRRL 181	XP_001260965.1	1 E-128	580/612
Eukaryota	Fungi	Aspergillus clavatus NRRL 1	XP_001270826.1	1 E-126	596/612
Eukaryota	Fungi	Aspergillus niger CBS 513.88	XP_001390806.1	1 E-125	579/612
Eukaryota	Fungi	Aspergillus fumigatus Af293	XP_755835.1	1 E-125	571/612
Eukaryota	Fungi	Sclerotinia sclerotiorum 1980 UF-70	XP_001598641.1	1 E-124	581/612
Eukaryota	Fungi	Aspergillus fumigatus A1163	EDP55006.1	1 E-124	571/612
Eukaryota	Fungi	Botryotinia fuckeliana B05.10	XP_001559357.1	1 E-123	581/612
Eukaryota	Fungi	Talaromyces stipitatus ATCC 10500	XP_002485882.1	1 E-123	578/612
Eukaryota	Fungi	Aspergillus flavus NRRL3357	XP_002375706.1	1 E-121	554/612
Eukaryota	Fungi	Aspergillus flavus NRRL3357	XP_002373620.1	1 E-121	583/612
Eukaryota	Fungi	Aspergillus oryzae RIB40	XP_001818394.1	1 E-121	583/612
Eukaryota	Fungi	Talaromyces stipitatus ATCC 10500	XP_002339960.1	1 E-120	608/612
Eukaryota	Fungi	Chaetomium globosum CBS 148.51	XP_001220751.1	1 E-119	586/612

Eukaryota	Fungi	Neosartorya fischeri NRRL 181	XP_001263633.1	1 E-118	595/612
Eukaryota	Fungi	Aspergillus fumigatus A1163	EDP52931.1	1 E-118	595/612
Eukaryota	Fungi	Pyrenophora tritici-repentis Pt-1C-BFP	XP_001940082.1	1 E-118	563/612
Eukaryota	Fungi	Aspergillus fumigatus Af293	XP_754807.1	1 E-118	595/612
Eukaryota	Fungi	Aspergillus terreus NIH2624	XP_001216476.1	1 E-117	529/612
Eukaryota	Fungi	Aspergillus nidulans FGSC A4	XP_661816.1	1 E-117	574/612
Eukaryota	Fungi	Penicillium marneffei ATCC 18224	XP_002148584.1	1 E-116	589/612
Eukaryota	Fungi	Aspergillus nidulans FGSC A4	XP_660833.1	1 E-115	600/612
Eukaryota	Fungi	Aspergillus oryzae RIB40	XP_001821530.1	1 E-113	570/612
Eukaryota	Fungi	Aspergillus flavus NRRL3357	XP_002379930.1	1 E-113	570/612
Eukaryota	Fungi	Aspergillus flavus	AAS90042.1	1 E-112	570/612
Eukaryota	Fungi	Botryotinia fuckeliana B05.10	XP_001545318.1	1 E-112	560/612
Eukaryota	Fungi	Aspergillus flavus	AAS90106.1	1 E-112	570/612
Eukaryota	Fungi	Aspergillus flavus	AAS90019.1	1 E-112	568/612
Eukaryota	Fungi	Aspergillus nidulans FGSC A4	XP_664049.1	1 E-112	576/612
Eukaryota	Fungi	Aspergillus parasiticus	AAC49318.1	1 E-111	568/612
Eukaryota	Fungi	Mycosphaerella pini	ABO72541.2	1 E-111	572/612
Eukaryota	Fungi	Podospora anserina DSM 980	XP_001911514.1	1 E-110	568/612
Eukaryota	Fungi	Aspergillus nomius	AAS90066.1	1 E-110	570/612
Eukaryota	Fungi	Aspergillus terreus NIH2624	XP_001215452.1	1 E-110	556/612
Eukaryota	Fungi	Pyrenophora tritici-repentis Pt-1C-BFP	XP_001935481.1	1 E-109	586/612
Eukaryota	Fungi	Aspergillus nidulans FGSC A4	tpeCBF80156.1	1 E-107	571/612
Eukaryota	Fungi	Neosartorya fischeri NRRL 181	XP_001261659.1	1 E-107	578/612
Eukaryota	Fungi	Talaromyces stipitatus ATCC 10500	XP_002479433.1	1 E-106	599/612
Eukaryota	Fungi	Aspergillus terreus NIH2624	XP_001209025.1	1 E-106	581/612
Eukaryota	Fungi	Magnaporthe grisea 70-15	XP_001414883.1	1 E-106	539/612
Eukaryota	Fungi	Aspergillus niger CBS 513.88	XP_001400283.1	1 E-106	578/612
Eukaryota	Fungi	Sclerotinia sclerotiorum 1980 UF-70	XP_001592756.1	1 E-105	490/612
Eukaryota	Fungi	Talaromyces stipitatus ATCC 10500	XP_002485672.1	1 E-105	561/612
Eukaryota	Fungi	Phaeosphaeria nodorum SN15	XP_001797048.1	1 E-104	562/612
Eukaryota	Fungi	Aspergillus ochraceoroseus	ACH72898.1	1 E-102	571/612
Eukaryota	Fungi	Botryotinia fuckeliana B05.10	XP_001556658.1	1 E-102	573/612
Eukaryota	Fungi	Phaeosphaeria nodorum SN15	XP_001800211.1	1 E-101	598/612
Eukaryota	Fungi	Ustilago maydis 521	XP_759393.1	7 E-99	587/612
Eukaryota	Fungi	Aspergillus nidulans FGSC A4	XP_681081.1	1 E-96	548/612
Eukaryota	Fungi	Phaeosphaeria nodorum SN15	XP_001793977.1	7 E-92	572/612
Eukaryota	Fungi	Penicillium chrysogenum Wisconsin 54-1255	XP_002567445.1	4 E-88	578/612
Eukaryota	Fungi	Aspergillus niger CBS 513.88	XP_001398522.1	1 E-87	555/612
Eukaryota	Fungi	Aspergillus terreus NIH2624	XP_001211074.1	3 E-83	531/612
Eukaryota	Fungi	Aspergillus niger CBS 513.88	XP_001397016.1	1 E-71	614/612
Bacteria	Proteobacteria	Pseudomonas putida F1	YP_001265431.1	3 E-65	533/612
Bacteria	Proteobacteria	Bradyrhizobium sp. BTAi1	YP_001238278.1	4 E-65	527/612
Bacteria	Proteobacteria	Bradyrhizobium sp. ORS278	YP_001203981.1	3 E-64	527/612
Bacteria	Proteobacteria	Azorhizobium caulinodans ORS 571	YP_001526184.1	3 E-64	532/612
Bacteria	Proteobacteria	Pseudomonas putida KT2440	NP_742226.1	5 E-64	538/612
Bacteria	Proteobacteria	Pseudomonas putida GB-1	YP_001666324.1	1 E-63	533/612
Bacteria	Proteobacteria	Oceanicaulis alexandrii HTCC2633	ZP_00957061.1	5 E-63	530/612
Bacteria	Proteobacteria	Pseudomonas entomophila L48	YP_605824.1	7 E-63	530/612
Bacteria	Candidatus	Candidatus Pelagibacter ubique HTCC1062	YP_266277.1	2 E-62	527/612
Bacteria	Proteobacteria	Ralstonia pickettii 12J	YP_001901091.1	2 E-62	527/612
Bacteria	Proteobacteria	Polynucleobacter necessarius subsp. asymbiotic	YP_001155137.1	4 E-62	535/612
Bacteria	Proteobacteria	Photobacterium profundum 3TCK	ZP_01222730.1	5 E-62	538/612
Bacteria	Proteobacteria	Bordetella petrii DSM 12804	YP_001633470.1	5 E-62	530/612
Bacteria	Proteobacteria	Burkholderia cenocepacia PC184	ZP_04939997.1	1 E-61	529/612
Bacteria	Proteobacteria	Ralstonia eutropha H16	YP_728088.1	1 E-61	534/612
Bacteria	Proteobacteria	Photobacterium profundum SS9	YP_130410.1	1 E-61	538/612
Bacteria	Proteobacteria	Burkholderia multivorans CGD2M	ZP_03575238.1	1 E-61	529/612
Bacteria	Proteobacteria	Rhodopseudomonas palustris BisA53	YP_782821.1	2 E-61	528/612

Bacteria	Proteobacteria	Stenotrophomonas maltophilia	BAF45126.1	2 E-61	530/612
Bacteria	Proteobacteria	Pseudomonas putida W619	YP_001746950.1	3 E-61	533/612
Bacteria	Proteobacteria	Sphingopyxis terrae	BAB61732.1	3 E-61	530/612
Bacteria	Proteobacteria	Pseudomonas fluorescens Pf0-1	YP_349087.1	4 E-61	527/612
Bacteria	Proteobacteria	Burkholderia cenocepacia J2315	YP_002229178.1	4 E-61	529/612
Bacteria	Proteobacteria	Sphingopyxis macrogoltabida	BAB07804.1	4 E-61	530/612
Bacteria	Proteobacteria	Burkholderia cenocepacia MC0-3	YP_001763402.1	5 E-61	529/612
Bacteria	Proteobacteria	Burkholderia sp. 383	YP_367509.1	5 E-61	529/612
Bacteria	Proteobacteria	Roseobacter sp. MED193	ZP_01055291.1	5 E-61	534/612
Bacteria	Proteobacteria	Burkholderia multivorans CGD1	ZP_03587285.1	5 E-61	529/612
Bacteria	Proteobacteria	Burkholderia multivorans ATCC 17616	YP_001578274.1	6 E-61	529/612
Bacteria	Proteobacteria	Burkholderia multivorans ATCC 17616	YP_001947593.1	6 E-61	529/612
Bacteria	Proteobacteria	Ralstonia solanacearum MolK2	YP_002252541.1	6 E-61	532/612
Bacteria	Proteobacteria	Sphingomonas sp. EK-1	BAF45123.1	8 E-61	530/612
Bacteria	Proteobacteria	Acidovorax delafieldii 2AN	ZP_04764988.1	8 E-61	548/612
Bacteria	Proteobacteria	Burkholderia phytofirmans PsJN	YP_001897374.1	9 E-61	535/612
Bacteria	Proteobacteria	Burkholderia ambifaria IOP40-10	ZP_02890074.1	1 E-60	529/612
Bacteria	Proteobacteria	Burkholderia cenocepacia AU 1054	YP_622842.1	2 E-60	529/612
Bacteria	Proteobacteria	Burkholderia vietnamiensis G4	YP_001117950.1	2 E-60	529/612
Bacteria	Proteobacteria	Ralstonia solanacearum GMI1000	NP_521464.1	2 E-60	528/612
Bacteria	Proteobacteria	Pseudomonas sp. PE-2	BAF45124.1	3 E-60	530/612
Bacteria	Actinobacteria	Mycobacterium gilvum PYR-GCK	YP_001134495.1	3 E-60	555/612
Bacteria	Proteobacteria	Rhodobacterales bacterium HTCC2255	ZP_01446736.1	3 E-60	535/612
Bacteria	Proteobacteria	Ralstonia solanacearum UW551	ZP_00946537.1	3 E-60	532/612
Bacteria	Proteobacteria	Ralstonia pickettii 12D	YP_002983153.1	3 E-60	527/612
Bacteria	Proteobacteria	Pseudovibrio sp. JE062	ZP_05085589.1	4 E-60	525/612
Bacteria	Proteobacteria	Burkholderia phytofirmans PsJN	YP_001897527.1	5 E-60	529/612
Bacteria	Proteobacteria	Burkholderia ubonensis Bu	ZP_02380339.1	5 E-60	529/612
Bacteria	Proteobacteria	Alteromonas macleodii 'Deep ecotype'	YP_002125469.1	5 E-60	537/612
Bacteria	Proteobacteria	Burkholderia dolosa AUO158	ZP_04944312.1	5 E-60	543/612
Bacteria	Proteobacteria	Caulobacter crescentus CB15	NP_419761.1	6 E-60	544/612
Bacteria	Proteobacteria	Ralstonia pickettii 12J	YP_001901188.1	8 E-60	540/612
Bacteria	Proteobacteria	Sphingopyxis macrogoltabida	BAF98451.1	9 E-60	530/612
Bacteria	Proteobacteria	Ralstonia eutropha JMP134	YP_297574.1	1 E-59	536/612
Bacteria	Proteobacteria	Burkholderia xenovorans LB400	YP_560809.1	1 E-59	536/612
Eukaryota	Fungi	Nectria haematococca mpVI 77-13-4	EEU37471.1	1 E-59	592/612
Bacteria	Proteobacteria	Burkholderia ambifaria MC40-6	YP_001806802.1	2 E-59	529/612
Bacteria	Proteobacteria	Burkholderia graminis C4D1M	ZP_02887167.1	2 E-59	535/612
Bacteria	Proteobacteria	Pseudomonas fluorescens Pf-5	YP_259594.1	2 E-59	526/612
Bacteria	Proteobacteria	Burkholderia sp. H160	ZP_03264459.1	2 E-59	529/612
Bacteria	Proteobacteria	Burkholderia sp. 383	YP_366469.1	2 E-59	533/612
Bacteria	Proteobacteria	Burkholderia ambifaria AMMD	YP_771968.1	3 E-59	529/612
Bacteria	Proteobacteria	Burkholderia ambifaria MEX-5	ZP_02905080.1	3 E-59	529/612
Bacteria	Proteobacteria	Sphingomonas wittichii RW1	YP_001265285.1	3 E-59	527/612
Bacteria	Proteobacteria	Cupriavidus taiwanensis	YP_002008190.1	3 E-59	536/612
Bacteria	Proteobacteria	Pseudomonas fluorescens SBW25	YP_002871776.1	3 E-59	525/612
Bacteria	Proteobacteria	Caulobacter sp. K31	YP_001682976.1	4 E-59	546/612
Bacteria	Proteobacteria	Pseudoalteromonas atlantica T6c	YP_662346.1	4 E-59	526/612
Bacteria	Proteobacteria	Burkholderia xenovorans LB400	YP_560963.1	5 E-59	529/612
Bacteria	Proteobacteria	Vibrio coralliilyticus ATCC BAA-450	ZP_05888080.1	8 E-59	541/612
Bacteria	Proteobacteria	Sphingomonas wittichii RW1	YP_001262083.1	9 E-59	524/612
Bacteria	Proteobacteria	Pseudomonas entomophila L48	YP_608181.1	1 E-58	539/612
Bacteria	Proteobacteria	Alcanivorax sp. DG881	ZP_05040520.1	1 E-58	522/612
Bacteria	Proteobacteria	Rhodopseudomonas palustris BisB18	YP_533645.1	1 E-58	528/612
Bacteria	Proteobacteria	Burkholderia graminis C4D1M	ZP_02885452.1	1 E-58	529/612
Bacteria	Proteobacteria	Curvibacter putative symbiont of	CBA30511.1	2 E-58	530/612
Bacteria	Proteobacteria	Ralstonia pickettii 12D	YP_002983249.1	2 E-58	540/612
Bacteria	Proteobacteria	Rhizobium leguminosarum bv. trifolii	YP_002278603.1	2 E-58	550/612

Bacteria	Proteobacteria	Ralstonia metallidurans CH34	YP_587146.1	3 E-58	537/612
Eukaryota	Fungi	Ajellomyces dermatitidis SLH14081	XP_002622246.1	3 E-58	591/612
Bacteria	Proteobacteria	Acidovorax citrulli AAC00-1	YP_968686.1	3 E-58	556/612
Bacteria	Proteobacteria	Burkholderia ambifaria MC40-6	YP_001811486.1	3 E-58	539/612
Bacteria	Proteobacteria	Roseovarius sp. HTCC2601	ZP_01444019.1	4 E-58	523/612
Bacteria	Proteobacteria	Pseudovibrio sp. JE062	ZP_05086419.1	4 E-58	525/612
Bacteria	Proteobacteria	Rhizobium sp. NGR234	YP_002822699.1	4 E-58	551/612
Bacteria	Proteobacteria	Pseudomonas stutzeri A1501	YP_001171160.1	5 E-58	525/612
Bacteria	Proteobacteria	Limnobacter sp. MED105	ZP_01916549.1	5 E-58	545/612
Bacteria	Proteobacteria	Burkholderia glumae BGR1	YP_002909966.1	5 E-58	529/612
Bacteria	Proteobacteria	Ralstonia eutropha H16	YP_841363.1	7 E-58	536/612
Bacteria	Proteobacteria	Ruegeria pomeroyi DSS-3	YP_166223.1	8 E-58	523/612
Bacteria	Proteobacteria	Oligotropha carboxidovorans OM5	YP_002289724.1	8 E-58	529/612
Bacteria	Proteobacteria	Bradyrhizobium japonicum USDA 110	NP_774525.1	9 E-58	525/612
Bacteria	Proteobacteria	Bermanella marisrubri	ZP_01306234.1	1 E-57	526/612
Bacteria	Proteobacteria	Ruegeria sp. R11	ZP_05089337.1	1 E-57	525/612
Bacteria	Proteobacteria	Cupriavidus taiwanensis	YP_002007099.1	1 E-57	536/612
Bacteria	Actinobacteria	Mycobacterium sp. MCS	YP_639792.1	1 E-57	554/612
Bacteria	Actinobacteria	Mycobacterium marinum M	YP_001851248.1	2 E-57	554/612
Bacteria	Proteobacteria	Roseobacter sp. GAI101	ZP_05101564.1	2 E-57	533/612
Bacteria	Proteobacteria	Burkholderia sp. 383	YP_372762.1	2 E-57	531/612
Bacteria	Proteobacteria	Burkholderia sp. H160	ZP_03264661.1	2 E-57	529/612
Bacteria	Proteobacteria	Bordetella avium 197N	YP_784649.1	2 E-57	520/612
Bacteria	Proteobacteria	Methylibium petroleiphilum PM1	YP_001022991.1	3 E-57	533/612
Bacteria	Proteobacteria	Marinobacter aquaeolei VT8	YP_957725.1	3 E-57	528/612
Bacteria	Proteobacteria	Burkholderia phymatum STM815	YP_001859265.1	4 E-57	529/612
Bacteria	Proteobacteria	Burkholderia multivorans CGD1	ZP_03585612.1	4 E-57	535/612
Bacteria	Proteobacteria	Burkholderia sp. H160	ZP_03266156.1	4 E-57	524/612
Bacteria	Proteobacteria	Pseudomonas aeruginosa 2192	ZP_04935724.1	4 E-57	533/612
Bacteria	Proteobacteria	Pseudomonas aeruginosa PACS2	ZP_01367142.1	5 E-57	533/612
Bacteria	Proteobacteria	Verminephrobacter eiseniae EF01-2	YP_999005.1	5 E-57	536/612
Bacteria	Proteobacteria	Marinobacter algicola DG893	ZP_01894771.1	6 E-57	544/612
Bacteria	Proteobacteria	Methylobacterium nodulans ORS 2060	YP_002500414.1	7 E-57	532/612
Bacteria	Proteobacteria	Verminephrobacter eiseniae EF01-2	YP_995708.1	8 E-57	550/612
Bacteria	Proteobacteria	Pseudomonas aeruginosa PA7	YP_001346810.1	8 E-57	533/612
Bacteria	Proteobacteria	Burkholderia xenovorans LB400	YP_554605.1	8 E-57	531/612
Bacteria	Proteobacteria	alpha proteobacterium BAL199	ZP_02191362.1	8 E-57	528/612
Bacteria	Proteobacteria	Psychrobacter cryohalolentis K5	YP_581107.1	1 E-56	530/612
Bacteria	Proteobacteria	Burkholderia cenocepacia J2315	YP_002233786.1	1 E-56	536/612
Bacteria	Proteobacteria	Ralstonia metallidurans CH34	YP_585662.1	1 E-56	531/612
Bacteria	Proteobacteria	Leptothrix cholodnii SP-6	YP_001789228.1	1 E-56	544/612
Bacteria	Proteobacteria	Pseudomonas aeruginosa C3719	ZP_04929943.1	1 E-56	533/612
Bacteria	Proteobacteria	Ruegeria pomeroyi DSS-3	YP_165460.1	1 E-56	530/612
Bacteria	Proteobacteria	Pseudomonas aeruginosa UCBPP-PA14	YP_789454.1	2 E-56	533/612
Bacteria	Proteobacteria	Burkholderia ubonensis Bu	ZP_02377531.1	2 E-56	525/612
Bacteria	Proteobacteria	Burkholderia ambifaria AMMD	YP_776180.1	2 E-56	536/612
Bacteria	Proteobacteria	Pseudomonas aeruginosa PAO1	NP_252399.1	2 E-56	533/612
Bacteria	Actinobacteria	Streptomyces hygroscopicus ATCC 53653	ZP_05520070.1	2 E-56	512/612
Bacteria	Actinobacteria	Streptomyces viridochromogenes DSM 40736	ZP_05536544.1	2 E-56	510/612
Bacteria	Proteobacteria	Pseudovibrio sp. JE062	ZP_05085930.1	2 E-56	538/612
Bacteria	Proteobacteria	Burkholderia thailandensis MSMB43	ZP_02461782.1	2 E-56	529/612
Bacteria	Proteobacteria	Burkholderia cenocepacia MC0-3	YP_001774488.1	2 E-56	529/612
Bacteria	Proteobacteria	Burkholderia thailandensis E264	YP_440752.1	2 E-56	525/612
Bacteria	Proteobacteria	Burkholderia thailandensis Bt4	ZP_02386160.1	3 E-56	525/612
Bacteria	Proteobacteria	Pseudomonas aeruginosa LESB58	YP_002438878.1	3 E-56	533/612
Bacteria	Proteobacteria	Burkholderia oklahomensis EO147	ZP_02354055.1	3 E-56	536/612
Bacteria	Proteobacteria	Halomonas sp. HTNK1	ACV84069.1	3 E-56	544/612
Bacteria	Proteobacteria	alpha proteobacterium BAL199	ZP_02187363.1	3 E-56	530/612

Bacteria	Proteobacteria	Pseudomonas mendocina ymp	YP_001188857.1	4 E-56	534/612
Bacteria	Proteobacteria	Vibrio shilonii AK1	ZP_01867788.1	4 E-56	526/612
Bacteria	Proteobacteria	Rhodopseudomonas palustris HaA2	YP_487451.1	4 E-56	503/612
Bacteria	Proteobacteria	Novosphingobium aromaticivorans DSM 12444	YP_001166065.1	4 E-56	528/612
Bacteria	Proteobacteria	Comamonas testosteroni KF-1	ZP_03545577.1	4 E-56	546/612
Bacteria	Proteobacteria	Ruegeria pomeroyi DSS-3	YP_167582.1	4 E-56	526/612
Bacteria	Proteobacteria	Oceanicola batsensis HTCC2597	ZP_00998644.1	5 E-56	524/612
Bacteria	Proteobacteria	Bradyrhizobium sp. ORS278	YP_001208258.1	5 E-56	524/612
Bacteria	Proteobacteria	Bradyrhizobium japonicum USDA 110	NP_769037.1	5 E-56	527/612
Bacteria	Proteobacteria	Psychrobacter arcticus 273-4	YP_264896.1	5 E-56	530/612
Bacteria	Proteobacteria	Pseudomonas putida	Q9WWW2.1	5 E-56	529/612
Bacteria	Proteobacteria	Burkholderia multivorans ATCC 17616	YP_001581248.1	5 E-56	525/612
Bacteria	Proteobacteria	Alcanivorax borkumensis SK2	YP_691907.1	6 E-56	528/612
Bacteria	Proteobacteria	Burkholderia multivorans ATCC 17616	YP_001584499.1	6 E-56	535/612
Bacteria	Candidatus	Candidatus Pelagibacter sp. HTCC7211	ZP_05069105.1	7 E-56	529/612
Bacteria	Proteobacteria	Burkholderia oklahomensis C6786	ZP_02361232.1	7 E-56	536/612
Bacteria	Proteobacteria	Delftia acidovorans SPH-1	YP_001566960.1	8 E-56	543/612
Bacteria	Proteobacteria	Rhodobacterales bacterium HTCC2150	ZP_01743515.1	9 E-56	535/612
Bacteria	Proteobacteria	Burkholderia multivorans CGD2M	ZP_03569823.1	1 E-55	535/612
Bacteria	Proteobacteria	Labrenzia aggregata IAM 12614	ZP_01545876.1	1 E-55	527/612
Bacteria	Proteobacteria	Marinobacter aquaeolei VT8	YP_957894.1	1 E-55	537/612
Bacteria	Actinobacteria	Mycobacterium smegmatis str. MC2	YP_887681.1	1 E-55	554/612
Bacteria	Proteobacteria	Burkholderia phytofirmans PsJN	YP_001888124.1	2 E-55	531/612
Bacteria	Proteobacteria	Burkholderia vietnamiensis G4	YP_001120981.1	2 E-55	529/612
Bacteria	Proteobacteria	Roseovarius sp. 217	ZP_01037150.1	2 E-55	538/612
Bacteria	Proteobacteria	Pseudomonas putida	BAD07371.1	2 E-55	523/612
Bacteria	Proteobacteria	Hyphomonas neptunium ATCC 15444	YP_760283.1	2 E-55	525/612
Bacteria	Proteobacteria	Burkholderia phymatum STM815	YP_001858600.1	2 E-55	541/612
Bacteria	Proteobacteria	Ralstonia solanacearum MolK2	YP_002255997.1	2 E-55	542/612
Bacteria	Proteobacteria	Burkholderia glumae BGR1	YP_002907583.1	3 E-55	526/612
Bacteria	Proteobacteria	Silicibacter lacuscaerulensis ITI-1157	ZP_05785341.1	4 E-55	527/612

AFUA_1G14510

Eukaryota	Fungi	Aspergillus fumigatus Af293	XP_752820.1	0.0	1087/1087
Eukaryota	Fungi	Neosartorya fischeri NRRL 181	XP_001264307.1	0.0	1086/1087
Eukaryota	Fungi	Aspergillus clavatus NRRL 1	XP_001268811.1	0.0	1105/1087
Eukaryota	Fungi	Aspergillus terreus NIH2624	XP_001210735.1	0.0	1099/1087
Eukaryota	Fungi	Aspergillus niger CBS 513.88	XP_001389655.1	0.0	1085/1087
Eukaryota	Fungi	Aspergillus oryzae RIB40	XP_001826007.1	0.0	1108/1087
Eukaryota	Fungi	Aspergillus flavus NRRL3357	XP_002377654.1	0.0	1108/1087
Eukaryota	Fungi	Penicillium chrysogenum Wisconsin 54-1255	XP_002561613.1	0.0	1092/1087
Eukaryota	Fungi	Aspergillus nidulans FGSC A4	XP_658386.1	0.0	1085/1087
Eukaryota	Fungi	Penicillium marneffei ATCC 18224	XP_002145964.1	0.0	1111/1087
Eukaryota	Fungi	Talaromyces stipitatus ATCC 10500	XP_002478261.1	0.0	1022/1087
Eukaryota	Fungi	Pyrenophora tritici-repentis Pt-1C-BFP	XP_001931440.1	0.0	1119/1087
Eukaryota	Fungi	Phaeosphaeria nodorum SN15	XP_001799438.1	0.0	1186/1087
Eukaryota	Fungi	Magnaporthe grisea 70-15	XP_001404116.1	0.0	1021/1087

AFUA_1G14520

Eukaryota	Fungi	Aspergillus fumigatus Af293	XP_752821.1	0.0	545/545
Eukaryota	Fungi	Neosartorya fischeri NRRL 181	XP_001264306.1	0.0	545/545
Eukaryota	Fungi	Aspergillus clavatus NRRL 1	XP_001268810.1	0.0	559/545
Eukaryota	Fungi	Aspergillus niger CBS 513.88	XP_001389656.1	0.0	530/545
Eukaryota	Fungi	Aspergillus terreus NIH2624	XP_001210739.1	0.0	524/545
Eukaryota	Fungi	Aspergillus oryzae RIB40	XP_001826006.1	0.0	524/545
Eukaryota	Fungi	Aspergillus flavus NRRL3357	XP_002377653.1	0.0	524/545

Eukaryota	Fungi	Penicillium chrysogenum Wisconsin 54-1255	XP_002561727.1	0.0	514/545
Eukaryota	Fungi	Aspergillus nidulans FGSC A4	tpeCBF82129.1	0.0	502/545
Eukaryota	Fungi	Talaromyces stipitatus ATCC 10500	XP_002478248.1	0.0	515/545
Eukaryota	Fungi	Penicillium marneffei ATCC 18224	XP_002145958.1	0.0	536/545
Eukaryota	Fungi	Ajellomyces dermatitidis SLH14081	XP_002622081.1	0.0	496/545
Eukaryota	Fungi	Ajellomyces dermatitidis ER-3	EEQ89733.1	0.0	496/545
Eukaryota	Fungi	Aspergillus nidulans FGSC A4	XP_662911.1	0.0	483/545
Eukaryota	Fungi	Coccidioides immitis RS;	XP_001243715.1	0.0	503/545
Eukaryota	Fungi	Coccidioides posadasii C735 delta	EER26826.1	0.0	503/545
Eukaryota	Fungi	Microsporum canis CBS 113480	EEQ33776.1	0.0	512/545
Eukaryota	Fungi	Ajellomyces capsulatus G186AR	EEH09736.1	1 E-177	497/545
Eukaryota	Fungi	Ajellomyces capsulatus NAm1	XP_001542133.1	1 E-163	452/545
Eukaryota	Fungi	Uncinocarpus reesii 1704	XP_002582259.1	1 E-158	447/545
Eukaryota	Fungi	Botryotinia fuckeliana B05.10	XP_001555551.1	1 E-117	542/545
Eukaryota	Fungi	Sclerotinia sclerotiorum 1980 UF-70	XP_001585896.1	1 E-116	534/545
Eukaryota	Fungi	Pyrenophora tritici-repentis Pt-1C-BFP	XP_001941949.1	1 E-105	480/545
Eukaryota	Fungi	Magnaporthe grisea 70-15	XP_359778.2	1 E-102	470/545
Eukaryota	Fungi	Neurospora crassa OR74A	XP_956666.2	1 E-93	497/545
Eukaryota	Fungi	Coprinopsis cinerea okayama7#130	XP_001837766.1	2 E-93	453/545
Eukaryota	Fungi	Nectria haematococca mpVI 77-13-4	EEU46578.1	2 E-92	454/545
Eukaryota	Fungi	Gibberella zeae PH-1	XP_387439.1	8 E-92	441/545
Eukaryota	Fungi	Laccaria bicolor S238N-H82	XP_001876977.1	5 E-89	442/545
Eukaryota	Fungi	Podospora anserina DSM 980	XP_001907841.1	1 E-86	495/545
Eukaryota	Fungi	Chaetomium globosum CBS 148.51	XP_001229437.1	2 E-85	501/545
Eukaryota	Fungi	Postia placenta Mad-698-R	XP_002473803.1	2 E-80	452/545
Eukaryota	Fungi	Botryotinia fuckeliana B05.10	XP_001554899.1	4 E-66	452/545
Eukaryota	stramenopiles	Phaeodactylum tricornutum CCAP 1055/1	XP_002179552.1	1 E-65	481/545
Eukaryota	Fungi	Sclerotinia sclerotiorum 1980 UF-70	XP_001585753.1	1 E-63	482/545
Eukaryota	Viridiplantae	Oryza sativa Japonica Group	NP_001058394.1	1 E-61	526/545
Eukaryota	Viridiplantae	Solanum tuberosum	CAB52797.1	5 E-61	540/545
Eukaryota	Viridiplantae	Vitis vinifera	XP_002274469.1	8 E-61	528/545
Eukaryota	Fungi	Ustilago maydis 521	XP_757559.1	2 E-60	490/545
Eukaryota	Viridiplantae	Vitis vinifera	CAN59739.1	2 E-60	526/545
Eukaryota	Fungi	Ustilago maydis 521	XP_758311.1	5 E-60	464/545
Eukaryota	Metazoa	Hydra magnipapillata	XP_002159552.1	1 E-59	543/545
Eukaryota	Fungi	Candida albicans WO-1	EEQ44387.1	2 E-59	488/545
Eukaryota	Fungi	Candida albicans SC5314	XP_720167.1	2 E-59	488/545
Eukaryota	Viridiplantae	Physcomitrella patens subsp. patens	XP_001766162.1	4 E-59	529/545
Eukaryota	Fungi	Ajellomyces capsulatus NAm1	XP_001544258.1	5 E-59	477/545
Eukaryota	Fungi	Candida dubliniensis CD36	XP_002419266.1	1 E-58	488/545
Eukaryota	Fungi	Pyrenophora tritici-repentis Pt-1C-BFP	XP_001940280.1	2 E-58	467/545
Eukaryota	Fungi	Pichia stipitis CBS 6054	XP_001386827.1	2 E-58	477/545
Eukaryota	Viridiplantae	Sorghum bicolor;	XP_002456448.1	3 E-58	522/545
Eukaryota	Fungi	Ajellomyces capsulatus G186AR	EEH03668.1	4 E-58	477/545
Eukaryota	Fungi	Clavispora lusitaniae ATCC 42720	XP_002615374.1	4 E-58	485/545
Eukaryota	Fungi	Aspergillus nidulans FGSC A4	XP_658698.1	1 E-57	468/545
Eukaryota	Viridiplantae	Oryza sativa Japonica Group	NP_001055220.1	2 E-57	520/545
Eukaryota	Viridiplantae	Zea mays;	NP_001142169.1	2 E-57	522/545
Eukaryota	Fungi	Botryotinia fuckeliana B05.10	XP_001553952.1	3 E-57	465/545
Eukaryota	Viridiplantae	Physcomitrella patens subsp. patens	XP_001764062.1	3 E-57	520/545
Eukaryota	Fungi	Candida tropicalis MYA-3404	XP_002548308.1	3 E-57	488/545
Eukaryota	Viridiplantae	Sorghum bicolor;	XP_002438881.1	3 E-57	526/545
Eukaryota	Viridiplantae	Micromonas pusilla CCMP1545	EEH59133.1	4 E-57	539/545
Eukaryota	Fungi	Podospora anserina DSM 980	XP_001907894.1	5 E-57	465/545
Eukaryota	Fungi	Laccaria bicolor S238N-H82	XP_001889510.1	5 E-57	498/545
Eukaryota	Fungi	Ajellomyces dermatitidis SLH14081	XP_002623361.1	6 E-57	477/545
Eukaryota	Fungi	Sclerotinia sclerotiorum 1980 UF-70	XP_001595102.1	7 E-57	465/545
Eukaryota	Viridiplantae	Vitis vinifera	XP_002274523.1	1 E-56	523/545

Eukaryota	Fungi	Coccidioides immitis RS;	XP_001239430.1	1 E-56	467/545
Eukaryota	Fungi	Coccidioides posadasii C735 delta	EER24894.1	1 E-56	467/545
Eukaryota	Fungi	Lodderomyces elongisporus NRRL YB-4239	XP_001527240.1	2 E-56	488/545
Eukaryota	Viridiplantae	Vitis vinifera	CAO41237.1	2 E-56	523/545
Eukaryota	Fungi	Paracoccidioides brasiliensis Pb01;	EEH40984.1	2 E-56	468/545
Eukaryota	Fungi	Paracoccidioides brasiliensis Pb03;	EEH21181.1	2 E-56	477/545
Eukaryota	Fungi	Chaetomium globosum CBS 148.51	XP_001229543.1	2 E-56	465/545
Eukaryota	Fungi	Debaryomyces hansenii	CAG86934.2	4 E-56	497/545
Eukaryota	Fungi	Lodderomyces elongisporus NRRL YB-4239	XP_001527696.1	4 E-56	483/545
Eukaryota	Fungi	Aspergillus clavatus NRRL 1	XP_001269074.1	4 E-56	517/545
Eukaryota	stramenopiles	Phaeodactylum tricornutum CCAP 1055/1	XP_002179088.1	5 E-56	441/545
Eukaryota	Viridiplantae	Populus trichocarpa	XP_002305005.1	5 E-56	437/545
Eukaryota	Fungi	Neurospora crassa OR74A	XP_959008.1	5 E-56	465/545
Eukaryota	Fungi	Neosartorya fischeri NRRL 181	XP_001264565.1	7 E-56	467/545
Eukaryota	Fungi	Candida tropicalis MYA-3404	XP_002547805.1	7 E-56	496/545
Eukaryota	Fungi	Vanderwaltozyma polyspora DSM 70294	XP_001645375.1	8 E-56	463/545
Eukaryota	Fungi	Penicillium marneffei ATCC 18224	XP_002149337.1	1 E-55	468/545
Eukaryota	Fungi	Magnaporthe grisea 70-15	XP_369761.1	1 E-55	487/545
Eukaryota	Fungi	Nectria haematococca mpVI 77-13-4	EEU39397.1	2 E-55	534/545
Eukaryota	Fungi	Debaryomyces hansenii CBS767	XP_458790.1	2 E-55	497/545
Eukaryota	Fungi	Penicillium chrysogenum Wisconsin 54-1255	XP_002558326.1	2 E-55	518/545
Eukaryota	Fungi	Aspergillus terreus NIH2624	XP_001210480.1	2 E-55	468/545
Eukaryota	Viridiplantae	Populus trichocarpa	XP_002319384.1	3 E-55	536/545
Eukaryota	Fungi	Pichia guilliermondii ATCC 6260	EDK38335.2	4 E-55	489/545
Eukaryota	Viridiplantae	Ricinus communis	XP_002521423.1	4 E-55	437/545
Eukaryota	Fungi	Pichia guilliermondii ATCC 6260	XP_001484704.1	6 E-55	489/545
Eukaryota	Amoebozoa	Dictyostelium discoideum AX4	XP_635825.1	7 E-55	532/545
Eukaryota	Fungi	Ajellomyces dermatitidis ER-3	EEQ90707.1	8 E-55	461/545
Eukaryota	Fungi	Nectria haematococca mpVI 77-13-4	EEU39104.1	1 E-54	516/545
Eukaryota	Viridiplantae	Populus trichocarpa	XP_002317236.1	4 E-54	436/545
Eukaryota	Fungi	Gibberella zeae PH-1	XP_382653.1	5 E-54	537/545
Eukaryota	Fungi	Aspergillus niger CBS 513.88	XP_001392541.1	5 E-54	466/545
Eukaryota	Fungi	Verticillium albo-atrum VaMs.102	EEY22232.1	1 E-53	565/545
Eukaryota	Fungi	Gibberella zeae PH-1	XP_384306.1	1 E-53	515/545
Eukaryota	Fungi	Candida albicans WO-1	EEQ46660.1	3 E-53	483/545
Eukaryota	Fungi	Talaromyces stipitatus ATCC 10500	XP_002484822.1	4 E-53	468/545
Eukaryota	Fungi	Paracoccidioides brasiliensis Pb18;	EEH45832.1	4 E-53	493/545
Eukaryota	Viridiplantae	Populus trichocarpa	XP_002319383.1	9 E-53	540/545
Eukaryota	Fungi	Candida albicans SC5314	XP_718052.1	1 E-52	483/545
Eukaryota	Fungi	Vanderwaltozyma polyspora DSM 70294	XP_001645632.1	1 E-52	463/545
Eukaryota	Viridiplantae	Vitis vinifera	XP_002274690.1	2 E-52	525/545
Eukaryota	Fungi	Phaeosphaeria nodorum SN15	XP_001801004.1	2 E-52	468/545
Eukaryota	Viridiplantae	Physcomitrella patens subsp. patens	XP_001769969.1	2 E-52	453/545
Eukaryota	Viridiplantae	Arabidopsis thaliana	NP_180560.1	3 E-52	465/545
Eukaryota	Viridiplantae	Vitis vinifera	XP_002277505.1	3 E-52	437/545
Eukaryota	Fungi	Aspergillus fumigatus Af293	XP_752562.1	3 E-52	489/545
Eukaryota	Fungi	Saccharomyces cerevisiae	NP_013586.1	5 E-52	482/545
Eukaryota	Fungi	Saccharomyces cerevisiae YJM789	EDN64274.1	6 E-52	482/545
Eukaryota	Fungi	Saccharomyces cerevisiae RM11-1a	EDV11392.1	7 E-52	482/545
Eukaryota	Viridiplantae	Populus trichocarpa	ABK95883.1	9 E-52	463/545
Eukaryota	Viridiplantae	Micromonas sp. RCC299	XP_002502044.1	1 E-51	526/545
Eukaryota	Viridiplantae	Arabidopsis thaliana	AAM61225.1	1 E-51	450/545
Eukaryota	Viridiplantae	Oryza sativa Japonica Group	EEE55618.1	2 E-51	436/545
Eukaryota	Viridiplantae	Populus trichocarpa	XP_002313376.1	2 E-51	437/545
Eukaryota	Viridiplantae	Oryza sativa Indica Group	EEC71737.1	2 E-51	436/545
Eukaryota	Fungi	Candida dubliniensis CD36	XP_002421074.1	2 E-51	483/545
Eukaryota	Viridiplantae	Arabidopsis thaliana	NP_563783.1	2 E-51	450/545
Eukaryota	Fungi	Yarrowia lipolytica CLIB122	XP_505856.1	3 E-51	471/545

Eukaryota	Viridiplantae	Ostreococcus tauri	CAL52475.1	4 E-51	524/545
Eukaryota	Fungi	Ustilago maydis 521	XP_759816.1	5 E-51	571/545
Eukaryota	Fungi	Malassezia globosa CBS 7966	XP_001729302.1	5 E-51	555/545
Eukaryota	Fungi	Zygosaccharomyces rouxii CBS 732	XP_002494725.1	1 E-50	459/545
Eukaryota	Viridiplantae	Micromonas sp. RCC299	XP_002502745.1	1 E-50	512/545
Eukaryota	Amoebozoa	Dictyostelium discoideum AX4	XP_645570.1	1 E-50	482/545
Eukaryota	Viridiplantae	Sorghum bicolor;	XP_002460866.1	1 E-50	438/545
Eukaryota	Viridiplantae	Vitis vinifera	CAN66469.1	1 E-50	525/545
Eukaryota	Viridiplantae	Vitis vinifera	XP_002262771.1	2 E-50	438/545
Eukaryota	Viridiplantae	Populus trichocarpa	XP_002319927.1	2 E-50	530/545
Eukaryota	Fungi	Candida glabrata CBS 138	XP_445076.1	2 E-50	460/545
Eukaryota	Fungi	Neurospora crassa	CAB41986.1	2 E-50	525/545
Eukaryota	Fungi	Saccharomyces cerevisiae AWRI1631	EDZ73312.1	2 E-50	461/545
Eukaryota	Fungi	Saccharomyces cerevisiae	NP_010198.1	2 E-50	461/545
Eukaryota	Fungi	Phaeosphaeria nodorum SN15	XP_001804157.1	3 E-50	531/545
Eukaryota	Fungi	Cryptococcus neoformans var. neoformans	XP_774102.1	3 E-50	530/545
Eukaryota	Fungi	Cryptococcus neoformans var. neoformans	XP_571799.1	3 E-50	530/545
Eukaryota	Fungi	Magnaporthe grisea 70-15	XP_361666.2	3 E-50	543/545
Eukaryota	Fungi	Pichia pastoris GS115	XP_002490420.1	4 E-50	493/545
Eukaryota	Fungi	Neurospora crassa OR74A	XP_961885.1	5 E-50	525/545
Eukaryota	Viridiplantae	Ricinus communis	XP_002517991.1	5 E-50	548/545
Eukaryota	Viridiplantae	Ricinus communis	XP_002524685.1	6 E-50	525/545
Eukaryota	Fungi	Saccharomyces cerevisiae JAY291	EEU07031.1	6 E-50	463/545
Eukaryota	Fungi	Saccharomyces cerevisiae	NP_013865.1	6 E-50	463/545
Eukaryota	Viridiplantae	Physcomitrella patens subsp. patens	XP_001757660.1	7 E-50	441/545
Eukaryota	Viridiplantae	Zea mays;	ACN29078.1	1 E-49	438/545
Eukaryota	Fungi	Penicillium marneffei ATCC 18224	XP_002146630.1	1 E-49	523/545
Eukaryota	Fungi	Schizosaccharomyces pombe	NP_595261.1	2 E-49	457/545
Eukaryota	Viridiplantae	Solanum tuberosum	CAB52796.1	3 E-49	436/545
Eukaryota	Fungi	Aspergillus clavatus NRRL 1	XP_001272658.1	4 E-49	525/545
Eukaryota	Fungi	Ajellomyces dermatitidis ER-3	EEQ83249.1	6 E-49	523/545
Eukaryota	Fungi	Ashbya gossypii ATCC 10895	NP_984358.1	9 E-49	462/545
Eukaryota	Viridiplantae	Arabidopsis thaliana	AAF82202.1	1 E-48	452/545
Eukaryota	Fungi	Pichia guilliermondii ATCC 6260	EDK40505.2	3 E-48	475/545
Eukaryota	Viridiplantae	Arabidopsis thaliana	NP_179673.1	3 E-48	535/545
Eukaryota	Fungi	Pichia guilliermondii ATCC 6260	XP_001483874.1	4 E-48	475/545
Eukaryota	Fungi	Ashbya gossypii ATCC 10895	NP_985994.1	4 E-48	462/545
Eukaryota	Viridiplantae	Sorghum bicolor;	XP_002456580.1	8 E-48	438/545
Eukaryota	Viridiplantae	Vitis vinifera	CAN59740.1	8 E-48	468/545
Eukaryota	Fungi	Talaromyces stipitatus ATCC 10500	XP_002478941.1	1 E-47	523/545
Eukaryota	Fungi	Aspergillus flavus NRRL3357	XP_002380323.1	1 E-47	448/545
Eukaryota	Fungi	Verticillium albo-atrum VaMs.102	EEY21711.1	2 E-47	458/545
Eukaryota	Fungi	Lachancea thermotolerans CBS 6340	XP_002556537.1	2 E-47	448/545
Eukaryota	Fungi	Coccidioides posadasii C735 delta	EER24176.1	2 E-47	531/545
Eukaryota	Fungi	Saccharomyces cerevisiae	CAA43787.1	2 E-47	482/545
Eukaryota	Viridiplantae	Chlamydomonas reinhardtii	XP_001698901.1	3 E-47	453/545
Eukaryota	Viridiplantae	Zea mays;	ACN30943.1	3 E-47	477/545
Eukaryota	Fungi	Neosartorya fischeri NRRL 181	XP_001260169.1	4 E-47	522/545
Eukaryota	Fungi	Coprinopsis cinerea okayama7#130	XP_001835053.1	4 E-47	530/545
Eukaryota	Fungi	Aspergillus fumigatus Af293	XP_749675.1	4 E-47	522/545
Eukaryota	Viridiplantae	Arabidopsis thaliana	NP_567801.1	4 E-47	519/545
Eukaryota	Fungi	Candida glabrata CBS 138	XP_447286.1	5 E-47	464/545
Eukaryota	Fungi	Schizosaccharomyces pombe	NP_594196.1	8 E-47	459/545
Eukaryota	Fungi	Schizosaccharomyces japonicus yFS275	XP_002175270.1	8 E-47	463/545
Eukaryota	Fungi	Aspergillus oryzae RIB40	XP_001819156.1	9 E-47	526/545
Eukaryota	Fungi	Aspergillus flavus NRRL3357	XP_002382154.1	9 E-47	526/545
Eukaryota	Fungi	Aspergillus nidulans FGSC A4	XP_680769.1	1 E-46	523/545
Eukaryota	Fungi	Kluyveromyces lactis NRRL Y-1140	XP_452480.1	2 E-46	457/545

Eukaryota	Fungi	Aspergillus fumigatus	CAE47920.1	2 E-46	456/545
Eukaryota	Fungi	Aspergillus nidulans FGSC A4	tpeCBF79508.1	2 E-46	523/545
Eukaryota	Fungi	Aspergillus niger CBS 513.88	XP_001394893.1	5 E-46	524/545
Eukaryota	Fungi	Cryptococcus neoformans var. neoformans	XP_567114.1	7 E-46	455/545
Eukaryota	Fungi	Uncinocarpus reesii 1704	XP_002541212.1	7 E-46	481/545
Eukaryota	Fungi	Kluyveromyces lactis NRRL Y-1140	XP_454942.1	9 E-46	447/545
Eukaryota	Fungi	Zygosaccharomyces rouxii CBS 732	XP_002496288.1	2 E-45	461/545
Eukaryota	Viridiplantae	Oryza sativa Indica Group	EAZ04357.1	2 E-45	448/545
Eukaryota	Viridiplantae	Populus trichocarpa	XP_002316663.1	2 E-45	525/545
Eukaryota	Viridiplantae	Oryza sativa Japonica Group	NP_001060003.1	2 E-45	448/545
Eukaryota	Fungi	Microsporum canis CBS 113480	EEQ30738.1	3 E-45	531/545
Eukaryota	Fungi	Lachancea thermotolerans CBS 6340	XP_002554507.1	3 E-45	457/545
Eukaryota	Fungi	Aspergillus terreus NIH2624	XP_001215912.1	3 E-45	531/545
Eukaryota	Fungi	Aspergillus oryzae RIB40	XP_001818887.1	3 E-45	467/545
Eukaryota	Fungi	Laccaria bicolor S238N-H82	XP_001874951.1	3 E-45	530/545
Eukaryota	Fungi	Ajellomyces dermatitidis SLH14081	XP_002620750.1	3 E-45	516/545
Eukaryota	Viridiplantae	Populus trichocarpa	XP_002305652.1	4 E-45	526/545
Eukaryota	Fungi	Botryotinia fuckeliana B05.10	XP_001552240.1	6 E-45	531/545
Eukaryota	Fungi	Botryotinia fuckeliana	CAJ15142.1	8 E-45	531/545
Eukaryota	Fungi	Coprinopsis cinerea okayama7#130	XP_001833080.1	1 E-44	548/545
Eukaryota	Fungi	Chaetomium globosum CBS 148.51	XP_001223002.1	1 E-44	529/545
Eukaryota	Fungi	Paracoccidioides brasiliensis Pb03;	EEH22566.1	1 E-44	528/545
Eukaryota	Fungi	Penicillium chrysogenum Wisconsin 54-1255	XP_002564927.1	7 E-44	524/545
Eukaryota	Fungi	Zygosaccharomyces rouxii CBS 732	XP_002498045.1	2 E-43	544/545
Eukaryota	Fungi	Uncinocarpus reesii 1704	XP_002543820.1	8 E-43	439/545
Eukaryota	stramenopiles	Phaeodactylum tricornutum CCAP 1055/1	XP_002184939.1	8 E-43	474/545
Eukaryota	Fungi	Ajellomyces capsulatus NAm1	XP_001543922.1	2 E-42	521/545
Eukaryota	Fungi	Ajellomyces capsulatus G186AR	EEH06739.1	7 E-42	519/545
Eukaryota	Alveolata	Toxoplasma gondii RH	CAJ20615.1	4 E-41	507/545
Eukaryota	Alveolata	Toxoplasma gondii GT1	EEE21222.1	4 E-41	507/545
Eukaryota	Fungi	Ajellomyces capsulatus H143	EER38400.1	2 E-40	501/545
Eukaryota	Alveolata	Toxoplasma gondii ME49	XP_002368319.1	3 E-40	499/545
Eukaryota	Alveolata	Toxoplasma gondii GT1	EEE22709.1	3 E-40	499/545
Eukaryota	Alveolata	Toxoplasma gondii	ABB17192.1	3 E-40	499/545
Eukaryota	Fungi	Sclerotinia sclerotiorum 1980 UF-70	XP_001590642.1	4 E-40	479/545
Eukaryota	Alveolata	Toxoplasma gondii ME49	XP_002369675.1	4 E-40	507/545

AFUA_1G14530

Eukaryota	Fungi	Aspergillus fumigatus Af293	XP_752822.1	0.0	577/577
Eukaryota	Fungi	Neosartorya fischeri NRRL 181	XP_001264305.1	0.0	577/577
Eukaryota	Fungi	Aspergillus fumigatus A1163	EDP56688.1	0.0	537/577
Eukaryota	Fungi	Aspergillus oryzae RIB40	XP_001819654.1	0.0	564/577
Eukaryota	Fungi	Aspergillus clavatus NRRL 1	XP_001268809.1	0.0	589/577
Eukaryota	Fungi	Aspergillus terreus NIH2624	XP_001210740.1	0.0	543/577
Eukaryota	Fungi	Penicillium chrysogenum Wisconsin 54-1255	XP_002561722.1	0.0	579/577
Eukaryota	Fungi	Aspergillus nidulans FGSC A4	XP_658387.1	0.0	572/577
Eukaryota	Fungi	Neosartorya fischeri NRRL 181	XP_001262921.1	0.0	571/577
Eukaryota	Fungi	Aspergillus flavus NRRL3357	XP_002374854.1	0.0	482/577
Eukaryota	Fungi	Aspergillus fumigatus Af293	XP_754101.1	0.0	571/577
Eukaryota	Fungi	Ajellomyces dermatitidis ER-3	EEQ89732.1	0.0	578/577
Eukaryota	Fungi	Ajellomyces dermatitidis SLH14081	XP_002622082.1	0.0	578/577
Eukaryota	Fungi	Coccidioides posadasii C735 delta	EER26827.1	0.0	572/577
Eukaryota	Fungi	Microsporum canis CBS 113480	EEQ27217.1	0.0	555/577
Eukaryota	Fungi	Paracoccidioides brasiliensis Pb03;	EEH15929.1	0.0	580/577
Eukaryota	Fungi	Uncinocarpus reesii 1704	XP_002582258.1	0.0	574/577
Eukaryota	Fungi	Aspergillus niger CBS 513.88	XP_001389657.1	0.0	577/577
Eukaryota	Fungi	Aspergillus nidulans FGSC A4	tpeCBF88792.1	0.0	532/577

Eukaryota	Fungi	Ajellomyces capsulatus H143	EER38076.1	0.0	533/577
Eukaryota	Fungi	Coccidioides immitis RS;	XP_001243714.1	0.0	523/577
Eukaryota	Fungi	Ajellomyces capsulatus G186AR	EEH09735.1	0.0	526/577
Eukaryota	Fungi	Penicillium marneffei ATCC 18224	XP_002145938.1	1 E-178	564/577
Eukaryota	Fungi	Paracoccidioides brasiliensis Pb18;	EEH50619.1	1 E-176	527/577
Eukaryota	Fungi	Paracoccidioides brasiliensis Pb01;	EEH34306.1	1 E-174	496/577
Eukaryota	Fungi	Ajellomyces capsulatus NAm1	XP_001542132.1	1 E-172	480/577
Eukaryota	Fungi	Talaromyces stipitatus ATCC 10500	XP_002478209.1	1 E-172	570/577
Eukaryota	Fungi	Pyrenophora tritici-repentis Pt-1C-BFP	XP_001940983.1	1 E-146	547/577
Eukaryota	Fungi	Botryotinia fuckeliana B05.10	XP_001546066.1	2 E-97	534/577
Eukaryota	Fungi	Penicillium marneffei ATCC 18224	XP_002148056.1	3 E-93	532/577
Eukaryota	Fungi	Neosartorya fischeri NRRL 181	XP_001267605.1	3 E-90	525/577
Eukaryota	Fungi	Aspergillus niger CBS 513.88	XP_001399172.1	3 E-87	533/577
Eukaryota	Fungi	Penicillium marneffei ATCC 18224	XP_002149809.1	5 E-87	538/577
Eukaryota	Fungi	Aspergillus oryzae RIB40	XP_001820564.1	3 E-85	538/577
Eukaryota	Fungi	Aspergillus oryzae RIB40	XP_001822891.1	3 E-85	542/577
Eukaryota	Fungi	Penicillium marneffei ATCC 18224	XP_002143333.1	3 E-85	533/577
Eukaryota	Fungi	Aspergillus terreus NIH2624	XP_001215343.1	3 E-85	483/577
Eukaryota	Fungi	Aspergillus fumigatus Af293	XP_746419.1	4 E-85	474/577
Eukaryota	Fungi	Talaromyces stipitatus ATCC 10500	XP_002479642.1	8 E-85	524/577
Eukaryota	Fungi	Coccidioides posadasii C735 delta	EER25918.1	3 E-84	528/577
Eukaryota	Fungi	Aspergillus fumigatus A1163	EDP47095.1	1 E-83	474/577
Eukaryota	Fungi	Aspergillus terreus	AAK31195.1	2 E-83	532/577
Eukaryota	Fungi	Emericella nidulans	AAK31199.1	2 E-83	478/577
Eukaryota	Fungi	Aspergillus terreus NIH2624	XP_001210024.1	2 E-83	465/577
Eukaryota	Fungi	Aspergillus nidulans FGSC A4	XP_660433.1	3 E-83	478/577
Eukaryota	Fungi	Phaeosphaeria nodorum SN15	XP_001792268.1	3 E-83	477/577
Eukaryota	Fungi	Penicillium chrysogenum Wisconsin 54-1255	XP_002557312.1	7 E-83	491/577
Eukaryota	Fungi	Aspergillus nidulans FGSC A4	XP_660266.1	1 E-82	528/577
Eukaryota	Fungi	Coccidioides immitis RS;	XP_001240408.1	2 E-82	470/577
Eukaryota	Fungi	Aspergillus nidulans FGSC A4	tpeCBF70903.1	1 E-81	501/577
Eukaryota	Fungi	Aspergillus nidulans FGSC A4	XP_664123.1	1 E-81	490/577
Eukaryota	Fungi	Pyrenophora tritici-repentis Pt-1C-BFP	XP_001934866.1	3 E-81	464/577
Eukaryota	Fungi	Aspergillus niger CBS 513.88	XP_001393013.1	4 E-81	536/577
Eukaryota	Fungi	Talaromyces stipitatus ATCC 10500	XP_002486483.1	6 E-81	545/577
Eukaryota	Fungi	Neosartorya fischeri NRRL 181	XP_001266411.1	9 E-81	543/577
Eukaryota	Fungi	Botryotinia fuckeliana B05.10	XP_001560291.1	2 E-80	516/577
Eukaryota	Fungi	Penicillium chrysogenum Wisconsin 54-1255	XP_002567163.1	4 E-80	474/577
Eukaryota	Fungi	Aspergillus niger CBS 513.88	XP_001396568.1	6 E-80	540/577
Eukaryota	Fungi	Aspergillus terreus NIH2624	XP_001214852.1	6 E-80	492/577
Eukaryota	Fungi	Aspergillus niger CBS 513.88	XP_001394850.1	1 E-79	475/577
Eukaryota	Fungi	Sclerotinia sclerotiorum 1980 UF-70	XP_001585430.1	1 E-79	489/577
Eukaryota	Fungi	Aspergillus clavatus NRRL 1	XP_001270046.1	2 E-79	490/577
Eukaryota	Fungi	Ajellomyces dermatitidis SLH14081	XP_002626547.1	3 E-79	465/577
Eukaryota	Fungi	Ustilago maydis 521	XP_756158.1	3 E-79	480/577
Eukaryota	Fungi	Aspergillus flavus NRRL3357	XP_002372468.1	4 E-79	538/577
Eukaryota	Fungi	Ajellomyces dermatitidis ER-3	EEQ84490.1	5 E-79	465/577
Eukaryota	Fungi	Phaeosphaeria nodorum SN15	XP_001801211.1	5 E-79	524/577
Eukaryota	Fungi	Nectria haematococca mpVI 77-13-4	EEU48206.1	5 E-79	489/577
Eukaryota	Fungi	Aspergillus oryzae RIB40	XP_001822929.1	6 E-79	507/577
Eukaryota	Fungi	Uncinocarpus reesii 1704	XP_002582938.1	8 E-79	482/577
Eukaryota	Fungi	Aspergillus oryzae RIB40	XP_001817397.1	1 E-78	465/577
Eukaryota	Fungi	Pyrenophora tritici-repentis Pt-1C-BFP	XP_001934102.1	1 E-78	487/577
Eukaryota	Fungi	Coccidioides posadasii C735 delta	EER24780.1	2 E-78	481/577
Eukaryota	Fungi	Ajellomyces dermatitidis ER-3	EEQ85902.1	2 E-78	490/577
Eukaryota	Fungi	Penicillium marneffei ATCC 18224	XP_002144885.1	2 E-78	491/577
Eukaryota	Fungi	Aspergillus flavus NRRL3357	XP_002378240.1	3 E-78	507/577
Eukaryota	Fungi	Ajellomyces dermatitidis ER-3	EEQ91602.1	3 E-78	487/577

Eukaryota	Fungi	Aspergillus clavatus NRRL 1	XP_001270530.1	3 E-78	475/577
Eukaryota	Fungi	Aspergillus fumigatus A1163	EDP47483.1	4 E-78	502/577
Eukaryota	Fungi	Aspergillus fumigatus Af293	XP_747576.1	9 E-78	502/577
Eukaryota	Fungi	Talaromyces stipitatus ATCC 10500	XP_002485567.1	9 E-78	490/577
Eukaryota	Fungi	Neosartorya fischeri NRRL 181	XP_001266529.1	3 E-77	537/577
Eukaryota	Fungi	Aspergillus flavus NRRL3357	XP_002373834.1	4 E-77	482/577
Eukaryota	Fungi	Pichia guilliermondii ATCC 6260	XP_001482617.1	5 E-77	489/577
Eukaryota	Fungi	Gibberella zeae PH-1	XP_389513.1	6 E-77	483/577
Eukaryota	Fungi	Ajellomyces capsulatus H143	EER40580.1	7 E-77	490/577
Eukaryota	Fungi	Pichia guilliermondii ATCC 6260	EDK41539.2	8 E-77	489/577
Eukaryota	Fungi	Schizosaccharomyces pombe	NP_588099.1	8 E-77	495/577
Eukaryota	Fungi	Paracoccidioides brasiliensis Pb01;	EEH41340.1	1 E-76	498/577
Eukaryota	Fungi	Ajellomyces capsulatus G186AR	EEH02605.1	1 E-76	490/577
Eukaryota	Fungi	Talaromyces stipitatus ATCC 10500	XP_002481430.1	1 E-76	541/577
Eukaryota	Fungi	Neosartorya fischeri NRRL 181	XP_001257710.1	2 E-76	502/577
Eukaryota	Fungi	Ustilago maydis 521	XP_761507.1	3 E-76	519/577
Eukaryota	Fungi	Gibberella zeae PH-1	XP_390494.1	7 E-76	465/577
Eukaryota	Fungi	Aspergillus nidulans FGSC A4	XP_660483.1	8 E-76	526/577
Eukaryota	Fungi	Aspergillus fumigatus Af293	XP_748292.1	1 E-75	537/577
Eukaryota	Fungi	Nectria haematococca mpVI 77-13-4	EEU44584.1	1 E-75	548/577
Eukaryota	Fungi	Ajellomyces capsulatus NAm1	XP_001539656.1	2 E-75	486/577
Eukaryota	Fungi	Aspergillus terreus NIH2624	XP_001210984.1	2 E-75	540/577
Eukaryota	Fungi	Pichia stipitis CBS 6054	XP_001384049.2	3 E-75	538/577
Eukaryota	Fungi	Coprinopsis cinerea okayama7#130	XP_001837854.1	4 E-75	567/577
Eukaryota	Fungi	Penicillium chrysogenum Wisconsin 54-1255	XP_002561084.1	7 E-75	524/577
Eukaryota	Fungi	Paracoccidioides brasiliensis Pb18;	EEH50121.1	1 E-74	498/577
Eukaryota	Fungi	Pyrenophora tritici-repentis Pt-1C-BFP	XP_001938035.1	2 E-74	478/577
Eukaryota	Fungi	Emericella unguis	AAK29062.1	3 E-74	519/577
Eukaryota	Fungi	Aspergillus fumigatus	AAK31196.1	4 E-74	533/577
Eukaryota	Fungi	Aspergillus flavus NRRL3357	XP_002373936.1	4 E-74	462/577
Eukaryota	Fungi	Aspergillus oryzae RIB40	XP_001820467.1	9 E-74	471/577
Eukaryota	Fungi	Microsporum canis CBS 113480	EEQ30996.1	1 E-73	517/577
Eukaryota	Fungi	Neurospora crassa OR74A	XP_961990.1	1 E-73	536/577
Eukaryota	Fungi	Coccidioides immitis RS;	XP_001243435.1	1 E-73	485/577
Eukaryota	Fungi	Neosartorya fischeri NRRL 181	XP_001262066.1	1 E-73	466/577
Eukaryota	Fungi	Nectria haematococca mpVI 77-13-4	EEU38629.1	2 E-73	539/577
Eukaryota	Fungi	Aspergillus oryzae RIB40	XP_001817728.1	3 E-73	478/577
Eukaryota	Fungi	Candida dubliniensis CD36	XP_002421039.1	5 E-73	560/577
Eukaryota	Fungi	Coccidioides immitis RS;	XP_001248856.1	6 E-73	489/577
Eukaryota	Fungi	Nectria haematococca mpVI 77-13-4	EEU34779.1	7 E-73	521/577
Eukaryota	Fungi	Uncinocarpus reesii 1704	XP_002583101.1	1 E-72	478/577
Eukaryota	Fungi	Botryotinia fuckeliana B05.10	XP_001546878.1	1 E-72	534/577
Eukaryota	Fungi	Gibberella zeae PH-1	XP_387788.1	2 E-72	552/577
Eukaryota	Fungi	Cryptococcus neoformans var. neoformans	XP_570813.1	2 E-72	510/577
Eukaryota	Fungi	Nectria haematococca mpVI 77-13-4	EEU35864.1	3 E-72	523/577
Eukaryota	Fungi	Aspergillus nidulans FGSC A4	tpeCBF85015.1	8 E-72	532/577
Eukaryota	Fungi	Ajellomyces dermatitidis SLH14081	XP_002624523.1	1 E-71	473/577
Eukaryota	Fungi	Ajellomyces capsulatus NAm1	XP_001539260.1	3 E-71	500/577
Eukaryota	Fungi	Gibberella zeae PH-1	XP_388254.1	3 E-71	545/577
Eukaryota	Fungi	Nectria haematococca mpVI 77-13-4	EEU34159.1	3 E-71	541/577
Eukaryota	Fungi	Aspergillus niger CBS 513.88	XP_001393811.1	5 E-71	523/577
Eukaryota	Fungi	Ajellomyces capsulatus NAm1	XP_001538851.1	5 E-71	465/577
Eukaryota	Fungi	Paracoccidioides brasiliensis Pb03;	EEH20415.1	5 E-71	464/577
Eukaryota	Fungi	Aspergillus niger CBS 513.88	XP_001390467.1	5 E-71	539/577
Eukaryota	Fungi	Microsporum canis CBS 113480	EEQ28671.1	8 E-71	504/577
Eukaryota	Fungi	Aspergillus fumigatus Af293	XP_748218.1	1 E-70	537/577
Eukaryota	Fungi	Paracoccidioides brasiliensis Pb18;	EEH44826.1	1 E-70	464/577
Eukaryota	Fungi	Penicillium chrysogenum Wisconsin 54-1255	XP_002562914.1	2 E-70	512/577

Eukaryota	Fungi	Sclerotinia sclerotiorum 1980 UF-70	XP_001584936.1	2 E-70	543/577
Eukaryota	Fungi	Pyrenophora tritici-repentis Pt-1C-BFP	XP_001941172.1	2 E-70	521/577
Eukaryota	Fungi	Sclerotinia sclerotiorum 1980 UF-70	XP_001596417.1	3 E-70	522/577
Eukaryota	Fungi	Aspergillus fumigatus A1163	EDP50970.1	4 E-70	537/577
Eukaryota	Fungi	Talaromyces stipitatus ATCC 10500	XP_002488660.1	7 E-70	528/577
Eukaryota	Fungi	Aspergillus flavus NRRL3357	XP_002375187.1	8 E-70	494/577
Eukaryota	Fungi	Gibberella zeae PH-1	XP_383522.1	8 E-70	480/577
Eukaryota	Fungi	Verticillium albo-atrum VaMs.102	EEY18374.1	8 E-70	503/577
Eukaryota	Fungi	Pyrenophora tritici-repentis Pt-1C-BFP	XP_001937124.1	9 E-70	480/577
Eukaryota	Fungi	Aspergillus niger CBS 513.88	XP_001394300.1	1 E-69	487/577
Eukaryota	Fungi	Aspergillus ustus	AAK31194.1	2 E-69	516/577
Eukaryota	Fungi	Aspergillus niger CBS 513.88	XP_001393419.1	2 E-69	470/577
Eukaryota	Fungi	Sclerotinia sclerotiorum 1980 UF-70	XP_001596081.1	2 E-69	534/577
Eukaryota	Fungi	Chaetomium globosum CBS 148.51	XP_001230011.1	4 E-69	482/577
Eukaryota	Fungi	Candida albicans SC5314	XP_719100.1	4 E-69	560/577
Eukaryota	Fungi	Aspergillus niger CBS 513.88	XP_001398050.1	5 E-69	488/577
Eukaryota	Fungi	Candida albicans SC5314	XP_719217.1	6 E-69	560/577
Eukaryota	Fungi	Laccaria bicolor S238N-H82	XP_001885198.1	9 E-69	535/577
Eukaryota	Fungi	Aspergillus clavatus NRRL 1	XP_001272500.1	1 E-68	482/577
Eukaryota	Fungi	Nectria haematococca mpVI 77-13-4	EEU41419.1	1 E-68	520/577
Eukaryota	Fungi	Aspergillus oryzae RIB40	XP_001819358.1	1 E-68	503/577
Eukaryota	Fungi	Phaeosphaeria nodorum SN15	XP_001799608.1	2 E-68	494/577
Eukaryota	Fungi	Nectria haematococca mpVI 77-13-4	EEU38485.1	2 E-68	546/577
Eukaryota	Fungi	Neosartorya fischeri NRRL 181	XP_001257921.1	2 E-68	549/577
Eukaryota	Fungi	Aspergillus ustus	AAK31193.1	2 E-68	518/577
Eukaryota	Fungi	Aspergillus clavatus NRRL 1	XP_001276630.1	3 E-68	469/577
Eukaryota	Fungi	Coccidioides posadasii C735 delta	EER23269.1	5 E-68	496/577
Eukaryota	Fungi	Candida albicans WO-1	EEQ46737.1	6 E-68	560/577
Eukaryota	Fungi	Penicillium chrysogenum Wisconsin 54-1255	XP_002557813.1	7 E-68	518/577
Eukaryota	Fungi	Coccidioides posadasii C735 delta	EER29023.1	9 E-68	522/577
Eukaryota	Fungi	Coccidioides immitis RS;	XP_001248096.1	2 E-67	496/577
Eukaryota	Fungi	Aspergillus fumigatus A1163	EDP49372.1	2 E-67	558/577
Eukaryota	Fungi	Aspergillus fumigatus Af293	XP_750691.1	2 E-67	558/577
Eukaryota	Fungi	Phaeosphaeria nodorum SN15	XP_001805796.1	3 E-67	478/577
Eukaryota	Fungi	Penicillium chrysogenum Wisconsin 54-1255	XP_002563603.1	3 E-67	463/577
Eukaryota	Fungi	Verticillium albo-atrum VaMs.102	EEY16874.1	4 E-67	469/577
Eukaryota	Fungi	Candida tropicalis MYA-3404	XP_002549642.1	7 E-67	477/577
Eukaryota	Fungi	Paracoccidioides brasiliensis Pb18;	EEH48675.1	8 E-67	484/577
Eukaryota	Fungi	Paracoccidioides brasiliensis Pb03;	EEH22153.1	9 E-67	484/577
Eukaryota	Fungi	Monascus purpureus	BAB84516.1	1 E-66	511/577
Eukaryota	Fungi	Gibberella zeae PH-1	XP_384139.1	1 E-66	578/577
Eukaryota	Fungi	Aspergillus flavus NRRL3357	XP_002381831.1	1 E-66	519/577
Eukaryota	Fungi	Aspergillus oryzae	Q12559.2	1 E-66	519/577
Eukaryota	Fungi	Podospora anserina DSM 980	XP_001907273.1	2 E-66	528/577
Eukaryota	Fungi	Coccidioides posadasii C735 delta	EER23853.1	3 E-66	545/577
Eukaryota	Fungi	Aspergillus niger CBS 513.88	XP_001400696.1	3 E-66	545/577
Eukaryota	Fungi	Aspergillus oryzae	BAA01373.1	9 E-66	519/577
Eukaryota	Fungi	Penicillium marneffei ATCC 18224	XP_002149724.1	1 E-65	530/577
Eukaryota	Fungi	Aspergillus nidulans FGSC A4	XP_661561.1	1 E-65	550/577
Eukaryota	Fungi	Aspergillus terreus NIH2624	XP_001212563.1	3 E-65	518/577
Eukaryota	Fungi	Aspergillus clavatus NRRL 1	XP_001267998.1	5 E-65	547/577
Eukaryota	Fungi	Phaeosphaeria nodorum SN15	XP_001803341.1	6 E-65	494/577
Eukaryota	Fungi	Aspergillus terreus NIH2624	XP_001215034.1	9 E-65	508/577
Eukaryota	Fungi	Paracoccidioides brasiliensis Pb03;	EEH22390.1	9 E-65	504/577
Eukaryota	Fungi	Penicillium marneffei ATCC 18224	XP_002145745.1	1 E-64	510/577
Eukaryota	Fungi	Malassezia globosa CBS 7966	XP_001729102.1	3 E-64	479/577
Eukaryota	Fungi	Paracoccidioides brasiliensis Pb18;	EEH48923.1	4 E-64	510/577
Eukaryota	Fungi	Uncinocarpus reesii 1704	XP_002583854.1	5 E-64	503/577

Eukaryota	Fungi	Aspergillus terreus NIH2624	XP_001215585.1	6 E-64	495/577
Eukaryota	Fungi	Ajellomyces capsulatus G186AR	EEH05571.1	6 E-64	494/577
Eukaryota	Fungi	Talaromyces stipitatus ATCC 10500	XP_002484306.1	8 E-64	515/577
Eukaryota	Fungi	Penicillium marneffei ATCC 18224	XP_002149962.1	8 E-64	495/577
Eukaryota	Fungi	Aspergillus clavatus NRRL 1	XP_001274802.1	1 E-63	524/577
Eukaryota	Fungi	Pichia guilliermondii ATCC 6260	XP_001487446.1	3 E-63	537/577
Eukaryota	Fungi	Aspergillus oryzae RIB40	XP_001826580.1	4 E-63	528/577
Eukaryota	Fungi	Coprinopsis cinerea okayama7#130	XP_001831504.1	4 E-63	501/577
Eukaryota	Fungi	Coccidioides immitis RS;	XP_001248603.1	5 E-63	498/577
Eukaryota	Fungi	Coccidioides posadasii C735 delta	EER27006.1	7 E-63	494/577
Eukaryota	Fungi	Ajellomyces dermatitidis SLH14081	XP_002620214.1	8 E-63	477/577
Eukaryota	Fungi	Paracoccidioides brasiliensis Pb01;	EEH34112.1	1 E-62	479/577
Eukaryota	Fungi	Penicillium chrysogenum Wisconsin 54-1255	XP_002559064.1	1 E-62	530/577
Eukaryota	Fungi	Ajellomyces capsulatus G186AR	EEH05772.1	2 E-62	492/577
Eukaryota	Fungi	Aspergillus nidulans FGSC A4	XP_682046.1	2 E-62	519/577
Eukaryota	Fungi	Coccidioides immitis RS;	XP_001239154.1	3 E-62	463/577
Eukaryota	Fungi	Coccidioides posadasii C735 delta	EER28750.1	3 E-62	498/577
Eukaryota	Fungi	Nectria haematococca mpVI 77-13-4	EEU35325.1	4 E-62	536/577
Eukaryota	Fungi	Neosartorya fischeri NRRL 181	XP_001262387.1	1 E-61	519/577
Eukaryota	Fungi	Aspergillus oryzae RIB40	XP_001822270.1	1 E-61	504/577
Eukaryota	Fungi	Uncinocarpus reesii 1704	XP_002545119.1	1 E-61	494/577
Eukaryota	Fungi	Botryotinia fuckeliana B05.10	XP_001554888.1	1 E-61	537/577
Eukaryota	Fungi	Aspergillus flavus NRRL3357	XP_002382352.1	1 E-61	504/577
Eukaryota	Fungi	Coccidioides posadasii C735 delta	EER29963.1	1 E-61	463/577
Eukaryota	Fungi	Nectria haematococca mpVI 77-13-4	EEU40007.1	2 E-61	518/577
Eukaryota	Fungi	Schizosaccharomyces pombe	NP_001018764.1	2 E-61	539/577
Eukaryota	Fungi	Aspergillus nidulans FGSC A4	XP_664641.1	2 E-61	536/577
Eukaryota	Fungi	Nectria haematococca mpVI 77-13-4	EEU35914.1	2 E-61	541/577
Eukaryota	Fungi	Talaromyces stipitatus ATCC 10500	XP_002484401.1	3 E-61	486/577
Eukaryota	Fungi	Nectria haematococca mpVI 77-13-4	EEU36819.1	4 E-61	537/577
Eukaryota	Fungi	Paracoccidioides brasiliensis Pb18;	EEH47555.1	4 E-61	484/577
Eukaryota	Fungi	Microsporum canis CBS 113480	EEQ34981.1	2 E-60	546/577
Eukaryota	Fungi	Emericella rugulosa	AAK29061.1	3 E-60	519/577
Eukaryota	Fungi	Penicillium marneffei ATCC 18224	XP_002149865.1	4 E-60	543/577
Eukaryota	Fungi	Lodderomyces elongisporus NRRL YB-4239	XP_001523741.1	6 E-59	471/577
Eukaryota	Fungi	Botryotinia fuckeliana B05.10	XP_001555270.1	1 E-58	536/577
Eukaryota	Fungi	Ajellomyces dermatitidis ER-3	EEQ85347.1	1 E-58	522/577
Eukaryota	Fungi	Clavispora lusitaniae ATCC 42720	XP_002618455.1	1 E-58	476/577
Eukaryota	Fungi	Aspergillus nidulans FGSC A4	XP_659116.1	2 E-58	476/577
Eukaryota	Fungi	Paracoccidioides brasiliensis Pb01;	EEH39235.1	2 E-58	499/577
Eukaryota	Fungi	Ajellomyces dermatitidis SLH14081	XP_002628820.1	2 E-58	522/577

AFUA_1G14540

Eukaryota	Fungi	Aspergillus fumigatus Af293	XP_752823.1	1 E-164	288/288
Eukaryota	Fungi	Neosartorya fischeri NRRL 181	XP_001264304.1	1 E-156	281/288
Eukaryota	Fungi	Aspergillus clavatus NRRL 1	XP_001268808.1	1 E-145	281/288
Eukaryota	Fungi	Aspergillus flavus NRRL3357	XP_002374853.1	1 E-134	292/288
Eukaryota	Fungi	Penicillium chrysogenum Wisconsin 54-1255	XP_002561721.1	1 E-129	281/288
Eukaryota	Fungi	Coccidioides immitis RS;	XP_001243713.1	1 E-126	280/288
Eukaryota	Fungi	Coccidioides posadasii C735 delta	EER26828.1	1 E-126	280/288
Eukaryota	Fungi	Uncinocarpus reesii 1704	XP_002582257.1	1 E-126	280/288
Eukaryota	Fungi	Aspergillus nidulans FGSC A4	XP_658388.1	1 E-123	281/288
Eukaryota	Fungi	Microsporum canis CBS 113480	EEQ27216.1	1 E-123	281/288
Eukaryota	Fungi	Aspergillus niger CBS 513.88	XP_001389658.1	1 E-116	274/288
Eukaryota	Fungi	Talaromyces stipitatus ATCC 10500	XP_002478212.1	1 E-111	277/288
Eukaryota	Fungi	Penicillium marneffei ATCC 18224	XP_002145947.1	1 E-110	277/288
Eukaryota	Fungi	Pyrenophora tritici-repentis Pt-1C-BFP	XP_001940870.1	1 E-103	279/288

Eukaryota	Fungi	Gibberella zeae PH-1	XP_382033.1	3 E-98	273/288
Eukaryota	Fungi	Botryotinia fuckeliana B05.10	XP_001557478.1	6 E-98	272/288
Eukaryota	Fungi	Nectria haematococca mpVI 77-13-4	EEU41860.1	1 E-97	273/288
Eukaryota	Fungi	Neurospora crassa OR74A	XP_961469.1	8 E-97	275/288
Eukaryota	Fungi	Podospora anserina DSM 980	XP_001912019.1	3 E-92	277/288
Eukaryota	Fungi	Magnaporthe grisea 70-15	XP_364373.1	1 E-91	272/288
Eukaryota	Fungi	Phaeosphaeria nodorum SN15	XP_001792826.1	9 E-91	273/288
Eukaryota	Fungi	Chaetomium globosum CBS 148.51	XP_001224970.1	2 E-88	277/288
Eukaryota	Fungi	Paracoccidioides brasiliensis Pb03;	EEH16435.1	5 E-72	267/288
Eukaryota	Fungi	Paracoccidioides brasiliensis Pb01;	EEH35088.1	5 E-72	267/288
Eukaryota	Fungi	Sclerotinia sclerotiorum 1980 UF-70	XP_001586547.1	5 E-72	238/288
Eukaryota	Fungi	Pyrenophora tritici-repentis Pt-1C-BFP	XP_001940615.1	4 E-71	265/288
Eukaryota	Fungi	Ajellomyces dermatitidis SLH14081	XP_002622769.1	2 E-70	264/288
Eukaryota	Fungi	Ajellomyces dermatitidis ER-3	EEQ87200.1	3 E-70	264/288
Eukaryota	Fungi	Uncinocarpus reesii 1704	XP_002545006.1	3 E-70	257/288
Eukaryota	Fungi	Phaeosphaeria nodorum SN15	XP_001800159.1	5 E-70	266/288
Eukaryota	Fungi	Verticillium albo-atrum VaMs.102	EEY17842.1	2 E-66	277/288
Eukaryota	Fungi	Aspergillus nidulans FGSC A4	XP_681672.1	4 E-63	267/288
Eukaryota	Fungi	Yarrowia lipolytica CLIB122	XP_501373.1	7 E-63	259/288
Eukaryota	Fungi	Candida dubliniensis CD36	XP_002417090.1	4 E-58	257/288
Eukaryota	Fungi	Candida albicans WO-1	EEQ42727.1	6 E-58	257/288
Eukaryota	Fungi	Candida albicans SC5314	XP_712226.1	6 E-58	257/288
Eukaryota	Fungi	Candida tropicalis MYA-3404	XP_002550414.1	7 E-58	254/288
Eukaryota	Fungi	Clavispora lusitaniae ATCC 42720	XP_002619547.1	6 E-57	263/288
Eukaryota	Fungi	Pichia guilliermondii ATCC 6260	XP_001486475.1	1 E-56	261/288
Eukaryota	Fungi	Debaryomyces hansenii	CAG85346.2	4 E-55	261/288
Eukaryota	Fungi	Debaryomyces hansenii CBS767	XP_457342.1	5 E-55	261/288
Eukaryota	Fungi	Lodderomyces elongisporus NRRL YB-4239	XP_001526988.1	8 E-55	259/288
Bacteria	Actinobacteria	Thermomonospora curvata DSM 43183	ZP_04034022.1	6 E-54	248/288
Eukaryota	Fungi	Pichia stipitis CBS 6054	XP_001385804.1	2 E-52	259/288
Bacteria	Actinobacteria	Mycobacterium smegmatis str. MC2	YP_884537.1	2 E-52	249/288
Bacteria	Proteobacteria	marine gamma proteobacterium HTCC2143	ZP_01617981.1	5 E-52	255/288
Bacteria	Actinobacteria	Geodermatophilus obscurus DSM 43160	ZP_03888442.1	2 E-51	248/288
Eukaryota	Fungi	Phaeosphaeria nodorum SN15	XP_001792351.1	6 E-51	262/288
Bacteria	Proteobacteria	Desulfatibacillum alkenivorans AK-01	YP_002432520.1	7 E-51	255/288
Bacteria	Actinobacteria	Streptomyces hygroscopicus ATCC 53653	ZP_05519416.1	9 E-51	248/288
Bacteria	Proteobacteria	Congregibacter litoralis KT71	ZP_01101314.1	2 E-50	248/288
Bacteria	Proteobacteria	marine gamma proteobacterium HTCC2148	ZP_05096873.1	8 E-50	256/288
Bacteria	Actinobacteria	Rhodococcus erythropolis SK121	ZP_04385393.1	8 E-50	254/288
Eukaryota	Fungi	Ajellomyces dermatitidis SLH14081	XP_002625057.1	2 E-49	258/288
Bacteria	Actinobacteria	Mycobacterium sp. JLS	YP_001068403.1	3 E-49	249/288
Eukaryota	Fungi	Debaryomyces hansenii CBS767	XP_457430.1	4 E-49	261/288
Bacteria	Actinobacteria	Rhodococcus erythropolis PR4	YP_002768928.1	4 E-49	254/288
Eukaryota	Fungi	Pichia guilliermondii ATCC 6260	EDK37616.2	6 E-49	261/288
Eukaryota	Fungi	Podospora anserina DSM 980	XP_001910841.1	6 E-49	262/288
Eukaryota	Fungi	Aspergillus clavatus NRRL 1	XP_001272204.1	7 E-49	259/288
Bacteria	Proteobacteria	marine gamma proteobacterium HTCC2143	ZP_01615142.1	9 E-49	263/288
Bacteria	Proteobacteria	marine gamma proteobacterium HTCC2080	ZP_01627907.1	9 E-49	253/288
Bacteria	Actinobacteria	Mycobacterium sp. JLS	YP_001070924.1	1 E-48	248/288
Eukaryota	Fungi	Pichia guilliermondii ATCC 6260	XP_001486043.1	1 E-48	261/288
Eukaryota	Fungi	Pichia pastoris GS115	XP_002491141.1	2 E-48	263/288
Eukaryota	Fungi	Penicillium chrysogenum Wisconsin 54-1255	XP_002564409.1	2 E-48	258/288
Bacteria	Proteobacteria	Oceanicola batsensis HTCC2597	ZP_00999734.1	2 E-48	253/288
Bacteria	Actinobacteria	Mycobacterium sp. MCS	YP_639787.1	2 E-48	248/288
Bacteria	Proteobacteria	Novosphingobium aromaticivorans DSM 12444	YP_496280.1	2 E-48	252/288
Eukaryota	Fungi	Neurospora crassa OR74A	XP_960628.1	2 E-48	281/288
Bacteria	Proteobacteria	Bradyrhizobium japonicum USDA 110	NP_767722.1	3 E-48	255/288
Eukaryota	Fungi	Aspergillus oryzae RIB40	XP_001816710.1	3 E-48	259/288

Eukaryota	Choanoflagellida	Monosiga brevicollis MX1	XP_001748577.1	4 E-48	266/288
Bacteria	Actinobacteria	Streptosporangium roseum DSM 43021	ZP_04476173.1	6 E-48	247/288
Eukaryota	Fungi	Aspergillus nidulans FGSC A4	XP_663241.1	6 E-48	261/288
Bacteria	Proteobacteria	Pseudoalteromonas atlantica T6c	YP_663396.1	7 E-48	257/288
Eukaryota	Fungi	Talaromyces stipitatus ATCC 10500	XP_002481690.1	9 E-48	261/288
Bacteria	Proteobacteria	Parvibaculum lavamentivorans DS-1	YP_001412954.1	2 E-47	252/288
Eukaryota	Fungi	Paracoccidioides brasiliensis Pb18;	EEH49927.1	3 E-47	279/288
Eukaryota	Fungi	Neosartorya fischeri NRRL 181	XP_001266693.1	4 E-47	259/288
Bacteria	Proteobacteria	Rhodobacteraceae bacterium KLH11	ZP_05122554.1	4 E-47	255/288
Eukaryota	Fungi	Aspergillus niger CBS 513.88	XP_001389351.1	5 E-47	261/288
Eukaryota	Fungi	Paracoccidioides brasiliensis Pb01;	EEH34944.1	5 E-47	279/288
Eukaryota	Fungi	Coccidioides immitis RS;	XP_001248226.1	5 E-47	258/288
Bacteria	Proteobacteria	gamma proteobacterium NOR5-3	ZP_05126130.1	6 E-47	255/288
Eukaryota	Fungi	Ajellomyces capsulatus G186AR	EEH07124.1	6 E-47	258/288
Bacteria	Proteobacteria	Desulfatibacillum alkenivorans AK-01	YP_002434168.1	7 E-47	257/288
Bacteria	Actinobacteria	Micromonospora sp. ATCC 39149	ZP_04608328.1	7 E-47	255/288
Bacteria	Proteobacteria	marine gamma proteobacterium HTCC2148	ZP_05096872.1	1 E-46	264/288
Eukaryota	Fungi	Penicillium marneffei ATCC 18224	XP_002147538.1	1 E-46	261/288
Eukaryota	Fungi	Aspergillus terreus NIH2624	XP_001212413.1	2 E-46	259/288
Eukaryota	Fungi	Ajellomyces capsulatus NAm1	XP_001544218.1	2 E-46	279/288
Bacteria	Proteobacteria	Erythrobacter sp. NAP1	ZP_01041803.1	3 E-46	252/288
Bacteria	Actinobacteria	Brevibacterium linens BL2	ZP_05912377.1	4 E-46	254/288
Eukaryota	Fungi	Aspergillus fumigatus Af293	XP_751476.1	5 E-46	259/288
Eukaryota	Fungi	Sclerotinia sclerotiorum 1980 UF-70	XP_001595757.1	8 E-46	258/288
Eukaryota	Fungi	Paracoccidioides brasiliensis Pb01;	EEH36535.1	9 E-46	260/288
Eukaryota	Fungi	Ajellomyces capsulatus G186AR	EEH03613.1	1 E-45	279/288
Eukaryota	Fungi	Ajellomyces capsulatus H143	EER37456.1	1 E-45	279/288
Bacteria	Proteobacteria	Sphingopyxis alaskensis RB2256	YP_618116.1	1 E-45	256/288
Bacteria	Proteobacteria	Bradyrhizobium sp. ORS278	YP_001208598.1	2 E-45	253/288
Eukaryota	Fungi	Pyrenophora tritici-repentis Pt-1C-BFP	XP_001933039.1	2 E-45	262/288
Bacteria	Proteobacteria	Burkholderia graminis C4D1M	ZP_02887243.1	2 E-45	248/288
Eukaryota	Fungi	Microsporum canis CBS 113480	EEQ28138.1	2 E-45	258/288
Bacteria	Proteobacteria	Acidovorax delafieldii 2AN	ZP_04764516.1	2 E-45	252/288
Bacteria	Proteobacteria	Roseobacter sp. SK209-2-6	ZP_01753463.1	3 E-45	253/288
Eukaryota	Fungi	Paracoccidioides brasiliensis Pb03;	EEH20737.1	3 E-45	260/288
Eukaryota	Fungi	Postia placenta Mad-698-R	XP_002472834.1	3 E-45	266/288
Bacteria	Proteobacteria	Erythrobacter sp. SD-21	ZP_01863917.1	5 E-45	252/288
Bacteria	Proteobacteria	Pseudoalteromonas atlantica T6c	YP_660985.1	6 E-45	252/288
Bacteria	Proteobacteria	Delftia acidovorans SPH-1	YP_001561297.1	8 E-45	252/288
Bacteria	Proteobacteria	Rhodopseudomonas palustris TIE-1	YP_001994234.1	1 E-44	253/288
Bacteria	Proteobacteria	Rhodopseudomonas palustris CGA009	NP_950120.1	1 E-44	253/288
Bacteria	Proteobacteria	Variovorax paradoxus S110	YP_002946610.1	1 E-44	247/288
Bacteria	Actinobacteria	Salinispora tropica CNB-440	YP_001158443.1	2 E-44	255/288
Bacteria	Proteobacteria	Erythrobacter litoralis HTCC2594	YP_458808.1	2 E-44	252/288
Bacteria	Proteobacteria	Rhodopseudomonas palustris BisA53	YP_779413.1	2 E-44	255/288
Bacteria	Actinobacteria	Nocardioides sp. JS614	YP_923032.1	3 E-44	250/288
Eukaryota	Fungi	Ajellomyces dermatitidis SLH14081	XP_002623324.1	6 E-44	272/288
Bacteria	Proteobacteria	Mesorhizobium opportunistum WSM2075	ZP_05808823.1	1 E-43	256/288
Bacteria	Proteobacteria	Roseovarius sp. HTCC2601	ZP_01441394.1	1 E-43	254/288
Bacteria	Proteobacteria	Rhodopseudomonas palustris BisB5	YP_568021.1	2 E-43	255/288
Bacteria	Actinobacteria	Salinispora arenicola CNS-205	YP_001536446.1	2 E-43	255/288
Bacteria	Proteobacteria	Oceanicaulis alexandrii HTCC2633	ZP_00953027.1	3 E-43	252/288
Bacteria	Proteobacteria	Maricaulis maris MCS10	YP_757840.1	3 E-43	250/288
Bacteria	Proteobacteria	Burkholderia sp. H160	ZP_03268927.1	4 E-43	251/288
Bacteria	Proteobacteria	Acidovorax citrulli AAC00-1	YP_971215.1	5 E-43	250/288
Bacteria	Proteobacteria	Pseudomonas fluorescens Pf0-1	YP_346919.1	5 E-43	249/288
Bacteria	Proteobacteria	Acidiphilium cryptum JF-5	YP_001234700.1	6 E-43	248/288
Bacteria	Proteobacteria	Silicibacter lacuscaerulensis ITI-1157	ZP_05787662.1	6 E-43	255/288

Bacteria	Proteobacteria	Bradyrhizobium sp. BTAi1	YP_001236922.1	7 E-43	253/288
Bacteria	Proteobacteria	Sulfitobacter sp. NAS-14.1	ZP_00963729.1	7 E-43	257/288
Bacteria	Firmicutes	Geobacillus sp. Y4.1MC1	ZP_05372178.1	8 E-43	248/288
Bacteria	Proteobacteria	Rhodopseudomonas palustris HaA2	YP_484392.1	1 E-42	253/288
Bacteria	Firmicutes	Bacillus sp. B14905	ZP_01723753.1	1 E-42	248/288
Bacteria	Proteobacteria	Pseudomonas aeruginosa UCBPP-PA14	YP_789762.1	1 E-42	249/288
Bacteria	Thermus	Meiothermus silvanus DSM 9946	ZP_04037318.1	2 E-42	249/288
Bacteria	Proteobacteria	Pseudomonas aeruginosa PAO1	NP_252077.1	2 E-42	249/288
Bacteria	Proteobacteria	Sulfitobacter sp. EE-36	ZP_00956681.1	2 E-42	257/288
Bacteria	Proteobacteria	Rhodopseudomonas palustris BisB18	YP_530140.1	2 E-42	255/288
Bacteria	Proteobacteria	Burkholderia phytofirmans PsJN	YP_001888767.1	3 E-42	251/288
Bacteria	Proteobacteria	Pseudomonas aeruginosa 2192	ZP_04935395.1	3 E-42	249/288
Bacteria	Proteobacteria	Pseudomonas aeruginosa PACS2	ZP_01366837.1	4 E-42	249/288
Bacteria	Actinobacteria	Rhodococcus opacus B4	YP_002778396.1	4 E-42	253/288
Bacteria	Proteobacteria	Pseudomonas aeruginosa LESB58	YP_002439277.1	4 E-42	249/288
Bacteria	Proteobacteria	Pseudomonas aeruginosa C3719	ZP_04929669.1	5 E-42	249/288
Bacteria	Proteobacteria	Roseobacter sp. GAI101	ZP_05102366.1	6 E-42	257/288
Bacteria	Actinobacteria	Rhodococcus jostii RHA1	YP_701469.1	9 E-42	256/288
Eukaryota	Fungi	Magnaporthe grisea 70-15	XP_368547.1	1 E-41	261/288
Eukaryota	Fungi	Uncinocarpus reesii 1704	XP_002583947.1	2 E-41	276/288
Bacteria	Proteobacteria	Variovorax paradoxus S110	YP_002946190.1	3 E-41	246/288
Bacteria	Proteobacteria	Ralstonia solanacearum GMI1000	NP_519884.1	4 E-41	256/288
Bacteria	Proteobacteria	Ruegeria pomeroyi DSS-3	YP_167201.1	4 E-41	262/288
Bacteria	Proteobacteria	Aurantimonas manganoxydans SI85-9A1	ZP_01228339.1	4 E-41	256/288
Bacteria	Proteobacteria	Comamonas testosteroni KF-1	ZP_03542718.1	7 E-41	250/288
Eukaryota	Fungi	Cryptococcus neoformans var. neoformans	XP_570273.1	7 E-41	257/288
Bacteria	Proteobacteria	Ralstonia pickettii 12J	YP_001899016.1	7 E-41	250/288
Bacteria	Proteobacteria	Ralstonia pickettii 12D	YP_002981443.1	8 E-41	250/288
Bacteria	Proteobacteria	Rhodobacterales bacterium HTCC2654	ZP_01012179.1	9 E-41	253/288
Bacteria	Proteobacteria	Curvibacter putative symbiont of	CBA26309.1	1 E-40	252/288
Bacteria	Proteobacteria	Hoeflea phototrophica DFL-43	ZP_02167324.1	1 E-40	259/288
Bacteria	Firmicutes	Geobacillus thermodenitrificans NG80-2	YP_001125014.1	1 E-40	250/288
Bacteria	Proteobacteria	Verminephrobacter eiseniae EF01-2	YP_997117.1	1 E-40	259/288
Bacteria	Proteobacteria	Polaromonas naphthalenivorans CJ2	YP_982075.1	2 E-40	253/288
Bacteria	Proteobacteria	Acidovorax delafieldii 2AN	ZP_04761399.1	2 E-40	255/288
Eukaryota	Fungi	Coprinopsis cinerea okayama7#130	XP_001839968.1	2 E-40	266/288
Bacteria	Proteobacteria	Ralstonia solanacearum MolK2	YP_002253944.1	3 E-40	256/288
Bacteria	Proteobacteria	Polaromonas sp. JS666	YP_549381.1	4 E-40	253/288
Bacteria	Proteobacteria	Rhodobacterales bacterium HTCC2083	ZP_05074922.1	5 E-40	257/288
Bacteria	Proteobacteria	Ralstonia solanacearum UW551	ZP_00945624.1	5 E-40	256/288
Bacteria	Proteobacteria	Delftia acidovorans SPH-1	YP_001564383.1	1 E-39	253/288
Bacteria	Proteobacteria	Oceanibulbus indolifex HEL-45	ZP_02154583.1	1 E-39	257/288
Bacteria	Proteobacteria	gamma proteobacterium NOR51-B	ZP_04958797.1	2 E-39	257/288
Bacteria	Proteobacteria	Pseudomonas fluorescens Pf-5	YP_258374.1	2 E-39	249/288
Bacteria	Proteobacteria	Rhodoferax ferrireducens T118	YP_523741.1	2 E-39	250/288
Bacteria	Proteobacteria	marine gamma proteobacterium HTCC2080	ZP_01627042.1	2 E-39	249/288
Bacteria	Proteobacteria	Ralstonia metallidurans CH34	YP_583999.1	3 E-39	256/288
Bacteria	Proteobacteria	Acidovorax delafieldii 2AN	ZP_04761940.1	3 E-39	252/288
Bacteria	Proteobacteria	Pseudomonas mendocina ymp	YP_001187200.1	4 E-39	249/288
Bacteria	Proteobacteria	Ralstonia eutropha JMP134	YP_295623.1	4 E-39	256/288
Bacteria	Proteobacteria	Variovorax paradoxus S110	YP_002944805.1	4 E-39	252/288
Bacteria	Proteobacteria	Ralstonia eutropha H16	YP_726031.1	5 E-39	256/288
Bacteria	Proteobacteria	Acidovorax sp. JS42	YP_985961.1	6 E-39	250/288
Bacteria	Proteobacteria	Mesorhizobium loti MAFF303099	NP_104279.1	9 E-39	256/288
Bacteria	Actinobacteria	Salinispora tropica CNB-440	YP_001159657.1	1 E-38	251/288
Bacteria	Thermus	Deinococcus radiodurans R1	NP_285523.1	1 E-38	252/288
Eukaryota	Fungi	Ustilago maydis 521	XP_758329.1	1 E-38	270/288
Bacteria	Firmicutes	Lysinibacillus sphaericus C3-41	YP_001698866.1	1 E-38	235/288

Bacteria	Proteobacteria	Cupriavidus taiwanensis	YP_002005488.1	1 E-38	256/288
Eukaryota	stramenopiles	Phaeodactylum tricornutum CCAP 1055/1	XP_002186286.1	1 E-38	252/288
Bacteria	Proteobacteria	Phenylobacterium zucineum HLK1	YP_002132054.1	2 E-38	248/288
Bacteria	Proteobacteria	Marinobacter algicola DG893	ZP_01895883.1	2 E-38	252/288
Bacteria	Proteobacteria	Marinobacter sp. ELB17	ZP_01736744.1	2 E-38	252/288
Eukaryota	Fungi	Phaeosphaeria nodorum SN15	XP_001806536.1	2 E-38	256/288
Eukaryota	Fungi	Laccaria bicolor S238N-H82	XP_001878090.1	2 E-38	269/288
Bacteria	Proteobacteria	Sphingomonas sp. SKA58	ZP_01301757.1	3 E-38	255/288
Bacteria	Proteobacteria	Ralstonia eutropha JMP134	YP_298243.1	3 E-38	253/288
Bacteria	Firmicutes	Geobacillus kaustophilus HTA426	YP_146882.1	3 E-38	250/288
Bacteria	Firmicutes	Geobacillus sp. Y412MC52	ZP_04393621.1	5 E-38	250/288
Bacteria	Firmicutes	Geobacillus sp. WCH70	YP_002949055.1	7 E-38	249/288
Bacteria	Proteobacteria	Thiomonas intermedia K12	ZP_05500579.1	2 E-37	249/288
Bacteria	Proteobacteria	Burkholderia ambifaria IOP40-10	ZP_02891583.1	2 E-37	258/288
Eukaryota	Viridiplantae	Micromonas sp. RCC299	XP_002502806.1	2 E-37	262/288
Bacteria	Proteobacteria	Cupriavidus taiwanensis	YP_002007805.1	3 E-37	253/288
Bacteria	Proteobacteria	Phenylobacterium zucineum HLK1	YP_002131973.1	5 E-37	248/288
Bacteria	Thermus	Deinococcus deserti VCD115	YP_002787974.1	5 E-37	252/288
Bacteria	Proteobacteria	Ralstonia eutropha H16	YP_840760.1	5 E-37	253/288
Eukaryota	Viridiplantae	Ostreococcus tauri	CAL55150.1	6 E-37	267/288
Bacteria	Firmicutes	Bacillus sp. NRRL B-14911	ZP_01172595.1	6 E-37	252/288
Bacteria	Proteobacteria	Congregibacter litoralis KT71	ZP_01101959.1	7 E-37	247/288
Bacteria	Proteobacteria	Caulobacter crescentus CB15	NP_420099.1	1 E-36	255/288
Bacteria	Firmicutes	Oceanobacillus iheyensis HTE831	NP_691596.1	2 E-35	249/288
Bacteria	Thermus	Deinococcus geothermalis DSM 11300	YP_593911.1	3 E-35	252/288
Bacteria	Proteobacteria	Caulobacter sp. K31	YP_001684688.1	3 E-35	250/288
Bacteria	Proteobacteria	Marinobacter algicola DG893	ZP_01896139.1	3 E-35	252/288
Bacteria	Proteobacteria	Roseobacter sp. MED193	ZP_01057466.1	3 E-35	256/288
Bacteria	Proteobacteria	Caulobacter sp. K31	YP_001682480.1	5 E-35	253/288
Bacteria	Proteobacteria	Labrenzia alexandrii DFL-11	ZP_05114714.1	6 E-35	255/288
Bacteria	Proteobacteria	Hyphomonas neptunium ATCC 15444	YP_759561.1	7 E-35	253/288
Bacteria	Proteobacteria	Ralstonia metallidurans CH34	YP_586876.1	1 E-34	253/288
Bacteria	Proteobacteria	Caulobacter crescentus CB15	NP_420382.1	1 E-34	250/288
Bacteria	Proteobacteria	gamma proteobacterium NOR5-3	ZP_05129117.1	1 E-34	251/288
Bacteria	Proteobacteria	Brevundimonas sp. BAL3	ZP_05032440.1	2 E-34	249/288
Eukaryota	Viridiplantae	Micromonas pusilla CCMP1545	EEH54571.1	2 E-34	265/288
Bacteria	Proteobacteria	Phenylobacterium zucineum HLK1	YP_002131393.1	7 E-34	251/288
Bacteria	Proteobacteria	Rhodopseudomonas palustris BisB5	YP_570332.1	1 E-33	253/288
Eukaryota	stramenopiles	Thalassiosira pseudonana CCMP1335	XP_002296326.1	1 E-33	269/288
Bacteria	Firmicutes	Bacillus coahuilensis m4-4	ZP_03227801.1	2 E-33	250/288
Bacteria	Firmicutes	Bacillus coagulans 36D1	ZP_04431112.1	3 E-33	252/288
Bacteria	Actinobacteria	Rhodococcus jostii RHA1	YP_706915.1	3 E-33	259/288
Bacteria	Proteobacteria	Pectobacterium carotovorum subsp. carotovorur	ZP_03832124.1	4 E-33	250/288
Bacteria	Proteobacteria	Pectobacterium carotovorum subsp. carotovorur	YP_003017815.1	5 E-33	250/288
Bacteria	Proteobacteria	Sphingomonas wittichii RW1	YP_001262803.1	5 E-33	250/288
Bacteria	Firmicutes	Blautia hydrogenotrophica DSM 10507	ZP_03782731.1	6 E-33	244/288
Bacteria	Proteobacteria	Pseudovibrio sp. JE062	ZP_05084561.1	6 E-33	262/288
Bacteria	Proteobacteria	Sphingomonas sp. SKA58	ZP_01301770.1	6 E-33	254/288
Bacteria	Proteobacteria	Rhodoferax ferrireducens T118	YP_525276.1	7 E-33	241/288
Bacteria	Proteobacteria	Sphingopyxis alaskensis RB2256	YP_618108.1	8 E-33	255/288
Bacteria	Proteobacteria	Acidiphilium cryptum JF-5	YP_001233759.1	1 E-32	252/288
Bacteria	Proteobacteria	Caulobacter crescentus CB15	NP_421870.1	2 E-32	253/288
Bacteria	Firmicutes	Ruminococcus obeum ATCC 29174	ZP_01965754.1	2 E-32	244/288
Bacteria	Firmicutes	Dorea formicigenerans ATCC 27755	ZP_02233341.1	2 E-32	244/288
Bacteria	Proteobacteria	Bradyrhizobium sp. BTAi1	YP_001242857.1	3 E-32	246/288
Bacteria	Proteobacteria	Enterobacter sp. 638	YP_001176766.1	4 E-32	241/288

AFUA_1G17550

Eukaryota	Fungi	Aspergillus fumigatus Af293	XP_753121.1	0.0	793/793
Eukaryota	Fungi	Aspergillus nidulans FGSC A4	XP_660012.1	2 E-47	648/793

AFUA_2G00160

Eukaryota	Fungi	Aspergillus fumigatus Af293	XP_749152.1	1 E-114	202/202
Eukaryota	Fungi	Neosartorya fischeri NRRL 181	XP_001265566.1	5 E-99	202/202
Eukaryota	Fungi	Talaromyces stipitatus ATCC 10500	XP_002482853.1	5 E-38	180/202
Eukaryota	Fungi	Aspergillus flavus NRRL3357	XP_002381450.1	3 E-30	175/202
Eukaryota	Fungi	Aspergillus fumigatus Af293	XP_746869.1	6 E-30	164/202
Eukaryota	Fungi	Neosartorya fischeri NRRL 181	XP_001262695.1	6 E-30	164/202
Eukaryota	Fungi	Aspergillus oryzae RIB40	XP_001824343.1	2 E-29	167/202
Eukaryota	Fungi	Botryotinia fuckeliana B05.10	XP_001549419.1	2 E-28	190/202
Eukaryota	Fungi	Phaeosphaeria nodorum SN15	XP_001791599.1	3 E-28	162/202
Eukaryota	Fungi	Aspergillus nidulans FGSC A4	XP_664347.1	4 E-24	173/202
Eukaryota	Fungi	Botryotinia fuckeliana B05.10	XP_001556396.1	1 E-23	163/202
Eukaryota	Fungi	Aspergillus flavus NRRL3357	XP_002372307.1	2 E-22	164/202
Eukaryota	Fungi	Penicillium chrysogenum Wisconsin 54-1255	XP_002560021.1	2 E-21	164/202
Eukaryota	Fungi	Aspergillus clavatus NRRL 1	XP_001273155.1	3 E-21	178/202
Eukaryota	Fungi	Magnaporthe grisea 70-15	XP_363197.1	2 E-20	165/202
Eukaryota	Fungi	Aspergillus fumigatus A1163	EDP53761.1	5 E-18	179/202
Eukaryota	Fungi	Neosartorya fischeri NRRL 181	XP_001265658.1	6 E-18	179/202
Eukaryota	Fungi	Ajellomyces capsulatus H143	EER38176.1	1 E-16	168/202
Eukaryota	Fungi	Neosartorya fischeri NRRL 181	XP_001262725.1	6 E-15	162/202
Eukaryota	Fungi	Chaetomium globosum CBS 148.51	XP_001222877.1	2 E-13	190/202

AFUA_2G00720

Eukaryota	Fungi	Aspergillus fumigatus Af293	XP_749209.1	0.0	492/492
Eukaryota	Fungi	Neosartorya fischeri NRRL 181	XP_001265630.1	0.0	492/492
Eukaryota	Fungi	Aspergillus clavatus NRRL 1	XP_001273140.1	0.0	489/492
Eukaryota	Fungi	Aspergillus oryzae RIB40	XP_001823180.1	0.0	491/492
Eukaryota	Fungi	Aspergillus flavus NRRL3357	XP_002378518.1	0.0	499/492
Eukaryota	Fungi	Penicillium chrysogenum Wisconsin 54-1255	XP_002566374.1	0.0	491/492
Eukaryota	Fungi	Aspergillus niger CBS 513.88	XP_001402476.1	0.0	493/492
Eukaryota	Fungi	Coccidioides posadasii C735 delta	EER27049.1	0.0	487/492
Eukaryota	Fungi	Coccidioides immitis RS;	XP_001240016.1	0.0	487/492
Eukaryota	Fungi	Gibberella zeae PH-1	XP_381935.1	1 E-180	488/492
Eukaryota	Fungi	Gibberella zeae PH-1	XP_382472.1	1 E-178	492/492
Eukaryota	Fungi	Nectria haematococca mpVI 77-13-4	EEU42663.1	1 E-176	491/492
Eukaryota	Fungi	Gibberella zeae PH-1	XP_386007.1	1 E-163	490/492
Eukaryota	Fungi	Uncinocarpus reesii 1704	XP_002545080.1	1 E-154	444/492
Eukaryota	Fungi	Phaeosphaeria nodorum SN15	XP_001802029.1	1 E-138	469/492
Eukaryota	Fungi	Neosartorya fischeri NRRL 181	XP_001258260.1	1 E-136	470/492
Eukaryota	Fungi	Neurospora crassa OR74A	XP_956862.1	1 E-134	470/492
Eukaryota	Fungi	Aspergillus fumigatus Af293	XP_751026.1	1 E-134	470/492
Eukaryota	Fungi	Aspergillus fumigatus Af293	XP_746831.1	1 E-133	474/492
Eukaryota	Fungi	Chaetomium globosum CBS 148.51	XP_001225795.1	1 E-133	470/492
Bacteria	Cyanobacteria	Cyanothece sp. ATCC 51142	YP_001804643.1	1 E-132	472/492
Bacteria	Cyanobacteria	Cyanothece sp. CCY0110	ZP_01726360.1	1 E-132	472/492
Eukaryota	Fungi	Botryotinia fuckeliana B05.10	XP_001554714.1	1 E-132	487/492
Eukaryota	Fungi	Aspergillus clavatus NRRL 1	XP_001272452.1	1 E-132	475/492
Eukaryota	Fungi	Nectria haematococca mpVI 77-13-4	EEU41877.1	1 E-132	485/492
Eukaryota	Fungi	Podospora anserina DSM 980	XP_001909997.1	1 E-131	470/492
Eukaryota	Fungi	Aspergillus oryzae RIB40	XP_001821011.1	1 E-131	474/492
Eukaryota	Fungi	Neosartorya fischeri NRRL 181	XP_001262737.1	1 E-131	474/492
Eukaryota	Fungi	Nectria haematococca mpVI 77-13-4	EEU48018.1	1 E-130	470/492

Eukaryota	Fungi	Pyrenophora tritici-repentis Pt-1C-BFP	XP_001931266.1	1 E-130	471/492
Eukaryota	Fungi	Aspergillus terreus NIH2624	XP_001214198.1	1 E-130	474/492
Eukaryota	Fungi	Beauveria bassiana	ABG77527.1	1 E-129	484/492
Eukaryota	Fungi	Ustilago maydis 521	XP_758655.1	1 E-129	487/492
Eukaryota	Fungi	Aspergillus clavatus NRRL 1	XP_001268240.1	1 E-129	470/492
Eukaryota	Fungi	Verticillium albo-atrum VaMs.102	EEY15433.1	1 E-129	472/492
Eukaryota	Fungi	Passalora fulva	AAF82789.1	1 E-129	470/492
Eukaryota	Fungi	Gibberella zeae PH-1	XP_382336.1	1 E-128	485/492
Bacteria	Cyanobacteria	Microcoleus chthonoplastes PCC 7420	ZP_05023575.1	1 E-128	472/492
Eukaryota	Fungi	Uncinocarpus reesii 1704	XP_002545205.1	1 E-128	471/492
Eukaryota	Fungi	Phaeosphaeria nodorum SN15	XP_001797286.1	1 E-128	471/492
Eukaryota	Fungi	Gibberella zeae PH-1	XP_381155.1	1 E-128	470/492
Eukaryota	Fungi	Penicillium chrysogenum Wisconsin 54-1255	XP_002556634.1	1 E-128	474/492
Bacteria	Cyanobacteria	Crocosphaera watsonii WH 8501	ZP_00518574.1	1 E-128	472/492
Eukaryota	Fungi	Magnaporthe grisea 70-15	XP_361426.1	1 E-128	487/492
Eukaryota	Fungi	Ajellomyces capsulatus G186AR	EEH05124.1	1 E-128	486/492
Eukaryota	Fungi	Ajellomyces capsulatus H143	EER40883.1	1 E-128	488/492
Eukaryota	Fungi	Cryptococcus neoformans var. neoformans	XP_571348.1	1 E-128	479/492
Eukaryota	Fungi	Alternaria alternata	P42041.2	1 E-127	487/492
Eukaryota	Fungi	Davidiella tassiana	P40108.2	1 E-127	486/492
Eukaryota	Fungi	Aciculosporium take	BAF57023.1	1 E-127	470/492
Eukaryota	Fungi	Aspergillus niger CBS 513.88	XP_001392844.1	1 E-127	486/492
Eukaryota	Fungi	Nectria haematococca mpVI 77-13-4	EEU36924.1	1 E-127	470/492
Eukaryota	Fungi	Microsporum canis CBS 113480	EEQ30142.1	1 E-127	471/492
Bacteria	Cyanobacteria	Cyanothece sp. PCC 7822	ZP_03156326.1	1 E-127	472/492
Eukaryota	Metazoa	Strongylocentrotus purpuratus	XP_786787.2	1 E-127	474/492
Eukaryota	Fungi	Emericella nidulans	AAK18073.1	1 E-126	474/492
Eukaryota	Fungi	Talaromyces stipitatus ATCC 10500	XP_002487946.1	1 E-126	487/492
Eukaryota	Fungi	Aspergillus nidulans FGSC A4	XP_658158.1	1 E-126	474/492
Eukaryota	Fungi	Podospora anserina DSM 980	XP_001907828.1	1 E-126	487/492
Eukaryota	Fungi	Aspergillus niger	P41751.1	1 E-126	486/492
Bacteria	Cyanobacteria	Cyanothece sp. PCC 7424	YP_002379008.1	1 E-126	472/492
Eukaryota	Fungi	Emericella nidulans	AAK18074.1	1 E-126	474/492
Bacteria	Cyanobacteria	Trichodesmium erythraeum IMS101	YP_722268.1	1 E-126	472/492
Eukaryota	Fungi	Penicillium chrysogenum Wisconsin 54-1255	XP_002569248.1	1 E-126	471/492
Eukaryota	Fungi	Nectria haematococca mpVI 77-13-4	EEU35791.1	1 E-126	484/492
Eukaryota	Fungi	Ajellomyces dermatitidis ER-3	EEQ89540.1	1 E-126	486/492
Eukaryota	Metazoa	Trichoplax adhaerens	XP_002110540.1	1 E-125	471/492
Eukaryota	Fungi	Laccaria bicolor S238N-H82	XP_001889968.1	1 E-125	477/492
Eukaryota	Fungi	Gibberella zeae PH-1	XP_382449.1	1 E-125	492/492
Eukaryota	Fungi	Verticillium albo-atrum VaMs.102	EEY19032.1	1 E-125	485/492
Eukaryota	Metazoa	Trichoplax adhaerens	XP_002110539.1	1 E-125	471/492
Eukaryota	Fungi	Coccidioides immitis RS;	XP_001240184.1	1 E-125	471/492
Eukaryota	Fungi	Nectria haematococca mpVI 77-13-4	EEU48181.1	1 E-125	470/492
Eukaryota	Metazoa	Danio rerio	AAI16560.1	1 E-125	470/492
Eukaryota	Fungi	Yarrowia lipolytica CLIB122	XP_504993.1	1 E-125	487/492
Eukaryota	Metazoa	Ixodes scapularis	XP_002415064.1	1 E-125	470/492
Eukaryota	Fungi	Paracoccidioides brasiliensis Pb03;	EEH18549.1	1 E-125	483/492
Eukaryota	Fungi	Paracoccidioides brasiliensis Pb01;	EEH34200.1	1 E-125	471/492
Eukaryota	Metazoa	Danio rerio	AAI07975.1	1 E-125	472/492
Eukaryota	Metazoa	Danio rerio	CAN87982.1	1 E-125	470/492
Eukaryota	Metazoa	Xenopus (Silurana) tropicalis	CAJ83424.1	1 E-125	474/492
Eukaryota	Metazoa	Danio rerio	NP_998466.2	1 E-125	470/492
Eukaryota	Fungi	Coccidioides posadasii C735 delta	EER26940.1	1 E-125	471/492
Eukaryota	Fungi	Laccaria bicolor S238N-H82	XP_001877085.1	1 E-124	486/492
Eukaryota	Metazoa	Xenopus (Silurana) tropicalis	NP_001004907.1	1 E-124	474/492
Eukaryota	Metazoa	Sus scrofa	XP_001924254.1	1 E-124	470/492
Bacteria	Cyanobacteria	Lyngbya sp. PCC 8106	ZP_01618896.1	1 E-124	471/492

Eukaryota	Fungi	Magnaporthe grisea 70-15	XP_359769.1	1 E-124	487/492
Eukaryota	Fungi	Aspergillus clavatus NRRL 1	XP_001275042.1	1 E-124	485/492
Eukaryota	Fungi	Neurospora crassa OR74A	XP_957264.1	1 E-124	487/492
Eukaryota	Fungi	Talaromyces stipitatus ATCC 10500	XP_002484160.1	1 E-124	482/492
Bacteria	Cyanobacteria	Cyanothece sp. PCC 8802	YP_003137302.1	1 E-124	472/492
Eukaryota	Metazoa	Schistosoma mansoni	XP_002573730.1	1 E-124	475/492
Eukaryota	Metazoa	Salmo salar	ACI33614.1	1 E-124	470/492
Eukaryota	Metazoa	Taeniopygia guttata	XP_002191253.1	1 E-124	476/492
Eukaryota	Metazoa	Caenorhabditis briggsae AF16	XP_001666639.1	1 E-123	472/492
Eukaryota	Fungi	Penicillium marneffei ATCC 18224	XP_002152345.1	1 E-123	471/492
Eukaryota	Metazoa	Canis lupus familiaris	XP_538742.2	1 E-123	474/492
Eukaryota	Fungi	Chaetomium globosum CBS 148.51	XP_001229422.1	1 E-123	487/492
Eukaryota	Fungi	Emericella nidulans	AAA33293.1	1 E-123	474/492
Eukaryota	Metazoa	Caenorhabditis elegans	NP_498081.2	1 E-123	472/492
Eukaryota	Fungi	Penicillium marneffei ATCC 18224	XP_002153479.1	1 E-123	487/492
Eukaryota	Metazoa	Ovis aries	NP_001009778.1	1 E-123	476/492
Eukaryota	Metazoa	Bos taurus	P48644.3	1 E-123	476/492
Eukaryota	Fungi	Coprinopsis cinerea okayama7#130	XP_001834689.1	1 E-123	475/492
Eukaryota	Metazoa	Pan troglodytes	XP_001170537.1	1 E-123	470/492
Eukaryota	Metazoa	Bos taurus	NP_776664.1	1 E-123	476/492
Eukaryota	Fungi	Gibberella zeae PH-1	XP_384370.1	1 E-122	480/492
Eukaryota	Fungi	Nectria haematococca mpVI 77-13-4	EEU39378.1	1 E-122	480/492
Eukaryota	Metazoa	Homo sapiens	BAG36147.1	1 E-122	470/492
Eukaryota	Metazoa	Homo sapiens	NP_000683.3	1 E-122	470/492
Eukaryota	Metazoa	Xenopus laevis	NP_001087022.1	1 E-122	474/492
Eukaryota	Metazoa	Ornithorhynchus anatinus	XP_001519422.1	1 E-122	474/492
Eukaryota	Metazoa	Tetraodon nigroviridis	CAF94009.1	1 E-122	470/492
Eukaryota	Fungi	Pyrenophora tritici-repentis Pt-1C-BFP	XP_001937452.1	1 E-122	488/492
Eukaryota	Metazoa	Mus musculus	BAE27044.1	1 E-122	470/492
Eukaryota	Metazoa	Mus musculus	NP_033786.1	1 E-122	470/492
Eukaryota	Metazoa	Pongo abelii	NP_001127576.1	1 E-122	470/492
Eukaryota	Metazoa	Bos taurus	NP_001068835.1	1 E-122	470/492
Eukaryota	Metazoa	Mus musculus	BAE31741.1	1 E-122	470/492
Eukaryota	Metazoa	Homo sapiens	AAA96830.1	1 E-122	470/492
Eukaryota	Metazoa	Oryctolagus cuniculus	Q8MI17.1	1 E-122	476/492
Eukaryota	Metazoa	Homo sapiens	P30837.2	1 E-122	470/492
Eukaryota	Fungi	Pyrenophora tritici-repentis Pt-1C-BFP	XP_001933249.1	1 E-122	486/492
Eukaryota	Fungi	Pichia pastoris GS115	XP_002493450.1	1 E-122	478/492
Eukaryota	Metazoa	Homo sapiens	AAT41621.1	1 E-122	470/492
Eukaryota	Metazoa	Homo sapiens	AAA51693.1	1 E-122	470/492
Eukaryota	Metazoa	Sus scrofa	NP_001038076.1	1 E-122	470/492
Eukaryota	Fungi	Neosartorya fischeri NRRL 181	XP_001267163.1	1 E-122	489/492
Eukaryota	Metazoa	Sus scrofa	XP_001924933.1	1 E-121	470/492
Eukaryota	Metazoa	Xenopus laevis	NP_001089074.1	1 E-121	470/492
Eukaryota	Metazoa	Homo sapiens	NP_000681.2	1 E-121	470/492
Eukaryota	Metazoa	Pongo abelii	NP_001124747.1	1 E-121	470/492
Eukaryota	Fungi	Fusarium oxysporum;	ACN65117.1	1 E-121	494/492
Eukaryota	Metazoa	Rattus norvegicus	EDL98822.1	1 E-121	470/492
Eukaryota	Metazoa	Mus musculus	BAE29958.1	1 E-121	470/492
Eukaryota	Metazoa	Macaca fascicularis	Q8HYE4.3	1 E-121	476/492
Eukaryota	Metazoa	Macaca mulatta	XP_001097512.1	1 E-121	476/492
Eukaryota	Fungi	Aspergillus flavus NRRL3357	XP_002379232.1	1 E-121	490/492
Eukaryota	Fungi	Aspergillus oryzae RIB40	XP_001822148.1	1 E-121	490/492
Eukaryota	Metazoa	Homo sapiens	BAD97093.1	1 E-121	470/492
Eukaryota	Metazoa	Gallus gallus	NP_989908.1	1 E-121	476/492
Eukaryota	Metazoa	Rattus norvegicus	AAM94394.2	1 E-121	470/492
Eukaryota	Metazoa	Homo sapiens	AAC51652.1	1 E-121	476/492
Eukaryota	Metazoa	Rattus norvegicus	NP_115792.1	1 E-121	470/492

Eukaryota	Fungi	Penicillium marneffei ATCC 18224	XP_002150118.1	1 E-121	477/492
Eukaryota	Metazoa	Homo sapiens	NP_000680.2	1 E-121	476/492
Eukaryota	Fungi	Malassezia globosa CBS 7966	XP_001731550.1	1 E-121	495/492
Eukaryota	Metazoa	Rattus norvegicus	EDM13724.1	1 E-121	470/492
Eukaryota	Metazoa	Mus musculus	BAE35596.1	1 E-121	470/492
Eukaryota	Metazoa	Rattus norvegicus	AAS75813.1	1 E-121	470/492
Eukaryota	Fungi	Schizosaccharomyces pombe	NP_595007.1	1 E-121	482/492
Eukaryota	Fungi	Botryotinia fuckeliana B05.10	XP_001559415.1	1 E-121	490/492
Eukaryota	Metazoa	Drosophila mojavensis	XP_002003812.1	1 E-121	471/492
Eukaryota	Metazoa	Rattus norvegicus	AAS75814.1	1 E-121	470/492
Eukaryota	Metazoa	Acyrthosiphon pisum	XP_001945750.1	1 E-121	472/492
Eukaryota	Fungi	Laccaria bicolor S238N-H82	XP_001883222.1	1 E-121	471/492
Eukaryota	Metazoa	Mesocricetus auratus	P81178.1	1 E-121	470/492
Eukaryota	Metazoa	Macaca mulatta	XP_001114412.1	1 E-121	475/492
Eukaryota	Metazoa	Sus scrofa	ACD02422.1	1 E-121	470/492
Eukaryota	Metazoa	Danio rerio	NP_956784.1	1 E-121	470/492
Eukaryota	Fungi	Nectria haematococca mpVI 77-13-4	EEU41589.1	1 E-121	474/492
Eukaryota	Metazoa	Danio rerio	AAM19352.1	1 E-121	470/492
Eukaryota	Metazoa	Gallus gallus	XP_415171.2	1 E-121	474/492
Eukaryota	Metazoa	Mus musculus	BAE30339.1	1 E-121	470/492
Eukaryota	Metazoa	Mus musculus	NP_082546.1	1 E-121	470/492
Eukaryota	Metazoa	Rattus norvegicus	NP_001011975.1	1 E-121	470/492
Eukaryota	Metazoa	Monodelphis domestica	XP_001372410.1	1 E-121	474/492
Eukaryota	Metazoa	Homo sapiens	CAG33272.1	1 E-121	470/492
Eukaryota	Fungi	Aspergillus terreus NIH2624	XP_001214516.1	1 E-121	489/492
Eukaryota	Metazoa	Rattus norvegicus	AAS75815.1	1 E-121	470/492
Eukaryota	Fungi	Coprinopsis cinerea okayama7#130	XP_001834665.1	1 E-121	480/492
Eukaryota	Fungi	Penicillium marneffei ATCC 18224	XP_002146930.1	1 E-120	493/492
Eukaryota	Metazoa	Mus musculus	NP_036051.1	1 E-120	476/492
Eukaryota	Fungi	Aspergillus niger CBS 513.88	XP_001401707.1	1 E-120	490/492
Eukaryota	Fungi	Pichia pastoris GS115	XP_002491418.1	1 E-120	487/492
Eukaryota	Fungi	Nectria haematococca mpVI 77-13-4	EEU45748.1	1 E-120	490/492
Eukaryota	Metazoa	Equus caballus	XP_001490960.1	1 E-120	470/492
Eukaryota	Fungi	Cryptococcus neoformans var. neoformans	XP_773561.1	1 E-120	479/492
Eukaryota	Fungi	Penicillium marneffei ATCC 18224	XP_002145748.1	1 E-120	467/492
Eukaryota	Metazoa	Danio rerio	CAM13323.1	1 E-120	470/492
Eukaryota	Metazoa	Taeniopygia guttata	XP_002196279.1	1 E-120	474/492
Eukaryota	Metazoa	Pongo abelii	NP_001127609.1	1 E-120	476/492
Eukaryota	Metazoa	Canis lupus familiaris	XP_853628.1	1 E-120	470/492
Eukaryota	Metazoa	Oryzias latipes	NP_001098291.1	1 E-120	472/492
Eukaryota	Fungi	Aspergillus fumigatus Af293	XP_751967.1	1 E-120	489/492
Eukaryota	Fungi	Aspergillus fumigatus A1163	EDP50237.1	1 E-120	489/492
Eukaryota	Metazoa	Nasonia vitripennis	XP_001604192.1	1 E-120	470/492
Eukaryota	Metazoa	Drosophila mojavensis	XP_002002963.1	1 E-120	472/492
Eukaryota	Metazoa	Macroscelides proboscideus	Q29490.1	1 E-120	476/492
Eukaryota	Metazoa	Xenopus laevis	NP_001084244.1	1 E-120	474/492
Eukaryota	Metazoa	Branchiostoma floridae	XP_002592527.1	1 E-120	472/492
Eukaryota	Metazoa	Homo sapiens	CAA68290.1	1 E-120	470/492
Eukaryota	Metazoa	Salmo salar	NP_001135258.1	1 E-120	476/492
Eukaryota	Metazoa	Rattus norvegicus	NP_058968.14	1 E-120	476/492
Eukaryota	Fungi	Sclerotinia sclerotiorum 1980 UF-70	XP_001598693.1	1 E-120	490/492
Eukaryota	Fungi	Aspergillus oryzae RIB40	XP_001821214.1	1 E-119	490/492
Eukaryota	Fungi	Aspergillus terreus NIH2624	XP_001216921.1	1 E-119	491/492
Eukaryota	Metazoa	Drosophila mojavensis	XP_002003813.1	1 E-119	471/492
Eukaryota	Metazoa	Mus musculus	AAH44729.1	1 E-119	476/492
Eukaryota	Metazoa	Mus musculus	NP_038495.2	1 E-119	476/492
Eukaryota	Fungi	Cryptococcus neoformans var. neoformans	XP_568049.1	1 E-119	479/492
Eukaryota	Metazoa	Rattus norvegicus	NP_446348.2	1 E-119	476/492

Eukaryota	Metazoa	Elephantulus edwardii	Q28399.1	1 E-119	476/492
Eukaryota	Metazoa	Drosophila melanogaster	AAY85116.1	1 E-119	472/492
Eukaryota	Metazoa	Xenopus (Silurana) tropicalis	NP_001039196.1	1 E-119	476/492
Eukaryota	Metazoa	Canis lupus familiaris	XP_535494.2	1 E-119	476/492
Eukaryota	Metazoa	Drosophila virilis	XP_002059255.1	1 E-119	473/492
Eukaryota	Metazoa	Homo sapiens	CAA28990.1	1 E-119	470/492
Eukaryota	Metazoa	Oryctolagus cuniculus	NP_001075482.1	1 E-119	476/492
Eukaryota	Fungi	Talaromyces stipitatus ATCC 10500	XP_002479224.1	1 E-119	493/492
Eukaryota	Metazoa	Mus musculus	NP_033048.2	1 E-119	476/492
Eukaryota	Metazoa	Mus musculus	CAA67666.1	1 E-119	476/492
Eukaryota	Metazoa	Drosophila grimshawi	XP_001993018.1	1 E-119	472/492
Eukaryota	Metazoa	Canis lupus familiaris	XP_533525.1	1 E-119	476/492
Eukaryota	Metazoa	Mus musculus	AAB32754.2	1 E-119	476/492
Eukaryota	Viridiplantae	Oryza sativa Indica Group	EAY74759.1	1 E-119	479/492
Eukaryota	Fungi	Penicillium chrysogenum Wisconsin 54-1255	XP_002562117.1	1 E-119	494/492
Eukaryota	Fungi	Penicillium chrysogenum Wisconsin 54-1255	XP_002565636.1	1 E-119	490/492
Eukaryota	Metazoa	Drosophila melanogaster	NP_609285.1	1 E-119	472/492
Eukaryota	Metazoa	Homo sapiens	NP_003879.2	1 E-119	476/492
Eukaryota	Metazoa	Drosophila grimshawi	XP_001993163.1	1 E-119	471/492
Eukaryota	Metazoa	Macaca mulatta	XP_001090504.1	1 E-119	474/492
Eukaryota	Metazoa	Drosophila sechellia	XP_002036327.1	1 E-119	472/492
Eukaryota	Metazoa	Pan troglodytes	XP_001172122.1	1 E-119	474/492
Eukaryota	Metazoa	Anopheles gambiae str. PEST	XP_313425.3	1 E-119	472/492
Eukaryota	Metazoa	Homo sapiens	BAA34785.1	1 E-119	476/492
Eukaryota	Metazoa	Equus caballus	P12762.1	1 E-119	470/492
Eukaryota	Metazoa	Drosophila willistoni	XP_002067091.1	1 E-119	495/492
Eukaryota	Viridiplantae	Oryza sativa Indica Group	EAY74764.1	1 E-119	479/492
Eukaryota	Fungi	Lachancea thermotolerans CBS 6340	XP_002554007.1	1 E-119	485/492
Eukaryota	Metazoa	Drosophila melanogaster	ABV69001.1	1 E-119	472/492
Eukaryota	Fungi	Aspergillus nidulans FGSC A4	XP_661730.1	1 E-119	485/492
Eukaryota	Metazoa	Monodelphis domestica	XP_001368154.1	1 E-119	476/492
Eukaryota	Fungi	Gibberella zeae PH-1	XP_380315.1	1 E-119	490/492
Eukaryota	Metazoa	Xenopus laevis	AAH77256.1	1 E-118	472/492
Eukaryota	Viridiplantae	Oryza sativa Japonica Group	NP_001043454.1	1 E-118	471/492
Eukaryota	Metazoa	Gallus gallus	O93344.2	1 E-118	476/492

AFUA_2G00730

Eukaryota	Fungi	Aspergillus fumigatus Af293	XP_749210.1	0.0	395/395
Eukaryota	Fungi	Neosartorya fischeri NRRL 181	XP_001265631.1	0.0	378/395
Eukaryota	Fungi	Aspergillus clavatus NRRL 1	XP_001273141.1	1 E-167	378/395
Eukaryota	Fungi	Aspergillus nidulans FGSC A4	XP_682577.1	1 E-151	386/395
Eukaryota	Fungi	Aspergillus flavus NRRL3357	XP_002378519.1	1 E-150	381/395
Eukaryota	Fungi	Aspergillus oryzae RIB40	XP_001823181.1	1 E-150	381/395
Eukaryota	Fungi	Penicillium chrysogenum Wisconsin 54-1255	XP_002566375.1	1 E-141	382/395
Eukaryota	Fungi	Aspergillus terreus NIH2624	XP_001216742.1	1 E-141	378/395
Eukaryota	Fungi	Coccidioides immitis RS;	XP_001247631.1	1 E-115	384/395
Eukaryota	Fungi	Uncinocarpus reesii 1704	XP_002541810.1	1 E-115	388/395
Eukaryota	Fungi	Coccidioides posadasii C735 delta	EER23656.1	1 E-114	384/395
Eukaryota	Fungi	Paracoccidioides brasiliensis Pb03;	EEH21462.1	1 E-110	387/395
Eukaryota	Fungi	Paracoccidioides brasiliensis Pb01;	EEH39164.1	1 E-110	385/395
Eukaryota	Fungi	Paracoccidioides brasiliensis Pb18;	EEH43859.1	1 E-109	385/395
Eukaryota	Fungi	Neosartorya fischeri NRRL 181	XP_001259062.1	2 E-87	362/395
Eukaryota	Fungi	Aspergillus terreus NIH2624	XP_001214152.1	8 E-82	362/395
Eukaryota	Fungi	Penicillium chrysogenum Wisconsin 54-1255	XP_002561260.1	2 E-81	361/395
Eukaryota	Fungi	Aspergillus niger CBS 513.88	XP_001398520.1	2 E-78	353/395
Eukaryota	Fungi	Neosartorya fischeri NRRL 181	XP_001261799.1	1 E-74	387/395
Eukaryota	Fungi	Botryotinia fuckeliana B05.10	XP_001558345.1	5 E-70	371/395

Eukaryota	Fungi	Aspergillus fumigatus Af293	XP_751370.1	4 E-69	350/395
Eukaryota	Fungi	Aspergillus fumigatus A1163	EDP50847.1	6 E-69	350/395
Eukaryota	Fungi	Neosartorya fischeri NRRL 181	XP_001266588.1	3 E-68	357/395
Eukaryota	Fungi	Nectria haematococca mpVI 77-13-4	EEU48765.1	1 E-67	386/395
Eukaryota	Fungi	Sclerotinia sclerotiorum 1980 UF-70	XP_001590963.1	5 E-67	372/395
Eukaryota	Fungi	Aspergillus nidulans FGSC A4	XP_680491.1	3 E-64	355/395
Eukaryota	Fungi	Talaromyces stipitatus ATCC 10500	XP_002484385.1	1 E-63	372/395
Eukaryota	Fungi	Talaromyces stipitatus ATCC 10500	XP_002484384.1	2 E-63	372/395
Eukaryota	Fungi	Gibberella zeae PH-1	XP_381071.1	2 E-63	387/395
Eukaryota	Fungi	Aspergillus terreus NIH2624	XP_001218425.1	3 E-62	351/395
Eukaryota	Fungi	Coprinopsis cinerea okayama7#130	XP_001837617.1	6 E-61	341/395
Eukaryota	Fungi	Moniliophthora perniciosa FA553	XP_002388174.1	4 E-59	330/395
Eukaryota	Fungi	Coprinopsis cinerea okayama7#130	XP_001837398.1	5 E-59	347/395
Eukaryota	Fungi	Aspergillus nidulans FGSC A4	tpeCBF70720.1	4 E-57	344/395
Eukaryota	Fungi	Aspergillus nidulans FGSC A4	XP_663450.1	5 E-57	344/395
Eukaryota	Fungi	Penicillium marneffei ATCC 18224	XP_002149879.1	6 E-57	372/395
Eukaryota	Fungi	Penicillium marneffei ATCC 18224	XP_002149881.1	9 E-57	372/395
Eukaryota	Fungi	Phaeosphaeria nodorum SN15	XP_001791014.1	2 E-56	358/395
Eukaryota	Fungi	Penicillium marneffei ATCC 18224	XP_002149880.1	4 E-56	344/395
Eukaryota	Fungi	Aspergillus niger CBS 513.88	XP_001398623.1	6 E-56	350/395
Eukaryota	Fungi	Aspergillus oryzae RIB40	XP_001824539.1	1 E-55	342/395
Eukaryota	Fungi	Aspergillus flavus NRRL3357	XP_002384098.1	2 E-55	342/395
Eukaryota	Fungi	Coprinopsis cinerea okayama7#130	XP_001839067.1	5 E-55	317/395
Bacteria	Proteobacteria	Rhizobium leguminosarum bv. trifolii	YP_002976019.1	5 E-52	351/395
Bacteria	Proteobacteria	Sinorhizobium meliloti 1021	NP_437782.1	5 E-50	350/395
Eukaryota	Fungi	Aspergillus niger CBS 513.88	XP_001390478.1	5 E-50	342/395
Bacteria	Proteobacteria	Sinorhizobium medicae WSM419	YP_001313158.1	5 E-50	351/395
Bacteria	Proteobacteria	Rhizobium leguminosarum bv. viciae	YP_768253.1	1 E-49	351/395
Bacteria	Chloroflexi	Thermomicrobium roseum DSM 5159	YP_002523802.1	2 E-49	338/395
Bacteria	Proteobacteria	Rhizobium etli CFN 42	YP_469861.1	4 E-49	351/395
Bacteria	Proteobacteria	Rhizobium etli GR56	ZP_03522723.1	4 E-49	351/395
Bacteria	Chloroflexi	Sphaerobacter thermophilus DSM 20745	ZP_04495069.1	8 E-49	353/395
Bacteria	Proteobacteria	Mesorhizobium loti MAFF303099	NP_107295.1	1 E-48	350/395
Bacteria	Proteobacteria	Rhizobium etli CIAT 652	YP_001978582.1	2 E-48	351/395
Bacteria	Proteobacteria	Vibrio parahaemolyticus RIMD 2210633	NP_800145.1	2 E-48	351/395
Bacteria	Proteobacteria	Vibrio sp. Ex25	ZP_04922811.1	3 E-48	357/395
Bacteria	Acidobacteria	Candidatus Solibacter usitatus Ellin6076	YP_824467.1	6 E-48	344/395
Bacteria	Proteobacteria	Vibrio parahaemolyticus AQ3810	ZP_01990258.1	8 E-48	356/395
Bacteria	Proteobacteria	Vibrio parahaemolyticus AQ4037	ZP_05909637.1	1 E-47	350/395
Bacteria	Verrucomicrobia	Verrucomicrobiae bacterium DG1235	ZP_05058211.1	2 E-47	352/395
Bacteria	Proteobacteria	Chelativorans sp. BNC1	YP_675008.1	3 E-47	350/395
Bacteria	Proteobacteria	Burkholderia graminis C4D1M	ZP_02884111.1	3 E-47	348/395
Bacteria	Proteobacteria	Polaromonas sp. JS666	YP_548352.1	4 E-47	349/395
Bacteria	Proteobacteria	Rhizobium leguminosarum bv. trifolii	YP_002281502.1	4 E-47	351/395
Bacteria	Actinobacteria	Thermomonospora curvata DSM 43183	ZP_04033051.1	4 E-47	352/395
Bacteria	Proteobacteria	Rhizobium etli 8C-3	ZP_03513849.1	6 E-47	375/395
Bacteria	Proteobacteria	Polaromonas naphthalenivorans CJ2	YP_982990.1	2 E-46	349/395
Bacteria	Actinobacteria	Arthrobacter chlorophenolicus A6	YP_002487830.1	2 E-46	351/395
Eukaryota	Fungi	Nectria haematococca mpVI 77-13-4	EEU39622.1	1 E-45	355/395
Bacteria	Proteobacteria	Rhizobium sp. NGR234	YP_002826943.1	2 E-45	351/395
Bacteria	Proteobacteria	Burkholderia phymatum STM815	YP_001861489.1	2 E-45	348/395
Bacteria	Proteobacteria	Anaeromyxobacter sp. Fw109-5	YP_001378794.1	2 E-45	344/395
Bacteria	Proteobacteria	Silicibacter lacuscaerulensis ITI-1157	ZP_05784878.1	3 E-45	355/395
Bacteria	Proteobacteria	Burkholderia sp. H160	ZP_03271025.1	4 E-45	349/395
Bacteria	Proteobacteria	Cupriavidus taiwanensis	YP_002005005.1	4 E-45	339/395
Bacteria	Proteobacteria	Roseobacter sp. GAI101	ZP_05102306.1	3 E-44	353/395
Bacteria	Proteobacteria	Shewanella loihica PV-4	YP_001093614.1	1 E-43	353/395
Bacteria	Proteobacteria	Ralstonia eutropha H16	YP_725507.1	1 E-43	342/395

Eukaryota	Fungi	Gibberella zeae PH-1	XP_382809.1	5 E-43	351/395
Bacteria	Proteobacteria	Rhodoferax ferrireducens T118	YP_523584.1	1 E-42	348/395
Bacteria	Firmicutes	Bacillus sp. SG-1	ZP_01859978.1	3 E-42	333/395
Bacteria	Actinobacteria	Geodermatophilus obscurus DSM 43160	ZP_03889897.1	6 E-42	352/395
Bacteria	Proteobacteria	Roseovarius nubinhibens ISM	ZP_00961244.1	7 E-42	349/395
Bacteria	Actinobacteria	Frankia sp. CcI3	YP_480196.1	8 E-42	353/395
Bacteria	Proteobacteria	Burkholderia phytofirmans PsJN	YP_001888200.1	4 E-41	346/395
Bacteria	Proteobacteria	Aromatoleum aromaticum EbN1	YP_160879.1	4 E-41	350/395
Eukaryota	Fungi	Aspergillus terreus NIH2624	XP_001215389.1	7 E-41	342/395
Bacteria	Proteobacteria	Ralstonia metallidurans CH34	YP_583923.1	3 E-40	345/395
Bacteria	Proteobacteria	Burkholderia xenovorans LB400	YP_554531.1	3 E-40	342/395
Archaea	Euryarchaeota	Halogeometricum borinquense DSM 11551	ZP_04001054.1	4 E-40	371/395
Bacteria	Proteobacteria	Photobacterium profundum 3TCK	ZP_01219354.1	5 E-40	353/395
Bacteria	Proteobacteria	Ralstonia metallidurans CH34	YP_585610.1	4 E-39	350/395
Bacteria	Actinobacteria	Nocardioides sp. JS614	YP_919354.1	7 E-39	351/395
Bacteria	Actinobacteria	Streptomyces maritimus	AAF81732.1	8 E-39	352/395
Bacteria	Actinobacteria	Geodermatophilus obscurus DSM 43160	ZP_03888778.1	2 E-38	341/395
Bacteria	Verrucomicrobia	bacterium Ellin514	ZP_03631275.1	3 E-38	333/395
Archaea	Euryarchaeota	Natrialba magadii ATCC 43099	ZP_03696126.1	2 E-37	369/395
Bacteria	Actinobacteria	Streptomyces roseosporus NRRL 15998	ZP_04694861.1	3 E-37	322/395
Eukaryota	Fungi	Phaeosphaeria nodorum SN15	XP_001795574.1	3 E-37	366/395
Eukaryota	Fungi	Nectria haematococca mpVI 77-13-4	EEU37530.1	3 E-36	330/395
Bacteria	Cyanobacteria	Cyanothece sp. PCC 7425	YP_002482592.1	9 E-36	322/395
Bacteria	Actinobacteria	Rhodococcus erythropolis PR4	YP_002765398.1	2 E-35	371/395
Archaea	Euryarchaeota	Halogeometricum borinquense DSM 11551	ZP_04000219.1	2 E-35	327/395
Bacteria	Actinobacteria	Rhodococcus erythropolis SK121	ZP_04383375.1	4 E-35	371/395
Bacteria	Actinobacteria	Saccharopolyspora erythraea NRRL 2338	YP_001109355.1	5 E-35	350/395
Bacteria	Actinobacteria	Rubrobacter xylanophilus DSM 9941	YP_643212.1	9 E-35	356/395
Eukaryota	Fungi	Postia placenta Mad-698-R	XP_002469800.1	4 E-34	377/395
Bacteria	Actinobacteria	Thermomonospora curvata DSM 43183	ZP_04032796.1	5 E-34	350/395
Bacteria	Proteobacteria	Pseudoalteromonas atlantica T6c	YP_661379.1	1 E-33	349/395
Eukaryota	Fungi	Neurospora crassa OR74A	XP_965608.1	2 E-33	399/395
Bacteria	Actinobacteria	Kribbella flavida DSM 17836	ZP_03861746.1	2 E-33	334/395
Bacteria	Proteobacteria	Rhodobacteraceae bacterium KLH11	ZP_05124703.1	4 E-33	368/395
Eukaryota	Fungi	Pyrenophora tritici-repentis Pt-1C-BFP	XP_001932221.1	5 E-33	328/395
Eukaryota	Fungi	Sordaria macrospora	CAQ58437.1	6 E-33	380/395
Bacteria	Proteobacteria	Octadecabacter antarcticus 238	ZP_05067354.1	1 E-32	348/395
Eukaryota	Fungi	Neurospora crassa OR74A	XP_001728023.1	3 E-32	387/395
Bacteria	Chloroflexi	Sphaerobacter thermophilus DSM 20745	ZP_04497488.1	3 E-32	320/395
Bacteria	Actinobacteria	Beutenbergia cavernae DSM 12333	YP_002880099.1	3 E-32	336/395
Eukaryota	Fungi	Magnaporthe grisea 70-15	XP_361884.1	4 E-32	342/395
Eukaryota	Fungi	Sclerotinia sclerotiorum 1980 UF-70	XP_001592758.1	3 E-31	330/395
Bacteria	Chloroflexi	Sphaerobacter thermophilus DSM 20745	ZP_04494680.1	3 E-31	346/395
Bacteria	Actinobacteria	Streptomyces sp. C	ZP_05505778.1	3 E-31	364/395
Eukaryota	Fungi	Aspergillus flavus NRRL3357	XP_002382116.1	7 E-31	371/395
Eukaryota	Fungi	Pyrenophora tritici-repentis Pt-1C-BFP	XP_001937757.1	8 E-31	381/395
Eukaryota	Fungi	Aspergillus oryzae RIB40	XP_001819119.1	1 E-30	371/395
Archaea	Euryarchaeota	Haloquadratum walsbyi DSM 16790	YP_658062.1	2 E-30	353/395
Bacteria	Firmicutes	Bacillus sp. B14905	ZP_01725273.1	3 E-30	366/395
Bacteria	Actinobacteria	Brachybacterium faecium DSM 4810	YP_003153937.1	4 E-30	366/395
Eukaryota	Fungi	Phaeosphaeria nodorum SN15	XP_001798371.1	4 E-30	384/395
Eukaryota	Fungi	Podospora anserina DSM 980	XP_001905462.1	1 E-29	381/395
Bacteria	Actinobacteria	Rubrobacter xylanophilus DSM 9941	YP_645806.1	1 E-29	318/395
Bacteria	Firmicutes	Bacillus cereus G9842;	YP_002446690.1	1 E-29	327/395
Bacteria	Firmicutes	Bacillus thuringiensis serovar israelensis	ZP_00743322.1	1 E-29	327/395
Eukaryota	Fungi	Aspergillus niger CBS 513.88	XP_001390005.1	2 E-29	351/395
Bacteria	Firmicutes	Bacillus thuringiensis IBL 4222	ZP_04065888.1	2 E-29	327/395
Eukaryota	Fungi	Penicillium chrysogenum Wisconsin 54-1255	XP_002564777.1	3 E-29	345/395

Bacteria	Actinobacteria	Rhodococcus jostii RHA1	YP_701527.1	3 E-29	325/395
Bacteria	Actinobacteria	Rhodococcus opacus B4	YP_002778450.1	6 E-29	332/395
Eukaryota	Fungi	Microsporum canis CBS 113480	EEQ27891.1	7 E-29	338/395
Bacteria	Actinobacteria	Kribbella flavida DSM 17836	ZP_03865197.1	8 E-29	319/395
Eukaryota	Fungi	Pyrenophora tritici-repentis Pt-1C-BFP	XP_001934478.1	2 E-28	391/395
Bacteria	Actinobacteria	Streptomyces sp. Mg1	ZP_05001981.1	2 E-28	349/395
Eukaryota	Fungi	Magnaporthe grisea 70-15	XP_366839.1	2 E-28	390/395
Bacteria	Firmicutes	Lysinibacillus sphaericus C3-41	YP_001698653.1	2 E-28	364/395
Bacteria	Actinobacteria	Streptomyces ghanaensis ATCC 14672	ZP_04683913.1	3 E-28	349/395
Eukaryota	Fungi	Microsporum canis CBS 113480	EEQ31208.1	7 E-28	338/395
Eukaryota	Fungi	Microsporum canis CBS 113480	EEQ35851.1	1 E-27	316/395
Eukaryota	Fungi	Aspergillus terreus NIH2624	XP_001212611.1	1 E-27	332/395
Eukaryota	Fungi	Talaromyces stipitatus ATCC 10500	XP_002479463.1	2 E-27	325/395
Bacteria	Actinobacteria	Sanguibacter keddieii DSM 10542	ZP_05818428.1	3 E-27	320/395
Bacteria	Actinobacteria	Streptomyces griseoflavus Tu4000	ZP_05536873.1	3 E-27	349/395
Eukaryota	Fungi	Coccidioides posadasii C735 delta	EER29452.1	3 E-27	382/395
Eukaryota	Fungi	Coccidioides immitis RS;	XP_001245245.1	4 E-27	382/395
Bacteria	Actinobacteria	Nocardiopsis dassonvillei subsp. dassonvillei	ZP_04332581.1	7 E-27	367/395
Eukaryota	Fungi	Ajellomyces capsulatus H143	EER43723.1	1 E-26	385/395
Bacteria	Actinobacteria	Cellulomonas flavigena DSM 20109	ZP_04367705.1	2 E-26	319/395
Eukaryota	Fungi	Sclerotinia sclerotiorum 1980 UF-70	XP_001585313.1	3 E-26	331/395
Eukaryota	Fungi	Magnaporthe grisea 70-15	XP_001523041.1	3 E-26	377/395
Eukaryota	Amoebozoa	Dictyostelium discoideum AX4	XP_646382.1	4 E-26	381/395
Eukaryota	Fungi	Ajellomyces capsulatus G186AR	EEH05600.1	5 E-26	385/395
Bacteria	Actinobacteria	Catenulispora acidiphila DSM 44928	YP_003111898.1	7 E-26	318/395
Eukaryota	Fungi	Gibberella zeae PH-1	XP_384074.1	1 E-25	389/395
Bacteria	Proteobacteria	Methylobacterium nodulans ORS 2060	YP_002499140.1	1 E-25	346/395

AFUA_2G00740

Eukaryota	Fungi	Aspergillus fumigatus Af293	XP_749211.1	0.0	337/337
Eukaryota	Fungi	Neosartorya fischeri NRRL 181	XP_001265632.1	1 E-124	310/337
Eukaryota	Fungi	Aspergillus clavatus NRRL 1	XP_001273142.1	6 E-76	305/337
Eukaryota	Fungi	Magnaporthe grisea 70-15	XP_361732.1	1 E-14	293/337

AFUA_2G00750

Eukaryota	Fungi	Aspergillus fumigatus Af293	XP_749212.1	0.0	364/364
Eukaryota	Fungi	Neosartorya fischeri NRRL 181	XP_001265633.1	0.0	347/364
Eukaryota	Fungi	Aspergillus clavatus NRRL 1	XP_001273143.1	1 E-180	347/364
Eukaryota	Fungi	Aspergillus terreus NIH2624	XP_001216743.1	1 E-173	347/364
Eukaryota	Fungi	Aspergillus flavus NRRL3357	XP_002378520.1	1 E-170	348/364
Eukaryota	Fungi	Aspergillus nidulans FGSC A4	XP_660272.1	1 E-168	353/364
Eukaryota	Fungi	Penicillium chrysogenum Wisconsin 54-1255	XP_002566376.1	1 E-158	343/364
Eukaryota	Fungi	Nectria haematococca mpVI 77-13-4	EEU42949.1	1 E-128	346/364
Eukaryota	Fungi	Talaromyces stipitatus ATCC 10500	XP_002478001.1	1 E-125	342/364
Eukaryota	Fungi	Aspergillus niger CBS 513.88	XP_001402477.1	1 E-117	317/364
Eukaryota	Fungi	Yarrowia lipolytica CLIB122	XP_503422.1	2 E-92	338/364
Eukaryota	Fungi	Podospora anserina DSM 980	XP_001903256.1	2 E-74	358/364
Eukaryota	Fungi	Verticillium albo-atrum VaMs.102	EEY23668.1	1 E-73	346/364
Eukaryota	Fungi	Coccidioides posadasii C735 delta	EER28514.1	4 E-72	348/364
Eukaryota	Fungi	Paracoccidioides brasiliensis Pb03;	EEH21417.1	5 E-71	352/364
Eukaryota	Fungi	Paracoccidioides brasiliensis Pb18;	EEH43911.1	8 E-70	351/364
Eukaryota	Fungi	Ajellomyces dermatitidis ER-3	EEQ86172.1	2 E-68	357/364
Eukaryota	Fungi	Ajellomyces dermatitidis SLH14081	XP_002620907.1	4 E-68	357/364
Eukaryota	Fungi	Coccidioides immitis RS;	XP_001241709.1	6 E-68	338/364
Eukaryota	Fungi	Paracoccidioides brasiliensis Pb01;	EEH39116.1	9 E-68	339/364
Eukaryota	Fungi	Ajellomyces capsulatus H143	EER43357.1	2 E-67	358/364

Eukaryota	Fungi	Nectria haematococca mpVI 77-13-4	EEU43834.1	2 E-65	341/364
Eukaryota	Fungi	Aspergillus nidulans FGSC A4	tpeCBF87050.1	9 E-65	357/364
Eukaryota	Fungi	Sclerotinia sclerotiorum 1980 UF-70	XP_001585007.1	1 E-64	359/364
Eukaryota	Fungi	Aspergillus oryzae RIB40	XP_001823315.1	1 E-64	363/364
Eukaryota	Fungi	Ajellomyces capsulatus G186AR	EEH03163.1	4 E-63	356/364
Eukaryota	Fungi	Aspergillus nidulans FGSC A4	XP_660140.1	7 E-63	349/364
Eukaryota	Fungi	Aspergillus terreus NIH2624	XP_001212704.1	7 E-63	350/364
Eukaryota	Fungi	Aspergillus fumigatus Af293	XP_754137.1	9 E-63	350/364
Eukaryota	Fungi	Ajellomyces capsulatus G186AR	EEH11311.1	1 E-62	393/364
Eukaryota	Fungi	Coccidioides posadasii C735 delta	EER25572.1	6 E-62	355/364
Eukaryota	Fungi	Microsporum canis CBS 113480	EEQ35524.1	7 E-62	349/364
Eukaryota	Fungi	Neosartorya fischeri NRRL 181	XP_001262973.1	1 E-61	350/364
Eukaryota	Fungi	Paracoccidioides brasiliensis Pb01;	EEH41019.1	2 E-61	352/364
Eukaryota	Fungi	Paracoccidioides brasiliensis Pb03;	EEH16915.1	4 E-61	352/364
Eukaryota	Fungi	Paracoccidioides brasiliensis Pb18;	EEH50439.1	7 E-61	352/364
Eukaryota	Fungi	Magnaporthe grisea 70-15	XP_363297.1	1 E-60	360/364
Eukaryota	Fungi	Coprinopsis cinerea okayama7#130	XP_001830929.1	4 E-60	347/364
Eukaryota	Fungi	Gibberella zeae PH-1	XP_381069.1	8 E-60	355/364
Eukaryota	Fungi	Podospora anserina DSM 980	XP_001912450.1	8 E-60	364/364
Eukaryota	Fungi	Penicillium marneffei ATCC 18224	XP_002152172.1	2 E-59	351/364
Eukaryota	Fungi	Laccaria bicolor S238N-H82	XP_001888922.1	7 E-59	361/364
Eukaryota	Fungi	Penicillium chrysogenum Wisconsin 54-1255	XP_002559982.1	8 E-59	356/364
Eukaryota	Fungi	Talaromyces stipitatus ATCC 10500	XP_002481206.1	9 E-59	351/364
Eukaryota	Fungi	Sclerotinia sclerotiorum 1980 UF-70	XP_001592931.1	3 E-58	355/364
Eukaryota	Fungi	Clavispora lusitaniae ATCC 42720	XP_002618603.1	5 E-58	370/364
Eukaryota	Fungi	Aspergillus niger CBS 513.88	XP_001393435.1	8 E-58	334/364
Eukaryota	Fungi	Aspergillus clavatus NRRL 1	XP_001271423.1	1 E-57	350/364
Eukaryota	Fungi	Nectria haematococca mpVI 77-13-4	EEU48766.1	3 E-57	355/364
Eukaryota	Fungi	Pichia stipitis CBS 6054	XP_001384889.2	3 E-56	364/364
Eukaryota	Fungi	Botryotinia fuckeliana B05.10	XP_001560083.1	9 E-56	355/364
Eukaryota	Fungi	Phaeosphaeria nodorum SN15	XP_001801829.1	1 E-54	356/364
Eukaryota	Fungi	Ajellomyces capsulatus NAm1	XP_001541452.1	2 E-54	358/364
Eukaryota	Fungi	Aspergillus oryzae RIB40	XP_001826817.1	6 E-54	362/364
Eukaryota	Fungi	Lodderomyces elongisporus NRRL YB-4239	XP_001527076.1	2 E-53	366/364
Eukaryota	Fungi	Candida tropicalis MYA-3404	XP_002545632.1	6 E-53	368/364
Eukaryota	Fungi	Pyrenophora tritici-repentis Pt-1C-BFP	XP_001934803.1	7 E-53	363/364
Bacteria	Proteobacteria	Methylocella silvestris BL2	YP_002361004.1	1 E-52	326/364
Eukaryota	Fungi	Zygosaccharomyces rouxii CBS 732	XP_002498964.1	2 E-52	361/364
Bacteria	Proteobacteria	Klebsiella pneumoniae subsp. rhinoscleromatis	EEW39581.1	4 E-52	339/364
Bacteria	Proteobacteria	Klebsiella pneumoniae subsp. pneumoniae	YP_001335316.1	7 E-52	339/364
Bacteria	Proteobacteria	Klebsiella pneumoniae 342	YP_002238550.1	2 E-51	339/364
Eukaryota	Fungi	Neurospora crassa OR74A	XP_965006.2	3 E-51	336/364
Eukaryota	Fungi	Candida dubliniensis CD36	XP_002422010.1	5 E-51	361/364
Eukaryota	Fungi	Debaryomyces hansenii	CAG87997.2	6 E-51	361/364
Eukaryota	Fungi	Candida albicans WO-1	EEQ43547.1	7 E-51	361/364
Eukaryota	Fungi	Debaryomyces hansenii CBS767	XP_459758.1	8 E-51	361/364
Eukaryota	Fungi	Candida albicans SC5314	XP_717496.1	6 E-50	361/364
Bacteria	Proteobacteria	Cronobacter turicensis	YP_003209425.1	1 E-49	326/364
Bacteria	Actinobacteria	Rhodococcus jostii RHA1	YP_701764.1	1 E-49	325/364
Eukaryota	Fungi	Gibberella zeae PH-1	XP_380224.1	1 E-49	361/364
Eukaryota	Fungi	Chaetomium globosum CBS 148.51	XP_001220920.1	1 E-49	337/364
Bacteria	Proteobacteria	Pseudomonas fluorescens Pf-5	YP_260295.1	4 E-49	331/364
Bacteria	Actinobacteria	Rhodococcus erythropolis PR4	YP_002769293.1	9 E-49	335/364
Bacteria	Actinobacteria	Rhodococcus erythropolis SK121	ZP_04388489.1	1 E-48	335/364
Bacteria	Proteobacteria	Dickeya dadantii Ech586	ZP_05724683.1	2 E-48	336/364
Bacteria	Proteobacteria	Pseudomonas fluorescens Pf0-1	YP_348066.1	3 E-48	330/364
Bacteria	Proteobacteria	Cronobacter sakazakii ATCC BAA-894	YP_001438882.1	8 E-48	327/364
Eukaryota	Fungi	Lachancea thermotolerans CBS 6340	XP_002553503.1	2 E-47	364/364

Eukaryota	Fungi	Aspergillus flavus NRRL3357	XP_002385244.1	4 E-47	296/364
Bacteria	Proteobacteria	Dickeya dadantii Ech703	YP_002986964.1	4 E-47	342/364
Bacteria	Actinobacteria	Rhodococcus opacus B4	YP_002778659.1	4 E-47	325/364
Bacteria	Proteobacteria	Yersinia aldovae ATCC 35236	ZP_04619121.1	5 E-47	333/364
Bacteria	Proteobacteria	Yersinia frederiksenii ATCC 33641	ZP_04633463.1	5 E-47	314/364
Bacteria	Proteobacteria	Yersinia ruckeri ATCC 29473	ZP_04614794.1	1 E-46	328/364
Bacteria	Proteobacteria	Pantoea sp. At-9b	ZP_05730050.1	5 E-46	329/364
Eukaryota	Fungi	Ajellomyces dermatitidis ER-3	EEQ91399.1	5 E-46	312/364
Bacteria	Proteobacteria	Yersinia rohdei ATCC 43380	ZP_04613314.1	9 E-46	329/364
Bacteria	Proteobacteria	Dickeya zeae Ech1591	YP_003004944.1	1 E-45	342/364
Bacteria	Proteobacteria	Campylobacter jejuni subsp. doylei	YP_001397708.1	2 E-45	327/364
Eukaryota	Fungi	Ajellomyces dermatitidis SLH14081	XP_002624327.1	3 E-45	312/364
Bacteria	Proteobacteria	Serratia proteamaculans 568	YP_001477857.1	7 E-45	331/364
Eukaryota	Fungi	Debaryomyces hansenii	CAG86660.2	1 E-44	366/364
Bacteria	Proteobacteria	Yersinia bercovieri ATCC 43970	ZP_04628914.1	2 E-44	313/364
Bacteria	Proteobacteria	Campylobacter coli RM2228	ZP_00367217.1	2 E-44	327/364
Bacteria	Proteobacteria	Rhodopseudomonas palustris TIE-1	YP_001991590.1	2 E-44	334/364
Bacteria	Actinobacteria	Streptomyces hygroscopicus subsp. yingchenge	AAP21658.1	3 E-44	338/364
Bacteria	Proteobacteria	Rhodopseudomonas palustris CGA009	NP_947703.1	3 E-44	334/364
Bacteria	Proteobacteria	Delftia acidovorans SPH-1	YP_001566629.1	3 E-44	328/364
Bacteria	Actinobacteria	Streptomyces sp. C	ZP_05506900.1	7 E-44	322/364
Eukaryota	Fungi	Debaryomyces hansenii CBS767	XP_458528.1	7 E-44	366/364
Bacteria	Actinobacteria	Streptomyces sp. Mg1	ZP_05001913.1	7 E-44	333/364
Bacteria	Proteobacteria	Yersinia intermedia ATCC 29909	ZP_04638468.1	1 E-43	313/364
Bacteria	Proteobacteria	Yersinia pestis biovar Microtus	NP_992642.1	1 E-43	314/364
Bacteria	Proteobacteria	Yersinia kristensenii ATCC 33638	ZP_04623011.1	1 E-43	315/364
Bacteria	Proteobacteria	Yersinia pseudotuberculosis IP 32953	YP_069879.1	1 E-43	314/364
Bacteria	Proteobacteria	Yersinia pseudotuberculosis IP 31758	YP_001401628.1	1 E-43	314/364
Bacteria	Proteobacteria	Yersinia pseudotuberculosis YPIII	YP_001721474.1	1 E-43	314/364
Bacteria	Proteobacteria	Methylobacterium extorquens DM4	YP_003069601.1	1 E-43	329/364
Bacteria	Proteobacteria	Bradyrhizobium sp. BTAi1	YP_001238574.1	1 E-43	335/364
Bacteria	Proteobacteria	Agrobacterium vitis S4	YP_002539578.1	2 E-43	331/364
Bacteria	Proteobacteria	Mesorhizobium opportunistum WSM2075	ZP_05810853.1	3 E-43	329/364
Bacteria	Proteobacteria	Mesorhizobium loti MAFF303099	NP_107308.1	6 E-43	333/364
Bacteria	Proteobacteria	Yersinia pestis KIM	NP_670168.1	7 E-43	305/364
Bacteria	Proteobacteria	Methylobacterium extorquens PA1	YP_001640789.1	1 E-42	329/364
Eukaryota	Fungi	Pichia pastoris GS115	XP_002492589.1	1 E-42	380/364
Bacteria	Proteobacteria	Yersinia mollaretii ATCC 43969	ZP_04639297.1	2 E-42	312/364
Bacteria	Proteobacteria	Yersinia enterocolitica subsp. enterocolitica	YP_001005772.1	2 E-42	325/364
Bacteria	Proteobacteria	Rhodospirillum rubrum ATCC 11170	YP_425866.1	2 E-42	314/364
Bacteria	Proteobacteria	Methylobacterium chloromethanicum CM4	YP_002422403.1	4 E-42	329/364
Eukaryota	Fungi	Talaromyces stipitatus ATCC 10500	XP_002481627.1	5 E-42	335/364
Bacteria	Proteobacteria	Methylobacterium extorquens AM1	YP_002964559.1	6 E-42	329/364
Bacteria	Actinobacteria	Mycobacterium smegmatis str. MC2	YP_884827.1	8 E-42	312/364
Bacteria	Actinobacteria	Mycobacterium intracellulare ATCC 13950	ZP_05225427.1	2 E-41	316/364
Bacteria	Proteobacteria	Ochrobactrum anthropi ATCC 49188	YP_001372324.1	2 E-41	329/364
Bacteria	Proteobacteria	Aeromonas salmonicida subsp. salmonicida	YP_001142143.1	1 E-40	328/364
Eukaryota	Fungi	Verticillium albo-atrum VaMs.102	EEY16857.1	2 E-40	353/364
Eukaryota	Viridiplantae	Ostreococcus lucimarinus CCE9901	XP_001415530.1	3 E-40	334/364
Bacteria	Proteobacteria	Campylobacter jejuni subsp. jejuni	ZP_01071207.1	3 E-40	327/364
Bacteria	Proteobacteria	Providencia rettgeri DSM 1131	ZP_03641427.1	6 E-40	334/364
Bacteria	Proteobacteria	Campylobacter jejuni subsp. jejuni	ZP_01068962.1	6 E-40	327/364
Bacteria	Proteobacteria	Campylobacter jejuni subsp. jejuni	ZP_01068632.1	3 E-39	327/364
Bacteria	Proteobacteria	Campylobacter jejuni RM1221	YP_179320.1	4 E-39	327/364
Bacteria	Proteobacteria	Campylobacter jejuni subsp. jejuni	YP_001000874.1	4 E-39	327/364
Bacteria	Actinobacteria	Catenulispora acidiphila DSM 44928	YP_003113918.1	5 E-39	327/364
Bacteria	Actinobacteria	Tsukamurella paurometabola DSM 20162	ZP_04028936.1	8 E-39	322/364
Bacteria	Proteobacteria	Campylobacter jejuni subsp. jejuni	ZP_01099539.1	9 E-39	327/364

Eukaryota	Viridiplantae	Micromonas sp. RCC299	XP_002504314.1	9 E-39	306/364
Bacteria	Actinobacteria	Mycobacterium abscessus	YP_001705158.1	2 E-38	311/364
Bacteria	Actinobacteria	marine actinobacterium PHSC20C1	ZP_01131310.1	2 E-38	335/364
Bacteria	Actinobacteria	Arthrobacter sp. KW	AAZ38135.1	3 E-38	339/364
Bacteria	Proteobacteria	Campylobacter jejuni subsp. jejuni	ZP_03222988.1	3 E-38	327/364
Bacteria	Actinobacteria	Mycobacterium sp. MCS	YP_640653.1	1 E-37	315/364
Bacteria	Actinobacteria	Mycobacterium sp. JLS	YP_001071771.1	2 E-37	315/364
Bacteria	Actinobacteria	Gordonia bronchialis DSM 43247	ZP_03885577.1	2 E-37	323/364
Bacteria	Actinobacteria	Mycobacterium avium subsp. paratuberculosis	NP_962629.1	6 E-37	302/364
Eukaryota	Viridiplantae	Physcomitrella patens subsp. patens	XP_001782343.1	1 E-36	352/364
Bacteria	Actinobacteria	Mycobacterium avium subsp. avium	ZP_05218868.1	1 E-36	320/364
Bacteria	Actinobacteria	Mycobacterium avium 104;	YP_884034.1	2 E-36	320/364
Bacteria	Proteobacteria	Shewanella sp. W3-18-1	YP_964811.1	2 E-35	324/364
Bacteria	Proteobacteria	Aeromonas hydrophila subsp. hydrophila	YP_856479.1	2 E-35	328/364
Bacteria	Actinobacteria	Brevibacterium linens BL2	ZP_05913703.1	3 E-35	322/364
Bacteria	Proteobacteria	Shewanella baltica OS223	YP_002356275.1	3 E-35	310/364
Bacteria	Actinobacteria	Frankia alni ACN14a	YP_715025.1	5 E-35	358/364
Bacteria	Actinobacteria	Arthrobacter chlorophenolicus A6	YP_002486758.1	7 E-35	339/364
Bacteria	Proteobacteria	Vibrio metschnikovii CIP 69.14	ZP_05882802.1	9 E-35	312/364
Bacteria	Actinobacteria	Streptomyces avermitilis MA-4680	NP_825037.1	2 E-34	329/364
Bacteria	Proteobacteria	Shewanella putrefaciens 200	ZP_01706711.1	4 E-34	324/364
Bacteria	Proteobacteria	Shewanella baltica OS155	YP_001048713.1	4 E-33	310/364
Bacteria	Actinobacteria	Streptomyces scabiei 87.22	CBG72177.1	5 E-33	326/364
Bacteria	Proteobacteria	Shewanella baltica OS195	YP_001552756.1	1 E-31	310/364
Bacteria	Proteobacteria	Idiomarina baltica OS145	ZP_01041965.1	6 E-31	318/364
Eukaryota	Viridiplantae	Glycine max	ACU18564.1	8 E-29	301/364
Bacteria	Proteobacteria	Idiomarina loihiensis L2TR	YP_154555.1	1 E-28	325/364
Bacteria	Actinobacteria	Frankia sp. EAN1pec	YP_001508546.1	1 E-28	380/364
Eukaryota	Fungi	Aspergillus niger CBS 513.88	XP_001397253.1	8 E-26	304/364
Bacteria	Actinobacteria	Mycobacterium sp. MCS	YP_639015.1	1 E-24	322/364
Eukaryota	Fungi	Sclerotinia sclerotiorum 1980 UF-70	XP_001588037.1	1 E-24	304/364
Bacteria	Proteobacteria	Burkholderia xenovorans LB400	YP_553500.1	2 E-23	319/364
Eukaryota	Fungi	Botryotinia fuckeliana B05.10	XP_001556004.1	2 E-23	301/364
Eukaryota	Fungi	Aspergillus clavatus NRRL 1	XP_001273700.1	2 E-23	294/364
Bacteria	Proteobacteria	Pseudomonas syringae pv. syringae	YP_233770.1	4 E-23	316/364
Eukaryota	Fungi	Neosartorya fischeri NRRL 181	XP_001258345.1	5 E-23	300/364
Eukaryota	Fungi	Penicillium marneffei ATCC 18224	XP_002143706.1	2 E-22	296/364

AFUA_2G04480

Eukaryota	Fungi	Aspergillus fumigatus Af293	XP_749579.1	0.0	372/372
Eukaryota	Fungi	Neosartorya fischeri NRRL 181	XP_001260077.1	1 E-174	326/372
Eukaryota	Fungi	Aspergillus clavatus NRRL 1	XP_001272742.1	1 E-140	350/372
Eukaryota	Fungi	Aspergillus nidulans FGSC A4	tpeCBF85727.1	1 E-136	341/372
Eukaryota	Fungi	Aspergillus terreus NIH2624	XP_001215237.1	1 E-133	316/372
Eukaryota	Fungi	Aspergillus flavus NRRL3357	XP_002375090.1	1 E-130	330/372
Eukaryota	Fungi	Aspergillus niger	ACF75334.1	1 E-128	317/372
Eukaryota	Fungi	Aspergillus niger CBS 513.88	XP_001394314.1	1 E-127	317/372
Eukaryota	Fungi	Ajellomyces dermatitidis ER-3	EEQ86113.1	2 E-96	310/372
Eukaryota	Fungi	Ajellomyces dermatitidis SLH14081	XP_002620509.1	4 E-96	309/372
Eukaryota	Fungi	Talaromyces stipitatus ATCC 10500	XP_002479001.1	5 E-96	315/372
Eukaryota	Fungi	Ajellomyces capsulatus G186AR	EEH03428.1	2 E-93	300/372
Bacteria	Bacteroidetes	Bacteroides fragilis 3_1_12	ZP_05280126.1	3 E-78	299/372
Bacteria	Bacteroidetes	Parabacteroides merdae ATCC 43184	ZP_02031981.1	7 E-78	326/372
Bacteria	Firmicutes	Paenibacillus sp. oral taxon	ZP_04852919.1	1 E-75	300/372
Bacteria	Bacteroidetes	Bacteroides ovatus ATCC 8483	ZP_02067600.1	4 E-75	327/372
Bacteria	Bacteroidetes	Bacteroides sp. 2_1_7	ZP_05285457.1	4 E-75	327/372
Bacteria	Bacteroidetes	Parabacteroides distasonis ATCC 8503	YP_001302720.1	7 E-75	327/372

Bacteria	Bacteroidetes	Parabacteroides sp. D13	ZP_05546357.1	7 E-75	327/372
Bacteria	Firmicutes	Paenibacillus sp. JDR-2	YP_003012914.1	8 E-75	305/372
Bacteria	Bacteroidetes	Bacteroides sp. 2_2_4	ZP_04550828.1	2 E-74	324/372
Bacteria	Bacteroidetes	Bacteroides ovatus ATCC 8483	ZP_02066340.1	3 E-74	313/372
Bacteria	Bacteroidetes	Bacteroides uniformis ATCC 8492	ZP_02071656.1	4 E-74	328/372
Bacteria	Bacteroidetes	Bacteroides stercoris ATCC 43183	ZP_02437481.1	5 E-74	328/372
Bacteria	Bacteroidetes	Bacteroides sp. D1	ZP_04547090.1	7 E-74	324/372
Bacteria	Bacteroidetes	Spirosoma linguale DSM 74	ZP_04493880.1	4 E-73	301/372
Bacteria	Bacteroidetes	Bacteroides sp. D2	ZP_05758766.1	5 E-73	313/372
Bacteria	Bacteroidetes	Bacteroides thetaiotaomicron VPI-5482	NP_812428.1	9 E-73	305/372
Bacteria	Bacteroidetes	Bacteroides sp. D1	ZP_04547835.1	5 E-72	340/372
Bacteria	Bacteroidetes	Leeuwenhoekiella blandensis MED217	ZP_01061955.1	6 E-70	300/372
Bacteria	Bacteroidetes	Chitinophaga pinensis DSM 2588	YP_003126332.1	2 E-69	299/372
Bacteria	Bacteroidetes	Bacteroides coprocola DSM 17136	ZP_03011577.1	3 E-69	319/372
Bacteria	Verrucomicrobia	bacterium Ellin514	ZP_03631860.1	2 E-68	308/372
Bacteria	Bacteroidetes	Bacteroides intestinalis DSM 17393	ZP_03016975.1	2 E-67	299/372
Bacteria	Bacteroidetes	Algoriphagus sp. PR1	ZP_01718741.1	6 E-63	310/372
Bacteria	Bacteroidetes	Pedobacter sp. BAL39	ZP_01884023.1	1 E-39	302/372
Bacteria	Bacteroidetes	Bacteroides thetaiotaomicron VPI-5482	NP_812586.1	3 E-38	309/372
Bacteria	Bacteroidetes	Bacteroides plebeius DSM 17135	ZP_03210041.1	7 E-35	306/372
Bacteria	Bacteroidetes	Bacteroides vulgatus ATCC 8482	YP_001298436.1	4 E-34	313/372
Bacteria	Bacteroidetes	Bacteroides sp. 4_3_47FAA	ZP_05253789.1	4 E-34	313/372
Bacteria	Bacteroidetes	Bacteroides sp. D4	ZP_04556652.1	5 E-34	313/372
Bacteria	Bacteroidetes	Leeuwenhoekiella blandensis MED217	ZP_01061223.1	1 E-33	340/372
Bacteria	Bacteroidetes	Bacteroides caccae ATCC 43185	ZP_01961597.1	2 E-33	307/372
Bacteria	Bacteroidetes	Bacteroides sp. 4_3_47FAA	ZP_05254941.1	3 E-33	298/372
Bacteria	Bacteroidetes	Bacteroides cellulosilyticus DSM 14838	ZP_03676533.1	1 E-32	304/372
Bacteria	Bacteroidetes	Bacteroides sp. 2_2_4	ZP_04552377.1	5 E-32	307/372
Bacteria	Bacteroidetes	Bacteroides sp. D2	ZP_05759376.1	9 E-32	306/372
Bacteria	Bacteroidetes	Bacteroides ovatus ATCC 8483	ZP_02065676.1	1 E-31	307/372
Bacteria	Firmicutes	Clostridium papyrosolvens DSM 2782	ZP_05496332.1	1 E-31	306/372
Bacteria	Bacteroidetes	Bacteroides intestinalis DSM 17393	ZP_03012795.1	3 E-31	302/372
Bacteria	Bacteroidetes	Bacteroides sp. 2_2_4	ZP_04550197.1	6 E-31	309/372
Bacteria	Bacteroidetes	Bacteroides sp. 4_3_47FAA	ZP_05255074.1	7 E-31	306/372
Bacteria	Bacteroidetes	Bacteroides vulgatus ATCC 8482	YP_001297829.1	8 E-31	306/372
Bacteria	Bacteroidetes	Flavobacterium johnsoniae UW101	YP_001195445.1	9 E-31	302/372
Bacteria	Bacteroidetes	Bacteroides sp. D4	ZP_04554585.1	1 E-30	335/372
Bacteria	Bacteroidetes	Bacteroides dorei DSM 17855	ZP_03299909.1	2 E-30	306/372
Bacteria	Bacteroidetes	Bacteroides intestinalis DSM 17393	ZP_03012973.1	4 E-30	316/372
Bacteria	Bacteroidetes	Bacteroides uniformis ATCC 8492	ZP_02071885.1	8 E-30	304/372
Bacteria	Bacteroidetes	Bacteroides cellulosilyticus DSM 14838	ZP_03677619.1	2 E-29	316/372
Bacteria	Bacteroidetes	Bacteroides cellulosilyticus DSM 14838	ZP_03678452.1	5 E-28	298/372
Eukaryota	Fungi	Pyrenophora tritici-repentis Pt-1C-BFP	XP_001941239.1	3 E-24	327/372
Bacteria	Proteobacteria	Sorangium cellulosum 'So ce	YP_001613757.1	3 E-24	298/372
Eukaryota	Fungi	Phaeosphaeria nodorum SN15	XP_001792127.1	2 E-23	339/372
Bacteria	Verrucomicrobia	Verrucomicrobiae bacterium DG1235	ZP_05056161.1	2 E-22	318/372
Bacteria	Bacteroidetes	Leeuwenhoekiella blandensis MED217	ZP_01060797.1	3 E-22	330/372
Bacteria	Firmicutes	Clostridium nexile DSM 1787	ZP_03288615.1	5 E-22	335/372
Eukaryota	Fungi	Magnaporthe grisea 70-15	XP_364253.1	2 E-21	310/372
Bacteria	Verrucomicrobia	Verrucomicrobiae bacterium DG1235	ZP_05055538.1	3 E-21	299/372
Bacteria	Bacteroidetes	Bacteroides eggerthii DSM 20697	ZP_03460707.1	4 E-21	304/372
Eukaryota	Fungi	Gibberella zeae PH-1	XP_383179.1	6 E-21	308/372
Bacteria	Bacteroidetes	Bacteroides ovatus ATCC 8483	ZP_02066439.1	1 E-20	299/372
Bacteria	Bacteroidetes	Bacteroides intestinalis DSM 17393	ZP_03013484.1	1 E-20	302/372
Bacteria	Bacteroidetes	Chryseobacterium gleum ATCC 35910	ZP_03852823.1	2 E-20	305/372
Bacteria	Bacteroidetes	Bacteroides intestinalis DSM 17393	ZP_03013394.1	2 E-20	298/372
Bacteria	Bacteroidetes	Bacteroides sp. D1	ZP_04545877.1	3 E-20	299/372
Bacteria	Bacteroidetes	Bacteroides cellulosilyticus DSM 14838	ZP_03677803.1	3 E-20	302/372

Bacteria	Actinobacteria	Bifidobacterium longum subsp. infantis	ZP_03976940.1	4 E-20	323/372
Bacteria	Proteobacteria	Shewanella sp. ANA-3	YP_869705.1	5 E-20	301/372
Bacteria	Proteobacteria	Shewanella sp. MR-4	YP_734112.1	6 E-20	301/372
Bacteria	Firmicutes	Geobacillus sp. Y412MC10	YP_003243347.1	6 E-20	326/372
Bacteria	Bacteroidetes	Bacteroides sp. 2_2_4	ZP_04550241.1	8 E-20	299/372
Bacteria	Actinobacteria	Bifidobacterium longum subsp. infantis	ZP_04664139.1	8 E-20	323/372
Bacteria	Bacteroidetes	Bacteroides sp. 2_2_4	ZP_04549019.1	9 E-20	309/372
Bacteria	Proteobacteria	Shewanella sp. MR-7	YP_738038.1	1 E-19	301/372
Bacteria	Bacteroidetes	Bacteroides eggerthii DSM 20697	ZP_03460708.1	2 E-19	299/372
Bacteria	Bacteroidetes	Bacteroides plebeius DSM 17135	ZP_03208331.1	2 E-19	303/372
Bacteria	Proteobacteria	Asticcacaulis excentricus CB 48	ZP_04769336.1	3 E-19	311/372
Bacteria	Bacteroidetes	Bacteroides sp. D2	ZP_05758655.1	8 E-19	312/372
Bacteria	Bacteroidetes	Bacteroides intestinalis DSM 17393	ZP_03013524.1	1 E-18	298/372
Bacteria	Bacteroidetes	Bacteroides sp. 4_3_47FAA	ZP_05254114.1	2 E-18	299/372
Bacteria	Bacteroidetes	Bacteroides vulgatus ATCC 8482	YP_001297384.1	2 E-18	299/372
Bacteria	Bacteroidetes	Bacteroides cellulosilyticus DSM 14838	ZP_03677737.1	4 E-18	306/372
Bacteria	Bacteroidetes	Bacteroides ovatus ATCC 8483	ZP_02064526.1	6 E-18	310/372
Bacteria	Firmicutes	Paenibacillus sp. JDR-2	YP_003012977.1	6 E-18	377/372
Bacteria	Bacteroidetes	Chryseobacterium gleum ATCC 35910	ZP_03852824.1	8 E-18	303/372
Bacteria	Actinobacteria	Bifidobacterium pseudocatenulatum DSM 2043[illegible]	ZP_03743585.1	4 E-17	326/372
Bacteria	Proteobacteria	Idiomarina loihiensis L2TR	YP_154474.1	2 E-16	304/372
Bacteria	Bacteroidetes	Bacteroides ovatus ATCC 8483	ZP_02063282.1	3 E-16	337/372
Eukaryota	Fungi	Neosartorya fischeri NRRL 181	XP_001261269.1	3 E-16	298/372
Bacteria	Firmicutes	Roseburia intestinalis L1-82	ZP_04743122.1	4 E-16	327/372
Bacteria	Actinobacteria	Bifidobacterium longum subsp. infantis	ZP_04665655.1	6 E-16	349/372
Bacteria	Actinobacteria	Bifidobacterium longum subsp. infantis	ZP_03975750.1	6 E-16	349/372
Eukaryota	Fungi	Aspergillus oryzae RIB40	XP_001827081.1	7 E-16	298/372
Eukaryota	Fungi	Aspergillus flavus NRRL3357	XP_002384310.1	7 E-16	298/372
Eukaryota	Fungi	Verticillium albo-atrum VaMs.102	EEY24093.1	8 E-16	322/372
Bacteria	Firmicutes	Geobacillus sp. Y412MC10	YP_003244786.1	1 E-15	313/372
Bacteria	Bacteroidetes	Bacteroides eggerthii DSM 20697	ZP_03459579.1	2 E-15	333/372
Eukaryota	Fungi	Podospora anserina DSM 980	XP_001905179.1	3 E-15	314/372
Bacteria	Bacteroidetes	Chitinophaga pinensis DSM 2588	YP_003122171.1	5 E-15	314/372
Bacteria	Bacteroidetes	Bacteroides caccae ATCC 43185	ZP_01961970.1	5 E-15	329/372
Bacteria	Bacteroidetes	Gramella forsetii KT0803	YP_860737.1	1 E-14	300/372
Bacteria	Bacteroidetes	Bacteroides cellulosilyticus DSM 14838	ZP_03676906.1	1 E-14	316/372
Bacteria	Bacteroidetes	Bacteroides ovatus ATCC 8483	ZP_02063312.1	1 E-14	306/372
Bacteria	Bacteroidetes	Bacteroides sp. 2_2_4	ZP_04549018.1	2 E-14	306/372
Bacteria	Firmicutes	Eubacterium siraeum DSM 15702	ZP_02423737.1	2 E-14	370/372
Bacteria	Bacteroidetes	Bacteroides sp. 2_1_7	ZP_05287753.1	2 E-14	357/372
Bacteria	Bacteroidetes	Spirosoma linguale DSM 74	ZP_04493931.1	2 E-14	324/372
Bacteria	Bacteroidetes	Bacteroides intestinalis DSM 17393	ZP_03013099.1	5 E-14	318/372
Bacteria	Dictyoglomi	Dictyoglomus thermophilum H-6-12	YP_002251602.1	5 E-14	322/372
Bacteria	Bacteroidetes	Parabacteroides distasonis ATCC 8503	YP_001303121.1	6 E-14	357/372

AFUA_2G04490

Eukaryota	Fungi	Aspergillus fumigatus Af293	XP_749580.1	0.0	635/635
Eukaryota	Fungi	Neosartorya fischeri NRRL 181	XP_001260078.1	0.0	582/635
Eukaryota	Fungi	Penicillium chrysogenum Wisconsin 54-1255	XP_002557876.1	0.0	588/635
Eukaryota	Fungi	Coccidioides posadasii C735 delta	EER23785.1	0.0	587/635
Eukaryota	Fungi	Coccidioides immitis RS;	XP_001247470.1	0.0	583/635
Eukaryota	Fungi	Ajellomyces capsulatus H143	EER41382.1	0.0	595/635
Eukaryota	Fungi	Ajellomyces capsulatus G186AR	EEH09552.1	0.0	595/635
Eukaryota	Fungi	Ajellomyces capsulatus NAm1	XP_001536069.1	0.0	595/635
Eukaryota	Fungi	Paracoccidioides brasiliensis Pb18;	EEH45017.1	0.0	596/635
Eukaryota	Fungi	Ajellomyces dermatitidis SLH14081	XP_002625466.1	0.0	591/635
Eukaryota	Fungi	Paracoccidioides brasiliensis Pb01;	EEH40061.1	0.0	594/635

Eukaryota	Fungi	Microsporum canis CBS 113480	EEQ35831.1	0.0	558/635
Eukaryota	Fungi	Phaeosphaeria nodorum SN15	XP_001803372.1	0.0	562/635
Eukaryota	Fungi	Uncinocarpus reesii 1704	XP_002541655.1	0.0	560/635
Eukaryota	Fungi	Pyrenophora tritici-repentis Pt-1C-BFP	XP_001937038.1	0.0	564/635
Eukaryota	Fungi	Magnaporthe grisea 70-15	XP_367282.1	1 E-176	554/635
Eukaryota	Fungi	Verticillium albo-atrum VaMs.102	EEY21990.1	1 E-166	540/635
Eukaryota	Fungi	Podospora anserina DSM 980	XP_001905881.1	1 E-163	571/635
Eukaryota	Fungi	Nectria haematococca mpVI 77-13-4	EEU40465.1	1 E-161	551/635
Eukaryota	Fungi	Chaetomium globosum CBS 148.51	XP_001226842.1	1 E-160	571/635
Eukaryota	Fungi	Gibberella zeae PH-1	XP_389659.1	1 E-156	551/635
Eukaryota	Viridiplantae	Picea sitchensis	ABK25346.1	1 E-106	525/635
Archaea	Euryarchaeota	Methanocorpusculum labreanum Z	YP_001030974.1	1 E-105	524/635
Eukaryota	Viridiplantae	Oryza sativa Indica Group	CAH68268.1	1 E-103	518/635
Eukaryota	Viridiplantae	Zea mays;	ACN36105.1	1 E-103	522/635
Eukaryota	Viridiplantae	Oryza sativa Japonica Group	EEE61811.1	1 E-103	518/635
Eukaryota	Viridiplantae	Oryza sativa Japonica Group	NP_001054094.1	1 E-103	518/635
Eukaryota	Viridiplantae	Sorghum bicolor;	XP_002447228.1	1 E-103	522/635
Eukaryota	Viridiplantae	Zea mays;	NP_001147127.1	1 E-102	522/635
Eukaryota	Viridiplantae	Populus trichocarpa	XP_002327177.1	1 E-102	527/635
Eukaryota	Viridiplantae	Sorghum bicolor;	XP_002445584.1	1 E-100	528/635
Eukaryota	Viridiplantae	Populus trichocarpa	XP_002301163.1	1 E-100	520/635
Eukaryota	Viridiplantae	Vitis vinifera	XP_002285358.1	1 E-100	528/635
Eukaryota	Viridiplantae	Vitis vinifera	CAO47860.1	1 E-100	528/635
Eukaryota	Viridiplantae	Zea mays;	ACR35566.1	1 E-100	513/635
Eukaryota	Viridiplantae	Populus trichocarpa	XP_002313870.1	1 E-100	523/635
Eukaryota	Viridiplantae	Ricinus communis	XP_002518687.1	1 E-100	529/635
Eukaryota	Viridiplantae	Populus trichocarpa	XP_002311914.1	1 E-99	523/635
Eukaryota	Viridiplantae	Zea mays;	ACF86477.1	1 E-99	538/635
Eukaryota	Viridiplantae	Zea mays;	NP_001147079.1	2 E-99	540/635
Eukaryota	Viridiplantae	Oryza sativa Indica Group	EAZ07169.1	5 E-99	526/635
Eukaryota	Viridiplantae	Oryza sativa Japonica Group	BAD09434.1	1 E-98	526/635
Eukaryota	Viridiplantae	Vitis vinifera	XP_002283022.1	1 E-98	534/635
Eukaryota	Viridiplantae	Arabidopsis thaliana	NP_195146.1	1 E-98	523/635
Eukaryota	Viridiplantae	Populus trichocarpa	XP_002316453.1	2 E-98	525/635
Eukaryota	Viridiplantae	Arabidopsis thaliana	AAM60833.1	4 E-98	523/635
Eukaryota	Viridiplantae	Populus trichocarpa	XP_002300235.1	4 E-98	529/635
Bacteria	Candidatus	Candidatus Methanoregula boonei 6A8	YP_001405321.1	7 E-98	521/635
Eukaryota	Viridiplantae	Arabidopsis thaliana	NP_564034.1	9 E-98	525/635
Eukaryota	Viridiplantae	Ricinus communis	XP_002525010.1	9 E-98	521/635
Archaea	Euryarchaeota	Methanosarcina acetivorans C2A	NP_615556.1	1 E-97	522/635
Eukaryota	Viridiplantae	Physcomitrella patens subsp. patens	XP_001751801.1	1 E-97	524/635
Eukaryota	Viridiplantae	Physcomitrella patens subsp. patens	XP_001766812.1	2 E-97	517/635
Eukaryota	Viridiplantae	Vitis vinifera	XP_002273552.1	2 E-97	531/635
Archaea	Euryarchaeota	Methanosarcina mazei Go1	NP_633777.1	4 E-97	522/635
Eukaryota	Viridiplantae	Ricinus communis	XP_002510158.1	6 E-97	538/635
Eukaryota	Viridiplantae	Vitis vinifera	CAO61403.1	1 E-96	511/635
Eukaryota	Viridiplantae	Oryza sativa Indica Group	EAZ01932.1	1 E-96	518/635
Eukaryota	Viridiplantae	Oryza sativa Japonica Group	EAZ37861.1	1 E-96	518/635
Eukaryota	Viridiplantae	Physcomitrella patens subsp. patens	XP_001759699.1	8 E-96	508/635
Bacteria	Cyanobacteria	Thermosynechococcus elongatus BP-1	NP_681115.1	8 E-96	526/635
Bacteria	Firmicutes	Mitsuokella multacida DSM 20544	ZP_05404709.2	6 E-95	533/635
Archea	Methanosphaerula	Methanosphaerula palustris E1-9c	YP_002465536.1	6 E-95	521/635
Archaea	Euryarchaeota	Methanoculleus marisnigri JR1	YP_001047758.1	1 E-94	520/635
Bacteria	Acidobacteria	uncultured Acidobacteria bacterium	AAP58615.1	2 E-94	530/635
Bacteria	Firmicutes	Paenibacillus sp. oral taxon	ZP_04853711.1	3 E-94	514/635
Archaea	Euryarchaeota	Methanosarcina barkeri str. Fusaro	YP_304970.1	3 E-94	522/635
Eukaryota	Viridiplantae	Oryza sativa Japonica Group	BAD37553.1	5 E-94	530/635
Bacteria	Cyanobacteria	Cyanothece sp. PCC 7425	YP_002484060.1	8 E-94	525/635

Bacteria	Cyanobacteria	Lyngbya sp. PCC 8106	ZP_01623360.1	1 E-93	526/635
Eukaryota	Viridiplantae	Oryza sativa Japonica Group	NP_001058248.1	3 E-93	534/635
Bacteria	Verrucomicrobia	Chthoniobacter flavus Ellin428	ZP_03132314.1	9 E-93	527/635
Bacteria	Nitrospirae	Leptospirillum rubarum	EAY55804.1	1 E-92	525/635
Bacteria	Nitrospirae	Leptospirillum sp. Group II	EDZ39364.1	1 E-92	525/635
Bacteria	Cyanobacteria	Acaryochloris marina MBIC11017	YP_001515625.1	2 E-92	525/635
Eukaryota	Viridiplantae	Sorghum bicolor;	XP_002438779.1	3 E-92	518/635
Eukaryota	Viridiplantae	Physcomitrella patens subsp. patens	XP_001753924.1	4 E-92	508/635
Bacteria	Firmicutes	Desulfotomaculum reducens MI-1	YP_001111391.1	1 E-91	525/635
Bacteria	Firmicutes	Dethiobacter alkaliphilus AHT 1	ZP_03728432.1	2 E-91	523/635
Archaea	Euryarchaeota	Archaeoglobus fulgidus DSM 4304	NP_069647.1	3 E-91	526/635
Archaea	Euryarchaeota	Methanococcoides burtonii DSM 6242	YP_566986.1	4 E-91	522/635
Archaea	Euryarchaeota	Methanospirillum hungatei JF-1	YP_504469.1	4 E-91	520/635
Bacteria	Cyanobacteria	Arthrospira maxima CS-328	ZP_03272375.1	7 E-91	526/635
Eukaryota	Viridiplantae	Oryza sativa Japonica Group	NP_001061929.1	1 E-90	571/635
Bacteria	Cyanobacteria	Trichodesmium erythraeum IMS101	YP_723673.1	2 E-90	526/635
Bacteria	Cyanobacteria	Synechocystis sp. PCC 6803	NP_441198.1	7 E-90	524/635
Bacteria	Cyanobacteria	Crocosphaera watsonii WH 8501	ZP_00514196.1	7 E-90	523/635
Bacteria	Firmicutes	Pelotomaculum thermopropionicum SI	YP_001210562.1	9 E-90	525/635
Bacteria	Firmicutes	Candidatus Desulforudis audaxviator MP104C	YP_001716213.1	1 E-89	525/635
Bacteria	Firmicutes	Brevibacillus brevis NBRC 100599	YP_002771903.1	2 E-89	525/635
Archaea	Euryarchaeota	Methanosaeta thermophila PT	YP_843643.1	3 E-89	522/635
Bacteria	Proteobacteria	Geobacter lovleyi SZ	YP_001952323.1	8 E-89	509/635
Bacteria	Firmicutes	Ammonifex degensii KC4	YP_003238037.1	2 E-88	525/635
Bacteria	Firmicutes	Thermoanaerobacterium thermosaccharolyticum	ZP_05337501.1	3 E-88	529/635
Bacteria	Firmicutes	Thermoanaerobacter tengcongensis MB4	NP_624129.1	8 E-88	529/635
Eukaryota	Viridiplantae	Zea mays;	ACF87704.1	2 E-87	530/635
Eukaryota	Viridiplantae	Zea mays;	NP_001148319.1	2 E-87	539/635
Bacteria	Firmicutes	Clostridium tetani E88	NP_781361.1	2 E-87	529/635
Bacteria	Cyanobacteria	Cyanothece sp. PCC 8801	YP_002372544.1	4 E-87	523/635
Archaea	Euryarchaeota	Ferroglobus placidus DSM 10642	ZP_05841118.1	5 E-87	526/635
Bacteria	Firmicutes	Thermoanaerobacter italicus Ab9	ZP_05334109.1	5 E-87	529/635
Bacteria	Firmicutes	Thermoanaerobacter pseudethanolicus ATCC 3	YP_001664154.1	5 E-87	528/635
Archaea	Euryarchaeota	Methanocaldococcus jannaschii DSM 2661	NP_248012.1	1 E-86	522/635
Bacteria	Cyanobacteria	Prochlorococcus marinus str. MIT	YP_397948.1	2 E-86	526/635
Bacteria	Firmicutes	Moorella thermoacetica ATCC 39073	YP_428903.1	2 E-86	524/635
Bacteria	Cyanobacteria	Synechococcus elongatus PCC 6301	YP_173196.1	2 E-86	528/635
Bacteria	Firmicutes	Thermoanaerobacter mathranii subsp. mathranii	ZP_05378580.1	2 E-86	529/635
Bacteria	Cyanobacteria	Synechococcus sp. JA-2-3B'a(2-13)	YP_477614.1	3 E-86	525/635
Bacteria	Firmicutes	Desulfotomaculum acetoxidans DSM 771	YP_003193626.1	4 E-86	525/635
Bacteria	Firmicutes	Heliobacterium modesticaldum Ice1	YP_001679721.1	1 E-85	524/635
Bacteria	Cyanobacteria	Cyanothece sp. ATCC 51142	YP_001803550.1	1 E-85	523/635
Bacteria	Cyanobacteria	Cyanothece sp. CCY0110	ZP_01728849.1	1 E-85	523/635
Bacteria	Firmicutes	Thermoanaerobacter sp. X514	YP_001663905.1	2 E-85	528/635
Bacteria	Cyanobacteria	Synechococcus sp. JA-3-3Ab	YP_474789.1	2 E-85	526/635
Bacteria	Cyanobacteria	Aphanothece halophytica	BAF91727.1	2 E-85	524/635
Bacteria	Firmicutes	Thermoanaerobacter ethanolicus CCSD1	ZP_05492511.1	2 E-85	528/635
Archaea	Euryarchaeota	Methanocaldococcus fervens AG86	YP_003127955.1	4 E-85	522/635
Bacteria	Cyanobacteria	Prochlorococcus marinus str. AS9601	YP_001009945.1	6 E-85	526/635
Bacteria	Firmicutes	Syntrophomonas wolfei subsp. wolfei	YP_752739.1	1 E-84	523/635
Bacteria	Cyanobacteria	Microcoleus chthonoplastes PCC 7420	ZP_05026373.1	1 E-84	525/635
Bacteria	Cyanobacteria	Cyanothece sp. PCC 7822	ZP_03154794.1	1 E-84	523/635
Bacteria	Cyanobacteria	Prochlorococcus marinus str. MIT	YP_001484782.1	1 E-84	526/635
Bacteria	Cyanobacteria	Synechococcus sp. PCC 7335	ZP_05035750.1	2 E-84	525/635
Bacteria	Cyanobacteria	Prochlorococcus marinus str. MIT	YP_001011829.1	2 E-84	526/635
Bacteria	Cyanobacteria	Prochlorococcus marinus str. MIT	ZP_05138923.1	4 E-84	526/635
Bacteria	Firmicutes	Staphylococcus carnosus subsp. carnosus	YP_002634420.1	5 E-84	526/635
Bacteria	Cyanobacteria	Nodularia spumigena CCY9414	ZP_01627985.1	5 E-84	524/635

Bacteria	Cyanobacteria	Synechococcus sp. PCC 7002	YP_001734500.1	6 E-84	523/635
Bacteria	Firmicutes	Selenomonas sputigena ATCC 35185	ZP_05900020.1	2 E-83	523/635
Bacteria	Cyanobacteria	Prochlorococcus marinus str. MIT	YP_001091764.1	2 E-83	526/635
Bacteria	Cyanobacteria	Prochlorococcus marinus subsp. pastoris	NP_893471.1	2 E-83	526/635
Archaea	Euryarchaeota	Methanocaldococcus infernus ME	ZP_04789769.1	3 E-83	522/635
Bacteria	Proteobacteria	Geobacter sp. M21	YP_003022063.1	5 E-83	511/635
Bacteria	Cyanobacteria	Nostoc azollae 0708	ZP_03767444.1	6 E-83	524/635
Bacteria	Proteobacteria	Methylocella silvestris BL2	YP_002361703.1	1 E-82	526/635

AFUA_2G04500

Eukaryota	Fungi	Aspergillus fumigatus Af293	XP_749581.1	3 E-50	97/97

AFUA_2G04510

Eukaryota	Fungi	Aspergillus fumigatus Af293	XP_749582.1	0.0	540/540
Eukaryota	Fungi	Neosartorya fischeri NRRL 181	XP_001260079.1	0.0	519/540
Eukaryota	Fungi	Penicillium chrysogenum Wisconsin 54-1255	XP_002557878.1	6 E-81	518/540

AFUA_2G04520

Eukaryota	Fungi	Aspergillus fumigatus Af293	XP_749583.1	0.0	488/488
Eukaryota	Fungi	Neosartorya fischeri NRRL 181	XP_001260080.1	0.0	488/488
Eukaryota	Fungi	Aspergillus flavus NRRL3357	XP_002375086.1	0.0	491/488
Eukaryota	Fungi	Aspergillus oryzae RIB40	XP_001819453.1	0.0	493/488
Eukaryota	Fungi	Aspergillus niger CBS 513.88	XP_001394313.1	0.0	493/488
Eukaryota	Fungi	Aspergillus terreus NIH2624	XP_001215236.1	0.0	471/488
Eukaryota	Fungi	Aspergillus nidulans FGSC A4	XP_659472.1	0.0	491/488
Eukaryota	Fungi	Talaromyces stipitatus ATCC 10500	XP_002340727.1	0.0	492/488
Eukaryota	Fungi	Penicillium chrysogenum Wisconsin 54-1255	XP_002557879.1	0.0	492/488
Eukaryota	Fungi	Aspergillus clavatus NRRL 1	XP_001272741.1	0.0	490/488
Eukaryota	Fungi	Penicillium marneffei ATCC 18224	XP_002144749.1	0.0	493/488
Eukaryota	Fungi	Paracoccidioides brasiliensis Pb18;	EEH45018.1	0.0	493/488
Eukaryota	Fungi	Coccidioides immitis RS;	XP_001247471.1	0.0	494/488
Eukaryota	Fungi	Ajellomyces dermatitidis SLH14081	XP_002625467.1	0.0	493/488
Eukaryota	Fungi	Paracoccidioides brasiliensis Pb01;	EEH40062.1	0.0	479/488
Eukaryota	Fungi	Pyrenophora tritici-repentis Pt-1C-BFP	XP_001937156.1	0.0	490/488
Eukaryota	Fungi	Microsporum canis CBS 113480	EEQ35832.1	0.0	493/488
Eukaryota	Fungi	Nectria haematococca mpVI 77-13-4	EEU41108.1	0.0	492/488
Eukaryota	Fungi	Uncinocarpus reesii 1704	XP_002541656.1	0.0	494/488
Eukaryota	Fungi	Ajellomyces capsulatus NAm1	XP_001536070.1	0.0	493/488
Eukaryota	Fungi	Ajellomyces capsulatus G186AR	EEH09553.1	0.0	493/488
Eukaryota	Fungi	Gibberella zeae PH-1	XP_389421.1	0.0	492/488
Eukaryota	Fungi	Sclerotinia sclerotiorum 1980 UF-70	XP_001585607.1	0.0	496/488
Eukaryota	Fungi	Chaetomium globosum CBS 148.51	XP_001227183.1	0.0	510/488
Eukaryota	Fungi	Magnaporthe grisea 70-15	XP_361350.1	0.0	490/488
Eukaryota	Fungi	Podospora anserina DSM 980	XP_001906205.1	0.0	504/488
Eukaryota	Fungi	Neurospora crassa OR74A	XP_957693.2	0.0	498/488
Eukaryota	Fungi	Paracoccidioides brasiliensis Pb03;	EEH18707.1	0.0	424/488
Eukaryota	Fungi	Phaeosphaeria nodorum SN15	XP_001803274.1	0.0	425/488
Eukaryota	Fungi	Ajellomyces capsulatus H143	EER41381.1	0.0	471/488
Eukaryota	Fungi	Verticillium albo-atrum VaMs.102	EEY21816.1	0.0	445/488
Eukaryota	Fungi	Yarrowia lipolytica CLIB122	XP_503997.1	1 E-163	484/488
Eukaryota	Fungi	Ustilago maydis 521	XP_758380.1	1 E-157	506/488
Eukaryota	Fungi	Coprinopsis cinerea okayama7#130	XP_001837165.1	1 E-152	490/488
Eukaryota	Fungi	Laccaria bicolor S238N-H82	XP_001876672.1	1 E-148	487/488
Eukaryota	Metazoa	Danio rerio	CAQ13689.1	1 E-144	449/488
Eukaryota	Metazoa	Danio rerio	NP_996969.1	1 E-143	449/488

Eukaryota	Metazoa	Ornithorhynchus anatinus	XP_001511833.1	1 E-142	444/488
Eukaryota	Metazoa	Tetraodon nigroviridis	CAF93117.1	1 E-141	446/488
Eukaryota	Metazoa	Nematostella vectensis	XP_001640433.1	1 E-139	417/488
Eukaryota	Metazoa	Rattus norvegicus	NP_001020594.1	1 E-137	465/488
Eukaryota	Fungi	Cryptococcus neoformans var. neoformans	XP_566553.1	1 E-137	518/488
Eukaryota	Fungi	Cryptococcus neoformans var. neoformans	XP_778178.1	1 E-137	518/488
Eukaryota	Metazoa	Ciona intestinalis	XP_002126466.1	1 E-136	421/488
Eukaryota	Metazoa	Gallus gallus	XP_424090.1	1 E-136	417/488
Eukaryota	Metazoa	Mus musculus	NP_780445.1	1 E-136	463/488
Eukaryota	Metazoa	Bos taurus	NP_001095357.1	1 E-135	462/488
Eukaryota	Metazoa	Xenopus (Silurana) tropicalis	NP_989277.1	1 E-135	460/488
Eukaryota	Metazoa	Culex quinquefasciatus	XP_001847094.1	1 E-135	454/488
Eukaryota	Metazoa	Monodelphis domestica	XP_001379063.1	1 E-134	443/488
Eukaryota	Metazoa	Taeniopygia guttata	XP_002198853.1	1 E-134	457/488
Eukaryota	Metazoa	Tribolium castaneum	XP_968236.1	1 E-133	459/488
Eukaryota	Metazoa	Macaca mulatta	XP_001094680.1	1 E-133	455/488
Eukaryota	Metazoa	Ixodes scapularis	XP_002411587.1	1 E-133	423/488
Eukaryota	Metazoa	Pongo abelii	NP_001128888.1	1 E-132	465/488
Eukaryota	Metazoa	Pongo abelii	Q5RF11.1	1 E-132	465/488
Eukaryota	Metazoa	Strongylocentrotus purpuratus	XP_780928.2	1 E-132	476/488
Eukaryota	Metazoa	Xenopus laevis	NP_001121274.1	1 E-132	453/488
Eukaryota	Metazoa	Equus caballus	XP_001915713.1	1 E-132	425/488
Eukaryota	Metazoa	Rattus norvegicus	EDM11562.1	1 E-132	417/488
Eukaryota	Metazoa	Anopheles gambiae str. PEST	XP_316676.3	1 E-131	456/488
Eukaryota	Metazoa	Homo sapiens	NP_653251.2	1 E-131	454/488
Eukaryota	Metazoa	Acyrthosiphon pisum	XP_001952147.1	1 E-131	457/488
Eukaryota	Metazoa	Mus musculus	BAC34108.1	1 E-130	417/488
Eukaryota	Metazoa	Aedes aegypti	XP_001664064.1	1 E-129	457/488
Eukaryota	Metazoa	Macaca mulatta	XP_001094799.1	1 E-129	417/488
Eukaryota	Metazoa	Pongo abelii	NP_001127410.1	1 E-128	417/488
Eukaryota	Metazoa	Canis lupus familiaris	XP_849448.1	1 E-128	417/488
Eukaryota	Metazoa	Homo sapiens	BAB71335.1	1 E-127	417/488
Eukaryota	Metazoa	Homo sapiens	EAW86904.1	1 E-127	417/488
Bacteria	Chloroflexi	Sphaerobacter thermophilus DSM 20745	ZP_04496071.1	1 E-126	419/488
Eukaryota	Metazoa	Homo sapiens	EAW86901.1	1 E-125	422/488
Eukaryota	Metazoa	Aedes aegypti	XP_001664065.1	1 E-125	425/488
Eukaryota	Metazoa	Canis lupus familiaris	XP_535091.2	1 E-123	395/488
Eukaryota	Metazoa	Drosophila persimilis	XP_002018122.1	1 E-123	458/488
Eukaryota	Metazoa	Drosophila ananassae	XP_001959750.1	1 E-123	459/488
Eukaryota	Metazoa	Drosophila simulans	XP_002082603.1	1 E-123	459/488
Eukaryota	Metazoa	Drosophila sechellia	XP_002039891.1	1 E-123	459/488
Eukaryota	Metazoa	Drosophila erecta	XP_001975186.1	1 E-122	459/488
Eukaryota	Metazoa	Drosophila yakuba	XP_002092406.1	1 E-122	459/488
Eukaryota	Metazoa	Drosophila melanogaster	NP_477209.2	1 E-121	459/488
Eukaryota	Metazoa	Drosophila melanogaster	ACN86075.1	1 E-121	459/488
Eukaryota	Metazoa	Drosophila mojavensis	XP_002005588.1	1 E-121	459/488
Eukaryota	Metazoa	Drosophila willistoni	XP_002061275.1	1 E-120	456/488
Eukaryota	Metazoa	Drosophila grimshawi	XP_001987221.1	1 E-120	459/488
Eukaryota	Metazoa	Drosophila melanogaster	AAM11277.1	1 E-120	459/488
Eukaryota	Metazoa	Drosophila melanogaster	AAL28462.1	1 E-119	422/488
Eukaryota	Metazoa	Drosophila virilis	XP_002050894.1	1 E-119	459/488
Eukaryota	Metazoa	Apis mellifera	XP_624450.1	1 E-118	415/488
Eukaryota	Metazoa	Homo sapiens	BAG57012.1	1 E-118	395/488
Eukaryota	Metazoa	Caenorhabditis briggsae AF16	XP_001679485.1	1 E-116	443/488
Eukaryota	Metazoa	Branchiostoma floridae	XP_002603113.1	1 E-116	421/488
Eukaryota	stramenopiles	Phaeodactylum tricornutum CCAP 1055/1	XP_002179196.1	1 E-115	425/488
Eukaryota	Metazoa	Caenorhabditis elegans	NP_496764.1	1 E-113	443/488
Eukaryota	Amoebozoa	Dictyostelium discoideum AX4	XP_635889.1	1 E-110	434/488

Bacteria	Chloroflexi	Thermomicrobium roseum DSM 5159	YP_002521449.1	1 E-106	411/488
Bacteria	Chloroflexi	Sphaerobacter thermophilus DSM 20745	ZP_04494342.1	1 E-104	411/488
Bacteria	Actinobacteria	Rubrobacter xylanophilus DSM 9941	YP_645121.1	1 E-100	418/488
Bacteria	Actinobacteria	Mycobacterium vanbaalenii PYR-1	YP_956582.1	7 E-97	403/488
Bacteria	Actinobacteria	Mycobacterium gilvum PYR-GCK	YP_001132296.1	4 E-95	419/488
Bacteria	Actinobacteria	Thermomonospora curvata DSM 43183	ZP_04030364.1	7 E-94	407/488
Bacteria	Actinobacteria	Saccharomonospora viridis DSM 43017	YP_003132745.1	1 E-92	417/488
Bacteria	Thermus	Deinococcus geothermalis DSM 11300	YP_594028.1	2 E-92	431/488
Bacteria	Actinobacteria	Nocardioides sp. JS614	YP_921936.1	9 E-89	416/488
Bacteria	Proteobacteria	Haliangium ochraceum DSM 14365	ZP_03878892.1	2 E-88	424/488
Bacteria	Actinobacteria	Rhodococcus opacus B4	YP_002780540.1	3 E-87	404/488
Bacteria	Actinobacteria	Streptomyces sp. AA4	ZP_05481151.1	4 E-85	403/488
Bacteria	Proteobacteria	Acidiphilium cryptum JF-5	YP_001234549.1	5 E-85	416/488
Archaea	Euryarchaeota	Thermoplasma volcanium GSS1	NP_111803.1	1 E-83	417/488
Archaea	Crenarchaeota	Sulfolobus islandicus Y.N.15.51	YP_002841012.1	2 E-83	415/488
Bacteria	Actinobacteria	Geodermatophilus obscurus DSM 43160	ZP_03889028.1	7 E-83	417/488
Bacteria	Actinobacteria	Streptomyces sp. AA4	ZP_05476541.1	6 E-81	422/488
Archaea	Crenarchaeota	Sulfolobus islandicus M.16.27	YP_002842829.1	2 E-79	415/488
Archaea	Euryarchaeota	Halorubrum lacusprofundi ATCC 49239	YP_002567361.1	2 E-48	408/488

AFUA_2G04530

Eukaryota	Fungi	Aspergillus fumigatus Af293	XP_749584.1	2 E-27	64/64

AFUA_2G04533

Eukaryota	Fungi	Aspergillus fumigatus Af293	XP_001481654.1	1 E-100	177/177
Eukaryota	Fungi	Neosartorya fischeri NRRL 181	XP_001260081.1	3 E-83	181/177
Eukaryota	Fungi	Aspergillus nidulans FGSC A4	XP_659467.1	4 E-40	194/177
Eukaryota	Fungi	Phaeosphaeria nodorum SN15	XP_001805006.1	7 E-31	153/177
Eukaryota	Fungi	Penicillium chrysogenum Wisconsin 54-1255	XP_002557880.1	8 E-31	145/177
Eukaryota	Fungi	Podospora anserina DSM 980	XP_001911468.1	9 E-23	144/177

AFUA_2G05150

Eukaryota	Fungi	Aspergillus fumigatus Af293	XP_749645.1	0.0	591/591

AFUA_2G06205

Eukaryota	Fungi	Aspergillus fumigatus Af293	XP_749750.2	1 E-124	214/214
Eukaryota	Fungi	Neosartorya fischeri NRRL 181	XP_001260245.1	7 E-79	174/214
Eukaryota	Fungi	Aspergillus oryzae RIB40	XP_001819229.1	5 E-63	229/214
Eukaryota	Fungi	Aspergillus flavus NRRL3357	XP_002382229.1	1 E-62	229/214
Eukaryota	Fungi	Aspergillus clavatus NRRL 1	XP_001275210.1	2 E-59	233/214
Eukaryota	Fungi	Penicillium chrysogenum Wisconsin 54-1255	XP_002568165.1	9 E-56	202/214
Eukaryota	Fungi	Ajellomyces capsulatus H143	EER43418.1	9 E-55	239/214
Eukaryota	Fungi	Aspergillus niger CBS 513.88	XP_001400614.1	4 E-54	227/214
Eukaryota	Fungi	Ajellomyces capsulatus NAm1	XP_001541526.1	1 E-52	242/214
Eukaryota	Fungi	Ajellomyces capsulatus G186AR	EEH11366.1	2 E-52	242/214
Eukaryota	Fungi	Penicillium marneffei ATCC 18224	XP_002144548.1	1 E-51	234/214
Eukaryota	Fungi	Paracoccidioides brasiliensis Pb18;	EEH47761.1	3 E-49	241/214
Eukaryota	Fungi	Paracoccidioides brasiliensis Pb03;	EEH18950.1	3 E-48	239/214
Eukaryota	Fungi	Talaromyces stipitatus ATCC 10500	XP_002340987.1	3 E-48	248/214
Eukaryota	Fungi	Ajellomyces dermatitidis ER-3	EEQ87737.1	4 E-47	240/214
Eukaryota	Fungi	Ajellomyces dermatitidis SLH14081	XP_002623884.1	4 E-47	240/214
Eukaryota	Fungi	Paracoccidioides brasiliensis Pb01;	EEH38061.1	2 E-46	238/214
Eukaryota	Fungi	Gibberella zeae PH-1	XP_390070.1	8 E-35	212/214
Eukaryota	Fungi	Phaeosphaeria nodorum SN15	XP_001799753.1	9 E-32	218/214

Eukaryota	Fungi	Nectria haematococca mpVI 77-13-4	EEU45044.1	6 E-30	191/214
Eukaryota	Fungi	Neurospora crassa OR74A	XP_957379.1	2 E-28	221/214
Eukaryota	Fungi	Verticillium albo-atrum VaMs.102	EEY14306.1	2 E-26	196/214
Eukaryota	Fungi	Podospora anserina DSM 980	XP_001903189.1	9 E-21	215/214

AFUA_2G06220

Eukaryota	Fungi	Aspergillus fumigatus Af293	XP_749751.1	0.0	632/632
Eukaryota	Fungi	Neosartorya fischeri NRRL 181	XP_001260247.1	0.0	632/632
Eukaryota	Fungi	Aspergillus clavatus NRRL 1	XP_001275208.1	0.0	631/632
Eukaryota	Fungi	Aspergillus oryzae RIB40	XP_001819230.1	0.0	634/632
Eukaryota	Fungi	Aspergillus nidulans FGSC A4	XP_680700.1	0.0	628/632
Eukaryota	Fungi	Aspergillus terreus NIH2624	XP_001214793.1	0.0	616/632
Eukaryota	Fungi	Aspergillus niger CBS 513.88	XP_001400616.1	0.0	626/632
Eukaryota	Fungi	Penicillium chrysogenum Wisconsin 54-1255	XP_002568167.1	0.0	619/632
Eukaryota	Fungi	Paracoccidioides brasiliensis Pb03;	EEH16311.1	0.0	636/632
Eukaryota	Fungi	Paracoccidioides brasiliensis Pb01;	EEH35960.1	0.0	637/632
Eukaryota	Fungi	Penicillium marneffei ATCC 18224	XP_002144547.1	0.0	645/632
Eukaryota	Fungi	Ajellomyces capsulatus G186AR	EEH02558.1	0.0	645/632
Eukaryota	Fungi	Ajellomyces dermatitidis ER-3	EEQ86223.1	0.0	638/632
Eukaryota	Fungi	Talaromyces stipitatus ATCC 10500	XP_002340988.1	0.0	644/632
Eukaryota	Fungi	Ajellomyces dermatitidis SLH14081	XP_002620159.1	0.0	621/632
Eukaryota	Fungi	Coccidioides immitis RS;	XP_001244808.1	0.0	598/632
Eukaryota	Fungi	Coccidioides posadasii C735 delta	EER26020.1	1 E-180	598/632
Eukaryota	Fungi	Microsporum canis CBS 113480	EEQ28499.1	1 E-178	609/632
Eukaryota	Fungi	Ajellomyces capsulatus H143	EER37721.1	1 E-173	600/632
Eukaryota	Fungi	Uncinocarpus reesii 1704	XP_002584983.1	1 E-170	589/632
Eukaryota	Fungi	Ajellomyces capsulatus NAm1	XP_001544989.1	1 E-166	507/632
Eukaryota	Fungi	Paracoccidioides brasiliensis Pb18;	EEH43026.1	1 E-166	587/632
Eukaryota	Fungi	Sclerotinia sclerotiorum 1980 UF-70	XP_001594046.1	1 E-137	625/632
Eukaryota	Viridiplantae	Arabidopsis thaliana	NP_193471.2	1 E-30	524/632
Eukaryota	Viridiplantae	Arabidopsis thaliana	BAE99012.1	1 E-30	524/632
Eukaryota	Viridiplantae	Sorghum bicolor;	XP_002467201.1	3 E-30	581/632

AFUA_2G06330

Eukaryota	Fungi	Aspergillus fumigatus Af293	XP_749762.1	0.0	696/696
Eukaryota	Fungi	Neosartorya fischeri NRRL 181	XP_001260257.1	0.0	732/696
Eukaryota	Fungi	Aspergillus clavatus NRRL 1	XP_001275198.1	0.0	727/696
Eukaryota	Fungi	Aspergillus oryzae RIB40	XP_001819242.1	0.0	725/696
Eukaryota	Fungi	Aspergillus niger CBS 513.88	XP_001400628.1	0.0	722/696
Eukaryota	Fungi	Aspergillus nidulans FGSC A4	XP_680691.1	0.0	695/696
Eukaryota	Fungi	Penicillium chrysogenum Wisconsin 54-1255	XP_002568177.1	0.0	721/696
Eukaryota	Fungi	Aspergillus flavus NRRL3357	XP_002382242.1	0.0	657/696
Eukaryota	Fungi	Ajellomyces dermatitidis SLH14081	XP_002620148.1	0.0	737/696
Eukaryota	Fungi	Talaromyces stipitatus ATCC 10500	XP_002340999.1	0.0	728/696
Eukaryota	Fungi	Penicillium marneffei ATCC 18224	XP_002144533.1	0.0	725/696
Eukaryota	Fungi	Penicillium marneffei ATCC 18224	XP_002144534.1	0.0	724/696
Eukaryota	Fungi	Aspergillus terreus NIH2624	XP_001214801.1	0.0	699/696
Eukaryota	Fungi	Paracoccidioides brasiliensis Pb01;	EEH35972.1	0.0	726/696
Eukaryota	Fungi	Ajellomyces capsulatus G186AR	EEH05978.1	0.0	727/696
Eukaryota	Fungi	Paracoccidioides brasiliensis Pb03;	EEH16300.1	0.0	721/696
Eukaryota	Fungi	Paracoccidioides brasiliensis Pb18;	EEH43014.1	0.0	721/696
Eukaryota	Fungi	Pyrenophora tritici-repentis Pt-1C-BFP	XP_001942015.1	0.0	745/696
Eukaryota	Fungi	Phaeosphaeria nodorum SN15	XP_001793477.1	0.0	745/696
Eukaryota	Fungi	Coccidioides posadasii C735 delta	EER28211.1	0.0	732/696
Eukaryota	Fungi	Sclerotinia sclerotiorum 1980 UF-70	XP_001588082.1	0.0	727/696
Eukaryota	Fungi	Microsporum canis CBS 113480	EEQ28489.1	0.0	723/696

Eukaryota	Fungi	Podospora anserina DSM 980	XP_001903598.1	0.0	722/696
Eukaryota	Fungi	Nectria haematococca mpVI 77-13-4	EEU39615.1	0.0	720/696
Eukaryota	Fungi	Chaetomium globosum CBS 148.51	XP_001226305.1	0.0	724/696
Eukaryota	Fungi	Magnaporthe grisea 70-15	XP_362778.1	0.0	727/696
Eukaryota	Fungi	Uncinocarpus reesii 1704	XP_002584985.1	0.0	703/696
Eukaryota	Fungi	Gibberella zeae PH-1	XP_382816.1	0.0	717/696
Eukaryota	Fungi	Neurospora crassa OR74A	XP_960037.1	0.0	731/696
Eukaryota	Fungi	Botryotinia fuckeliana B05.10	XP_001550856.1	0.0	637/696
Eukaryota	Fungi	Coccidioides immitis RS;	XP_001243340.1	0.0	633/696
Eukaryota	Fungi	Ajellomyces capsulatus H143	EER37710.1	0.0	626/696
Eukaryota	Fungi	Yarrowia lipolytica CLIB122	XP_501002.1	1 E-157	702/696
Eukaryota	Fungi	Laccaria bicolor S238N-H82	XP_001889939.1	1 E-154	741/696
Eukaryota	Fungi	Postia placenta Mad-698-R	XP_002469027.1	1 E-153	670/696
Eukaryota	Fungi	Cryptococcus neoformans var. neoformans	XP_775041.1	1 E-151	736/696
Eukaryota	Fungi	Cryptococcus neoformans var. neoformans	XP_571361.1	1 E-151	736/696
Eukaryota	Fungi	Cryptococcus neoformans var. neoformans	XP_571362.1	1 E-147	723/696
Eukaryota	Fungi	Coprinopsis cinerea okayama7#130	XP_001834663.1	1 E-143	702/696
Eukaryota	Fungi	Schizosaccharomyces japonicus yFS275	XP_002171918.1	1 E-143	704/696
Eukaryota	Fungi	Ustilago maydis 521	XP_756445.1	1 E-142	798/696
Eukaryota	Fungi	Schizosaccharomyces pombe	NP_596085.1	1 E-139	718/696
Eukaryota	Fungi	Pichia pastoris GS115	XP_002493173.1	1 E-135	732/696
Eukaryota	Fungi	Pichia stipitis CBS 6054	XP_001384426.2	1 E-131	738/696
Eukaryota	Fungi	Malassezia globosa CBS 7966	XP_001730762.1	1 E-127	699/696
Eukaryota	Fungi	Pichia guilliermondii ATCC 6260	EDK38619.2	1 E-127	713/696
Eukaryota	Fungi	Pichia guilliermondii ATCC 6260	XP_001484988.1	1 E-126	713/696
Eukaryota	Fungi	Candida tropicalis MYA-3404	XP_002547120.1	1 E-124	731/696
Eukaryota	Fungi	Debaryomyces hansenii CBS767	XP_461931.1	1 E-123	729/696
Eukaryota	Fungi	Candida albicans SC5314	XP_722422.1	1 E-122	735/696
Eukaryota	Fungi	Candida albicans WO-1	EEQ45641.1	1 E-122	736/696
Eukaryota	Fungi	Candida albicans SC5314	XP_722561.1	1 E-121	735/696
Eukaryota	Fungi	Candida dubliniensis CD36	XP_002418161.1	1 E-120	729/696
Eukaryota	Fungi	Clavispora lusitaniae ATCC 42720	XP_002619161.1	1 E-120	714/696
Eukaryota	Metazoa	Gallus gallus	XP_416513.2	1 E-117	771/696
Eukaryota	Metazoa	Taeniopygia guttata	XP_002196552.1	1 E-115	772/696
Eukaryota	Fungi	Lodderomyces elongisporus NRRL YB-4239	XP_001528598.1	1 E-114	756/696
Eukaryota	Metazoa	Pan troglodytes	XP_001163218.1	1 E-114	773/696
Eukaryota	Metazoa	Monodelphis domestica	XP_001370137.1	1 E-114	796/696
Eukaryota	Metazoa	Canis lupus familiaris	XP_867393.1	1 E-114	773/696
Eukaryota	Metazoa	Mus musculus	BAE32312.1	1 E-113	773/696
Eukaryota	Metazoa	Taeniopygia guttata	XP_002196564.1	1 E-113	795/696
Eukaryota	Metazoa	Macaca mulatta	XP_001110679.1	1 E-112	773/696
Eukaryota	Metazoa	Homo sapiens	NP_003472.2	1 E-112	773/696
Eukaryota	Metazoa	Pan troglodytes	XP_508970.2	1 E-112	796/696
Eukaryota	Metazoa	Homo sapiens	AAA78934.1	1 E-112	773/696
Eukaryota	Metazoa	Rattus norvegicus	NP_001100089.1	1 E-112	796/696
Eukaryota	Metazoa	Macaca mulatta	XP_001110571.1	1 E-112	772/696
Eukaryota	Metazoa	Canis lupus familiaris	XP_543845.2	1 E-112	796/696
Eukaryota	Viridiplantae	Physcomitrella patens subsp. patens	XP_001785646.1	1 E-112	742/696
Eukaryota	Metazoa	Mus musculus	NP_038728.1	1 E-112	796/696
Eukaryota	Metazoa	Equus caballus	XP_001915559.1	1 E-112	792/696
Eukaryota	Metazoa	Homo sapiens	prf2124276A	1 E-111	772/696
Eukaryota	Metazoa	Macaca mulatta	XP_001110639.1	1 E-111	796/696
Eukaryota	Metazoa	Homo sapiens	NP_001092006.1	1 E-111	796/696
Eukaryota	Metazoa	Homo sapiens	CAA62690.1	1 E-111	796/696
Eukaryota	Metazoa	Nematostella vectensis	XP_001641941.1	1 E-110	726/696
Eukaryota	Metazoa	Pongo abelii	NP_001127051.1	1 E-110	796/696
Eukaryota	Metazoa	Bos taurus	XP_581544.3	1 E-109	796/696
Eukaryota	Metazoa	Canis lupus familiaris	XP_867414.1	1 E-109	748/696

Eukaryota	Metazoa	Drosophila willistoni	XP_002068928.1	1 E-109	598/696
Eukaryota	Viridiplantae	Arabidopsis thaliana	AAG42755.1	1 E-107	742/696
Eukaryota	Viridiplantae	Arabidopsis thaliana	NP_566666.2	1 E-107	742/696
Eukaryota	Viridiplantae	Arabidopsis thaliana	BAB01171.1	1 E-107	742/696
Eukaryota	Metazoa	Xenopus (Silurana) tropicalis	NP_001116956.1	1 E-105	764/696
Eukaryota	Metazoa	Drosophila mojavensis	XP_002007042.1	1 E-105	597/696
Eukaryota	Metazoa	Xenopus laevis	NP_001083082.1	1 E-105	764/696
Eukaryota	Metazoa	Xenopus laevis	NP_001087432.1	1 E-105	786/696
Eukaryota	Viridiplantae	Oryza sativa Japonica Group	NP_001042185.1	1 E-105	739/696
Eukaryota	Viridiplantae	Oryza sativa Indica Group	EEC70038.1	1 E-104	739/696
Eukaryota	Viridiplantae	Vitis vinifera	CAO39183.1	1 E-104	753/696
Eukaryota	Metazoa	Drosophila grimshawi	XP_001983288.1	1 E-104	597/696
Eukaryota	Metazoa	Drosophila virilis	XP_002046103.1	1 E-104	600/696
Eukaryota	Viridiplantae	Vitis vinifera	XP_002284309.1	1 E-103	740/696
Eukaryota	Metazoa	Tribolium castaneum	XP_967156.1	1 E-103	578/696
Eukaryota	Metazoa	Drosophila pseudoobscura pseudoobscura	XP_001352737.1	1 E-103	599/696
Eukaryota	Metazoa	Salmo salar	NP_001135286.1	1 E-103	791/696
Eukaryota	Metazoa	Xenopus laevis	AAQ86957.1	1 E-103	787/696
Eukaryota	Metazoa	Drosophila ananassae	XP_001957031.1	1 E-103	597/696
Eukaryota	Alveolata	Tetrahymena thermophila	XP_001020768.1	1 E-103	747/696
Eukaryota	Metazoa	Drosophila sechellia	XP_002035139.1	1 E-103	597/696
Eukaryota	Viridiplantae	Populus trichocarpa	XP_002330384.1	1 E-102	752/696
Eukaryota	Metazoa	Drosophila simulans	XP_002083433.1	1 E-102	597/696
Eukaryota	Metazoa	Drosophila melanogaster	NP_647773.1	1 E-102	597/696
Eukaryota	Metazoa	Drosophila yakuba	XP_002094142.1	1 E-102	597/696
Eukaryota	Metazoa	Drosophila erecta	XP_001971348.1	1 E-101	597/696
Eukaryota	Metazoa	Taeniopygia guttata	XP_002193106.1	1 E-101	776/696
Eukaryota	Metazoa	Danio rerio	NP_999920.2	1 E-101	773/696
Eukaryota	Metazoa	Danio rerio	AAH66694.1	1 E-101	773/696
Eukaryota	Metazoa	Nasonia vitripennis	XP_001606298.1	1 E-101	585/696
Eukaryota	Metazoa	Trichoplax adhaerens	XP_002113846.1	1 E-101	598/696
Eukaryota	Metazoa	Apis mellifera	XP_624702.1	1 E-100	738/696
Eukaryota	Metazoa	Danio rerio	CAK04985.1	1 E-100	792/696
Eukaryota	Viridiplantae	Sorghum bicolor;	XP_002457247.1	2 E-99	738/696
Eukaryota	Metazoa	Strongylocentrotus purpuratus	XP_001189314.1	2 E-99	577/696
Eukaryota	Viridiplantae	Populus trichocarpa	XP_002317532.1	5 E-99	754/696
Eukaryota	Viridiplantae	Ricinus communis	XP_002525787.1	2 E-98	735/696
Eukaryota	Viridiplantae	Zea mays;	ACN32026.1	2 E-98	738/696
Eukaryota	Metazoa	Gallus gallus	XP_426842.2	4 E-97	786/696
Eukaryota	Metazoa	Mus musculus	NP_001013042.1	9 E-97	779/696
Eukaryota	Metazoa	Pan troglodytes	XP_526393.2	1 E-96	773/696
Eukaryota	Metazoa	Culex quinquefasciatus	XP_001847129.1	2 E-96	601/696
Eukaryota	Metazoa	Hydra magnipapillata	XP_002168139.1	3 E-96	588/696
Eukaryota	Fungi	Lachancea thermotolerans CBS 6340	XP_002552544.1	4 E-96	693/696
Eukaryota	Metazoa	Danio rerio	NP_001091856.1	5 E-96	799/696
Eukaryota	Metazoa	Bos taurus	XP_001787414.1	8 E-96	782/696
Eukaryota	Metazoa	Monodelphis domestica	XP_001368216.1	9 E-96	787/696
Eukaryota	Fungi	Candida glabrata CBS 138	XP_447240.1	1 E-95	624/696
Eukaryota	Metazoa	Homo sapiens	AAC63405.1	1 E-95	782/696
Eukaryota	Metazoa	Anopheles gambiae str. PEST	XP_320294.3	1 E-95	611/696
Eukaryota	Metazoa	Homo sapiens	BAG63715.1	2 E-95	782/696
Eukaryota	Metazoa	Homo sapiens	NP_003931.2	2 E-95	782/696
Eukaryota	Metazoa	Equus caballus	XP_001496365.2	2 E-95	782/696
Eukaryota	Metazoa	Canis lupus familiaris	XP_535813.2	6 E-95	782/696
Eukaryota	Metazoa	Acyrthosiphon pisum	XP_001952484.1	6 E-95	580/696
Eukaryota	Metazoa	Macaca mulatta	XP_001107760.1	7 E-95	782/696
Eukaryota	Fungi	Zygosaccharomyces rouxii CBS 732	XP_002497253.1	1 E-94	695/696
Eukaryota	Metazoa	Rattus norvegicus	NP_001101135.1	2 E-94	779/696

Eukaryota	Metazoa	Aedes aegypti	XP_001652416.1	3 E-94	600/696
Eukaryota	Metazoa	Homo sapiens	BAF83027.1	7 E-94	782/696
Eukaryota	Amoebozoa	Dictyostelium discoideum	AAC71068.1	2 E-92	781/696
Eukaryota	Amoebozoa	Dictyostelium discoideum	P54201.2	4 E-92	781/696
Eukaryota	Amoebozoa	Dictyostelium discoideum AX4	XP_635198.2	5 E-92	781/696
Eukaryota	Viridiplantae	Ostreococcus tauri	CAL51849.1	1 E-90	605/696
Eukaryota	Fungi	Saccharomyces cerevisiae YJM789	EDN64671.1	2 E-90	563/696
Eukaryota	Fungi	Saccharomyces cerevisiae	CAA85001.1	3 E-90	563/696
Eukaryota	Fungi	Saccharomyces cerevisiae	NP_009614.2	3 E-90	563/696
Eukaryota	Fungi	Saccharomyces cerevisiae RM11-1a	EDV12044.1	4 E-90	563/696
Eukaryota	Metazoa	Tetraodon nigroviridis	CAG08062.1	1 E-89	772/696
Eukaryota	Metazoa	Pediculus humanus corporis	XP_002425026.1	1 E-88	585/696
Eukaryota	Metazoa	Bos taurus	ABM06095.1	2 E-87	624/696
Eukaryota	Metazoa	Acyrthosiphon pisum	XP_001946579.1	2 E-87	573/696
Eukaryota	Metazoa	Tetraodon nigroviridis	CAF97823.1	6 E-87	626/696
Eukaryota	Metazoa	Ciona intestinalis	tpdFAA00239.1	5 E-86	779/696
Eukaryota	Metazoa	Ciona intestinalis	XP_002122471.1	8 E-85	796/696
Eukaryota	Alveolata	Paramecium tetraurelia strain d4-2	XP_001436845.1	2 E-84	709/696
Eukaryota	Viridiplantae	Ostreococcus lucimarinus CCE9901	XP_001417342.1	2 E-83	607/696
Eukaryota	Fungi	Ashbya gossypii ATCC 10895	NP_986688.1	2 E-83	566/696
Eukaryota	Fungi	Kluyveromyces lactis NRRL Y-1140	XP_455918.1	1 E-81	559/696
Eukaryota	Viridiplantae	Micromonas sp. RCC299	XP_002504065.1	4 E-81	641/696
Eukaryota	Metazoa	Schistosoma japonicum	CAX72904.1	4 E-79	683/696
Eukaryota	Choanoflagellida	Monosiga brevicollis MX1	XP_001748436.1	1 E-78	587/696
Eukaryota	Viridiplantae	Micromonas pusilla CCMP1545	EEH56731.1	2 E-76	713/696
Eukaryota	Alveolata	Paramecium tetraurelia strain d4-2	XP_001451097.1	3 E-76	702/696
Eukaryota	Metazoa	Caenorhabditis briggsae AF16	XP_001669074.1	2 E-74	569/696
Eukaryota	Metazoa	Schistosoma mansoni	XP_002578206.1	2 E-70	682/696
Eukaryota	Metazoa	Caenorhabditis elegans	NP_491765.2	8 E-68	568/696
Eukaryota	stramenopiles	Phaeodactylum tricornutum CCAP 1055/1	XP_002177539.1	3 E-66	611/696
Eukaryota	Metazoa	Brugia malayi	XP_001902802.1	4 E-66	561/696
Eukaryota	stramenopiles	Thalassiosira pseudonana CCMP1335	XP_002291061.1	2 E-64	620/696
Eukaryota	Viridiplantae	Chlamydomonas reinhardtii	XP_001701682.1	1 E-63	595/696
Eukaryota	Parabasalidea	Trichomonas vaginalis G3	XP_001312940.1	2 E-57	630/696
Eukaryota	Alveolata	Babesia bovis T2Bo	XP_001610364.1	2 E-51	560/696
Eukaryota	Alveolata	Cryptosporidium muris RN66	XP_002142684.1	4 E-50	597/696
Eukaryota	Alveolata	Cryptosporidium hominis TU502	XP_667847.1	5 E-47	703/696
Eukaryota	Alveolata	Cryptosporidium parvum Iowa II	XP_627786.1	6 E-47	704/696
Eukaryota	Alveolata	Plasmodium falciparum 3D7	XP_001351447.1	2 E-38	778/696
Eukaryota	Alveolata	Plasmodium berghei str. ANKA	XP_679287.1	2 E-37	791/696
Eukaryota	Alveolata	Plasmodium vivax SaI-1	XP_001615021.1	6 E-33	661/696
Eukaryota	Alveolata	Plasmodium yoelii yoelii str.	XP_725205.1	3 E-31	759/696

AFUA_2G07440

Eukaryota	Fungi	Aspergillus fumigatus Af293	XP_755080.1	4 E-80	145/145
Eukaryota	Fungi	Aspergillus clavatus NRRL 1	XP_001267687.1	2 E-59	145/145
Eukaryota	Fungi	Aspergillus terreus NIH2624	XP_001209538.1	1 E-58	143/145
Eukaryota	Fungi	Aspergillus oryzae RIB40	XP_001826506.1	8 E-58	143/145
Eukaryota	Fungi	Aspergillus nidulans FGSC A4	tpeCBF70812.1	4 E-52	142/145
Eukaryota	Fungi	Aspergillus nidulans FGSC A4	XP_663409.1	1 E-51	142/145
Eukaryota	Fungi	Talaromyces stipitatus ATCC 10500	XP_002478731.1	9 E-47	140/145
Eukaryota	Fungi	Penicillium chrysogenum Wisconsin 54-1255	XP_002568453.1	2 E-38	143/145
Eukaryota	Fungi	Botryotinia fuckeliana B05.10	XP_001554378.1	5 E-35	141/145
Eukaryota	Fungi	Sclerotinia sclerotiorum 1980 UF-70	XP_001588146.1	6 E-35	141/145
Eukaryota	Fungi	Aspergillus flavus NRRL3357	XP_002372650.1	4 E-34	145/145
Eukaryota	Fungi	Phaeosphaeria nodorum SN15	XP_001805116.1	1 E-29	140/145
Eukaryota	Fungi	Pyrenophora tritici-repentis Pt-1C-BFP	XP_001932262.1	1 E-28	140/145

Eukaryota	Fungi	Podospora anserina DSM 980	XP_001911216.1	2 E-28	142/145
Eukaryota	Fungi	Nectria haematococca mpVI 77-13-4	EEU39919.1	6 E-28	137/145
Eukaryota	Fungi	Gibberella zeae PH-1	XP_386699.1	2 E-25	134/145
Eukaryota	Fungi	Magnaporthe grisea 70-15	XP_363268.1	5 E-25	142/145
Eukaryota	Fungi	Neurospora crassa OR74A	XP_961581.1	1 E-23	159/145
Eukaryota	Fungi	Ustilago maydis 521	XP_760354.1	3 E-20	131/145
Eukaryota	Fungi	Penicillium marneffei ATCC 18224	XP_002148082.1	6 E-19	135/145
Eukaryota	Fungi	Talaromyces stipitatus ATCC 10500	XP_002482323.1	5 E-18	135/145
Eukaryota	Fungi	Ajellomyces dermatitidis SLH14081	XP_002620335.1	2 E-17	135/145
Eukaryota	Fungi	Paracoccidioides brasiliensis Pb01;	EEH35286.1	5 E-17	135/145
Eukaryota	Viridiplantae	Picea sitchensis	ABK25910.1	3 E-16	119/145
Eukaryota	Fungi	Talaromyces stipitatus ATCC 10500	XP_002482324.1	1 E-15	126/145
Eukaryota	Fungi	Penicillium chrysogenum Wisconsin 54-1255	XP_002559089.1	2 E-15	130/145
Eukaryota	Fungi	Coccidioides immitis RS;	XP_001241469.1	5 E-15	143/145
Eukaryota	Metazoa	Danio rerio	CAK05423.1	7 E-15	126/145
Eukaryota	Metazoa	Nasonia vitripennis	XP_001600888.1	9 E-15	128/145
Eukaryota	Fungi	Yarrowia lipolytica CLIB122	XP_002143077.1	2 E-14	137/145
Eukaryota	Fungi	Aspergillus niger CBS 513.88	XP_001400678.1	1 E-13	155/145
Eukaryota	Fungi	Neurospora crassa OR74A	XP_960484.2	2 E-13	117/145
Eukaryota	Fungi	Schizosaccharomyces pombe	NP_596564.1	2 E-13	136/145
Eukaryota	Metazoa	Danio rerio	NP_001074272.1	3 E-13	129/145
Eukaryota	Metazoa	Xenopus laevis	NP_001108254.1	3 E-13	122/145
Eukaryota	Metazoa	Salmo salar	NP_001139872.1	4 E-13	126/145
Eukaryota	Metazoa	Canis lupus familiaris	XP_853542.1	5 E-13	118/145
Eukaryota	Viridiplantae	Populus trichocarpa	XP_002306007.1	5 E-13	116/145
Eukaryota	Viridiplantae	Ricinus communis	XP_002518008.1	8 E-13	119/145
Eukaryota	Metazoa	Homo sapiens	NP_060943.1	1 E-12	135/145
Eukaryota	Metazoa	Mus musculus	NP_080066.1	1 E-12	135/145
Eukaryota	Viridiplantae	Arabidopsis thaliana	ABY67504.1	2 E-12	116/145
Eukaryota	Fungi	Aspergillus fumigatus Af293	XP_001481548.1	2 E-12	146/145
Eukaryota	Viridiplantae	Arabidopsis thaliana	AAK32936.1	3 E-12	116/145
Eukaryota	Metazoa	Xenopus (Silurana) tropicalis	NP_001005098.1	3 E-12	122/145
Eukaryota	Viridiplantae	Arabidopsis thaliana	NP_563705.1	3 E-12	116/145
Eukaryota	Viridiplantae	Arabidopsis thaliana	ABY67490.1	3 E-12	116/145
Eukaryota	Fungi	Microsporum canis CBS 113480	EEQ29490.1	3 E-12	129/145
Eukaryota	Viridiplantae	Glycine max	ACU19163.1	4 E-12	118/145
Eukaryota	Viridiplantae	Arabidopsis thaliana	ABY67521.1	5 E-12	116/145
Eukaryota	Metazoa	Nematostella vectensis	XP_001629422.1	5 E-12	137/145
Eukaryota	Metazoa	Rattus norvegicus	NP_001099581.1	6 E-12	135/145
Eukaryota	Fungi	Podospora anserina DSM 980	XP_001905741.1	7 E-12	119/145
Eukaryota	Metazoa	Pan troglodytes	XP_001171713.1	7 E-12	135/145
Eukaryota	Fungi	Schizosaccharomyces japonicus yFS275	XP_002172171.1	1 E-11	136/145
Eukaryota	Metazoa	Ciona intestinalis	XP_002119944.1	4 E-11	121/145
Eukaryota	Metazoa	Culex quinquefasciatus	XP_001866940.1	6 E-11	120/145
Eukaryota	Fungi	Paracoccidioides brasiliensis Pb03;	EEH20510.1	7 E-11	148/145
Eukaryota	Fungi	Paracoccidioides brasiliensis Pb18;	EEH45082.1	8 E-11	148/145

AFUA_2G07450

Eukaryota	Fungi	Aspergillus fumigatus Af293	XP_755081.1	0.0	981/981
Eukaryota	Fungi	Aspergillus terreus NIH2624	XP_001209539.1	0.0	931/981
Eukaryota	Fungi	Aspergillus oryzae RIB40	XP_001826505.1	0.0	971/981
Eukaryota	Fungi	Penicillium chrysogenum Wisconsin 54-1255	XP_002558984.1	0.0	899/981
Eukaryota	Fungi	Aspergillus nidulans FGSC A4	tpeCBF70814.1	1 E-180	954/981
Eukaryota	Fungi	Aspergillus nidulans FGSC A4	XP_663409.1	1 E-179	954/981
Eukaryota	Fungi	Ajellomyces capsulatus G186AR	EEH08671.1	1 E-159	965/981
Eukaryota	Fungi	Ajellomyces capsulatus H143	EER43449.1	1 E-159	965/981
Eukaryota	Fungi	Paracoccidioides brasiliensis Pb01;	EEH37083.1	1 E-158	939/981

Eukaryota	Fungi	Talaromyces stipitatus ATCC 10500	XP_002478732.1	1 E-157	923/981
Eukaryota	Fungi	Ajellomyces dermatitidis ER-3	EEQ83501.1	1 E-156	933/981
Eukaryota	Fungi	Ajellomyces dermatitidis SLH14081	XP_002624950.1	1 E-156	933/981
Eukaryota	Fungi	Paracoccidioides brasiliensis Pb18;	EEH49115.1	1 E-153	941/981
Eukaryota	Fungi	Nectria haematococca mpVI 77-13-4	EEU45492.1	1 E-147	978/981
Eukaryota	Fungi	Microsporum canis CBS 113480	EEQ28476.1	1 E-139	944/981
Eukaryota	Fungi	Uncinocarpus reesii 1704	XP_002584974.1	1 E-134	947/981
Eukaryota	Fungi	Coccidioides posadasii C735 delta	EER26029.1	1 E-132	984/981
Eukaryota	Fungi	Coccidioides immitis RS;	XP_001244796.1	1 E-132	984/981

AFUA_2G07710

Eukaryota	Fungi	Aspergillus fumigatus Af293	XP_755106.1	0.0	1120/1120
Eukaryota	Fungi	Neosartorya fischeri NRRL 181	XP_001260289.1	0.0	1118/1120
Eukaryota	Fungi	Aspergillus niger CBS 513.88	XP_001390603.1	0.0	1128/1120
Eukaryota	Fungi	Aspergillus oryzae RIB40	XP_001826484.1	0.0	1119/1120
Eukaryota	Fungi	Aspergillus clavatus NRRL 1	XP_001267713.1	0.0	1129/1120
Eukaryota	Fungi	Penicillium chrysogenum Wisconsin 54-1255	XP_002558957.1	0.0	1124/1120
Eukaryota	Fungi	Aspergillus nidulans FGSC A4	XP_663431.1	0.0	1128/1120
Eukaryota	Fungi	Aspergillus terreus NIH2624	XP_001209582.1	0.0	1113/1120
Eukaryota	Fungi	Talaromyces stipitatus ATCC 10500	XP_002478754.1	0.0	1136/1120
Eukaryota	Fungi	Ajellomyces capsulatus G186AR	EEH07778.1	0.0	1129/1120
Eukaryota	Fungi	Ajellomyces dermatitidis ER-3	EEQ83450.1	0.0	1138/1120
Eukaryota	Fungi	Ajellomyces capsulatus H143	EER36487.1	0.0	1129/1120
Eukaryota	Fungi	Coccidioides immitis RS;	XP_001247168.1	0.0	1105/1120
Eukaryota	Fungi	Coccidioides posadasii C735 delta	EER24038.1	0.0	1105/1120
Eukaryota	Fungi	Ajellomyces dermatitidis SLH14081	XP_002621583.1	0.0	1116/1120
Eukaryota	Fungi	Uncinocarpus reesii 1704	XP_002541373.1	0.0	1088/1120
Eukaryota	Fungi	Penicillium marneffei ATCC 18224	XP_002146456.1	0.0	1130/1120
Eukaryota	Fungi	Paracoccidioides brasiliensis Pb18;	EEH49213.1	0.0	1119/1120
Eukaryota	Fungi	Microsporum canis CBS 113480	EEQ27381.1	0.0	1093/1120
Eukaryota	Fungi	Paracoccidioides brasiliensis Pb01;	EEH38797.1	0.0	904/1120
Eukaryota	Fungi	Paracoccidioides brasiliensis Pb03;	EEH22456.1	0.0	1092/1120
Eukaryota	Fungi	Nectria haematococca mpVI 77-13-4	EEU34332.1	0.0	1003/1120
Eukaryota	Fungi	Gibberella zeae PH-1	XP_390567.1	0.0	1006/1120
Eukaryota	Fungi	Verticillium albo-atrum VaMs.102	EEY14447.1	0.0	940/1120
Eukaryota	Fungi	Magnaporthe grisea 70-15	XP_367590.1	0.0	1011/1120
Eukaryota	Fungi	Botryotinia fuckeliana B05.10	XP_001556045.1	0.0	985/1120
Eukaryota	Fungi	Sclerotinia sclerotiorum 1980 UF-70	XP_001588001.1	0.0	933/1120
Eukaryota	Fungi	Coprinopsis cinerea okayama7#130	XP_001835890.1	0.0	1059/1120
Eukaryota	Fungi	Ustilago maydis 521	XP_761890.1	0.0	911/1120
Eukaryota	Fungi	Schizosaccharomyces pombe	NP_595686.2	0.0	1046/1120
Eukaryota	Fungi	Cryptococcus neoformans var. neoformans	XP_776430.1	0.0	1052/1120
Eukaryota	Fungi	Schizosaccharomyces japonicus yFS275	XP_002172564.1	0.0	900/1120
Eukaryota	Fungi	Cryptococcus neoformans var. neoformans	XP_569522.1	0.0	1052/1120
Eukaryota	Fungi	Yarrowia lipolytica CLIB122	XP_502638.1	0.0	905/1120
Eukaryota	Viridiplantae	Arabidopsis thaliana	NP_181077.3	0.0	1038/1120
Eukaryota	Viridiplantae	Arabidopsis thaliana	BAE98860.1	0.0	1038/1120
Eukaryota	Metazoa	Danio rerio	NP_956318.1	0.0	1046/1120
Eukaryota	Viridiplantae	Sorghum bicolor;	XP_002457621.1	0.0	1036/1120
Eukaryota	Viridiplantae	Vitis vinifera	XP_002284415.1	0.0	1046/1120
Eukaryota	Viridiplantae	Arabidopsis thaliana	AAC36188.1	0.0	1081/1120
Eukaryota	Metazoa	Sus scrofa	XP_001927998.1	0.0	899/1120
Eukaryota	Metazoa	Equus caballus	XP_001491503.1	0.0	899/1120
Eukaryota	Metazoa	Sus scrofa	NP_001116578.1	0.0	899/1120
Eukaryota	Viridiplantae	Arabidopsis thaliana	AAF81347.1	0.0	918/1120
Eukaryota	Metazoa	Caenorhabditis elegans	NP_497027.1	0.0	941/1120
Eukaryota	Alveolata	Toxoplasma gondii VEG	EEE27953.1	0.0	914/1120

Eukaryota	Alveolata	Toxoplasma gondii GT1	EEE20099.1	0.0	926/1120
Eukaryota	Alveolata	Toxoplasma gondii ME49	XP_002365465.1	0.0	914/1120

AFUA_2G08060

Eukaryota	Fungi	Aspergillus fumigatus Af293	XP_755141.1	0.0	5538/5538
Eukaryota	Fungi	Neosartorya fischeri NRRL 181	XP_001260316.1	0.0	5556/5538

AFUA_2G08670

Eukaryota	Fungi	Aspergillus fumigatus Af293	XP_755201.1	0.0	2292/2292
Eukaryota	Fungi	Neosartorya fischeri NRRL 181	XP_001260373.1	0.0	2291/2292
Eukaryota	Fungi	Aspergillus clavatus NRRL 1	XP_001267796.1	0.0	2292/2292
Eukaryota	Fungi	Aspergillus oryzae RIB40	XP_001826411.1	0.0	2282/2292
Eukaryota	Fungi	Aspergillus niger CBS 513.88	XP_001395476.1	0.0	2281/2292
Eukaryota	Fungi	Aspergillus niger	CAG38356.1	0.0	2277/2292
Eukaryota	Fungi	Aspergillus terreus NIH2624	XP_001218324.1	0.0	2274/2292
Eukaryota	Fungi	Aspergillus nidulans FGSC A4	XP_663730.1	0.0	2287/2292
Eukaryota	Fungi	Penicillium chrysogenum Wisconsin 54-1255	XP_002558828.1	0.0	2265/2292
Eukaryota	Fungi	Aspergillus flavus NRRL3357	XP_002378098.1	0.0	2091/2292
Eukaryota	Fungi	Ajellomyces capsulatus H143	EER45832.1	0.0	2292/2292
Eukaryota	Fungi	Ajellomyces capsulatus G186AR	EEH07675.1	0.0	2292/2292
Eukaryota	Fungi	Paracoccidioides brasiliensis Pb03;	EEH22491.1	0.0	2298/2292
Eukaryota	Fungi	Ajellomyces dermatitidis SLH14081	XP_002621459.1	0.0	2291/2292
Eukaryota	Fungi	Talaromyces stipitatus ATCC 10500	XP_002478864.1	0.0	2283/2292
Eukaryota	Fungi	Penicillium marneffei ATCC 18224	XP_002146561.1	0.0	2288/2292
Eukaryota	Fungi	Coccidioides immitis RS;	XP_001247056.1	0.0	2282/2292
Eukaryota	Fungi	Coccidioides posadasii C735 delta	EER24112.1	0.0	2282/2292
Eukaryota	Fungi	Uncinocarpus reesii 1704	XP_002541275.1	0.0	2284/2292
Eukaryota	Fungi	Paracoccidioides brasiliensis Pb01;	EEH39462.1	0.0	2264/2292
Eukaryota	Fungi	Paracoccidioides brasiliensis Pb18;	EEH49248.1	0.0	2184/2292
Eukaryota	Fungi	Microsporum canis CBS 113480	EEQ31661.1	0.0	2282/2292
Eukaryota	Fungi	Sclerotinia sclerotiorum 1980 UF-70	XP_001592109.1	0.0	2274/2292
Eukaryota	Fungi	Neurospora crassa OR74A	XP_963017.1	0.0	2262/2292
Eukaryota	Fungi	Pyrenophora tritici-repentis Pt-1C-BFP	XP_001932248.1	0.0	2263/2292
Eukaryota	Fungi	Podospora anserina DSM 980	XP_001907634.1	0.0	2270/2292
Eukaryota	Fungi	Nectria haematococca mpVI 77-13-4	EEU39887.1	0.0	2258/2292
Eukaryota	Fungi	Gibberella zeae PH-1	XP_386756.1	0.0	2249/2292
Eukaryota	Fungi	Phaeosphaeria nodorum SN15	XP_001793413.1	0.0	2232/2292
Eukaryota	Fungi	Magnaporthe grisea 70-15	XP_367702.2	0.0	2219/2292
Eukaryota	Fungi	Yarrowia lipolytica CLIB122	XP_501721.1	0.0	2231/2292
Eukaryota	Fungi	Candida albicans SC5314	XP_718624.1	0.0	2204/2292
Eukaryota	Fungi	Candida albicans WO-1	EEQ43196.1	0.0	2204/2292
Eukaryota	Fungi	Candida dubliniensis CD36	XP_002421671.1	0.0	2204/2292
Eukaryota	Fungi	Pichia guilliermondii ATCC 6260	EDK36707.2	0.0	2205/2292
Eukaryota	Fungi	Schizosaccharomyces japonicus yFS275	XP_002174469.1	0.0	2245/2292
Eukaryota	Fungi	Pichia guilliermondii ATCC 6260	XP_001487428.1	0.0	2205/2292
Eukaryota	Fungi	Candida tropicalis MYA-3404	XP_002546225.1	0.0	2202/2292
Eukaryota	Fungi	Debaryomyces hansenii CBS767	XP_457211.1	0.0	2202/2292
Eukaryota	Fungi	Lodderomyces elongisporus NRRL YB-4239	XP_001528007.1	0.0	2201/2292
Eukaryota	Fungi	Pichia stipitis CBS 6054	XP_001386775.1	0.0	2205/2292
Eukaryota	Fungi	Clavispora lusitaniae ATCC 42720	XP_002616419.1	0.0	2203/2292
Eukaryota	Fungi	Schizosaccharomyces pombe	NP_593271.1	0.0	2247/2292
Eukaryota	Fungi	Vanderwaltozyma polyspora DSM 70294	XP_001647339.1	0.0	2213/2292
Eukaryota	Fungi	Lachancea thermotolerans CBS 6340	XP_002551722.1	0.0	2211/2292
Eukaryota	Fungi	Schizosaccharomyces pombe	BAA11238.1	0.0	2246/2292
Eukaryota	Fungi	Candida glabrata CBS 138	XP_449236.1	0.0	2215/2292
Eukaryota	Fungi	Ashbya gossypii ATCC 10895	NP_982612.1	0.0	2209/2292

Eukaryota	Fungi	Saccharomyces cerevisiae YJM789	EDN62822.1	0.0	2215/2292
Eukaryota	Fungi	Saccharomyces cerevisiae	NP_014413.1	0.0	2215/2292
Eukaryota	Fungi	Saccharomyces cerevisiae EC1118	CAY82209.1	0.0	2215/2292
Eukaryota	Fungi	Pichia pastoris GS115	XP_002490365.1	0.0	2203/2292
Eukaryota	Fungi	Kluyveromyces lactis NRRL Y-1140	XP_455355.1	0.0	2208/2292
Eukaryota	Fungi	Zygosaccharomyces rouxii CBS 732	XP_002496821.1	0.0	2213/2292
Eukaryota	Fungi	Saccharomyces cerevisiae	AAA20073.1	0.0	2219/2292
Eukaryota	Fungi	Zygosaccharomyces rouxii	CAQ43571.1	0.0	2213/2292
Eukaryota	Fungi	Saccharomyces cerevisiae AWRI1631	EDZ69596.1	0.0	2021/2292
Eukaryota	Fungi	Amylomyces rouxii	ABQ28729.1	0.0	2209/2292
Eukaryota	Fungi	Laccaria bicolor S238N-H82	XP_001875210.1	0.0	2116/2292
Eukaryota	Fungi	Cryptococcus neoformans var. neoformans	XP_571316.1	0.0	2229/2292
Eukaryota	Fungi	Cryptococcus neoformans var. neoformans	XP_774823.1	0.0	2229/2292
Eukaryota	Fungi	Ustilago maydis	CAA86983.1	0.0	2167/2292
Eukaryota	Fungi	Ustilago maydis 521	XP_760776.1	0.0	2167/2292
Eukaryota	Fungi	Saccharomyces cerevisiae	BAA24410.1	0.0	2150/2292
Eukaryota	Fungi	Saccharomyces cerevisiae	P32874.2	0.0	2150/2292
Eukaryota	Fungi	Saccharomyces cerevisiae YJM789	EDN64143.1	0.0	2099/2292
Eukaryota	Fungi	Saccharomyces cerevisiae EC1118	CAY82038.1	0.0	2099/2292
Eukaryota	Fungi	Saccharomyces cerevisiae JAY291	EEU06674.1	0.0	2099/2292
Eukaryota	Fungi	Saccharomyces cerevisiae	NP_013934.1	0.0	2099/2292
Eukaryota	Fungi	Saccharomyces cerevisiae RM11-1a	EDV11698.1	0.0	2099/2292
Eukaryota	Fungi	Saccharomyces cerevisiae AWRI1631	EDZ70018.1	0.0	2002/2292
Eukaryota	Metazoa	Danio rerio	XP_001919815.1	0.0	2240/2292
Eukaryota	Metazoa	Gallus gallus	NP_990836.1	0.0	2215/2292
Eukaryota	Metazoa	Canis lupus familiaris	XP_548250.2	0.0	2215/2292
Eukaryota	Metazoa	Xenopus (Silurana) tropicalis	NP_001131086.1	0.0	2215/2292
Eukaryota	Metazoa	Canis lupus familiaris	XP_867576.1	0.0	2215/2292
Eukaryota	Metazoa	Canis lupus familiaris	XP_867544.1	0.0	2210/2292
Eukaryota	Metazoa	Canis lupus familiaris	XP_867594.1	0.0	2215/2292
Eukaryota	Metazoa	Homo sapiens	O00763.2	0.0	2207/2292
Eukaryota	Metazoa	Homo sapiens	NP_001084.3	0.0	2207/2292
Eukaryota	Metazoa	Macaca mulatta	XP_001111634.1	0.0	2219/2292
Eukaryota	Metazoa	Homo sapiens	ABF48723.1	0.0	2207/2292
Eukaryota	Metazoa	Macaca mulatta	XP_001111672.1	0.0	2219/2292
Eukaryota	Metazoa	Homo sapiens	AAR37018.1	0.0	2207/2292
Eukaryota	Metazoa	Canis lupus familiaris	XP_867536.1	0.0	2209/2292
Eukaryota	Metazoa	Ovis aries	NP_001009256.1	0.0	2238/2292
Eukaryota	Metazoa	Monodelphis domestica	XP_001371374.1	0.0	2238/2292
Eukaryota	Metazoa	Homo sapiens	NP_942134.1	0.0	2238/2292
Eukaryota	Metazoa	Mus musculus	NP_598665.2	0.0	2207/2292
Eukaryota	Metazoa	Macaca mulatta	XP_001111553.1	0.0	2238/2292
Eukaryota	Metazoa	Homo sapiens	NP_942135.1	0.0	2238/2292
Eukaryota	Metazoa	Equus caballus	XP_001918337.1	0.0	2238/2292
Eukaryota	Metazoa	Macaca mulatta	XP_001111746.1	0.0	2238/2292
Eukaryota	Metazoa	Homo sapiens	NP_942133.1	0.0	2238/2292
Eukaryota	Metazoa	Canis lupus familiaris	XP_867554.1	0.0	2208/2292
Eukaryota	Metazoa	Bos taurus	NP_776649.1	0.0	2238/2292
Eukaryota	Metazoa	Homo sapiens	NP_942131.1	0.0	2238/2292
Eukaryota	Metazoa	Macaca mulatta	XP_001111823.1	0.0	2238/2292
Eukaryota	Metazoa	Sus scrofa	ABQ85554.1	0.0	2238/2292
Eukaryota	Metazoa	Pan troglodytes	XP_511428.2	0.0	2238/2292
Eukaryota	Metazoa	Homo sapiens	AAP94122.1	0.0	2238/2292
Eukaryota	Metazoa	Mus musculus	NP_579938.2	0.0	2238/2292
Eukaryota	Metazoa	Sus scrofa	NP_001107741.1	0.0	2238/2292
Eukaryota	Metazoa	Canis lupus familiaris	XP_867566.1	0.0	2225/2292
Eukaryota	Metazoa	Mus musculus	AAS13686.1	0.0	2207/2292
Eukaryota	Metazoa	Homo sapiens	pirS41121	0.0	2230/2292

Eukaryota	Metazoa	Rattus norvegicus	ABF48724.1	0.0	2207/2292
Eukaryota	Metazoa	Mus musculus	AAS13685.1	0.0	2238/2292
Eukaryota	Metazoa	Taeniopygia guttata	XP_002199465.1	0.0	2225/2292
Eukaryota	Metazoa	Mus musculus	EDL15735.1	0.0	2175/2292
Eukaryota	Metazoa	Equus caballus	XP_001496980.1	0.0	2207/2292
Eukaryota	Metazoa	Bos taurus	XP_873014.3	0.0	2207/2292
Eukaryota	Metazoa	Culex quinquefasciatus	XP_001847001.1	0.0	2220/2292
Eukaryota	Metazoa	Anopheles gambiae str. PEST	XP_001688518.1	0.0	2214/2292
Eukaryota	Metazoa	Ornithorhynchus anatinus	XP_001508230.1	0.0	2084/2292
Eukaryota	Metazoa	Sus scrofa	ACL80208.1	0.0	2084/2292
Eukaryota	Metazoa	Sus scrofa	XP_001929305.1	0.0	2207/2292
Eukaryota	Metazoa	Rattus norvegicus	NP_071529.1	0.0	2238/2292
Eukaryota	Metazoa	Danio rerio	XP_684081.3	0.0	2209/2292
Eukaryota	Metazoa	Danio rerio	CAM14140.1	0.0	2209/2292
Eukaryota	Metazoa	Aedes aegypti	XP_001651879.1	0.0	2217/2292
Eukaryota	Metazoa	Rattus norvegicus	EDM13946.1	0.0	2077/2292
Eukaryota	Metazoa	Homo sapiens	AAC50139.1	0.0	2238/2292
Eukaryota	Metazoa	Tetraodon nigroviridis	CAG08536.1	0.0	2176/2292
Eukaryota	Metazoa	Pediculus humanus corporis	XP_002429216.1	0.0	2210/2292
Eukaryota	Metazoa	Trichoplax adhaerens	XP_002111053.1	0.0	2115/2292
Eukaryota	Metazoa	Acyrthosiphon pisum	XP_001946412.1	0.0	2113/2292
Eukaryota	Metazoa	Homo sapiens	AAB58382.1	0.0	2233/2292
Eukaryota	Metazoa	Tribolium castaneum	XP_969851.2	0.0	2222/2292
Eukaryota	Metazoa	Drosophila sechellia	XP_002032844.1	0.0	2193/2292
Eukaryota	Metazoa	Drosophila erecta	XP_001970537.1	0.0	2193/2292
Eukaryota	Metazoa	Drosophila melanogaster	NP_001097227.1	0.0	2193/2292
Eukaryota	Metazoa	Drosophila melanogaster	NP_724636.1	0.0	2193/2292
Eukaryota	Metazoa	Drosophila yakuba	XP_002089560.1	0.0	2193/2292
Eukaryota	Metazoa	Drosophila ananassae	XP_001961005.1	0.0	2192/2292
Eukaryota	Metazoa	Drosophila melanogaster	NP_610342.1	0.0	2193/2292
Eukaryota	Metazoa	Drosophila mojavensis	XP_002005267.1	0.0	2204/2292
Eukaryota	Metazoa	Drosophila grimshawi	XP_001987388.1	0.0	2204/2292
Eukaryota	Metazoa	Drosophila willistoni	XP_002063097.1	0.0	2194/2292
Eukaryota	Metazoa	Drosophila virilis	XP_002050377.1	0.0	2194/2292
Eukaryota	Metazoa	Drosophila pseudoobscura pseudoobscura	XP_001360655.2	0.0	2193/2292
Eukaryota	Metazoa	Rattus norvegicus	NP_446374.1	0.0	2204/2292
Eukaryota	Metazoa	Nasonia vitripennis	XP_001606974.1	0.0	2113/2292
Eukaryota	Metazoa	Apis mellifera	XP_624665.1	0.0	2084/2292
Eukaryota	Metazoa	Drosophila persimilis	XP_002016234.1	0.0	2179/2292
Eukaryota	Metazoa	Rattus norvegicus	EDM05509.1	0.0	2138/2292
Eukaryota	Metazoa	Rattus norvegicus	EDM05508.1	0.0	2075/2292
Eukaryota	Amoebozoa	Dictyostelium discoideum AX4	XP_636722.1	0.0	2273/2292
Eukaryota	Choanoflagellida	Monosiga brevicollis MX1	XP_001748674.1	0.0	2069/2292
Eukaryota	Viridiplantae	Oryza sativa Japonica Group	AAM18728.1	0.0	2251/2292
Eukaryota	Viridiplantae	Physcomitrella patens subsp. patens	XP_001773073.1	0.0	2147/2292
Eukaryota	Viridiplantae	Arabidopsis thaliana	NP_174849.2	0.0	2250/2292
Eukaryota	Viridiplantae	Arabidopsis thaliana	AAG40563.1	0.0	2250/2292
Eukaryota	Viridiplantae	Vitis vinifera	XP_002285808.1	0.0	2156/2292
Eukaryota	Viridiplantae	Triticum urartu;	ACD46670.1	0.0	2241/2292
Eukaryota	Viridiplantae	Physcomitrella patens subsp. patens	XP_001754424.1	0.0	2143/2292
Eukaryota	Viridiplantae	Arabidopsis thaliana	AAF18638.2	0.0	2253/2292
Eukaryota	Viridiplantae	Arabidopsis thaliana	AAG40564.1	0.0	2246/2292
Eukaryota	Viridiplantae	Aegilops tauschii;	ACD46664.1	0.0	2242/2292
Eukaryota	Viridiplantae	Triticum aestivum;	ACD46674.1	0.0	2241/2292
Eukaryota	Viridiplantae	Triticum aestivum;	ACD46685.1	0.0	2177/2292
Eukaryota	Viridiplantae	Ricinus communis	XP_002513881.1	0.0	2135/2292
Eukaryota	Viridiplantae	Aegilops tauschii;	ACD46679.1	0.0	2177/2292
Eukaryota	Viridiplantae	Triticum urartu;	ACD46677.1	0.0	2177/2292

Eukaryota	Viridiplantae	Arabidopsis thaliana	NP_174850.3	0.0	2247/2292
Eukaryota	Viridiplantae	Triticum aestivum;	AAC39330.1	0.0	2177/2292
Eukaryota	Viridiplantae	Triticum aestivum;	ACD46683.1	0.0	2177/2292
Eukaryota	Viridiplantae	Triticum turgidum subsp. durum	ACD46681.1	0.0	2177/2292
Eukaryota	Viridiplantae	Arabidopsis thaliana	AAG51252.1	0.0	2250/2292
Eukaryota	Viridiplantae	Triticum turgidum subsp. durum	ACD46682.1	0.0	2177/2292
Eukaryota	Viridiplantae	Glycine max	AAA75528.1	0.0	2140/2292
Eukaryota	Viridiplantae	Zea mays;	AAP78896.1	0.0	2108/2292
Eukaryota	stramenopiles	Thalassiosira pseudonana CCMP1335	XP_002287470.1	0.0	2247/2292
Eukaryota	Viridiplantae	Alopecurus myosuroides;	CAC84161.1	0.0	2100/2292
Eukaryota	Viridiplantae	Alopecurus myosuroides;	CAL63610.1	0.0	2100/2292
Eukaryota	Viridiplantae	Setaria italica;	AAO62903.1	0.0	2106/2292
Eukaryota	Viridiplantae	Setaria italica;	AAL02056.1	0.0	2106/2292
Eukaryota	Viridiplantae	Setaria viridis;	CAL63609.1	0.0	2106/2292
Eukaryota	Viridiplantae	Alopecurus myosuroides;	CAL63611.1	0.0	2100/2292
Eukaryota	Viridiplantae	Triticum aestivum;	ACD46686.1	0.0	2242/2292
Eukaryota	Viridiplantae	Triticum aestivum;	ACD46667.1	0.0	2242/2292
Eukaryota	Viridiplantae	Sorghum bicolor;	XP_002442242.1	0.0	2223/2292
Eukaryota	Viridiplantae	Zea mays;	AAP78897.1	0.0	2109/2292
Eukaryota	Viridiplantae	Setaria italica;	AAO62902.1	0.0	2106/2292
Eukaryota	Viridiplantae	Sorghum bicolor;	XP_002446178.1	0.0	2130/2292
Eukaryota	Viridiplantae	Triticum aestivum;	AAC49275.1	0.0	2241/2292
Eukaryota	Viridiplantae	Zea mays;	NP_001105373.1	0.0	2109/2292
Eukaryota	Viridiplantae	Triticum aestivum;	AAA19970.1	0.0	2241/2292
Eukaryota	Viridiplantae	Triticum turgidum subsp. durum	ACD46672.1	0.0	2233/2292
Eukaryota	Viridiplantae	Oryza sativa Indica Group	EAY97401.1	0.0	2145/2292
Eukaryota	Viridiplantae	Populus trichocarpa	XP_002302277.1	0.0	2143/2292
Eukaryota	Viridiplantae	Brassica napus	CAA54683.1	0.0	2158/2292
Eukaryota	Viridiplantae	Oryza sativa Japonica Group	AAP53321.2	0.0	2213/2292
Eukaryota	Viridiplantae	Brassica napus	CAC19875.1	0.0	2204/2292
Eukaryota	Viridiplantae	Oryza sativa Japonica Group	EEE50826.1	0.0	2198/2292
Eukaryota	Viridiplantae	Oryza sativa Japonica Group	EEE63127.1	0.0	2132/2292
Eukaryota	Viridiplantae	Medicago sativa	AAB42144.1	0.0	2141/2292
Eukaryota	Viridiplantae	Populus trichocarpa	XP_002306591.1	0.0	2214/2292
Eukaryota	stramenopiles	Phaeodactylum tricornutum CCAP 1055/1	XP_002185458.1	0.0	2249/2292
Eukaryota	Metazoa	Schistosoma mansoni	XP_002571765.1	0.0	2177/2292
Eukaryota	Viridiplantae	Ostreococcus lucimarinus CCE9901	XP_001415874.1	0.0	1932/2292
Eukaryota	Viridiplantae	Vitis vinifera	CAN64563.1	0.0	2077/2292
Eukaryota	Viridiplantae	Glycine max	AAA81578.1	0.0	1958/2292
Eukaryota	Viridiplantae	Arabidopsis thaliana	AAG51250.1	0.0	1865/2292

AFUA_2G09490

Eukaryota	Fungi	Aspergillus fumigatus Af293	XP_755283.1	0.0	1525/1525
Eukaryota	Fungi	Neosartorya fischeri NRRL 181	XP_001260442.1	0.0	1527/1525
Eukaryota	Fungi	Aspergillus clavatus NRRL 1	XP_001267866.1	0.0	1545/1525
Eukaryota	Fungi	Aspergillus oryzae RIB40	XP_001826305.1	0.0	1517/1525
Eukaryota	Fungi	Aspergillus nidulans FGSC A4	tpeCBF70267.1	0.0	1518/1525
Eukaryota	Fungi	Aspergillus flavus NRRL3357	XP_002377995.1	0.0	1315/1525
Eukaryota	Fungi	Aspergillus niger CBS 513.88	XP_001397987.1	0.0	1315/1525
Eukaryota	Fungi	Aspergillus nidulans FGSC A4	XP_663664.1	0.0	1447/1525
Eukaryota	Fungi	Talaromyces stipitatus ATCC 10500	XP_002482542.1	0.0	1497/1525
Eukaryota	Fungi	Penicillium marneffei ATCC 18224	XP_002148280.1	0.0	1495/1525
Eukaryota	Fungi	Ajellomyces capsulatus H143	EER40362.1	0.0	1516/1525
Eukaryota	Fungi	Paracoccidioides brasiliensis Pb18;	EEH44168.1	0.0	1528/1525
Eukaryota	Fungi	Ajellomyces capsulatus G186AR	EEH03073.1	0.0	1516/1525
Eukaryota	Fungi	Ajellomyces capsulatus NAm1	XP_001537100.1	0.0	1512/1525
Eukaryota	Fungi	Paracoccidioides brasiliensis Pb01;	EEH35473.1	0.0	1530/1525

Eukaryota	Fungi	Paracoccidioides brasiliensis Pb03;	EEH19800.1	0.0	1448/1525
Eukaryota	Fungi	Penicillium chrysogenum Wisconsin 54-1255	XP_002565819.1	0.0	1272/1525
Eukaryota	Fungi	Uncinocarpus reesii 1704	XP_002542660.1	0.0	1454/1525
Eukaryota	Fungi	Microsporum canis CBS 113480	EEQ31944.1	0.0	1516/1525
Eukaryota	Fungi	Ajellomyces dermatitidis SLH14081	XP_002622462.1	0.0	1506/1525
Eukaryota	Fungi	Ajellomyces dermatitidis ER-3	EEQ92044.1	0.0	1507/1525
Eukaryota	Fungi	Nectria haematococca mpVI 77-13-4	EEU43103.1	0.0	1525/1525
Eukaryota	Fungi	Gibberella zeae PH-1	XP_385598.1	0.0	1510/1525
Eukaryota	Fungi	Magnaporthe grisea 70-15	XP_369881.2	0.0	1456/1525

AFUA_2G10360

Eukaryota	Fungi	Aspergillus fumigatus Af293	XP_755369.1	0.0	509/509
Eukaryota	Fungi	Neosartorya fischeri NRRL 181	XP_001260518.1	0.0	487/509
Eukaryota	Fungi	Aspergillus clavatus NRRL 1	XP_001275335.1	0.0	485/509
Eukaryota	Fungi	Aspergillus flavus NRRL3357	XP_002377888.1	0.0	487/509
Eukaryota	Fungi	Aspergillus oryzae RIB40	XP_001826210.1	0.0	487/509
Eukaryota	Fungi	Aspergillus nidulans FGSC A4	XP_663556.1	0.0	483/509
Eukaryota	Fungi	Penicillium chrysogenum Wisconsin 54-1255	XP_002565957.1	0.0	484/509
Eukaryota	Fungi	Aspergillus terreus NIH2624	XP_001213652.1	0.0	467/509
Eukaryota	Fungi	Aspergillus niger CBS 513.88	XP_001397772.1	0.0	453/509
Eukaryota	Fungi	Talaromyces stipitatus ATCC 10500	XP_002480950.1	0.0	490/509
Eukaryota	Fungi	Ajellomyces dermatitidis SLH14081	XP_002622571.1	0.0	468/509
Eukaryota	Fungi	Ajellomyces dermatitidis ER-3	EEQ92146.1	0.0	468/509
Eukaryota	Fungi	Coccidioides immitis RS;	XP_001244916.1	0.0	463/509
Eukaryota	Fungi	Coccidioides posadasii C735 delta	EER29692.1	0.0	463/509
Eukaryota	Fungi	Paracoccidioides brasiliensis Pb01;	EEH38957.1	0.0	466/509
Eukaryota	Fungi	Paracoccidioides brasiliensis Pb18;	EEH44060.1	0.0	466/509
Eukaryota	Fungi	Paracoccidioides brasiliensis Pb03;	EEH19706.1	0.0	466/509
Eukaryota	Fungi	Ajellomyces capsulatus G186AR	EEH04472.1	0.0	470/509
Eukaryota	Fungi	Uncinocarpus reesii 1704	XP_002542086.1	1 E-180	460/509
Eukaryota	Fungi	Microsporum canis CBS 113480	EEQ27321.1	1 E-157	460/509
Eukaryota	Fungi	Sclerotinia sclerotiorum 1980 UF-70	XP_001590483.1	1 E-151	446/509
Eukaryota	Fungi	Botryotinia fuckeliana B05.10	XP_001557886.1	1 E-150	445/509
Eukaryota	Fungi	Aspergillus flavus NRRL3357	XP_002374095.1	1 E-147	449/509
Eukaryota	Fungi	Aspergillus oryzae RIB40	XP_001820319.1	1 E-146	449/509
Eukaryota	Fungi	Phaeosphaeria nodorum SN15	XP_001798697.1	1 E-146	467/509
Eukaryota	Fungi	Pyrenophora tritici-repentis Pt-1C-BFP	XP_001939020.1	1 E-145	488/509
Eukaryota	Fungi	Nectria haematococca mpVI 77-13-4	EEU35954.1	1 E-143	471/509
Eukaryota	Fungi	Gibberella zeae PH-1	XP_385005.1	1 E-142	482/509
Eukaryota	Fungi	Aspergillus terreus NIH2624	XP_001210844.1	1 E-139	446/509
Eukaryota	Fungi	Aspergillus clavatus NRRL 1	XP_001271861.1	1 E-139	449/509
Eukaryota	Fungi	Penicillium marneffei ATCC 18224	XP_002149280.1	1 E-138	443/509
Eukaryota	Fungi	Verticillium albo-atrum VaMs.102	EEY23556.1	1 E-138	458/509
Eukaryota	Fungi	Aspergillus nidulans FGSC A4	tpeCBF85703.1	1 E-137	447/509
Eukaryota	Fungi	Penicillium chrysogenum Wisconsin 54-1255	XP_002565133.1	1 E-137	447/509
Eukaryota	Fungi	Aspergillus nidulans FGSC A4	XP_659461.1	1 E-136	443/509
Eukaryota	Fungi	Neurospora crassa OR74A	XP_959093.1	1 E-135	456/509
Eukaryota	Fungi	Talaromyces stipitatus ATCC 10500	XP_002484882.1	1 E-133	436/509
Eukaryota	Fungi	Neosartorya fischeri NRRL 181	XP_001267030.1	1 E-133	449/509
Eukaryota	Fungi	Aspergillus fumigatus Af293	XP_751842.1	1 E-133	448/509
Eukaryota	Fungi	Nectria haematococca mpVI 77-13-4	EEU39105.1	1 E-132	454/509
Eukaryota	Fungi	Neurospora crassa OR74A	XP_956782.2	1 E-129	459/509
Eukaryota	Fungi	Aspergillus niger CBS 513.88	XP_001402081.1	1 E-127	440/509
Eukaryota	Fungi	Podospora anserina DSM 980	XP_001907979.1	1 E-126	465/509
Eukaryota	Fungi	Chaetomium globosum CBS 148.51	XP_001229573.1	1 E-125	436/509
Eukaryota	Fungi	Gibberella zeae PH-1	XP_384307.1	1 E-124	448/509
Eukaryota	Fungi	Magnaporthe grisea 70-15	XP_361892.1	1 E-124	474/509

Eukaryota	Fungi	Podospora anserina DSM 980	XP_001905477.1	1 E-122	444/509
Eukaryota	Fungi	Pyrenophora tritici-repentis Pt-1C-BFP	XP_001932253.1	1 E-122	444/509
Eukaryota	Fungi	Phaeosphaeria nodorum SN15	XP_001793409.1	1 E-118	444/509
Eukaryota	Metazoa	Danio rerio	XP_700804.3	1 E-111	425/509
Eukaryota	Fungi	Chaetomium globosum CBS 148.51	XP_001221498.1	1 E-107	510/509
Eukaryota	Metazoa	Gallus gallus	XP_422147.1	1 E-106	428/509
Eukaryota	Metazoa	Nematostella vectensis	XP_001630669.1	1 E-106	446/509
Eukaryota	Metazoa	Branchiostoma floridae	XP_002593904.1	1 E-104	428/509
Eukaryota	Choanoflagellida	Monosiga brevicollis MX1	XP_001747110.1	1 E-104	433/509
Bacteria	Proteobacteria	Kangiella koreensis DSM 16069	YP_003145565.1	1 E-103	416/509
Eukaryota	Fungi	Pichia stipitis CBS 6054	XP_001383218.1	1 E-102	456/509
Eukaryota	Fungi	Pichia guilliermondii ATCC 6260	XP_001483663.1	1 E-102	411/509
Eukaryota	Fungi	Candida albicans SC5314	XP_711911.1	1 E-102	435/509
Eukaryota	Fungi	Candida albicans WO-1	EEQ42368.1	1 E-102	435/509
Bacteria	Acidobacteria	Candidatus Koribacter versatilis Ellin345	YP_589759.1	1 E-102	410/509
Eukaryota	Fungi	Candida dubliniensis CD36	XP_002417446.1	1 E-102	422/509
Eukaryota	Fungi	Lachancea thermotolerans CBS 6340	XP_002554552.1	1 E-101	451/509
Eukaryota	Fungi	Candida tropicalis MYA-3404	XP_002548865.1	1 E-100	440/509
Eukaryota	Fungi	Debaryomyces hansenii CBS767	XP_462152.1	1 E-100	451/509
Bacteria	Bacteroidetes	Flavobacteria bacterium BBFL7	ZP_01200814.1	1 E-100	410/509
Bacteria	Proteobacteria	Stigmatella aurantiaca DW4/3-1	ZP_01464021.1	2 E-99	411/509
Bacteria	Bacteroidetes	Flavobacterium johnsoniae UW101	YP_001192860.1	3 E-99	412/509
Eukaryota	Fungi	Clavispora lusitaniae ATCC 42720	XP_002616657.1	5 E-99	440/509
Eukaryota	Fungi	Vanderwaltozyma polyspora DSM 70294	XP_001643105.1	7 E-99	431/509
Bacteria	Bacteroidetes	Flavobacteriales bacterium ALC-1	ZP_02182053.1	8 E-99	409/509
Eukaryota	Metazoa	Branchiostoma floridae	XP_002587535.1	1 E-98	430/509
Bacteria	Proteobacteria	Myxococcus xanthus DK 1622	YP_629178.1	2 E-98	408/509
Bacteria	Bacteroidetes	Psychroflexus torquis ATCC 700755	ZP_01254382.1	1 E-97	410/509
Eukaryota	Metazoa	Mus musculus	NP_081828.1	2 E-97	432/509
Eukaryota	Metazoa	Mus musculus	Q9CXF0.3	2 E-97	432/509
Bacteria	Bacteroidetes	Polaribacter filamentus	AAQ86995.1	1 E-96	408/509
Eukaryota	Metazoa	Bos taurus	XP_001788737.1	3 E-96	425/509
Eukaryota	Metazoa	Canis lupus familiaris	XP_541027.2	6 E-96	432/509
Bacteria	Bacteroidetes	Algoriphagus sp. PR1	ZP_01718885.1	7 E-96	419/509
Eukaryota	Metazoa	Homo sapiens	BAF84784.1	1 E-95	432/509
Eukaryota	Metazoa	Rattus sp. Sciurognathi; Muroidea;	AAB35497.1	1 E-95	430/509
Eukaryota	Metazoa	Rattus norvegicus	P70712.2	2 E-95	430/509
Eukaryota	Metazoa	Rattus norvegicus	NP_446354.1	2 E-95	430/509
Eukaryota	Fungi	Saccharomyces cerevisiae JAY291	EEU06787.1	2 E-95	451/509
Eukaryota	Metazoa	Pan troglodytes	XP_515818.2	3 E-95	432/509
Eukaryota	Fungi	Saccharomyces cerevisiae	NP_013332.1	3 E-95	451/509
Eukaryota	Fungi	Saccharomyces cerevisiae YJM789	EDN59450.1	4 E-95	451/509
Eukaryota	Metazoa	Homo sapiens	NP_003928.1	4 E-95	432/509
Bacteria	Proteobacteria	Shewanella woodyi ATCC 51908	YP_001759792.1	4 E-95	409/509
Eukaryota	Metazoa	Homo sapiens	BAG37742.1	4 E-95	432/509
Bacteria	Bacteroidetes	Polaribacter sp. MED152	ZP_05108319.1	4 E-95	408/509
Eukaryota	Metazoa	Homo sapiens	BAF83589.1	8 E-95	432/509
Eukaryota	Amoebozoa	Dictyostelium discoideum AX4	XP_638641.1	1 E-94	428/509
Eukaryota	Metazoa	Homo sapiens	BAD97146.1	2 E-94	432/509
Bacteria	Bacteroidetes	Gramella forsetii KT0803	YP_862743.1	8 E-94	412/509
Eukaryota	Metazoa	Monodelphis domestica	XP_001371113.1	1 E-93	435/509
Eukaryota	Metazoa	Ciona intestinalis	XP_002119397.1	3 E-93	420/509
Eukaryota	Metazoa	Trichoplax adhaerens	XP_002114057.1	3 E-93	434/509
Bacteria	Bacteroidetes	Chitinophaga pinensis DSM 2588	YP_003120846.1	8 E-93	408/509
Bacteria	Bacteroidetes	Flavobacterium psychrophilum JIP02/86	YP_001297072.1	3 E-92	411/509
Bacteria	Proteobacteria	Xanthomonas campestris pv. vesicatoria	YP_363373.1	4 E-92	411/509
Bacteria	Bacteroidetes	Flavobacteria bacterium BAL38	ZP_01733380.1	2 E-91	417/509
Eukaryota	Fungi	Lodderomyces elongisporus NRRL YB-4239	XP_001528120.1	2 E-91	410/509

Eukaryota	Metazoa	Equus caballus	XP_001490835.1	2 E-91	432/509
Bacteria	Bacteroidetes	Pedobacter sp. BAL39	ZP_01882509.1	1 E-90	412/509
Bacteria	Proteobacteria	Stenotrophomonas sp. SKA14	ZP_05135633.1	2 E-88	412/509
Bacteria	Proteobacteria	Stenotrophomonas maltophilia K279a	YP_001972895.1	9 E-88	412/509
Bacteria	Proteobacteria	Stenotrophomonas maltophilia R551-3	YP_002028981.1	1 E-87	412/509
Bacteria	Bacteroidetes	Cytophaga hutchinsonii ATCC 33406	YP_679960.1	1 E-86	411/509
Bacteria	Bacteroidetes	Flavobacteria bacterium MS024-3C	ZP_03701382.1	2 E-84	418/509
Eukaryota	Metazoa	Hydra magnipapillata	XP_002168253.1	1 E-81	451/509
Eukaryota	Metazoa	Schistosoma mansoni	XP_002573785.1	2 E-81	468/509
Eukaryota	Fungi	Ustilago maydis 521	XP_760993.1	2 E-79	510/509
Eukaryota	Euglenozoa	Trypanosoma cruzi strain CL	XP_816513.1	2 E-76	430/509
Eukaryota	Metazoa	Schistosoma japonicum	Q5DGJ1.1	3 E-76	417/509
Eukaryota	Euglenozoa	Trypanosoma cruzi strain CL	XP_807119.1	1 E-75	430/509
Eukaryota	Euglenozoa	Trypanosoma brucei gambiense DAL972	CBH13998.1	3 E-69	433/509
Eukaryota	Metazoa	Ciona intestinalis	XP_002126394.1	3 E-69	431/509
Eukaryota	Euglenozoa	Trypanosoma brucei TREU927	XP_803513.1	6 E-68	429/509
Eukaryota	Euglenozoa	Leishmania braziliensis MHOM/BR/75/M2904	XP_001562446.1	8 E-68	444/509
Eukaryota	Euglenozoa	Leishmania infantum JPCM5	XP_001470562.1	9 E-65	440/509
Eukaryota	Euglenozoa	Leishmania major strain Friedlin	XP_001684230.1	2 E-62	440/509
Bacteria	Firmicutes	Clostridium hylemonae DSM 15053	ZP_03778242.1	1 E-55	410/509

AFUA_2G10370

Eukaryota	Fungi	Aspergillus fumigatus Af293	XP_755370.1	1 E-155	267/267

AFUA_2G12700

Eukaryota	Fungi	Aspergillus fumigatus Af293	XP_755601.1	0.0	621/621
Eukaryota	Fungi	Neosartorya fischeri NRRL 181	XP_001260726.1	0.0	610/621
Eukaryota	Fungi	Aspergillus clavatus NRRL 1	XP_001275552.1	0.0	580/621
Eukaryota	Fungi	Ajellomyces dermatitidis ER-3	EEQ88711.1	0.0	582/621
Eukaryota	Fungi	Ajellomyces dermatitidis SLH14081	XP_002627054.1	1 E-180	582/621
Eukaryota	Fungi	Microsporum canis CBS 113480	EEQ31190.1	1 E-177	559/621
Eukaryota	Fungi	Nectria haematococca mpVI 77-13-4	EEU33927.1	1 E-149	565/621

AFUA_2G12710

Eukaryota	Fungi	Aspergillus fumigatus Af293	XP_755602.1	0.0	419/419
Eukaryota	Fungi	Neosartorya fischeri NRRL 181	XP_001260727.1	0.0	403/419
Eukaryota	Fungi	Aspergillus clavatus NRRL 1	XP_001275551.1	0.0	388/419
Eukaryota	Fungi	Nectria haematococca mpVI 77-13-4	EEU34079.1	1 E-125	390/419
Eukaryota	Fungi	Penicillium chrysogenum Wisconsin 54-1255	XP_002563959.1	1 E-122	385/419
Eukaryota	Fungi	Aspergillus niger CBS 513.88	XP_001390756.1	1 E-118	386/419
Eukaryota	Fungi	Penicillium marneffei ATCC 18224	XP_002153522.1	1 E-117	391/419
Eukaryota	Fungi	Talaromyces stipitatus ATCC 10500	XP_002487895.1	1 E-114	393/419
Eukaryota	Fungi	Neosartorya fischeri NRRL 181	XP_001258636.1	1 E-101	394/419
Eukaryota	Fungi	Aspergillus nidulans FGSC A4	XP_682058.1	5 E-99	385/419
Eukaryota	Fungi	Aspergillus niger CBS 513.88	XP_001400794.1	4 E-97	390/419
Eukaryota	Fungi	Aspergillus terreus NIH2624	XP_001217806.1	2 E-95	388/419
Eukaryota	Fungi	Verticillium albo-atrum VaMs.102	EEY17956.1	9 E-89	354/419
Eukaryota	Fungi	Ajellomyces dermatitidis SLH14081	XP_002627272.1	5 E-54	357/419
Eukaryota	Fungi	Pichia guilliermondii ATCC 6260	XP_001487655.1	8 E-54	350/419
Eukaryota	Fungi	Pichia guilliermondii ATCC 6260	EDK36934.2	2 E-53	350/419
Eukaryota	Fungi	Debaryomyces hansenii CBS767	XP_461841.1	3 E-53	355/419
Eukaryota	Fungi	Debaryomyces hansenii	CAG90302.2	1 E-52	355/419
Eukaryota	Fungi	Candida tropicalis MYA-3404	XP_002546475.1	5 E-42	354/419
Eukaryota	Fungi	Penicillium chrysogenum Wisconsin 54-1255	XP_002564221.1	6 E-34	341/419
Eukaryota	Fungi	Paracoccidioides brasiliensis Pb18;	EEH48808.1	5 E-33	347/419

Eukaryota	Fungi	Ajellomyces dermatitidis SLH14081	XP_002629066.1	1 E-32	347/419
Eukaryota	Fungi	Ajellomyces dermatitidis ER-3	EEQ85103.1	1 E-32	347/419
Eukaryota	Fungi	Ajellomyces capsulatus G186AR	EEH09155.1	2 E-31	347/419
Eukaryota	Fungi	Ajellomyces capsulatus NAm1	XP_001539789.1	3 E-31	347/419
Eukaryota	Fungi	Aspergillus flavus NRRL3357	XP_002381734.1	3 E-30	348/419
Eukaryota	Fungi	Aspergillus oryzae RIB40	XP_001825057.1	6 E-30	348/419
Eukaryota	Fungi	Nectria haematococca mpVI 77-13-4	EEU35610.1	2 E-29	342/419
Eukaryota	Fungi	Cryptococcus neoformans var. neoformans	XP_567535.1	2 E-28	348/419
Eukaryota	Fungi	Nectria haematococca mpVI 77-13-4	EEU43564.1	5 E-28	339/419
Eukaryota	Fungi	Aspergillus terreus NIH2624	XP_001218667.1	2 E-27	346/419
Eukaryota	Fungi	Aspergillus flavus NRRL3357	XP_002375427.1	3 E-27	337/419
Eukaryota	Fungi	Aspergillus niger CBS 513.88	XP_001393867.1	4 E-27	357/419
Eukaryota	Fungi	Penicillium chrysogenum Wisconsin 54-1255	XP_002561128.1	5 E-27	340/419
Eukaryota	Fungi	Gibberella zeae PH-1	XP_389385.1	6 E-27	340/419
Eukaryota	Fungi	Aspergillus oryzae RIB40	XP_001823160.1	6 E-27	341/419
Eukaryota	Fungi	Aspergillus oryzae RIB40	XP_001820577.1	9 E-27	342/419
Eukaryota	Fungi	Aspergillus flavus NRRL3357	XP_002373816.1	9 E-27	342/419
Eukaryota	Fungi	Laccaria bicolor S238N-H82	XP_001887593.1	1 E-26	355/419
Eukaryota	Fungi	Aspergillus niger CBS 513.88	XP_001397482.1	3 E-26	358/419
Eukaryota	Fungi	Yarrowia lipolytica CLIB122	XP_501302.1	4 E-26	346/419
Eukaryota	Fungi	Aspergillus niger CBS 513.88	XP_001397979.1	1 E-25	339/419
Eukaryota	Fungi	Gibberella zeae PH-1	XP_390828.1	2 E-25	364/419
Eukaryota	Fungi	Pichia stipitis CBS 6054	XP_001384289.2	2 E-25	350/419
Eukaryota	Fungi	Microsporum canis CBS 113480	EEQ30417.1	8 E-25	349/419
Eukaryota	Fungi	Aspergillus terreus NIH2624	XP_001217046.1	2 E-24	342/419
Eukaryota	Fungi	Nectria haematococca mpVI 77-13-4	EEU43362.1	2 E-24	349/419
Eukaryota	Fungi	Talaromyces stipitatus ATCC 10500	XP_002341678.1	5 E-24	338/419
Eukaryota	Fungi	Cryptococcus neoformans var. neoformans	XP_777930.1	6 E-24	358/419
Eukaryota	Fungi	Gibberella zeae PH-1	XP_389552.1	7 E-24	338/419
Eukaryota	Fungi	Verticillium albo-atrum VaMs.102	EEY17121.1	7 E-24	346/419
Eukaryota	Fungi	Neosartorya fischeri NRRL 181	XP_001259072.1	1 E-23	347/419
Eukaryota	Fungi	Aspergillus niger CBS 513.88	XP_001394030.1	1 E-23	357/419
Eukaryota	Fungi	Aspergillus fumigatus Af293	XP_754331.2	2 E-23	349/419
Eukaryota	Fungi	Aspergillus fumigatus Af293	XP_749847.1	3 E-23	360/419
Eukaryota	Fungi	Talaromyces stipitatus ATCC 10500	XP_002479904.1	5 E-23	341/419
Eukaryota	Fungi	Pyrenophora tritici-repentis Pt-1C-BFP	XP_001931289.1	5 E-23	390/419
Eukaryota	Fungi	Aspergillus oryzae RIB40	XP_001817712.1	1 E-22	340/419
Eukaryota	Fungi	Kluyveromyces lactis NRRL Y-1140	XP_451322.1	2 E-22	360/419
Eukaryota	Fungi	Candida dubliniensis CD36	XP_002418214.1	2 E-22	365/419
Eukaryota	Fungi	Candida albicans WO-1	EEQ45694.1	2 E-22	365/419
Eukaryota	Fungi	Nectria haematococca mpVI 77-13-4	EEU48223.1	2 E-22	344/419
Eukaryota	Fungi	Aspergillus terreus NIH2624	XP_001210389.1	2 E-22	362/419
Eukaryota	Fungi	Sclerotinia sclerotiorum 1980 UF-70	XP_001589189.1	2 E-22	337/419
Eukaryota	Fungi	Neurospora crassa OR74A	XP_956353.2	4 E-22	357/419
Eukaryota	Fungi	Aspergillus niger CBS 513.88	XP_001395054.1	6 E-22	356/419
Eukaryota	Fungi	Neosartorya fischeri NRRL 181	XP_001263175.1	7 E-22	349/419
Eukaryota	Fungi	Penicillium chrysogenum Wisconsin 54-1255	XP_002559067.1	7 E-22	338/419
Eukaryota	Fungi	Ajellomyces capsulatus G186AR	EEH07851.1	7 E-22	356/419
Eukaryota	Fungi	Penicillium chrysogenum Wisconsin 54-1255	XP_002559085.1	7 E-22	340/419
Eukaryota	Fungi	Coccidioides posadasii C735 delta	EER28670.1	8 E-22	365/419
Eukaryota	Fungi	Ajellomyces capsulatus H143	EER42807.1	8 E-22	340/419
Eukaryota	Fungi	Chaetomium globosum CBS 148.51	XP_001228061.1	1 E-21	355/419
Eukaryota	Fungi	Yarrowia lipolytica CLIB122	XP_503579.1	1 E-21	349/419
Eukaryota	Metazoa	Nematostella vectensis	XP_001641564.1	1 E-21	355/419
Eukaryota	Fungi	Candida albicans SC5314	XP_722626.1	2 E-21	365/419
Eukaryota	Metazoa	Ciona intestinalis	XP_002129951.1	2 E-21	360/419
Eukaryota	Fungi	Penicillium marneffei ATCC 18224	XP_002151297.1	2 E-21	340/419
Eukaryota	Fungi	Aspergillus flavus NRRL3357	XP_002381611.1	2 E-21	367/419

Eukaryota	Fungi	Aspergillus oryzae RIB40	XP_001825613.1	2 E-21	367/419
Eukaryota	Fungi	Sclerotinia sclerotiorum 1980 UF-70	XP_001592457.1	2 E-21	356/419
Eukaryota	Fungi	Nectria haematococca mpVI 77-13-4	EEU46261.1	5 E-21	360/419
Eukaryota	Fungi	Nectria haematococca mpVI 77-13-4	EEU34720.1	5 E-21	352/419
Eukaryota	Fungi	Microsporum canis CBS 113480	EEQ35853.1	6 E-21	344/419
Eukaryota	Fungi	Podospora anserina DSM 980	XP_001905598.1	8 E-21	351/419
Eukaryota	Fungi	Botryotinia fuckeliana B05.10	XP_001549407.1	9 E-21	356/419
Eukaryota	Fungi	Aspergillus nidulans FGSC A4	XP_681776.1	9 E-21	348/419
Eukaryota	Fungi	Saccharomyces cerevisiae EC1118	CAY82479.1	9 E-21	403/419
Eukaryota	Fungi	Saccharomyces cerevisiae RM11-1a	EDV12376.1	1 E-20	403/419
Eukaryota	Fungi	Aspergillus fumigatus Af293	XP_748643.1	1 E-20	347/419

AFUA_2G12720

Eukaryota	Fungi	Aspergillus fumigatus Af293	XP_755603.2	1 E-127	221/221

AFUA_2G13025

Eukaryota	Fungi	Aspergillus fumigatus Af293	XP_755634.1	1 E-167	282/282
Eukaryota	Fungi	Neosartorya fischeri NRRL 181	XP_001260761.1	1 E-126	273/282
Eukaryota	Fungi	Aspergillus clavatus NRRL 1	XP_001275577.1	1 E-82	265/282
Eukaryota	Fungi	Aspergillus niger CBS 513.88	XP_001401725.1	6 E-79	263/282
Eukaryota	Fungi	Aspergillus nidulans FGSC A4	XP_659280.1	4 E-77	260/282
Eukaryota	Fungi	Aspergillus flavus NRRL3357	XP_002376967.1	3 E-75	265/282
Eukaryota	Fungi	Aspergillus oryzae RIB40	XP_001821205.1	3 E-75	265/282
Eukaryota	Fungi	Aspergillus terreus NIH2624	XP_001214506.1	3 E-74	271/282
Eukaryota	Fungi	Penicillium chrysogenum Wisconsin 54-1255	XP_002559127.1	4 E-66	294/282
Eukaryota	Fungi	Talaromyces stipitatus ATCC 10500	XP_002483119.1	3 E-60	249/282
Eukaryota	Fungi	Penicillium marneffei ATCC 18224	XP_002150973.1	1 E-57	263/282
Eukaryota	Fungi	Aspergillus flavus NRRL3357	XP_002384331.1	4 E-57	287/282
Eukaryota	Fungi	Aspergillus clavatus NRRL 1	XP_001269041.1	6 E-51	277/282
Eukaryota	Fungi	Paracoccidioides brasiliensis Pb03;	EEH19091.1	3 E-48	253/282
Eukaryota	Fungi	Aspergillus terreus NIH2624	XP_001214992.1	1 E-44	246/282
Eukaryota	Fungi	Paracoccidioides brasiliensis Pb01;	EEH37902.1	1 E-41	237/282
Eukaryota	Fungi	Nectria haematococca mpVI 77-13-4	EEU34126.1	8 E-31	236/282
Eukaryota	Fungi	Gibberella zeae PH-1	XP_390568.1	1 E-30	234/282
Eukaryota	Fungi	Coccidioides posadasii C735 delta	EER23348.1	6 E-25	243/282
Eukaryota	Fungi	Coccidioides immitis RS;	XP_001248001.1	1 E-24	243/282
Eukaryota	Fungi	Botryotinia fuckeliana B05.10	XP_001552391.1	3 E-23	272/282
Eukaryota	Fungi	Magnaporthe grisea 70-15	XP_368291.2	4 E-23	281/282
Eukaryota	Fungi	Verticillium albo-atrum VaMs.102	EEY14535.1	5 E-23	254/282
Eukaryota	Fungi	Sclerotinia sclerotiorum 1980 UF-70	XP_001587425.1	2 E-20	272/282
Eukaryota	Fungi	Podospora anserina DSM 980	XP_001911795.1	1 E-19	245/282
Eukaryota	Fungi	Neurospora crassa	CAD11389.1	2 E-17	314/282
Eukaryota	Fungi	Neurospora crassa OR74A	XP_961251.2	2 E-17	314/282

AFUA_2G13060

Eukaryota	Fungi	Aspergillus fumigatus Af293	XP_755638.1	1 E-162	288/288
Eukaryota	Fungi	Neosartorya fischeri NRRL 181	XP_001260765.1	1 E-146	288/288
Eukaryota	Fungi	Aspergillus flavus NRRL3357	XP_002379610.1	1 E-104	281/288
Eukaryota	Fungi	Aspergillus oryzae RIB40	XP_001821801.1	1 E-104	281/288
Eukaryota	Fungi	Penicillium chrysogenum Wisconsin 54-1255	XP_002557571.1	1 E-101	276/288
Eukaryota	Fungi	Aspergillus clavatus NRRL 1	XP_001275581.1	2 E-99	270/288
Eukaryota	Fungi	Aspergillus terreus NIH2624	XP_001208640.1	8 E-99	260/288
Eukaryota	Fungi	Aspergillus niger CBS 513.88	XP_001399317.1	4 E-97	284/288
Eukaryota	Fungi	Aspergillus nidulans FGSC A4	tpeCBF69857.1	1 E-87	268/288
Eukaryota	Fungi	Aspergillus nidulans FGSC A4	XP_663853.1	1 E-87	268/288

Eukaryota	Fungi	Coccidioides posadasii C735 delta	EER29120.1	3 E-72	257/288
Eukaryota	Fungi	Coccidioides immitis RS;	XP_001245666.1	7 E-72	257/288
Eukaryota	Fungi	Ajellomyces dermatitidis SLH14081	XP_002624021.1	5 E-71	298/288
Eukaryota	Fungi	Uncinocarpus reesii 1704	XP_002583482.1	2 E-70	287/288
Eukaryota	Fungi	Ajellomyces capsulatus G186AR	EEH11495.1	3 E-66	311/288
Eukaryota	Fungi	Penicillium marneffei ATCC 18224	XP_002147106.1	8 E-66	271/288
Eukaryota	Fungi	Ajellomyces capsulatus NAm1	XP_001541658.1	2 E-65	304/288
Eukaryota	Fungi	Paracoccidioides brasiliensis Pb03;	EEH23064.1	3 E-65	300/288
Eukaryota	Fungi	Paracoccidioides brasiliensis Pb01;	EEH35213.1	1 E-64	299/288
Eukaryota	Fungi	Talaromyces stipitatus ATCC 10500	XP_002479374.1	1 E-63	283/288
Eukaryota	Fungi	Microsporum canis CBS 113480	EEQ31536.1	2 E-57	244/288
Eukaryota	Fungi	Botryotinia fuckeliana B05.10	XP_001554496.1	4 E-48	240/288
Eukaryota	Fungi	Magnaporthe grisea 70-15	XP_360675.1	9 E-46	233/288
Eukaryota	Fungi	Sclerotinia sclerotiorum 1980 UF-70	XP_001593917.1	9 E-44	238/288
Eukaryota	Fungi	Chaetomium globosum CBS 148.51	XP_001228347.1	4 E-43	242/288
Eukaryota	Fungi	Neurospora crassa OR74A	XP_956953.1	2 E-40	261/288
Eukaryota	Fungi	Gibberella zeae PH-1	XP_381626.1	2 E-38	231/288
Eukaryota	Fungi	Verticillium albo-atrum VaMs.102	EEY19312.1	8 E-38	251/288
Eukaryota	Fungi	Podospora anserina DSM 980	XP_001909744.1	1 E-36	257/288
Eukaryota	Fungi	Nectria haematococca mpVI 77-13-4	EEU44185.1	1 E-35	231/288
Eukaryota	Fungi	Phaeosphaeria nodorum SN15	XP_001797869.1	3 E-31	241/288

AFUA_2G13295

Eukaryota	Fungi	Aspergillus fumigatus Af293	XP_001481672.1	0.0	453/453
Eukaryota	Fungi	Neosartorya fischeri NRRL 181	XP_001260789.1	0.0	452/453
Eukaryota	Fungi	Aspergillus clavatus NRRL 1	XP_001275603.1	0.0	423/453
Eukaryota	Fungi	Aspergillus terreus NIH2624	XP_001215304.1	1 E-178	440/453
Eukaryota	Fungi	Neosartorya fischeri NRRL 181	XP_001258057.1	1 E-176	444/453
Eukaryota	Fungi	Aspergillus niger CBS 513.88	XP_001390787.1	1 E-175	439/453
Eukaryota	Fungi	Aspergillus oryzae RIB40	XP_001821768.1	1 E-174	448/453
Eukaryota	Fungi	Aspergillus fumigatus A1163	EDP49523.1	1 E-174	444/453
Eukaryota	Fungi	Aspergillus flavus NRRL3357	XP_002379647.1	1 E-172	457/453
Eukaryota	Fungi	Aspergillus flavus NRRL3357	XP_002380809.1	1 E-165	449/453
Eukaryota	Fungi	Penicillium chrysogenum Wisconsin 54-1255	XP_002557972.1	1 E-151	446/453
Eukaryota	Fungi	Talaromyces stipitatus ATCC 10500	XP_002481350.1	1 E-148	442/453
Eukaryota	Fungi	Penicillium marneffei ATCC 18224	XP_002147219.1	1 E-148	442/453
Eukaryota	Fungi	Paracoccidioides brasiliensis Pb01;	EEH36303.1	1 E-147	447/453
Eukaryota	Fungi	Paracoccidioides brasiliensis Pb03;	EEH23098.1	1 E-146	447/453
Eukaryota	Fungi	Paracoccidioides brasiliensis Pb18;	EEH42124.1	1 E-146	447/453
Eukaryota	Fungi	Ajellomyces dermatitidis SLH14081	XP_002626123.1	1 E-141	421/453
Eukaryota	Fungi	Coccidioides posadasii C735 delta	EER29148.1	1 E-140	452/453
Eukaryota	Fungi	Ajellomyces capsulatus H143	EER41771.1	1 E-133	425/453
Eukaryota	Fungi	Aspergillus nidulans FGSC A4	XP_663831.1	1 E-131	449/453
Eukaryota	Fungi	Ajellomyces capsulatus G186AR	EEH05868.1	1 E-131	407/453
Eukaryota	Fungi	Ajellomyces capsulatus NAm1	XP_001538357.1	1 E-131	407/453
Eukaryota	Fungi	Aspergillus niger CBS 513.88	XP_001399344.1	1 E-127	454/453
Eukaryota	Fungi	Uncinocarpus reesii 1704	XP_002583522.1	1 E-123	417/453
Eukaryota	Fungi	Microsporum canis CBS 113480	EEQ31467.1	1 E-123	465/453
Eukaryota	Fungi	Sclerotinia sclerotiorum 1980 UF-70	XP_001595595.1	1 E-113	427/453
Eukaryota	Fungi	Phaeosphaeria nodorum SN15	XP_001796379.1	1 E-112	442/453
Eukaryota	Fungi	Pyrenophora tritici-repentis Pt-1C-BFP	XP_001935429.1	1 E-108	455/453
Eukaryota	Fungi	Neotyphodium uncinatum	AAV68709.1	1 E-108	446/453
Eukaryota	Fungi	Neurospora crassa OR74A	XP_001728131.1	1 E-107	447/453
Eukaryota	Fungi	Gibberella zeae PH-1	XP_382643.1	1 E-106	428/453
Eukaryota	Fungi	Podospora anserina DSM 980	XP_001903278.1	1 E-106	432/453
Eukaryota	Fungi	Neotyphodium coenophialum	ABQ57514.1	1 E-106	446/453
Eukaryota	Fungi	Neotyphodium uncinatum	AAV68700.1	1 E-105	446/453

Eukaryota	Fungi	Penicillium marneffei ATCC 18224	XP_002148421.1	1 E-104	419/453
Eukaryota	Fungi	Talaromyces stipitatus ATCC 10500	XP_002482701.1	1 E-101	426/453
Eukaryota	Fungi	Botryotinia fuckeliana B05.10	XP_001560486.1	3 E-99	432/453
Eukaryota	Fungi	Nectria haematococca mpVI 77-13-4	EEU39402.1	7 E-99	420/453
Eukaryota	Fungi	Botryotinia fuckeliana B05.10	XP_001557939.1	2 E-98	405/453
Eukaryota	Fungi	Sclerotinia sclerotiorum 1980 UF-70	XP_001589723.1	5 E-97	432/453
Eukaryota	Fungi	Magnaporthe grisea 70-15	XP_364581.1	5 E-94	430/453
Eukaryota	Fungi	Nectria haematococca mpVI 77-13-4	EEU48236.1	5 E-92	400/453
Eukaryota	Fungi	Microsporum canis CBS 113480	EEQ35769.1	1 E-90	437/453
Eukaryota	Fungi	Chaetomium globosum CBS 148.51	XP_001224417.1	2 E-88	448/453
Eukaryota	Fungi	Verticillium albo-atrum VaMs.102	EEY23451.1	2 E-78	402/453
Eukaryota	Fungi	Ustilago maydis 521	XP_760275.1	4 E-78	435/453
Eukaryota	Fungi	Coprinopsis cinerea okayama7#130	XP_001834727.1	2 E-69	412/453
Eukaryota	Fungi	Laccaria bicolor S238N-H82	XP_001881883.1	2 E-68	405/453
Eukaryota	Fungi	Cryptococcus neoformans var. neoformans	XP_772243.1	3 E-67	435/453
Eukaryota	Fungi	Cryptococcus neoformans var. neoformans	XP_772244.1	4 E-67	435/453
Eukaryota	Fungi	Coprinopsis cinerea okayama7#130	XP_001840655.1	5 E-67	415/453
Eukaryota	Fungi	Cryptococcus neoformans var. neoformans	XP_568355.1	6 E-67	435/453
Eukaryota	Fungi	Cryptococcus neoformans var. neoformans	XP_568356.1	7 E-67	435/453
Eukaryota	Fungi	Pichia pastoris GS115	XP_002493550.1	1 E-66	405/453
Eukaryota	Fungi	Candida dubliniensis CD36	XP_002416949.1	9 E-63	419/453
Eukaryota	Fungi	Clavispora lusitaniae ATCC 42720	XP_002615578.1	1 E-62	416/453
Eukaryota	Fungi	Yarrowia lipolytica CLIB122	XP_503727.1	4 E-62	398/453
Eukaryota	Fungi	Lodderomyces elongisporus NRRL YB-4239	XP_001526674.1	2 E-61	417/453
Eukaryota	Fungi	Candida albicans WO-1	EEQ42864.1	2 E-61	406/453
Eukaryota	Fungi	Pichia stipitis CBS 6054	XP_001385424.2	2 E-61	415/453
Eukaryota	Fungi	Pichia stipitis CBS 6054	XP_001386538.2	1 E-60	415/453
Eukaryota	Fungi	Yarrowia lipolytica CLIB122	XP_501088.1	2 E-60	401/453
Eukaryota	Fungi	Candida albicans SC5314	XP_721486.1	5 E-60	406/453
Eukaryota	Fungi	Candida albicans	CAA21971.1	8 E-60	406/453
Eukaryota	Fungi	Candida tropicalis MYA-3404	XP_002550032.1	2 E-59	395/453
Eukaryota	Fungi	Debaryomyces hansenii	CAG86972.2	4 E-57	422/453
Eukaryota	Fungi	Debaryomyces hansenii CBS767	XP_458826.1	4 E-56	422/453
Eukaryota	Fungi	Pichia guilliermondii ATCC 6260	XP_001487138.1	5 E-54	395/453
Eukaryota	Fungi	Pichia guilliermondii ATCC 6260	EDK36417.2	7 E-54	395/453
Eukaryota	Fungi	Lachancea thermotolerans CBS 6340	XP_002552787.1	2 E-53	411/453
Eukaryota	Fungi	Candida tropicalis MYA-3404	XP_002550030.1	2 E-50	385/453
Eukaryota	Fungi	Kluyveromyces lactis NRRL Y-1140	XP_456124.1	2 E-50	418/453
Eukaryota	Fungi	Pichia guilliermondii ATCC 6260	EDK36577.2	1 E-49	404/453
Eukaryota	Fungi	Debaryomyces hansenii	CAG87243.2	3 E-49	420/453
Eukaryota	Fungi	Pichia guilliermondii ATCC 6260	XP_001487298.1	7 E-49	404/453
Eukaryota	Fungi	Schizosaccharomyces pombe	NP_595091.1	1 E-48	390/453
Eukaryota	Fungi	Debaryomyces hansenii CBS767	XP_459075.1	1 E-48	413/453
Eukaryota	Fungi	Zygosaccharomyces rouxii CBS 732	XP_002496453.1	4 E-48	409/453
Eukaryota	Fungi	Clavispora lusitaniae ATCC 42720	XP_002616148.1	2 E-47	406/453
Bacteria	Proteobacteria	alpha proteobacterium BAL199	ZP_02187140.1	1 E-44	389/453
Eukaryota	Metazoa	Branchiostoma floridae	XP_002604134.1	7 E-43	387/453
Bacteria	Cyanobacteria	Lyngbya sp. PCC 8106	ZP_01623525.1	1 E-42	390/453
Bacteria	Cyanobacteria	Synechococcus sp. JA-3-3Ab	YP_474286.1	5 E-41	374/453
Bacteria	Verrucomicrobia	Chthoniobacter flavus Ellin428	ZP_03132331.1	5 E-41	389/453
Bacteria	Cyanobacteria	Microcystis aeruginosa PCC 7806	CAO90795.1	8 E-40	381/453
Bacteria	Proteobacteria	Sorangium cellulosum 'So ce	YP_001618780.1	3 E-39	378/453
Bacteria	Cyanobacteria	Cyanothece sp. PCC 7425	YP_002481469.1	3 E-39	381/453
Bacteria	Cyanobacteria	Microcystis aeruginosa NIES-843	YP_001660305.1	5 E-39	381/453
Eukaryota	Alveolata	Paramecium tetraurelia strain d4-2	XP_001460222.1	3 E-38	371/453
Eukaryota	Alveolata	Paramecium tetraurelia strain d4-2	XP_001454622.1	7 E-38	371/453
Bacteria	Thermus	Meiothermus ruber DSM 1279	ZP_04039199.1	8 E-38	384/453
Bacteria	Proteobacteria	Myxococcus xanthus DK 1622	YP_633895.1	1 E-37	387/453

Eukaryota	Fungi	Schizosaccharomyces japonicus yFS275	XP_002174984.1	3 E-37	382/453
Bacteria	Chloroflexi	Chloroflexus aurantiacus J-10-fl	YP_001635266.1	6 E-37	373/453
Eukaryota	Metazoa	Nematostella vectensis	XP_001633118.1	8 E-37	392/453
Eukaryota	Viridiplantae	Populus trichocarpa	XP_002329994.1	2 E-36	398/453
Eukaryota	Metazoa	Nematostella vectensis	XP_001635489.1	2 E-36	394/453
Bacteria	Cyanobacteria	Cyanothece sp. PCC 7822	ZP_03155664.1	3 E-36	382/453
Eukaryota	Viridiplantae	Arabidopsis thaliana	NP_850886.1	1 E-35	399/453
Eukaryota	Metazoa	Trichoplax adhaerens	XP_002111932.1	3 E-35	378/453
Bacteria	Cyanobacteria	Synechococcus sp. JA-2-3B'a(2-13)	YP_478092.1	7 E-35	374/453
Eukaryota	Viridiplantae	Zea mays;	NP_001151142.1	3 E-34	406/453
Eukaryota	Viridiplantae	Populus trichocarpa	XP_002318924.1	4 E-34	398/453
Eukaryota	Viridiplantae	Vitis vinifera	XP_002279795.1	8 E-34	395/453
Eukaryota	Viridiplantae	Zea mays;	NP_001152215.1	2 E-33	403/453
Eukaryota	Alveolata	Tetrahymena thermophila	XP_001030031.1	3 E-33	382/453
Eukaryota	Viridiplantae	Vitis vinifera	CAO40231.1	6 E-33	391/453
Eukaryota	Viridiplantae	Oryza sativa Japonica Group	NP_001042786.1	8 E-33	397/453
Eukaryota	Viridiplantae	Ricinus communis	XP_002515182.1	9 E-33	399/453
Eukaryota	Viridiplantae	Oryza sativa Indica Group	EEC70448.1	1 E-32	397/453
Eukaryota	Viridiplantae	Vitis vinifera	XP_002281415.1	2 E-32	397/453
Eukaryota	Viridiplantae	Populus trichocarpa	XP_002302806.1	4 E-32	414/453
Eukaryota	Viridiplantae	Picea sitchensis	ABR18137.1	9 E-32	395/453
Bacteria	Planctomycetes	Rhodopirellula baltica SH 1	NP_867887.1	9 E-32	386/453
Eukaryota	Viridiplantae	Arabidopsis thaliana	NP_191772.1	3 E-31	395/453
Bacteria	Actinobacteria	Stackebrandtia nassauensis DSM 44728	ZP_04485177.1	5 E-31	382/453
Eukaryota	Metazoa	Ciona intestinalis	XP_002122357.1	7 E-31	380/453
Eukaryota	Viridiplantae	Physcomitrella patens subsp. patens	XP_001763635.1	5 E-30	410/453
Eukaryota	Viridiplantae	Physcomitrella patens subsp. patens	XP_001754646.1	6 E-30	404/453
Eukaryota	Viridiplantae	Sorghum bicolor;	XP_002455490.1	1 E-29	414/453
Eukaryota	Viridiplantae	Populus trichocarpa	XP_002320258.1	1 E-29	407/453
Bacteria	Actinobacteria	Micromonospora sp. ATCC 39149	ZP_04608025.1	1 E-27	368/453
Eukaryota	Viridiplantae	Oryza sativa Japonica Group	NP_001042788.1	1 E-27	411/453
Bacteria	Actinobacteria	Streptomyces hygroscopicus ATCC 53653	ZP_05512777.1	1 E-27	398/453
Eukaryota	Viridiplantae	Oryza sativa Japonica Group	BAD86870.1	2 E-27	411/453
Bacteria	Actinobacteria	Streptomyces sp. SPB78	ZP_05485253.1	2 E-27	382/453
Bacteria	Planctomycetes	Gemmata obscuriglobus UQM 2246	ZP_02732865.1	2 E-26	392/453
Eukaryota	Viridiplantae	Zea mays;	NP_001131731.1	6 E-26	411/453
Eukaryota	Viridiplantae	Solanum demissum	AAT39966.1	6 E-26	385/453
Bacteria	Actinobacteria	Kribbella flavida DSM 17836	ZP_03862630.1	9 E-25	367/453
Eukaryota	Viridiplantae	Oryza sativa Japonica Group	EAZ11522.1	2 E-24	412/453
Bacteria	Actinobacteria	Mobiluncus curtisii ATCC 43063	ZP_03924407.1	4 E-24	380/453
Bacteria	Actinobacteria	Catenulispora acidiphila DSM 44928	YP_003116259.1	7 E-24	380/453
Eukaryota	Euglenozoa	Trypanosoma brucei gambiense DAL972	CBH17719.1	3 E-23	395/453
Eukaryota	Euglenozoa	Trypanosoma cruzi strain CL	XP_815545.1	9 E-23	384/453
Eukaryota	Euglenozoa	Trypanosoma brucei TREU927	XP_828812.1	9 E-23	395/453
Bacteria	Proteobacteria	Lysobacter lactamgenus	CAA39986.1	2 E-22	394/453
Eukaryota	Euglenozoa	Trypanosoma cruzi strain CL	XP_813763.1	7 E-22	384/453
Eukaryota	Choanoflagellida	Monosiga brevicollis MX1	XP_001743892.1	1 E-21	390/453
Bacteria	Actinobacteria	Amycolatopsis lactamdurans	Q03046.1	6 E-21	388/453
Bacteria	Actinobacteria	Streptomyces clavuligerus ATCC 27064	ZP_05002808.1	1 E-20	390/453
Bacteria	Actinobacteria	Streptosporangium roseum DSM 43021	ZP_04476107.1	5 E-20	369/453
Bacteria	Actinobacteria	Streptomyces sp. SPB74	ZP_04992197.1	8 E-18	377/453
Bacteria	Actinobacteria	marine actinobacterium PHSC20C1	ZP_01129534.1	5 E-17	383/453
Bacteria	Proteobacteria	Acidovorax citrulli AAC00-1	YP_969493.1	2 E-14	407/453

AFUA_2G13300

Eukaryota	Fungi	Aspergillus fumigatus Af293	XP_755663.1	0.0	547/547
Eukaryota	Fungi	Neosartorya fischeri NRRL 181	XP_001260790.1	0.0	545/547

Eukaryota	Fungi	Aspergillus clavatus NRRL 1	XP_001275604.1	0.0	567/547
Eukaryota	Fungi	Aspergillus terreus NIH2624	XP_001208672.1	0.0	524/547
Eukaryota	Fungi	Aspergillus oryzae RIB40	XP_001821769.1	0.0	528/547
Eukaryota	Fungi	Aspergillus flavus NRRL3357	XP_002379646.1	0.0	528/547
Eukaryota	Fungi	Aspergillus nidulans FGSC A4	tpeCBF69908.1	0.0	547/547
Eukaryota	Fungi	Aspergillus nidulans FGSC A4	XP_663830.1	0.0	529/547
Eukaryota	Fungi	Penicillium marneffei ATCC 18224	XP_002145540.1	0.0	540/547
Eukaryota	Fungi	Talaromyces stipitatus ATCC 10500	XP_002486992.1	0.0	539/547
Eukaryota	Fungi	Neosartorya fischeri NRRL 181	XP_001266384.1	1 E-178	531/547
Eukaryota	Fungi	Aspergillus fumigatus A1163	EDP51000.1	1 E-175	530/547
Eukaryota	Fungi	Aspergillus fumigatus Af293	XP_748194.1	1 E-175	530/547
Eukaryota	Fungi	Aspergillus clavatus NRRL 1	XP_001276390.1	1 E-175	531/547
Eukaryota	Fungi	Pyrenophora tritici-repentis Pt-1C-BFP	XP_001939833.1	1 E-174	535/547
Eukaryota	Fungi	Phaeosphaeria nodorum SN15	XP_001805595.1	1 E-168	535/547
Eukaryota	Fungi	Podospora anserina DSM 980	XP_001912749.1	1 E-164	544/547
Eukaryota	Fungi	Nectria haematococca mpVI 77-13-4	EEU38176.1	1 E-164	530/547
Eukaryota	Fungi	Gibberella zeae PH-1	XP_388184.1	1 E-161	525/547
Eukaryota	Fungi	Nectria haematococca mpVI 77-13-4	EEU43836.1	1 E-160	535/547
Eukaryota	Fungi	Neurospora crassa OR74A	XP_964556.1	1 E-160	563/547
Eukaryota	Fungi	Penicillium chrysogenum Wisconsin 54-1255	XP_002564206.1	1 E-160	532/547
Eukaryota	Fungi	Penicillium chrysogenum Wisconsin 54-1255	XP_002557540.1	1 E-159	528/547
Eukaryota	Fungi	Aspergillus oryzae RIB40	XP_001825761.1	1 E-158	536/547
Eukaryota	Fungi	Aspergillus flavus NRRL3357	XP_002377350.1	1 E-158	536/547
Eukaryota	Fungi	Aspergillus terreus NIH2624	XP_001217039.1	1 E-155	532/547
Eukaryota	Fungi	Gibberella zeae PH-1	XP_381889.1	1 E-152	534/547
Eukaryota	Fungi	Aspergillus nidulans FGSC A4	XP_664325.1	1 E-152	537/547
Eukaryota	Fungi	Penicillium chrysogenum Wisconsin 54-1255	XP_002568439.1	1 E-151	532/547
Eukaryota	Fungi	Magnaporthe grisea 70-15	XP_362883.1	1 E-150	543/547
Eukaryota	Fungi	Nectria haematococca mpVI 77-13-4	EEU42621.1	1 E-142	533/547
Eukaryota	Fungi	Gibberella zeae PH-1	XP_385974.1	1 E-138	527/547
Eukaryota	Fungi	Ustilago maydis 521	XP_759158.1	1 E-122	533/547
Bacteria	Actinobacteria	Rhodococcus erythropolis SK121	ZP_04382279.1	1 E-103	508/547
Bacteria	Actinobacteria	Rhodococcus erythropolis PR4	YP_002768176.1	1 E-103	508/547
Bacteria	Actinobacteria	Rhodococcus jostii RHA1	YP_705541.1	2 E-97	511/547
Bacteria	Actinobacteria	Rhodococcus opacus B4	YP_002782860.1	2 E-95	511/547
Bacteria	Actinobacteria	Streptomyces sp. AA4	ZP_05479238.1	9 E-93	525/547
Bacteria	Actinobacteria	Renibacterium salmoninarum ATCC 33209	YP_001624021.1	4 E-92	539/547
Bacteria	Actinobacteria	Arthrobacter aurescens TC1	YP_949628.1	8 E-90	536/547
Bacteria	Actinobacteria	Tsukamurella paurometabola DSM 20162	ZP_04026681.1	2 E-84	542/547
Bacteria	Cyanobacteria	Gloeobacter violaceus PCC 7421	NP_923321.1	6 E-55	531/547
Bacteria	Firmicutes	Bacillus sp. SG-1	ZP_01859717.1	8 E-55	530/547
Bacteria	Chloroflexi	Chloroflexus aurantiacus J-10-fl	YP_001635359.1	2 E-54	531/547
Bacteria	Actinobacteria	Streptomyces scabiei 87.22	CBG67920.1	6 E-54	499/547
Bacteria	Proteobacteria	Burkholderia cenocepacia J2315	YP_002234543.1	3 E-52	530/547
Bacteria	Cyanobacteria	Cyanothece sp. PCC 7425	YP_002482097.1	7 E-51	536/547
Bacteria	Firmicutes	Carboxydibrachium pacificum DSM 12653	ZP_05091422.1	5 E-50	529/547
Bacteria	Actinobacteria	Streptomyces sviceus ATCC 29083	ZP_05020273.1	5 E-50	521/547
Bacteria	Proteobacteria	Burkholderia sp. 383	YP_371776.1	1 E-49	530/547
Bacteria	Firmicutes	Brevibacillus brevis NBRC 100599	YP_002770253.1	2 E-49	539/547
Bacteria	Actinobacteria	Streptomyces hygroscopicus ATCC 53653	ZP_05521088.1	4 E-48	500/547
Bacteria	Firmicutes	Thermoanaerobacter tengcongensis MB4	NP_622711.1	6 E-48	529/547
Bacteria	Actinobacteria	Rhodococcus jostii RHA1	YP_700152.1	1 E-47	497/547
Bacteria	Chloroflexi	Chloroflexus aggregans DSM 9485	YP_002462664.1	2 E-47	498/547
Bacteria	Proteobacteria	Ruegeria pomeroyi DSS-3	YP_167744.1	2 E-47	484/547
Bacteria	Firmicutes	Bacillus thuringiensis serovar pondicheriensis	ZP_04091143.1	4 E-47	509/547
Bacteria	Chloroflexi	Chloroflexus aggregans DSM 9485	YP_002464069.1	6 E-47	515/547
Bacteria	Firmicutes	Bacillus cereus R309803;	ZP_04289907.1	6 E-47	539/547
Bacteria	Proteobacteria	Sagittula stellata E-37	ZP_01747278.1	6 E-47	527/547

Bacteria	Firmicutes	Bacillus thuringiensis serovar pakistani	ZP_04123444.1	6 E-47	539/547
Bacteria	Proteobacteria	Erythrobacter litoralis HTCC2594	YP_457213.1	6 E-47	523/547
Bacteria	Thermobaculum	Thermobaculum terrenum ATCC BAA-798	ZP_03857601.1	8 E-47	509/547
Bacteria	Firmicutes	Bacillus cereus 95/8201;	ZP_04251804.1	8 E-47	509/547
Bacteria	Firmicutes	Bacillus cereus W;	ZP_03099887.1	9 E-47	509/547
Bacteria	Firmicutes	Bacillus cereus AH820;	YP_002452003.1	1 E-46	509/547
Bacteria	Planctomycetes	Gemmata obscuriglobus UQM 2246	ZP_02735100.1	1 E-46	508/547
Bacteria	Actinobacteria	Rhodococcus jostii RHA1	YP_703816.1	1 E-46	518/547
Bacteria	Proteobacteria	Kangiella koreensis DSM 16069	YP_003147050.1	2 E-46	518/547
Bacteria	Proteobacteria	Congregibacter litoralis KT71	ZP_01102402.1	5 E-46	495/547
Bacteria	Actinobacteria	Cryptobacterium curtum DSM 15641	YP_003151312.1	6 E-46	518/547
Bacteria	Firmicutes	Bacillus thuringiensis serovar monterrey	ZP_04108964.1	1 E-45	509/547
Bacteria	Actinobacteria	Streptosporangium roseum DSM 43021	ZP_04474520.1	1 E-45	487/547
Bacteria	Actinobacteria	Streptomyces sp. C	ZP_05510321.1	2 E-45	521/547
Bacteria	Firmicutes	Thermosinus carboxydivorans Nor1	ZP_01665166.1	3 E-45	504/547
Bacteria	Proteobacteria	Plesiocystis pacifica SIR-1	ZP_01906096.1	6 E-45	454/547
Bacteria	Proteobacteria	Shewanella amazonensis SB2B	YP_929004.1	1 E-44	509/547
Bacteria	Chloroflexi	Chloroflexus aurantiacus J-10-fl	YP_001636197.1	2 E-44	532/547
Bacteria	Bacteroidetes	Salinibacter ruber DSM 13855	YP_445294.1	2 E-44	522/547
Bacteria	Actinobacteria	Arthrobacter aurescens TC1	YP_946610.1	8 E-44	548/547
Bacteria	Proteobacteria	Rhodobacter sp. SW2	ZP_05842014.1	8 E-44	543/547
Bacteria	Firmicutes	Brevibacillus brevis NBRC 100599	YP_002772467.1	9 E-44	512/547
Eukaryota	Viridiplantae	Ricinus communis	XP_002513056.1	1 E-43	531/547
Bacteria	Chloroflexi	Roseiflexus sp. RS-1	YP_001276288.1	1 E-43	523/547
Bacteria	Chloroflexi	Sphaerobacter thermophilus DSM 20745	ZP_04494924.1	2 E-43	526/547
Bacteria	Actinobacteria	Saccharopolyspora erythraea NRRL 2338	YP_001106119.1	2 E-43	483/547
Bacteria	Proteobacteria	Sorangium cellulosum 'So ce	YP_001613707.1	4 E-43	502/547
Bacteria	Firmicutes	Clostridium sp. L2-50	ZP_02075458.1	6 E-43	493/547
Archaea	Euryarchaeota	Thermococcus sibiricus MM 739	YP_002993823.1	7 E-43	502/547
Bacteria	Proteobacteria	Erythrobacter sp. SD-21	ZP_01864550.1	8 E-43	504/547
Bacteria	Proteobacteria	gamma proteobacterium NOR5-3	ZP_05129313.1	1 E-42	525/547
Eukaryota	Viridiplantae	Sorghum bicolor;	XP_002456945.1	1 E-42	519/547
Bacteria	Actinobacteria	Eggerthella lenta DSM 2243	YP_003181648.1	2 E-42	523/547
Bacteria	Firmicutes	Clostridium carboxidivorans P7	ZP_05394604.1	2 E-42	532/547
Bacteria	Gemmatimonadet	Gemmatimonas aurantiaca T-27	YP_002762316.1	2 E-42	521/547
Bacteria	Proteobacteria	Phenylobacterium zucineum HLK1	YP_002131627.1	3 E-42	519/547
Bacteria	Acidobacteria	Acidobacterium capsulatum ATCC 51196	YP_002756515.1	3 E-42	522/547
Bacteria	Actinobacteria	marine actinobacterium PHSC20C1	ZP_01129268.1	4 E-42	544/547
Eukaryota	Viridiplantae	Arabidopsis thaliana	NP_974445.2	7 E-42	529/547
Eukaryota	Viridiplantae	Arabidopsis thaliana	AAP55750.1	8 E-42	529/547
Eukaryota	Viridiplantae	Arabidopsis thaliana	AAP55749.1	8 E-42	529/547
Eukaryota	Viridiplantae	Arabidopsis thaliana	NP_567027.2	9 E-42	529/547
Bacteria	Proteobacteria	Erythrobacter sp. NAP1	ZP_01038788.1	1 E-41	506/547
Bacteria	Actinobacteria	Saccharopolyspora erythraea NRRL 2338	YP_001106071.1	1 E-41	494/547
Bacteria	Proteobacteria	Shewanella loihica PV-4	YP_001095149.1	1 E-41	485/547
Bacteria	Proteobacteria	Hirschia baltica ATCC 49814	YP_003060939.1	2 E-41	536/547
Eukaryota	Viridiplantae	Populus trichocarpa	XP_002304801.1	2 E-41	484/547
Bacteria	Acidobacteria	Candidatus Koribacter versatilis Ellin345	YP_592518.1	2 E-41	500/547
Bacteria	Chloroflexi	Herpetosiphon aurantiacus ATCC 23779	YP_001545195.1	2 E-41	526/547
Bacteria	Proteobacteria	Shewanella loihica PV-4	YP_001093057.1	4 E-41	539/547
Bacteria	Chloroflexi	Roseiflexus castenholzii DSM 13941	YP_001433888.1	6 E-41	503/547
Bacteria	Proteobacteria	Glaciecola sp. HTCC2999	ZP_03559757.1	6 E-41	504/547
Eukaryota	Viridiplantae	Vitis vinifera	CAO42828.1	7 E-41	531/547
Eukaryota	Viridiplantae	Vitis vinifera	XP_002265500.1	1 E-40	531/547
Bacteria	Proteobacteria	Myxococcus xanthus DK 1622	YP_628927.1	1 E-40	486/547
Bacteria	Proteobacteria	Idiomarina loihiensis L2TR	YP_155596.1	2 E-40	507/547
Eukaryota	Viridiplantae	Oryza sativa Japonica Group	NP_001045422.1	2 E-40	517/547
Bacteria	Proteobacteria	Idiomarina baltica OS145	ZP_01042109.1	4 E-40	532/547

Bacteria	Proteobacteria	Pectobacterium carotovorum subsp. carotovorur	YP_003019065.1	5 E-40	549/547
Bacteria	Bacteroidetes	Algoriphagus sp. PR1	ZP_01720735.1	5 E-40	502/547
Bacteria	Bacteroidetes	Microscilla marina ATCC 23134	ZP_01689700.1	7 E-40	528/547
Bacteria	Firmicutes	Bacillus cereus Rock3-44;	ZP_04217159.1	8 E-40	528/547
Bacteria	Actinobacteria	Streptosporangium roseum DSM 43021	ZP_04472152.1	1 E-39	490/547
Bacteria	Bacteroidetes	Microscilla marina ATCC 23134	ZP_01687397.1	1 E-39	501/547
Bacteria	Proteobacteria	Novosphingobium aromaticivorans DSM 12444	YP_496376.1	1 E-39	478/547
Bacteria	Proteobacteria	Pectobacterium atrosepticum SCRI1043	YP_051779.1	2 E-39	541/547
Bacteria	Acidobacteria	Candidatus Koribacter versatilis Ellin345	YP_590517.1	2 E-39	511/547
Bacteria	Proteobacteria	Stigmatella aurantiaca DW4/3-1	ZP_01459367.1	3 E-39	486/547
Bacteria	Actinobacteria	Eggerthella lenta DSM 2243	YP_003181331.1	3 E-39	546/547
Bacteria	Firmicutes	Clostridium bolteae ATCC BAA-613	ZP_02087145.1	4 E-39	537/547
Bacteria	Firmicutes	Bacillus mycoides DSM 2048	ZP_04170976.1	4 E-39	475/547
Bacteria	Proteobacteria	Novosphingobium aromaticivorans DSM 12444	YP_496333.1	6 E-39	536/547
Bacteria	Firmicutes	Alkaliphilus metalliredigens QYMF	YP_001320870.1	6 E-39	539/547
Bacteria	Actinobacteria	Mycobacterium gilvum PYR-GCK	YP_001134195.1	7 E-39	537/547
Bacteria	Actinobacteria	marine actinobacterium PHSC20C1	ZP_01129586.1	9 E-39	545/547
Bacteria	Acidobacteria	Candidatus Solibacter usitatus Ellin6076	YP_828563.1	1 E-38	537/547
Bacteria	Firmicutes	Clostridium sp. M62/1	ZP_03732546.1	1 E-38	537/547
Bacteria	Actinobacteria	Arthrobacter aurescens TC1	YP_947787.1	1 E-38	496/547
Bacteria	Proteobacteria	Shewanella woodyi ATCC 51908	YP_001761769.1	2 E-38	524/547
Bacteria	Actinobacteria	Mycobacterium sp. KMS	YP_939364.1	2 E-38	538/547
Bacteria	Actinobacteria	Rhodococcus jostii RHA1	YP_705538.1	2 E-38	505/547
Bacteria	Firmicutes	Bacillus cereus AH621;	ZP_04297080.1	2 E-38	498/547
Bacteria	Bacteroidetes	Algoriphagus sp. PR1	ZP_01721121.1	3 E-38	539/547
Bacteria	Proteobacteria	Shewanella sediminis HAW-EB3	YP_001474990.1	3 E-38	504/547
Bacteria	Actinobacteria	Eggerthella lenta DSM 2243	YP_003182412.1	4 E-38	523/547
Bacteria	Firmicutes	Bacillus cereus BDRD-ST196;	ZP_04264267.1	4 E-38	498/547
Bacteria	Actinobacteria	Mycobacterium sp. MCS	YP_640481.1	5 E-38	538/547
Bacteria	Firmicutes	Bacillus cereus AH603;	ZP_04199648.1	5 E-38	498/547
Bacteria	Proteobacteria	Pectobacterium wasabiae WPP163	YP_003261005.1	6 E-38	541/547
Bacteria	Actinobacteria	Collinsella intestinalis DSM 13280	ZP_04447014.1	9 E-38	494/547
Bacteria	Actinobacteria	Brevibacterium linens BL2	ZP_05912243.1	9 E-38	491/547
Bacteria	Incertae Sedis	Symbiobacterium thermophilum IAM 14863	YP_075066.1	1 E-37	506/547
Bacteria	Synergistetes	Anaerobaculum hydrogeniformans ATCC BAA	ZP_05799804.1	1 E-37	495/547
Bacteria	Firmicutes	Bacillus cereus AH1134;	ZP_03230639.1	2 E-37	509/547
Bacteria	Proteobacteria	Cupriavidus taiwanensis	YP_002008822.1	2 E-37	525/547
Bacteria	Actinobacteria	Streptomyces sp. AA4	ZP_05479960.1	2 E-37	513/547
Bacteria	Actinobacteria	Nocardioides sp. JS614	YP_924412.1	2 E-37	482/547
Bacteria	Firmicutes	Bacillus cereus ATCC 10876	ZP_04319805.1	2 E-37	508/547
Bacteria	Proteobacteria	gamma proteobacterium HTCC2207	ZP_01224504.1	3 E-37	506/547
Bacteria	Proteobacteria	Methylobacterium radiotolerans JCM 2831	YP_001756706.1	4 E-37	538/547
Bacteria	Actinobacteria	Mycobacterium smegmatis str. MC2	YP_884858.1	4 E-37	531/547
Bacteria	Firmicutes	Bacillus weihenstephanensis KBAB4;	YP_001647260.1	4 E-37	498/547
Bacteria	Proteobacteria	Ralstonia metallidurans CH34	YP_586784.1	5 E-37	525/547
Bacteria	Firmicutes	Bacillus cereus Rock4-2;	ZP_04214388.1	6 E-37	508/547
Bacteria	Proteobacteria	Ralstonia eutropha H16	YP_841985.1	6 E-37	525/547
Bacteria	Proteobacteria	Shewanella piezotolerans WP3	YP_002313288.1	7 E-37	524/547
Bacteria	Bacteroidetes	Pedobacter heparinus DSM 2366	YP_003090766.1	7 E-37	519/547
Bacteria	Firmicutes	Granulicatella elegans ATCC 700633	ZP_05852815.1	7 E-37	513/547
Bacteria	Proteobacteria	Alteromonas macleodii 'Deep ecotype'	YP_002125086.1	7 E-37	503/547
Bacteria	Firmicutes	Bacillus cereus 172560W;	ZP_04308297.1	7 E-37	508/547
Bacteria	Proteobacteria	Stigmatella aurantiaca DW4/3-1	ZP_01463971.1	8 E-37	504/547
Bacteria	Firmicutes	Bacillus thuringiensis serovar kurstaki	ZP_04116931.1	9 E-37	509/547
Bacteria	Actinobacteria	Mycobacterium vanbaalenii PYR-1	YP_952025.1	1 E-36	524/547
Bacteria	Actinobacteria	Mycobacterium sp. MCS	YP_641530.1	2 E-36	529/547
Bacteria	Firmicutes	Bacillus thuringiensis IBL 200	ZP_04074284.1	2 E-36	498/547
Bacteria	Actinobacteria	Mycobacterium smegmatis str. MC2	YP_888565.1	2 E-36	542/547

Bacteria	Proteobacteria	Alteromonas macleodii ATCC 27126	ZP_04715128.1	2 E-36	485/547
Bacteria	Proteobacteria	marine gamma proteobacterium HTCC2080	ZP_01625930.1	2 E-36	532/547
Bacteria	Actinobacteria	Brevibacterium linens BL2	ZP_05915622.1	2 E-36	547/547
Bacteria	Proteobacteria	Caulobacter crescentus CB15	NP_419475.1	2 E-36	510/547
Bacteria	Proteobacteria	Sphingomonas sp. SKA58	ZP_01305028.1	3 E-36	497/547
Bacteria	Proteobacteria	Caulobacter sp. K31	YP_001685747.1	3 E-36	506/547
Bacteria	Gemmatimonadet	Gemmatimonas aurantiaca T-27	YP_002760055.1	3 E-36	516/547
Bacteria	Firmicutes	Clostridium bartlettii DSM 16795	ZP_02210512.1	3 E-36	527/547
Bacteria	Proteobacteria	Alteromonadales bacterium TW-7	ZP_01611936.1	3 E-36	507/547
Bacteria	Firmicutes	Bacillus thuringiensis serovar huazhongensis	ZP_04086680.1	4 E-36	498/547
Bacteria	Synergistetes	Thermanaerovibrio acidaminovorans DSM 658	ZP_04468602.1	4 E-36	478/547
Bacteria	Firmicutes	Bacillus cereus F65185;	ZP_04205355.1	4 E-36	508/547
Bacteria	Firmicutes	Bacillus cereus B4264;	YP_002369438.1	4 E-36	501/547
Bacteria	Actinobacteria	Rhodococcus opacus B4	YP_002782857.1	4 E-36	531/547
Bacteria	Actinobacteria	Mycobacterium sp. MCS	YP_637918.1	5 E-36	520/547
Bacteria	Chloroflexi	Thermomicrobium roseum DSM 5159	YP_002521637.1	6 E-36	525/547
Bacteria	Actinobacteria	Streptomyces sp. AA4	ZP_05482384.1	6 E-36	521/547
Bacteria	Actinobacteria	Slackia heliotrinireducens DSM 20476	YP_003143100.1	6 E-36	498/547
Bacteria	Bacteroidetes	Sphingobacterium spiritivorum ATCC 33861	ZP_04777470.1	6 E-36	501/547
Bacteria	Firmicutes	Bacillus mycoides Rock3-17;	ZP_04159007.1	6 E-36	498/547
Bacteria	Firmicutes	Bacillus thuringiensis serovar berliner	ZP_04104371.1	7 E-36	508/547
Bacteria	Proteobacteria	Pseudoalteromonas tunicata D2	ZP_01132311.1	8 E-36	529/547
Archaea	Crenarchaeota	Desulfurococcus kamchatkensis 1221n	YP_002428423.1	8 E-36	516/547
Bacteria	Firmicutes	Clostridium sp. M62/1	ZP_03733037.1	1 E-35	532/547
Bacteria	Firmicutes	Bacillus cereus E33L;	YP_085965.1	1 E-35	508/547
Bacteria	Proteobacteria	Mesorhizobium opportunistum WSM2075	ZP_05812826.1	1 E-35	509/547
Bacteria	Proteobacteria	Rhodospirillum centenum SW	YP_002299313.1	1 E-35	528/547
Bacteria	Firmicutes	Bacillus thuringiensis serovar pakistani	ZP_04122544.1	1 E-35	498/547
Bacteria	Firmicutes	Bacillus sp. NRRL B-14911	ZP_01172950.1	2 E-35	483/547
Bacteria	Actinobacteria	Arthrobacter sp. FB24	YP_829754.1	2 E-35	526/547
Bacteria	Firmicutes	Bacillus cereus ATCC 10987	NP_981072.1	2 E-35	508/547
Bacteria	Firmicutes	Enterococcus casseliflavus EC30	ZP_05645049.1	2 E-35	511/547
Bacteria	Firmicutes	Bacillus sp. SG-1	ZP_01859601.1	2 E-35	482/547
Bacteria	Proteobacteria	Rhodobacter sphaeroides KD131	YP_002520098.1	2 E-35	533/547
Bacteria	Firmicutes	Bacillus cereus Rock1-15;	ZP_04241652.1	2 E-35	498/547
Bacteria	Proteobacteria	marine gamma proteobacterium HTCC2148	ZP_05095596.1	2 E-35	502/547
Bacteria	Proteobacteria	Sphingomonas wittichii RW1	YP_001262893.1	2 E-35	478/547
Bacteria	Bacteroidetes	Robiginitalea biformata HTCC2501	YP_003195083.1	2 E-35	489/547
Bacteria	Firmicutes	Bacillus cereus ATCC 14579	NP_834349.1	3 E-35	498/547
Bacteria	Bacteroidetes	Flavobacteriaceae bacterium 3519-10	YP_003095244.1	3 E-35	497/547
Bacteria	Firmicutes	Clostridium bartlettii DSM 16795	ZP_02212829.1	3 E-35	538/547
Bacteria	Firmicutes	Bacillus cereus BDRD-Cer4;	ZP_04258893.1	3 E-35	498/547
Bacteria	Actinobacteria	Mycobacterium avium 104;	YP_883720.1	3 E-35	519/547
Bacteria	Proteobacteria	Rhodobacter sphaeroides 2.4.1	YP_354522.1	3 E-35	545/547
Bacteria	Chloroflexi	Sphaerobacter thermophilus DSM 20745	ZP_04496396.1	3 E-35	526/547
Bacteria	Bacteroidetes	Flavobacteriales bacterium ALC-1	ZP_02180816.1	4 E-35	523/547
Bacteria	Bacteroidetes	Flavobacteria bacterium BBFL7	ZP_01202131.1	4 E-35	500/547
Bacteria	Firmicutes	Eubacterium biforme DSM 3989	ZP_03488701.1	4 E-35	533/547
Bacteria	Actinobacteria	Mycobacterium avium subsp. avium	ZP_05218540.1	4 E-35	519/547
Bacteria	Actinobacteria	Collinsella stercoris DSM 13279	ZP_03296464.1	4 E-35	520/547
Bacteria	Proteobacteria	gamma proteobacterium NOR5-3	ZP_05127742.1	5 E-35	501/547
Bacteria	Firmicutes	Bacillus cereus m1550;	ZP_04281021.1	5 E-35	501/547
Bacteria	Bacteroidetes	Psychroflexus torquis ATCC 700755	ZP_01251755.1	5 E-35	500/547
Bacteria	Actinobacteria	Mycobacterium avium subsp. paratuberculosis	NP_962983.1	5 E-35	518/547
Bacteria	Proteobacteria	Rhodobacter sphaeroides ATCC 17029	YP_001045396.1	5 E-35	545/547
Bacteria	Firmicutes	Bacillus cereus Rock3-42;	ZP_04224838.1	6 E-35	508/547
Bacteria	Proteobacteria	marine gamma proteobacterium HTCC2148	ZP_05093902.1	8 E-35	505/547
Bacteria	Firmicutes	Bacillus thuringiensis serovar konkukian	YP_038692.1	8 E-35	508/547

Bacteria	Actinobacteria	Mycobacterium abscessus	YP_001703281.1	9 E-35	537/547
Bacteria	Proteobacteria	Sphingomonas wittichii RW1	YP_001262556.1	9 E-35	512/547
Bacteria	Firmicutes	Bacillus cereus ATCC 4342	ZP_04286318.1	1 E-34	476/547
Bacteria	Firmicutes	Bacillus thuringiensis serovar israelensis	ZP_00740772.1	1 E-34	476/547
Bacteria	Firmicutes	Bacillus cereus BDRD-ST24;	ZP_04275562.1	1 E-34	498/547
Bacteria	Firmicutes	Bacillus pseudomycoides DSM 12442	ZP_04153291.1	1 E-34	498/547
Bacteria	Firmicutes	Bacillus cereus G9842;	YP_002448209.1	1 E-34	476/547
Bacteria	Firmicutes	Bacillus cereus 95/8201;	ZP_04253405.1	1 E-34	508/547
Bacteria	Proteobacteria	Shewanella frigidimarina NCIMB 400	YP_752621.1	1 E-34	486/547
Bacteria	Firmicutes	Bacillus thuringiensis serovar andalousiensis	ZP_04098763.1	1 E-34	508/547
Bacteria	Firmicutes	Bacillus anthracis str. A0193	ZP_02396420.1	1 E-34	508/547
Bacteria	Bacteroidetes	Flavobacteria bacterium MS024-2A	ZP_03702347.1	2 E-34	513/547
Bacteria	Firmicutes	Brevibacillus brevis NBRC 100599	YP_002773179.1	2 E-34	528/547

AFUA_2G13310

Eukaryota	Fungi	Aspergillus fumigatus Af293	XP_755664.1	0.0	444/444
Eukaryota	Fungi	Neosartorya fischeri NRRL 181	XP_001260791.1	0.0	453/444
Eukaryota	Fungi	Aspergillus clavatus NRRL 1	XP_001275605.1	1 E-170	449/444
Eukaryota	Fungi	Aspergillus oryzae RIB40	XP_001821770.1	1 E-127	462/444
Eukaryota	Fungi	Aspergillus flavus NRRL3357	XP_002379645.1	1 E-113	412/444
Eukaryota	Fungi	Aspergillus niger CBS 513.88	XP_001399359.1	8 E-88	389/444
Eukaryota	Fungi	Talaromyces stipitatus ATCC 10500	XP_002487293.1	1 E-75	471/444
Eukaryota	Fungi	Talaromyces stipitatus ATCC 10500	XP_002487294.1	1 E-73	473/444
Eukaryota	Fungi	Penicillium marneffei ATCC 18224	XP_002145261.1	3 E-68	468/444
Eukaryota	Fungi	Microsporum canis CBS 113480	EEQ31466.1	1 E-66	426/444
Eukaryota	Fungi	Talaromyces stipitatus ATCC 10500	XP_002487295.1	4 E-66	364/444
Eukaryota	Fungi	Aspergillus nidulans FGSC A4	tpeCBF69910.1	3 E-61	435/444
Eukaryota	Fungi	Penicillium chrysogenum Wisconsin 54-1255	XP_002557952.1	3 E-61	405/444
Eukaryota	Fungi	Coccidioides immitis RS;	XP_001245623.1	2 E-60	389/444
Eukaryota	Fungi	Phaeosphaeria nodorum SN15	XP_001796285.1	9 E-46	364/444
Eukaryota	Fungi	Pyrenophora tritici-repentis Pt-1C-BFP	XP_001935349.1	7 E-44	432/444
Eukaryota	Fungi	Ajellomyces dermatitidis ER-3	EEQ92479.1	6 E-29	369/444
Eukaryota	Fungi	Ajellomyces dermatitidis SLH14081	XP_002626117.1	1 E-28	369/444

AFUA_2G16510

Eukaryota	Fungi	Aspergillus fumigatus Af293	XP_755988.1	0.0	675/675
Eukaryota	Fungi	Neosartorya fischeri NRRL 181	XP_001261114.1	0.0	675/675
Eukaryota	Fungi	Aspergillus clavatus NRRL 1	XP_001275929.1	0.0	663/675
Eukaryota	Fungi	Aspergillus oryzae RIB40	XP_001822433.1	0.0	684/675
Eukaryota	Fungi	Aspergillus flavus NRRL3357	XP_002382524.1	0.0	683/675
Eukaryota	Fungi	Aspergillus nidulans FGSC A4	tpeCBF78594.1	0.0	665/675
Eukaryota	Fungi	Aspergillus nidulans FGSC A4	XP_680604.1	0.0	665/675
Eukaryota	Fungi	Aspergillus niger CBS 513.88	XP_001397226.1	0.0	648/675
Eukaryota	Fungi	Aspergillus terreus NIH2624	XP_001211467.1	0.0	615/675
Eukaryota	Fungi	Penicillium chrysogenum Wisconsin 54-1255	XP_002567211.1	0.0	640/675
Eukaryota	Fungi	Talaromyces stipitatus ATCC 10500	XP_002486938.1	0.0	606/675
Eukaryota	Fungi	Penicillium marneffei ATCC 18224	XP_002145584.1	0.0	627/675
Eukaryota	Fungi	Paracoccidioides brasiliensis Pb01;	EEH35566.1	1 E-154	662/675
Eukaryota	Fungi	Ajellomyces dermatitidis ER-3	EEQ84448.1	1 E-154	663/675
Eukaryota	Fungi	Ajellomyces dermatitidis SLH14081	XP_002626589.1	1 E-154	663/675
Eukaryota	Fungi	Paracoccidioides brasiliensis Pb03;	EEH20372.1	1 E-154	667/675
Eukaryota	Fungi	Ajellomyces capsulatus G186AR	EEH04255.1	1 E-153	660/675
Eukaryota	Fungi	Ajellomyces capsulatus NAm1	XP_001538883.1	1 E-139	616/675
Eukaryota	Fungi	Uncinocarpus reesii 1704	XP_002584145.1	1 E-139	626/675
Eukaryota	Fungi	Coccidioides posadasii C735 delta	EER25245.1	1 E-135	638/675
Eukaryota	Fungi	Coccidioides immitis RS;	XP_001239864.1	1 E-134	638/675

Eukaryota	Fungi	Microsporum canis CBS 113480	EEQ31032.1	1 E-129	632/675
Eukaryota	Fungi	Phaeosphaeria nodorum SN15	XP_001800718.1	1 E-96	652/675
Eukaryota	Fungi	Pyrenophora tritici-repentis Pt-1C-BFP	XP_001940252.1	9 E-95	651/675
Eukaryota	Fungi	Nectria haematococca mpVI 77-13-4	EEU43052.1	6 E-56	578/675
Eukaryota	Fungi	Magnaporthe grisea 70-15	XP_362226.2	4 E-49	585/675
Eukaryota	Fungi	Podospora anserina DSM 980	XP_001907967.1	5 E-47	578/675

AFUA_2G17000

Eukaryota	Fungi	Aspergillus fumigatus Af293	XP_756037.2	0.0	2170/2170

AFUA_2G17040

Eukaryota	Fungi	Aspergillus fumigatus Af293	XP_756038.1	0.0	951/951
Eukaryota	Fungi	Neosartorya fischeri NRRL 181	XP_001261171.1	0.0	970/951
Eukaryota	Fungi	Aspergillus clavatus NRRL 1	XP_001275987.1	0.0	874/951
Eukaryota	Fungi	Aspergillus terreus NIH2624	XP_001209343.1	1 E-88	791/951
Eukaryota	Fungi	Aspergillus nidulans FGSC A4	XP_680530.1	5 E-86	872/951
Eukaryota	Fungi	Aspergillus flavus NRRL3357	XP_002382444.1	5 E-76	840/951
Eukaryota	Fungi	Aspergillus oryzae RIB40	XP_001822355.1	2 E-71	796/951

AFUA_2G17610

Eukaryota	Fungi	Aspergillus fumigatus Af293	XP_756096.1	0.0	888/888
Eukaryota	Fungi	Neosartorya fischeri NRRL 181	XP_001261236.1	0.0	887/888
Eukaryota	Fungi	Aspergillus clavatus NRRL 1	XP_001276036.1	0.0	875/888
Eukaryota	Fungi	Aspergillus terreus NIH2624	XP_001209305.1	0.0	839/888
Eukaryota	Fungi	Aspergillus nidulans FGSC A4	XP_680498.1	0.0	831/888
Eukaryota	Fungi	Aspergillus oryzae RIB40	XP_001822303.1	0.0	765/888
Eukaryota	Fungi	Aspergillus flavus NRRL3357	XP_002382391.1	0.0	763/888
Eukaryota	Fungi	Penicillium chrysogenum Wisconsin 54-1255	XP_002562730.1	0.0	854/888
Eukaryota	Fungi	Penicillium chrysogenum Wisconsin 54-1255	XP_002568997.1	0.0	827/888
Eukaryota	Fungi	Penicillium chrysogenum Wisconsin 54-1255	XP_002559718.1	0.0	852/888
Eukaryota	Fungi	Penicillium chrysogenum Wisconsin 54-1255	XP_002568970.1	0.0	895/888
Eukaryota	Fungi	Nectria haematococca mpVI 77-13-4	EEU43447.1	1 E-154	821/888
Eukaryota	Fungi	Nectria haematococca mpVI 77-13-4	EEU37702.1	1 E-151	843/888
Eukaryota	Fungi	Nectria haematococca mpVI 77-13-4	EEU33414.1	1 E-147	838/888
Eukaryota	Fungi	Gibberella zeae PH-1	XP_383077.1	1 E-142	837/888
Eukaryota	Fungi	Talaromyces stipitatus ATCC 10500	XP_002481940.1	1 E-140	815/888
Eukaryota	Fungi	Gibberella zeae PH-1	XP_383497.1	1 E-137	836/888
Eukaryota	Fungi	Talaromyces stipitatus ATCC 10500	XP_002485727.1	1 E-134	807/888
Eukaryota	Fungi	Gibberella zeae PH-1	XP_391306.1	1 E-132	832/888
Eukaryota	Fungi	Ajellomyces capsulatus G186AR	EEH04081.1	1 E-129	856/888
Eukaryota	Fungi	Coccidioides posadasii C735 delta	EER23016.1	1 E-126	858/888
Eukaryota	Fungi	Talaromyces stipitatus ATCC 10500	XP_002488506.1	1 E-126	841/888
Eukaryota	Fungi	Gibberella zeae PH-1	XP_383628.1	1 E-126	868/888
Eukaryota	Fungi	Talaromyces stipitatus ATCC 10500	XP_002485183.1	1 E-125	844/888
Eukaryota	Fungi	Penicillium chrysogenum Wisconsin 54-1255	XP_002562126.1	1 E-119	867/888
Eukaryota	Fungi	Gibberella zeae PH-1	XP_385256.1	1 E-119	900/888
Eukaryota	Fungi	Nectria haematococca mpVI 77-13-4	EEU40272.1	1 E-118	888/888
Eukaryota	Fungi	Ajellomyces dermatitidis SLH14081	XP_002622429.1	1 E-116	857/888
Eukaryota	Fungi	Ajellomyces dermatitidis ER-3	EEQ85506.1	1 E-115	857/888
Eukaryota	Fungi	Gibberella zeae PH-1	XP_383399.1	1 E-113	915/888
Eukaryota	Fungi	Nectria haematococca mpVI 77-13-4	EEU41749.1	1 E-112	889/888
Eukaryota	Fungi	Penicillium chrysogenum Wisconsin 54-1255	XP_002564657.1	1 E-106	715/888
Eukaryota	Fungi	Microsporum canis CBS 113480	EEQ29345.1	1 E-102	760/888

AFUA_2G17620

Eukaryota	Fungi	Aspergillus fumigatus Af293	XP_756097.1	0.0	805/805
Eukaryota	Fungi	Neosartorya fischeri NRRL 181	XP_001261237.1	0.0	791/805
Eukaryota	Fungi	Aspergillus clavatus NRRL 1	XP_001276037.1	0.0	790/805
Eukaryota	Fungi	Aspergillus oryzae RIB40	XP_001822302.1	0.0	789/805
Eukaryota	Fungi	Aspergillus flavus NRRL3357	XP_002382390.1	0.0	807/805
Eukaryota	Fungi	Magnaporthe grisea 70-15	XP_360402.2	0.0	801/805
Eukaryota	Fungi	Neurospora crassa OR74A	XP_956591.1	0.0	787/805
Eukaryota	Fungi	Podospora anserina DSM 980	XP_001907754.1	0.0	786/805
Eukaryota	Fungi	Myceliophthora thermophila	AAC26221.1	0.0	767/805
Eukaryota	Fungi	Myriococcum thermophilum	ABS45567.2	0.0	767/805
Eukaryota	Fungi	Myriococcum thermophilum	ABS45566.1	0.0	767/805
Eukaryota	Fungi	Aspergillus nidulans FGSC A4	XP_680499.1	0.0	763/805
Eukaryota	Fungi	Neurospora crassa OR74A	XP_958234.1	0.0	811/805
Eukaryota	Fungi	Chaetomium globosum CBS 148.51	XP_001229896.1	0.0	764/805
Eukaryota	Fungi	Gibberella zeae PH-1	XP_389261.1	0.0	749/805
Eukaryota	Fungi	Podospora anserina DSM 980	XP_001903875.1	0.0	790/805
Eukaryota	Fungi	Humicola insolens	AAF69005.1	0.0	783/805
Eukaryota	Fungi	Magnaporthe grisea 70-15	XP_001404273.1	0.0	749/805
Eukaryota	Fungi	Pyrenophora tritici-repentis Pt-1C-BFP	XP_001939778.1	0.0	783/805
Eukaryota	Fungi	Phaeosphaeria nodorum SN15	XP_001801490.1	0.0	778/805
Eukaryota	Fungi	Sclerotinia sclerotiorum 1980 UF-70	XP_001591237.1	0.0	802/805
Eukaryota	Fungi	Botryotinia fuckeliana B05.10	XP_001553707.1	0.0	772/805
Eukaryota	Fungi	Coprinopsis cinerea okayama7#130	XP_001835032.1	1 E-130	742/805
Eukaryota	Fungi	Trametes versicolor	AAC50004.1	1 E-123	749/805
Eukaryota	Fungi	Phanerochaete chrysosporium	CAA61359.1	1 E-123	746/805
Eukaryota	Fungi	Phanerochaete chrysosporium	Q01738.1	1 E-123	747/805
Eukaryota	Fungi	Irpex lacteus	BAD36748.1	1 E-122	751/805
Eukaryota	Fungi	Athelia rolfsii	AAO64483.1	1 E-122	768/805
Eukaryota	Fungi	Grifola frondosa	BAC20641.1	1 E-121	750/805
Eukaryota	Fungi	Coniophora puteana	BAD32781.1	1 E-121	747/805
Eukaryota	Fungi	Pycnoporus cinnabarinus	AAC32197.1	1 E-120	750/805
Eukaryota	Fungi	Trametes versicolor	AAO32063.1	1 E-119	749/805
Eukaryota	Fungi	Phanerochaete chrysosporium	prf2118247A	1 E-117	744/805
Eukaryota	Fungi	Ceriporiopsis subvermispora	ACF60617.1	1 E-115	748/805
Eukaryota	Fungi	Neosartorya fischeri NRRL 181	XP_001265679.1	1 E-111	734/805
Eukaryota	Fungi	Nectria haematococca mpVI 77-13-4	EEU36349.1	1 E-111	737/805
Eukaryota	Fungi	Aspergillus clavatus NRRL 1	XP_001273175.1	1 E-109	732/805
Eukaryota	Fungi	Aspergillus fumigatus Af293	XP_749254.1	1 E-109	734/805
Eukaryota	Fungi	Botryotinia fuckeliana B05.10	XP_001558156.1	1 E-106	732/805
Eukaryota	Fungi	Gibberella zeae PH-1	XP_383918.1	1 E-104	744/805
Eukaryota	Fungi	Gibberella zeae PH-1	XP_385048.1	1 E-104	757/805
Eukaryota	Fungi	Nectria haematococca mpVI 77-13-4	EEU37222.1	1 E-102	720/805
Eukaryota	Fungi	Verticillium albo-atrum VaMs.102	EEY20241.1	2 E-93	677/805
Eukaryota	Fungi	Pyrenophora tritici-repentis Pt-1C-BFP	XP_001937164.1	9 E-93	761/805
Eukaryota	Fungi	Sclerotinia sclerotiorum 1980 UF-70	XP_001593342.1	7 E-92	743/805
Eukaryota	Fungi	Botryotinia fuckeliana B05.10	XP_001559563.1	5 E-90	733/805
Eukaryota	Fungi	Aspergillus niger CBS 513.88	XP_001402432.1	3 E-84	705/805

AFUA_2G18070

Eukaryota	Fungi	Aspergillus fumigatus Af293	XP_756144.1	1 E-128	222/222
Eukaryota	Fungi	Aspergillus nidulans FGSC A4	XP_681581.1	1 E-105	210/222
Eukaryota	Fungi	Aspergillus oryzae RIB40	XP_001825086.1	2 E-96	203/222
Eukaryota	Fungi	Aspergillus flavus NRRL3357	XP_002381783.1	3 E-96	203/222
Eukaryota	Fungi	Aspergillus flavus NRRL3357	XP_002373501.1	6 E-42	198/222
Eukaryota	Fungi	Aspergillus oryzae RIB40	XP_001818280.1	2 E-40	194/222
Eukaryota	Fungi	Aspergillus flavus NRRL3357	XP_002384685.1	2 E-21	179/222

Eukaryota	Fungi	Aspergillus oryzae	AAB19701.1	3 E-21	179/222
Eukaryota	Fungi	Aspergillus oryzae RIB40	XP_001827477.1	3 E-21	179/222
Eukaryota	Fungi	Aspergillus oryzae RIB40	XP_001823175.1	5 E-19	178/222
Eukaryota	Fungi	Aspergillus flavus NRRL3357	XP_002378511.1	9 E-19	178/222
Eukaryota	Fungi	Penicillium citrinum	P47189.1	2 E-18	192/222
Eukaryota	Fungi	Aspergillus nidulans FGSC A4	XP_660997.1	7 E-18	190/222
Eukaryota	Fungi	Microsporum canis CBS 113480	EEQ34336.1	4 E-16	193/222
Eukaryota	Fungi	Coccidioides posadasii;	AAY45752.1	6 E-16	204/222
Eukaryota	Fungi	Coccidioides immitis RS;	XP_001243453.1	8 E-16	178/222
Eukaryota	Fungi	Aspergillus nidulans FGSC A4	XP_661563.1	2 E-15	184/222
Eukaryota	Fungi	Coccidioides posadasii C735 delta	EER28352.1	3 E-15	190/222
Eukaryota	Fungi	Coccidioides posadasii;	AAY45757.1	3 E-15	190/222
Eukaryota	Fungi	Sclerotinia sclerotiorum 1980 UF-70	XP_001585173.1	2 E-14	186/222
Eukaryota	Fungi	Coccidioides immitis RS;	XP_001241450.1	3 E-14	180/222
Eukaryota	Fungi	Aspergillus flavus	P46073.1	5 E-14	189/222
Eukaryota	Fungi	Aspergillus terreus NIH2624	XP_001214119.1	5 E-14	178/222
Eukaryota	Fungi	Coccidioides posadasii;	AAY45758.1	7 E-14	195/222
Eukaryota	Fungi	Microsporum canis CBS 113480	EEQ31438.1	8 E-14	181/222
Eukaryota	Fungi	Aspergillus nidulans FGSC A4	XP_681231.1	1 E-13	182/222
Eukaryota	Fungi	Aspergillus fumigatus A1163	EDP47444.1	3 E-13	189/222
Eukaryota	Fungi	Botryotinia fuckeliana B05.10	XP_001551183.1	4 E-13	186/222
Eukaryota	Fungi	Coccidioides immitis RS;	XP_001239079.1	5 E-13	190/222
Eukaryota	Fungi	Neosartorya fischeri NRRL 181	XP_001262576.1	8 E-13	186/222
Eukaryota	Fungi	Aspergillus fumigatus Af293	XP_746456.2	9 E-13	186/222
Eukaryota	Fungi	Ajellomyces capsulatus NAm1	XP_001538183.1	3 E-12	179/222
Eukaryota	Fungi	Verticillium albo-atrum VaMs.102	EEY14413.1	8 E-12	187/222

AFUA_3G01280

Eukaryota	Fungi	Aspergillus fumigatus Af293	XP_748449.1	1 E-180	308/308
Eukaryota	Fungi	Neosartorya fischeri NRRL 181	XP_001258770.1	1 E-170	308/308
Eukaryota	Fungi	Aspergillus flavus NRRL3357	XP_002377221.1	1 E-112	300/308
Eukaryota	Fungi	Aspergillus clavatus NRRL 1	XP_001273767.1	1 E-101	267/308
Eukaryota	Fungi	Microsporum canis CBS 113480	EEQ29423.1	3 E-98	297/308
Eukaryota	Fungi	Ajellomyces dermatitidis ER-3	EEQ87567.1	2 E-96	296/308
Eukaryota	Fungi	Ajellomyces dermatitidis SLH14081	XP_002624057.1	3 E-96	296/308
Eukaryota	Fungi	Uncinocarpus reesii 1704	XP_002584759.1	2 E-94	298/308
Eukaryota	Fungi	Coccidioides posadasii C735 delta	EER25369.1	6 E-85	294/308
Eukaryota	Fungi	Aspergillus niger CBS 513.88	XP_001400378.1	6 E-77	283/308
Eukaryota	Fungi	Penicillium marneffei ATCC 18224	XP_002143380.1	1 E-76	318/308
Eukaryota	Fungi	Talaromyces stipitatus ATCC 10500	XP_002479701.1	1 E-76	309/308
Eukaryota	Fungi	Penicillium marneffei ATCC 18224	XP_002143415.1	5 E-52	298/308
Eukaryota	Fungi	Aspergillus niger CBS 513.88	XP_001397904.1	5 E-45	290/308
Eukaryota	Fungi	Penicillium marneffei ATCC 18224	XP_002145485.1	2 E-43	303/308
Eukaryota	Fungi	Aspergillus clavatus NRRL 1	XP_001269506.1	1 E-40	298/308
Eukaryota	Fungi	Neosartorya fischeri NRRL 181	XP_001262288.1	3 E-40	298/308
Eukaryota	Fungi	Ajellomyces capsulatus G186AR	EEH11439.1	4 E-39	309/308
Eukaryota	Fungi	Botryotinia fuckeliana B05.10	XP_001551986.1	8 E-39	302/308
Eukaryota	Fungi	Ajellomyces capsulatus NAm1	XP_001541601.1	2 E-38	305/308
Eukaryota	Fungi	Aspergillus flavus NRRL3357	XP_002377446.1	5 E-38	303/308
Eukaryota	Fungi	Ajellomyces capsulatus H143	EER39255.1	1 E-37	309/308
Eukaryota	Fungi	Talaromyces stipitatus ATCC 10500	XP_002482733.1	2 E-37	302/308
Eukaryota	Fungi	Aspergillus oryzae RIB40	XP_001825849.1	2 E-37	303/308
Eukaryota	Fungi	Phaeosphaeria nodorum SN15	XP_001798901.1	3 E-36	296/308
Eukaryota	Fungi	Penicillium marneffei ATCC 18224	XP_002149315.1	2 E-35	302/308
Eukaryota	Fungi	Paracoccidioides brasiliensis Pb03;	EEH19323.1	4 E-33	302/308
Eukaryota	Fungi	Paracoccidioides brasiliensis Pb01;	EEH38137.1	4 E-33	302/308
Eukaryota	Fungi	Paracoccidioides brasiliensis Pb18;	EEH47689.1	4 E-33	302/308

Eukaryota	Fungi	Ajellomyces dermatitidis SLH14081	XP_002623969.1	4 E-32	301/308
Eukaryota	Fungi	Pyrenophora tritici-repentis Pt-1C-BFP	XP_001935039.1	1 E-29	259/308
Eukaryota	Fungi	Gibberella zeae PH-1	XP_380220.1	5 E-28	278/308
Bacteria	Acidobacteria	Candidatus Solibacter usitatus Ellin6076	YP_824464.1	3 E-18	282/308
Bacteria	Cyanobacteria	Gloeobacter violaceus PCC 7421	NP_924256.1	1 E-17	284/308
Bacteria	Actinobacteria	Kineococcus radiotolerans SRS30216	YP_001364039.1	2 E-17	269/308
Bacteria	Chlorobi	Chlorobium phaeobacteroides BS1	YP_001959908.1	1 E-14	291/308
Bacteria	Cyanobacteria	Synechococcus sp. PCC 7335	ZP_05039576.1	2 E-13	282/308
Bacteria	Proteobacteria	Variovorax paradoxus S110	YP_002948166.1	3 E-13	272/308
Bacteria	Proteobacteria	Geobacter uraniireducens Rf4	YP_001231304.1	3 E-13	275/308
Bacteria	Chloroflexi	Herpetosiphon aurantiacus ATCC 23779	YP_001543820.1	1 E-12	262/308

AFUA_3G01290

Eukaryota	Fungi	Aspergillus fumigatus Af293	XP_748450.1	0.0	382/382
Eukaryota	Fungi	Neosartorya fischeri NRRL 181	XP_001258772.1	0.0	382/382
Eukaryota	Fungi	Penicillium chrysogenum Wisconsin 54-1255	XP_002567696.1	1 E-128	382/382
Eukaryota	Fungi	Aspergillus terreus NIH2624	XP_001217432.1	1 E-122	377/382
Eukaryota	Fungi	Aspergillus terreus NIH2624	XP_001208580.1	1 E-116	376/382
Eukaryota	Fungi	Penicillium chrysogenum Wisconsin 54-1255	XP_002556784.1	1 E-115	382/382
Eukaryota	Fungi	Paracoccidioides brasiliensis Pb18;	EEH49677.1	1 E-82	391/382
Eukaryota	Fungi	Paracoccidioides brasiliensis Pb03;	EEH23229.1	3 E-82	391/382
Eukaryota	Fungi	Talaromyces stipitatus ATCC 10500	XP_002481727.1	5 E-80	373/382
Eukaryota	Fungi	Paracoccidioides brasiliensis Pb01;	EEH36833.1	5 E-80	391/382
Eukaryota	Fungi	Penicillium marneffei ATCC 18224	XP_002147577.1	3 E-79	373/382
Eukaryota	Fungi	Ajellomyces capsulatus NAm1	XP_001543389.1	6 E-79	386/382
Eukaryota	Fungi	Ajellomyces capsulatus G186AR	EEH05294.1	7 E-79	386/382
Eukaryota	Fungi	Ajellomyces capsulatus H143	EER41059.1	2 E-78	386/382
Eukaryota	Fungi	Aspergillus terreus NIH2624	XP_001217552.1	4 E-75	387/382
Eukaryota	Fungi	Ajellomyces dermatitidis SLH14081	XP_002628565.1	1 E-74	387/382
Eukaryota	Fungi	Ajellomyces dermatitidis ER-3	EEQ89374.1	2 E-74	387/382
Eukaryota	Fungi	Talaromyces stipitatus ATCC 10500	XP_002340601.1	3 E-72	358/382
Eukaryota	Fungi	Aspergillus niger CBS 513.88	XP_001389943.1	1 E-64	379/382
Eukaryota	Fungi	Aspergillus niger CBS 513.88	XP_001398627.1	8 E-61	333/382
Eukaryota	Fungi	Sclerotinia sclerotiorum 1980 UF-70	XP_001592890.1	4 E-49	359/382
Eukaryota	Fungi	Nectria haematococca mpVI 77-13-4	EEU39457.1	1 E-47	370/382
Eukaryota	Fungi	Penicillium marneffei ATCC 18224	XP_002143222.1	2 E-45	385/382
Eukaryota	Fungi	Botryotinia fuckeliana B05.10	XP_001548259.1	3 E-45	366/382
Eukaryota	Fungi	Verticillium albo-atrum VaMs.102	EEY14776.1	5 E-45	367/382
Eukaryota	Fungi	Phaeosphaeria nodorum SN15	XP_001804025.1	1 E-44	389/382
Eukaryota	Fungi	Nectria haematococca mpVI 77-13-4	EEU35710.1	2 E-44	369/382
Eukaryota	Fungi	Sclerotinia sclerotiorum 1980 UF-70	XP_001589086.1	3 E-44	357/382
Eukaryota	Fungi	Pyrenophora tritici-repentis Pt-1C-BFP	XP_001939615.1	1 E-43	390/382
Eukaryota	Fungi	Nectria haematococca mpVI 77-13-4	EEU40290.1	2 E-43	368/382
Eukaryota	Fungi	Talaromyces stipitatus ATCC 10500	XP_002479517.1	9 E-43	372/382
Eukaryota	Fungi	Gibberella zeae PH-1	XP_382871.1	4 E-41	370/382
Eukaryota	Fungi	Debaryomyces hansenii	CAG85518.2	4 E-41	370/382
Eukaryota	Fungi	Aspergillus nidulans FGSC A4	XP_682584.1	6 E-41	400/382
Eukaryota	Fungi	Botryotinia fuckeliana B05.10	XP_001558960.1	3 E-40	409/382
Eukaryota	Fungi	Debaryomyces hansenii CBS767	XP_457512.1	3 E-40	370/382
Eukaryota	Fungi	Gibberella zeae PH-1	XP_389896.1	1 E-39	367/382
Eukaryota	Fungi	Phaeosphaeria nodorum SN15	XP_001800345.1	4 E-39	381/382
Eukaryota	Fungi	Aspergillus niger CBS 513.88	XP_001390841.1	4 E-39	381/382
Eukaryota	Fungi	Sclerotinia sclerotiorum 1980 UF-70	XP_001597603.1	5 E-39	404/382
Eukaryota	Fungi	Aspergillus oryzae RIB40	XP_001823840.1	9 E-39	400/382
Eukaryota	Fungi	Magnaporthe grisea 70-15	XP_369285.1	1 E-38	373/382
Eukaryota	Fungi	Pyrenophora tritici-repentis Pt-1C-BFP	XP_001935714.1	7 E-38	389/382
Eukaryota	Fungi	Talaromyces stipitatus ATCC 10500	XP_002478102.1	8 E-37	405/382

Eukaryota	Fungi	Phaeosphaeria nodorum SN15	XP_001797766.1	9 E-37	390/382
Eukaryota	Fungi	Chaetomium globosum CBS 148.51	XP_001225470.1	2 E-35	395/382
Eukaryota	Fungi	Verticillium albo-atrum VaMs.102	EEY14021.1	2 E-35	363/382
Eukaryota	Fungi	Debaryomyces hansenii	CAG85519.2	2 E-35	370/382
Eukaryota	Fungi	Aspergillus flavus NRRL3357	XP_002380895.1	3 E-35	392/382
Eukaryota	Fungi	Nectria haematococca mpVI 77-13-4	EEU42306.1	4 E-35	372/382
Eukaryota	Fungi	Debaryomyces hansenii CBS767	XP_457513.1	4 E-35	370/382
Eukaryota	Fungi	Pichia guilliermondii ATCC 6260	XP_001481913.1	1 E-34	365/382
Eukaryota	Fungi	Candida dubliniensis CD36	XP_002418703.1	1 E-34	354/382
Eukaryota	Fungi	Candida tropicalis MYA-3404	XP_002547502.1	1 E-34	363/382
Eukaryota	Fungi	Pichia guilliermondii ATCC 6260	EDK41578.2	3 E-34	365/382
Eukaryota	Fungi	Candida albicans WO-1	EEQ47333.1	2 E-33	354/382
Eukaryota	Fungi	Candida albicans SC5314	XP_716016.1	2 E-33	354/382
Eukaryota	Fungi	Lachancea thermotolerans CBS 6340	XP_002555879.1	2 E-33	359/382
Eukaryota	Fungi	Chaetomium globosum CBS 148.51	XP_001223101.1	3 E-33	375/382
Eukaryota	Fungi	Magnaporthe grisea 70-15	XP_362613.2	4 E-33	391/382
Eukaryota	Fungi	Nectria haematococca mpVI 77-13-4	EEU44267.1	1 E-32	370/382
Eukaryota	Fungi	Podospora anserina DSM 980	XP_001906597.1	3 E-31	314/382
Eukaryota	Metazoa	Ciona intestinalis	XP_002125889.1	7 E-31	348/382
Eukaryota	Metazoa	Ciona intestinalis	XP_002129080.1	1 E-29	349/382
Eukaryota	Metazoa	Strongylocentrotus purpuratus	XP_781398.2	8 E-29	350/382
Eukaryota	Fungi	Pichia stipitis CBS 6054	XP_001384153.1	3 E-28	365/382
Eukaryota	Fungi	Clavispora lusitaniae ATCC 42720	XP_002618453.1	1 E-26	358/382
Eukaryota	Fungi	Nectria haematococca mpVI 77-13-4	EEU41748.1	1 E-26	358/382
Eukaryota	Metazoa	Branchiostoma floridae	XP_002593442.1	2 E-26	341/382
Eukaryota	Metazoa	Ciona intestinalis	XP_002126981.1	1 E-25	355/382
Eukaryota	Metazoa	Branchiostoma floridae	XP_002593446.1	3 E-24	349/382
Eukaryota	Metazoa	Nematostella vectensis	XP_001637313.1	2 E-23	351/382
Eukaryota	Viridiplantae	Glycine max	ACU24451.1	3 E-23	341/382
Bacteria	Proteobacteria	Pseudovibrio sp. JE062	ZP_05086043.1	4 E-23	355/382
Eukaryota	Fungi	Gibberella zeae PH-1	XP_387565.1	6 E-23	382/382
Eukaryota	Viridiplantae	Ricinus communis	XP_002523879.1	4 E-22	347/382
Eukaryota	Viridiplantae	Sorghum bicolor;	XP_002449028.1	5 E-22	350/382
Eukaryota	Fungi	Postia placenta Mad-698-R	XP_002472936.1	1 E-21	365/382
Eukaryota	Viridiplantae	Vitis vinifera	CAO62380.1	3 E-21	338/382
Eukaryota	Viridiplantae	Vitis vinifera	CAN79702.1	4 E-21	338/382
Eukaryota	Viridiplantae	Populus trichocarpa	XP_002299779.1	5 E-21	349/382
Eukaryota	Fungi	Aspergillus oryzae RIB40	XP_001825770.1	1 E-20	325/382
Eukaryota	Metazoa	Oncorhynchus mykiss	NP_001158717.1	1 E-20	326/382
Eukaryota	Fungi	Aspergillus flavus NRRL3357	XP_002377361.1	1 E-20	325/382
Eukaryota	Viridiplantae	Micromonas sp. RCC299	XP_002502564.1	2 E-20	320/382
Eukaryota	Viridiplantae	Arabidopsis thaliana	BAC42443.1	4 E-20	340/382
Eukaryota	Viridiplantae	Arabidopsis thaliana	NP_680200.1	4 E-20	340/382
Eukaryota	Metazoa	Tetraodon nigroviridis	CAG05093.1	6 E-20	326/382
Eukaryota	Fungi	Cryptococcus neoformans var. neoformans	XP_569706.1	8 E-20	411/382
Bacteria	Proteobacteria	Sulfitobacter sp. EE-36	ZP_00955936.1	1 E-19	348/382
Eukaryota	Fungi	Aspergillus clavatus NRRL 1	XP_001273196.1	1 E-19	318/382
Eukaryota	Fungi	Cryptococcus neoformans var. neoformans	XP_776770.1	2 E-19	411/382
Eukaryota	Fungi	Neosartorya fischeri NRRL 181	XP_001265575.1	3 E-19	327/382
Eukaryota	Fungi	Aspergillus terreus NIH2624	XP_001214106.1	1 E-18	346/382
Eukaryota	Metazoa	Ciona intestinalis	XP_002123031.1	1 E-18	327/382
Eukaryota	Fungi	Lodderomyces elongisporus NRRL YB-4239	XP_001526009.1	1 E-18	391/382
Eukaryota	Fungi	Penicillium marneffei ATCC 18224	XP_002145838.1	3 E-18	374/382
Eukaryota	Viridiplantae	Solanum demissum	AAU90286.1	4 E-18	332/382
Eukaryota	Viridiplantae	Arabidopsis thaliana	NP_190005.1	4 E-18	340/382
Eukaryota	Viridiplantae	Solanum demissum	AAU90296.1	4 E-18	332/382
Eukaryota	Fungi	Cryptococcus neoformans var. neoformans	XP_773988.1	5 E-18	343/382
Eukaryota	Fungi	Neurospora crassa OR74A	XP_958599.1	5 E-18	372/382

Eukaryota	Fungi	Cryptococcus neoformans var. neoformans	XP_572726.1	7 E-18	343/382
Eukaryota	Viridiplantae	Oryza sativa Japonica Group	NP_001065804.1	2 E-17	352/382
Eukaryota	Fungi	Postia placenta Mad-698-R	XP_002472841.1	2 E-17	334/382
Eukaryota	Fungi	Aspergillus fumigatus Af293	XP_749159.1	2 E-17	327/382
Eukaryota	Amoebozoa	Dictyostelium discoideum AX4	XP_639989.1	4 E-17	374/382
Eukaryota	Metazoa	Ciona intestinalis	XP_002124648.1	5 E-17	349/382
Eukaryota	Metazoa	Taeniopygia guttata	B5FXE5.1	5 E-17	339/382
Eukaryota	Metazoa	Taeniopygia guttata	XP_002193831.1	5 E-17	308/382
Eukaryota	Fungi	Uncinocarpus reesii 1704	XP_002542193.1	5 E-17	356/382
Eukaryota	Viridiplantae	Sorghum bicolor;	XP_002451553.1	8 E-17	351/382
Eukaryota	Metazoa	Branchiostoma floridae	XP_002593444.1	2 E-16	327/382
Eukaryota	Fungi	Penicillium chrysogenum Wisconsin 54-1255	XP_002560441.1	3 E-16	327/382
Eukaryota	Viridiplantae	Physcomitrella patens subsp. patens	XP_001780589.1	3 E-16	328/382
Bacteria	Actinobacteria	Streptomyces sviceus ATCC 29083	ZP_05019640.1	5 E-16	326/382
Eukaryota	Metazoa	Ciona intestinalis	XP_002121166.1	6 E-16	336/382
Eukaryota	Fungi	Gibberella zeae PH-1	XP_383039.1	8 E-16	327/382
Eukaryota	Fungi	Laccaria bicolor S238N-H82	XP_001883163.1	1 E-15	357/382
Bacteria	Proteobacteria	Oceanibulbus indolifex HEL-45	ZP_02152607.1	1 E-15	348/382
Eukaryota	Fungi	Microsporum canis CBS 113480	EEQ27949.1	2 E-15	361/382
Eukaryota	Fungi	Ustilago maydis 521	XP_758114.1	2 E-15	394/382
Eukaryota	Amoebozoa	Dictyostelium discoideum AX4	XP_639988.1	4 E-15	324/382
Eukaryota	Metazoa	Xenopus (Silurana) tropicalis	NP_001135491.1	5 E-15	309/382
Eukaryota	Fungi	Podospora anserina DSM 980	XP_001905548.1	8 E-15	312/382
Eukaryota	Fungi	Aspergillus niger CBS 513.88	XP_001397262.1	9 E-15	325/382
Eukaryota	Fungi	Nectria haematococca mpVI 77-13-4	EEU38339.1	1 E-14	323/382
Eukaryota	Fungi	Talaromyces stipitatus ATCC 10500	XP_002481064.1	1 E-14	317/382
Bacteria	Cyanobacteria	Prochlorococcus marinus str. MIT	YP_001549976.1	2 E-14	368/382
Eukaryota	Metazoa	Danio rerio	XP_685679.2	2 E-14	318/382
Eukaryota	Fungi	Nectria haematococca mpVI 77-13-4	EEU37195.1	2 E-14	327/382
Eukaryota	Fungi	Uncinocarpus reesii 1704	XP_002582950.1	4 E-14	308/382
Bacteria	Actinobacteria	Stackebrandtia nassauensis DSM 44728	ZP_04480432.1	5 E-14	348/382
Bacteria	Proteobacteria	Congregibacter litoralis KT71	ZP_01102534.1	9 E-14	310/382
Eukaryota	stramenopiles	Phytophthora infestans	CAI72284.1	1 E-13	345/382
Bacteria	Cyanobacteria	Arthrospira maxima CS-328	ZP_03272371.1	2 E-13	349/382
Bacteria	Actinobacteria	Actinomyces odontolyticus ATCC 17982	ZP_02044394.1	3 E-13	353/382
Eukaryota	Viridiplantae	Micromonas pusilla CCMP1545	EEH59110.1	7 E-13	328/382
Eukaryota	Metazoa	Xenopus laevis	NP_001091397.1	1 E-12	309/382
Eukaryota	Fungi	Chaetomium globosum CBS 148.51	XP_001225988.1	2 E-12	310/382
Eukaryota	Fungi	Nectria haematococca mpVI 77-13-4	EEU42238.1	3 E-12	308/382
Bacteria	Cyanobacteria	Synechococcus sp. PCC 7002	YP_001735357.1	3 E-12	311/382
Bacteria	Actinobacteria	Brachybacterium faecium DSM 4810	YP_003156102.1	3 E-12	337/382
Bacteria	Proteobacteria	marine gamma proteobacterium HTCC2080	ZP_01627026.1	4 E-12	315/382
Eukaryota	Fungi	Ustilago maydis 521	tpgDAA04934.1	4 E-12	321/382
Bacteria	Bacteroidetes	Capnocytophaga gingivalis ATCC 33624	ZP_04058377.1	7 E-12	327/382
Eukaryota	Metazoa	Pan troglodytes	XP_001170630.1	9 E-12	325/382
Bacteria	Actinobacteria	Saccharopolyspora erythraea NRRL 2338	YP_001102692.1	9 E-12	322/382
Eukaryota	Fungi	Ustilago maydis 521	XP_757577.1	1 E-11	321/382
Bacteria	Proteobacteria	Rhodopseudomonas palustris BisA53	YP_780605.1	1 E-11	307/382
Eukaryota	Metazoa	Homo sapiens	NP_116186.1	1 E-11	325/382
Eukaryota	Metazoa	Macaca mulatta	XP_001108666.1	1 E-11	325/382
Eukaryota	Metazoa	Rattus norvegicus	NP_001132955.1	5 E-11	306/382
Eukaryota	Metazoa	Mus musculus	NP_835159.1	5 E-11	306/382
Eukaryota	Fungi	Coprinopsis cinerea okayama7#130	XP_001834811.1	8 E-11	334/382

AFUA_3G02450

Eukaryota	Fungi	Aspergillus fumigatus Af293	XP_748568.1	0.0	1178/1178
Eukaryota	Fungi	Neosartorya fischeri NRRL 181	XP_001258907.1	0.0	958/1178

Eukaryota	Fungi	Nectria haematococca mpVI 77-13-4	EEU37420.1	0.0	1140/1178
Eukaryota	Fungi	Aspergillus flavus NRRL3357	XP_002380817.1	0.0	1159/1178
Eukaryota	Fungi	Aspergillus oryzae RIB40	XP_001823690.1	0.0	1141/1178
Bacteria	Chloroflexi	Herpetosiphon aurantiacus ATCC 23779	YP_001544967.1	4 E-23	1063/1178

AFUA_3G03540

Eukaryota	Fungi	Aspergillus fumigatus Af293	XP_748674.1	0.0	808/808

AFUA_3G03550

Eukaryota	Fungi	Aspergillus fumigatus Af293	XP_748675.1	1 E-116	203/203
Eukaryota	Fungi	Aspergillus nidulans FGSC A4	XP_659640.1	6 E-57	180/203
Eukaryota	Fungi	Penicillium marneffei ATCC 18224	XP_002149768.1	2 E-16	183/203
Eukaryota	Fungi	Talaromyces stipitatus ATCC 10500	XP_002340039.1	1 E-13	183/203
Eukaryota	Fungi	Aspergillus nidulans FGSC A4	XP_660989.1	1 E-12	179/203

AFUA_3G04100

Eukaryota	Fungi	Aspergillus fumigatus Af293	XP_748728.1	4 E-48	94/94

AFUA_3G04300

Eukaryota	Fungi	Aspergillus fumigatus Af293	XP_748747.1	0.0	1282/1282
Eukaryota	Fungi	Neosartorya fischeri NRRL 181	XP_001259186.1	0.0	1277/1282
Eukaryota	Fungi	Aspergillus clavatus NRRL 1	XP_001273523.1	0.0	1269/1282
Eukaryota	Fungi	Aspergillus terreus NIH2624	XP_001213430.1	0.0	1235/1282
Eukaryota	Fungi	Penicillium chrysogenum Wisconsin 54-1255	XP_002560076.1	0.0	1209/1282
Eukaryota	Fungi	Talaromyces stipitatus ATCC 10500	XP_002486429.1	0.0	1161/1282
Eukaryota	Fungi	Penicillium marneffei ATCC 18224	XP_002152718.1	0.0	1159/1282
Eukaryota	Fungi	Ajellomyces dermatitidis SLH14081	XP_002620493.1	0.0	1245/1282
Eukaryota	Fungi	Ajellomyces dermatitidis ER-3	EEQ86097.1	0.0	1189/1282
Eukaryota	Fungi	Coccidioides immitis RS;	XP_001241329.1	0.0	1206/1282
Eukaryota	Fungi	Coccidioides posadasii C735 delta	EER28284.1	0.0	1206/1282
Eukaryota	Fungi	Ajellomyces capsulatus G186AR	EEH03411.1	0.0	1271/1282
Eukaryota	Fungi	Paracoccidioides brasiliensis Pb18;	EEH43979.1	0.0	1249/1282
Eukaryota	Fungi	Paracoccidioides brasiliensis Pb01;	EEH39051.1	0.0	1277/1282
Eukaryota	Fungi	Aspergillus nidulans FGSC A4	tpeCBF81166.1	0.0	1153/1282
Eukaryota	Fungi	Microsporum canis CBS 113480	EEQ29965.1	0.0	1118/1282
Eukaryota	Fungi	Aspergillus nidulans FGSC A4	XP_663401.1	0.0	1135/1282
Eukaryota	Fungi	Ajellomyces capsulatus NAm1	XP_001537336.1	0.0	1211/1282
Eukaryota	Fungi	Ajellomyces capsulatus H143	EER44833.1	0.0	1232/1282
Eukaryota	Fungi	Uncinocarpus reesii 1704	XP_002543048.1	0.0	1125/1282
Eukaryota	Fungi	Sclerotinia sclerotiorum 1980 UF-70	XP_001589667.1	0.0	1214/1282
Eukaryota	Fungi	Gibberella zeae PH-1	XP_385255.1	0.0	1229/1282
Eukaryota	Fungi	Nectria haematococca mpVI 77-13-4	EEU40273.1	0.0	1237/1282
Eukaryota	Fungi	Podospora anserina DSM 980	XP_001906698.1	0.0	1286/1282
Eukaryota	Fungi	Neurospora crassa OR74A	XP_961831.2	0.0	1181/1282
Eukaryota	Fungi	Phaeosphaeria nodorum SN15	XP_001798765.1	0.0	1138/1282

AFUA_3G07850

Eukaryota	Fungi	Aspergillus fumigatus Af293	XP_754828.2	0.0	919/919
Eukaryota	Fungi	Neosartorya fischeri NRRL 181	XP_001263657.1	0.0	919/919
Eukaryota	Fungi	Aspergillus clavatus NRRL 1	XP_001270801.1	0.0	906/919
Eukaryota	Fungi	Aspergillus terreus NIH2624	XP_001209038.1	0.0	903/919
Eukaryota	Fungi	Aspergillus niger	CAC41019.1	0.0	878/919
Eukaryota	Fungi	Aspergillus niger CBS 513.88	XP_001400269.1	0.0	878/919

Eukaryota	Fungi	Aspergillus flavus NRRL3357	XP_002373630.1	0.0	907/919
Eukaryota	Fungi	Aspergillus oryzae RIB40	XP_001818403.1	0.0	878/919
Eukaryota	Fungi	Penicillium chrysogenum Wisconsin 54-1255	XP_002563136.1	0.0	893/919
Eukaryota	Fungi	Aspergillus nidulans FGSC A4	tpeCBF83695.1	0.0	899/919
Eukaryota	Fungi	Emericella nidulans	tpgDAA01787.1	0.0	868/919
Eukaryota	Fungi	Aspergillus nidulans FGSC A4	XP_660550.1	0.0	866/919
Eukaryota	Fungi	Penicillium marneffei ATCC 18224	XP_002153462.1	0.0	890/919
Eukaryota	Fungi	Talaromyces stipitatus ATCC 10500	XP_002487962.1	0.0	888/919
Eukaryota	Fungi	Uncinocarpus reesii 1704	XP_002541894.1	0.0	857/919
Eukaryota	Fungi	Paracoccidioides brasiliensis Pb18;	EEH43764.1	0.0	842/919
Eukaryota	Fungi	Paracoccidioides brasiliensis Pb03;	EEH21555.1	0.0	841/919
Eukaryota	Fungi	Paracoccidioides brasiliensis Pb01;	EEH39262.1	0.0	848/919
Eukaryota	Fungi	Ajellomyces dermatitidis SLH14081	XP_002628849.1	0.0	844/919
Eukaryota	Fungi	Ajellomyces capsulatus H143	EER37061.1	0.0	809/919
Eukaryota	Fungi	Ajellomyces capsulatus G186AR	EEH04721.1	0.0	809/919
Eukaryota	Fungi	Ajellomyces capsulatus NAm1	XP_001538713.1	0.0	810/919
Eukaryota	Fungi	Microsporum canis CBS 113480	EEQ34741.1	0.0	877/919
Eukaryota	Fungi	Coccidioides immitis RS;	XP_001248569.1	0.0	825/919
Eukaryota	Fungi	Coccidioides posadasii C735 delta	EER28722.1	0.0	821/919
Eukaryota	Fungi	Sclerotinia sclerotiorum 1980 UF-70	XP_001591119.1	0.0	868/919
Eukaryota	Fungi	Botryotinia fuckeliana B05.10	XP_001547950.1	0.0	913/919
Eukaryota	Fungi	Phaeosphaeria nodorum SN15	XP_001794630.1	0.0	823/919
Eukaryota	Fungi	Neurospora crassa OR74A	XP_965646.1	0.0	881/919
Eukaryota	Fungi	Podospora anserina DSM 980	XP_001913070.1	0.0	858/919
Eukaryota	Fungi	Magnaporthe grisea 70-15	XP_367841.1	0.0	862/919
Eukaryota	Fungi	Pyrenophora tritici-repentis Pt-1C-BFP	XP_001937877.1	0.0	819/919
Eukaryota	Fungi	Chaetomium globosum CBS 148.51	XP_001220335.1	0.0	898/919
Eukaryota	Fungi	Gibberella zeae PH-1	XP_385797.1	0.0	858/919
Eukaryota	Fungi	Nectria haematococca mpVI 77-13-4	EEU43005.1	0.0	858/919
Eukaryota	Fungi	Verticillium albo-atrum VaMs.102	EEY18938.1	0.0	825/919
Eukaryota	Fungi	Yarrowia lipolytica CLIB122	XP_500440.1	0.0	867/919
Eukaryota	Fungi	Pichia pastoris GS115	XP_002493132.1	0.0	792/919
Eukaryota	Fungi	Saccharomyces cerevisiae	AAU09739.1	1 E-176	788/919
Eukaryota	Fungi	Saccharomyces cerevisiae	NP_011893.1	1 E-176	788/919
Eukaryota	Fungi	Saccharomyces cerevisiae RM11-1a	EDV09080.1	1 E-176	788/919
Eukaryota	Fungi	Saccharomyces cerevisiae EC1118	CAY80312.1	1 E-175	788/919
Eukaryota	Fungi	Saccharomyces cerevisiae YJM789	EDN62265.1	1 E-175	788/919
Eukaryota	Fungi	Debaryomyces hansenii	CAG88320.2	1 E-173	855/919
Eukaryota	Fungi	Debaryomyces hansenii CBS767	XP_460062.1	1 E-173	855/919
Eukaryota	Fungi	Saccharomyces cerevisiae	CAA33512.1	1 E-173	778/919
Eukaryota	Fungi	Lachancea thermotolerans CBS 6340	XP_002555343.1	1 E-171	742/919
Eukaryota	Fungi	Vanderwaltozyma polyspora DSM 70294	XP_001644232.1	1 E-169	745/919
Eukaryota	Fungi	Candida glabrata CBS 138	XP_447737.1	1 E-167	792/919
Eukaryota	Fungi	Pichia stipitis CBS 6054	XP_001385880.2	1 E-166	752/919
Eukaryota	Fungi	Kluyveromyces lactis NRRL Y-1140	XP_454468.1	1 E-166	803/919
Eukaryota	Fungi	Ashbya gossypii ATCC 10895	NP_983475.1	1 E-164	757/919
Eukaryota	Fungi	Zygosaccharomyces rouxii CBS 732	XP_002498678.1	1 E-161	808/919
Eukaryota	Fungi	Candida albicans SC5314	XP_720628.1	1 E-155	802/919
Eukaryota	Fungi	Candida albicans SC5314	XP_720755.1	1 E-154	802/919
Eukaryota	Fungi	Candida albicans WO-1	EEQ46331.1	1 E-154	802/919
Eukaryota	Fungi	Lodderomyces elongisporus NRRL YB-4239	XP_001527713.1	1 E-149	738/919
Eukaryota	Fungi	Pichia pastoris GS115	XP_002491876.1	1 E-145	803/919
Eukaryota	Fungi	Postia placenta Mad-698-R	XP_002471010.1	1 E-140	865/919
Eukaryota	Fungi	Postia placenta Mad-698-R	XP_002475839.1	1 E-140	792/919
Eukaryota	Fungi	Coprinopsis cinerea okayama7#130	XP_001831130.1	1 E-134	757/919
Eukaryota	Fungi	Cryptococcus neoformans var. neoformans	XP_568605.1	1 E-130	767/919
Eukaryota	Fungi	Candida tropicalis MYA-3404	XP_002545795.1	1 E-120	882/919
Eukaryota	Fungi	Candida albicans WO-1	EEQ43630.1	1 E-119	845/919

Eukaryota	Fungi	Candida albicans SC5314	XP_710817.1	1 E-118	905/919
Eukaryota	Fungi	Candida dubliniensis CD36	XP_002422088.1	1 E-118	848/919
Eukaryota	Fungi	Ashbya gossypii ATCC 10895	NP_983505.1	1 E-117	792/919
Eukaryota	Fungi	Lodderomyces elongisporus NRRL YB-4239	XP_001525354.1	1 E-113	889/919
Eukaryota	Fungi	Candida glabrata CBS 138	XP_448886.1	1 E-108	804/919
Eukaryota	Fungi	Ustilago maydis 521	XP_761420.1	1 E-107	851/919
Eukaryota	Metazoa	Equus caballus	XP_001494049.1	5 E-93	763/919
Eukaryota	Metazoa	Gallus gallus	NP_001026426.1	3 E-92	754/919

AFUA_3G07860

Eukaryota	Fungi	Aspergillus fumigatus Af293	XP_754827.1	0.0	3014/3014
Eukaryota	Fungi	Neosartorya fischeri NRRL 181	XP_001263656.1	0.0	2860/3014
Eukaryota	Fungi	Aspergillus clavatus NRRL 1	XP_001270802.1	0.0	2857/3014
Eukaryota	Fungi	Aspergillus oryzae RIB40	XP_001818404.1	0.0	2825/3014
Eukaryota	Fungi	Aspergillus terreus NIH2624	XP_001209039.1	0.0	2805/3014
Eukaryota	Fungi	Aspergillus nidulans FGSC A4	XP_660559.1	0.0	2852/3014
Eukaryota	Fungi	Penicillium chrysogenum Wisconsin 54-1255	XP_002563137.1	0.0	2840/3014
Eukaryota	Fungi	Pyrenophora tritici-repentis Pt-1C-BFP	XP_001935073.1	0.0	2789/3014
Eukaryota	Fungi	Neosartorya fischeri NRRL 181	XP_001266638.1	0.0	2718/3014
Eukaryota	Fungi	Aspergillus fumigatus A1163	EDP50781.1	0.0	2718/3014
Eukaryota	Fungi	Aspergillus fumigatus Af293	XP_751425.1	0.0	2719/3014
Eukaryota	Fungi	Aspergillus flavus NRRL3357	XP_002384474.1	0.0	2738/3014
Eukaryota	Fungi	Aspergillus nidulans FGSC A4	XP_681638.1	0.0	2739/3014
Eukaryota	Fungi	Aspergillus niger CBS 513.88	XP_001396090.1	0.0	2714/3014
Eukaryota	Fungi	Aspergillus oryzae RIB40	XP_001827225.1	0.0	2665/3014
Eukaryota	Fungi	Nectria haematococca mpVI 77-13-4	EEU43691.1	0.0	2582/3014
Eukaryota	Fungi	Neurospora crassa OR74A	XP_956630.1	0.0	2521/3014

AFUA_3G08120

Eukaryota	Fungi	Aspergillus fumigatus Af293	XP_754802.1	0.0	533/533
Eukaryota	Fungi	Aspergillus clavatus NRRL 1	XP_001270831.1	1 E-118	530/533
Eukaryota	Fungi	Neosartorya fischeri NRRL 181	XP_001263628.1	1 E-101	482/533
Eukaryota	Fungi	Aspergillus niger CBS 513.88	XP_001400291.1	2 E-88	436/533

AFUA_3G09450

Eukaryota	Fungi	Aspergillus fumigatus Af293	XP_754674.2	1 E-169	292/292
Eukaryota	Fungi	Penicillium chrysogenum Wisconsin 54-1255	XP_002569210.1	1 E-116	287/292
Eukaryota	Fungi	Penicillium marneffei ATCC 18224	XP_002144318.1	1 E-109	281/292
Eukaryota	Fungi	Phaeosphaeria nodorum SN15	XP_001805882.1	7 E-83	273/292
Eukaryota	Fungi	Nectria haematococca mpVI 77-13-4	EEU42167.1	8 E-77	274/292
Eukaryota	Fungi	Pyrenophora tritici-repentis Pt-1C-BFP	XP_001933550.1	5 E-58	293/292
Bacteria	Actinobacteria	Micromonospora sp. ATCC 39149	ZP_04604430.1	8 E-21	249/292
Bacteria	Proteobacteria	Bordetella bronchiseptica RB50	NP_889027.1	2 E-19	255/292
Bacteria	Proteobacteria	alpha proteobacterium BAL199	ZP_02189092.1	2 E-19	252/292
Bacteria	Proteobacteria	Ralstonia eutropha H16	YP_724667.1	1 E-18	256/292
Bacteria	Proteobacteria	Cupriavidus taiwanensis	YP_001795485.1	2 E-18	256/292
Bacteria	Proteobacteria	Bordetella pertussis Tohama I	NP_880772.1	3 E-18	256/292
Bacteria	Proteobacteria	Bordetella parapertussis 12822	NP_883718.1	4 E-18	255/292
Bacteria	Actinobacteria	Mycobacterium gilvum PYR-GCK	YP_001132618.1	2 E-15	240/292
Bacteria	Proteobacteria	Ralstonia eutropha JMP134	YP_294341.1	2 E-15	262/292
Bacteria	Proteobacteria	Ralstonia solanacearum MolK2	YP_002256131.1	3 E-15	253/292
Bacteria	Proteobacteria	Ralstonia solanacearum UW551	ZP_00945534.1	3 E-15	253/292
Bacteria	Actinobacteria	Nocardiopsis dassonvillei subsp. dassonvillei	ZP_04335977.1	3 E-15	241/292
Bacteria	Proteobacteria	Burkholderia graminis C4D1M	ZP_02884229.1	2 E-14	255/292
Bacteria	Proteobacteria	Ralstonia pickettii 12J	YP_001897747.1	2 E-14	267/292

Bacteria	Proteobacteria	Nitrobacter sp. Nb-311A	ZP_01046573.1	2 E-14	237/292
Bacteria	Proteobacteria	Ralstonia metallidurans CH34	YP_582239.1	3 E-14	235/292
Bacteria	Firmicutes	Thermosinus carboxydivorans Nor1	ZP_01665252.1	7 E-14	253/292
Bacteria	Proteobacteria	Ralstonia pickettii 12D	YP_002980141.1	8 E-14	253/292
Bacteria	Proteobacteria	Nitrobacter hamburgensis X14	YP_577851.1	1 E-13	237/292
Bacteria	Proteobacteria	Burkholderia xenovorans LB400	YP_554657.1	5 E-13	255/292
Bacteria	Proteobacteria	Granulibacter bethesdensis CGDNIH1	YP_744976.1	5 E-13	276/292
Bacteria	Proteobacteria	Marinomonas sp. MWYL1	YP_001341895.1	7 E-13	239/292
Bacteria	Actinobacteria	Rhodococcus opacus B4	YP_002778246.1	9 E-13	236/292
Bacteria	Proteobacteria	Burkholderia phytofirmans PsJN	YP_001888062.1	1 E-12	255/292
Bacteria	Proteobacteria	Nitrobacter winogradskyi Nb-255	YP_318813.1	1 E-12	237/292
Bacteria	Actinobacteria	Stackebrandtia nassauensis DSM 44728	ZP_04485053.1	2 E-12	250/292
Bacteria	Firmicutes	Clostridium beijerinckii NCIMB 8052	YP_001310323.1	2 E-12	274/292
Bacteria	Actinobacteria	Rhodococcus opacus	AAC38246.1	3 E-12	235/292
Bacteria	Actinobacteria	Rhodococcus sp. DK17	ABD14383.1	3 E-12	236/292
Bacteria	Proteobacteria	Burkholderia sp. H160	ZP_03269292.1	4 E-12	257/292
Bacteria	Actinobacteria	Rhodococcus jostii RHA1	YP_701320.1	4 E-12	236/292
Bacteria	Proteobacteria	Pseudomonas syringae pv. oryzae	ZP_04587312.1	4 E-12	246/292
Bacteria	Proteobacteria	Burkholderia xenovorans LB400	YP_554705.1	4 E-12	236/292
Bacteria	Proteobacteria	Bradyrhizobium sp. BTAi1	YP_001241207.1	7 E-12	237/292
Bacteria	Bacteroidetes	Salinibacter ruber DSM 13855	YP_446779.1	2 E-11	258/292
Bacteria	Proteobacteria	Azorhizobium caulinodans ORS 571	YP_001525259.1	2 E-11	245/292
Bacteria	Proteobacteria	Ralstonia solanacearum GMI1000	NP_518419.1	2 E-11	253/292
Bacteria	Actinobacteria	Mycobacterium sp. MCS	YP_637580.1	2 E-11	255/292
Bacteria	Proteobacteria	Burkholderia cenocepacia AU 1054	YP_621110.1	4 E-11	256/292
Bacteria	Proteobacteria	Granulibacter bethesdensis CGDNIH1	YP_744346.1	4 E-11	253/292
Bacteria	Proteobacteria	Bradyrhizobium sp. ORS278	YP_001204901.1	5 E-11	237/292

AFUA_3G09460

Eukaryota	Fungi	Aspergillus fumigatus Af293	XP_754673.1	0.0	319/319
Eukaryota	Fungi	Penicillium chrysogenum Wisconsin 54-1255	XP_002569209.1	1 E-112	296/319
Eukaryota	Fungi	Nectria haematococca mpVI 77-13-4	EEU45734.1	3 E-59	307/319
Eukaryota	Fungi	Phaeosphaeria nodorum SN15	XP_001796106.1	1 E-56	310/319
Eukaryota	Fungi	Aspergillus terreus NIH2624	XP_001209302.1	4 E-56	310/319
Eukaryota	Fungi	Nectria haematococca mpVI 77-13-4	EEU37364.1	1 E-55	313/319
Eukaryota	Fungi	Nectria haematococca mpVI 77-13-4	EEU41999.1	7 E-55	283/319
Eukaryota	Fungi	Nectria haematococca mpVI 77-13-4	EEU45733.1	1 E-54	339/319
Eukaryota	Fungi	Uncinocarpus reesii 1704	XP_002542329.1	1 E-53	296/319
Eukaryota	Fungi	Podospora anserina DSM 980	XP_001903155.1	2 E-53	315/319
Eukaryota	Fungi	Penicillium marneffei ATCC 18224	XP_002149353.1	4 E-52	326/319
Eukaryota	Fungi	Coccidioides posadasii C735 delta	EER29483.1	9 E-52	296/319
Eukaryota	Fungi	Coccidioides immitis RS;	XP_001245207.1	1 E-51	296/319
Eukaryota	Fungi	Nectria haematococca mpVI 77-13-4	EEU41703.1	9 E-50	294/319
Eukaryota	Fungi	Coprinopsis cinerea okayama7#130	XP_001835719.1	2 E-45	283/319
Eukaryota	Fungi	Postia placenta Mad-698-R	XP_002471773.1	4 E-43	284/319
Eukaryota	Fungi	Coccidioides immitis RS;	XP_001248654.1	6 E-39	279/319
Eukaryota	Fungi	Coprinopsis cinerea okayama7#130	XP_001835718.1	2 E-38	282/319
Eukaryota	Fungi	Aspergillus nidulans FGSC A4	XP_681904.1	1 E-34	282/319
Eukaryota	Fungi	Postia placenta Mad-698-R	XP_002474071.1	3 E-28	285/319
Eukaryota	Fungi	Postia placenta Mad-698-R	XP_002474180.1	3 E-28	285/319
Eukaryota	Fungi	Penicillium marneffei ATCC 18224	XP_002144319.1	2 E-27	284/319
Eukaryota	Fungi	Penicillium marneffei ATCC 18224	XP_002149483.1	1 E-18	291/319
Eukaryota	Fungi	Nectria haematococca mpVI 77-13-4	EEU41996.1	5 E-18	275/319
Eukaryota	Fungi	Aspergillus nidulans FGSC A4	CBF90346.1	7 E-16	296/319
Eukaryota	Fungi	Talaromyces stipitatus ATCC 10500	XP_002488715.1	3 E-15	294/319

AFUA_3G11840

Eukaryota	Fungi	Aspergillus fumigatus Af293	XP_754437.1	0.0	575/575
Eukaryota	Fungi	Neosartorya fischeri NRRL 181	XP_001263281.1	0.0	556/575
Eukaryota	Fungi	Aspergillus oryzae RIB40	XP_001819890.1	0.0	565/575
Eukaryota	Fungi	Aspergillus clavatus NRRL 1	XP_001271183.1	0.0	570/575
Eukaryota	Fungi	Penicillium chrysogenum Wisconsin 54-1255	XP_002561985.1	0.0	533/575
Eukaryota	Fungi	Aspergillus niger CBS 513.88	XP_001399899.1	0.0	530/575
Eukaryota	Fungi	Ajellomyces dermatitidis ER-3	EEQ84009.1	0.0	528/575
Eukaryota	Fungi	Ajellomyces dermatitidis SLH14081	XP_002623399.1	0.0	527/575
Eukaryota	Fungi	Coccidioides posadasii C735 delta	EER26464.1	0.0	528/575
Eukaryota	Fungi	Penicillium marneffei ATCC 18224	XP_002143325.1	0.0	520/575
Eukaryota	Fungi	Coccidioides immitis RS;	XP_001244197.1	0.0	528/575
Eukaryota	Fungi	Talaromyces stipitatus ATCC 10500	XP_002479625.1	0.0	521/575
Eukaryota	Fungi	Ajellomyces capsulatus G186AR	EEH04777.1	0.0	534/575
Eukaryota	Fungi	Ajellomyces capsulatus H143	EER37116.1	0.0	536/575
Eukaryota	Fungi	Uncinocarpus reesii 1704	XP_002582630.1	0.0	519/575
Eukaryota	Fungi	Microsporum canis CBS 113480	EEQ27906.1	0.0	518/575
Eukaryota	Fungi	Paracoccidioides brasiliensis Pb18;	EEH44393.1	0.0	521/575
Eukaryota	Fungi	Paracoccidioides brasiliensis Pb01;	EEH39823.1	0.0	518/575
Eukaryota	Fungi	Paracoccidioides brasiliensis Pb03;	EEH20013.1	1 E-179	521/575
Eukaryota	Fungi	Aspergillus nidulans FGSC A4	XP_660470.1	1 E-174	558/575
Eukaryota	Fungi	Ajellomyces capsulatus NAm1	XP_001536485.1	1 E-171	526/575
Eukaryota	Fungi	Neurospora crassa OR74A	XP_963705.2	1 E-151	526/575
Eukaryota	Fungi	Chaetomium globosum CBS 148.51	XP_001229229.1	1 E-150	507/575
Eukaryota	Fungi	Podospora anserina DSM 980	XP_001905131.1	1 E-146	526/575
Eukaryota	Fungi	Sclerotinia sclerotiorum 1980 UF-70	XP_001596937.1	1 E-144	505/575
Eukaryota	Fungi	Nectria haematococca mpVI 77-13-4	EEU48657.1	1 E-143	490/575
Eukaryota	Fungi	Pyrenophora tritici-repentis Pt-1C-BFP	XP_001938643.1	1 E-142	502/575
Eukaryota	Fungi	Gibberella zeae PH-1	XP_380565.1	1 E-135	490/575
Eukaryota	Fungi	Magnaporthe grisea 70-15	XP_001404275.1	1 E-127	491/575
Eukaryota	Fungi	Gibberella zeae PH-1	XP_384718.1	1 E-119	504/575

AFUA_3G11850

Eukaryota	Fungi	Aspergillus fumigatus Af293	XP_754436.1	0.0	563/563
Eukaryota	Fungi	Neosartorya fischeri NRRL 181	XP_001263280.1	0.0	563/563
Eukaryota	Fungi	Aspergillus clavatus NRRL 1	XP_001271184.1	1 E-128	582/563
Eukaryota	Fungi	Uncinocarpus reesii 1704	XP_002582763.1	2 E-12	466/563

AFUA_3G14680

Eukaryota	Fungi	Aspergillus fumigatus Af293	XP_754157.1	0.0	630/630
Eukaryota	Fungi	Neosartorya fischeri NRRL 181	XP_001262993.1	0.0	630/630
Eukaryota	Fungi	Aspergillus clavatus NRRL 1	XP_001271420.1	0.0	612/630
Eukaryota	Fungi	Aspergillus oryzae RIB40	XP_001823316.1	0.0	604/630
Eukaryota	Fungi	Aspergillus niger CBS 513.88	XP_001393442.1	0.0	604/630
Eukaryota	Fungi	Aspergillus terreus NIH2624	XP_001214505.1	0.0	601/630
Eukaryota	Fungi	Aspergillus fumigatus Af293	XP_751952.1	0.0	586/630
Eukaryota	Fungi	Ajellomyces capsulatus G186AR	EEH02551.1	0.0	616/630
Eukaryota	Fungi	Neosartorya fischeri NRRL 181	XP_001267149.1	0.0	598/630
Eukaryota	Fungi	Ajellomyces capsulatus H143	EER45250.1	0.0	616/630
Eukaryota	Fungi	Aspergillus nidulans FGSC A4	XP_681061.1	0.0	589/630
Eukaryota	Fungi	Penicillium chrysogenum Wisconsin 54-1255	XP_002562131.1	0.0	600/630
Eukaryota	Fungi	Ajellomyces capsulatus NAm1	XP_001538591.1	0.0	616/630
Eukaryota	Fungi	Ajellomyces dermatitidis SLH14081	XP_002629279.1	0.0	620/630
Eukaryota	Fungi	Penicillium chrysogenum;	P39457.1	0.0	600/630
Eukaryota	Fungi	Emericella nidulans	BAD95522.1	0.0	589/630
Eukaryota	Fungi	Aspergillus clavatus NRRL 1	XP_001271763.1	0.0	608/630

Eukaryota	Fungi	Aspergillus flavus NRRL3357	XP_002375951.1	0.0	575/630
Eukaryota	Fungi	Aspergillus niger CBS 513.88	XP_001400453.1	0.0	613/630
Eukaryota	Fungi	Aspergillus niger CBS 513.88	XP_001401726.1	0.0	579/630
Eukaryota	Fungi	Aspergillus oryzae RIB40	XP_001727653.1	0.0	575/630
Eukaryota	Fungi	Aspergillus flavus NRRL3357	XP_002376965.1	0.0	598/630
Eukaryota	Fungi	Aspergillus oryzae RIB40	XP_001821203.1	0.0	598/630
Eukaryota	Fungi	Aspergillus nidulans FGSC A4	XP_659279.1	0.0	599/630
Eukaryota	Fungi	Paracoccidioides brasiliensis Pb03;	EEH18113.1	0.0	605/630
Eukaryota	Fungi	Paracoccidioides brasiliensis Pb01;	EEH41672.1	0.0	605/630
Eukaryota	Fungi	Paracoccidioides brasiliensis Pb18;	EEH47014.1	0.0	605/630
Eukaryota	Fungi	Uncinocarpus reesii 1704	XP_002542963.1	0.0	609/630
Eukaryota	Fungi	Coccidioides immitis RS;	XP_001241137.1	0.0	585/630
Eukaryota	Fungi	Coccidioides posadasii;	ABA12208.1	0.0	585/630
Eukaryota	Fungi	Coccidioides posadasii C735 delta	EER22941.1	0.0	585/630
Eukaryota	Fungi	Talaromyces stipitatus ATCC 10500	XP_002483683.1	0.0	607/630
Eukaryota	Fungi	Trichophyton tonsurans	ACL37324.1	0.0	606/630
Eukaryota	Fungi	Trichophyton rubrum	ABG67901.1	0.0	591/630
Eukaryota	Fungi	Microsporum canis CBS 113480	EEQ29833.1	0.0	612/630
Eukaryota	Fungi	Penicillium marneffei ATCC 18224	XP_002150506.1	0.0	602/630
Eukaryota	Fungi	Aspergillus fumigatus Af293	XP_748208.1	0.0	582/630
Eukaryota	Fungi	Sclerotinia sclerotiorum 1980 UF-70	XP_001594722.1	0.0	592/630
Eukaryota	Fungi	Talaromyces stipitatus ATCC 10500	XP_002483684.1	0.0	536/630
Eukaryota	Fungi	Phaeosphaeria nodorum SN15	XP_001796806.1	0.0	638/630
Eukaryota	Fungi	Neurospora crassa OR74A	XP_964190.2	0.0	588/630
Eukaryota	Fungi	Gibberella zeae PH-1	XP_384051.1	1 E-180	586/630
Eukaryota	Fungi	Gibberella zeae PH-1	XP_382249.1	1 E-179	608/630
Eukaryota	Fungi	Neurospora crassa	AAC03052.1	1 E-179	587/630
Eukaryota	Fungi	Magnaporthe grisea 70-15	XP_367362.1	1 E-178	621/630
Eukaryota	Fungi	Neurospora crassa	AAC03053.1	1 E-178	587/630
Eukaryota	Fungi	Nectria haematococca mpVI 77-13-4	EEU33926.1	1 E-178	593/630
Eukaryota	Fungi	Nectria haematococca mpVI 77-13-4	EEU45269.1	1 E-172	618/630
Eukaryota	Fungi	Nectria haematococca mpVI 77-13-4	EEU41951.1	1 E-168	578/630
Eukaryota	Fungi	Pyrenophora tritici-repentis Pt-1C-BFP	XP_001931817.1	1 E-167	548/630
Eukaryota	Fungi	Gibberella zeae PH-1	XP_390869.1	1 E-162	562/630
Eukaryota	Fungi	Aspergillus niger CBS 513.88	XP_001390678.1	1 E-161	595/630
Eukaryota	Fungi	Podospora anserina DSM 980	XP_001908815.1	1 E-149	586/630
Eukaryota	Fungi	Debaryomyces hansenii CBS767	XP_459527.1	1 E-148	556/630
Eukaryota	Fungi	Torulaspora delbrueckii	Q11121.1	1 E-148	559/630
Eukaryota	Fungi	Saccharomyces cerevisiae EC1118	CAY86279.1	1 E-148	563/630
Eukaryota	Fungi	Saccharomyces cerevisiae JAY291	EEU08907.1	1 E-148	563/630
Eukaryota	Fungi	Saccharomyces cerevisiae	NP_014632.1	1 E-148	563/630
Eukaryota	Fungi	Saccharomyces cerevisiae YJM789	EDN63860.1	1 E-148	563/630
Eukaryota	Fungi	Vanderwaltozyma polyspora DSM 70294	XP_001645274.1	1 E-146	575/630
Eukaryota	Fungi	Lachancea thermotolerans CBS 6340	XP_002552683.1	1 E-146	575/630
Eukaryota	Fungi	Kluyveromyces lactis NRRL Y-1140	XP_452462.1	1 E-146	561/630
Eukaryota	Fungi	Saccharomyces cerevisiae	NP_013721.1	1 E-146	587/630
Eukaryota	Fungi	Chaetomium globosum CBS 148.51	XP_001220104.1	1 E-146	546/630
Eukaryota	Fungi	Saccharomyces cerevisiae RM11-1a	EDV11512.1	1 E-146	587/630
Eukaryota	Fungi	Saccharomyces cerevisiae YJM789	EDN64399.1	1 E-146	587/630
Eukaryota	Fungi	Saccharomyces cerevisiae EC1118	CAY81830.1	1 E-146	587/630
Eukaryota	Fungi	Saccharomyces cerevisiae JAY291	EEU08383.1	1 E-146	587/630
Eukaryota	Fungi	Candida tropicalis MYA-3404	XP_002546411.1	1 E-146	597/630
Eukaryota	Fungi	Saccharomyces cerevisiae	AAT92807.1	1 E-145	587/630
Eukaryota	Fungi	Saccharomyces cerevisiae	AAA61611.1	1 E-145	587/630
Eukaryota	Fungi	Candida glabrata CBS 138	XP_448225.1	1 E-145	565/630
Eukaryota	Fungi	Pichia jadinii	BAC79383.1	1 E-144	543/630
Eukaryota	Fungi	Zygosaccharomyces rouxii CBS 732	XP_002495964.1	1 E-144	577/630
Eukaryota	Fungi	Clavispora lusitaniae ATCC 42720	XP_002618066.1	1 E-143	562/630

Eukaryota	Fungi	Lodderomyces elongisporus NRRL YB-4239	XP_001523506.1	1 E-143	560/630
Eukaryota	Fungi	Zygosaccharomyces rouxii	BAH70356.1	1 E-142	578/630
Eukaryota	Fungi	Pichia stipitis CBS 6054	XP_001383860.2	1 E-142	558/630
Eukaryota	Fungi	Candida glabrata	AAM16160.1	1 E-141	563/630
Eukaryota	Fungi	Candida albicans SC5314	XP_711295.1	1 E-141	556/630
Eukaryota	Fungi	Pichia pastoris GS115	XP_002493557.1	1 E-140	558/630
Eukaryota	Fungi	Yarrowia lipolytica CLIB122	XP_504006.1	1 E-139	563/630
Eukaryota	Fungi	Saccharomyces cerevisiae RM11-1a	EDV11510.1	1 E-136	560/630
Eukaryota	Fungi	Saccharomyces cerevisiae JAY291	EEU08421.1	1 E-136	560/630
Eukaryota	Fungi	Saccharomyces cerevisiae EC1118	CAY81828.1	1 E-136	560/630
Eukaryota	Fungi	Saccharomyces cerevisiae YJM789	EDN64398.1	1 E-136	560/630
Eukaryota	Fungi	Candida versatilis	BAH70499.1	1 E-135	556/630
Eukaryota	Fungi	Saccharomyces cerevisiae	NP_013719.1	1 E-135	560/630
Eukaryota	Fungi	Candida glabrata CBS 138	XP_445790.1	1 E-134	573/630
Eukaryota	Fungi	Pichia stipitis CBS 6054	XP_001382679.2	1 E-134	556/630
Eukaryota	Fungi	Vanderwaltozyma polyspora DSM 70294	XP_001646712.1	1 E-134	556/630
Eukaryota	Fungi	Clavispora lusitaniae ATCC 42720	XP_002619005.1	1 E-134	569/630
Eukaryota	Fungi	Candida dubliniensis CD36	XP_002422435.1	1 E-134	553/630
Eukaryota	Fungi	Candida tropicalis MYA-3404	XP_002548856.1	1 E-131	563/630
Eukaryota	Fungi	Pichia guilliermondii ATCC 6260	EDK37190.2	1 E-131	556/630
Eukaryota	Fungi	Pichia guilliermondii ATCC 6260	XP_001485617.1	1 E-130	556/630
Eukaryota	Fungi	Candida albicans	AAF08980.1	1 E-129	560/630
Eukaryota	Fungi	Candida albicans SC5314	XP_717565.1	1 E-129	560/630
Eukaryota	Fungi	Candida albicans	Q9UVX1.2	1 E-129	560/630
Eukaryota	Fungi	Candida albicans WO-1	EEQ42389.1	1 E-129	560/630
Eukaryota	Fungi	Candida albicans SC5314	XP_717639.1	1 E-129	560/630
Eukaryota	Fungi	Candida dubliniensis CD36	XP_002417423.1	1 E-129	560/630
Eukaryota	Fungi	Debaryomyces hansenii CBS767	XP_460544.1	1 E-128	553/630
Eukaryota	Fungi	Candida dubliniensis CD36	XP_002420975.1	1 E-127	547/630
Eukaryota	Fungi	Candida albicans WO-1	EEQ46802.1	1 E-126	548/630
Eukaryota	Fungi	Verticillium albo-atrum VaMs.102	EEY21067.1	1 E-126	564/630
Eukaryota	Fungi	Candida albicans SC5314	XP_713822.1	1 E-126	548/630
Eukaryota	Fungi	Candida albicans	AAC72296.1	1 E-126	548/630
Eukaryota	Fungi	Candida albicans SC5314	XP_713863.1	1 E-126	548/630
Eukaryota	Fungi	Schizosaccharomyces japonicus yFS275	XP_002173310.1	1 E-124	571/630
Eukaryota	Fungi	Candida glabrata CBS 138	XP_448224.1	1 E-124	565/630
Eukaryota	Fungi	Candida glabrata	AAM19335.1	1 E-124	565/630
Eukaryota	Fungi	Pichia stipitis CBS 6054	XP_001386013.2	1 E-124	556/630
Eukaryota	Fungi	Candida albicans WO-1	EEQ46801.1	1 E-123	546/630
Eukaryota	Fungi	Lodderomyces elongisporus NRRL YB-4239	XP_001526480.1	1 E-122	557/630
Eukaryota	Fungi	Saccharomyces cerevisiae AWRI1631	EDZ70212.1	1 E-121	523/630
Eukaryota	Fungi	Lodderomyces elongisporus NRRL YB-4239	XP_001527860.1	1 E-121	555/630
Eukaryota	Fungi	Candida tropicalis MYA-3404	XP_002548597.1	1 E-121	562/630
Eukaryota	Fungi	Candida albicans SC5314	XP_713865.1	1 E-121	546/630
Eukaryota	Fungi	Schizosaccharomyces pombe	NP_592772.1	1 E-121	568/630
Eukaryota	Fungi	Candida albicans	O93795.1	1 E-120	545/630
Eukaryota	Fungi	Candida dubliniensis CD36	XP_002420976.1	1 E-120	546/630
Eukaryota	Fungi	Debaryomyces hansenii	CAG90378.2	1 E-120	569/630
Eukaryota	Fungi	Debaryomyces hansenii CBS767	XP_461915.1	1 E-119	569/630
Eukaryota	Fungi	Candida tropicalis MYA-3404	XP_002548600.1	1 E-117	543/630
Eukaryota	Fungi	Malassezia pachydermatis	BAH09386.1	1 E-113	565/630
Eukaryota	Fungi	Pichia guilliermondii ATCC 6260	EDK38597.2	1 E-113	581/630
Eukaryota	Fungi	Pichia stipitis CBS 6054	XP_001382291.2	1 E-113	628/630
Eukaryota	Fungi	Pichia guilliermondii ATCC 6260	XP_001484966.1	1 E-113	581/630
Eukaryota	Fungi	Candida versatilis	BAH70500.1	1 E-112	553/630
Eukaryota	Fungi	Schizosaccharomyces pombe	NP_593194.1	1 E-112	562/630
Eukaryota	Fungi	Schizosaccharomyces pombe	O13857.2	1 E-112	562/630
Eukaryota	Fungi	Candida tropicalis MYA-3404	XP_002546924.1	1 E-111	594/630

Eukaryota	Fungi	Schizosaccharomyces pombe	NP_588308.1	1 E-110	559/630
Eukaryota	Fungi	Schizosaccharomyces pombe	Q9Y7N6.3	1 E-110	559/630
Eukaryota	Fungi	Malassezia furfur	BAG16390.1	1 E-109	565/630
Eukaryota	Fungi	Ustilago maydis 521	XP_757182.1	1 E-107	577/630
Eukaryota	Fungi	Malassezia globosa CBS 7966	XP_001730207.1	1 E-107	565/630
Eukaryota	Fungi	Candida dubliniensis CD36	XP_002418104.1	1 E-105	587/630
Eukaryota	Fungi	Clavispora lusitaniae ATCC 42720	XP_002618756.1	1 E-100	563/630
Eukaryota	Fungi	Yarrowia lipolytica CLIB122	XP_502563.1	3 E-99	569/630
Eukaryota	Fungi	Candida albicans WO-1	EEQ45582.1	7 E-97	602/630
Eukaryota	Fungi	Schizosaccharomyces pombe	NP_594024.1	4 E-96	616/630
Eukaryota	Fungi	Schizosaccharomyces japonicus yFS275	XP_002174365.1	2 E-94	597/630
Eukaryota	Fungi	Schizosaccharomyces japonicus yFS275	XP_002173309.1	2 E-91	559/630
Eukaryota	Fungi	Candida tropicalis MYA-3404	XP_002547327.1	3 E-90	530/630
Eukaryota	Fungi	Filobasidiella neoformans	ACB41996.1	4 E-90	564/630
Eukaryota	Fungi	Filobasidiella neoformans	ACB41992.1	5 E-90	608/630
Eukaryota	Fungi	Filobasidiella neoformans	ACB41987.1	6 E-90	564/630
Eukaryota	Fungi	Aspergillus niger CBS 513.88	XP_001398573.1	7 E-90	586/630
Eukaryota	Fungi	Filobasidiella neoformans	ACB41981.1	1 E-89	564/630
Eukaryota	Fungi	Filobasidiella neoformans	ACB41991.1	1 E-89	541/630
Eukaryota	Fungi	Filobasidiella neoformans	ACB41984.1	8 E-89	564/630
Eukaryota	Fungi	Cryptococcus neoformans var. neoformans	AAF61964.1	9 E-89	571/630
Eukaryota	Fungi	Cryptococcus neoformans var. neoformans	ACB42004.1	1 E-88	571/630
Eukaryota	Fungi	Cryptococcus neoformans var. grubii	Q9P8P2.1	1 E-88	567/630
Eukaryota	Fungi	Cryptococcus bacillisporus	CAB90748.2	2 E-88	565/630
Eukaryota	Fungi	Filobasidiella neoformans	ACB42002.1	2 E-88	571/630
Eukaryota	Fungi	Cryptococcus bacillisporus	ACB42015.1	2 E-88	565/630
Eukaryota	Fungi	Cryptococcus bacillisporus	ACB42025.1	3 E-88	566/630
Eukaryota	Fungi	Cryptococcus bacillisporus	CAC83081.1	3 E-88	565/630
Eukaryota	Fungi	Filobasidiella neoformans	ACB42001.1	4 E-88	565/630
Eukaryota	Fungi	Cryptococcus bacillisporus	ACB42022.1	5 E-88	565/630
Eukaryota	Fungi	Cryptococcus bacillisporus	ACB42048.1	7 E-88	565/630
Eukaryota	Fungi	Cryptococcus bacillisporus	ACB42019.1	1 E-87	565/630
Eukaryota	Fungi	Cryptococcus bacillisporus	ACB42024.1	2 E-87	565/630
Eukaryota	Fungi	Cryptococcus bacillisporus	ACB42035.1	2 E-87	565/630
Eukaryota	Fungi	Cryptococcus bacillisporus	ACB42026.1	2 E-87	565/630
Eukaryota	Fungi	Cryptococcus neoformans var. neoformans	XP_568399.1	4 E-87	574/630
Eukaryota	Fungi	Cryptococcus bacillisporus	ACB42039.1	6 E-87	565/630
Eukaryota	Fungi	Cryptococcus bacillisporus	ACB42038.1	8 E-87	565/630
Eukaryota	Fungi	Cryptococcus bacillisporus	ACB42057.1	1 E-86	565/630
Eukaryota	Fungi	Cryptococcus bacillisporus	ACB42044.1	1 E-86	565/630
Eukaryota	Fungi	Sclerotinia sclerotiorum 1980 UF-70	XP_001594223.1	2 E-86	516/630
Eukaryota	Fungi	Cryptococcus bacillisporus	ACB42055.1	2 E-86	565/630
Eukaryota	Fungi	Schizosaccharomyces pombe	NP_593196.1	3 E-86	601/630
Eukaryota	Fungi	Botryotinia fuckeliana B05.10	XP_001548519.1	4 E-86	533/630
Eukaryota	Fungi	Yarrowia lipolytica CLIB122	XP_002142997.1	3 E-84	530/630
Eukaryota	Fungi	Gibberella zeae PH-1	XP_388326.1	3 E-78	547/630
Eukaryota	Fungi	Yarrowia lipolytica CLIB122	XP_505398.1	1 E-76	522/630
Eukaryota	Fungi	Clavispora lusitaniae ATCC 42720	XP_002614360.1	7 E-76	611/630
Eukaryota	Fungi	Yarrowia lipolytica CLIB122	XP_504269.1	1 E-72	533/630
Eukaryota	Fungi	Postia placenta Mad-698-R	XP_002471132.1	6 E-71	626/630
Eukaryota	Fungi	Coprinopsis cinerea okayama7#130	XP_001830555.1	3 E-69	540/630
Eukaryota	Fungi	Nectria haematococca mpVI 77-13-4	EEU37192.1	2 E-67	574/630
Eukaryota	Fungi	Gibberella zeae PH-1	XP_382999.1	1 E-66	589/630
Eukaryota	Fungi	Candida tropicalis MYA-3404	XP_002548382.1	4 E-61	582/630

AFUA_3G14690

Eukaryota	Fungi	Aspergillus fumigatus Af293	XP_754156.1	0.0	470/470

Eukaryota	Fungi	Neosartorya fischeri NRRL 181	XP_001262992.1	0.0	467/470
Eukaryota	Fungi	Aspergillus clavatus NRRL 1	XP_001275050.1	0.0	469/470
Eukaryota	Fungi	Nectria haematococca mpVI 77-13-4	EEU36833.1	1 E-143	458/470
Eukaryota	Fungi	Aspergillus niger CBS 513.88	XP_001394542.1	1 E-140	448/470
Eukaryota	Fungi	Botryotinia fuckeliana B05.10	XP_001546556.1	1 E-119	426/470
Eukaryota	Fungi	Aspergillus terreus NIH2624	XP_001216790.1	1 E-118	435/470
Eukaryota	Fungi	Aspergillus clavatus NRRL 1	XP_001269932.1	1 E-117	444/470
Eukaryota	Fungi	Verticillium albo-atrum VaMs.102	EEY16854.1	1 E-112	482/470
Eukaryota	Fungi	Aspergillus niger CBS 513.88	XP_001395181.1	4 E-90	408/470
Eukaryota	Fungi	Aspergillus flavus NRRL3357	XP_002380650.1	2 E-84	413/470
Eukaryota	Fungi	Neosartorya fischeri NRRL 181	XP_001257570.1	7 E-82	388/470
Eukaryota	Fungi	Aspergillus fumigatus A1163	EDP47619.1	4 E-81	388/470
Eukaryota	Fungi	Phaeosphaeria nodorum SN15	XP_001801325.1	9 E-81	401/470
Eukaryota	Fungi	Aspergillus niger CBS 513.88	XP_001401479.1	2 E-80	411/470
Eukaryota	Fungi	Aspergillus fumigatus Af293	XP_747713.1	4 E-80	388/470
Eukaryota	Fungi	Penicillium chrysogenum Wisconsin 54-1255	XP_002559674.1	6 E-80	406/470
Eukaryota	Fungi	Aspergillus terreus NIH2624	XP_001209173.1	2 E-79	393/470
Eukaryota	Fungi	Aspergillus clavatus NRRL 1	XP_001270182.1	6 E-79	398/470
Eukaryota	Fungi	Aspergillus nidulans FGSC A4	CBF71163.1	2 E-78	405/470
Eukaryota	Fungi	Aspergillus flavus NRRL3357	XP_002383037.1	3 E-77	397/470
Eukaryota	Fungi	Aspergillus oryzae RIB40	XP_001817022.1	4 E-77	397/470
Eukaryota	Fungi	Aspergillus niger CBS 513.88	XP_001396737.1	2 E-76	401/470
Eukaryota	Fungi	Paracoccidioides brasiliensis Pb03;	EEH17388.1	3 E-76	380/470
Eukaryota	Fungi	Paracoccidioides brasiliensis Pb01;	EEH38428.1	3 E-76	380/470
Eukaryota	Fungi	Paracoccidioides brasiliensis Pb18;	EEH43635.1	8 E-76	380/470
Eukaryota	Fungi	Aspergillus terreus NIH2624	XP_001209789.1	3 E-75	393/470
Eukaryota	Fungi	Laccaria bicolor S238N-H82	XP_001878539.1	2 E-72	387/470
Eukaryota	Fungi	Penicillium chrysogenum Wisconsin 54-1255	XP_002563821.1	1 E-70	407/470
Eukaryota	Fungi	Microsporum canis CBS 113480	EEQ27646.1	3 E-70	381/470
Eukaryota	Fungi	Postia placenta Mad-698-R	XP_002473209.1	8 E-70	403/470
Eukaryota	Fungi	Postia placenta Mad-698-R	XP_002473362.1	1 E-68	407/470
Eukaryota	Fungi	Pyrenophora tritici-repentis Pt-1C-BFP	XP_001930482.1	1 E-67	391/470
Eukaryota	Fungi	Penicillium marneffei ATCC 18224	XP_002143160.1	3 E-67	410/470
Eukaryota	Fungi	Talaromyces stipitatus ATCC 10500	XP_002479457.1	1 E-65	400/470
Eukaryota	Fungi	Nectria haematococca mpVI 77-13-4	EEU41173.1	4 E-62	387/470
Eukaryota	Fungi	Coprinopsis cinerea okayama7#130	XP_001836705.1	1 E-58	419/470
Eukaryota	Fungi	Podospora anserina DSM 980	XP_001903515.1	4 E-58	393/470
Eukaryota	Fungi	Ustilago maydis 521	XP_757344.1	7 E-57	407/470
Eukaryota	Fungi	Yarrowia lipolytica CLIB122	XP_504066.1	1 E-53	384/470
Eukaryota	Fungi	Chaetomium globosum CBS 148.51	XP_001219505.1	2 E-52	411/470
Eukaryota	Fungi	Sclerotinia sclerotiorum 1980 UF-70	XP_001590700.1	1 E-50	437/470
Eukaryota	Fungi	Gibberella zeae PH-1	XP_389225.1	1 E-50	381/470
Bacteria	Bacteroidetes	Dokdonia donghaensis MED134	ZP_01049451.1	1 E-47	384/470
Bacteria	Bacteroidetes	Gramella forsetii KT0803	YP_863566.1	2 E-44	380/470
Eukaryota	Fungi	Malassezia globosa CBS 7966	XP_001729177.1	3 E-44	386/470
Eukaryota	Fungi	Botryotinia fuckeliana B05.10	XP_001558100.1	1 E-39	405/470
Bacteria	Bacteroidetes	Leeuwenhoekiella blandensis MED217	ZP_01062148.1	1 E-39	381/470
Bacteria	Proteobacteria	Rhodopseudomonas palustris BisB5	YP_571358.1	2 E-38	386/470
Eukaryota	stramenopiles	Phaeodactylum tricornutum CCAP 1055/1	XP_002180694.1	9 E-34	394/470
Bacteria	Proteobacteria	Ruegeria pomeroyi DSS-3	YP_165023.1	4 E-33	396/470
Bacteria	Thermotogae	Kosmotoga olearia TBF 19.5.1	YP_002941807.1	7 E-33	390/470
Bacteria	Firmicutes	Alicyclobacillus acidocaldarius subsp. acidocalc	YP_003186249.1	7 E-32	379/470
Bacteria	Proteobacteria	Sideroxydans lithotrophicus ES-1	ZP_05339299.1	1 E-30	383/470
Bacteria	Proteobacteria	Alkalilimnicola ehrlichii MLHE-1	YP_743265.1	1 E-30	384/470
Bacteria	Bacteroidetes	Rhodothermus marinus DSM 4252	ZP_04422695.1	7 E-30	390/470
Bacteria	Proteobacteria	Neisseria flavescens NRL30031/H210	ZP_03718946.1	1 E-29	377/470
Bacteria	Proteobacteria	Haemophilus influenzae 7P49H1	ZP_04467236.1	1 E-29	377/470
Bacteria	Proteobacteria	Photobacterium profundum	Q6LPR3.2	1 E-29	385/470

Bacteria	Proteobacteria	Photobacterium profundum SS9	YP_130515.1	2 E-29	385/470
Bacteria	Proteobacteria	Neisseria meningitidis Z2491	YP_002343276.1	2 E-29	377/470
Bacteria	Proteobacteria	Neisseria meningitidis alpha14	YP_003083721.1	2 E-29	377/470
Bacteria	Proteobacteria	Neisseria meningitidis alpha275	CBA06229.1	3 E-29	377/470
Bacteria	Bacteroidetes	Parabacteroides merdae ATCC 43184	ZP_02034070.1	3 E-29	379/470
Bacteria	Proteobacteria	Haemophilus influenzae 22.1-21	ZP_01785522.1	5 E-29	377/470
Bacteria	Proteobacteria	Haemophilus influenzae PittAA	ZP_01790587.1	5 E-29	377/470

AFUA_3G15300

Eukaryota	Fungi	Aspergillus fumigatus Af293	XP_754094.1	1 E-125	224/224
Eukaryota	Fungi	Neosartorya fischeri NRRL 181	XP_001262915.1	5 E-98	262/224
Eukaryota	Fungi	Penicillium chrysogenum Wisconsin 54-1255	XP_002566379.1	5 E-30	185/224
Eukaryota	Fungi	Aspergillus fumigatus A1163	EDP53743.1	6 E-27	199/224
Eukaryota	Fungi	Aspergillus fumigatus Af293	XP_749215.1	7 E-27	199/224
Eukaryota	Fungi	Penicillium chrysogenum Wisconsin 54-1255	XP_002567192.1	8 E-27	188/224
Eukaryota	Fungi	Neosartorya fischeri NRRL 181	XP_001265637.1	5 E-26	198/224
Eukaryota	Fungi	Aspergillus clavatus NRRL 1	XP_001273145.1	3 E-24	184/224
Eukaryota	Fungi	Penicillium marneffei ATCC 18224	XP_002150940.1	3 E-22	182/224
Eukaryota	Fungi	Aspergillus niger CBS 513.88	XP_001391280.1	3 E-22	193/224
Eukaryota	Fungi	Talaromyces stipitatus ATCC 10500	XP_002483142.1	1 E-20	206/224
Eukaryota	Fungi	Phaeosphaeria nodorum SN15	XP_001799236.1	2 E-17	184/224
Eukaryota	Fungi	Microsporum canis CBS 113480	EEQ30206.1	2 E-16	189/224
Eukaryota	Fungi	Paracoccidioides brasiliensis Pb03;	EEH21090.1	2 E-15	222/224
Eukaryota	Fungi	Sclerotinia sclerotiorum 1980 UF-70	XP_001585435.1	3 E-15	195/224
Eukaryota	Fungi	Ajellomyces dermatitidis SLH14081	XP_002623798.1	5 E-15	194/224
Eukaryota	Fungi	Ajellomyces dermatitidis ER-3	EEQ90373.1	5 E-15	194/224
Eukaryota	Fungi	Talaromyces stipitatus ATCC 10500	XP_002486165.1	1 E-14	185/224
Eukaryota	Fungi	Penicillium marneffei ATCC 18224	XP_002152540.1	2 E-13	187/224
Eukaryota	Fungi	Paracoccidioides brasiliensis Pb01;	EEH36450.1	5 E-12	188/224
Eukaryota	Fungi	Cryptococcus neoformans var. neoformans	XP_772683.1	2 E-11	210/224
Eukaryota	Fungi	Paracoccidioides brasiliensis Pb18;	EEH48243.1	2 E-11	188/224
Eukaryota	Fungi	Aspergillus clavatus NRRL 1	XP_001268183.1	2 E-11	204/224
Eukaryota	Fungi	Paracoccidioides brasiliensis Pb03;	EEH18823.1	2 E-11	188/224
Eukaryota	Fungi	Ajellomyces dermatitidis SLH14081	XP_002625982.1	5 E-11	187/224
Eukaryota	Fungi	Penicillium chrysogenum Wisconsin 54-1255	XP_002569053.1	5 E-11	186/224
Eukaryota	Fungi	Ajellomyces dermatitidis ER-3	EEQ86751.1	7 E-11	187/224

AFUA_3G15350

Eukaryota	Fungi	Aspergillus fumigatus Af293	XP_754090.1	1 E-139	243/243
Eukaryota	Fungi	Neosartorya fischeri NRRL 181	XP_001262911.1	1 E-137	243/243
Eukaryota	Fungi	Nectria haematococca mpVI 77-13-4	EEU41807.1	5 E-96	242/243
Eukaryota	Fungi	Gibberella zeae PH-1	XP_383644.1	3 E-80	239/243
Eukaryota	Fungi	Aspergillus nidulans FGSC A4	XP_659973.1	4 E-73	243/243
Eukaryota	Fungi	Aspergillus flavus NRRL3357	XP_002372369.1	3 E-60	235/243
Eukaryota	Fungi	Aspergillus oryzae RIB40	XP_001817310.1	7 E-60	237/243
Eukaryota	Fungi	Penicillium marneffei ATCC 18224	XP_002143939.1	1 E-57	250/243
Eukaryota	Fungi	Talaromyces stipitatus ATCC 10500	XP_002480320.1	1 E-56	249/243
Eukaryota	Fungi	Talaromyces stipitatus ATCC 10500	XP_002481505.1	1 E-48	241/243
Eukaryota	Fungi	Penicillium marneffei ATCC 18224	XP_002147372.1	4 E-48	246/243
Bacteria	Actinobacteria	Frankia alni ACN14a	YP_710447.1	8 E-42	242/243
Eukaryota	Fungi	Nectria haematococca mpVI 77-13-4	EEU48604.1	3 E-40	239/243
Bacteria	Proteobacteria	Zymomonas mobilis subsp. mobilis	YP_161904.1	4 E-38	240/243
Bacteria	Proteobacteria	Burkholderia xenovorans LB400	YP_556046.1	1 E-37	244/243
Eukaryota	Fungi	Podospora anserina DSM 980	XP_001912992.1	1 E-37	249/243
Bacteria	Actinobacteria	Frankia sp. CcI3	YP_479269.1	2 E-36	240/243
Bacteria	Proteobacteria	Rhizobium leguminosarum bv. trifolii	YP_002974507.1	2 E-36	240/243

Bacteria	Actinobacteria	Kribbella flavida DSM 17836	ZP_03862277.1	7 E-36	241/243
Bacteria	Proteobacteria	Agrobacterium tumefaciens str. C58	NP_396088.2	9 E-36	229/243
Bacteria	Proteobacteria	Agrobacterium tumefaciens str. C58	AAD44003.1	1 E-35	229/243
Eukaryota	Fungi	Gibberella zeae PH-1	XP_383967.1	1 E-34	248/243
Bacteria	Actinobacteria	Kytococcus sedentarius DSM 20547	YP_003148472.1	2 E-34	237/243
Bacteria	Actinobacteria	Clavibacter michiganensis subsp. sepedonicus	YP_001711561.1	3 E-34	240/243
Bacteria	Proteobacteria	Burkholderia sp. H160	ZP_03268997.1	3 E-34	248/243
Bacteria	Proteobacteria	Burkholderia xenovorans LB400	YP_558336.1	4 E-34	240/243
Bacteria	Proteobacteria	Burkholderia ambifaria MEX-5	ZP_02908044.1	6 E-34	248/243
Bacteria	Proteobacteria	Xanthobacter autotrophicus Py2	YP_001415962.1	7 E-34	236/243
Bacteria	Firmicutes	Lactobacillus casei ATCC 334	YP_806025.1	1 E-33	240/243
Bacteria	Firmicutes	Lactobacillus paracasei subsp. paracasei	ZP_03964493.1	1 E-33	240/243
Bacteria	Proteobacteria	Burkholderia phytofirmans PsJN	YP_001890030.1	1 E-33	249/243
Bacteria	Firmicutes	Listeria grayi DSM 20601	ZP_04445220.1	1 E-33	250/243
Bacteria	Actinobacteria	Streptomyces sp. SPB78	ZP_05489236.1	2 E-33	231/243
Bacteria	Bacteroidetes	Pedobacter sp. BAL39	ZP_01886309.1	3 E-33	240/243
Bacteria	Proteobacteria	Xanthomonas axonopodis pv. citri	NP_641818.1	4 E-33	238/243
Bacteria	Proteobacteria	Xanthomonas campestris pv. vesicatoria	YP_363273.1	4 E-33	238/243
Bacteria	Actinobacteria	Nocardiopsis dassonvillei subsp. dassonvillei	ZP_04333275.1	4 E-33	231/243
Bacteria	Firmicutes	Lactobacillus paracasei subsp. paracasei	ZP_04672709.1	4 E-33	240/243
Bacteria	Acidobacteria	Candidatus Solibacter usitatus Ellin6076	YP_826006.1	2 E-32	242/243
Bacteria	Proteobacteria	Stenotrophomonas maltophilia K279a	YP_001971656.1	2 E-32	238/243
Bacteria	Bacteroidetes	Leeuwenhoekiella blandensis MED217	ZP_01061402.1	4 E-32	243/243
Bacteria	Actinobacteria	Kribbella flavida DSM 17836	ZP_03863529.1	4 E-32	226/243
Bacteria	Firmicutes	Lactobacillus buchneri ATCC 11577	ZP_03942814.1	6 E-32	249/243
Bacteria	Bacteroidetes	Chitinophaga pinensis DSM 2588	YP_003124260.1	7 E-32	241/243
Bacteria	Proteobacteria	Idiomarina baltica OS145	ZP_01042022.1	7 E-32	239/243
Bacteria	Cyanobacteria	Microcoleus chthonoplastes PCC 7420	ZP_05024776.1	1 E-31	240/243
Bacteria	Bacteroidetes	Flavobacteriaceae bacterium 3519-10	YP_003095586.1	1 E-31	242/243
Bacteria	Actinobacteria	Streptomyces ambofaciens ATCC 23877	CAI78063.1	1 E-31	237/243
Bacteria	Actinobacteria	Streptomyces ambofaciens	CAK50996.1	1 E-31	237/243
Bacteria	Proteobacteria	Vibrio shilonii AK1	ZP_01870643.1	1 E-31	245/243
Bacteria	Proteobacteria	Xanthomonas campestris pv. campestris	NP_636814.1	1 E-31	241/243
Bacteria	Proteobacteria	Stenotrophomonas sp. SKA14	ZP_05134238.1	2 E-31	238/243
Bacteria	Firmicutes	Lactobacillus brevis subsp. gravesensis	ZP_03939878.1	3 E-31	249/243
Bacteria	Firmicutes	Lactobacillus hilgardii ATCC 8290	ZP_03952938.1	4 E-31	249/243
Bacteria	Actinobacteria	Nocardia farcinica IFM 10152	YP_116710.1	6 E-31	248/243
Bacteria	Acidobacteria	Acidobacterium capsulatum ATCC 51196	YP_002754373.1	9 E-31	238/243
Bacteria	Proteobacteria	Stenotrophomonas maltophilia R551-3	YP_002027957.1	1 E-30	238/243
Eukaryota	Fungi	Pyrenophora tritici-repentis Pt-1C-BFP	XP_001940982.1	1 E-30	203/243
Bacteria	Actinobacteria	Beutenbergia cavernae DSM 12333	YP_002880541.1	1 E-30	242/243
Bacteria	Actinobacteria	Streptomyces avermitilis MA-4680	NP_822086.1	1 E-30	246/243
Bacteria	Proteobacteria	Oxalobacter formigenes HOxBLS	ZP_04576576.1	2 E-30	237/243
Bacteria	Firmicutes	Paenibacillus sp. JDR-2	YP_003013001.1	2 E-30	242/243
Bacteria	Actinobacteria	Kribbella flavida DSM 17836	ZP_03865279.1	3 E-30	231/243
Bacteria	Proteobacteria	Caulobacter sp. K31	YP_001684494.1	3 E-30	250/243
Bacteria	Bacteroidetes	Chitinophaga pinensis DSM 2588	YP_003125289.1	3 E-30	244/243
Bacteria	Proteobacteria	Ralstonia eutropha H16	YP_841027.1	4 E-30	234/243
Bacteria	Proteobacteria	Aromatoleum aromaticum EbN1	YP_159365.1	1 E-29	234/243
Bacteria	Actinobacteria	Streptomyces sp. AA4	ZP_05480251.1	1 E-29	232/243
Eukaryota	Fungi	Neosartorya fischeri NRRL 181	XP_001262718.1	3 E-29	207/243
Bacteria	Actinobacteria	Streptomyces sp. SPB78	ZP_05486732.1	4 E-29	226/243
Bacteria	Actinobacteria	Streptomyces tubercidicus	AAT45284.1	5 E-29	232/243
Eukaryota	Fungi	Aspergillus niger CBS 513.88	XP_001401148.1	5 E-29	221/243
Bacteria	Spirochaetes	Leptospira biflexa serovar Patoc	YP_001837630.1	5 E-29	248/243
Eukaryota	Fungi	Aspergillus niger CBS 513.88	XP_001401752.1	5 E-29	229/243
Eukaryota	Fungi	Nectria haematococca mpVI 77-13-4	EEU38522.1	8 E-29	250/243
Bacteria	Actinobacteria	Actinosynnema mirum DSM 43827	YP_003102795.1	1 E-28	238/243

Eukaryota	Fungi	Aspergillus niger CBS 513.88	XP_001397550.1	1 E-28	230/243
Bacteria	Actinobacteria	Catenulispora acidiphila DSM 44928	YP_003116601.1	1 E-28	236/243
Bacteria	Actinobacteria	Streptomyces sviceus ATCC 29083	ZP_05019637.1	1 E-28	231/243
Bacteria	Actinobacteria	Frankia alni ACN14a	YP_714629.1	1 E-28	243/243
Bacteria	Bacteroidetes	Pedobacter sp. BAL39	ZP_01886518.1	2 E-28	244/243
Bacteria	Cyanobacteria	Cyanothece sp. PCC 7425	YP_002481105.1	2 E-28	233/243
Eukaryota	Fungi	Botryotinia fuckeliana B05.10	XP_001545304.1	4 E-28	239/243
Bacteria	Cyanobacteria	Synechococcus sp. PCC 7335	ZP_05039392.1	4 E-28	232/243
Eukaryota	Fungi	Aspergillus clavatus NRRL 1	XP_001274647.1	5 E-28	239/243
Eukaryota	Fungi	Verticillium albo-atrum VaMs.102	EEY19422.1	6 E-28	251/243
Bacteria	Actinobacteria	Streptomyces avermitilis MA-4680	NP_822455.1	8 E-28	232/243
Bacteria	Proteobacteria	Mesorhizobium loti MAFF303099	NP_103600.1	1 E-27	240/243
Eukaryota	Fungi	Moniliophthora perniciosa FA553	XP_002389689.1	1 E-27	229/243
Bacteria	Actinobacteria	Actinosynnema mirum DSM 43827	YP_003098662.1	1 E-27	244/243
Bacteria	Actinobacteria	Catenulispora acidiphila DSM 44928	YP_003115460.1	1 E-27	226/243
Eukaryota	Fungi	Verticillium albo-atrum VaMs.102	EEY23709.1	1 E-27	266/243
Eukaryota	Fungi	Phaeosphaeria nodorum SN15	XP_001792842.1	1 E-27	255/243
Bacteria	Actinobacteria	Streptomyces sp. Mg1	ZP_04997147.1	1 E-27	242/243
Bacteria	Firmicutes	Staphylococcus aureus A9635	ZP_05687757.1	2 E-27	231/243
Eukaryota	Fungi	Aspergillus niger CBS 513.88	XP_001389240.1	2 E-27	228/243
Bacteria	Actinobacteria	Streptomyces sp. AA4	ZP_05479058.1	3 E-27	245/243
Bacteria	Proteobacteria	Gluconacetobacter diazotrophicus PAl 5	YP_001602523.1	3 E-27	245/243
Bacteria	Firmicutes	Staphylococcus aureus subsp. aureus	NP_373103.1	3 E-27	231/243
Bacteria	Firmicutes	Staphylococcus aureus subsp. aureus	YP_001247953.1	3 E-27	231/243
Bacteria	Actinobacteria	Streptomyces sviceus ATCC 29083	ZP_05021431.1	4 E-27	239/243
Bacteria	Actinobacteria	Stackebrandtia nassauensis DSM 44728	ZP_04483890.1	4 E-27	251/243
Bacteria	Firmicutes	Staphylococcus aureus RF122	YP_417902.1	6 E-27	231/243
Bacteria	Actinobacteria	Streptomyces flavogriseus ATCC 33331	ZP_05805459.1	6 E-27	227/243
Bacteria	Firmicutes	Staphylococcus aureus A5948	ZP_05699549.1	1 E-26	231/243
Bacteria	Bacteroidetes	Spirosoma linguale DSM 74	ZP_04490099.1	1 E-26	241/243
Bacteria	Proteobacteria	Jannaschia sp. CCS1	YP_509264.1	1 E-26	229/243
Bacteria	Actinobacteria	Saccharopolyspora spinosa	AAG23281.1	1 E-26	236/243
Eukaryota	Fungi	Penicillium marneffei ATCC 18224	XP_002148524.1	1 E-26	216/243
Bacteria	Proteobacteria	Gluconacetobacter diazotrophicus PAl 5	YP_002274908.1	2 E-26	245/243
Eukaryota	Fungi	Penicillium marneffei ATCC 18224	XP_002144093.1	2 E-26	245/243
Bacteria	Actinobacteria	Streptomyces coelicolor A3(2)	NP_625189.1	2 E-26	231/243
Bacteria	Proteobacteria	Mesorhizobium opportunistum WSM2075	ZP_05808903.1	2 E-26	242/243
Eukaryota	Fungi	Aspergillus terreus NIH2624	XP_001216642.1	2 E-26	244/243
Bacteria	Firmicutes	Staphylococcus epidermidis M23864:W1	ZP_04817800.1	2 E-26	234/243
Bacteria	Firmicutes	Staphylococcus aureus subsp. aureus	YP_042001.1	3 E-26	231/243
Bacteria	Actinobacteria	Streptosporangium roseum DSM 43021	ZP_04475165.1	3 E-26	234/243
Bacteria	Bacteroidetes	Spirosoma linguale DSM 74	ZP_04489250.1	4 E-26	241/243
Eukaryota	Fungi	Aspergillus nidulans FGSC A4	XP_680537.1	6 E-26	243/243
Eukaryota	Fungi	Aspergillus oryzae RIB40	XP_001819120.1	7 E-26	243/243
Bacteria	Actinobacteria	Streptomyces hygroscopicus subsp. yingchengei	AAP21670.1	8 E-26	238/243
Eukaryota	Metazoa	Adineta vaga	ACD54816.1	8 E-26	235/243
Bacteria	Cyanobacteria	Gloeobacter violaceus PCC 7421	NP_923239.1	8 E-26	234/243
Eukaryota	Fungi	Nectria haematococca mpVI 77-13-4	EEU36907.1	9 E-26	248/243
Eukaryota	Fungi	Aspergillus flavus NRRL3357	XP_002381900.1	9 E-26	219/243
Eukaryota	Euglenozoa	Leishmania braziliensis MHOM/BR/75/M2904	XP_001568225.1	1 E-25	250/243
Eukaryota	Fungi	Aspergillus flavus NRRL3357	XP_002374911.1	1 E-25	219/243
Eukaryota	Euglenozoa	Leishmania major strain Friedlin	XP_843230.1	1 E-25	254/243
Eukaryota	Fungi	Aspergillus nidulans FGSC A4	XP_658129.1	1 E-25	215/243
Bacteria	Proteobacteria	Azoarcus sp. BH72	YP_933955.1	2 E-25	235/243
Eukaryota	Euglenozoa	Leishmania infantum JPCM5	XP_001468936.1	2 E-25	254/243
Eukaryota	Euglenozoa	Trypanosoma cruzi strain CL	XP_808880.1	2 E-25	248/243
Eukaryota	Fungi	Aspergillus oryzae RIB40	XP_001819601.1	2 E-25	219/243
Bacteria	Actinobacteria	Catenulispora acidiphila DSM 44928	YP_003115736.1	3 E-25	239/243

Eukaryota	Fungi	Aspergillus oryzae RIB40	XP_001823710.1	4 E-25	232/243
Eukaryota	Fungi	Aspergillus flavus NRRL3357	XP_002380795.1	4 E-25	232/243
Bacteria	Bacteroidetes	Flavobacterium johnsoniae UW101	YP_001196158.1	4 E-25	243/243
Eukaryota	Fungi	Aspergillus flavus NRRL3357	XP_002379763.1	5 E-25	280/243
Bacteria	Cyanobacteria	Anabaena variabilis ATCC 29413	YP_321499.1	6 E-25	235/243
Bacteria	Cyanobacteria	Synechococcus sp. PCC 7335	ZP_05040385.1	6 E-25	223/243
Bacteria	Firmicutes	Bacillus thuringiensis serovar tochigiensis	ZP_04145079.1	7 E-25	235/243
Bacteria	Bacteroidetes	Chitinophaga pinensis DSM 2588	YP_003125011.1	7 E-25	231/243
Bacteria	Bacteroidetes	Microscilla marina ATCC 23134	ZP_01690397.1	8 E-25	243/243
Bacteria	Proteobacteria	Haliangium ochraceum DSM 14365	ZP_03876425.1	1 E-24	229/243
Bacteria	Firmicutes	Staphylococcus warneri L37603	ZP_04676866.1	1 E-24	231/243
Eukaryota	Fungi	Aspergillus clavatus NRRL 1	XP_001276386.1	1 E-24	230/243
Eukaryota	Fungi	Aspergillus fumigatus A1163	EDP51005.1	2 E-24	216/243
Bacteria	Firmicutes	Staphylococcus capitis SK14	ZP_03612504.1	2 E-24	231/243
Bacteria	Proteobacteria	Stigmatella aurantiaca DW4/3-1	ZP_01461306.1	2 E-24	229/243
Bacteria	Firmicutes	Bacillus cereus AH1273;	ZP_04174016.1	2 E-24	235/243
Eukaryota	Fungi	Aspergillus flavus NRRL3357	XP_002382117.1	2 E-24	238/243
Bacteria	Actinobacteria	Frankia alni ACN14a	YP_713842.1	3 E-24	227/243
Eukaryota	Euglenozoa	Leishmania major strain Friedlin	XP_843229.1	3 E-24	230/243
Eukaryota	Fungi	Gibberella zeae PH-1	XP_388045.1	4 E-24	224/243
Eukaryota	Fungi	Botryotinia fuckeliana B05.10	XP_001555550.1	4 E-24	218/243
Eukaryota	Fungi	Aspergillus nidulans FGSC A4	XP_660073.1	5 E-24	218/243
Eukaryota	Fungi	Neosartorya fischeri NRRL 181	XP_001266377.1	5 E-24	220/243
Eukaryota	Euglenozoa	Trypanosoma cruzi strain CL	XP_809584.1	6 E-24	249/243
Eukaryota	Fungi	Talaromyces stipitatus ATCC 10500	XP_002480486.1	6 E-24	245/243
Eukaryota	Fungi	Aspergillus fumigatus Af293	XP_748189.2	7 E-24	216/243
Bacteria	Actinobacteria	Nocardia farcinica IFM 10152	YP_119534.1	8 E-24	240/243
Eukaryota	Fungi	Phaeosphaeria nodorum SN15	XP_001798597.1	9 E-24	223/243
Eukaryota	Euglenozoa	Leishmania braziliensis MHOM/BR/75/M2904	XP_001568224.1	1 E-23	230/243
Bacteria	Bacteroidetes	Spirosoma linguale DSM 74	ZP_04490485.1	1 E-23	228/243
Bacteria	Proteobacteria	Stigmatella aurantiaca DW4/3-1	ZP_01459208.1	2 E-23	234/243
Eukaryota	Euglenozoa	Leishmania infantum JPCM5	XP_001468935.1	2 E-23	230/243
Bacteria	Proteobacteria	Mesorhizobium loti MAFF303099	NP_103485.1	2 E-23	230/243
Bacteria	Actinobacteria	Kribbella flavida DSM 17836	ZP_03861235.1	2 E-23	247/243
Bacteria	Firmicutes	Bacillus cereus ATCC 4342	ZP_04283505.1	2 E-23	235/243
Bacteria	Actinobacteria	Rubrobacter xylanophilus DSM 9941	YP_643299.1	2 E-23	226/243
Archaea	Euryarchaeota	Haloarcula marismortui ATCC 43049	YP_137863.1	2 E-23	224/243
Eukaryota	Fungi	Aspergillus oryzae RIB40	XP_001821671.1	3 E-23	264/243
Bacteria	Verrucomicrobia	Verrucomicrobium spinosum DSM 4136	ZP_02926811.1	4 E-23	229/243
Bacteria	Proteobacteria	Rhizobium etli Brasil 5	ZP_03506605.1	4 E-23	243/243
Bacteria	Proteobacteria	Bdellovibrio bacteriovorus HD100	NP_970287.1	4 E-23	235/243
Bacteria	Firmicutes	Bacillus cereus G9241;	ZP_00236538.1	4 E-23	235/243
Bacteria	Firmicutes	Bacillus thuringiensis serovar huazhongensis	ZP_04083869.1	5 E-23	235/243
Bacteria	Firmicutes	Bacillus cereus 172560W;	ZP_04305594.1	5 E-23	235/243
Bacteria	Actinobacteria	Streptomyces sp. SPB78	ZP_05489807.1	5 E-23	233/243
Bacteria	Proteobacteria	Alkalilimnicola ehrlichii MLHE-1	YP_742161.1	5 E-23	234/243
Eukaryota	Fungi	Nectria haematococca mpVI 77-13-4	EEU37788.1	6 E-23	267/243
Bacteria	Bacteroidetes	Chitinophaga pinensis DSM 2588	YP_003125752.1	7 E-23	231/243
Bacteria	Actinobacteria	Salinispora arenicola CNS-205	YP_001539344.1	7 E-23	229/243
Bacteria	Firmicutes	Bacillus cereus AH1134;	ZP_03231641.1	8 E-23	235/243
Bacteria	Actinobacteria	Frankia alni ACN14a	YP_710722.1	8 E-23	229/243
Bacteria	Firmicutes	Bacillus thuringiensis serovar berliner	ZP_04101531.1	1 E-22	235/243
Bacteria	Actinobacteria	Streptomyces sp. C	ZP_05511065.1	1 E-22	236/243
Bacteria	Firmicutes	Bacillus cereus B4264;	YP_002366501.1	1 E-22	235/243
Bacteria	Firmicutes	Staphylococcus carnosus subsp. carnosus	YP_002635321.1	1 E-22	231/243
Bacteria	Bacteroidetes	Flavobacterium johnsoniae UW101	YP_001192829.1	2 E-22	243/243
Bacteria	Actinobacteria	Streptomyces sp. C	ZP_05511119.1	2 E-22	232/243
Bacteria	Actinobacteria	Streptomyces scabiei 87.22	CBG67771.1	2 E-22	229/243

Bacteria	Firmicutes	Bacillus cereus ATCC 14579	NP_831486.1	2 E-22	235/243
Bacteria	Firmicutes	Bacillus thuringiensis serovar kurstaki	ZP_04114290.1	2 E-22	235/243
Bacteria	Actinobacteria	Streptomyces sp. SPB74	ZP_04994078.1	3 E-22	228/243
Bacteria	Spirochaetes	Leptospira biflexa serovar Patoc	YP_001840227.1	3 E-22	227/243
Eukaryota	Fungi	Phaeosphaeria nodorum SN15	XP_001801482.1	7 E-22	263/243
Bacteria	Bacteroidetes	Chryseobacterium gleum ATCC 35910	ZP_03850137.1	9 E-22	236/243
Bacteria	Firmicutes	Bacillus thuringiensis serovar konkukian	YP_035948.1	9 E-22	235/243
Bacteria	Cyanobacteria	Cyanothece sp. PCC 7822	ZP_03156950.1	9 E-22	234/243
Eukaryota	Fungi	Talaromyces stipitatus ATCC 10500	XP_002482002.1	1 E-21	213/243
Bacteria	Firmicutes	Bacillus cereus m1550;	ZP_04278245.1	1 E-21	235/243
Bacteria	Actinobacteria	Frankia alni ACN14a	YP_715757.1	1 E-21	229/243
Bacteria	Cyanobacteria	Cyanothece sp. PCC 7424	YP_002377067.1	2 E-21	234/243
Eukaryota	Fungi	Verticillium albo-atrum VaMs.102	EEY20363.1	2 E-21	261/243
Eukaryota	Fungi	Sclerotinia sclerotiorum 1980 UF-70	XP_001591696.1	2 E-21	224/243
Bacteria	Actinobacteria	Actinosynnema mirum DSM 43827	YP_003100176.1	2 E-21	236/243
Eukaryota	Fungi	Podospora anserina DSM 980	XP_001903810.1	3 E-21	207/243
Bacteria	Actinobacteria	Streptomyces hygroscopicus ATCC 53653	ZP_05519547.1	3 E-21	229/243
Eukaryota	Fungi	Aspergillus nidulans FGSC A4	CBF73678.1	3 E-21	223/243
Bacteria	Actinobacteria	Streptomyces sviceus ATCC 29083	ZP_05016911.1	3 E-21	227/243
Bacteria	Firmicutes	Bacillus thuringiensis IBL 200	ZP_04071308.1	4 E-21	235/243
Eukaryota	Fungi	Moniliophthora perniciosa FA553	XP_002397072.1	4 E-21	240/243
Bacteria	Firmicutes	Bacillus coahuilensis m4-4	ZP_03225002.1	5 E-21	235/243
Bacteria	Bacteroidetes	Salinibacter ruber DSM 13855	YP_446386.1	5 E-21	227/243
Eukaryota	Fungi	Pyrenophora tritici-repentis Pt-1C-BFP	XP_001933347.1	5 E-21	269/243
Bacteria	Firmicutes	Bacillus thuringiensis serovar israelensis	ZP_00739083.1	5 E-21	235/243
Eukaryota	Fungi	Talaromyces stipitatus ATCC 10500	XP_002487425.1	6 E-21	244/243
Eukaryota	Viridiplantae	Nandina domestica	ACN87275.1	7 E-21	281/243
Bacteria	Actinobacteria	Salinispora tropica CNB-440	YP_001159019.1	9 E-21	237/243
Bacteria	Candidatus	Candidatus Accumulibacter phosphatis clade	YP_003166252.1	1 E-20	240/243
Bacteria	Verrucomicrobia	Chthoniobacter flavus Ellin428	ZP_03128557.1	1 E-20	231/243
Bacteria	Proteobacteria	Haliangium ochraceum DSM 14365	ZP_03879719.1	2 E-20	259/243
Bacteria	Cyanobacteria	Acaryochloris marina MBIC11017	YP_001515673.1	3 E-20	224/243
Eukaryota	Fungi	Moniliophthora perniciosa FA553	XP_002393362.1	3 E-20	228/243
Eukaryota	Fungi	Aspergillus nidulans FGSC A4	XP_681268.1	5 E-20	232/243
Eukaryota	Fungi	Penicillium marneffei ATCC 18224	XP_002151584.1	5 E-20	225/243
Bacteria	Proteobacteria	Nitrococcus mobilis Nb-231	ZP_01128541.1	5 E-20	240/243
Bacteria	Actinobacteria	Nocardiopsis dassonvillei subsp. dassonvillei	ZP_04333204.1	6 E-20	198/243
Bacteria	Proteobacteria	Alteromonas macleodii ATCC 27126	ZP_04717051.1	8 E-20	230/243
Bacteria	Proteobacteria	Chromohalobacter salexigens DSM 3043	YP_573140.1	8 E-20	245/243
Bacteria	Proteobacteria	Vibrio shilonii AK1	ZP_01867044.1	9 E-20	217/243
Bacteria	Cyanobacteria	Acaryochloris marina MBIC11017	YP_001518192.1	9 E-20	236/243
Bacteria	Proteobacteria	beta proteobacterium KB13	ZP_05082435.1	1 E-19	240/243
Bacteria	Proteobacteria	Rhizobium leguminosarum bv. viciae	YP_764658.1	2 E-19	213/243
Bacteria	Actinobacteria	Xylanimonas cellulosilytica DSM 15894	ZP_03911857.1	2 E-19	227/243
Bacteria	Proteobacteria	Anaeromyxobacter dehalogenans 2CP-1	YP_002493161.1	2 E-19	228/243
Bacteria	Actinobacteria	Streptomyces sp. SPB78	ZP_05486001.1	2 E-19	228/243
Bacteria	Firmicutes	Bacillus cereus Rock3-29;	ZP_04227273.1	2 E-19	235/243
Bacteria	Proteobacteria	Phenylobacterium zucineum HLK1	YP_002130457.1	2 E-19	198/243
Bacteria	Proteobacteria	Anaeromyxobacter sp. K	YP_002135017.1	3 E-19	228/243

AFUA_3G15390

Eukaryota	Fungi	Aspergillus fumigatus Af293	XP_754087.1	0.0	449/449
Eukaryota	Fungi	Neosartorya fischeri NRRL 181	XP_001261645.1	0.0	446/449
Eukaryota	Fungi	Aspergillus terreus NIH2624	XP_001216517.1	0.0	442/449
Eukaryota	Fungi	Aspergillus oryzae RIB40	XP_001827631.1	0.0	445/449
Eukaryota	Fungi	Aspergillus flavus NRRL3357	XP_002384872.1	0.0	445/449
Eukaryota	Fungi	Penicillium chrysogenum Wisconsin 54-1255	XP_002567630.1	0.0	438/449

Eukaryota	Fungi	Botryotinia fuckeliana B05.10	XP_001548734.1	0.0	439/449
Eukaryota	Fungi	Sclerotinia sclerotiorum 1980 UF-70	XP_001595416.1	0.0	427/449
Eukaryota	Fungi	Phaeosphaeria nodorum SN15	XP_001795260.1	1 E-169	432/449
Eukaryota	Fungi	Aspergillus oryzae RIB40	XP_001826757.1	9 E-94	427/449
Eukaryota	Fungi	Aspergillus flavus NRRL3357	XP_002385314.1	2 E-93	424/449
Eukaryota	Fungi	Aspergillus niger CBS 513.88	XP_001400980.1	2 E-92	424/449
Eukaryota	Fungi	Sclerotinia sclerotiorum 1980 UF-70	XP_001591465.1	2 E-91	439/449
Eukaryota	Fungi	Coccidioides posadasii C735 delta	EER25920.1	2 E-90	434/449
Eukaryota	Fungi	Schizosaccharomyces japonicus yFS275	XP_002173352.1	7 E-90	432/449
Eukaryota	Fungi	Coccidioides immitis RS;	XP_001240406.1	8 E-90	434/449
Eukaryota	Fungi	Neosartorya fischeri NRRL 181	XP_001260343.1	9 E-90	444/449
Eukaryota	Fungi	Aspergillus fumigatus Af293	XP_755169.1	1 E-89	431/449
Eukaryota	Fungi	Penicillium chrysogenum Wisconsin 54-1255	XP_002557078.1	2 E-89	429/449
Eukaryota	Fungi	Botryotinia fuckeliana B05.10	XP_001557082.1	6 E-89	394/449
Eukaryota	Fungi	Nectria haematococca mpVI 77-13-4	EEU37410.1	1 E-88	427/449
Eukaryota	Fungi	Magnaporthe grisea 70-15	XP_361625.2	1 E-88	423/449
Eukaryota	Fungi	Aspergillus niger CBS 513.88	XP_001396718.1	1 E-88	405/449
Eukaryota	Fungi	Aspergillus flavus NRRL3357	XP_002376696.1	2 E-88	428/449
Eukaryota	Fungi	Aspergillus oryzae RIB40	XP_001820961.1	2 E-88	428/449
Eukaryota	Fungi	Aspergillus oryzae RIB40	XP_001826442.1	3 E-88	447/449
Eukaryota	Fungi	Penicillium chrysogenum Wisconsin 54-1255	XP_002558878.1	5 E-88	435/449
Eukaryota	Fungi	Aspergillus niger CBS 513.88	XP_001395430.1	1 E-87	435/449
Eukaryota	Fungi	Uncinocarpus reesii 1704	XP_002582940.1	2 E-87	420/449
Eukaryota	Fungi	Aspergillus clavatus NRRL 1	XP_001267760.1	4 E-87	444/449
Eukaryota	Fungi	Gibberella zeae PH-1	XP_380395.1	1 E-86	437/449
Eukaryota	Fungi	Aspergillus terreus NIH2624	XP_001218296.1	3 E-86	435/449
Eukaryota	Fungi	Aspergillus niger CBS 513.88	XP_001391430.1	3 E-86	418/449
Eukaryota	Fungi	Aspergillus terreus NIH2624	XP_001209783.1	3 E-86	418/449
Eukaryota	Fungi	Gibberella zeae PH-1	XP_381968.1	7 E-86	444/449
Eukaryota	Fungi	Microsporum canis CBS 113480	EEQ32054.1	8 E-86	415/449
Eukaryota	Fungi	Penicillium marneffei ATCC 18224	XP_002150919.1	2 E-85	441/449
Eukaryota	Fungi	Coccidioides immitis RS;	XP_001247092.1	3 E-85	441/449
Eukaryota	Fungi	Penicillium marneffei ATCC 18224	XP_002147862.1	6 E-85	418/449
Eukaryota	Fungi	Pyrenophora tritici-repentis Pt-1C-BFP	XP_001932358.1	1 E-84	435/449
Eukaryota	Fungi	Uncinocarpus reesii 1704	XP_002541309.1	1 E-84	428/449
Eukaryota	Fungi	Coccidioides posadasii C735 delta	EER24082.1	2 E-84	440/449
Eukaryota	Fungi	Gibberella zeae PH-1	XP_384031.1	2 E-84	429/449
Eukaryota	Fungi	Aspergillus nidulans FGSC A4	XP_868838.1	2 E-84	401/449
Eukaryota	Fungi	Aspergillus flavus NRRL3357	XP_002380181.1	2 E-84	427/449
Eukaryota	Fungi	Talaromyces stipitatus ATCC 10500	XP_002478820.1	3 E-84	426/449
Eukaryota	Fungi	Sclerotinia sclerotiorum 1980 UF-70	XP_001596012.1	1 E-83	396/449
Eukaryota	Fungi	Talaromyces stipitatus ATCC 10500	XP_002484725.1	1 E-83	436/449
Eukaryota	Fungi	Schizosaccharomyces pombe	BAG68905.1	2 E-83	398/449
Eukaryota	Fungi	Talaromyces stipitatus ATCC 10500	XP_002483160.1	3 E-83	432/449
Eukaryota	Fungi	Penicillium chrysogenum Wisconsin 54-1255	XP_002567161.1	4 E-83	400/449
Eukaryota	Fungi	Penicillium marneffei ATCC 18224	XP_002149454.1	4 E-83	437/449
Eukaryota	Fungi	Aspergillus fumigatus Af293	XP_746813.2	6 E-83	407/449
Eukaryota	Fungi	Aspergillus oryzae RIB40	XP_001827145.1	7 E-83	450/449
Eukaryota	Fungi	Neosartorya fischeri NRRL 181	XP_001257578.1	9 E-83	428/449
Eukaryota	Fungi	Talaromyces stipitatus ATCC 10500	XP_002482066.1	1 E-82	431/449
Eukaryota	Fungi	Aspergillus flavus NRRL3357	XP_002384382.1	1 E-82	450/449
Eukaryota	Fungi	Aspergillus flavus NRRL3357	XP_002373851.1	2 E-82	434/449
Eukaryota	Fungi	Aspergillus flavus NRRL3357	XP_002372171.1	2 E-82	433/449
Eukaryota	Fungi	Schizosaccharomyces japonicus yFS275	XP_002171495.1	2 E-82	365/449
Eukaryota	Fungi	Neosartorya fischeri NRRL 181	XP_001262757.1	2 E-82	407/449
Eukaryota	Fungi	Penicillium chrysogenum Wisconsin 54-1255	XP_002565581.1	4 E-82	442/449
Eukaryota	Fungi	Aspergillus fumigatus A1163	EDP48067.1	5 E-82	407/449
Eukaryota	Fungi	Aspergillus terreus NIH2624	XP_001215681.1	5 E-82	423/449

Eukaryota	Fungi	Nectria haematococca mpVI 77-13-4	EEU35468.1	5 E-82	432/449
Eukaryota	Fungi	Aspergillus niger CBS 513.88	XP_001395282.1	7 E-82	407/449
Eukaryota	Fungi	Pyrenophora tritici-repentis Pt-1C-BFP	XP_001941559.1	1 E-81	437/449
Eukaryota	Fungi	Aspergillus niger CBS 513.88	XP_001394848.1	3 E-81	395/449
Eukaryota	Fungi	Aspergillus terreus NIH2624	XP_001215206.1	5 E-81	408/449
Eukaryota	Fungi	Aspergillus oryzae RIB40	XP_001818760.1	6 E-81	427/449
Eukaryota	Fungi	Aspergillus clavatus NRRL 1	XP_001274108.1	6 E-81	418/449
Eukaryota	Fungi	Yarrowia lipolytica CLIB122	XP_502460.1	1 E-80	434/449
Eukaryota	Fungi	Aspergillus terreus NIH2624	XP_001217783.1	2 E-80	436/449
Eukaryota	Fungi	Nectria haematococca mpVI 77-13-4	EEU35132.1	3 E-80	400/449
Eukaryota	Fungi	Penicillium chrysogenum Wisconsin 54-1255	XP_002565373.1	3 E-80	427/449
Eukaryota	Fungi	Candida tropicalis MYA-3404	XP_002548230.1	4 E-80	431/449
Eukaryota	Fungi	Aspergillus oryzae RIB40	XP_001816586.1	5 E-80	400/449
Eukaryota	Fungi	Aspergillus niger CBS 513.88	XP_001389570.1	6 E-80	404/449
Eukaryota	Fungi	Penicillium chrysogenum Wisconsin 54-1255	XP_002558186.1	8 E-80	403/449
Eukaryota	Fungi	Aspergillus fumigatus Af293	XP_753692.1	9 E-80	416/449
Eukaryota	Fungi	Nectria haematococca mpVI 77-13-4	EEU42186.1	1 E-79	401/449
Eukaryota	Fungi	Aspergillus nidulans FGSC A4	XP_680649.1	2 E-79	439/449
Eukaryota	Fungi	Nectria haematococca mpVI 77-13-4	EEU43631.1	2 E-79	400/449
Eukaryota	Fungi	Neosartorya fischeri NRRL 181	XP_001259742.1	2 E-79	416/449
Eukaryota	Fungi	Aspergillus clavatus NRRL 1	XP_001272427.1	5 E-79	398/449
Eukaryota	Fungi	Gibberella zeae PH-1	XP_381888.1	5 E-79	434/449
Eukaryota	Fungi	Nectria haematococca mpVI 77-13-4	EEU43837.1	5 E-79	437/449
Eukaryota	Fungi	Ustilago maydis 521	XP_756521.1	6 E-79	451/449
Eukaryota	Fungi	Gibberella zeae PH-1	XP_387760.1	8 E-79	429/449
Eukaryota	Fungi	Aspergillus niger CBS 513.88	XP_001402514.1	8 E-79	421/449
Eukaryota	Fungi	Penicillium marneffei ATCC 18224	XP_002146530.1	9 E-79	389/449
Eukaryota	Fungi	Lodderomyces elongisporus NRRL YB-4239	XP_001525551.1	9 E-79	434/449
Eukaryota	Fungi	Microsporum canis CBS 113480	EEQ33925.1	1 E-78	453/449
Eukaryota	Fungi	Magnaporthe grisea 70-15	XP_359884.1	1 E-78	429/449
Eukaryota	Fungi	Debaryomyces hansenii CBS767	XP_460392.1	1 E-78	417/449
Eukaryota	Fungi	Aspergillus oryzae RIB40	XP_001821708.1	2 E-78	401/449
Eukaryota	Fungi	Aspergillus flavus NRRL3357	XP_002375055.1	2 E-78	397/449
Eukaryota	Fungi	Gibberella zeae PH-1	XP_380521.1	2 E-78	439/449
Eukaryota	Fungi	Pichia guilliermondii ATCC 6260	EDK41700.2	2 E-78	437/449
Eukaryota	Fungi	Aspergillus nidulans FGSC A4	XP_661107.1	3 E-78	405/449
Eukaryota	Fungi	Aspergillus nidulans FGSC A4	CBF81557.1	4 E-78	399/449
Eukaryota	Fungi	Aspergillus flavus NRRL3357	XP_002379720.1	7 E-78	401/449
Eukaryota	Fungi	Aspergillus terreus NIH2624	XP_001209942.1	7 E-78	444/449
Eukaryota	Fungi	Pichia guilliermondii ATCC 6260	EDK41588.2	8 E-78	437/449
Eukaryota	Fungi	Pichia guilliermondii ATCC 6260	XP_001482035.1	1 E-77	437/449
Eukaryota	Fungi	Talaromyces stipitatus ATCC 10500	XP_002478263.1	1 E-77	400/449
Eukaryota	Fungi	Aspergillus flavus NRRL3357	XP_002385354.1	2 E-77	443/449
Eukaryota	Fungi	Aspergillus oryzae RIB40	XP_001824868.1	2 E-77	409/449
Eukaryota	Fungi	Nectria haematococca mpVI 77-13-4	EEU33950.1	2 E-77	396/449
Eukaryota	Fungi	Aspergillus niger CBS 513.88	XP_001389913.1	2 E-77	433/449
Eukaryota	Fungi	Nectria haematococca mpVI 77-13-4	EEU45196.1	3 E-77	404/449
Eukaryota	Fungi	Schizosaccharomyces pombe	NP_587688.1	4 E-77	416/449
Eukaryota	Fungi	Candida albicans SC5314	XP_720136.1	4 E-77	423/449
Eukaryota	Fungi	Pichia guilliermondii ATCC 6260	XP_001481923.1	5 E-77	437/449
Eukaryota	Fungi	Nectria haematococca mpVI 77-13-4	EEU37655.1	5 E-77	400/449
Eukaryota	Fungi	Aspergillus nidulans FGSC A4	XP_664065.1	5 E-77	452/449
Eukaryota	Fungi	Aspergillus flavus NRRL3357	XP_002383726.1	6 E-77	437/449
Eukaryota	Fungi	Clavispora lusitaniae ATCC 42720	XP_002616761.1	7 E-77	425/449
Eukaryota	Fungi	Aspergillus nidulans FGSC A4	CBF77966.1	8 E-77	421/449
Eukaryota	Fungi	Aspergillus flavus NRRL3357	XP_002383516.1	9 E-77	403/449
Eukaryota	Fungi	Candida albicans SC5314	XP_714757.1	9 E-77	400/449
Eukaryota	Fungi	Aspergillus niger CBS 513.88	XP_001392249.1	9 E-77	396/449

Eukaryota	Fungi	Phaeosphaeria nodorum SN15	XP_001806066.1	1 E-76	446/449
Eukaryota	Fungi	Aspergillus clavatus NRRL 1	XP_001268786.1	1 E-76	442/449
Eukaryota	Fungi	Aspergillus flavus NRRL3357	XP_002380074.1	2 E-76	440/449
Eukaryota	Fungi	Lodderomyces elongisporus NRRL YB-4239	XP_001523276.1	2 E-76	449/449
Eukaryota	Fungi	Nectria haematococca mpVI 77-13-4	EEU38145.1	3 E-76	427/449
Eukaryota	Fungi	Aspergillus flavus NRRL3357	XP_002379901.1	3 E-76	442/449
Eukaryota	Fungi	Nectria haematococca mpVI 77-13-4	EEU42123.1	3 E-76	443/449
Eukaryota	Fungi	Magnaporthe grisea 70-15	XP_366174.2	7 E-76	421/449
Eukaryota	Fungi	Nectria haematococca mpVI 77-13-4	EEU35596.1	8 E-76	400/449
Eukaryota	Fungi	Penicillium marneffei ATCC 18224	XP_002145966.1	8 E-76	405/449
Eukaryota	Fungi	Schizosaccharomyces pombe	NP_588287.1	9 E-76	400/449
Eukaryota	Fungi	Penicillium chrysogenum Wisconsin 54-1255	XP_002557154.1	1 E-75	422/449
Eukaryota	Fungi	Aspergillus niger CBS 513.88	XP_001391951.1	1 E-75	432/449
Eukaryota	Fungi	Debaryomyces hansenii CBS767	XP_458208.1	1 E-75	421/449
Eukaryota	Fungi	Microsporum canis CBS 113480	EEQ35405.1	1 E-75	442/449
Eukaryota	Fungi	Lachancea thermotolerans CBS 6340	XP_002553625.1	1 E-75	420/449
Eukaryota	Fungi	Candida albicans SC5314	XP_723075.1	2 E-75	396/449
Eukaryota	Fungi	Aspergillus terreus NIH2624	XP_001211787.1	2 E-75	444/449
Eukaryota	Fungi	Aspergillus oryzae RIB40	XP_001821553.1	2 E-75	442/449
Eukaryota	Fungi	Candida dubliniensis CD36	XP_002419328.1	2 E-75	396/449
Eukaryota	Fungi	Candida albicans WO-1	EEQ44461.1	3 E-75	396/449
Eukaryota	Fungi	Yarrowia lipolytica CLIB122	XP_502810.1	3 E-75	433/449
Eukaryota	Fungi	Candida albicans WO-1	EEQ44366.1	4 E-75	423/449
Eukaryota	Fungi	Candida dubliniensis CD36	XP_002419285.1	4 E-75	423/449
Eukaryota	Fungi	Candida albicans WO-1	EEQ44413.1	4 E-75	423/449
Eukaryota	Fungi	Aspergillus niger CBS 513.88	XP_001397379.1	5 E-75	407/449
Eukaryota	Fungi	Aspergillus nidulans FGSC A4	XP_682170.1	5 E-75	394/449
Eukaryota	Fungi	Penicillium chrysogenum Wisconsin 54-1255	XP_002566225.1	7 E-75	396/449
Eukaryota	Fungi	Magnaporthe grisea 70-15	XP_001462606.1	8 E-75	437/449
Eukaryota	Fungi	Lodderomyces elongisporus NRRL YB-4239	XP_001527654.1	8 E-75	421/449
Eukaryota	Fungi	Penicillium marneffei ATCC 18224	XP_002152600.1	1 E-74	420/449
Eukaryota	Fungi	Candida albicans SC5314	XP_712757.1	1 E-74	423/449
Eukaryota	Fungi	Aspergillus fumigatus Af293	XP_752839.1	2 E-74	447/449
Eukaryota	Fungi	Neosartorya fischeri NRRL 181	XP_001264286.1	2 E-74	447/449
Eukaryota	Fungi	Pichia guilliermondii ATCC 6260	EDK40451.2	2 E-74	424/449
Eukaryota	Fungi	Aspergillus niger CBS 513.88	XP_001401651.1	2 E-74	422/449
Eukaryota	Fungi	Nectria haematococca mpVI 77-13-4	EEU48676.1	3 E-74	436/449
Eukaryota	Fungi	Pichia guilliermondii ATCC 6260	XP_001483820.1	3 E-74	424/449
Eukaryota	Fungi	Talaromyces stipitatus ATCC 10500	XP_002486298.1	4 E-74	413/449
Eukaryota	Fungi	Coccidioides posadasii C735 delta	EER24843.1	7 E-74	421/449
Eukaryota	Fungi	Coccidioides immitis RS;	XP_001239364.1	7 E-74	409/449
Eukaryota	Fungi	Aspergillus terreus NIH2624	XP_001214171.1	8 E-74	417/449
Eukaryota	Fungi	Aspergillus oryzae RIB40	XP_001825867.1	1 E-73	397/449
Eukaryota	Fungi	Yarrowia lipolytica CLIB122	XP_505496.1	1 E-73	423/449
Eukaryota	Fungi	Pichia stipitis CBS 6054	XP_001382424.1	2 E-73	403/449
Eukaryota	Fungi	Nectria haematococca mpVI 77-13-4	EEU36590.1	2 E-73	406/449
Eukaryota	Fungi	Aspergillus terreus NIH2624	XP_001208701.1	2 E-73	418/449
Eukaryota	Fungi	Aspergillus oryzae RIB40	XP_001816979.1	2 E-73	404/449
Eukaryota	Fungi	Nectria haematococca mpVI 77-13-4	EEU37957.1	3 E-73	414/449
Eukaryota	Fungi	Aspergillus niger CBS 513.88	XP_001396347.1	4 E-73	403/449
Eukaryota	Fungi	Yarrowia lipolytica CLIB122	XP_501799.1	4 E-73	418/449
Eukaryota	Fungi	Postia placenta Mad-698-R	XP_002475499.1	4 E-73	445/449
Eukaryota	Fungi	Penicillium marneffei ATCC 18224	XP_002143194.1	6 E-73	395/449
Eukaryota	Fungi	Debaryomyces hansenii CBS767	XP_462626.1	9 E-73	421/449
Eukaryota	Fungi	Penicillium chrysogenum Wisconsin 54-1255	XP_002561028.1	1 E-72	431/449
Eukaryota	Fungi	Microsporum canis CBS 113480	EEQ30684.1	1 E-72	438/449
Eukaryota	Fungi	Gibberella zeae PH-1	XP_383121.1	1 E-72	397/449
Eukaryota	Fungi	Gibberella zeae PH-1	XP_380284.1	1 E-72	425/449

Eukaryota	Fungi	Cryptococcus neoformans var. neoformans	XP_773020.1	2 E-72	405/449
Eukaryota	Fungi	Aspergillus niger CBS 513.88	XP_001399011.1	2 E-72	440/449
Eukaryota	Fungi	Cryptococcus neoformans var. neoformans	XP_567308.1	2 E-72	405/449
Eukaryota	Fungi	Aspergillus nidulans FGSC A4	XP_682083.1	2 E-72	416/449
Eukaryota	Fungi	Aspergillus oryzae RIB40	XP_001818664.1	2 E-72	443/449
Eukaryota	Fungi	Penicillium chrysogenum Wisconsin 54-1255	XP_002559484.1	3 E-72	398/449
Eukaryota	Fungi	Lachancea thermotolerans CBS 6340	XP_002552234.1	4 E-72	447/449
Eukaryota	Fungi	Aspergillus clavatus NRRL 1	XP_001273703.1	4 E-72	403/449
Eukaryota	Fungi	Penicillium chrysogenum Wisconsin 54-1255	XP_002569127.1	5 E-72	394/449
Eukaryota	Fungi	Talaromyces stipitatus ATCC 10500	XP_002482683.1	6 E-72	441/449
Eukaryota	Fungi	Aspergillus oryzae RIB40	XP_001820853.1	6 E-72	429/449
Eukaryota	Fungi	Pyrenophora tritici-repentis Pt-1C-BFP	XP_001932146.1	7 E-72	444/449
Eukaryota	Fungi	Aspergillus niger CBS 513.88	XP_001390406.1	2 E-71	440/449
Eukaryota	Fungi	Ajellomyces dermatitidis ER-3	EEQ88654.1	2 E-71	442/449
Eukaryota	Fungi	Neurospora crassa OR74A	XP_965426.1	2 E-71	440/449
Eukaryota	Fungi	Neosartorya fischeri NRRL 181	XP_001258860.1	2 E-71	403/449
Eukaryota	Fungi	Ajellomyces dermatitidis SLH14081	XP_002627115.1	2 E-71	442/449
Eukaryota	Fungi	Aspergillus niger CBS 513.88	XP_001395707.1	2 E-71	416/449
Eukaryota	Fungi	Penicillium marneffei ATCC 18224	XP_002152265.1	2 E-71	448/449
Eukaryota	Fungi	Cryptococcus neoformans var. neoformans	XP_571744.1	2 E-71	408/449
Eukaryota	Fungi	Gibberella zeae PH-1	XP_382256.1	3 E-71	397/449
Eukaryota	Fungi	Lodderomyces elongisporus NRRL YB-4239	XP_001524969.1	4 E-71	421/449
Eukaryota	Fungi	Talaromyces stipitatus ATCC 10500	XP_002487211.1	5 E-71	394/449
Eukaryota	Fungi	Aspergillus oryzae RIB40	XP_001825038.1	6 E-71	415/449
Eukaryota	Fungi	Aspergillus flavus NRRL3357	XP_002383084.1	6 E-71	419/449
Eukaryota	Fungi	Aspergillus oryzae RIB40	XP_001820549.1	7 E-71	402/449
Eukaryota	Fungi	Pichia pastoris GS115	XP_002489943.1	7 E-71	418/449
Eukaryota	Fungi	Aspergillus terreus NIH2624	XP_001210770.1	8 E-71	441/449
Eukaryota	Fungi	Aspergillus oryzae RIB40	XP_001818726.1	9 E-71	420/449
Eukaryota	Fungi	Cryptococcus neoformans var. neoformans	XP_571059.1	9 E-71	427/449
Eukaryota	Fungi	Aspergillus fumigatus Af293	XP_748528.1	9 E-71	441/449
Eukaryota	Fungi	Cryptococcus neoformans var. neoformans	XP_571481.1	1 E-70	414/449
Eukaryota	Fungi	Penicillium chrysogenum Wisconsin 54-1255	XP_002567504.1	1 E-70	403/449
Eukaryota	Fungi	Pichia stipitis CBS 6054	XP_001384207.2	1 E-70	418/449
Eukaryota	Fungi	Zygosaccharomyces rouxii CBS 732	XP_002494970.1	2 E-70	428/449
Eukaryota	Fungi	Nectria haematococca mpVI 77-13-4	EEU35737.1	2 E-70	422/449
Eukaryota	Fungi	Debaryomyces hansenii	CAG84407.2	2 E-70	438/449
Eukaryota	Fungi	Pyrenophora tritici-repentis Pt-1C-BFP	XP_001932699.1	2 E-70	394/449
Eukaryota	Fungi	Penicillium marneffei ATCC 18224	XP_002143914.1	2 E-70	420/449
Eukaryota	Fungi	Penicillium marneffei ATCC 18224	XP_002149541.1	2 E-70	453/449
Eukaryota	Fungi	Uncinocarpus reesii 1704	XP_002543870.1	2 E-70	435/449
Eukaryota	Fungi	Kluyveromyces lactis NRRL Y-1140	XP_451180.1	2 E-70	405/449
Eukaryota	Fungi	Debaryomyces hansenii CBS767	XP_456455.1	2 E-70	438/449
Eukaryota	Fungi	Yarrowia lipolytica CLIB122	XP_501924.1	3 E-70	421/449
Eukaryota	Fungi	Lodderomyces elongisporus NRRL YB-4239	XP_001524593.1	4 E-70	397/449
Eukaryota	Fungi	Nectria haematococca mpVI 77-13-4	EEU34922.1	4 E-70	406/449
Eukaryota	Fungi	Nectria haematococca mpVI 77-13-4	EEU40122.1	4 E-70	416/449
Eukaryota	Fungi	Pichia stipitis CBS 6054	EAZ63032.2	4 E-70	439/449
Eukaryota	Fungi	Lachancea thermotolerans CBS 6340	XP_002554597.1	5 E-70	401/449
Eukaryota	Fungi	Aspergillus oryzae RIB40	XP_001819672.1	5 E-70	447/449
Eukaryota	Fungi	Aspergillus nidulans FGSC A4	CBF90344.1	6 E-70	449/449
Eukaryota	Fungi	Ajellomyces capsulatus NAm1	XP_001539635.1	8 E-70	414/449
Eukaryota	Fungi	Cryptococcus neoformans var. neoformans	XP_774645.1	1 E-69	414/449
Eukaryota	Fungi	Verticillium albo-atrum VaMs.102	EEY19852.1	1 E-69	443/449
Eukaryota	Fungi	Aspergillus flavus NRRL3357	XP_002374831.1	1 E-69	447/449
Eukaryota	Fungi	Ustilago maydis 521	XP_758091.1	1 E-69	421/449
Eukaryota	Fungi	Verticillium albo-atrum VaMs.102	EEY22924.1	2 E-69	450/449
Eukaryota	Fungi	Debaryomyces hansenii	CAG84621.2	2 E-69	421/449

Eukaryota	Fungi	Debaryomyces hansenii CBS767	XP_456665.1	2 E-69	421/449
Eukaryota	Fungi	Aspergillus nidulans FGSC A4	XP_657633.1	2 E-69	434/449
Eukaryota	Fungi	Penicillium chrysogenum Wisconsin 54-1255	XP_002561693.1	3 E-69	440/449
Eukaryota	Fungi	Pichia stipitis CBS 6054	XP_001387055.1	3 E-69	439/449
Eukaryota	Fungi	Phaeosphaeria nodorum SN15	XP_001806492.1	4 E-69	437/449
Eukaryota	Fungi	Nectria haematococca mpVI 77-13-4	EEU37396.1	5 E-69	426/449
Eukaryota	Fungi	Verticillium albo-atrum VaMs.102	EEY23343.1	9 E-69	429/449
Eukaryota	Fungi	Aspergillus oryzae RIB40	XP_001826720.1	1 E-68	417/449
Eukaryota	Fungi	Gibberella zeae PH-1	XP_388275.1	1 E-68	381/449
Eukaryota	Fungi	Nectria haematococca mpVI 77-13-4	EEU39443.1	1 E-68	421/449
Eukaryota	Fungi	Pichia guilliermondii ATCC 6260	XP_001486993.1	2 E-68	401/449

AFUA_4G00340

Eukaryota	Fungi	Aspergillus fumigatus Af293	XP_746422.1	0.0	348/348
Eukaryota	Fungi	Neosartorya fischeri NRRL 181	XP_001267608.1	0.0	348/348
Eukaryota	Fungi	Aspergillus clavatus NRRL 1	XP_001270533.1	1 E-173	345/348
Eukaryota	Fungi	Aspergillus oryzae RIB40	XP_001727288.1	1 E-162	348/348
Eukaryota	Fungi	Aspergillus niger CBS 513.88	XP_001389497.1	1 E-161	347/348
Eukaryota	Fungi	Penicillium chrysogenum Wisconsin 54-1255	XP_002563826.1	1 E-154	348/348
Eukaryota	Fungi	Aspergillus nidulans FGSC A4	XP_658553.1	1 E-152	342/348
Eukaryota	Fungi	Aspergillus terreus NIH2624	XP_001211639.1	1 E-150	341/348
Eukaryota	Fungi	Aspergillus niger CBS 513.88	XP_001398577.1	1 E-137	346/348
Eukaryota	Fungi	Penicillium chrysogenum Wisconsin 54-1255	XP_002568690.1	1 E-136	345/348
Eukaryota	Fungi	Aspergillus oryzae RIB40	XP_001820475.1	1 E-134	348/348
Eukaryota	Fungi	Talaromyces stipitatus ATCC 10500	XP_002481188.1	1 E-134	344/348
Eukaryota	Fungi	Aspergillus terreus NIH2624	XP_001210026.1	1 E-134	346/348
Eukaryota	Fungi	Aspergillus flavus NRRL3357	XP_002373929.1	1 E-134	348/348
Eukaryota	Fungi	Aspergillus fumigatus Af293	XP_753577.1	1 E-133	341/348
Eukaryota	Fungi	Aspergillus clavatus NRRL 1	XP_001274375.1	1 E-132	344/348
Eukaryota	Fungi	Penicillium marneffei ATCC 18224	XP_002152154.1	1 E-128	346/348
Eukaryota	Fungi	Aspergillus flavus NRRL3357	XP_002377541.1	1 E-128	348/348
Eukaryota	Fungi	Aspergillus nidulans FGSC A4	XP_660434.1	1 E-126	348/348
Eukaryota	Fungi	Neosartorya fischeri NRRL 181	XP_001259621.1	1 E-125	341/348
Eukaryota	Fungi	Gibberella zeae PH-1	XP_391620.1	1 E-124	345/348
Eukaryota	Fungi	Penicillium chrysogenum Wisconsin 54-1255	XP_002558940.1	1 E-122	345/348
Eukaryota	Fungi	Aspergillus oryzae RIB40	XP_001825913.1	1 E-122	339/348
Eukaryota	Fungi	Talaromyces stipitatus ATCC 10500	XP_002340193.1	1 E-120	317/348
Eukaryota	Fungi	Aspergillus flavus NRRL3357	XP_002379912.1	1 E-118	338/348
Eukaryota	Fungi	Aspergillus terreus NIH2624	XP_001211677.1	1 E-117	342/348
Eukaryota	Fungi	Penicillium chrysogenum Wisconsin 54-1255	XP_002559544.1	1 E-113	342/348
Eukaryota	Fungi	Aspergillus oryzae RIB40	XP_001826877.1	1 E-109	341/348
Eukaryota	Fungi	Aspergillus flavus NRRL3357	XP_002385173.1	1 E-109	338/348
Eukaryota	Fungi	Ustilago maydis 521	XP_760176.1	2 E-69	338/348
Eukaryota	Fungi	Ustilago maydis 521	XP_757553.1	6 E-67	331/348
Eukaryota	Fungi	Ustilago maydis 521	XP_761766.1	2 E-63	345/348
Eukaryota	Fungi	Ustilago maydis 521	XP_761217.1	2 E-55	338/348
Eukaryota	Fungi	Ustilago maydis 521	XP_760328.1	1 E-34	322/348

AFUA_4G00610

Eukaryota	Fungi	Aspergillus fumigatus Af293	XP_746395.1	0.0	601/601
Eukaryota	Fungi	Neosartorya fischeri NRRL 181	XP_001267585.1	0.0	601/601
Eukaryota	Fungi	Penicillium chrysogenum Wisconsin 54-1255	XP_002564952.1	0.0	600/601
Eukaryota	Fungi	Aspergillus clavatus NRRL 1	XP_001270520.1	0.0	616/601
Eukaryota	Fungi	Aspergillus niger CBS 513.88	XP_001399178.1	0.0	604/601
Eukaryota	Fungi	Aspergillus oryzae RIB40	XP_001820465.1	0.0	599/601
Eukaryota	Fungi	Aspergillus terreus NIH2624	XP_001215580.1	0.0	599/601

Eukaryota	Fungi	Podospora anserina DSM 980	XP_001905344.1	1 E-164	592/601
Eukaryota	Fungi	Chaetomium globosum CBS 148.51	XP_001226145.1	1 E-151	561/601
Eukaryota	Fungi	Aspergillus niger CBS 513.88	XP_001392939.1	1 E-150	595/601
Eukaryota	Fungi	Penicillium marneffei ATCC 18224	XP_002151382.1	1 E-150	596/601
Eukaryota	Fungi	Neurospora crassa OR74A	XP_960108.2	1 E-145	588/601
Eukaryota	Fungi	Aspergillus nidulans FGSC A4	XP_681598.1	1 E-138	586/601
Eukaryota	Fungi	Ajellomyces capsulatus G186AR	EEH08424.1	1 E-125	593/601
Eukaryota	Fungi	Nectria haematococca mpVI 77-13-4	EEU39492.1	1 E-123	590/601
Eukaryota	Fungi	Ajellomyces capsulatus NAm1	XP_001544836.1	1 E-122	593/601
Eukaryota	Fungi	Podospora anserina DSM 980	XP_001904207.1	1 E-121	603/601
Eukaryota	Fungi	Aspergillus niger CBS 513.88	XP_001395781.1	1 E-121	589/601
Eukaryota	Fungi	Phaeosphaeria nodorum SN15	XP_001791642.1	1 E-112	590/601
Eukaryota	Fungi	Botryotinia fuckeliana B05.10	XP_001548196.1	1 E-110	585/601
Eukaryota	Fungi	Verticillium albo-atrum VaMs.102	EEY16122.1	1 E-108	585/601
Eukaryota	Fungi	Aspergillus oryzae RIB40	XP_001817583.1	1 E-105	595/601
Eukaryota	Fungi	Aspergillus flavus NRRL3357	XP_002372700.1	1 E-105	595/601
Eukaryota	Fungi	Verticillium albo-atrum VaMs.102	EEY18875.1	1 E-103	596/601
Eukaryota	Fungi	Coprinopsis cinerea okayama7#130	XP_001834096.1	1 E-101	589/601
Eukaryota	Fungi	Botryotinia fuckeliana B05.10	XP_001552368.1	1 E-100	604/601
Eukaryota	Fungi	Aspergillus flavus NRRL3357	XP_002385344.1	1 E-100	590/601
Eukaryota	Fungi	Ustilago maydis 521	XP_757858.1	2 E-97	588/601
Eukaryota	Fungi	Nectria haematococca mpVI 77-13-4	EEU37471.1	4 E-96	584/601
Eukaryota	Fungi	Monascus aurantiacus	ACA34720.1	8 E-95	597/601
Eukaryota	Fungi	Aspergillus niger CBS 513.88	XP_001396334.1	5 E-94	550/601
Eukaryota	Fungi	Coccidioides posadasii C735 delta	EER29992.1	6 E-94	591/601
Eukaryota	Fungi	Chaetomium globosum CBS 148.51	XP_001223662.1	4 E-93	596/601
Eukaryota	Fungi	Coccidioides immitis RS;	XP_001239189.1	1 E-91	591/601
Eukaryota	Fungi	Aspergillus oryzae RIB40	XP_001826731.1	3 E-90	604/601
Eukaryota	Fungi	Aspergillus clavatus NRRL 1	XP_001276775.1	4 E-90	532/601
Eukaryota	Fungi	Aspergillus niger CBS 513.88	XP_001401665.1	9 E-89	522/601
Eukaryota	Fungi	Ustilago maydis 521	XP_760191.1	4 E-88	575/601
Eukaryota	Fungi	Aspergillus terreus NIH2624	XP_001214030.1	7 E-88	586/601
Eukaryota	Fungi	Talaromyces stipitatus ATCC 10500	XP_002486243.1	9 E-88	588/601
Eukaryota	Fungi	Nectria haematococca mpVI 77-13-4	EEU43534.1	1 E-86	570/601
Eukaryota	Fungi	Aspergillus niger CBS 513.88	XP_001396051.1	1 E-86	599/601
Eukaryota	Fungi	Gibberella zeae PH-1	XP_383651.1	3 E-86	583/601
Eukaryota	Fungi	Penicillium chrysogenum Wisconsin 54-1255	XP_002560665.1	1 E-84	591/601
Eukaryota	Fungi	Aspergillus nidulans FGSC A4	XP_682280.1	1 E-84	588/601
Eukaryota	Fungi	Gibberella zeae PH-1	XP_383802.1	3 E-84	573/601
Eukaryota	Fungi	Podospora anserina DSM 980	XP_001910548.1	6 E-84	580/601
Eukaryota	Fungi	Phaeosphaeria nodorum SN15	XP_001805040.1	7 E-84	592/601
Eukaryota	Fungi	Aspergillus terreus NIH2624	XP_001216916.1	5 E-83	560/601
Eukaryota	Fungi	Aspergillus terreus NIH2624	XP_001218113.1	9 E-83	577/601
Eukaryota	Fungi	Nectria haematococca mpVI 77-13-4	EEU36182.1	1 E-82	575/601
Eukaryota	Fungi	Aspergillus flavus NRRL3357	XP_002376612.1	3 E-81	584/601
Eukaryota	Fungi	Gibberella zeae PH-1	XP_383916.1	4 E-81	574/601
Eukaryota	Fungi	Phaeosphaeria nodorum SN15	XP_001804484.1	5 E-81	564/601
Eukaryota	Fungi	Fusarium oxysporum f. sp.	CAJ85791.1	6 E-81	575/601
Eukaryota	Fungi	Aspergillus nidulans FGSC A4	XP_661610.1	1 E-80	578/601
Eukaryota	Fungi	Pyrenophora tritici-repentis Pt-1C-BFP	XP_001937082.1	3 E-80	564/601
Eukaryota	Fungi	Aspergillus oryzae RIB40	XP_001820890.1	2 E-79	583/601
Eukaryota	Fungi	Botryotinia fuckeliana	CAD88590.1	3 E-78	564/601
Eukaryota	Fungi	Malassezia globosa CBS 7966	XP_001732157.1	7 E-78	576/601
Eukaryota	Fungi	Aspergillus niger CBS 513.88	XP_001394544.1	2 E-77	558/601
Eukaryota	Fungi	Malassezia sympodialis	CAI43283.4	3 E-77	573/601
Eukaryota	Fungi	Pyrenophora tritici-repentis Pt-1C-BFP	XP_001939413.1	2 E-75	568/601
Eukaryota	Fungi	Coccidioides immitis RS;	XP_001240784.1	3 E-75	570/601
Eukaryota	Fungi	Postia placenta Mad-698-R	XP_002469715.1	4 E-75	562/601

Eukaryota	Fungi	Coccidioides posadasii C735 delta	EER22976.1	4 E-75	570/601
Eukaryota	Fungi	Aspergillus niger CBS 513.88	XP_001391138.1	6 E-75	560/601
Eukaryota	Fungi	Sclerotinia sclerotiorum 1980 UF-70	XP_001584680.1	1 E-74	562/601
Bacteria	Proteobacteria	Pseudomonas aeruginosa PACS2	ZP_01367534.1	2 E-74	532/601
Bacteria	Proteobacteria	Pseudomonas aeruginosa C3719	ZP_04930310.1	2 E-74	532/601
Bacteria	Proteobacteria	Pseudomonas aeruginosa PAO1	NP_252789.1	2 E-74	532/601
Bacteria	Proteobacteria	Pseudomonas aeruginosa LESB58	YP_002438481.1	4 E-74	531/601
Eukaryota	Fungi	Podospora anserina DSM 980	XP_001907031.1	5 E-74	569/601
Bacteria	Proteobacteria	Pseudomonas aeruginosa UCBPP-PA14	YP_789017.1	9 E-74	531/601
Eukaryota	Fungi	Verticillium albo-atrum VaMs.102	EEY23008.1	1 E-73	549/601
Eukaryota	Fungi	Chaetomium globosum CBS 148.51	XP_001224472.1	3 E-73	564/601
Eukaryota	Fungi	Aspergillus oryzae RIB40	XP_001826806.1	3 E-73	560/601
Eukaryota	Fungi	Gibberella zeae PH-1	XP_391162.1	1 E-72	564/601
Eukaryota	Fungi	Aspergillus flavus NRRL3357	XP_002372599.1	1 E-72	561/601
Bacteria	Proteobacteria	Pseudomonas aeruginosa PA7	YP_001346382.1	2 E-72	531/601
Eukaryota	Fungi	Botryotinia fuckeliana B05.10	XP_001558188.1	2 E-72	587/601
Eukaryota	Fungi	Aspergillus oryzae RIB40	XP_001817515.1	6 E-72	556/601
Eukaryota	Fungi	Penicillium marneffei ATCC 18224	XP_002147168.1	5 E-71	559/601
Eukaryota	Fungi	Moniliophthora perniciosa FA553	XP_002388554.1	1 E-70	584/601
Eukaryota	Fungi	Podospora anserina DSM 980	XP_001904543.1	3 E-70	574/601
Bacteria	Proteobacteria	Pseudomonas fluorescens Pf-5	YP_260472.1	7 E-70	530/601
Eukaryota	Fungi	Ustilago maydis 521	XP_759762.1	9 E-70	588/601
Eukaryota	Fungi	Verticillium albo-atrum VaMs.102	EEY22366.1	2 E-69	575/601
Eukaryota	Fungi	Laccaria bicolor S238N-H82	XP_001885676.1	4 E-69	555/601
Eukaryota	Fungi	Coprinopsis cinerea okayama7#130	XP_001840660.1	3 E-68	592/601
Eukaryota	Fungi	Ustilago maydis 521	XP_761104.1	1 E-67	550/601
Eukaryota	Fungi	Sclerotinia sclerotiorum 1980 UF-70	XP_001586361.1	2 E-67	559/601
Eukaryota	Fungi	Chaetomium globosum CBS 148.51	XP_001227424.1	2 E-67	604/601
Bacteria	Proteobacteria	Thalassiobium sp. R2A62	ZP_05343380.1	2 E-66	527/601
Eukaryota	Fungi	Penicillium chrysogenum Wisconsin 54-1255	XP_002565293.1	3 E-66	566/601
Eukaryota	Fungi	Coprinopsis cinerea okayama7#130	XP_001836517.1	5 E-66	568/601
Bacteria	Proteobacteria	Burkholderia graminis C4D1M	ZP_02883654.1	6 E-66	531/601
Bacteria	Proteobacteria	Marinomonas sp. MED121	ZP_01075202.1	1 E-65	534/601
Eukaryota	Fungi	Uncinocarpus reesii 1704	XP_002542728.1	1 E-65	581/601
Bacteria	Proteobacteria	Burkholderia phymatum STM815	YP_001862312.1	2 E-65	528/601
Eukaryota	Fungi	Aspergillus clavatus NRRL 1	XP_001273036.1	2 E-65	551/601
Eukaryota	Fungi	Botryotinia fuckeliana B05.10	XP_001549389.1	2 E-65	547/601
Eukaryota	Fungi	Penicillium marneffei ATCC 18224	XP_002148263.1	3 E-65	582/601
Bacteria	Proteobacteria	Roseobacter sp. MED193	ZP_01057973.1	3 E-65	527/601
Bacteria	Proteobacteria	Verminephrobacter eiseniae EF01-2	YP_996713.1	3 E-65	527/601
Bacteria	Proteobacteria	Burkholderia phytofirmans PsJN	YP_001895624.1	3 E-65	531/601
Bacteria	Proteobacteria	Roseobacter denitrificans OCh 114	YP_684227.1	4 E-65	527/601
Eukaryota	Fungi	Talaromyces stipitatus ATCC 10500	XP_002482522.1	6 E-65	563/601
Eukaryota	Fungi	Aspergillus niger CBS 513.88	XP_001394546.1	6 E-65	548/601
Bacteria	Proteobacteria	Silicibacter lacuscaerulensis ITI-1157	ZP_05785341.1	7 E-65	525/601
Bacteria	Proteobacteria	Citreicella sp. SE45	ZP_05781295.1	8 E-65	525/601
Bacteria	Proteobacteria	Jannaschia sp. CCS1	YP_511622.1	9 E-65	531/601
Eukaryota	Fungi	Verticillium albo-atrum VaMs.102	EEY22930.1	1 E-64	565/601
Bacteria	Proteobacteria	Roseobacter sp. SK209-2-6	ZP_01754259.1	1 E-64	530/601
Eukaryota	Fungi	Postia placenta Mad-698-R	XP_002475513.1	1 E-64	592/601
Eukaryota	Fungi	Malassezia globosa CBS 7966	XP_001732090.1	1 E-64	574/601
Eukaryota	Fungi	Cryptococcus neoformans var. neoformans	XP_568317.1	1 E-64	594/601
Bacteria	Proteobacteria	Roseobacter sp. AzwK-3b	ZP_01901081.1	2 E-64	525/601
Bacteria	Proteobacteria	Octadecabacter antarcticus 307	ZP_05052326.1	2 E-64	526/601
Bacteria	Proteobacteria	Octadecabacter antarcticus 238	ZP_05067451.1	2 E-64	527/601
Bacteria	Proteobacteria	Roseobacter sp. SK209-2-6	ZP_01754024.1	2 E-64	526/601
Bacteria	Proteobacteria	Roseobacter litoralis Och 149	ZP_02140274.1	3 E-64	531/601
Eukaryota	Fungi	Aspergillus niger CBS 513.88	XP_001395522.1	9 E-64	554/601

Bacteria	Proteobacteria	Burkholderia xenovorans LB400	YP_554605.1	2 E-63	528/601
Bacteria	Proteobacteria	Rhodobacteraceae bacterium KLH11	ZP_05124197.1	3 E-63	536/601
Bacteria	Proteobacteria	Burkholderia sp. 383	YP_372762.1	3 E-63	527/601
Eukaryota	Metazoa	Monodelphis domestica	XP_001368717.1	6 E-63	533/601
Bacteria	Proteobacteria	Verminephrobacter eiseniae EF01-2	YP_999236.1	7 E-63	531/601
Bacteria	Proteobacteria	Chelativorans sp. BNC1	YP_676241.1	8 E-63	522/601
Eukaryota	Fungi	Nectria haematococca mpVI 77-13-4	EEU41487.1	8 E-63	558/601
Bacteria	Proteobacteria	Rhodobacterales bacterium HTCC2255	ZP_01446884.1	9 E-63	527/601
Bacteria	Proteobacteria	Burkholderia multivorans CGD1	ZP_03585612.1	1 E-62	527/601
Eukaryota	Fungi	Verticillium albo-atrum VaMs.102	EEY21549.1	1 E-62	542/601
Eukaryota	Fungi	Pyrenophora tritici-repentis Pt-1C-BFP	XP_001935810.1	2 E-62	565/601
Bacteria	Proteobacteria	Curvibacter putative symbiont of	CBA30511.1	2 E-62	532/601
Bacteria	Proteobacteria	Burkholderia multivorans CGD2M	ZP_03569823.1	2 E-62	527/601
Bacteria	Proteobacteria	Pseudomonas putida	BAD07371.1	2 E-62	522/601
Eukaryota	Fungi	Postia placenta Mad-698-R	XP_002471526.1	3 E-62	582/601
Bacteria	Proteobacteria	Roseovarius nubinhibens ISM	ZP_00960121.1	3 E-62	526/601
Bacteria	Proteobacteria	Legionella drancourtii LLAP12	ZP_05110316.1	3 E-62	534/601
Bacteria	Proteobacteria	Burkholderia cenocepacia J2315	YP_002233786.1	1 E-61	527/601
Bacteria	Proteobacteria	Burkholderia multivorans ATCC 17616	YP_001584499.1	1 E-61	527/601
Eukaryota	Fungi	Postia placenta Mad-698-R	XP_002470068.1	2 E-61	565/601
Bacteria	Proteobacteria	Thalassiobium sp. R2A62	ZP_05342702.1	2 E-61	533/601
Bacteria	Proteobacteria	Ruegeria pomeroyi DSS-3	YP_167582.1	2 E-61	527/601
Bacteria	Actinobacteria	Actinomadura madurae	ABY65992.1	3 E-61	515/601
Eukaryota	Fungi	Coprinopsis cinerea okayama7#130	XP_001840719.1	3 E-61	616/601
Bacteria	Proteobacteria	Cupriavidus taiwanensis	YP_002008190.1	3 E-61	525/601
Bacteria	Proteobacteria	Roseobacter sp. MED193	ZP_01055291.1	3 E-61	532/601
Bacteria	Proteobacteria	Rhodobacterales bacterium HTCC2150	ZP_01740154.1	4 E-61	527/601
Eukaryota	Fungi	Pyrenophora tritici-repentis Pt-1C-BFP	XP_001940604.1	4 E-61	584/601
Bacteria	Proteobacteria	Rhodobacteraceae bacterium KLH11	ZP_05124765.1	4 E-61	530/601
Bacteria	Proteobacteria	Dinoroseobacter shibae DFL 12	YP_001532150.1	5 E-61	527/601
Eukaryota	Fungi	Talaromyces stipitatus ATCC 10500	XP_002339960.1	5 E-61	574/601
Bacteria	Proteobacteria	Burkholderia xenovorans LB400	YP_557446.1	5 E-61	532/601
Bacteria	Proteobacteria	Burkholderia multivorans CGD1	ZP_03583651.1	7 E-61	532/601
Bacteria	Proteobacteria	Marinobacter sp. ELB17	ZP_01736903.1	7 E-61	528/601
Bacteria	Proteobacteria	Shewanella baltica OS155	YP_001049712.1	7 E-61	524/601
Bacteria	Proteobacteria	Burkholderia phytofirmans PsJN	YP_001888124.1	7 E-61	528/601
Bacteria	Proteobacteria	Shewanella baltica OS195	YP_001553786.1	8 E-61	524/601
Eukaryota	Fungi	Aspergillus fumigatus A1163	EDP54987.1	9 E-61	581/601
Bacteria	Proteobacteria	Mesorhizobium loti MAFF303099	NP_102692.1	1 E-60	522/601
Eukaryota	Fungi	Aspergillus fumigatus Af293	XP_755816.1	1 E-60	581/601
Bacteria	Proteobacteria	Roseobacter sp. CCS2	ZP_01749397.1	1 E-60	527/601
Bacteria	Proteobacteria	Pseudovibrio sp. JE062	ZP_05086419.1	1 E-60	519/601
Bacteria	Proteobacteria	Labrenzia aggregata IAM 12614	ZP_01548976.1	2 E-60	534/601
Bacteria	Proteobacteria	Silicibacter lacuscaerulensis ITI-1157	ZP_05784784.1	2 E-60	526/601
Eukaryota	Fungi	Podospora anserina DSM 980	XP_001906345.1	2 E-60	582/601
Bacteria	Actinobacteria	Streptomyces hygroscopicus ATCC 53653	ZP_05521027.1	2 E-60	531/601
Bacteria	Proteobacteria	Marinomonas sp. MED121	ZP_01076637.1	3 E-60	547/601
Eukaryota	Fungi	Malassezia globosa CBS 7966	XP_001732158.1	3 E-60	572/601
Bacteria	Proteobacteria	Bermanella marisrubri	ZP_01305514.1	3 E-60	528/601
Eukaryota	Fungi	Podospora anserina DSM 980	XP_001906627.1	4 E-60	584/601
Bacteria	Proteobacteria	Ruegeria sp. R11	ZP_05090872.1	4 E-60	530/601
Bacteria	Proteobacteria	Roseovarius sp. HTCC2601	ZP_01441735.1	4 E-60	531/601
Eukaryota	Fungi	Coprinopsis cinerea okayama7#130	XP_001836512.1	4 E-60	571/601
Bacteria	Actinobacteria	Thermomonospora curvata DSM 43183	ZP_04031134.1	4 E-60	520/601
Bacteria	Proteobacteria	Marinobacter aquaeolei VT8	YP_961096.1	6 E-60	527/601
Eukaryota	Fungi	Neosartorya fischeri NRRL 181	XP_001265740.1	6 E-60	558/601
Bacteria	Proteobacteria	Bordetella petrii DSM 12804	YP_001633470.1	9 E-60	524/601
Bacteria	Proteobacteria	Burkholderia ambifaria MC40-6	YP_001816113.1	1 E-59	526/601

Eukaryota	Fungi	Sclerotinia sclerotiorum 1980 UF-70	XP_001590695.1	1 E-59	566/601
Bacteria	Proteobacteria	Roseovarius sp. 217	ZP_01035570.1	1 E-59	525/601
Eukaryota	Fungi	Magnaporthe grisea 70-15	XP_367669.2	1 E-59	552/601
Bacteria	Candidatus	Candidatus Pelagibacter sp. HTCC7211	ZP_05068964.1	1 E-59	526/601
Bacteria	Proteobacteria	Pseudomonas syringae pv. tabaci	ZP_05636703.1	1 E-59	526/601
Bacteria	Proteobacteria	Roseovarius sp. TM1035	ZP_01878091.1	1 E-59	525/601
Bacteria	Proteobacteria	Citreicella sp. SE45	ZP_05781490.1	1 E-59	529/601
Bacteria	Proteobacteria	Phaeobacter gallaeciensis 2.10	ZP_02149954.1	2 E-59	533/601
Bacteria	Proteobacteria	Mesorhizobium loti MAFF303099	NP_107484.1	2 E-59	524/601
Bacteria	Proteobacteria	Pseudomonas fluorescens Pf-5	YP_259594.1	2 E-59	526/601
Bacteria	Proteobacteria	Alcanivorax sp. DG881	ZP_05040520.1	2 E-59	526/601
Bacteria	Proteobacteria	Burkholderia ambifaria AMMD	YP_778292.1	2 E-59	526/601
Bacteria	Proteobacteria	Pseudomonas syringae	AAU95210.1	2 E-59	526/601
Bacteria	Proteobacteria	Pseudomonas syringae pv. oryzae	ZP_04590345.1	2 E-59	526/601
Bacteria	Proteobacteria	Azorhizobium caulinodans ORS 571	YP_001526184.1	2 E-59	528/601
Bacteria	Proteobacteria	Pseudoalteromonas haloplanktis TAC125	YP_341748.1	2 E-59	530/601
Bacteria	Proteobacteria	Phaeobacter gallaeciensis BS107	ZP_02145452.1	2 E-59	533/601
Bacteria	Proteobacteria	Roseovarius sp. TM1035	ZP_01878466.1	2 E-59	532/601
Eukaryota	Fungi	Aspergillus flavus NRRL3357	XP_002373928.1	2 E-59	573/601
Bacteria	Proteobacteria	Burkholderia sp. 383	YP_366469.1	3 E-59	523/601
Bacteria	Proteobacteria	Chelativorans sp. BNC1	YP_676287.1	3 E-59	533/601
Bacteria	Proteobacteria	Shewanella piezotolerans WP3	YP_002313077.1	3 E-59	531/601
Bacteria	Proteobacteria	Agrobacterium radiobacter K84	YP_002541437.1	4 E-59	524/601
Bacteria	Proteobacteria	Hyphomonas neptunium ATCC 15444	YP_761348.1	4 E-59	531/601
Bacteria	Proteobacteria	Paracoccus denitrificans PD1222	YP_918038.1	4 E-59	523/601
Bacteria	Proteobacteria	Shewanella sediminis HAW-EB3	YP_001472858.1	5 E-59	526/601
Bacteria	Proteobacteria	Alcanivorax sp. DG881	ZP_05042898.1	5 E-59	529/601
Bacteria	Proteobacteria	Burkholderia phymatum STM815	YP_001862883.1	5 E-59	529/601
Bacteria	Proteobacteria	Bradyrhizobium sp. ORS278	YP_001208258.1	5 E-59	524/601
Eukaryota	Fungi	Sclerotinia sclerotiorum 1980 UF-70	XP_001587168.1	8 E-59	575/601
Bacteria	Proteobacteria	Ralstonia eutropha H16	YP_841363.1	8 E-59	525/601
Eukaryota	Fungi	Phaeosphaeria nodorum SN15	XP_001805358.1	9 E-59	578/601
Eukaryota	Fungi	Magnaporthe grisea 70-15	XP_359722.1	1 E-58	586/601
Bacteria	Proteobacteria	Paracoccus denitrificans PD1222	YP_915505.1	1 E-58	527/601
Eukaryota	Metazoa	Rattus norvegicus	pirJC8009	1 E-58	535/601
Eukaryota	Metazoa	Rattus norvegicus	NP_942026.1	1 E-58	535/601
Bacteria	Proteobacteria	Rhizobium leguminosarum bv. trifolii	YP_002984725.1	1 E-58	530/601
Bacteria	Proteobacteria	Shewanella halifaxensis HAW-EB4	YP_001673285.1	2 E-58	532/601
Bacteria	Proteobacteria	Ruegeria pomeroyi DSS-3	YP_165213.1	2 E-58	534/601
Eukaryota	Fungi	Pleurotus pulmonarius	AAF31169.1	2 E-58	561/601
Eukaryota	Fungi	Laccaria bicolor S238N-H82	XP_001884302.1	2 E-58	561/601
Bacteria	Proteobacteria	marine gamma proteobacterium HTCC2080	ZP_01626020.1	2 E-58	528/601
Eukaryota	Fungi	Ustilago maydis 521	XP_758019.1	2 E-58	578/601
Bacteria	Proteobacteria	Rhizobium leguminosarum bv. viciae	YP_771439.1	2 E-58	530/601
Bacteria	Bacteroidetes	Spirosoma linguale DSM 74	ZP_04491139.1	2 E-58	544/601
Bacteria	Proteobacteria	Pseudovibrio sp. JE062	ZP_05085930.1	2 E-58	534/601
Bacteria	Proteobacteria	Novosphingobium aromaticivorans DSM 12444	YP_001166065.1	3 E-58	527/601
Bacteria	Proteobacteria	Burkholderia sp. 383	YP_373593.1	3 E-58	532/601
Bacteria	Proteobacteria	Shewanella denitrificans OS217	YP_561728.1	3 E-58	525/601
Eukaryota	Fungi	Pleurotus eryngii	AAC72747.1	3 E-58	561/601
Bacteria	Proteobacteria	Verminephrobacter eiseniae EF01-2	YP_997664.1	3 E-58	529/601
Eukaryota	Metazoa	Strongylocentrotus purpuratus	XP_796478.1	4 E-58	532/601
Bacteria	Proteobacteria	Silicibacter lacuscaerulensis ITI-1157	ZP_05785860.1	4 E-58	534/601
Bacteria	Proteobacteria	Roseovarius sp. 217	ZP_01037150.1	4 E-58	530/601
Bacteria	Proteobacteria	Caulobacter crescentus CB15	NP_419761.1	5 E-58	545/601
Bacteria	Proteobacteria	Ruegeria pomeroyi DSS-3	YP_166223.1	5 E-58	524/601
Eukaryota	Fungi	Phaeosphaeria nodorum SN15	XP_001796868.1	6 E-58	586/601
Bacteria	Proteobacteria	Shewanella pealeana ATCC 700345	YP_001500869.1	6 E-58	532/601

Bacteria	Proteobacteria	Rhizobium etli CIAT 652	YP_001985833.1	7 E-58	530/601
Eukaryota	Metazoa	Trichoplax adhaerens	XP_002109312.1	7 E-58	559/601
Eukaryota	Metazoa	Hydra magnipapillata	XP_002156758.1	8 E-58	530/601
Eukaryota	Metazoa	Mus musculus	NP_780552.1	8 E-58	534/601
Eukaryota	Metazoa	Strongylocentrotus purpuratus	XP_792493.1	8 E-58	530/601
Eukaryota	Fungi	Pyrenophora tritici-repentis Pt-1C-BFP	XP_001937272.1	9 E-58	576/601

AFUA_4G00620

Eukaryota	Fungi	Aspergillus fumigatus Af293	XP_746394.1	0.0	453/453
Eukaryota	Fungi	Neosartorya fischeri NRRL 181	XP_001267584.1	0.0	451/453
Eukaryota	Fungi	Aspergillus clavatus NRRL 1	XP_001270519.1	0.0	443/453
Eukaryota	Fungi	Aspergillus terreus NIH2624	XP_001210023.1	0.0	441/453
Eukaryota	Fungi	Aspergillus flavus NRRL3357	XP_002373939.1	1 E-171	439/453
Eukaryota	Fungi	Aspergillus oryzae RIB40	XP_001820464.1	1 E-171	439/453
Eukaryota	Fungi	Aspergillus niger CBS 513.88	XP_001398601.1	1 E-165	434/453
Eukaryota	Fungi	Aspergillus nidulans FGSC A4	CBF83933.1	1 E-165	459/453
Eukaryota	Fungi	Talaromyces stipitatus ATCC 10500	XP_002486572.1	1 E-164	453/453
Eukaryota	Fungi	Penicillium marneffei ATCC 18224	XP_002152839.1	1 E-159	449/453
Eukaryota	Fungi	Penicillium chrysogenum Wisconsin 54-1255	XP_002558151.1	1 E-159	441/453
Eukaryota	Fungi	Aspergillus nidulans FGSC A4	XP_660429.1	1 E-155	397/453
Eukaryota	Fungi	Botryotinia fuckeliana B05.10	XP_001554597.1	1 E-142	430/453
Eukaryota	Fungi	Uncinocarpus reesii 1704	XP_002543276.1	1 E-141	435/453
Eukaryota	Fungi	Coccidioides posadasii C735 delta	EER28519.1	1 E-141	429/453
Eukaryota	Fungi	Coccidioides immitis RS;	XP_001241715.1	1 E-140	428/453
Eukaryota	Fungi	Sclerotinia sclerotiorum 1980 UF-70	XP_001593682.1	1 E-140	430/453
Eukaryota	Fungi	Paracoccidioides brasiliensis Pb03;	EEH21423.1	1 E-138	423/453
Eukaryota	Fungi	Microsporum canis CBS 113480	EEQ29922.1	1 E-135	441/453
Eukaryota	Fungi	Botryotinia fuckeliana B05.10	XP_001551024.1	1 E-132	431/453
Eukaryota	Fungi	Aspergillus niger CBS 513.88	XP_001389977.1	1 E-132	438/453
Eukaryota	Fungi	Aspergillus terreus NIH2624	XP_001209844.1	1 E-131	428/453
Eukaryota	Fungi	Sclerotinia sclerotiorum 1980 UF-70	XP_001586857.1	1 E-131	431/453
Eukaryota	Fungi	Coccidioides posadasii C735 delta	EER29898.1	1 E-131	427/453
Eukaryota	Fungi	Coccidioides immitis RS;	XP_001239061.1	1 E-131	427/453
Eukaryota	Fungi	Pyrenophora tritici-repentis Pt-1C-BFP	XP_001935983.1	1 E-129	434/453
Eukaryota	Fungi	Aspergillus clavatus NRRL 1	XP_001270236.1	1 E-127	400/453
Eukaryota	Fungi	Paracoccidioides brasiliensis Pb18;	EEH48614.1	1 E-127	425/453
Eukaryota	Fungi	Nectria haematococca mpVI 77-13-4	EEU44369.1	1 E-126	428/453
Eukaryota	Fungi	Paracoccidioides brasiliensis Pb01;	EEH33484.1	1 E-126	425/453
Eukaryota	Fungi	Uncinocarpus reesii 1704	XP_002585267.1	1 E-126	417/453
Eukaryota	Fungi	Gibberella zeae PH-1	XP_387192.1	1 E-126	451/453
Eukaryota	Fungi	Paracoccidioides brasiliensis Pb03;	EEH22098.1	1 E-124	416/453
Eukaryota	Fungi	Botryotinia fuckeliana B05.10	XP_001556145.1	1 E-123	430/453
Eukaryota	Fungi	Ajellomyces dermatitidis SLH14081	XP_002620911.1	1 E-122	432/453
Eukaryota	Fungi	Aspergillus clavatus NRRL 1	XP_001273784.1	1 E-122	420/453
Eukaryota	Fungi	Ajellomyces dermatitidis ER-3	EEQ86176.1	1 E-122	430/453
Eukaryota	Fungi	Ajellomyces capsulatus NAm1	XP_001537244.1	1 E-120	421/453
Eukaryota	Fungi	Aspergillus fumigatus A1163	EDP53590.1	1 E-120	386/453
Eukaryota	Fungi	Neosartorya fischeri NRRL 181	XP_001258678.1	1 E-119	418/453
Eukaryota	Fungi	Ajellomyces capsulatus G186AR	EEH09347.1	1 E-119	435/453
Eukaryota	Fungi	Aspergillus fumigatus Af293	XP_748390.1	1 E-119	386/453
Eukaryota	Fungi	Ajellomyces capsulatus H143	EER44919.1	1 E-118	431/453
Eukaryota	Fungi	Microsporum canis CBS 113480	EEQ30249.1	1 E-118	430/453
Eukaryota	Fungi	Magnaporthe grisea 70-15	XP_361541.1	1 E-117	444/453
Eukaryota	Fungi	Kluyveromyces lactis NRRL Y-1140	XP_454297.1	1 E-117	443/453
Eukaryota	Fungi	Ajellomyces dermatitidis ER-3	EEQ91658.1	1 E-117	435/453
Eukaryota	Fungi	Neurospora crassa	CAC10089.2	1 E-117	448/453
Eukaryota	Fungi	Ajellomyces dermatitidis SLH14081	XP_002624579.1	1 E-117	435/453

Eukaryota	Fungi	Nectria haematococca mpVI 77-13-4	EEU47465.1	1 E-116	425/453
Eukaryota	Fungi	Neurospora crassa OR74A	XP_964654.2	1 E-116	422/453
Eukaryota	Fungi	Sclerotinia sclerotiorum 1980 UF-70	XP_001596462.1	1 E-116	430/453
Eukaryota	Fungi	Sclerotinia sclerotiorum 1980 UF-70	XP_001593423.1	1 E-115	439/453
Eukaryota	Fungi	Chaetomium globosum CBS 148.51	XP_001224891.1	1 E-115	446/453
Eukaryota	Fungi	Ajellomyces capsulatus G186AR	EEH03489.1	1 E-115	414/453
Eukaryota	Fungi	Magnaporthe grisea 70-15	XP_001522439.1	1 E-114	448/453
Eukaryota	Fungi	Magnaporthe grisea 70-15	XP_366717.2	1 E-114	448/453
Eukaryota	Fungi	Podospora anserina DSM 980	XP_001911797.1	1 E-114	423/453
Eukaryota	Fungi	Sclerotinia sclerotiorum 1980 UF-70	XP_001584600.1	1 E-113	384/453
Eukaryota	Fungi	Magnaporthe grisea 70-15	XP_368293.2	1 E-112	443/453
Eukaryota	Fungi	Lachancea thermotolerans CBS 6340	XP_002553061.1	1 E-111	434/453
Eukaryota	Fungi	Podospora anserina DSM 980	XP_001907411.1	1 E-111	423/453
Eukaryota	Fungi	Kluyveromyces lactis NRRL Y-1140	XP_456190.1	1 E-110	441/453
Eukaryota	Fungi	Candida tropicalis MYA-3404	XP_002546922.1	1 E-110	436/453
Eukaryota	Fungi	Neurospora crassa	CAD11391.1	1 E-110	427/453
Eukaryota	Fungi	Neurospora crassa OR74A	XP_961253.2	1 E-109	422/453
Eukaryota	Fungi	Saccharomyces cerevisiae	NP_013965.1	1 E-109	442/453
Eukaryota	Fungi	Zygosaccharomyces rouxii CBS 732	XP_002497731.1	1 E-109	447/453
Eukaryota	Fungi	Candida dubliniensis CD36	XP_002418102.1	1 E-109	441/453
Eukaryota	Fungi	Ashbya gossypii ATCC 10895	NP_986077.1	1 E-109	446/453
Eukaryota	Fungi	Lachancea thermotolerans CBS 6340	XP_002553658.1	1 E-108	447/453
Eukaryota	Fungi	Saccharomyces cerevisiae	NP_012878.1	1 E-108	429/453
Eukaryota	Fungi	Paracoccidioides brasiliensis Pb18;	EEH43904.1	1 E-108	378/453
Eukaryota	Fungi	Candida albicans SC5314	XP_719567.1	1 E-108	447/453
Eukaryota	Fungi	Saccharomyces cerevisiae	AAU09756.1	1 E-108	429/453
Eukaryota	Fungi	Ashbya gossypii ATCC 10895	NP_983204.1	1 E-107	443/453
Eukaryota	Fungi	Candida glabrata CBS 138	XP_449567.1	1 E-107	445/453
Eukaryota	Fungi	Lodderomyces elongisporus NRRL YB-4239	XP_001528626.1	1 E-107	448/453
Eukaryota	Fungi	Chaetomium globosum CBS 148.51	XP_001222615.1	1 E-107	392/453
Eukaryota	Fungi	Ashbya gossypii ATCC 10895	NP_983203.1	1 E-106	443/453
Eukaryota	Fungi	Clavispora lusitaniae ATCC 42720	XP_002617444.1	1 E-106	443/453
Eukaryota	Fungi	Ashbya gossypii ATCC 10895	NP_983006.1	1 E-106	430/453
Eukaryota	Fungi	Sclerotinia sclerotiorum 1980 UF-70	XP_001596282.1	1 E-105	417/453
Eukaryota	Fungi	Pichia pastoris GS115	XP_002489792.1	1 E-104	441/453
Eukaryota	Fungi	Pichia stipitis CBS 6054	XP_001382820.1	1 E-104	444/453
Eukaryota	Fungi	Phaeosphaeria nodorum SN15	XP_001805692.1	1 E-104	377/453
Eukaryota	Fungi	Gibberella zeae PH-1	XP_390563.1	1 E-104	448/453
Eukaryota	Fungi	Nectria haematococca mpVI 77-13-4	EEU34130.1	1 E-104	438/453
Eukaryota	Fungi	Verticillium albo-atrum VaMs.102	EEY14538.1	1 E-103	378/453
Eukaryota	Fungi	Yarrowia lipolytica CLIB122	XP_502330.1	1 E-103	455/453
Eukaryota	Fungi	Botryotinia fuckeliana B05.10	XP_001552606.1	1 E-103	389/453
Eukaryota	Fungi	Pichia guilliermondii ATCC 6260	XP_001483001.1	1 E-103	444/453
Eukaryota	Fungi	Vanderwaltozyma polyspora DSM 70294	XP_001644662.1	1 E-102	420/453
Eukaryota	Fungi	Pichia guilliermondii ATCC 6260	EDK40858.2	1 E-102	444/453
Eukaryota	Fungi	Candida albicans WO-1	EEQ45504.1	1 E-102	441/453
Eukaryota	Fungi	Candida albicans SC5314	XP_719646.1	1 E-102	441/453
Eukaryota	Fungi	Candida dubliniensis CD36	XP_002418028.1	1 E-102	441/453
Eukaryota	Fungi	Schizosaccharomyces japonicus yFS275	XP_002175924.1	1 E-101	434/453
Eukaryota	Fungi	Aspergillus terreus NIH2624	XP_001217756.1	1 E-101	367/453
Eukaryota	Fungi	Podospora anserina DSM 980	XP_001905291.1	1 E-101	420/453
Eukaryota	Fungi	Candida glabrata CBS 138	XP_448845.1	1 E-101	414/453
Eukaryota	Fungi	Pichia guilliermondii ATCC 6260	XP_001484975.1	1 E-100	441/453
Eukaryota	Fungi	Pichia guilliermondii ATCC 6260	EDK38606.2	1 E-100	438/453
Eukaryota	Fungi	Zygosaccharomyces rouxii CBS 732	XP_002499200.1	3 E-99	434/453
Eukaryota	Fungi	Ajellomyces capsulatus NAm1	XP_001539610.1	3 E-99	386/453
Eukaryota	Fungi	Gibberella zeae PH-1	XP_384760.1	3 E-99	431/453
Eukaryota	Fungi	Neosartorya fischeri NRRL 181	XP_001265477.1	4 E-99	432/453

Eukaryota	Fungi	Aspergillus fumigatus Af293	XP_749923.1	6 E-99	432/453
Eukaryota	Fungi	Ashbya gossypii ATCC 10895	NP_983202.1	1 E-98	414/453
Eukaryota	Fungi	Neurospora crassa OR74A	XP_961116.1	1 E-98	478/453
Eukaryota	Fungi	Candida tropicalis MYA-3404	XP_002546817.1	9 E-98	440/453
Eukaryota	Fungi	Ajellomyces capsulatus H143	EER43882.1	1 E-97	386/453
Eukaryota	Fungi	Schizosaccharomyces japonicus yFS275	XP_002173762.1	1 E-97	389/453
Eukaryota	Fungi	Yarrowia lipolytica CLIB122	XP_505591.1	2 E-97	454/453
Eukaryota	Fungi	Debaryomyces hansenii CBS767	XP_456419.1	3 E-97	434/453
Eukaryota	Fungi	Aspergillus flavus NRRL3357	XP_002379836.1	4 E-97	452/453
Eukaryota	Fungi	Botryotinia fuckeliana B05.10	XP_001550175.1	5 E-97	376/453
Eukaryota	Fungi	Aspergillus clavatus NRRL 1	XP_001269969.1	1 E-96	434/453
Eukaryota	Fungi	Aspergillus oryzae RIB40	XP_001821610.1	1 E-96	460/453
Eukaryota	Fungi	Yarrowia lipolytica CLIB122	XP_500844.1	4 E-96	437/453
Eukaryota	Fungi	Nectria haematococca mpVI 77-13-4	EEU42734.1	2 E-95	431/453
Eukaryota	Fungi	Chaetomium globosum CBS 148.51	XP_001228681.1	4 E-95	430/453
Eukaryota	Fungi	Pichia stipitis CBS 6054	XP_001382781.2	4 E-95	447/453
Eukaryota	Fungi	Schizosaccharomyces pombe	NP_587853.1	5 E-95	397/453
Eukaryota	Fungi	Neurospora crassa OR74A	XP_959670.1	5 E-95	425/453
Eukaryota	Fungi	Neurospora crassa OR74A	XP_962581.2	1 E-94	435/453
Eukaryota	Fungi	Pichia pastoris GS115	XP_002490358.1	2 E-94	432/453
Eukaryota	Fungi	Aspergillus flavus NRRL3357	XP_002373097.1	4 E-94	435/453
Eukaryota	Fungi	Podospora anserina DSM 980	XP_001904041.1	1 E-93	433/453
Eukaryota	Fungi	Aspergillus niger CBS 513.88	XP_001389075.1	4 E-93	426/453
Eukaryota	Fungi	Lodderomyces elongisporus NRRL YB-4239	XP_001528696.1	5 E-93	442/453
Eukaryota	Fungi	Aspergillus oryzae RIB40	XP_001817932.1	8 E-93	435/453
Eukaryota	Fungi	Aspergillus terreus NIH2624	XP_001214095.1	9 E-93	451/453
Eukaryota	Fungi	Debaryomyces hansenii CBS767	XP_461921.1	1 E-92	438/453
Eukaryota	Fungi	Penicillium marneffei ATCC 18224	XP_002149887.1	3 E-92	452/453
Eukaryota	Fungi	Neurospora crassa OR74A	XP_959431.1	1 E-91	439/453
Eukaryota	Fungi	Yarrowia lipolytica CLIB122	XP_505725.1	3 E-91	446/453
Eukaryota	Fungi	Aspergillus niger CBS 513.88	XP_001398107.1	3 E-91	448/453
Eukaryota	Fungi	Clavispora lusitaniae ATCC 42720	XP_002619165.1	1 E-90	438/453
Eukaryota	Fungi	Aspergillus oryzae RIB40	XP_001827140.1	7 E-90	446/453
Eukaryota	Fungi	Talaromyces stipitatus ATCC 10500	XP_002484373.1	7 E-90	454/453
Eukaryota	Fungi	Aspergillus clavatus NRRL 1	XP_001273708.1	2 E-89	429/453
Eukaryota	Fungi	Magnaporthe grisea 70-15	XP_362320.2	3 E-89	456/453
Eukaryota	Fungi	Neosartorya fischeri NRRL 181	XP_001258856.1	8 E-89	439/453
Eukaryota	Fungi	Podospora anserina DSM 980	XP_001906578.1	1 E-88	382/453
Eukaryota	Fungi	Penicillium chrysogenum Wisconsin 54-1255	XP_002558147.1	2 E-88	449/453
Eukaryota	Fungi	Aspergillus fumigatus Af293	XP_748524.1	8 E-88	429/453
Eukaryota	Fungi	Penicillium chrysogenum Wisconsin 54-1255	XP_002561268.1	1 E-87	433/453
Eukaryota	Fungi	Phaeosphaeria nodorum SN15	XP_001796694.1	1 E-87	388/453
Eukaryota	Fungi	Aspergillus oryzae RIB40	XP_001817610.1	3 E-87	438/453
Eukaryota	Fungi	Pyrenophora tritici-repentis Pt-1C-BFP	XP_001932831.1	4 E-87	391/453
Eukaryota	Fungi	Pyrenophora tritici-repentis Pt-1C-BFP	XP_001932510.1	5 E-87	393/453
Eukaryota	Fungi	Penicillium marneffei ATCC 18224	XP_002145673.1	1 E-86	432/453
Eukaryota	Fungi	Sclerotinia sclerotiorum 1980 UF-70	XP_001587586.1	2 E-86	376/453
Eukaryota	Fungi	Aspergillus oryzae RIB40	XP_001826713.1	2 E-86	421/453
Eukaryota	Fungi	Neurospora crassa OR74A	XP_957931.2	2 E-86	381/453
Eukaryota	Fungi	Penicillium chrysogenum Wisconsin 54-1255	XP_002560638.1	2 E-86	424/453
Eukaryota	Fungi	Talaromyces stipitatus ATCC 10500	XP_002477877.1	4 E-86	430/453
Eukaryota	Fungi	Aspergillus flavus NRRL3357	XP_002372730.1	5 E-86	422/453
Eukaryota	Fungi	Penicillium chrysogenum Wisconsin 54-1255	XP_002566141.1	1 E-84	444/453
Eukaryota	Fungi	Aspergillus flavus NRRL3357	XP_002385368.1	1 E-84	417/453
Eukaryota	Fungi	Aspergillus terreus NIH2624	XP_001217670.1	3 E-84	419/453
Eukaryota	Fungi	Chaetomium globosum CBS 148.51	XP_001224711.1	4 E-84	381/453
Eukaryota	Fungi	Nectria haematococca mpVI 77-13-4	EEU40986.1	5 E-84	368/453
Eukaryota	Fungi	Schizosaccharomyces japonicus yFS275	XP_002175923.1	1 E-83	373/453

Eukaryota	Fungi	Schizosaccharomyces japonicus yFS275	XP_002171597.1	2 E-83	382/453
Eukaryota	Fungi	Aspergillus nidulans FGSC A4	XP_681690.1	1 E-82	438/453
Eukaryota	Fungi	Schizosaccharomyces pombe	NP_595076.1	2 E-82	414/453
Eukaryota	Fungi	Chaetomium globosum CBS 148.51	XP_001223139.1	3 E-82	372/453
Eukaryota	Fungi	Aspergillus terreus NIH2624	XP_001208717.1	6 E-82	412/453
Eukaryota	Fungi	Magnaporthe grisea 70-15	XP_367176.2	1 E-81	398/453
Eukaryota	Fungi	Aspergillus oryzae RIB40	XP_001827674.1	7 E-81	427/453
Eukaryota	Fungi	Paracoccidioides brasiliensis Pb18;	EEH45344.1	1 E-80	410/453
Eukaryota	Fungi	Aspergillus niger CBS 513.88	XP_001399407.1	1 E-80	449/453
Eukaryota	Fungi	Verticillium albo-atrum VaMs.102	EEY23504.1	1 E-80	410/453
Eukaryota	Fungi	Chaetomium globosum CBS 148.51	XP_001226209.1	2 E-80	419/453
Eukaryota	Fungi	Verticillium albo-atrum VaMs.102	EEY14877.1	3 E-80	368/453
Eukaryota	Fungi	Gibberella zeae PH-1	XP_389824.1	4 E-80	379/453
Eukaryota	Fungi	Paracoccidioides brasiliensis Pb03;	EEH20743.1	4 E-80	410/453
Eukaryota	Fungi	Paracoccidioides brasiliensis;	AAP74717.2	6 E-80	409/453
Eukaryota	Fungi	Phaeosphaeria nodorum SN15	XP_001793438.1	8 E-80	402/453
Eukaryota	Fungi	Magnaporthe grisea	AAX07733.1	3 E-79	391/453
Eukaryota	Fungi	Aspergillus flavus NRRL3357	XP_002382495.1	3 E-79	426/453
Eukaryota	Fungi	Podospora anserina DSM 980	XP_001910691.1	4 E-79	394/453
Eukaryota	Fungi	Verticillium albo-atrum VaMs.102	EEY17943.1	4 E-79	432/453
Eukaryota	Fungi	Paracoccidioides brasiliensis Pb01;	EEH36542.1	2 E-78	439/453
Eukaryota	Fungi	Neurospora crassa	CAD11780.1	3 E-78	367/453
Eukaryota	Fungi	Aspergillus oryzae RIB40	XP_001822403.1	5 E-78	419/453
Eukaryota	Fungi	Aspergillus niger CBS 513.88	XP_001400977.1	6 E-77	432/453
Eukaryota	Fungi	Aspergillus oryzae RIB40	XP_001827585.1	2 E-76	443/453
Eukaryota	Fungi	Aspergillus niger CBS 513.88	XP_001390864.1	3 E-75	395/453
Eukaryota	Fungi	Aspergillus fumigatus Af293	XP_748356.1	1 E-74	429/453
Eukaryota	Fungi	Aspergillus flavus NRRL3357	XP_002384814.1	2 E-74	409/453
Eukaryota	Fungi	Aspergillus clavatus NRRL 1	XP_001273860.1	3 E-74	428/453
Eukaryota	Fungi	Aspergillus nidulans FGSC A4	XP_660653.1	1 E-73	416/453
Eukaryota	Fungi	Neosartorya fischeri NRRL 181	XP_001258640.1	1 E-73	429/453
Eukaryota	Fungi	Yarrowia lipolytica CLIB122	XP_500286.1	1 E-71	367/453
Eukaryota	Fungi	Schizosaccharomyces pombe	NP_595075.1	1 E-70	440/453
Eukaryota	Fungi	Aspergillus nidulans FGSC A4	XP_657997.1	9 E-69	437/453
Eukaryota	Fungi	Phaeosphaeria nodorum SN15	XP_001799814.1	2 E-68	376/453
Eukaryota	Fungi	Aspergillus terreus NIH2624	XP_001217238.1	4 E-68	417/453
Eukaryota	Fungi	Nectria haematococca mpVI 77-13-4	EEU36298.1	4 E-64	376/453
Eukaryota	Fungi	Penicillium chrysogenum Wisconsin 54-1255	XP_002568269.1	2 E-62	455/453
Eukaryota	Fungi	Aspergillus fumigatus Af293	XP_746458.1	2 E-60	394/453
Eukaryota	Fungi	Aspergillus fumigatus A1163	EDP47441.1	5 E-60	394/453
Eukaryota	Fungi	Chaetomium globosum CBS 148.51	XP_001223543.1	7 E-52	389/453
Eukaryota	Fungi	Aspergillus niger CBS 513.88	XP_001394131.1	1 E-27	378/453

AFUA_4G03510

Eukaryota	Fungi	Aspergillus fumigatus Af293	XP_746538.1	0.0	535/535
Eukaryota	Fungi	Neosartorya fischeri NRRL 181	XP_001262458.1	0.0	533/535
Eukaryota	Fungi	Aspergillus clavatus NRRL 1	XP_001274876.1	0.0	539/535
Eukaryota	Fungi	Aspergillus terreus NIH2624	XP_001211335.1	0.0	537/535
Eukaryota	Fungi	Aspergillus flavus NRRL3357	XP_002377507.1	0.0	536/535
Eukaryota	Fungi	Aspergillus oryzae RIB40	XP_001825905.1	0.0	536/535
Eukaryota	Fungi	Aspergillus niger CBS 513.88	XP_001400885.1	0.0	536/535
Eukaryota	Fungi	Aspergillus nidulans FGSC A4	XP_664754.1	0.0	519/535
Eukaryota	Fungi	Penicillium chrysogenum Wisconsin 54-1255	XP_002565635.1	0.0	533/535
Eukaryota	Fungi	Penicillium chrysogenum Wisconsin 54-1255	XP_002558271.1	0.0	485/535
Eukaryota	Fungi	Penicillium marneffei ATCC 18224	XP_002153477.1	0.0	531/535
Eukaryota	Fungi	Talaromyces stipitatus ATCC 10500	XP_002487948.1	0.0	526/535
Eukaryota	Fungi	Nectria haematococca mpVI 77-13-4	EEU40016.1	0.0	538/535

Eukaryota	Fungi	Gibberella zeae PH-1	XP_382448.1	0.0	523/535
Eukaryota	Fungi	Nectria haematococca mpVI 77-13-4	EEU34659.1	0.0	511/535
Eukaryota	Fungi	Nectria haematococca mpVI 77-13-4	EEU44508.1	0.0	519/535
Eukaryota	Fungi	Magnaporthe grisea 70-15	XP_366604.1	0.0	511/535
Eukaryota	Fungi	Magnaporthe grisea 70-15	XP_001522306.1	0.0	502/535
Eukaryota	Fungi	Gibberella zeae PH-1	XP_386035.1	0.0	517/535
Eukaryota	Fungi	Verticillium albo-atrum VaMs.102	EEY14162.1	1 E-180	504/535
Eukaryota	Fungi	Gibberella zeae PH-1	XP_388523.1	1 E-80	492/535
Eukaryota	Fungi	Nectria haematococca mpVI 77-13-4	EEU46773.1	4 E-77	493/535
Eukaryota	Fungi	Penicillium chrysogenum Wisconsin 54-1255	XP_002563153.1	9 E-76	485/535
Eukaryota	Fungi	Phaeosphaeria nodorum SN15	XP_001792956.1	3 E-74	510/535
Eukaryota	Fungi	Penicillium chrysogenum Wisconsin 54-1255	XP_002563838.1	8 E-74	477/535
Eukaryota	Fungi	Talaromyces stipitatus ATCC 10500	XP_002487952.1	1 E-73	506/535
Eukaryota	Fungi	Botryotinia fuckeliana B05.10	XP_001552432.1	4 E-73	472/535
Eukaryota	Fungi	Cryptococcus neoformans var. neoformans	XP_775382.1	6 E-73	482/535
Eukaryota	Fungi	Cryptococcus neoformans var. neoformans	XP_570845.1	8 E-73	482/535
Eukaryota	Fungi	Gibberella zeae PH-1	XP_382166.1	8 E-71	517/535
Eukaryota	Fungi	Aspergillus fumigatus Af293	XP_754815.1	1 E-69	471/535
Eukaryota	Fungi	Penicillium marneffei ATCC 18224	XP_002146484.1	2 E-69	490/535
Eukaryota	Fungi	Aspergillus niger CBS 513.88	XP_001393569.1	4 E-69	479/535
Eukaryota	Fungi	Verticillium albo-atrum VaMs.102	EEY20751.1	8 E-68	462/535
Eukaryota	Fungi	Neosartorya fischeri NRRL 181	XP_001263641.1	1 E-67	462/535
Eukaryota	Fungi	Pyrenophora tritici-repentis Pt-1C-BFP	XP_001940474.1	1 E-67	501/535
Eukaryota	Fungi	Nectria haematococca mpVI 77-13-4	EEU45709.1	9 E-67	472/535
Eukaryota	Fungi	Aspergillus terreus NIH2624	XP_001209814.1	3 E-66	468/535
Eukaryota	Fungi	Emericella nidulans	CAB43936.1	5 E-66	484/535
Eukaryota	Fungi	Nectria haematococca mpVI 77-13-4	EEU48319.1	8 E-66	490/535
Eukaryota	Fungi	Aspergillus niger CBS 513.88	XP_001393813.1	9 E-66	502/535
Eukaryota	Fungi	Nectria haematococca mpVI 77-13-4	EEU40643.1	7 E-65	470/535
Eukaryota	Fungi	Aspergillus nidulans FGSC A4	XP_660566.1	8 E-65	487/535
Eukaryota	Fungi	Nectria haematococca mpVI 77-13-4	EEU43613.1	2 E-64	488/535
Eukaryota	Fungi	Pichia stipitis CBS 6054	XP_001382779.1	1 E-63	510/535
Eukaryota	Fungi	Penicillium marneffei ATCC 18224	XP_002153473.1	8 E-62	449/535
Eukaryota	Fungi	Neosartorya fischeri NRRL 181	XP_001261865.1	2 E-61	489/535
Eukaryota	Fungi	Aspergillus fumigatus Af293	XP_747459.1	3 E-61	481/535
Eukaryota	Fungi	Nectria haematococca mpVI 77-13-4	EEU37164.1	1 E-60	471/535
Eukaryota	Fungi	Coccidioides posadasii C735 delta	EER29905.1	2 E-60	499/535
Eukaryota	Fungi	Neosartorya fischeri NRRL 181	XP_001262187.1	2 E-60	470/535
Eukaryota	Fungi	Sclerotinia sclerotiorum 1980 UF-70	XP_001584635.1	2 E-60	436/535
Eukaryota	Fungi	Coccidioides immitis RS;	XP_001239074.1	2 E-60	499/535
Eukaryota	Fungi	Paracoccidioides brasiliensis Pb18;	EEH48626.1	2 E-60	519/535
Eukaryota	Fungi	Ajellomyces capsulatus NAm1	XP_001539619.1	4 E-60	496/535
Eukaryota	Fungi	Paracoccidioides brasiliensis Pb03;	EEH22110.1	4 E-60	526/535
Eukaryota	Fungi	Paracoccidioides brasiliensis Pb01;	EEH33496.1	1 E-59	499/535
Eukaryota	Fungi	Verticillium albo-atrum VaMs.102	EEY23768.1	1 E-59	498/535
Eukaryota	Fungi	Magnaporthe grisea 70-15	XP_363086.1	1 E-59	480/535
Eukaryota	Fungi	Aspergillus fumigatus Af293	XP_746988.2	1 E-59	481/535
Eukaryota	Fungi	Ajellomyces capsulatus G186AR	EEH09335.1	2 E-59	489/535
Eukaryota	Fungi	Gibberella zeae PH-1	XP_391498.1	2 E-59	500/535
Eukaryota	Fungi	Nectria haematococca mpVI 77-13-4	EEU34979.1	2 E-59	490/535
Eukaryota	Fungi	Cryptococcus neoformans var. neoformans	XP_773568.1	3 E-59	476/535
Eukaryota	Fungi	Neosartorya fischeri NRRL 181	XP_001266237.1	1 E-58	515/535
Eukaryota	Fungi	Ustilago maydis 521	XP_758293.1	1 E-58	534/535
Eukaryota	Fungi	Nectria haematococca mpVI 77-13-4	EEU37454.1	1 E-58	472/535
Eukaryota	Fungi	Aspergillus oryzae RIB40	XP_001822657.1	4 E-58	508/535
Eukaryota	Fungi	Ajellomyces dermatitidis SLH14081	XP_002624567.1	5 E-58	486/535
Eukaryota	Fungi	Aspergillus oryzae RIB40	XP_001823364.1	7 E-58	484/535
Eukaryota	Fungi	Pichia stipitis CBS 6054	XP_001382810.2	1 E-57	493/535

Eukaryota	Fungi	Aspergillus niger CBS 513.88	XP_001397513.1	2 E-57	469/535
Eukaryota	Fungi	Gibberella zeae PH-1	XP_385055.1	9 E-57	505/535
Eukaryota	Fungi	Yarrowia lipolytica CLIB122	XP_500191.1	2 E-56	526/535
Eukaryota	Fungi	Aspergillus niger CBS 513.88	XP_001400084.1	2 E-56	466/535
Eukaryota	Fungi	Cryptococcus neoformans var. neoformans	XP_568040.1	2 E-56	465/535
Eukaryota	Fungi	Vanderwaltozyma polyspora DSM 70294	XP_001645594.1	3 E-56	500/535
Eukaryota	Fungi	Talaromyces stipitatus ATCC 10500	XP_002478423.1	3 E-56	495/535
Eukaryota	Fungi	Aspergillus niger CBS 513.88	XP_001396729.1	3 E-56	500/535
Eukaryota	Fungi	Penicillium marneffei ATCC 18224	XP_002146128.1	3 E-56	484/535
Eukaryota	Fungi	Pichia guilliermondii ATCC 6260	EDK38604.2	6 E-56	496/535
Eukaryota	Fungi	Pichia guilliermondii ATCC 6260	XP_001484973.1	8 E-56	496/535
Eukaryota	Fungi	Lodderomyces elongisporus NRRL YB-4239	XP_001528698.1	9 E-56	514/535
Eukaryota	Fungi	Aspergillus oryzae RIB40	XP_001821192.1	9 E-56	486/535
Eukaryota	Fungi	Neosartorya fischeri NRRL 181	XP_001263314.1	1 E-55	503/535
Eukaryota	Fungi	Gibberella zeae PH-1	XP_391287.1	2 E-55	481/535
Eukaryota	Fungi	Aspergillus flavus NRRL3357	XP_002378749.1	3 E-55	506/535
Eukaryota	Fungi	Aspergillus clavatus NRRL 1	XP_001270188.1	3 E-55	512/535
Eukaryota	Fungi	Penicillium chrysogenum Wisconsin 54-1255	XP_002562141.1	5 E-55	488/535
Eukaryota	Fungi	Candida glabrata CBS 138	XP_446941.1	5 E-55	504/535
Eukaryota	Fungi	Clavispora lusitaniae ATCC 42720	XP_002618289.1	9 E-55	488/535
Eukaryota	Fungi	Neosartorya fischeri NRRL 181	XP_001262041.1	1 E-54	510/535
Eukaryota	Fungi	Aspergillus fumigatus A1163	EDP52602.1	2 E-54	504/535
Eukaryota	Fungi	Aspergillus fumigatus Af293	XP_754470.2	3 E-54	523/535
Eukaryota	Fungi	Aspergillus clavatus NRRL 1	XP_001269019.1	3 E-54	507/535
Eukaryota	Fungi	Clavispora lusitaniae ATCC 42720	XP_002619163.1	6 E-54	525/535
Eukaryota	Fungi	Penicillium chrysogenum Wisconsin 54-1255	XP_002560090.1	7 E-54	489/535
Eukaryota	Fungi	Debaryomyces hansenii CBS767	XP_458651.1	9 E-54	491/535
Eukaryota	Fungi	Neosartorya fischeri NRRL 181	XP_001267135.1	1 E-53	512/535
Eukaryota	Fungi	Neosartorya fischeri NRRL 181	XP_001264529.1	1 E-53	500/535
Eukaryota	Fungi	Nectria haematococca mpVI 77-13-4	EEU43648.1	2 E-53	501/535
Eukaryota	Fungi	Talaromyces stipitatus ATCC 10500	XP_002487445.1	2 E-53	491/535
Eukaryota	Fungi	Talaromyces stipitatus ATCC 10500	XP_002478604.1	2 E-53	503/535
Eukaryota	Fungi	Microsporum canis CBS 113480	EEQ30267.1	2 E-53	492/535
Eukaryota	Fungi	Aspergillus fumigatus Af293	XP_752599.1	2 E-53	500/535
Eukaryota	Fungi	Aspergillus terreus NIH2624	XP_001217892.1	3 E-53	467/535
Eukaryota	Fungi	Penicillium chrysogenum Wisconsin 54-1255	XP_002559695.1	4 E-53	511/535
Eukaryota	Fungi	Aspergillus terreus NIH2624	XP_001217215.1	4 E-53	460/535
Eukaryota	Fungi	Pyrenophora tritici-repentis Pt-1C-BFP	XP_001933856.1	5 E-53	492/535
Eukaryota	Fungi	Pyrenophora tritici-repentis Pt-1C-BFP	XP_001932370.1	1 E-52	494/535
Eukaryota	Fungi	Aspergillus niger CBS 513.88	XP_001392611.1	1 E-52	484/535
Eukaryota	Fungi	Lachancea thermotolerans CBS 6340	XP_002556074.1	1 E-52	493/535
Eukaryota	Fungi	Talaromyces stipitatus ATCC 10500	XP_002341513.1	2 E-52	481/535
Eukaryota	Fungi	Aspergillus flavus NRRL3357	XP_002377501.1	2 E-52	457/535
Eukaryota	Fungi	Aspergillus oryzae RIB40	XP_001825899.1	3 E-52	457/535
Eukaryota	Fungi	Neurospora crassa OR74A	XP_960036.2	3 E-52	503/535
Eukaryota	Fungi	Candida tropicalis MYA-3404	XP_002546820.1	3 E-52	488/535
Eukaryota	Fungi	Aspergillus nidulans FGSC A4	XP_681995.1	5 E-52	469/535
Eukaryota	Fungi	Phaeosphaeria nodorum SN15	XP_001792257.1	5 E-52	483/535
Eukaryota	Fungi	Microsporum canis CBS 113480	EEQ35748.1	6 E-52	433/535
Eukaryota	Fungi	Neosartorya fischeri NRRL 181	XP_001262162.1	8 E-52	466/535
Eukaryota	Fungi	Aspergillus fumigatus	CAD27309.1	1 E-51	502/535
Eukaryota	Fungi	Coccidioides posadasii C735 delta	EER27950.1	1 E-51	487/535
Eukaryota	Fungi	Aspergillus nidulans FGSC A4	XP_658665.1	1 E-51	517/535
Eukaryota	Fungi	Aspergillus fumigatus Af293	XP_747411.1	2 E-51	460/535
Eukaryota	Fungi	Sclerotinia sclerotiorum 1980 UF-70	XP_001592589.1	3 E-51	495/535
Eukaryota	Fungi	Sclerotinia sclerotiorum 1980 UF-70	XP_001592106.1	3 E-51	517/535
Eukaryota	Fungi	Penicillium marneffei ATCC 18224	XP_002152422.1	3 E-51	481/535
Eukaryota	Fungi	Microsporum canis CBS 113480	EEQ28373.1	4 E-51	492/535

Eukaryota	Fungi	Penicillium marneffei ATCC 18224	XP_002151053.1	5 E-51	491/535
Eukaryota	Fungi	Pyrenophora tritici-repentis Pt-1C-BFP	XP_001935688.1	5 E-51	505/535
Eukaryota	Fungi	Candida tropicalis MYA-3404	XP_002550327.1	6 E-51	492/535
Eukaryota	Fungi	Pichia pastoris GS115	XP_002492739.1	7 E-51	514/535
Eukaryota	Fungi	Gibberella zeae PH-1	XP_386649.1	7 E-51	501/535
Eukaryota	Fungi	Aspergillus oryzae RIB40	XP_001823267.1	8 E-51	496/535
Eukaryota	Fungi	Aspergillus terreus NIH2624	XP_001214472.1	8 E-51	515/535
Eukaryota	Fungi	Aspergillus oryzae RIB40	XP_001818837.1	1 E-50	516/535
Eukaryota	Fungi	Candida albicans SC5314	XP_719643.1	1 E-50	516/535
Eukaryota	Fungi	Ashbya gossypii ATCC 10895	NP_985467.1	2 E-50	494/535
Eukaryota	Fungi	Magnaporthe grisea 70-15	XP_363459.2	2 E-50	490/535
Eukaryota	Fungi	Penicillium chrysogenum Wisconsin 54-1255	XP_002568422.1	2 E-50	522/535
Eukaryota	Fungi	Aspergillus clavatus NRRL 1	XP_001271782.1	2 E-50	507/535
Eukaryota	Fungi	Ajellomyces dermatitidis SLH14081	XP_002626402.1	2 E-50	488/535
Eukaryota	Fungi	Saccharomyces cerevisiae	NP_011438.1	3 E-50	489/535
Eukaryota	Fungi	Penicillium marneffei ATCC 18224	XP_002146292.1	4 E-50	491/535
Eukaryota	Fungi	Verticillium albo-atrum VaMs.102	EEY23055.1	4 E-50	461/535
Eukaryota	Fungi	Kluyveromyces lactis NRRL Y-1140	XP_452894.1	5 E-50	502/535
Eukaryota	Fungi	Pichia guilliermondii ATCC 6260	EDK40872.2	5 E-50	480/535
Eukaryota	Fungi	Magnaporthe grisea 70-15	XP_001402594.1	6 E-50	519/535
Eukaryota	Fungi	Aspergillus niger CBS 513.88	XP_001399966.1	1 E-49	492/535
Eukaryota	Fungi	Neosartorya fischeri NRRL 181	XP_001265698.1	1 E-49	493/535
Eukaryota	Fungi	Coccidioides immitis RS;	XP_001244747.1	1 E-49	511/535
Eukaryota	Fungi	Aspergillus fumigatus Af293	XP_749273.2	1 E-49	497/535
Eukaryota	Fungi	Pichia guilliermondii ATCC 6260	XP_001483015.1	1 E-49	480/535
Eukaryota	Fungi	Aspergillus fumigatus Af293	XP_751937.1	1 E-49	511/535
Eukaryota	Fungi	Aspergillus niger CBS 513.88	XP_001398088.1	1 E-49	470/535
Eukaryota	Fungi	Aspergillus flavus NRRL3357	XP_002379882.1	1 E-49	500/535
Eukaryota	Fungi	Ajellomyces capsulatus H143	EER39658.1	2 E-49	500/535
Eukaryota	Fungi	Aspergillus oryzae RIB40	XP_001816990.1	2 E-49	478/535
Eukaryota	Fungi	Nectria haematococca mpVI 77-13-4	EEU39941.1	2 E-49	498/535
Eukaryota	Fungi	Paracoccidioides brasiliensis Pb01;	EEH41502.1	2 E-49	480/535
Eukaryota	Fungi	Aspergillus flavus NRRL3357	XP_002377532.1	2 E-49	450/535
Eukaryota	Fungi	Aspergillus oryzae RIB40	XP_001821570.1	2 E-49	494/535
Eukaryota	Fungi	Aspergillus nidulans FGSC A4	XP_680661.1	2 E-49	480/535
Eukaryota	Fungi	Uncinocarpus reesii 1704	XP_002544083.1	3 E-49	472/535
Eukaryota	Fungi	Aspergillus nidulans FGSC A4	XP_868823.1	3 E-49	519/535
Eukaryota	Fungi	Coccidioides posadasii C735 delta	EER26072.1	3 E-49	511/535
Eukaryota	Fungi	Aspergillus nidulans FGSC A4	XP_682259.1	3 E-49	498/535
Eukaryota	Fungi	Candida tropicalis MYA-3404	XP_002546816.1	4 E-49	505/535
Eukaryota	Fungi	Aspergillus niger CBS 513.88	XP_001393652.1	5 E-49	502/535
Eukaryota	Fungi	Zygosaccharomyces rouxii CBS 732	XP_002498370.1	7 E-49	510/535
Eukaryota	Fungi	Aspergillus clavatus NRRL 1	XP_001273078.1	7 E-49	485/535
Eukaryota	Fungi	Podospora anserina DSM 980	XP_001905552.1	7 E-49	475/535
Eukaryota	Fungi	Ajellomyces capsulatus G186AR	EEH08267.1	7 E-49	492/535
Eukaryota	Fungi	Penicillium chrysogenum Wisconsin 54-1255	XP_002569244.1	8 E-49	485/535
Eukaryota	Fungi	Neosartorya fischeri NRRL 181	XP_001262668.1	8 E-49	495/535
Eukaryota	Fungi	Aspergillus flavus NRRL3357	XP_002383071.1	1 E-48	478/535
Eukaryota	Fungi	Coccidioides posadasii C735 delta	EER23230.1	1 E-48	498/535
Eukaryota	Fungi	Lodderomyces elongisporus NRRL YB-4239	XP_001528630.1	1 E-48	480/535
Eukaryota	Fungi	Aspergillus clavatus NRRL 1	XP_001269027.1	1 E-48	495/535
Eukaryota	Fungi	Sclerotinia sclerotiorum 1980 UF-70	XP_001591233.1	2 E-48	452/535
Eukaryota	Fungi	Aspergillus niger CBS 513.88	XP_001397505.1	2 E-48	486/535
Eukaryota	Fungi	Aspergillus flavus NRRL3357	XP_002378626.1	2 E-48	508/535
Eukaryota	Fungi	Penicillium chrysogenum Wisconsin 54-1255	XP_002558381.1	2 E-48	498/535
Eukaryota	Fungi	Uncinocarpus reesii 1704	XP_002583200.1	2 E-48	499/535
Eukaryota	Fungi	Penicillium chrysogenum Wisconsin 54-1255	XP_002560202.1	3 E-48	506/535
Eukaryota	Fungi	Aspergillus flavus NRRL3357	XP_002372816.1	3 E-48	528/535

Eukaryota	Fungi	Gibberella zeae PH-1	XP_384848.1	5 E-48	493/535
Eukaryota	Fungi	Pyrenophora tritici-repentis Pt-1C-BFP	XP_001939419.1	5 E-48	500/535
Eukaryota	Fungi	Aspergillus clavatus NRRL 1	XP_001271150.1	6 E-48	494/535
Eukaryota	Fungi	Nectria haematococca mpVI 77-13-4	EEU39429.1	8 E-48	488/535
Eukaryota	Fungi	Laccaria bicolor S238N-H82	XP_001886817.1	1 E-47	498/535
Eukaryota	Fungi	Ustilago maydis 521	XP_758938.1	1 E-47	528/535
Eukaryota	Fungi	Uncinocarpus reesii 1704	XP_002584932.1	2 E-47	516/535
Eukaryota	Fungi	Aspergillus flavus NRRL3357	XP_002374116.1	2 E-47	484/535
Eukaryota	Fungi	Aspergillus fumigatus Af293	XP_747297.1	2 E-47	460/535
Eukaryota	Fungi	Aspergillus flavus NRRL3357	XP_002380841.1	2 E-47	457/535
Eukaryota	Fungi	Aspergillus oryzae RIB40	XP_001820297.1	2 E-47	484/535
Eukaryota	Fungi	Penicillium chrysogenum Wisconsin 54-1255	XP_002559989.1	2 E-47	465/535
Eukaryota	Fungi	Candida tropicalis MYA-3404	XP_002546916.1	3 E-47	496/535
Eukaryota	Fungi	Aspergillus fumigatus Af293	XP_748049.1	5 E-47	442/535
Eukaryota	Fungi	Nectria haematococca mpVI 77-13-4	EEU47256.1	1 E-46	470/535
Eukaryota	Fungi	Aspergillus niger CBS 513.88	XP_001390387.1	1 E-46	493/535
Eukaryota	Fungi	Penicillium marneffei ATCC 18224	XP_002151771.1	1 E-46	501/535
Eukaryota	Fungi	Paracoccidioides brasiliensis Pb03;	EEH17962.1	2 E-46	439/535
Eukaryota	Fungi	Coccidioides immitis RS;	XP_001242995.1	2 E-46	468/535
Eukaryota	Fungi	Penicillium marneffei ATCC 18224	XP_002148101.1	2 E-46	503/535
Eukaryota	Fungi	Aspergillus niger CBS 513.88	XP_001389950.1	2 E-46	472/535
Eukaryota	Fungi	Talaromyces stipitatus ATCC 10500	XP_002478822.1	2 E-46	494/535
Eukaryota	Fungi	Aspergillus clavatus NRRL 1	XP_001271885.1	2 E-46	464/535
Eukaryota	Fungi	Phaeosphaeria nodorum SN15	XP_001799730.1	2 E-46	502/535
Eukaryota	Fungi	Aspergillus terreus NIH2624	XP_001209052.1	3 E-46	478/535
Eukaryota	Fungi	Aspergillus terreus NIH2624	XP_001210516.1	3 E-46	484/535
Eukaryota	Fungi	Candida dubliniensis CD36	XP_002418030.1	3 E-46	516/535
Eukaryota	Fungi	Candida albicans SC5314	XP_721445.1	3 E-46	495/535
Eukaryota	Fungi	Ajellomyces capsulatus NAm1	XP_001543239.1	4 E-46	507/535
Eukaryota	Fungi	Aspergillus niger CBS 513.88	XP_001389930.1	4 E-46	484/535
Eukaryota	Fungi	Gibberella zeae PH-1	XP_387877.1	4 E-46	496/535
Eukaryota	Fungi	Candida albicans WO-1	EEQ42901.1	4 E-46	495/535
Eukaryota	Fungi	Gibberella zeae PH-1	XP_382615.1	4 E-46	483/535
Eukaryota	Fungi	Aspergillus flavus NRRL3357	XP_002380263.1	5 E-46	465/535
Eukaryota	Fungi	Ustilago maydis 521	XP_760842.1	5 E-46	481/535
Eukaryota	Fungi	Phaeosphaeria nodorum SN15	XP_001801125.1	6 E-46	439/535
Eukaryota	Fungi	Candida albicans SC5314	XP_719581.1	7 E-46	471/535
Eukaryota	Fungi	Candida albicans SC5314	XP_719453.1	7 E-46	471/535
Eukaryota	Fungi	Aspergillus fumigatus A1163	EDP50382.1	8 E-46	517/535
Bacteria	Actinobacteria	Streptomyces avermitilis MA-4680	NP_825299.1	8 E-46	504/535
Bacteria	Actinobacteria	Streptomyces coelicolor A3(2)	NP_733625.1	8 E-46	499/535
Eukaryota	Fungi	Verticillium albo-atrum VaMs.102	EEY17097.1	9 E-46	478/535
Eukaryota	Fungi	Aspergillus oryzae RIB40	XP_001827651.1	1 E-45	469/535
Eukaryota	Fungi	Aspergillus nidulans FGSC A4	CBF79670.1	1 E-45	472/535
Eukaryota	Fungi	Aspergillus fumigatus Af293	XP_751817.1	1 E-45	517/535
Eukaryota	Fungi	Coccidioides posadasii C735 delta	EER26127.1	1 E-45	508/535
Eukaryota	Fungi	Coccidioides immitis RS;	XP_001244672.1	1 E-45	489/535
Eukaryota	Fungi	Gibberella zeae PH-1	XP_382454.1	1 E-45	496/535
Eukaryota	Fungi	Botryotinia fuckeliana B05.10	XP_001549824.1	2 E-45	513/535
Eukaryota	Fungi	Lachancea thermotolerans CBS 6340	XP_002555500.1	2 E-45	516/535
Eukaryota	Fungi	Pichia guilliermondii ATCC 6260	EDK40038.2	2 E-45	495/535
Eukaryota	Fungi	Neosartorya fischeri NRRL 181	XP_001257317.1	2 E-45	484/535
Bacteria	Actinobacteria	Streptomyces sp. AA4	ZP_05484035.1	2 E-45	477/535
Eukaryota	Fungi	Ajellomyces dermatitidis ER-3	EEQ89488.1	3 E-45	466/535
Eukaryota	Fungi	Ajellomyces dermatitidis SLH14081	XP_002628685.1	3 E-45	466/535
Eukaryota	Fungi	Pichia guilliermondii ATCC 6260	XP_001483407.1	3 E-45	503/535
Eukaryota	Fungi	Aspergillus clavatus NRRL 1	XP_001268741.1	3 E-45	500/535

AFUA_4G03520

Eukaryota	Fungi	Aspergillus fumigatus Af293	XP_746539.1	0.0	328/328
Eukaryota	Fungi	Neosartorya fischeri NRRL 181	XP_001262457.1	1 E-168	328/328
Eukaryota	Fungi	Aspergillus flavus NRRL3357	XP_002375622.1	2 E-86	323/328
Eukaryota	Fungi	Aspergillus oryzae RIB40	XP_001727374.1	4 E-86	323/328
Eukaryota	Fungi	Aspergillus terreus NIH2624	XP_001211838.1	1 E-81	318/328
Eukaryota	Fungi	Aspergillus nidulans FGSC A4	XP_868913.1	2 E-71	333/328
Eukaryota	Fungi	Nectria haematococca mpVI 77-13-4	EEU37234.1	6 E-71	319/328
Eukaryota	Fungi	Talaromyces stipitatus ATCC 10500	XP_002478450.1	3 E-67	331/328
Eukaryota	Fungi	Gibberella zeae PH-1	XP_383032.1	2 E-66	321/328
Eukaryota	Fungi	Talaromyces stipitatus ATCC 10500	XP_002480979.1	6 E-45	326/328
Eukaryota	Fungi	Gibberella zeae PH-1	XP_389114.1	2 E-43	332/328
Eukaryota	Fungi	Aspergillus flavus NRRL3357	XP_002383814.1	7 E-43	328/328
Eukaryota	Fungi	Nectria haematococca mpVI 77-13-4	EEU47617.1	2 E-41	340/328
Eukaryota	Fungi	Nectria haematococca mpVI 77-13-4	EEU35981.1	4 E-41	328/328
Eukaryota	Fungi	Aspergillus oryzae RIB40	XP_001824788.1	7 E-40	323/328
Eukaryota	Fungi	Nectria haematococca mpVI 77-13-4	EEU45214.1	8 E-40	326/328
Eukaryota	Fungi	Nectria haematococca mpVI 77-13-4	EEU34860.1	8 E-40	330/328
Eukaryota	Fungi	Penicillium chrysogenum Wisconsin 54-1255	XP_002556641.1	1 E-39	329/328
Eukaryota	Fungi	Chaetomium globosum CBS 148.51	XP_001221418.1	3 E-38	336/328
Eukaryota	Fungi	Gibberella zeae PH-1	XP_384929.1	4 E-38	330/328
Eukaryota	Fungi	Gibberella zeae PH-1	XP_390504.1	5 E-38	329/328
Eukaryota	Fungi	Botryotinia fuckeliana B05.10	XP_001560280.1	2 E-37	344/328
Eukaryota	Fungi	Pyrenophora tritici-repentis Pt-1C-BFP	XP_001939716.1	1 E-36	326/328
Eukaryota	Fungi	Podospora anserina DSM 980	XP_001905519.1	6 E-36	335/328
Eukaryota	Fungi	Sclerotinia sclerotiorum 1980 UF-70	XP_001586141.1	3 E-35	342/328
Eukaryota	Fungi	Phaeosphaeria nodorum SN15	XP_001801328.1	8 E-35	336/328
Eukaryota	Fungi	Neosartorya fischeri NRRL 181	XP_001261787.1	1 E-34	307/328
Eukaryota	Fungi	Aspergillus niger CBS 513.88	XP_001389101.1	3 E-33	338/328
Eukaryota	Fungi	Neurospora crassa OR74A	XP_958003.1	3 E-32	267/328
Eukaryota	Fungi	Coccidioides immitis RS;	XP_001240786.1	3 E-31	334/328
Eukaryota	Fungi	Coccidioides posadasii C735 delta	EER22977.1	3 E-31	334/328
Eukaryota	Fungi	Nectria haematococca mpVI 77-13-4	EEU35184.1	4 E-31	333/328
Eukaryota	Fungi	Aspergillus flavus NRRL3357	XP_002379662.1	8 E-31	334/328
Eukaryota	Fungi	Uncinocarpus reesii 1704	XP_002542729.1	1 E-30	334/328
Eukaryota	Fungi	Talaromyces stipitatus ATCC 10500	XP_002481474.1	2 E-30	320/328
Eukaryota	Fungi	Gibberella zeae PH-1	XP_383700.1	7 E-30	334/328
Eukaryota	Fungi	Aspergillus oryzae RIB40	XP_001821756.1	2 E-29	298/328
Eukaryota	Fungi	Botryotinia fuckeliana B05.10	XP_001561343.1	2 E-29	307/328
Eukaryota	Fungi	Phaeosphaeria nodorum SN15	XP_001791825.1	3 E-29	297/328
Eukaryota	Fungi	Penicillium marneffei ATCC 18224	XP_002150675.1	5 E-29	338/328
Eukaryota	Fungi	Nectria haematococca mpVI 77-13-4	EEU36390.1	3 E-28	301/328
Eukaryota	Fungi	Talaromyces stipitatus ATCC 10500	XP_002483465.1	1 E-27	338/328
Eukaryota	Fungi	Aspergillus niger CBS 513.88	XP_001395342.1	2 E-26	285/328
Eukaryota	Fungi	Aspergillus nidulans FGSC A4	XP_682601.1	2 E-26	283/328
Eukaryota	Fungi	Podospora anserina DSM 980	XP_001905531.1	4 E-25	319/328
Eukaryota	Fungi	Talaromyces stipitatus ATCC 10500	XP_002479404.1	8 E-25	322/328
Eukaryota	Fungi	Aspergillus niger CBS 513.88	XP_001402390.1	1 E-24	266/328
Eukaryota	Fungi	Verticillium albo-atrum VaMs.102	EEY22452.1	1 E-23	298/328
Eukaryota	Fungi	Neurospora crassa OR74A	XP_959079.1	1 E-23	319/328
Eukaryota	Fungi	Ajellomyces dermatitidis SLH14081	XP_002620908.1	8 E-23	273/328
Eukaryota	Fungi	Ajellomyces capsulatus NAm1	XP_001537249.1	9 E-23	273/328
Eukaryota	Fungi	Talaromyces stipitatus ATCC 10500	XP_002340593.1	1 E-22	306/328
Eukaryota	Fungi	Ajellomyces capsulatus H143	EER44916.1	1 E-22	273/328
Eukaryota	Fungi	Ajellomyces capsulatus G186AR	EEH03484.1	2 E-22	273/328
Eukaryota	Fungi	Ajellomyces dermatitidis ER-3	EEQ86173.1	3 E-22	273/328
Eukaryota	Fungi	Gibberella zeae PH-1	XP_389548.1	3 E-22	278/328

Eukaryota	Fungi	Talaromyces stipitatus ATCC 10500	XP_002340017.1	3 E-22	293/328
Eukaryota	Fungi	Sclerotinia sclerotiorum 1980 UF-70	XP_001587235.1	6 E-22	282/328
Eukaryota	Fungi	Microsporum canis CBS 113480	EEQ29167.1	7 E-22	277/328
Eukaryota	Fungi	Debaryomyces hansenii	CAG84741.2	1 E-21	271/328
Eukaryota	Fungi	Magnaporthe grisea 70-15	XP_369088.1	1 E-21	310/328
Eukaryota	Fungi	Talaromyces stipitatus ATCC 10500	XP_002479219.1	2 E-21	328/328
Eukaryota	Fungi	Aspergillus terreus NIH2624	XP_001215701.1	2 E-21	304/328
Eukaryota	Fungi	Debaryomyces hansenii CBS767	XP_456778.1	4 E-21	270/328
Eukaryota	Fungi	Verticillium albo-atrum VaMs.102	EEY17848.1	7 E-21	292/328
Eukaryota	Fungi	Aspergillus fumigatus A1163	EDP48530.1	7 E-21	301/328
Eukaryota	Fungi	Aspergillus fumigatus Af293	XP_748819.1	3 E-20	301/328
Eukaryota	Fungi	Botryotinia fuckeliana B05.10	XP_001559720.1	3 E-20	286/328
Eukaryota	Fungi	Aspergillus niger CBS 513.88	XP_001397387.1	1 E-19	269/328
Eukaryota	Fungi	Nectria haematococca mpVI 77-13-4	EEU48852.1	1 E-19	301/328
Eukaryota	Fungi	Aspergillus nidulans FGSC A4	XP_662212.1	4 E-19	297/328
Eukaryota	Fungi	Penicillium marneffei ATCC 18224	XP_002148633.1	1 E-18	308/328
Eukaryota	Fungi	Talaromyces stipitatus ATCC 10500	XP_002485609.1	2 E-18	274/328
Eukaryota	Fungi	Aspergillus oryzae RIB40	XP_001816657.1	2 E-18	304/328
Eukaryota	Fungi	Nectria haematococca mpVI 77-13-4	EEU45747.1	2 E-18	276/328
Eukaryota	Fungi	Aspergillus flavus NRRL3357	XP_002383428.1	3 E-18	304/328
Eukaryota	Amoebozoa	Dictyostelium discoideum AX4	XP_644302.1	8 E-18	302/328
Eukaryota	Metazoa	Gallus gallus	NP_001025816.1	5 E-17	288/328
Eukaryota	Fungi	Botryotinia fuckeliana B05.10	XP_001549390.1	6 E-17	307/328
Eukaryota	Fungi	Debaryomyces hansenii	CAR65717.1	1 E-16	276/328
Eukaryota	Fungi	Debaryomyces hansenii CBS767	XP_459312.1	1 E-16	278/328
Eukaryota	Fungi	Botryotinia fuckeliana B05.10	XP_001554652.1	5 E-16	272/328
Eukaryota	Fungi	Uncinocarpus reesii 1704	XP_002543273.1	6 E-16	270/328
Eukaryota	Metazoa	Monodelphis domestica	XP_001377212.1	7 E-16	303/328
Eukaryota	Fungi	Coccidioides posadasii C735 delta	EER28515.1	1 E-15	270/328
Eukaryota	Metazoa	Macaca mulatta	XP_001093249.1	1 E-15	289/328
Eukaryota	Metazoa	Ornithorhynchus anatinus	XP_001507495.1	1 E-15	288/328
Eukaryota	Metazoa	Pan troglodytes	XP_001152180.1	2 E-15	289/328
Eukaryota	Fungi	Coccidioides immitis RS;	XP_001241710.1	2 E-15	270/328
Eukaryota	Metazoa	Monodelphis domestica	XP_001377245.1	2 E-14	289/328
Eukaryota	Metazoa	Rana catesbeiana	ACO51603.1	2 E-14	287/328
Eukaryota	Metazoa	Xenopus laevis	AAH68857.1	2 E-14	284/328
Eukaryota	Fungi	Chaetomium globosum CBS 148.51	XP_001223491.1	3 E-14	280/328
Eukaryota	Amoebozoa	Dictyostelium discoideum AX4	XP_643826.1	3 E-14	301/328
Eukaryota	Fungi	Penicillium marneffei ATCC 18224	XP_002153197.1	3 E-14	273/328
Eukaryota	Fungi	Aspergillus terreus NIH2624	XP_001209779.1	1 E-13	278/328
Bacteria	Cyanobacteria	Synechococcus sp. CC9605	YP_381144.1	3 E-13	324/328
Eukaryota	Metazoa	Equus caballus	XP_001497893.2	3 E-13	279/328
Eukaryota	Fungi	Phaeosphaeria nodorum SN15	XP_001791268.1	6 E-13	301/328
Eukaryota	Fungi	Pichia guilliermondii ATCC 6260	XP_001487009.1	8 E-13	279/328
Eukaryota	Fungi	Pichia guilliermondii ATCC 6260	EDK36288.2	9 E-13	279/328
Eukaryota	Fungi	Pyrenophora tritici-repentis Pt-1C-BFP	XP_001940929.1	1 E-12	312/328
Bacteria	Cyanobacteria	Acaryochloris marina MBIC11017	YP_001516988.1	3 E-12	303/328
Eukaryota	Fungi	Aspergillus terreus NIH2624	XP_001215113.1	4 E-12	295/328
Eukaryota	Metazoa	Taeniopygia guttata	XP_002194655.1	4 E-12	289/328
Eukaryota	Metazoa	Pan troglodytes	XP_526413.2	1 E-11	277/328
Bacteria	Actinobacteria	Streptomyces hygroscopicus ATCC 53653	ZP_05514701.1	3 E-11	318/328
Bacteria	Proteobacteria	Yersinia frederiksenii ATCC 33641	ZP_04632045.1	3 E-11	309/328

AFUA_4G04820

Eukaryota	Fungi	Aspergillus fumigatus Af293	XP_746668.1	1 E-174	296/296
Eukaryota	Fungi	Aspergillus nidulans FGSC A4	CBF79344.1	1 E-158	296/296
Eukaryota	Fungi	Aspergillus oryzae RIB40	XP_001826514.1	1 E-158	296/296

Eukaryota	Fungi	Penicillium chrysogenum;	ACG56268.1	1 E-153	295/296
Eukaryota	Fungi	Penicillium chrysogenum Wisconsin 54-1255	XP_002558991.1	1 E-152	294/296
Eukaryota	Fungi	Penicillium marneffei ATCC 18224	XP_002152703.1	1 E-149	287/296
Eukaryota	Fungi	Penicillium marneffei ATCC 18224	XP_002152704.1	1 E-149	286/296
Eukaryota	Fungi	Talaromyces stipitatus ATCC 10500	XP_002486406.1	1 E-147	287/296
Eukaryota	Fungi	Coccidioides posadasii C735 delta	EER28298.1	1 E-147	298/296
Eukaryota	Fungi	Penicillium chrysogenum Wisconsin 54-1255	XP_002569104.1	1 E-145	292/296
Eukaryota	Fungi	Penicillium chrysogenum;;	ABA70590.1	1 E-145	291/296
Eukaryota	Fungi	Penicillium chrysogenum;;	ABR12622.1	1 E-141	319/296
Eukaryota	Fungi	Ajellomyces dermatitidis SLH14081	XP_002620488.1	1 E-140	296/296
Eukaryota	Fungi	Aspergillus terreus NIH2624	XP_001211953.1	1 E-136	246/296
Eukaryota	Fungi	Aspergillus clavatus NRRL 1	XP_001275196.1	1 E-135	249/296
Eukaryota	Fungi	Paracoccidioides brasiliensis Pb18;	EEH43985.1	1 E-133	291/296
Eukaryota	Fungi	Microsporum canis CBS 113480	EEQ29986.1	1 E-133	263/296
Eukaryota	Fungi	Uncinocarpus reesii 1704	XP_002582293.1	1 E-132	252/296
Eukaryota	Fungi	Botryotinia fuckeliana B05.10	XP_001547478.1	1 E-130	294/296
Eukaryota	Fungi	Sclerotinia sclerotiorum 1980 UF-70	XP_001585991.1	1 E-130	294/296
Eukaryota	Fungi	Coccidioides immitis RS;	XP_001241345.1	1 E-126	247/296
Eukaryota	Fungi	Uncinocarpus reesii 1704	XP_002543059.1	1 E-125	240/296
Eukaryota	Fungi	Phaeosphaeria nodorum SN15	XP_001798717.1	1 E-125	288/296
Eukaryota	Fungi	Pyrenophora tritici-repentis Pt-1C-BFP	XP_001938772.1	1 E-125	289/296
Eukaryota	Fungi	Verticillium albo-atrum VaMs.102	EEY19070.1	1 E-124	279/296
Eukaryota	Fungi	Ajellomyces capsulatus G186AR	EEH03406.1	1 E-123	259/296
Eukaryota	Fungi	Chaetomium globosum	ABF84060.1	1 E-122	301/296
Eukaryota	Fungi	Podospora anserina DSM 980	XP_001910102.1	1 E-121	301/296
Eukaryota	Fungi	Nectria haematococca mpVI 77-13-4	EEU40354.1	1 E-120	302/296
Eukaryota	Fungi	Microsporum canis CBS 113480	EEQ32084.1	1 E-118	272/296
Eukaryota	Fungi	Magnaporthe grisea 70-15	XP_369331.1	1 E-118	286/296
Eukaryota	Fungi	Gibberella zeae PH-1	XP_390006.1	1 E-117	299/296
Eukaryota	Fungi	Neurospora crassa OR74A	XP_962240.1	1 E-117	289/296
Eukaryota	Fungi	Magnaporthe grisea 70-15	XP_360827.1	1 E-113	276/296
Eukaryota	Fungi	Nectria haematococca mpVI 77-13-4	EEU42161.1	1 E-112	292/296
Eukaryota	Fungi	Neurospora crassa OR74A	XP_001728201.1	1 E-107	248/296
Eukaryota	Fungi	Pichia stipitis CBS 6054	XP_001384753.1	1 E-102	274/296
Eukaryota	Fungi	Lachancea thermotolerans CBS 6340	XP_002553288.1	1 E-101	296/296
Eukaryota	Fungi	Ashbya gossypii ATCC 10895	NP_986119.1	1 E-100	284/296
Eukaryota	Fungi	Candida dubliniensis CD36	XP_002421808.1	1 E-100	289/296
Eukaryota	Fungi	Schizosaccharomyces pombe	NP_592903.1	1 E-100	287/296
Eukaryota	Fungi	Candida albicans SC5314	XP_713456.1	1 E-100	289/296
Eukaryota	Fungi	Ustilago maydis 521	XP_762027.1	1 E-100	270/296
Eukaryota	Fungi	Candida tropicalis MYA-3404	XP_002546126.1	2 E-99	297/296
Eukaryota	Fungi	Chaetomium globosum CBS 148.51	XP_001223958.1	2 E-98	264/296
Eukaryota	Fungi	Pichia pastoris GS115	XP_002492261.1	4 E-98	295/296
Eukaryota	Fungi	Saccharomyces cerevisiae	AAS56153.1	5 E-98	295/296
Eukaryota	Fungi	Saccharomyces cerevisiae EC1118	CAY79820.1	5 E-98	295/296
Eukaryota	Fungi	Candida glabrata CBS 138	XP_448420.1	6 E-98	295/296
Eukaryota	Fungi	Saccharomyces cerevisiae	NP_011574.1	6 E-98	295/296
Eukaryota	Fungi	Schizosaccharomyces japonicus yFS275	XP_002174187.1	1 E-97	270/296
Eukaryota	Fungi	Pichia guilliermondii ATCC 6260	EDK37810.2	3 E-97	287/296
Eukaryota	Fungi	Lodderomyces elongisporus NRRL YB-4239	XP_001527555.1	5 E-97	270/296
Eukaryota	Fungi	Pichia guilliermondii ATCC 6260	XP_001486237.1	6 E-97	287/296
Eukaryota	Fungi	Vanderwaltozyma polyspora DSM 70294	XP_001643341.1	3 E-96	295/296
Eukaryota	Fungi	Zygosaccharomyces rouxii CBS 732	XP_002495261.1	7 E-96	269/296
Eukaryota	Fungi	Yarrowia lipolytica CLIB122	XP_505281.1	1 E-95	288/296
Eukaryota	Fungi	Kluyveromyces lactis NRRL Y-1140	XP_451890.1	7 E-95	277/296
Eukaryota	Fungi	Vanderwaltozyma polyspora DSM 70294	XP_001642831.1	2 E-94	294/296
Eukaryota	Fungi	Clavispora lusitaniae ATCC 42720	XP_002619218.1	6 E-94	287/296
Eukaryota	Fungi	Zygosaccharomyces rouxii CBS 732	XP_002495286.1	7 E-94	274/296

Eukaryota	Fungi	Debaryomyces hansenii	CAG84836.2	1 E-93	287/296
Eukaryota	Fungi	Debaryomyces hansenii CBS767	XP_456861.1	2 E-93	287/296
Eukaryota	Fungi	Pichia stipitis CBS 6054	XP_001387513.1	3 E-92	279/296
Eukaryota	Fungi	Pichia guilliermondii ATCC 6260	EDK41155.2	3 E-90	287/296
Eukaryota	Fungi	Glomus intraradices	ACR83862.1	8 E-90	281/296
Eukaryota	Fungi	Clavispora lusitaniae ATCC 42720	XP_002618654.1	3 E-89	286/296
Eukaryota	Fungi	Aspergillus fumigatus Af293	XP_746962.1	4 E-88	299/296
Eukaryota	Fungi	Coprinopsis cinerea okayama7#130	XP_001838294.1	5 E-88	272/296
Eukaryota	Fungi	Cryptococcus neoformans var. neoformans	XP_569526.1	2 E-87	271/296
Eukaryota	Fungi	Laccaria bicolor S238N-H82	XP_001876145.1	2 E-87	271/296
Eukaryota	Fungi	Pichia guilliermondii ATCC 6260	XP_001482233.1	4 E-87	287/296
Eukaryota	Fungi	Candida tropicalis MYA-3404	XP_002545417.1	6 E-87	300/296
Eukaryota	Fungi	Debaryomyces hansenii	CAG90106.2	5 E-86	279/296
Eukaryota	Fungi	Aspergillus niger CBS 513.88	XP_001390516.1	8 E-86	289/296
Eukaryota	Fungi	Debaryomyces hansenii CBS767	XP_461658.1	3 E-85	279/296
Eukaryota	Fungi	Candida dubliniensis CD36	XP_002419818.1	1 E-84	301/296
Eukaryota	Fungi	Aspergillus clavatus NRRL 1	XP_001275068.1	8 E-84	294/296
Eukaryota	Fungi	Lodderomyces elongisporus NRRL YB-4239	XP_001524278.1	9 E-84	270/296
Eukaryota	Fungi	Aspergillus terreus NIH2624	XP_001213994.1	2 E-83	284/296
Eukaryota	Fungi	Candida albicans SC5314	XP_722849.1	2 E-83	293/296
Eukaryota	Fungi	Candida albicans SC5314	XP_722703.1	3 E-83	293/296
Eukaryota	Fungi	Aspergillus oryzae RIB40	XP_001827624.1	6 E-83	272/296
Eukaryota	Fungi	Neosartorya fischeri NRRL 181	XP_001261890.1	4 E-82	299/296
Eukaryota	Fungi	Aspergillus flavus NRRL3357	XP_002384865.1	1 E-80	255/296
Eukaryota	Fungi	Aspergillus nidulans FGSC A4	CBF84688.1	1 E-80	280/296
Eukaryota	Fungi	Aspergillus nidulans FGSC A4	XP_682176.1	3 E-80	277/296
Eukaryota	Amoebozoa	Dictyostelium discoideum AX4	XP_646277.1	1 E-55	263/296
Eukaryota	Viridiplantae	Micromonas sp. RCC299	XP_002508762.1	9 E-54	239/296
Eukaryota	Amoebozoa	Dictyostelium discoideum	Q55D52.2	5 E-53	265/296
Eukaryota	Viridiplantae	Zea mays;	ACF82703.1	6 E-49	259/296
Eukaryota	Viridiplantae	Sorghum bicolor;	XP_002465927.1	7 E-49	265/296
Eukaryota	Viridiplantae	Zea mays;	NP_001148435.1	1 E-48	260/296
Eukaryota	Viridiplantae	Ostreococcus tauri	CAL54207.1	2 E-48	245/296
Eukaryota	Viridiplantae	Zea mays;	ACL53689.1	2 E-48	265/296
Eukaryota	Viridiplantae	Zea mays;	ACG34890.1	4 E-48	265/296
Eukaryota	Viridiplantae	Zea mays;	NP_001105744.1	4 E-48	262/296
Eukaryota	Viridiplantae	Gossypium arboreum	AAO13795.1	6 E-48	251/296
Eukaryota	Viridiplantae	Ostreococcus lucimarinus CCE9901	XP_001418099.1	8 E-48	239/296
Eukaryota	Viridiplantae	Zea mays;	NP_001148153.1	8 E-48	241/296
Eukaryota	Viridiplantae	Oryza sativa Japonica Group	NP_001068557.1	1 E-47	261/296
Eukaryota	Viridiplantae	Boechera divaricarpa	ABW74580.1	2 E-47	243/296
Eukaryota	Metazoa	Monodelphis domestica	XP_001365974.1	3 E-47	243/296
Eukaryota	Viridiplantae	Oryza sativa Indica Group	EEC81368.1	4 E-47	242/296
Eukaryota	Viridiplantae	Physcomitrella patens subsp. patens	XP_001758083.1	4 E-47	242/296
Eukaryota	Viridiplantae	Oryza sativa Japonica Group	NP_001058678.1	5 E-47	242/296
Eukaryota	Viridiplantae	Capsella rubella	ABW81143.1	5 E-47	243/296
Eukaryota	Viridiplantae	Physcomitrella patens subsp. patens	XP_001770841.1	6 E-47	245/296
Eukaryota	Viridiplantae	Picea sitchensis	ABK24231.1	7 E-47	243/296
Eukaryota	Viridiplantae	Populus trichocarpa	XP_002298337.1	8 E-47	251/296
Eukaryota	Viridiplantae	Arabidopsis thaliana	AAF79571.1	8 E-47	250/296
Eukaryota	Viridiplantae	Arabidopsis thaliana	NP_563789.1	8 E-47	250/296
Eukaryota	Viridiplantae	Populus trichocarpa	XP_002313418.1	1 E-46	251/296
Eukaryota	Viridiplantae	Populus trichocarpa	ABK93133.1	1 E-46	251/296
Eukaryota	Viridiplantae	Arabidopsis lyrata subsp. lyrata	ABW81040.1	1 E-46	243/296
Eukaryota	Metazoa	Homo sapiens	NP_006736.1	1 E-46	240/296
Eukaryota	Metazoa	Macaca mulatta	XP_001101334.1	1 E-46	240/296
Eukaryota	Metazoa	Sus scrofa	NP_998917.1	1 E-46	240/296
Eukaryota	Metazoa	Rattus norvegicus	NP_543162.1	1 E-46	240/296

Eukaryota	Viridiplantae	Arabidopsis thaliana	AAM64821.1	2 E-46	250/296
Eukaryota	Viridiplantae	Vitis vinifera	XP_002282305.1	2 E-46	250/296
Eukaryota	Viridiplantae	Arabidopsis thaliana	NP_973559.1	2 E-46	243/296
Eukaryota	Viridiplantae	Arabidopsis thaliana	NP_850133.1	3 E-46	243/296
Eukaryota	Metazoa	Bos taurus	NP_001092333.1	3 E-46	240/296
Eukaryota	Metazoa	Mus musculus	NP_079712.1	5 E-46	237/296
Eukaryota	Viridiplantae	Vitis vinifera	XP_002282653.1	9 E-46	255/296
Eukaryota	Viridiplantae	Vitis vinifera	CAO69146.1	1 E-45	255/296
Eukaryota	Viridiplantae	Ricinus communis	XP_002520505.1	1 E-45	251/296
Eukaryota	Metazoa	Trichoplax adhaerens	XP_002109654.1	1 E-45	242/296
Eukaryota	Viridiplantae	Glycine max	ACU21580.1	2 E-45	243/296
Eukaryota	Metazoa	Xenopus (Silurana) tropicalis	NP_001072809.1	2 E-45	241/296
Eukaryota	Viridiplantae	Glycine max	ACU23987.1	2 E-45	243/296
Eukaryota	Metazoa	Equus caballus	XP_001499719.1	8 E-45	240/296
Eukaryota	Metazoa	Ornithorhynchus anatinus	XP_001507700.1	3 E-44	243/296
Eukaryota	Metazoa	Gallus gallus	NP_001006438.1	1 E-43	243/296
Eukaryota	Viridiplantae	Arabidopsis thaliana	NP_565681.1	1 E-43	255/296
Eukaryota	Metazoa	Branchiostoma floridae	XP_002612908.1	1 E-43	241/296
Eukaryota	Metazoa	Strongylocentrotus purpuratus	XP_787442.1	2 E-43	243/296
Eukaryota	Viridiplantae	Oryza sativa Japonica Group	AAX95419.1	4 E-43	270/296
Eukaryota	Metazoa	Strongylocentrotus purpuratus	XP_001178153.1	2 E-35	239/296
Eukaryota	Amoebozoa	Dictyostelium discoideum AX4	XP_641553.1	3 E-35	242/296
Eukaryota	Viridiplantae	Populus trichocarpa	XP_002304440.1	4 E-34	258/296
Eukaryota	Viridiplantae	Arabidopsis thaliana	AAM64961.1	7 E-33	252/296
Eukaryota	Viridiplantae	Arabidopsis thaliana	NP_192948.1	7 E-33	252/296
Eukaryota	Viridiplantae	Physcomitrella patens subsp. patens	XP_001780893.1	8 E-33	248/296
Eukaryota	Viridiplantae	Arabidopsis thaliana	CAB40952.1	9 E-33	252/296
Eukaryota	Viridiplantae	Arabidopsis thaliana	AAQ13424.1	2 E-32	252/296
Eukaryota	Viridiplantae	Arabidopsis thaliana	AAK61361.1	8 E-32	254/296
Eukaryota	Viridiplantae	Arabidopsis thaliana	NP_567670.1	1 E-31	254/296
Eukaryota	Viridiplantae	Populus trichocarpa	XP_002329848.1	1 E-31	271/296
Eukaryota	Viridiplantae	Arabidopsis thaliana	AAM65428.1	4 E-31	254/296
Eukaryota	Viridiplantae	Glycine max	ACU17994.1	5 E-31	264/296
Eukaryota	Viridiplantae	Arabidopsis thaliana	CAA16560.1	7 E-30	264/296
Eukaryota	Viridiplantae	Populus trichocarpa	XP_002320723.1	2 E-29	249/296
Eukaryota	Alveolata	Paramecium tetraurelia strain d4-2	XP_001448034.1	7 E-24	237/296
Eukaryota	Alveolata	Tetrahymena thermophila	XP_001032917.1	2 E-22	240/296
Eukaryota	Metazoa	Bos taurus	NP_001068711.1	7 E-22	250/296
Eukaryota	Metazoa	Mus musculus	NP_034020.1	8 E-22	241/296
Eukaryota	Metazoa	Mus musculus	AAH39919.1	1 E-21	241/296
Eukaryota	Metazoa	Mus musculus	BAE35945.1	2 E-21	241/296
Eukaryota	Metazoa	Danio rerio	NP_001076393.1	1 E-19	248/296
Eukaryota	Metazoa	Salmo salar	NP_001134334.1	3 E-19	255/296
Eukaryota	Metazoa	Macaca mulatta	XP_001083208.1	1 E-18	253/296
Eukaryota	Metazoa	Branchiostoma floridae	XP_002587132.1	2 E-18	249/296

AFUA_4G06410

Eukaryota	Fungi	Aspergillus fumigatus Af293	XP_752183.1	1 E-154	265/265
Eukaryota	Fungi	Neosartorya fischeri NRRL 181	XP_001267377.1	1 E-100	256/265
Eukaryota	Fungi	Aspergillus flavus NRRL3357	XP_002377224.1	2 E-31	235/265

AFUA_4G06420

Eukaryota	Fungi	Aspergillus fumigatus Af293	XP_752182.1	0.0	798/798
Eukaryota	Fungi	Neosartorya fischeri NRRL 181	XP_001267376.1	0.0	793/798
Eukaryota	Fungi	Penicillium chrysogenum Wisconsin 54-1255	XP_002565640.1	0.0	809/798
Eukaryota	Fungi	Aspergillus flavus NRRL3357	XP_002377220.1	0.0	693/798

Eukaryota	Fungi	Talaromyces stipitatus ATCC 10500	XP_002341090.1	0.0	797/798
Eukaryota	Fungi	Penicillium marneffei ATCC 18224	XP_002144445.1	0.0	774/798
Eukaryota	Fungi	Aspergillus terreus NIH2624	XP_001214760.1	0.0	675/798
Eukaryota	Fungi	Aspergillus niger CBS 513.88	XP_001401443.1	0.0	718/798
Eukaryota	Fungi	Aspergillus nidulans FGSC A4	CBF77703.1	0.0	773/798
Eukaryota	Fungi	Aspergillus nidulans FGSC A4	XP_661954.1	0.0	763/798
Eukaryota	Fungi	Nectria haematococca mpVI 77-13-4	EEU44999.1	0.0	836/798
Eukaryota	Fungi	Gibberella zeae PH-1	XP_390206.1	0.0	837/798
Eukaryota	Fungi	Podospora anserina DSM 980	XP_001911808.1	0.0	821/798
Eukaryota	Fungi	Pyrenophora tritici-repentis Pt-1C-BFP	XP_001934500.1	0.0	809/798
Eukaryota	Fungi	Verticillium albo-atrum VaMs.102	EEY14349.1	1 E-176	713/798
Eukaryota	Fungi	Chaetomium globosum CBS 148.51	XP_001224687.1	1 E-173	770/798
Eukaryota	Fungi	Neurospora crassa OR74A	XP_961085.1	1 E-142	687/798
Eukaryota	Fungi	Pyrenophora tritici-repentis Pt-1C-BFP	XP_001933904.1	9 E-37	669/798
Eukaryota	Fungi	Verticillium albo-atrum VaMs.102	EEY20636.1	2 E-33	678/798
Eukaryota	Fungi	Talaromyces stipitatus ATCC 10500	XP_002480487.1	2 E-31	737/798
Eukaryota	Fungi	Ajellomyces dermatitidis ER-3	EEQ83765.1	4 E-31	653/798
Eukaryota	Fungi	Ajellomyces dermatitidis SLH14081	XP_002625152.1	5 E-30	653/798
Eukaryota	Fungi	Nectria haematococca mpVI 77-13-4	EEU43058.1	7 E-30	657/798
Eukaryota	Fungi	Neosartorya fischeri NRRL 181	XP_001265095.1	8 E-30	652/798
Eukaryota	Fungi	Aspergillus fumigatus Af293	XP_750323.1	5 E-29	652/798
Eukaryota	Fungi	Aspergillus fumigatus A1163	EDP55910.1	9 E-29	652/798
Eukaryota	Fungi	Gibberella zeae PH-1	XP_385679.1	2 E-27	822/798
Eukaryota	Fungi	Sclerotinia sclerotiorum 1980 UF-70	XP_001586545.1	8 E-23	705/798

AFUA_4G07710

Eukaryota	Fungi	Aspergillus fumigatus Af293	XP_752054.1	0.0	1193/1193
Eukaryota	Fungi	Neosartorya fischeri NRRL 181	XP_001267251.1	0.0	1193/1193
Eukaryota	Fungi	Aspergillus clavatus NRRL 1	XP_001271664.1	0.0	1192/1193
Eukaryota	Fungi	Aspergillus oryzae RIB40	XP_001821306.1	0.0	1193/1193
Eukaryota	Fungi	Aspergillus niger CBS 513.88	XP_001401577.1	0.0	1191/1193
Eukaryota	Fungi	Aspergillus terreus	O93918.1	0.0	1193/1193
Eukaryota	Fungi	Aspergillus niger	Q9HES8.1	0.0	1191/1193
Eukaryota	Fungi	Aspergillus terreus NIH2624	Q0CLK1.2	0.0	1193/1193
Eukaryota	Fungi	Aspergillus nidulans FGSC A4	XP_662066.1	0.0	1188/1193
Eukaryota	Fungi	Penicillium chrysogenum Wisconsin 54-1255	XP_002559154.1	0.0	1192/1193
Eukaryota	Fungi	Talaromyces stipitatus ATCC 10500	XP_002485193.1	0.0	1182/1193
Eukaryota	Fungi	Penicillium marneffei ATCC 18224	XP_002148985.1	0.0	1182/1193
Eukaryota	Fungi	Aspergillus terreus NIH2624	XP_001214611.1	0.0	1146/1193
Eukaryota	Fungi	Ajellomyces capsulatus G186AR	EEH10652.1	0.0	1186/1193
Eukaryota	Fungi	Ajellomyces dermatitidis ER-3	EEQ88600.1	0.0	1185/1193
Eukaryota	Fungi	Coccidioides posadasii C735 delta	EER25315.1	0.0	1184/1193
Eukaryota	Fungi	Coccidioides immitis RS;	XP_001239952.1	0.0	1184/1193
Eukaryota	Fungi	Paracoccidioides brasiliensis Pb01;	EEH37805.1	0.0	1188/1193
Eukaryota	Fungi	Paracoccidioides brasiliensis Pb18;	EEH48019.1	0.0	1185/1193
Eukaryota	Fungi	Paracoccidioides brasiliensis Pb03;	EEH19179.1	0.0	1185/1193
Eukaryota	Fungi	Microsporum canis CBS 113480	EEQ33318.1	0.0	1185/1193
Eukaryota	Fungi	Ajellomyces capsulatus NAm1	XP_001540387.1	0.0	1171/1193
Eukaryota	Fungi	Ajellomyces dermatitidis SLH14081	XP_002627170.1	0.0	1154/1193
Eukaryota	Fungi	Pyrenophora tritici-repentis Pt-1C-BFP	XP_001939124.1	0.0	1184/1193
Eukaryota	Fungi	Phaeosphaeria nodorum SN15	XP_001794065.1	0.0	1191/1193
Eukaryota	Fungi	Botryotinia fuckeliana B05.10	XP_001556558.1	0.0	1181/1193
Eukaryota	Fungi	Sclerotinia sclerotiorum 1980 UF-70	XP_001586261.1	0.0	1179/1193
Eukaryota	Fungi	Uncinocarpus reesii 1704	XP_002584060.1	0.0	1126/1193
Eukaryota	Fungi	Magnaporthe grisea 70-15	XP_367852.2	0.0	1182/1193
Eukaryota	Fungi	Podospora anserina DSM 980	XP_001913060.1	0.0	1113/1193
Eukaryota	Fungi	Ajellomyces capsulatus H143	EER44717.1	0.0	1117/1193

Eukaryota	Fungi	Neurospora crassa OR74A	XP_965636.1	0.0	1156/1193
Eukaryota	Fungi	Nectria haematococca mpVI 77-13-4	EEU46408.1	0.0	1137/1193
Eukaryota	Fungi	Yarrowia lipolytica CLIB122	XP_502210.1	0.0	1187/1193
Eukaryota	Fungi	Candida glabrata CBS 138	XP_448518.1	0.0	1157/1193
Eukaryota	Fungi	Pichia angusta	Q8X1T3.1	0.0	1167/1193
Eukaryota	Fungi	Pichia pastoris GS115	XP_002492193.1	0.0	1163/1193
Eukaryota	Fungi	Pichia guilliermondii ATCC 6260	EDK39659.2	0.0	1163/1193
Eukaryota	Fungi	Pichia guilliermondii ATCC 6260	XP_001484376.1	0.0	1163/1193
Eukaryota	Fungi	Lodderomyces elongisporus NRRL YB-4239	XP_001524462.1	0.0	1169/1193
Eukaryota	Fungi	Pichia stipitis CBS 6054	XP_001386229.1	0.0	1171/1193
Eukaryota	Fungi	Saccharomyces cerevisiae	NP_009777.1	0.0	1157/1193
Eukaryota	Fungi	Saccharomyces cerevisiae YJM789	EDN64827.1	0.0	1157/1193
Eukaryota	Fungi	Saccharomyces cerevisiae RM11-1a	EDV11895.1	0.0	1157/1193
Eukaryota	Fungi	Lachancea thermotolerans CBS 6340	XP_002556166.1	0.0	1158/1193
Eukaryota	Fungi	Schizosaccharomyces pombe	NP_595900.1	0.0	1172/1193
Eukaryota	Fungi	Schizosaccharomyces pombe	BAA11239.1	0.0	1172/1193
Eukaryota	Fungi	Clavispora lusitaniae ATCC 42720	XP_002617098.1	0.0	1163/1193
Eukaryota	Fungi	Debaryomyces hansenii	CAG86153.2	0.0	1173/1193
Eukaryota	Fungi	Candida tropicalis MYA-3404	XP_002545478.1	0.0	1169/1193
Eukaryota	Fungi	Candida dubliniensis CD36	XP_002420024.1	0.0	1163/1193
Eukaryota	Fungi	Debaryomyces hansenii CBS767	XP_458082.1	0.0	1173/1193
Eukaryota	Fungi	Candida glabrata CBS 138	XP_446270.1	0.0	1158/1193
Eukaryota	Fungi	Saccharomyces cerevisiae	CAA42544.1	0.0	1157/1193
Eukaryota	Fungi	Vanderwaltozyma polyspora DSM 70294	XP_001643109.1	0.0	1160/1193
Eukaryota	Fungi	Saccharomyces cerevisiae YJM789	EDN62052.1	0.0	1157/1193
Eukaryota	Fungi	Saccharomyces cerevisiae EC1118	CAY79701.1	0.0	1157/1193
Eukaryota	Fungi	Kluyveromyces lactis NRRL Y-1140	XP_452456.1	0.0	1162/1193
Eukaryota	Fungi	Saccharomyces cerevisiae	NP_011453.1	0.0	1157/1193
Eukaryota	Fungi	Pichia pastoris	P78992.1	0.0	1167/1193
Eukaryota	Fungi	Saccharomyces cerevisiae	AAA34843.1	0.0	1157/1193
Eukaryota	Fungi	Ashbya gossypii ATCC 10895	NP_982705.1	0.0	1157/1193
Eukaryota	Fungi	Zygosaccharomyces rouxii CBS 732	XP_002495867.1	0.0	1163/1193
Eukaryota	Fungi	Vanderwaltozyma polyspora DSM 70294	XP_001645424.1	0.0	1154/1193
Eukaryota	Fungi	Cryptococcus neoformans var. neoformans	XP_774724.1	0.0	1148/1193
Eukaryota	Fungi	Ustilago maydis 521	XP_757201.1	0.0	1165/1193
Eukaryota	Fungi	Laccaria bicolor S238N-H82	XP_001883738.1	0.0	1152/1193
Eukaryota	Fungi	Malassezia globosa CBS 7966	XP_001730267.1	0.0	1164/1193
Eukaryota	Fungi	Coprinopsis cinerea okayama7#130	XP_001831593.1	0.0	1106/1193
Eukaryota	Fungi	Cryptococcus neoformans var. neoformans	XP_571482.1	0.0	1050/1193
Eukaryota	Fungi	Candida albicans SC5314	XP_721034.1	0.0	981/1193
Eukaryota	Fungi	Schizosaccharomyces japonicus yFS275	XP_002171743.1	0.0	1088/1193
Eukaryota	Metazoa	Branchiostoma floridae	XP_002596432.1	0.0	1141/1193
Bacteria	Proteobacteria	Plesiocystis pacifica SIR-1	ZP_01909586.1	0.0	1146/1193
Eukaryota	Metazoa	Caenorhabditis elegans	NP_505977.1	0.0	1156/1193
Eukaryota	Metazoa	Trichoplax adhaerens	XP_002115759.1	0.0	1145/1193
Eukaryota	Metazoa	Caenorhabditis briggsae AF16	XP_001673713.1	0.0	1158/1193
Eukaryota	Metazoa	Aedes aegypti	XP_001653941.1	0.0	1141/1193
Eukaryota	Metazoa	Nasonia vitripennis	XP_001600219.1	0.0	1170/1193
Eukaryota	Metazoa	Aedes aegypti	AAB64306.1	0.0	1157/1193
Eukaryota	Metazoa	Nematostella vectensis	XP_001640547.1	0.0	1180/1193
Eukaryota	Metazoa	Drosophila willistoni	XP_002061562.1	0.0	1158/1193
Eukaryota	Metazoa	Culex quinquefasciatus	XP_001844183.1	0.0	1160/1193
Eukaryota	Metazoa	Drosophila virilis	XP_002049926.1	0.0	1157/1193
Eukaryota	Metazoa	Anopheles gambiae str. PEST	XP_318078.2	0.0	1141/1193
Eukaryota	Metazoa	Anopheles gambiae str. PEST	XP_001689096.1	0.0	1157/1193
Eukaryota	Metazoa	Nematostella vectensis	XP_001640546.1	0.0	1136/1193
Eukaryota	Metazoa	Drosophila mojavensis	XP_002005916.1	0.0	1157/1193
Eukaryota	Metazoa	Drosophila grimshawi	XP_001986999.1	0.0	1158/1193

Eukaryota	Metazoa	Ciona intestinalis	XP_002125037.1	0.0	1161/1193
Eukaryota	Metazoa	Tribolium castaneum	XP_973877.1	0.0	1141/1193
Eukaryota	Metazoa	Drosophila melanogaster	NP_610527.1	0.0	1142/1193
Eukaryota	Metazoa	Drosophila pseudoobscura pseudoobscura	XP_001361830.1	0.0	1158/1193
Eukaryota	Metazoa	Drosophila ananassae	XP_001959980.1	0.0	1159/1193
Eukaryota	Metazoa	Danio rerio	AAI62583.1	0.0	1142/1193
Eukaryota	Metazoa	Drosophila yakuba	XP_002089853.1	0.0	1158/1193
Eukaryota	Metazoa	Drosophila melanogaster	NP_724841.1	0.0	1158/1193
Eukaryota	Metazoa	Drosophila erecta	XP_001969139.1	0.0	1158/1193
Eukaryota	Metazoa	Drosophila sechellia	XP_002045332.1	0.0	1158/1193
Eukaryota	Metazoa	Xenopus laevis	NP_001083226.1	0.0	1139/1193
Eukaryota	Metazoa	Danio rerio	NP_571625.1	0.0	1141/1193
Eukaryota	Metazoa	Pagrus major	BAC23138.1	0.0	1140/1193
Eukaryota	Metazoa	Drosophila virilis	XP_002049925.1	0.0	1138/1193
Eukaryota	Metazoa	Monodelphis domestica	XP_001364528.1	0.0	1139/1193
Eukaryota	Metazoa	Pediculus humanus corporis	XP_002432924.1	0.0	1163/1193
Eukaryota	Metazoa	Canis lupus familiaris	XP_852197.1	0.0	1169/1193
Eukaryota	Metazoa	Canis lupus familiaris	XP_540825.2	0.0	1161/1193
Eukaryota	Metazoa	Macaca mulatta	XP_001107749.1	0.0	1140/1193
Eukaryota	Metazoa	Bos taurus	Q29RK2.2	0.0	1140/1193
Eukaryota	Metazoa	Bos taurus	NP_808815.2	0.0	1140/1193
Eukaryota	Metazoa	Sus scrofa	NP_999514.1	0.0	1140/1193
Eukaryota	Metazoa	Drosophila grimshawi	XP_001986371.1	0.0	1155/1193
Eukaryota	Metazoa	Homo sapiens	NP_071504.2	0.0	1140/1193
Eukaryota	Metazoa	Bos taurus	AAO27903.1	0.0	1140/1193
Eukaryota	Metazoa	Rattus norvegicus	NP_036876.2	0.0	1140/1193
Eukaryota	Metazoa	Homo sapiens	AAA82937.1	0.0	1140/1193
Eukaryota	Metazoa	Mus musculus	BAE41902.1	0.0	1140/1193
Eukaryota	Metazoa	Mus musculus	NP_001156418.1	0.0	1140/1193
Eukaryota	Metazoa	Mus musculus	NP_032823.2	0.0	1140/1193
Eukaryota	Metazoa	Equus caballus	XP_001917468.1	0.0	1140/1193
Eukaryota	Metazoa	Mus musculus	BAE42943.1	0.0	1140/1193
Eukaryota	Metazoa	Mus musculus	Q05920.1	0.0	1140/1193
Eukaryota	Metazoa	Mus musculus	BAE28484.1	0.0	1140/1193
Eukaryota	Metazoa	Rattus norvegicus	AAA96256.1	0.0	1140/1193
Eukaryota	Metazoa	Gallus gallus	NP_989677.1	0.0	1147/1193
Eukaryota	Metazoa	Homo sapiens	AAB31500.1	0.0	1140/1193
Eukaryota	Metazoa	Drosophila mojavensis	XP_002005917.1	0.0	1134/1193
Eukaryota	Metazoa	Acyrthosiphon pisum	XP_001944200.1	0.0	1126/1193
Eukaryota	stramenopiles	Phaeodactylum tricornutum CCAP 1055/1	XP_002184364.1	0.0	1160/1193
Eukaryota	Viridiplantae	Micromonas sp. RCC299	XP_002503347.1	0.0	1153/1193
Eukaryota	Viridiplantae	Ostreococcus tauri	CAL54832.1	0.0	1156/1193
Eukaryota	Choanoflagellida	Monosiga brevicollis MX1	XP_001748229.1	0.0	1132/1193
Bacteria	Firmicutes	Geobacillus kaustophilus HTA426	YP_146932.1	0.0	1143/1193
Bacteria	Firmicutes	Geobacillus sp. WCH70	YP_002949107.1	0.0	1143/1193
Bacteria	Firmicutes	Geobacillus sp. Y412MC52	ZP_04391949.1	0.0	1143/1193
Bacteria	Firmicutes	Bacillus halodurans C-125	NP_243491.1	0.0	1145/1193
Eukaryota	Viridiplantae	Micromonas pusilla CCMP1545	EEH56848.1	0.0	1151/1193
Bacteria	Firmicutes	Geobacillus sp. Y4.1MC1	ZP_05371747.1	0.0	1143/1193
Bacteria	Firmicutes	Bacillus clausii KSM-K16	YP_175891.1	0.0	1148/1193
Bacteria	Firmicutes	Geobacillus thermodenitrificans NG80-2	YP_001125067.1	0.0	1143/1193
Eukaryota	Fungi	Uncinocarpus reesii 1704	XP_002543315.1	0.0	1093/1193
Bacteria	Firmicutes	Bacillus licheniformis ATCC 14580	YP_091292.1	0.0	1141/1193
Bacteria	Firmicutes	Thermosinus carboxydivorans Nor1	ZP_01665809.1	0.0	1139/1193
Bacteria	Firmicutes	Anoxybacillus flavithermus WK1	YP_002316217.1	0.0	1143/1193
Bacteria	Firmicutes	Geobacillus stearothermophilus	BAA12072.1	0.0	1143/1193
Bacteria	Firmicutes	Bacillus subtilis subsp. subtilis	NP_389369.1	0.0	1143/1193
Bacteria	Firmicutes	Bacillus cytotoxicus NVH 391-98	YP_001375878.1	0.0	1147/1193

Bacteria	Firmicutes	Bacillus thuringiensis serovar monterrey	ZP_04109956.1	0.0	1145/1193
Bacteria	Firmicutes	Bacillus anthracis str. Ames	NP_846395.1	0.0	1145/1193
Bacteria	Firmicutes	Bacillus cereus Rock3-44;	ZP_04218683.1	0.0	1147/1193
Bacteria	Firmicutes	Bacillus cereus 03BB102;	YP_002751307.1	0.0	1145/1193
Bacteria	Firmicutes	Bacillus methanolicus	AAY89103.1	0.0	1144/1193
Bacteria	Firmicutes	Bacillus thuringiensis serovar konkukian	YP_038009.1	0.0	1145/1193
Eukaryota	stramenopiles	Phaeodactylum tricornutum CCAP 1055/1	XP_002183906.1	0.0	1171/1193
Bacteria	Firmicutes	Bacillus cereus NVH0597-99;	ZP_03106164.1	0.0	1145/1193
Bacteria	Firmicutes	Bacillus thuringiensis str. Al	YP_896315.1	0.0	1145/1193
Bacteria	Firmicutes	Bacillus methanolicus	AAY89102.1	0.0	1142/1193
Eukaryota	Metazoa	Schistosoma mansoni	XP_002582167.1	0.0	1148/1193
Bacteria	Firmicutes	Bacillus cereus BGSC 6E1	ZP_04313356.1	0.0	1145/1193
Bacteria	Firmicutes	Bacillus cereus 95/8201;	ZP_04252667.1	0.0	1145/1193
Bacteria	Firmicutes	Bacillus cereus 03BB108;	ZP_03113000.1	0.0	1145/1193
Bacteria	Firmicutes	Bacillus cereus W;	ZP_03103306.1	0.0	1145/1193
Bacteria	Firmicutes	Bacillus thuringiensis serovar pondicheriensis	ZP_04092026.1	0.0	1145/1193
Bacteria	Firmicutes	Bacillus cereus ATCC 4342	ZP_04285601.1	0.0	1145/1193
Bacteria	Firmicutes	Bacillus anthracis str. A0442	ZP_02392096.1	0.0	1145/1193
Bacteria	Firmicutes	Bacillus cereus AH820;	YP_002452912.1	0.0	1145/1193
Bacteria	Firmicutes	Bacillus thuringiensis serovar andalousiensis	ZP_04098088.1	0.0	1145/1193
Bacteria	Firmicutes	Bacillus cereus Rock4-18;	ZP_04208686.1	0.0	1145/1193
Bacteria	Firmicutes	Bacillus cereus Rock3-29;	ZP_04229362.1	0.0	1145/1193
Bacteria	Firmicutes	Bacillus cereus E33L;	YP_085288.1	0.0	1145/1193
Bacteria	Firmicutes	Bacillus cereus G9241;	ZP_00236861.1	0.0	1145/1193
Bacteria	Firmicutes	Bacillus thuringiensis serovar pulsiensis	ZP_04080150.1	0.0	1145/1193
Bacteria	Firmicutes	Bacillus cereus Rock3-28;	ZP_04235176.1	0.0	1145/1193
Eukaryota	Viridiplantae	Ostreococcus lucimarinus CCE9901	XP_001419085.1	0.0	1127/1193
Bacteria	Firmicutes	Bacillus cereus ATCC 10987	NP_980289.1	0.0	1145/1193
Bacteria	Firmicutes	Bacillus cereus R309803;	ZP_04290838.1	0.0	1145/1193
Bacteria	Firmicutes	Bacillus cereus Q1;	YP_002531452.1	0.0	1145/1193
Bacteria	Firmicutes	Bacillus thuringiensis serovar tochigiensis	ZP_04147215.1	0.0	1145/1193
Bacteria	Firmicutes	Bacillus sp. SG-1	ZP_01860197.1	0.0	1143/1193
Bacteria	Firmicutes	Bacillus cereus AH187;	YP_002340000.1	0.0	1145/1193
Bacteria	Firmicutes	Bacillus anthracis str. A1055	ZP_05183338.1	0.0	1145/1193
Bacteria	Firmicutes	Bacillus cereus AH1134;	ZP_03231929.1	0.0	1145/1193
Bacteria	Firmicutes	Bacillus cereus G9842;	YP_002447514.1	0.0	1145/1193
Bacteria	Firmicutes	Bacillus cereus AH1271;	ZP_04187577.1	0.0	1145/1193
Bacteria	Firmicutes	Bacillus cereus F65185;	ZP_04204668.1	0.0	1145/1193
Bacteria	Firmicutes	Bacillus cereus BDRD-ST196;	ZP_04263564.1	0.0	1145/1193
Bacteria	Firmicutes	Bacillus thuringiensis serovar huazhongensis	ZP_04085997.1	0.0	1145/1193
Bacteria	Firmicutes	Bacillus thuringiensis serovar kurstaki	ZP_04116251.1	0.0	1145/1193
Bacteria	Firmicutes	Bacillus thuringiensis IBL 200	ZP_04073596.1	0.0	1145/1193
Bacteria	Firmicutes	Bacillus mycoides DSM 2048	ZP_04170296.1	0.0	1145/1193
Bacteria	Firmicutes	Bacillus weihenstephanensis KBAB4;	YP_001646570.1	0.0	1145/1193
Bacteria	Firmicutes	Bacillus thuringiensis serovar berliner	ZP_04103665.1	0.0	1145/1193
Bacteria	Firmicutes	Bacillus cereus ATCC 10876	ZP_04319123.1	0.0	1145/1193
Bacteria	Firmicutes	Bacillus amyloliquefaciens FZB42	YP_001421066.1	0.0	1142/1193
Bacteria	Firmicutes	Bacillus cereus ATCC 14579	NP_833666.1	0.0	1145/1193
Bacteria	Firmicutes	Bacillus thuringiensis serovar pakistani	ZP_04121866.1	0.0	1145/1193
Bacteria	Firmicutes	Bacillus cereus MM3;	ZP_04302143.1	0.0	1145/1193
Bacteria	Firmicutes	Bacillus cereus B4264;	YP_002368748.1	0.0	1145/1193
Bacteria	Firmicutes	Bacillus cereus AH1273;	ZP_04175983.1	0.0	1145/1193
Bacteria	Firmicutes	Staphylococcus hominis SK119	ZP_04060970.1	0.0	1146/1193
Bacteria	Firmicutes	Staphylococcus aureus subsp. aureus	YP_001332013.1	0.0	1154/1193
Bacteria	Firmicutes	Bacillus thuringiensis IBL 4222	ZP_04066607.1	0.0	1145/1193
Bacteria	Firmicutes	Bacillus cereus AH621;	ZP_04296408.1	0.0	1145/1193
Bacteria	Proteobacteria	Desulfuromonas acetoxidans DSM 684	ZP_01312174.1	0.0	1144/1193
Bacteria	Firmicutes	Brevibacillus brevis NBRC 100599	YP_002773577.1	0.0	1143/1193

Bacteria	Firmicutes	Listeria innocua Clip11262	NP_470397.1	0.0	1143/1193
Bacteria	Firmicutes	Oceanobacillus iheyensis HTE831	NP_692351.1	0.0	1141/1193
Eukaryota	stramenopiles	Thalassiosira pseudonana CCMP1335	XP_002294371.1	0.0	1153/1193
Bacteria	Firmicutes	Staphylococcus aureus subsp. aureus	YP_493712.1	0.0	1146/1193
Bacteria	Firmicutes	Staphylococcus aureus subsp. aureus	YP_185987.1	0.0	1146/1193
Bacteria	Firmicutes	Bacillus mycoides Rock3-17;	ZP_04158314.1	0.0	1147/1193
Bacteria	Firmicutes	Bacillus sp. NRRL B-14911	ZP_01172425.1	0.0	1143/1193
Bacteria	Firmicutes	Bacillus pseudomycoides DSM 12442	ZP_04152609.1	0.0	1147/1193
Bacteria	Firmicutes	Listeria monocytogenes HCC23	YP_002350516.1	0.0	1143/1193
Bacteria	Firmicutes	Staphylococcus aureus subsp. aureus	YP_040501.1	0.0	1146/1193
Bacteria	Firmicutes	Staphylococcus aureus A5948	ZP_05699955.1	0.0	1146/1193
Bacteria	Firmicutes	Staphylococcus aureus subsp. aureus	ZP_04867816.1	0.0	1146/1193
Eukaryota	Haptophyceae	Emiliania huxleyi	BAH22705.1	0.0	1181/1193
Bacteria	Firmicutes	Staphylococcus aureus A9635	ZP_05686504.1	0.0	1146/1193
Bacteria	Firmicutes	Staphylococcus aureus subsp. aureus	NP_371638.1	0.0	1146/1193
Bacteria	Firmicutes	Staphylococcus aureus A6300	ZP_05693302.1	0.0	1146/1193
Bacteria	Firmicutes	Staphylococcus aureus subsp. aureus	ZP_04018268.1	0.0	1146/1193
Bacteria	Firmicutes	Listeria welshimeri serovar 6b	YP_849249.1	0.0	1143/1193
Bacteria	Firmicutes	Staphylococcus aureus A9299	ZP_05689120.1	0.0	1146/1193
Bacteria	Firmicutes	Listeria monocytogenes FSL N1-017	ZP_05246000.1	0.0	1139/1193
Bacteria	Firmicutes	Listeria grayi DSM 20601	ZP_04443958.1	0.0	1151/1193
Bacteria	Firmicutes	Staphylococcus aureus subsp. aureus	ZP_04865890.1	0.0	1146/1193
Bacteria	Firmicutes	Staphylococcus aureus subsp. aureus	NP_645814.1	0.0	1146/1193
Bacteria	Firmicutes	Listeria monocytogenes FSL J1-194	ZP_05228659.1	0.0	1143/1193
Bacteria	Firmicutes	Listeria monocytogenes str. 4b	YP_013689.1	0.0	1143/1193
Bacteria	Firmicutes	Bacillus pumilus SAFR-032	YP_001486620.1	0.0	1137/1193
Bacteria	Proteobacteria	Anaeromyxobacter dehalogenans 2CP-C	YP_466222.1	0.0	1141/1193
Bacteria	Firmicutes	Listeria monocytogenes EGD-e	NP_464597.1	0.0	1143/1193
Bacteria	Firmicutes	Staphylococcus aureus RF122	YP_416463.1	0.0	1146/1193
Bacteria	Proteobacteria	Anaeromyxobacter dehalogenans 2CP-1	YP_002493616.1	0.0	1140/1193
Bacteria	Firmicutes	Veillonella parvula DSM 2008	ZP_03854901.1	0.0	1143/1193
Bacteria	Proteobacteria	Geobacter sulfurreducens PCA	NP_953474.1	0.0	1144/1193
Bacteria	Proteobacteria	Anaeromyxobacter sp. K	YP_002135464.1	0.0	1140/1193
Bacteria	Firmicutes	Staphylococcus haemolyticus JCSC1435	YP_253753.1	0.0	1146/1193
Bacteria	Firmicutes	Staphylococcus epidermidis M23864:W1	ZP_04818910.1	0.0	1147/1193
Bacteria	Firmicutes	Syntrophomonas wolfei subsp. wolfei	YP_753224.1	0.0	1140/1193
Bacteria	Firmicutes	Staphylococcus capitis SK14	ZP_03613876.1	0.0	1145/1193
Bacteria	Proteobacteria	Geobacter lovleyi SZ	YP_001952783.1	0.0	1142/1193
Eukaryota	Metazoa	Pagrus major	BAC44998.1	0.0	1027/1193
Bacteria	Exiguobacterium	Exiguobacterium sp. AT1b;	YP_002887164.1	0.0	1140/1193

AFUA_4G09420

Eukaryota	Fungi	Aspergillus fumigatus Af293	XP_751882.1	4 E-90	165/165
Eukaryota	Fungi	Neosartorya fischeri NRRL 181	XP_001267068.1	6 E-84	165/165
Eukaryota	Fungi	Talaromyces stipitatus ATCC 10500	XP_002480450.1	2 E-57	163/165
Eukaryota	Fungi	Aspergillus terreus NIH2624	XP_001209372.1	4 E-55	162/165
Eukaryota	Fungi	Penicillium marneffei ATCC 18224	XP_002144067.1	2 E-54	163/165
Eukaryota	Fungi	Candida albicans WO-1	EEQ43380.1	8 E-52	162/165
Eukaryota	Fungi	Pichia stipitis CBS 6054	XP_001383682.1	8 E-52	161/165
Eukaryota	Fungi	Candida dubliniensis CD36	XP_002421843.1	2 E-51	162/165
Eukaryota	Fungi	Gibberella zeae PH-1	XP_383146.1	6 E-51	154/165
Eukaryota	Fungi	Candida albicans SC5314	XP_715817.1	2 E-50	162/165
Eukaryota	Fungi	Nectria haematococca mpVI 77-13-4	EEU37120.1	6 E-49	159/165
Bacteria	Proteobacteria	Sorangium cellulosum 'So ce	YP_001611951.1	2 E-48	165/165
Eukaryota	Fungi	Nectria haematococca mpVI 77-13-4	EEU34094.1	3 E-48	158/165
Eukaryota	Fungi	Gibberella zeae PH-1	XP_390629.1	2 E-47	176/165
Eukaryota	Fungi	Nectria haematococca mpVI 77-13-4	EEU36360.1	4 E-46	159/165

Eukaryota	Fungi	Cadophora finlandica	ACP19538.1	2 E-43	155/165
Bacteria	Thermobaculum	Thermobaculum terrenum ATCC BAA-798	ZP_03857501.1	1 E-42	165/165
Bacteria	Chloroflexi	Roseiflexus castenholzii DSM 13941	YP_001431447.1	5 E-42	159/165
Eukaryota	Fungi	Podospora anserina DSM 980	XP_001911575.1	8 E-42	163/165
Eukaryota	Fungi	Botryotinia fuckeliana B05.10	XP_001561102.1	5 E-41	159/165
Eukaryota	Fungi	Neurospora crassa OR74A	XP_961715.1	6 E-41	162/165
Eukaryota	Fungi	Sordaria macrospora	CAT00782.1	7 E-41	165/165
Bacteria	Chloroflexi	Roseiflexus sp. RS-1	YP_001277992.1	1 E-40	135/165
Eukaryota	Fungi	Phaeosphaeria nodorum SN15	XP_001805507.1	1 E-40	160/165
Eukaryota	Fungi	Chaetomium globosum CBS 148.51	XP_001225170.1	5 E-40	163/165
Eukaryota	Fungi	Magnaporthe grisea 70-15	XP_364389.1	6 E-40	165/165
Bacteria	Firmicutes	Streptococcus equi subsp. zooepidemicus	YP_002745252.1	7 E-40	155/165
Bacteria	Firmicutes	Streptococcus equi subsp. zooepidemicus	YP_002122616.1	9 E-40	155/165
Bacteria	Firmicutes	Streptococcus sanguinis SK36	YP_001036072.1	1 E-39	160/165
Bacteria	Firmicutes	Streptococcus pyogenes MGAS9429	YP_595934.1	2 E-39	163/165
Bacteria	Firmicutes	Streptococcus pyogenes NZ131	YP_002285238.1	2 E-39	163/165
Bacteria	Firmicutes	Streptococcus pyogenes MGAS10394	YP_059550.1	2 E-39	163/165
Bacteria	Firmicutes	Streptococcus pyogenes MGAS10270	YP_597809.1	3 E-39	163/165
Bacteria	Firmicutes	Streptococcus pyogenes M1 GAS	NP_268596.1	3 E-39	163/165
Bacteria	Firmicutes	Streptococcus thermophilus LMG 18311	YP_140234.1	3 E-39	163/165
Bacteria	Firmicutes	Streptococcus pyogenes MGAS10750	YP_601690.1	4 E-39	163/165
Bacteria	Firmicutes	Streptococcus salivarius SK126	ZP_04062697.1	5 E-39	163/165
Bacteria	Firmicutes	Streptococcus pyogenes MGAS8232	NP_606509.1	8 E-39	163/165
Bacteria	Actinobacteria	Janibacter sp. HTCC2649	ZP_00995467.1	1 E-38	150/165
Bacteria	Firmicutes	Streptococcus pyogenes MGAS6180	YP_279664.1	1 E-38	163/165
Bacteria	Firmicutes	Streptococcus pyogenes str. Manfredo	YP_001127776.1	1 E-38	163/165
Bacteria	Firmicutes	Streptococcus pyogenes MGAS315	NP_663972.1	1 E-38	163/165
Eukaryota	Fungi	Uncinocarpus reesii 1704	XP_002543090.1	2 E-38	157/165
Bacteria	Firmicutes	Streptococcus dysgalactiae subsp. equisimilis	YP_002997668.1	4 E-38	152/165
Eukaryota	Fungi	Coccidioides posadasii C735 delta	EER28329.1	5 E-38	158/165
Bacteria	Firmicutes	Streptococcus uberis 0140J	YP_002562955.1	5 E-38	155/165
Eukaryota	Fungi	Coccidioides immitis RS;	XP_001241380.1	5 E-38	158/165
Bacteria	Firmicutes	Streptococcus agalactiae COH1	ZP_00786382.1	1 E-37	153/165
Bacteria	Firmicutes	Streptococcus agalactiae A909	YP_328835.1	1 E-37	153/165
Bacteria	Firmicutes	Streptococcus pneumoniae TIGR4	NP_344575.1	1 E-37	160/165
Bacteria	Firmicutes	Streptococcus pneumoniae SP6-BS73	ZP_01819205.1	1 E-37	160/165
Bacteria	Firmicutes	Streptococcus pneumoniae TIGR4	ZP_01408019.1	1 E-37	151/165
Bacteria	Firmicutes	Streptococcus pneumoniae CGSP14	YP_001834741.1	2 E-37	151/165
Bacteria	Firmicutes	Streptococcus suis 05ZYH33	YP_001199398.1	2 E-37	163/165
Bacteria	Actinobacteria	Frankia alni ACN14a	YP_715072.1	2 E-37	162/165
Bacteria	Firmicutes	Streptococcus agalactiae 2603V/R	NP_687147.1	2 E-37	153/165
Bacteria	Firmicutes	Streptococcus pneumoniae R6	NP_357620.1	2 E-37	151/165
Bacteria	Nitrospirae	Leptospirillum sp. Group II	EDZ39801.1	4 E-37	177/165
Bacteria	Firmicutes	Streptococcus agalactiae NEM316	NP_734580.1	5 E-37	153/165
Bacteria	Actinobacteria	Tsukamurella paurometabola DSM 20162	ZP_04028648.1	1 E-36	158/165
Bacteria	Actinobacteria	Nakamurella multipartita DSM 44233	YP_003203631.1	2 E-36	159/165
Bacteria	Actinobacteria	Mycobacterium abscessus	YP_001704853.1	4 E-36	150/165
Bacteria	Firmicutes	Streptococcus mutans UA159	NP_720781.1	4 E-36	151/165
Eukaryota	Fungi	Ajellomyces capsulatus G186AR	EEH03356.1	5 E-36	181/165
Bacteria	Firmicutes	Streptococcus mutans NN2025	BAH88648.1	5 E-36	151/165
Bacteria	Nitrospirae	Leptospirillum rubarum	EAY57989.1	1 E-35	177/165
Bacteria	Firmicutes	Streptococcus gordonii str. Challis	YP_001449494.1	1 E-35	160/165
Bacteria	Nitrospirae	Leptospirillum ferrodiazotrophum	EES52281.1	2 E-35	177/165
Bacteria	Actinobacteria	Rhodococcus jostii RHA1	YP_706337.1	2 E-35	153/165
Bacteria	Actinobacteria	Mycobacterium intracellulare ATCC 13950	ZP_05226266.1	2 E-35	158/165
Bacteria	Actinobacteria	Catenulispora acidiphila DSM 44928	YP_003113528.1	3 E-35	148/165
Bacteria	Actinobacteria	Rubrobacter xylanophilus DSM 9941	YP_645093.1	3 E-35	149/165
Bacteria	Actinobacteria	Rhodococcus opacus B4	YP_002783652.1	3 E-35	153/165

Bacteria	Actinobacteria	Mycobacterium sp. MCS	YP_641082.1	3 E-35	153/165
Eukaryota	Fungi	Ajellomyces capsulatus H143	EER44774.1	4 E-35	183/165
Eukaryota	Fungi	Ajellomyces capsulatus NAm1	XP_001537386.1	4 E-35	186/165
Bacteria	Firmicutes	Geobacillus sp. Y4.1MC1	ZP_05373312.1	4 E-35	177/165
Bacteria	Actinobacteria	Mycobacterium ulcerans Agy99	YP_907531.1	4 E-35	158/165
Eukaryota	Fungi	Pyrenophora tritici-repentis Pt-1C-BFP	XP_001931649.1	5 E-35	160/165
Bacteria	Actinobacteria	Gordonia bronchialis DSM 43247	ZP_03887211.1	1 E-34	150/165
Bacteria	Actinobacteria	Mycobacterium kansasii ATCC 12478	ZP_04748198.1	1 E-34	158/165
Bacteria	Firmicutes	Streptococcus infantarius subsp. infantarius	ZP_02920011.1	1 E-34	160/165
Bacteria	Actinobacteria	Mycobacterium avium subsp. paratuberculosis	NP_961421.1	2 E-34	158/165
Bacteria	Cyanobacteria	Cyanothece sp. PCC 7425	YP_002483538.1	3 E-34	174/165
Bacteria	Actinobacteria	Rhodococcus erythropolis PR4	YP_002765753.1	3 E-34	148/165
Bacteria	Proteobacteria	Methylococcus capsulatus str. Bath	YP_113395.1	4 E-34	178/165
Bacteria	Actinobacteria	Mycobacterium avium 104;	YP_880676.1	6 E-34	158/165
Bacteria	Actinobacteria	Mycobacterium gilvum PYR-GCK	YP_001133536.1	6 E-34	148/165
Bacteria	Actinobacteria	Rhodococcus erythropolis SK121	ZP_04384816.1	7 E-34	148/165
Eukaryota	Fungi	Paracoccidioides brasiliensis Pb18;	EEH44026.1	2 E-33	169/165
Bacteria	Actinobacteria	Mycobacterium vanbaalenii PYR-1	YP_955206.1	2 E-33	148/165
Bacteria	Proteobacteria	Beijerinckia indica subsp. indica	YP_001833004.1	2 E-33	178/165
Bacteria	Proteobacteria	Nitrosococcus oceani ATCC 19707	YP_343366.1	2 E-33	177/165
Bacteria	Proteobacteria	Nitrosococcus oceani AFC27	ZP_05048343.1	2 E-33	177/165
Bacteria	Actinobacteria	Mycobacterium smegmatis str. MC2	YP_889236.1	3 E-33	153/165
Bacteria	Verrucomicrobia	Methylacidiphilum infernorum V4	YP_001941020.1	3 E-33	180/165
Bacteria	Proteobacteria	Acidithiobacillus ferrooxidans ATCC 53993	YP_002219870.1	4 E-33	183/165
Bacteria	Proteobacteria	Psychromonas ingrahamii 37	YP_942938.1	5 E-33	179/165
Bacteria	Proteobacteria	Marinobacter sp. ELB17	ZP_01739190.1	5 E-33	179/165
Bacteria	Actinobacteria	Renibacterium salmoninarum ATCC 33209	YP_001624876.1	7 E-33	148/165
Bacteria	Cyanobacteria	Synechococcus elongatus PCC 7942	YP_398717.1	9 E-33	182/165
Bacteria	Verrucomicrobia	Verrucomicrobiae bacterium DG1235	ZP_05058548.1	1 E-32	178/165
Bacteria	Proteobacteria	Stigmatella aurantiaca DW4/3-1	ZP_01464073.1	2 E-32	177/165
Bacteria	Actinobacteria	Streptomyces clavuligerus ATCC 27064	ZP_05004681.1	4 E-32	151/165
Bacteria	Actinobacteria	Frankia sp. CcI3	YP_479334.1	5 E-32	161/165
Bacteria	Proteobacteria	gamma proteobacterium NOR51-B	ZP_04957227.1	6 E-32	177/165
Bacteria	Proteobacteria	Burkholderia sp. H160	ZP_03267608.1	8 E-32	184/165
Bacteria	Actinobacteria	Mycobacterium tuberculosis H37Rv;	NP_215800.1	1 E-31	158/165
Bacteria	Cyanobacteria	Fremyella diplosiphon Fd33	AAT41924.1	2 E-30	179/165
Bacteria	Firmicutes	Selenomonas flueggei ATCC 43531	ZP_04659246.1	5 E-30	168/165
Bacteria	Proteobacteria	Nitrobacter hamburgensis X14	YP_576228.1	2 E-29	174/165
Bacteria	Proteobacteria	Photorhabdus luminescens subsp. laumondii	NP_929505.1	3 E-29	183/165
Bacteria	Acidobacteria	Candidatus Koribacter versatilis Ellin345	YP_590677.1	5 E-28	160/165
Bacteria	Proteobacteria	Photorhabdus asymbiotica	YP_003039657.1	1 E-27	180/165
Bacteria	Actinobacteria	Streptomyces hygroscopicus ATCC 53653	ZP_05518557.1	4 E-26	149/165
Bacteria	Actinobacteria	Streptomyces avermitilis MA-4680	NP_827289.1	2 E-25	153/165
Bacteria	Actinobacteria	Streptomyces sp. SPB78	ZP_05486336.1	3 E-25	162/165
Bacteria	Actinobacteria	Streptosporangium roseum DSM 43021	ZP_04472006.1	7 E-25	164/165
Bacteria	Actinobacteria	Streptomyces viridochromogenes DSM 40736	ZP_05530865.1	1 E-24	153/165
Bacteria	Actinobacteria	Janibacter sp. HTCC2649	ZP_00993842.1	1 E-24	157/165
Bacteria	Actinobacteria	Streptomyces clavuligerus ATCC 27064	ZP_05006784.1	2 E-24	153/165
Bacteria	Actinobacteria	Streptomyces griseoflavus Tu4000	ZP_05541787.1	2 E-24	153/165
Bacteria	Actinobacteria	Streptomyces scabiei 87.22	CBG73789.1	2 E-24	153/165
Bacteria	Actinobacteria	Streptomyces ghanaensis ATCC 14672	ZP_04688753.1	3 E-24	153/165
Bacteria	Actinobacteria	Streptomyces albus J1074	ZP_04701504.1	3 E-24	153/165
Bacteria	Actinobacteria	Streptomyces sp. C	ZP_05506085.1	3 E-24	154/165
Eukaryota	Fungi	Paracoccidioides brasiliensis Pb01;	EEH39003.1	3 E-24	146/165
Eukaryota	Fungi	Paracoccidioides brasiliensis Pb03;	EEH19673.1	4 E-24	143/165
Bacteria	Actinobacteria	Frankia alni ACN14a	YP_711475.1	4 E-24	136/165
Bacteria	Actinobacteria	Streptomyces sviceus ATCC 29083	ZP_05021450.1	6 E-24	153/165
Bacteria	Actinobacteria	Frankia sp. CcI3	YP_479821.1	1 E-23	136/165

Bacteria	Actinobacteria	Streptomyces coelicolor A3(2)	NP_626352.1	1 E-23	153/165
Bacteria	Actinobacteria	Streptomyces sviceus ATCC 29083	ZP_05022703.1	2 E-23	158/165
Bacteria	Actinobacteria	Geodermatophilus obscurus DSM 43160	ZP_03890951.1	2 E-23	157/165
Bacteria	Actinobacteria	Frankia sp. EAN1pec	YP_001510156.1	3 E-23	136/165
Bacteria	Actinobacteria	Streptomyces sp. Mg1	ZP_04997959.1	4 E-23	153/165
Bacteria	Actinobacteria	Kineococcus radiotolerans SRS30216	YP_001360779.1	1 E-22	155/165
Bacteria	Actinobacteria	Streptomyces flavogriseus ATCC 33331	ZP_05806629.1	1 E-22	148/165
Bacteria	Actinobacteria	Saccharopolyspora erythraea NRRL 2338	YP_001102707.1	2 E-22	140/165
Bacteria	Actinobacteria	Streptomyces roseosporus NRRL 15998	ZP_04692516.1	4 E-22	154/165
Bacteria	Actinobacteria	Streptomyces sp. AA4	ZP_05483677.1	6 E-22	133/165
Bacteria	Actinobacteria	Streptomyces griseus subsp. griseus	YP_001826924.1	2 E-21	154/165
Bacteria	Actinobacteria	Saccharomonospora viridis DSM 43017	YP_003135317.1	4 E-21	133/165
Archaea	Euryarchaeota	Methanosphaera stadtmanae DSM 3091	YP_447632.1	4 E-21	145/165
Bacteria	Cyanobacteria	Cyanothece sp. PCC 7822	ZP_03157888.1	1 E-20	158/165
Bacteria	Actinobacteria	Acidothermus cellulolyticus 11B	YP_873430.1	1 E-20	155/165
Bacteria	Actinobacteria	Thermobifida fusca YX	YP_290188.1	6 E-20	161/165
Bacteria	Actinobacteria	Actinomyces urogenitalis DSM 15434	ZP_03927569.1	2 E-19	136/165
Bacteria	Actinobacteria	Kribbella flavida DSM 17836	ZP_03863924.1	2 E-19	158/165
Bacteria	Actinobacteria	Nakamurella multipartita DSM 44233	YP_003200261.1	5 E-19	147/165
Archaea	Euryarchaeota	Haloarcula marismortui ATCC 43049	YP_135635.1	6 E-19	156/165
Bacteria	Actinobacteria	Actinosynnema mirum DSM 43827	YP_003098159.1	8 E-19	133/165
Bacteria	Actinobacteria	Nocardiopsis dassonvillei subsp. dassonvillei	ZP_04336113.1	1 E-18	161/165
Archaea	Euryarchaeota	Haloquadratum walsbyi DSM 16790	YP_657802.1	4 E-18	155/165
Archaea	Euryarchaeota	Methanococcus maripaludis S2	NP_988419.1	4 E-18	143/165
Bacteria	Actinobacteria	Nocardioides sp. JS614	YP_924141.1	5 E-18	158/165
Archaea	Euryarchaeota	Methanococcus vannielii SB	YP_001323127.1	3 E-17	143/165
Archaea	Euryarchaeota	Methanococcus maripaludis C7	YP_001329763.1	3 E-17	143/165
Archaea	Crenarchaeota	Sulfolobus solfataricus P2	NP_342671.1	5 E-17	159/165
Archaea	Euryarchaeota	Methanococcus maripaludis C6	YP_001549420.1	6 E-17	143/165
Archaea	Crenarchaeota	Sulfolobus islandicus L.S.2.15	YP_002831649.1	8 E-17	159/165
Archaea	Euryarchaeota	Methanocaldococcus fervens AG86	YP_003127971.1	2 E-16	143/165
Archaea	Euryarchaeota	Methanococcus maripaludis C5	YP_001096825.1	4 E-16	143/165
Bacteria	Actinobacteria	Catenulispora acidiphila DSM 44928	YP_003114021.1	5 E-16	153/165
Eukaryota	Fungi	Talaromyces stipitatus ATCC 10500	XP_002480271.1	7 E-16	157/165
Bacteria	Actinobacteria	Mobiluncus curtisii ATCC 43063	ZP_03922744.1	2 E-15	157/165
Archaea	Euryarchaeota	Methanococcus vannielii SB	YP_001322729.1	2 E-15	143/165
Archaea	Euryarchaeota	Methanocaldococcus infernus ME	ZP_04789678.1	4 E-15	143/165
Bacteria	Proteobacteria	Geobacter sp. M21	YP_003022128.1	5 E-14	173/165
Bacteria	Firmicutes	Clostridium ramosum DSM 1402	ZP_02428106.1	6 E-14	155/165
Bacteria	Actinobacteria	Frankia alni ACN14a	YP_713017.1	7 E-14	164/165
Archaea	Euryarchaeota	Methanococcus aeolicus Nankai-3	YP_001324776.1	1 E-13	143/165
Bacteria	Actinobacteria	Catenulispora acidiphila DSM 44928	YP_003115078.1	2 E-13	163/165
Bacteria	Actinobacteria	Mycobacterium marinum M	YP_001850848.1	3 E-13	158/165
Bacteria	Bacteroidetes	Bacteroides plebeius DSM 17135	ZP_03208838.1	3 E-13	154/165
Bacteria	Actinobacteria	Mycobacterium marinum M	YP_001849733.1	4 E-13	158/165
Bacteria	Proteobacteria	Geobacter sp. FRC-32	YP_002537384.1	4 E-13	165/165
Bacteria	Proteobacteria	Kingella oralis ATCC 51147	ZP_04600902.1	6 E-13	182/165
Bacteria	Firmicutes	Bryantella formatexigens DSM 14469	ZP_05347955.1	6 E-13	173/165
Bacteria	Proteobacteria	Geobacter bemidjiensis Bem	YP_002138697.1	1 E-12	173/165
Bacteria	Bacteroidetes	Bacteroides sp. 2_2_4	ZP_04553761.1	1 E-12	165/165
Bacteria	Firmicutes	Clostridiales bacterium 1_7_47FAA	ZP_04669070.1	2 E-12	152/165
Bacteria	Firmicutes	Coprococcus comes ATCC 27758	ZP_03799259.1	2 E-12	173/165
Bacteria	Bacteroidetes	Bacteroides thetaiotaomicron VPI-5482	NP_810594.1	2 E-12	153/165
Archaea	Euryarchaeota	Methanococcus voltae A3	ZP_02193346.1	2 E-12	143/165
Bacteria	Firmicutes	Anaerofustis stercorihominis DSM 17244	ZP_02861314.1	2 E-12	150/165
Archaea	Euryarchaeota	Methanobrevibacter smithii ATCC 35061	YP_001273796.1	3 E-12	168/165
Bacteria	Firmicutes	Clostridium sp. SS2/1	ZP_02438854.1	3 E-12	150/165
Archaea	Euryarchaeota	Methanobrevibacter smithii DSM 2375	ZP_03608030.1	3 E-12	168/165

Bacteria	Firmicutes	Clostridium phytofermentans ISDg	YP_001558577.1	3 E-12	153/165
Archaea	Euryarchaeota	Methanobrevibacter smithii DSM 2374	ZP_05975341.1	4 E-12	168/165
Bacteria	Bacteroidetes	Bacteroides ovatus ATCC 8483	ZP_02067418.1	4 E-12	165/165
Bacteria	Bacteroidetes	Bacteroides finegoldii DSM 17565	ZP_05416837.1	5 E-12	153/165
Eukaryota	Fungi	Talaromyces stipitatus ATCC 10500	XP_002481470.1	5 E-12	179/165
Bacteria	Bacteroidetes	Bacteroides sp. D2	ZP_05759867.1	5 E-12	165/165
Bacteria	Firmicutes	Anaerostipes caccae DSM 14662	ZP_02418378.1	6 E-12	151/165
Bacteria	Firmicutes	Clostridium butyricum 5521	ZP_02950678.1	7 E-12	157/165
Archaea	Euryarchaeota	Methanothermobacter thermautotrophicus	AAC44811.1	7 E-12	138/165
Archaea	Euryarchaeota	Methanothermobacter thermautotrophicus str. D	NP_276694.1	8 E-12	138/165
Bacteria	Proteobacteria	Geobacter uraniireducens Rf4	YP_001232050.1	1 E-11	167/165
Bacteria	Proteobacteria	Geobacter sp. M18	ZP_05313714.1	1 E-11	173/165
Bacteria	Bacteroidetes	Bacteroides caccae ATCC 43185	ZP_01961152.1	1 E-11	153/165
Bacteria	Bacteroidetes	Bacteroides coprocola DSM 17136	ZP_03008800.1	1 E-11	153/165
Bacteria	Bacteroidetes	Prevotella melaninogenica ATCC 25845	ZP_04833234.1	1 E-11	178/165
Bacteria	Exiguobacterium	Exiguobacterium sibiricum 255-15;	YP_001813727.1	2 E-11	154/165
Bacteria	Exiguobacterium	Exiguobacterium sp. AT1b;	YP_002886743.1	3 E-11	150/165

AFUA_4G09440

Eukaryota	Fungi	Aspergillus fumigatus Af293	XP_751881.1	0.0	1048/1048
Eukaryota	Fungi	Neosartorya fischeri NRRL 181	XP_001267067.1	0.0	1033/1048
Eukaryota	Fungi	Aspergillus flavus NRRL3357	XP_002376874.1	0.0	1034/1048
Eukaryota	Fungi	Aspergillus nidulans FGSC A4	CBF85251.1	0.0	1032/1048
Eukaryota	Fungi	Aspergillus oryzae RIB40	XP_001821124.1	0.0	1043/1048
Eukaryota	Fungi	Aspergillus nidulans FGSC A4	XP_659232.1	0.0	1022/1048
Eukaryota	Fungi	Penicillium chrysogenum Wisconsin 54-1255	XP_002561735.1	0.0	1031/1048
Eukaryota	Fungi	Aspergillus niger CBS 513.88	XP_001393393.1	0.0	1029/1048
Eukaryota	Fungi	Coccidioides posadasii C735 delta	EER26862.1	0.0	1013/1048
Eukaryota	Fungi	Ajellomyces dermatitidis ER-3	EEQ89701.1	0.0	1008/1048
Eukaryota	Fungi	Ajellomyces capsulatus G186AR	EEH09703.1	0.0	1008/1048
Eukaryota	Fungi	Ajellomyces capsulatus NAm1	XP_001542093.1	0.0	1014/1048
Eukaryota	Fungi	Ajellomyces dermatitidis SLH14081	XP_002622113.1	0.0	1005/1048
Eukaryota	Fungi	Paracoccidioides brasiliensis Pb18;	EEH50593.1	0.0	1023/1048
Eukaryota	Fungi	Talaromyces stipitatus ATCC 10500	XP_002486686.1	0.0	955/1048
Eukaryota	Fungi	Neosartorya fischeri NRRL 181	XP_001257574.1	0.0	1015/1048
Eukaryota	Fungi	Paracoccidioides brasiliensis Pb03;	EEH15954.1	0.0	1033/1048
Eukaryota	Fungi	Uncinocarpus reesii 1704	XP_002543333.1	0.0	945/1048
Eukaryota	Fungi	Aspergillus oryzae RIB40	XP_001817024.1	0.0	1014/1048
Eukaryota	Fungi	Coccidioides immitis RS;	XP_001243667.1	0.0	946/1048
Eukaryota	Fungi	Aspergillus fumigatus Af293	XP_747708.1	0.0	1015/1048
Eukaryota	Fungi	Aspergillus clavatus NRRL 1	XP_001270181.1	0.0	1017/1048
Eukaryota	Fungi	Microsporum canis CBS 113480	EEQ27156.1	0.0	997/1048
Eukaryota	Fungi	Aspergillus nidulans FGSC A4	XP_664246.1	0.0	1018/1048
Eukaryota	Fungi	Aspergillus terreus NIH2624	XP_001209786.1	0.0	1016/1048
Eukaryota	Fungi	Penicillium chrysogenum Wisconsin 54-1255	XP_002569134.1	0.0	1025/1048
Eukaryota	Fungi	Penicillium chrysogenum Wisconsin 54-1255	XP_002563820.1	0.0	1023/1048
Eukaryota	Fungi	Aspergillus flavus NRRL3357	XP_002383034.1	0.0	951/1048
Eukaryota	Fungi	Paracoccidioides brasiliensis Pb01;	EEH34280.1	0.0	983/1048
Eukaryota	Fungi	Aspergillus terreus NIH2624	XP_001214443.1	0.0	875/1048
Eukaryota	Fungi	Aspergillus niger CBS 513.88	XP_001396722.1	0.0	1029/1048
Eukaryota	Fungi	Pyrenophora tritici-repentis Pt-1C-BFP	XP_001941857.1	0.0	1019/1048
Eukaryota	Fungi	Phaeosphaeria nodorum SN15	XP_001799925.1	0.0	1004/1048
Eukaryota	Fungi	Nectria haematococca mpVI 77-13-4	EEU41227.1	0.0	1028/1048
Eukaryota	Fungi	Chaetomium globosum CBS 148.51	XP_001219310.1	0.0	1041/1048
Eukaryota	Fungi	Neurospora crassa OR74A	XP_956354.1	0.0	1034/1048
Eukaryota	Fungi	Podospora anserina DSM 980	XP_001905220.1	0.0	1038/1048
Eukaryota	Fungi	Botryotinia fuckeliana B05.10	XP_001556812.1	0.0	1018/1048

Eukaryota	Fungi	Sclerotinia sclerotiorum 1980 UF-70	XP_001592311.1	0.0	1033/1048
Eukaryota	Fungi	Nectria haematococca mpVI 77-13-4	EEU48249.1	0.0	1025/1048
Eukaryota	Fungi	Magnaporthe grisea 70-15	XP_360418.1	0.0	1026/1048
Eukaryota	Fungi	Magnaporthe grisea 70-15	XP_365372.2	0.0	1020/1048
Eukaryota	Fungi	Fusarium oxysporum f. sp.	AAR01872.1	0.0	1028/1048
Eukaryota	Fungi	Gibberella zeae PH-1	XP_385095.1	0.0	1028/1048
Eukaryota	Fungi	Phaeosphaeria nodorum SN15	XP_001805854.1	0.0	996/1048
Eukaryota	Fungi	Botryotinia fuckeliana B05.10	XP_001549707.1	0.0	865/1048
Eukaryota	Fungi	Ustilago maydis	CAQ86600.1	0.0	1028/1048
Eukaryota	Fungi	Yarrowia lipolytica CLIB122	XP_499639.1	0.0	1016/1048
Eukaryota	Fungi	Nectria haematococca mpVI 77-13-4	EEU39439.1	0.0	1035/1048
Eukaryota	Fungi	Gibberella zeae PH-1	XP_382853.1	0.0	1012/1048
Eukaryota	Fungi	Nectria haematococca mpVI 77-13-4	EEU46760.1	0.0	1019/1048
Eukaryota	Fungi	Verticillium albo-atrum VaMs.102	EEY16846.1	0.0	1027/1048
Eukaryota	Fungi	Pichia pastoris GS115	XP_002490087.1	0.0	1018/1048
Eukaryota	Fungi	Microsporum canis CBS 113480	EEQ33554.1	0.0	905/1048
Eukaryota	Fungi	Ashbya gossypii ATCC 10895	NP_986570.1	0.0	1028/1048
Eukaryota	Fungi	Lachancea thermotolerans CBS 6340	XP_002554551.1	0.0	1042/1048
Eukaryota	Fungi	Candida glabrata CBS 138	XP_448738.1	0.0	1036/1048
Eukaryota	Fungi	Lodderomyces elongisporus NRRL YB-4239	XP_001526650.1	0.0	1021/1048
Eukaryota	Fungi	Chaetomium globosum CBS 148.51	XP_001222926.1	0.0	1031/1048
Eukaryota	Fungi	Pichia stipitis CBS 6054	XP_001387351.1	0.0	1019/1048
Eukaryota	Fungi	Candida albicans SC5314	XP_719032.1	0.0	1002/1048
Eukaryota	Fungi	Candida albicans WO-1	EEQ43097.1	0.0	1002/1048
Eukaryota	Fungi	Candida tropicalis MYA-3404	XP_002550010.1	0.0	1017/1048
Eukaryota	Fungi	Torulaspora delbrueckii	AAZ04389.1	0.0	1025/1048
Eukaryota	Fungi	Debaryomyces hansenii CBS767	XP_461943.1	0.0	1021/1048
Eukaryota	Fungi	Debaryomyces hansenii	CAG90411.2	0.0	1021/1048
Eukaryota	Fungi	Candida dubliniensis CD36	XP_002416711.1	0.0	1012/1048
Eukaryota	Fungi	Vanderwaltozyma polyspora DSM 70294	XP_001644531.1	0.0	1022/1048
Eukaryota	Fungi	Saccharomyces cerevisiae YJM789	EDN60386.1	0.0	1044/1048
Eukaryota	Fungi	Debaryomyces hansenii	AAK28385.2	0.0	1007/1048
Eukaryota	Fungi	Saccharomyces cerevisiae EC1118	CAY78549.1	0.0	1044/1048
Eukaryota	Fungi	Saccharomyces cerevisiae AWRI1631	EDZ73203.1	0.0	1044/1048
Eukaryota	Fungi	Debaryomyces hansenii	CAG85830.2	0.0	1007/1048
Eukaryota	Fungi	Talaromyces stipitatus ATCC 10500	XP_002341868.1	0.0	1036/1048
Eukaryota	Fungi	Saccharomyces cerevisiae	NP_010323.1	0.0	1044/1048
Eukaryota	Fungi	Saccharomyces cerevisiae	ACI43585.1	0.0	1044/1048
Eukaryota	Fungi	Saccharomyces cerevisiae	NP_010324.1	0.0	1041/1048
Eukaryota	Fungi	Debaryomyces hansenii CBS767	XP_457790.1	0.0	1007/1048
Eukaryota	Fungi	Pichia guilliermondii ATCC 6260	EDK38630.2	0.0	1005/1048
Eukaryota	Fungi	Saccharomyces cerevisiae YJM789	EDN60387.1	0.0	1041/1048
Eukaryota	Fungi	Saccharomyces cerevisiae RM11-1a	EDV08256.1	0.0	1041/1048
Eukaryota	Fungi	Pichia guilliermondii ATCC 6260	XP_001484999.1	0.0	1005/1048
Eukaryota	Fungi	Zygosaccharomyces rouxii CBS 732	XP_002499224.1	0.0	998/1048
Eukaryota	Fungi	Zygosaccharomyces rouxii	BAA11411.1	0.0	998/1048
Eukaryota	Fungi	Podospora anserina DSM 980	XP_001907578.1	0.0	1020/1048
Eukaryota	Fungi	Saccharomyces cerevisiae	NP_010325.1	0.0	1041/1048
Eukaryota	Fungi	Debaryomyces hansenii	AAK52600.2	0.0	1021/1048
Eukaryota	Fungi	Kluyveromyces lactis NRRL Y-1140	XP_454607.1	0.0	1021/1048
Eukaryota	Fungi	Penicillium marneffei ATCC 18224	XP_002151171.1	0.0	1027/1048
Eukaryota	Fungi	Kluyveromyces lactis NRRL Y-1140	XP_456007.1	0.0	1033/1048
Eukaryota	Fungi	Pichia stipitis CBS 6054	XP_001385604.2	0.0	961/1048
Eukaryota	Fungi	Yarrowia lipolytica CLIB122	XP_504141.1	0.0	1023/1048
Eukaryota	Fungi	Debaryomyces occidentalis	AAB86427.1	0.0	1010/1048
Eukaryota	Fungi	Coccidioides posadasii C735 delta	EER24302.1	0.0	1065/1048
Eukaryota	Fungi	Ajellomyces capsulatus G186AR	EEH07235.1	0.0	1049/1048
Eukaryota	Fungi	Debaryomyces occidentalis	AAB86426.1	0.0	1007/1048

Eukaryota	Fungi	Verticillium albo-atrum VaMs.102	EEY17441.1	0.0	855/1048
Eukaryota	Fungi	Uncinocarpus reesii 1704	XP_002541078.1	0.0	1067/1048
Eukaryota	Fungi	Candida tropicalis MYA-3404	XP_002550693.1	0.0	955/1048
Eukaryota	Fungi	Botryotinia fuckeliana B05.10	XP_001546156.1	0.0	1036/1048
Eukaryota	Fungi	Sclerotinia sclerotiorum 1980 UF-70	XP_001588929.1	0.0	1059/1048
Eukaryota	Fungi	Schizosaccharomyces pombe	NP_595246.1	0.0	1007/1048
Eukaryota	Fungi	Malassezia globosa CBS 7966	XP_001729255.1	0.0	987/1048
Eukaryota	Fungi	Candida albicans SC5314	XP_716992.1	0.0	945/1048
Eukaryota	Fungi	Candida dubliniensis CD36	XP_002421471.1	0.0	945/1048
Eukaryota	Fungi	Coccidioides immitis RS;	XP_001246801.1	0.0	985/1048
Eukaryota	Fungi	Nectria haematococca mpVI 77-13-4	EEU38936.1	0.0	1050/1048
Eukaryota	Fungi	Neurospora crassa OR74A	XP_962099.1	0.0	1042/1048
Eukaryota	Fungi	Podospora anserina DSM 980	XP_001903430.1	0.0	1052/1048
Eukaryota	Fungi	Chaetomium globosum CBS 148.51	XP_001224140.1	0.0	1060/1048
Eukaryota	Fungi	Magnaporthe grisea 70-15	XP_359699.1	0.0	1050/1048
Eukaryota	Fungi	Gibberella zeae PH-1	XP_384421.1	0.0	1052/1048
Eukaryota	Fungi	Microsporum canis CBS 113480	EEQ27293.1	0.0	970/1048
Eukaryota	Fungi	Verticillium albo-atrum VaMs.102	EEY21470.1	0.0	1030/1048
Eukaryota	Fungi	Ajellomyces dermatitidis ER-3	EEQ88153.1	0.0	964/1048
Eukaryota	Fungi	Ajellomyces dermatitidis SLH14081	XP_002627622.1	0.0	964/1048
Eukaryota	Fungi	Ajellomyces capsulatus G186AR	EEH07522.1	0.0	973/1048
Eukaryota	Fungi	Ajellomyces capsulatus H143	EER45674.1	0.0	973/1048
Eukaryota	Fungi	Podospora anserina DSM 980	XP_001904052.1	0.0	995/1048
Eukaryota	Fungi	Paracoccidioides brasiliensis Pb03;	EEH20843.1	0.0	968/1048
Eukaryota	Fungi	Schizosaccharomyces japonicus yFS275	XP_002175285.1	0.0	996/1048
Eukaryota	Fungi	Paracoccidioides brasiliensis Pb01;	EEH36659.1	0.0	968/1048
Eukaryota	Fungi	Ajellomyces dermatitidis ER-3	EEQ91163.1	0.0	979/1048
Eukaryota	Fungi	Ajellomyces dermatitidis SLH14081	XP_002629501.1	0.0	979/1048
Eukaryota	Fungi	Coccidioides posadasii C735 delta	EER24612.1	0.0	972/1048
Eukaryota	Fungi	Coccidioides immitis RS;	XP_001246391.1	0.0	972/1048
Eukaryota	Fungi	Neosartorya fischeri NRRL 181	XP_001265693.1	0.0	967/1048
Eukaryota	Fungi	Saccharomyces cerevisiae	CAA90778.1	0.0	917/1048
Eukaryota	Fungi	Saccharomyces cerevisiae	CAA98865.1	0.0	917/1048
Eukaryota	Fungi	Aspergillus fumigatus Af293	XP_749268.1	0.0	981/1048
Eukaryota	Fungi	Aspergillus fumigatus A1163	EDP53796.1	0.0	967/1048
Eukaryota	Fungi	Penicillium chrysogenum Wisconsin 54-1255	XP_002563111.1	0.0	962/1048
Eukaryota	Fungi	Aspergillus clavatus NRRL 1	XP_001273189.1	0.0	979/1048
Eukaryota	Fungi	Aspergillus flavus NRRL3357	XP_002378610.1	0.0	979/1048
Eukaryota	Fungi	Neurospora crassa	CAB65297.1	0.0	1010/1048
Eukaryota	Fungi	Uncinocarpus reesii 1704	XP_002540744.1	0.0	936/1048
Eukaryota	Fungi	Coprinopsis cinerea okayama7#130	XP_001837092.1	0.0	1024/1048
Eukaryota	Fungi	Magnaporthe grisea 70-15	XP_001404752.1	0.0	976/1048
Eukaryota	Fungi	Aspergillus oryzae RIB40	XP_001823251.1	0.0	967/1048
Eukaryota	Fungi	Gibberella zeae PH-1	XP_385171.1	0.0	959/1048
Eukaryota	Fungi	Ustilago maydis 521	XP_756351.1	0.0	991/1048
Eukaryota	Fungi	Nectria haematococca mpVI 77-13-4	EEU41274.1	0.0	958/1048
Eukaryota	Fungi	Hortaea werneckii;	ABD64571.1	0.0	979/1048
Eukaryota	Fungi	Coprinopsis cinerea okayama7#130	XP_001832079.1	0.0	1014/1048
Eukaryota	Fungi	Paracoccidioides brasiliensis Pb03;	EEH18274.1	0.0	894/1048
Eukaryota	Fungi	Aspergillus nidulans FGSC A4	CBF79858.1	0.0	948/1048
Eukaryota	Fungi	Hortaea werneckii;	ABD64570.1	0.0	993/1048
Eukaryota	Fungi	Chaetomium globosum CBS 148.51	XP_001228662.1	0.0	939/1048
Eukaryota	Fungi	Aspergillus nidulans FGSC A4	XP_680933.1	0.0	949/1048
Eukaryota	Fungi	Ajellomyces capsulatus NAm1	XP_001538484.1	0.0	861/1048
Eukaryota	Fungi	Neurospora crassa OR74A	XP_959558.2	0.0	984/1048
Eukaryota	Fungi	Pyrenophora tritici-repentis Pt-1C-BFP	XP_001939766.1	0.0	936/1048
Eukaryota	Fungi	Phaeosphaeria nodorum SN15	XP_001801404.1	0.0	946/1048
Eukaryota	Fungi	Aspergillus terreus NIH2624	XP_001216782.1	0.0	943/1048

Eukaryota	Viridiplantae	Physcomitrella patens subsp. patens	CAX20544.1	0.0	953/1048
Eukaryota	Viridiplantae	Physcomitrella patens subsp. patens	XP_001752194.1	0.0	953/1048
Eukaryota	Fungi	Cryptococcus neoformans var. neoformans	XP_566813.1	0.0	1039/1048
Eukaryota	Viridiplantae	Marchantia polymorpha	CAX27437.1	0.0	934/1048
Eukaryota	Viridiplantae	Marchantia polymorpha	CAX27440.1	0.0	936/1048
Eukaryota	Viridiplantae	Physcomitrella patens subsp. patens	XP_001760095.1	0.0	943/1048
Eukaryota	Viridiplantae	Physcomitrella patens	CAD91924.1	0.0	956/1048
Eukaryota	Viridiplantae	Physcomitrella patens subsp. patens	XP_001779936.1	0.0	956/1048
Eukaryota	Viridiplantae	Physcomitrella patens	CAD91919.1	0.0	956/1048
Eukaryota	Viridiplantae	Physcomitrella patens	CAD91920.1	0.0	956/1048
Eukaryota	Viridiplantae	Physcomitrella patens	CAD91923.1	0.0	956/1048
Eukaryota	Viridiplantae	Physcomitrella patens	CAD91918.1	0.0	924/1048
Eukaryota	Euglenozoa	Trypanosoma brucei TREU927	XP_827683.1	1 E-173	919/1048
Eukaryota	Euglenozoa	Trypanosoma cruzi strain CL	XP_817442.1	1 E-171	933/1048
Eukaryota	Euglenozoa	Trypanosoma cruzi strain CL	XP_817886.1	1 E-171	933/1048
Eukaryota	Euglenozoa	Leishmania donovani	AAC19126.1	1 E-169	956/1048
Eukaryota	Euglenozoa	Leishmania infantum JPCM5	XP_001469025.1	1 E-169	922/1048
Eukaryota	Euglenozoa	Leishmania braziliensis MHOM/BR/75/M2904	XP_001568308.1	1 E-168	922/1048
Eukaryota	Euglenozoa	Leishmania major strain Friedlin	XP_843313.1	1 E-167	956/1048
Bacteria	Firmicutes	Paenibacillus sp. JDR-2	YP_003012485.1	1 E-145	897/1048
Bacteria	Firmicutes	Caldicellulosiruptor saccharolyticus DSM 8903	YP_001179201.1	1 E-143	848/1048
Bacteria	Chlorobi	Chlorobaculum parvum NCIB 8327	YP_001998545.1	1 E-143	846/1048
Archaea	Euryarchaeota	Methanosarcina acetivorans C2A	NP_618950.1	1 E-142	900/1048
Bacteria	Chloroflexi	Roseiflexus castenholzii DSM 13941	YP_001430522.1	1 E-141	903/1048
Archaea	Euryarchaeota	Methanosarcina mazei Go1	NP_632859.1	1 E-141	892/1048
Bacteria	Chloroflexi	Chloroflexus aggregans DSM 9485	YP_002463491.1	1 E-141	864/1048
Bacteria	Firmicutes	Thermoanaerobacter pseudethanolicus ATCC 3[illegible]	YP_001664239.1	1 E-141	888/1048
Bacteria	Chloroflexi	Chloroflexus aurantiacus J-10-fl	YP_001634830.1	1 E-140	881/1048
Bacteria	Firmicutes	Thermoanaerobacter sp. X514	YP_001662309.1	1 E-140	888/1048
Bacteria	Firmicutes	Paenibacillus larvae subsp. larvae	ZP_02327910.1	1 E-140	870/1048
Bacteria	Firmicutes	Halothermothrix orenii H 168	YP_002509936.1	1 E-139	877/1048
Bacteria	Firmicutes	Thermoanaerobacter mathranii subsp. mathranii	ZP_05378441.1	1 E-138	888/1048
Bacteria	Firmicutes	Thermoanaerobacter italicus Ab9	ZP_05333104.1	1 E-138	888/1048
Archaea	Euryarchaeota	Methanosarcina barkeri str. Fusaro	YP_304062.1	1 E-138	888/1048
Bacteria	Firmicutes	Bacillus thuringiensis serovar kurstaki	ZP_04113077.1	1 E-138	881/1048
Bacteria	Firmicutes	Desulfitobacterium hafniense Y51	YP_518982.1	1 E-138	895/1048
Bacteria	Chlorobi	Chlorobium ferrooxidans DSM 13031	ZP_01385799.1	1 E-137	880/1048
Bacteria	Firmicutes	Bacillus thuringiensis serovar tochigiensis	ZP_04143863.1	1 E-137	875/1048
Bacteria	Thermotogae	Thermotoga lettingae TMO	YP_001469904.1	1 E-137	867/1048
Bacteria	Chlorobi	Chlorobium limicola DSM 245	YP_001943627.1	1 E-137	875/1048
Bacteria	Incertae Sedis	Symbiobacterium thermophilum IAM 14863	YP_075164.1	1 E-137	930/1048
Bacteria	Cyanobacteria	Nostoc azollae 0708	ZP_03764209.1	1 E-137	940/1048
Bacteria	Firmicutes	Bacillus cereus m1293;	ZP_04321570.1	1 E-137	875/1048
Bacteria	Firmicutes	Bacillus cereus G9241;	ZP_00237944.1	1 E-136	875/1048
Bacteria	Firmicutes	Syntrophomonas wolfei subsp. wolfei	YP_753920.1	1 E-136	886/1048
Bacteria	Firmicutes	Bacillus cereus F65185;	ZP_04201460.1	1 E-136	875/1048
Bacteria	Cyanobacteria	Cyanothece sp. PCC 7425	YP_002482969.1	1 E-136	934/1048
Bacteria	Proteobacteria	Pelobacter propionicus DSM 2379	YP_902877.1	1 E-136	888/1048
Bacteria	Firmicutes	Thermosinus carboxydivorans Nor1	ZP_01665540.1	1 E-136	888/1048
Bacteria	Firmicutes	Paenibacillus sp. oral taxon	ZP_04853102.1	1 E-136	899/1048
Bacteria	Firmicutes	Bacillus cereus B4264;	YP_002365248.1	1 E-136	875/1048
Bacteria	Firmicutes	Bacillus cereus ATCC 4342	ZP_04282292.1	1 E-136	875/1048
Bacteria	Firmicutes	Clostridium sporogenes ATCC 15579	ZP_02994099.1	1 E-136	860/1048
Bacteria	Firmicutes	Heliobacterium modesticaldum Ice1	YP_001680690.1	1 E-136	958/1048
Bacteria	Firmicutes	Bacillus cereus m1550;	ZP_04277051.1	1 E-136	875/1048
Bacteria	Firmicutes	Bacillus cereus ATCC 10987	NP_976846.1	1 E-136	875/1048
Bacteria	Firmicutes	Bacillus cereus Q1;	YP_002528257.1	1 E-136	875/1048
Bacteria	Firmicutes	Bacillus cereus Rock1-15;	ZP_04237696.1	1 E-135	875/1048

Bacteria	Firmicutes	Bacillus cereus AH676;	ZP_04190056.1	1 E-135	875/1048
Bacteria	Incertae Sedis	Anaerococcus tetradius ATCC 35098	ZP_03930914.1	1 E-135	880/1048
Bacteria	Firmicutes	Bacillus thuringiensis serovar berliner	ZP_04100329.1	1 E-135	875/1048
Bacteria	Chlorobi	Chlorobium phaeobacteroides DSM 266	YP_910664.1	1 E-135	875/1048
Bacteria	Firmicutes	Bacillus thuringiensis serovar huazhongensis	ZP_04082703.1	1 E-135	875/1048
Bacteria	Firmicutes	Bacillus cereus H3081.97;	ZP_03235268.1	1 E-135	875/1048
Bacteria	Firmicutes	Bacillus cereus ATCC 10876	ZP_04315709.1	1 E-135	875/1048
Bacteria	Firmicutes	Geobacillus sp. Y412MC10	YP_003242030.1	1 E-135	898/1048
Bacteria	Firmicutes	Bacillus cereus ATCC 14579	NP_830287.1	1 E-135	875/1048
Bacteria	Nitrospirae	Thermodesulfovibrio yellowstonii DSM 11347	YP_002248415.1	1 E-135	884/1048
Bacteria	Firmicutes	Bacillus cereus AH1134;	ZP_03229297.1	1 E-135	875/1048
Bacteria	Firmicutes	Bacillus thuringiensis serovar pakistani	ZP_04118663.1	1 E-135	875/1048
Bacteria	Firmicutes	Bacillus cereus AH1273;	ZP_04172766.1	1 E-134	875/1048
Bacteria	Firmicutes	Bacillus cereus AH1271;	ZP_04184392.1	1 E-134	875/1048
Bacteria	Firmicutes	Bacillus cereus AH1272;	ZP_04178553.1	1 E-134	875/1048
Bacteria	Cyanobacteria	Nostoc sp. PCC 7120	NP_487415.1	1 E-134	938/1048
Bacteria	Firmicutes	Pelotomaculum thermopropionicum SI	YP_001212351.1	1 E-134	880/1048
Bacteria	Firmicutes	Bacillus cereus 172560W;	ZP_04304385.1	1 E-134	875/1048

AFUA_4G09450

Eukaryota	Fungi	Aspergillus fumigatus Af293	XP_751880.1	4 E-94	172/172
Eukaryota	Fungi	Neosartorya fischeri NRRL 181	XP_001267066.1	6 E-40	159/172

AFUA_4G09560

Eukaryota	Fungi	Aspergillus fumigatus Af293	XP_751869.1	0.0	521/521
Eukaryota	Fungi	Neosartorya fischeri NRRL 181	XP_001267053.1	0.0	489/521
Eukaryota	Fungi	Aspergillus clavatus NRRL 1	XP_001271839.1	0.0	491/521
Eukaryota	Fungi	Aspergillus oryzae RIB40	XP_001817943.1	0.0	492/521
Eukaryota	Fungi	Aspergillus flavus NRRL3357	XP_002373107.1	0.0	492/521
Eukaryota	Fungi	Aspergillus terreus NIH2624	XP_001210879.1	0.0	485/521
Eukaryota	Fungi	Penicillium chrysogenum Wisconsin 54-1255	XP_002562293.1	1 E-175	459/521
Eukaryota	Fungi	Aspergillus nidulans FGSC A4	XP_659437.1	1 E-165	449/521
Eukaryota	Fungi	Ajellomyces dermatitidis SLH14081	XP_002624455.1	1 E-142	471/521
Eukaryota	Fungi	Ajellomyces dermatitidis ER-3	EEQ91531.1	1 E-141	471/521
Eukaryota	Fungi	Ajellomyces capsulatus NAm1	XP_001536241.1	1 E-136	476/521
Eukaryota	Fungi	Paracoccidioides brasiliensis Pb01;	EEH41133.1	1 E-134	475/521
Eukaryota	Fungi	Paracoccidioides brasiliensis Pb18;	EEH50338.1	1 E-132	474/521
Eukaryota	Fungi	Sclerotinia sclerotiorum 1980 UF-70	XP_001590789.1	1 E-128	485/521
Eukaryota	Fungi	Microsporum canis CBS 113480	EEQ32789.1	1 E-126	495/521
Eukaryota	Fungi	Paracoccidioides brasiliensis Pb03;	EEH17011.1	1 E-124	470/521
Eukaryota	Fungi	Paracoccidioides brasiliensis;	ABH03462.1	1 E-122	483/521
Eukaryota	Fungi	Pichia pastoris GS115	XP_002492744.1	1 E-116	436/521
Eukaryota	Fungi	Magnaporthe grisea 70-15	XP_363936.2	1 E-116	492/521
Eukaryota	Fungi	Pyrenophora tritici-repentis Pt-1C-BFP	XP_001932429.1	1 E-113	465/521
Eukaryota	Fungi	Phaeosphaeria nodorum SN15	XP_001793585.1	1 E-113	457/521
Eukaryota	Fungi	Pichia stipitis CBS 6054	XP_001386213.2	1 E-112	428/521
Eukaryota	Fungi	Gibberella zeae PH-1	XP_382424.1	1 E-110	464/521
Eukaryota	Fungi	Coccidioides immitis RS;	XP_001241167.1	1 E-110	442/521
Eukaryota	Fungi	Gibberella zeae PH-1	XP_386507.1	1 E-109	473/521
Eukaryota	Fungi	Coccidioides posadasii C735 delta	EER28227.1	1 E-107	495/521
Eukaryota	Fungi	Lodderomyces elongisporus NRRL YB-4239	XP_001523151.1	1 E-104	422/521
Eukaryota	Fungi	Candida tropicalis MYA-3404	XP_002549455.1	1 E-103	445/521
Eukaryota	Fungi	Nectria haematococca mpVI 77-13-4	EEU40564.1	1 E-103	449/521
Eukaryota	Fungi	Candida albicans SC5314	XP_715491.1	1 E-98	430/521
Eukaryota	Fungi	Candida albicans WO-1	EEQ44837.1	3 E-98	430/521

AFUA_4G13630

Eukaryota	Fungi	Aspergillus fumigatus Af293	XP_751468.1	0.0	1187/1187

AFUA_4G13765

Eukaryota	Fungi	Aspergillus fumigatus Af293	XP_001481572.1	1 E-128	227/227
Eukaryota	Fungi	Neosartorya fischeri NRRL 181	XP_001266673.1	1 E-114	227/227
Eukaryota	Fungi	Aspergillus clavatus NRRL 1	XP_001272227.1	1 E-77	219/227
Eukaryota	Fungi	Penicillium chrysogenum Wisconsin 54-1255	XP_002556622.1	3 E-69	215/227
Eukaryota	Fungi	Aspergillus niger CBS 513.88	XP_001393615.1	4 E-68	222/227
Eukaryota	Fungi	Aspergillus flavus NRRL3357	XP_002384871.1	2 E-49	225/227
Eukaryota	Fungi	Aspergillus oryzae RIB40	XP_001827630.1	9 E-49	225/227
Eukaryota	Fungi	Aspergillus niger CBS 513.88	XP_001400459.1	7 E-47	219/227
Bacteria	Proteobacteria	Rhodoferax ferrireducens T118	YP_525411.1	2 E-40	223/227
Eukaryota	Fungi	Chaetomium globosum CBS 148.51	XP_001225034.1	7 E-35	214/227
Eukaryota	Fungi	Ajellomyces dermatitidis ER-3	EEQ91274.1	1 E-34	215/227
Eukaryota	Fungi	Ajellomyces dermatitidis SLH14081	XP_002629388.1	2 E-34	215/227
Eukaryota	Fungi	Aspergillus oryzae RIB40	XP_001825535.1	5 E-33	219/227
Eukaryota	Fungi	Neosartorya fischeri NRRL 181	XP_001258292.1	6 E-33	219/227
Eukaryota	Fungi	Aspergillus flavus NRRL3357	XP_002381525.1	1 E-32	219/227
Eukaryota	Fungi	Penicillium chrysogenum Wisconsin 54-1255	XP_002562563.1	3 E-32	219/227
Eukaryota	Fungi	Cryptococcus neoformans var. neoformans	XP_571223.1	1 E-30	216/227
Bacteria	Proteobacteria	Rhodoferax ferrireducens T118	YP_525416.1	2 E-29	218/227
Eukaryota	Fungi	Aspergillus niger CBS 513.88	XP_001395669.1	9 E-28	207/227

AFUA_4G13770

Eukaryota	Fungi	Aspergillus fumigatus Af293	XP_751454.1	0.0	354/354
Eukaryota	Fungi	Neosartorya fischeri NRRL 181	XP_001266672.1	1 E-172	353/354
Eukaryota	Fungi	Aspergillus clavatus NRRL 1	XP_001272229.1	1 E-131	328/354
Eukaryota	Fungi	Penicillium chrysogenum Wisconsin 54-1255	XP_002557099.1	1 E-117	357/354
Eukaryota	Fungi	Penicillium marneffei ATCC 18224	XP_002150945.1	1 E-109	334/354
Eukaryota	Fungi	Aspergillus terreus NIH2624	XP_001214141.1	1 E-109	326/354
Eukaryota	Fungi	Microsporum canis CBS 113480	EEQ35001.1	1 E-107	351/354
Eukaryota	Fungi	Aspergillus nidulans FGSC A4	XP_660964.1	1 E-106	323/354
Eukaryota	Fungi	Talaromyces stipitatus ATCC 10500	XP_002483140.1	1 E-103	324/354
Bacteria	Actinobacteria	Streptomyces sp. AA4	ZP_05482814.1	9 E-83	356/354
Eukaryota	Fungi	Aspergillus niger CBS 513.88	XP_001391067.1	2 E-82	352/354
Bacteria	Actinobacteria	Renibacterium salmoninarum ATCC 33209	YP_001624514.1	1 E-78	323/354
Bacteria	Actinobacteria	Catenulispora acidiphila DSM 44928	YP_003113916.1	5 E-77	333/354
Bacteria	Firmicutes	Coprothermobacter proteolyticus DSM 5265	YP_002246485.1	3 E-34	316/354
Bacteria	Firmicutes	Lysinibacillus sphaericus C3-41	YP_001698115.1	4 E-33	310/354
Bacteria	Firmicutes	Bacillus sp. B14905	ZP_01723118.1	7 E-33	301/354
Bacteria	Firmicutes	Paenibacillus sp. JDR-2	YP_003011886.1	7 E-33	306/354
Bacteria	Firmicutes	Paenibacillus sp. oral taxon	ZP_04853176.1	3 E-32	317/354
Bacteria	Firmicutes	Thermoanaerobacter tengcongensis MB4	NP_621912.1	3 E-31	313/354
Bacteria	Firmicutes	Oceanobacillus iheyensis HTE831	NP_694128.1	3 E-31	318/354
Bacteria	Incertae Sedis	Symbiobacterium thermophilum IAM 14863	YP_074129.1	4 E-31	297/354
Bacteria	Firmicutes	Clostridium acetobutylicum ATCC 824	NP_346824.1	4 E-31	306/354
Sedis	Exiguobacterium	Exiguobacterium sibiricum 255-15;	YP_001813820.1	5 E-31	319/354
Bacteria	Firmicutes	Paenibacillus larvae subsp. larvae	ZP_02326240.1	7 E-31	322/354
Bacteria	Firmicutes	Paenibacillus sp. JDR-2	YP_003010221.1	9 E-31	321/354
Bacteria	Firmicutes	Clostridium phytofermentans ISDg	YP_001558242.1	1 E-30	316/354
Bacteria	Firmicutes	Bacillus amyloliquefaciens FZB42	YP_001419850.1	1 E-30	314/354
Bacteria	Firmicutes	Thermoanaerobacter italicus Ab9	ZP_05333001.1	2 E-30	303/354
Bacteria	Firmicutes	Geobacillus sp. G11MC16	ZP_03147184.1	2 E-30	305/354
Bacteria	Firmicutes	Geobacillus thermodenitrificans NG80-2	YP_001125404.1	3 E-30	305/354

Bacteria	Actinobacteria	Rubrobacter xylanophilus DSM 9941	YP_644752.1	5 E-30	297/354
Bacteria	Firmicutes	Thermoanaerobacter sp. X513	ZP_04802166.1	7 E-30	332/354
Bacteria	Firmicutes	Thermoanaerobacter sp. X514	YP_001661837.1	8 E-30	303/354
Bacteria	Firmicutes	Brevibacillus brevis NBRC 100599	YP_002774186.1	9 E-30	317/354
Bacteria	Actinobacteria	Clavibacter michiganensis subsp. michiganensis	YP_001220788.1	1 E-29	334/354
Bacteria	Firmicutes	Eubacterium dolichum DSM 3991	ZP_02078274.1	1 E-29	325/354
Bacteria	Firmicutes	Oceanobacillus iheyensis HTE831	NP_691191.1	1 E-29	300/354
Bacteria	Spirochaetes	Leptospira interrogans serovar Lai	NP_712743.1	1 E-29	299/354
Bacteria	Spirochaetes	Leptospira interrogans serovar Copenhageni	YP_001376.1	1 E-29	299/354
Bacteria	Firmicutes	Geobacillus sp. WCH70	YP_002949469.1	2 E-29	305/354
Bacteria	Firmicutes	Bacillus halodurans C-125	NP_241541.1	2 E-29	305/354
Bacteria	Firmicutes	Bacillus licheniformis ATCC 14580	YP_077449.1	2 E-29	314/354
Bacteria	Firmicutes	Clostridium thermocellum ATCC 27405	YP_001036753.1	2 E-29	291/354
Bacteria	Firmicutes	Thermoanaerobacter mathranii subsp. mathranii	ZP_05380021.1	2 E-29	313/354
Bacteria	Firmicutes	Desulfitobacterium hafniense DCB-2	YP_002457585.1	2 E-29	326/354
Bacteria	Firmicutes	Desulfitobacterium hafniense Y51	YP_520467.1	3 E-29	326/354
Bacteria	Exiguobacterium	Exiguobacterium sp. AT1b;	YP_002885774.1	6 E-29	311/354
Bacteria	Proteobacteria	Bdellovibrio bacteriovorus HD100	NP_970596.1	7 E-29	356/354
Archaea	Euryarchaeota	Halomicrobium mukohataei DSM 12286	YP_003178986.1	8 E-29	317/354
Bacteria	Firmicutes	Geobacillus sp. G11MC16	ZP_03149141.1	8 E-29	314/354
Bacteria	Firmicutes	Clostridium perfringens E str.	ZP_02632637.1	9 E-29	341/354
Bacteria	Proteobacteria	Sodalis glossinidius str. 'morsitans'	YP_455633.1	1 E-28	331/354
Bacteria	Firmicutes	Clostridium perfringens ATCC 13124	YP_694721.1	2 E-28	313/354
Bacteria	Proteobacteria	Bdellovibrio bacteriovorus HD100	NP_967163.1	2 E-28	304/354
Bacteria	Firmicutes	Geobacillus thermodenitrificans NG80-2	YP_001127327.1	2 E-28	314/354
Bacteria	Firmicutes	Alkaliphilus oremlandii OhILAs	YP_001513901.1	2 E-28	305/354
Bacteria	Spirochaetes	Borrelia recurrentis A1	YP_002223070.1	3 E-28	317/354
Bacteria	Firmicutes	Clostridium perfringens C str.	ZP_02864170.1	3 E-28	313/354
Bacteria	Firmicutes	Bacillus sp. NRRL B-14911	ZP_01168824.1	3 E-28	306/354
Bacteria	Firmicutes	Geobacillus sp. Y4.1MC1	ZP_05374173.1	4 E-28	306/354
Bacteria	Actinobacteria	Actinosynnema mirum DSM 43827	YP_003098088.1	4 E-28	310/354
Bacteria	Spirochaetes	Borrelia hermsii DAH	YP_001884042.1	4 E-28	317/354
Bacteria	Spirochaetes	Borrelia turicatae 91E135	YP_945610.1	5 E-28	317/354
Bacteria	Firmicutes	Bacillus pumilus SAFR-032	YP_001485413.1	6 E-28	314/354
Bacteria	Proteobacteria	Beijerinckia indica subsp. indica	YP_001832004.1	6 E-28	329/354
Bacteria	Firmicutes	Selenomonas sputigena ATCC 35185	ZP_05899806.1	6 E-28	300/354
Bacteria	Firmicutes	Clostridium perfringens str. 13	NP_561182.1	7 E-28	313/354
Bacteria	Actinobacteria	Stackebrandtia nassauensis DSM 44728	ZP_04484289.1	8 E-28	302/354
Bacteria	Firmicutes	Bacillus subtilis subsp. subtilis	NP_388047.1	1 E-27	315/354
Bacteria	Spirochaetes	Borrelia duttonii Ly	YP_002222261.1	1 E-27	317/354
Bacteria	Firmicutes	Paenibacillus sp. oral taxon	ZP_04852929.1	2 E-27	316/354
Bacteria	Firmicutes	Bacillus pumilus ATCC 7061	ZP_03056242.1	2 E-27	314/354
Bacteria	Firmicutes	Eubacterium dolichum DSM 3991	ZP_02078428.1	3 E-27	333/354
Bacteria	Firmicutes	Clostridium spiroforme DSM 1552	ZP_02867185.1	3 E-27	338/354
Bacteria	Proteobacteria	Vibrio coralliilyticus ATCC BAA-450	ZP_05885149.1	3 E-27	334/354
Bacteria	Firmicutes	Clostridium paraputrificum	BAC56177.1	4 E-27	319/354
Bacteria	Firmicutes	Holdemania filiformis DSM 12042	ZP_03634351.1	5 E-27	304/354
Bacteria	Thermobaculum	Thermobaculum terrenum ATCC BAA-798	ZP_03859171.1	5 E-27	321/354
Bacteria	Firmicutes	Geobacillus sp. Y412MC10	YP_003242159.1	5 E-27	318/354
Bacteria	Actinobacteria	marine actinobacterium PHSC20C1	ZP_01129732.1	5 E-27	299/354
Bacteria	Actinobacteria	Kribbella flavida DSM 17836	ZP_03859977.1	7 E-27	288/354
Bacteria	Cyanobacteria	Cyanothece sp. PCC 8801	YP_002371440.1	8 E-27	304/354
Bacteria	Proteobacteria	Pseudoalteromonas atlantica T6c	YP_663715.1	1 E-26	335/354
Bacteria	Proteobacteria	Nitrosomonas eutropha C91	YP_747973.1	1 E-26	291/354
Bacteria	Proteobacteria	Desulfurivibrio alkaliphilus AHT2	ZP_05711220.1	1 E-26	311/354
Bacteria	Firmicutes	Macrococcus caseolyticus JCSC5402	YP_002560691.1	1 E-26	329/354
Bacteria	Proteobacteria	Pseudoalteromonas haloplanktis TAC125	YP_341561.1	1 E-26	334/354
Bacteria	Firmicutes	Clostridium sp. 7_2_43FAA	ZP_05132017.1	1 E-26	293/354

Bacteria	Proteobacteria	Methylocella silvestris BL2	YP_002362544.1	1 E-26	296/354
Bacteria	Firmicutes	Clostridium spiroforme DSM 1552	ZP_02867814.1	2 E-26	316/354
Bacteria	Actinobacteria	Streptomyces avermitilis	ACB59179.1	2 E-26	302/354
Bacteria	Actinobacteria	Streptomyces avermitilis MA-4680	NP_824195.1	2 E-26	329/354
Bacteria	Spirochaetes	Treponema vincentii ATCC 35580	ZP_05623408.1	2 E-26	316/354
Bacteria	Firmicutes	Clostridium sp. 7_2_43FAA	ZP_05129838.1	2 E-26	319/354
Bacteria	Firmicutes	Bacillus coagulans 36D1	ZP_04431820.1	2 E-26	307/354
Bacteria	Fibrobacteres	Fibrobacter succinogenes subsp. succinogenes	YP_003249826.1	2 E-26	293/354
Bacteria	Actinobacteria	Frankia sp. EAN1pec	YP_001509540.1	2 E-26	300/354
Bacteria	Firmicutes	Lactobacillus salivarius UCC118	YP_536455.1	3 E-26	318/354
Bacteria	Proteobacteria	marine gamma proteobacterium HTCC2148	ZP_05096463.1	3 E-26	324/354
Bacteria	Actinobacteria	Streptomyces ghanaensis ATCC 14672	ZP_04688097.1	3 E-26	304/354
Bacteria	Proteobacteria	Stigmatella aurantiaca DW4/3-1	ZP_01465216.1	3 E-26	304/354
Bacteria	Firmicutes	Clostridium papyrosolvens DSM 2782	ZP_05496739.1	3 E-26	300/354
Bacteria	Proteobacteria	Geobacter lovleyi SZ	YP_001950963.1	4 E-26	335/354
Bacteria	Proteobacteria	Coxiella burnetii CbuG_Q212	YP_002303445.1	5 E-26	291/354
Bacteria	Actinobacteria	Streptomyces sp. AA4	ZP_05483762.1	5 E-26	302/354
Bacteria	Proteobacteria	Coxiella burnetii RSA 493	NP_820076.1	5 E-26	291/354
Bacteria	Proteobacteria	Bdellovibrio bacteriovorus	AAR17736.1	5 E-26	303/354
Bacteria	Actinobacteria	Streptomyces ghanaensis ATCC 14672	ZP_04683901.1	6 E-26	302/354
Bacteria	Proteobacteria	Glaciecola sp. HTCC2999	ZP_03560274.1	6 E-26	334/354
Bacteria	Actinobacteria	Streptomyces albus J1074	ZP_04702072.1	6 E-26	301/354
Bacteria	Firmicutes	Clostridium tetani E88	NP_782714.1	6 E-26	320/354
Bacteria	Spirochaetes	Borrelia burgdorferi B31	NP_212754.1	7 E-26	317/354
Bacteria	Spirochaetes	Borrelia burgdorferi 72a	ZP_03589447.1	9 E-26	317/354
Bacteria	Spirochaetes	Borrelia burgdorferi 156a	ZP_03436541.1	9 E-26	317/354
Bacteria	Spirochaetes	Borrelia burgdorferi 80a	ZP_03086822.1	9 E-26	317/354
Bacteria	Actinobacteria	Streptomyces roseosporus NRRL 15998	ZP_04696119.1	1 E-25	309/354
Bacteria	Tenericutes	Acholeplasma laidlawii PG-8A	YP_001620280.1	1 E-25	330/354
Bacteria	Proteobacteria	Coxiella burnetii 'MSU Goat	ZP_01946273.1	1 E-25	291/354
Bacteria	Actinobacteria	Streptomyces lividans TK24	ZP_05522609.1	1 E-25	306/354
Bacteria	Spirochaetes	Borrelia garinii PBi	YP_073061.1	1 E-25	317/354
Bacteria	Cyanobacteria	Microcoleus chthonoplastes PCC 7420	ZP_05025562.1	1 E-25	305/354
Bacteria	Cyanobacteria	Nostoc azollae 0708	ZP_03768092.1	1 E-25	295/354
Bacteria	Actinobacteria	Streptomyces ambofaciens ATCC 23877	CAJ88089.1	1 E-25	302/354
Bacteria	Firmicutes	Eubacterium hallii DSM 3353	ZP_03716458.1	2 E-25	293/354
Bacteria	Proteobacteria	Sulfurovum sp. NBC37-1	YP_001357764.1	2 E-25	361/354
Bacteria	Cyanobacteria	Cyanothece sp. PCC 7424	YP_002377168.1	2 E-25	311/354
Bacteria	Actinobacteria	Rubrobacter xylanophilus DSM 9941	YP_643411.1	2 E-25	303/354
Bacteria	Proteobacteria	Coxiella burnetii Dugway 5J108-111	YP_001424543.1	2 E-25	291/354
Bacteria	Firmicutes	Lactobacillus paracasei subsp. paracasei	ZP_04672298.1	2 E-25	311/354
Bacteria	Firmicutes	Lactobacillus rhamnosus Lc 705	YP_003175676.1	2 E-25	311/354
Bacteria	Spirochaetes	Borrelia burgdorferi 94a	ZP_03769945.1	2 E-25	317/354
Bacteria	Firmicutes	Clostridium sp. L2-50	ZP_02075497.1	2 E-25	309/354
Bacteria	Proteobacteria	Myxococcus xanthus DK 1622	YP_634739.1	2 E-25	324/354
Eukaryota	Viridiplantae	Micromonas sp. RCC299	XP_002502223.1	3 E-25	300/354
Bacteria	Proteobacteria	Mesorhizobium sp. CJ1	ABR12885.1	3 E-25	303/354
Bacteria	Spirochaetes	Borrelia garinii Far04	ZP_03540552.1	3 E-25	317/354
Bacteria	Actinobacteria	Streptomyces griseus subsp. griseus	YP_001823790.1	3 E-25	309/354
Bacteria	Firmicutes	Epulopiscium sp. 'N.t. morphotype	ZP_02691915.1	3 E-25	306/354
Bacteria	Incertae Sedis	Symbiobacterium thermophilum IAM 14863	YP_076327.1	3 E-25	315/354
Bacteria	Spirochaetes	Borrelia sp. SV1	ZP_03772753.1	3 E-25	317/354
Bacteria	Actinobacteria	Streptomyces griseus	BAB79300.1	3 E-25	309/354
Bacteria	Actinobacteria	Streptomyces coelicolor A3(2)	NP_630397.1	3 E-25	306/354
Bacteria	Firmicutes	Bacillus clausii KSM-K16	YP_176621.1	3 E-25	317/354
Bacteria	Firmicutes	Ruminococcus lactaris ATCC 29176	ZP_03167784.1	4 E-25	296/354
Bacteria	Chlorobi	Chlorobium chlorochromatii CaD3	YP_380263.1	4 E-25	315/354
Bacteria	Spirochaetes	Borrelia garinii PBr	ZP_03539263.1	4 E-25	317/354

Bacteria	Cyanobacteria	Nostoc punctiforme PCC 73102	YP_001866251.1	5 E-25	295/354
Bacteria	Firmicutes	Bacillus selenitireducens MLS10	ZP_02168805.1	5 E-25	303/354
Bacteria	Proteobacteria	Syntrophobacter fumaroxidans MPOB	YP_846636.1	6 E-25	312/354
Bacteria	Firmicutes	Clostridium spiroforme DSM 1552	ZP_02866995.1	6 E-25	338/354
Bacteria	Proteobacteria	Alcanivorax borkumensis SK2	YP_692738.1	6 E-25	305/354
Bacteria	Firmicutes	Streptococcus sanguinis SK36	YP_001035029.1	6 E-25	315/354
Bacteria	Firmicutes	Clostridium sp. SS2/1	ZP_02439655.1	6 E-25	294/354
Bacteria	Actinobacteria	Streptomyces sviceus ATCC 29083	ZP_05015787.1	8 E-25	306/354
Bacteria	Proteobacteria	Desulfotalea psychrophila LSv54	YP_066617.1	9 E-25	324/354
Bacteria	Actinobacteria	Streptomyces flavogriseus ATCC 33331	ZP_05803720.1	9 E-25	304/354
Bacteria	Spirochaetes	Borrelia afzelii PKo	YP_710068.1	9 E-25	317/354
Bacteria	Actinobacteria	Streptomyces flavogriseus ATCC 33331	ZP_05804726.1	9 E-25	309/354
Bacteria	Proteobacteria	Colwellia psychrerythraea 34H	YP_271256.1	1 E-24	332/354
Bacteria	Spirochaetes	Borrelia spielmanii A14S	ZP_03675148.1	1 E-24	319/354
Bacteria	Spirochaetes	Borrelia valaisiana VS116	ZP_03672259.1	1 E-24	319/354
Bacteria	Proteobacteria	Geobacter lovleyi SZ	YP_001950965.1	1 E-24	306/354
Bacteria	Thermus	Meiothermus ruber DSM 1279	ZP_04040009.1	1 E-24	317/354
Bacteria	Firmicutes	Mitsuokella multacida DSM 20544	ZP_05404229.2	2 E-24	318/354
Bacteria	Actinobacteria	Streptosporangium roseum DSM 43021	ZP_04471956.1	2 E-24	304/354
Bacteria	Firmicutes	Blautia hydrogenotrophica DSM 10507	ZP_03781398.1	2 E-24	300/354
Bacteria	Firmicutes	Eubacterium siraeum DSM 15702	ZP_02423873.1	2 E-24	301/354
Bacteria	Actinobacteria	Streptomyces clavuligerus ATCC 27064	ZP_05004225.1	2 E-24	316/354
Bacteria	Actinobacteria	Nocardiopsis dassonvillei subsp. dassonvillei	ZP_04332967.1	2 E-24	300/354
Bacteria	Actinobacteria	Streptomyces sp. SPB74	ZP_04992950.1	2 E-24	326/354
Bacteria	Actinobacteria	Streptomyces sp. Mg1	ZP_05000073.1	2 E-24	301/354
Bacteria	Proteobacteria	Alteromonadales bacterium TW-7	ZP_01613372.1	3 E-24	339/354
Bacteria	Proteobacteria	Sulfurimonas denitrificans DSM 1251	YP_394578.1	3 E-24	319/354
Bacteria	Proteobacteria	Desulfomicrobium baculatum DSM 4028	YP_003159419.1	3 E-24	321/354
Bacteria	Actinobacteria	Streptomyces thermoviolaceus	BAA32403.1	4 E-24	304/354
Bacteria	Actinobacteria	Streptomyces coelicolor A3(2)	NP_629382.1	4 E-24	311/354
Bacteria	Proteobacteria	Desulfovibrio salexigens DSM 2638	YP_002990692.1	4 E-24	363/354
Bacteria	Actinobacteria	Streptomyces lividans TK24	ZP_05523717.1	4 E-24	311/354
Bacteria	Chlorobi	Chloroherpeton thalassium ATCC 35110	YP_001995163.1	4 E-24	313/354
Bacteria	Proteobacteria	Pelobacter carbinolicus DSM 2380	YP_356737.1	4 E-24	315/354
Bacteria	Actinobacteria	Saccharomonospora viridis DSM 43017	YP_003135377.1	6 E-24	315/354
Bacteria	Proteobacteria	Xylella fastidiosa Dixon	ZP_00652055.1	7 E-24	330/354
Bacteria	Actinobacteria	Streptomyces sp. SPB78	ZP_05489674.1	7 E-24	311/354
Bacteria	Firmicutes	Roseburia inulinivorans DSM 16841	ZP_03754447.1	7 E-24	299/354
Bacteria	Proteobacteria	Desulfovibrio vulgaris str. Hildenborough	YP_011452.1	7 E-24	359/354
Bacteria	Cyanobacteria	Anabaena variabilis ATCC 29413	YP_325339.1	7 E-24	295/354
Bacteria	Firmicutes	Clostridium ramosum DSM 1402	ZP_02428963.1	8 E-24	330/354
Bacteria	Proteobacteria	Anaeromyxobacter dehalogenans 2CP-C	YP_467465.1	9 E-24	318/354
Bacteria	Tenericutes	Mollicutes bacterium D7	ZP_04565030.1	9 E-24	330/354
Bacteria	Firmicutes	Clostridium bolteae ATCC BAA-613	ZP_02089392.1	1 E-23	299/354
Bacteria	Firmicutes	Geobacillus sp. Y412MC10	YP_003244017.1	1 E-23	286/354
Bacteria	Firmicutes	Catenibacterium mitsuokai DSM 15897	ZP_03683733.1	1 E-23	312/354
Bacteria	Proteobacteria	Pelobacter propionicus DSM 2379	YP_902022.1	1 E-23	338/354
Bacteria	Firmicutes	Coprococcus eutactus ATCC 27759	ZP_02206011.1	1 E-23	287/354
Bacteria	Actinobacteria	Streptomyces roseosporus NRRL 15998	ZP_04693310.1	1 E-23	304/354
Bacteria	Cyanobacteria	Synechococcus sp. JA-2-3B'a(2-13)	YP_478540.1	1 E-23	308/354
Bacteria	Actinobacteria	Janibacter sp. HTCC2649	ZP_00994295.1	1 E-23	295/354
Bacteria	Actinobacteria	Janibacter sp. HTCC2649	ZP_00994410.1	1 E-23	303/354
Bacteria	Actinobacteria	Janibacter sp. HTCC2649	ZP_00996980.1	1 E-23	305/354
Bacteria	Firmicutes	Veillonella dispar ATCC 17748	ZP_04598734.1	2 E-23	298/354
Bacteria	Proteobacteria	Campylobacter curvus 525.92	YP_001408361.1	2 E-23	350/354
Bacteria	Actinobacteria	Streptomyces sp. Mg1	ZP_04999988.1	2 E-23	309/354
Bacteria	Firmicutes	Clostridiales bacterium 1_7_47FAA	ZP_04669557.1	2 E-23	296/354
Bacteria	Proteobacteria	Anaeromyxobacter sp. Fw109-5	YP_001381581.1	2 E-23	307/354

Bacteria	Proteobacteria	Haemophilus influenzae 6P18H1	ZP_04464121.1	2 E-23	293/354
Bacteria	Proteobacteria	Hyphomicrobium denitrificans ATCC 51888	ZP_05377536.1	2 E-23	326/354
Bacteria	Actinobacteria	Kribbella flavida DSM 17836	ZP_03865612.1	2 E-23	313/354
Bacteria	Actinobacteria	Streptomyces sp. C	ZP_05506627.1	2 E-23	301/354
Bacteria	Cyanobacteria	Cyanothece sp. ATCC 51142	YP_001806087.1	2 E-23	303/354
Bacteria	Actinobacteria	Streptomyces avermitilis MA-4680	NP_826479.1	3 E-23	304/354
Eukaryota	Fungi	Penicillium chrysogenum Wisconsin 54-1255	XP_002561976.1	3 E-23	313/354
Bacteria	Proteobacteria	Sorangium cellulosum 'So ce	YP_001614935.1	3 E-23	315/354
Bacteria	Actinobacteria	Streptomyces sviceus ATCC 29083	ZP_05023121.1	3 E-23	298/354
Bacteria	Actinobacteria	Collinsella stercoris DSM 13279	ZP_03298131.1	4 E-23	323/354
Bacteria	Chlorobi	Chlorobaculum parvum NCIB 8327	YP_001999587.1	4 E-23	312/354
Bacteria	Proteobacteria	Chromobacterium violaceum ATCC 12472	NP_899929.1	4 E-23	290/354
Bacteria	Proteobacteria	Anaeromyxobacter sp. K	YP_002136731.1	4 E-23	318/354
Bacteria	Proteobacteria	Desulfuromonas acetoxidans DSM 684	ZP_01312384.1	4 E-23	320/354
Bacteria	Proteobacteria	Haemophilus influenzae NT127	ZP_05849756.1	4 E-23	293/354
Bacteria	Chlorobi	Chlorobium tepidum TLS	NP_662998.1	5 E-23	302/354
Bacteria	Spirochaetes	Leptospira biflexa serovar Patoc	YP_001838361.1	5 E-23	287/354
Bacteria	Actinobacteria	Streptomyces pristinaespiralis ATCC 25486	ZP_05011919.1	6 E-23	304/354
Bacteria	Proteobacteria	Haemophilus influenzae 3655	ZP_01788080.1	6 E-23	293/354
Bacteria	Proteobacteria	Vibrio mimicus VM603	ZP_05720893.1	6 E-23	301/354
Bacteria	Cyanobacteria	Synechocystis sp. PCC 6803	NP_441754.1	7 E-23	311/354
Bacteria	Proteobacteria	Nitrosomonas europaea ATCC 19718	NP_842317.1	7 E-23	285/354

AFUA_4G13780

Eukaryota	Fungi	Aspergillus fumigatus Af293	XP_751453.1	3 E-84	151/151
Eukaryota	Fungi	Neosartorya fischeri NRRL 181	XP_001266671.1	7 E-74	169/151
Eukaryota	Fungi	Aspergillus clavatus NRRL 1	XP_001272230.1	3 E-46	168/151
Eukaryota	Fungi	Aspergillus niger CBS 513.88	XP_001393614.1	6 E-29	157/151
Eukaryota	Fungi	Aspergillus flavus NRRL3357	XP_002382379.1	1 E-28	169/151
Eukaryota	Fungi	Aspergillus oryzae RIB40	XP_001821641.1	1 E-27	169/151
Eukaryota	Fungi	Aspergillus flavus NRRL3357	XP_002379803.1	3 E-27	169/151
Eukaryota	Fungi	Penicillium chrysogenum Wisconsin 54-1255	XP_002560324.1	4 E-26	171/151
Eukaryota	Fungi	Aspergillus nidulans FGSC A4	XP_657835.1	7 E-26	167/151
Eukaryota	Fungi	Aspergillus terreus NIH2624	XP_001212742.1	2 E-23	170/151
Eukaryota	Fungi	Neosartorya fischeri NRRL 181	XP_001267634.1	4 E-23	154/151
Eukaryota	Fungi	Aspergillus clavatus NRRL 1	XP_001273482.1	9 E-21	156/151
Eukaryota	Fungi	Aspergillus clavatus NRRL 1	XP_001276726.1	4 E-20	173/151
Eukaryota	Fungi	Neurospora crassa OR74A	XP_001728070.1	4 E-19	157/151
Eukaryota	Fungi	Uncinocarpus reesii 1704	XP_002583089.1	4 E-19	156/151
Eukaryota	Fungi	Coccidioides posadasii C735 delta	EER24767.1	8 E-18	159/151
Eukaryota	Fungi	Coccidioides immitis RS;	XP_001243418.1	1 E-17	159/151
Eukaryota	Fungi	Aspergillus nidulans FGSC A4	CBF69359.1	2 E-17	157/151
Eukaryota	Fungi	Aspergillus nidulans FGSC A4	XP_664090.1	3 E-17	157/151
Eukaryota	Fungi	Botryotinia fuckeliana B05.10	XP_001559474.1	3 E-17	154/151
Eukaryota	Fungi	Penicillium chrysogenum Wisconsin 54-1255	XP_002565755.1	4 E-17	151/151
Eukaryota	Fungi	Aspergillus niger CBS 513.88	XP_001393824.1	1 E-16	158/151
Eukaryota	Fungi	Nectria haematococca mpVI 77-13-4	EEU41888.1	2 E-16	155/151
Eukaryota	Fungi	Uncinocarpus reesii 1704	XP_002544706.1	3 E-16	173/151
Eukaryota	Fungi	Aspergillus oryzae RIB40	XP_001818908.1	2 E-15	153/151
Eukaryota	Fungi	Magnaporthe grisea 70-15	XP_369295.2	5 E-15	153/151
Eukaryota	Fungi	Aspergillus flavus NRRL3357	XP_002379066.1	8 E-15	156/151
Eukaryota	Fungi	Aspergillus oryzae RIB40	XP_001823642.1	8 E-15	156/151
Eukaryota	Fungi	Talaromyces stipitatus ATCC 10500	XP_002483103.1	2 E-14	151/151
Eukaryota	Fungi	Penicillium marneffei ATCC 18224	XP_002146483.1	2 E-14	155/151
Eukaryota	Fungi	Uncinocarpus reesii 1704	XP_002541521.1	4 E-14	155/151
Eukaryota	Fungi	Botryotinia fuckeliana B05.10	XP_001545651.1	9 E-14	164/151
Eukaryota	Fungi	Paracoccidioides brasiliensis Pb03;	EEH21308.1	9 E-14	153/151

Eukaryota	Fungi	Aspergillus niger CBS 513.88	XP_001389342.1	1 E-13	153/151
Eukaryota	Fungi	Phaeosphaeria nodorum SN15	XP_001803687.1	2 E-13	156/151
Eukaryota	Fungi	Gibberella zeae PH-1	XP_382164.1	2 E-13	159/151
Eukaryota	Fungi	Paracoccidioides brasiliensis Pb01;	EEH36257.1	2 E-13	153/151
Eukaryota	Fungi	Penicillium marneffei ATCC 18224	XP_002143248.1	3 E-13	139/151
Eukaryota	Fungi	Aspergillus terreus NIH2624	XP_001208556.1	3 E-13	155/151
Eukaryota	Fungi	Phaeosphaeria nodorum SN15	XP_001794983.1	3 E-13	153/151
Eukaryota	Fungi	Verticillium albo-atrum VaMs.102	EEY21152.1	5 E-13	154/151
Eukaryota	Fungi	Chaetomium globosum CBS 148.51	XP_001227853.1	1 E-12	158/151
Eukaryota	Fungi	Verticillium albo-atrum VaMs.102	EEY15740.1	3 E-12	153/151
Eukaryota	Fungi	Ajellomyces dermatitidis ER-3	EEQ84436.1	3 E-12	162/151
Eukaryota	Fungi	Paracoccidioides brasiliensis Pb03;	EEH20362.1	3 E-12	163/151
Eukaryota	Fungi	Ajellomyces dermatitidis SLH14081	XP_002626603.1	5 E-12	163/151
Eukaryota	Fungi	Chaetomium globosum CBS 148.51	XP_001227696.1	6 E-12	151/151
Eukaryota	Fungi	Paracoccidioides brasiliensis Pb18;	EEH44766.1	7 E-12	163/151
Eukaryota	Fungi	Paracoccidioides brasiliensis Pb01;	EEH35553.1	7 E-12	163/151
Eukaryota	Fungi	Ajellomyces capsulatus H143	EER42061.1	8 E-12	162/151
Eukaryota	Fungi	Gibberella zeae PH-1	XP_391704.1	1 E-11	153/151
Eukaryota	Fungi	Ajellomyces capsulatus G186AR	EEH04242.1	1 E-11	162/151
Eukaryota	Fungi	Pyrenophora tritici-repentis Pt-1C-BFP	XP_001932707.1	1 E-11	154/151
Eukaryota	Fungi	Ajellomyces capsulatus NAm1	XP_001538894.1	2 E-11	162/151
Eukaryota	Fungi	Magnaporthe grisea 70-15	XP_367508.1	2 E-11	152/151

AFUA_4G13800

Eukaryota	Fungi	Aspergillus fumigatus Af293	XP_751452.2	0.0	406/406
Eukaryota	Fungi	Neosartorya fischeri NRRL 181	XP_001266669.1	0.0	406/406
Eukaryota	Fungi	Aspergillus terreus NIH2624	XP_001214142.1	0.0	403/406
Eukaryota	Fungi	Microsporum canis CBS 113480	EEQ30548.1	1 E-163	394/406
Eukaryota	Fungi	Chaetomium globosum CBS 148.51	XP_001226947.1	1 E-159	365/406
Eukaryota	Fungi	Nectria haematococca mpVI 77-13-4	EEU33886.1	1 E-152	367/406
Bacteria	Bacteroidetes	Pedobacter heparinus DSM 2366	YP_003092493.1	2 E-67	364/406
Bacteria	Planctomycetes	Planctomyces maris DSM 8797	ZP_01856792.1	1 E-34	358/406
Bacteria	Planctomycetes	Blastopirellula marina DSM 3645	ZP_01092135.1	9 E-34	339/406
Bacteria	Bacteroidetes	Pedobacter heparinus DSM 2366	YP_003091976.1	1 E-27	383/406
Bacteria	Actinobacteria	Saccharopolyspora erythraea NRRL 2338	YP_001107328.1	2 E-26	343/406
Bacteria	Lentisphaerae	Lentisphaera araneosa HTCC2155	ZP_01876025.1	1 E-25	339/406
Bacteria	Bacteroidetes	Flavobacteriales bacterium HTCC2170	ZP_01105517.1	5 E-25	353/406
Bacteria	Actinobacteria	Streptomyces avermitilis MA-4680	NP_827111.1	6 E-25	341/406
Bacteria	Actinobacteria	Streptomyces sp. AA4	ZP_05479105.1	2 E-24	325/406
Bacteria	Bacteroidetes	Spirosoma linguale DSM 74	ZP_04492889.1	2 E-24	352/406
Bacteria	Actinobacteria	Kribbella flavida DSM 17836	ZP_03862019.1	4 E-24	345/406
Bacteria	Actinobacteria	Streptomyces hygroscopicus ATCC 53653	ZP_05513783.1	4 E-24	353/406
Bacteria	Actinobacteria	Streptomyces avermitilis MA-4680	NP_826783.1	2 E-23	348/406
Bacteria	Verrucomicrobia	Opitutaceae bacterium TAV2	ZP_03723513.1	2 E-23	349/406
Bacteria	Actinobacteria	Streptomyces avermitilis MA-4680	NP_823018.1	1 E-22	352/406
Bacteria	Actinobacteria	Streptomyces viridochromogenes DSM 40736	ZP_05535391.1	2 E-22	352/406
Bacteria	Bacteroidetes	Dyadobacter fermentans DSM 18053	YP_003087400.1	3 E-22	371/406
Bacteria	Actinobacteria	Streptomyces lividans TK24	ZP_05522410.1	4 E-22	387/406
Bacteria	Bacteroidetes	Pedobacter heparinus DSM 2366	YP_003092480.1	1 E-21	353/406
Bacteria	Actinobacteria	Streptomyces sp. AA4	ZP_05482020.1	3 E-21	345/406
Bacteria	Actinobacteria	Streptomyces coelicolor A3(2)	NP_630638.1	6 E-21	387/406
Bacteria	Planctomycetes	Planctomyces limnophilus DSM 3776	ZP_04427442.1	8 E-21	340/406
Bacteria	Actinobacteria	Streptomyces viridochromogenes DSM 40736	ZP_05531364.1	1 E-20	348/406
Bacteria	Bacteroidetes	Sphingobacterium spiritivorum ATCC 33300	ZP_03970398.1	2 E-20	345/406
Bacteria	Bacteroidetes	Sphingobacterium spiritivorum ATCC 33861	ZP_04779737.1	3 E-20	345/406
Bacteria	Actinobacteria	Streptomyces sp. C	ZP_05508226.1	2 E-19	326/406
Bacteria	Bacteroidetes	Flavobacteriales bacterium HTCC2170	ZP_01105317.1	6 E-19	363/406

Bacteria	Actinobacteria	Stackebrandtia nassauensis DSM 44728	ZP_04485950.1	1 E-18	338/406
Bacteria	Planctomycetes	Rhodopirellula baltica SH 1	NP_865457.1	5 E-18	352/406
Bacteria	Actinobacteria	Streptomyces sviceus ATCC 29083	ZP_05021542.1	6 E-18	354/406
Bacteria	Verrucomicrobia	Verrucomicrobium spinosum DSM 4136	ZP_02926484.1	5 E-17	369/406
Bacteria	Bacteroidetes	Bacteroides uniformis ATCC 8492	ZP_02071388.1	4 E-16	353/406
Bacteria	Bacteroidetes	Parabacteroides merdae ATCC 43184	ZP_02031636.1	4 E-16	354/406
Bacteria	Verrucomicrobia	Opitutaceae bacterium TAV2	ZP_03723717.1	1 E-15	353/406
Bacteria	Actinobacteria	Streptomyces ghanaensis ATCC 14672	ZP_04684862.1	2 E-14	349/406
Bacteria	Actinobacteria	Streptomyces griseus subsp. griseus	YP_001827941.1	4 E-14	344/406
Bacteria	Bacteroidetes	Bacteroides sp. 3_2_5	ZP_04843806.1	6 E-14	354/406
Bacteria	Bacteroidetes	Bacteroides fragilis NCTC 9343	YP_213298.1	7 E-14	354/406
Bacteria	Bacteroidetes	Bacteroides fragilis YCH46	YP_101212.1	8 E-14	354/406
Bacteria	Planctomycetes	Rhodopirellula baltica SH 1	NP_868606.1	1 E-13	332/406
Bacteria	Bacteroidetes	Bacteroides coprocola DSM 17136	ZP_03010490.1	2 E-13	327/406
Bacteria	Bacteroidetes	Capnocytophaga ochracea DSM 7271	YP_003140143.1	2 E-13	338/406
Bacteria	Verrucomicrobia	Opitutaceae bacterium TAV2	ZP_03726339.1	4 E-13	362/406
Bacteria	Actinobacteria	Micromonospora viridifaciens	pdb1EURA	5 E-13	339/406
Bacteria	Actinobacteria	Micromonospora viridifaciens	Q02834.1	6 E-13	339/406
Bacteria	Actinobacteria	Micromonospora viridifaciens	pdb1EUTA	7 E-13	339/406
Bacteria	Bacteroidetes	Capnocytophaga sputigena ATCC 33612	ZP_03392334.1	7 E-13	338/406
Bacteria	Bacteroidetes	Prevotella sp. oral taxon	ZP_05917053.1	7 E-13	329/406
Bacteria	Bacteroidetes	Capnocytophaga canimorsus	ACA13563.1	1 E-12	335/406
Bacteria	Bacteroidetes	Bacteroides sp. 4_3_47FAA	ZP_05255794.1	1 E-12	354/406
Bacteria	Actinobacteria	Streptomyces pristinaespiralis ATCC 25486	ZP_05013561.1	1 E-12	349/406
Bacteria	Verrucomicrobia	Chthoniobacter flavus Ellin428	ZP_03130341.1	6 E-12	363/406
Bacteria	Bacteroidetes	Parabacteroides distasonis ATCC 8503	YP_001304276.1	8 E-12	329/406
Bacteria	Bacteroidetes	Parabacteroides sp. D13	ZP_05543775.1	8 E-12	329/406
Bacteria	Bacteroidetes	Bacteroides sp. 2_1_7	ZP_05285301.1	8 E-12	329/406
Bacteria	Bacteroidetes	Bacteroides plebeius DSM 17135	ZP_03206975.1	1 E-11	329/406
Bacteria	Bacteroidetes	Bacteroides stercoris ATCC 43183	ZP_02435734.1	3 E-11	339/406
Bacteria	Bacteroidetes	Bacteroides ovatus ATCC 8483	ZP_02063613.1	4 E-11	329/406
Bacteria	Bacteroidetes	Bacteroides thetaiotaomicron VPI-5482	NP_809368.1	4 E-11	329/406
Bacteria	Bacteroidetes	Bacteroides sp. 2_2_4	ZP_04548243.1	7 E-11	329/406
Bacteria	Bacteroidetes	Bacteroides sp. D2	ZP_05757165.1	9 E-11	329/406

AFUA_4G14130

Eukaryota	Fungi	Aspergillus fumigatus Af293	XP_751419.1	0.0	1297/1297
Eukaryota	Fungi	Neosartorya fischeri NRRL 181	XP_001266631.1	0.0	1297/1297
Eukaryota	Fungi	Aspergillus flavus NRRL3357	XP_002383625.1	0.0	1300/1297
Eukaryota	Fungi	Aspergillus oryzae RIB40	XP_001824955.1	0.0	1300/1297
Eukaryota	Fungi	Aspergillus flavus	AAB88655.1	0.0	1300/1297
Eukaryota	Fungi	Aspergillus niger CBS 513.88	XP_001402192.1	0.0	1294/1297
Eukaryota	Fungi	Aspergillus terreus NIH2624	XP_001208711.1	0.0	1285/1297
Eukaryota	Fungi	Neosartorya fischeri NRRL 181	XP_001266028.1	0.0	1271/1297
Eukaryota	Fungi	Aspergillus fumigatus	AAB88657.1	0.0	1272/1297
Eukaryota	Fungi	Aspergillus fumigatus Af293	XP_754025.1	0.0	1272/1297
Eukaryota	Fungi	Aspergillus clavatus NRRL 1	XP_001273942.1	0.0	1271/1297
Eukaryota	Fungi	Emericella nidulans	AAD43626.1	0.0	1264/1297
Eukaryota	Fungi	Talaromyces stipitatus ATCC 10500	XP_002482272.1	0.0	1307/1297
Eukaryota	Fungi	Aspergillus niger CBS 513.88	XP_001398406.1	0.0	1262/1297
Eukaryota	Fungi	Aspergillus oryzae RIB40	XP_001817075.1	0.0	1275/1297
Eukaryota	Fungi	Penicillium chrysogenum Wisconsin 54-1255	XP_002562681.1	0.0	1272/1297
Eukaryota	Fungi	Aspergillus nidulans FGSC A4	XP_659904.1	0.0	1259/1297
Eukaryota	Fungi	Penicillium marneffei ATCC 18224	XP_002148014.1	0.0	1289/1297
Eukaryota	Fungi	Coccidioides immitis RS;	XP_001242301.1	0.0	1274/1297
Eukaryota	Fungi	Coccidioides posadasii C735 delta	EER27407.1	0.0	1270/1297
Eukaryota	Fungi	Aspergillus terreus NIH2624	XP_001218046.1	0.0	1271/1297

Eukaryota	Fungi	Microsporum canis CBS 113480	EEQ28926.1	0.0	1280/1297
Eukaryota	Fungi	Trichophyton rubrum	AAG01549.3	0.0	1268/1297
Eukaryota	Fungi	Paracoccidioides brasiliensis Pb18;	EEH49756.1	0.0	1297/1297
Eukaryota	Fungi	Paracoccidioides brasiliensis Pb03;	EEH23309.1	0.0	1297/1297
Eukaryota	Fungi	Paracoccidioides brasiliensis Pb01;	EEH36917.1	0.0	1297/1297
Eukaryota	Fungi	Ajellomyces capsulatus G186AR	EEH10458.1	0.0	1289/1297
Eukaryota	Fungi	Ajellomyces dermatitidis ER-3	EEQ85957.1	0.0	1274/1297
Eukaryota	Fungi	Ajellomyces dermatitidis SLH14081	XP_002621139.1	0.0	1274/1297
Eukaryota	Fungi	Sclerotinia sclerotiorum 1980 UF-70	XP_001591841.1	0.0	1271/1297
Eukaryota	Fungi	Nectria haematococca mpVI 77-13-4	EEU46536.1	0.0	1252/1297
Eukaryota	Fungi	Aspergillus clavatus NRRL 1	XP_001273593.1	0.0	1269/1297
Eukaryota	Fungi	Magnaporthe grisea 70-15	XP_369103.2	0.0	1259/1297
Eukaryota	Fungi	Podospora anserina DSM 980	XP_001908137.1	0.0	1273/1297
Eukaryota	Fungi	Aspergillus flavus NRRL3357	XP_002377398.1	0.0	1269/1297
Eukaryota	Fungi	Uncinocarpus reesii 1704	XP_002544671.1	0.0	1239/1297
Eukaryota	Fungi	Microsporum canis CBS 113480	EEQ34671.1	0.0	1287/1297
Eukaryota	Fungi	Aspergillus oryzae RIB40	XP_001825800.1	0.0	1269/1297
Eukaryota	Fungi	Neosartorya fischeri NRRL 181	XP_001261790.1	0.0	1269/1297
Eukaryota	Fungi	Venturia inaequalis	AAL57243.1	0.0	1268/1297
Eukaryota	Fungi	Neurospora crassa OR74A	XP_959059.2	0.0	1275/1297
Eukaryota	Fungi	Verticillium albo-atrum VaMs.102	EEY16463.1	0.0	1205/1297
Eukaryota	Fungi	Penicillium chrysogenum Wisconsin 54-1255	XP_002557969.1	0.0	1265/1297
Eukaryota	Fungi	Microsporum canis CBS 113480	EEQ28924.1	0.0	1203/1297
Eukaryota	Fungi	Penicillium marneffei ATCC 18224	XP_002146111.1	0.0	1246/1297
Eukaryota	Fungi	Aspergillus terreus NIH2624	XP_001216792.1	0.0	1220/1297
Eukaryota	Fungi	Talaromyces stipitatus ATCC 10500	XP_002480836.1	0.0	1244/1297
Eukaryota	Fungi	Pyrenophora tritici-repentis Pt-1C-BFP	XP_001932865.1	0.0	1247/1297
Eukaryota	Fungi	Nectria haematococca mpVI 77-13-4	EEU33797.1	0.0	1241/1297
Eukaryota	Fungi	Penicillium chrysogenum Wisconsin 54-1255	XP_002564092.1	0.0	1257/1297
Eukaryota	Fungi	Yarrowia lipolytica CLIB122	XP_499981.1	0.0	1244/1297
Eukaryota	Fungi	Microsporum canis CBS 113480	EEQ27964.1	0.0	1259/1297
Eukaryota	Fungi	Schizosaccharomyces pombe	NP_588265.1	0.0	1298/1297
Eukaryota	Fungi	Schizosaccharomyces pombe	BAA01537.1	0.0	1298/1297
Eukaryota	Fungi	Yarrowia lipolytica CLIB122	XP_500789.1	0.0	1252/1297
Eukaryota	Fungi	Phaeosphaeria nodorum SN15	XP_001796035.1	0.0	1181/1297
Eukaryota	Fungi	Schizosaccharomyces japonicus yFS275	XP_002174515.1	0.0	1249/1297
Eukaryota	Fungi	Yarrowia lipolytica CLIB122	XP_505651.1	0.0	1253/1297
Eukaryota	Fungi	Pichia pastoris	CAY67034.1	0.0	1249/1297
Eukaryota	Fungi	Pichia pastoris GS115	XP_002490931.1	0.0	1250/1297
Eukaryota	Fungi	Leptosphaeria maculans	AAR11078.1	0.0	1259/1297
Eukaryota	Fungi	Laccaria bicolor S238N-H82	XP_001889044.1	0.0	1281/1297
Eukaryota	Fungi	Coprinopsis cinerea okayama7#130	XP_001835937.1	0.0	1259/1297
Eukaryota	Fungi	Coprinopsis cinerea okayama7#130	XP_001832249.1	0.0	1270/1297
Eukaryota	Fungi	Laccaria bicolor S238N-H82	XP_001876178.1	0.0	1258/1297
Eukaryota	Fungi	Aspergillus fumigatus Af293	XP_747730.1	0.0	1270/1297
Eukaryota	Fungi	Aspergillus fumigatus A1163	EDP47635.1	0.0	1270/1297
Eukaryota	Fungi	Gibberella zeae PH-1	XP_381860.1	0.0	1245/1297
Eukaryota	Fungi	Neosartorya fischeri NRRL 181	XP_001263980.1	0.0	1268/1297
Eukaryota	Fungi	Laccaria bicolor S238N-H82	XP_001881840.1	0.0	1266/1297
Eukaryota	Fungi	Coccidioides posadasii C735 delta	EER24151.1	0.0	1231/1297
Eukaryota	Fungi	Coprinopsis cinerea okayama7#130	XP_001841011.1	0.0	1257/1297
Eukaryota	Fungi	Coccidioides immitis RS;	XP_001247009.1	0.0	1231/1297
Eukaryota	Fungi	Ustilago maydis 521	XP_762156.1	0.0	1297/1297
Eukaryota	Fungi	Microsporum canis CBS 113480	EEQ30704.1	0.0	1238/1297
Eukaryota	Fungi	Uncinocarpus reesii 1704	XP_002541236.1	0.0	1197/1297
Eukaryota	Fungi	Microsporum canis CBS 113480	EEQ32397.1	0.0	1107/1297
Eukaryota	Fungi	Aspergillus flavus NRRL3357	XP_002381514.1	0.0	1177/1297
Eukaryota	Fungi	Malassezia globosa CBS 7966	XP_001731885.1	0.0	1261/1297

Eukaryota	Fungi	Filobasidiella neoformans	AAC49889.1	0.0	1268/1297
Eukaryota	Fungi	Cryptococcus neoformans var. neoformans	XP_567121.1	0.0	1268/1297
Eukaryota	Fungi	Pyrenophora tritici-repentis Pt-1C-BFP	XP_001935446.1	0.0	1230/1297
Eukaryota	Fungi	Ajellomyces capsulatus G186AR	EEH08704.1	0.0	1259/1297
Eukaryota	Fungi	Gibberella zeae PH-1	XP_382962.1	0.0	1182/1297
Eukaryota	Fungi	Neosartorya fischeri NRRL 181	XP_001262902.1	0.0	1262/1297
Eukaryota	Metazoa	Pan troglodytes	XP_001163380.1	0.0	1227/1297
Eukaryota	Metazoa	Macaca fascicularis	AAN07780.2	0.0	1244/1297
Eukaryota	Metazoa	Cricetulus griseus	P21448.2	0.0	1239/1297
Eukaryota	Fungi	Nectria haematococca mpVI 77-13-4	EEU44424.1	0.0	1212/1297
Eukaryota	Metazoa	Macaca mulatta	NP_001028059.1	0.0	1244/1297
Eukaryota	Fungi	Aspergillus flavus NRRL3357	XP_002373865.1	0.0	1272/1297
Eukaryota	Metazoa	Chlorocebus aethiops	AAX18881.1	0.0	1241/1297
Eukaryota	Metazoa	Cricetulus sp.	AAA37004.1	0.0	1239/1297
Eukaryota	Metazoa	Canis lupus familiaris	AAS91647.1	0.0	1243/1297
Eukaryota	Metazoa	Macaca mulatta	AAS91648.1	0.0	1244/1297
Eukaryota	Metazoa	Homo sapiens	BAF82848.1	0.0	1241/1297
Eukaryota	Metazoa	Homo sapiens	NP_000918.2	0.0	1241/1297
Eukaryota	Metazoa	Pan troglodytes	XP_001163271.1	0.0	1236/1297
Eukaryota	Metazoa	Canis lupus familiaris	ACM77791.1	0.0	1243/1297
Eukaryota	Metazoa	Canis lupus familiaris	NP_001003215.1	0.0	1243/1297
Eukaryota	Metazoa	Canis lupus familiaris	AAY67840.1	0.0	1243/1297
Eukaryota	Metazoa	Ovis aries	CAM33439.1	0.0	1242/1297
Eukaryota	Metazoa	Homo sapiens	AAA59576.1	0.0	1241/1297
Eukaryota	Metazoa	Homo sapiens	AAI30425.1	0.0	1241/1297
Eukaryota	Metazoa	Rattus norvegicus	NP_596892.1	0.0	1234/1297
Eukaryota	Metazoa	Pan troglodytes	XP_519183.2	0.0	1241/1297
Eukaryota	Metazoa	Oryctolagus cuniculus	NP_001075628.1	0.0	1242/1297
Eukaryota	Metazoa	Canis lupus familiaris	AAN05645.1	0.0	1243/1297
Eukaryota	Metazoa	Rattus norvegicus	AAS91649.1	0.0	1234/1297
Eukaryota	Metazoa	Danio rerio	XP_001922717.1	0.0	1297/1297
Eukaryota	Metazoa	Mus musculus	NP_035205.1	0.0	1237/1297
Eukaryota	Metazoa	Gallus gallus	XP_418636.2	0.0	1250/1297
Eukaryota	Metazoa	Homo sapiens	AAB69423.1	0.0	1240/1297
Eukaryota	Metazoa	Homo sapiens	AAA59575.1	0.0	1241/1297
Eukaryota	Metazoa	Bos taurus	XP_001787923.1	0.0	1226/1297
Eukaryota	Metazoa	Pan troglodytes	XP_001163208.1	0.0	1241/1297
Eukaryota	Fungi	Ajellomyces dermatitidis ER-3	EEQ83552.1	0.0	1244/1297
Eukaryota	Metazoa	Pan troglodytes	XP_001163142.1	0.0	1243/1297
Eukaryota	Metazoa	Xenopus laevis	NP_001081394.1	0.0	1235/1297
Eukaryota	Metazoa	Mytilus californianus	ABS83556.1	0.0	1264/1297
Eukaryota	Metazoa	Bos taurus	XP_590317.4	0.0	1240/1297
Eukaryota	Metazoa	Mus musculus	NP_035206.2	0.0	1238/1297
Eukaryota	Metazoa	Mus musculus	AAA39514.1	0.0	1238/1297
Eukaryota	Metazoa	Monodelphis domestica	XP_001377623.1	0.0	1243/1297
Eukaryota	Metazoa	Pan troglodytes	XP_001163342.1	0.0	1248/1297
Eukaryota	Fungi	Ajellomyces dermatitidis SLH14081	XP_002623024.1	0.0	1244/1297
Eukaryota	Metazoa	Mus musculus	AAA39517.1	0.0	1238/1297
Eukaryota	Fungi	Aspergillus nidulans FGSC A4	XP_661212.1	0.0	1261/1297
Eukaryota	Metazoa	Ovis aries	NP_001009790.1	0.0	1279/1297
Eukaryota	Metazoa	Rattus norvegicus	EDL84316.1	0.0	1237/1297
Eukaryota	Metazoa	Equus caballus	XP_001492073.2	0.0	1238/1297
Eukaryota	Metazoa	Rattus norvegicus	NP_036755.2	0.0	1237/1297
Eukaryota	Fungi	Nectria haematococca mpVI 77-13-4	EEU35319.1	0.0	1183/1297
Eukaryota	Metazoa	Pan troglodytes	XP_001163300.1	0.0	1244/1297
Eukaryota	Metazoa	Gallus gallus	NP_990225.1	0.0	1233/1297
Eukaryota	Metazoa	Equus caballus	XP_001497272.2	0.0	1238/1297
Eukaryota	Metazoa	Canis lupus familiaris	XP_539461.2	0.0	1236/1297

Eukaryota	Metazoa	Trichoplax adhaerens	XP_002110955.1	0.0	1252/1297
Eukaryota	Fungi	Microsporum canis CBS 113480	EEQ28955.1	0.0	1251/1297
Eukaryota	Metazoa	Cricetulus griseus	P21449.2	0.0	1237/1297
Eukaryota	Metazoa	Pan troglodytes	XP_001163178.1	0.0	1189/1297
Eukaryota	Fungi	Penicillium chrysogenum Wisconsin 54-1255	XP_002568389.1	0.0	1232/1297
Eukaryota	Metazoa	Monodelphis domestica	XP_001377612.1	0.0	1235/1297
Eukaryota	Metazoa	Cricetulus sp.	AAA37005.1	0.0	1164/1297
Eukaryota	Fungi	Aspergillus niger CBS 513.88	XP_001392653.1	0.0	1230/1297
Eukaryota	Metazoa	Rattus norvegicus	P43245.1	0.0	1239/1297
Eukaryota	Metazoa	Takifugu rubripes	AAO20901.1	0.0	1248/1297
Eukaryota	Metazoa	Ciona intestinalis	XP_002131382.1	0.0	1283/1297
Eukaryota	Metazoa	Platichthys flesus	CAC86600.1	0.0	1243/1297
Eukaryota	Metazoa	Mus musculus	NP_084237.1	0.0	1233/1297
Eukaryota	Fungi	Emericella nidulans	AAD25925.1	0.0	1261/1297
Eukaryota	Fungi	Gibberella zeae PH-1	XP_388999.1	0.0	1240/1297
Eukaryota	Fungi	Verticillium albo-atrum VaMs.102	EEY23473.1	0.0	1272/1297
Eukaryota	Fungi	Phaeosphaeria nodorum SN15	XP_001805258.1	0.0	1257/1297
Eukaryota	Fungi	Podospora anserina DSM 980	XP_001906793.1	0.0	1244/1297
Eukaryota	Metazoa	Homo sapiens	AAP55848.1	0.0	1226/1297
Eukaryota	Metazoa	Homo sapiens	NP_001157413.1	0.0	1226/1297
Eukaryota	Fungi	Microsporum canis CBS 113480	EEQ33365.1	0.0	1204/1297
Eukaryota	Metazoa	Mus musculus	NP_032856.2	0.0	1234/1297
Eukaryota	Fungi	Aspergillus fumigatus Af293	XP_747768.1	0.0	1262/1297
Eukaryota	Metazoa	Cricetulus griseus	P23174.1	0.0	1236/1297
Eukaryota	Metazoa	Ciona intestinalis	XP_002126447.1	0.0	1276/1297
Eukaryota	Metazoa	Xenopus (Silurana) tropicalis	NP_989254.1	0.0	1247/1297
Eukaryota	Metazoa	Rattus norvegicus	EDL84315.1	0.0	1236/1297
Eukaryota	Metazoa	Mus musculus	P21440.1	0.0	1234/1297
Eukaryota	Fungi	Neosartorya fischeri NRRL 181	XP_001257500.1	0.0	1247/1297
Eukaryota	Metazoa	Rattus norvegicus	NP_036822.1	0.0	1236/1297
Eukaryota	Fungi	Pyrenophora tritici-repentis Pt-1C-BFP	XP_001942410.1	0.0	1249/1297
Eukaryota	Metazoa	Macaca mulatta	XP_001102010.1	0.0	1129/1297
Eukaryota	Metazoa	Pan troglodytes	XP_001152831.1	0.0	1171/1297
Eukaryota	Fungi	Nectria haematococca mpVI 77-13-4	EEU46758.1	0.0	1228/1297
Eukaryota	Fungi	Neosartorya fischeri NRRL 181	XP_001258969.1	0.0	1219/1297
Eukaryota	Metazoa	Tribolium castaneum	XP_001810982.1	0.0	1226/1297
Eukaryota	Fungi	Coccidioides posadasii C735 delta	EER24709.1	0.0	1270/1297
Eukaryota	Viridiplantae	Catharanthus roseus	ABG56413.1	0.0	1246/1297
Eukaryota	Metazoa	Homo sapiens	EAW76949.1	0.0	1251/1297
Eukaryota	Fungi	Nectria haematococca mpVI 77-13-4	EEU41707.1	0.0	1249/1297
Eukaryota	Metazoa	Homo sapiens	NP_000434.1	0.0	1251/1297
Eukaryota	Metazoa	Pan troglodytes	XP_001160982.1	0.0	1252/1297
Eukaryota	Metazoa	Danio rerio	XP_001337724.2	0.0	1285/1297
Eukaryota	Fungi	Aspergillus nidulans FGSC A4	XP_659953.1	0.0	1275/1297
Eukaryota	Fungi	Aspergillus oryzae RIB40	XP_001819360.1	0.0	1208/1297
Eukaryota	Viridiplantae	Oryza sativa Japonica Group	CAD59586.1	0.0	1235/1297
Eukaryota	Fungi	Aspergillus fumigatus A1163	EDP53377.1	0.0	1207/1297
Eukaryota	Viridiplantae	Vitis vinifera	CAO66875.1	0.0	1220/1297
Eukaryota	Metazoa	Homo sapiens	NP_061337.1	0.0	1258/1297
Eukaryota	Metazoa	Rattus norvegicus	XP_234725.4	0.0	1232/1297
Eukaryota	Metazoa	Homo sapiens	ACF94688.1	0.0	1177/1297
Eukaryota	Fungi	Aspergillus fumigatus Af293	XP_748598.1	0.0	1207/1297
Eukaryota	Metazoa	Homo sapiens	AAH42531.1	0.0	1258/1297
Eukaryota	Viridiplantae	Oryza sativa Indica Group	EAY75483.1	0.0	1235/1297
Eukaryota	Viridiplantae	Vitis vinifera	XP_002271185.1	0.0	1247/1297
Eukaryota	Metazoa	Ornithorhynchus anatinus	XP_001513897.1	0.0	1272/1297
Eukaryota	Fungi	Gibberella zeae PH-1	XP_383499.1	0.0	1261/1297
Eukaryota	Metazoa	Homo sapiens	EAX11287.1	0.0	1309/1297

Eukaryota	Fungi	Aspergillus flavus NRRL3357	XP_002379996.1	0.0	1239/1297
Eukaryota	Metazoa	Pan troglodytes	XP_526100.2	0.0	1300/1297
Eukaryota	Metazoa	Equus caballus	XP_001497606.2	0.0	1291/1297
Eukaryota	Fungi	Aspergillus oryzae RIB40	XP_001818587.1	0.0	1219/1297
Eukaryota	Viridiplantae	Ricinus communis	XP_002519757.1	0.0	1245/1297
Eukaryota	Fungi	Nectria haematococca mpVI 77-13-4	EEU47023.1	0.0	1232/1297
Eukaryota	Metazoa	Homo sapiens	NP_003733.2	0.0	1300/1297
Eukaryota	Fungi	Emericella nidulans	AAD43625.1	0.0	1275/1297
Eukaryota	Metazoa	Homo sapiens	AAD28285.1	0.0	1300/1297
Eukaryota	Metazoa	Homo sapiens	EAX11286.1	0.0	1285/1297
Eukaryota	Viridiplantae	Populus trichocarpa	XP_002320942.1	0.0	1270/1297
Eukaryota	Fungi	Aspergillus oryzae RIB40	XP_001820535.1	0.0	1109/1297
Eukaryota	Viridiplantae	Triticum aestivum;	BAB85651.1	0.0	1238/1297
Eukaryota	Metazoa	Mus musculus	AAA03243.1	0.0	1097/1297
Eukaryota	Metazoa	Homo sapiens	O95342.1	0.0	1300/1297
Eukaryota	Viridiplantae	Ricinus communis	XP_002519759.1	0.0	1251/1297
Eukaryota	Metazoa	Canis lupus familiaris	NP_001137404.1	0.0	1285/1297
Eukaryota	Fungi	Nectria haematococca mpVI 77-13-4	EEU36332.1	0.0	1261/1297
Eukaryota	Viridiplantae	Populus trichocarpa	XP_002320939.1	0.0	1270/1297
Eukaryota	Metazoa	Acyrthosiphon pisum	XP_001947434.1	0.0	1268/1297
Eukaryota	Fungi	Neosartorya fischeri NRRL 181	XP_001266548.1	0.0	1259/1297
Eukaryota	Viridiplantae	Populus trichocarpa	XP_002331877.1	0.0	1247/1297
Eukaryota	Viridiplantae	Vitis vinifera	CAO70937.1	0.0	1230/1297
Eukaryota	Viridiplantae	Vitis vinifera	XP_002271305.1	0.0	1240/1297
Eukaryota	Metazoa	Homo sapiens	NP_061338.1	0.0	1204/1297
Eukaryota	Metazoa	Aedes aegypti	XP_001654492.1	0.0	1248/1297
Eukaryota	Viridiplantae	Oryza sativa Indica Group	EEC73043.1	0.0	1243/1297
Eukaryota	Metazoa	Taeniopygia guttata	XP_002194908.1	0.0	1285/1297
Eukaryota	Viridiplantae	Arabidopsis thaliana	NP_199466.1	0.0	1233/1297
Eukaryota	Viridiplantae	Oryza sativa Japonica Group	EEE55238.1	0.0	1228/1297
Eukaryota	Viridiplantae	Vitis vinifera	CAO66886.1	0.0	1216/1297
Eukaryota	Viridiplantae	Oryza sativa Indica Group	EEC71330.1	0.0	1228/1297
Eukaryota	Viridiplantae	Oryza sativa Japonica Group	CAD59587.1	0.0	1231/1297
Eukaryota	Fungi	Aspergillus flavus NRRL3357	XP_002381771.1	0.0	1245/1297
Eukaryota	Metazoa	Mus musculus	Q9QY30.1	0.0	1285/1297
Eukaryota	Fungi	Rhizopus stolonifer	CAI47726.2	0.0	1280/1297
Eukaryota	Fungi	Pyrenophora tritici-repentis Pt-1C-BFP	XP_001940759.1	0.0	1263/1297
Eukaryota	Metazoa	Mus musculus	NP_066302.2	0.0	1285/1297
Eukaryota	Fungi	Aspergillus oryzae RIB40	XP_001825076.1	0.0	1239/1297
Eukaryota	Fungi	Neosartorya fischeri NRRL 181	XP_001262673.1	0.0	1290/1297
Eukaryota	Fungi	Ajellomyces capsulatus G186AR	EEH07483.1	0.0	1259/1297
Eukaryota	Viridiplantae	Populus trichocarpa	XP_002301547.1	0.0	1218/1297
Eukaryota	Viridiplantae	Oryza sativa Japonica Group	BAD15946.1	0.0	1249/1297
Eukaryota	Viridiplantae	Vitis vinifera	CAN77320.1	0.0	1218/1297
Eukaryota	Viridiplantae	Vitis vinifera	XP_002273987.1	0.0	1246/1297
Eukaryota	Metazoa	Cavia porcellus	ACU12846.1	0.0	1284/1297
Eukaryota	Metazoa	Pediculus humanus corporis	XP_002432260.1	0.0	1234/1297
Eukaryota	Viridiplantae	Oryza sativa Japonica Group	CAD59585.1	0.0	1240/1297
Eukaryota	Metazoa	Danio rerio	XP_699562.3	0.0	1285/1297
Eukaryota	Viridiplantae	Oryza sativa Japonica Group	BAA96612.1	0.0	1231/1297

AFUA_4G14310

Eukaryota	Fungi	Aspergillus fumigatus Af293	XP_751401.1	1 E-162	274/274
Eukaryota	Fungi	Microsporum canis CBS 113480	EEQ31848.1	1 E-101	281/274
Eukaryota	Fungi	Penicillium chrysogenum Wisconsin 54-1255	XP_002566455.1	6 E-94	280/274
Eukaryota	Fungi	Nectria haematococca mpVI 77-13-4	EEU34230.1	6 E-93	276/274
Eukaryota	Fungi	Verticillium albo-atrum VaMs.102	EEY19569.1	6 E-93	281/274

Eukaryota	Fungi	Aspergillus terreus NIH2624	XP_001213918.1	9 E-81	254/274
Eukaryota	Fungi	Phaeosphaeria nodorum SN15	XP_001798147.1	1 E-49	283/274
Eukaryota	Fungi	Microsporum canis CBS 113480	EEQ31341.1	3 E-36	273/274
Eukaryota	Fungi	Microsporum canis CBS 113480	EEQ35034.1	3 E-35	260/274
Eukaryota	Fungi	Coccidioides posadasii C735 delta	EER26442.1	9 E-35	262/274
Eukaryota	Fungi	Talaromyces stipitatus ATCC 10500	XP_002484450.1	1 E-34	289/274
Eukaryota	Fungi	Coccidioides immitis RS;	XP_001244223.1	2 E-34	262/274
Eukaryota	Fungi	Penicillium chrysogenum Wisconsin 54-1255	XP_002564312.1	1 E-33	291/274
Eukaryota	Fungi	Ajellomyces capsulatus NAm1	XP_001535918.1	4 E-33	264/274
Eukaryota	Fungi	Coccidioides immitis RS;	XP_001244002.1	4 E-33	258/274
Eukaryota	Fungi	Ajellomyces dermatitidis ER-3	EEQ90661.1	6 E-33	264/274
Eukaryota	Fungi	Ajellomyces capsulatus G186AR	EEH06650.1	6 E-33	290/274
Eukaryota	Fungi	Ajellomyces capsulatus G186AR	EEH10318.1	7 E-33	264/274
Eukaryota	Fungi	Ajellomyces dermatitidis SLH14081	XP_002622961.1	2 E-32	264/274
Eukaryota	Fungi	Aspergillus clavatus NRRL 1	XP_001272007.1	2 E-32	258/274
Eukaryota	Fungi	Ajellomyces capsulatus H143	EER39163.1	2 E-32	264/274
Eukaryota	Fungi	Coccidioides posadasii C735 delta	EER26615.1	2 E-31	257/274
Eukaryota	Fungi	Coccidioides posadasii C735 delta	EER26471.1	8 E-31	290/274
Eukaryota	Fungi	Ajellomyces capsulatus NAm1	XP_001543247.1	4 E-30	293/274
Eukaryota	Fungi	Neosartorya fischeri NRRL 181	XP_001262202.1	1 E-29	270/274
Eukaryota	Fungi	Coccidioides immitis RS;	XP_001239606.1	2 E-29	229/274
Eukaryota	Fungi	Gibberella zeae PH-1	XP_390838.1	4 E-29	285/274
Eukaryota	Fungi	Chaetomium globosum CBS 148.51	XP_001225691.1	2 E-28	310/274
Eukaryota	Fungi	Coccidioides posadasii C735 delta	EER24811.1	4 E-28	275/274
Eukaryota	Fungi	Ajellomyces capsulatus NAm1	XP_001540377.1	3 E-26	243/274
Eukaryota	Fungi	Pyrenophora tritici-repentis Pt-1C-BFP	XP_001937101.1	2 E-25	239/274
Eukaryota	Fungi	Podospora anserina DSM 980	XP_001909358.1	1 E-23	292/274
Eukaryota	Fungi	Aspergillus fumigatus A1163	EDP53281.1	1 E-22	274/274
Eukaryota	Fungi	Pyrenophora tritici-repentis Pt-1C-BFP	XP_001932536.1	3 E-22	235/274
Eukaryota	Fungi	Penicillium chrysogenum Wisconsin 54-1255	XP_002568335.1	7 E-22	230/274
Eukaryota	Fungi	Ajellomyces dermatitidis SLH14081	XP_002628189.1	3 E-20	234/274
Eukaryota	Fungi	Ajellomyces capsulatus NAm1	XP_001544572.1	8 E-20	234/274
Eukaryota	Fungi	Ajellomyces dermatitidis ER-3	EEQ88985.1	2 E-19	230/274
Eukaryota	Fungi	Penicillium chrysogenum Wisconsin 54-1255	XP_002562493.1	2 E-17	228/274
Eukaryota	Fungi	Postia placenta Mad-698-R	XP_002472004.1	9 E-17	221/274
Eukaryota	Fungi	Ajellomyces capsulatus H143	EER42869.1	8 E-14	224/274
Eukaryota	Fungi	Ajellomyces capsulatus H143	EER36361.1	1 E-13	235/274
Eukaryota	Fungi	Nectria haematococca mpVI 77-13-4	EEU47096.1	3 E-12	231/274

AFUA_4G14410

Eukaryota	Fungi	Aspergillus fumigatus Af293	XP_751391.1	1 E-146	256/256
Eukaryota	Fungi	Talaromyces stipitatus ATCC 10500	XP_002482093.1	4 E-52	265/256
Eukaryota	Fungi	Talaromyces stipitatus ATCC 10500	XP_002488384.1	4 E-50	248/256
Eukaryota	Fungi	Penicillium marneffei ATCC 18224	XP_002147883.1	2 E-49	267/256
Eukaryota	Fungi	Talaromyces stipitatus ATCC 10500	XP_002488468.1	1 E-39	265/256
Eukaryota	Fungi	Neosartorya fischeri NRRL 181	XP_001259864.1	3 E-34	262/256
Eukaryota	Fungi	Coccidioides posadasii C735 delta	EER27216.1	2 E-33	260/256
Eukaryota	Fungi	Aspergillus fumigatus Af293	XP_753814.1	3 E-33	262/256
Eukaryota	Fungi	Coccidioides immitis RS;	XP_001242066.1	4 E-33	260/256
Eukaryota	Fungi	Coccidioides immitis RS;	XP_001241584.1	6 E-32	261/256
Eukaryota	Fungi	Aspergillus nidulans FGSC A4	XP_680983.1	1 E-31	259/256
Eukaryota	Fungi	Aspergillus clavatus NRRL 1	XP_001274227.1	2 E-30	245/256
Eukaryota	Fungi	Aspergillus terreus NIH2624	XP_001216925.1	8 E-30	263/256
Eukaryota	Fungi	Paracoccidioides brasiliensis Pb18;	EEH46038.1	4 E-27	251/256
Eukaryota	Fungi	Paracoccidioides brasiliensis Pb03;	EEH16672.1	5 E-27	251/256
Eukaryota	Fungi	Paracoccidioides brasiliensis Pb01;	EEH36173.1	2 E-26	252/256
Eukaryota	Fungi	Microsporum canis CBS 113480	EEQ34032.1	3 E-25	282/256

Eukaryota	Fungi	Aspergillus oryzae RIB40	XP_001823540.1	2 E-22	255/256
Eukaryota	Fungi	Aspergillus flavus NRRL3357	XP_002378946.1	4 E-22	254/256
Eukaryota	Fungi	Ajellomyces dermatitidis ER-3	EEQ87082.1	4 E-21	243/256
Eukaryota	Fungi	Aspergillus niger CBS 513.88	XP_001390324.1	1 E-20	246/256
Eukaryota	Fungi	Sclerotinia sclerotiorum 1980 UF-70	XP_001591380.1	9 E-19	245/256
Eukaryota	Fungi	Pyrenophora tritici-repentis Pt-1C-BFP	XP_001941102.1	4 E-11	207/256
Eukaryota	Fungi	Podospora anserina DSM 980	XP_001906908.1	5 E-11	248/256

AFUA_4G14560

Eukaryota	Fungi	Aspergillus fumigatus Af293	XP_751377.1	0.0	1782/1782
Eukaryota	Fungi	Neosartorya fischeri NRRL 181	XP_001266594.1	0.0	1785/1782
Eukaryota	Fungi	Aspergillus terreus NIH2624	XP_001217072.1	0.0	1771/1782
Eukaryota	Fungi	Aspergillus nidulans FGSC A4	XP_657754.1	0.0	1803/1782
Eukaryota	Fungi	Penicillium marneffei ATCC 18224	XP_002144865.1	0.0	1793/1782
Eukaryota	Fungi	Talaromyces stipitatus ATCC 10500	XP_002482902.1	0.0	1768/1782
Eukaryota	Fungi	Talaromyces stipitatus ATCC 10500	XP_002482968.1	0.0	1803/1782
Eukaryota	Fungi	Penicillium marneffei ATCC 18224	XP_002149615.1	0.0	1754/1782
Eukaryota	Fungi	Neosartorya fischeri NRRL 181	XP_001266579.1	0.0	1808/1782
Eukaryota	Fungi	Pyrenophora tritici-repentis Pt-1C-BFP	XP_001933041.1	0.0	1787/1782
Eukaryota	Fungi	Cochliobolus heterostrophus	AAR90273.1	0.0	1797/1782
Eukaryota	Fungi	Neosartorya fischeri NRRL 181	XP_001267621.1	0.0	1770/1782
Eukaryota	Fungi	Aspergillus fumigatus Af293	XP_746435.1	0.0	1768/1782
Eukaryota	Fungi	Aspergillus fumigatus A1163	EDP47078.1	0.0	1769/1782
Eukaryota	Fungi	Neosartorya fischeri NRRL 181	XP_001262597.1	0.0	1787/1782
Eukaryota	Fungi	Microsporum canis CBS 113480	EEQ30779.1	0.0	1789/1782
Eukaryota	Fungi	Aspergillus fumigatus Af293	XP_746913.1	0.0	1787/1782
Eukaryota	Fungi	Aspergillus fumigatus A1163	EDP47964.1	0.0	1787/1782
Eukaryota	Fungi	Aspergillus nidulans FGSC A4	XP_663604.1	0.0	1782/1782
Eukaryota	Fungi	Aspergillus terreus NIH2624	XP_001211612.1	0.0	1758/1782
Eukaryota	Fungi	Aspergillus niger CBS 513.88	XP_001394705.1	0.0	1787/1782
Eukaryota	Fungi	Aspergillus terreus	BAB88752.1	0.0	1758/1782
Eukaryota	Fungi	Botryotinia fuckeliana	AAR90250.1	0.0	1750/1782
Eukaryota	Fungi	Aspergillus clavatus NRRL 1	XP_001275038.1	0.0	1749/1782
Eukaryota	Fungi	Microsporum canis CBS 113480	EEQ31623.1	0.0	1786/1782
Eukaryota	Fungi	Aspergillus flavus NRRL3357	XP_002376725.1	0.0	1743/1782
Eukaryota	Fungi	Aspergillus oryzae RIB40	XP_001820992.1	0.0	1723/1782
Eukaryota	Fungi	Botryotinia fuckeliana B05.10	XP_001553397.1	0.0	1646/1782
Eukaryota	Fungi	Aspergillus niger CBS 513.88	XP_001402309.1	0.0	1644/1782
Eukaryota	Fungi	Aspergillus oryzae RIB40	XP_001823362.1	0.0	1859/1782
Eukaryota	Fungi	Bipolaris oryzae	BAD22832.1	0.0	1731/1782
Eukaryota	Fungi	Ophiostoma piceae	ABD47522.2	0.0	1706/1782
Eukaryota	Fungi	Sclerotinia sclerotiorum 1980 UF-70	XP_001586760.1	0.0	1713/1782
Eukaryota	Fungi	Sclerotinia sclerotiorum 1980 UF-70	XP_001585805.1	0.0	1709/1782
Eukaryota	Fungi	Phaeosphaeria nodorum SN15	XP_001805964.1	0.0	1494/1782
Eukaryota	Fungi	Cochliobolus heterostrophus	AAR90272.1	0.0	1696/1782
Eukaryota	Fungi	Botryotinia fuckeliana B05.10	XP_001554288.1	0.0	1712/1782
Eukaryota	Fungi	Elsinoe fawcettii	ABU63483.1	0.0	1722/1782
Eukaryota	Fungi	Magnaporthe grisea 70-15	XP_367294.2	0.0	1709/1782
Eukaryota	Fungi	Glarea lozoyensis	AAN59953.1	0.0	1696/1782
Eukaryota	Fungi	Phaeosphaeria nodorum SN15	XP_001802212.1	0.0	1714/1782
Eukaryota	Fungi	Aspergillus nidulans FGSC A4	XP_664675.1	0.0	1840/1782
Eukaryota	Fungi	Ceratocystis resinifera;	AAO60166.1	0.0	1698/1782
Eukaryota	Fungi	Nodulisporium sp. ATCC74245	AAD38786.1	0.0	1695/1782
Eukaryota	Fungi	Monascus purpureus	CAC94008.1	0.0	1592/1782
Eukaryota	Fungi	Xylaria sp. BCC 1067	AAM93545.1	0.0	1699/1782
Eukaryota	Fungi	Botryotinia fuckeliana	AAR90249.1	0.0	1709/1782
Eukaryota	Fungi	Podospora anserina DSM 980	XP_001910795.1	0.0	1710/1782

Eukaryota	Fungi	Botryotinia fuckeliana B05.10	XP_001547095.1	0.0	1709/1782
Eukaryota	Fungi	Penicillium marneffei ATCC 18224	XP_002149119.1	0.0	1721/1782
Eukaryota	Fungi	Colletotrichum lagenarium;	BAA18956.1	0.0	1719/1782
Eukaryota	Fungi	Aspergillus terreus	BAB88688.1	0.0	1719/1782
Eukaryota	Fungi	Glomerella graminicola;	ACN32207.1	0.0	1673/1782
Eukaryota	Fungi	Chaetomium globosum CBS 148.51	XP_001219763.1	0.0	1702/1782
Eukaryota	Fungi	Sordaria macrospora	CAM35471.1	0.0	1721/1782
Eukaryota	Fungi	Aspergillus terreus NIH2624	XP_001210231.1	0.0	1569/1782
Eukaryota	Fungi	Aspergillus flavus NRRL3357	XP_002382817.1	0.0	1714/1782
Eukaryota	Fungi	Pyrenophora tritici-repentis Pt-1C-BFP	XP_001933656.1	0.0	1552/1782
Eukaryota	Fungi	Penicillium chrysogenum Wisconsin 54-1255	XP_002568608.1	0.0	1706/1782
Eukaryota	Fungi	Xanthoria elegans	ABG91136.3	0.0	1501/1782
Eukaryota	Fungi	Exophiala dermatitidis	AAD31436.3	0.0	1732/1782
Eukaryota	Fungi	Aspergillus oryzae RIB40	XP_001822700.1	0.0	1714/1782
Eukaryota	Fungi	Aspergillus niger CBS 513.88	XP_001393884.1	0.0	1711/1782
Eukaryota	Fungi	Neurospora crassa OR74A	XP_960586.2	0.0	1600/1782
Eukaryota	Fungi	Neosartorya fischeri NRRL 181	XP_001261235.1	0.0	1697/1782
Eukaryota	Fungi	Aspergillus fumigatus	ACJ13038.1	0.0	1699/1782
Eukaryota	Fungi	Aspergillus fumigatus	ACJ13035.1	0.0	1699/1782
Eukaryota	Fungi	Aspergillus fumigatus	ACJ13039.1	0.0	1699/1782
Eukaryota	Fungi	Aspergillus fumigatus	ACJ13034.1	0.0	1699/1782
Eukaryota	Fungi	Aspergillus fumigatus Af293	XP_756095.1	0.0	1699/1782
Eukaryota	Fungi	Aspergillus fumigatus A1163	EDP55264.1	0.0	1699/1782
Eukaryota	Fungi	Aspergillus fumigatus	AAC39471.1	0.0	1699/1782
Eukaryota	Fungi	Aspergillus fumigatus	CAA76740.1	0.0	1692/1782
Eukaryota	Fungi	Emericella nidulans	prf1905375A	0.0	1719/1782
Eukaryota	Fungi	Emericella nidulans	Q03149.2	0.0	1719/1782
Eukaryota	Fungi	Aspergillus clavatus NRRL 1	XP_001276035.1	0.0	1697/1782
Eukaryota	Fungi	Penicillium marneffei ATCC 18224	XP_002147717.1	0.0	1696/1782
Eukaryota	Fungi	Talaromyces stipitatus ATCC 10500	XP_002481882.1	0.0	1686/1782
Eukaryota	Fungi	Aspergillus fumigatus	ACJ13036.1	0.0	1699/1782
Eukaryota	Fungi	Cochliobolus heterostrophus	AAR90274.1	0.0	1445/1782
Eukaryota	Fungi	Aspergillus fumigatus	ACJ13037.1	0.0	1658/1782
Eukaryota	Fungi	Gibberella zeae	AAU10633.1	0.0	1709/1782
Eukaryota	Fungi	Exophiala lecanii-corni	AAN74983.1	0.0	1707/1782
Eukaryota	Fungi	Microsporum canis CBS 113480	EEQ32235.1	0.0	1700/1782
Eukaryota	Fungi	Aspergillus niger CBS 513.88	XP_001390425.1	0.0	1707/1782
Eukaryota	Fungi	Talaromyces stipitatus ATCC 10500	XP_002483594.1	0.0	1702/1782
Eukaryota	Fungi	Exophiala lecanii-corni	AAN75188.1	0.0	1705/1782
Eukaryota	Fungi	Talaromyces stipitatus ATCC 10500	XP_002478062.1	0.0	1655/1782
Eukaryota	Fungi	Nectria haematococca	AAS48892.1	0.0	1700/1782
Eukaryota	Fungi	Penicillium marneffei ATCC 18224	XP_002152334.1	0.0	1725/1782
Eukaryota	Fungi	Penicillium marneffei ATCC 18224	XP_002145792.1	0.0	1711/1782
Eukaryota	Fungi	Aspergillus flavus NRRL3357	XP_002384329.1	0.0	1685/1782
Eukaryota	Fungi	Nectria haematococca mpVI 77-13-4	EEU40203.1	0.0	1700/1782
Eukaryota	Fungi	Aspergillus oryzae RIB40	XP_001827098.1	0.0	1702/1782
Eukaryota	Fungi	Podospora anserina DSM 980	XP_001911464.1	0.0	1668/1782
Eukaryota	Fungi	Aspergillus flavus NRRL3357	XP_002373130.1	0.0	1704/1782
Eukaryota	Fungi	Aspergillus oryzae RIB40	XP_001817959.1	0.0	1708/1782
Eukaryota	Fungi	Gibberella moniliformis	AAR92210.1	0.0	1703/1782
Eukaryota	Fungi	Nectria haematococca mpVI 77-13-4	EEU34216.1	0.0	1704/1782
Eukaryota	Fungi	Gibberella fujikuroi	CAB92399.1	0.0	1683/1782
Eukaryota	Fungi	Talaromyces stipitatus ATCC 10500	XP_002483004.1	0.0	1692/1782
Eukaryota	Fungi	Aspergillus terreus	BAB88689.1	0.0	1733/1782
Eukaryota	Fungi	Gibberella moniliformis	AAR92211.1	0.0	1536/1782
Eukaryota	Fungi	Microsporum canis CBS 113480	EEQ35716.1	0.0	1674/1782
Eukaryota	Fungi	Aspergillus flavus	AAS89999.1	0.0	1755/1782
Eukaryota	Fungi	Aspergillus sp. L	AAR32704.2	0.0	1755/1782

Eukaryota	Fungi	Aspergillus parasiticus	Q12053.1	0.0	1755/1782
Eukaryota	Fungi	Aspergillus flavus	AAS90022.1	0.0	1755/1782
Eukaryota	Fungi	Aspergillus flavus NRRL3357	XP_002379951.1	0.0	1755/1782
Eukaryota	Fungi	Aspergillus oryzae RIB40	XP_001821511.1	0.0	1755/1782
Eukaryota	Fungi	Aspergillus oryzae	BAE71314.1	0.0	1755/1782
Eukaryota	Fungi	Aspergillus sojae	AAU08792.1	0.0	1755/1782
Eukaryota	Fungi	Aspergillus nomius	AAS90047.1	0.0	1747/1782
Eukaryota	Fungi	Podospora anserina DSM 980	XP_001911528.1	0.0	1743/1782
Eukaryota	Fungi	Aspergillus nidulans FGSC A4	XP_681094.1	0.0	1757/1782
Eukaryota	Fungi	Emericella nidulans	Q12397.2	0.0	1757/1782
Eukaryota	Fungi	Coccidioides immitis RS;	XP_001241406.1	0.0	1807/1782
Eukaryota	Fungi	Coccidioides posadasii C735 delta	EER28351.1	0.0	1791/1782
Eukaryota	Fungi	Mycosphaerella pini	AAZ95017.1	0.0	1807/1782
Eukaryota	Fungi	Aspergillus ochraceoroseus	ACH72912.1	0.0	1751/1782
Eukaryota	Fungi	Cercospora nicotianae	AAT69682.1	0.0	1738/1782
Eukaryota	Fungi	Emericella nidulans	AAA81586.1	0.0	1746/1782
Eukaryota	Fungi	Leptosphaeria maculans	AAS92537.1	0.0	1719/1782
Eukaryota	Fungi	Phaeosphaeria nodorum SN15	XP_001798923.1	0.0	1511/1782
Eukaryota	Fungi	Penicillium marneffei ATCC 18224	XP_002146110.1	0.0	1672/1782
Eukaryota	Fungi	Talaromyces stipitatus ATCC 10500	XP_002488697.1	0.0	1744/1782
Eukaryota	Fungi	Botryotinia fuckeliana	AAR90251.1	0.0	1667/1782
Eukaryota	Fungi	Penicillium marneffei ATCC 18224	XP_002151003.1	0.0	1762/1782
Eukaryota	Fungi	Chaetomium chiversii	ACM42403.1	0.0	1729/1782
Eukaryota	Fungi	Gibberella zeae	ABB90282.1	0.0	1677/1782
Eukaryota	Fungi	Hypomyces subiculosus	ACD39753.1	0.0	1691/1782
Eukaryota	Fungi	Hypomyces subiculosus	ACD39762.1	0.0	1691/1782
Eukaryota	Fungi	Penicillium chrysogenum Wisconsin 54-1255	XP_002568275.1	0.0	1720/1782
Eukaryota	Fungi	Aspergillus nidulans FGSC A4	XP_681178.1	0.0	1691/1782
Eukaryota	Fungi	Pochonia chlamydosporia	ACD39770.1	0.0	1662/1782
Eukaryota	Fungi	Chaetomium globosum CBS 148.51	XP_001225797.1	0.0	1626/1782
Eukaryota	Fungi	Magnaporthe grisea 70-15	XP_369003.2	0.0	1665/1782
Eukaryota	Fungi	Talaromyces stipitatus ATCC 10500	XP_002486858.1	1 E-173	1641/1782
Eukaryota	Fungi	Gibberella zeae PH-1	XP_384140.1	1 E-170	1620/1782
Eukaryota	Fungi	Aspergillus flavus NRRL3357	XP_002377153.1	1 E-157	1655/1782
Eukaryota	Fungi	Aspergillus terreus NIH2624	XP_001210065.1	1 E-156	1535/1782
Eukaryota	Fungi	Aspergillus fumigatus A1163	EDP49937.1	1 E-156	1613/1782
Eukaryota	Fungi	Aspergillus oryzae RIB40	XP_001821376.1	1 E-143	1652/1782

AFUA_5G00240

Eukaryota	Fungi	Aspergillus fumigatus Af293	XP_748316.1	4 E-77	139/139
Eukaryota	Fungi	Aspergillus terreus NIH2624	XP_001209778.1	8 E-17	113/139

AFUA_5G00250

Eukaryota	Fungi	Aspergillus fumigatus Af293	XP_748315.1	1 E-149	258/258
Eukaryota	Fungi	Candida dubliniensis CD36	XP_002418310.1	5 E-15	222/258
Eukaryota	Fungi	Candida albicans SC5314	XP_720983.1	2 E-14	222/258
Eukaryota	Fungi	Candida albicans SC5314	XP_721104.1	2 E-14	222/258
Eukaryota	Fungi	Pichia stipitis CBS 6054	XP_001384084.2	4 E-13	223/258
Eukaryota	Fungi	Candida dubliniensis CD36	XP_002418122.1	4 E-13	210/258
Eukaryota	Fungi	Pichia stipitis CBS 6054	XP_001384582.2	3 E-12	224/258

AFUA_5G00650

Eukaryota	Fungi	Aspergillus fumigatus Af293	XP_748274.1	0.0	340/340
Eukaryota	Fungi	Penicillium chrysogenum Wisconsin 54-1255	XP_002560250.1	1 E-173	340/340
Eukaryota	Fungi	Gibberella zeae PH-1	XP_384922.1	8 E-98	308/340

Eukaryota	Fungi	Pyrenophora tritici-repentis Pt-1C-BFP	XP_001934326.1	3 E-95	337/340
Eukaryota	Fungi	Pyrenophora tritici-repentis Pt-1C-BFP	XP_001931879.1	1 E-93	341/340
Eukaryota	Fungi	Neosartorya fischeri NRRL 181	XP_001262587.1	6 E-93	333/340
Eukaryota	Fungi	Aspergillus niger CBS 513.88	XP_001391222.1	6 E-92	338/340
Eukaryota	Fungi	Aspergillus clavatus NRRL 1	XP_001273884.1	1 E-91	332/340
Eukaryota	Fungi	Phaeosphaeria nodorum SN15	XP_001790791.1	2 E-91	340/340
Eukaryota	Fungi	Penicillium chrysogenum Wisconsin 54-1255	XP_002566697.1	7 E-91	338/340
Eukaryota	Fungi	Penicillium chrysogenum Wisconsin 54-1255	XP_002557659.1	8 E-91	334/340
Eukaryota	Fungi	Aspergillus clavatus NRRL 1	XP_001267786.1	9 E-74	327/340
Eukaryota	Fungi	Microsporum canis CBS 113480	EEQ34790.1	4 E-16	280/340

AFUA_5G00660

Eukaryota	Fungi	Aspergillus fumigatus Af293	XP_748273.1	0.0	479/479

AFUA_5G01680

Eukaryota	Fungi	Aspergillus fumigatus Af293	XP_748174.1	0.0	515/515
Eukaryota	Fungi	Aspergillus fumigatus A1163	EDP51019.1	0.0	515/515
Eukaryota	Fungi	Neosartorya fischeri NRRL 181	XP_001266362.1	0.0	487/515
Eukaryota	Fungi	Aspergillus niger CBS 513.88	XP_001397413.1	0.0	486/515
Eukaryota	Fungi	Aspergillus terreus NIH2624	XP_001218400.1	0.0	478/515
Eukaryota	Fungi	Aspergillus oryzae RIB40	XP_001820425.1	0.0	471/515
Eukaryota	Fungi	Penicillium chrysogenum Wisconsin 54-1255	XP_002568441.1	0.0	457/515
Eukaryota	Fungi	Talaromyces stipitatus ATCC 10500	XP_002478354.1	0.0	504/515
Eukaryota	Fungi	Penicillium marneffei ATCC 18224	XP_002146061.1	0.0	482/515
Eukaryota	Fungi	Sclerotinia sclerotiorum 1980 UF-70	XP_001595338.1	0.0	483/515
Eukaryota	Fungi	Podospora anserina DSM 980	XP_001903567.1	1 E-177	474/515
Eukaryota	Fungi	Botryotinia fuckeliana B05.10	XP_001553454.1	1 E-174	507/515
Eukaryota	Fungi	Neurospora crassa OR74A	XP_001728106.1	1 E-172	481/515
Eukaryota	Fungi	Schizosaccharomyces pombe	NP_595068.1	1 E-124	470/515
Eukaryota	Fungi	Schizosaccharomyces pombe	NP_593504.1	1 E-120	462/515
Eukaryota	Fungi	Lachancea thermotolerans CBS 6340	XP_002552222.1	1 E-118	490/515
Eukaryota	Fungi	Saccharomyces cerevisiae EC1118	CAY82272.1	1 E-116	500/515
Eukaryota	Fungi	Kluyveromyces lactis NRRL Y-1140	XP_453048.1	1 E-115	457/515
Eukaryota	Fungi	Schizosaccharomyces pombe	NP_592813.1	1 E-115	487/515
Eukaryota	Fungi	Zygosaccharomyces bailii	CAX63267.1	1 E-115	500/515
Eukaryota	Fungi	Saccharomyces cerevisiae JAY291	EEU07467.1	1 E-115	500/515
Eukaryota	Fungi	Schizosaccharomyces japonicus yFS275	XP_002175790.1	1 E-114	458/515
Eukaryota	Fungi	Lachancea thermotolerans CBS 6340	XP_002555882.1	1 E-110	490/515
Eukaryota	Fungi	Zygosaccharomyces rouxii CBS 732	XP_002498025.1	1 E-110	492/515
Eukaryota	Fungi	Nectria haematococca mpVI 77-13-4	EEU41129.1	1 E-105	489/515
Eukaryota	Fungi	Penicillium chrysogenum Wisconsin 54-1255	XP_002558669.1	3 E-96	416/515
Eukaryota	Fungi	Aspergillus clavatus NRRL 1	XP_001270259.1	1 E-95	486/515
Eukaryota	Fungi	Clavispora lusitaniae ATCC 42720	XP_002618822.1	2 E-89	453/515
Eukaryota	Fungi	Aspergillus fumigatus Af293	XP_747804.1	4 E-89	487/515
Eukaryota	Fungi	Neosartorya fischeri NRRL 181	XP_001257464.1	2 E-88	485/515
Eukaryota	Fungi	Aspergillus nidulans FGSC A4	CBF78114.1	4 E-88	471/515
Eukaryota	Fungi	Aspergillus nidulans FGSC A4	XP_682014.1	8 E-88	457/515
Eukaryota	Fungi	Penicillium chrysogenum Wisconsin 54-1255	XP_002566305.1	4 E-87	482/515
Eukaryota	Fungi	Aspergillus terreus NIH2624	XP_001209868.1	3 E-86	462/515
Eukaryota	Fungi	Aspergillus nidulans FGSC A4	CBF81477.1	2 E-84	443/515
Eukaryota	Fungi	Phaeosphaeria nodorum SN15	XP_001799104.1	5 E-84	416/515
Eukaryota	Fungi	Uncinocarpus reesii 1704	XP_002545174.1	1 E-82	465/515
Eukaryota	Fungi	Gibberella zeae PH-1	XP_391245.1	1 E-82	448/515
Eukaryota	Fungi	Nectria haematococca mpVI 77-13-4	EEU40075.1	9 E-82	435/515
Eukaryota	Fungi	Gibberella zeae PH-1	XP_390985.1	1 E-81	463/515
Eukaryota	Fungi	Pyrenophora tritici-repentis Pt-1C-BFP	XP_001932746.1	2 E-81	478/515

Eukaryota	Fungi	Aspergillus nidulans FGSC A4	XP_658597.1	5 E-81	429/515
Eukaryota	Fungi	Aspergillus oryzae RIB40	XP_001823914.1	1 E-80	489/515
Eukaryota	Fungi	Aspergillus niger CBS 513.88	XP_001395883.1	7 E-80	488/515
Eukaryota	Fungi	Verticillium albo-atrum VaMs.102	EEY14812.1	1 E-79	446/515
Eukaryota	Fungi	Verticillium albo-atrum VaMs.102	EEY17356.1	1 E-79	448/515
Eukaryota	Fungi	Coccidioides posadasii C735 delta	EER26968.1	1 E-79	472/515
Eukaryota	Fungi	Coccidioides immitis RS;	XP_001240142.1	6 E-79	472/515
Eukaryota	Fungi	Aspergillus nidulans FGSC A4	XP_663254.1	9 E-79	422/515
Eukaryota	Fungi	Verticillium albo-atrum VaMs.102	EEY22343.1	2 E-78	482/515
Eukaryota	Fungi	Aspergillus terreus NIH2624	XP_001214217.1	2 E-76	484/515
Eukaryota	Fungi	Cryptococcus neoformans var. neoformans	XP_568590.1	3 E-76	437/515
Eukaryota	Fungi	Podospora anserina DSM 980	XP_001903172.1	1 E-75	473/515
Eukaryota	Fungi	Aspergillus clavatus NRRL 1	XP_001268230.1	2 E-75	467/515
Eukaryota	Fungi	Ajellomyces capsulatus G186AR	EEH05165.1	3 E-75	432/515
Eukaryota	Fungi	Neosartorya fischeri NRRL 181	XP_001259142.1	5 E-75	464/515
Eukaryota	Fungi	Aspergillus fumigatus Af293	XP_748702.2	5 E-75	468/515
Eukaryota	Fungi	Cryptococcus neoformans var. neoformans	XP_771875.1	9 E-74	438/515
Eukaryota	Fungi	Nectria haematococca mpVI 77-13-4	EEU41775.1	1 E-73	432/515
Eukaryota	Fungi	Aspergillus fumigatus Af293	XP_751016.1	2 E-73	458/515
Eukaryota	Fungi	Neurospora crassa OR74A	XP_960768.1	2 E-73	494/515
Eukaryota	Fungi	Ajellomyces dermatitidis SLH14081	XP_002628694.1	3 E-73	414/515
Eukaryota	Fungi	Penicillium chrysogenum Wisconsin 54-1255	XP_002569166.1	5 E-73	467/515
Eukaryota	Fungi	Ajellomyces dermatitidis ER-3	EEQ89497.1	1 E-72	414/515
Eukaryota	Fungi	Yarrowia lipolytica CLIB122	XP_504181.1	5 E-72	511/515
Eukaryota	Fungi	Yarrowia lipolytica CLIB122	XP_502728.1	6 E-72	495/515
Eukaryota	Fungi	Aspergillus clavatus NRRL 1	XP_001273552.1	9 E-72	464/515
Eukaryota	Fungi	Aspergillus oryzae RIB40	XP_001817321.1	1 E-71	491/515
Eukaryota	Fungi	Magnaporthe grisea 70-15	XP_360948.1	3 E-71	424/515
Eukaryota	Fungi	Aspergillus oryzae RIB40	XP_001821025.1	2 E-70	441/515
Eukaryota	Fungi	Gibberella zeae PH-1	XP_391395.1	4 E-70	486/515
Eukaryota	Fungi	Ashbya gossypii ATCC 10895	NP_984426.1	5 E-70	474/515
Eukaryota	Fungi	Gibberella zeae PH-1	XP_382162.1	6 E-70	510/515
Eukaryota	Fungi	Nectria haematococca mpVI 77-13-4	EEU40206.1	2 E-69	478/515
Eukaryota	Fungi	Ajellomyces capsulatus G186AR	EEH09066.1	2 E-69	456/515
Eukaryota	Fungi	Ajellomyces capsulatus H143	EER44176.1	2 E-69	456/515
Eukaryota	Fungi	Verticillium albo-atrum VaMs.102	EEY23096.1	2 E-69	440/515
Eukaryota	Fungi	Aspergillus niger CBS 513.88	XP_001392855.1	2 E-69	425/515
Eukaryota	Fungi	Botryotinia fuckeliana B05.10	XP_001560573.1	3 E-69	443/515
Eukaryota	Fungi	Aspergillus terreus NIH2624	XP_001210467.1	3 E-69	487/515
Eukaryota	Fungi	Microsporum canis CBS 113480	EEQ27183.1	3 E-69	443/515
Eukaryota	Fungi	Gibberella zeae PH-1	XP_385414.1	4 E-69	479/515
Eukaryota	Fungi	Magnaporthe grisea 70-15	XP_001522931.1	4 E-69	467/515
Eukaryota	Fungi	Nectria haematococca mpVI 77-13-4	EEU35406.1	7 E-69	447/515
Eukaryota	Fungi	Phaeosphaeria nodorum SN15	XP_001794417.1	1 E-68	493/515
Eukaryota	Fungi	Nectria haematococca mpVI 77-13-4	EEU41890.1	2 E-68	469/515
Eukaryota	Fungi	Ajellomyces dermatitidis SLH14081	XP_002629167.1	2 E-68	456/515
Eukaryota	Fungi	Ajellomyces dermatitidis ER-3	EEQ85804.1	3 E-68	456/515
Eukaryota	Fungi	Penicillium marneffei ATCC 18224	XP_002153612.1	3 E-68	457/515
Eukaryota	Fungi	Nectria haematococca mpVI 77-13-4	EEU38654.1	4 E-68	500/515
Eukaryota	Fungi	Sclerotinia sclerotiorum 1980 UF-70	XP_001598403.1	4 E-68	474/515
Eukaryota	Fungi	Aspergillus flavus NRRL3357	XP_002379738.1	6 E-68	505/515
Eukaryota	Fungi	Aspergillus oryzae RIB40	XP_001821692.1	9 E-68	505/515
Eukaryota	Fungi	Aspergillus flavus NRRL3357	XP_002372380.1	1 E-67	509/515
Eukaryota	Fungi	Magnaporthe grisea 70-15	XP_369040.1	1 E-67	457/515
Eukaryota	Fungi	Verticillium albo-atrum VaMs.102	EEY15295.1	2 E-67	416/515
Eukaryota	Fungi	Nectria haematococca mpVI 77-13-4	EEU43754.1	2 E-67	440/515
Eukaryota	Fungi	Penicillium marneffei ATCC 18224	XP_002151032.1	2 E-67	492/515
Eukaryota	Fungi	Sclerotinia sclerotiorum 1980 UF-70	XP_001596684.1	2 E-67	488/515

Eukaryota	Fungi	Aspergillus fumigatus Af293	XP_749930.2	3 E-67	494/515
Eukaryota	Fungi	Ajellomyces dermatitidis ER-3	EEQ90684.1	3 E-67	447/515
Eukaryota	Fungi	Talaromyces stipitatus ATCC 10500	XP_002482696.1	3 E-67	485/515
Eukaryota	Fungi	Magnaporthe grisea 70-15	XP_365417.1	4 E-67	474/515
Eukaryota	Fungi	Nectria haematococca mpVI 77-13-4	EEU33699.1	4 E-67	470/515
Eukaryota	Fungi	Vanderwaltozyma polyspora DSM 70294	XP_001643892.1	5 E-67	426/515
Eukaryota	Fungi	Nectria haematococca mpVI 77-13-4	EEU33931.1	5 E-67	466/515
Eukaryota	Fungi	Cryptococcus neoformans var. neoformans	XP_570093.1	6 E-67	431/515
Eukaryota	Fungi	Penicillium marneffei ATCC 18224	XP_002151499.1	7 E-67	490/515
Eukaryota	Fungi	Ajellomyces dermatitidis SLH14081	XP_002622994.1	1 E-66	447/515
Eukaryota	Fungi	Neosartorya fischeri NRRL 181	XP_001267558.1	1 E-66	501/515
Eukaryota	Fungi	Talaromyces stipitatus ATCC 10500	XP_002482697.1	2 E-66	457/515
Eukaryota	Fungi	Aspergillus niger CBS 513.88	XP_001390468.1	2 E-66	461/515
Eukaryota	Fungi	Kluyveromyces lactis NRRL Y-1140	XP_451413.1	2 E-66	451/515
Eukaryota	Fungi	Aspergillus nidulans FGSC A4	XP_657979.1	4 E-66	508/515
Eukaryota	Fungi	Talaromyces stipitatus ATCC 10500	XP_002487817.1	4 E-66	452/515
Eukaryota	Fungi	Aspergillus clavatus NRRL 1	XP_001269961.1	1 E-65	505/515
Eukaryota	Fungi	Candida glabrata CBS 138	XP_446332.1	1 E-65	482/515
Eukaryota	Fungi	Yarrowia lipolytica CLIB122	XP_505625.1	2 E-65	455/515
Eukaryota	Fungi	Nectria haematococca mpVI 77-13-4	EEU37009.1	3 E-65	455/515
Eukaryota	Fungi	Nectria haematococca mpVI 77-13-4	EEU44477.1	4 E-65	477/515
Eukaryota	Fungi	Phaeosphaeria nodorum SN15	XP_001796110.1	5 E-65	440/515
Eukaryota	Fungi	Phaeosphaeria nodorum SN15	XP_001792077.1	6 E-65	511/515
Eukaryota	Fungi	Talaromyces stipitatus ATCC 10500	XP_002484776.1	6 E-65	478/515
Eukaryota	Fungi	Neurospora crassa OR74A	XP_964938.1	8 E-65	492/515
Eukaryota	Fungi	Aspergillus nidulans FGSC A4	XP_682279.1	9 E-65	501/515
Eukaryota	Fungi	Laccaria bicolor S238N-H82	XP_001882726.1	1 E-64	453/515
Eukaryota	Fungi	Ajellomyces capsulatus G186AR	EEH03681.1	1 E-64	447/515
Eukaryota	Fungi	Coprinopsis cinerea okayama7#130	XP_001836599.1	2 E-64	467/515
Eukaryota	Fungi	Ajellomyces capsulatus G186AR	EEH04161.1	2 E-64	432/515
Eukaryota	Fungi	Coprinopsis cinerea okayama7#130	XP_001839739.1	2 E-64	462/515
Eukaryota	Fungi	Aspergillus terreus NIH2624	XP_001215462.1	2 E-64	468/515
Eukaryota	Fungi	Cryptococcus neoformans var. neoformans	XP_570441.1	2 E-64	426/515
Eukaryota	Fungi	Pyrenophora tritici-repentis Pt-1C-BFP	XP_001932888.1	3 E-64	491/515
Eukaryota	Fungi	Ajellomyces dermatitidis ER-3	EEQ86804.1	3 E-64	494/515
Eukaryota	Fungi	Cryptococcus neoformans var. neoformans	XP_776091.1	3 E-64	428/515
Eukaryota	Fungi	Coccidioides immitis RS;	XP_001247633.1	4 E-64	438/515
Eukaryota	Fungi	Paracoccidioides brasiliensis Pb01;	EEH35030.1	4 E-64	505/515
Eukaryota	Fungi	Paracoccidioides brasiliensis Pb18;	EEH45853.1	4 E-64	472/515
Eukaryota	Fungi	Penicillium chrysogenum Wisconsin 54-1255	XP_002559425.1	4 E-64	458/515
Eukaryota	Fungi	Coccidioides posadasii C735 delta	EER23654.1	5 E-64	438/515
Eukaryota	Fungi	Penicillium chrysogenum Wisconsin 54-1255	XP_002560051.1	9 E-64	467/515
Eukaryota	Fungi	Botryotinia fuckeliana B05.10	XP_001556070.1	1 E-63	508/515
Eukaryota	Fungi	Nectria haematococca mpVI 77-13-4	EEU41579.1	1 E-63	493/515
Eukaryota	Fungi	Ajellomyces dermatitidis SLH14081	XP_002625919.1	1 E-63	496/515
Eukaryota	Fungi	Ajellomyces capsulatus H143	EER42143.1	1 E-63	432/515
Eukaryota	Fungi	Coccidioides immitis RS;	XP_001242175.1	1 E-63	470/515
Eukaryota	Fungi	Aspergillus nidulans FGSC A4	CBF89610.1	1 E-63	492/515
Eukaryota	Fungi	Phaeosphaeria nodorum SN15	XP_001803475.1	2 E-63	458/515
Eukaryota	Fungi	Chaetomium globosum CBS 148.51	XP_001220439.1	2 E-63	475/515
Eukaryota	Fungi	Podospora anserina DSM 980	XP_001912789.1	2 E-63	494/515
Eukaryota	Fungi	Phaeosphaeria nodorum SN15	XP_001800057.1	2 E-63	551/515
Eukaryota	Fungi	Aspergillus nidulans FGSC A4	XP_659886.1	2 E-63	497/515
Eukaryota	Fungi	Botryotinia fuckeliana B05.10	XP_001559126.1	3 E-63	436/515
Eukaryota	Fungi	Gibberella zeae PH-1	XP_384393.1	3 E-63	493/515
Eukaryota	Fungi	Aspergillus terreus NIH2624	XP_001216196.1	3 E-63	487/515
Eukaryota	Fungi	Talaromyces stipitatus ATCC 10500	XP_002486254.1	3 E-63	505/515
Eukaryota	Fungi	Nectria haematococca mpVI 77-13-4	EEU39883.1	4 E-63	440/515

Eukaryota	Fungi	Talaromyces stipitatus ATCC 10500	XP_002483025.1	5 E-63	456/515
Eukaryota	Fungi	Aspergillus nidulans FGSC A4	XP_658714.1	5 E-63	485/515
Eukaryota	Fungi	Yarrowia lipolytica CLIB122	XP_505981.1	6 E-63	508/515
Eukaryota	Fungi	Penicillium marneffei ATCC 18224	XP_002149363.1	6 E-63	496/515
Eukaryota	Fungi	Aspergillus niger CBS 513.88	XP_001389066.1	6 E-63	510/515
Eukaryota	Fungi	Neosartorya fischeri NRRL 181	XP_001265470.1	1 E-62	505/515
Eukaryota	Fungi	Aspergillus flavus NRRL3357	XP_002375962.1	1 E-62	457/515
Eukaryota	Fungi	Yarrowia lipolytica CLIB122	XP_505987.1	1 E-62	498/515
Eukaryota	Fungi	Aspergillus clavatus NRRL 1	XP_001273963.1	2 E-62	465/515
Eukaryota	Fungi	Coccidioides posadasii C735 delta	EER28402.1	2 E-62	501/515
Eukaryota	Fungi	Saccharomyces cerevisiae AWRI1631	EDZ71891.1	3 E-62	504/515
Eukaryota	Fungi	Coprinopsis cinerea okayama7#130	XP_001839608.1	3 E-62	455/515
Eukaryota	Fungi	Paracoccidioides brasiliensis Pb01;	EEH38879.1	3 E-62	481/515
Eukaryota	Fungi	Uncinocarpus reesii 1704	XP_002541812.1	5 E-62	437/515
Eukaryota	Fungi	Saccharomyces cerevisiae	NP_011776.1	5 E-62	498/515
Eukaryota	Fungi	Aspergillus clavatus NRRL 1	XP_001269091.1	5 E-62	437/515
Eukaryota	Fungi	Uncinocarpus reesii 1704	XP_002585160.1	5 E-62	467/515
Eukaryota	Fungi	Saccharomyces cerevisiae EC1118	CAY80018.1	6 E-62	498/515
Eukaryota	Fungi	Aspergillus oryzae RIB40	XP_001727404.1	7 E-62	491/515
Eukaryota	Fungi	Magnaporthe grisea 70-15	XP_361210.2	9 E-62	419/515
Eukaryota	Fungi	Botryotinia fuckeliana B05.10	XP_001559197.1	1 E-61	474/515
Eukaryota	Fungi	Saccharomyces cerevisiae JAY291	EEU07648.1	1 E-61	498/515
Eukaryota	Fungi	Penicillium chrysogenum Wisconsin 54-1255	XP_002559510.1	2 E-61	432/515
Eukaryota	Fungi	Coccidioides immitis RS;	XP_001241518.1	2 E-61	457/515
Eukaryota	Fungi	Aspergillus oryzae RIB40	XP_001817055.1	2 E-61	453/515
Eukaryota	Fungi	Paracoccidioides brasiliensis Pb01;	EEH38500.1	2 E-61	472/515
Eukaryota	Fungi	Ustilago maydis 521	XP_758851.1	2 E-61	439/515
Eukaryota	Fungi	Paracoccidioides brasiliensis Pb03;	EEH21202.1	3 E-61	444/515
Eukaryota	Fungi	Coccidioides posadasii C735 delta	EER23741.1	3 E-61	431/515
Eukaryota	Fungi	Saccharomyces cerevisiae YJM789	EDN61845.1	3 E-61	498/515
Eukaryota	Fungi	Coprinopsis cinerea okayama7#130	XP_001830878.1	3 E-61	490/515
Eukaryota	Fungi	Aspergillus fumigatus Af293	XP_752548.1	3 E-61	458/515
Eukaryota	Fungi	Microsporum canis CBS 113480	EEQ34172.1	3 E-61	482/515
Eukaryota	Fungi	Yarrowia lipolytica CLIB122	XP_504459.1	4 E-61	469/515
Eukaryota	Fungi	Pyrenophora tritici-repentis Pt-1C-BFP	XP_001931676.1	7 E-61	479/515
Eukaryota	Fungi	Nectria haematococca mpVI 77-13-4	EEU42842.1	7 E-61	493/515
Eukaryota	Fungi	Phaeosphaeria nodorum SN15	XP_001792080.1	7 E-61	448/515
Eukaryota	Fungi	Phaeosphaeria nodorum SN15	XP_001792895.1	8 E-61	448/515
Eukaryota	Fungi	Podospora anserina DSM 980	XP_001904926.1	1 E-60	487/515
Eukaryota	Fungi	Penicillium chrysogenum Wisconsin 54-1255	XP_002566136.1	1 E-60	500/515
Eukaryota	Fungi	Pyrenophora tritici-repentis Pt-1C-BFP	XP_001939028.1	2 E-60	441/515
Eukaryota	Fungi	Gibberella zeae PH-1	XP_383291.1	2 E-60	416/515
Eukaryota	Fungi	Gibberella zeae PH-1	XP_384065.1	2 E-60	452/515
Eukaryota	Fungi	Microsporum canis CBS 113480	EEQ34529.1	3 E-60	519/515
Eukaryota	Fungi	Yarrowia lipolytica CLIB122	XP_504188.1	5 E-60	454/515
Eukaryota	Fungi	Clavispora lusitaniae ATCC 42720	XP_002616708.1	6 E-60	519/515
Eukaryota	Fungi	Paracoccidioides brasiliensis Pb18;	EEH43221.1	6 E-60	465/515
Eukaryota	Fungi	Gibberella zeae PH-1	XP_384607.1	7 E-60	493/515
Eukaryota	Fungi	Verticillium albo-atrum VaMs.102	EEY16792.1	1 E-59	502/515
Eukaryota	Fungi	Aspergillus clavatus NRRL 1	XP_001270398.1	1 E-59	437/515
Eukaryota	Fungi	Aspergillus fumigatus Af293	XP_748898.2	2 E-59	462/515
Eukaryota	Fungi	Aspergillus fumigatus A1163	EDP48453.1	2 E-59	462/515
Eukaryota	Fungi	Gibberella zeae PH-1	XP_387947.1	2 E-59	449/515
Eukaryota	Fungi	Aspergillus nidulans FGSC A4	XP_659948.1	3 E-59	439/515
Eukaryota	Fungi	Yarrowia lipolytica CLIB122	XP_505694.1	4 E-59	510/515
Eukaryota	Fungi	Coccidioides posadasii C735 delta	EER28038.1	4 E-59	451/515
Eukaryota	Fungi	Aspergillus oryzae RIB40	XP_001818910.1	4 E-59	461/515
Eukaryota	Fungi	Pyrenophora tritici-repentis Pt-1C-BFP	XP_001936718.1	4 E-59	455/515

Eukaryota	Fungi	Ajellomyces capsulatus H143	EER36908.1	5 E-59	481/515
Eukaryota	Fungi	Magnaporthe grisea 70-15	XP_363176.1	6 E-59	510/515
Eukaryota	Fungi	Uncinocarpus reesii 1704	XP_002541710.1	9 E-59	425/515
Eukaryota	Fungi	Coccidioides posadasii C735 delta	EER29478.1	1 E-58	466/515
Eukaryota	Fungi	Neosartorya fischeri NRRL 181	XP_001264579.1	1 E-58	425/515
Eukaryota	Fungi	Coccidioides immitis RS;	XP_001245211.1	1 E-58	466/515
Eukaryota	Fungi	Neurospora crassa OR74A	XP_956418.2	1 E-58	503/515
Eukaryota	Fungi	Pyrenophora tritici-repentis Pt-1C-BFP	XP_001935385.1	2 E-58	497/515
Eukaryota	Fungi	Sclerotinia sclerotiorum 1980 UF-70	XP_001589646.1	2 E-58	497/515
Eukaryota	Fungi	Gibberella zeae PH-1	XP_383822.1	2 E-58	509/515
Eukaryota	Fungi	Sclerotinia sclerotiorum 1980 UF-70	XP_001585100.1	2 E-58	433/515
Eukaryota	Fungi	Ustilago maydis 521	XP_762630.1	3 E-58	441/515
Eukaryota	Fungi	Ajellomyces dermatitidis ER-3	EEQ85240.1	3 E-58	435/515
Eukaryota	Fungi	Aspergillus oryzae RIB40	XP_001727662.1	3 E-58	472/515
Eukaryota	Fungi	Ajellomyces dermatitidis SLH14081	XP_002628931.1	4 E-58	435/515
Eukaryota	Fungi	Ajellomyces capsulatus NAm1	XP_001541196.1	4 E-58	494/515

AFUA_5G01690

Eukaryota	Fungi	Aspergillus fumigatus Af293	XP_748173.1	1 E-177	301/301
Eukaryota	Fungi	Neosartorya fischeri NRRL 181	XP_001266361.1	1 E-156	281/301
Eukaryota	Fungi	Aspergillus terreus NIH2624	XP_001218401.1	1 E-112	284/301
Eukaryota	Fungi	Aspergillus oryzae RIB40	XP_001820424.1	1 E-108	265/301
Eukaryota	Fungi	Aspergillus flavus NRRL3357	XP_002373984.1	1 E-108	265/301
Eukaryota	Fungi	Penicillium chrysogenum Wisconsin 54-1255	XP_002568440.1	1 E-106	266/301
Eukaryota	Fungi	Aspergillus niger CBS 513.88	XP_001397414.1	2 E-97	274/301
Eukaryota	Fungi	Talaromyces stipitatus ATCC 10500	XP_002486618.1	9 E-76	286/301
Eukaryota	Fungi	Kluyveromyces lactis NRRL Y-1140	XP_453049.1	4 E-71	272/301
Eukaryota	Fungi	Nectria haematococca mpVI 77-13-4	EEU46414.1	9 E-70	279/301
Eukaryota	Fungi	Lachancea thermotolerans CBS 6340	XP_002552224.1	4 E-69	264/301
Eukaryota	Fungi	Botryotinia fuckeliana B05.10	XP_001553452.1	4 E-67	273/301
Eukaryota	Fungi	Sclerotinia sclerotiorum 1980 UF-70	XP_001595335.1	4 E-67	278/301
Eukaryota	Fungi	Verticillium albo-atrum VaMs.102	EEY14610.1	1 E-66	269/301
Eukaryota	Fungi	Clavispora lusitaniae ATCC 42720	XP_002618823.1	4 E-66	263/301
Eukaryota	Fungi	Magnaporthe grisea 70-15	XP_361373.1	3 E-65	294/301
Eukaryota	Fungi	Zygosaccharomyces rouxii CBS 732	XP_002495675.1	3 E-63	256/301
Eukaryota	Fungi	Cryptococcus neoformans var. neoformans	XP_571051.1	1 E-62	261/301
Eukaryota	Fungi	Schizosaccharomyces pombe	NP_592814.1	2 E-60	250/301
Bacteria	Firmicutes	Staphylococcus haemolyticus JCSC1435	YP_252189.1	1 E-52	256/301
Bacteria	Proteobacteria	Rhodobacterales bacterium HTCC2150	ZP_01742079.1	9 E-52	261/301
Bacteria	Firmicutes	Bacillus licheniformis ATCC 14580	YP_077736.1	1 E-51	250/301
Bacteria	Proteobacteria	Rhizobium sp. NGR234	YP_002824185.1	1 E-51	266/301
Eukaryota	Fungi	Penicillium marneffei ATCC 18224	XP_002152861.1	1 E-51	245/301
Bacteria	Actinobacteria	Corynebacterium accolens ATCC 49725	ZP_03932895.1	2 E-51	247/301
Bacteria	Planctomycetes	Planctomyces maris DSM 8797	ZP_01857454.1	2 E-51	257/301
Bacteria	Proteobacteria	Bradyrhizobium sp. ORS278	YP_001204595.1	3 E-51	274/301
Bacteria	Firmicutes	Macrococcus caseolyticus JCSC5402	YP_002560885.1	4 E-51	248/301
Bacteria	Proteobacteria	Bordetella petrii DSM 12804	YP_001630844.1	4 E-51	250/301
Bacteria	Proteobacteria	Bradyrhizobium sp. ORS278	A4YR32.2	6 E-51	272/301
Bacteria	Actinobacteria	Corynebacterium aurimucosum ATCC 700975	YP_002834123.1	6 E-51	247/301
Bacteria	Proteobacteria	Azorhizobium caulinodans ORS 571	YP_001526996.1	7 E-51	243/301
Bacteria	Proteobacteria	Campylobacter coli RM2228	ZP_00370842.1	2 E-50	245/301
Bacteria	Candidatus	Candidatus Pelagibacter ubique HTCC1062	YP_266083.1	2 E-50	257/301
Bacteria	Proteobacteria	Roseobacter sp. AzwK-3b	ZP_01901298.1	3 E-50	268/301
Bacteria	Actinobacteria	Corynebacterium urealyticum DSM 7109	YP_001800975.1	6 E-50	257/301
Bacteria	Proteobacteria	Rhodobacterales bacterium HTCC2083	ZP_05073336.1	1 E-49	261/301
Bacteria	Proteobacteria	Bradyrhizobium japonicum USDA 110	NP_769561.1	2 E-49	241/301
Bacteria	Proteobacteria	Neptuniibacter caesariensis	ZP_01166283.1	2 E-49	245/301

Bacteria	Proteobacteria	Bordetella pertussis	Q7VXB5Y1875	3 E-49	254/301
Bacteria	Proteobacteria	Bordetella parapertussis 12822	NP_885216.1	3 E-49	254/301
Bacteria	Proteobacteria	Bordetella pertussis Tohama I	NP_880567.1	3 E-49	255/301
Bacteria	Actinobacteria	Corynebacterium tuberculostearicum SK141	ZP_05364997.1	3 E-49	246/301
Bacteria	Firmicutes	Thermosinus carboxydivorans Nor1	ZP_01666056.1	4 E-49	256/301
Bacteria	Chloroflexi	Sphaerobacter thermophilus DSM 20745	ZP_04495871.1	4 E-49	263/301
Bacteria	Actinobacteria	Corynebacterium pseudogenitalium ATCC 3303	ZP_03920114.1	4 E-49	246/301
Bacteria	Firmicutes	Bacillus subtilis subsp. subtilis	NP_388289.3	7 E-49	252/301
Bacteria	Proteobacteria	Methylobacterium nodulans ORS 2060	YP_002496407.1	7 E-49	247/301
Bacteria	Proteobacteria	Rhodopseudomonas palustris TIE-1	YP_001990945.1	7 E-49	254/301
Bacteria	Proteobacteria	Rhodopseudomonas palustris CGA009	NP_947092.1	8 E-49	254/301
Bacteria	Firmicutes	Clostridium sp. M62/1	ZP_03730968.1	9 E-49	265/301
Bacteria	Proteobacteria	gamma proteobacterium NOR51-B	ZP_04958270.1	1 E-48	245/301
Bacteria	Proteobacteria	Rhodopseudomonas palustris BisB18	YP_531546.1	1 E-48	254/301
Bacteria	Proteobacteria	Rhizobium leguminosarum bv. viciae	YP_768034.1	1 E-48	257/301
Bacteria	Proteobacteria	Polaromonas sp. JS666	YP_548610.1	2 E-48	273/301
Bacteria	Proteobacteria	Rhodopseudomonas palustris BisB5	YP_568983.1	2 E-48	254/301
Bacteria	Proteobacteria	Congregibacter litoralis KT71	ZP_01101328.1	2 E-48	262/301
Bacteria	Actinobacteria	Corynebacterium jeikeium K411	YP_250178.1	3 E-48	245/301
Bacteria	Proteobacteria	Bradyrhizobium sp. BTAi1	YP_001238916.1	4 E-48	254/301
Bacteria	Firmicutes	Geobacillus kaustophilus HTA426	YP_147956.1	4 E-48	256/301
Bacteria	Proteobacteria	marine gamma proteobacterium HTCC2080	ZP_01625389.1	5 E-48	243/301
Bacteria	Firmicutes	Bacillus amyloliquefaciens FZB42	YP_001420060.1	5 E-48	263/301
Bacteria	Proteobacteria	Aliivibrio salmonicida LFI1238	YP_002262883.1	7 E-48	251/301
Bacteria	Firmicutes	Staphylococcus carnosus subsp. carnosus	YP_002635298.1	1 E-47	247/301
Bacteria	Firmicutes	Staphylococcus saprophyticus subsp. saprophyti	YP_300398.1	1 E-47	244/301
Bacteria	Actinobacteria	Arthrobacter sp. FB24	YP_833051.1	1 E-47	266/301
Bacteria	Proteobacteria	Variovorax paradoxus S110	YP_002945176.1	2 E-47	257/301
Bacteria	Actinobacteria	Bifidobacterium adolescentis L2-32	ZP_02027839.1	2 E-47	247/301
Bacteria	Proteobacteria	Rhodopseudomonas palustris BisA53	YP_780621.1	3 E-47	254/301
Bacteria	Actinobacteria	Brevibacterium linens BL2	ZP_05915630.1	4 E-47	243/301
Bacteria	Proteobacteria	Vibrio fischeri MJ11	YP_002156177.1	4 E-47	260/301
Bacteria	Proteobacteria	Gluconacetobacter diazotrophicus PAl 5	YP_002276628.1	5 E-47	249/301
Bacteria	Proteobacteria	Rhodopseudomonas palustris HaA2	YP_487227.1	7 E-47	254/301
Bacteria	Proteobacteria	Roseovarius sp. HTCC2601	ZP_01445064.1	1 E-46	247/301
Bacteria	Proteobacteria	Roseovarius sp. 217	ZP_01036570.1	1 E-46	248/301
Bacteria	Actinobacteria	Bifidobacterium adolescentis ATCC 15703	YP_908940.1	2 E-46	247/301
Bacteria	Proteobacteria	Vibrio fischeri ES114	YP_204760.1	2 E-46	251/301
Bacteria	Proteobacteria	Xanthobacter autotrophicus Py2	YP_001416408.1	3 E-46	250/301
Bacteria	Firmicutes	Acidaminococcus sp. D21	ZP_03929152.1	3 E-46	263/301
Bacteria	Actinobacteria	Bifidobacterium adolescentis ATCC 15703	A0ZZH5.2	3 E-46	247/301
Bacteria	Proteobacteria	Agrobacterium tumefaciens str. C58	NP_356723.1	3 E-46	261/301
Bacteria	Firmicutes	Dorea formicigenerans ATCC 27755	ZP_02233325.1	4 E-46	258/301
Bacteria	Proteobacteria	Gluconacetobacter diazotrophicus PAl 5	YP_001600478.1	5 E-46	249/301
Bacteria	Actinobacteria	Nocardioides sp. JS614	YP_921325.1	6 E-46	260/301
Bacteria	Proteobacteria	Gluconacetobacter diazotrophicus PAl 5	A9H2B4.2	6 E-46	249/301
Bacteria	Firmicutes	Bacillus subtilis subsp. subtilis	ZP_03590071.1	7 E-46	259/301
Bacteria	Firmicutes	Bacillus pumilus SAFR-032	YP_001485636.1	8 E-46	251/301
Bacteria	Firmicutes	Thermoanaerobacter ethanolicus CCSD1	ZP_05493686.1	9 E-46	242/301
Bacteria	Firmicutes	Thermoanaerobacter pseudethanolicus ATCC 3:	YP_001665151.1	1 E-45	242/301
Bacteria	Proteobacteria	Roseovarius nubinhibens ISM	ZP_00960231.1	1 E-45	243/301
Bacteria	Proteobacteria	Methylobacterium sp. 4-46	YP_001769021.1	1 E-45	256/301
Bacteria	Firmicutes	Bacillus subtilis	BAA07360.1	5 E-45	252/301
Bacteria	Actinobacteria	Eggerthella lenta DSM 2243	YP_003180557.1	1 E-44	242/301
Bacteria	Proteobacteria	Syntrophobacter fumaroxidans MPOB	YP_847501.1	1 E-44	248/301
Bacteria	Firmicutes	Bacillus pumilus ATCC 7061	ZP_03054571.1	1 E-44	251/301
Bacteria	Firmicutes	Clostridium hylemonae DSM 15053	ZP_03780211.1	1 E-44	249/301
Bacteria	Proteobacteria	Acinetobacter sp. RUH2624	ZP_05823817.1	2 E-44	246/301

Bacteria	Proteobacteria	Acinetobacter baumannii AYE	YP_001714270.1	2 E-44	246/301
Bacteria	Actinobacteria	Bifidobacterium gallicum DSM 20093	ZP_05966578.1	2 E-44	248/301
Bacteria	Proteobacteria	Acinetobacter baumannii ATCC 17978	A3M452.2	3 E-44	246/301
Bacteria	Proteobacteria	Acinetobacter baumannii SDF	YP_001707542.1	3 E-44	246/301
Bacteria	Proteobacteria	Acinetobacter baumannii ATCC 19606	ZP_05827768.1	3 E-44	246/301
Bacteria	Proteobacteria	Pseudomonas syringae pv. tomato	ZP_03398938.1	4 E-44	246/301
Bacteria	Proteobacteria	Acinetobacter baumannii ACICU	YP_001845927.1	6 E-44	246/301
Bacteria	Actinobacteria	Geodermatophilus obscurus DSM 43160	ZP_03892541.1	6 E-44	241/301
Bacteria	Proteobacteria	Bermanella marisrubri	ZP_01306927.1	6 E-44	241/301
Bacteria	Proteobacteria	Providencia alcalifaciens DSM 30120	ZP_03320500.1	6 E-44	246/301
Bacteria	Proteobacteria	Marinomonas sp. MED121	ZP_01077023.1	6 E-44	261/301
Bacteria	Proteobacteria	Providencia rettgeri DSM 1131	ZP_03639441.1	9 E-44	246/301
Bacteria	Proteobacteria	Acinetobacter sp. ADP1	YP_047108.1	9 E-44	248/301
Bacteria	Proteobacteria	Pseudomonas syringae pv. tomato	NP_795106.1	9 E-44	246/301
Bacteria	Proteobacteria	Providencia rustigianii DSM 4541	ZP_05973761.1	2 E-43	246/301
Bacteria	Proteobacteria	Psychrobacter arcticus 273-4	YP_264388.1	3 E-43	258/301
Bacteria	Firmicutes	Clostridium butyricum 5521	ZP_02950477.1	6 E-43	257/301
Bacteria	Synergistetes	Dethiosulfovibrio peptidovorans DSM 11002	ZP_04340475.1	9 E-43	249/301
Bacteria	Proteobacteria	Pseudomonas aeruginosa PA7	YP_001348554.1	9 E-43	241/301
Bacteria	Firmicutes	Clostridium beijerinckii NCIMB 8052	YP_001309867.1	1 E-42	250/301
Bacteria	Proteobacteria	Pseudomonas aeruginosa UCBPP-PA14	YP_791130.1	1 E-42	247/301
Bacteria	Proteobacteria	Pseudomonas aeruginosa C3719	ZP_04928466.1	1 E-42	247/301
Bacteria	Actinobacteria	Mycobacterium gilvum PYR-GCK	YP_001136448.1	2 E-42	258/301
Bacteria	Proteobacteria	Pseudomonas aeruginosa PACS2	ZP_01365455.1	2 E-42	247/301
Bacteria	Proteobacteria	Pseudomonas aeruginosa LESB58	YP_002440801.1	2 E-42	247/301
Bacteria	Proteobacteria	Pseudomonas aeruginosa 2192	ZP_04933865.1	2 E-42	247/301
Bacteria	Actinobacteria	Catenulispora acidiphila DSM 44928	YP_003115129.1	3 E-42	243/301
Bacteria	Proteobacteria	Pseudomonas aeruginosa PAO1	NP_250806.1	6 E-42	247/301
Bacteria	Proteobacteria	Pseudomonas syringae pv. oryzae	ZP_04586659.1	2 E-41	255/301
Bacteria	Actinobacteria	Streptomyces coelicolor A3(2)	NP_625694.1	3 E-41	242/301
Bacteria	Actinobacteria	Streptomyces lividans TK24	ZP_05527417.1	4 E-41	242/301
Bacteria	Proteobacteria	Proteus penneri ATCC 35198	ZP_03804982.1	4 E-41	253/301
Bacteria	Actinobacteria	Streptomyces griseoflavus Tu4000	ZP_05542540.1	1 E-40	242/301
Bacteria	Proteobacteria	Pseudomonas syringae pv. syringae	YP_233606.1	3 E-40	255/301
Bacteria	Actinobacteria	Streptomyces scabiei 87.22	CBG74575.1	3 E-40	252/301
Bacteria	Actinobacteria	Micrococcus luteus NCTC 2665	YP_002957958.1	8 E-40	259/301
Bacteria	Actinobacteria	Streptomyces ambofaciens ATCC 23877	CAJ90355.1	3 E-39	242/301
Bacteria	Proteobacteria	Paracoccus denitrificans PD1222	YP_914131.1	6 E-39	250/301
Bacteria	Actinobacteria	Streptomyces avermitilis MA-4680	NP_828116.1	1 E-38	252/301
Bacteria	Proteobacteria	Oligotropha carboxidovorans OM5	YP_002290327.1	4 E-38	259/301
Bacteria	Actinobacteria	Streptomyces ghanaensis ATCC 14672	ZP_04689453.1	2 E-37	242/301
Eukaryota	stramenopiles	Phaeodactylum tricornutum CCAP 1055/1	XP_002181781.1	2 E-37	243/301
Eukaryota	Metazoa	Xenopus (Silurana) tropicalis	CAJ82045.1	5 E-37	245/301
Eukaryota	Metazoa	Xenopus (Silurana) tropicalis	NP_001016461.2	5 E-37	245/301
Eukaryota	Metazoa	Nematostella vectensis	XP_001640633.1	7 E-37	243/301
Eukaryota	Metazoa	Danio rerio	XP_001923891.1	8 E-36	244/301
Eukaryota	Metazoa	Danio rerio	XP_001923890.1	2 E-33	250/301
Eukaryota	Alveolata	Perkinsus marinus ATCC 50983	EER17334.1	2 E-33	243/301
Eukaryota	Metazoa	Ornithorhynchus anatinus	XP_001514386.1	1 E-28	262/301
Eukaryota	Metazoa	Gallus gallus	XP_421319.2	6 E-28	244/301
Eukaryota	Metazoa	Strongylocentrotus purpuratus	XP_782739.1	6 E-26	246/301
Eukaryota	Metazoa	Homo sapiens	CAD66589.1	3 E-24	248/301
Eukaryota	Metazoa	Homo sapiens	CAD62323.1	3 E-24	248/301
Eukaryota	Metazoa	Homo sapiens	NP_001095838.1	3 E-24	248/301
Eukaryota	Metazoa	Homo sapiens	BAG53908.1	3 E-24	248/301
Eukaryota	Metazoa	Homo sapiens	CAG33570.1	3 E-24	248/301
Eukaryota	Metazoa	Homo sapiens	BAE06113.1	4 E-24	248/301
Eukaryota	Metazoa	Homo sapiens	CAD62622.1	5 E-24	242/301

Eukaryota	Metazoa	Homo sapiens	CAD97934.1	9 E-24	248/301
Eukaryota	Metazoa	Pan troglodytes	XP_510121.2	3 E-23	248/301
Eukaryota	Metazoa	Homo sapiens	EAW81442.1	4 E-22	243/301

AFUA_5G01700

Eukaryota	Fungi	Aspergillus fumigatus Af293	XP_748172.1	0.0	662/662
Eukaryota	Fungi	Neosartorya fischeri NRRL 181	XP_001266360.1	0.0	575/662
Eukaryota	Fungi	Aspergillus clavatus NRRL 1	XP_001276374.1	0.0	726/662
Eukaryota	Fungi	Aspergillus terreus NIH2624	XP_001218408.1	0.0	684/662
Eukaryota	Fungi	Aspergillus niger CBS 513.88	XP_001397415.1	0.0	693/662
Eukaryota	Fungi	Penicillium chrysogenum Wisconsin 54-1255	XP_002568442.1	0.0	696/662
Eukaryota	Fungi	Aspergillus nidulans FGSC A4	CBF73839.1	0.0	647/662
Eukaryota	Fungi	Aspergillus flavus NRRL3357	XP_002373985.1	0.0	629/662
Eukaryota	Fungi	Aspergillus nidulans FGSC A4	XP_681348.1	0.0	639/662
Eukaryota	Fungi	Talaromyces stipitatus ATCC 10500	XP_002482594.1	1 E-170	775/662
Eukaryota	Fungi	Penicillium marneffei ATCC 18224	XP_002148335.1	1 E-164	632/662
Eukaryota	Fungi	Neosartorya fischeri NRRL 181	XP_001267398.1	1 E-145	706/662
Eukaryota	Fungi	Coccidioides posadasii C735 delta	EER26054.1	1 E-144	695/662
Eukaryota	Fungi	Coccidioides immitis RS;	XP_001244765.1	1 E-144	695/662
Eukaryota	Fungi	Aspergillus flavus NRRL3357	XP_002377246.1	1 E-142	705/662
Eukaryota	Fungi	Aspergillus clavatus NRRL 1	XP_001271512.1	1 E-141	706/662
Eukaryota	Fungi	Penicillium marneffei ATCC 18224	XP_002144424.1	1 E-139	657/662
Eukaryota	Fungi	Ajellomyces capsulatus H143	EER43475.1	1 E-139	742/662
Eukaryota	Fungi	Ajellomyces capsulatus G186AR	EEH08697.1	1 E-139	742/662
Eukaryota	Fungi	Talaromyces stipitatus ATCC 10500	XP_002341114.1	1 E-138	663/662
Eukaryota	Fungi	Aspergillus nidulans FGSC A4	CBF77756.1	1 E-138	687/662
Eukaryota	Fungi	Paracoccidioides brasiliensis Pb01;	EEH33345.1	1 E-135	715/662
Eukaryota	Fungi	Ajellomyces dermatitidis SLH14081	XP_002623031.1	1 E-134	716/662
Eukaryota	Fungi	Ajellomyces dermatitidis ER-3	EEQ83543.1	1 E-134	716/662
Eukaryota	Fungi	Aspergillus oryzae RIB40	XP_001821461.1	1 E-133	720/662
Eukaryota	Fungi	Pyrenophora tritici-repentis Pt-1C-BFP	XP_001931155.1	1 E-133	710/662
Eukaryota	Fungi	Paracoccidioides brasiliensis Pb03;	EEH21987.1	1 E-132	715/662
Eukaryota	Fungi	Uncinocarpus reesii 1704	XP_002584945.1	1 E-127	594/662
Eukaryota	Fungi	Microsporum canis CBS 113480	EEQ28444.1	1 E-123	605/662
Eukaryota	Fungi	Paracoccidioides brasiliensis Pb18;	EEH48484.1	1 E-123	697/662
Eukaryota	Fungi	Penicillium chrysogenum Wisconsin 54-1255	XP_002565595.1	1 E-122	695/662
Eukaryota	Fungi	Aspergillus niger CBS 513.88	XP_001401414.1	1 E-122	659/662
Eukaryota	Fungi	Aspergillus fumigatus Af293	XP_752205.1	1 E-121	649/662
Eukaryota	Fungi	Aspergillus nidulans FGSC A4	XP_661930.1	1 E-121	633/662
Eukaryota	Fungi	Aspergillus terreus NIH2624	XP_001214780.1	1 E-120	586/662
Eukaryota	Fungi	Gibberella zeae PH-1	XP_388556.1	1 E-114	655/662
Eukaryota	Fungi	Nectria haematococca mpVI 77-13-4	EEU47277.1	1 E-113	641/662
Eukaryota	Fungi	Verticillium albo-atrum VaMs.102	EEY23157.1	1 E-112	593/662
Eukaryota	Fungi	Neurospora crassa OR74A	XP_960498.1	1 E-103	607/662
Eukaryota	Fungi	Podospora anserina DSM 980	XP_001905737.1	2 E-90	550/662
Eukaryota	Fungi	Aspergillus flavus NRRL3357	XP_002378695.1	7 E-88	585/662
Eukaryota	Fungi	Verticillium albo-atrum VaMs.102	EEY23059.1	1 E-87	617/662
Eukaryota	Fungi	Penicillium chrysogenum Wisconsin 54-1255	XP_002559987.1	2 E-85	606/662
Eukaryota	Fungi	Magnaporthe grisea 70-15	XP_368926.1	1 E-79	575/662
Eukaryota	Fungi	Neosartorya fischeri NRRL 181	XP_001263004.1	2 E-79	704/662
Eukaryota	Fungi	Aspergillus clavatus NRRL 1	XP_001271412.1	4 E-79	596/662
Eukaryota	Fungi	Talaromyces stipitatus ATCC 10500	XP_002482571.1	1 E-78	591/662
Eukaryota	Fungi	Aspergillus niger CBS 513.88	XP_001393471.1	5 E-78	606/662
Eukaryota	Fungi	Penicillium marneffei ATCC 18224	XP_002148308.1	3 E-73	608/662
Eukaryota	Fungi	Podospora anserina DSM 980	XP_001905172.1	4 E-72	633/662
Eukaryota	Fungi	Nectria haematococca mpVI 77-13-4	EEU37687.1	4 E-68	583/662
Eukaryota	Fungi	Phaeosphaeria nodorum SN15	XP_001797511.1	1 E-65	601/662

Eukaryota	Fungi	Nectria haematococca mpVI 77-13-4	EEU42920.1	9 E-64	540/662
Eukaryota	Fungi	Penicillium marneffei ATCC 18224	XP_002148420.1	6 E-55	642/662
Eukaryota	Fungi	Talaromyces stipitatus ATCC 10500	XP_002482702.1	5 E-54	680/662
Eukaryota	Fungi	Nectria haematococca mpVI 77-13-4	EEU34682.1	3 E-30	627/662
Eukaryota	Fungi	Gibberella zeae PH-1	XP_387814.1	6 E-27	622/662
Eukaryota	Fungi	Sclerotinia sclerotiorum 1980 UF-70	XP_001588792.1	7 E-11	531/662

AFUA_5G01710

Eukaryota	Fungi	Aspergillus fumigatus Af293	XP_748171.1	0.0	515/515
Eukaryota	Fungi	Neosartorya fischeri NRRL 181	XP_001266359.1	0.0	515/515
Eukaryota	Fungi	Aspergillus terreus NIH2624	XP_001218407.1	0.0	513/515
Eukaryota	Fungi	Aspergillus clavatus NRRL 1	XP_001276373.1	0.0	513/515
Eukaryota	Fungi	Aspergillus flavus NRRL3357	XP_002373986.1	0.0	498/515
Eukaryota	Fungi	Aspergillus niger CBS 513.88	XP_001397416.1	0.0	513/515
Eukaryota	Fungi	Aspergillus oryzae	BAG68501.1	0.0	513/515
Eukaryota	Fungi	Aspergillus nidulans FGSC A4	XP_681347.1	0.0	514/515
Eukaryota	Fungi	Penicillium chrysogenum;;	AAF21760.1	0.0	513/515
Eukaryota	Fungi	Aspergillus oryzae RIB40	XP_001820421.1	0.0	498/515
Eukaryota	Fungi	Penicillium chrysogenum;;	AAF21759.1	0.0	513/515
Eukaryota	Fungi	Penicillium chrysogenum Wisconsin 54-1255	XP_002568443.1	0.0	513/515
Eukaryota	Fungi	Penicillium marneffei ATCC 18224	XP_002148333.1	0.0	514/515
Eukaryota	Fungi	Aspergillus terreus	AAD34565.1	0.0	513/515
Eukaryota	Fungi	Aspergillus terreus NIH2624	XP_001209275.1	0.0	513/515
Eukaryota	Fungi	Talaromyces stipitatus ATCC 10500	XP_002482593.1	0.0	514/515
Eukaryota	Fungi	Ajellomyces capsulatus G186AR	EEH08392.1	0.0	513/515
Eukaryota	Fungi	Ajellomyces capsulatus H143	EER42365.1	0.0	513/515
Eukaryota	Fungi	Ajellomyces dermatitidis SLH14081	XP_002628930.1	0.0	513/515
Eukaryota	Fungi	Coccidioides immitis RS;	XP_001243121.1	0.0	513/515
Eukaryota	Fungi	Coccidioides posadasii C735 delta	EER28040.1	0.0	513/515
Eukaryota	Fungi	Microsporum canis CBS 113480	EEQ30995.1	0.0	514/515
Eukaryota	Fungi	Paracoccidioides brasiliensis Pb18;	EEH42449.1	0.0	513/515
Eukaryota	Fungi	Paracoccidioides brasiliensis Pb03;	EEH21615.1	0.0	513/515
Eukaryota	Fungi	Uncinocarpus reesii 1704	XP_002585159.1	0.0	513/515
Eukaryota	Fungi	Sclerotinia sclerotiorum 1980 UF-70	XP_001592633.1	0.0	513/515
Eukaryota	Fungi	Nectria haematococca mpVI 77-13-4	EEU37697.1	0.0	510/515
Eukaryota	Fungi	Magnaporthe grisea 70-15	XP_362239.1	0.0	510/515
Eukaryota	Fungi	Phaeosphaeria nodorum SN15	XP_001797234.1	0.0	475/515
Eukaryota	Fungi	Pyrenophora tritici-repentis Pt-1C-BFP	XP_001931215.1	0.0	422/515
Eukaryota	Fungi	Gibberella zeae PH-1	XP_383917.1	1 E-179	436/515
Eukaryota	Fungi	Yarrowia lipolytica CLIB122	XP_504958.1	1 E-139	512/515
Eukaryota	Fungi	Nectria haematococca mpVI 77-13-4	EEU35728.1	1 E-135	520/515
Eukaryota	Fungi	Penicillium marneffei ATCC 18224	XP_002148107.1	1 E-134	523/515
Eukaryota	Fungi	Talaromyces stipitatus ATCC 10500	XP_002482351.1	1 E-134	523/515
Eukaryota	Fungi	Aspergillus niger CBS 513.88	XP_001395495.1	1 E-133	525/515
Eukaryota	Fungi	Penicillium chrysogenum Wisconsin 54-1255	XP_002564267.1	1 E-133	505/515
Eukaryota	Fungi	Ustilago maydis 521	XP_757571.1	1 E-125	506/515
Eukaryota	Fungi	Talaromyces stipitatus ATCC 10500	XP_002486664.1	1 E-118	519/515
Eukaryota	Fungi	Penicillium marneffei ATCC 18224	XP_002149440.1	1 E-117	517/515
Eukaryota	Fungi	Aspergillus terreus NIH2624	XP_001216534.1	1 E-110	436/515
Eukaryota	Fungi	Penicillium chrysogenum Wisconsin 54-1255	XP_002560551.1	1 E-109	492/515
Eukaryota	Fungi	Aspergillus terreus NIH2624	XP_001217085.1	1 E-109	497/515
Eukaryota	Fungi	Aspergillus oryzae RIB40	XP_001818548.1	1 E-108	493/515
Eukaryota	Fungi	Emericella nidulans	ABB20530.1	1 E-107	490/515
Eukaryota	Fungi	Uncinocarpus reesii 1704	XP_002585013.1	1 E-107	492/515
Eukaryota	Fungi	Aspergillus nidulans FGSC A4	XP_659001.1	1 E-106	482/515
Eukaryota	Fungi	Magnaporthe grisea 70-15	XP_360809.1	1 E-105	507/515
Eukaryota	Fungi	Coccidioides posadasii C735 delta	EER28194.1	1 E-104	490/515

Eukaryota	Fungi	Coccidioides immitis RS;	XP_001243308.1	1 E-104	490/515
Eukaryota	Fungi	Neosartorya fischeri NRRL 181	XP_001264888.1	1 E-103	489/515
Eukaryota	Fungi	Aspergillus clavatus NRRL 1	XP_001269380.1	1 E-103	492/515
Eukaryota	Fungi	Aspergillus fumigatus Af293	XP_752247.1	1 E-103	471/515
Eukaryota	Fungi	Microsporum canis CBS 113480	EEQ28516.1	1 E-102	493/515
Eukaryota	Fungi	Ustilago maydis 521	XP_761221.1	1 E-102	499/515
Eukaryota	Fungi	Aspergillus niger CBS 513.88	XP_001392136.1	2 E-99	486/515
Eukaryota	Fungi	Aspergillus niger CBS 513.88	XP_001396973.1	1 E-98	517/515
Eukaryota	Fungi	Penicillium chrysogenum Wisconsin 54-1255	XP_002569225.1	3 E-97	414/515
Eukaryota	Fungi	Nectria haematococca mpVI 77-13-4	EEU37587.1	4 E-97	488/515
Eukaryota	Fungi	Gibberella zeae PH-1	XP_386623.1	3 E-96	486/515
Eukaryota	Fungi	Pyrenophora tritici-repentis Pt-1C-BFP	XP_001939402.1	1 E-95	458/515
Eukaryota	Fungi	Debaryomyces hansenii CBS767	XP_458605.1	3 E-95	525/515
Eukaryota	Fungi	Debaryomyces hansenii	CAG86740.2	3 E-95	525/515
Eukaryota	Fungi	Talaromyces stipitatus ATCC 10500	XP_002341949.1	1 E-93	498/515
Eukaryota	Fungi	Nectria haematococca mpVI 77-13-4	EEU38898.1	2 E-93	493/515
Eukaryota	Fungi	Phaeosphaeria nodorum SN15	XP_001804114.1	2 E-93	489/515
Eukaryota	Fungi	Nectria haematococca mpVI 77-13-4	EEU36189.1	3 E-93	514/515
Eukaryota	Fungi	Pichia stipitis CBS 6054	XP_001386239.2	2 E-92	502/515
Eukaryota	Fungi	Verticillium albo-atrum VaMs.102	EEY23053.1	6 E-89	464/515
Eukaryota	Fungi	Pichia guilliermondii ATCC 6260	EDK37436.2	1 E-87	493/515
Eukaryota	Fungi	Pichia guilliermondii ATCC 6260	XP_001485863.1	3 E-87	493/515
Eukaryota	Fungi	Pichia guilliermondii ATCC 6260	EDK41071.2	4 E-86	491/515
Eukaryota	Fungi	Pichia pastoris GS115	XP_002493097.1	5 E-86	507/515
Eukaryota	Fungi	Pichia guilliermondii ATCC 6260	XP_001483214.1	8 E-86	491/515
Eukaryota	Fungi	Penicillium marneffei ATCC 18224	XP_002152937.1	4 E-76	482/515
Eukaryota	Fungi	Candida dubliniensis CD36	XP_002420060.1	4 E-71	574/515
Eukaryota	Fungi	Clavispora lusitaniae ATCC 42720	XP_002615031.1	3 E-70	518/515
Eukaryota	Fungi	Candida tropicalis MYA-3404	XP_002549633.1	6 E-69	472/515
Eukaryota	Fungi	Candida albicans WO-1	EEQ45062.1	7 E-69	537/515
Eukaryota	Fungi	Candida albicans SC5314	XP_711539.1	7 E-69	537/515
Eukaryota	Fungi	Penicillium marneffei ATCC 18224	XP_002144322.1	2 E-41	516/515
Eukaryota	Fungi	Aspergillus clavatus NRRL 1	XP_001268010.1	2 E-40	508/515
Eukaryota	Fungi	Gibberella zeae PH-1	XP_391565.1	5 E-38	527/515
Eukaryota	Fungi	Neosartorya fischeri NRRL 181	XP_001261696.1	2 E-36	512/515
Eukaryota	Fungi	Nectria haematococca mpVI 77-13-4	EEU38733.1	4 E-35	508/515
Eukaryota	Fungi	Postia placenta Mad-698-R	XP_002470318.1	6 E-35	471/515
Eukaryota	Fungi	Pyrenophora tritici-repentis Pt-1C-BFP	XP_001938019.1	1 E-34	524/515
Eukaryota	Fungi	Neosartorya fischeri NRRL 181	XP_001262548.1	1 E-34	527/515
Eukaryota	Fungi	Pyrenophora tritici-repentis Pt-1C-BFP	XP_001933549.1	2 E-34	502/515
Eukaryota	Fungi	Aspergillus fumigatus Af293	XP_746459.1	2 E-34	527/515
Eukaryota	Fungi	Nectria haematococca mpVI 77-13-4	EEU41997.1	3 E-34	498/515
Eukaryota	Fungi	Aspergillus fumigatus A1163	EDP47440.1	3 E-34	527/515
Eukaryota	Fungi	Postia placenta Mad-698-R	XP_002471433.1	9 E-34	511/515
Eukaryota	Metazoa	Branchiostoma floridae	XP_002611375.1	9 E-34	457/515
Eukaryota	Fungi	Postia placenta Mad-698-R	XP_002474169.1	1 E-33	470/515
Eukaryota	Fungi	Microsporum canis CBS 113480	EEQ29777.1	1 E-33	510/515
Eukaryota	Fungi	Coprinopsis cinerea okayama7#130	XP_001828262.1	2 E-33	481/515
Eukaryota	Fungi	Podospora anserina DSM 980	XP_001912243.1	2 E-33	512/515
Eukaryota	Metazoa	Pagrus major	ABX10186.1	4 E-33	471/515
Eukaryota	Metazoa	Phalacrocorax carbo	BAE93470.1	6 E-33	453/515
Eukaryota	Fungi	Talaromyces stipitatus ATCC 10500	XP_002478228.1	1 E-32	473/515
Eukaryota	Metazoa	Branchiostoma floridae	XP_002590181.1	1 E-32	484/515
Eukaryota	Fungi	Laccaria bicolor S238N-H82	XP_001879813.1	5 E-32	468/515
Eukaryota	Fungi	Podospora anserina DSM 980	XP_001904426.1	6 E-32	466/515
Eukaryota	Fungi	Nectria haematococca mpVI 77-13-4	EEU33964.1	7 E-32	489/515
Eukaryota	Metazoa	Lithognathus mormyrus	AAK69390.1	7 E-32	469/515
Eukaryota	Metazoa	Stenotomus chrysops	Q92116.1	9 E-32	469/515

Eukaryota	Fungi	Neurospora crassa OR74A	XP_964142.1	9 E-32	514/515
Eukaryota	Amoebozoa	Dictyostelium discoideum AX4	XP_640271.1	1 E-31	465/515
Eukaryota	Metazoa	Monodelphis domestica	XP_001373113.1	1 E-31	495/515
Eukaryota	Metazoa	Phalacrocorax carbo	BAE93469.1	1 E-31	472/515
Eukaryota	Fungi	Penicillium chrysogenum Wisconsin 54-1255	XP_002563123.1	1 E-31	472/515
Eukaryota	Metazoa	Liza aurata	O42231.1	2 E-31	463/515
Eukaryota	Metazoa	Chaetodon capistratus	Q92039.1	2 E-31	469/515
Eukaryota	Metazoa	Macaca fascicularis	BAA04500.1	2 E-31	460/515
Eukaryota	Metazoa	Acanthopagrus schlegelii	ABI54450.1	2 E-31	469/515
Eukaryota	Metazoa	Limanda limanda	O42430.1	2 E-31	470/515
Eukaryota	Metazoa	Macaca mulatta	NP_001035328.1	2 E-31	460/515
Eukaryota	Metazoa	Macaca fascicularis	P33616.1	2 E-31	460/515
Eukaryota	Fungi	Laccaria bicolor S238N-H82	XP_001882712.1	3 E-31	504/515
Eukaryota	Fungi	Nectria haematococca mpVI 77-13-4	EEU36875.1	3 E-31	471/515
Eukaryota	Metazoa	Mesocricetus auratus	BAA01096.1	4 E-31	480/515
Eukaryota	Metazoa	Ovis aries	P56591.1	4 E-31	470/515
Eukaryota	Metazoa	Anguilla anguilla	AAL99905.1	4 E-31	471/515
Eukaryota	Metazoa	Sus scrofa	NP_999577.1	4 E-31	457/515
Eukaryota	Metazoa	Mesocricetus auratus	Q00557.2	4 E-31	480/515
Eukaryota	Metazoa	Ciona intestinalis	ABV54639.1	4 E-31	483/515
Eukaryota	Fungi	Coccidioides posadasii C735 delta	EER29259.1	4 E-31	463/515
Eukaryota	Metazoa	Branchiostoma floridae	XP_002607749.1	5 E-31	462/515
Eukaryota	Fungi	Gibberella zeae PH-1	XP_389262.1	5 E-31	515/515
Eukaryota	Metazoa	Paralichthys olivaceus	ABO38813.1	5 E-31	469/515
Eukaryota	Amoebozoa	Dictyostelium discoideum AX4	XP_646732.1	6 E-31	456/515
Eukaryota	Metazoa	Cavia porcellus	Q06367.1	7 E-31	457/515
Eukaryota	Metazoa	Bos taurus	XP_588298.2	7 E-31	465/515
Eukaryota	Metazoa	Anguilla japonica	BAA88242.1	8 E-31	471/515
Eukaryota	Metazoa	Nematostella vectensis	XP_001639062.1	9 E-31	434/515
Eukaryota	Metazoa	Opsanus tau	Q92095.1	1 E-30	477/515
Eukaryota	Metazoa	Oryctolagus cuniculus	P00187.3	1 E-30	456/515
Eukaryota	Metazoa	Anguilla japonica	BAA88248.1	1 E-30	469/515
Eukaryota	Viridiplantae	Pisum sativum	AAP69988.1	1 E-30	468/515
Eukaryota	Metazoa	Cyprinus carpio	AAR87723.1	1 E-30	494/515
Eukaryota	Metazoa	Micropogonias undulatus	ACD44944.1	2 E-30	454/515
Eukaryota	Metazoa	Sparus aurata	O42457.1	2 E-30	469/515
Eukaryota	Metazoa	Sus scrofa	XP_001927881.1	2 E-30	457/515
Eukaryota	Fungi	Penicillium marneffei ATCC 18224	XP_002145188.1	2 E-30	470/515
Eukaryota	Fungi	Coprinopsis cinerea	BAA33717.1	2 E-30	477/515
Eukaryota	Metazoa	Monodelphis domestica	XP_001379519.1	2 E-30	453/515
Eukaryota	Fungi	Postia placenta Mad-698-R	XP_002475242.1	2 E-30	492/515
Eukaryota	Viridiplantae	Arnebia euchroma	ABD77493.2	2 E-30	444/515
Eukaryota	Metazoa	Platichthys flesus	Q9YH64.1	3 E-30	456/515
Eukaryota	Metazoa	Ciona intestinalis	XP_002131197.1	3 E-30	483/515
Eukaryota	Metazoa	Oryctolagus cuniculus	CAA29171.1	3 E-30	456/515
Eukaryota	Metazoa	Cyprinus carpio	AAR87724.1	4 E-30	470/515
Eukaryota	Metazoa	Danio rerio	XP_001919231.1	5 E-30	473/515
Eukaryota	Metazoa	Fundulus heteroclitus	AAD01809.1	5 E-30	456/515
Eukaryota	Metazoa	Xenopus laevis	BAA37079.1	5 E-30	456/515
Eukaryota	Fungi	Talaromyces stipitatus ATCC 10500	XP_002478210.1	5 E-30	456/515
Eukaryota	Metazoa	Oryctolagus cuniculus	P05176.1	5 E-30	482/515
Eukaryota	Metazoa	Homo sapiens	BAD96828.1	6 E-30	453/515
Eukaryota	Metazoa	Pleuronectes platessa	Q92100.1	6 E-30	456/515
Eukaryota	Metazoa	Takifugu obscurus	ABV24057.1	6 E-30	470/515
Eukaryota	Fungi	Phaeosphaeria nodorum SN15	XP_001805883.1	6 E-30	463/515
Eukaryota	Metazoa	Pseudopleuronectes yokohamae	BAC87834.1	6 E-30	456/515
Eukaryota	Fungi	Penicillium marneffei ATCC 18224	XP_002149505.1	6 E-30	494/515
Eukaryota	Metazoa	Liza saliens	Q9W683.1	6 E-30	463/515

Eukaryota	Fungi	Coccidioides immitis RS;	XP_001245498.1	6 E-30	455/515
Eukaryota	Fungi	Aspergillus terreus NIH2624	XP_001210010.1	7 E-30	473/515
Eukaryota	Metazoa	Homo sapiens	CAA26458.1	7 E-30	453/515
Eukaryota	Fungi	Postia placenta Mad-698-R	XP_002469779.1	7 E-30	486/515
Eukaryota	Metazoa	Pan troglodytes	XP_001137654.1	8 E-30	453/515
Eukaryota	Metazoa	Lagenorhynchus acutus	AAV34440.1	8 E-30	457/515
Eukaryota	Metazoa	Rattus norvegicus	NP_036672.2	8 E-30	460/515
Eukaryota	Fungi	Penicillium marneffei ATCC 18224	XP_002145973.1	8 E-30	447/515
Eukaryota	Metazoa	Mus musculus	AAA37506.1	8 E-30	455/515
Eukaryota	Metazoa	Balaenoptera acutorostrata	Q3LFU0.1	9 E-30	457/515
Eukaryota	Metazoa	Rattus norvegicus	P00185.1	9 E-30	460/515
Eukaryota	Metazoa	Homo sapiens	BAD96833.1	9 E-30	453/515
Eukaryota	Fungi	Coprinopsis cinerea okayama7#130	XP_001831767.1	9 E-30	475/515
Eukaryota	Metazoa	Homo sapiens	NP_000490.1	9 E-30	453/515
Eukaryota	Fungi	Aspergillus fumigatus Af293	XP_747129.1	9 E-30	472/515
Eukaryota	Metazoa	Rattus norvegicus	AAA41025.1	9 E-30	460/515
Eukaryota	Metazoa	Mus musculus	NP_034122.1	1 E-29	455/515
Eukaryota	Metazoa	Anguilla anguilla	AAL99904.1	1 E-29	469/515
Eukaryota	Metazoa	Anguilla japonica	BAA88241.1	1 E-29	469/515
Eukaryota	Metazoa	Danio rerio	NP_571954.1	1 E-29	456/515
Eukaryota	Metazoa	Danio rerio	AAQ97766.1	1 E-29	456/515
Eukaryota	Metazoa	Tetraodon nigroviridis	CAG03127.1	2 E-29	456/515
Eukaryota	Fungi	Coprinopsis cinerea okayama7#130	XP_001839948.1	2 E-29	463/515
Eukaryota	Metazoa	Xenopus laevis	NP_001090541.1	2 E-29	453/515
Eukaryota	Fungi	Chaetomium globosum CBS 148.51	XP_001221290.1	2 E-29	459/515
Eukaryota	Metazoa	Equus caballus	XP_001493959.1	2 E-29	457/515
Eukaryota	Metazoa	Homo sapiens	AAA52139.1	2 E-29	453/515
Eukaryota	Metazoa	Chelon labrosus	ABD95933.1	3 E-29	456/515
Eukaryota	Metazoa	Oryctolagus cuniculus	prf1307202A	3 E-29	482/515
Eukaryota	Fungi	Aspergillus clavatus NRRL 1	XP_001270497.1	3 E-29	467/515
Eukaryota	Fungi	Nectria haematococca mpVI 77-13-4	EEU42001.1	4 E-29	441/515
Eukaryota	Metazoa	Branchiostoma floridae	XP_002601369.1	4 E-29	476/515
Eukaryota	Metazoa	Phoca fasciata	BAB20377.2	4 E-29	457/515
Eukaryota	Viridiplantae	Oryza sativa Japonica Group	AAK63940.1	5 E-29	491/515
Eukaryota	Metazoa	Phoca sibirica	BAF58167.1	5 E-29	457/515
Eukaryota	Metazoa	Salmo salar	ACI33928.1	5 E-29	456/515
Eukaryota	Metazoa	Xenopus (Silurana) tropicalis	NP_001090813.1	5 E-29	452/515
Eukaryota	Viridiplantae	Oryza sativa Japonica Group	EEE59969.1	6 E-29	491/515
Eukaryota	Metazoa	Danio rerio	AAH94977.1	6 E-29	455/515
Eukaryota	Metazoa	Equus caballus	ABW86891.1	8 E-29	457/515
Eukaryota	Fungi	Coprinopsis cinerea okayama7#130	XP_001834734.1	8 E-29	440/515
Eukaryota	Fungi	Aspergillus nidulans FGSC A4	CBF80376.1	8 E-29	478/515
Eukaryota	Viridiplantae	Arabidopsis thaliana	NP_189261.1	9 E-29	455/515
Eukaryota	Viridiplantae	Arabidopsis thaliana	NP_189262.1	1 E-28	460/515
Eukaryota	Metazoa	Tetraodon nigroviridis	CAG10688.1	1 E-28	463/515
Eukaryota	Metazoa	Dicentrarchus labrax	CAB63650.1	1 E-28	456/515
Eukaryota	Viridiplantae	Oryza sativa Japonica Group	EEE65905.1	1 E-28	435/515
Eukaryota	Viridiplantae	Oryza sativa Japonica Group	NP_001057909.1	2 E-28	429/515
Eukaryota	Fungi	Aspergillus niger CBS 513.88	XP_001394343.1	2 E-28	468/515
Eukaryota	Viridiplantae	Stevia rebaudiana	AAQ63464.1	2 E-28	492/515
Eukaryota	Metazoa	Halichoerus grypus	CAF18539.1	2 E-28	457/515
Eukaryota	Viridiplantae	Vitis vinifera	XP_002282014.1	2 E-28	503/515
Eukaryota	Viridiplantae	Cucurbita maxima	AAG41776.1	2 E-28	432/515
Eukaryota	Viridiplantae	Oryza sativa Japonica Group	AAT81230.1	2 E-28	429/515
Eukaryota	Fungi	Coprinopsis cinerea okayama7#130	XP_001834855.1	2 E-28	512/515
Eukaryota	Viridiplantae	Capsicum annuum	ACF19421.1	2 E-28	444/515
Eukaryota	Fungi	Nectria haematococca mpVI 77-13-4	EEU41755.1	2 E-28	468/515
Eukaryota	Metazoa	Salvelinus namaycush	AAQ10900.1	3 E-28	456/515

Eukaryota	Metazoa	Fundulus heteroclitus	ACO51072.1	3 E-28	457/515
Eukaryota	Fungi	Uncinocarpus reesii 1704	XP_002583646.1	3 E-28	441/515
Eukaryota	Viridiplantae	Stevia rebaudiana	AAY42951.1	3 E-28	492/515
Eukaryota	Metazoa	Oncorhynchus mykiss	NP_001118226.1	3 E-28	456/515
Eukaryota	Metazoa	Oncorhynchus mykiss	AAD14035.1	3 E-28	456/515
Eukaryota	Metazoa	Xenopus (Silurana) tropicalis	NP_001120285.1	3 E-28	474/515
Eukaryota	Viridiplantae	Helianthus tuberosus	CAA04117.1	3 E-28	428/515
Eukaryota	Metazoa	Phoca groenlandica	CAF18541.1	3 E-28	457/515
Eukaryota	Metazoa	Corvus macrorhynchos	BAE75841.1	3 E-28	459/515
Eukaryota	Metazoa	Equus caballus	XP_001493936.1	4 E-28	456/515
Eukaryota	Fungi	Coprinopsis cinerea okayama7#130	XP_001835122.1	4 E-28	483/515
Eukaryota	Viridiplantae	Zea mays;	NP_001145908.1	4 E-28	475/515
Eukaryota	Metazoa	Salvelinus fontinalis	AAQ10899.1	5 E-28	456/515
Eukaryota	Viridiplantae	Lithospermum erythrorhizon	BAC44836.1	5 E-28	487/515
Eukaryota	Fungi	Aspergillus niger CBS 513.88	XP_001402405.1	5 E-28	488/515
Eukaryota	Metazoa	Rattus norvegicus	CAA35039.1	6 E-28	465/515
Eukaryota	Metazoa	Papio ursinus	ABY57765.1	6 E-28	461/515

AFUA_5G01730

Eukaryota	Fungi	Aspergillus fumigatus Af293	XP_748169.1	0.0	2031/2031
Eukaryota	Fungi	Neosartorya fischeri NRRL 181	XP_001266358.1	0.0	2028/2031
Eukaryota	Fungi	Aspergillus oryzae RIB40	XP_001820413.1	0.0	1748/2031
Eukaryota	Fungi	Aspergillus niger CBS 513.88	XP_001397460.1	0.0	1812/2031
Eukaryota	Fungi	Aspergillus nidulans FGSC A4	XP_681345.1	0.0	1753/2031
Eukaryota	Fungi	Coccidioides posadasii C735 delta	EER27556.1	0.0	1738/2031
Eukaryota	Fungi	Coccidioides immitis RS;	XP_001242490.1	0.0	1737/2031
Eukaryota	Fungi	Ajellomyces capsulatus G186AR	EEH05531.1	0.0	1641/2031

AFUA_5G03960

Eukaryota	Fungi	Aspergillus fumigatus Af293	XP_747948.2	0.0	1424/1424
Eukaryota	Fungi	Neosartorya fischeri NRRL 181	XP_001266139.1	0.0	1432/1424
Eukaryota	Fungi	Microsporum canis CBS 113480	EEQ32193.1	0.0	1347/1424
Eukaryota	Fungi	Hypocrea jecorina	DAA05849.1	0.0	1324/1424

AFUA_5G04440

Eukaryota	Fungi	Aspergillus fumigatus Af293	XP_747899.1	0.0	373/373
Eukaryota	Fungi	Neosartorya fischeri NRRL 181	XP_001266085.1	0.0	372/373
Eukaryota	Fungi	Penicillium marneffei ATCC 18224	XP_002149133.1	3 E-77	329/373
Eukaryota	Fungi	Coccidioides posadasii C735 delta	EER27516.1	1 E-71	315/373
Eukaryota	Fungi	Coccidioides immitis RS;	XP_001242444.1	2 E-71	315/373
Eukaryota	Fungi	Microsporum canis CBS 113480	EEQ29101.1	2 E-68	343/373
Eukaryota	Fungi	Uncinocarpus reesii 1704	XP_002544543.1	3 E-68	303/373
Eukaryota	Fungi	Ajellomyces dermatitidis SLH14081	XP_002621616.1	4 E-67	311/373
Eukaryota	Fungi	Ajellomyces capsulatus H143	EER40533.1	3 E-66	300/373
Eukaryota	Fungi	Ajellomyces capsulatus NAm1	XP_001539311.1	4 E-66	318/373
Eukaryota	Fungi	Ajellomyces capsulatus G186AR	EEH02970.1	9 E-66	300/373

AFUA_5G06800

Eukaryota	Fungi	Aspergillus fumigatus Af293	XP_753953.1	0.0	606/606
Eukaryota	Fungi	Neosartorya fischeri NRRL 181	XP_001265957.1	0.0	594/606
Eukaryota	Fungi	Aspergillus clavatus NRRL 1	XP_001274017.1	0.0	613/606
Eukaryota	Fungi	Aspergillus terreus NIH2624	XP_001217968.1	0.0	579/606
Eukaryota	Fungi	Aspergillus flavus NRRL3357	XP_002378422.1	0.0	569/606
Eukaryota	Fungi	Aspergillus oryzae RIB40	XP_001823087.1	0.0	569/606

Eukaryota	Fungi	Aspergillus niger CBS 513.88	XP_001398308.1	0.0	578/606
Eukaryota	Fungi	Penicillium chrysogenum Wisconsin 54-1255	XP_002568794.1	0.0	631/606
Eukaryota	Fungi	Aspergillus nidulans FGSC A4	CBF86446.1	0.0	581/606
Eukaryota	Fungi	Aspergillus nidulans FGSC A4	XP_659845.1	1 E-170	505/606
Eukaryota	Fungi	Penicillium marneffei ATCC 18224	XP_002150673.1	1 E-143	571/606
Eukaryota	Fungi	Talaromyces stipitatus ATCC 10500	XP_002483468.1	1 E-142	587/606
Eukaryota	Fungi	Sclerotinia sclerotiorum 1980 UF-70	XP_001588429.1	1 E-135	537/606
Eukaryota	Fungi	Neosartorya fischeri NRRL 181	XP_001261746.1	1 E-130	549/606
Eukaryota	Fungi	Aspergillus fumigatus A1163	EDP49088.1	1 E-126	549/606
Eukaryota	Fungi	Aspergillus fumigatus Af293	XP_747082.1	1 E-125	549/606
Eukaryota	Fungi	Nectria haematococca mpVI 77-13-4	EEU48207.1	1 E-123	529/606
Eukaryota	Fungi	Gibberella zeae PH-1	XP_389509.1	1 E-122	539/606
Eukaryota	Fungi	Aspergillus terreus NIH2624	XP_001211571.1	1 E-121	531/606
Eukaryota	Fungi	Aspergillus flavus NRRL3357	XP_002381694.1	1 E-118	526/606
Eukaryota	Fungi	Aspergillus oryzae RIB40	XP_001825022.1	1 E-118	526/606
Eukaryota	Fungi	Penicillium chrysogenum Wisconsin 54-1255	XP_002562072.1	1 E-117	536/606
Eukaryota	Fungi	Aspergillus nidulans FGSC A4	XP_661722.1	1 E-113	551/606
Eukaryota	Fungi	Aspergillus niger CBS 513.88	XP_001390754.1	1 E-108	528/606
Eukaryota	Fungi	Aspergillus oryzae RIB40	XP_001824319.1	7 E-92	530/606
Eukaryota	Fungi	Aspergillus flavus NRRL3357	XP_002381424.1	5 E-91	530/606
Eukaryota	Fungi	Aspergillus flavus NRRL3357	XP_002378655.1	5 E-85	491/606
Eukaryota	Fungi	Aspergillus oryzae RIB40	XP_001823292.1	4 E-84	493/606
Eukaryota	Fungi	Talaromyces stipitatus ATCC 10500	XP_002486360.1	9 E-75	514/606
Eukaryota	Fungi	Sclerotinia sclerotiorum 1980 UF-70	XP_001586920.1	6 E-70	514/606
Eukaryota	Fungi	Aspergillus fumigatus A1163	EDP48624.1	3 E-59	511/606
Eukaryota	Fungi	Aspergillus fumigatus Af293	XP_747516.2	9 E-59	511/606
Eukaryota	Fungi	Lachancea thermotolerans CBS 6340	XP_002552676.1	2 E-58	522/606
Eukaryota	Fungi	Aspergillus clavatus NRRL 1	XP_001276777.1	2 E-55	513/606
Eukaryota	Fungi	Sclerotinia sclerotiorum 1980 UF-70	XP_001596839.1	5 E-54	517/606
Eukaryota	Fungi	Sclerotinia sclerotiorum 1980 UF-70	XP_001596565.1	6 E-54	552/606
Eukaryota	Fungi	Saccharomyces cerevisiae YJM789	EDN63697.1	4 E-52	526/606
Eukaryota	Fungi	Saccharomyces cerevisiae	NP_015025.1	8 E-52	526/606
Eukaryota	Fungi	Penicillium marneffei ATCC 18224	XP_002146900.1	4 E-47	565/606
Eukaryota	Fungi	Talaromyces stipitatus ATCC 10500	XP_002479194.1	1 E-45	585/606
Eukaryota	Fungi	Nectria haematococca mpVI 77-13-4	EEU43662.1	6 E-40	512/606
Eukaryota	Fungi	Penicillium chrysogenum Wisconsin 54-1255	XP_002558456.1	2 E-36	495/606
Eukaryota	Fungi	Aspergillus niger CBS 513.88	XP_001395767.1	7 E-36	536/606
Eukaryota	Fungi	Penicillium marneffei ATCC 18224	XP_002144986.1	6 E-33	505/606
Eukaryota	Fungi	Aspergillus oryzae RIB40	XP_001827262.1	1 E-32	547/606
Eukaryota	Fungi	Aspergillus clavatus NRRL 1	XP_001272851.1	2 E-31	554/606
Eukaryota	Fungi	Neosartorya fischeri NRRL 181	XP_001262280.1	3 E-31	528/606

AFUA_5G06840

Eukaryota	Fungi	Aspergillus fumigatus Af293	XP_753949.1	0.0	1153/1153
Eukaryota	Fungi	Aspergillus terreus NIH2624	XP_001209460.1	0.0	1201/1153
Eukaryota	Fungi	Penicillium marneffei ATCC 18224	XP_002143918.1	0.0	1196/1153
Eukaryota	Fungi	Uncinocarpus reesii 1704	XP_002544597.1	0.0	1204/1153
Eukaryota	Fungi	Hypocrea jecorina	DAA05856.1	0.0	1211/1153
Eukaryota	Fungi	Aspergillus terreus NIH2624	XP_001215392.1	0.0	1184/1153
Eukaryota	Fungi	Hypocrea jecorina	DAA05858.1	0.0	1171/1153
Eukaryota	Fungi	Uncinocarpus reesii 1704	XP_002583760.1	0.0	1134/1153
Eukaryota	Fungi	Aspergillus terreus NIH2624	XP_001214802.1	1 E-176	963/1153
Eukaryota	Fungi	Penicillium marneffei ATCC 18224	XP_002146945.1	1 E-174	941/1153

AFUA_5G06850

Eukaryota	Fungi	Aspergillus fumigatus Af293	XP_753948.1	0.0	626/626

Eukaryota	Fungi	Aspergillus terreus NIH2624	XP_001209461.1	0.0	620/626
Eukaryota	Fungi	Penicillium marneffei ATCC 18224	XP_002143919.1	0.0	643/626
Eukaryota	Fungi	Uncinocarpus reesii 1704	XP_002544596.1	1 E-159	518/626

AFUA_5G06860

Eukaryota	Fungi	Aspergillus fumigatus Af293	XP_753947.1	0.0	362/362

AFUA_5G07510

Eukaryota	Fungi	Aspergillus fumigatus Af293	XP_753883.1	0.0	866/866
Eukaryota	Fungi	Neosartorya fischeri NRRL 181	XP_001259931.1	0.0	878/866
Eukaryota	Fungi	Aspergillus clavatus NRRL 1	XP_001274077.1	0.0	878/866
Eukaryota	Fungi	Penicillium chrysogenum Wisconsin 54-1255	XP_002569247.1	0.0	803/866
Eukaryota	Fungi	Aspergillus oryzae RIB40	XP_001727118.1	1 E-176	811/866
Eukaryota	Fungi	Penicillium marneffei ATCC 18224	XP_002152352.1	1 E-174	745/866
Eukaryota	Fungi	Emericella nidulans	P21228.2ALCR_E	1 E-174	820/866
Eukaryota	Fungi	Aspergillus nidulans FGSC A4	XP_682247.1	1 E-163	801/866
Eukaryota	Fungi	Gibberella zeae PH-1	XP_382923.1	9 E-96	898/866
Eukaryota	Fungi	Aspergillus terreus NIH2624	XP_001217059.1	6 E-92	819/866
Eukaryota	Fungi	Aspergillus terreus NIH2624	XP_001212805.1	4 E-78	797/866
Eukaryota	Fungi	Ustilago maydis 521	XP_759767.1	4 E-52	817/866

AFUA_5G07570

Eukaryota	Fungi	Aspergillus fumigatus Af293	XP_753877.1	0.0	646/646
Eukaryota	Fungi	Neosartorya fischeri NRRL 181	XP_001259925.1	0.0	644/646
Eukaryota	Fungi	Penicillium chrysogenum Wisconsin 54-1255	XP_002565436.1	0.0	660/646
Eukaryota	Fungi	Aspergillus terreus NIH2624	XP_001216988.1	0.0	638/646
Eukaryota	Fungi	Aspergillus flavus NRRL3357	XP_002378876.1	0.0	634/646
Eukaryota	Fungi	Aspergillus niger CBS 513.88	XP_001396820.1	0.0	636/646
Eukaryota	Fungi	Coccidioides immitis RS;	XP_001245609.1	0.0	664/646
Eukaryota	Fungi	Aspergillus nidulans FGSC A4	CBF80060.1	0.0	634/646
Eukaryota	Fungi	Uncinocarpus reesii 1704	XP_002583543.1	0.0	657/646
Eukaryota	Fungi	Aspergillus nidulans FGSC A4	XP_681032.1	0.0	645/646
Eukaryota	Fungi	Ajellomyces dermatitidis ER-3	EEQ92456.1	0.0	663/646
Eukaryota	Fungi	Ajellomyces dermatitidis SLH14081	XP_002626099.1	0.0	663/646
Eukaryota	Fungi	Paracoccidioides brasiliensis Pb03;	EEH23118.1	0.0	658/646
Eukaryota	Fungi	Paracoccidioides brasiliensis Pb01;	EEH36280.1	0.0	659/646
Eukaryota	Fungi	Ajellomyces capsulatus NAm1	XP_001538374.1	0.0	653/646
Eukaryota	Fungi	Coccidioides posadasii C735 delta	EER29163.1	0.0	606/646
Eukaryota	Fungi	Paracoccidioides brasiliensis Pb18;	EEH42103.1	1 E-180	608/646
Eukaryota	Fungi	Ajellomyces capsulatus H143	EER41789.1	1 E-176	612/646
Eukaryota	Fungi	Botryotinia fuckeliana B05.10	XP_001558985.1	1 E-163	628/646
Eukaryota	Fungi	Ajellomyces capsulatus G186AR	EEH05888.1	1 E-141	578/646
Eukaryota	Fungi	Coprinopsis cinerea okayama7#130	XP_001838959.1	1 E-112	633/646
Bacteria	Actinobacteria	Bifidobacterium angulatum DSM 20098	ZP_04448559.1	2 E-87	606/646
Bacteria	Chloroflexi	Herpetosiphon aurantiacus ATCC 23779	YP_001546967.1	2 E-85	564/646
Bacteria	Actinobacteria	Mycobacterium smegmatis str. MC2	YP_884747.1	3 E-85	636/646
Bacteria	Actinobacteria	Mycobacterium vanbaalenii PYR-1	YP_951199.1	7 E-85	635/646
Eukaryota	Metazoa	Nematostella vectensis	XP_001636842.1	4 E-83	649/646
Bacteria	Actinobacteria	Bifidobacterium breve DSM 20213	ZP_03618010.1	5 E-83	613/646

AFUA_5G07580

Eukaryota	Fungi	Aspergillus fumigatus Af293	XP_753876.1	0.0	559/559
Eukaryota	Fungi	Neosartorya fischeri NRRL 181	XP_001259924.1	0.0	559/559
Eukaryota	Fungi	Aspergillus niger CBS 513.88	XP_001396821.1	0.0	549/559

Eukaryota	Fungi	Paracoccidioides brasiliensis Pb01;	EEH36279.1	0.0	571/559
Eukaryota	Fungi	Aspergillus flavus NRRL3357	XP_002378875.1	0.0	555/559
Eukaryota	Fungi	Paracoccidioides brasiliensis Pb18;	EEH42102.1	0.0	571/559
Eukaryota	Fungi	Paracoccidioides brasiliensis Pb03;	EEH23119.1	0.0	571/559
Eukaryota	Fungi	Penicillium chrysogenum Wisconsin 54-1255	XP_002565435.1	0.0	569/559
Eukaryota	Fungi	Ajellomyces dermatitidis ER-3	EEQ92455.1	0.0	563/559
Eukaryota	Fungi	Ajellomyces dermatitidis SLH14081	XP_002626098.1	0.0	563/559
Eukaryota	Fungi	Coccidioides posadasii C735 delta	EER29162.1	0.0	560/559
Eukaryota	Fungi	Pyrenophora tritici-repentis Pt-1C-BFP	XP_001936879.1	0.0	568/559
Eukaryota	Fungi	Ajellomyces capsulatus G186AR	EEH05889.1	0.0	521/559
Eukaryota	Fungi	Botryotinia fuckeliana B05.10	XP_001558986.1	0.0	566/559
Eukaryota	Fungi	Sclerotinia sclerotiorum 1980 UF-70	XP_001597586.1	0.0	568/559
Eukaryota	Fungi	Coprinopsis cinerea okayama7#130	XP_001838958.1	1 E-126	510/559
Bacteria	Proteobacteria	Novosphingobium aromaticivorans DSM 12444	YP_497863.1	1 E-102	468/559
Bacteria	Proteobacteria	Diaphorobacter sp. TPSY	YP_002554846.1	1 E-101	468/559
Bacteria	Proteobacteria	Rhodospirillum centenum SW	YP_002296488.1	3 E-99	468/559
Bacteria	Proteobacteria	Erythrobacter sp. SD-21	ZP_01864054.1	3 E-98	476/559
Bacteria	Proteobacteria	Erythrobacter sp. NAP1	ZP_01040330.1	3 E-97	489/559
Bacteria	Proteobacteria	Erythrobacter litoralis HTCC2594	YP_458870.1	9 E-96	476/559
Bacteria	Proteobacteria	Ralstonia eutropha JMP134	YP_298092.1	1 E-95	468/559
Bacteria	Proteobacteria	Delftia acidovorans SPH-1	YP_001562129.1	2 E-95	471/559
Bacteria	Proteobacteria	Sphingopyxis alaskensis RB2256	YP_616899.1	3 E-95	478/559
Bacteria	Actinobacteria	Streptomyces sp. AA4	ZP_05481659.1	5 E-95	477/559
Bacteria	Proteobacteria	Verminephrobacter eiseniae EF01-2	YP_995629.1	2 E-93	469/559
Bacteria	Proteobacteria	Hyphomonas neptunium ATCC 15444	YP_758830.1	2 E-92	478/559
Bacteria	Proteobacteria	Rhodopseudomonas palustris BisB18	YP_532958.1	2 E-90	474/559
Bacteria	Proteobacteria	Rhodopseudomonas palustris BisA53	YP_781299.1	3 E-90	474/559
Bacteria	Proteobacteria	Cupriavidus taiwanensis	YP_002008524.1	1 E-89	468/559
Bacteria	Proteobacteria	Oceanicaulis alexandrii HTCC2633	ZP_00952171.1	2 E-89	468/559
Bacteria	Proteobacteria	Rhodopseudomonas palustris TIE-1	YP_001992916.1	4 E-86	474/559
Bacteria	Proteobacteria	Rhodopseudomonas palustris CGA009	NP_948794.1	6 E-86	474/559
Bacteria	Proteobacteria	marine gamma proteobacterium HTCC2080	ZP_01625932.1	7 E-86	478/559
Bacteria	Proteobacteria	Bradyrhizobium japonicum USDA 110	NP_770580.1	2 E-81	474/559
Bacteria	Proteobacteria	Acidovorax delafieldii 2AN	ZP_04762275.1	3 E-81	483/559
Bacteria	Proteobacteria	Phenylobacterium zucineum HLK1	YP_002131398.1	5 E-81	471/559
Bacteria	Proteobacteria	marine gamma proteobacterium HTCC2143	ZP_01618328.1	1 E-80	473/559
Bacteria	Proteobacteria	Ruegeria pomeroyi DSS-3	YP_167756.1	2 E-80	478/559
Bacteria	Proteobacteria	Bradyrhizobium sp. ORS278	YP_001205165.1	4 E-79	474/559
Bacteria	Proteobacteria	Bradyrhizobium sp. BTAi1	YP_001239569.1	4 E-78	474/559
Bacteria	Proteobacteria	Parvibaculum lavamentivorans DS-1	YP_001413250.1	4 E-76	471/559
Bacteria	Chloroflexi	Chloroflexus aurantiacus J-10-fl	YP_001635958.1	3 E-65	463/559
Bacteria	Thermobaculum	Thermobaculum terrenum ATCC BAA-798	ZP_03857787.1	4 E-63	490/559
Bacteria	Chloroflexi	Roseiflexus castenholzii DSM 13941	YP_001434152.1	4 E-62	470/559
Archaea	Crenarchaeota	Hyperthermus butylicus DSM 5456	YP_001013337.1	2 E-61	461/559
Archaea	Crenarchaeota	Staphylothermus marinus F1	YP_001041424.1	2 E-61	461/559
Bacteria	Actinobacteria	Acidimicrobium ferrooxidans DSM 10331	YP_003108873.1	3 E-61	473/559
Bacteria	Chloroflexi	Chloroflexus aurantiacus J-10-fl	YP_001637009.1	4 E-61	480/559
Bacteria	Chloroflexi	Chloroflexus aggregans DSM 9485	YP_002461761.1	6 E-61	460/559
Archaea	Euryarchaeota	Natronomonas pharaonis DSM 2160	YP_330911.1	7 E-61	462/559
Bacteria	Proteobacteria	Haliangium ochraceum DSM 14365	ZP_03876638.1	1 E-60	465/559
Bacteria	Actinobacteria	Rubrobacter xylanophilus DSM 9941	YP_645205.1	1 E-60	483/559
Archaea	Euryarchaeota	Aciduliprofundum boonei T469	ZP_04874632.1	1 E-60	462/559
Archaea	Euryarchaeota	Aciduliprofundum boonei T469	ZP_04873860.1	4 E-60	462/559
Bacteria	Proteobacteria	Thauera sp. MZ1T	YP_002890455.1	1 E-59	486/559
Bacteria	Proteobacteria	Rhodopseudomonas palustris CGA009	NP_948542.1	2 E-59	458/559
Bacteria	Bacteroidetes	Flavobacteriales bacterium ALC-1	ZP_02183137.1	2 E-59	462/559
Bacteria	Actinobacteria	Bifidobacterium dentium ATCC 27678	ZP_02917152.1	3 E-59	470/559
Bacteria	Actinobacteria	Bifidobacterium animalis subsp. lactis	ZP_02963251.1	4 E-59	472/559

Bacteria	Chloroflexi	Roseiflexus sp. RS-1	YP_001277602.1	4 E-59	463/559
Archaea	Euryarchaeota	Halomicrobium mukohataei DSM 12286	YP_003178386.1	5 E-59	479/559
Bacteria	Actinobacteria	Streptomyces ghanaensis ATCC 14672	ZP_04685696.1	6 E-59	499/559
Bacteria	Actinobacteria	Streptomyces scabiei 87.22	CBG69782.1	8 E-59	495/559
Bacteria	Actinobacteria	Streptomyces griseoflavus Tu4000	ZP_05538537.1	8 E-59	495/559
Bacteria	Actinobacteria	Streptomyces hygroscopicus ATCC 53653	ZP_05514554.1	9 E-59	495/559
Bacteria	Actinobacteria	Bifidobacterium adolescentis L2-32	ZP_02028076.1	9 E-59	495/559
Bacteria	Acidobacteria	Candidatus Solibacter usitatus Ellin6076	YP_822769.1	1 E-58	467/559
Bacteria	Proteobacteria	Azoarcus sp. BH72	YP_932191.1	1 E-58	462/559
Bacteria	Actinobacteria	Streptomyces coelicolor A3(2)	NP_629669.1	1 E-58	495/559
Bacteria	Actinobacteria	Streptomyces ghanaensis ATCC 14672	ZP_04691260.1	1 E-58	475/559
Bacteria	Chloroflexi	Herpetosiphon aurantiacus ATCC 23779	YP_001543720.1	1 E-58	475/559
Bacteria	Actinobacteria	Streptomyces lividans TK24	ZP_05523443.1	1 E-58	476/559
Bacteria	Actinobacteria	Bifidobacterium adolescentis ATCC 15703	YP_909118.1	2 E-58	495/559
Bacteria	Proteobacteria	Magnetospirillum gryphiswaldense MSR-1	CAM76410.1	2 E-58	463/559
Bacteria	Proteobacteria	Rhodopseudomonas palustris BisB18	YP_533256.1	2 E-58	482/559
Archaea	Crenarchaeota	Sulfolobus tokodaii str. 7	NP_376479.1	2 E-58	500/559
Bacteria	Proteobacteria	Magnetospirillum magneticum AMB-1	YP_423606.1	2 E-58	465/559
Bacteria	Proteobacteria	Rhodopseudomonas palustris BisA53	YP_782451.1	2 E-58	458/559
Bacteria	Proteobacteria	Dechloromonas aromatica RCB	YP_287201.1	3 E-58	483/559
Bacteria	Proteobacteria	Anaeromyxobacter sp. Fw109-5	YP_001377473.1	4 E-58	463/559
Bacteria	Proteobacteria	Magnetospirillum magnetotacticum MS-1	ZP_00053942.1	4 E-58	465/559
Archaea	Crenarchaeota	Sulfolobus solfataricus 98/2	ACX90564.1	6 E-58	494/559
Bacteria	Proteobacteria	Stigmatella aurantiaca DW4/3-1	ZP_01461629.1	6 E-58	463/559
Archaea	Euryarchaeota	Haloarcula marismortui ATCC 43049	YP_135585.1	8 E-58	462/559
Archaea	Euryarchaeota	Halogeometricum borinquense DSM 11551	ZP_03999578.1	8 E-58	461/559
Bacteria	Actinobacteria	Streptomyces griseus subsp. griseus	YP_001823456.1	8 E-58	492/559
Bacteria	Proteobacteria	Rhodopseudomonas palustris HaA2	YP_485958.1	1 E-57	458/559
Archaea	Euryarchaeota	Halomicrobium mukohataei DSM 12286	YP_003178100.1	1 E-57	462/559
Bacteria	Synergistetes	Jonquetella anthropi E3_33 E1	ZP_05860432.1	1 E-57	467/559
Bacteria	Actinobacteria	Streptomyces antibioticus	CAG14973.1	1 E-57	485/559
Bacteria	Proteobacteria	Comamonas testosteroni KF-1	ZP_03543062.1	1 E-57	486/559
Bacteria	Proteobacteria	Rhodopseudomonas palustris BisB5	YP_570249.1	2 E-57	458/559
Bacteria	Proteobacteria	Methylobacterium sp. 4-46	YP_001770622.1	2 E-57	460/559
Bacteria	Actinobacteria	Kocuria rhizophila DC2201	YP_001855537.1	2 E-57	476/559
Bacteria	Actinobacteria	Bifidobacterium pseudocatenulatum DSM 2043	ZP_03742149.1	3 E-57	509/559
Archaea	Crenarchaeota	Desulfurococcus kamchatkensis 1221n	YP_002428353.1	4 E-57	486/559
Bacteria	Proteobacteria	Nitrobacter sp. Nb-311A	ZP_01046080.1	4 E-57	460/559
Bacteria	Bacteroidetes	Porphyromonas gingivalis W83	NP_905741.1	4 E-57	461/559
Bacteria	Proteobacteria	Methylobacterium sp. 4-46	YP_001772386.1	4 E-57	463/559
Bacteria	Bacteroidetes	Bacteroides coprophilus DSM 18228	ZP_03644584.1	4 E-57	456/559
Bacteria	Actinobacteria	Thermobifida fusca YX	YP_289276.1	4 E-57	486/559
Bacteria	Firmicutes	Heliobacterium modesticaldum Ice1	YP_001679008.1	5 E-57	458/559
Bacteria	Actinobacteria	Streptomyces cinnamonensis	CAL34080.1	5 E-57	485/559
Archaea	Crenarchaeota	Sulfolobus islandicus Y.N.15.51	YP_002841684.1	5 E-57	466/559
Bacteria	Proteobacteria	Delftia acidovorans SPH-1	YP_001564772.1	5 E-57	486/559
Bacteria	Bacteroidetes	Bacteroides coprocola DSM 17136	ZP_03011881.1	5 E-57	456/559
Archaea	Crenarchaeota	Sulfolobus islandicus M.14.25	YP_002828440.1	6 E-57	466/559
Bacteria	Actinobacteria	Streptomyces flavogriseus ATCC 33331	ZP_05806824.1	6 E-57	502/559
Bacteria	Proteobacteria	Hyphomicrobium denitrificans ATCC 51888	ZP_05376987.1	6 E-57	461/559
Bacteria	Bacteroidetes	Bacteroides finegoldii DSM 17565	ZP_05414863.1	6 E-57	457/559
Bacteria	Actinobacteria	Streptomyces murayamaensis	AAO65356.1	7 E-57	498/559
Bacteria	Synergistetes	Thermanaerovibrio acidaminovorans DSM 658	ZP_04468044.1	7 E-57	477/559
Bacteria	Synergistetes	Anaerobaculum hydrogeniformans ATCC BAA	ZP_05800404.1	7 E-57	484/559
Bacteria	Actinobacteria	Streptomyces avermitilis MA-4680	NP_823879.1	7 E-57	491/559
Bacteria	Actinobacteria	Bifidobacterium catenulatum DSM 16992	ZP_03324817.1	7 E-57	463/559
Bacteria	Actinobacteria	Streptomyces roseosporus NRRL 15998	ZP_04696495.1	8 E-57	502/559
Bacteria	Actinobacteria	Streptomyces ambofaciens	AAR30168.1	8 E-57	475/559

Bacteria	Actinobacteria	Bifidobacterium angulatum DSM 20098	ZP_04448560.1	1 E-56	491/559
Bacteria	Bacteroidetes	Bacteroides plebeius DSM 17135	ZP_03208256.1	1 E-56	456/559
Bacteria	Actinobacteria	Streptomyces sviceus ATCC 29083	ZP_05015363.1	1 E-56	491/559
Bacteria	Actinobacteria	Streptomyces ambofaciens	CAK51046.1	1 E-56	475/559
Bacteria	Firmicutes	Acidaminococcus sp. D21	ZP_03989726.1	1 E-56	463/559
Bacteria	Proteobacteria	Methylobacterium nodulans ORS 2060	YP_002500657.1	1 E-56	460/559
Bacteria	Proteobacteria	Nitrobacter hamburgensis X14	YP_576860.1	1 E-56	482/559
Bacteria	Actinobacteria	Tropheryma whipplei str. Twist	NP_787151.1	1 E-56	473/559
Bacteria	Proteobacteria	Methylobacterium populi BJ001	YP_001923071.1	1 E-56	483/559
Eukaryota	Metazoa	Xenopus laevis	NP_001083656.1	2 E-56	459/559
Bacteria	Proteobacteria	Methylobacterium radiotolerans JCM 2831	YP_001755502.1	2 E-56	482/559
Bacteria	Proteobacteria	Bradyrhizobium sp. BTAi1	YP_001240933.1	2 E-56	482/559
Bacteria	Chloroflexi	Sphaerobacter thermophilus DSM 20745	ZP_04496473.1	2 E-56	462/559
Bacteria	Actinobacteria	Rothia mucilaginosa ATCC 25296	ZP_05368342.1	2 E-56	480/559
Bacteria	Bacteroidetes	Bacteroides vulgatus ATCC 8482	YP_001298774.1	2 E-56	456/559
Bacteria	Proteobacteria	Bradyrhizobium sp. ORS278	YP_001206303.1	2 E-56	482/559
Bacteria	Actinobacteria	Bifidobacterium bifidum NCIMB 41171	ZP_03646961.1	2 E-56	483/559
Archaea	Crenarchaeota	Sulfolobus solfataricus P2	NP_343812.1	3 E-56	466/559
Archaea	Euryarchaeota	Haloquadratum walsbyi DSM 16790	YP_658717.1	3 E-56	462/559
Bacteria	Proteobacteria	Nitrobacter winogradskyi Nb-255	YP_318014.1	3 E-56	458/559
Bacteria	Bacteroidetes	Salinibacter ruber DSM 13855	YP_446351.1	3 E-56	470/559
Bacteria	Proteobacteria	Magnetococcus sp. MC-1	YP_864318.1	3 E-56	461/559
Bacteria	Actinobacteria	Streptomyces toxytricini	ACM44494.1	3 E-56	489/559
Eukaryota	Metazoa	Xenopus laevis	AAH61665.1	3 E-56	459/559
Archaea	Euryarchaeota	Halobacterium sp. NRC-1	NP_280337.1	4 E-56	461/559
Bacteria	Bacteroidetes	Bacteroides uniformis ATCC 8492	ZP_02071470.1	4 E-56	456/559
Bacteria	Actinobacteria	Streptomyces sp. Mg1	ZP_04998604.1	4 E-56	495/559
Bacteria	Actinobacteria	Streptomyces sp. C	ZP_05508633.1	5 E-56	474/559
Bacteria	Proteobacteria	Methylobacterium chloromethanicum CM4	YP_002419191.1	5 E-56	483/559
Bacteria	Proteobacteria	Methylobacterium extorquens PA1	YP_001637777.1	6 E-56	483/559
Bacteria	Bacteroidetes	Bacteroides fragilis YCH46	YP_100808.1	6 E-56	456/559
Bacteria	Chloroflexi	Roseiflexus sp. RS-1	YP_001278000.1	6 E-56	459/559
Bacteria	Actinobacteria	Streptomyces sviceus ATCC 29083	ZP_05020741.1	6 E-56	488/559
Bacteria	Actinobacteria	Streptomyces sp. AA4	ZP_05482497.1	6 E-56	487/559
Bacteria	Bacteroidetes	Bacteroides ovatus ATCC 8483	ZP_02064497.1	7 E-56	457/559
Bacteria	Actinobacteria	Streptomyces clavuligerus ATCC 27064	ZP_05005113.1	7 E-56	473/559
Bacteria	Actinobacteria	Streptomyces sp. Mg1	ZP_05001652.1	7 E-56	487/559
Bacteria	Firmicutes	Thermoanaerobacter tengcongensis MB4	NP_622846.1	7 E-56	476/559
Bacteria	Bacteroidetes	Bacteroides fragilis NCTC 9343	YP_212950.1	9 E-56	456/559
Eukaryota	Metazoa	Xenopus (Silurana) tropicalis	AAH96507.1	9 E-56	459/559
Bacteria	Bacteroidetes	Bacteroides sp. 2_2_4	ZP_04550746.1	9 E-56	457/559
Bacteria	Proteobacteria	Anaeromyxobacter dehalogenans 2CP-C	YP_463463.1	9 E-56	463/559
Archaea	Euryarchaeota	Halorubrum lacusprofundi ATCC 49239	YP_002567179.1	9 E-56	461/559
Bacteria	Bacteroidetes	Bacteroides sp. D2	ZP_05760322.1	9 E-56	457/559
Bacteria	Bacteroidetes	Bacteroides fragilis 3_1_12	ZP_05284051.1	9 E-56	456/559
Bacteria	Acidobacteria	Candidatus Koribacter versatilis Ellin345	YP_593771.1	1 E-55	463/559
Archaea	Thaumarchaeota	Nitrosopumilus maritimus SCM1	YP_001581606.1	1 E-55	469/559
Bacteria	Proteobacteria	Acidovorax sp. JS42	YP_986294.1	1 E-55	486/559
Bacteria	Chloroflexi	Roseiflexus castenholzii DSM 13941	YP_001431082.1	1 E-55	459/559
Bacteria	Actinobacteria	Catenulispora acidiphila DSM 44928	YP_003117193.1	1 E-55	463/559
Bacteria	Bacteroidetes	Bacteroides thetaiotaomicron VPI-5482	NP_810830.1	1 E-55	457/559
Bacteria	Proteobacteria	Methylobacterium extorquens DM4	YP_003065890.1	1 E-55	458/559
Bacteria	Bacteroidetes	Prevotella melaninogenica ATCC 25845	ZP_04832966.1	2 E-55	480/559
Eukaryota	Metazoa	Xenopus (Silurana) tropicalis	AAI18687.1	2 E-55	459/559
Bacteria	Firmicutes	Dethiobacter alkaliphilus AHT 1	ZP_03729978.1	2 E-55	461/559
Bacteria	Actinobacteria	Streptomyces pristinaespiralis ATCC 25486	ZP_05013396.1	2 E-55	485/559
Bacteria	Firmicutes	Pelotomaculum thermopropionicum SI	YP_001211914.1	2 E-55	481/559
Bacteria	Actinobacteria	Streptomyces lividans TK24	ZP_05524025.1	2 E-55	504/559

Bacteria	Actinobacteria	Catenulispora acidiphila DSM 44928	YP_003114287.1	2 E-55	483/559
Bacteria	Bacteroidetes	Bacteroides sp. D1	ZP_04544623.1	2 E-55	457/559
Bacteria	Proteobacteria	Stigmatella aurantiaca DW4/3-1	ZP_01465745.1	2 E-55	477/559
Bacteria	Proteobacteria	Diaphorobacter sp. TPSY	YP_002553308.1	2 E-55	486/559
Bacteria	Bacteroidetes	Porphyromonas uenonis 60-3	ZP_04056043.1	2 E-55	483/559
Archaea	Euryarchaeota	Halogeometricum borinquense DSM 11551	ZP_03999965.1	2 E-55	483/559
Bacteria	Actinobacteria	Bifidobacterium breve DSM 20213	ZP_03618011.1	2 E-55	464/559
Bacteria	Actinobacteria	Streptomyces viridochromogenes DSM 40736	ZP_05534436.1	3 E-55	485/559
Bacteria	Proteobacteria	Brevundimonas sp. BAL3	ZP_05033122.1	3 E-55	460/559
Bacteria	Bacteroidetes	Prevotella sp. oral taxon	ZP_05918127.1	3 E-55	499/559
Bacteria	Proteobacteria	Acidovorax citrulli AAC00-1	YP_970860.1	3 E-55	486/559
Bacteria	Actinobacteria	Streptomyces sp. AM-7161	BAC79026.1	3 E-55	471/559
Bacteria	Proteobacteria	Myxococcus xanthus DK 1622	YP_629373.1	4 E-55	463/559
Bacteria	Candidatus	Candidatus Accumulibacter phosphatis clade	YP_003166397.1	4 E-55	477/559
Bacteria	Bacteroidetes	Prevotella veroralis F0319	ZP_05857947.1	4 E-55	480/559
Bacteria	Actinobacteria	Bifidobacterium longum NCC2705	NP_696692.1	4 E-55	464/559
Bacteria	Proteobacteria	Acidovorax delafieldii 2AN	ZP_04763112.1	4 E-55	486/559
Bacteria	Actinobacteria	Bifidobacterium longum subsp. infantis	YP_002323721.1	4 E-55	464/559
Bacteria	Proteobacteria	Bradyrhizobium japonicum USDA 110	NP_771996.1	5 E-55	458/559
Bacteria	Proteobacteria	Anaeromyxobacter sp. K	YP_002132631.1	5 E-55	463/559
Bacteria	Bacteroidetes	Parabacteroides distasonis ATCC 8503	YP_001301492.1	5 E-55	463/559
Bacteria	Chlorobi	Chloroherpeton thalassium ATCC 35110	YP_001995096.1	5 E-55	464/559
Bacteria	Actinobacteria	Streptomyces coelicolor A3(2)	NP_629079.1	5 E-55	504/559
Bacteria	Actinobacteria	Kytococcus sedentarius DSM 20547	YP_003148653.1	5 E-55	497/559
Bacteria	Bacteroidetes	Bacteroides sp. 2_1_7	ZP_05284862.1	6 E-55	456/559
Bacteria	Proteobacteria	Mesorhizobium opportunistum WSM2075	ZP_05807789.1	6 E-55	482/559
Bacteria	Bacteroidetes	Gramella forsetii KT0803	YP_861017.1	6 E-55	463/559
Bacteria	Proteobacteria	Anaeromyxobacter dehalogenans 2CP-1	YP_002490696.1	7 E-55	463/559
Bacteria	Proteobacteria	Aurantimonas manganoxydans SI85-9A1	ZP_01225645.1	8 E-55	482/559
Bacteria	Firmicutes	Thermoanaerobacterium thermosaccharolyticum	ZP_05336044.1	8 E-55	476/559
Bacteria	Proteobacteria	Sinorhizobium medicae WSM419	YP_001312977.1	8 E-55	463/559
Bacteria	Actinobacteria	Streptomyces coelicolor A3(2)	NP_630382.1	8 E-55	477/559
Bacteria	Synergistetes	Dethiosulfovibrio peptidovorans DSM 11002	ZP_04340078.1	9 E-55	468/559
Bacteria	Bacteroidetes	Bacteroides plebeius DSM 17135	ZP_03209394.1	9 E-55	462/559
Bacteria	Actinobacteria	Bifidobacterium gallicum DSM 20093	ZP_05966224.1	1 E-54	467/559
Bacteria	Bacteroidetes	Parabacteroides sp. D13	ZP_05544318.1	1 E-54	456/559
Bacteria	Actinobacteria	Streptomyces viridochromogenes DSM 40736	ZP_05533861.1	1 E-54	508/559
Bacteria	Actinobacteria	Streptomyces sp. C	ZP_05509270.1	1 E-54	492/559
Bacteria	Proteobacteria	Mesorhizobium loti MAFF303099	NP_102162.1	1 E-54	482/559
Bacteria	Proteobacteria	Rhizobium sp. NGR234	YP_002824288.1	1 E-54	463/559
Bacteria	Actinobacteria	Streptomyces scabiei 87.22	CBG70551.1	1 E-54	509/559
Bacteria	Actinobacteria	Streptomyces albus J1074	ZP_04705037.1	1 E-54	475/559
Bacteria	Deferribacteres	Denitrovibrio acetiphilus DSM 12809	ZP_03906752.1	2 E-54	463/559
Bacteria	Bacteroidetes	Croceibacter atlanticus HTCC2559	ZP_00950919.1	2 E-54	462/559
Bacteria	Proteobacteria	Aromatoleum aromaticum EbN1	YP_159133.1	2 E-54	485/559
Eukaryota	Metazoa	Danio rerio	NP_998090.1	2 E-54	461/559
Eukaryota	Metazoa	Danio rerio	CAQ13492.1	2 E-54	461/559
Bacteria	Proteobacteria	Desulfococcus oleovorans Hxd3	YP_001527964.1	2 E-54	497/559
Bacteria	Proteobacteria	Verminephrobacter eiseniae EF01-2	YP_998140.1	2 E-54	485/559
Bacteria	Bacteroidetes	Bacteroides dorei DSM 17855	ZP_03303587.1	2 E-54	456/559
Bacteria	Actinobacteria	Salinispora arenicola CNS-205	YP_001535712.1	2 E-54	470/559
Bacteria	Actinobacteria	Streptomyces cyanogenus	AAD13544.1	2 E-54	491/559
Bacteria	Actinobacteria	Corynebacterium kroppenstedtii DSM 44385	YP_002905385.1	2 E-54	467/559
Bacteria	Actinobacteria	Streptomyces sp. SPB78	ZP_05489921.1	2 E-54	471/559
Bacteria	Bacteroidetes	Bacteroides vulgatus ATCC 8482	YP_001300358.1	2 E-54	456/559
Bacteria	Proteobacteria	Labrenzia aggregata IAM 12614	ZP_01548528.1	2 E-54	482/559
Bacteria	Proteobacteria	gamma proteobacterium HTCC2207	ZP_01225349.1	2 E-54	458/559
Bacteria	Actinobacteria	Arthrobacter chlorophenolicus A6	YP_002487404.1	2 E-54	468/559

Bacteria	Bacteroidetes	Bacteroides caccae ATCC 43185	ZP_01962068.1	2 E-54	457/559
Bacteria	Proteobacteria	Methylocella silvestris BL2	YP_002364031.1	2 E-54	457/559
Bacteria	Proteobacteria	Rhodospirillum rubrum ATCC 11170	YP_425145.1	3 E-54	474/559
Bacteria	Bacteroidetes	Bacteroides coprocola DSM 17136	ZP_03009519.1	3 E-54	489/559

AFUA_5G07620

Eukaryota	Fungi	Aspergillus fumigatus Af293	XP_753873.1	0.0	660/660
Eukaryota	Fungi	Neosartorya fischeri NRRL 181	XP_001259920.1	0.0	663/660
Eukaryota	Fungi	Aspergillus clavatus NRRL 1	XP_001274083.1	0.0	661/660
Eukaryota	Fungi	Aspergillus nidulans FGSC A4	XP_661083.1	0.0	609/660
Eukaryota	Fungi	Aspergillus terreus NIH2624	XP_001218362.1	0.0	643/660
Eukaryota	Fungi	Penicillium chrysogenum Wisconsin 54-1255	XP_002561453.1	0.0	631/660
Eukaryota	Fungi	Aspergillus fumigatus A1163	EDP53515.1	0.0	628/660
Eukaryota	Fungi	Aspergillus fumigatus Af293	XP_748465.2	0.0	628/660
Eukaryota	Fungi	Aspergillus niger CBS 513.88	XP_001396423.1	0.0	565/660
Eukaryota	Fungi	Neosartorya fischeri NRRL 181	XP_001258786.1	0.0	629/660
Eukaryota	Fungi	Penicillium chrysogenum Wisconsin 54-1255	XP_002564841.1	0.0	598/660
Eukaryota	Fungi	Aspergillus clavatus NRRL 1	XP_001273759.1	0.0	638/660
Eukaryota	Fungi	Aspergillus flavus NRRL3357	XP_002383537.1	0.0	623/660
Eukaryota	Fungi	Aspergillus nidulans FGSC A4	XP_681764.1	0.0	549/660
Eukaryota	Fungi	Penicillium marneffei ATCC 18224	XP_002152770.1	0.0	572/660
Eukaryota	Fungi	Talaromyces stipitatus ATCC 10500	XP_002486494.1	1 E-176	578/660
Eukaryota	Fungi	Aspergillus terreus NIH2624	XP_001211319.1	1 E-175	637/660
Eukaryota	Fungi	Aspergillus flavus NRRL3357	XP_002377443.1	1 E-175	561/660
Eukaryota	Fungi	Penicillium chrysogenum Wisconsin 54-1255	XP_002558258.1	1 E-173	541/660
Eukaryota	Fungi	Neosartorya fischeri NRRL 181	XP_001262478.1	1 E-171	563/660
Eukaryota	Fungi	Penicillium marneffei ATCC 18224	XP_002153057.1	1 E-167	623/660
Eukaryota	Fungi	Talaromyces stipitatus ATCC 10500	XP_002487527.1	1 E-165	625/660
Eukaryota	Fungi	Aspergillus flavus NRRL3357	XP_002376027.1	1 E-161	572/660
Eukaryota	Fungi	Aspergillus terreus NIH2624	XP_001213215.1	1 E-159	574/660
Eukaryota	Fungi	Aspergillus clavatus NRRL 1	XP_001271350.1	1 E-158	576/660
Eukaryota	Fungi	Neosartorya fischeri NRRL 181	XP_001263069.1	1 E-156	573/660
Eukaryota	Fungi	Aspergillus niger CBS 513.88	XP_001393791.1	1 E-156	571/660
Eukaryota	Fungi	Aspergillus fumigatus Af293	XP_754230.1	1 E-155	573/660
Eukaryota	Fungi	Aspergillus nidulans FGSC A4	XP_660747.1	1 E-152	577/660
Eukaryota	Fungi	Penicillium chrysogenum Wisconsin 54-1255	XP_002559937.1	1 E-151	568/660
Eukaryota	Fungi	Sclerotinia sclerotiorum 1980 UF-70	XP_001598955.1	1 E-147	581/660
Eukaryota	Fungi	Talaromyces stipitatus ATCC 10500	XP_002479840.1	1 E-146	590/660
Eukaryota	Fungi	Botryotinia fuckeliana B05.10	XP_001547557.1	1 E-141	582/660
Eukaryota	Fungi	Phaeosphaeria nodorum SN15	XP_001803898.1	1 E-140	532/660
Eukaryota	Fungi	Coccidioides posadasii C735 delta	EER24686.1	1 E-139	544/660
Eukaryota	Fungi	Microsporum canis CBS 113480	EEQ33786.1	1 E-139	624/660
Eukaryota	Fungi	Ashbya gossypii ATCC 10895	NP_985622.1	1 E-131	547/660
Eukaryota	Fungi	Clavispora lusitaniae ATCC 42720	XP_002614443.1	1 E-130	583/660
Eukaryota	Fungi	Pichia stipitis CBS 6054	XP_001385940.2	1 E-130	605/660
Eukaryota	Fungi	Pichia guilliermondii ATCC 6260	XP_001482058.1	1 E-127	597/660
Eukaryota	Fungi	Pichia guilliermondii ATCC 6260	EDK41723.2	1 E-127	603/660
Eukaryota	Fungi	Candida tropicalis MYA-3404	XP_002550871.1	1 E-127	604/660
Eukaryota	Fungi	Kluyveromyces lactis NRRL Y-1140	XP_455207.1	1 E-126	550/660
Eukaryota	Fungi	Debaryomyces hansenii	CAG85871.2	1 E-126	610/660
Eukaryota	Fungi	Debaryomyces hansenii CBS767	XP_457826.1	1 E-126	610/660
Eukaryota	Fungi	Candida glabrata CBS 138	XP_446552.1	1 E-126	650/660
Eukaryota	Fungi	Lachancea thermotolerans CBS 6340	XP_002554147.1	1 E-126	567/660
Eukaryota	Fungi	Schizosaccharomyces pombe	NP_593846.1	1 E-124	602/660
Eukaryota	Fungi	Paracoccidioides brasiliensis Pb01;	EEH42087.1	1 E-124	620/660
Eukaryota	Fungi	Ajellomyces dermatitidis SLH14081	XP_002623141.1	1 E-124	650/660
Eukaryota	Fungi	Ajellomyces dermatitidis ER-3	EEQ90916.1	1 E-124	650/660

Eukaryota	Fungi	Paracoccidioides brasiliensis Pb03;	EEH18455.1	1 E-123	623/660
Eukaryota	Fungi	Nectria haematococca mpVI 77-13-4	EEU40142.1	1 E-122	625/660
Eukaryota	Fungi	Saccharomyces cerevisiae YJM789	EDN63271.1	1 E-121	619/660
Eukaryota	Fungi	Saccharomyces cerevisiae EC1118	CAY80622.1	1 E-121	619/660
Eukaryota	Fungi	Zygosaccharomyces rouxii CBS 732	XP_002498726.1	1 E-120	574/660
Eukaryota	Fungi	Pyrenophora tritici-repentis Pt-1C-BFP	XP_001936983.1	1 E-116	588/660
Eukaryota	Fungi	Verticillium albo-atrum VaMs.102	EEY20110.1	1 E-114	541/660
Eukaryota	Fungi	Pichia farinosa	CAB62252.1	1 E-113	589/660
Eukaryota	Fungi	Schizosaccharomyces japonicus yFS275	XP_002176026.1	1 E-113	601/660
Eukaryota	Fungi	Podospora anserina DSM 980	XP_001912741.1	1 E-113	550/660
Eukaryota	Fungi	Ustilago maydis 521	XP_761924.1	2 E-82	606/660
Eukaryota	Fungi	Malassezia globosa CBS 7966	XP_001732181.1	4 E-70	552/660

AFUA_5G09980

Eukaryota	Fungi	Aspergillus fumigatus Af293	XP_753642.2	0.0	647/647
Eukaryota	Fungi	Neosartorya fischeri NRRL 181	XP_001259693.1	0.0	639/647
Eukaryota	Fungi	Aspergillus clavatus NRRL 1	XP_001274307.1	0.0	640/647
Eukaryota	Fungi	Aspergillus oryzae RIB40	XP_001817542.1	0.0	624/647
Eukaryota	Fungi	Aspergillus flavus NRRL3357	XP_002372645.1	0.0	624/647
Eukaryota	Fungi	Aspergillus terreus NIH2624	XP_001215488.1	0.0	626/647
Eukaryota	Fungi	Penicillium chrysogenum Wisconsin 54-1255	XP_002566402.1	0.0	626/647
Eukaryota	Fungi	Aspergillus nidulans FGSC A4	XP_664359.1	0.0	639/647
Eukaryota	Fungi	Penicillium chrysogenum Wisconsin 54-1255	XP_002559610.1	0.0	603/647
Eukaryota	Fungi	Aspergillus terreus NIH2624	XP_001211103.1	1 E-176	606/647
Eukaryota	Fungi	Paracoccidioides brasiliensis Pb01;	EEH34736.1	1 E-115	593/647
Eukaryota	Fungi	Paracoccidioides brasiliensis Pb03;	EEH16264.1	1 E-114	610/647
Eukaryota	Fungi	Paracoccidioides brasiliensis Pb18;	EEH42784.1	1 E-114	610/647
Eukaryota	Fungi	Penicillium marneffei ATCC 18224	XP_002151916.1	1 E-111	605/647
Eukaryota	Fungi	Ajellomyces capsulatus NAm1	XP_001540639.1	1 E-108	611/647
Eukaryota	Fungi	Ajellomyces capsulatus G186AR	EEH10948.1	1 E-106	611/647
Eukaryota	Fungi	Microsporum canis CBS 113480	EEQ32860.1	1 E-106	607/647
Eukaryota	Fungi	Ajellomyces dermatitidis SLH14081	XP_002624878.1	1 E-105	617/647
Eukaryota	Fungi	Coccidioides posadasii C735 delta	EER29604.1	1 E-104	607/647
Eukaryota	Fungi	Coccidioides immitis RS;	XP_001245039.1	1 E-104	607/647
Eukaryota	Fungi	Ajellomyces dermatitidis ER-3	EEQ86697.1	1 E-104	617/647
Eukaryota	Fungi	Talaromyces stipitatus ATCC 10500	XP_002480899.1	1 E-100	607/647
Eukaryota	Fungi	Nectria haematococca mpVI 77-13-4	EEU45093.1	2 E-99	621/647
Eukaryota	Fungi	Gibberella zeae PH-1	XP_390383.1	1 E-98	596/647
Eukaryota	Fungi	Ajellomyces capsulatus H143	EER42980.1	1 E-96	551/647
Eukaryota	Fungi	Penicillium marneffei ATCC 18224	XP_002146316.1	3 E-90	559/647
Eukaryota	Fungi	Podospora anserina DSM 980	XP_001912080.1	2 E-88	572/647
Eukaryota	Fungi	Phaeosphaeria nodorum SN15	XP_001792459.1	2 E-87	574/647
Eukaryota	Fungi	Neurospora crassa	CAD11366.1	7 E-87	542/647
Eukaryota	Fungi	Neurospora crassa OR74A	XP_961222.2	7 E-87	542/647
Eukaryota	Fungi	Yarrowia lipolytica CLIB122	XP_500511.1	1 E-84	572/647
Eukaryota	Fungi	Verticillium albo-atrum VaMs.102	EEY18668.1	3 E-83	581/647
Eukaryota	Fungi	Aspergillus niger CBS 513.88	XP_001393689.1	6 E-83	585/647
Eukaryota	Metazoa	Salmo salar	NP_001133849.1	1 E-73	536/647
Eukaryota	Amoebozoa	Dictyostelium discoideum AX4	XP_646730.1	8 E-72	565/647
Eukaryota	Metazoa	Monodelphis domestica	XP_001380274.1	1 E-70	519/647
Eukaryota	Metazoa	Caenorhabditis elegans	NP_001033378.1	2 E-69	537/647
Eukaryota	Metazoa	Caenorhabditis elegans	NP_001033379.1	3 E-69	537/647
Eukaryota	Metazoa	Gallus gallus	XP_415303.2	1 E-67	533/647
Eukaryota	Metazoa	Caenorhabditis briggsae AF16	XP_001665949.1	2 E-67	559/647
Eukaryota	Fungi	Coprinopsis cinerea okayama7#130	XP_001835802.1	3 E-67	577/647
Eukaryota	Metazoa	Monodelphis domestica	XP_001380314.1	4 E-67	519/647
Bacteria	Proteobacteria	Sorangium cellulosum 'So ce	YP_001616402.1	4 E-66	529/647

Eukaryota	Metazoa	Branchiostoma floridae	XP_002596671.1	1 E-65	524/647
Eukaryota	Metazoa	Monodelphis domestica	XP_001380303.1	9 E-65	524/647
Eukaryota	Metazoa	Xenopus laevis	NP_001086464.1	3 E-64	539/647
Eukaryota	Metazoa	Xenopus laevis	AAH80048.1	1 E-63	539/647
Eukaryota	Metazoa	Xenopus (Silurana) tropicalis	CAJ82159.1	2 E-63	539/647
Eukaryota	Metazoa	Xenopus (Silurana) tropicalis	NP_001025577.1	2 E-63	539/647
Eukaryota	Fungi	Laccaria bicolor S238N-H82	XP_001875865.1	5 E-63	550/647
Eukaryota	Metazoa	Tetraodon nigroviridis	CAG08549.1	2 E-61	532/647
Bacteria	Chloroflexi	Roseiflexus sp. RS-1	YP_001278600.1	5 E-60	524/647
Eukaryota	Alveolata	Tetrahymena thermophila	XP_001027832.1	1 E-56	574/647
Archaea	Euryarchaeota	Natrialba magadii ATCC 43099	ZP_03692141.1	1 E-35	558/647

AFUA_5G09990

Eukaryota	Fungi	Aspergillus fumigatus Af293	XP_753641.1	0.0	555/555
Eukaryota	Fungi	Neosartorya fischeri NRRL 181	XP_001259692.1	0.0	521/555
Eukaryota	Fungi	Aspergillus clavatus NRRL 1	XP_001274308.1	0.0	553/555
Eukaryota	Fungi	Penicillium chrysogenum Wisconsin 54-1255	XP_002566400.1	1 E-164	595/555
Eukaryota	Fungi	Paracoccidioides brasiliensis Pb01;	EEH40097.1	2 E-96	452/555
Eukaryota	Fungi	Paracoccidioides brasiliensis Pb03;	EEH18658.1	2 E-96	451/555
Eukaryota	Fungi	Uncinocarpus reesii 1704	XP_002542869.1	3 E-96	466/555
Eukaryota	Fungi	Coccidioides immitis RS;	XP_001240951.1	9 E-95	460/555
Eukaryota	Fungi	Coccidioides posadasii C735 delta	EER23101.1	1 E-94	460/555
Eukaryota	Fungi	Phaeosphaeria nodorum SN15	XP_001795484.1	3 E-94	476/555
Eukaryota	Fungi	Ajellomyces dermatitidis SLH14081	XP_002620837.1	4 E-92	450/555
Eukaryota	Fungi	Ajellomyces capsulatus G186AR	EEH04686.1	5 E-92	450/555
Eukaryota	Fungi	Penicillium chrysogenum Wisconsin 54-1255	XP_002559851.1	3 E-85	446/555
Eukaryota	Fungi	Ajellomyces capsulatus H143	EER40123.1	7 E-84	471/555
Eukaryota	Fungi	Ajellomyces dermatitidis ER-3	EEQ92136.1	1 E-74	460/555
Eukaryota	Fungi	Ajellomyces dermatitidis SLH14081	XP_002622561.1	1 E-74	460/555
Eukaryota	Fungi	Paracoccidioides brasiliensis Pb03;	EEH19716.1	4 E-74	463/555
Eukaryota	Fungi	Paracoccidioides brasiliensis Pb01;	EEH38945.1	8 E-74	463/555
Eukaryota	Fungi	Gibberella zeae PH-1	XP_386636.1	5 E-68	481/555
Eukaryota	Fungi	Paracoccidioides brasiliensis Pb18;	EEH44070.1	7 E-66	451/555
Eukaryota	Fungi	Neosartorya fischeri NRRL 181	XP_001264098.1	2 E-23	452/555
Eukaryota	Fungi	Pichia pastoris GS115	XP_002489620.1	1 E-21	451/555
Eukaryota	Fungi	Candida tropicalis MYA-3404	XP_002546974.1	5 E-18	452/555
Eukaryota	Fungi	Pichia guilliermondii ATCC 6260	EDK39068.2	3 E-17	453/555
Eukaryota	Fungi	Pichia stipitis CBS 6054	XP_001383436.1	9 E-17	449/555
Eukaryota	Fungi	Lodderomyces elongisporus NRRL YB-4239	XP_001528426.1	3 E-16	449/555
Eukaryota	Fungi	Pichia guilliermondii ATCC 6260	XP_001485437.1	6 E-14	445/555

AFUA_5G10120

Eukaryota	Fungi	Aspergillus fumigatus Af293	XP_753630.1	0.0	1274/1274
Eukaryota	Fungi	Neosartorya fischeri NRRL 181	XP_001259681.1	0.0	1273/1274
Eukaryota	Fungi	Aspergillus clavatus NRRL 1	XP_001274334.1	0.0	1274/1274
Eukaryota	Fungi	Aspergillus terreus NIH2624	XP_001211581.1	0.0	1273/1274
Eukaryota	Fungi	Aspergillus niger CBS 513.88	XP_001399159.1	0.0	1276/1274
Eukaryota	Fungi	Aspergillus nidulans FGSC A4	XP_662922.1	0.0	1270/1274
Eukaryota	Fungi	Aspergillus oryzae RIB40	XP_001817726.1	0.0	1278/1274
Eukaryota	Fungi	Aspergillus flavus NRRL3357	XP_002372857.1	0.0	1278/1274
Eukaryota	Fungi	Ajellomyces dermatitidis SLH14081	XP_002625314.1	0.0	1275/1274
Eukaryota	Fungi	Ajellomyces dermatitidis ER-3	EEQ84688.1	0.0	1275/1274
Eukaryota	Fungi	Talaromyces stipitatus ATCC 10500	XP_002477944.1	0.0	1277/1274
Eukaryota	Fungi	Penicillium marneffei ATCC 18224	XP_002145684.1	0.0	1275/1274
Eukaryota	Fungi	Penicillium chrysogenum Wisconsin 54-1255	XP_002561885.1	0.0	1276/1274
Eukaryota	Fungi	Paracoccidioides brasiliensis Pb01;	EEH33786.1	0.0	1276/1274

Eukaryota	Fungi	Ajellomyces capsulatus H143	EER39104.1	0.0	1276/1274
Eukaryota	Fungi	Paracoccidioides brasiliensis Pb03;	EEH17613.1	0.0	1276/1274
Eukaryota	Fungi	Ajellomyces capsulatus G186AR	EEH10260.1	0.0	1276/1274
Eukaryota	Fungi	Leptosphaeria maculans	AAO49458.1	0.0	1282/1274
Eukaryota	Fungi	Pyrenophora tritici-repentis Pt-1C-BFP	XP_001930780.1	0.0	1282/1274
Eukaryota	Fungi	Cochliobolus heterostrophus	AAX09992.1	0.0	1282/1274
Eukaryota	Fungi	Phaeosphaeria nodorum SN15	XP_001797479.1	0.0	1249/1274
Eukaryota	Fungi	Magnaporthe grisea 70-15	XP_360747.1	0.0	1280/1274
Eukaryota	Fungi	Nectria haematococca mpVI 77-13-4	EEU39792.1	0.0	1282/1274
Eukaryota	Fungi	Verticillium albo-atrum VaMs.102	EEY16460.1	0.0	1284/1274
Eukaryota	Fungi	Gibberella moniliformis	AAX11423.1	0.0	1284/1274
Eukaryota	Fungi	Gibberella zeae PH-1	XP_386683.1	0.0	1282/1274
Eukaryota	Fungi	Ustilago maydis 521	XP_759255.1	0.0	1320/1274
Eukaryota	Metazoa	Trichoplax adhaerens	XP_002112422.1	0.0	1203/1274
Eukaryota	stramenopiles	Phaeodactylum tricornutum CCAP 1055/1	XP_002181204.1	0.0	1363/1274
Eukaryota	Metazoa	Branchiostoma floridae	XP_002608488.1	0.0	1321/1274
Eukaryota	stramenopiles	Thalassiosira pseudonana CCMP1335	XP_002290027.1	0.0	1221/1274
Eukaryota	Metazoa	Acyrthosiphon pisum	XP_001942617.1	0.0	1223/1274
Eukaryota	Fungi	Aspergillus flavus NRRL3357	XP_002384042.1	1 E-83	1020/1274
Eukaryota	Fungi	Microsporum canis CBS 113480	EEQ33597.1	5 E-79	1141/1274
Eukaryota	Fungi	Coccidioides immitis RS;	XP_001247720.1	5 E-75	1059/1274
Eukaryota	Fungi	Coccidioides posadasii C735 delta	EER23582.1	3 E-74	1059/1274
Eukaryota	Fungi	Yarrowia lipolytica CLIB122	XP_503627.1	1 E-73	1039/1274
Eukaryota	Fungi	Laccaria bicolor S238N-H82	XP_001879618.1	9 E-73	1042/1274
Eukaryota	Fungi	Penicillium marneffei ATCC 18224	XP_002143711.1	5 E-72	1028/1274
Eukaryota	Fungi	Candida glabrata CBS 138	XP_448559.1	2 E-71	1034/1274
Eukaryota	Fungi	Talaromyces stipitatus ATCC 10500	XP_002480033.1	3 E-71	1031/1274

AFUA_5G13190

Eukaryota	Fungi	Aspergillus fumigatus Af293	XP_753335.1	0.0	1769/1769
Eukaryota	Fungi	Neosartorya fischeri NRRL 181	XP_001259365.1	0.0	1748/1769
Eukaryota	Fungi	Aspergillus clavatus NRRL 1	XP_001274597.1	0.0	1748/1769
Eukaryota	Fungi	Aspergillus terreus NIH2624	XP_001215369.1	0.0	1745/1769
Eukaryota	Fungi	Aspergillus niger CBS 513.88	XP_001401215.1	0.0	1750/1769
Eukaryota	Fungi	Aspergillus oryzae RIB40	XP_001824166.1	0.0	1738/1769
Eukaryota	Fungi	Aspergillus flavus NRRL3357	XP_002381254.1	0.0	1755/1769
Eukaryota	Fungi	Aspergillus nidulans FGSC A4	XP_664479.1	0.0	1660/1769
Eukaryota	Fungi	Penicillium chrysogenum Wisconsin 54-1255	XP_002567839.1	0.0	1751/1769
Eukaryota	Fungi	Coccidioides posadasii C735 delta	EER26982.1	0.0	1783/1769
Eukaryota	Fungi	Coccidioides immitis RS;	XP_001240121.1	0.0	1787/1769
Eukaryota	Fungi	Ajellomyces dermatitidis SLH14081	XP_002628673.1	0.0	1785/1769
Eukaryota	Fungi	Ajellomyces capsulatus G186AR	EEH05193.1	0.0	1789/1769
Eukaryota	Fungi	Ajellomyces capsulatus H143	EER40951.1	0.0	1789/1769
Eukaryota	Fungi	Uncinocarpus reesii 1704	XP_002545150.1	0.0	1781/1769
Eukaryota	Fungi	Paracoccidioides brasiliensis Pb01;	EEH34142.1	0.0	1784/1769
Eukaryota	Fungi	Penicillium marneffei ATCC 18224	XP_002145322.1	0.0	1741/1769
Eukaryota	Fungi	Paracoccidioides brasiliensis Pb18;	EEH48985.1	0.0	1783/1769
Eukaryota	Fungi	Ajellomyces capsulatus NAm1	XP_001543271.1	0.0	1740/1769
Eukaryota	Fungi	Talaromyces stipitatus ATCC 10500	XP_002487228.1	0.0	1694/1769
Eukaryota	Fungi	Paracoccidioides brasiliensis Pb03;	EEH22413.1	0.0	1584/1769
Eukaryota	Fungi	Microsporum canis CBS 113480	EEQ30101.1	0.0	1733/1769
Eukaryota	Fungi	Botryotinia fuckeliana B05.10	XP_001556354.1	0.0	1701/1769
Eukaryota	Fungi	Sclerotinia sclerotiorum 1980 UF-70	XP_001596260.1	0.0	1695/1769
Eukaryota	Fungi	Nectria haematococca mpVI 77-13-4	EEU44050.1	0.0	1683/1769
Eukaryota	Fungi	Chaetomium globosum CBS 148.51	XP_001228119.1	0.0	1804/1769
Eukaryota	Fungi	Podospora anserina DSM 980	XP_001909542.1	0.0	1736/1769
Eukaryota	Fungi	Verticillium albo-atrum VaMs.102	EEY19550.1	0.0	1743/1769

Eukaryota	Fungi	Botryotinia fuckeliana	AAO59285.1	0.0	1526/1769
Eukaryota	Fungi	Neurospora crassa OR74A	XP_963673.1	0.0	1751/1769
Eukaryota	Fungi	Pyrenophora tritici-repentis Pt-1C-BFP	XP_001931100.1	0.0	1712/1769
Eukaryota	Fungi	Neurospora crassa	CAD21208.1	0.0	1706/1769
Eukaryota	Fungi	Cochliobolus heterostrophus	AAO59296.1	0.0	1695/1769
Eukaryota	Fungi	Gibberella moniliformis	AAO59307.1	0.0	1503/1769
Eukaryota	Fungi	Gibberella zeae PH-1	XP_381866.1	0.0	1498/1769

AFUA_5G14270

Eukaryota	Fungi	Aspergillus fumigatus Af293	XP_753230.2	0.0	533/533
Eukaryota	Fungi	Neosartorya fischeri NRRL 181	XP_001259246.1	0.0	528/533
Eukaryota	Fungi	Penicillium chrysogenum Wisconsin 54-1255	XP_002566366.1	0.0	527/533
Eukaryota	Fungi	Aspergillus flavus NRRL3357	XP_002380165.1	0.0	530/533
Eukaryota	Fungi	Aspergillus oryzae RIB40	XP_001818748.1	0.0	516/533
Eukaryota	Fungi	Aspergillus nidulans FGSC A4	XP_660278.1	0.0	520/533
Eukaryota	Fungi	Uncinocarpus reesii 1704	XP_002544754.1	0.0	524/533
Eukaryota	Fungi	Paracoccidioides brasiliensis Pb01;	EEH33848.1	0.0	534/533
Eukaryota	Fungi	Coccidioides posadasii C735 delta	EER27347.1	0.0	532/533
Eukaryota	Fungi	Coccidioides immitis RS;	XP_001242226.1	0.0	518/533
Eukaryota	Fungi	Aspergillus niger CBS 513.88	XP_001401186.1	0.0	530/533
Eukaryota	Fungi	Paracoccidioides brasiliensis Pb18;	EEH46537.1	0.0	534/533
Eukaryota	Fungi	Ajellomyces capsulatus G186AR	EEH10327.1	0.0	518/533
Eukaryota	Fungi	Ajellomyces dermatitidis ER-3	EEQ84619.1	0.0	532/533
Eukaryota	Fungi	Ajellomyces dermatitidis SLH14081	XP_002625251.1	0.0	532/533
Eukaryota	Fungi	Aspergillus terreus NIH2624	XP_001215281.1	1 E-177	444/533
Eukaryota	Fungi	Aspergillus oryzae RIB40	XP_001822323.1	1 E-177	527/533
Eukaryota	Fungi	Aspergillus flavus NRRL3357	XP_002382411.1	1 E-177	527/533
Eukaryota	Fungi	Ajellomyces capsulatus H143	EER39172.1	1 E-177	524/533
Eukaryota	Fungi	Ajellomyces capsulatus NAm1	XP_001542729.1	1 E-176	498/533
Eukaryota	Fungi	Aspergillus niger CBS 513.88	XP_001393497.1	1 E-162	520/533
Eukaryota	Fungi	Paracoccidioides brasiliensis Pb03;	EEH17670.1	1 E-157	457/533
Eukaryota	Fungi	Penicillium marneffei ATCC 18224	XP_002146968.1	1 E-149	533/533
Eukaryota	Fungi	Alternaria alternata	BAH83502.1	1 E-142	503/533
Eukaryota	Fungi	Phaeosphaeria nodorum SN15	XP_001793602.1	1 E-133	516/533
Eukaryota	Fungi	Aspergillus fumigatus Af293	XP_753401.1	2 E-98	528/533
Eukaryota	Fungi	Penicillium chrysogenum Wisconsin 54-1255	XP_002567821.1	3 E-95	532/533
Eukaryota	Fungi	Penicillium marneffei ATCC 18224	XP_002143719.1	3 E-95	542/533
Eukaryota	Fungi	Aspergillus oryzae RIB40	XP_001824095.1	3 E-94	534/533
Eukaryota	Fungi	Neosartorya fischeri NRRL 181	XP_001259446.1	8 E-94	524/533
Eukaryota	Fungi	Aspergillus terreus NIH2624	XP_001211201.1	6 E-93	536/533
Eukaryota	Fungi	Aspergillus nidulans FGSC A4	XP_657658.1	8 E-93	526/533
Eukaryota	Fungi	Aspergillus nidulans FGSC A4	CBF82169.1	5 E-92	526/533
Eukaryota	Fungi	Aspergillus niger CBS 513.88	XP_001401799.1	9 E-92	530/533
Eukaryota	Fungi	Aspergillus nidulans FGSC A4	XP_662890.1	2 E-90	518/533
Eukaryota	Fungi	Aspergillus niger CBS 513.88	XP_001401293.1	2 E-90	536/533
Eukaryota	Fungi	Aspergillus clavatus NRRL 1	XP_001274541.1	6 E-90	539/533
Eukaryota	Fungi	Talaromyces stipitatus ATCC 10500	XP_002480055.1	3 E-89	532/533
Eukaryota	Fungi	Coccidioides posadasii C735 delta	EER27897.1	2 E-87	527/533
Eukaryota	Fungi	Pyrenophora tritici-repentis Pt-1C-BFP	XP_001940616.1	1 E-86	523/533
Eukaryota	Fungi	Aspergillus terreus NIH2624	XP_001209989.1	1 E-86	530/533
Eukaryota	Fungi	Coccidioides immitis RS;	XP_001242925.1	1 E-85	537/533
Eukaryota	Fungi	Aspergillus oryzae RIB40	XP_001727572.1	1 E-85	498/533
Eukaryota	Fungi	Coccidioides posadasii C735 delta	EER30068.1	6 E-85	525/533
Eukaryota	Fungi	Coccidioides immitis RS;	XP_001239283.1	6 E-85	525/533
Eukaryota	Fungi	Aspergillus niger CBS 513.88	XP_001392724.1	3 E-84	503/533
Eukaryota	Fungi	Aspergillus flavus NRRL3357	XP_002375856.1	2 E-83	498/533
Eukaryota	Fungi	Aspergillus nidulans FGSC A4	XP_658253.1	9 E-83	505/533

Eukaryota	Fungi	Pyrenophora tritici-repentis Pt-1C-BFP	XP_001940180.1	1 E-81	530/533
Eukaryota	Fungi	Gibberella zeae PH-1	XP_383765.1	3 E-81	542/533
Eukaryota	Fungi	Ajellomyces dermatitidis SLH14081	XP_002628017.1	3 E-81	500/533
Eukaryota	Fungi	Nectria haematococca mpVI 77-13-4	EEU45163.1	4 E-81	536/533
Eukaryota	Fungi	Nectria haematococca mpVI 77-13-4	EEU37414.1	5 E-81	546/533
Eukaryota	Fungi	Aspergillus oryzae RIB40	XP_001822397.1	5 E-81	529/533
Eukaryota	Fungi	Phaeosphaeria nodorum SN15	XP_001800160.1	8 E-81	501/533
Eukaryota	Fungi	Ajellomyces dermatitidis ER-3	EEQ88814.1	9 E-81	500/533
Eukaryota	Fungi	Phaeosphaeria nodorum SN15	XP_001800661.1	2 E-80	525/533
Eukaryota	Fungi	Aspergillus clavatus NRRL 1	XP_001268948.1	3 E-80	507/533
Eukaryota	Fungi	Neosartorya fischeri NRRL 181	XP_001264449.1	5 E-80	507/533
Eukaryota	Fungi	Aspergillus fumigatus Af293	XP_752681.1	7 E-80	511/533
Eukaryota	Fungi	Neurospora crassa OR74A	XP_959842.1	1 E-79	511/533
Eukaryota	Fungi	Aspergillus oryzae RIB40	XP_001817913.1	1 E-79	518/533
Eukaryota	Fungi	Aspergillus niger CBS 513.88	XP_001394929.1	3 E-79	507/533
Eukaryota	Fungi	Aspergillus nidulans FGSC A4	CBF89623.1	3 E-79	521/533
Eukaryota	Fungi	Aspergillus clavatus NRRL 1	XP_001271444.1	6 E-79	509/533
Eukaryota	Fungi	Penicillium chrysogenum Wisconsin 54-1255	XP_002559643.1	2 E-77	516/533
Eukaryota	Fungi	Paracoccidioides brasiliensis Pb01;	EEH39401.1	2 E-77	525/533
Eukaryota	Fungi	Aspergillus terreus NIH2624	XP_001215262.1	3 E-77	525/533
Eukaryota	Fungi	Neosartorya fischeri NRRL 181	XP_001262313.1	5 E-77	554/533
Eukaryota	Fungi	Botryotinia fuckeliana B05.10	XP_001560123.1	5 E-77	515/533
Eukaryota	Fungi	Microsporum canis CBS 113480	EEQ28839.1	3 E-76	518/533
Eukaryota	Fungi	Aspergillus fumigatus Af293	XP_751267.1	5 E-76	551/533
Eukaryota	Fungi	Aspergillus fumigatus A1163	EDP55392.1	6 E-76	551/533
Eukaryota	Fungi	Aspergillus terreus NIH2624	XP_001210592.1	2 E-75	528/533
Eukaryota	Metazoa	Strongylocentrotus purpuratus	XP_001200865.1	3 E-75	480/533
Eukaryota	Fungi	Podospora anserina DSM 980	XP_001910943.1	4 E-75	541/533
Eukaryota	Fungi	Aspergillus flavus NRRL3357	XP_002373072.1	7 E-75	537/533
Eukaryota	Metazoa	Trichoplax adhaerens	XP_002112311.1	7 E-75	505/533
Eukaryota	Fungi	Aspergillus nidulans FGSC A4	XP_682350.1	1 E-74	512/533
Eukaryota	Fungi	Neurospora crassa	CAD70894.1	3 E-74	522/533
Eukaryota	Fungi	Gibberella zeae PH-1	XP_390406.1	5 E-74	539/533
Eukaryota	Metazoa	Trichoplax adhaerens	XP_002112331.1	8 E-74	484/533
Eukaryota	Metazoa	Trichoplax adhaerens	XP_002108496.1	2 E-73	464/533
Eukaryota	Fungi	Aspergillus fumigatus	CAF32026.1	2 E-73	517/533
Eukaryota	Fungi	Aspergillus terreus NIH2624	XP_001211888.1	4 E-73	490/533
Eukaryota	Fungi	Neurospora crassa OR74A	XP_960912.2	7 E-73	511/533
Eukaryota	Fungi	Uncinocarpus reesii 1704	XP_002543918.1	2 E-72	523/533
Eukaryota	Fungi	Sclerotinia sclerotiorum 1980 UF-70	XP_001586224.1	2 E-72	521/533
Eukaryota	Metazoa	Trichoplax adhaerens	XP_002107768.1	3 E-72	498/533
Eukaryota	Fungi	Aspergillus terreus NIH2624	XP_001214079.1	4 E-72	518/533
Eukaryota	Fungi	Nectria haematococca mpVI 77-13-4	EEU37978.1	4 E-72	548/533
Eukaryota	Fungi	Sclerotinia sclerotiorum 1980 UF-70	XP_001589574.1	6 E-72	476/533
Eukaryota	Fungi	Verticillium albo-atrum VaMs.102	EEY20127.1	9 E-72	524/533
Eukaryota	Fungi	Verticillium albo-atrum VaMs.102	EEY18669.1	9 E-72	513/533
Eukaryota	Metazoa	Branchiostoma floridae	XP_002587331.1	1 E-71	482/533
Eukaryota	Fungi	Paracoccidioides brasiliensis Pb01;	EEH34942.1	2 E-71	518/533
Eukaryota	Fungi	Penicillium marneffei ATCC 18224	XP_002148217.1	3 E-71	549/533
Eukaryota	Fungi	Penicillium marneffei ATCC 18224	XP_002146352.1	4 E-71	534/533
Eukaryota	Metazoa	Branchiostoma floridae	XP_002610164.1	6 E-71	489/533
Eukaryota	Fungi	Magnaporthe grisea 70-15	XP_370522.2	6 E-71	535/533
Eukaryota	Fungi	Paracoccidioides brasiliensis Pb18;	EEH49929.1	1 E-70	518/533
Eukaryota	Fungi	Paracoccidioides brasiliensis Pb03;	EEH23445.1	2 E-70	520/533
Eukaryota	Metazoa	Branchiostoma floridae	XP_002612162.1	3 E-70	535/533
Eukaryota	Metazoa	Trichoplax adhaerens	XP_002108494.1	3 E-70	505/533
Eukaryota	Fungi	Talaromyces stipitatus ATCC 10500	XP_002482472.1	4 E-70	546/533
Eukaryota	Viridiplantae	Picea sitchensis	ABR18186.1	4 E-70	512/533

Eukaryota	Metazoa	Nematostella vectensis	XP_001638857.1	9 E-70	491/533
Eukaryota	Viridiplantae	Populus trichocarpa	XP_002311981.1	1 E-69	484/533
Eukaryota	Fungi	Chaetomium globosum CBS 148.51	XP_001219930.1	1 E-69	532/533
Eukaryota	Metazoa	Trichoplax adhaerens	XP_002107769.1	1 E-69	498/533
Eukaryota	Metazoa	Trichoplax adhaerens	XP_002114490.1	3 E-69	502/533
Eukaryota	Fungi	Aspergillus flavus NRRL3357	XP_002382394.1	3 E-69	504/533
Eukaryota	Fungi	Ajellomyces dermatitidis SLH14081	XP_002623321.1	5 E-69	519/533
Eukaryota	Fungi	Paracoccidioides brasiliensis Pb03;	EEH23467.1	6 E-69	501/533
Eukaryota	Viridiplantae	Populus trichocarpa	XP_002315339.1	6 E-69	486/533
Eukaryota	Fungi	Pyrenophora tritici-repentis Pt-1C-BFP	XP_001932297.1	9 E-69	520/533
Eukaryota	Fungi	Ajellomyces capsulatus G186AR	EEH03610.1	4 E-68	520/533
Eukaryota	Metazoa	Nematostella vectensis	XP_001625358.1	5 E-68	461/533
Eukaryota	Viridiplantae	Picea sitchensis	ABR17274.1	6 E-68	512/533
Eukaryota	Metazoa	Branchiostoma floridae	XP_002610165.1	7 E-68	503/533
Eukaryota	Viridiplantae	Ricinus communis	XP_002523698.1	8 E-68	505/533
Eukaryota	Fungi	Ajellomyces capsulatus H143	EER37458.1	8 E-68	520/533
Eukaryota	Fungi	Chaetomium globosum CBS 148.51	XP_001220786.1	9 E-68	503/533
Eukaryota	Fungi	Coccidioides posadasii C735 delta	EER26683.1	1 E-67	503/533
Eukaryota	Fungi	Coccidioides posadasii C735 delta	EER25935.1	2 E-67	537/533
Eukaryota	Fungi	Coccidioides immitis RS;	XP_001246761.1	2 E-67	451/533
Eukaryota	Fungi	Coccidioides immitis RS;	XP_001240389.1	2 E-67	537/533
Bacteria	Actinobacteria	Geodermatophilus obscurus DSM 43160	ZP_03888392.1	2 E-67	488/533
Eukaryota	Fungi	Coccidioides posadasii C735 delta	EER24333.1	3 E-67	475/533
Eukaryota	Fungi	Botryotinia fuckeliana B05.10	XP_001556516.1	4 E-67	521/533
Eukaryota	Fungi	Gibberella zeae PH-1	XP_383049.1	4 E-67	516/533
Eukaryota	Fungi	Nectria haematococca mpVI 77-13-4	EEU43201.1	6 E-67	524/533
Eukaryota	Fungi	Penicillium marneffei ATCC 18224	XP_002150740.1	8 E-67	497/533
Eukaryota	Fungi	Coccidioides immitis RS;	XP_001243909.1	1 E-66	503/533
Eukaryota	Fungi	Nectria haematococca mpVI 77-13-4	EEU47537.1	3 E-66	504/533
Bacteria	Cyanobacteria	Nostoc punctiforme PCC 73102	YP_001869555.1	3 E-66	483/533
Eukaryota	Fungi	Pyrenophora tritici-repentis Pt-1C-BFP	XP_001932329.1	3 E-66	503/533
Eukaryota	Fungi	Talaromyces stipitatus ATCC 10500	XP_002478646.1	3 E-66	525/533
Eukaryota	Fungi	Ajellomyces capsulatus NAm1	XP_001544216.1	5 E-66	535/533
Eukaryota	Fungi	Laccaria bicolor S238N-H82	XP_001880147.1	5 E-66	559/533
Eukaryota	Fungi	Ajellomyces capsulatus G186AR	EEH04398.1	6 E-66	537/533
Eukaryota	Viridiplantae	Ricinus communis	XP_002532625.1	9 E-66	501/533
Bacteria	Proteobacteria	Ruegeria pomeroyi DSS-3	YP_166054.1	9 E-66	502/533
Eukaryota	Fungi	Ustilago maydis 521	XP_757318.1	9 E-66	526/533
Bacteria	Proteobacteria	Roseobacter denitrificans OCh 114	YP_682165.1	2 E-65	503/533
Eukaryota	Fungi	Magnaporthe grisea 70-15	XP_362103.1	2 E-65	526/533
Eukaryota	Metazoa	Trichoplax adhaerens	XP_002112308.1	2 E-65	481/533
Eukaryota	Fungi	Coprinopsis cinerea okayama7#130	XP_001836025.1	3 E-65	487/533
Eukaryota	Metazoa	Apis mellifera	XP_394579.2	4 E-65	483/533
Eukaryota	Fungi	Coprinopsis cinerea okayama7#130	XP_001830320.1	5 E-65	550/533
Eukaryota	Fungi	Aspergillus terreus NIH2624	XP_001209307.1	6 E-65	490/533
Eukaryota	Fungi	Penicillium chrysogenum Wisconsin 54-1255	XP_002565410.1	6 E-65	485/533
Eukaryota	Metazoa	Trichoplax adhaerens	XP_002115672.1	8 E-65	501/533
Eukaryota	Fungi	Neurospora crassa OR74A	XP_964221.1	9 E-65	489/533
Eukaryota	Viridiplantae	Vitis vinifera	XP_002276353.1	9 E-65	502/533
Eukaryota	Fungi	Uncinocarpus reesii 1704	XP_002544135.1	9 E-65	481/533
Eukaryota	Fungi	Podospora anserina DSM 980	XP_001910650.1	1 E-64	459/533
Eukaryota	Viridiplantae	Vitis vinifera	XP_002274994.1	1 E-64	501/533
Eukaryota	Metazoa	Trichoplax adhaerens	XP_002114172.1	1 E-64	516/533
Eukaryota	Metazoa	Nematostella vectensis	XP_001638455.1	2 E-64	488/533
Eukaryota	Metazoa	Ciona intestinalis	XP_002121433.1	2 E-64	480/533
Eukaryota	Fungi	Ajellomyces capsulatus NAm1	XP_001538766.1	2 E-64	520/533
Eukaryota	Metazoa	Trichoplax adhaerens	XP_002114173.1	2 E-64	511/533
Eukaryota	Metazoa	Nasonia vitripennis	XP_001604903.1	3 E-64	478/533

Eukaryota	Fungi	Aspergillus oryzae RIB40	XP_001823173.1	3 E-64	496/533
Eukaryota	Metazoa	Tribolium castaneum	XP_973874.2	4 E-64	469/533
Bacteria	Proteobacteria	Dinoroseobacter shibae DFL 12	YP_001531603.1	4 E-64	488/533
Bacteria	Actinobacteria	Streptomyces ghanaensis ATCC 14672	ZP_04687548.1	5 E-64	485/533
Eukaryota	Metazoa	Drosophila simulans	XP_002106957.1	5 E-64	496/533
Eukaryota	Viridiplantae	Populus trichocarpa	XP_002300662.1	6 E-64	485/533
Eukaryota	Metazoa	Ciona intestinalis	XP_002127963.1	7 E-64	474/533
Eukaryota	Fungi	Aspergillus fumigatus Af293	XP_755349.1	7 E-64	534/533
Eukaryota	Metazoa	Pediculus humanus corporis	XP_002429109.1	8 E-64	486/533
Bacteria	Bacteroidetes	Rhodothermus marinus DSM 4252	ZP_04424017.1	9 E-64	478/533
Eukaryota	Fungi	Aspergillus niger CBS 513.88	XP_001397791.1	1 E-63	529/533
Eukaryota	Metazoa	Nasonia vitripennis	XP_001604694.1	1 E-63	518/533
Eukaryota	Fungi	Aspergillus fumigatus A1163	EDP54539.1	1 E-63	534/533
Eukaryota	Fungi	Ajellomyces dermatitidis SLH14081	XP_002621797.1	1 E-63	513/533
Eukaryota	Metazoa	Drosophila ananassae	XP_001966071.1	1 E-63	465/533
Eukaryota	Fungi	Ajellomyces dermatitidis SLH14081	XP_002623346.1	2 E-63	482/533
Eukaryota	Fungi	Ajellomyces dermatitidis ER-3	EEQ91892.1	2 E-63	513/533
Eukaryota	Metazoa	Drosophila erecta	XP_001978137.1	2 E-63	493/533
Eukaryota	Fungi	Gibberella zeae PH-1	XP_389019.1	2 E-63	498/533
Eukaryota	Viridiplantae	Populus trichocarpa	XP_002305282.1	2 E-63	506/533
Bacteria	Actinobacteria	Streptomyces coelicolor A3(2)	NP_628552.1	2 E-63	480/533
Bacteria	Proteobacteria	Roseobacter litoralis Och 149	ZP_02142464.1	2 E-63	489/533
Eukaryota	Fungi	Paracoccidioides brasiliensis Pb18;	EEH42456.1	2 E-63	453/533
Eukaryota	Viridiplantae	Ricinus communis	XP_002520028.1	2 E-63	509/533
Eukaryota	Viridiplantae	Populus trichocarpa	XP_002329323.1	2 E-63	506/533
Eukaryota	Fungi	Aspergillus fumigatus Af293	XP_753893.2	2 E-63	509/533
Eukaryota	Viridiplantae	Ricinus communis	XP_002519487.1	3 E-63	474/533
Eukaryota	Viridiplantae	Paulownia fortunei	ACL31667.1	4 E-63	473/533
Eukaryota	Fungi	Cryptococcus neoformans var. neoformans	XP_568637.1	4 E-63	512/533
Eukaryota	Amoebozoa	Dictyostelium discoideum AX4	XP_638380.1	5 E-63	486/533
Eukaryota	Fungi	Coccidioides immitis RS;	XP_001242526.1	5 E-63	481/533
Eukaryota	Fungi	Paracoccidioides brasiliensis Pb03;	EEH19618.1	5 E-63	510/533
Eukaryota	Viridiplantae	Oryza sativa Indica Group	EAY87168.1	6 E-63	497/533
Eukaryota	Metazoa	Trichoplax adhaerens	XP_002112310.1	6 E-63	471/533
Eukaryota	Fungi	Aspergillus nidulans FGSC A4	CBF88756.1	8 E-63	534/533
Eukaryota	Fungi	Paracoccidioides brasiliensis Pb18;	EEH46276.1	8 E-63	510/533
Eukaryota	Metazoa	Drosophila melanogaster	NP_572988.1	8 E-63	493/533
Bacteria	Actinobacteria	Streptomyces scabiei 87.22	CBG72172.1	8 E-63	480/533
Eukaryota	Viridiplantae	Physcomitrella patens subsp. magdalenae	ABY21313.1	9 E-63	468/533
Eukaryota	Viridiplantae	Arabidopsis thaliana	NP_192425.1	9 E-63	512/533
Eukaryota	Viridiplantae	Populus trichocarpa	XP_002297838.1	9 E-63	484/533
Eukaryota	Metazoa	Luciola cruciata	BAE80729.1	9 E-63	489/533
Eukaryota	Metazoa	Suberites domuncula	CAR31336.1	9 E-63	488/533
Eukaryota	Fungi	Paracoccidioides brasiliensis Pb18;	EEH46636.1	1 E-62	469/533
Eukaryota	Metazoa	Drosophila grimshawi	XP_001991632.1	1 E-62	465/533
Bacteria	Actinobacteria	Streptomyces lividans TK24	ZP_05524585.1	1 E-62	480/533
Eukaryota	Fungi	Aspergillus terreus NIH2624	XP_001213629.1	1 E-62	522/533
Eukaryota	Fungi	Uncinocarpus reesii 1704	XP_002541043.1	1 E-62	438/533
Eukaryota	Viridiplantae	Vitis vinifera	XP_002271586.1	1 E-62	488/533
Eukaryota	Viridiplantae	Oryza sativa Japonica Group	NP_001047819.1	2 E-62	497/533
Eukaryota	Viridiplantae	Glycine max	AAC97389.1	2 E-62	499/533
Eukaryota	Fungi	Neosartorya fischeri NRRL 181	XP_001259941.1	2 E-62	495/533
Eukaryota	Viridiplantae	Populus tremuloides	AAC24504.1	2 E-62	509/533
Eukaryota	Fungi	Aspergillus terreus NIH2624	XP_001218379.1	2 E-62	484/533
Eukaryota	Fungi	Coccidioides posadasii C735 delta	EER27585.1	3 E-62	487/533
Eukaryota	Viridiplantae	Arabidopsis thaliana	AAP03017.1	3 E-62	484/533
Eukaryota	Metazoa	Culex quinquefasciatus	XP_001845435.1	3 E-62	472/533
Eukaryota	Fungi	Neosartorya fischeri NRRL 181	XP_001260500.1	4 E-62	534/533

Eukaryota	Fungi	Paracoccidioides brasiliensis Pb18;	EEH49905.1	4 E-62	461/533
Eukaryota	Viridiplantae	Arabidopsis thaliana	AAP03016.1	4 E-62	510/533
Eukaryota	Viridiplantae	Physcomitrella patens subsp. patens	ABV60448.1	4 E-62	468/533
Eukaryota	Metazoa	Culex quinquefasciatus	XP_001857251.1	4 E-62	483/533
Eukaryota	Viridiplantae	Physcomitrella patens subsp. patens	XP_001755943.1	4 E-62	468/533
Eukaryota	Viridiplantae	Oryza sativa Japonica Group	Q7XXL2.24CLL9_	5 E-62	506/533
Eukaryota	Viridiplantae	Arabidopsis thaliana	NP_173472.1	5 E-62	510/533
Eukaryota	Fungi	Uncinocarpus reesii 1704	XP_002544469.1	6 E-62	491/533
Eukaryota	Viridiplantae	Ruta graveolens	ABY81910.1	6 E-62	490/533
Eukaryota	Viridiplantae	Arabidopsis thaliana	AAF79612.1AC02	6 E-62	474/533
Eukaryota	Viridiplantae	Arabidopsis thaliana	AAF79612.1AC02	2 E-55	453/533
Eukaryota	Viridiplantae	Arabidopsis thaliana	AAP03021.1	7 E-62	501/533
Eukaryota	Viridiplantae	Arabidopsis thaliana	NP_564115.1	7 E-62	501/533
Eukaryota	Viridiplantae	Arabidopsis thaliana	NP_193636.1	9 E-62	484/533
Eukaryota	Fungi	Ustilago maydis 521	XP_760626.1	1 E-61	493/533
Eukaryota	Metazoa	Drosophila sechellia	XP_002043794.1	1 E-61	493/533
Eukaryota	Fungi	Aspergillus clavatus NRRL 1	XP_001274067.1	1 E-61	495/533
Eukaryota	Viridiplantae	Physcomitrella patens subsp. californica	ABY21309.1	1 E-61	468/533
Eukaryota	Metazoa	Nematostella vectensis	XP_001623060.1	2 E-61	461/533
Eukaryota	Viridiplantae	Ricinus communis	XP_002510640.1	2 E-61	505/533
Bacteria	Actinobacteria	Rhodococcus jostii RHA1	YP_705267.1	2 E-61	495/533
Eukaryota	Fungi	Ajellomyces capsulatus H143	EER39157.1	2 E-61	496/533
Eukaryota	Fungi	Phaeosphaeria nodorum SN15	XP_001793248.1	2 E-61	514/533
Eukaryota	Viridiplantae	Arabidopsis thaliana	BAC42032.1	2 E-61	484/533

AFUA_5G14840

Eukaryota	Fungi	Aspergillus fumigatus Af293	XP_753173.2	1 E-130	226/226
Eukaryota	Fungi	Aspergillus terreus NIH2624	XP_001210816.1	1 E-75	224/226
Eukaryota	Fungi	Aspergillus niger CBS 513.88	XP_001397070.1	2 E-75	224/226
Eukaryota	Fungi	Aspergillus nidulans FGSC A4	CBF74648.1	5 E-72	227/226
Eukaryota	Fungi	Neosartorya fischeri NRRL 181	XP_001259212.1	8 E-72	220/226
Eukaryota	Fungi	Aspergillus fumigatus Af293	XP_753189.1	1 E-71	220/226
Eukaryota	Fungi	Aspergillus fumigatus A1163	EDP52196.1	4 E-71	220/226
Eukaryota	Fungi	Aspergillus clavatus NRRL 1	XP_001274721.1	8 E-70	217/226
Eukaryota	Fungi	Talaromyces stipitatus ATCC 10500	XP_002478828.1	5 E-54	206/226
Eukaryota	Fungi	Aspergillus nidulans FGSC A4	XP_661731.1	2 E-53	214/226
Eukaryota	Fungi	Penicillium marneffei ATCC 18224	XP_002145675.1	7 E-50	217/226
Eukaryota	Fungi	Talaromyces stipitatus ATCC 10500	XP_002477879.1	2 E-46	207/226
Eukaryota	Fungi	Nectria haematococca mpVI 77-13-4	EEU33580.1	1 E-17	200/226
Eukaryota	Fungi	Gibberella zeae PH-1	XP_390618.1	9 E-17	201/226
Eukaryota	Fungi	Chaetomium globosum CBS 148.51	XP_001228673.1	4 E-15	222/226
Eukaryota	Fungi	Gibberella zeae PH-1	XP_387967.1	5 E-14	201/226

AFUA_6G00440

Eukaryota	Fungi	Aspergillus fumigatus Af293	XP_731511.1	0.0	538/538
Eukaryota	Fungi	Neosartorya fischeri NRRL 181	XP_001257320.1	0.0	515/538
Eukaryota	Fungi	Aspergillus clavatus NRRL 1	XP_001270414.1	0.0	511/538
Eukaryota	Fungi	Aspergillus terreus NIH2624	XP_001213348.1	0.0	503/538
Eukaryota	Fungi	Aspergillus flavus NRRL3357	XP_002374548.1	1 E-165	522/538
Eukaryota	Fungi	Aspergillus nidulans FGSC A4	XP_662653.1	1 E-159	494/538
Eukaryota	Fungi	Sclerotinia sclerotiorum 1980 UF-70	XP_001594869.1	1 E-148	443/538
Eukaryota	Fungi	Pyrenophora tritici-repentis Pt-1C-BFP	XP_001931435.1	1 E-139	575/538
Eukaryota	Fungi	Podospora anserina DSM 980	XP_001905108.1	1 E-130	442/538
Eukaryota	Fungi	Talaromyces stipitatus ATCC 10500	XP_002486315.1	1 E-129	492/538
Eukaryota	Fungi	Penicillium marneffei ATCC 18224	XP_002152616.1	1 E-129	491/538
Eukaryota	Fungi	Neurospora crassa OR74A	XP_963778.1	1 E-125	510/538

Eukaryota	Fungi	Magnaporthe grisea 70-15	XP_362178.1	1 E-119	531/538
Eukaryota	Fungi	Nectria haematococca mpVI 77-13-4	EEU47732.1	1 E-118	472/538
Eukaryota	Fungi	Gibberella zeae PH-1	XP_381338.1	1 E-115	477/538
Eukaryota	Fungi	Phaeosphaeria nodorum SN15	XP_001799581.1	1 E-115	522/538
Eukaryota	Fungi	Verticillium albo-atrum VaMs.102	EEY19033.1	8 E-98	483/538
Eukaryota	Fungi	Pichia stipitis CBS 6054	XP_001385911.1	7 E-82	433/538
Eukaryota	Fungi	Candida dubliniensis CD36	XP_002422147.1	4 E-77	438/538
Eukaryota	Fungi	Candida albicans WO-1	EEQ43689.1	4 E-77	438/538
Eukaryota	Fungi	Candida albicans SC5314	XP_713540.1	5 E-77	438/538
Eukaryota	Fungi	Debaryomyces hansenii	CAR66338.1	5 E-77	439/538
Eukaryota	Fungi	Pichia guilliermondii ATCC 6260	EDK36837.2	4 E-75	433/538
Eukaryota	Fungi	Pichia guilliermondii ATCC 6260	XP_001487558.1	1 E-74	433/538
Eukaryota	Fungi	Candida tropicalis MYA-3404	XP_002545850.1	1 E-74	459/538
Eukaryota	Fungi	Clavispora lusitaniae ATCC 42720	XP_002619984.1	1 E-72	431/538
Eukaryota	Metazoa	Caenorhabditis elegans	NP_001024066.1	8 E-40	445/538
Eukaryota	Metazoa	Caenorhabditis briggsae AF16	XP_001672608.1	2 E-35	457/538

AFUA_6G00450

Eukaryota	Fungi	Aspergillus fumigatus Af293	XP_731510.1	0.0	528/528
Eukaryota	Fungi	Neosartorya fischeri NRRL 181	XP_001257321.1	0.0	525/528
Eukaryota	Fungi	Aspergillus oryzae RIB40	XP_001818628.1	0.0	506/528
Eukaryota	Fungi	Penicillium marneffei ATCC 18224	XP_002149574.1	0.0	544/528
Eukaryota	Fungi	Aspergillus fumigatus Af293	XP_750792.1	0.0	509/528
Eukaryota	Fungi	Aspergillus fumigatus A1163	EDP49472.1	0.0	509/528
Eukaryota	Fungi	Penicillium chrysogenum Wisconsin 54-1255	XP_002557661.1	0.0	522/528
Eukaryota	Fungi	Neosartorya fischeri NRRL 181	XP_001258021.1	0.0	505/528
Eukaryota	Fungi	Aspergillus clavatus NRRL 1	XP_001268067.1	0.0	505/528
Eukaryota	Fungi	Aspergillus oryzae RIB40	XP_001819091.1	1 E-174	504/528
Eukaryota	Fungi	Aspergillus flavus NRRL3357	XP_002382080.1	1 E-173	504/528
Eukaryota	Fungi	Aspergillus niger CBS 513.88	XP_001396085.1	1 E-170	515/528
Eukaryota	Fungi	Aspergillus niger	Q8WZI8.1	1 E-167	500/528
Eukaryota	Fungi	Talaromyces stipitatus ATCC 10500	XP_002340484.1	1 E-164	523/528
Eukaryota	Fungi	Aspergillus terreus NIH2624	XP_001211390.1	1 E-164	521/528
Eukaryota	Fungi	Aspergillus nidulans FGSC A4	XP_659376.1	1 E-160	525/528
Eukaryota	Fungi	Aspergillus terreus NIH2624	XP_001215841.1	1 E-155	470/528
Eukaryota	Fungi	Nectria haematococca mpVI 77-13-4	EEU37962.1	1 E-152	520/528
Eukaryota	Fungi	Phaeosphaeria nodorum SN15	XP_001801563.1	1 E-143	510/528
Eukaryota	Fungi	Gibberella zeae PH-1	XP_383127.1	1 E-139	522/528
Eukaryota	Fungi	Pyrenophora tritici-repentis Pt-1C-BFP	XP_001934990.1	1 E-139	528/528
Eukaryota	Fungi	Magnaporthe grisea 70-15	XP_363153.1	1 E-136	525/528
Eukaryota	Fungi	Nectria haematococca mpVI 77-13-4	EEU35594.1	1 E-134	507/528
Eukaryota	Fungi	Verticillium albo-atrum VaMs.102	EEY23366.1	1 E-134	473/528
Eukaryota	Fungi	Magnaporthe grisea 70-15	XP_361297.1	1 E-131	520/528
Eukaryota	Fungi	Aspergillus oryzae RIB40	XP_001822262.1	1 E-129	518/528
Eukaryota	Fungi	Nectria haematococca mpVI 77-13-4	EEU35764.1	1 E-126	513/528
Eukaryota	Fungi	Aspergillus flavus NRRL3357	XP_002382345.1	1 E-124	503/528
Eukaryota	Fungi	Nectria haematococca mpVI 77-13-4	EEU43324.1	1 E-123	516/528
Eukaryota	Fungi	Aspergillus terreus NIH2624	XP_001211593.1	1 E-116	505/528
Eukaryota	Fungi	Nectria haematococca mpVI 77-13-4	EEU41741.1	1 E-112	523/528
Eukaryota	Fungi	Pyrenophora tritici-repentis Pt-1C-BFP	XP_001930528.1	1 E-110	475/528
Eukaryota	Fungi	Magnaporthe grisea 70-15	XP_364522.1	1 E-110	504/528
Eukaryota	Fungi	Verticillium albo-atrum VaMs.102	EEY19048.1	2 E-98	530/528
Eukaryota	Fungi	Gibberella zeae PH-1	XP_382309.1	1 E-96	498/528
Eukaryota	Fungi	Pyrenophora tritici-repentis Pt-1C-BFP	XP_001936734.1	5 E-84	506/528
Eukaryota	Fungi	Magnaporthe grisea 70-15	XP_360155.1	1 E-82	557/528
Eukaryota	Fungi	Phaeosphaeria nodorum SN15	XP_001790831.1	2 E-75	475/528
Eukaryota	Fungi	Gibberella zeae PH-1	XP_383163.1	4 E-74	505/528

Eukaryota	Fungi	Aspergillus niger CBS 513.88	XP_001401646.1	6 E-74	538/528
Eukaryota	Fungi	Aspergillus flavus NRRL3357	XP_002384763.1	2 E-71	476/528
Eukaryota	Fungi	Pyrenophora tritici-repentis Pt-1C-BFP	XP_001941383.1	1 E-68	481/528
Eukaryota	Fungi	Aspergillus niger CBS 513.88	XP_001393845.1	2 E-68	460/528
Eukaryota	Fungi	Aspergillus oryzae RIB40	XP_001827546.1	1 E-67	457/528
Eukaryota	Fungi	Botryotinia fuckeliana B05.10	XP_001560611.1	5 E-67	519/528
Eukaryota	Fungi	Phaeosphaeria nodorum SN15	XP_001804005.1	1 E-65	469/528
Eukaryota	Fungi	Aspergillus niger CBS 513.88	XP_001397058.1	8 E-64	485/528
Eukaryota	Fungi	Magnaporthe grisea 70-15	XP_360959.1	6 E-63	468/528
Eukaryota	Fungi	Postia placenta Mad-698-R	XP_002472645.1	5 E-59	469/528
Eukaryota	Fungi	Neurospora crassa OR74A	XP_958973.1	6 E-59	458/528
Eukaryota	Fungi	Postia placenta Mad-698-R	XP_002477224.1	3 E-58	464/528
Eukaryota	Fungi	Aspergillus niger CBS 513.88	XP_001392929.1	9 E-58	533/528
Eukaryota	Fungi	Magnaporthe grisea 70-15	XP_359991.1	1 E-57	555/528
Eukaryota	Fungi	Chaetomium globosum CBS 148.51	XP_001230230.1	1 E-55	512/528
Eukaryota	Fungi	Botryotinia fuckeliana B05.10	XP_001551789.1	1 E-53	508/528
Eukaryota	Fungi	Aspergillus terreus NIH2624	XP_001217735.1	2 E-52	501/528
Eukaryota	Fungi	Podospora anserina DSM 980	XP_001904816.1	2 E-51	486/528
Bacteria	Proteobacteria	Burkholderia sp. H160	ZP_03263997.1	1 E-50	445/528
Bacteria	Proteobacteria	Burkholderia ubonensis Bu	ZP_02376789.1	6 E-50	526/528
Eukaryota	Fungi	Aspergillus niger CBS 513.88	XP_001389907.1	9 E-50	511/528
Eukaryota	Fungi	Aspergillus niger CBS 513.88	XP_001398581.1	1 E-47	497/528
Bacteria	Acidobacteria	Candidatus Solibacter usitatus Ellin6076	YP_825464.1	3 E-47	436/528
Bacteria	Proteobacteria	Burkholderia multivorans CGD1	ZP_03582488.1	5 E-47	485/528
Eukaryota	Fungi	Magnaporthe grisea 70-15	XP_365559.2	2 E-46	455/528
Eukaryota	Fungi	Nectria haematococca mpVI 77-13-4	EEU33441.1	3 E-46	479/528
Bacteria	Proteobacteria	Acetobacter pasteurianus IFO 3283-01	YP_003188040.1	2 E-45	434/528
Eukaryota	Fungi	Aspergillus niger CBS 513.88	XP_001396750.1	5 E-45	428/528
Eukaryota	Fungi	Verticillium albo-atrum VaMs.102	EEY17219.1	1 E-44	483/528
Bacteria	Proteobacteria	Agrobacterium radiobacter K84	YP_002541515.1	2 E-44	440/528
Bacteria	Proteobacteria	Burkholderia sp. 383	YP_367255.1	2 E-44	431/528
Bacteria	Proteobacteria	Caulobacter sp. K31	YP_001682224.1	3 E-44	512/528
Bacteria	Proteobacteria	Burkholderia multivorans CGD2	ZP_03578248.1	8 E-44	432/528
Bacteria	Proteobacteria	Burkholderia multivorans ATCC 17616	YP_001585986.1	9 E-44	432/528
Eukaryota	Fungi	Phaeosphaeria nodorum SN15	XP_001802169.1	9 E-44	503/528
Bacteria	Proteobacteria	Burkholderia multivorans CGD2M	ZP_03572017.1	1 E-43	432/528
Bacteria	Proteobacteria	Burkholderia multivorans CGD1	ZP_03585074.1	2 E-43	432/528
Eukaryota	Fungi	Nectria haematococca mpVI 77-13-4	EEU41317.1	3 E-43	461/528
Eukaryota	Fungi	Aspergillus flavus NRRL3357	XP_002376896.1	2 E-42	455/528
Eukaryota	Fungi	Verticillium albo-atrum VaMs.102	EEY21819.1	2 E-42	463/528
Eukaryota	Fungi	Aspergillus oryzae RIB40	XP_001821143.1	2 E-42	455/528
Bacteria	Proteobacteria	Xanthomonas campestris pv. campestris	Q8P8Y5.1	6 E-42	517/528
Bacteria	Proteobacteria	Xanthomonas campestris pv. campestris	NP_637458.2	7 E-42	517/528
Eukaryota	Fungi	Sclerotinia sclerotiorum 1980 UF-70	XP_001589546.1	3 E-41	459/528
Eukaryota	Fungi	Nectria haematococca mpVI 77-13-4	EEU36370.1	4 E-41	426/528
Bacteria	Actinobacteria	Streptomyces sp. SPB78	ZP_05485377.1	1 E-40	470/528
Eukaryota	Fungi	Talaromyces stipitatus ATCC 10500	XP_002341414.1	2 E-40	496/528
Eukaryota	Fungi	Microsporum canis CBS 113480	EEQ34025.1	8 E-40	521/528
Eukaryota	Fungi	Gibberella zeae PH-1	XP_391535.1	9 E-40	446/528
Eukaryota	Fungi	Penicillium marneffei ATCC 18224	XP_002151622.1	1 E-39	518/528
Eukaryota	Fungi	Aspergillus flavus NRRL3357	XP_002376356.1	1 E-39	535/528
Bacteria	Proteobacteria	Teredinibacter turnerae T7901	YP_003072832.1	2 E-39	438/528
Bacteria	Proteobacteria	Burkholderia phymatum STM815	YP_001856368.1	2 E-39	522/528
Eukaryota	Fungi	Nectria haematococca mpVI 77-13-4	EEU43572.1	2 E-39	482/528
Bacteria	Proteobacteria	Burkholderia ubonensis Bu	ZP_02381052.1	5 E-39	538/528
Bacteria	Proteobacteria	Ralstonia eutropha JMP134	YP_299065.1	7 E-39	539/528
Eukaryota	Fungi	Gibberella zeae PH-1	XP_391738.1	7 E-39	465/528
Eukaryota	Fungi	Aspergillus oryzae RIB40	XP_001820636.1	1 E-38	535/528

Bacteria	Proteobacteria	Burkholderia glumae BGR1	YP_002908690.1	2 E-38	536/528
Eukaryota	Fungi	Botryotinia fuckeliana B05.10	XP_001560161.1	3 E-38	455/528
Eukaryota	Fungi	Podospora anserina DSM 980	XP_001905213.1	3 E-38	512/528
Eukaryota	Fungi	Magnaporthe grisea 70-15	XP_360218.2	5 E-38	452/528
Bacteria	Proteobacteria	Burkholderia sp. H160	ZP_03270179.1	6 E-38	522/528
Eukaryota	Fungi	Aspergillus niger CBS 513.88	XP_001399173.1	8 E-38	556/528
Bacteria	Proteobacteria	Burkholderia sp. 383	YP_370816.1	9 E-38	544/528
Bacteria	Proteobacteria	Klebsiella pneumoniae 342	YP_002240994.1	9 E-38	461/528
Bacteria	Proteobacteria	Burkholderia ambifaria MC40-6	YP_001812119.1	1 E-37	546/528
Eukaryota	Fungi	Aspergillus flavus NRRL3357	XP_002379564.1	2 E-37	454/528
Bacteria	Proteobacteria	Klebsiella pneumoniae NTUH-K2044	YP_002917272.1	2 E-37	454/528
Eukaryota	Fungi	Aspergillus nidulans FGSC A4	XP_661644.1	2 E-37	461/528
Eukaryota	Fungi	Aspergillus fumigatus Af293	XP_748245.1	3 E-37	455/528
Bacteria	Proteobacteria	Burkholderia ambifaria IOP40-10	ZP_02888928.1	6 E-37	546/528
Bacteria	Proteobacteria	Pseudomonas syringae pv. tomato	ZP_03398785.1	1 E-36	447/528
Bacteria	Proteobacteria	Burkholderia cenocepacia AU 1054	YP_625075.1	1 E-36	468/528
Bacteria	Proteobacteria	Burkholderia phytofirmans PsJN	YP_001887774.1	3 E-36	543/528
Bacteria	Proteobacteria	Burkholderia pseudomallei 305	ZP_01765732.1	3 E-36	456/528
Bacteria	Proteobacteria	Burkholderia cenocepacia MC0-3	YP_001778227.1	4 E-36	538/528
Eukaryota	Fungi	Nectria haematococca mpVI 77-13-4	EEU35373.1	4 E-36	503/528
Bacteria	Proteobacteria	Burkholderia pseudomallei 1106a	YP_001075108.1	5 E-36	456/528
Bacteria	Proteobacteria	Burkholderia pseudomallei 406e	ZP_04967554.1	5 E-36	456/528
Bacteria	Proteobacteria	Burkholderia pseudomallei S13	ZP_04899247.1	5 E-36	456/528
Bacteria	Proteobacteria	Variovorax paradoxus S110	YP_002947343.1	6 E-36	454/528
Eukaryota	Fungi	Pyrenophora tritici-repentis Pt-1C-BFP	XP_001932100.1	6 E-36	444/528
Bacteria	Proteobacteria	Burkholderia pseudomallei 576	ZP_03450548.1	7 E-36	456/528
Bacteria	Proteobacteria	Burkholderia pseudomallei MSHR346	ZP_04523013.1	7 E-36	456/528
Bacteria	Proteobacteria	Burkholderia pseudomallei 1710a	ZP_04956051.1	7 E-36	456/528
Bacteria	Proteobacteria	Burkholderia ambifaria AMMD	YP_776794.1	8 E-36	546/528
Bacteria	Proteobacteria	Burkholderia pseudomallei Pasteur 52237	ZP_04896346.1	8 E-36	456/528
Bacteria	Proteobacteria	Burkholderia pseudomallei BCC215	ZP_02509324.1	9 E-36	456/528
Bacteria	Proteobacteria	Burkholderia pseudomallei 14	ZP_02414806.1	1 E-35	456/528
Bacteria	Proteobacteria	Burkholderia pseudomallei 7894	ZP_02485064.1	1 E-35	456/528
Bacteria	Proteobacteria	Burkholderia pseudomallei 1655	ZP_04889801.1	1 E-35	456/528
Bacteria	Proteobacteria	Burkholderia pseudomallei 9	ZP_02459063.1	1 E-35	456/528
Bacteria	Proteobacteria	Burkholderia pseudomallei 1710b	YP_337516.1	1 E-35	456/528
Bacteria	Proteobacteria	Burkholderia pseudomallei DM98	ZP_02406289.1	1 E-35	456/528
Bacteria	Proteobacteria	Burkholderia mallei GB8 horse	ZP_00442445.1	1 E-35	456/528
Bacteria	Proteobacteria	Burkholderia pseudomallei 91	ZP_02450897.1	1 E-35	456/528
Bacteria	Proteobacteria	Burkholderia mallei ATCC 23344	YP_105387.1	2 E-35	456/528
Eukaryota	Fungi	Aspergillus niger CBS 513.88	XP_001390411.1	2 E-35	479/528
Bacteria	Proteobacteria	Burkholderia pseudomallei K96243	YP_110793.1	2 E-35	456/528
Bacteria	Proteobacteria	Burkholderia cenocepacia PC184	ZP_04942913.1	2 E-35	528/528
Bacteria	Proteobacteria	Burkholderia sp. H160	ZP_03265733.1	2 E-35	472/528
Eukaryota	Fungi	Magnaporthe grisea 70-15	XP_001521975.1	2 E-35	503/528
Eukaryota	Fungi	Magnaporthe grisea 70-15	XP_364832.2	2 E-35	503/528
Eukaryota	Fungi	Aspergillus niger CBS 513.88	XP_001394358.1	3 E-35	485/528
Eukaryota	Fungi	Aspergillus oryzae RIB40	XP_001821840.1	3 E-35	454/528
Bacteria	Proteobacteria	Burkholderia graminis C4D1M	ZP_02881286.1	4 E-35	524/528
Bacteria	Proteobacteria	Marinomonas sp. MWYL1	YP_001341773.1	4 E-35	466/528
Eukaryota	Fungi	Aspergillus fumigatus Af293	XP_748839.1	8 E-35	534/528
Bacteria	Proteobacteria	Burkholderia vietnamiensis G4	YP_001116296.1	1 E-34	468/528
Bacteria	Proteobacteria	Burkholderia dolosa AUO158	ZP_04948100.1	1 E-34	468/528
Eukaryota	Fungi	Neosartorya fischeri NRRL 181	XP_001261622.1	1 E-34	545/528
Bacteria	Proteobacteria	Burkholderia cenocepacia J2315	YP_002235388.1	1 E-34	538/528
Bacteria	Proteobacteria	Pseudomonas syringae pv. tomato	NP_792779.1	1 E-34	445/528
Bacteria	Proteobacteria	Delftia acidovorans SPH-1	YP_001564554.1	1 E-34	502/528
Eukaryota	Fungi	Gibberella zeae PH-1	XP_383393.1	1 E-34	490/528

Bacteria	Proteobacteria	Shewanella putrefaciens 200	ZP_01704193.1	2 E-34	530/528
Bacteria	Proteobacteria	Burkholderia phytofirmans PsJN	YP_001889750.1	2 E-34	517/528
Bacteria	Actinobacteria	Catenulispora acidiphila DSM 44928	YP_003117801.1	2 E-34	491/528
Bacteria	Fusobacteria	Leptotrichia buccalis DSM 1135	YP_003164505.1	3 E-34	436/528
Bacteria	Proteobacteria	Burkholderia cenocepacia MC0-3	YP_001773752.1	3 E-34	530/528
Eukaryota	Fungi	Aspergillus flavus NRRL3357	XP_002382292.1	3 E-34	454/528
Bacteria	Proteobacteria	Burkholderia ambifaria MEX-5	ZP_02907124.1	4 E-34	546/528
Eukaryota	Fungi	Phaeosphaeria nodorum SN15	XP_001805090.1	4 E-34	461/528
Eukaryota	Fungi	Aspergillus niger CBS 513.88	XP_001393089.1	5 E-34	552/528
Bacteria	Proteobacteria	Burkholderia thailandensis MSMB43	ZP_02467978.1	5 E-34	538/528
Eukaryota	Fungi	Aspergillus oryzae RIB40	XP_001822863.1	8 E-34	454/528
Bacteria	Proteobacteria	Burkholderia graminis C4D1M	ZP_02884950.1	8 E-34	545/528
Bacteria	Proteobacteria	Shewanella putrefaciens CN-32	YP_001184896.1	8 E-34	530/528
Eukaryota	Fungi	Aspergillus flavus NRRL3357	XP_002385227.1	9 E-34	487/528
Bacteria	Proteobacteria	Delftia acidovorans SPH-1	YP_001565552.1	9 E-34	519/528
Bacteria	Fusobacteria	Leptotrichia hofstadii F0254	ZP_05901451.1	1 E-33	436/528
Eukaryota	Fungi	Aspergillus niger CBS 513.88	XP_001389868.1	1 E-33	534/528
Bacteria	Proteobacteria	Ralstonia metallidurans CH34	YP_585978.1	2 E-33	460/528
Bacteria	Proteobacteria	Shewanella sp. W3-18-1	YP_961962.1	2 E-33	530/528
Eukaryota	Fungi	Aspergillus terreus NIH2624	XP_001211829.1	2 E-33	524/528
Eukaryota	Fungi	Chaetomium globosum CBS 148.51	XP_001222975.1	2 E-33	481/528
Eukaryota	Fungi	Botryotinia fuckeliana B05.10	XP_001555592.1	3 E-33	466/528
Bacteria	Proteobacteria	Pantoea sp. At-9b	ZP_05730130.1	3 E-33	430/528
Bacteria	Proteobacteria	Burkholderia xenovorans LB400	YP_553057.1	4 E-33	530/528
Bacteria	Proteobacteria	Burkholderia cenocepacia AU 1054	YP_621180.1	8 E-33	530/528
Bacteria	Proteobacteria	Burkholderia ambifaria MEX-5	ZP_02905140.1	2 E-32	464/528
Bacteria	Proteobacteria	Burkholderia ambifaria AMMD	YP_777907.1	3 E-32	464/528
Eukaryota	Fungi	Aspergillus niger CBS 513.88	XP_001401809.1	3 E-32	475/528
Bacteria	Proteobacteria	Burkholderia graminis C4D1M	ZP_02883781.1	4 E-32	520/528
Eukaryota	Fungi	Nectria haematococca mpVI 77-13-4	EEU43622.1	4 E-32	445/528
Bacteria	Proteobacteria	Shewanella baltica OS185	YP_001365371.1	5 E-32	466/528
Bacteria	Proteobacteria	Bradyrhizobium japonicum USDA 110	NP_774602.1	5 E-32	486/528
Bacteria	Proteobacteria	Burkholderia graminis C4D1M	ZP_02883355.1	6 E-32	480/528
Eukaryota	Fungi	Botryotinia fuckeliana B05.10	XP_001560056.1	8 E-32	479/528
Bacteria	Proteobacteria	Burkholderia multivorans ATCC 17616	YP_001583195.1	1 E-31	546/528
Bacteria	Proteobacteria	Agrobacterium radiobacter K84	YP_002544289.1	2 E-31	459/528
Bacteria	Proteobacteria	Shewanella frigidimarina NCIMB 400	YP_752362.1	2 E-31	465/528
Bacteria	Proteobacteria	Burkholderia multivorans CGD1	ZP_03583425.1	2 E-31	546/528
Eukaryota	Fungi	Botryotinia fuckeliana B05.10	XP_001547571.1	3 E-31	505/528
Eukaryota	Fungi	Aspergillus niger CBS 513.88	XP_001402482.1	3 E-31	475/528
Bacteria	Proteobacteria	Burkholderia multivorans CGD2M	ZP_03573639.1	3 E-31	546/528
Bacteria	Proteobacteria	Burkholderia ambifaria IOP40-10	ZP_02888495.1	3 E-31	464/528
Eukaryota	Fungi	Chaetomium globosum CBS 148.51	XP_001224411.1	5 E-31	501/528
Eukaryota	Fungi	Chaetomium globosum CBS 148.51	XP_001228631.1	5 E-31	466/528
Bacteria	Proteobacteria	Burkholderia ambifaria MC40-6	YP_001815781.1	6 E-31	464/528
Eukaryota	Fungi	Aspergillus niger CBS 513.88	XP_001398312.1	9 E-31	493/528
Bacteria	Firmicutes	Clostridium carboxidivorans P7	ZP_05394636.1	1 E-30	439/528
Eukaryota	Fungi	Aspergillus nidulans FGSC A4	XP_680482.1	1 E-30	477/528
Eukaryota	Fungi	Aspergillus oryzae RIB40	XP_001826830.1	2 E-30	474/528
Eukaryota	Fungi	Sclerotinia sclerotiorum 1980 UF-70	XP_001594065.1	3 E-30	475/528
Eukaryota	Fungi	Nectria haematococca mpVI 77-13-4	EEU38248.1	3 E-30	453/528
Eukaryota	Fungi	Aspergillus niger CBS 513.88	XP_001396513.1	5 E-30	495/528
Bacteria	Proteobacteria	Burkholderia cenocepacia PC184	ZP_04941998.1	6 E-30	474/528
Bacteria	Proteobacteria	Dickeya dadantii Ech703	YP_002987908.1	1 E-29	494/528
Bacteria	Proteobacteria	Burkholderia cenocepacia MC0-3	YP_001777179.1	2 E-29	491/528
Eukaryota	Fungi	Aspergillus niger CBS 513.88	XP_001389566.1	4 E-29	480/528
Eukaryota	Fungi	Sclerotinia sclerotiorum 1980 UF-70	XP_001590621.1	4 E-29	504/528
Bacteria	Proteobacteria	Mannheimia succiniciproducens MBEL55E	YP_089433.1	5 E-29	534/528

Bacteria	Proteobacteria	Burkholderia xenovorans LB400	YP_556500.1	8 E-29	490/528
Bacteria	Proteobacteria	Ralstonia eutropha JMP134	YP_295720.1	1 E-28	498/528
Bacteria	Proteobacteria	Bradyrhizobium sp. BTAi1	YP_001239214.1	2 E-28	549/528
Bacteria	Proteobacteria	Bradyrhizobium sp. BTAi1	YP_001238757.1	2 E-28	463/528
Bacteria	Proteobacteria	Burkholderia graminis C4D1M	ZP_02886709.1	3 E-28	464/528
Bacteria	Proteobacteria	Bradyrhizobium sp. ORS278	YP_001206797.1	3 E-28	460/528
Bacteria	Proteobacteria	Campylobacter gracilis RM3268	ZP_05626107.1	5 E-28	430/528

AFUA_6G01790

Eukaryota	Fungi	Aspergillus fumigatus Af293	XP_747898.1	0.0	332/332
Eukaryota	Fungi	Neosartorya fischeri NRRL 181	XP_001257356.1	1 E-180	332/332
Eukaryota	Fungi	Aspergillus clavatus NRRL 1	XP_001270377.1	1 E-170	332/332
Eukaryota	Fungi	Penicillium chrysogenum Wisconsin 54-1255	XP_002569039.1	1 E-133	319/332
Eukaryota	Fungi	Neurospora crassa OR74A	XP_957130.2	2 E-91	299/332
Eukaryota	Fungi	Aspergillus nidulans FGSC A4	XP_681868.1	3 E-91	313/332
Eukaryota	Fungi	Talaromyces stipitatus ATCC 10500	XP_002486264.1	2 E-85	302/332
Eukaryota	Fungi	Chaetomium globosum CBS 148.51	XP_001221831.1	4 E-85	308/332
Eukaryota	Fungi	Podospora anserina DSM 980	XP_001908328.1	4 E-85	307/332
Eukaryota	Fungi	Magnaporthe grisea 70-15	XP_369871.1	2 E-84	305/332
Eukaryota	Fungi	Penicillium marneffei ATCC 18224	XP_002152558.1	3 E-84	302/332
Eukaryota	Fungi	Postia placenta Mad-698-R	XP_002473771.1	2 E-81	309/332
Eukaryota	Fungi	Pyrenophora tritici-repentis Pt-1C-BFP	XP_001936698.1	6 E-81	307/332
Eukaryota	Fungi	Phaeosphaeria nodorum SN15	XP_001802479.1	1 E-78	307/332
Eukaryota	Fungi	Penicillium chrysogenum Wisconsin 54-1255	XP_002568018.1	5 E-76	289/332
Eukaryota	Fungi	Aspergillus terreus NIH2624	XP_001217033.1	3 E-64	272/332
Eukaryota	Fungi	Coprinopsis cinerea okayama7#130	XP_001828426.1	3 E-63	279/332
Bacteria	Actinobacteria	Kineococcus radiotolerans SRS30216	YP_001363290.1	2 E-55	277/332
Bacteria	Proteobacteria	Plesiocystis pacifica SIR-1	ZP_01911481.1	5 E-21	270/332

AFUA_6G01860

Eukaryota	Fungi	Aspergillus fumigatus Af293	XP_747891.1	0.0	554/554
Eukaryota	Fungi	Neosartorya fischeri NRRL 181	XP_001257366.1	0.0	551/554
Eukaryota	Fungi	Aspergillus clavatus NRRL 1	XP_001270350.1	0.0	549/554
Eukaryota	Fungi	Aspergillus flavus NRRL3357	XP_002383101.1	0.0	513/554
Eukaryota	Fungi	Aspergillus terreus NIH2624	XP_001209961.1	0.0	520/554
Eukaryota	Fungi	Aspergillus nidulans FGSC A4	CBF85148.1	0.0	537/554
Eukaryota	Fungi	Aspergillus niger CBS 513.88	XP_001395979.1	0.0	512/554
Eukaryota	Fungi	Aspergillus nidulans FGSC A4	XP_659181.1	0.0	514/554
Eukaryota	Fungi	Phaeosphaeria nodorum SN15	XP_001790779.1	0.0	508/554
Eukaryota	Fungi	Pyrenophora tritici-repentis Pt-1C-BFP	XP_001937764.1	0.0	488/554
Eukaryota	Fungi	Sclerotinia sclerotiorum 1980 UF-70	XP_001597329.1	1 E-159	506/554
Eukaryota	Fungi	Nectria haematococca mpVI 77-13-4	EEU41662.1	1 E-130	501/554
Eukaryota	Fungi	Neosartorya fischeri NRRL 181	XP_001259225.1	1 E-129	489/554
Eukaryota	Fungi	Aspergillus terreus NIH2624	XP_001218592.1	1 E-127	489/554
Eukaryota	Fungi	Gibberella zeae PH-1	XP_383448.1	1 E-127	497/554
Eukaryota	Fungi	Aspergillus fumigatus Af293	XP_753203.1	1 E-126	489/554
Eukaryota	Fungi	Chaetomium globosum CBS 148.51	XP_001220480.1	1 E-122	478/554
Eukaryota	Fungi	Podospora anserina DSM 980	XP_001912722.1	1 E-122	486/554
Eukaryota	Fungi	Neurospora crassa OR74A	XP_964352.1	1 E-120	525/554
Eukaryota	Fungi	Verticillium albo-atrum VaMs.102	EEY22433.1	1 E-119	504/554
Eukaryota	Fungi	Aspergillus nidulans FGSC A4	XP_660803.1	1 E-116	516/554
Eukaryota	Fungi	Pyrenophora tritici-repentis Pt-1C-BFP	XP_001933299.1	1 E-116	514/554
Eukaryota	Fungi	Magnaporthe grisea 70-15	XP_369575.2	1 E-114	502/554
Eukaryota	Fungi	Gibberella zeae PH-1	XP_391543.1	1 E-113	494/554
Eukaryota	Fungi	Phaeosphaeria nodorum SN15	XP_001794107.1	1 E-111	488/554
Eukaryota	Fungi	Verticillium albo-atrum VaMs.102	EEY16671.1	1 E-110	496/554

Eukaryota	Fungi	Verticillium albo-atrum VaMs.102	EEY16566.1	1 E-109	492/554
Eukaryota	Fungi	Nectria haematococca mpVI 77-13-4	EEU43515.1	1 E-108	496/554
Eukaryota	Fungi	Penicillium marneffei ATCC 18224	XP_002151471.1	1 E-107	479/554
Eukaryota	Fungi	Talaromyces stipitatus ATCC 10500	XP_002341594.1	1 E-107	495/554
Eukaryota	Fungi	Botryotinia fuckeliana B05.10	XP_001556149.1	1 E-107	484/554
Eukaryota	Fungi	Gibberella zeae PH-1	XP_384852.1	1 E-106	499/554
Eukaryota	Fungi	Magnaporthe grisea 70-15	XP_369159.1	1 E-104	495/554
Eukaryota	Fungi	Verticillium albo-atrum VaMs.102	EEY23835.1	1 E-102	534/554
Eukaryota	Fungi	Pyrenophora tritici-repentis Pt-1C-BFP	XP_001940774.1	1 E-102	520/554
Eukaryota	Fungi	Botryotinia fuckeliana B05.10	XP_001555138.1	1 E-101	491/554
Eukaryota	Fungi	Sclerotinia sclerotiorum 1980 UF-70	XP_001585622.1	1 E-100	507/554
Eukaryota	Fungi	Sclerotinia sclerotiorum 1980 UF-70	XP_001595903.1	2 E-99	506/554
Eukaryota	Fungi	Nectria haematococca mpVI 77-13-4	EEU33794.1	4 E-98	516/554
Eukaryota	Fungi	Aspergillus nidulans FGSC A4	XP_664435.1	6 E-98	504/554
Eukaryota	Fungi	Talaromyces stipitatus ATCC 10500	XP_002480620.1	9 E-98	509/554
Eukaryota	Fungi	Aspergillus clavatus NRRL 1	XP_001273463.1	2 E-97	534/554
Eukaryota	Fungi	Aspergillus oryzae RIB40	XP_001824503.1	5 E-97	500/554
Eukaryota	Fungi	Aspergillus nidulans FGSC A4	XP_660418.1	3 E-96	520/554
Eukaryota	Fungi	Gibberella zeae PH-1	XP_383600.1	8 E-96	515/554
Eukaryota	Fungi	Aspergillus flavus NRRL3357	XP_002384138.1	9 E-96	505/554
Eukaryota	Fungi	Nectria haematococca mpVI 77-13-4	EEU36719.1	9 E-96	505/554
Eukaryota	Fungi	Coccidioides posadasii C735 delta	EER29768.1	2 E-95	501/554
Eukaryota	Fungi	Aspergillus oryzae RIB40	XP_001817400.1	9 E-95	490/554
Eukaryota	Fungi	Magnaporthe grisea 70-15	XP_364883.1	1 E-94	518/554
Eukaryota	Fungi	Penicillium marneffei ATCC 18224	XP_002144908.1	3 E-94	514/554
Eukaryota	Fungi	Penicillium chrysogenum Wisconsin 54-1255	XP_002566754.1	1 E-93	523/554
Eukaryota	Fungi	Neurospora crassa OR74A	XP_963801.1	3 E-93	524/554
Eukaryota	Fungi	Aspergillus clavatus NRRL 1	XP_001268541.1	5 E-93	517/554
Eukaryota	Fungi	Nectria haematococca mpVI 77-13-4	EEU34649.1	1 E-92	476/554
Eukaryota	Fungi	Chaetomium globosum CBS 148.51	XP_001220469.1	3 E-92	503/554
Eukaryota	Fungi	Coccidioides immitis RS;	XP_001243560.1	4 E-92	521/554
Eukaryota	Fungi	Verticillium albo-atrum VaMs.102	EEY21884.1	6 E-91	456/554
Eukaryota	Fungi	Cryptococcus neoformans var. neoformans	XP_566468.1	3 E-90	482/554
Eukaryota	Fungi	Debaryomyces hansenii CBS767	XP_462624.1	3 E-90	499/554
Eukaryota	Fungi	Neosartorya fischeri NRRL 181	XP_001264024.1	1 E-89	506/554
Eukaryota	Fungi	Aspergillus terreus NIH2624	XP_001211430.1	2 E-89	537/554
Eukaryota	Fungi	Talaromyces stipitatus ATCC 10500	XP_002479550.1	3 E-89	515/554
Eukaryota	Fungi	Aspergillus fumigatus Af293	XP_753099.1	2 E-88	528/554
Eukaryota	Fungi	Penicillium chrysogenum Wisconsin 54-1255	XP_002559288.1	2 E-88	508/554
Eukaryota	Fungi	Podospora anserina DSM 980	XP_001912766.1	7 E-88	508/554
Eukaryota	Fungi	Phaeosphaeria nodorum SN15	XP_001791292.1	1 E-87	511/554
Eukaryota	Fungi	Kluyveromyces lactis NRRL Y-1140	XP_452193.1	2 E-87	493/554
Eukaryota	Fungi	Candida intermedia	CAO79524.1	6 E-84	505/554
Eukaryota	Fungi	Penicillium marneffei ATCC 18224	XP_002149658.1	2 E-83	507/554
Eukaryota	Fungi	Aspergillus oryzae RIB40	XP_001817774.1	4 E-83	501/554
Eukaryota	Fungi	Verticillium albo-atrum VaMs.102	EEY13991.1	2 E-82	484/554
Eukaryota	Fungi	Coprinopsis cinerea okayama7#130	XP_001832384.1	3 E-81	477/554
Eukaryota	Fungi	Uncinocarpus reesii 1704	XP_002543423.1	3 E-81	445/554
Eukaryota	Fungi	Pichia stipitis CBS 6054	EAZ63208.2	1 E-79	484/554
Eukaryota	Fungi	Paxillus involutus	AAT91304.1	2 E-79	478/554
Eukaryota	Fungi	Paxillus involutus	AAT91305.1	2 E-79	478/554
Eukaryota	Fungi	Paxillus involutus	AAT91303.1	2 E-79	478/554
Eukaryota	Fungi	Aspergillus flavus NRRL3357	XP_002374068.1	2 E-79	482/554
Eukaryota	Fungi	Paxillus involutus	AAT91253.1	2 E-79	478/554
Eukaryota	Fungi	Aspergillus oryzae RIB40	XP_001820343.1	5 E-79	482/554
Eukaryota	Fungi	Aspergillus terreus NIH2624	XP_001210859.1	5 E-79	491/554
Eukaryota	Fungi	Pichia stipitis CBS 6054	XP_001387231.1	5 E-79	484/554
Eukaryota	Fungi	Phaeosphaeria nodorum SN15	XP_001800292.1	8 E-79	457/554

Eukaryota	Fungi	Neurospora crassa OR74A	XP_963873.1	1 E-77	491/554
Eukaryota	Fungi	Aspergillus terreus NIH2624	XP_001218616.1	2 E-77	496/554
Eukaryota	Fungi	Gibberella zeae PH-1	XP_391358.1	3 E-77	458/554
Eukaryota	Fungi	Paxillus involutus	AAT91307.1	4 E-77	478/554
Eukaryota	Fungi	Paxillus involutus	AAT91306.1	5 E-77	478/554
Eukaryota	Fungi	Podospora anserina DSM 980	XP_001908539.1	4 E-76	482/554
Eukaryota	Fungi	Neosartorya fischeri NRRL 181	XP_001261997.1	6 E-76	452/554
Eukaryota	Fungi	Aspergillus flavus NRRL3357	XP_002372913.1	1 E-75	459/554
Eukaryota	Fungi	Pyrenophora tritici-repentis Pt-1C-BFP	XP_001935811.1	1 E-75	483/554
Eukaryota	Fungi	Sclerotinia sclerotiorum 1980 UF-70	XP_001594466.1	4 E-75	494/554
Eukaryota	Fungi	Laccaria bicolor S238N-H82	XP_001881032.1	5 E-75	475/554
Eukaryota	Fungi	Chaetomium globosum CBS 148.51	XP_001220290.1	7 E-75	490/554
Eukaryota	Fungi	Magnaporthe grisea 70-15	XP_364462.2	3 E-74	509/554
Eukaryota	Fungi	Gibberella zeae PH-1	XP_387792.1	4 E-74	534/554
Eukaryota	Fungi	Aspergillus oryzae RIB40	XP_001820663.1	5 E-74	459/554
Eukaryota	Fungi	Aspergillus fumigatus Af293	XP_747255.1	1 E-73	491/554
Eukaryota	Fungi	Aspergillus fumigatus A1163	EDP48858.1	1 E-73	491/554
Eukaryota	Fungi	Aspergillus flavus NRRL3357	XP_002376381.1	1 E-73	459/554
Eukaryota	Fungi	Pichia stipitis CBS 6054	XP_001383110.1	2 E-73	475/554
Eukaryota	Fungi	Nectria haematococca mpVI 77-13-4	EEU33403.1	3 E-73	519/554
Eukaryota	Fungi	Nectria haematococca mpVI 77-13-4	EEU33934.1	4 E-73	471/554
Eukaryota	Fungi	Coprinopsis cinerea okayama7#130	XP_001840091.1	4 E-73	448/554
Eukaryota	Fungi	Gibberella zeae PH-1	XP_387866.1	2 E-72	476/554
Eukaryota	Fungi	Verticillium albo-atrum VaMs.102	EEY16517.1	2 E-72	452/554
Eukaryota	Fungi	Nectria haematococca mpVI 77-13-4	EEU36335.1	8 E-72	469/554
Eukaryota	Fungi	Verticillium albo-atrum VaMs.102	EEY20323.1	3 E-71	479/554
Eukaryota	Fungi	Penicillium chrysogenum Wisconsin 54-1255	XP_002568019.1	5 E-71	502/554
Eukaryota	Fungi	Talaromyces stipitatus ATCC 10500	XP_002481129.1	2 E-70	471/554
Eukaryota	Fungi	Nectria haematococca mpVI 77-13-4	EEU45208.1	5 E-70	472/554
Eukaryota	Fungi	Nectria haematococca mpVI 77-13-4	EEU38316.1	8 E-70	513/554
Eukaryota	Fungi	Nectria haematococca mpVI 77-13-4	EEU42511.1	2 E-69	502/554
Eukaryota	Fungi	Talaromyces stipitatus ATCC 10500	XP_002477997.1	2 E-69	495/554
Eukaryota	Fungi	Penicillium chrysogenum Wisconsin 54-1255	XP_002561406.1	3 E-69	508/554
Eukaryota	Fungi	Laccaria bicolor S238N-H82	XP_001880629.1	4 E-69	512/554
Eukaryota	Fungi	Gibberella zeae PH-1	XP_385528.1	5 E-69	514/554
Eukaryota	Fungi	Pichia stipitis CBS 6054	XP_001387757.1	1 E-68	465/554
Eukaryota	Fungi	Penicillium marneffei ATCC 18224	XP_002147172.1	3 E-68	502/554
Eukaryota	Fungi	Phaeosphaeria nodorum SN15	XP_001793796.1	4 E-68	496/554
Eukaryota	Fungi	Gibberella zeae PH-1	XP_383784.1	1 E-67	466/554
Eukaryota	Fungi	Magnaporthe grisea 70-15	XP_369535.1	3 E-67	522/554
Eukaryota	Fungi	Gibberella moniliformis	ABV60280.1	4 E-67	505/554
Eukaryota	Fungi	Gibberella zeae PH-1	XP_391671.1	5 E-67	470/554
Eukaryota	Fungi	Cryptococcus neoformans var. neoformans	XP_567678.1	1 E-66	458/554
Eukaryota	Fungi	Cryptococcus neoformans var. neoformans	XP_772778.1	1 E-66	458/554
Eukaryota	Fungi	Cryptococcus neoformans var. neoformans	XP_771814.1	5 E-66	491/554
Eukaryota	Fungi	Nectria haematococca mpVI 77-13-4	EEU35801.1	5 E-66	519/554
Eukaryota	Fungi	Pichia stipitis CBS 6054	XP_001383677.2	1 E-65	498/554
Eukaryota	Fungi	Penicillium marneffei ATCC 18224	XP_002150980.1	2 E-65	494/554
Eukaryota	Fungi	Aspergillus oryzae RIB40	XP_001821535.1	2 E-65	489/554
Eukaryota	Fungi	Pyrenophora tritici-repentis Pt-1C-BFP	XP_001932382.1	2 E-65	467/554
Eukaryota	Fungi	Aspergillus flavus NRRL3357	XP_002379924.1	3 E-65	489/554
Eukaryota	Fungi	Aspergillus terreus NIH2624	XP_001210244.1	4 E-65	468/554
Eukaryota	Fungi	Gibberella zeae PH-1	XP_387807.1	4 E-65	516/554
Eukaryota	Fungi	Nectria haematococca mpVI 77-13-4	EEU36861.1	1 E-64	485/554
Eukaryota	Fungi	Nectria haematococca mpVI 77-13-4	EEU35723.1	1 E-64	486/554
Eukaryota	Fungi	Nectria haematococca mpVI 77-13-4	EEU35085.1	3 E-64	473/554
Eukaryota	Fungi	Aspergillus terreus NIH2624	XP_001209810.1	3 E-64	509/554
Eukaryota	Fungi	Verticillium albo-atrum VaMs.102	EEY23963.1	7 E-64	496/554

Eukaryota	Fungi	Magnaporthe grisea 70-15	XP_366707.1	1 E-63	487/554
Eukaryota	Fungi	Podospora anserina DSM 980	XP_001906434.1	2 E-63	510/554
Eukaryota	Fungi	Aspergillus terreus NIH2624	XP_001214260.1	2 E-63	476/554
Eukaryota	Fungi	Gibberella zeae PH-1	XP_380263.1	3 E-63	483/554
Eukaryota	Fungi	Pichia guilliermondii ATCC 6260	EDK38126.2	3 E-63	481/554
Eukaryota	Fungi	Neosartorya fischeri NRRL 181	XP_001258822.1	3 E-63	482/554
Eukaryota	Fungi	Gibberella zeae PH-1	XP_391233.1	3 E-63	484/554
Eukaryota	Fungi	Gibberella zeae PH-1	XP_390861.1	4 E-63	489/554
Eukaryota	Fungi	Nectria haematococca mpVI 77-13-4	EEU33784.1	4 E-63	492/554
Eukaryota	Fungi	Penicillium chrysogenum Wisconsin 54-1255	XP_002568054.1	4 E-63	514/554
Eukaryota	Fungi	Pichia guilliermondii ATCC 6260	XP_001486553.1	7 E-63	481/554
Eukaryota	Fungi	Aspergillus parasiticus	AAS66028.1	9 E-63	477/554
Eukaryota	Fungi	Botryotinia fuckeliana B05.10	XP_001548958.1	9 E-63	456/554
Eukaryota	Fungi	Saccharomyces cerevisiae JAY291	EEU05584.1	1 E-62	482/554
Eukaryota	Fungi	Aspergillus clavatus NRRL 1	XP_001273720.1	1 E-62	487/554
Eukaryota	Fungi	Aspergillus flavus NRRL3357	XP_002378613.1	2 E-62	462/554
Eukaryota	Fungi	Aspergillus nidulans FGSC A4	XP_660218.1	2 E-62	471/554
Eukaryota	Fungi	Magnaporthe grisea 70-15	XP_361760.2	2 E-62	456/554
Eukaryota	Fungi	Aspergillus fumigatus A1163	EDP55331.1	3 E-62	471/554
Eukaryota	Fungi	Aspergillus fumigatus Af293	XP_751330.1	3 E-62	471/554
Eukaryota	Fungi	Podospora anserina DSM 980	XP_001910685.1	4 E-62	470/554
Eukaryota	Fungi	Aspergillus oryzae RIB40	XP_001823255.1	4 E-62	462/554
Eukaryota	Fungi	Aspergillus oryzae RIB40	XP_001826848.1	5 E-62	476/554
Eukaryota	Fungi	Pichia stipitis CBS 6054	XP_001384653.2	9 E-62	484/554
Eukaryota	Fungi	Gibberella zeae PH-1	XP_383320.1	1 E-61	490/554
Eukaryota	Fungi	Debaryomyces hansenii CBS767	XP_461825.1	2 E-61	471/554
Eukaryota	Fungi	Aspergillus clavatus NRRL 1	XP_001268489.1	3 E-61	474/554
Eukaryota	Fungi	Aspergillus nidulans FGSC A4	XP_657617.1	3 E-61	476/554
Eukaryota	Fungi	Aspergillus flavus NRRL3357	XP_002385201.1	4 E-61	476/554
Eukaryota	Fungi	Neosartorya fischeri NRRL 181	XP_001258591.1	5 E-61	471/554
Eukaryota	Fungi	Debaryomyces hansenii	CAG90290.2	5 E-61	471/554
Eukaryota	Fungi	Debaryomyces hansenii CBS767	XP_461829.1	6 E-61	471/554
Eukaryota	Fungi	Pichia stipitis CBS 6054	XP_001386873.1	7 E-61	482/554
Eukaryota	Fungi	Magnaporthe grisea 70-15	XP_364788.2	1 E-60	445/554
Eukaryota	Fungi	Pichia stipitis CBS 6054	XP_001385684.1	1 E-60	465/554
Eukaryota	Fungi	Magnaporthe grisea 70-15	XP_001521940.1	1 E-60	445/554
Eukaryota	Fungi	Pichia stipitis CBS 6054	EAZ62850.2	2 E-60	482/554
Eukaryota	Fungi	Penicillium marneffei ATCC 18224	XP_002145010.1	3 E-60	485/554
Eukaryota	Fungi	Aspergillus niger CBS 513.88	XP_001400787.1	7 E-60	463/554
Eukaryota	Fungi	Aspergillus oryzae	BAC20337.1	1 E-59	496/554
Eukaryota	Fungi	Aspergillus nidulans FGSC A4	XP_681381.1	1 E-59	452/554
Eukaryota	Fungi	Penicillium marneffei ATCC 18224	XP_002147677.1	1 E-59	477/554
Eukaryota	Fungi	Nectria haematococca mpVI 77-13-4	EEU34432.1	2 E-59	461/554
Eukaryota	Fungi	Nectria haematococca mpVI 77-13-4	EEU42797.1	2 E-59	454/554
Eukaryota	Fungi	Nectria haematococca mpVI 77-13-4	EEU36514.1	2 E-59	463/554
Eukaryota	Fungi	Nectria haematococca mpVI 77-13-4	EEU46706.1	3 E-59	450/554
Eukaryota	Fungi	Cryptococcus neoformans var. neoformans	XP_568685.1	3 E-59	511/554
Eukaryota	Fungi	Verticillium albo-atrum VaMs.102	EEY17151.1	4 E-59	486/554
Eukaryota	Fungi	Pichia guilliermondii ATCC 6260	EDK41291.2	6 E-59	463/554
Eukaryota	Fungi	Pichia guilliermondii ATCC 6260	XP_001482369.1	6 E-59	462/554
Eukaryota	Fungi	Phaeosphaeria nodorum SN15	XP_001801375.1	8 E-59	471/554
Eukaryota	Fungi	Pichia stipitis CBS 6054	XP_001387362.1	9 E-59	495/554
Eukaryota	Fungi	Nectria haematococca mpVI 77-13-4	EEU37335.1	1 E-58	506/554
Eukaryota	Fungi	Penicillium chrysogenum Wisconsin 54-1255	XP_002561986.1	2 E-58	450/554
Eukaryota	Fungi	Gibberella zeae PH-1	XP_387526.1	2 E-58	459/554
Eukaryota	Fungi	Verticillium albo-atrum VaMs.102	EEY17463.1	3 E-58	464/554
Eukaryota	Fungi	Nectria haematococca mpVI 77-13-4	EEU35096.1	4 E-58	461/554
Eukaryota	Fungi	Magnaporthe grisea 70-15	XP_366289.1	4 E-58	487/554

Eukaryota	Fungi	Aspergillus niger CBS 513.88	XP_001390413.1	5 E-58	458/554
Eukaryota	Fungi	Talaromyces stipitatus ATCC 10500	XP_002487579.1	6 E-58	477/554
Eukaryota	Fungi	Nectria haematococca mpVI 77-13-4	EEU39954.1	8 E-58	469/554
Eukaryota	Fungi	Coprinopsis cinerea okayama7#130	XP_001839222.1	1 E-57	499/554
Eukaryota	Fungi	Aspergillus nidulans FGSC A4	XP_682158.1	3 E-57	478/554
Eukaryota	Fungi	Pichia guilliermondii ATCC 6260	XP_001487010.1	5 E-57	477/554
Eukaryota	Fungi	Nectria haematococca mpVI 77-13-4	EEU37083.1	7 E-57	498/554
Eukaryota	Fungi	Gibberella zeae PH-1	XP_380243.1	1 E-56	449/554
Eukaryota	Fungi	Aspergillus clavatus NRRL 1	XP_001268640.1	1 E-56	487/554
Eukaryota	Fungi	Penicillium chrysogenum Wisconsin 54-1255	XP_002561138.1	1 E-56	495/554
Eukaryota	Fungi	Neosartorya fischeri NRRL 181	XP_001264124.1	1 E-56	499/554
Eukaryota	Fungi	Neosartorya fischeri NRRL 181	XP_001258602.1	2 E-56	473/554
Eukaryota	Fungi	Talaromyces stipitatus ATCC 10500	XP_002481846.1	2 E-56	478/554
Eukaryota	Fungi	Clavispora lusitaniae ATCC 42720	XP_002620019.1	2 E-56	512/554
Eukaryota	Fungi	Clavispora lusitaniae ATCC 42720	XP_002620020.1	3 E-56	509/554
Eukaryota	Fungi	Aspergillus fumigatus Af293	XP_748488.1	1 E-55	465/554
Eukaryota	Fungi	Penicillium marneffei ATCC 18224	XP_002150540.1	2 E-55	508/554
Eukaryota	Fungi	Candida tropicalis MYA-3404	XP_002550006.1	2 E-55	492/554
Eukaryota	Fungi	Verticillium albo-atrum VaMs.102	EEY17963.1	2 E-55	469/554
Eukaryota	Fungi	Neurospora crassa OR74A	XP_001728155.1	3 E-55	449/554
Eukaryota	Fungi	Pyrenophora tritici-repentis Pt-1C-BFP	XP_001941095.1	3 E-55	498/554
Eukaryota	Fungi	Nectria haematococca mpVI 77-13-4	EEU35516.1	4 E-55	473/554
Eukaryota	Fungi	Aspergillus terreus NIH2624	XP_001213315.1	7 E-55	483/554
Eukaryota	Fungi	Aspergillus nidulans FGSC A4	XP_660709.1	8 E-55	466/554
Eukaryota	Fungi	Neosartorya fischeri NRRL 181	XP_001264000.1	9 E-55	492/554
Eukaryota	Fungi	Podospora anserina DSM 980	XP_001903608.1	1 E-54	507/554
Eukaryota	Fungi	Aspergillus flavus NRRL3357	XP_002384316.1	1 E-54	457/554
Eukaryota	Fungi	Nectria haematococca mpVI 77-13-4	EEU35678.1	1 E-54	473/554
Eukaryota	Fungi	Aspergillus fumigatus Af293	XP_753005.1	2 E-54	511/554

AFUA_6G01900

Eukaryota	Fungi	Aspergillus fumigatus Af293	XP_747888.1	0.0	600/600
Eukaryota	Fungi	Neosartorya fischeri NRRL 181	XP_001257368.1	0.0	592/600
Eukaryota	Fungi	Aspergillus clavatus NRRL 1	XP_001270348.1	0.0	587/600
Eukaryota	Fungi	Penicillium chrysogenum Wisconsin 54-1255	XP_002566344.1	0.0	584/600
Eukaryota	Fungi	Aspergillus flavus NRRL3357	XP_002383102.1	0.0	581/600
Eukaryota	Fungi	Aspergillus oryzae RIB40	XP_001816962.1	0.0	581/600
Eukaryota	Fungi	Aspergillus terreus NIH2624	XP_001209959.1	0.0	587/600
Eukaryota	Fungi	Aspergillus nidulans FGSC A4	XP_681929.1	0.0	646/600
Eukaryota	Fungi	Nectria haematococca mpVI 77-13-4	EEU39086.1	1 E-173	584/600
Eukaryota	Fungi	Chaetomium globosum CBS 148.51	XP_001223533.1	1 E-168	588/600
Eukaryota	Fungi	Podospora anserina DSM 980	XP_001910678.1	1 E-168	593/600
Eukaryota	Fungi	Penicillium marneffei ATCC 18224	XP_002153112.1	1 E-167	586/600
Eukaryota	Fungi	Talaromyces stipitatus ATCC 10500	XP_002487580.1	1 E-165	589/600
Eukaryota	Fungi	Coccidioides posadasii C735 delta	EER25801.1	1 E-162	569/600
Eukaryota	Fungi	Microsporum canis CBS 113480	EEQ27605.1	1 E-161	582/600
Eukaryota	Fungi	Neurospora crassa OR74A	XP_962558.2	1 E-160	611/600
Eukaryota	Fungi	Sclerotinia sclerotiorum 1980 UF-70	XP_001587404.1	1 E-156	559/600
Eukaryota	Fungi	Ajellomyces dermatitidis SLH14081	XP_002628394.1	1 E-152	591/600
Eukaryota	Fungi	Magnaporthe grisea 70-15	XP_365662.2	1 E-149	625/600
Eukaryota	Fungi	Coccidioides immitis RS;	XP_001240556.1	1 E-146	547/600
Eukaryota	Fungi	Gibberella zeae PH-1	XP_389230.1	1 E-141	608/600
Eukaryota	Fungi	Aspergillus terreus NIH2624	XP_001217474.1	1 E-141	548/600
Eukaryota	Fungi	Nectria haematococca mpVI 77-13-4	EEU34666.1	1 E-140	548/600
Eukaryota	Fungi	Gibberella zeae PH-1	XP_387812.1	1 E-137	550/600
Eukaryota	Fungi	Ajellomyces capsulatus NAm1	XP_001543541.1	1 E-137	556/600
Eukaryota	Fungi	Uncinocarpus reesii 1704	XP_002582799.1	1 E-136	505/600

Eukaryota	Fungi	Nectria haematococca mpVI 77-13-4	EEU44563.1	1 E-136	500/600
Eukaryota	Fungi	Ajellomyces capsulatus G186AR	EEH05446.1	1 E-133	550/600
Eukaryota	Fungi	Ajellomyces capsulatus H143	EER41214.1	1 E-133	551/600
Eukaryota	Fungi	Chaetomium globosum CBS 148.51	XP_001227590.1	1 E-130	509/600
Eukaryota	Fungi	Aspergillus flavus NRRL3357	XP_002380053.1	1 E-125	600/600
Eukaryota	Fungi	Aspergillus oryzae RIB40	XP_001818644.1	1 E-124	599/600
Eukaryota	Fungi	Aspergillus nidulans FGSC A4	XP_660233.1	1 E-118	593/600
Eukaryota	Fungi	Pyrenophora tritici-repentis Pt-1C-BFP	XP_001933690.1	1 E-110	579/600
Eukaryota	Fungi	Aspergillus niger CBS 513.88	XP_001395706.1	1 E-105	563/600
Eukaryota	Fungi	Neurospora crassa OR74A	XP_963610.1	3 E-99	594/600
Eukaryota	Fungi	Phaeosphaeria nodorum SN15	XP_001802464.1	7 E-98	560/600
Eukaryota	Fungi	Aspergillus oryzae RIB40	XP_001825037.1	4 E-96	546/600
Eukaryota	Fungi	Aspergillus terreus NIH2624	XP_001211874.1	7 E-91	514/600
Eukaryota	Fungi	Aspergillus flavus NRRL3357	XP_002381712.1	3 E-90	527/600
Eukaryota	Fungi	Magnaporthe grisea 70-15	XP_364538.2	1 E-85	611/600
Eukaryota	Fungi	Talaromyces stipitatus ATCC 10500	XP_002480266.1	4 E-64	555/600
Eukaryota	Fungi	Chaetomium globosum CBS 148.51	XP_001219466.1	1 E-62	555/600
Eukaryota	Fungi	Talaromyces stipitatus ATCC 10500	XP_002482122.1	2 E-62	550/600
Eukaryota	Fungi	Uncinocarpus reesii 1704	XP_002541851.1	3 E-61	555/600
Eukaryota	Fungi	Penicillium marneffei ATCC 18224	XP_002152962.1	8 E-60	553/600
Eukaryota	Fungi	Coccidioides posadasii C735 delta	EER28767.1	1 E-59	552/600
Eukaryota	Fungi	Neosartorya fischeri NRRL 181	XP_001261663.1	4 E-59	557/600
Eukaryota	Fungi	Coccidioides immitis RS;	XP_001248626.1	6 E-59	547/600
Eukaryota	Fungi	Sclerotinia sclerotiorum 1980 UF-70	XP_001585682.1	2 E-56	569/600
Eukaryota	Fungi	Talaromyces stipitatus ATCC 10500	XP_002341958.1	2 E-54	563/600
Eukaryota	Fungi	Phaeosphaeria nodorum SN15	XP_001803278.1	8 E-47	566/600
Eukaryota	Fungi	Talaromyces stipitatus ATCC 10500	XP_002485406.1	1 E-42	552/600
Eukaryota	Fungi	Botryotinia fuckeliana B05.10	XP_001546458.1	1 E-41	527/600
Eukaryota	Fungi	Aspergillus clavatus NRRL 1	XP_001273755.1	2 E-41	618/600
Eukaryota	Fungi	Penicillium chrysogenum Wisconsin 54-1255	XP_002560017.1	1 E-39	519/600
Eukaryota	Fungi	Microsporum canis CBS 113480	EEQ35343.1	1 E-38	481/600
Eukaryota	Fungi	Botryotinia fuckeliana B05.10	XP_001548370.1	5 E-33	570/600
Eukaryota	Fungi	Coprinopsis cinerea okayama7#130	XP_001840861.1	6 E-19	599/600

AFUA_6G01905

Eukaryota	Fungi	Aspergillus fumigatus Af293	XP_747887.1	1 E-154	269/269
Eukaryota	Fungi	Aspergillus niger CBS 513.88	XP_001395970.1	5 E-12	218/269

AFUA_6G01910

Eukaryota	Fungi	Aspergillus fumigatus Af293	XP_747886.1	0.0	419/419
Eukaryota	Fungi	Neosartorya fischeri NRRL 181	XP_001257370.1	0.0	422/419
Eukaryota	Fungi	Aspergillus clavatus NRRL 1	XP_001270347.1	0.0	446/419
Eukaryota	Fungi	Aspergillus flavus NRRL3357	XP_002383103.1	1 E-119	412/419
Eukaryota	Fungi	Aspergillus niger CBS 513.88	XP_001395971.1	1 E-112	410/419
Eukaryota	Fungi	Penicillium chrysogenum Wisconsin 54-1255	XP_002566342.1	2 E-91	387/419
Eukaryota	Fungi	Penicillium marneffei ATCC 18224	XP_002148886.1	1 E-82	411/419
Eukaryota	Fungi	Talaromyces stipitatus ATCC 10500	XP_002485303.1	4 E-79	411/419
Eukaryota	Fungi	Aspergillus nidulans FGSC A4	XP_681932.1	1 E-78	412/419
Eukaryota	Fungi	Ajellomyces capsulatus H143	EER43829.1	6 E-77	410/419
Eukaryota	Fungi	Ajellomyces dermatitidis SLH14081	XP_002621021.1	8 E-74	397/419
Eukaryota	Fungi	Botryotinia fuckeliana B05.10	XP_001548571.1	3 E-48	409/419
Eukaryota	Fungi	Sclerotinia sclerotiorum 1980 UF-70	XP_001593822.1	8 E-46	422/419

AFUA_6G03480

Eukaryota	Fungi	Aspergillus fumigatus Af293	XP_747729.1	0.0	1480/1480

AFUA_6G03630

Eukaryota	Fungi	Aspergillus fumigatus Af293	XP_747714.2	0.0	632/632
Eukaryota	Fungi	Neosartorya fischeri NRRL 181	XP_001257568.1	0.0	613/632
Eukaryota	Fungi	Aspergillus clavatus NRRL 1	XP_001270187.1	0.0	614/632
Eukaryota	Fungi	Aspergillus oryzae RIB40	XP_001817018.1	0.0	632/632
Eukaryota	Fungi	Penicillium chrysogenum Wisconsin 54-1255	XP_002563829.1	0.0	632/632
Eukaryota	Fungi	Aspergillus flavus NRRL3357	XP_002383042.1	0.0	617/632
Eukaryota	Fungi	Aspergillus niger CBS 513.88	XP_001396734.1	0.0	611/632
Eukaryota	Fungi	Microsporum canis CBS 113480	EEQ34355.1	0.0	627/632
Eukaryota	Fungi	Aspergillus nidulans FGSC A4	XP_664253.1	0.0	638/632
Eukaryota	Fungi	Ajellomyces dermatitidis SLH14081	XP_002624559.1	0.0	612/632
Eukaryota	Fungi	Paracoccidioides brasiliensis Pb18;	EEH48635.1	0.0	625/632
Eukaryota	Fungi	Paracoccidioides brasiliensis Pb03;	EEH22116.1	0.0	615/632
Eukaryota	Fungi	Coccidioides immitis RS;	XP_001239112.1	0.0	612/632
Eukaryota	Fungi	Ajellomyces capsulatus G186AR	EEH09328.1	0.0	593/632
Eukaryota	Fungi	Uncinocarpus reesii 1704	XP_002544059.1	0.0	608/632
Eukaryota	Fungi	Microsporum canis CBS 113480	EEQ30277.1	0.0	622/632
Eukaryota	Fungi	Paracoccidioides brasiliensis Pb01;	EEH33504.1	0.0	589/632
Eukaryota	Fungi	Ajellomyces dermatitidis ER-3	EEQ91639.1	0.0	592/632
Eukaryota	Fungi	Penicillium marneffei ATCC 18224	XP_002143348.1	0.0	605/632
Eukaryota	Fungi	Ajellomyces capsulatus NAm1	XP_001539625.1	0.0	580/632
Eukaryota	Fungi	Talaromyces stipitatus ATCC 10500	XP_002479654.1	0.0	605/632
Eukaryota	Fungi	Coccidioides posadasii C735 delta	EER29930.1	0.0	545/632
Eukaryota	Fungi	Phaeosphaeria nodorum SN15	XP_001799306.1	0.0	614/632
Eukaryota	Fungi	Aspergillus clavatus NRRL 1	XP_001273689.1	0.0	631/632
Eukaryota	Fungi	Pyrenophora tritici-repentis Pt-1C-BFP	XP_001931539.1	0.0	614/632
Eukaryota	Fungi	Pyrenophora tritici-repentis Pt-1C-BFP	XP_001935974.1	0.0	648/632
Eukaryota	Fungi	Botryotinia fuckeliana B05.10	XP_001551612.1	0.0	640/632
Eukaryota	Fungi	Sclerotinia sclerotiorum 1980 UF-70	XP_001593853.1	1 E-179	624/632
Eukaryota	Fungi	Magnaporthe grisea 70-15	XP_359752.1	1 E-170	613/632
Eukaryota	Fungi	Acremonium chrysogenum;	CAD45625.1	1 E-160	583/632
Eukaryota	Fungi	Podospora anserina DSM 980	XP_001903228.1	1 E-128	628/632
Eukaryota	Fungi	Microsporum canis CBS 113480	EEQ29348.1	1 E-127	632/632
Eukaryota	Fungi	Podospora anserina DSM 980	XP_001905325.1	1 E-126	620/632
Eukaryota	Fungi	Penicillium marneffei ATCC 18224	XP_002150629.1	1 E-124	627/632
Eukaryota	Fungi	Aspergillus terreus NIH2624	XP_001211043.1	1 E-124	625/632
Eukaryota	Fungi	Aspergillus niger CBS 513.88	XP_001391993.1	1 E-123	643/632
Eukaryota	Fungi	Aspergillus flavus NRRL3357	XP_002373722.1	1 E-122	647/632
Eukaryota	Fungi	Aspergillus clavatus NRRL 1	XP_001267878.1	1 E-122	645/632
Eukaryota	Fungi	Talaromyces stipitatus ATCC 10500	XP_002483523.1	1 E-121	634/632
Eukaryota	Fungi	Uncinocarpus reesii 1704	XP_002542773.1	1 E-121	645/632
Eukaryota	Fungi	Coccidioides immitis RS;	XP_001240844.1	1 E-121	645/632
Eukaryota	Fungi	Coccidioides posadasii C735 delta	EER23017.1	1 E-121	645/632
Eukaryota	Fungi	Nectria haematococca mpVI 77-13-4	EEU38169.1	1 E-121	615/632
Eukaryota	Fungi	Neosartorya fischeri NRRL 181	XP_001257846.1	1 E-120	637/632
Eukaryota	Fungi	Aspergillus fumigatus Af293	XP_750620.1	1 E-120	637/632
Eukaryota	Fungi	Gibberella zeae PH-1	XP_387715.1	1 E-120	628/632
Eukaryota	Fungi	Chaetomium globosum CBS 148.51	XP_001226163.1	1 E-120	624/632
Eukaryota	Fungi	Aspergillus nidulans FGSC A4	XP_662796.1	1 E-120	637/632
Eukaryota	Fungi	Ajellomyces dermatitidis ER-3	EEQ85503.1	1 E-119	646/632
Eukaryota	Fungi	Ajellomyces dermatitidis SLH14081	XP_002622431.1	1 E-119	646/632
Eukaryota	Fungi	Ajellomyces capsulatus G186AR	EEH04085.1	1 E-119	646/632
Eukaryota	Fungi	Aspergillus oryzae RIB40	XP_001818484.1	1 E-119	624/632
Eukaryota	Fungi	Ajellomyces capsulatus NAm1	XP_001537495.1	1 E-119	646/632
Eukaryota	Fungi	Aspergillus terreus NIH2624	XP_001212885.1	1 E-118	644/632
Eukaryota	Fungi	Paracoccidioides brasiliensis Pb01;	EEH35359.1	1 E-117	646/632

Eukaryota	Fungi	Ustilago maydis 521	XP_758730.1	1 E-116	626/632
Eukaryota	Fungi	Penicillium chrysogenum Wisconsin 54-1255	XP_002562398.1	1 E-116	633/632
Eukaryota	Fungi	Cochliobolus heterostrophus	O42633.1	1 E-116	610/632
Eukaryota	Fungi	Paracoccidioides brasiliensis Pb03;	EEH21872.1	1 E-113	629/632
Eukaryota	Fungi	Paracoccidioides brasiliensis Pb18;	EEH42723.1	1 E-113	629/632
Eukaryota	Fungi	Magnaporthe grisea 70-15	XP_362791.1	1 E-113	557/632
Eukaryota	Fungi	Ajellomyces dermatitidis SLH14081	XP_002628472.1	1 E-112	614/632
Eukaryota	Fungi	Ajellomyces dermatitidis ER-3	EEQ89281.1	1 E-112	614/632
Eukaryota	Fungi	Sclerotinia sclerotiorum 1980 UF-70	XP_001596429.1	1 E-111	625/632
Eukaryota	Fungi	Ajellomyces capsulatus G186AR	EEH05375.1	1 E-111	630/632
Eukaryota	Fungi	Paracoccidioides brasiliensis Pb01;	EEH33260.1	1 E-106	608/632
Eukaryota	Fungi	Yarrowia lipolytica CLIB122	XP_504004.1	1 E-106	625/632
Eukaryota	Fungi	Botryotinia fuckeliana B05.10	XP_001560276.1	1 E-104	554/632
Eukaryota	Fungi	Monascus aurantiacus	ACA34725.1	1 E-101	603/632
Eukaryota	Fungi	Clavispora lusitaniae ATCC 42720	XP_002616453.1	1 E-101	646/632
Eukaryota	Fungi	Pyrenophora tritici-repentis Pt-1C-BFP	XP_001930790.1	1 E-99	534/632
Eukaryota	Fungi	Lodderomyces elongisporus NRRL YB-4239	XP_001523894.1	3 E-98	647/632
Eukaryota	Fungi	Verticillium albo-atrum VaMs.102	EEY21003.1	7 E-98	540/632
Bacteria	Proteobacteria	Hyphomonas neptunium ATCC 15444	YP_761262.1	2 E-97	595/632
Eukaryota	Fungi	Pichia guilliermondii ATCC 6260	XP_001484426.1	3 E-97	652/632
Eukaryota	Fungi	Pichia guilliermondii ATCC 6260	EDK39709.2	3 E-97	652/632
Eukaryota	Fungi	Debaryomyces hansenii CBS767	XP_461022.1	5 E-97	652/632
Eukaryota	Fungi	Pichia stipitis CBS 6054	XP_001385064.2	2 E-96	647/632
Eukaryota	Fungi	Aspergillus fumigatus Af293	XP_755469.1	6 E-96	624/632
Eukaryota	Metazoa	Branchiostoma floridae	XP_002588223.1	2 E-95	615/632
Eukaryota	Fungi	Neosartorya fischeri NRRL 181	XP_001260605.1	1 E-94	624/632
Eukaryota	Fungi	Aspergillus niger CBS 513.88	XP_001399469.1	2 E-94	612/632
Eukaryota	Fungi	Candida albicans SC5314	XP_711373.1	3 E-94	647/632
Eukaryota	Fungi	Aspergillus flavus NRRL3357	XP_002379433.1	6 E-94	622/632
Eukaryota	Fungi	Candida dubliniensis CD36	XP_002420867.1	1 E-93	647/632
Eukaryota	Fungi	Aspergillus oryzae RIB40	XP_001821953.1	2 E-93	622/632
Eukaryota	Fungi	Aspergillus clavatus NRRL 1	XP_001275426.1	2 E-93	624/632
Bacteria	Proteobacteria	Brevundimonas sp. BAL3	ZP_05034855.1	2 E-93	561/632
Eukaryota	Fungi	Candida tropicalis MYA-3404	XP_002548639.1	5 E-93	647/632
Eukaryota	Fungi	Penicillium marneffei ATCC 18224	XP_002152074.1	5 E-93	570/632
Bacteria	Proteobacteria	Parvibaculum lavamentivorans DS-1	YP_001412921.1	6 E-93	560/632
Eukaryota	Fungi	Neurospora crassa OR74A	XP_959622.2	9 E-93	524/632
Eukaryota	Metazoa	Bos taurus	XP_615837.3	1 E-92	570/632
Eukaryota	Metazoa	Macaca mulatta	XP_001114099.1	5 E-92	562/632
Eukaryota	Fungi	Talaromyces stipitatus ATCC 10500	XP_002481107.1	9 E-92	624/632
Eukaryota	Metazoa	Xenopus laevis	AAH41746.1	2 E-91	596/632
Eukaryota	Metazoa	Xenopus (Silurana) tropicalis	AAH98084.1	9 E-91	587/632
Eukaryota	Metazoa	Pan troglodytes	XP_510394.2	2 E-90	562/632
Eukaryota	Metazoa	Homo sapiens	NP_003636.2	2 E-90	562/632
Eukaryota	Metazoa	Homo sapiens	O14975.1	2 E-90	562/632
Bacteria	Proteobacteria	Hirschia baltica ATCC 49814	YP_003058519.1	3 E-90	506/632
Eukaryota	Metazoa	Monodelphis domestica	XP_001369939.1	3 E-90	552/632
Eukaryota	Fungi	Aspergillus nidulans FGSC A4	XP_663481.1	4 E-90	615/632
Eukaryota	Metazoa	Homo sapiens	BAD96579.1	6 E-90	562/632
Eukaryota	Metazoa	Branchiostoma floridae	XP_002613260.1	2 E-89	563/632
Eukaryota	Fungi	Penicillium chrysogenum Wisconsin 54-1255	XP_002560269.1	3 E-89	628/632
Bacteria	Proteobacteria	Methylobacterium extorquens AM1	YP_002965938.1	3 E-89	538/632
Eukaryota	Metazoa	Mus musculus	O35488.1	3 E-89	562/632
Eukaryota	Metazoa	Mus musculus	CAA11687.1	4 E-89	562/632
Bacteria	Proteobacteria	Methylobacterium extorquens PA1	YP_001642045.1	5 E-89	538/632
Eukaryota	Metazoa	Mus musculus	NP_036108.2	5 E-89	562/632
Bacteria	Actinobacteria	Mycobacterium tuberculosis T85;	ZP_03432133.1	8 E-89	534/632
Bacteria	Actinobacteria	Mycobacterium tuberculosis str. Haarlem	ZP_04980153.1	8 E-89	534/632

Eukaryota	Metazoa	Sus scrofa	XP_001928401.1	1 E-88	549/632
Eukaryota	Metazoa	Branchiostoma floridae	XP_002602011.1	1 E-88	576/632
Eukaryota	Metazoa	Branchiostoma floridae	XP_002587137.1	1 E-88	587/632
Eukaryota	Metazoa	Branchiostoma floridae	XP_002587138.1	2 E-88	621/632
Bacteria	Proteobacteria	Methylobacterium extorquens DM4	YP_003071067.1	2 E-88	538/632
Eukaryota	Fungi	Aspergillus terreus NIH2624	XP_001208808.1	2 E-88	610/632
Bacteria	Proteobacteria	Phenylobacterium zucineum HLK1	YP_002129440.1	2 E-88	536/632
Bacteria	Actinobacteria	Mycobacterium tuberculosis H37Rv;	NP_215722.1	3 E-88	534/632
Eukaryota	Metazoa	Equus caballus	XP_001502057.1	3 E-88	562/632
Bacteria	Proteobacteria	Caulobacter sp. K31	YP_001685165.1	3 E-88	537/632
Eukaryota	Metazoa	Strongylocentrotus purpuratus	XP_797528.2	4 E-88	563/632
Bacteria	Actinobacteria	Mycobacterium marinum M	YP_001852495.1	5 E-88	534/632
Bacteria	Proteobacteria	Thauera sp. E7	ACB12933.1	5 E-88	533/632
Eukaryota	Metazoa	Nasonia vitripennis	XP_001603871.1	5 E-88	622/632
Eukaryota	Metazoa	Rattus norvegicus	AAH81766.1	6 E-88	577/632
Bacteria	Actinobacteria	Mycobacterium ulcerans Agy99	YP_905050.1	8 E-88	534/632
Bacteria	Actinobacteria	Mycobacterium tuberculosis T92;	ZP_03424431.1	9 E-88	511/632
Eukaryota	Metazoa	Mus musculus	BAE25684.1	1 E-87	530/632
Bacteria	Proteobacteria	marine gamma proteobacterium HTCC2148	ZP_05094987.1	1 E-87	543/632
Eukaryota	Metazoa	Danio rerio	NP_001020470.1	2 E-87	558/632
Eukaryota	Metazoa	Rattus norvegicus	NP_113924.1	2 E-87	567/632
Eukaryota	Metazoa	Danio rerio	AAH68405.1	2 E-87	558/632
Eukaryota	Fungi	Lachancea thermotolerans CBS 6340	XP_002552576.1	4 E-87	628/632
Bacteria	Proteobacteria	gamma proteobacterium HTCC2207	ZP_01225081.1	5 E-87	558/632
Bacteria	Proteobacteria	Methylobacterium nodulans ORS 2060	YP_002498564.1	1 E-86	535/632
Bacteria	Proteobacteria	Methylobacterium chloromethanicum CM4	YP_002423760.1	2 E-86	538/632
Eukaryota	Metazoa	Salmo salar	NP_001135269.1	3 E-86	561/632
Eukaryota	Fungi	Pichia pastoris GS115	XP_002490190.1	3 E-86	624/632
Eukaryota	Metazoa	Anopheles gambiae str. PEST	XP_309835.2	4 E-86	577/632
Bacteria	Proteobacteria	Oceanicaulis alexandrii HTCC2633	ZP_00953260.1	5 E-86	567/632
Eukaryota	Metazoa	Canis lupus familiaris	XP_535473.2	5 E-86	549/632
Bacteria	Proteobacteria	Rhodopseudomonas palustris BisB5	YP_568242.1	7 E-86	560/632
Eukaryota	Metazoa	Strongylocentrotus purpuratus	XP_794803.2	2 E-85	624/632
Eukaryota	Metazoa	Danio rerio	NP_001076488.1	2 E-85	561/632
Eukaryota	Fungi	Saccharomyces cerevisiae	NP_009597.2	3 E-85	591/632
Bacteria	Proteobacteria	Sphingomonas wittichii RW1	YP_001263800.1	3 E-85	546/632
Eukaryota	Fungi	Saccharomyces cerevisiae YJM789	EDN64654.1	3 E-85	591/632
Bacteria	Actinobacteria	Mycobacterium avium 104;	YP_880595.1	3 E-85	531/632
Bacteria	Actinobacteria	Mycobacterium intracellulare ATCC 13950	ZP_05227034.1	3 E-85	531/632
Bacteria	Proteobacteria	Limnobacter sp. MED105	ZP_01915065.1	5 E-85	540/632
Bacteria	Actinobacteria	Mycobacterium avium subsp. paratuberculosis	NP_961505.1	6 E-85	531/632
Bacteria	Proteobacteria	Asticcacaulis excentricus CB 48	ZP_04768960.1	6 E-85	512/632
Bacteria	Actinobacteria	Mycobacterium kansasii ATCC 12478	ZP_04747674.1	1 E-84	530/632
Eukaryota	Fungi	Kluyveromyces lactis NRRL Y-1140	XP_451837.1	1 E-84	561/632
Bacteria	Actinobacteria	Rhodococcus jostii RHA1	YP_705902.1	2 E-84	538/632
Bacteria	Proteobacteria	Methylobacterium sp. 4-46	YP_001771095.1	2 E-84	525/632
Eukaryota	Metazoa	Tetraodon nigroviridis	CAF99800.1	3 E-84	578/632
Eukaryota	Metazoa	Trichoplax adhaerens	XP_002109359.1	3 E-84	565/632
Bacteria	Actinobacteria	Rhodococcus opacus B4	YP_002783219.1	3 E-84	538/632
Eukaryota	Metazoa	Aedes aegypti	XP_001658965.1	4 E-84	578/632
Bacteria	Proteobacteria	Caulobacter crescentus NA1000	YP_002517757.1	4 E-84	537/632
Bacteria	Proteobacteria	Bradyrhizobium sp. ORS278	YP_001208146.1	5 E-84	524/632
Bacteria	Proteobacteria	Maricaulis maris MCS10	YP_757539.1	6 E-84	557/632
Bacteria	Proteobacteria	Caulobacter crescentus CB15	NP_421102.1	6 E-84	582/632
Eukaryota	Fungi	Candida glabrata CBS 138	XP_447451.1	1 E-83	628/632
Bacteria	Actinobacteria	Rhodococcus erythropolis SK121	ZP_04387617.1	1 E-83	535/632
Eukaryota	Fungi	Ajellomyces capsulatus G186AR	EEH07417.1	2 E-83	645/632
Eukaryota	Metazoa	Tetraodon nigroviridis	CAF99827.1	3 E-83	561/632

Eukaryota	Metazoa	Xenopus laevis	NP_001090443.1	3 E-83	562/632
Eukaryota	Metazoa	Aedes aegypti	XP_001648665.1	4 E-83	565/632
Eukaryota	Metazoa	Apis mellifera	XP_392108.2	4 E-83	590/632
Bacteria	Actinobacteria	Mycobacterium sp. MCS	YP_641168.1	5 E-83	526/632
Eukaryota	Metazoa	Tribolium castaneum	XP_967675.1	6 E-83	563/632
Bacteria	Actinobacteria	Mycobacterium sp. JLS	YP_001072499.1	6 E-83	526/632
Bacteria	Proteobacteria	Rhodopseudomonas palustris HaA2	YP_484621.1	7 E-83	559/632
Eukaryota	Metazoa	Culex quinquefasciatus	XP_001867718.1	8 E-83	536/632
Eukaryota	Fungi	Ashbya gossypii ATCC 10895	NP_983230.1	9 E-83	559/632
Eukaryota	Metazoa	Culex quinquefasciatus	XP_001848670.1	1 E-82	564/632
Bacteria	Actinobacteria	Mycobacterium abscessus	YP_001702084.1	1 E-82	551/632
Bacteria	Proteobacteria	Rhodopseudomonas palustris TIE-1	YP_001994036.1	2 E-82	557/632
Eukaryota	Metazoa	Canis lupus familiaris	XP_862254.1	2 E-82	549/632
Eukaryota	Metazoa	Nematostella vectensis	XP_001635484.1	2 E-82	561/632
Bacteria	Actinobacteria	Mycobacterium vanbaalenii PYR-1	YP_955291.1	4 E-82	536/632
Bacteria	Actinobacteria	Rhodococcus erythropolis PR4	YP_002767615.1	4 E-82	535/632
Eukaryota	Fungi	Zygosaccharomyces rouxii CBS 732	XP_002494527.1	5 E-82	629/632
Eukaryota	Metazoa	Gallus gallus	XP_415504.2	1 E-81	591/632
Eukaryota	Metazoa	Ixodes scapularis	XP_002399547.1	2 E-81	538/632
Bacteria	Proteobacteria	Rhodopseudomonas palustris CGA009	NP_949926.1	4 E-81	553/632
Bacteria	Proteobacteria	Bradyrhizobium japonicum USDA 110	NP_774425.1	4 E-81	555/632
Bacteria	Proteobacteria	Acinetobacter sp. ADP1	YP_045199.1	4 E-81	568/632
Eukaryota	Metazoa	Gallus gallus	XP_001233248.1	6 E-81	561/632
Bacteria	Proteobacteria	Acinetobacter radioresistens SK82	ZP_05362419.1	6 E-81	561/632
Eukaryota	Metazoa	Drosophila willistoni	XP_002066144.1	8 E-81	570/632
Eukaryota	Metazoa	Xenopus (Silurana) tropicalis	NP_001011348.2	8 E-81	562/632
Bacteria	Actinobacteria	Mycobacterium gilvum PYR-GCK	YP_001133451.1	8 E-81	535/632
Eukaryota	Metazoa	Xenopus (Silurana) tropicalis	AAH88505.1	1 E-80	562/632
Eukaryota	Fungi	Saccharomyces cerevisiae	CAA84983.1	1 E-80	534/632
Eukaryota	Metazoa	Manduca sexta	ACT22576.1	1 E-80	561/632
Eukaryota	Fungi	Ajellomyces capsulatus NAm1	XP_001537702.1	1 E-80	560/632
Eukaryota	Metazoa	Taeniopygia guttata	XP_002188490.1	1 E-80	561/632
Bacteria	Proteobacteria	Pseudoalteromonas haloplanktis TAC125	YP_339420.1	3 E-80	533/632
Eukaryota	Metazoa	Strongylocentrotus purpuratus	XP_792970.2	4 E-80	567/632
Eukaryota	Metazoa	Drosophila yakuba	XP_002092800.1	4 E-80	569/632
Eukaryota	Metazoa	Drosophila yakuba	XP_002088240.1	6 E-80	535/632
Eukaryota	Metazoa	Taeniopygia guttata	XP_002197433.1	1 E-79	595/632
Eukaryota	Metazoa	Drosophila ananassae	XP_001960831.1	1 E-79	555/632
Bacteria	Proteobacteria	Pseudomonas fluorescens Pf0-1	YP_347460.1	2 E-79	567/632
Eukaryota	Metazoa	Danio rerio	NP_001008639.1	2 E-79	562/632
Eukaryota	Metazoa	Drosophila melanogaster	NP_995926.1	3 E-79	555/632
Eukaryota	Metazoa	Drosophila simulans	XP_002082942.1	3 E-79	548/632
Eukaryota	Metazoa	Drosophila erecta	XP_001976580.1	4 E-79	569/632
Eukaryota	Metazoa	Equus caballus	XP_001504525.1	4 E-79	560/632
Eukaryota	Metazoa	Drosophila melanogaster	NP_611749.2	4 E-79	556/632
Bacteria	Proteobacteria	marine gamma proteobacterium HTCC2080	ZP_01626876.1	4 E-79	563/632
Eukaryota	Metazoa	Mus musculus	AAC40186.1	4 E-79	565/632
Eukaryota	Metazoa	Drosophila melanogaster	NP_995925.1	5 E-79	556/632
Bacteria	Proteobacteria	Marinobacter aquaeolei VT8	YP_960071.1	5 E-79	543/632
Eukaryota	Metazoa	Acyrthosiphon pisum	XP_001944545.1	5 E-79	544/632
Eukaryota	Metazoa	Drosophila erecta	XP_001976336.1	5 E-79	555/632
Eukaryota	Fungi	Vanderwaltozyma polyspora DSM 70294	XP_001647123.1	6 E-79	564/632
Eukaryota	Metazoa	Pediculus humanus corporis	XP_002431863.1	7 E-79	559/632
Eukaryota	Metazoa	Drosophila virilis	XP_002049432.1	8 E-79	555/632
Eukaryota	Metazoa	Drosophila melanogaster	NP_524723.2	9 E-79	535/632
Bacteria	Proteobacteria	marine gamma proteobacterium HTCC2080	ZP_01625347.1	9 E-79	565/632
Eukaryota	Metazoa	Drosophila simulans	XP_002078981.1	9 E-79	535/632
Eukaryota	Metazoa	Strongylocentrotus purpuratus	XP_784241.2	9 E-79	594/632

Eukaryota	Metazoa	Drosophila yakuba	XP_002092546.1	9 E-79	555/632
Eukaryota	Metazoa	Drosophila simulans	XP_002082716.1	1 E-78	555/632
Eukaryota	Metazoa	Strongylocentrotus purpuratus	XP_794818.2	1 E-78	624/632
Eukaryota	Metazoa	Drosophila erecta	AAO01012.1	1 E-78	556/632
Bacteria	Proteobacteria	Pseudomonas citronellolis	ABC69247.1	1 E-78	533/632
Bacteria	Proteobacteria	Acinetobacter baumannii ACICU	YP_001845068.1	1 E-78	568/632
Eukaryota	Metazoa	Anopheles gambiae str. PEST	XP_321320.4	1 E-78	575/632
Eukaryota	Metazoa	Drosophila melanogaster	NP_726437.2	1 E-78	569/632
Eukaryota	Metazoa	Drosophila melanogaster	NP_611906.1	1 E-78	569/632
Bacteria	Proteobacteria	Acinetobacter baumannii SDF	YP_001708229.1	2 E-78	568/632
Bacteria	Proteobacteria	Acinetobacter baumannii ATCC 17978	YP_001083461.1	2 E-78	568/632
Eukaryota	Metazoa	Drosophila erecta	XP_001969961.1	2 E-78	535/632
Eukaryota	Metazoa	Drosophila ananassae	XP_001963147.1	2 E-78	535/632
Bacteria	Proteobacteria	Acinetobacter baumannii ATCC 19606	ZP_05828459.1	3 E-78	553/632
Eukaryota	Metazoa	Drosophila ananassae	XP_001960659.1	3 E-78	569/632
Eukaryota	Metazoa	Drosophila virilis	XP_002049448.1	3 E-78	619/632

AFUA_6G05350

Eukaryota	Fungi	Aspergillus fumigatus Af293	XP_747542.1	0.0	485/485
Eukaryota	Fungi	Neosartorya fischeri NRRL 181	XP_001257744.1	0.0	485/485
Eukaryota	Fungi	Aspergillus clavatus NRRL 1	XP_001270014.1	0.0	484/485
Eukaryota	Fungi	Aspergillus flavus NRRL3357	XP_002378210.1	0.0	487/485
Eukaryota	Fungi	Aspergillus oryzae RIB40	XP_001822899.1	0.0	487/485
Eukaryota	Fungi	Aspergillus terreus NIH2624	XP_001214806.1	0.0	471/485
Eukaryota	Fungi	Aspergillus nidulans FGSC A4	XP_664091.1	1 E-165	451/485
Eukaryota	Fungi	Penicillium chrysogenum Wisconsin 54-1255	XP_002557331.1	1 E-161	475/485
Eukaryota	Fungi	Penicillium marneffei ATCC 18224	XP_002153232.1	1 E-151	464/485
Eukaryota	Fungi	Talaromyces stipitatus ATCC 10500	XP_002488208.1	1 E-140	438/485
Eukaryota	Fungi	Ajellomyces dermatitidis SLH14081	XP_002620209.1	1 E-128	410/485
Eukaryota	Fungi	Botryotinia fuckeliana B05.10	XP_001555017.1	1 E-127	434/485
Eukaryota	Fungi	Sclerotinia sclerotiorum 1980 UF-70	XP_001598535.1	1 E-126	411/485
Eukaryota	Fungi	Uncinocarpus reesii 1704	XP_002543607.1	1 E-122	460/485
Eukaryota	Fungi	Pyrenophora tritici-repentis Pt-1C-BFP	XP_001933970.1	1 E-121	412/485
Eukaryota	Fungi	Paracoccidioides brasiliensis Pb03;	EEH16860.1	1 E-120	439/485
Eukaryota	Fungi	Magnaporthe grisea 70-15	XP_369812.1	1 E-119	435/485
Eukaryota	Fungi	Ajellomyces capsulatus G186AR	EEH02610.1	1 E-118	437/485
Eukaryota	Fungi	Nectria haematococca mpVI 77-13-4	EEU43268.1	1 E-117	423/485
Eukaryota	Fungi	Coccidioides immitis RS;	XP_001248862.1	1 E-116	440/485
Eukaryota	Fungi	Coccidioides posadasii;	ABA54910.1	1 E-116	440/485
Eukaryota	Fungi	Verticillium albo-atrum VaMs.102	EEY20672.1	1 E-115	418/485
Eukaryota	Fungi	Paracoccidioides brasiliensis Pb01;	EEH41333.1	1 E-114	418/485
Eukaryota	Fungi	Paracoccidioides brasiliensis Pb18;	EEH50128.1	1 E-113	407/485
Eukaryota	Fungi	Podospora anserina DSM 980	XP_001908191.1	1 E-112	433/485
Eukaryota	Fungi	Microsporum canis CBS 113480	EEQ32553.1	1 E-112	455/485
Eukaryota	Fungi	Chaetomium globosum CBS 148.51	XP_001229789.1	1 E-112	451/485
Eukaryota	Fungi	Phaeosphaeria nodorum SN15	XP_001792431.1	1 E-111	411/485
Eukaryota	Fungi	Ajellomyces capsulatus NAm1	XP_001539255.1	1 E-110	457/485
Eukaryota	Fungi	Gibberella zeae PH-1	XP_385647.1	1 E-104	467/485
Eukaryota	Fungi	Aspergillus oryzae	BAC00849.1	3 E-99	424/485
Eukaryota	Fungi	Aspergillus oryzae RIB40	XP_001816654.1	3 E-99	419/485
Eukaryota	Fungi	Aspergillus flavus NRRL3357	XP_002383432.1	3 E-99	419/485
Eukaryota	Fungi	Aspergillus niger CBS 513.88	XP_001396351.1	1 E-97	406/485
Eukaryota	Fungi	Aspergillus niger CBS 513.88	XP_001398592.1	7 E-91	403/485
Eukaryota	Fungi	Neosartorya fischeri NRRL 181	XP_001262266.1	3 E-90	437/485
Eukaryota	Fungi	Aspergillus clavatus NRRL 1	XP_001276766.1	4 E-90	392/485
Eukaryota	Fungi	Aspergillus nidulans FGSC A4	XP_659177.1	4 E-85	468/485
Eukaryota	Fungi	Penicillium marneffei ATCC 18224	XP_002147216.1	9 E-81	446/485

Eukaryota	Fungi	Talaromyces stipitatus ATCC 10500	XP_002481346.1	2 E-78	418/485
Eukaryota	Fungi	Chaetomium globosum CBS 148.51	XP_001220576.1	6 E-78	400/485
Eukaryota	Fungi	Podospora anserina DSM 980	XP_001912861.1	1 E-77	417/485
Eukaryota	Fungi	Neurospora crassa OR74A	XP_965343.1	2 E-74	405/485
Eukaryota	Fungi	Nectria haematococca mpVI 77-13-4	EEU44215.1	7 E-73	415/485
Eukaryota	Fungi	Sclerotinia sclerotiorum 1980 UF-70	XP_001585877.1	3 E-71	413/485
Eukaryota	Fungi	Gibberella zeae PH-1	XP_381566.1	1 E-70	395/485
Eukaryota	Fungi	Neurospora crassa OR74A	XP_964574.1	2 E-60	418/485
Eukaryota	Fungi	Magnaporthe grisea 70-15	XP_366938.1	8 E-60	476/485
Eukaryota	Fungi	Verticillium albo-atrum VaMs.102	EEY17782.1	1 E-59	389/485
Eukaryota	Fungi	Yarrowia lipolytica CLIB122	XP_503768.1	1 E-59	468/485
Eukaryota	Fungi	Penicillium marneffei ATCC 18224	XP_002150565.1	3 E-58	424/485
Eukaryota	Fungi	Talaromyces stipitatus ATCC 10500	XP_002483627.1	1 E-57	429/485
Eukaryota	Fungi	Nectria haematococca mpVI 77-13-4	EEU46918.1	1 E-51	408/485
Eukaryota	Fungi	Podospora anserina DSM 980	XP_001907160.1	4 E-50	409/485
Eukaryota	Fungi	Yarrowia lipolytica CLIB122	XP_505251.1	3 E-49	412/485
Eukaryota	Fungi	Candida glabrata CBS 138	XP_445766.1	6 E-49	430/485
Eukaryota	Fungi	Botryotinia fuckeliana B05.10	XP_001560386.1	2 E-47	435/485
Eukaryota	Fungi	Hypocrea lixii	CAL30188.1	3 E-47	400/485
Eukaryota	Fungi	Yarrowia lipolytica CLIB122	XP_500335.1	4 E-47	417/485
Eukaryota	Fungi	Yarrowia lipolytica CLIB122	XP_500144.1	3 E-46	418/485
Eukaryota	Fungi	Zygosaccharomyces rouxii CBS 732	XP_002497646.1	2 E-45	452/485
Eukaryota	Fungi	Yarrowia lipolytica CLIB122	XP_504265.1	4 E-45	399/485
Eukaryota	Fungi	Aspergillus clavatus NRRL 1	XP_001276381.1	7 E-45	416/485
Eukaryota	Fungi	Ashbya gossypii ATCC 10895	NP_986475.1	8 E-45	431/485
Eukaryota	Fungi	Zygosaccharomyces rouxii CBS 732	XP_002498127.1	8 E-45	449/485
Eukaryota	Fungi	Saccharomyces cerevisiae	NP_012305.1	3 E-44	443/485
Eukaryota	Fungi	Saccharomyces cerevisiae JAY291	EEU08237.1	3 E-44	443/485
Eukaryota	Fungi	Zygosaccharomyces rouxii CBS 732	XP_002497643.1	4 E-44	422/485
Eukaryota	Fungi	Saccharomyces cerevisiae RM11-1a	EDV09452.1	1 E-43	443/485
Eukaryota	Fungi	Kluyveromyces lactis NRRL Y-1140	XP_453761.1	1 E-43	409/485
Eukaryota	Fungi	Candida glabrata CBS 138	XP_445767.1	4 E-43	409/485
Eukaryota	Fungi	Saccharomyces cerevisiae YJM789	EDN61534.1	6 E-43	443/485
Eukaryota	Fungi	Candida albicans WO-1	EEQ43824.1	7 E-43	388/485
Eukaryota	Fungi	Candida albicans	P28871.1	1 E-42	388/485
Eukaryota	Fungi	Candida albicans	prf2124256A	2 E-42	388/485
Eukaryota	Fungi	Candida albicans	AAM21050.1	3 E-42	388/485
Eukaryota	Fungi	Candida albicans SC5314	XP_711061.1	3 E-42	388/485
Eukaryota	Fungi	Saccharomyces cerevisiae RM11-1a	EDV10953.1	1 E-41	395/485
Eukaryota	Fungi	Ashbya gossypii ATCC 10895	NP_986906.1	2 E-41	402/485
Eukaryota	Fungi	Lachancea thermotolerans CBS 6340	XP_002554498.1	5 E-41	420/485
Eukaryota	Fungi	Candida glabrata CBS 138	XP_445765.1	7 E-41	412/485
Eukaryota	Fungi	Candida albicans SC5314	XP_719265.1	1 E-40	411/485
Eukaryota	Fungi	Saccharomyces cerevisiae	AAC49112.1	3 E-40	433/485
Eukaryota	Fungi	Saccharomyces cerevisiae	NP_010428.1	4 E-40	431/485
Eukaryota	Fungi	Saccharomyces cerevisiae RM11-1a	EDV08165.1	5 E-40	431/485
Eukaryota	Fungi	Saccharomyces cerevisiae EC1118	CAY78649.1	5 E-40	431/485
Eukaryota	Fungi	Candida albicans WO-1	EEQ46701.1	6 E-40	411/485
Eukaryota	Fungi	Saccharomyces cerevisiae YJM789	EDN60484.1	2 E-39	431/485
Eukaryota	Fungi	Pichia guilliermondii ATCC 6260	EDK37827.2	1 E-38	475/485
Eukaryota	Fungi	Candida glabrata CBS 138	XP_445771.1	1 E-38	415/485
Eukaryota	Fungi	Candida glabrata CBS 138	XP_447804.1	2 E-38	396/485
Eukaryota	Fungi	Candida glabrata CBS 138	XP_445770.1	3 E-38	424/485
Eukaryota	Fungi	Candida glabrata CBS 138	XP_445768.1	2 E-37	428/485
Eukaryota	Fungi	Debaryomyces hansenii	CAR65599.1	3 E-37	453/485
Eukaryota	Fungi	Pichia guilliermondii ATCC 6260	EDK35904.2	4 E-37	451/485
Eukaryota	Fungi	Candida glabrata CBS 138	XP_445769.1	7 E-37	470/485
Eukaryota	Fungi	Pichia stipitis CBS 6054	XP_001385957.2	1 E-36	401/485

Eukaryota	Fungi	Candida dubliniensis CD36	XP_002419429.1	2 E-36	400/485
Eukaryota	Fungi	Pichia guilliermondii ATCC 6260	XP_001486625.1	4 E-36	451/485
Eukaryota	Fungi	Yarrowia lipolytica CLIB122	XP_504725.1	8 E-35	392/485
Eukaryota	Fungi	Pichia guilliermondii ATCC 6260	XP_001486254.1	5 E-34	475/485
Eukaryota	Fungi	Penicillium chrysogenum Wisconsin 54-1255	XP_002564956.1	7 E-34	395/485
Eukaryota	Fungi	Hypocrea lixii	ABK64120.1	4 E-33	392/485
Eukaryota	Fungi	Magnaporthe grisea 70-15	XP_001406951.1	3 E-31	390/485

AFUA_6G06350

Eukaryota	Fungi	Aspergillus fumigatus Af293	XP_750527.1	1 E-157	278/278
Eukaryota	Fungi	Neosartorya fischeri NRRL 181	XP_001257750.1	1 E-146	278/278
Eukaryota	Fungi	Aspergillus clavatus NRRL 1	XP_001272550.1	1 E-136	277/278
Eukaryota	Fungi	Aspergillus terreus NIH2624	XP_001216026.1	1 E-127	290/278
Eukaryota	Fungi	Aspergillus nidulans FGSC A4	XP_663397.1	1 E-127	277/278
Eukaryota	Fungi	Penicillium chrysogenum Wisconsin 54-1255	XP_002560108.1	1 E-127	253/278
Eukaryota	Fungi	Aspergillus flavus NRRL3357	XP_002375302.1	1 E-126	277/278
Eukaryota	Fungi	Aspergillus oryzae RIB40	XP_001819247.1	1 E-126	277/278
Eukaryota	Fungi	Aspergillus niger CBS 513.88	XP_001399133.1	1 E-124	253/278
Eukaryota	Fungi	Uncinocarpus reesii 1704	XP_002583051.1	1 E-122	274/278
Eukaryota	Fungi	Microsporum canis CBS 113480	EEQ34459.1	1 E-121	254/278
Eukaryota	Fungi	Ajellomyces capsulatus NAm1	XP_001540250.1	1 E-120	256/278
Eukaryota	Fungi	Ajellomyces dermatitidis SLH14081	XP_002627037.1	1 E-119	254/278
Eukaryota	Fungi	Paracoccidioides brasiliensis Pb18;	EEH47898.1	1 E-119	254/278
Eukaryota	Fungi	Paracoccidioides brasiliensis Pb03;	EEH19067.1	1 E-118	253/278
Eukaryota	Fungi	Phaeosphaeria nodorum SN15	XP_001794484.1	1 E-118	256/278
Eukaryota	Fungi	Talaromyces stipitatus ATCC 10500	XP_002480789.1	1 E-117	238/278
Eukaryota	Fungi	Coccidioides immitis RS;	XP_001243364.1	1 E-116	254/278
Eukaryota	Fungi	Coccidioides posadasii C735 delta	EER24725.1	1 E-116	254/278
Eukaryota	Fungi	Paracoccidioides brasiliensis Pb01;	EEH37931.1	1 E-116	307/278
Eukaryota	Fungi	Penicillium marneffei ATCC 18224	XP_002151821.1	1 E-115	238/278
Eukaryota	Fungi	Magnaporthe grisea 70-15	XP_367823.1	1 E-113	256/278
Eukaryota	Fungi	Podospora anserina DSM 980	XP_001912669.1	1 E-112	254/278
Eukaryota	Fungi	Pyrenophora tritici-repentis Pt-1C-BFP	XP_001939049.1	1 E-112	256/278
Eukaryota	Fungi	Sclerotinia sclerotiorum 1980 UF-70	XP_001586244.1	1 E-111	254/278
Eukaryota	Fungi	Botryotinia fuckeliana B05.10	XP_001553547.1	1 E-110	277/278
Eukaryota	Fungi	Neurospora crassa OR74A	XP_965087.1	1 E-105	276/278
Eukaryota	Fungi	Nectria haematococca mpVI 77-13-4	EEU48568.1	1 E-104	250/278
Eukaryota	Fungi	Ajellomyces capsulatus H143	EER36721.1	1 E-104	229/278
Eukaryota	Fungi	Gibberella zeae PH-1	XP_380740.1	1 E-102	247/278
Eukaryota	Fungi	Verticillium albo-atrum VaMs.102	EEY18916.1	1 E-88	233/278
Eukaryota	Fungi	Yarrowia lipolytica CLIB122	XP_505120.1	2 E-80	231/278
Eukaryota	Fungi	Chaetomium globosum CBS 148.51	XP_001220368.1	3 E-80	270/278
Eukaryota	Fungi	Kluyveromyces lactis	CAH02741.2	5 E-79	225/278
Eukaryota	Fungi	Ashbya gossypii ATCC 10895	NP_984496.1	4 E-75	253/278
Eukaryota	Fungi	Pichia guilliermondii ATCC 6260	XP_001481983.1	1 E-74	227/278
Eukaryota	Fungi	Schizosaccharomyces pombe	NP_588040.1	3 E-74	229/278
Eukaryota	Fungi	Saccharomyces cerevisiae	NP_015007.1	9 E-74	228/278
Eukaryota	Fungi	Zygosaccharomyces rouxii CBS 732	XP_002495696.1	4 E-73	228/278
Eukaryota	Fungi	Candida glabrata CBS 138	XP_447060.1	4 E-73	227/278
Eukaryota	Fungi	Schizosaccharomyces japonicus yFS275	XP_002172045.1	3 E-72	231/278
Eukaryota	Fungi	Ustilago maydis 521	XP_760923.1	1 E-68	230/278
Eukaryota	Fungi	Vanderwaltozyma polyspora DSM 70294	XP_001645138.1	5 E-68	227/278
Eukaryota	Metazoa	Bombyx mori	NP_001040387.1	6 E-68	234/278
Eukaryota	Metazoa	Ixodes scapularis	XP_002435379.1	1 E-66	232/278
Eukaryota	Metazoa	Taeniopygia guttata	XP_002200417.1	1 E-66	235/278
Eukaryota	Metazoa	Gallus gallus	NP_001006491.1	2 E-66	234/278
Eukaryota	Metazoa	Rattus norvegicus	CAE48381.1	2 E-66	234/278

Eukaryota	Metazoa	Strongylocentrotus purpuratus	XP_796247.2	3 E-66	238/278
Eukaryota	Metazoa	Pediculus humanus corporis	XP_002432892.1	5 E-66	233/278
Eukaryota	Metazoa	Mus musculus	NP_035314.3	9 E-66	234/278
Eukaryota	Metazoa	Mus musculus	EDL36567.1	1 E-65	234/278
Eukaryota	Metazoa	Mus musculus	BAB22424.1	2 E-65	234/278
Eukaryota	Metazoa	Papilio xuthus	BAG30797.1	2 E-65	234/278
Eukaryota	Metazoa	Rattus norvegicus	NP_058976.1	2 E-65	234/278
Eukaryota	Metazoa	Equus caballus	XP_001496754.1	3 E-65	234/278
Eukaryota	Metazoa	Sus scrofa	XP_001928025.1	3 E-65	234/278
Eukaryota	Metazoa	Homo sapiens	AAH29402.1	4 E-65	234/278
Eukaryota	Metazoa	Canis lupus familiaris	XP_537460.2	5 E-65	234/278
Eukaryota	Metazoa	Nematostella vectensis	XP_001628601.1	7 E-65	233/278
Eukaryota	Metazoa	Homo sapiens	NP_002779.1	8 E-65	234/278
Eukaryota	Metazoa	Monodelphis domestica	XP_001369098.1	8 E-65	234/278
Eukaryota	Metazoa	Homo sapiens	CAG33214.1	8 E-65	234/278
Eukaryota	Metazoa	Bos taurus	NP_001029407.1	1 E-64	234/278
Eukaryota	Amoebozoa	Acanthamoeba castellanii	P90513.1	1 E-64	223/278
Eukaryota	Viridiplantae	Populus trichocarpa	XP_002299648.1	4 E-64	229/278
Eukaryota	Viridiplantae	Populus trichocarpa	XP_002314241.1	8 E-64	229/278
Eukaryota	Viridiplantae	Medicago truncatula	ACJ84360.1	3 E-63	228/278
Eukaryota	Metazoa	Sus scrofa	XP_001928042.1	3 E-63	227/278
Eukaryota	Metazoa	Equus caballus	XP_001496772.1	4 E-63	227/278
Eukaryota	Metazoa	Canis lupus familiaris	XP_864852.1	4 E-63	227/278
Eukaryota	Metazoa	Rattus norvegicus	EDM05372.1	5 E-63	227/278
Eukaryota	Viridiplantae	Arabidopsis thaliana	NP_180270.1	6 E-63	229/278
Eukaryota	Metazoa	Homo sapiens	NP_687033.1	7 E-63	227/278
Eukaryota	Viridiplantae	Arabidopsis thaliana	CAA74027.1	7 E-63	229/278
Eukaryota	Viridiplantae	Zea mays;	ACN34897.1	7 E-63	229/278
Eukaryota	Viridiplantae	Zea mays;	NP_001150029.1	2 E-62	229/278
Eukaryota	Metazoa	Pan troglodytes	XP_001164624.1	2 E-62	233/278
Eukaryota	Metazoa	Trichoplax adhaerens	XP_002117216.1	3 E-62	232/278
Eukaryota	Metazoa	Tribolium castaneum	XP_969972.1	7 E-62	232/278
Eukaryota	Metazoa	Homo sapiens	EAW80717.1	1 E-61	228/278
Eukaryota	Viridiplantae	Oryza sativa Japonica Group	NP_001044588.1	7 E-61	229/278
Eukaryota	Viridiplantae	Zea mays;	ACG48183.1	7 E-61	229/278
Eukaryota	Metazoa	Euprymna scolopes	AAT36639.1	1 E-60	232/278
Eukaryota	Viridiplantae	Oryza sativa Japonica Group	AAT69640.1	2 E-60	229/278
Eukaryota	Metazoa	Apis mellifera	XP_392518.1	2 E-60	233/278
Eukaryota	Metazoa	Mus musculus	EDK98066.1	3 E-60	231/278
Eukaryota	Metazoa	Mus musculus	XP_001477646.1	5 E-60	228/278
Eukaryota	Viridiplantae	Spinacia oleracea	O24362.1	5 E-60	229/278
Eukaryota	Metazoa	Culex quinquefasciatus	XP_001845254.1	6 E-60	232/278
Eukaryota	Metazoa	Ciona intestinalis	XP_002132081.1	1 E-59	231/278
Eukaryota	Metazoa	Aedes aegypti	XP_001664262.1	4 E-59	232/278
Eukaryota	Amoebozoa	Dictyostelium discoideum AX4	XP_647103.1	9 E-59	224/278
Eukaryota	Metazoa	Schistosoma japonicum	CAX70764.1	3 E-58	233/278
Eukaryota	Metazoa	Schistosoma mansoni	XP_002569974.1	4 E-58	233/278
Eukaryota	Fungi	Moniliophthora perniciosa FA553	XP_002389422.1	9 E-58	225/278
Eukaryota	Metazoa	Ornithorhynchus anatinus	XP_001516758.1	9 E-58	234/278
Eukaryota	Amoebozoa	Dictyostelium discoideum	AAB03671.1	2 E-57	224/278
Eukaryota	Viridiplantae	Picea sitchensis	ABK22658.1	7 E-57	229/278
Eukaryota	Viridiplantae	Oryza sativa Japonica Group	NP_001055904.1	6 E-56	256/278
Eukaryota	Metazoa	Acyrthosiphon pisum	NP_001156106.1	9 E-56	227/278
Eukaryota	Metazoa	Acyrthosiphon pisum	XP_001950162.1	2 E-55	227/278
Eukaryota	Viridiplantae	Chlamydomonas reinhardtii	XP_001690302.1	4 E-55	229/278
Eukaryota	Metazoa	Clonorchis sinensis	ABK91802.1	7 E-55	233/278
Eukaryota	Viridiplantae	Micromonas pusilla CCMP1545	EEH60821.1	2 E-54	231/278
Eukaryota	Viridiplantae	Micromonas sp. RCC299	XP_002503561.1	4 E-54	228/278

Eukaryota	Alveolata	Alexandrium fundyense	ABO47863.1	2 E-53	234/278
Eukaryota	Metazoa	Drosophila sechellia	XP_002045330.1	3 E-51	231/278
Eukaryota	Metazoa	Drosophila melanogaster	NP_724834.1	4 E-51	231/278
Eukaryota	Metazoa	Drosophila yakuba	XP_002089851.1	3 E-50	231/278
Eukaryota	Metazoa	Drosophila persimilis	XP_002026016.1	3 E-50	232/278
Eukaryota	Metazoa	Lepeophtheirus salmonis	ACO12975.1	4 E-50	234/278
Eukaryota	stramenopiles	Thalassiosira pseudonana CCMP1335	XP_002287079.1	6 E-50	237/278
Eukaryota	Metazoa	Lepeophtheirus salmonis	ACO12968.1	1 E-49	234/278
Eukaryota	Metazoa	Drosophila erecta	XP_001969141.1	2 E-49	231/278
Eukaryota	Metazoa	Caligus rogercresseyi	ACO10381.1	3 E-49	225/278
Eukaryota	Metazoa	Acyrthosiphon pisum	XP_001945150.1	4 E-49	225/278
Eukaryota	Alveolata	Perkinsus marinus ATCC 50983	EER19220.1	4 E-49	239/278
Eukaryota	Metazoa	Brugia malayi	XP_001894710.1	9 E-49	230/278
Eukaryota	stramenopiles	Phaeodactylum tricornutum CCAP 1055/1	XP_002184927.1	2 E-48	238/278
Eukaryota	Metazoa	Drosophila ananassae	XP_001959982.1	5 E-48	231/278
Eukaryota	Metazoa	Drosophila ananassae	XP_001961771.1	7 E-48	231/278
Eukaryota	Metazoa	Drosophila mojavensis	XP_002005919.1	2 E-47	232/278
Eukaryota	Metazoa	Drosophila willistoni	XP_002061564.1	8 E-47	228/278
Eukaryota	Choanoflagellida	Monosiga brevicollis MX1	XP_001748847.1	1 E-46	254/278
Eukaryota	Alveolata	Paramecium tetraurelia strain d4-2	XP_001436797.1	1 E-46	227/278
Eukaryota	Alveolata	Paramecium tetraurelia strain d4-2	XP_001437849.1	9 E-46	225/278
Eukaryota	Metazoa	Drosophila grimshawi	XP_001986373.1	1 E-45	232/278
Eukaryota	Metazoa	Tetraodon nigroviridis	CAF89630.1	2 E-43	241/278
Eukaryota	Metazoa	Drosophila virilis	XP_002049923.1	2 E-43	231/278
Eukaryota	Metazoa	Caenorhabditis elegans	NP_496177.2	6 E-43	230/278
Eukaryota	Metazoa	Caenorhabditis briggsae AF16	XP_001678494.1	7 E-41	229/278
Eukaryota	Alveolata	Toxoplasma gondii ME49	XP_002370955.1	2 E-35	240/278
Eukaryota	Alveolata	Toxoplasma gondii GT1	EEE20214.1	6 E-35	240/278
Eukaryota	Alveolata	Tetrahymena thermophila	XP_001032923.2	7 E-32	226/278
Eukaryota	Alveolata	Babesia bovis T2Bo	XP_001610702.1	4 E-30	231/278
Eukaryota	Alveolata	Theileria annulata strain Ankara	XP_954982.1	4 E-29	235/278
Eukaryota	Alveolata	Plasmodium berghei str. ANKA	XP_676733.1	1 E-26	224/278
Eukaryota	Alveolata	Plasmodium knowlesi strain H	XP_002258889.1	3 E-26	233/278
Eukaryota	Diplomonadida	Giardia lamblia ATCC 50803	XP_001706995.1	8 E-26	230/278

AFUA_6G08630

Eukaryota	Fungi	Aspergillus fumigatus Af293	XP_750752.1	1 E-141	247/247
Eukaryota	Fungi	Neosartorya fischeri NRRL 181	XP_001257979.1	1 E-127	246/247
Eukaryota	Fungi	Aspergillus clavatus NRRL 1	XP_001268109.1	1 E-112	240/247
Eukaryota	Fungi	Aspergillus flavus NRRL3357	XP_002382018.1	1 E-109	244/247
Eukaryota	Fungi	Aspergillus oryzae RIB40	XP_001819035.1	1 E-109	244/247
Eukaryota	Fungi	Penicillium chrysogenum Wisconsin 54-1255	XP_002557955.1	1 E-108	252/247
Eukaryota	Fungi	Aspergillus terreus NIH2624	XP_001215782.1	1 E-105	245/247
Eukaryota	Fungi	Aspergillus niger CBS 513.88	XP_001394556.1	1 E-104	252/247
Eukaryota	Fungi	Talaromyces stipitatus ATCC 10500	XP_002340825.1	5 E-94	246/247
Eukaryota	Fungi	Penicillium marneffei ATCC 18224	XP_002144673.1	4 E-93	246/247
Eukaryota	Fungi	Coccidioides posadasii C735 delta	EER23913.1	2 E-77	254/247
Eukaryota	Fungi	Coccidioides immitis RS;	XP_001247314.1	7 E-77	254/247
Eukaryota	Fungi	Uncinocarpus reesii 1704	XP_002541507.1	2 E-74	258/247
Eukaryota	Fungi	Aspergillus nidulans FGSC A4	XP_868894.1	3 E-72	226/247
Eukaryota	Fungi	Ajellomyces dermatitidis SLH14081	XP_002624007.1	2 E-64	217/247
Eukaryota	Fungi	Ajellomyces dermatitidis ER-3	EEQ87619.1	3 E-64	212/247
Eukaryota	Fungi	Ajellomyces capsulatus G186AR	EEH11482.1	1 E-63	222/247
Eukaryota	Fungi	Microsporum canis CBS 113480	EEQ31700.1	7 E-63	235/247
Eukaryota	Fungi	Ajellomyces capsulatus NAm1	XP_001541645.1	7 E-62	219/247
Eukaryota	Fungi	Ajellomyces capsulatus H143	EER39298.1	1 E-61	216/247
Eukaryota	Fungi	Paracoccidioides brasiliensis Pb18;	EEH47636.1	1 E-58	225/247

Eukaryota	Fungi	Paracoccidioides brasiliensis Pb01;	EEH38190.1	1 E-58	223/247
Eukaryota	Fungi	Paracoccidioides brasiliensis Pb03;	EEH19368.1	2 E-58	225/247
Eukaryota	Fungi	Sclerotinia sclerotiorum 1980 UF-70	XP_001597101.1	8 E-57	224/247
Eukaryota	Fungi	Magnaporthe grisea 70-15	XP_368756.1	4 E-55	254/247
Eukaryota	Fungi	Botryotinia fuckeliana B05.10	XP_001549091.1	3 E-54	224/247
Eukaryota	Fungi	Phaeosphaeria nodorum SN15	XP_001805911.1	2 E-53	244/247
Eukaryota	Fungi	Podospora anserina DSM 980	XP_001908720.1	5 E-52	262/247
Eukaryota	Fungi	Verticillium albo-atrum VaMs.102	EEY18463.1	3 E-50	255/247
Eukaryota	Fungi	Nectria haematococca mpVI 77-13-4	EEU46463.1	3 E-49	249/247
Eukaryota	Fungi	Neurospora crassa OR74A	XP_964591.1	2 E-47	261/247
Eukaryota	Fungi	Pyrenophora tritici-repentis Pt-1C-BFP	XP_001933532.1	5 E-47	220/247
Eukaryota	Fungi	Gibberella zeae PH-1	XP_387040.1	1 E-46	229/247
Eukaryota	Fungi	Debaryomyces hansenii	CAG87883.2	4 E-11	233/247

AFUA_6G08640

Eukaryota	Fungi	Aspergillus fumigatus Af293	XP_750753.1	0.0	801/801
Eukaryota	Fungi	Neosartorya fischeri NRRL 181	XP_001257980.1	0.0	801/801
Eukaryota	Fungi	Aspergillus clavatus NRRL 1	XP_001268108.1	0.0	801/801
Eukaryota	Fungi	Aspergillus oryzae RIB40	XP_001819036.1	0.0	800/801
Eukaryota	Fungi	Aspergillus flavus NRRL3357	XP_002382019.1	0.0	800/801
Eukaryota	Fungi	Aspergillus terreus NIH2624	Q0CI79.2	0.0	798/801
Eukaryota	Fungi	Aspergillus terreus NIH2624	XP_001215783.1	0.0	795/801
Eukaryota	Fungi	Aspergillus niger CBS 513.88	XP_001394557.1	0.0	798/801
Eukaryota	Fungi	Penicillium chrysogenum Wisconsin 54-1255	XP_002557954.1	0.0	795/801
Eukaryota	Fungi	Penicillium marneffei ATCC 18224	XP_002144672.1	0.0	797/801
Eukaryota	Fungi	Talaromyces stipitatus ATCC 10500	XP_002340824.1	0.0	786/801
Eukaryota	Fungi	Microsporum canis CBS 113480	EEQ31701.1	0.0	792/801
Eukaryota	Fungi	Coccidioides posadasii C735 delta	EER23914.1	0.0	793/801
Eukaryota	Fungi	Coccidioides immitis RS;	XP_001247313.1	0.0	793/801
Eukaryota	Fungi	Ajellomyces dermatitidis ER-3	EEQ87620.1	0.0	794/801
Eukaryota	Fungi	Ajellomyces dermatitidis SLH14081	XP_002624006.1	0.0	794/801
Eukaryota	Fungi	Paracoccidioides brasiliensis Pb01;	EEH38189.1	0.0	798/801
Eukaryota	Fungi	Ajellomyces capsulatus G186AR	EEH11481.1	0.0	795/801
Eukaryota	Fungi	Paracoccidioides brasiliensis Pb18;	EEH47637.1	0.0	798/801
Eukaryota	Fungi	Paracoccidioides brasiliensis Pb03;	EEH19367.1	0.0	798/801
Eukaryota	Fungi	Sclerotinia sclerotiorum 1980 UF-70	A7E7L8.2	0.0	783/801
Eukaryota	Fungi	Botryotinia fuckeliana B05.10	A6SHZ5.2	0.0	760/801
Eukaryota	Fungi	Phaeosphaeria nodorum SN15	XP_001805910.1	0.0	775/801
Eukaryota	Fungi	Magnaporthe grisea 70-15	XP_368757.1	0.0	803/801
Eukaryota	Fungi	Neurospora crassa OR74A	XP_964019.1	0.0	794/801
Eukaryota	Fungi	Nectria haematococca mpVI 77-13-4	EEU46016.1	0.0	776/801
Eukaryota	Fungi	Podospora anserina DSM 980	XP_001908719.1	0.0	783/801
Eukaryota	Fungi	Gibberella zeae PH-1	XP_387041.1	0.0	777/801
Eukaryota	Fungi	Chaetomium globosum CBS 148.51	XP_001220207.1	0.0	760/801
Eukaryota	Fungi	Yarrowia lipolytica CLIB122	XP_499926.1	1 E-166	736/801
Eukaryota	Fungi	Lachancea thermotolerans CBS 6340	XP_002552928.1	1 E-148	747/801
Eukaryota	Fungi	Candida glabrata CBS 138	XP_445506.1	1 E-146	716/801
Eukaryota	Fungi	Vanderwaltozyma polyspora DSM 70294	XP_001642605.1	1 E-145	737/801
Eukaryota	Fungi	Kluyveromyces lactis NRRL Y-1140	XP_452082.1	1 E-143	734/801
Eukaryota	Fungi	Coprinopsis cinerea okayama7#130	XP_001829155.1	1 E-141	760/801
Eukaryota	Fungi	Coprinopsis scobicola	Q6Y5M7.1	1 E-141	751/801
Eukaryota	Fungi	Zygosaccharomyces rouxii CBS 732	XP_002498853.1	1 E-140	742/801
Eukaryota	Fungi	Pleurotus djamor	Q6Y5M5.1	1 E-139	720/801
Eukaryota	Fungi	Saccharomyces cerevisiae YJM789	A6ZZI7.1	1 E-139	728/801
Eukaryota	Fungi	Saccharomyces cerevisiae RM11-1a	EDV12969.1	1 E-139	728/801
Eukaryota	Fungi	Saccharomyces cerevisiae EC1118	CAY80958.1	1 E-139	728/801
Eukaryota	Fungi	Saccharomyces cerevisiae	P35999.2	1 E-139	728/801

Eukaryota	Fungi	Malassezia globosa CBS 7966	A8QB25.2	1 E-138	778/801
Eukaryota	Fungi	Ashbya gossypii ATCC 10895	NP_985745.1	1 E-137	732/801
Eukaryota	Fungi	Pholiota nameko	BAH22601.1	1 E-135	718/801
Eukaryota	Fungi	Postia placenta Mad-698-R	XP_002469602.1	1 E-134	757/801
Eukaryota	Fungi	Postia placenta Mad-698-R	XP_002475688.1	1 E-133	748/801
Eukaryota	Fungi	Coprinellus disseminatus	Q6Y5M6.1	1 E-133	701/801
Eukaryota	Fungi	Pichia pastoris GS115	XP_002491343.1	1 E-133	783/801
Eukaryota	Fungi	Schizosaccharomyces pombe	NP_593013.1	1 E-132	726/801
Eukaryota	Fungi	Laccaria bicolor S238N-H82	XP_001874001.1	1 E-131	725/801
Eukaryota	Fungi	Leucoagaricus gongylophorus	Q6VMB4.1	1 E-131	722/801
Eukaryota	Fungi	Candida albicans SC5314	XP_712456.1	1 E-130	771/801
Eukaryota	Fungi	Cryptococcus neoformans var. neoformans	XP_569591.1	1 E-130	716/801
Eukaryota	Fungi	Cryptococcus neoformans var. neoformans	XP_776864.1	1 E-130	716/801
Eukaryota	Fungi	Candida dubliniensis CD36	XP_002420831.1	1 E-128	773/801
Eukaryota	Fungi	Saccharomyces cerevisiae	NP_012788.1	1 E-128	726/801
Eukaryota	Fungi	Pichia stipitis CBS 6054	XP_001384676.2	1 E-127	751/801
Eukaryota	Fungi	Debaryomyces hansenii	Q6BJ61.2	1 E-127	757/801
Eukaryota	Fungi	Schizosaccharomyces japonicus yFS275	XP_002172272.1	1 E-126	718/801
Eukaryota	Fungi	Debaryomyces hansenii CBS767	XP_461760.1	1 E-126	757/801
Eukaryota	Fungi	Pichia guilliermondii	A5DI46.2	1 E-126	774/801
Eukaryota	Fungi	Filobasidiella neoformans	Q5KMC8.2	1 E-125	770/801
Eukaryota	Fungi	Schizophyllum commune	P37932.2	1 E-125	719/801
Eukaryota	Fungi	Pichia guilliermondii ATCC 6260	XP_001485218.1	1 E-124	774/801
Eukaryota	Fungi	Ustilago maydis 521	XP_758582.1	1 E-124	844/801
Eukaryota	Fungi	Ustilago maydis	Q4PBS8.2	1 E-124	844/801
Eukaryota	Fungi	Candida tropicalis MYA-3404	XP_002548706.1	1 E-122	766/801
Eukaryota	Fungi	Malassezia globosa CBS 7966	XP_001729086.1	1 E-122	646/801
Eukaryota	Fungi	Cryptococcus neoformans var. neoformans	XP_569144.1	1 E-122	708/801
Eukaryota	Fungi	Clavispora lusitaniae ATCC 42720	XP_002618100.1	1 E-118	739/801
Eukaryota	Fungi	Lodderomyces elongisporus NRRL YB-4239	XP_001523832.1	1 E-113	728/801
Eukaryota	Metazoa	Apis mellifera	XP_396237.3	5 E-90	648/801
Eukaryota	Viridiplantae	Ostreococcus lucimarinus CCE9901	XP_001420059.1	7 E-84	657/801
Eukaryota	Metazoa	Acyrthosiphon pisum	XP_001950913.1	2 E-79	661/801
Eukaryota	Viridiplantae	Physcomitrella patens subsp. patens	XP_001768859.1	2 E-72	656/801
Eukaryota	Viridiplantae	Sorghum bicolor;	XP_002437475.1	4 E-72	674/801
Eukaryota	Viridiplantae	Populus trichocarpa	XP_002321840.1	5 E-72	681/801
Eukaryota	Viridiplantae	Ricinus communis	XP_002510927.1	2 E-71	664/801
Eukaryota	Viridiplantae	Vitis vinifera	XP_002265587.1	4 E-69	664/801
Eukaryota	Metazoa	Schistosoma mansoni	XP_002578698.1	3 E-66	652/801
Eukaryota	stramenopiles	Phaeodactylum tricornutum CCAP 1055/1	XP_002185286.1	3 E-66	681/801
Bacteria	Bacteroidetes	Prevotella melaninogenica ATCC 25845	ZP_04833277.1	8 E-47	666/801
Bacteria	Proteobacteria	Nitrosomonas europaea ATCC 19718	NP_841697.1	2 E-46	656/801
Bacteria	Bacteroidetes	Robiginitalea biformata HTCC2501	YP_003194952.1	1 E-44	643/801

AFUA_6G08650

Eukaryota	Fungi	Aspergillus fumigatus Af293	XP_750754.1	1 E-135	236/236
Eukaryota	Fungi	Neosartorya fischeri NRRL 181	XP_001257981.1	1 E-75	241/236

AFUA_6G08660

Eukaryota	Fungi	Aspergillus fumigatus Af293	XP_750755.1	0.0	1239/1239
Eukaryota	Fungi	Neosartorya fischeri NRRL 181	XP_001257982.1	0.0	1151/1239
Eukaryota	Fungi	Aspergillus clavatus NRRL 1	XP_001268106.1	0.0	1134/1239
Eukaryota	Fungi	Aspergillus flavus NRRL3357	XP_002382021.1	0.0	1163/1239
Eukaryota	Fungi	Aspergillus oryzae RIB40	XP_001819037.1	0.0	1163/1239
Eukaryota	Fungi	Aspergillus terreus NIH2624	XP_001215785.1	0.0	1138/1239
Eukaryota	Fungi	Aspergillus nidulans FGSC A4	XP_661510.1	0.0	1102/1239

Eukaryota	Fungi	Penicillium chrysogenum Wisconsin 54-1255	XP_002557945.1	0.0	1207/1239
Eukaryota	Fungi	Aspergillus niger CBS 513.88	XP_001394559.1	0.0	1283/1239
Eukaryota	Fungi	Penicillium marneffei ATCC 18224	XP_002145890.1	1 E-154	1189/1239
Eukaryota	Fungi	Talaromyces stipitatus ATCC 10500	XP_002478151.1	1 E-140	1196/1239
Eukaryota	Fungi	Coccidioides posadasii C735 delta	EER23683.1	1 E-131	1222/1239
Eukaryota	Fungi	Ajellomyces capsulatus H143	EER41510.1	1 E-129	1225/1239
Eukaryota	Fungi	Ajellomyces capsulatus G186AR	EEH05646.1	1 E-128	1225/1239
Eukaryota	Fungi	Coccidioides immitis RS;	XP_001247598.1	1 E-124	1187/1239
Eukaryota	Fungi	Uncinocarpus reesii 1704	XP_002541780.1	2 E-96	1153/1239
Eukaryota	Fungi	Microsporum canis CBS 113480	EEQ27654.1	6 E-94	1120/1239
Eukaryota	Fungi	Gibberella zeae PH-1	XP_389403.1	2 E-26	1120/1239
Eukaryota	Fungi	Chaetomium globosum CBS 148.51	XP_001226838.1	2 E-22	1127/1239

AFUA_6G09500

Eukaryota	Fungi	Aspergillus fumigatus Af293	XP_750839.1	1 E-138	241/241
Eukaryota	Fungi	Neosartorya fischeri NRRL 181	XP_001258063.1	9 E-91	197/241
Eukaryota	Fungi	Aspergillus niger CBS 513.88	XP_001395752.1	2 E-60	220/241
Eukaryota	Fungi	Aspergillus niger CBS 513.88	XP_001390781.1	5 E-60	218/241
Eukaryota	Fungi	Coccidioides immitis RS;	XP_001244683.1	1 E-18	193/241
Eukaryota	Fungi	Coccidioides posadasii C735 delta	EER26117.1	5 E-18	193/241
Eukaryota	Fungi	Uncinocarpus reesii 1704	XP_002585061.1	1 E-16	243/241

AFUA_6G09570

Eukaryota	Fungi	Aspergillus fumigatus Af293	XP_750846.1	0.0	384/384
Eukaryota	Fungi	Penicillium chrysogenum Wisconsin 54-1255	XP_002562731.1	5 E-48	350/384
Eukaryota	Fungi	Aspergillus flavus NRRL3357	XP_002372190.1	4 E-45	352/384
Eukaryota	Fungi	Aspergillus oryzae RIB40	XP_001817154.1	4 E-45	352/384

AFUA_6G09580

Eukaryota	Fungi	Aspergillus fumigatus Af293	XP_750847.2	0.0	499/499
Eukaryota	Fungi	Aspergillus nidulans FGSC A4	CBF70180.1	1 E-110	480/499
Eukaryota	Fungi	Aspergillus nidulans FGSC A4	XP_663706.1	1 E-110	480/499
Eukaryota	Fungi	Aspergillus terreus NIH2624	XP_001209820.1	1 E-106	448/499
Eukaryota	Fungi	Aspergillus flavus NRRL3357	XP_002376851.1	9 E-74	496/499
Eukaryota	Fungi	Penicillium marneffei ATCC 18224	XP_002143233.1	2 E-68	503/499
Eukaryota	Fungi	Penicillium chrysogenum Wisconsin 54-1255	XP_002561475.1	6 E-64	498/499
Eukaryota	Fungi	Talaromyces stipitatus ATCC 10500	XP_002481664.1	4 E-63	503/499
Eukaryota	Fungi	Neosartorya fischeri NRRL 181	XP_001258300.1	6 E-62	471/499
Eukaryota	Fungi	Aspergillus nidulans FGSC A4	XP_659958.1	1 E-56	472/499
Eukaryota	Fungi	Talaromyces stipitatus ATCC 10500	XP_002485725.1	8 E-47	401/499
Eukaryota	Fungi	Talaromyces stipitatus ATCC 10500	XP_002481840.1	7 E-18	412/499
Eukaryota	Fungi	Aspergillus flavus NRRL3357	XP_002384736.1	2 E-16	415/499
Eukaryota	Fungi	Aspergillus oryzae RIB40	XP_001827524.1	3 E-16	415/499
Eukaryota	Fungi	Ajellomyces capsulatus H143	EER42062.1	2 E-15	400/499
Eukaryota	Fungi	Nectria haematococca mpVI 77-13-4	EEU45821.1	2 E-14	434/499
Eukaryota	Fungi	Gibberella zeae PH-1	XP_386500.1	6 E-14	438/499
Eukaryota	Fungi	Nectria haematococca mpVI 77-13-4	EEU35050.1	2 E-12	421/499

AFUA_6G09590

Eukaryota	Fungi	Aspergillus fumigatus Af293	XP_750848.1	0.0	337/337
Eukaryota	Fungi	Neosartorya fischeri NRRL 181	XP_001258074.1	1 E-176	330/337
Eukaryota	Fungi	Aspergillus clavatus NRRL 1	XP_001268039.1	1 E-138	330/337
Eukaryota	Fungi	Penicillium chrysogenum Wisconsin 54-1255	XP_002557601.1	1 E-124	334/337
Eukaryota	Fungi	Aspergillus terreus NIH2624	XP_001215877.1	1 E-112	318/337

Eukaryota	Fungi	Aspergillus oryzae RIB40	XP_001727394.1	1 E-110	333/337
Eukaryota	Fungi	Aspergillus flavus NRRL3357	XP_002375649.1	1 E-110	336/337
Eukaryota	Fungi	Aspergillus niger CBS 513.88	XP_001394744.1	8 E-98	333/337
Eukaryota	Fungi	Talaromyces stipitatus ATCC 10500	XP_002477977.1	1 E-91	333/337
Eukaryota	Fungi	Aspergillus nidulans FGSC A4	XP_680796.1	2 E-91	349/337
Eukaryota	Fungi	Aspergillus nidulans FGSC A4	CBF79566.1	3 E-91	349/337
Eukaryota	Fungi	Podospora anserina DSM 980	XP_001903375.1	8 E-91	335/337
Eukaryota	Fungi	Penicillium marneffei ATCC 18224	XP_002145726.1	1 E-88	335/337
Eukaryota	Fungi	Nectria haematococca mpVI 77-13-4	EEU34257.1	3 E-80	333/337
Eukaryota	Fungi	Pyrenophora tritici-repentis Pt-1C-BFP	XP_001936927.1	4 E-75	337/337
Eukaryota	Fungi	Phaeosphaeria nodorum SN15	XP_001802687.1	6 E-75	319/337
Eukaryota	Fungi	Neurospora crassa OR74A	XP_959388.1	1 E-74	344/337
Eukaryota	Fungi	Aspergillus terreus NIH2624	XP_001215387.1	3 E-72	347/337
Eukaryota	Fungi	Gibberella zeae PH-1	XP_388301.1	2 E-68	336/337
Eukaryota	Fungi	Pyrenophora tritici-repentis Pt-1C-BFP	XP_001936340.1	2 E-68	333/337
Eukaryota	Fungi	Nectria haematococca mpVI 77-13-4	EEU43570.1	4 E-68	338/337
Eukaryota	Fungi	Nectria haematococca mpVI 77-13-4	EEU36918.1	7 E-68	340/337
Eukaryota	Fungi	Phaeosphaeria nodorum SN15	XP_001800222.1	3 E-67	332/337
Eukaryota	Fungi	Gibberella zeae PH-1	XP_384089.1	6 E-67	337/337
Eukaryota	Fungi	Nectria haematococca mpVI 77-13-4	EEU35890.1	3 E-64	330/337
Eukaryota	Fungi	Phaeosphaeria nodorum SN15	XP_001802812.1	1 E-61	335/337
Eukaryota	Fungi	Aspergillus flavus NRRL3357	XP_002377526.1	2 E-60	332/337
Eukaryota	Fungi	Magnaporthe grisea 70-15	XP_364162.1	2 E-59	335/337
Eukaryota	Fungi	Verticillium albo-atrum VaMs.102	EEY20400.1	9 E-58	302/337
Eukaryota	Fungi	Nectria haematococca mpVI 77-13-4	EEU37991.1	1 E-56	347/337
Eukaryota	Fungi	Pyrenophora tritici-repentis Pt-1C-BFP	XP_001932916.1	2 E-56	334/337
Eukaryota	Fungi	Aspergillus fumigatus Af293	XP_748533.1	3 E-51	303/337
Eukaryota	Fungi	Verticillium albo-atrum VaMs.102	EEY22965.1	5 E-49	340/337
Eukaryota	Fungi	Postia placenta Mad-698-R	XP_002476262.1	7 E-48	348/337
Eukaryota	Fungi	Postia placenta Mad-698-R	XP_002469276.1	2 E-47	349/337
Eukaryota	Fungi	Verticillium albo-atrum VaMs.102	EEY17467.1	9 E-47	289/337
Eukaryota	Fungi	Schizosaccharomyces pombe	NP_596787.1	2 E-45	334/337
Eukaryota	Fungi	Schizosaccharomyces japonicus yFS275	XP_002175486.1	1 E-41	309/337
Eukaryota	Fungi	Laccaria bicolor S238N-H82	XP_001878260.1	2 E-40	346/337
Eukaryota	Fungi	Ajellomyces dermatitidis ER-3	EEQ88330.1	7 E-38	363/337
Eukaryota	Fungi	Ajellomyces dermatitidis SLH14081	XP_002627443.1	5 E-37	363/337
Eukaryota	Fungi	Phaeosphaeria nodorum SN15	XP_001805829.1	1 E-36	291/337
Eukaryota	Fungi	Coprinopsis cinerea okayama7#130	XP_001833704.1	4 E-36	344/337
Bacteria	Firmicutes	Geobacillus sp. Y412MC10	YP_003243273.1	8 E-36	308/337
Bacteria	Cyanobacteria	Nodularia spumigena CCY9414	ZP_01628442.1	2 E-35	283/337
Eukaryota	Viridiplantae	Physcomitrella patens subsp. patens	XP_001760922.1	1 E-34	328/337
Eukaryota	Fungi	Nectria haematococca mpVI 77-13-4	EEU37660.1	2 E-34	326/337
Bacteria	Bacteroidetes	Microscilla marina ATCC 23134	ZP_01687425.1	1 E-33	307/337
Sedis	Exiguobacterium	Exiguobacterium sibiricum 255-15;	YP_001814010.1	1 E-33	304/337
Bacteria	Cyanobacteria	Cyanothece sp. ATCC 51142	YP_001806153.1	1 E-33	288/337
Eukaryota	Fungi	Coprinopsis cinerea okayama7#130	XP_001833703.1	2 E-33	297/337
Eukaryota	Fungi	Coprinopsis cinerea okayama7#130	XP_001833703.1	1 E-31	317/337
Bacteria	Proteobacteria	Pseudomonas putida F1	YP_001268022.1	3 E-33	315/337
Eukaryota	Fungi	Ajellomyces capsulatus NAm1	XP_001538186.1	4 E-33	365/337
Bacteria	Proteobacteria	Shewanella woodyi ATCC 51908	YP_001761333.1	5 E-33	284/337
Eukaryota	Fungi	Ajellomyces capsulatus G186AR	EEH05665.1	7 E-33	365/337
Bacteria	Cyanobacteria	Cyanothece sp. PCC 7822	ZP_03154199.1	8 E-33	291/337
Bacteria	Firmicutes	Staphylococcus carnosus subsp. carnosus	YP_002635103.1	2 E-32	303/337
Bacteria	Proteobacteria	Pseudomonas putida KT2440	NP_745132.1	3 E-32	315/337
Bacteria	Cyanobacteria	Lyngbya sp. PCC 8106	ZP_01621620.1	1 E-31	289/337
Bacteria	Firmicutes	Staphylococcus capitis SK14	ZP_03614325.1	1 E-31	307/337
Eukaryota	Fungi	Ajellomyces capsulatus H143	EER41526.1	2 E-31	365/337
Bacteria	Actinobacteria	Nakamurella multipartita DSM 44233	YP_003199829.1	3 E-31	311/337

Bacteria	Firmicutes	Bacillus sp. NRRL B-14911	ZP_01169858.1	3 E-31	303/337
Bacteria	Cyanobacteria	Cyanothece sp. PCC 7424	YP_002379676.1	3 E-31	289/337
Bacteria	Cyanobacteria	Nostoc azollae 0708	ZP_03766843.1	4 E-31	286/337
Bacteria	Actinobacteria	Salinispora tropica CNB-440	YP_001160316.1	4 E-31	297/337
Bacteria	Firmicutes	Bacillus cereus R309803;	ZP_04290315.1	5 E-31	316/337
Bacteria	Firmicutes	Bacillus cereus AH1271;	ZP_04187171.1	6 E-31	316/337
Eukaryota	Fungi	Penicillium chrysogenum Wisconsin 54-1255	XP_002559407.1	7 E-31	344/337
Bacteria	Firmicutes	Bacillus thuringiensis serovar kurstaki	ZP_04115806.1	9 E-31	316/337
Bacteria	Firmicutes	Bacillus cereus Rock3-29;	ZP_04228902.1	9 E-31	305/337
Bacteria	Firmicutes	Bacillus cereus Rock4-18;	ZP_04208397.1	1 E-30	305/337
Bacteria	Firmicutes	Bacillus thuringiensis serovar huazhongensis	ZP_04085516.1	1 E-30	316/337
Bacteria	Proteobacteria	Alcanivorax borkumensis SK2	YP_691781.1	1 E-30	312/337
Bacteria	Firmicutes	Bacillus cereus NVH0597-99;	ZP_03105726.1	1 E-30	316/337
Bacteria	Firmicutes	Bacillus cereus 172560W;	ZP_04307127.1	2 E-30	312/337
Bacteria	Firmicutes	Bacillus cereus AH676;	ZP_04192793.1	2 E-30	316/337
Bacteria	Firmicutes	Bacillus cereus AH1134;	ZP_03229907.1	2 E-30	312/337
Bacteria	Proteobacteria	Stenotrophomonas maltophilia R551-3	YP_002028395.1	2 E-30	301/337
Bacteria	Firmicutes	Weissella paramesenteroides ATCC 33313	ZP_04783397.1	2 E-30	285/337
Bacteria	Firmicutes	Bacillus thuringiensis serovar pulsiensis	ZP_04079697.1	2 E-30	316/337
Bacteria	Firmicutes	Bacillus thuringiensis serovar tochigiensis	ZP_04146728.1	2 E-30	316/337
Eukaryota	Viridiplantae	Physcomitrella patens subsp. patens	XP_001770323.1	2 E-30	330/337
Eukaryota	Viridiplantae	Vitis vinifera	XP_002270493.1	2 E-30	306/337
Bacteria	Cyanobacteria	Trichodesmium erythraeum IMS101	YP_722551.1	3 E-30	284/337
Bacteria	Firmicutes	Bacillus cereus Rock3-28;	ZP_04234718.1	3 E-30	305/337
Bacteria	Firmicutes	Bacillus cereus G9241;	ZP_00235676.1	4 E-30	316/337
Bacteria	Firmicutes	Bacillus thuringiensis serovar pondicheriensis	ZP_04091588.1	4 E-30	316/337
Bacteria	Firmicutes	Bacillus cereus ATCC 10876	ZP_04318573.1	5 E-30	312/337
Bacteria	Cyanobacteria	Acaryochloris marina MBIC11017	YP_001520351.1	5 E-30	303/337
Bacteria	Firmicutes	Bacillus thuringiensis serovar israelensis	ZP_00742707.1	6 E-30	316/337
Bacteria	Firmicutes	Bacillus cereus ATCC 10987	NP_979819.1	6 E-30	305/337
Bacteria	Firmicutes	Bacillus cereus m1550;	ZP_04279886.1	6 E-30	316/337
Bacteria	Firmicutes	Bacillus cereus B4264;	YP_002368211.1	6 E-30	316/337
Bacteria	Firmicutes	Bacillus thuringiensis serovar sotto	ZP_04127434.1	7 E-30	316/337
Bacteria	Firmicutes	Bacillus cereus ATCC 4342	ZP_04285172.1	7 E-30	316/337
Bacteria	Firmicutes	Bacillus cereus G9842;	YP_002446970.1	8 E-30	316/337
Bacteria	Firmicutes	Bacillus cereus F65185;	ZP_04206380.1	8 E-30	316/337
Bacteria	Firmicutes	Bacillus cereus H3081.97;	ZP_03236441.1	8 E-30	316/337
Bacteria	Bacteroidetes	Psychroflexus torquis ATCC 700755	ZP_01252257.1	8 E-30	299/337
Bacteria	Firmicutes	Lactobacillus salivarius ATCC 11741	ZP_04008770.1	9 E-30	321/337
Bacteria	Proteobacteria	Rhodobacterales bacterium HTCC2255	ZP_01446835.1	1 E-29	306/337
Bacteria	Bacteroidetes	Gramella forsetii KT0803	YP_861961.1	1 E-29	300/337
Eukaryota	Alveolata	Perkinsus marinus ATCC 50983	EER01757.1	1 E-29	320/337
Bacteria	Firmicutes	Bacillus anthracis str. A0442	ZP_02390347.1	1 E-29	316/337
Bacteria	Firmicutes	Bacillus thuringiensis serovar konkukian	YP_037595.1	1 E-29	316/337
Eukaryota	Viridiplantae	Zea mays;	NP_001151204.1	1 E-29	310/337
Bacteria	Actinobacteria	Salinispora arenicola CNS-205	YP_001538665.1	1 E-29	301/337
Bacteria	Firmicutes	Bacillus anthracis str. A1055	ZP_05185266.1	1 E-29	316/337
Eukaryota	Alveolata	Perkinsus marinus ATCC 50983	EER14551.1	1 E-29	324/337
Bacteria	Firmicutes	Paenibacillus sp. JDR-2	YP_003009322.1	2 E-29	298/337
Bacteria	Firmicutes	Bacillus cereus 03BB102;	YP_002750853.1	2 E-29	316/337
Bacteria	Proteobacteria	Geobacter bemidjiensis Bem	YP_002137331.1	2 E-29	300/337
Bacteria	Firmicutes	Bacillus thuringiensis serovar pakistani	ZP_04121373.1	2 E-29	316/337
Bacteria	Firmicutes	Bacillus anthracis str. Ames	NP_845838.1	2 E-29	316/337
Bacteria	Firmicutes	Bacillus cereus Rock1-15;	ZP_04240473.1	2 E-29	316/337
Bacteria	Chloroflexi	Herpetosiphon aurantiacus ATCC 23779	YP_001544275.1	2 E-29	301/337
Bacteria	Firmicutes	Leuconostoc citreum KM20	YP_001727408.1	2 E-29	288/337
Bacteria	Firmicutes	Pediococcus pentosaceus ATCC 25745	YP_803792.1	2 E-29	285/337
Bacteria	Proteobacteria	Pseudomonas fluorescens Pf0-1	YP_348207.1	2 E-29	310/337

Bacteria	Firmicutes	Bacillus cereus m1293;	ZP_04324345.1	3 E-29	316/337
Bacteria	Chloroflexi	Herpetosiphon aurantiacus ATCC 23779	YP_001546656.1	3 E-29	311/337
Bacteria	Firmicutes	Bacillus sp. B14905	ZP_01725910.1	3 E-29	316/337
Bacteria	Firmicutes	Bacillus cereus MM3;	ZP_04301700.1	3 E-29	316/337
Bacteria	Firmicutes	Bacillus thuringiensis serovar thuringiensis	ZP_04134073.1	3 E-29	316/337
Bacteria	Planctomycetes	Blastopirellula marina DSM 3645	ZP_01092492.1	3 E-29	289/337
Bacteria	Firmicutes	Bacillus cereus W;	ZP_03102995.1	3 E-29	316/337
Eukaryota	Alveolata	Perkinsus marinus ATCC 50983	EER11328.1	4 E-29	324/337
Bacteria	Firmicutes	Bacillus cereus 03BB108;	ZP_03110101.1	4 E-29	316/337
Bacteria	Firmicutes	Bacillus thuringiensis str. Al	YP_895917.1	4 E-29	316/337
Bacteria	Cyanobacteria	Acaryochloris marina MBIC11017	YP_001520333.1	4 E-29	302/337
Bacteria	Firmicutes	Bacillus cereus E33L;	YP_084807.1	4 E-29	316/337
Eukaryota	Viridiplantae	Physcomitrella patens subsp. patens	XP_001780366.1	5 E-29	325/337
Bacteria	Candidatus	Candidatus Pelagibacter ubique HTCC1002	ZP_01265024.1	6 E-29	306/337
Bacteria	Firmicutes	Bacillus cereus BDRD-Cer4;	ZP_04257744.1	6 E-29	316/337
Bacteria	Firmicutes	Bacillus thuringiensis serovar berliner	ZP_04103137.1	6 E-29	313/337
Bacteria	Firmicutes	Bacillus cereus ATCC 14579	NP_833231.1	6 E-29	316/337
Eukaryota	Viridiplantae	Sorghum bicolor;	XP_002450886.1	7 E-29	327/337
Bacteria	Firmicutes	Oceanobacillus iheyensis HTE831	NP_691739.1	7 E-29	291/337
Bacteria	Proteobacteria	Dickeya dadantii Ech586	ZP_05722503.1	7 E-29	332/337
Bacteria	Firmicutes	Bacillus cereus AH603;	ZP_04198443.1	8 E-29	305/337
Bacteria	Firmicutes	Enterococcus faecalis TX1322	ZP_04435749.1	9 E-29	320/337
Bacteria	Firmicutes	Enterococcus faecalis HIP11704	ZP_05568252.1	9 E-29	320/337
Bacteria	Spirochaetes	Leptospira biflexa serovar Patoc	YP_001838927.1	9 E-29	319/337
Eukaryota	Fungi	Yarrowia lipolytica CLIB122	XP_503674.1	1 E-28	330/337
Bacteria	Firmicutes	Lactobacillus salivarius UCC118	YP_535863.1	1 E-28	321/337
Bacteria	Bacteroidetes	Flavobacterium johnsoniae UW101	YP_001195437.1	1 E-28	333/337
Bacteria	Proteobacteria	Alcanivorax sp. DG881	ZP_05040853.1	1 E-28	313/337
Bacteria	Firmicutes	Enterococcus faecalis T8	ZP_05560446.1	2 E-28	320/337
Eukaryota	Viridiplantae	Zea mays;	ACG31381.1	2 E-28	329/337
Bacteria	Firmicutes	Enterococcus faecalis T1	ZP_05422238.1	2 E-28	313/337
Bacteria	Actinobacteria	Rubrobacter xylanophilus DSM 9941	YP_643474.1	2 E-28	295/337
Bacteria	Firmicutes	Bacillus mycoides DSM 2048	ZP_04169876.1	2 E-28	305/337
Bacteria	Cyanobacteria	Microcystis aeruginosa NIES-843	YP_001660876.1	2 E-28	301/337
Bacteria	Firmicutes	Bacillus cereus BGSC 6E1	ZP_04312907.1	2 E-28	316/337
Bacteria	Firmicutes	Bacillus cereus MM3;	ZP_04301595.1	2 E-28	288/337
Bacteria	Firmicutes	Enterococcus faecalis DS5	ZP_05561083.1	2 E-28	315/337
Bacteria	Firmicutes	Enterococcus faecalis T3	ZP_05502538.1	2 E-28	320/337
Bacteria	Firmicutes	Lactobacillus brevis ATCC 367	YP_796003.1	2 E-28	313/337
Bacteria	Firmicutes	Enterococcus faecalis D6	ZP_05582292.1	2 E-28	320/337
Bacteria	Proteobacteria	Burkholderia sp. 383	YP_372247.1	2 E-28	309/337
Bacteria	Firmicutes	Enterococcus faecalis TUSoD Ef11	ZP_04648034.1	2 E-28	320/337
Bacteria	Actinobacteria	Micromonospora sp. ATCC 39149	ZP_04608995.1	3 E-28	282/337
Bacteria	Proteobacteria	Roseovarius sp. 217	ZP_01035742.1	3 E-28	315/337
Bacteria	Firmicutes	Enterococcus faecalis ATCC 29200	ZP_04439323.1	3 E-28	320/337
Bacteria	Proteobacteria	Leptothrix cholodnii SP-6	YP_001792666.1	3 E-28	303/337
Eukaryota	Viridiplantae	Zea mays;	ACL53936.1	3 E-28	328/337
Bacteria	Firmicutes	Enterococcus faecalis AR01/DG	ZP_05594524.1	3 E-28	315/337
Bacteria	Actinobacteria	Geodermatophilus obscurus DSM 43160	ZP_03890727.1	3 E-28	298/337
Bacteria	Actinobacteria	Streptosporangium roseum DSM 43021	ZP_04471054.1	3 E-28	304/337
Bacteria	Firmicutes	Enterococcus faecalis TX0104	ZP_03950000.1	4 E-28	316/337
Bacteria	Firmicutes	Enterococcus faecalis HH22	ZP_03985838.1	4 E-28	315/337
Eukaryota	Fungi	Phaeosphaeria nodorum SN15	XP_001800637.1	4 E-28	337/337
Bacteria	Firmicutes	Enterococcus faecalis V583	NP_813914.1	4 E-28	315/337
Bacteria	Proteobacteria	Serratia proteamaculans 568	YP_001479410.1	5 E-28	305/337
Eukaryota	Viridiplantae	Populus trichocarpa	XP_002330717.1	5 E-28	294/337
Bacteria	Proteobacteria	Roseobacter litoralis Och 149	ZP_02140012.1	6 E-28	297/337
Bacteria	Firmicutes	Enterococcus faecalis Merz96	ZP_05564886.1	6 E-28	315/337

Bacteria	Proteobacteria	Methylotenera mobilis JLW8	YP_003048535.1	6 E-28	332/337
Bacteria	Proteobacteria	Geobacter sp. FRC-32	YP_002536431.1	6 E-28	280/337
Bacteria	Firmicutes	Bacillus thuringiensis serovar berliner	ZP_04103011.1	6 E-28	288/337
Bacteria	Firmicutes	Enterococcus faecalis T2	ZP_05425203.1	7 E-28	320/337
Bacteria	Proteobacteria	Burkholderia sp. H160	ZP_03264012.1	7 E-28	306/337
Bacteria	Firmicutes	Leuconostoc mesenteroides subsp. mesenteroide	YP_818427.1	7 E-28	283/337
Bacteria	Exiguobacterium	Exiguobacterium sp. AT1b;	YP_002886726.1	7 E-28	284/337
Eukaryota	Viridiplantae	Oryza sativa Japonica Group	NP_001063592.1	9 E-28	334/337
Bacteria	Firmicutes	Bacillus cereus BDRD-ST196;	ZP_04263146.1	9 E-28	305/337
Bacteria	Firmicutes	Bacillus cereus m1293;	ZP_04324227.1	9 E-28	288/337
Eukaryota	Viridiplantae	Spinacia oleracea	Q8H0M1.1	1 E-27	298/337
Eukaryota	Viridiplantae	Populus trichocarpa	ABK96194.1	1 E-27	294/337
Bacteria	Firmicutes	Enterococcus faecalis Fly1	ZP_05578466.1	1 E-27	315/337
Bacteria	Proteobacteria	Pseudomonas fluorescens Pf-5	AAX56379.1	1 E-27	333/337
Bacteria	Firmicutes	Bacillus cereus H3081.97;	ZP_03236258.1	1 E-27	288/337
Bacteria	Firmicutes	Bacillus cereus E33L;	YP_084672.1	1 E-27	288/337
Bacteria	Firmicutes	Bacillus cereus BDRD-ST26;	ZP_04268564.1	1 E-27	288/337
Eukaryota	Alveolata	Perkinsus marinus ATCC 50983	EER06102.1	1 E-27	326/337
Bacteria	Firmicutes	Enterococcus faecalis CH188	ZP_05582948.1	2 E-27	315/337
Bacteria	Proteobacteria	Burkholderia ambifaria MEX-5	ZP_02905878.1	2 E-27	306/337
Bacteria	Firmicutes	Enterococcus faecalis E1Sol	ZP_05575810.1	2 E-27	315/337
Eukaryota	Viridiplantae	Ricinus communis	XP_002525379.1	2 E-27	310/337
Bacteria	Verrucomicrobia	Verrucomicrobium spinosum DSM 4136	ZP_02926987.1	2 E-27	305/337
Bacteria	Firmicutes	Bacillus thuringiensis serovar andalousiensis	ZP_04097498.1	2 E-27	288/337
Eukaryota	Viridiplantae	Oryza sativa Indica Group	EAZ09652.1	2 E-27	334/337
Eukaryota	Viridiplantae	Oryza sativa Japonica Group	NP_001061691.1	2 E-27	311/337
Bacteria	Cyanobacteria	Anabaena variabilis ATCC 29413	YP_320385.1	3 E-27	292/337
Bacteria	Firmicutes	Bacillus sp. SG-1	ZP_01858629.1	3 E-27	285/337
Bacteria	Bacteroidetes	Pedobacter sp. BAL39	ZP_01882889.1	3 E-27	305/337
Eukaryota	Viridiplantae	Sorghum bicolor;	XP_002446245.1	4 E-27	328/337
Bacteria	Actinobacteria	Geodermatophilus obscurus DSM 43160	ZP_03890011.1	4 E-27	326/337
Bacteria	Proteobacteria	Methylobacterium nodulans ORS 2060	YP_002501525.1	4 E-27	305/337
Bacteria	Bacteroidetes	Algoriphagus sp. PR1	ZP_01719141.1	4 E-27	285/337
Bacteria	Bacteroidetes	Chitinophaga pinensis DSM 2588	YP_003121622.1	4 E-27	289/337
Bacteria	Proteobacteria	Pseudomonas fluorescens Pf-5	YP_259909.1	5 E-27	308/337
Bacteria	Firmicutes	Lactobacillus casei BL23	YP_001988898.1	5 E-27	310/337
Bacteria	Firmicutes	Bacillus thuringiensis serovar pulsiensis	ZP_04079566.1	5 E-27	288/337
Bacteria	Proteobacteria	Rhodoferax ferrireducens T118	YP_521590.1	6 E-27	298/337
Bacteria	Firmicutes	Bacillus thuringiensis IBL 200	ZP_04072320.1	7 E-27	282/337
Bacteria	Proteobacteria	Rhizobium leguminosarum bv. trifolii	YP_002984619.1	7 E-27	333/337
Bacteria	Chloroflexi	Herpetosiphon aurantiacus ATCC 23779	YP_001544276.1	8 E-27	322/337
Bacteria	Proteobacteria	Pseudomonas fluorescens SBW25	YP_002872908.1	8 E-27	315/337
Bacteria	Firmicutes	Lactobacillus paracasei subsp. paracasei	ZP_04673688.1	8 E-27	305/337
Bacteria	Proteobacteria	Burkholderia ambifaria IOP40-10	ZP_02889111.1	9 E-27	306/337
Bacteria	Proteobacteria	Burkholderia ambifaria MC40-6	YP_001811928.1	9 E-27	306/337
Bacteria	Firmicutes	Bacillus anthracis str. Ames	NP_845719.1	9 E-27	288/337
Bacteria	Firmicutes	Bacillus cereus ATCC 4342	ZP_04284416.1	1 E-26	282/337
Bacteria	Firmicutes	Lactobacillus casei ATCC 334	YP_807943.1	1 E-26	305/337
Bacteria	Cyanobacteria	Lyngbya sp. PCC 8106	ZP_01623442.1	1 E-26	290/337
Bacteria	Actinobacteria	Kribbella flavida DSM 17836	ZP_03866121.1	2 E-26	314/337
Eukaryota	Viridiplantae	Populus trichocarpa	XP_002300061.1	2 E-26	300/337
Bacteria	Firmicutes	Bacillus cereus Rock3-29;	ZP_04228225.1	2 E-26	282/337
Eukaryota	Fungi	Cryptococcus neoformans var. neoformans	XP_776684.1	2 E-26	339/337
Bacteria	Actinobacteria	Salinispora arenicola CNS-205	YP_001535551.1	2 E-26	306/337
Bacteria	Firmicutes	Paenibacillus sp. JDR-2	YP_003010649.1	2 E-26	306/337
Eukaryota	Viridiplantae	Picea sitchensis	ABR16979.1	2 E-26	313/337
Bacteria	Cyanobacteria	Microcystis aeruginosa PCC 7806	CAO86444.1	2 E-26	287/337
Bacteria	Actinobacteria	Streptomyces avermitilis MA-4680	NP_828629.1	2 E-26	313/337

Bacteria	Firmicutes	Bacillus cereus Rock1-3;	ZP_04245649.1	3 E-26	282/337
Bacteria	Firmicutes	Bacillus cereus Rock3-28;	ZP_04234034.1	3 E-26	282/337

AFUA_6G09600

Eukaryota	Fungi	Aspergillus fumigatus Af293	XP_750849.1	0.0	421/421
Eukaryota	Fungi	Neosartorya fischeri NRRL 181	XP_001258075.1	0.0	421/421
Eukaryota	Fungi	Aspergillus flavus NRRL3357	XP_002382111.1	0.0	413/421
Eukaryota	Fungi	Aspergillus oryzae RIB40	XP_001819114.1	0.0	413/421
Eukaryota	Fungi	Aspergillus terreus NIH2624	XP_001215872.1	0.0	415/421
Eukaryota	Fungi	Aspergillus niger CBS 513.88	XP_001394745.1	1 E-173	415/421
Eukaryota	Fungi	Penicillium chrysogenum Wisconsin 54-1255	XP_002557602.1	1 E-170	415/421
Eukaryota	Fungi	Aspergillus nidulans FGSC A4	XP_680798.1	1 E-170	413/421
Eukaryota	Fungi	Aspergillus niger CBS 513.88	XP_001399236.1	1 E-120	421/421
Eukaryota	Fungi	Aspergillus nidulans FGSC A4	XP_660428.1	1 E-118	403/421
Eukaryota	Fungi	Aspergillus terreus NIH2624	XP_001213591.1	1 E-117	415/421
Eukaryota	Fungi	Neosartorya fischeri NRRL 181	XP_001264641.1	1 E-117	416/421
Eukaryota	Fungi	Aspergillus oryzae RIB40	XP_001822115.1	1 E-115	418/421
Eukaryota	Fungi	Aspergillus clavatus NRRL 1	XP_001271446.1	1 E-115	418/421
Eukaryota	Fungi	Aspergillus flavus NRRL3357	XP_002379274.1	1 E-115	418/421
Eukaryota	Fungi	Aspergillus fumigatus Af293	XP_752490.1	1 E-114	416/421
Eukaryota	Fungi	Penicillium chrysogenum Wisconsin 54-1255	XP_002565631.1	1 E-113	419/421
Bacteria	Proteobacteria	Burkholderia vietnamiensis G4	YP_001115040.1	1 E-111	406/421
Bacteria	Proteobacteria	Nitrobacter hamburgensis X14	YP_579103.1	1 E-111	403/421
Eukaryota	Fungi	Penicillium marneffei ATCC 18224	XP_002151927.1	1 E-110	416/421
Bacteria	Proteobacteria	Nitrosospira multiformis ATCC 25196	YP_411548.1	1 E-109	422/421
Bacteria	Actinobacteria	Streptomyces lividans TK24	ZP_05524861.1	1 E-109	416/421
Bacteria	Proteobacteria	Ralstonia pickettii 12J	YP_001900085.1	1 E-108	411/421
Bacteria	Actinobacteria	Streptomyces coelicolor A3(2)	NP_628283.1	1 E-108	416/421
Bacteria	Proteobacteria	Anaeromyxobacter dehalogenans 2CP-C	YP_466377.1	1 E-108	412/421
Eukaryota	Fungi	Gibberella zeae PH-1	XP_389314.1	1 E-107	424/421
Bacteria	Proteobacteria	Anaeromyxobacter dehalogenans 2CP-1	YP_002493764.1	1 E-107	412/421
Bacteria	Proteobacteria	Geobacter bemidjiensis Bem	YP_002138957.1	1 E-106	405/421
Eukaryota	Fungi	Botryotinia fuckeliana B05.10	XP_001550386.1	1 E-106	427/421
Bacteria	Actinobacteria	Mycobacterium sp. MCS	YP_638505.1	1 E-105	389/421
Bacteria	Proteobacteria	Ralstonia eutropha JMP134	YP_299407.1	1 E-105	417/421
Bacteria	Actinobacteria	Mycobacterium abscessus	YP_001705460.1	1 E-104	408/421
Eukaryota	Fungi	Uncinocarpus reesii 1704	XP_002544082.1	1 E-104	425/421
Bacteria	Actinobacteria	Mycobacterium sp. JLS	YP_001069666.1	1 E-104	389/421
Eukaryota	Fungi	Talaromyces stipitatus ATCC 10500	XP_002480910.1	1 E-103	406/421
Bacteria	Actinobacteria	Mycobacterium avium 104;	YP_882674.1	1 E-103	397/421
Bacteria	Actinobacteria	Arthrobacter aurescens TC1	YP_946771.1	1 E-103	399/421
Eukaryota	Fungi	Sclerotinia sclerotiorum 1980 UF-70	XP_001591340.1	1 E-103	416/421
Eukaryota	Fungi	Coccidioides posadasii C735 delta	EER27951.1	1 E-102	420/421
Bacteria	Actinobacteria	Streptomyces ghanaensis ATCC 14672	ZP_04684856.1	1 E-102	383/421
Bacteria	Actinobacteria	Streptomyces griseoflavus Tu4000	ZP_05542967.1	1 E-101	416/421
Bacteria	Actinobacteria	Mycobacterium gilvum PYR-GCK	YP_001133239.1	1 E-101	395/421
Bacteria	Actinobacteria	Rhodococcus opacus B4	YP_002779825.1	1 E-101	396/421
Eukaryota	Fungi	Phaeosphaeria nodorum SN15	XP_001790877.1	1 E-101	417/421
Bacteria	Actinobacteria	Mycobacterium vanbaalenii PYR-1	YP_955522.1	1 E-100	413/421
Bacteria	Actinobacteria	Jonesia denitrificans DSM 20603	YP_003160005.1	1 E-100	399/421
Bacteria	Actinobacteria	Corynebacterium pseudogenitalium ATCC 3303	ZP_03920089.1	1 E-98	385/421
Eukaryota	Fungi	Pyrenophora tritici-repentis Pt-1C-BFP	XP_001937636.1	2 E-98	418/421
Bacteria	Actinobacteria	Corynebacterium tuberculostearicum SK141	ZP_05364976.1	2 E-98	385/421
Bacteria	Proteobacteria	Methylobacterium extorquens DM4	YP_003069923.1	4 E-98	402/421
Bacteria	Proteobacteria	Myxococcus xanthus DK 1622	YP_633251.1	1 E-97	408/421
Bacteria	Actinobacteria	Brevibacterium linens BL2	ZP_05913351.1	3 E-97	387/421
Bacteria	Actinobacteria	Arthrobacter chlorophenolicus A6	YP_002489713.1	3 E-97	393/421

Eukaryota	Fungi	Botryotinia fuckeliana B05.10	XP_001547166.1	4 E-97	426/421
Eukaryota	Fungi	Ajellomyces dermatitidis ER-3	EEQ87857.1	7 E-97	446/421
Bacteria	Fusobacteria	Fusobacterium sp. 3_1_5R	ZP_05617426.1	1 E-96	388/421
Eukaryota	Fungi	Ajellomyces capsulatus G186AR	EEH08865.1	1 E-96	450/421
Bacteria	Fusobacteria	Fusobacterium gonidiaformans ATCC 25563	ZP_05630390.1	6 E-96	388/421
Eukaryota	Fungi	Ajellomyces dermatitidis SLH14081	XP_002627896.1	8 E-96	446/421
Bacteria	Actinobacteria	Sanguibacter keddieii DSM 10542	ZP_05819450.1	9 E-96	397/421
Bacteria	Actinobacteria	Corynebacterium urealyticum DSM 7109	YP_001800858.1	2 E-95	420/421
Bacteria	Actinobacteria	Streptomyces sp. AA4	ZP_05478552.1	1 E-94	400/421
Bacteria	Firmicutes	Enterococcus faecalis Merz96	ZP_05563871.1	3 E-94	386/421
Bacteria	Proteobacteria	Geobacter metallireducens GS-15	YP_386379.1	4 E-94	354/421
Bacteria	Actinobacteria	Nocardioides sp. JS614	YP_924902.1	4 E-94	402/421
Bacteria	Actinobacteria	Rhodococcus erythropolis SK121	ZP_04386128.1	2 E-93	397/421
Bacteria	Actinobacteria	Corynebacterium jeikeium ATCC 43734	ZP_05847541.1	4 E-93	417/421
Bacteria	Actinobacteria	Rhodococcus erythropolis PR4	YP_002764683.1	8 E-93	397/421
Bacteria	Actinobacteria	Mobiluncus curtisii ATCC 43063	ZP_03923959.1	1 E-92	386/421
Bacteria	Actinobacteria	Corynebacterium amycolatum SK46	ZP_03393508.1	1 E-92	417/421
Bacteria	Actinobacteria	Arthrobacter aurescens TC1	YP_946652.1	2 E-92	383/421
Bacteria	Firmicutes	Streptococcus uberis 0140J	YP_002562236.1	2 E-92	385/421
Bacteria	Actinobacteria	Nocardiopsis dassonvillei subsp. dassonvillei	ZP_04331567.1	7 E-92	418/421
Bacteria	Actinobacteria	Corynebacterium jeikeium K411	YP_250275.1	2 E-91	415/421
Bacteria	Actinobacteria	Catenulispora acidiphila DSM 44928	YP_003111906.1	1 E-90	418/421
Bacteria	Proteobacteria	Stigmatella aurantiaca DW4/3-1	ZP_01465842.1	2 E-90	392/421
Bacteria	Actinobacteria	Corynebacterium efficiens YS-314	NP_739352.1	6 E-90	376/421
Eukaryota	Fungi	Ajellomyces capsulatus H143	EER43655.1	1 E-89	441/421
Bacteria	Actinobacteria	Corynebacterium glutamicum R	YP_001139750.1	3 E-89	388/421
Bacteria	Actinobacteria	Rhodococcus opacus B4	YP_002779838.1	1 E-88	383/421
Eukaryota	Fungi	Podospora anserina DSM 980	XP_001904854.1	7 E-88	406/421
Bacteria	Actinobacteria	Corynebacterium accolens ATCC 49725	ZP_03933384.1	7 E-88	398/421
Bacteria	Acidobacteria	Candidatus Koribacter versatilis Ellin345	YP_591131.1	2 E-87	383/421
Bacteria	Actinobacteria	Corynebacterium kroppenstedtii DSM 44385	YP_002906099.1	2 E-87	414/421
Bacteria	Actinobacteria	Streptomyces pristinaespiralis ATCC 25486	ZP_05012546.1	2 E-87	398/421
Bacteria	Actinobacteria	Clavibacter michiganensis subsp. michiganensis	YP_001222680.1	3 E-87	392/421
Bacteria	Actinobacteria	Rhodococcus jostii RHA1	YP_702887.1	4 E-87	381/421
Bacteria	Actinobacteria	Corynebacterium jeikeium ATCC 43734	ZP_05845952.1	2 E-86	399/421
Bacteria	Actinobacteria	Corynebacterium efficiens YS-314	NP_739205.1	6 E-86	391/421
Bacteria	Actinobacteria	Corynebacterium jeikeium K411	YP_250582.1	7 E-86	399/421
Bacteria	Actinobacteria	Corynebacterium glucuronolyticum ATCC 5186	ZP_03971150.1	3 E-85	381/421
Bacteria	Actinobacteria	Corynebacterium pseudogenitalium ATCC 3303	ZP_03921530.1	4 E-85	384/421
Bacteria	Actinobacteria	Corynebacterium glucuronolyticum ATCC 5186	ZP_03917665.1	6 E-85	381/421
Bacteria	Actinobacteria	Corynebacterium diphtheriae NCTC 13129	NP_939893.1	8 E-85	395/421
Bacteria	Actinobacteria	Corynebacterium aurimucosum ATCC 700975	YP_002834483.1	3 E-84	365/421
Bacteria	Actinobacteria	Corynebacterium lipophiloflavum DSM 44291	ZP_03979033.1	3 E-84	412/421
Bacteria	Actinobacteria	Corynebacterium efficiens YS-314	NP_738596.1	1 E-83	416/421
Bacteria	Actinobacteria	Corynebacterium glucuronolyticum ATCC 5186	ZP_03973238.1	2 E-83	363/421
Bacteria	Actinobacteria	Streptomyces sviceus ATCC 29083	ZP_05017610.1	2 E-83	392/421
Bacteria	Actinobacteria	Corynebacterium tuberculostearicum SK141	ZP_05366388.1	2 E-83	391/421
Bacteria	Actinobacteria	Corynebacterium glucuronolyticum ATCC 5186	ZP_03918901.1	3 E-83	363/421
Bacteria	Proteobacteria	Geobacter sp. M18	ZP_05311875.1	4 E-83	419/421
Bacteria	Verrucomicrobia	Chthoniobacter flavus Ellin428	ZP_03132057.1	4 E-83	399/421
Bacteria	Actinobacteria	Beutenbergia cavernae DSM 12333	YP_002880568.1	7 E-83	422/421
Bacteria	Proteobacteria	Geobacter sp. M21	YP_003021809.1	9 E-83	420/421
Bacteria	Bacteroidetes	Microscilla marina ATCC 23134	ZP_01692144.1	2 E-82	403/421
Bacteria	Proteobacteria	Geobacter bemidjiensis Bem	YP_002139036.1	2 E-82	420/421
Bacteria	Proteobacteria	Pseudoalteromonas haloplanktis TAC125	YP_340836.1	2 E-82	404/421
Bacteria	Actinobacteria	Gardnerella vaginalis ATCC 14019	ZP_03937675.1	2 E-82	385/421
Bacteria	Actinobacteria	Salinispora arenicola CNS-205	YP_001537401.1	3 E-82	396/421
Bacteria	Proteobacteria	gamma proteobacterium NOR5-3	ZP_05126978.1	4 E-82	394/421

Bacteria	Actinobacteria	Corynebacterium glutamicum ATCC 13032	NP_601283.1	7 E-82	402/421
Bacteria	Actinobacteria	Corynebacterium glutamicum R	YP_001138867.1	7 E-82	402/421
Bacteria	Actinobacteria	Brevibacterium linens BL2	ZP_05915100.1	7 E-82	378/421
Bacteria	Actinobacteria	Atopobium vaginae DSM 15829	ZP_03946510.1	9 E-82	368/421
Bacteria	Proteobacteria	Pseudoalteromonas tunicata D2	ZP_01133833.1	1 E-81	406/421
Bacteria	Proteobacteria	Erythrobacter litoralis HTCC2594	YP_457946.1	1 E-81	415/421
Bacteria	Proteobacteria	Caulobacter crescentus CB15	NP_421605.1	2 E-81	399/421
Bacteria	Planctomycetes	Blastopirellula marina DSM 3645	ZP_01092850.1	3 E-81	408/421
Bacteria	Proteobacteria	Caulobacter crescentus NA1000	YP_002518273.1	6 E-81	399/421
Bacteria	Actinobacteria	Corynebacterium striatum ATCC 6940	ZP_03934797.1	7 E-81	382/421
Bacteria	Proteobacteria	Congregibacter litoralis KT71	ZP_01103486.1	2 E-80	404/421
Bacteria	Actinobacteria	Corynebacterium urealyticum DSM 7109	YP_001800554.1	4 E-80	385/421
Bacteria	Actinobacteria	Corynebacterium aurimucosum ATCC 700975	YP_002835141.1	5 E-80	382/421
Bacteria	Proteobacteria	Ralstonia pickettii 12J	YP_001900334.1	5 E-80	391/421
Bacteria	Proteobacteria	Pseudoalteromonas atlantica T6c	YP_662352.1	5 E-80	389/421
Bacteria	Proteobacteria	Erythrobacter sp. SD-21	ZP_01864958.1	1 E-79	416/421
Bacteria	Proteobacteria	Maricaulis maris MCS10	YP_757052.1	2 E-79	404/421
Bacteria	Proteobacteria	Sphingomonas sp. SKA58	ZP_01302332.1	2 E-79	407/421
Bacteria	Proteobacteria	Colwellia psychrerythraea 34H	YP_267603.1	2 E-79	411/421
Bacteria	Actinobacteria	Thermobifida fusca YX	YP_290493.1	3 E-79	402/421
Bacteria	Proteobacteria	Shewanella frigidimarina NCIMB 400	YP_752271.1	3 E-79	407/421
Bacteria	Proteobacteria	Erythrobacter sp. NAP1	ZP_01039538.1	4 E-79	384/421
Bacteria	Proteobacteria	marine gamma proteobacterium HTCC2143	ZP_01615163.1	4 E-79	423/421
Bacteria	Proteobacteria	Desulfovibrio salexigens DSM 2638	YP_002991546.1	4 E-79	414/421
Bacteria	Actinobacteria	Corynebacterium matruchotii ATCC 14266	ZP_04836716.1	1 E-78	410/421
Bacteria	Actinobacteria	Corynebacterium lipophiloflavum DSM 44291	ZP_03979900.1	1 E-78	371/421
Bacteria	Proteobacteria	Comamonas testosteroni KF-1	ZP_03541058.1	2 E-78	406/421
Bacteria	Acidobacteria	Candidatus Koribacter versatilis Ellin345	YP_590673.1	2 E-78	400/421
Bacteria	Proteobacteria	Sphingomonas wittichii RW1	YP_001263918.1	2 E-78	412/421
Bacteria	Actinobacteria	Corynebacterium matruchotii ATCC 33806	ZP_03711178.1	2 E-78	410/421
Bacteria	Proteobacteria	Ralstonia pickettii 12D	YP_002982319.1	3 E-78	391/421
Bacteria	Proteobacteria	Shewanella loihica PV-4	YP_001094813.1	4 E-78	416/421
Bacteria	Proteobacteria	Shewanella halifaxensis HAW-EB4	YP_001675227.1	5 E-78	414/421
Bacteria	Proteobacteria	Sphingopyxis alaskensis RB2256	YP_615991.1	6 E-78	416/421
Bacteria	Proteobacteria	Shewanella piezotolerans WP3	YP_002312845.1	8 E-78	419/421
Bacteria	Actinobacteria	Streptomyces sp. AA4	ZP_05484596.1	2 E-77	369/421
Bacteria	Proteobacteria	gamma proteobacterium HTCC2207	ZP_01224716.1	6 E-77	398/421
Bacteria	Actinobacteria	Salinispora tropica CNB-440	YP_001159241.1	7 E-77	398/421
Bacteria	Proteobacteria	Congregibacter litoralis KT71	ZP_01101248.1	9 E-77	401/421
Eukaryota	Fungi	Coccidioides immitis RS;	XP_001242996.1	4 E-76	338/421
Bacteria	Proteobacteria	Shewanella pealeana ATCC 700345	YP_001502780.1	2 E-75	404/421
Bacteria	Proteobacteria	Shewanella woodyi ATCC 51908	YP_001761738.1	2 E-75	403/421
Bacteria	Proteobacteria	gamma proteobacterium NOR51-B	ZP_04959291.1	3 E-75	399/421
Bacteria	Proteobacteria	Oceanicaulis alexandrii HTCC2633	ZP_00952133.1	9 E-75	401/421
Bacteria	Actinobacteria	Rhodococcus erythropolis SK121	ZP_04386646.1	1 E-74	389/421
Bacteria	Actinobacteria	Corynebacterium striatum ATCC 6940	ZP_03935484.1	2 E-74	390/421
Bacteria	Actinobacteria	Corynebacterium striatum	AAN77164.1	3 E-74	399/421
Bacteria	Proteobacteria	Asticcacaulis excentricus CB 48	ZP_04771118.1	4 E-74	406/421
Bacteria	Proteobacteria	gamma proteobacterium NOR5-3	ZP_05126195.1	5 E-74	399/421
Bacteria	Proteobacteria	Xanthomonas campestris pv. vesicatoria	YP_361892.1	5 E-73	405/421
Bacteria	Proteobacteria	Xanthomonas oryzae pv. oryzicola	ZP_02241341.1	6 E-73	405/421
Bacteria	Proteobacteria	Alcanivorax sp. DG881	ZP_05043315.1	1 E-72	395/421
Bacteria	Proteobacteria	Hirschia baltica ATCC 49814	YP_003060940.1	1 E-72	418/421
Bacteria	Proteobacteria	Hyphomonas neptunium ATCC 15444	YP_759423.1	3 E-72	418/421
Bacteria	Proteobacteria	Syntrophus aciditrophicus SB	YP_462610.1	2 E-71	400/421
Bacteria	Proteobacteria	marine gamma proteobacterium HTCC2080	ZP_01626086.1	3 E-71	402/421
Bacteria	Planctomycetes	Gemmata obscuriglobus UQM 2246	ZP_02733537.1	3 E-71	395/421
Eukaryota	Fungi	Podospora anserina DSM 980	XP_001905306.1	6 E-70	432/421

Bacteria	Actinobacteria	Rhodococcus erythropolis PR4	YP_002763602.1	1 E-69	389/421
Bacteria	Proteobacteria	Brevundimonas sp. BAL3	ZP_05033618.1	8 E-69	383/421
Bacteria	Actinobacteria	Corynebacterium genitalium ATCC 33030	ZP_05707333.1	3 E-68	378/421
Archea	Methanosphaerula	Methanosphaerula palustris E1-9c	YP_002467716.1	2 E-67	392/421
Bacteria	Proteobacteria	Chelativorans sp. BNC1	YP_675081.1	4 E-67	414/421
Bacteria	Actinobacteria	Catenulispora acidiphila DSM 44928	YP_003114441.1	4 E-67	395/421
Bacteria	Actinobacteria	Streptomyces albus J1074	ZP_04704278.1	1 E-66	390/421
Eukaryota	Fungi	Chaetomium globosum CBS 148.51	XP_001223671.1	3 E-65	347/421
Bacteria	Proteobacteria	Acinetobacter baumannii AYE	YP_001714873.1	9 E-65	380/421
Bacteria	Proteobacteria	Acinetobacter baumannii AB900	ZP_04660649.1	1 E-64	380/421
Bacteria	Proteobacteria	Acinetobacter baumannii ACICU	YP_001845331.1	5 E-64	380/421
Bacteria	Actinobacteria	Catenulispora acidiphila DSM 44928	YP_003114904.1	3 E-63	389/421
Bacteria	Proteobacteria	Congregibacter litoralis KT71	ZP_01102183.1	6 E-63	406/421
Bacteria	Proteobacteria	Acinetobacter baumannii ATCC 17978	ABO11171.2	6 E-63	380/421
Bacteria	Proteobacteria	Alteromonadales bacterium TW-7	ZP_01613755.1	2 E-61	389/421
Bacteria	Proteobacteria	Pseudoalteromonas haloplanktis TAC125	YP_340907.1	2 E-60	389/421
Bacteria	Proteobacteria	Shewanella sp. MR-4	YP_735863.1	8 E-60	387/421
Bacteria	Proteobacteria	Shewanella sp. MR-7	YP_739850.1	8 E-60	387/421
Bacteria	Proteobacteria	Campylobacter jejuni subsp. jejuni	ZP_01809788.1	2 E-59	350/421
Bacteria	Proteobacteria	Ralstonia eutropha JMP134	YP_295572.1	2 E-59	384/421
Bacteria	Proteobacteria	Xanthomonas campestris pv. vesicatoria	YP_365695.1	3 E-59	390/421
Bacteria	Proteobacteria	Xanthomonas campestris pv. campestris	NP_639131.1	4 E-59	390/421
Bacteria	Proteobacteria	Ochrobactrum anthropi ATCC 49188	YP_001372882.1	5 E-59	353/421
Bacteria	Proteobacteria	Acinetobacter baumannii ATCC 17978	YP_001083773.1	9 E-59	363/421
Bacteria	Proteobacteria	Ralstonia eutropha H16	YP_725956.1	1 E-58	384/421
Bacteria	Proteobacteria	Pseudomonas aeruginosa C3719	ZP_04929226.1	1 E-58	340/421
Bacteria	Proteobacteria	Xanthomonas oryzae pv. oryzae	YP_202826.1	2 E-58	390/421
Bacteria	Proteobacteria	Xanthomonas oryzae pv. oryzae	YP_452985.1	2 E-58	390/421
Bacteria	Proteobacteria	Escherichia coli 536	YP_672035.1	2 E-58	339/421
Bacteria	Actinobacteria	Streptomyces griseus subsp. griseus	YP_001825931.1	3 E-58	382/421
Bacteria	Proteobacteria	Escherichia coli CFT073	NP_756778.1	3 E-58	339/421
Bacteria	Proteobacteria	Cupriavidus taiwanensis	YP_002005390.1	4 E-58	384/421
Bacteria	Proteobacteria	Xanthomonas oryzae pv. oryzicola	ZP_02241643.1	5 E-58	390/421
Bacteria	Proteobacteria	Escherichia coli O157:H7 EDL933	NP_290600.1	6 E-58	339/421
Bacteria	Proteobacteria	Stenotrophomonas sp. SKA14	ZP_05136920.1	6 E-58	369/421
Bacteria	Proteobacteria	Xanthomonas axonopodis pv. citri	NP_644153.1	8 E-58	390/421
Bacteria	Proteobacteria	Escherichia fergusonii ATCC 35469	YP_002384837.1	9 E-58	339/421
Bacteria	Proteobacteria	Escherichia albertii TW07627	ZP_02904069.1	9 E-58	339/421
Bacteria	Proteobacteria	Burkholderia sp. 383	YP_371706.1	1 E-57	357/421
Bacteria	Proteobacteria	Stenotrophomonas maltophilia R551-3	YP_002030070.1	1 E-57	369/421
Bacteria	Proteobacteria	Stenotrophomonas maltophilia K279a	YP_001973948.1	1 E-57	369/421
Bacteria	Proteobacteria	gamma proteobacterium NOR5-3	ZP_05129380.1	1 E-57	376/421
Bacteria	Proteobacteria	Burkholderia dolosa AUO158	ZP_04948256.1	1 E-57	349/421
Bacteria	Proteobacteria	Psychrobacter cryohalolentis K5	YP_580523.1	2 E-57	389/421

AFUA_6G09610

Eukaryota	Fungi	Aspergillus fumigatus Af293	XP_750850.1	0.0	1135/1135
Eukaryota	Fungi	Neosartorya fischeri NRRL 181	XP_001258077.1	0.0	1106/1135
Eukaryota	Fungi	Aspergillus terreus NIH2624	XP_001218653.1	0.0	1107/1135
Eukaryota	Fungi	Aspergillus clavatus NRRL 1	XP_001270091.1	0.0	1101/1135
Eukaryota	Fungi	Aspergillus fumigatus A1163	EDP49776.1	0.0	1105/1135
Eukaryota	Fungi	Aspergillus fumigatus Af293	XP_751087.1	0.0	1105/1135
Eukaryota	Fungi	Aspergillus clavatus NRRL 1	XP_001268512.1	0.0	1130/1135
Eukaryota	Fungi	Aspergillus terreus NIH2624	XP_001217641.1	1 E-180	1122/1135
Eukaryota	Fungi	Microsporum canis CBS 113480	EEQ31968.1	1 E-176	1096/1135
Eukaryota	Fungi	Aspergillus flavus NRRL3357	XP_002382646.1	1 E-171	1118/1135
Eukaryota	Fungi	Aspergillus oryzae RIB40	XP_001822532.1	1 E-168	1072/1135

Eukaryota	Fungi	Neosartorya fischeri NRRL 181	XP_001264733.1	1 E-164	1095/1135
Eukaryota	Fungi	Neosartorya fischeri NRRL 181	XP_001264733.1	1 E-152	1116/1135
Eukaryota	Fungi	Aspergillus nidulans FGSC A4	XP_681153.1	1 E-162	1104/1135
Eukaryota	Fungi	Xylaria sp. BCC 1067	ABF29402.1	1 E-162	1075/1135
Eukaryota	Fungi	Metarhizium anisopliae	CAA61605.1	1 E-160	1193/1135
Eukaryota	Fungi	Metarhizium anisopliae	CAA61605.1	1 E-157	1095/1135
Eukaryota	Fungi	Aspergillus niger CBS 513.88	XP_001392347.1	1 E-160	1115/1135
Eukaryota	Fungi	Aspergillus niger CBS 513.88	XP_001392347.1	1 E-137	1095/1135
Eukaryota	Fungi	Aspergillus oryzae RIB40	XP_001825738.1	1 E-160	1119/1135
Eukaryota	Fungi	Aspergillus fumigatus Af293	XP_752404.1	1 E-160	1092/1135
Eukaryota	Fungi	Aspergillus fumigatus Af293	XP_752404.1	1 E-153	1116/1135
Eukaryota	Fungi	Aspergillus fumigatus A1163	EDP56272.1	1 E-159	1092/1135
Eukaryota	Fungi	Aspergillus fumigatus A1163	EDP56272.1	1 E-154	1116/1135
Eukaryota	Fungi	Aspergillus flavus NRRL3357	XP_002385122.1	1 E-159	1105/1135
Eukaryota	Fungi	Aspergillus oryzae RIB40	XP_001826925.1	1 E-159	1105/1135
Eukaryota	Fungi	Talaromyces stipitatus ATCC 10500	XP_002483280.1	1 E-158	1088/1135
Eukaryota	Fungi	Talaromyces stipitatus ATCC 10500	XP_002483280.1	1 E-143	1083/1135
Eukaryota	Fungi	Talaromyces stipitatus ATCC 10500	XP_002483280.1	1 E-141	1099/1135
Eukaryota	Fungi	Pyrenophora tritici-repentis Pt-1C-BFP	XP_001940765.1	1 E-156	1109/1135
Eukaryota	Fungi	Penicillium marneffei ATCC 18224	XP_002150827.1	1 E-155	1095/1135
Eukaryota	Fungi	Penicillium marneffei ATCC 18224	XP_002150827.1	1 E-145	1102/1135
Eukaryota	Fungi	Penicillium marneffei ATCC 18224	XP_002150827.1	1 E-135	1095/1135
Eukaryota	Fungi	Penicillium chrysogenum Wisconsin 54-1255	XP_002568111.1	1 E-153	1090/1135
Eukaryota	Fungi	Aspergillus clavatus NRRL 1	XP_001269225.1	1 E-152	1116/1135
Eukaryota	Fungi	Aspergillus clavatus NRRL 1	XP_001269225.1	1 E-149	1104/1135
Eukaryota	Fungi	Aspergillus nidulans FGSC A4	XP_657620.1	1 E-152	1110/1135
Eukaryota	Fungi	Aspergillus nidulans FGSC A4	XP_657620.1	1 E-136	1109/1135
Eukaryota	Fungi	Uncinocarpus reesii 1704	XP_002545160.1	1 E-152	1101/1135
Eukaryota	Fungi	Aspergillus clavatus NRRL 1	XP_001273105.1	1 E-151	1113/1135
Eukaryota	Fungi	Aspergillus clavatus NRRL 1	XP_001270326.1	1 E-151	1096/1135
Eukaryota	Fungi	Aspergillus clavatus NRRL 1	XP_001270326.1	1 E-150	1054/1135
Eukaryota	Fungi	Aspergillus clavatus NRRL 1	XP_001270326.1	1 E-103	1064/1135
Eukaryota	Fungi	Aspergillus oryzae RIB40	XP_001825318.1	1 E-151	1111/1135
Eukaryota	Fungi	Aspergillus oryzae RIB40	XP_001825318.1	1 E-141	1108/1135
Eukaryota	Fungi	Aspergillus flavus NRRL3357	XP_002380492.1	1 E-151	1114/1135
Eukaryota	Fungi	Aspergillus flavus NRRL3357	XP_002380492.1	1 E-142	1105/1135
Eukaryota	Fungi	Uncinocarpus reesii 1704	XP_002544944.1	1 E-151	1079/1135
Eukaryota	Fungi	Penicillium chrysogenum Wisconsin 54-1255	XP_002560820.1	1 E-151	1116/1135
Eukaryota	Fungi	Penicillium chrysogenum Wisconsin 54-1255	XP_002560820.1	1 E-139	1137/1135
Eukaryota	Fungi	Hypocrea virens	AAX63399.1	1 E-150	1158/1135
Eukaryota	Fungi	Aspergillus terreus NIH2624	XP_001210314.1	1 E-150	1091/1135
Eukaryota	Fungi	Aspergillus terreus NIH2624	XP_001210314.1	1 E-139	1084/1135
Eukaryota	Fungi	Coccidioides posadasii C735 delta	EER26975.1	1 E-149	1104/1135
Eukaryota	Fungi	Coccidioides posadasii C735 delta	EER26975.1	1 E-130	1114/1135
Eukaryota	Fungi	Aspergillus niger CBS 513.88	XP_001395363.1	1 E-149	1093/1135
Eukaryota	Fungi	Aspergillus nidulans FGSC A4	XP_658846.1	1 E-149	1115/1135
Eukaryota	Fungi	Aspergillus nidulans FGSC A4	XP_658846.1	1 E-140	1075/1135
Eukaryota	Fungi	Nectria haematococca mpVI 77-13-4	EEU33981.1	1 E-149	1102/1135
Eukaryota	Fungi	Nectria haematococca mpVI 77-13-4	EEU33981.1	1 E-143	1113/1135
Eukaryota	Fungi	Nectria haematococca mpVI 77-13-4	EEU33981.1	1 E-127	1116/1135
Eukaryota	Fungi	Podospora anserina DSM 980	XP_001906396.1	1 E-148	1257/1135
Eukaryota	Fungi	Podospora anserina DSM 980	XP_001906396.1	1 E-119	1144/1135
Eukaryota	Fungi	Microsporum canis CBS 113480	EEQ30108.1	1 E-148	1115/1135
Eukaryota	Fungi	Microsporum canis CBS 113480	EEQ30108.1	1 E-147	1119/1135
Eukaryota	Fungi	Microsporum canis CBS 113480	EEQ30108.1	1 E-135	1106/1135
Eukaryota	Fungi	Penicillium marneffei ATCC 18224	XP_002152236.1	1 E-146	1130/1135
Eukaryota	Fungi	Penicillium marneffei ATCC 18224	XP_002152236.1	1 E-146	1118/1135
Eukaryota	Fungi	Microsporum canis CBS 113480	EEQ35622.1	1 E-146	1101/1135

Eukaryota	Fungi	Microsporum canis CBS 113480	EEQ27963.1	1 E-146	1118/1135
Eukaryota	Fungi	Microsporum canis CBS 113480	EEQ34799.1	1 E-146	1016/1135
Eukaryota	Fungi	Aspergillus niger CBS 513.88	XP_001397974.1	1 E-145	1083/1135
Eukaryota	Fungi	Neosartorya fischeri NRRL 181	XP_001267502.1	1 E-145	1124/1135
Eukaryota	Fungi	Phaeosphaeria nodorum SN15	XP_001805009.1	1 E-144	1086/1135
Eukaryota	Fungi	Phaeosphaeria nodorum SN15	XP_001805009.1	1 E-136	1115/1135
Eukaryota	Fungi	Phaeosphaeria nodorum SN15	XP_001799383.1	1 E-144	1066/1135
Eukaryota	Fungi	Phaeosphaeria nodorum SN15	XP_001799383.1	7 E-95	922/1135
Eukaryota	Fungi	Pyrenophora tritici-repentis Pt-1C-BFP	XP_001942140.1	1 E-144	1059/1135
Eukaryota	Fungi	Chaetomium globosum CBS 148.51	XP_001227984.1	1 E-143	1110/1135
Eukaryota	Fungi	Chaetomium globosum CBS 148.51	XP_001227984.1	1 E-119	1101/1135
Eukaryota	Fungi	Gibberella zeae PH-1	XP_382491.1	1 E-142	1117/1135
Eukaryota	Fungi	Gibberella zeae PH-1	XP_382491.1	1 E-142	1178/1135
Eukaryota	Fungi	Gibberella zeae PH-1	XP_382491.1	1 E-121	1124/1135
Eukaryota	Fungi	Cochliobolus heterostrophus	AAX09986.1	1 E-142	1097/1135
Eukaryota	Fungi	Cochliobolus heterostrophus	AAX09986.1	1 E-130	1112/1135
Eukaryota	Fungi	Coccidioides immitis RS;	XP_001240129.1	1 E-141	1081/1135
Eukaryota	Fungi	Coccidioides immitis RS;	XP_001240129.1	1 E-116	1043/1135
Eukaryota	Fungi	Alternaria brassicae	AAP78735.1	1 E-141	1097/1135
Eukaryota	Fungi	Alternaria brassicae	AAP78735.1	1 E-129	1116/1135
Eukaryota	Fungi	Talaromyces stipitatus ATCC 10500	XP_002477837.1	1 E-140	1073/1135
Eukaryota	Fungi	Talaromyces stipitatus ATCC 10500	XP_002477837.1	1 E-102	1007/1135
Eukaryota	Fungi	Pyrenophora tritici-repentis Pt-1C-BFP	XP_001932133.1	1 E-140	1104/1135
Eukaryota	Fungi	Aspergillus niger CBS 513.88	XP_001393486.1	1 E-139	1103/1135
Eukaryota	Fungi	Aspergillus terreus NIH2624	XP_001208367.1	1 E-138	1087/1135
Eukaryota	Fungi	Pyrenophora tritici-repentis Pt-1C-BFP	XP_001939433.1	1 E-136	1107/1135
Eukaryota	Fungi	Phaeosphaeria nodorum SN15	XP_001804295.1	1 E-135	1087/1135
Eukaryota	Fungi	Phaeosphaeria nodorum SN15	XP_001804295.1	1 E-128	1126/1135
Eukaryota	Fungi	Aspergillus oryzae RIB40	XP_001822635.1	1 E-132	1180/1135
Eukaryota	Fungi	Ajellomyces capsulatus G186AR	EEH02661.1	1 E-132	1064/1135
Eukaryota	Fungi	Verticillium albo-atrum VaMs.102	EEY22270.1	1 E-131	1110/1135
Eukaryota	Fungi	Chaetomium globosum CBS 148.51	XP_001226467.1	1 E-131	1103/1135
Eukaryota	Fungi	Gibberella zeae PH-1	XP_390720.1	1 E-129	1089/1135
Eukaryota	Fungi	Gibberella zeae PH-1	XP_390720.1	1 E-127	1089/1135
Eukaryota	Fungi	Gibberella zeae PH-1	XP_390720.1	1 E-126	1105/1135
Eukaryota	Fungi	Gibberella zeae PH-1	XP_390720.1	1 E-118	1080/1135
Eukaryota	Fungi	Gibberella zeae PH-1	XP_391164.1	1 E-129	1080/1135
Eukaryota	Fungi	Gibberella zeae PH-1	XP_391164.1	1 E-123	1056/1135
Eukaryota	Fungi	Gibberella zeae PH-1	XP_391164.1	1 E-116	1101/1135
Eukaryota	Fungi	Gibberella zeae PH-1	XP_391164.1	1 E-108	1062/1135
Eukaryota	Fungi	Gibberella zeae PH-1	XP_391164.1	1 E-91	940/1135
Eukaryota	Fungi	Aspergillus niger CBS 513.88	XP_001390682.1	1 E-129	1080/1135
Eukaryota	Fungi	Aspergillus niger CBS 513.88	XP_001390682.1	1 E-97	1015/1135
Eukaryota	Fungi	Chaetomium globosum CBS 148.51	XP_001222884.1	1 E-129	1129/1135
Eukaryota	Fungi	Chaetomium globosum CBS 148.51	XP_001222884.1	1 E-114	923/1135
Eukaryota	Fungi	Aspergillus nidulans FGSC A4	CBF87069.1	1 E-128	1091/1135
Eukaryota	Fungi	Aspergillus nidulans FGSC A4	XP_660149.1	1 E-128	1091/1135
Eukaryota	Fungi	Cochliobolus heterostrophus	AAX09987.1	1 E-127	1168/1135
Eukaryota	Fungi	Aspergillus fumigatus Af293	XP_753380.1	1 E-126	1067/1135
Eukaryota	Fungi	Aspergillus fumigatus Af293	XP_753380.1	1 E-105	1127/1135
Eukaryota	Fungi	Aspergillus terreus NIH2624	XP_001213501.1	1 E-124	1095/1135
Eukaryota	Fungi	Aspergillus fumigatus A1163	EDP52010.1	1 E-122	1005/1135
Eukaryota	Fungi	Phaeosphaeria nodorum SN15	XP_001791762.1	1 E-120	1080/1135
Eukaryota	Fungi	Cochliobolus heterostrophus	AAX09990.1	1 E-117	1079/1135
Eukaryota	Fungi	Penicillium marneffei ATCC 18224	XP_002153534.1	1 E-115	1077/1135
Eukaryota	Fungi	Phaeosphaeria nodorum SN15	XP_001799780.1	1 E-113	994/1135
Eukaryota	Fungi	Phaeosphaeria nodorum SN15	XP_001799780.1	3 E-87	1079/1135
Eukaryota	Fungi	Pyrenophora tritici-repentis Pt-1C-BFP	XP_001942346.1	1 E-112	1096/1135

Eukaryota	Fungi	Claviceps purpurea	ABR23346.1	1 E-112	1118/1135
Bacteria	Firmicutes	Brevibacillus brevis NBRC 100599	YP_002772375.1	1 E-104	1079/1135
Bacteria	Firmicutes	Brevibacillus parabrevis	Q70LM5.1	1 E-101	1083/1135
Bacteria	Firmicutes	Brevibacillus parabrevis	Q70LM5.1	2 E-87	1078/1135
Bacteria	Firmicutes	Brevibacillus parabrevis	Q70LM5.1	1 E-81	1069/1135
Eukaryota	Fungi	Cochliobolus carbonum	Q01886.2	1 E-100	1096/1135
Bacteria	Cyanobacteria	Planktothrix agardhii NIVA-CYA 126	CAJ21198.2	1 E-97	1062/1135
Bacteria	Cyanobacteria	Planktothrix rubescens NIVA-CYA 98	CAQ48266.1	5 E-97	1055/1135
Bacteria	Cyanobacteria	Cyanothece sp. PCC 8802	YP_003138772.1	3 E-95	1156/1135
Bacteria	Cyanobacteria	Cyanothece sp. PCC 8801	YP_002373167.1	3 E-95	1156/1135
Eukaryota	Fungi	Aspergillus nidulans FGSC A4	CBF77087.1	1 E-94	978/1135
Eukaryota	Fungi	Aspergillus nidulans FGSC A4	XP_662245.1	1 E-94	978/1135
Bacteria	Cyanobacteria	Nodularia spumigena CCY9414	ZP_01632061.1	2 E-94	1074/1135
Bacteria	Cyanobacteria	Cyanothece sp. CCY0110	ZP_01728834.1	6 E-93	1091/1135
Bacteria	Cyanobacteria	Nostoc sp. ATCC 53789	AAO23333.1	6 E-93	1134/1135
Bacteria	Cyanobacteria	Nostoc punctiforme PCC 73102	YP_001866468.1	1 E-92	1094/1135
Bacteria	Cyanobacteria	Nostoc punctiforme PCC 73102	YP_001866468.1	2 E-90	1087/1135
Bacteria	Firmicutes	Brevibacillus parabrevis	O30408.1	4 E-92	1067/1135
Bacteria	Cyanobacteria	Nostoc sp. PCC 7120	NP_486689.1	5 E-92	1088/1135
Bacteria	Firmicutes	Bacillus cereus Rock1-15;	ZP_04242463.1	7 E-92	1050/1135
Bacteria	Proteobacteria	Cellvibrio japonicus Ueda107	YP_001982344.1	7 E-92	1072/1135
Bacteria	Cyanobacteria	Microcystis aeruginosa NIES-843	YP_001660672.1	1 E-91	1006/1135
Bacteria	Firmicutes	Brevibacillus brevis NBRC 100599	YP_002772430.1	2 E-91	1066/1135
Bacteria	Firmicutes	Bacillus cereus AH676;	ZP_04195022.1	7 E-91	1050/1135
Bacteria	Proteobacteria	Delftia acidovorans SPH-1	YP_001565767.1	1 E-90	1073/1135
Bacteria	Cyanobacteria	Planktothrix agardhii NIES-205	ABW84363.1	8 E-90	1081/1135
Bacteria	Firmicutes	Bacillus cereus F65185;	ZP_04206252.1	8 E-90	1050/1135
Bacteria	Cyanobacteria	Crocosphaera watsonii WH 8501	ZP_00514129.1	9 E-90	1011/1135
Bacteria	Firmicutes	Bacillus cereus Rock4-2;	ZP_04212084.1	1 E-89	1050/1135
Bacteria	Firmicutes	Bacillus cereus ATCC 10876	ZP_04321077.1	2 E-89	1060/1135
Bacteria	Firmicutes	Bacillus thuringiensis serovar pulsiensis	ZP_04081707.1	3 E-89	1071/1135
Bacteria	Firmicutes	Bacillus thuringiensis serovar pondicheriensis	ZP_04094244.1	3 E-89	1048/1135
Bacteria	Firmicutes	Bacillus cereus ATCC 10876	ZP_04317463.1	3 E-89	1050/1135

AFUA_6G09650

Eukaryota	Fungi	Aspergillus fumigatus Af293	XP_750854.2	0.0	388/388
Eukaryota	Fungi	Neosartorya fischeri NRRL 181	XP_001258082.1	0.0	386/388
Eukaryota	Fungi	Penicillium lilacinoechinulatum	ABV48726.1	1 E-132	391/388
Eukaryota	Fungi	Magnaporthe grisea 70-15	XP_367835.1	1 E-106	375/388
Eukaryota	Fungi	Aspergillus terreus NIH2624	XP_001212651.1	1 E-105	406/388
Eukaryota	Fungi	Leptosphaeria maculans	AAS92542.1	7 E-96	372/388
Eukaryota	Fungi	Ajellomyces dermatitidis SLH14081	XP_002628471.1	7 E-94	350/388
Eukaryota	Fungi	Penicillium marneffei ATCC 18224	XP_002144835.1	2 E-92	371/388
Eukaryota	Fungi	Aspergillus oryzae RIB40	XP_001818607.1	2 E-91	346/388
Eukaryota	Fungi	Microsporum canis CBS 113480	EEQ29186.1	4 E-91	342/388
Eukaryota	Fungi	Ajellomyces dermatitidis ER-3	EEQ89280.1	1 E-89	334/388
Eukaryota	Fungi	Aspergillus flavus NRRL3357	XP_002380015.1	3 E-89	363/388
Eukaryota	Fungi	Coccidioides posadasii C735 delta	EER24847.1	1 E-84	361/388
Eukaryota	Fungi	Coccidioides immitis RS;	XP_001239367.1	1 E-84	361/388
Eukaryota	Fungi	Microsporum canis CBS 113480	EEQ30099.1	9 E-83	382/388
Eukaryota	Metazoa	Pediculus humanus corporis	XP_002424639.1	2 E-82	351/388
Eukaryota	Fungi	Aspergillus nidulans FGSC A4	XP_657828.1	6 E-82	355/388
Eukaryota	Fungi	Neurospora crassa OR74A	XP_958757.1	9 E-82	396/388
Eukaryota	Fungi	Coccidioides posadasii C735 delta	EER24951.1	4 E-81	348/388
Eukaryota	Fungi	Podospora anserina DSM 980	XP_001911660.1	3 E-80	402/388
Eukaryota	Fungi	Penicillium chrysogenum Wisconsin 54-1255	XP_002556625.1	6 E-80	388/388
Eukaryota	Metazoa	Trichoplax adhaerens	XP_002112019.1	7 E-80	338/388

Eukaryota	Metazoa	Apis mellifera	XP_393772.2	8 E-80	370/388
Eukaryota	Fungi	Nectria haematococca mpVI 77-13-4	EEU36188.1	9 E-80	379/388
Eukaryota	Fungi	Aspergillus niger CBS 513.88	XP_001389584.1	3 E-79	392/388
Eukaryota	Fungi	Paracoccidioides brasiliensis Pb03;	EEH21146.1	1 E-78	384/388
Eukaryota	Metazoa	Tetraodon nigroviridis	CAF94449.1	3 E-78	337/388
Eukaryota	Fungi	Verticillium albo-atrum VaMs.102	EEY18081.1	3 E-78	373/388
Eukaryota	Fungi	Penicillium marneffei ATCC 18224	XP_002150108.1	4 E-78	382/388
Eukaryota	Metazoa	Anoplopoma fimbria	ACQ57922.1	5 E-78	342/388
Eukaryota	Fungi	Talaromyces stipitatus ATCC 10500	XP_002341951.1	5 E-78	364/388
Eukaryota	Fungi	Magnaporthe grisea 70-15	XP_364685.1	8 E-78	389/388
Eukaryota	Fungi	Aspergillus oryzae RIB40	XP_001821078.1	8 E-78	382/388
Eukaryota	Fungi	Gibberella zeae PH-1	XP_380394.1	3 E-77	355/388
Eukaryota	Metazoa	Nasonia vitripennis	XP_001604934.1	3 E-77	351/388
Eukaryota	Metazoa	Danio rerio	NP_001070805.1	5 E-77	355/388
Eukaryota	Fungi	Aspergillus flavus NRRL3357	XP_002376826.1	5 E-77	382/388
Eukaryota	Fungi	Uncinocarpus reesii 1704	XP_002543865.1	6 E-77	347/388
Eukaryota	Fungi	Laccaria bicolor S238N-H82	XP_001880209.1	1 E-76	358/388
Eukaryota	Metazoa	Oryctolagus cuniculus	NP_001095167.1	2 E-76	346/388
Eukaryota	Fungi	Talaromyces stipitatus ATCC 10500	XP_002484171.1	2 E-76	382/388
Eukaryota	Fungi	Botryotinia fuckeliana B05.10	XP_001555190.1	6 E-76	393/388
Eukaryota	Fungi	Nectria haematococca mpVI 77-13-4	EEU44621.1	1 E-75	395/388
Eukaryota	Metazoa	Nematostella vectensis	XP_001628029.1	2 E-75	337/388
Eukaryota	Fungi	Aspergillus clavatus NRRL 1	XP_001268248.1	3 E-75	395/388
Eukaryota	Fungi	Coprinopsis cinerea okayama7#130	XP_001830401.1	3 E-75	362/388
Eukaryota	Metazoa	Equus caballus	XP_001488357.2	4 E-75	348/388
Eukaryota	Metazoa	Trichoplax adhaerens	XP_002111525.1	6 E-75	338/388
Eukaryota	Fungi	Aspergillus terreus NIH2624	XP_001214188.1	7 E-75	402/388
Eukaryota	Metazoa	Macaca mulatta	XP_001091399.1	1 E-74	348/388
Eukaryota	Fungi	Schizosaccharomyces japonicus yFS275	XP_002172794.1	1 E-74	361/388
Eukaryota	Metazoa	Branchiostoma floridae	XP_002614025.1	1 E-74	357/388
Eukaryota	Metazoa	Monodelphis domestica	XP_001377591.1	7 E-74	338/388
Eukaryota	Metazoa	Homo sapiens	NP_004404.1	8 E-74	348/388
Eukaryota	Metazoa	Homo sapiens	BAA02430.1	8 E-74	348/388
Eukaryota	Fungi	Zygosaccharomyces rouxii CBS 732	XP_002496830.1	2 E-73	354/388
Eukaryota	Metazoa	Homo sapiens	BAA02431.1	2 E-73	348/388
Eukaryota	Metazoa	Homo sapiens	CAG33685.1	7 E-73	348/388
Eukaryota	Fungi	Schizosaccharomyces pombe	NP_588522.1	2 E-72	352/388
Eukaryota	Fungi	Aspergillus flavus NRRL3357	XP_002376711.1	3 E-72	392/388
Eukaryota	Fungi	Lachancea thermotolerans CBS 6340	XP_002555788.1	6 E-72	359/388
Eukaryota	Fungi	Phaeosphaeria nodorum SN15	XP_001794172.1	8 E-72	382/388
Eukaryota	Metazoa	Ornithorhynchus anatinus	XP_001511283.1	1 E-71	340/388
Eukaryota	Fungi	Schizosaccharomyces pombe	NP_594193.1	1 E-71	342/388
Eukaryota	Metazoa	Pediculus humanus corporis	XP_002430277.1	1 E-71	347/388
Eukaryota	Metazoa	Strongylocentrotus purpuratus	XP_780043.1	2 E-71	354/388
Eukaryota	Metazoa	Osmerus mordax	ACO08892.1	2 E-71	337/388
Eukaryota	Fungi	Pichia guilliermondii ATCC 6260	EDK40587.2	4 E-71	357/388
Eukaryota	Fungi	Pichia guilliermondii ATCC 6260	XP_001482730.1	4 E-71	357/388
Eukaryota	Metazoa	Felis catus	NP_001036821.1	5 E-71	348/388
Bacteria	Actinobacteria	Geodermatophilus obscurus DSM 43160	ZP_03888728.1	8 E-71	372/388
Eukaryota	Fungi	Gibberella zeae PH-1	XP_387613.1	8 E-71	387/388
Eukaryota	Metazoa	Nasonia vitripennis	XP_001599068.1	2 E-70	354/388
Eukaryota	Metazoa	Sus scrofa	NP_999273.1	4 E-70	344/388
Eukaryota	Metazoa	Canis lupus familiaris	XP_536748.2	4 E-70	348/388
Eukaryota	Metazoa	Drosophila willistoni	XP_002073133.1	5 E-70	350/388
Eukaryota	Fungi	Clavispora lusitaniae ATCC 42720	XP_002619520.1	6 E-70	370/388
Eukaryota	Metazoa	Drosophila melanogaster	NP_724101.3	1 E-69	359/388
Eukaryota	Metazoa	Drosophila grimshawi	XP_001990554.1	1 E-69	350/388
Eukaryota	Metazoa	Drosophila melanogaster	AAQ23576.1	2 E-69	350/388

Bacteria	Actinobacteria	Saccharopolyspora erythraea NRRL 2338	YP_001106245.1	2 E-69	379/388
Eukaryota	Metazoa	Drosophila erecta	XP_001981685.1	2 E-69	350/388
Eukaryota	Fungi	Debaryomyces hansenii	CAG89141.2	2 E-69	354/388
Eukaryota	Metazoa	Drosophila persimilis	XP_002016869.1	2 E-69	350/388
Eukaryota	Metazoa	Drosophila yakuba	XP_002098925.1	2 E-69	350/388
Bacteria	Actinobacteria	Rhodococcus erythropolis PR4	YP_002768569.1	3 E-69	327/388
Bacteria	Proteobacteria	Phenylobacterium zucineum HLK1	YP_002129131.1	4 E-69	391/388
Eukaryota	Metazoa	Drosophila simulans	XP_002105037.1	4 E-69	343/388
Eukaryota	Metazoa	Drosophila melanogaster	NP_651471.1	5 E-69	343/388
Eukaryota	Metazoa	Mus musculus	NP_031902.2	5 E-69	347/388
Eukaryota	Metazoa	Drosophila virilis	XP_002053410.1	6 E-69	350/388
Eukaryota	Metazoa	Rattus norvegicus	AAA41093.1	6 E-69	348/388
Bacteria	Verrucomicrobia	bacterium Ellin514	ZP_03630514.1	7 E-69	382/388
Eukaryota	Metazoa	Drosophila ananassae	XP_001955843.1	7 E-69	343/388
Eukaryota	Metazoa	Mus musculus	AAH03492.1	7 E-69	347/388
Eukaryota	Metazoa	Rattus norvegicus	NP_446043.1	8 E-69	348/388
Eukaryota	Metazoa	Rattus norvegicus	EDL92430.1	8 E-69	335/388
Eukaryota	Metazoa	Mus musculus	EDL11339.1	9 E-69	365/388
Eukaryota	Metazoa	Drosophila mojavensis	XP_002000266.1	1 E-68	345/388
Bacteria	Actinobacteria	Rhodococcus erythropolis SK121	ZP_04382643.1	1 E-68	327/388
Eukaryota	Metazoa	Mus musculus	P31428.1	1 E-68	347/388
Eukaryota	Metazoa	Aedes aegypti	XP_001650949.1	2 E-68	353/388
Eukaryota	Metazoa	Ovis aries	P43477.1	2 E-68	348/388
Eukaryota	Metazoa	Tribolium castaneum	XP_966938.1	2 E-68	348/388
Eukaryota	Fungi	Debaryomyces hansenii CBS767	XP_460800.1	2 E-68	354/388
Bacteria	Proteobacteria	Brevundimonas sp. BAL3	ZP_05032445.1	3 E-68	367/388
Eukaryota	Metazoa	Monodelphis domestica	XP_001373840.1	3 E-68	339/388
Eukaryota	Metazoa	Bos taurus	NP_001029644.1	3 E-68	348/388
Eukaryota	Metazoa	Tribolium castaneum	XP_974577.1	3 E-68	353/388
Eukaryota	Metazoa	Mus musculus	Q8C255.1	6 E-68	362/388
Eukaryota	Metazoa	Mus musculus	BAE41559.1	6 E-68	362/388
Eukaryota	Metazoa	Pediculus humanus corporis	XP_002428930.1	7 E-68	350/388
Eukaryota	Metazoa	Equus caballus	XP_001496757.2	8 E-68	363/388
Eukaryota	Metazoa	Strongylocentrotus purpuratus	XP_797192.2	1 E-67	353/388
Eukaryota	Metazoa	Homo sapiens	AAQ88819.1	1 E-67	371/388
Eukaryota	Metazoa	Homo sapiens	EAW83203.1	1 E-67	371/388
Eukaryota	Fungi	Aspergillus oryzae RIB40	XP_001820977.1	1 E-67	372/388
Eukaryota	Metazoa	Mus musculus	BAE42354.1	2 E-67	362/388
Eukaryota	Metazoa	Homo sapiens	NP_071750.1	2 E-67	371/388
Bacteria	Planctomycetes	Planctomyces limnophilus DSM 3776	ZP_04427876.1	2 E-67	329/388
Eukaryota	Metazoa	Rattus norvegicus	NP_001011928.1	2 E-67	332/388
Bacteria	Proteobacteria	Sphingopyxis alaskensis RB2256	YP_617354.1	3 E-67	372/388
Eukaryota	Metazoa	Anopheles gambiae str. PEST	XP_552611.3	3 E-67	361/388
Eukaryota	Metazoa	Macaca mulatta	XP_001091274.1	1 E-66	320/388
Bacteria	Actinobacteria	Streptomyces scabiei 87.22	CBG72518.1	2 E-66	377/388
Eukaryota	Fungi	Kluyveromyces lactis NRRL Y-1140	XP_453094.1	2 E-66	344/388
Eukaryota	Metazoa	Canis lupus familiaris	XP_853810.1	2 E-66	364/388
Bacteria	Proteobacteria	Erythrobacter sp. NAP1	ZP_01040289.1	4 E-66	373/388
Eukaryota	Metazoa	Mus musculus	BAE42559.1	4 E-66	362/388
Eukaryota	Metazoa	Macaca mulatta	XP_001097111.1	6 E-66	379/388
Eukaryota	Metazoa	Apis mellifera	XP_623520.2	7 E-66	327/388
Bacteria	Actinobacteria	Streptomyces coelicolor A3(2)	NP_627279.1	8 E-66	375/388
Eukaryota	Metazoa	Mus musculus	NP_795887.2	1 E-65	376/388
Eukaryota	Metazoa	Apis mellifera	XP_396377.2	1 E-65	344/388
Bacteria	Actinobacteria	Streptomyces lividans TK24	ZP_05525744.1	2 E-65	373/388
Eukaryota	Metazoa	Tribolium castaneum	XP_969566.1	3 E-65	346/388
Bacteria	Gemmatimonadete	Gemmatimonas aurantiaca T-27	YP_002763281.1	3 E-65	366/388
Eukaryota	Fungi	Cryptococcus neoformans var. neoformans	XP_569812.1	4 E-65	355/388

Eukaryota	Metazoa	Rattus norvegicus	NP_001008384.1	6 E-65	332/388
Eukaryota	Metazoa	Bos taurus	XP_586714.3	1 E-64	363/388
Eukaryota	Metazoa	Aedes aegypti	XP_001651295.1	2 E-64	344/388
Eukaryota	Fungi	Sclerotinia sclerotiorum 1980 UF-70	XP_001596864.1	2 E-64	376/388
Bacteria	Planctomycetes	Planctomyces maris DSM 8797	ZP_01855927.1	3 E-64	379/388
Bacteria	Actinobacteria	Streptomyces ghanaensis ATCC 14672	ZP_04687805.1	3 E-64	373/388
Eukaryota	Metazoa	Mus musculus	NP_082236.1	5 E-64	332/388
Bacteria	Actinobacteria	Streptomyces sviceus ATCC 29083	ZP_05018869.1	5 E-64	373/388
Bacteria	Actinobacteria	Streptomyces hygroscopicus ATCC 53653	ZP_05517242.1	6 E-64	388/388
Bacteria	Actinobacteria	Streptomyces griseoflavus Tu4000	ZP_05540848.1	6 E-64	378/388
Bacteria	Planctomycetes	Gemmata obscuriglobus UQM 2246	ZP_02733712.1	6 E-64	375/388
Eukaryota	Metazoa	Taeniopygia guttata	XP_002189576.1	7 E-64	334/388
Eukaryota	Metazoa	Anopheles gambiae str. PEST	XP_320858.4	9 E-64	344/388
Bacteria	Actinobacteria	Streptomyces sp. Mg1	ZP_05000243.1	1 E-63	372/388
Bacteria	Actinobacteria	Streptomyces pristinaespiralis ATCC 25486	ZP_05014179.1	1 E-63	382/388
Eukaryota	Metazoa	Canis lupus familiaris	XP_546868.2	1 E-63	341/388
Eukaryota	Metazoa	Aedes aegypti	XP_001651296.1	2 E-63	345/388
Eukaryota	Metazoa	Apis mellifera	XP_623768.1	2 E-63	364/388
Bacteria	Actinobacteria	Streptomyces avermitilis MA-4680	NP_826200.1	2 E-63	373/388
Eukaryota	Fungi	Ustilago maydis 521	XP_758089.1	2 E-63	364/388
Eukaryota	Fungi	Paracoccidioides brasiliensis Pb18;	EEH45793.1	2 E-63	324/388
Bacteria	Actinobacteria	Streptomyces sp. AA4	ZP_05483580.1	3 E-63	368/388
Bacteria	Actinobacteria	Streptomyces sp. SPB74	ZP_04993098.1	3 E-63	395/388
Eukaryota	Metazoa	Drosophila grimshawi	XP_001984865.1	5 E-63	331/388
Bacteria	Actinobacteria	Streptomyces sp. C	ZP_05506970.1	5 E-63	383/388
Bacteria	Actinobacteria	Streptomyces sp. SPB78	ZP_05489177.1	8 E-63	395/388
Bacteria	Actinobacteria	Kytococcus sedentarius DSM 20547	YP_003149575.1	9 E-63	383/388
Eukaryota	Metazoa	Drosophila virilis	XP_002047906.1	9 E-63	331/388
Bacteria	Actinobacteria	Actinosynnema mirum DSM 43827	YP_003099346.1	1 E-62	383/388
Eukaryota	Fungi	Paracoccidioides brasiliensis Pb01;	EEH40939.1	2 E-62	318/388
Eukaryota	Metazoa	Gallus gallus	XP_414081.2	3 E-62	316/388
Eukaryota	Metazoa	Equus caballus	XP_001915918.1	3 E-62	345/388
Eukaryota	Metazoa	Drosophila mojavensis	XP_002008995.1	3 E-62	331/388
Bacteria	Proteobacteria	Erythrobacter sp. SD-21	ZP_01863904.1	4 E-62	383/388
Eukaryota	Fungi	Pyrenophora tritici-repentis Pt-1C-BFP	XP_001939111.1	8 E-62	367/388
Bacteria	Proteobacteria	gamma proteobacterium NOR5-3	ZP_05126756.1	1 E-61	372/388
Eukaryota	Metazoa	Drosophila ananassae	XP_001956606.1	1 E-61	330/388
Bacteria	Actinobacteria	Kribbella flavida DSM 17836	ZP_03864838.1	2 E-61	356/388
Eukaryota	Metazoa	Drosophila erecta	XP_001972454.1	2 E-61	330/388
Eukaryota	Metazoa	Nasonia vitripennis	XP_001607127.1	2 E-61	365/388
Eukaryota	Metazoa	Drosophila simulans	XP_002084560.1	2 E-61	330/388
Eukaryota	Metazoa	Drosophila yakuba	XP_002094503.1	2 E-61	330/388
Bacteria	Actinobacteria	Mycobacterium smegmatis str. MC2	YP_888031.1	2 E-61	356/388
Eukaryota	Metazoa	Nasonia vitripennis	XP_001607093.1	2 E-61	365/388
Eukaryota	Metazoa	Drosophila melanogaster	NP_001097590.1	3 E-61	330/388
Eukaryota	Metazoa	Acyrthosiphon pisum	XP_001944458.1	3 E-61	337/388
Bacteria	Actinobacteria	Streptomyces flavogriseus ATCC 33331	ZP_05803173.1	3 E-61	389/388
Bacteria	Actinobacteria	Streptomyces griseus subsp. griseus	YP_001825991.1	4 E-61	374/388
Bacteria	Proteobacteria	Congregibacter litoralis KT71	ZP_01104258.1	5 E-61	369/388
Eukaryota	Metazoa	Anopheles gambiae str. PEST	XP_312292.4	6 E-61	332/388
Eukaryota	Metazoa	Drosophila sechellia	XP_002030193.1	9 E-61	330/388
Eukaryota	Metazoa	Ciona intestinalis	XP_002121245.1	1 E-60	398/388
Bacteria	Actinobacteria	Streptomyces roseosporus NRRL 15998	ZP_04693703.1	2 E-60	374/388
Eukaryota	Metazoa	Mus musculus	BAE42280.1	2 E-60	342/388
Eukaryota	Metazoa	Anopheles gambiae str. PEST	XP_317639.4	2 E-60	362/388
Bacteria	Actinobacteria	Streptomyces clavuligerus ATCC 27064	ZP_05006838.1	2 E-60	376/388
Bacteria	Actinobacteria	Streptomyces albus J1074	ZP_04704346.1	3 E-60	382/388
Eukaryota	Metazoa	Bos taurus	XP_875681.2	3 E-60	332/388

Eukaryota	Metazoa	Danio rerio	XP_001345532.2	3 E-60	330/388
Eukaryota	Metazoa	Homo sapiens	Q9H4B8.2	9 E-60	332/388
Bacteria	Actinobacteria	Clavibacter michiganensis subsp. michiganensis	YP_001221062.1	9 E-60	392/388
Eukaryota	Metazoa	Homo sapiens	NP_071752.3	1 E-59	332/388
Eukaryota	Metazoa	Homo sapiens	AAH57789.1	1 E-59	332/388
Eukaryota	Metazoa	Macaca mulatta	XP_001096561.1	2 E-59	335/388
Eukaryota	Metazoa	Macaca fascicularis	Q4R7M2.2	4 E-59	335/388
Bacteria	Actinobacteria	Nakamurella multipartita DSM 44233	YP_003200234.1	5 E-59	379/388
Bacteria	Actinobacteria	Janibacter sp. HTCC2649	ZP_00995765.1	5 E-59	377/388
Eukaryota	Metazoa	Macaca fascicularis	BAE00900.1	6 E-59	335/388
Eukaryota	Metazoa	Homo sapiens	EAW83197.1	8 E-59	328/388
Bacteria	Actinobacteria	Clavibacter michiganensis subsp. sepedonicus	YP_001709645.1	1 E-58	383/388
Bacteria	Proteobacteria	Erythrobacter litoralis HTCC2594	YP_458822.1	2 E-58	372/388
Bacteria	Actinobacteria	Streptomyces viridochromogenes DSM 40736	ZP_05531962.1	3 E-58	349/388
Eukaryota	Metazoa	Homo sapiens	NP_001123230.1	3 E-58	331/388
Bacteria	Proteobacteria	Sphingomonas sp. SKA58	ZP_01305323.1	7 E-58	371/388
Eukaryota	Metazoa	Drosophila willistoni	XP_002068364.1	7 E-58	317/388
Eukaryota	Metazoa	Drosophila virilis	ABD64798.1	7 E-58	349/388
Eukaryota	Metazoa	Homo sapiens	AAQ88756.1	2 E-57	331/388
Eukaryota	Metazoa	Macaca mulatta	XP_001096449.1	6 E-56	344/388
Eukaryota	Metazoa	Branchiostoma floridae	XP_002591083.1	8 E-56	353/388
Bacteria	Actinobacteria	Stackebrandtia nassauensis DSM 44728	ZP_04486407.1	4 E-55	375/388
Eukaryota	Metazoa	Pan troglodytes	XP_523398.2	5 E-55	331/388
Eukaryota	Metazoa	Branchiostoma floridae	XP_002603639.1	9 E-55	346/388
Bacteria	Proteobacteria	Novosphingobium aromaticivorans DSM 12444	YP_496084.1	6 E-54	380/388
Bacteria	Bacteroidetes	Rhodothermus marinus DSM 4252	ZP_04423068.1	1 E-52	362/388
Bacteria	Actinobacteria	Kineococcus radiotolerans SRS30216	YP_001361587.1	2 E-52	354/388
Bacteria	Actinobacteria	Brevibacterium linens BL2	ZP_05914141.1	9 E-52	336/388
Eukaryota	Metazoa	Macaca mulatta	XP_001096773.1	1 E-51	316/388
Bacteria	Bacteroidetes	Rhodothermus marinus DSM 4252	ZP_04422571.1	4 E-49	371/388
Bacteria	Acidobacteria	Candidatus Koribacter versatilis Ellin345	YP_591654.1	3 E-48	373/388
Bacteria	Acidobacteria	Candidatus Solibacter usitatus Ellin6076	YP_825636.1	4 E-46	373/388
Bacteria	Proteobacteria	Idiomarina loihiensis L2TR	YP_156307.1	1 E-44	368/388

AFUA_6G09660

Eukaryota	Fungi	Aspergillus fumigatus Af293	XP_750855.1	0.0	2135/2135
Eukaryota	Fungi	Neosartorya fischeri NRRL 181	XP_001258083.1	0.0	2135/2135
Eukaryota	Fungi	Penicillium lilacinoechinulatum	ABV48729.1	0.0	2125/2135
Eukaryota	Fungi	Leptosphaeria maculans	AAS92545.1	0.0	2126/2135
Eukaryota	Fungi	Talaromyces stipitatus ATCC 10500	XP_002487175.1	0.0	2130/2135
Eukaryota	Fungi	Aspergillus fumigatus A1163	EDP52461.1	0.0	2131/2135
Eukaryota	Fungi	Neosartorya fischeri NRRL 181	XP_001263173.1	0.0	2131/2135
Eukaryota	Fungi	Aspergillus fumigatus Af293	XP_754329.2	0.0	2131/2135
Eukaryota	Fungi	Aspergillus terreus NIH2624	XP_001217048.1	0.0	2135/2135
Eukaryota	Fungi	Microsporum canis CBS 113480	EEQ35857.1	0.0	2095/2135
Eukaryota	Fungi	Penicillium marneffei ATCC 18224	XP_002144838.1	0.0	2032/2135
Eukaryota	Fungi	Hypocrea virens	ABV48720.1	0.0	2006/2135
Bacteria	Proteobacteria	Ralstonia solanacearum GMI1000	NP_522202.1	1 E-166	2092/2135
Bacteria	Proteobacteria	Ralstonia solanacearum GMI1000	NP_522202.1	1 E-141	2028/2135
Bacteria	Firmicutes	Brevibacillus parabrevis	O30409.1	1 E-154	1976/2135
Bacteria	Firmicutes	Brevibacillus parabrevis	O30409.1	1 E-143	2014/2135
Bacteria	Firmicutes	Brevibacillus parabrevis	O30409.1	1 E-130	2021/2135
Bacteria	Firmicutes	Brevibacillus parabrevis	O30409.1	1 E-130	2004/2135
Bacteria	Firmicutes	Bacillus licheniformis	CAA06324.1	1 E-152	2069/2135
Bacteria	Firmicutes	Bacillus licheniformis	CAA06324.1	1 E-111	2050/2135
Bacteria	Cyanobacteria	Nostoc punctiforme PCC 73102	YP_001866470.1	1 E-152	2132/2135
Bacteria	Firmicutes	Bacillus licheniformis	AAD04758.1	1 E-151	2069/2135

Bacteria	Firmicutes	Bacillus licheniformis	AAD04758.1	1 E-113	2050/2135
Bacteria	Firmicutes	Bacillus licheniformis ATCC 14580	YP_077641.1	1 E-151	2039/2135
Bacteria	Firmicutes	Bacillus licheniformis ATCC 14580	YP_077641.1	1 E-113	2049/2135
Bacteria	Proteobacteria	Photorhabdus luminescens subsp. laumondii	NP_929905.1	1 E-149	2083/2135
Bacteria	Proteobacteria	Photorhabdus luminescens subsp. laumondii	NP_929905.1	1 E-143	2024/2135
Bacteria	Proteobacteria	Photorhabdus luminescens subsp. laumondii	NP_929905.1	1 E-143	2028/2135
Bacteria	Proteobacteria	Photorhabdus luminescens subsp. laumondii	NP_929905.1	1 E-143	2076/2135
Bacteria	Proteobacteria	Photorhabdus luminescens subsp. laumondii	NP_929905.1	1 E-121	2059/2135
Bacteria	Proteobacteria	Photorhabdus luminescens subsp. laumondii	NP_929905.1	1 E-117	2034/2135
Bacteria	Proteobacteria	Photorhabdus luminescens subsp. laumondii	NP_929905.1	1 E-114	2019/2135
Bacteria	Proteobacteria	Photorhabdus luminescens subsp. laumondii	NP_929905.1	1 E-113	1980/2135
Bacteria	Proteobacteria	Photorhabdus luminescens subsp. laumondii	NP_929905.1	1 E-113	1976/2135
Bacteria	Proteobacteria	Photorhabdus luminescens subsp. laumondii	NP_929905.1	1 E-112	1973/2135
Bacteria	Proteobacteria	Photorhabdus luminescens subsp. laumondii	NP_929905.1	1 E-105	2008/2135
Bacteria	Proteobacteria	Pseudomonas syringae pv. syringae	AAO72425.1	1 E-148	2076/2135
Bacteria	Proteobacteria	Pseudomonas syringae pv. syringae	AAO72425.1	1 E-127	1994/2135
Bacteria	Proteobacteria	Pseudomonas syringae pv. syringae	AAO72425.1	1 E-121	1999/2135
Bacteria	Proteobacteria	Pseudomonas syringae pv. syringae	AAO72425.1	1 E-113	1984/2135
Bacteria	Proteobacteria	Pseudomonas syringae pv. syringae	AAO72425.1	1 E-102	2012/2135
Bacteria	Cyanobacteria	Microcystis aeruginosa K-139	BAH22764.1	1 E-146	2146/2135
Bacteria	Proteobacteria	Pseudomonas syringae pv. syringae	YP_235693.1	1 E-146	2076/2135
Bacteria	Proteobacteria	Pseudomonas syringae pv. syringae	YP_235693.1	1 E-125	1995/2135
Bacteria	Proteobacteria	Pseudomonas syringae pv. syringae	YP_235693.1	1 E-120	1999/2135
Bacteria	Proteobacteria	Pseudomonas syringae pv. syringae	YP_235693.1	1 E-111	1984/2135
Bacteria	Proteobacteria	Pseudomonas syringae pv. syringae	YP_235693.1	1 E-104	2012/2135
Bacteria	Firmicutes	Bacillus subtilis subsp. subtilis	NP_388231.2	1 E-146	1965/2135
Bacteria	Firmicutes	Clostridium cellulovorans 743B	ZP_04803479.1	1 E-146	1978/2135
Bacteria	Firmicutes	Clostridium cellulovorans 743B	ZP_04803479.1	1 E-126	2041/2135
Bacteria	Proteobacteria	Pseudomonas syringae pv. syringae	YP_236792.1	1 E-145	2083/2135
Bacteria	Proteobacteria	Pseudomonas syringae pv. syringae	YP_236792.1	1 E-145	2100/2135
Bacteria	Proteobacteria	Pseudomonas syringae pv. syringae	YP_236792.1	1 E-140	2093/2135
Bacteria	Cyanobacteria	Microcystis aeruginosa PCC 7806	CAO90637.1	1 E-145	2146/2135
Bacteria	Proteobacteria	Burkholderia pseudomallei Pakistan 9	ZP_03795572.1	1 E-145	2097/2135
Bacteria	Proteobacteria	Burkholderia pseudomallei Pakistan 9	ZP_03795572.1	1 E-136	2130/2135
Bacteria	Proteobacteria	Burkholderia pseudomallei Pakistan 9	ZP_03795572.1	1 E-102	2060/2135
Bacteria	Cyanobacteria	Microcystis sp. NIVA-CYA 172/5	AAZ03552.1	1 E-144	2137/2135
Bacteria	Firmicutes	Clostridium cellulovorans 743B	ZP_04807650.1	1 E-144	2037/2135
Bacteria	Firmicutes	Clostridium cellulovorans 743B	ZP_04807650.1	1 E-131	2061/2135
Bacteria	Firmicutes	Aneurinibacillus migulanus	P0C063.2	1 E-144	2005/2135
Bacteria	Firmicutes	Aneurinibacillus migulanus	P0C063.2	1 E-134	1986/2135
Bacteria	Actinobacteria	Rhodococcus opacus B4	YP_002783350.1	1 E-144	2116/2135
Bacteria	Actinobacteria	Rhodococcus opacus B4	YP_002783350.1	1 E-134	2039/2135
Bacteria	Proteobacteria	Photorhabdus luminescens subsp. laumondii	NP_930489.1	1 E-143	2113/2135
Bacteria	Cyanobacteria	Microcystis aeruginosa NIES-843	YP_001661015.1	1 E-143	2146/2135
Bacteria	Firmicutes	Brevibacillus brevis	P0C064.2	1 E-143	2005/2135
Bacteria	Firmicutes	Brevibacillus brevis	P0C064.2	1 E-136	1985/2135
Bacteria	Firmicutes	Brevibacillus texasporus	AAY29580.1	1 E-142	2035/2135
Bacteria	Firmicutes	Brevibacillus texasporus	AAY29580.1	1 E-132	2033/2135
Bacteria	Proteobacteria	Myxococcus xanthus DK 1622	YP_632180.1	1 E-142	2046/2135
Bacteria	Proteobacteria	Myxococcus xanthus DK 1622	YP_631823.1	1 E-141	2034/2135
Bacteria	Proteobacteria	Myxococcus xanthus DK 1622	YP_631823.1	1 E-141	2117/2135
Bacteria	Proteobacteria	Myxococcus xanthus DK 1622	YP_631823.1	1 E-136	2108/2135
Bacteria	Bacteroidetes	Kordia algicida OT-1	ZP_02164246.1	1 E-141	2029/2135
Bacteria	Proteobacteria	Ralstonia solanacearum GMI1000	NP_522203.1	1 E-141	2118/2135
Bacteria	Proteobacteria	Ralstonia solanacearum GMI1000	NP_522203.1	1 E-137	2086/2135
Bacteria	Proteobacteria	Burkholderia pseudomallei 305	ZP_01768847.1	1 E-141	2115/2135
Bacteria	Cyanobacteria	Planktothrix agardhii NIVA-CYA 116	ABI26078.1	1 E-141	2148/2135
Bacteria	Actinobacteria	Rhodococcus opacus B4	YP_002777453.1	1 E-140	2094/2135

Bacteria	Actinobacteria	Rhodococcus opacus B4	YP_002777453.1	1 E-125	2108/2135
Bacteria	Firmicutes	Bacillus subtilis	CAA49817.1	1 E-140	1969/2135
Bacteria	Proteobacteria	Sorangium cellulosum 'So ce	YP_001618906.1	1 E-140	2162/2135
Bacteria	Firmicutes	Clostridium botulinum A2 str.	YP_002802628.1	1 E-139	2067/2135
Bacteria	Firmicutes	Clostridium botulinum A2 str.	YP_002802628.1	1 E-136	2027/2135
Bacteria	Cyanobacteria	Planktothrix agardhii NIES-205	ABW84365.1	1 E-139	2146/2135
Bacteria	Firmicutes	Bacillus subtilis	BAA02523.1	1 E-139	1964/2135
Bacteria	Proteobacteria	Burkholderia pseudomallei 668	YP_001063288.1	1 E-139	2131/2135
Bacteria	Proteobacteria	Burkholderia pseudomallei 668	YP_001063288.1	1 E-106	2051/2135
Bacteria	Proteobacteria	Burkholderia pseudomallei 1710a	ZP_04953332.1	1 E-139	2128/2135
Bacteria	Proteobacteria	Burkholderia pseudomallei 1710a	ZP_04953332.1	1 E-134	2114/2135
Bacteria	Proteobacteria	Burkholderia pseudomallei 1710b	YP_335852.1	1 E-139	2128/2135
Bacteria	Proteobacteria	Burkholderia pseudomallei 1710b	YP_335852.1	1 E-134	2114/2135
Bacteria	Proteobacteria	Burkholderia pseudomallei K96243	YP_111640.1	1 E-139	2128/2135
Bacteria	Actinobacteria	Rhodococcus jostii RHA1	YP_705390.1	1 E-139	1919/2135
Bacteria	Actinobacteria	Rhodococcus jostii RHA1	YP_705390.1	1 E-121	1998/2135
Bacteria	Actinobacteria	Rhodococcus jostii RHA1	YP_705390.1	1 E-114	1938/2135
Bacteria	Firmicutes	Bacillus pumilus ATCC 7061	ZP_03054591.1	1 E-139	2007/2135
Bacteria	Firmicutes	Bacillus pumilus ATCC 7061	ZP_03054591.1	1 E-110	1794/2135
Bacteria	Cyanobacteria	Planktothrix rubescens NIVA-CYA 98	CAQ48255.1	1 E-139	2133/2135
Bacteria	Proteobacteria	Burkholderia pseudomallei 305	ZP_01764939.1	1 E-138	2196/2135
Bacteria	Proteobacteria	Burkholderia pseudomallei 305	ZP_01764939.1	1 E-117	2060/2135
Bacteria	Firmicutes	Bacillus pumilus SAFR-032	YP_001485577.1	1 E-138	2007/2135
Bacteria	Firmicutes	Bacillus pumilus SAFR-032	YP_001485577.1	1 E-108	1794/2135
Bacteria	Proteobacteria	Burkholderia pseudomallei 576	ZP_03453851.1	1 E-138	2129/2135
Bacteria	Proteobacteria	Burkholderia pseudomallei 576	ZP_03453851.1	1 E-134	2116/2135
Bacteria	Proteobacteria	Burkholderia pseudomallei 576	ZP_03453851.1	1 E-103	2037/2135
Bacteria	Proteobacteria	Burkholderia pseudomallei 1106b	ZP_04811762.1	1 E-138	2130/2135
Bacteria	Proteobacteria	Burkholderia pseudomallei 1106a	YP_001076245.1	1 E-137	2130/2135
Bacteria	Proteobacteria	Burkholderia pseudomallei 1106a	YP_001076245.1	1 E-132	2121/2135
Bacteria	Proteobacteria	Burkholderia pseudomallei 1106a	YP_001076245.1	1 E-104	2042/2135
Bacteria	Proteobacteria	Photorhabdus asymbiotica	YP_003041849.1	1 E-137	2056/2135
Bacteria	Proteobacteria	Photorhabdus asymbiotica	YP_003041849.1	1 E-119	1990/2135
Bacteria	Proteobacteria	Photorhabdus asymbiotica	YP_003041849.1	1 E-116	1988/2135
Bacteria	Proteobacteria	Burkholderia pseudomallei K96243	YP_111641.1	1 E-137	2222/2135
Bacteria	Proteobacteria	Burkholderia pseudomallei K96243	YP_111641.1	1 E-119	2046/2135
Bacteria	Firmicutes	Bacillus subtilis	ACI22672.1	1 E-136	2091/2135
Bacteria	Firmicutes	Brevibacillus texasporus	AAY29582.1	1 E-136	1756/2135
Bacteria	Proteobacteria	Burkholderia mallei NCTC 10229	YP_001025738.1	1 E-136	2128/2135
Bacteria	Proteobacteria	Burkholderia mallei GB8 horse	ZP_00439305.2	1 E-136	2128/2135
Bacteria	Proteobacteria	Burkholderia mallei NCTC 10247	YP_001077822.1	1 E-136	2128/2135
Bacteria	Proteobacteria	Burkholderia mallei ATCC 23344	YP_106216.1	1 E-136	2128/2135
Bacteria	Proteobacteria	Myxococcus xanthus DK 1622	YP_631961.1	1 E-136	2169/2135
Bacteria	Proteobacteria	Myxococcus xanthus DK 1622	YP_631961.1	1 E-127	2083/2135
Bacteria	Proteobacteria	Myxococcus xanthus DK 1622	YP_631961.1	1 E-124	2087/2135
Bacteria	Proteobacteria	Pectobacterium atrosepticum SCRI1043	YP_049593.1	1 E-136	2102/2135
Bacteria	Proteobacteria	Burkholderia pseudomallei 576	ZP_03453529.1	1 E-135	2113/2135
Bacteria	Firmicutes	Clostridium cellulolyticum H10	YP_002506694.1	1 E-135	1985/2135
Bacteria	Actinobacteria	Rhodococcus erythropolis SK121	ZP_04387369.1	1 E-135	1996/2135
Bacteria	Actinobacteria	Rhodococcus erythropolis SK121	ZP_04387369.1	1 E-101	1856/2135
Bacteria	Proteobacteria	Pseudomonas syringae pv. tomato	NP_794446.1	1 E-135	2083/2135
Bacteria	Proteobacteria	Burkholderia pseudomallei MSHR346	ZP_04522105.1	1 E-135	2113/2135
Bacteria	Proteobacteria	Burkholderia pseudomallei MSHR346	ZP_04522105.1	1 E-101	2067/2135
Bacteria	Firmicutes	Bacillus amyloliquefaciens FZB42	YP_001419996.1	1 E-134	1996/2135
Bacteria	Firmicutes	Bacillus amyloliquefaciens FZB42	YP_001419996.1	1 E-108	1775/2135
Bacteria	Firmicutes	Brevibacillus brevis	CAA43838.1	1 E-134	2006/2135
Bacteria	Firmicutes	Brevibacillus brevis	CAA43838.1	1 E-130	1993/2135
Bacteria	Firmicutes	Paenibacillus polymyxa	ACA09733.1	1 E-134	1814/2135

Bacteria	Proteobacteria	Teredinibacter turnerae T7901	YP_003073782.1	1 E-134	2096/2135
Bacteria	Proteobacteria	Teredinibacter turnerae T7901	YP_003073782.1	7 E-77	1812/2135
Bacteria	Actinobacteria	Rhodococcus erythropolis SK121	ZP_04385775.1	1 E-133	2008/2135
Bacteria	Firmicutes	Bacillus subtilis	AAN07013.1	1 E-133	2010/2135
Bacteria	Bacteroidetes	Chitinophaga pinensis DSM 2588	YP_003124822.1	1 E-133	1992/2135
Bacteria	Bacteroidetes	Chitinophaga pinensis DSM 2588	YP_003124822.1	1 E-126	1991/2135
Bacteria	Bacteroidetes	Chitinophaga pinensis DSM 2588	YP_003124822.1	1 E-114	2006/2135
Bacteria	Bacteroidetes	Chitinophaga pinensis DSM 2588	YP_003124822.1	1 E-109	2073/2135
Bacteria	Bacteroidetes	Chitinophaga pinensis DSM 2588	YP_003124822.1	1 E-108	2008/2135
Bacteria	Actinobacteria	Rhodococcus erythropolis PR4	YP_002765284.1	1 E-133	2044/2135
Bacteria	Actinobacteria	Rhodococcus erythropolis PR4	YP_002765284.1	1 E-130	2149/2135
Bacteria	Actinobacteria	Rhodococcus erythropolis PR4	YP_002765284.1	1 E-119	2088/2135
Bacteria	Actinobacteria	Rhodococcus erythropolis PR4	YP_002765284.1	4 E-92	1740/2135
Bacteria	Proteobacteria	Myxococcus xanthus DK 1622	YP_631805.1	1 E-133	2117/2135
Bacteria	Firmicutes	Bacillus amyloliquefaciens FZB42	YP_001421411.1	1 E-133	2078/2135
Bacteria	Firmicutes	Bacillus amyloliquefaciens FZB42	CAE11249.1	1 E-133	2078/2135
Bacteria	Firmicutes	Brevibacillus brevis NBRC 100599	YP_002772270.1	1 E-133	2049/2135
Bacteria	Firmicutes	Brevibacillus brevis NBRC 100599	YP_002772270.1	1 E-127	2037/2135
Bacteria	Firmicutes	Brevibacillus brevis NBRC 100599	YP_002772270.1	1 E-127	2066/2135
Bacteria	Firmicutes	Brevibacillus parabrevis	O30408.1	1 E-133	1767/2135
Bacteria	Firmicutes	Brevibacillus parabrevis	O30408.1	1 E-130	2018/2135
Bacteria	Actinobacteria	Rhodococcus opacus B4	YP_002780511.1	1 E-133	2020/2135
Bacteria	Firmicutes	Bacillus subtilis	ABB80123.1	1 E-132	2061/2135
Bacteria	Firmicutes	Bacillus subtilis	ABB80123.1	1 E-111	2060/2135
Bacteria	Firmicutes	Paenibacillus polymyxa	ACA97576.1	1 E-132	2070/2135
Bacteria	Actinobacteria	Streptomyces hygroscopicus	AAU34202.1	3 E-93	2159/2135
Bacteria	Firmicutes	Brevibacillus brevis NBRC 100599	YP_002772269.1	1 E-131	2041/2135
Bacteria	Firmicutes	Brevibacillus brevis NBRC 100599	YP_002772269.1	1 E-131	1769/2135
Bacteria	Firmicutes	Bacillus licheniformis	O68006.1	1 E-131	2060/2135
Bacteria	Firmicutes	Bacillus licheniformis	O68006.1	1 E-110	2060/2135
Bacteria	Proteobacteria	Ralstonia solanacearum MolK2	YP_002254772.1	1 E-131	1966/2135
Bacteria	Firmicutes	Bacillus amyloliquefaciens FZB42	CAE11274.1	1 E-130	1986/2135
Bacteria	Firmicutes	Bacillus amyloliquefaciens FZB42	CAE11274.1	1 E-112	1769/2135
Bacteria	Firmicutes	Brevibacillus texasporus	AAY29579.1	1 E-130	1801/2135
Bacteria	Firmicutes	Bacillus amyloliquefaciens FZB42	YP_001421437.1	1 E-130	1986/2135
Bacteria	Firmicutes	Bacillus amyloliquefaciens FZB42	YP_001421437.1	1 E-112	1769/2135
Bacteria	Actinobacteria	Rhodococcus jostii RHA1	YP_706038.1	1 E-130	2127/2135
Bacteria	Cyanobacteria	Anabaena variabilis ATCC 29413	YP_322129.1	1 E-130	2113/2135
Bacteria	Firmicutes	Bacillus licheniformis	CAA71582.1	1 E-130	2027/2135
Bacteria	Actinobacteria	Mycobacterium intracellulare ATCC 13950	ZP_05227517.1	1 E-129	2020/2135
Bacteria	Firmicutes	Paenibacillus polymyxa	ACA97580.1	1 E-129	2053/2135
Bacteria	Firmicutes	Paenibacillus polymyxa	ACA97580.1	1 E-128	2056/2135
Bacteria	Firmicutes	Paenibacillus polymyxa	ACA97580.1	1 E-111	1901/2135
Bacteria	Cyanobacteria	Cyanothece sp. PCC 7424	YP_002381149.1	1 E-129	1892/2135
Bacteria	Firmicutes	Bacillus licheniformis	AAD04757.1	1 E-129	2026/2135
Bacteria	Firmicutes	Bacillus licheniformis	AAD04757.1	1 E-113	1767/2135
Bacteria	Firmicutes	Bacillus thuringiensis serovar huazhongensis	ZP_04087603.1	1 E-129	2013/2135
Bacteria	Firmicutes	Bacillus thuringiensis serovar huazhongensis	ZP_04087603.1	1 E-112	1845/2135
Bacteria	Firmicutes	Bacillus licheniformis ATCC 14580	YP_077640.1	1 E-129	2024/2135
Bacteria	Firmicutes	Bacillus licheniformis ATCC 14580	YP_077640.1	1 E-110	1767/2135
Bacteria	Proteobacteria	Pectobacterium carotovorum subsp. brasiliensis	ZP_03829072.1	1 E-128	2000/2135
Bacteria	Firmicutes	Bacillus licheniformis	O68008.1	1 E-128	1769/2135
Bacteria	Firmicutes	Bacillus licheniformis	O68008.1	1 E-105	1853/2135
Bacteria	Actinobacteria	Mycobacterium avium subsp. avium	ZP_05217024.1	1 E-128	2064/2135
Bacteria	Actinobacteria	Streptomyces fungicidicus	ABD65958.1	1 E-128	2119/2135
Bacteria	Actinobacteria	Mycobacterium avium subsp. paratuberculosis	NP_960354.1	1 E-128	2093/2135
Bacteria	Proteobacteria	Myxococcus xanthus DK 1622	YP_629859.1	1 E-128	2012/2135
Bacteria	Proteobacteria	Myxococcus xanthus DK 1622	YP_629859.1	1 E-105	1980/2135

Bacteria	Actinobacteria	Rhodococcus jostii RHA1	YP_700138.1	1 E-127	2082/2135
Bacteria	Actinobacteria	Rhodococcus jostii RHA1	YP_700138.1	1 E-127	2082/2135
Bacteria	Actinobacteria	Rhodococcus jostii RHA1	YP_700138.1	1 E-117	2015/2135
Bacteria	Actinobacteria	Rhodococcus jostii RHA1	YP_700138.1	2 E-97	1719/2135
Bacteria	Cyanobacteria	Planktothrix rubescens NIVA-CYA 98	CAQ48252.1	1 E-127	1879/2135
Bacteria	Actinobacteria	Mycobacterium avium 104;	YP_882241.1	1 E-127	2064/2135
Bacteria	Proteobacteria	Pectobacterium atrosepticum SCRI1043	YP_049592.1	1 E-127	1993/2135
Bacteria	Proteobacteria	Pectobacterium atrosepticum SCRI1043	YP_049592.1	1 E-125	1996/2135
Bacteria	Proteobacteria	Pectobacterium atrosepticum SCRI1043	YP_049592.1	1 E-118	1996/2135
Bacteria	Proteobacteria	Pectobacterium atrosepticum SCRI1043	YP_049592.1	1 E-116	1996/2135
Bacteria	Actinobacteria	Rhodococcus erythropolis SK121	ZP_04385774.1	1 E-126	2083/2135
Bacteria	Firmicutes	Bacillus cereus BGSC 6E1	ZP_04315008.1	1 E-126	2069/2135
Bacteria	Firmicutes	Bacillus cereus m1550;	ZP_04278914.1	1 E-126	2034/2135
Bacteria	Firmicutes	Bacillus cereus Rock1-15;	ZP_04242463.1	1 E-126	2034/2135
Bacteria	Firmicutes	Bacillus thuringiensis serovar kurstaki	ZP_04114817.1	1 E-126	2034/2135
Bacteria	Firmicutes	Bacillus thuringiensis IBL 200	ZP_04072087.1	1 E-125	2034/2135
Bacteria	Actinobacteria	Streptomyces griseus subsp. griseus	YP_001824100.1	1 E-125	2052/2135
Bacteria	Firmicutes	Bacillus cereus BDRD-Cer4;	ZP_04256876.1	1 E-125	2034/2135
Bacteria	Firmicutes	Bacillus pumilus SAFR-032	YP_001485576.1	1 E-125	2038/2135
Bacteria	Firmicutes	Bacillus pumilus SAFR-032	YP_001485576.1	1 E-101	1797/2135
Bacteria	Firmicutes	Bacillus thuringiensis serovar pakistani	ZP_04120463.1	1 E-125	2034/2135
Bacteria	Firmicutes	Bacillus thuringiensis serovar huazhongensis	ZP_04084523.1	1 E-125	2001/2135
Bacteria	Firmicutes	Bacillus cereus Rock4-2;	ZP_04212084.1	1 E-125	2034/2135
Bacteria	Firmicutes	Bacillus cereus BDRD-ST24;	ZP_04273489.1	1 E-125	2034/2135
Bacteria	Firmicutes	Bacillus cereus 172560W;	ZP_04306184.1	1 E-125	2034/2135
Bacteria	Firmicutes	Bacillus cereus B4264;	YP_002367179.1	1 E-125	2034/2135
Bacteria	Firmicutes	Bacillus cereus F65185;	ZP_04206252.1	1 E-125	2034/2135
Bacteria	Proteobacteria	Pseudomonas fluorescens SBW25	YP_002872132.1	1 E-125	2037/2135
Bacteria	Firmicutes	Bacillus cereus G9842;	YP_002445847.1	1 E-125	2034/2135
Bacteria	Firmicutes	Bacillus cereus AH676;	ZP_04195022.1	1 E-124	2034/2135
Bacteria	Firmicutes	Bacillus thuringiensis serovar pulsiensis	ZP_04081707.1	1 E-124	2034/2135
Bacteria	Proteobacteria	Marinomonas sp. MWYL1	YP_001341426.1	1 E-124	2062/2135
Bacteria	Proteobacteria	Marinomonas sp. MWYL1	YP_001341426.1	5 E-97	1736/2135
Bacteria	Actinobacteria	Streptomyces albus J1074	ZP_04705330.1	1 E-124	1779/2135
Bacteria	Firmicutes	Bacillus cereus AH1134;	ZP_03232328.1	1 E-124	2034/2135
Bacteria	Firmicutes	Bacillus cereus ATCC 10876	ZP_04317463.1	1 E-124	2034/2135
Bacteria	Firmicutes	Bacillus thuringiensis serovar sotto	ZP_04130612.1	1 E-124	2034/2135
Bacteria	Proteobacteria	Pseudomonas putida	ABW17377.1	1 E-124	1984/2135
Bacteria	Firmicutes	Bacillus thuringiensis serovar berliner	ZP_04105675.1	1 E-124	2033/2135
Bacteria	Proteobacteria	Photorhabdus asymbiotica	YP_003042186.1	1 E-124	1999/2135
Bacteria	Cyanobacteria	Cyanothece sp. PCC 7822	ZP_03156735.1	1 E-124	1840/2135
Bacteria	Firmicutes	Bacillus subtilis	BAB69699.1	1 E-124	2010/2135
Bacteria	Firmicutes	Bacillus cereus BDRD-ST196;	ZP_04265092.1	1 E-123	2060/2135
Bacteria	Firmicutes	Bacillus weihenstephanensis KBAB4;	YP_001642560.1	1 E-123	2060/2135
Bacteria	Firmicutes	Bacillus pumilus ATCC 7061	ZP_03054623.1	1 E-123	2038/2135
Bacteria	Firmicutes	Bacillus pumilus ATCC 7061	ZP_03054623.1	1 E-102	1797/2135
Bacteria	Firmicutes	Clostridium cellulovorans 743B	ZP_04803466.1	1 E-123	1950/2135
Bacteria	Firmicutes	Bacillus subtilis subsp. subtilis	NP_388230.2	1 E-123	2025/2135
Bacteria	Firmicutes	Bacillus subtilis subsp. subtilis	NP_388230.2	1 E-121	1816/2135
Bacteria	Proteobacteria	Pseudomonas syringae pv. syringae	YP_235685.1	1 E-123	2234/2135
Bacteria	Proteobacteria	Pseudomonas syringae pv. syringae	YP_235685.1	1 E-113	2066/2135
Bacteria	Proteobacteria	Pseudomonas syringae pv. syringae	YP_235685.1	1 E-111	2069/2135
Bacteria	Proteobacteria	Pseudomonas syringae pv. syringae	YP_235685.1	1 E-108	1996/2135
Bacteria	Actinobacteria	Nocardia farcinica IFM 10152	YP_121249.1	1 E-123	2117/2135
Bacteria	Actinobacteria	Nocardia farcinica IFM 10152	YP_121249.1	1 E-121	2071/2135
Bacteria	Bacteroidetes	Flavobacterium johnsoniae UW101	YP_001194437.1	1 E-123	1948/2135
Bacteria	Firmicutes	Bacillus cereus Rock1-3;	ZP_04248847.1	1 E-123	2034/2135
Bacteria	Cyanobacteria	Nostoc punctiforme PCC 73102	YP_001865964.1	1 E-123	1720/2135

Bacteria	Actinobacteria	Rhodococcus erythropolis SK121	ZP_04384595.1	1 E-123	2036/2135
Bacteria	Cyanobacteria	Nostoc sp. ATCC 53789	AAO23334.1	1 E-122	2148/2135
Bacteria	Cyanobacteria	Nostoc sp. ATCC 53789	AAO23334.1	1 E-101	1722/2135
Bacteria	Actinobacteria	Rhodococcus erythropolis PR4	YP_002765285.1	1 E-122	2116/2135
Bacteria	Actinobacteria	Rhodococcus erythropolis PR4	YP_002765285.1	1 E-113	2018/2135
Bacteria	Firmicutes	Bacillus subtilis subsp. subtilis	ZP_03591570.1	1 E-122	1994/2135
Bacteria	Firmicutes	Bacillus cereus AH603;	ZP_04200914.1	1 E-122	2061/2135
Bacteria	Bacteroidetes	Chitinophaga pinensis DSM 2588	YP_003123067.1	1 E-122	2007/2135
Bacteria	Bacteroidetes	Chitinophaga pinensis DSM 2588	YP_003123067.1	1 E-121	2041/2135
Bacteria	Bacteroidetes	Chitinophaga pinensis DSM 2588	YP_003123067.1	1 E-120	2007/2135
Bacteria	Firmicutes	Bacillus subtilis subsp. subtilis	ZP_03590009.1	1 E-121	1819/2135
Bacteria	Actinobacteria	Actinosynnema mirum DSM 43827	YP_003101337.1	1 E-121	2051/2135
Bacteria	Firmicutes	Bacillus subtilis subsp. subtilis	CAA84363.1	1 E-121	1994/2135
Bacteria	Firmicutes	Bacillus subtilis	ABB80125.1	1 E-121	1809/2135
Bacteria	Firmicutes	Bacillus subtilis	ABB80125.1	1 E-105	1816/2135
Bacteria	Firmicutes	Bacillus subtilis	CAA49816.1	1 E-121	1819/2135
Bacteria	Firmicutes	Bacillus subtilis	CAA49816.1	1 E-116	2023/2135
Bacteria	Proteobacteria	Pseudoalteromonas tunicata D2	ZP_01131645.1	1 E-121	2176/2135
Bacteria	Firmicutes	Bacillus subtilis	BAA02522.1	1 E-121	2025/2135
Bacteria	Firmicutes	Bacillus subtilis	BAA02522.1	1 E-116	1816/2135
Bacteria	Cyanobacteria	Crocosphaera watsonii WH 8501	ZP_00515514.1	1 E-121	1985/2135
Bacteria	Actinobacteria	Rhodococcus jostii RHA1	YP_705042.1	1 E-121	2045/2135
Bacteria	Actinobacteria	Streptomyces griseus subsp. griseus	YP_001822165.1	1 E-120	2048/2135
Bacteria	Proteobacteria	Xenorhabdus bovienii	AAL57600.1	1 E-113	2013/2135
Bacteria	Actinobacteria	Rhodococcus opacus B4	YP_002782714.1	1 E-120	1973/2135
Bacteria	Actinobacteria	Rhodococcus opacus B4	YP_002782714.1	2 E-99	1896/2135
Bacteria	Actinobacteria	Saccharothrix mutabilis subsp. capreolus	AAM47273.1	1 E-120	1726/2135
Bacteria	Proteobacteria	Myxococcus xanthus DK 1622	YP_632257.1	1 E-120	1998/2135
Bacteria	Firmicutes	Bacillus thuringiensis serovar huazhongensis	ZP_04088042.1	1 E-120	2103/2135
Bacteria	Firmicutes	Bacillus thuringiensis serovar huazhongensis	ZP_04088042.1	1 E-92	1752/2135
Bacteria	Proteobacteria	Burkholderia ambifaria MEX-5	ZP_02907414.1	1 E-120	2047/2135
Bacteria	Firmicutes	Bacillus mycoides DSM 2048	ZP_04171786.1	1 E-119	2061/2135
Bacteria	Proteobacteria	Pseudomonas syringae pv. tomato	NP_794272.1	1 E-119	2084/2135
Bacteria	Firmicutes	Bacillus thuringiensis serovar huazhongensis	ZP_04087605.1	1 E-119	1767/2135
Bacteria	Firmicutes	Bacillus thuringiensis serovar huazhongensis	ZP_04087605.1	1 E-107	1941/2135
Bacteria	Firmicutes	Lactobacillus plantarum WCFS1	NP_784351.1	1 E-119	1825/2135
Bacteria	Firmicutes	Lactobacillus plantarum WCFS1	NP_784351.1	1 E-97	1858/2135
Bacteria	Proteobacteria	Pseudomonas syringae pv. tomato	ZP_03394827.1	1 E-119	2084/2135
Bacteria	Proteobacteria	Pseudomonas syringae pv. syringae	AAC80285.1	1 E-118	2234/2135
Bacteria	Proteobacteria	Pseudomonas syringae pv. syringae	AAC80285.1	1 E-112	2066/2135
Bacteria	Proteobacteria	Pseudomonas syringae pv. syringae	AAC80285.1	1 E-109	2067/2135
Bacteria	Proteobacteria	Pseudomonas syringae pv. syringae	AAC80285.1	1 E-105	1996/2135
Bacteria	Bacteroidetes	Chitinophaga pinensis DSM 2588	YP_003124820.1	1 E-118	1971/2135
Bacteria	Bacteroidetes	Chitinophaga pinensis DSM 2588	YP_003124820.1	1 E-109	1924/2135
Bacteria	Bacteroidetes	Chitinophaga pinensis DSM 2588	YP_003124820.1	1 E-103	1968/2135
Bacteria	Actinobacteria	Streptomyces sp. AA4	ZP_05479630.1	1 E-118	1914/2135
Bacteria	Proteobacteria	Photorhabdus asymbiotica	YP_003041897.1	1 E-116	1979/2135
Bacteria	Proteobacteria	Photorhabdus luminescens subsp. laumondii	NP_930752.1	1 E-118	2127/2135
Bacteria	Proteobacteria	Pseudomonas syringae pv. syringae	YP_235654.1	1 E-118	2067/2135
Bacteria	Proteobacteria	Pseudomonas syringae pv. syringae	YP_235654.1	1 E-110	2091/2135
Bacteria	Proteobacteria	Pseudomonas syringae pv. syringae	YP_235654.1	1 E-104	1969/2135
Bacteria	Actinobacteria	Rhodococcus jostii RHA1	YP_702360.1	1 E-118	2068/2135
Bacteria	Firmicutes	Bacillus subtilis	ABY89499.1	1 E-118	2010/2135
Bacteria	Actinobacteria	Nocardia farcinica IFM 10152	YP_116926.1	1 E-117	2001/2135
Bacteria	Actinobacteria	Rhodococcus opacus B4	YP_002779116.1	1 E-117	2045/2135
Bacteria	Actinobacteria	Nocardia farcinica IFM 10152	YP_116929.1	1 E-117	2027/2135
Bacteria	Cyanobacteria	Cyanothece sp. ATCC 51142	YP_001804469.1	1 E-117	1845/2135
Bacteria	Actinobacteria	Rhodococcus erythropolis SK121	ZP_04385773.1	1 E-117	2097/2135

Bacteria	Actinobacteria	Streptomyces griseus subsp. griseus	YP_001821962.1	1 E-117	1961/2135
Bacteria	Proteobacteria	Erwinia tasmaniensis Et1/99	YP_001908832.1	1 E-117	2015/2135
Bacteria	Firmicutes	Brevibacillus brevis NBRC 100599	YP_002772375.1	1 E-117	1872/2135
Bacteria	Firmicutes	Brevibacillus brevis NBRC 100599	YP_002772375.1	1 E-108	1840/2135
Bacteria	Firmicutes	Brevibacillus parabrevis	Q70LM5.1	1 E-116	1793/2135
Bacteria	Proteobacteria	Erwinia pyrifoliae Ep1/96	YP_002648141.1	1 E-116	2033/2135
Bacteria	Proteobacteria	Erwinia pyrifoliae Ep1/96	YP_002648141.1	1 E-100	1968/2135

AFUA_6G09720

Eukaryota	Fungi	Aspergillus fumigatus Af293	XP_750861.1	1 E-164	282/282
Eukaryota	Fungi	Neosartorya fischeri NRRL 181	XP_001258089.1	1 E-157	282/282
Eukaryota	Fungi	Leptosphaeria maculans	AAR11079.1	3 E-78	272/282
Eukaryota	Fungi	Hypocrea virens	ABV48717.1	5 E-37	262/282
Eukaryota	Fungi	Talaromyces stipitatus ATCC 10500	XP_002486679.1	4 E-29	260/282
Eukaryota	Fungi	Penicillium marneffei ATCC 18224	XP_002144829.1	5 E-28	260/282
Eukaryota	Fungi	Penicillium marneffei ATCC 18224	XP_002152919.1	2 E-27	260/282
Eukaryota	Fungi	Talaromyces stipitatus ATCC 10500	XP_002486598.1	4 E-26	247/282
Eukaryota	Fungi	Penicillium marneffei ATCC 18224	XP_002144836.1	5 E-26	229/282
Eukaryota	Fungi	Postia placenta Mad-698-R	XP_002477008.1	2 E-21	253/282
Eukaryota	Fungi	Nectria haematococca mpVI 77-13-4	EEU37653.1	8 E-21	270/282
Eukaryota	Fungi	Microsporum canis CBS 113480	EEQ31194.1	2 E-19	265/282
Eukaryota	Fungi	Chaetomium globosum CBS 148.51	XP_001223306.1	1 E-18	282/282
Eukaryota	Fungi	Aspergillus clavatus NRRL 1	XP_001276487.1	1 E-18	238/282
Eukaryota	Fungi	Ajellomyces capsulatus H143	EER37581.1	2 E-18	273/282
Eukaryota	Fungi	Coccidioides immitis RS;	XP_001243664.1	2 E-18	271/282
Eukaryota	Fungi	Magnaporthe grisea 70-15	XP_365572.2	4 E-18	271/282
Eukaryota	Fungi	Nectria haematococca mpVI 77-13-4	EEU40385.1	5 E-18	272/282
Eukaryota	Fungi	Nectria haematococca mpVI 77-13-4	EEU43360.1	5 E-18	272/282
Eukaryota	Fungi	Aspergillus fumigatus Af293	XP_747150.1	7 E-18	238/282
Eukaryota	Fungi	Ajellomyces capsulatus G186AR	EEH06108.1	1 E-17	271/282
Eukaryota	Fungi	Coccidioides posadasii C735 delta	EER26865.1	1 E-17	270/282
Eukaryota	Fungi	Magnaporthe grisea 70-15	XP_367317.1	3 E-17	267/282
Eukaryota	Fungi	Nectria haematococca mpVI 77-13-4	EEU34612.1	5 E-17	267/282
Eukaryota	Fungi	Coccidioides posadasii C735 delta	EER22978.1	1 E-16	250/282
Eukaryota	Fungi	Ajellomyces dermatitidis SLH14081	XP_002625587.1	2 E-16	273/282
Eukaryota	Fungi	Nectria haematococca mpVI 77-13-4	EEU38862.1	2 E-16	272/282
Eukaryota	Fungi	Paracoccidioides brasiliensis Pb01;	EEH36101.1	2 E-16	245/282
Eukaryota	Fungi	Nectria haematococca mpVI 77-13-4	EEU37518.1	2 E-16	267/282
Eukaryota	Fungi	Uncinocarpus reesii 1704	XP_002545217.1	3 E-16	274/282
Eukaryota	Fungi	Coccidioides immitis RS;	XP_001248741.1	5 E-16	273/282
Eukaryota	Fungi	Coccidioides immitis RS;	XP_001244425.1	6 E-16	268/282
Eukaryota	Fungi	Nectria haematococca mpVI 77-13-4	EEU34221.1	7 E-16	272/282
Eukaryota	Fungi	Coccidioides posadasii C735 delta	EER29095.1	1 E-15	273/282
Eukaryota	Fungi	Aspergillus terreus NIH2624	XP_001215191.1	1 E-15	281/282
Eukaryota	Fungi	Nectria haematococca mpVI 77-13-4	EEU37333.1	2 E-15	271/282
Eukaryota	Fungi	Sclerotinia sclerotiorum 1980 UF-70	XP_001596769.1	2 E-15	273/282
Eukaryota	Fungi	Ajellomyces capsulatus H143	EER37082.1	3 E-15	226/282
Eukaryota	Fungi	Paracoccidioides brasiliensis Pb03;	EEH16613.1	3 E-15	229/282
Eukaryota	Fungi	Nectria haematococca mpVI 77-13-4	EEU33860.1	3 E-15	272/282
Eukaryota	Fungi	Aspergillus nidulans FGSC A4	XP_663020.1	4 E-15	232/282
Eukaryota	Fungi	Aspergillus oryzae RIB40	XP_001822510.1	5 E-15	273/282
Eukaryota	Fungi	Chaetomium globosum CBS 148.51	XP_001219899.1	7 E-15	229/282
Eukaryota	Fungi	Paracoccidioides brasiliensis Pb18;	EEH46104.1	7 E-15	229/282
Eukaryota	Fungi	Aspergillus fumigatus Af293	XP_754131.2	8 E-15	229/282
Eukaryota	Fungi	Aspergillus flavus NRRL3357	XP_002372233.1	9 E-15	226/282
Eukaryota	Fungi	Botryotinia fuckeliana B05.10	XP_001547359.1	1 E-14	272/282
Eukaryota	Fungi	Chaetomium globosum CBS 148.51	XP_001224099.1	1 E-14	264/282

Eukaryota	Fungi	Aspergillus oryzae RIB40	XP_001819496.1	1 E-14	281/282
Eukaryota	Fungi	Gibberella zeae PH-1	XP_385013.1	2 E-14	272/282
Eukaryota	Fungi	Aspergillus flavus NRRL3357	XP_002375036.1	2 E-14	281/282
Eukaryota	Fungi	Sclerotinia sclerotiorum 1980 UF-70	XP_001592906.1	3 E-14	268/282
Eukaryota	Fungi	Microsporum canis CBS 113480	EEQ28992.1	4 E-14	255/282
Eukaryota	Fungi	Coccidioides posadasii C735 delta	EER26301.1	5 E-14	250/282
Eukaryota	Fungi	Nectria haematococca mpVI 77-13-4	EEU45312.1	7 E-14	277/282
Eukaryota	Fungi	Chaetomium globosum CBS 148.51	XP_001221417.1	7 E-14	239/282
Eukaryota	Fungi	Nectria haematococca mpVI 77-13-4	EEU38112.1	8 E-14	270/282
Eukaryota	Fungi	Aspergillus flavus NRRL3357	XP_002372462.1	9 E-14	227/282
Eukaryota	Fungi	Aspergillus flavus NRRL3357	XP_002385142.1	2 E-13	266/282
Eukaryota	Fungi	Ajellomyces dermatitidis SLH14081	XP_002629376.1	2 E-13	263/282
Eukaryota	Fungi	Uncinocarpus reesii 1704	XP_002543689.1	2 E-13	273/282
Eukaryota	Fungi	Nectria haematococca mpVI 77-13-4	EEU36652.1	2 E-13	274/282
Eukaryota	Fungi	Postia placenta Mad-698-R	XP_002474101.1	3 E-13	263/282
Eukaryota	Fungi	Neosartorya fischeri NRRL 181	XP_001260113.1	4 E-13	281/282
Eukaryota	Fungi	Botryotinia fuckeliana B05.10	XP_001560054.1	5 E-13	251/282
Eukaryota	Fungi	Ajellomyces capsulatus H143	EER44411.1	5 E-13	272/282
Eukaryota	Fungi	Aspergillus clavatus NRRL 1	XP_001272710.1	5 E-13	281/282
Eukaryota	Fungi	Aspergillus clavatus NRRL 1	XP_001275870.1	7 E-13	269/282
Eukaryota	Fungi	Podospora anserina DSM 980	XP_001907152.1	8 E-13	270/282
Eukaryota	Fungi	Aspergillus flavus NRRL3357	XP_002375525.1	9 E-13	268/282
Eukaryota	Fungi	Aspergillus oryzae RIB40	XP_001727278.1	1 E-12	268/282
Eukaryota	Fungi	Aspergillus fumigatus Af293	XP_755921.1	1 E-12	268/282
Eukaryota	Fungi	Laccaria bicolor S238N-H82	XP_001884234.1	2 E-12	268/282
Eukaryota	Fungi	Ajellomyces capsulatus NAm1	XP_001541137.1	3 E-12	271/282
Eukaryota	Fungi	Neosartorya fischeri NRRL 181	XP_001261049.1	4 E-12	246/282
Eukaryota	Fungi	Gibberella zeae PH-1	XP_389769.1	5 E-12	262/282
Eukaryota	Fungi	Talaromyces stipitatus ATCC 10500	XP_002488071.1	5 E-12	274/282
Eukaryota	Fungi	Ajellomyces dermatitidis ER-3	EEQ87337.1	6 E-12	271/282
Eukaryota	Fungi	Chaetomium globosum CBS 148.51	XP_001219290.1	6 E-12	228/282
Eukaryota	Fungi	Uncinocarpus reesii 1704	XP_002543336.1	6 E-12	232/282
Eukaryota	Fungi	Aspergillus nidulans FGSC A4	XP_681202.1	6 E-12	226/282
Eukaryota	Fungi	Ajellomyces capsulatus G186AR	EEH07412.1	6 E-12	271/282
Eukaryota	Fungi	Gibberella zeae PH-1	XP_383946.1	6 E-12	272/282
Eukaryota	Fungi	Nectria haematococca mpVI 77-13-4	EEU37522.1	7 E-12	256/282
Eukaryota	Fungi	Aspergillus flavus NRRL3357	XP_002375105.1	1 E-11	278/282
Eukaryota	Fungi	Nectria haematococca mpVI 77-13-4	EEU46849.1	1 E-11	278/282
Eukaryota	Fungi	Aspergillus fumigatus A1163	EDP48581.1	1 E-11	244/282
Eukaryota	Fungi	Aspergillus niger CBS 513.88	XP_001391626.1	2 E-11	305/282
Eukaryota	Fungi	Nectria haematococca mpVI 77-13-4	EEU46554.1	3 E-11	267/282
Eukaryota	Fungi	Gibberella zeae PH-1	XP_383811.1	3 E-11	264/282
Eukaryota	Fungi	Ajellomyces capsulatus G186AR	EEH06366.1	3 E-11	271/282
Eukaryota	Fungi	Gibberella zeae PH-1	XP_390366.1	3 E-11	230/282
Eukaryota	Fungi	Aspergillus nidulans FGSC A4	XP_659769.1	3 E-11	268/282
Eukaryota	Fungi	Aspergillus nidulans FGSC A4	CBF86301.1	3 E-11	273/282
Eukaryota	Fungi	Nectria haematococca mpVI 77-13-4	EEU39245.1	3 E-11	272/282
Eukaryota	Fungi	Coccidioides immitis RS;	XP_001246309.1	4 E-11	229/282
Eukaryota	Fungi	Coccidioides immitis RS;	XP_001238876.1	4 E-11	271/282
Eukaryota	Fungi	Penicillium marneffei ATCC 18224	XP_002144385.1	4 E-11	271/282
Eukaryota	Fungi	Penicillium marneffei ATCC 18224	XP_002144384.1	5 E-11	271/282
Eukaryota	Fungi	Ajellomyces capsulatus G186AR	EEH03849.1	5 E-11	254/282
Eukaryota	Fungi	Nectria haematococca mpVI 77-13-4	EEU36252.1	5 E-11	272/282
Eukaryota	Fungi	Phaeosphaeria nodorum SN15	XP_001790808.1	8 E-11	279/282
Eukaryota	Fungi	Talaromyces stipitatus ATCC 10500	XP_002479048.1	8 E-11	270/282
Eukaryota	Fungi	Penicillium chrysogenum Wisconsin 54-1255	XP_002563489.1	9 E-11	274/282
Eukaryota	Fungi	Gibberella zeae PH-1	XP_382596.1	9 E-11	252/282

AFUA_6G09730

Eukaryota	Fungi	Aspergillus fumigatus Af293	XP_750862.1	0.0	504/504
Eukaryota	Fungi	Neosartorya fischeri NRRL 181	XP_001258090.1	0.0	504/504
Eukaryota	Fungi	Aspergillus terreus NIH2624	XP_001212649.1	1 E-141	481/504
Eukaryota	Fungi	Sclerotinia sclerotiorum 1980 UF-70	XP_001589517.1	7 E-67	509/504
Eukaryota	Fungi	Sclerotinia sclerotiorum 1980 UF-70	XP_001589913.1	2 E-66	504/504
Eukaryota	Fungi	Botryotinia fuckeliana	CAP58781.1	1 E-64	468/504
Eukaryota	Fungi	Botryotinia fuckeliana B05.10	XP_001545699.1	1 E-64	501/504
Eukaryota	Fungi	Sclerotinia sclerotiorum 1980 UF-70	XP_001589514.1	2 E-63	480/504
Eukaryota	Fungi	Microsporum canis CBS 113480	EEQ35339.1	2 E-60	507/504
Eukaryota	Fungi	Botryotinia fuckeliana B05.10	XP_001545655.1	2 E-59	510/504
Eukaryota	Fungi	Microsporum canis CBS 113480	EEQ29819.1	2 E-58	453/504
Eukaryota	Fungi	Penicillium chrysogenum Wisconsin 54-1255	XP_002557476.1	2 E-57	445/504
Eukaryota	Fungi	Nectria haematococca mpVI 77-13-4	EEU42024.1	6 E-57	498/504
Eukaryota	Fungi	Neosartorya fischeri NRRL 181	XP_001264200.1	2 E-56	490/504
Eukaryota	Fungi	Aspergillus nidulans FGSC A4	XP_659202.1	3 E-56	448/504
Eukaryota	Fungi	Aspergillus nidulans FGSC A4	CBF85188.1	4 E-56	448/504
Eukaryota	Fungi	Verticillium albo-atrum VaMs.102	EEY23335.1	5 E-56	496/504
Eukaryota	Fungi	Gibberella zeae PH-1	XP_382044.1	2 E-55	492/504
Eukaryota	Fungi	Uncinocarpus reesii 1704	XP_002543353.1	7 E-53	443/504
Eukaryota	Fungi	Coccidioides posadasii C735 delta	EER26878.1	1 E-52	441/504
Eukaryota	Fungi	Penicillium marneffei ATCC 18224	XP_002151521.1	5 E-51	494/504
Eukaryota	Fungi	Gibberella fujikuroi	O94142.1	6 E-51	433/504
Eukaryota	Fungi	Phoma betae	BAD29968.1	7 E-51	446/504
Eukaryota	Fungi	Phaeosphaeria nodorum SN15	XP_001790996.1	3 E-50	493/504
Eukaryota	Fungi	Gibberella moniliformis	CAQ16961.1	4 E-50	435/504
Eukaryota	Fungi	Fusarium proliferatum	Q701P2.1	1 E-49	433/504
Eukaryota	Fungi	Microsporum canis CBS 113480	EEQ29648.1	1 E-49	421/504
Eukaryota	Fungi	Neosartorya fischeri NRRL 181	XP_001262311.1	5 E-49	443/504
Eukaryota	Fungi	Aspergillus flavus NRRL3357	XP_002379999.1	2 E-48	447/504
Eukaryota	Fungi	Phanerochaete chrysosporium	BAD94562.1	5 E-47	445/504
Eukaryota	Fungi	Taiwanofungus camphoratus	AAU05113.1	1 E-46	439/504
Eukaryota	Fungi	Aspergillus nidulans FGSC A4	XP_660857.1	2 E-46	440/504
Eukaryota	Fungi	Aspergillus clavatus NRRL 1	XP_001274956.1	3 E-46	449/504
Eukaryota	Fungi	Microsporum canis CBS 113480	EEQ28921.1	3 E-46	480/504
Eukaryota	Fungi	Aspergillus clavatus NRRL 1	XP_001270544.1	4 E-45	459/504
Eukaryota	Fungi	Aspergillus niger CBS 513.88	XP_001398729.1	6 E-45	447/504
Eukaryota	Fungi	Gibberella zeae PH-1	XP_391458.1	9 E-45	490/504
Eukaryota	Fungi	Aspergillus oryzae RIB40	XP_001826049.1	1 E-44	447/504
Eukaryota	Fungi	Pyrenophora tritici-repentis Pt-1C-BFP	XP_001942213.1	4 E-44	416/504
Eukaryota	Fungi	Trametes versicolor	BAB59027.1	1 E-43	492/504
Eukaryota	Fungi	Aspergillus nidulans FGSC A4	CBF83053.1	1 E-43	432/504
Eukaryota	Fungi	Penicillium marneffei ATCC 18224	XP_002151527.1	2 E-43	438/504
Eukaryota	Fungi	Aspergillus nidulans FGSC A4	XP_660878.1	2 E-43	432/504
Eukaryota	Fungi	Sphaceloma manihoticola	CAP07652.1	4 E-43	506/504
Eukaryota	Fungi	Magnaporthe grisea 70-15	XP_362671.1	6 E-43	489/504
Eukaryota	Fungi	Aspergillus clavatus NRRL 1	XP_001267987.1	1 E-42	433/504
Eukaryota	Fungi	Gibberella zeae PH-1	XP_382848.1	1 E-42	443/504
Eukaryota	Fungi	Aspergillus nidulans FGSC A4	XP_681799.1	1 E-42	428/504
Eukaryota	Fungi	Penicillium marneffei ATCC 18224	XP_002149366.1	3 E-42	473/504
Eukaryota	Fungi	Chaetomium globosum CBS 148.51	XP_001221376.1	4 E-42	493/504
Eukaryota	Fungi	Gibberella fujikuroi	CAA75565.1	7 E-42	424/504
Eukaryota	Fungi	Fusarium proliferatum	ABC46410.2	8 E-42	424/504
Eukaryota	Fungi	Talaromyces stipitatus ATCC 10500	XP_002484399.1	8 E-42	421/504
Eukaryota	Fungi	Laccaria bicolor S238N-H82	XP_001874991.1	1 E-41	434/504
Eukaryota	Fungi	Magnaporthe grisea 70-15	XP_364353.2	1 E-41	495/504
Eukaryota	Fungi	Penicillium paxilli	AAK11528.1	2 E-41	436/504

Eukaryota	Fungi	Aspergillus fumigatus A1163	EDP49385.1	2 E-41	429/504
Eukaryota	Fungi	Verticillium albo-atrum VaMs.102	EEY15296.1	2 E-41	426/504
Eukaryota	Fungi	Nectria haematococca mpVI 77-13-4	EEU35509.1	3 E-41	459/504
Eukaryota	Fungi	Fusarium proliferatum	CAF31353.1	3 E-41	424/504
Eukaryota	Fungi	Aspergillus terreus NIH2624	XP_001209385.1	3 E-41	421/504
Eukaryota	Fungi	Penicillium marneffei ATCC 18224	XP_002149867.1	4 E-41	431/504
Eukaryota	Fungi	Aspergillus fumigatus Af293	XP_750704.2	5 E-41	429/504
Eukaryota	Fungi	Podospora anserina DSM 980	XP_001904206.1	6 E-41	413/504
Eukaryota	Fungi	Phanerochaete chrysosporium	BAD94560.1	1 E-40	450/504
Eukaryota	Fungi	Nectria haematococca mpVI 77-13-4	EEU38241.1	1 E-40	445/504
Eukaryota	Fungi	Talaromyces stipitatus ATCC 10500	XP_002482605.1	3 E-40	436/504
Eukaryota	Fungi	Neotyphodium lolii	AAW88512.1	6 E-40	433/504
Eukaryota	Fungi	Sphaceloma manihoticola	CAP07651.1	8 E-40	434/504
Eukaryota	Fungi	Laccaria bicolor S238N-H82	XP_001887937.1	1 E-39	441/504
Eukaryota	Fungi	Ajellomyces dermatitidis SLH14081	XP_002625800.1	2 E-39	443/504
Eukaryota	Fungi	Chaetomium globosum CBS 148.51	XP_001228644.1	2 E-39	455/504
Eukaryota	Fungi	Gibberella zeae PH-1	XP_391712.1	4 E-39	411/504
Eukaryota	Fungi	Ajellomyces dermatitidis ER-3	EEQ91250.1	7 E-39	479/504
Eukaryota	Fungi	Podospora anserina DSM 980	XP_001904516.1	9 E-39	426/504
Eukaryota	Fungi	Ajellomyces dermatitidis SLH14081	XP_002629412.1	1 E-38	479/504
Eukaryota	Fungi	Magnaporthe grisea 70-15	XP_361360.2	1 E-38	461/504
Eukaryota	Fungi	Magnaporthe grisea 70-15	XP_363465.2	2 E-38	407/504
Eukaryota	Fungi	Penicillium chrysogenum Wisconsin 54-1255	XP_002568640.1	3 E-38	445/504
Eukaryota	Fungi	Neurospora crassa OR74A	XP_963189.1	5 E-38	431/504
Eukaryota	Fungi	Laccaria bicolor S238N-H82	XP_001885921.1	7 E-38	435/504
Eukaryota	Fungi	Paracoccidioides brasiliensis Pb03;	EEH18213.1	7 E-38	448/504
Eukaryota	Fungi	Chaetomium globosum CBS 148.51	XP_001227007.1	9 E-38	471/504
Eukaryota	Fungi	Magnaporthe grisea 70-15	XP_370137.2	1 E-37	441/504
Eukaryota	Fungi	Ajellomyces capsulatus H143	EER45596.1	1 E-37	429/504
Eukaryota	Fungi	Coprinopsis cinerea okayama7#130	XP_001833906.1	1 E-37	451/504
Eukaryota	Fungi	Aspergillus flavus NRRL3357	XP_002384778.1	1 E-37	421/504
Eukaryota	Fungi	Magnaporthe grisea 70-15	XP_369220.1	1 E-37	442/504
Eukaryota	Fungi	Penicillium marneffei ATCC 18224	XP_002148347.1	1 E-37	408/504
Eukaryota	Fungi	Ajellomyces dermatitidis ER-3	EEQ86913.1	3 E-37	420/504
Eukaryota	Fungi	Aspergillus niger CBS 513.88	XP_001394122.1	3 E-37	446/504
Eukaryota	Fungi	Penicillium chrysogenum Wisconsin 54-1255	XP_002564993.1	3 E-37	451/504
Eukaryota	Fungi	Aspergillus clavatus NRRL 1	XP_001276220.1	3 E-37	439/504
Eukaryota	Fungi	Pyrenophora tritici-repentis Pt-1C-BFP	XP_001936495.1	5 E-37	420/504
Eukaryota	Fungi	Nectria haematococca mpVI 77-13-4	EEU44957.1	5 E-37	427/504
Eukaryota	Fungi	Coprinopsis cinerea okayama7#130	XP_001833961.1	5 E-37	447/504
Eukaryota	Fungi	Talaromyces stipitatus ATCC 10500	XP_002340886.1	7 E-37	461/504
Eukaryota	Fungi	Penicillium chrysogenum Wisconsin 54-1255	XP_002569197.1	2 E-36	433/504
Eukaryota	Fungi	Paracoccidioides brasiliensis Pb01;	EEH41802.1	2 E-36	448/504
Eukaryota	Fungi	Talaromyces stipitatus ATCC 10500	XP_002486599.1	2 E-36	443/504
Eukaryota	Fungi	Botryotinia fuckeliana B05.10	XP_001553637.1	4 E-36	432/504
Eukaryota	Fungi	Phanerochaete chrysosporium	BAD94556.1	6 E-36	435/504
Eukaryota	Fungi	Ajellomyces capsulatus NAm1	XP_001537727.1	7 E-36	434/504
Eukaryota	Fungi	Penicillium chrysogenum Wisconsin 54-1255	XP_002560434.1	8 E-36	490/504
Eukaryota	Fungi	Microsporum canis CBS 113480	EEQ34674.1	9 E-36	521/504
Eukaryota	Fungi	Aspergillus clavatus NRRL 1	XP_001273589.1	1 E-35	467/504
Eukaryota	Fungi	Magnaporthe grisea 70-15	XP_363774.2	2 E-35	435/504
Eukaryota	Fungi	Verticillium albo-atrum VaMs.102	EEY21752.1	2 E-35	471/504
Eukaryota	Fungi	Paracoccidioides brasiliensis Pb18;	EEH47128.1	2 E-35	444/504
Eukaryota	Fungi	Podospora anserina DSM 980	XP_001906083.1	4 E-35	459/504
Eukaryota	Fungi	Gibberella fujikuroi	CAA75566.1	6 E-35	418/504
Eukaryota	Fungi	Neosartorya fischeri NRRL 181	XP_001266222.1	8 E-35	440/504
Eukaryota	Fungi	Neotyphodium lolii	ABF20221.1	1 E-34	441/504
Eukaryota	Fungi	Talaromyces stipitatus ATCC 10500	XP_002485883.1	1 E-34	441/504

Eukaryota	Fungi	Gibberella zeae PH-1	XP_384893.1	2 E-34	432/504
Eukaryota	Fungi	Magnaporthe grisea 70-15	XP_001523050.1	2 E-34	422/504
Eukaryota	Fungi	Neurospora crassa OR74A	XP_958493.1	2 E-34	496/504
Eukaryota	Fungi	Magnaporthe grisea 70-15	XP_367028.2	5 E-34	443/504
Eukaryota	Fungi	Aspergillus clavatus NRRL 1	XP_001270548.1	6 E-34	448/504
Eukaryota	Fungi	Aspergillus oryzae RIB40	XP_001822674.1	8 E-34	436/504
Eukaryota	Fungi	Coprinopsis cinerea okayama7#130	XP_001833905.1	9 E-34	448/504
Eukaryota	Fungi	Coprinopsis cinerea okayama7#130	XP_001833945.1	1 E-33	479/504
Eukaryota	Fungi	Podospora anserina DSM 980	XP_001910956.1	1 E-33	441/504
Eukaryota	Fungi	Penicillium paxilli	AAK11527.1	1 E-33	443/504
Eukaryota	Fungi	Penicillium marneffei ATCC 18224	XP_002152244.1	2 E-33	475/504
Eukaryota	Fungi	Sphaceloma manihoticola	CAP07653.1	2 E-33	432/504
Eukaryota	Fungi	Talaromyces stipitatus ATCC 10500	XP_002477798.1	2 E-33	444/504
Eukaryota	Fungi	Talaromyces stipitatus ATCC 10500	XP_002485808.1	3 E-33	486/504
Eukaryota	Fungi	Coccidioides immitis RS;	XP_001243722.1	3 E-33	495/504
Eukaryota	Fungi	Nectria haematococca mpVI 77-13-4	EEU40020.1	4 E-33	418/504
Eukaryota	Fungi	Aspergillus flavus NRRL3357	XP_002378016.1	6 E-33	436/504
Eukaryota	Fungi	Coccidioides posadasii C735 delta	EER26820.1	8 E-33	455/504
Eukaryota	Fungi	Fusarium sporotrichioides;	AAK77224.1	2 E-32	435/504
Eukaryota	Fungi	Gibberella zeae PH-1	XP_380247.1	3 E-32	413/504
Eukaryota	Fungi	Laccaria bicolor S238N-H82	XP_001888861.1	4 E-32	450/504
Eukaryota	Fungi	Botryotinia fuckeliana B05.10	XP_001553254.1	5 E-32	444/504
Eukaryota	Fungi	Fusarium sporotrichioides;	AAK77933.1	6 E-32	435/504
Eukaryota	Fungi	Magnaporthe grisea 70-15	XP_365570.1	7 E-32	441/504
Eukaryota	Fungi	Gibberella zeae PH-1	XP_382293.1	1 E-31	465/504
Eukaryota	Fungi	Aspergillus flavus	CAP53941.1	2 E-31	436/504
Eukaryota	Fungi	Phanerochaete chrysosporium	BAD94566.1	2 E-31	469/504
Eukaryota	Fungi	Pyrenophora tritici-repentis Pt-1C-BFP	XP_001933345.1	2 E-31	457/504
Eukaryota	Fungi	Aspergillus flavus NRRL3357	XP_002382783.1	3 E-31	452/504
Eukaryota	Fungi	Chaetomium globosum CBS 148.51	XP_001227324.1	4 E-31	423/504
Eukaryota	Fungi	Aspergillus niger CBS 513.88	XP_001394884.1	4 E-31	421/504
Eukaryota	Fungi	Fusarium sporotrichioides;	AAO64248.1	6 E-31	429/504
Eukaryota	Fungi	Aspergillus fumigatus Af293	XP_748033.1	7 E-31	460/504
Eukaryota	Fungi	Aspergillus oryzae RIB40	XP_001826327.1	7 E-31	436/504
Eukaryota	Fungi	Aspergillus flavus NRRL3357	XP_002380007.1	9 E-31	432/504
Eukaryota	Fungi	Phaeosphaeria sp. L487	BAF33070.1	9 E-31	414/504
Eukaryota	Fungi	Aspergillus fumigatus A1163	EDP51159.1	1 E-30	460/504
Eukaryota	Fungi	Neurospora crassa OR74A	XP_958581.1	7 E-30	470/504
Eukaryota	Fungi	Neotyphodium lolii	ABF20222.1	7 E-30	437/504
Eukaryota	Fungi	Magnaporthe grisea 70-15	XP_368672.1	4 E-29	473/504
Eukaryota	Fungi	Magnaporthe grisea 70-15	XP_366125.1	4 E-29	438/504
Eukaryota	Fungi	Magnaporthe grisea 70-15	XP_366308.1	9 E-29	406/504
Eukaryota	Fungi	Aspergillus terreus NIH2624	XP_001213596.1	1 E-28	469/504
Eukaryota	Fungi	Coprinopsis cinerea okayama7#130	XP_001833943.1	3 E-28	452/504
Eukaryota	Fungi	Aspergillus terreus NIH2624	XP_001211630.1	3 E-28	440/504
Eukaryota	Fungi	Penicillium chrysogenum Wisconsin 54-1255	XP_002564021.1	7 E-28	438/504
Eukaryota	Fungi	Phaeosphaeria nodorum SN15	XP_001801175.1	8 E-28	447/504
Eukaryota	Fungi	Microsporum canis CBS 113480	EEQ32730.1	1 E-27	435/504
Eukaryota	Fungi	Aspergillus clavatus NRRL 1	XP_001272469.1	2 E-27	445/504
Eukaryota	Fungi	Aspergillus nidulans FGSC A4	XP_661101.1	2 E-27	459/504
Eukaryota	Fungi	Podospora anserina DSM 980	XP_001905687.1	3 E-27	433/504
Eukaryota	Fungi	Moniliophthora perniciosa FA553	XP_002390475.1	6 E-27	423/504
Eukaryota	Fungi	Aspergillus oryzae RIB40	XP_001818600.1	7 E-27	435/504

AFUA_6G09740

Eukaryota	Fungi	Aspergillus fumigatus Af293	XP_750863.1	0.0	334/334
Eukaryota	Fungi	Neosartorya fischeri NRRL 181	XP_001258091.1	0.0	334/334

Eukaryota	Fungi	Aspergillus terreus NIH2624	XP_001214313.1	1 E-160	335/334
Eukaryota	Fungi	Aspergillus oryzae RIB40	XP_001820833.1	5 E-94	305/334
Eukaryota	Fungi	Aspergillus flavus NRRL3357	XP_002376556.1	2 E-93	305/334
Eukaryota	Fungi	Talaromyces stipitatus ATCC 10500	XP_002484596.1	2 E-92	311/334
Eukaryota	Fungi	Penicillium marneffei ATCC 18224	XP_002149524.1	1 E-88	309/334
Eukaryota	Fungi	Aspergillus nidulans FGSC A4	XP_661567.1	5 E-82	305/334
Eukaryota	Fungi	Nectria haematococca mpVI 77-13-4	EEU39801.1	1 E-81	306/334
Eukaryota	Fungi	Magnaporthe grisea 70-15	XP_367837.1	3 E-80	315/334
Eukaryota	Fungi	Gibberella zeae PH-1	XP_386674.1	1 E-75	306/334
Eukaryota	Fungi	Verticillium albo-atrum VaMs.102	EEY21344.1	6 E-74	313/334
Eukaryota	Fungi	Botryotinia fuckeliana B05.10	XP_001557506.1	9 E-70	304/334
Eukaryota	Fungi	Aspergillus clavatus NRRL 1	XP_001272470.1	4 E-68	309/334
Eukaryota	Fungi	Aspergillus flavus NRRL3357	XP_002380010.1	1 E-67	306/334
Eukaryota	Fungi	Aspergillus flavus NRRL3357	XP_002380008.1	2 E-67	309/334
Eukaryota	Fungi	Aspergillus flavus NRRL3357	XP_002381675.1	2 E-67	306/334
Eukaryota	Fungi	Aspergillus oryzae RIB40	XP_001818602.1	3 E-67	306/334
Eukaryota	Fungi	Aspergillus clavatus NRRL 1	XP_001272472.1	4 E-67	304/334
Eukaryota	Fungi	Phaeosphaeria nodorum SN15	XP_001796489.1	1 E-66	311/334
Eukaryota	Fungi	Leptosphaeria maculans	AAS92555.1	3 E-64	308/334
Eukaryota	Fungi	Hypocrea virens	ABV48723.1	2 E-63	312/334
Eukaryota	Fungi	Aspergillus niger CBS 513.88	XP_001395364.1	8 E-58	324/334
Eukaryota	Fungi	Aspergillus oryzae RIB40	XP_001825009.1	5 E-57	290/334
Eukaryota	Fungi	Gibberella zeae PH-1	XP_380219.1	5 E-53	300/334
Eukaryota	Fungi	Phaeosphaeria nodorum SN15	XP_001797128.1	9 E-50	312/334
Eukaryota	Fungi	Aspergillus niger CBS 513.88	XP_001402401.1	1 E-38	308/334
Eukaryota	Fungi	Chaetomium globosum CBS 148.51	XP_001226010.1	1 E-35	318/334
Bacteria	Bacteroidetes	Flavobacterium johnsoniae UW101	YP_001193282.1	1 E-34	290/334
Bacteria	Bacteroidetes	Chryseobacterium gleum ATCC 35910	ZP_03851675.1	2 E-34	295/334
Eukaryota	Fungi	Aspergillus flavus NRRL3357	XP_002382835.1	3 E-34	313/334
Eukaryota	Fungi	Aspergillus oryzae RIB40	XP_001822715.1	3 E-34	313/334
Eukaryota	Fungi	Aspergillus terreus NIH2624	XP_001216679.1	6 E-33	292/334
Eukaryota	Fungi	Aspergillus nidulans FGSC A4	XP_681487.1	6 E-33	292/334
Bacteria	Firmicutes	Bacillus pumilus SAFR-032	YP_001485916.1	1 E-32	300/334
Bacteria	Firmicutes	Bacillus pumilus ATCC 7061	ZP_03054633.1	1 E-32	293/334
Eukaryota	Fungi	Aspergillus nidulans FGSC A4	XP_664567.1	2 E-32	314/334
Eukaryota	Fungi	Gibberella zeae PH-1	XP_383434.1	2 E-32	298/334
Eukaryota	Fungi	Aspergillus flavus NRRL3357	XP_002380011.1	2 E-32	313/334
Eukaryota	Fungi	Aspergillus oryzae RIB40	XP_001818603.1	5 E-32	313/334
Eukaryota	Fungi	Aspergillus niger CBS 513.88	XP_001393905.1	1 E-31	315/334
Eukaryota	Fungi	Aspergillus niger	CAA62502.1	2 E-31	315/334
Bacteria	Bacteroidetes	Spirosoma linguale DSM 74	ZP_04487064.1	2 E-31	298/334
Bacteria	Verrucomicrobia	Verrucomicrobium spinosum DSM 4136	ZP_02928570.1	3 E-31	293/334
Bacteria	Proteobacteria	Oceanicola batsensis HTCC2597	ZP_01000665.1	1 E-30	284/334
Eukaryota	Fungi	Neosartorya fischeri NRRL 181	XP_001258605.1	1 E-30	307/334
Eukaryota	Fungi	Aspergillus terreus NIH2624	XP_001210799.1	1 E-30	291/334
Bacteria	Bacteroidetes	unidentified eubacterium SCB49	ZP_01891335.1	2 E-30	290/334
Eukaryota	Fungi	Aspergillus oryzae RIB40	XP_001824605.1	2 E-30	307/334
Eukaryota	Fungi	Aspergillus flavus	ABY86217.1	3 E-30	307/334
Eukaryota	Fungi	Aspergillus flavus	ABY86216.1	3 E-30	307/334
Bacteria	Bacteroidetes	Kordia algicida OT-1	ZP_02164341.1	3 E-30	282/334
Eukaryota	Fungi	Aspergillus flavus NRRL3357	XP_002384027.1	4 E-30	307/334
Bacteria	Bacteroidetes	Flavobacteria bacterium BBFL7	ZP_01202064.1	7 E-30	290/334
Eukaryota	Fungi	Aspergillus fumigatus Af293	XP_747990.1	1 E-29	291/334
Bacteria	Bacteroidetes	Pedobacter heparinus DSM 2366	YP_003093411.1	2 E-29	282/334
Bacteria	Bacteroidetes	Pedobacter sp. BAL39	ZP_01886302.1	2 E-29	290/334
Bacteria	Bacteroidetes	Chitinophaga pinensis DSM 2588	YP_003123102.1	3 E-29	281/334
Eukaryota	Fungi	Penicillium chrysogenum Wisconsin 54-1255	XP_002566175.1	6 E-29	289/334
Bacteria	Firmicutes	Brevibacillus brevis NBRC 100599	YP_002770834.1	7 E-29	296/334

Bacteria	Bacteroidetes	Cytophaga hutchinsonii ATCC 33406	YP_679167.1	7 E-29	270/334
Eukaryota	Fungi	Neosartorya fischeri NRRL 181	XP_001266180.1	9 E-29	291/334
Bacteria	Bacteroidetes	Chitinophaga pinensis DSM 2588	YP_003125253.1	1 E-28	282/334
Bacteria	Firmicutes	Geobacillus sp. Y412MC10	YP_003242698.1	1 E-28	271/334
Eukaryota	Fungi	Penicillium chrysogenum Wisconsin 54-1255	XP_002557249.1	2 E-28	306/334
Eukaryota	Fungi	Aspergillus clavatus NRRL 1	XP_001272473.1	5 E-28	315/334
Eukaryota	Fungi	Paracoccidioides brasiliensis Pb18;	EEH47549.1	6 E-28	315/334
Eukaryota	Fungi	Talaromyces stipitatus ATCC 10500	XP_002482758.1	6 E-28	289/334
Eukaryota	Fungi	Aspergillus clavatus NRRL 1	XP_001276178.1	1 E-27	293/334
Eukaryota	Fungi	Magnaporthe grisea 70-15	XP_001408027.1	2 E-27	322/334
Eukaryota	Fungi	Ajellomyces dermatitidis SLH14081	XP_002625018.1	2 E-27	292/334
Eukaryota	Fungi	Paracoccidioides brasiliensis Pb01;	EEH38268.1	3 E-27	315/334
Bacteria	Bacteroidetes	Algoriphagus sp. PR1	ZP_01720085.1	5 E-27	295/334
Bacteria	Proteobacteria	Citreicella sp. SE45	ZP_05782582.1	6 E-27	284/334
Eukaryota	Metazoa	Danio rerio	XP_001922162.1	1 E-26	287/334
Bacteria	Proteobacteria	Cellvibrio japonicus Ueda107	YP_001984188.1	1 E-26	287/334
Bacteria	Firmicutes	Bacillus halodurans C-125	NP_241523.1	1 E-26	295/334
Bacteria	Proteobacteria	Asticcacaulis excentricus CB 48	ZP_04770069.1	1 E-26	288/334
Eukaryota	Fungi	Ajellomyces capsulatus G186AR	EEH05762.1	2 E-26	283/334
Eukaryota	Fungi	Ajellomyces capsulatus H143	EER41620.1	2 E-26	292/334
Bacteria	Proteobacteria	Pseudomonas aeruginosa UCBPP-PA14	YP_791878.1	2 E-26	290/334
Eukaryota	Fungi	Penicillium marneffei ATCC 18224	XP_002148465.1	4 E-26	276/334
Eukaryota	Fungi	Penicillium chrysogenum Wisconsin 54-1255	XP_002563696.1	4 E-26	286/334
Eukaryota	Fungi	Microsporum canis CBS 113480	EEQ29680.1	5 E-26	297/334
Bacteria	Proteobacteria	Jannaschia sp. CCS1	YP_509249.1	7 E-26	280/334
Eukaryota	Fungi	Ajellomyces capsulatus NAm1	XP_001538266.1	7 E-26	292/334
Bacteria	Proteobacteria	Paracoccus denitrificans PD1222	YP_915220.1	1 E-25	275/334
Bacteria	Firmicutes	Bacillus licheniformis ATCC 14580	YP_079255.1	1 E-25	271/334
Bacteria	Thermus	Deinococcus geothermalis DSM 11300	YP_604444.1	1 E-25	279/334
Bacteria	Proteobacteria	Agrobacterium radiobacter K84	YP_002543147.1	2 E-25	284/334
Eukaryota	Fungi	Nectria haematococca mpVI 77-13-4	EEU38161.1	2 E-25	268/334
Bacteria	Spirochaetes	Leptospira biflexa serovar Patoc	YP_001839955.1	3 E-25	286/334
Bacteria	Proteobacteria	Rhizobium etli CFN 42	YP_471986.1	4 E-25	290/334
Bacteria	Actinobacteria	Mycobacterium marinum M	YP_001850206.1	6 E-25	294/334
Bacteria	Proteobacteria	Marinomonas sp. MWYL1	YP_001339240.1	7 E-25	290/334
Bacteria	Proteobacteria	Burkholderia ambifaria MC40-6	YP_001808691.1	8 E-25	292/334
Bacteria	Proteobacteria	Agrobacterium radiobacter K84	YP_002543106.1	9 E-25	291/334
Bacteria	Actinobacteria	Janibacter sp. HTCC2649	ZP_00996393.1	9 E-25	292/334
Bacteria	Proteobacteria	Acinetobacter radioresistens SK82	ZP_05361086.1	9 E-25	284/334
Eukaryota	Fungi	Ajellomyces dermatitidis ER-3	EEQ87358.1	1 E-24	282/334
Bacteria	Proteobacteria	Burkholderia thailandensis Bt4	ZP_02388616.1	2 E-24	285/334
Bacteria	Proteobacteria	Burkholderia thailandensis E264	YP_442880.1	2 E-24	285/334
Bacteria	Proteobacteria	Burkholderia cenocepacia MC0-3	YP_001773904.1	3 E-24	290/334
Bacteria	Proteobacteria	Xanthomonas campestris pv. campestris	YP_001902056.1	3 E-24	282/334
Bacteria	Proteobacteria	Chelativorans sp. BNC1	YP_674763.1	4 E-24	292/334
Bacteria	Proteobacteria	Ralstonia eutropha JMP134	YP_298639.1	4 E-24	290/334
Bacteria	Firmicutes	Carnobacterium sp. AT7	ZP_02184487.1	5 E-24	291/334
Bacteria	Proteobacteria	Pseudomonas fluorescens SBW25	YP_002873199.1	5 E-24	286/334
Bacteria	Proteobacteria	Burkholderia cenocepacia MC0-3	YP_001764417.1	6 E-24	290/334
Bacteria	Proteobacteria	Burkholderia ambifaria MEX-5	ZP_02909512.1	6 E-24	290/334
Bacteria	Proteobacteria	Burkholderia cenocepacia PC184	ZP_04939213.1	9 E-24	290/334
Eukaryota	Fungi	Debaryomyces hansenii CBS767	XP_462485.1	1 E-23	291/334
Bacteria	Chloroflexi	Herpetosiphon aurantiacus ATCC 23779	YP_001546557.1	2 E-23	271/334
Bacteria	Proteobacteria	Burkholderia ambifaria IOP40-10	ZP_02888303.1	2 E-23	290/334
Bacteria	Proteobacteria	Pseudomonas fluorescens Pf0-1	YP_348786.1	2 E-23	290/334
Bacteria	Proteobacteria	Xanthomonas campestris pv. campestris	NP_638890.1	2 E-23	282/334
Bacteria	Firmicutes	Bacillus clausii KSM-K16	YP_174796.1	3 E-23	293/334
Bacteria	Thermus	Deinococcus deserti VCD115	YP_002785541.1	3 E-23	279/334

Bacteria	Proteobacteria	Pseudomonas aeruginosa 2192	ZP_04934978.1	5 E-23	280/334
Bacteria	Proteobacteria	Burkholderia cenocepacia J2315	YP_002231676.1	5 E-23	279/334
Bacteria	Proteobacteria	Burkholderia thailandensis MSMB43	ZP_02468192.1	6 E-23	285/334
Bacteria	Proteobacteria	Rhizobium leguminosarum bv. trifolii	YP_002284283.1	6 E-23	290/334
Bacteria	Proteobacteria	Rhizobium etli CIAT 652	YP_001985957.1	7 E-23	290/334
Bacteria	Proteobacteria	Pseudomonas aeruginosa 2192	ZP_04936176.1	8 E-23	287/334
Bacteria	Proteobacteria	Burkholderia sp. H160	ZP_03269081.1	9 E-23	290/334
Bacteria	Firmicutes	Paenibacillus sp. oral taxon	ZP_04851037.1	1 E-22	286/334
Bacteria	Proteobacteria	Rhizobium etli Brasil 5	ZP_03508282.1	2 E-22	290/334
Bacteria	Proteobacteria	Bordetella petrii DSM 12804	YP_001631595.1	2 E-22	270/334
Bacteria	Proteobacteria	Pseudomonas aeruginosa UCBPP-PA14	YP_788944.1	2 E-22	287/334
Bacteria	Proteobacteria	Janthinobacterium sp. Marseille	YP_001353597.1	2 E-22	290/334
Bacteria	Proteobacteria	Dickeya zeae Ech1591	YP_003003514.1	3 E-22	290/334
Bacteria	Proteobacteria	Pseudomonas aeruginosa LESB58	YP_002438365.1	3 E-22	287/334
Bacteria	Proteobacteria	Pseudomonas aeruginosa PAO1	NP_252859.1	3 E-22	287/334
Bacteria	Actinobacteria	Kineococcus radiotolerans SRS30216	YP_001360261.1	3 E-22	274/334
Bacteria	Proteobacteria	Agrobacterium tumefaciens str. C58	NP_353269.1	3 E-22	270/334
Eukaryota	Fungi	Kluyveromyces lactis NRRL Y-1140	XP_455756.1	4 E-22	280/334
Bacteria	Proteobacteria	Rhizobium leguminosarum bv. viciae	YP_771095.1	4 E-22	284/334
Bacteria	Proteobacteria	Methylobacterium populi BJ001	YP_001927164.1	4 E-22	268/334
Bacteria	Actinobacteria	Brachybacterium faecium DSM 4810	YP_003153889.1	4 E-22	280/334
Bacteria	Proteobacteria	Coxiella burnetii Dugway 5J108-111	YP_001424133.1	5 E-22	286/334
Bacteria	Proteobacteria	Agrobacterium tumefaciens str. C58	NP_354617.1	6 E-22	290/334
Bacteria	Proteobacteria	Rhizobium leguminosarum bv. trifolii	YP_002985080.1	7 E-22	287/334
Bacteria	Proteobacteria	Pseudomonas aeruginosa PACS2	ZP_01367605.1	8 E-22	286/334
Bacteria	Proteobacteria	Sinorhizobium meliloti 1021	NP_384466.1	9 E-22	282/334
Bacteria	Proteobacteria	Brevundimonas sp. BAL3	ZP_05033159.1	1 E-21	292/334
Bacteria	Proteobacteria	Stigmatella aurantiaca DW4/3-1	ZP_01460381.1	1 E-21	269/334
Bacteria	Proteobacteria	Acidovorax delafieldii 2AN	ZP_04762652.1	1 E-21	280/334
Bacteria	Proteobacteria	Variovorax paradoxus S110	YP_002942028.1	1 E-21	287/334
Bacteria	Proteobacteria	Pseudomonas aeruginosa C3719	ZP_04930373.1	2 E-21	287/334
Eukaryota	Fungi	Aspergillus terreus NIH2624	XP_001208363.1	2 E-21	300/334
Bacteria	Verrucomicrobia	Opitutus terrae PB90-1	YP_001817661.1	2 E-21	268/334
Bacteria	Actinobacteria	Mycobacterium sp. MCS	YP_642353.1	2 E-21	294/334
Bacteria	Proteobacteria	Methylobacterium chloromethanicum CM4	YP_002423123.1	3 E-21	268/334
Bacteria	Proteobacteria	Methylobacterium extorquens PA1	YP_001641490.1	3 E-21	268/334
Bacteria	Proteobacteria	Burkholderia cenocepacia MC0-3	YP_001777590.1	3 E-21	289/334
Bacteria	Proteobacteria	Methylobacterium extorquens DM4	YP_003070471.1	3 E-21	268/334
Bacteria	Actinobacteria	Corynebacterium efficiens YS-314	ZP_05751275.1	3 E-21	291/334
Bacteria	Actinobacteria	Mycobacterium sp. KMS	YP_941261.1	3 E-21	294/334
Bacteria	Actinobacteria	Corynebacterium efficiens YS-314	NP_739495.1	4 E-21	291/334
Bacteria	Proteobacteria	Coxiella burnetii 'MSU Goat	ZP_01946065.1	4 E-21	286/334
Bacteria	Proteobacteria	Jannaschia sp. CCS1	YP_510197.1	5 E-21	295/334
Bacteria	Actinobacteria	Mycobacterium sp. MCS	YP_640503.1	5 E-21	292/334
Bacteria	Proteobacteria	Sinorhizobium medicae WSM419	YP_001329218.1	9 E-21	289/334
Bacteria	Proteobacteria	Haliangium ochraceum DSM 14365	ZP_03876959.1	9 E-21	268/334
Bacteria	Proteobacteria	Acinetobacter sp. ATCC 27244	ZP_03823252.1	9 E-21	279/334
Bacteria	Actinobacteria	Mycobacterium smegmatis str. MC2	YP_888580.1	1 E-20	277/334
Bacteria	Proteobacteria	Coxiella burnetii RSA 493	NP_819745.1	1 E-20	286/334
Bacteria	Actinobacteria	Clavibacter michiganensis subsp. sepedonicus	YP_001709638.1	1 E-20	279/334
Bacteria	Proteobacteria	Burkholderia cenocepacia HI2424	YP_837203.1	1 E-20	289/334
Bacteria	Actinobacteria	Mycobacterium vanbaalenii PYR-1	YP_952006.1	1 E-20	279/334
Bacteria	Actinobacteria	Streptosporangium roseum DSM 43021	ZP_04478739.1	2 E-20	287/334
Bacteria	Proteobacteria	Aurantimonas manganoxydans SI85-9A1	ZP_01227975.1	2 E-20	285/334
Bacteria	Proteobacteria	Pseudovibrio sp. JE062	ZP_05086488.1	2 E-20	280/334
Bacteria	Proteobacteria	Hyphomonas neptunium ATCC 15444	YP_761418.1	2 E-20	281/334
Bacteria	Actinobacteria	Brevibacterium linens BL2	ZP_05914137.1	3 E-20	327/334
Bacteria	Proteobacteria	Methylobacterium extorquens AM1	YP_002965347.1	4 E-20	268/334

Bacteria	Firmicutes	Paenibacillus sp. JDR-2	YP_003009141.1	5 E-20	295/334
Bacteria	Proteobacteria	Burkholderia cenocepacia J2315	YP_002233222.1	5 E-20	286/334
Bacteria	Proteobacteria	Burkholderia cenocepacia PC184	ZP_04942387.1	6 E-20	289/334
Bacteria	Actinobacteria	marine actinobacterium PHSC20C1	ZP_01131402.1	7 E-20	270/334
Bacteria	Proteobacteria	Burkholderia ambifaria MEX-5	ZP_02906969.1	7 E-20	286/334
Bacteria	Proteobacteria	Paracoccus denitrificans PD1222	YP_916897.1	8 E-20	284/334
Bacteria	Proteobacteria	Ochrobactrum intermedium LMG 3301	ZP_04682214.1	1 E-19	284/334
Bacteria	Proteobacteria	Burkholderia dolosa AUO158	ZP_04948285.1	1 E-19	276/334
Bacteria	Proteobacteria	Burkholderia sp. 383	YP_373303.1	1 E-19	285/334
Bacteria	Proteobacteria	Shewanella sp. ANA-3	YP_870170.1	2 E-19	285/334
Bacteria	Firmicutes	Bacillus cereus BDRD-ST196;	ZP_04262485.1	2 E-19	287/334
Bacteria	Firmicutes	Clostridium thermocellum DSM 2360	ZP_05430168.1	3 E-19	289/334
Bacteria	Proteobacteria	Ochrobactrum anthropi ATCC 49188	YP_001372120.1	4 E-19	284/334
Bacteria	Firmicutes	Clostridium thermocellum ATCC 27405	YP_001038356.1	5 E-19	289/334
Bacteria	Proteobacteria	Chromobacterium violaceum	ABP57752.1	7 E-19	283/334
Bacteria	Actinobacteria	Rhodococcus erythropolis PR4	YP_002765762.1	8 E-19	274/334
Bacteria	Actinobacteria	Rhodococcus erythropolis SK121	ZP_04384782.1	8 E-19	274/334
Bacteria	Planctomycetes	Blastopirellula marina DSM 3645	ZP_01091013.1	9 E-19	285/334
Bacteria	Proteobacteria	Burkholderia ambifaria MC40-6	YP_001810169.1	9 E-19	286/334
Bacteria	Actinobacteria	Corynebacterium aurimucosum ATCC 700975	YP_002833654.1	9 E-19	276/334
Bacteria	Actinobacteria	Mycobacterium gilvum PYR-GCK	YP_001136419.1	1 E-18	280/334
Bacteria	Thermus	Deinococcus radiodurans R1	NP_294135.1	1 E-18	295/334
Bacteria	Firmicutes	Clostridium papyrosolvens DSM 2782	ZP_05494361.1	1 E-18	288/334
Bacteria	Firmicutes	Bacillus cereus AH603;	ZP_04197804.1	2 E-18	286/334
Bacteria	Firmicutes	Bacillus weihenstephanensis KBAB4;	YP_001645402.1	2 E-18	286/334
Bacteria	Proteobacteria	Burkholderia ambifaria AMMD	YP_777186.1	2 E-18	286/334
Bacteria	Proteobacteria	Rhizobium leguminosarum bv. trifolii	YP_002278234.1	2 E-18	284/334
Bacteria	Proteobacteria	Burkholderia ambifaria IOP40-10	ZP_02888383.1	2 E-18	286/334
Bacteria	Proteobacteria	Acidovorax citrulli AAC00-1	YP_971669.1	2 E-18	280/334
Bacteria	Firmicutes	Bacillus mycoides DSM 2048	ZP_04169204.1	2 E-18	286/334
Bacteria	Firmicutes	Bacillus sp. SG-1	ZP_01858381.1	2 E-18	286/334
Bacteria	Firmicutes	Bacillus sp. NRRL B-14911	ZP_01171394.1	2 E-18	294/334
Bacteria	Proteobacteria	Rhodopseudomonas palustris HaA2	YP_487555.1	3 E-18	284/334
Bacteria	Proteobacteria	Xanthomonas campestris pv. vesicatoria	YP_362378.1	3 E-18	282/334
Bacteria	Firmicutes	Ruminococcus obeum ATCC 29174	ZP_01963629.1	3 E-18	305/334
Bacteria	Actinobacteria	Streptosporangium roseum DSM 43021	ZP_04477450.1	4 E-18	282/334
Bacteria	Actinobacteria	Stackebrandtia nassauensis DSM 44728	ZP_04485621.1	5 E-18	310/334
Bacteria	Gemmatimonadet	Gemmatimonas aurantiaca T-27	YP_002760464.1	9 E-18	283/334
Bacteria	Bacteroidetes	Microscilla marina ATCC 23134	ZP_01692936.1	9 E-18	298/334
Bacteria	Firmicutes	Paenibacillus sp. JDR-2	YP_003011705.1	1 E-17	292/334
Bacteria	Proteobacteria	Polaromonas naphthalenivorans CJ2	YP_973576.1	1 E-17	274/334
Bacteria	Actinobacteria	Salinispora arenicola CNS-205	YP_001539561.1	1 E-17	280/334
Bacteria	Firmicutes	Bacillus anthracis str. Sterne	YP_028840.1	2 E-17	286/334
Bacteria	Proteobacteria	Roseobacter sp. GAI101	ZP_05102780.1	2 E-17	283/334
Bacteria	Firmicutes	Bacillus cereus 03BB108;	ZP_03110899.1	2 E-17	286/334
Bacteria	Firmicutes	Bacillus anthracis str. Ames	NP_845117.1	2 E-17	286/334
Bacteria	Proteobacteria	Roseovarius sp. TM1035	ZP_01881142.1	2 E-17	280/334
Bacteria	Firmicutes	Bacillus halodurans C-125	NP_242359.1	2 E-17	286/334
Bacteria	Firmicutes	Bacillus thuringiensis str. Al	YP_895281.1	2 E-17	286/334
Bacteria	Firmicutes	Bacillus cereus BGSC 6E1	ZP_04312177.1	2 E-17	286/334
Bacteria	Firmicutes	Bacillus cereus 03BB102;	YP_002750122.1	3 E-17	286/334
Bacteria	Actinobacteria	Clavibacter michiganensis subsp. sepedonicus	YP_001710156.1	4 E-17	273/334

AFUA_6G10100

Eukaryota	Fungi	Aspergillus fumigatus Af293	XP_750899.1	0.0	422/422
Eukaryota	Fungi	Neosartorya fischeri NRRL 181	XP_001258137.1	0.0	403/422
Eukaryota	Fungi	Aspergillus clavatus NRRL 1	XP_001267950.1	0.0	404/422

Eukaryota	Fungi	Aspergillus flavus NRRL3357	XP_002380810.1	1 E-158	403/422
Eukaryota	Fungi	Aspergillus oryzae RIB40	XP_001823697.1	1 E-158	403/422
Eukaryota	Fungi	Talaromyces stipitatus ATCC 10500	XP_002341321.1	1 E-134	404/422
Eukaryota	Fungi	Penicillium chrysogenum Wisconsin 54-1255	XP_002560011.1	1 E-127	404/422
Eukaryota	Fungi	Penicillium chrysogenum Wisconsin 54-1255	XP_002563514.1	1 E-123	404/422
Eukaryota	Fungi	Ajellomyces capsulatus G186AR	EEH03614.1	1 E-122	405/422
Eukaryota	Fungi	Aspergillus clavatus NRRL 1	XP_001273745.1	1 E-121	404/422
Eukaryota	Fungi	Nectria haematococca mpVI 77-13-4	EEU45684.1	1 E-121	417/422
Eukaryota	Fungi	Pyrenophora tritici-repentis Pt-1C-BFP	XP_001933558.1	1 E-121	401/422
Eukaryota	Fungi	Gibberella zeae PH-1	XP_380371.1	1 E-120	412/422
Eukaryota	Fungi	Neurospora crassa OR74A	XP_957769.1	1 E-119	452/422
Eukaryota	Fungi	Ajellomyces dermatitidis SLH14081	XP_002623325.1	1 E-118	405/422
Eukaryota	Fungi	Ajellomyces dermatitidis ER-3	EEQ90738.1	1 E-117	405/422
Eukaryota	Fungi	Aspergillus oryzae RIB40	XP_001826689.1	1 E-115	404/422
Eukaryota	Fungi	Verticillium albo-atrum VaMs.102	EEY20622.1	1 E-115	415/422
Eukaryota	Fungi	Aspergillus flavus NRRL3357	XP_002385394.1	1 E-115	404/422
Eukaryota	Fungi	Penicillium chrysogenum Wisconsin 54-1255	XP_002564959.1	1 E-115	406/422
Eukaryota	Fungi	Pyrenophora tritici-repentis Pt-1C-BFP	XP_001941634.1	1 E-114	402/422
Eukaryota	Fungi	Phaeosphaeria nodorum SN15	XP_001803866.1	1 E-111	380/422
Eukaryota	Fungi	Aspergillus niger CBS 513.88	XP_001399597.1	1 E-106	398/422
Eukaryota	Fungi	Aspergillus fumigatus Af293	XP_748475.1	1 E-106	408/422
Eukaryota	Fungi	Aspergillus fumigatus A1163	EDP53505.1	1 E-105	408/422
Eukaryota	Fungi	Ajellomyces capsulatus NAm1	XP_001544219.1	1 E-103	348/422
Eukaryota	Fungi	Neosartorya fischeri NRRL 181	XP_001258799.1	1 E-100	350/422
Eukaryota	Fungi	Chaetomium globosum CBS 148.51	XP_001228642.1	1 E-100	404/422
Eukaryota	Fungi	Uncinocarpus reesii 1704	XP_002584294.1	2 E-94	361/422
Eukaryota	Fungi	Chaetomium globosum CBS 148.51	XP_001227447.1	3 E-93	376/422
Eukaryota	Fungi	Gibberella zeae PH-1	XP_389385.1	2 E-90	395/422
Eukaryota	Fungi	Aspergillus oryzae RIB40	XP_001821967.1	3 E-90	412/422
Eukaryota	Fungi	Penicillium chrysogenum Wisconsin 54-1255	XP_002560285.1	1 E-87	414/422
Eukaryota	Fungi	Aspergillus clavatus NRRL 1	XP_001275411.1	1 E-86	417/422
Eukaryota	Fungi	Aspergillus fumigatus A1163	EDP50801.1	7 E-83	413/422
Eukaryota	Fungi	Aspergillus fumigatus Af293	XP_751406.2	2 E-82	413/422
Eukaryota	Fungi	Aspergillus niger CBS 513.88	XP_001399455.1	5 E-78	410/422
Eukaryota	Fungi	Gibberella zeae PH-1	XP_383641.1	5 E-77	395/422
Eukaryota	Fungi	Gibberella zeae PH-1	XP_384391.1	2 E-72	394/422
Eukaryota	Fungi	Nectria haematococca mpVI 77-13-4	EEU39352.1	1 E-70	394/422
Eukaryota	Fungi	Verticillium albo-atrum VaMs.102	EEY20743.1	1 E-69	395/422
Eukaryota	Fungi	Neosartorya fischeri NRRL 181	XP_001266616.1	1 E-65	358/422
Eukaryota	Fungi	Phaeosphaeria nodorum SN15	XP_001803193.1	1 E-62	387/422
Eukaryota	Fungi	Nectria haematococca mpVI 77-13-4	EEU44952.1	3 E-62	389/422
Eukaryota	Fungi	Nectria haematococca mpVI 77-13-4	EEU37016.1	4 E-58	394/422
Eukaryota	Fungi	Botryotinia fuckeliana B05.10	XP_001560647.1	4 E-48	406/422
Eukaryota	Fungi	Phaeosphaeria nodorum SN15	XP_001796212.1	5 E-47	418/422
Eukaryota	Fungi	Botryotinia fuckeliana B05.10	XP_001557586.1	2 E-46	349/422
Eukaryota	Fungi	Cryptococcus neoformans var. neoformans	XP_571619.1	2 E-44	397/422
Eukaryota	Fungi	Cryptococcus neoformans var. neoformans	XP_774845.1	3 E-44	397/422
Eukaryota	Fungi	Cryptococcus neoformans var. neoformans	XP_571446.1	5 E-43	399/422
Eukaryota	Fungi	Cryptococcus neoformans var. neoformans	XP_571631.1	6 E-43	399/422
Eukaryota	Fungi	Talaromyces stipitatus ATCC 10500	XP_002478514.1	4 E-42	394/422
Eukaryota	Fungi	Cryptococcus neoformans var. neoformans	XP_774847.1	1 E-41	396/422
Eukaryota	Fungi	Cryptococcus neoformans var. neoformans	XP_571444.1	2 E-41	396/422
Eukaryota	Fungi	Gibberella zeae PH-1	XP_391749.1	8 E-40	364/422
Eukaryota	Fungi	Aspergillus nidulans FGSC A4	XP_660195.1	1 E-36	371/422
Eukaryota	Fungi	Cryptococcus neoformans var. neoformans	XP_774033.1	9 E-36	399/422
Eukaryota	Fungi	Pyrenophora tritici-repentis Pt-1C-BFP	XP_001935736.1	4 E-33	344/422
Eukaryota	Fungi	Ustilago maydis 521	XP_761589.1	9 E-33	405/422
Eukaryota	Fungi	Ajellomyces dermatitidis SLH14081	XP_002627272.1	3 E-29	349/422

Eukaryota	Fungi	Pichia guilliermondii ATCC 6260	XP_001487655.1	4 E-29	357/422
Eukaryota	Fungi	Debaryomyces hansenii	CAG90302.2	6 E-29	359/422
Eukaryota	Fungi	Cryptococcus neoformans var. neoformans	XP_572815.1	7 E-29	358/422
Eukaryota	Fungi	Debaryomyces hansenii CBS767	XP_461841.1	1 E-28	359/422
Eukaryota	Fungi	Penicillium chrysogenum Wisconsin 54-1255	XP_002563959.1	2 E-28	367/422
Eukaryota	Fungi	Talaromyces stipitatus ATCC 10500	XP_002487895.1	4 E-28	391/422
Eukaryota	Fungi	Pichia guilliermondii ATCC 6260	EDK36934.2	7 E-28	357/422
Eukaryota	Fungi	Neosartorya fischeri NRRL 181	XP_001260727.1	5 E-27	358/422
Eukaryota	Fungi	Aspergillus clavatus NRRL 1	XP_001275551.1	6 E-27	360/422
Eukaryota	Fungi	Penicillium marneffei ATCC 18224	XP_002153522.1	1 E-26	376/422
Eukaryota	Fungi	Microsporum canis CBS 113480	EEQ30417.1	3 E-24	339/422
Eukaryota	Fungi	Talaromyces stipitatus ATCC 10500	XP_002484943.1	1 E-23	353/422
Eukaryota	Fungi	Nectria haematococca mpVI 77-13-4	EEU34040.1	4 E-23	351/422
Eukaryota	Fungi	Aspergillus niger CBS 513.88	XP_001390756.1	4 E-23	367/422
Eukaryota	Fungi	Neosartorya fischeri NRRL 181	XP_001258636.1	6 E-23	365/422
Eukaryota	Fungi	Aspergillus niger CBS 513.88	XP_001394030.1	1 E-22	374/422
Eukaryota	Fungi	Coccidioides immitis RS;	XP_001242811.1	2 E-22	382/422
Eukaryota	Fungi	Neosartorya fischeri NRRL 181	XP_001259308.1	4 E-22	360/422
Eukaryota	Fungi	Aspergillus oryzae RIB40	XP_001820577.1	5 E-22	352/422
Eukaryota	Fungi	Nectria haematococca mpVI 77-13-4	EEU34079.1	5 E-22	367/422
Eukaryota	Fungi	Aspergillus flavus NRRL3357	XP_002373816.1	5 E-22	352/422
Eukaryota	Fungi	Laccaria bicolor S238N-H82	XP_001887593.1	6 E-22	357/422
Eukaryota	Fungi	Penicillium marneffei ATCC 18224	XP_002153696.1	7 E-22	353/422
Eukaryota	Fungi	Aspergillus clavatus NRRL 1	XP_001276477.1	1 E-21	380/422
Eukaryota	Fungi	Coccidioides posadasii C735 delta	EER29800.1	1 E-21	349/422
Eukaryota	Fungi	Ustilago maydis 521	XP_756624.1	1 E-21	381/422
Eukaryota	Fungi	Postia placenta Mad-698-R	XP_002476221.1	2 E-21	372/422
Eukaryota	Fungi	Ajellomyces dermatitidis SLH14081	XP_002624207.1	6 E-21	354/422
Eukaryota	Fungi	Aspergillus terreus NIH2624	XP_001211658.1	7 E-21	380/422
Eukaryota	Fungi	Aspergillus flavus NRRL3357	XP_002381495.1	9 E-21	358/422
Eukaryota	Fungi	Aspergillus terreus NIH2624	XP_001217046.1	9 E-21	390/422
Eukaryota	Fungi	Aspergillus terreus NIH2624	XP_001217806.1	1 E-20	354/422
Eukaryota	Fungi	Verticillium albo-atrum VaMs.102	EEY23800.1	1 E-20	385/422
Eukaryota	Fungi	Ustilago maydis 521	XP_762089.1	1 E-20	393/422
Eukaryota	Fungi	Podospora anserina DSM 980	XP_001912856.1	1 E-20	338/422
Eukaryota	Fungi	Chaetomium globosum CBS 148.51	XP_001225690.1	2 E-20	376/422
Eukaryota	Fungi	Aspergillus terreus NIH2624	XP_001216245.1	3 E-20	355/422
Eukaryota	Fungi	Pyrenophora tritici-repentis Pt-1C-BFP	XP_001937517.1	4 E-20	378/422
Eukaryota	Fungi	Talaromyces stipitatus ATCC 10500	XP_002341678.1	5 E-20	362/422
Eukaryota	Fungi	Aspergillus flavus NRRL3357	XP_002375427.1	7 E-20	361/422
Eukaryota	Fungi	Aspergillus nidulans FGSC A4	XP_664033.1	3 E-19	380/422
Eukaryota	Fungi	Podospora anserina DSM 980	XP_001909355.1	4 E-19	366/422
Eukaryota	Fungi	Verticillium albo-atrum VaMs.102	EEY17956.1	6 E-19	368/422
Eukaryota	Fungi	Cryptococcus neoformans var. neoformans	XP_567535.1	6 E-19	358/422
Eukaryota	Fungi	Aspergillus nidulans FGSC A4	XP_682058.1	8 E-19	396/422
Eukaryota	Fungi	Magnaporthe grisea 70-15	XP_367898.2	1 E-18	383/422
Eukaryota	Fungi	Ajellomyces capsulatus G186AR	EEH11688.1	2 E-18	354/422
Eukaryota	Fungi	Microsporum canis CBS 113480	EEQ31490.1	2 E-18	354/422
Eukaryota	Fungi	Nectria haematococca mpVI 77-13-4	EEU43129.1	2 E-18	377/422
Eukaryota	Fungi	Aspergillus flavus NRRL3357	XP_002381734.1	2 E-18	357/422
Eukaryota	Fungi	Ajellomyces capsulatus H143	EER39524.1	2 E-18	354/422
Eukaryota	Fungi	Aspergillus fumigatus Af293	XP_747462.1	2 E-18	343/422
Eukaryota	Fungi	Penicillium marneffei ATCC 18224	XP_002152756.1	3 E-18	366/422
Eukaryota	Fungi	Penicillium chrysogenum Wisconsin 54-1255	XP_002561311.1	3 E-18	374/422
Eukaryota	Fungi	Aspergillus niger CBS 513.88	XP_001393867.1	3 E-18	366/422
Eukaryota	Fungi	Aspergillus oryzae RIB40	XP_001825057.1	3 E-18	357/422
Eukaryota	Fungi	Nectria haematococca mpVI 77-13-4	EEU41498.1	4 E-18	389/422
Eukaryota	Fungi	Aspergillus fumigatus Af293	XP_748643.1	5 E-18	379/422

Eukaryota	Fungi	Aspergillus flavus NRRL3357	XP_002379213.1	5 E-18	361/422
Eukaryota	Fungi	Malassezia globosa CBS 7966	XP_001729091.1	6 E-18	355/422
Eukaryota	Fungi	Penicillium chrysogenum Wisconsin 54-1255	XP_002567235.1	8 E-18	358/422
Eukaryota	Fungi	Neosartorya fischeri NRRL 181	XP_001263175.1	1 E-17	397/422
Eukaryota	Fungi	Paracoccidioides brasiliensis Pb01;	EEH35743.1	2 E-17	354/422
Eukaryota	Fungi	Aspergillus flavus NRRL3357	XP_002372458.1	2 E-17	377/422
Eukaryota	Fungi	Magnaporthe grisea 70-15	XP_365300.1	2 E-17	346/422
Eukaryota	Fungi	Ajellomyces capsulatus NAm1	XP_001541845.1	2 E-17	354/422
Eukaryota	Fungi	Neosartorya fischeri NRRL 181	XP_001259072.1	2 E-17	379/422
Eukaryota	Fungi	Gibberella zeae PH-1	XP_391541.1	2 E-17	355/422
Eukaryota	Fungi	Aspergillus clavatus NRRL 1	XP_001273582.1	2 E-17	358/422
Eukaryota	Fungi	Aspergillus niger CBS 513.88	XP_001402113.1	3 E-17	367/422
Eukaryota	Fungi	Ajellomyces capsulatus G186AR	EEH07851.1	3 E-17	380/422
Eukaryota	Fungi	Aspergillus niger CBS 513.88	XP_001397979.1	3 E-17	356/422
Eukaryota	Fungi	Coccidioides posadasii C735 delta	EER23151.1	3 E-17	380/422
Eukaryota	Fungi	Coccidioides immitis RS;	XP_001248279.1	4 E-17	380/422
Eukaryota	Fungi	Aspergillus terreus NIH2624	XP_001210759.1	4 E-17	395/422
Eukaryota	Fungi	Talaromyces stipitatus ATCC 10500	XP_002486889.1	4 E-17	350/422
Eukaryota	Fungi	Aspergillus clavatus NRRL 1	XP_001276008.1	4 E-17	358/422
Eukaryota	Fungi	Talaromyces stipitatus ATCC 10500	XP_002478152.1	5 E-17	339/422
Eukaryota	Fungi	Aspergillus oryzae RIB40	XP_001822166.1	6 E-17	361/422
Eukaryota	Fungi	Aspergillus oryzae RIB40	XP_001824382.1	6 E-17	346/422
Eukaryota	Fungi	Pichia stipitis CBS 6054	XP_001385138.2	8 E-17	360/422
Eukaryota	Fungi	Aspergillus fumigatus A1163	EDP55239.1	9 E-17	357/422
Eukaryota	Fungi	Aspergillus fumigatus Af293	XP_754331.2	1 E-16	397/422
Eukaryota	Fungi	Magnaporthe grisea 70-15	XP_367978.2	1 E-16	370/422
Eukaryota	Fungi	Sclerotinia sclerotiorum 1980 UF-70	XP_001589189.1	1 E-16	352/422
Eukaryota	Fungi	Aspergillus fumigatus Af293	XP_748652.1	1 E-16	341/422
Eukaryota	Fungi	Aspergillus flavus NRRL3357	XP_002377394.1	1 E-16	368/422
Eukaryota	Fungi	Verticillium albo-atrum VaMs.102	EEY17950.1	2 E-16	356/422
Eukaryota	Fungi	Penicillium marneffei ATCC 18224	XP_002153620.1	2 E-16	356/422
Eukaryota	Fungi	Phaeosphaeria nodorum SN15	XP_001791751.1	2 E-16	351/422
Eukaryota	Fungi	Neosartorya fischeri NRRL 181	XP_001258699.1	2 E-16	355/422
Eukaryota	Fungi	Aspergillus fumigatus Af293	XP_756071.1	2 E-16	357/422
Eukaryota	Fungi	Microsporum canis CBS 113480	EEQ35853.1	3 E-16	401/422
Eukaryota	Fungi	Ajellomyces capsulatus H143	EER42807.1	3 E-16	381/422
Eukaryota	Fungi	Pyrenophora tritici-repentis Pt-1C-BFP	XP_001939781.1	3 E-16	350/422
Eukaryota	Fungi	Botryotinia fuckeliana B05.10	XP_001556136.1	3 E-16	355/422
Eukaryota	Fungi	Aspergillus nidulans FGSC A4	XP_681682.1	4 E-16	355/422
Eukaryota	Fungi	Talaromyces stipitatus ATCC 10500	XP_002484919.1	4 E-16	339/422
Eukaryota	Fungi	Aspergillus niger CBS 513.88	XP_001394787.1	4 E-16	363/422
Eukaryota	Fungi	Aspergillus terreus NIH2624	XP_001217295.1	6 E-16	370/422
Eukaryota	Fungi	Magnaporthe grisea 70-15	XP_360868.1	6 E-16	347/422
Eukaryota	Fungi	Aspergillus oryzae RIB40	XP_001818031.1	6 E-16	363/422
Eukaryota	Fungi	Aspergillus oryzae RIB40	XP_001825796.1	6 E-16	368/422
Eukaryota	Fungi	Chaetomium globosum CBS 148.51	XP_001220562.1	7 E-16	363/422
Eukaryota	Fungi	Microsporum canis CBS 113480	EEQ35443.1	7 E-16	380/422
Eukaryota	Fungi	Penicillium marneffei ATCC 18224	XP_002151297.1	8 E-16	379/422

AFUA_6G10120

Eukaryota	Fungi	Aspergillus fumigatus Af293	XP_750901.1	0.0	347/347
Eukaryota	Fungi	Neosartorya fischeri NRRL 181	XP_001258138.1	0.0	348/347
Eukaryota	Fungi	Aspergillus clavatus NRRL 1	XP_001267951.1	1 E-165	348/347
Eukaryota	Fungi	Aspergillus niger CBS 513.88	XP_001395692.1	1 E-159	347/347
Eukaryota	Fungi	Aspergillus oryzae RIB40	XP_001817596.1	1 E-150	348/347
Eukaryota	Fungi	Aspergillus nidulans FGSC A4	CBF78253.1	1 E-146	348/347
Eukaryota	Fungi	Aspergillus terreus NIH2624	XP_001214445.1	1 E-145	348/347

Eukaryota	Fungi	Aspergillus oryzae RIB40	XP_001821126.1	1 E-143	347/347
Eukaryota	Fungi	Penicillium chrysogenum Wisconsin 54-1255	XP_002563112.1	1 E-142	344/347
Eukaryota	Fungi	Aspergillus flavus NRRL3357	XP_002376876.1	1 E-141	347/347
Eukaryota	Fungi	Aspergillus nidulans FGSC A4	XP_681920.1	1 E-139	335/347
Eukaryota	Fungi	Aspergillus niger CBS 513.88	XP_001392499.1	1 E-133	348/347
Eukaryota	Fungi	Aspergillus flavus NRRL3357	XP_002379017.1	1 E-129	347/347
Eukaryota	Fungi	Aspergillus terreus NIH2624	XP_001209166.1	1 E-129	332/347
Eukaryota	Fungi	Aspergillus oryzae RIB40	XP_001823606.1	1 E-129	347/347
Eukaryota	Fungi	Aspergillus niger CBS 513.88	XP_001401814.1	1 E-118	342/347
Eukaryota	Fungi	Talaromyces stipitatus ATCC 10500	XP_002485889.1	1 E-116	346/347
Eukaryota	Fungi	Penicillium marneffei ATCC 18224	XP_002152303.1	1 E-113	347/347
Eukaryota	Fungi	Botryotinia fuckeliana B05.10	XP_001561009.1	1 E-110	350/347
Eukaryota	Fungi	Pichia pastoris	CAY67035.1	1 E-109	345/347
Eukaryota	Fungi	Sclerotinia sclerotiorum 1980 UF-70	XP_001594867.1	1 E-105	350/347
Eukaryota	Fungi	Aspergillus niger CBS 513.88	XP_001390368.1	1 E-105	342/347
Eukaryota	Fungi	Aspergillus niger CBS 513.88	XP_001400820.1	1 E-103	347/347
Eukaryota	Fungi	Nectria haematococca mpVI 77-13-4	EEU33591.1	1 E-101	349/347
Eukaryota	Fungi	Penicillium chrysogenum Wisconsin 54-1255	XP_002560735.1	6 E-99	348/347
Eukaryota	Fungi	Aspergillus flavus NRRL3357	XP_002380434.1	7 E-95	345/347
Eukaryota	Fungi	Chaetomium globosum CBS 148.51	XP_001224248.1	2 E-94	358/347
Eukaryota	Fungi	Aspergillus oryzae RIB40	XP_001825265.1	3 E-94	345/347
Eukaryota	Fungi	Aspergillus terreus NIH2624	XP_001217360.1	3 E-92	344/347
Eukaryota	Fungi	Paracoccidioides brasiliensis Pb01;	EEH35063.1	4 E-90	342/347
Eukaryota	Fungi	Microsporum canis CBS 113480	EEQ32050.1	1 E-89	329/347
Eukaryota	Fungi	Aspergillus niger CBS 513.88	XP_001394422.1	1 E-89	352/347
Eukaryota	Fungi	Verticillium albo-atrum VaMs.102	EEY19938.1	2 E-88	353/347
Eukaryota	Fungi	Talaromyces stipitatus ATCC 10500	XP_002487096.1	2 E-87	353/347
Eukaryota	Fungi	Phaeosphaeria nodorum SN15	XP_001802688.1	6 E-87	342/347
Eukaryota	Fungi	Penicillium marneffei ATCC 18224	XP_002145441.1	8 E-86	349/347
Eukaryota	Fungi	Pyrenophora tritici-repentis Pt-1C-BFP	XP_001936930.1	2 E-85	341/347
Eukaryota	Fungi	Ajellomyces capsulatus H143	EER36868.1	2 E-84	336/347
Eukaryota	Fungi	Aspergillus niger CBS 513.88	XP_001395332.1	2 E-83	349/347
Eukaryota	Fungi	Ajellomyces capsulatus G186AR	EEH06393.1	2 E-83	336/347
Eukaryota	Fungi	Aspergillus clavatus NRRL 1	XP_001271460.1	1 E-82	349/347
Eukaryota	Fungi	Magnaporthe grisea 70-15	XP_359645.1	2 E-81	357/347
Eukaryota	Fungi	Neosartorya fischeri NRRL 181	XP_001261771.1	5 E-80	352/347
Eukaryota	Fungi	Podospora anserina DSM 980	XP_001903443.1	2 E-77	310/347
Eukaryota	Fungi	Aspergillus fumigatus A1163	EDP49068.1	3 E-77	369/347
Eukaryota	Fungi	Aspergillus fumigatus Af293	XP_747063.1	1 E-76	369/347
Eukaryota	Fungi	Talaromyces stipitatus ATCC 10500	XP_002485880.1	3 E-75	351/347
Eukaryota	Fungi	Sclerotinia sclerotiorum 1980 UF-70	XP_001596372.1	1 E-73	285/347
Eukaryota	Fungi	Nectria haematococca mpVI 77-13-4	EEU35549.1	3 E-73	281/347
Eukaryota	Fungi	Aspergillus flavus NRRL3357	XP_002380251.1	8 E-73	347/347
Eukaryota	Fungi	Talaromyces stipitatus ATCC 10500	XP_002486605.1	3 E-72	347/347
Eukaryota	Fungi	Aspergillus clavatus NRRL 1	XP_001268503.1	3 E-69	338/347
Eukaryota	Fungi	Phaeosphaeria nodorum SN15	XP_001799427.1	3 E-68	286/347
Eukaryota	Fungi	Aspergillus niger CBS 513.88	XP_001392491.1	9 E-68	357/347
Eukaryota	Fungi	Aspergillus clavatus NRRL 1	XP_001270375.1	1 E-67	352/347
Eukaryota	Fungi	Penicillium marneffei ATCC 18224	XP_002149825.1	2 E-67	331/347
Eukaryota	Fungi	Neosartorya fischeri NRRL 181	XP_001258704.1	4 E-67	347/347
Eukaryota	Fungi	Magnaporthe grisea 70-15	XP_362669.1	1 E-65	328/347
Eukaryota	Fungi	Penicillium chrysogenum Wisconsin 54-1255	XP_002561690.1	2 E-65	354/347
Eukaryota	Fungi	Coccidioides immitis RS;	XP_001242735.1	6 E-64	353/347
Eukaryota	Fungi	Coccidioides posadasii C735 delta	EER27754.1	8 E-64	353/347
Eukaryota	Fungi	Aspergillus clavatus NRRL 1	XP_001270547.1	2 E-63	333/347
Eukaryota	Fungi	Botryotinia fuckeliana B05.10	XP_001547208.1	2 E-63	298/347
Eukaryota	Fungi	Aspergillus oryzae RIB40	XP_001818826.1	4 E-63	326/347
Eukaryota	Fungi	Aspergillus clavatus NRRL 1	XP_001270450.1	4 E-62	389/347

Eukaryota	Fungi	Aspergillus niger CBS 513.88	XP_001394623.1	7 E-61	354/347
Eukaryota	Fungi	Botryotinia fuckeliana	CAP58785.1	3 E-59	372/347
Eukaryota	Fungi	Microsporum canis CBS 113480	EEQ30015.1	5 E-59	326/347
Eukaryota	Fungi	Aspergillus clavatus NRRL 1	XP_001269054.1	4 E-58	332/347
Eukaryota	Fungi	Botryotinia fuckeliana B05.10	XP_001560668.1	4 E-58	356/347
Eukaryota	Fungi	Aspergillus niger CBS 513.88	XP_001399963.1	4 E-58	341/347
Eukaryota	Fungi	Chaetomium globosum CBS 148.51	XP_001221378.1	5 E-58	332/347
Eukaryota	Fungi	Aspergillus nidulans FGSC A4	XP_681678.1	1 E-57	326/347
Eukaryota	Fungi	Ustilago maydis 521	XP_758722.1	8 E-57	358/347
Eukaryota	Fungi	Verticillium albo-atrum VaMs.102	EEY15225.1	9 E-57	349/347
Eukaryota	Fungi	Paracoccidioides brasiliensis Pb18;	EEH43188.1	2 E-56	310/347
Eukaryota	Fungi	Postia placenta Mad-698-R	XP_002473263.1	7 E-56	343/347
Eukaryota	Fungi	Penicillium chrysogenum Wisconsin 54-1255	XP_002560004.1	1 E-55	336/347
Eukaryota	Fungi	Chaetomium globosum CBS 148.51	XP_001227950.1	2 E-55	328/347
Eukaryota	Fungi	Uncinocarpus reesii 1704	XP_002583531.1	3 E-55	385/347
Eukaryota	Fungi	Uncinocarpus reesii 1704	XP_002544296.1	6 E-55	317/347
Eukaryota	Fungi	Magnaporthe grisea 70-15	XP_362658.2	7 E-55	332/347
Eukaryota	Fungi	Aspergillus terreus	AAD34554.1	9 E-55	328/347
Eukaryota	Fungi	Aspergillus terreus NIH2624	XP_001209265.1	1 E-54	328/347
Eukaryota	Fungi	Penicillium expansum	CAO91860.1	3 E-54	347/347
Eukaryota	Fungi	Microsporum canis CBS 113480	EEQ27574.1	4 E-54	330/347
Eukaryota	Fungi	Aspergillus niger CBS 513.88	XP_001396429.1	1 E-53	351/347
Eukaryota	Fungi	Talaromyces stipitatus ATCC 10500	XP_002484500.1	2 E-53	369/347
Eukaryota	Fungi	Microsporum canis CBS 113480	EEQ35633.1	7 E-53	360/347
Eukaryota	Fungi	Monascus pilosus	ABA02243.1	1 E-52	328/347
Eukaryota	Fungi	Nectria haematococca mpVI 77-13-4	EEU33592.1	2 E-52	340/347
Eukaryota	Fungi	Aspergillus clavatus NRRL 1	XP_001274944.1	3 E-52	342/347
Eukaryota	Fungi	Podospora anserina DSM 980	XP_001905186.1	2 E-51	352/347
Eukaryota	Fungi	Penicillium citrinum	BAC20562.1	4 E-51	324/347
Eukaryota	Fungi	Talaromyces stipitatus ATCC 10500	XP_002478244.1	3 E-50	319/347
Eukaryota	Fungi	Chaetomium globosum CBS 148.51	XP_001220461.1	6 E-50	336/347
Eukaryota	Fungi	Botryotinia fuckeliana B05.10	XP_001545338.1	1 E-49	332/347
Eukaryota	Fungi	Cryptococcus neoformans var. neoformans	XP_569052.1	2 E-49	333/347
Eukaryota	Fungi	Nectria haematococca mpVI 77-13-4	EEU35059.1	3 E-49	326/347
Eukaryota	Fungi	Cryptococcus neoformans var. neoformans	XP_567047.1	3 E-49	351/347
Eukaryota	Fungi	Cryptococcus neoformans var. neoformans	XP_567470.1	7 E-49	333/347
Eukaryota	Fungi	Coprinopsis cinerea okayama7#130	XP_001834735.1	5 E-48	345/347
Eukaryota	Fungi	Aspergillus fumigatus Af293	XP_753140.1	9 E-48	336/347
Eukaryota	Fungi	Verticillium albo-atrum VaMs.102	EEY20226.1	2 E-47	321/347
Eukaryota	Fungi	Magnaporthe grisea 70-15	XP_360881.1	3 E-47	324/347
Eukaryota	Fungi	Penicillium chrysogenum Wisconsin 54-1255	XP_002556643.1	4 E-47	325/347
Eukaryota	Fungi	Neosartorya fischeri NRRL 181	XP_001257534.1	1 E-46	321/347
Eukaryota	Fungi	Pichia stipitis CBS 6054	XP_001383294.1	5 E-46	347/347
Eukaryota	Fungi	Candida dubliniensis CD36	XP_002418897.1	6 E-46	344/347
Eukaryota	Fungi	Clavispora lusitaniae ATCC 42720	XP_002618860.1	7 E-46	359/347
Eukaryota	Fungi	Talaromyces stipitatus ATCC 10500	XP_002341175.1	8 E-46	345/347
Eukaryota	Fungi	Aspergillus fumigatus A1163	EDP47644.1	1 E-45	330/347
Eukaryota	Fungi	Botryotinia fuckeliana B05.10	XP_001546009.1	2 E-45	379/347
Eukaryota	Fungi	Phaeosphaeria nodorum SN15	XP_001791001.1	2 E-45	315/347
Eukaryota	Fungi	Aspergillus fumigatus Af293	XP_747740.1	3 E-45	330/347
Eukaryota	Fungi	Cordyceps bassiana	CAL69596.1	3 E-45	375/347
Eukaryota	Fungi	Magnaporthe grisea 70-15	XP_361765.2	4 E-45	337/347
Eukaryota	Fungi	Malassezia globosa CBS 7966	XP_001728637.1	5 E-44	358/347
Eukaryota	Fungi	Candida dubliniensis CD36	XP_002420223.1	5 E-44	337/347
Eukaryota	Fungi	Nectria haematococca mpVI 77-13-4	EEU38995.1	5 E-44	312/347
Eukaryota	Fungi	Candida albicans SC5314	XP_713278.1	8 E-44	360/347
Eukaryota	Fungi	Candida albicans WO-1	EEQ47510.1	1 E-43	361/347
Eukaryota	Fungi	Aspergillus oryzae RIB40	XP_001727397.1	1 E-43	324/347

Eukaryota	Fungi	Aspergillus flavus NRRL3357	XP_002375652.1	1 E-43	324/347
Eukaryota	Fungi	Candida albicans SC5314	XP_713227.1	2 E-43	360/347
Eukaryota	Fungi	Candida albicans SC5314	XP_712257.1	3 E-43	353/347
Eukaryota	Fungi	Candida albicans WO-1	EEQ43428.1	3 E-43	358/347
Eukaryota	Fungi	Chaetomium globosum CBS 148.51	XP_001228886.1	4 E-43	368/347
Eukaryota	Fungi	Candida albicans SC5314	XP_714368.1	5 E-43	361/347
Eukaryota	Fungi	Candida albicans SC5314	XP_714408.1	9 E-43	361/347
Eukaryota	Fungi	Candida tropicalis MYA-3404	XP_002545990.1	1 E-42	354/347
Eukaryota	Fungi	Candida albicans WO-1	EEQ43426.1	3 E-42	337/347
Eukaryota	Fungi	Candida dubliniensis CD36	XP_002421198.1	3 E-42	356/347
Eukaryota	Fungi	Candida albicans WO-1	EEQ46583.1	3 E-42	352/347
Eukaryota	Fungi	Podospora anserina DSM 980	XP_001904545.1	4 E-42	345/347
Eukaryota	Fungi	Candida albicans SC5314	XP_715619.1	4 E-42	337/347
Eukaryota	Fungi	Candida albicans WO-1	EEQ44901.1	6 E-42	335/347
Eukaryota	Fungi	Sclerotinia sclerotiorum 1980 UF-70	XP_001594316.1	6 E-42	330/347
Eukaryota	Fungi	Pichia stipitis CBS 6054	XP_001383293.2	1 E-41	349/347
Eukaryota	Fungi	Candida albicans SC5314	XP_713280.1	2 E-41	337/347
Eukaryota	Fungi	Aspergillus niger CBS 513.88	XP_001398493.1	2 E-41	305/347
Eukaryota	Fungi	Candida dubliniensis CD36	XP_002421892.1	2 E-41	360/347
Eukaryota	Fungi	Gibberella zeae PH-1	XP_383589.1	3 E-41	350/347
Eukaryota	Fungi	Sclerotinia sclerotiorum 1980 UF-70	XP_001589515.1	4 E-41	304/347
Eukaryota	Fungi	Vanderwaltozyma polyspora DSM 70294	XP_001644106.1	1 E-40	362/347
Eukaryota	Fungi	Candida dubliniensis CD36	XP_002421890.1	1 E-40	349/347
Eukaryota	Fungi	Saccharomyces cerevisiae	NP_014265.1	2 E-40	365/347
Eukaryota	Fungi	Saccharomyces cerevisiae JAY291	EEU08736.1	3 E-40	365/347
Eukaryota	Fungi	Kluyveromyces lactis NRRL Y-1140	XP_456170.1	3 E-40	363/347
Eukaryota	Fungi	Malassezia globosa CBS 7966	XP_001728638.1	5 E-40	354/347
Eukaryota	Fungi	Saccharomyces cerevisiae RM11-1a	EDV08761.1	6 E-40	365/347
Eukaryota	Fungi	Aspergillus oryzae RIB40	XP_001822879.1	1 E-39	340/347
Eukaryota	Fungi	Pichia pastoris GS115	XP_002494199.1	1 E-39	371/347
Eukaryota	Fungi	Saccharomyces cerevisiae	NP_010026.1	2 E-39	347/347
Eukaryota	Fungi	Saccharomyces cerevisiae AWRI1631	EDZ73463.1	2 E-39	347/347
Eukaryota	Fungi	Nectria haematococca mpVI 77-13-4	EEU45296.1	2 E-39	323/347
Eukaryota	Fungi	Saccharomyces cerevisiae RM11-1a	EDV09786.1	3 E-39	347/347
Eukaryota	Fungi	Saccharomyces cerevisiae YJM789	EDN62223.1	3 E-39	347/347
Eukaryota	Fungi	Vanderwaltozyma polyspora DSM 70294	XP_001642565.1	4 E-39	363/347
Eukaryota	Fungi	Gibberella zeae PH-1	XP_387425.1	1 E-38	334/347
Eukaryota	Fungi	Pichia guilliermondii ATCC 6260	EDK41027.2	1 E-38	403/347
Eukaryota	Fungi	Aspergillus terreus NIH2624	XP_001212786.1	1 E-38	312/347
Eukaryota	Fungi	Saccharomyces cerevisiae YJM789	EDN59698.1	1 E-38	365/347
Eukaryota	Fungi	Debaryomyces hansenii CBS767	XP_457007.1	3 E-38	363/347
Eukaryota	Fungi	Pichia guilliermondii ATCC 6260	XP_001483170.1	3 E-38	403/347
Eukaryota	Fungi	Aspergillus flavus NRRL3357	XP_002382270.1	4 E-38	323/347
Eukaryota	Fungi	Debaryomyces hansenii CBS767	XP_457006.1	5 E-38	367/347
Eukaryota	Fungi	Nectria haematococca mpVI 77-13-4	EEU43354.1	5 E-38	323/347
Eukaryota	Fungi	Saccharomyces cerevisiae YJM789	EDN59358.1	6 E-38	365/347
Eukaryota	Fungi	Candida glabrata CBS 138	XP_448640.1	7 E-38	365/347
Eukaryota	Fungi	Pichia guilliermondii ATCC 6260	EDK39569.2	7 E-38	359/347
Eukaryota	Fungi	Debaryomyces hansenii	CAG84991.2	1 E-37	366/347
Eukaryota	Fungi	Talaromyces stipitatus ATCC 10500	XP_002340067.1	1 E-37	317/347
Eukaryota	Fungi	Pichia guilliermondii ATCC 6260	XP_001484286.1	1 E-37	359/347
Eukaryota	Fungi	Saccharomyces cerevisiae	NP_013565.1	1 E-37	365/347
Eukaryota	Fungi	Magnaporthe grisea 70-15	XP_362685.2	2 E-37	283/347
Eukaryota	Fungi	Penicillium marneffei ATCC 18224	XP_002149740.1	2 E-37	317/347
Eukaryota	Fungi	Aspergillus clavatus NRRL 1	XP_001276295.1	2 E-37	342/347
Eukaryota	Fungi	Talaromyces stipitatus ATCC 10500	XP_002484737.1	2 E-37	341/347
Eukaryota	Fungi	Pichia guilliermondii ATCC 6260	EDK36332.2	7 E-37	359/347
Eukaryota	Fungi	Penicillium marneffei ATCC 18224	XP_002149765.1	8 E-37	337/347

Eukaryota	Fungi	Talaromyces stipitatus ATCC 10500	XP_002340043.1	1 E-36	337/347
Eukaryota	Fungi	Aspergillus nidulans FGSC A4	CBF82230.1	1 E-36	323/347
Eukaryota	Fungi	Talaromyces stipitatus ATCC 10500	XP_002483873.1	1 E-36	343/347
Eukaryota	Fungi	Pichia guilliermondii ATCC 6260	XP_001487053.1	1 E-36	360/347
Eukaryota	Fungi	Debaryomyces hansenii	CAG84940.2	2 E-36	355/347
Eukaryota	Fungi	Debaryomyces hansenii	CAG84311.2	2 E-36	357/347
Eukaryota	Fungi	Podospora anserina DSM 980	XP_001904948.1	6 E-36	339/347
Eukaryota	Fungi	Pichia pastoris GS115	XP_002491528.1	6 E-36	370/347
Eukaryota	Fungi	Debaryomyces hansenii CBS767	XP_456366.1	6 E-36	357/347
Eukaryota	Fungi	Debaryomyces hansenii CBS767	XP_456961.1	6 E-36	355/347
Eukaryota	Fungi	Penicillium marneffei ATCC 18224	XP_002153039.1	6 E-36	358/347
Eukaryota	Fungi	Pichia guilliermondii ATCC 6260	EDK38229.2	9 E-36	356/347
Eukaryota	Fungi	Lachancea thermotolerans CBS 6340	XP_002552031.1	1 E-35	363/347
Eukaryota	Fungi	Candida glabrata CBS 138	XP_448339.1	1 E-35	364/347
Eukaryota	Fungi	Schizosaccharomyces pombe	NP_593982.1	1 E-35	346/347
Eukaryota	Fungi	Aspergillus niger CBS 513.88	XP_001398003.1	3 E-35	319/347
Eukaryota	Fungi	Yarrowia lipolytica CLIB122	XP_502298.1	3 E-35	307/347
Eukaryota	Fungi	Pichia guilliermondii ATCC 6260	XP_001484598.1	8 E-35	356/347
Eukaryota	Fungi	Aspergillus oryzae RIB40	XP_001824200.1	9 E-35	320/347
Eukaryota	Fungi	Debaryomyces hansenii	CAG84990.2	9 E-35	345/347
Eukaryota	Fungi	Pyrenophora tritici-repentis Pt-1C-BFP	XP_001934282.1	1 E-34	340/347
Eukaryota	Fungi	Debaryomyces hansenii CBS767	XP_457005.1	1 E-34	345/347
Eukaryota	Fungi	Lodderomyces elongisporus NRRL YB-4239	XP_001527734.1	2 E-34	340/347
Eukaryota	Fungi	Laccaria bicolor S238N-H82	XP_001889320.1	7 E-34	338/347
Eukaryota	Fungi	Phaeosphaeria nodorum SN15	XP_001798191.1	7 E-34	339/347
Eukaryota	Fungi	Laccaria bicolor S238N-H82	XP_001883322.1	3 E-33	345/347
Eukaryota	Fungi	Ashbya gossypii ATCC 10895	NP_985613.1	3 E-33	344/347
Eukaryota	Fungi	Aspergillus clavatus NRRL 1	XP_001273676.1	5 E-33	322/347
Eukaryota	Fungi	Pichia guilliermondii ATCC 6260	XP_001482167.1	6 E-33	401/347
Eukaryota	Fungi	Pichia guilliermondii ATCC 6260	EDK41089.2	8 E-33	401/347
Eukaryota	Fungi	Aspergillus niger CBS 513.88	XP_001391209.1	2 E-32	324/347
Eukaryota	Fungi	Pyrenophora tritici-repentis Pt-1C-BFP	XP_001940599.1	2 E-32	322/347
Eukaryota	Fungi	Postia placenta Mad-698-R	XP_002474689.1	9 E-32	353/347
Eukaryota	Fungi	Coccidioides posadasii C735 delta	EER30056.1	3 E-31	328/347
Eukaryota	Fungi	Zygosaccharomyces rouxii CBS 732	XP_002498257.1	4 E-31	364/347
Eukaryota	Fungi	Sclerotinia sclerotiorum 1980 UF-70	XP_001584696.1	6 E-31	355/347
Eukaryota	Fungi	Nectria haematococca mpVI 77-13-4	EEU38123.1	1 E-30	351/347
Eukaryota	Fungi	Laccaria bicolor S238N-H82	XP_001890400.1	1 E-30	290/347
Eukaryota	Fungi	Aspergillus fumigatus A1163	EDP47130.1	3 E-30	315/347
Eukaryota	Fungi	Penicillium chrysogenum Wisconsin 54-1255	XP_002561337.1	4 E-30	350/347
Eukaryota	Fungi	Ustilago maydis 521	XP_759132.1	4 E-30	342/347
Eukaryota	Fungi	Aspergillus niger CBS 513.88	XP_001393503.1	6 E-30	342/347
Eukaryota	Fungi	Coccidioides immitis RS;	XP_001243192.1	7 E-30	336/347
Eukaryota	Fungi	Aspergillus oryzae RIB40	XP_001823631.1	8 E-30	351/347
Eukaryota	Fungi	Aspergillus nidulans FGSC A4	XP_660943.1	9 E-30	311/347
Eukaryota	Fungi	Talaromyces stipitatus ATCC 10500	XP_002480278.1	1 E-29	346/347
Eukaryota	Fungi	Aspergillus fumigatus Af293	XP_746386.1	1 E-29	315/347
Eukaryota	Fungi	Aspergillus oryzae RIB40	XP_001820536.1	2 E-29	320/347
Eukaryota	Fungi	Phaeosphaeria nodorum SN15	XP_001790885.1	2 E-29	322/347
Eukaryota	Fungi	Aspergillus oryzae RIB40	XP_001816827.1	4 E-29	325/347
Eukaryota	Fungi	Neosartorya fischeri NRRL 181	XP_001261417.1	5 E-29	343/347
Eukaryota	Fungi	Penicillium marneffei ATCC 18224	XP_002144973.1	5 E-29	328/347
Eukaryota	Fungi	Laccaria bicolor S238N-H82	XP_001883576.1	5 E-29	336/347

AFUA_6G11580

Eukaryota	Fungi	Aspergillus fumigatus Af293	XP_751041.1	0.0	377/377
Eukaryota	Fungi	Neosartorya fischeri NRRL 181	XP_001258275.1	0.0	362/377

Eukaryota	Fungi	Aspergillus clavatus NRRL 1	XP_001268254.1	0.0	362/377
Eukaryota	Fungi	Aspergillus oryzae RIB40	XP_001820969.1	1 E-176	360/377
Eukaryota	Fungi	Aspergillus niger CBS 513.88	XP_001389578.1	1 E-174	352/377
Eukaryota	Fungi	Aspergillus nidulans FGSC A4	XP_658755.1	1 E-172	360/377
Eukaryota	Fungi	Penicillium chrysogenum Wisconsin 54-1255	XP_002562760.1	1 E-172	360/377
Eukaryota	Fungi	Aspergillus terreus NIH2624	XP_001214183.1	1 E-167	364/377
Eukaryota	Fungi	Talaromyces stipitatus ATCC 10500	XP_002487830.1	1 E-159	360/377
Eukaryota	Fungi	Penicillium marneffei ATCC 18224	XP_002153593.1	1 E-158	360/377
Eukaryota	Fungi	Ajellomyces dermatitidis ER-3	EEQ90359.1	1 E-153	360/377
Eukaryota	Fungi	Paracoccidioides brasiliensis Pb18;	EEH45748.1	1 E-153	369/377
Eukaryota	Fungi	Paracoccidioides brasiliensis Pb01;	EEH40897.1	1 E-153	369/377
Eukaryota	Fungi	Ajellomyces dermatitidis SLH14081	XP_002623812.1	1 E-152	360/377
Eukaryota	Fungi	Paracoccidioides brasiliensis Pb03;	EEH21103.1	1 E-151	360/377
Eukaryota	Fungi	Ajellomyces capsulatus G186AR	EEH07950.1	1 E-149	360/377
Eukaryota	Fungi	Ajellomyces capsulatus H143	EER42918.1	1 E-146	358/377
Eukaryota	Fungi	Neurospora crassa OR74A	XP_957686.1	1 E-135	363/377
Eukaryota	Fungi	Botryotinia fuckeliana B05.10	XP_001559250.1	1 E-135	360/377
Eukaryota	Fungi	Sclerotinia sclerotiorum 1980 UF-70	XP_001596557.1	1 E-134	360/377
Eukaryota	Fungi	Ajellomyces capsulatus NAm1	XP_001541290.1	1 E-132	337/377
Eukaryota	Fungi	Phaeosphaeria nodorum SN15	XP_001793813.1	1 E-132	361/377
Eukaryota	Fungi	Pyrenophora tritici-repentis Pt-1C-BFP	XP_001932578.1	1 E-131	361/377
Eukaryota	Fungi	Podospora anserina DSM 980	XP_001906119.1	1 E-131	363/377
Eukaryota	Fungi	Nectria haematococca mpVI 77-13-4	EEU41044.1	1 E-129	360/377
Eukaryota	Fungi	Gibberella zeae PH-1	XP_389661.1	1 E-127	361/377
Eukaryota	Fungi	Verticillium albo-atrum VaMs.102	EEY21992.1	1 E-127	360/377
Eukaryota	Fungi	Magnaporthe grisea 70-15	XP_363409.2	1 E-123	369/377
Eukaryota	Fungi	Epichloe festucae	BAE06851.1	1 E-119	364/377
Eukaryota	Fungi	Chaetomium globosum CBS 148.51	XP_001227169.1	1 E-115	359/377
Eukaryota	Fungi	Aspergillus oryzae RIB40	XP_001817424.1	3 E-43	353/377
Eukaryota	Fungi	Nectria haematococca mpVI 77-13-4	EEU38778.1	1 E-42	350/377

AFUA_6G11630

Eukaryota	Fungi	Aspergillus fumigatus Af293	XP_751046.2	0.0	557/557
Eukaryota	Fungi	Penicillium marneffei ATCC 18224	XP_002153033.1	1 E-122	559/557
Eukaryota	Fungi	Podospora anserina	CAP65464.1	1 E-121	561/557
Eukaryota	Fungi	Talaromyces stipitatus ATCC 10500	XP_002483865.1	1 E-116	586/557
Eukaryota	Fungi	Gibberella zeae PH-1	XP_391370.1	1 E-113	551/557
Eukaryota	Fungi	Neurospora crassa OR74A	XP_961264.1	1 E-112	540/557
Eukaryota	Fungi	Aspergillus nidulans FGSC A4	XP_664685.1	1 E-111	565/557
Eukaryota	Fungi	Sclerotinia sclerotiorum 1980 UF-70	XP_001595905.1	1 E-110	563/557
Eukaryota	Fungi	Talaromyces stipitatus ATCC 10500	XP_002483521.1	1 E-109	558/557
Eukaryota	Fungi	Pyrenophora tritici-repentis Pt-1C-BFP	XP_001931607.1	1 E-108	552/557
Eukaryota	Fungi	Aspergillus oryzae RIB40	XP_001824933.1	1 E-108	538/557
Eukaryota	Fungi	Aspergillus nidulans FGSC A4	XP_660252.1	1 E-107	536/557
Eukaryota	Fungi	Penicillium marneffei ATCC 18224	XP_002150632.1	1 E-106	547/557
Eukaryota	Fungi	Gibberella zeae	ABB90284.1	1 E-106	546/557
Eukaryota	Fungi	Aspergillus fumigatus Af293	XP_001481692.1	1 E-105	554/557
Eukaryota	Fungi	Pyrenophora tritici-repentis Pt-1C-BFP	XP_001934279.1	1 E-105	533/557
Eukaryota	Fungi	Aspergillus niger CBS 513.88	XP_001394372.1	1 E-104	537/557
Eukaryota	Fungi	Nectria haematococca mpVI 77-13-4	EEU37929.1	1 E-104	534/557
Eukaryota	Fungi	Neosartorya fischeri NRRL 181	XP_001265527.1	1 E-104	543/557
Eukaryota	Fungi	Aspergillus clavatus NRRL 1	XP_001269998.1	1 E-103	556/557
Eukaryota	Fungi	Aspergillus niger CBS 513.88	XP_001390502.1	1 E-103	535/557
Eukaryota	Fungi	Gibberella zeae PH-1	XP_388014.1	1 E-102	535/557
Eukaryota	Fungi	Aspergillus terreus NIH2624	XP_001209796.1	1 E-102	562/557
Eukaryota	Fungi	Talaromyces stipitatus ATCC 10500	XP_002341000.1	1 E-101	535/557
Eukaryota	Fungi	Sclerotinia sclerotiorum 1980 UF-70	XP_001595996.1	1 E-101	553/557

Eukaryota	Fungi	Aspergillus nidulans FGSC A4	CBF81578.1	1 E-100	535/557
Eukaryota	Fungi	Podospora anserina DSM 980	XP_001911121.1	1 E-100	541/557
Eukaryota	Fungi	Aspergillus oryzae RIB40	XP_001817422.1	1 E-100	559/557
Eukaryota	Fungi	Penicillium chrysogenum Wisconsin 54-1255	XP_002565565.1	1 E-99	540/557
Eukaryota	Fungi	Aspergillus flavus NRRL3357	XP_002372496.1	2 E-99	559/557
Eukaryota	Fungi	Aspergillus clavatus NRRL 1	XP_001276774.1	7 E-99	550/557
Eukaryota	Fungi	Nectria haematococca mpVI 77-13-4	EEU43345.1	9 E-99	560/557
Eukaryota	Fungi	Penicillium chrysogenum Wisconsin 54-1255	XP_002563824.1	3 E-97	537/557
Eukaryota	Fungi	Aspergillus niger CBS 513.88	XP_001396733.1	4 E-97	538/557
Eukaryota	Fungi	Botryotinia fuckeliana B05.10	XP_001557065.1	7 E-97	548/557
Eukaryota	Fungi	Penicillium marneffei ATCC 18224	XP_002145855.1	1 E-96	536/557
Eukaryota	Fungi	Aspergillus clavatus NRRL 1	XP_001273097.1	1 E-95	565/557
Eukaryota	Fungi	Aspergillus terreus NIH2624	XP_001208636.1	4 E-95	545/557
Eukaryota	Fungi	Pyrenophora tritici-repentis Pt-1C-BFP	XP_001934578.1	9 E-95	534/557
Eukaryota	Fungi	Aspergillus fumigatus Af293	XP_747715.1	4 E-94	538/557
Eukaryota	Fungi	Aspergillus fumigatus A1163	EDP47621.1	4 E-94	538/557
Eukaryota	Fungi	Neosartorya fischeri NRRL 181	XP_001257567.1	8 E-93	538/557
Eukaryota	Fungi	Phaeosphaeria nodorum SN15	XP_001793119.1	1 E-92	465/557
Eukaryota	Fungi	Nectria haematococca mpVI 77-13-4	EEU38742.1	4 E-92	556/557
Eukaryota	Fungi	Aspergillus flavus NRRL3357	XP_002383046.1	4 E-92	546/557
Eukaryota	Fungi	Verticillium albo-atrum VaMs.102	EEY17127.1	1 E-91	517/557
Eukaryota	Fungi	Aspergillus oryzae RIB40	XP_001817015.1	1 E-91	546/557
Eukaryota	Fungi	Penicillium chrysogenum Wisconsin 54-1255	XP_002566160.1	1 E-91	559/557
Eukaryota	Fungi	Microsporum canis CBS 113480	EEQ31308.1	1 E-90	541/557
Eukaryota	Fungi	Penicillium griseofulvum	ABQ01445.1	1 E-89	550/557
Eukaryota	Fungi	Aspergillus flavus NRRL3357	XP_002383651.1	6 E-89	466/557
Eukaryota	Fungi	Botryotinia fuckeliana B05.10	XP_001555842.1	2 E-88	543/557
Eukaryota	Fungi	Pyrenophora tritici-repentis Pt-1C-BFP	XP_001934587.1	4 E-86	485/557
Eukaryota	Fungi	Aspergillus nidulans FGSC A4	XP_664757.1	4 E-83	555/557
Eukaryota	Fungi	Phaeosphaeria nodorum SN15	XP_001806091.1	4 E-81	472/557
Eukaryota	Fungi	Aspergillus flavus NRRL3357	XP_002384068.1	1 E-80	547/557
Eukaryota	Fungi	Aspergillus oryzae RIB40	XP_001824564.1	2 E-80	547/557
Eukaryota	Fungi	Botryotinia fuckeliana B05.10	XP_001551855.1	4 E-80	528/557
Eukaryota	Fungi	Verticillium albo-atrum VaMs.102	EEY16254.1	1 E-79	506/557
Eukaryota	Fungi	Phaeosphaeria nodorum SN15	XP_001799898.1	4 E-78	470/557
Eukaryota	Fungi	Talaromyces stipitatus ATCC 10500	XP_002479467.1	2 E-77	549/557
Eukaryota	Fungi	Pyrenophora tritici-repentis Pt-1C-BFP	XP_001941500.1	2 E-77	557/557
Eukaryota	Fungi	Verticillium albo-atrum VaMs.102	EEY21424.1	4 E-77	463/557
Eukaryota	Fungi	Aspergillus niger CBS 513.88	XP_001388495.1	5 E-77	556/557
Eukaryota	Fungi	Nectria haematococca mpVI 77-13-4	EEU33866.1	9 E-77	545/557
Eukaryota	Fungi	Botryotinia fuckeliana B05.10	XP_001555136.1	1 E-76	451/557
Eukaryota	Fungi	Podospora anserina DSM 980	XP_001906356.1	2 E-75	573/557
Eukaryota	Fungi	Aspergillus niger CBS 513.88	XP_001390807.1	3 E-75	542/557
Eukaryota	Fungi	Neosartorya fischeri NRRL 181	XP_001263737.1	4 E-75	568/557
Eukaryota	Fungi	Pyrenophora tritici-repentis Pt-1C-BFP	XP_001937513.1	4 E-75	542/557
Eukaryota	Fungi	Nectria haematococca mpVI 77-13-4	EEU41417.1	7 E-75	546/557
Eukaryota	Fungi	Talaromyces stipitatus ATCC 10500	XP_002482842.1	1 E-74	551/557
Eukaryota	Fungi	Phaeosphaeria nodorum SN15	XP_001802939.1	2 E-74	547/557
Eukaryota	Fungi	Gibberella zeae PH-1	XP_383483.1	1 E-73	544/557
Eukaryota	Fungi	Neosartorya fischeri NRRL 181	XP_001266456.1	6 E-73	557/557
Eukaryota	Fungi	Aspergillus terreus NIH2624	XP_001209125.1	9 E-73	545/557
Eukaryota	Fungi	Penicillium chrysogenum Wisconsin 54-1255	XP_002562785.1	1 E-72	539/557
Eukaryota	Fungi	Penicillium marneffei ATCC 18224	XP_002143167.1	2 E-72	539/557
Eukaryota	Fungi	Phaeosphaeria nodorum SN15	XP_001806615.1	4 E-72	545/557
Eukaryota	Fungi	Talaromyces stipitatus ATCC 10500	XP_002482839.1	7 E-72	562/557
Eukaryota	Fungi	Nectria haematococca mpVI 77-13-4	EEU40561.1	2 E-71	565/557
Eukaryota	Fungi	Nectria haematococca mpVI 77-13-4	EEU41465.1	2 E-71	541/557
Eukaryota	Fungi	Pyrenophora tritici-repentis Pt-1C-BFP	XP_001939417.1	7 E-71	539/557

Eukaryota	Fungi	Aspergillus flavus NRRL3357	XP_002373522.1	2 E-70	545/557
Eukaryota	Fungi	Aspergillus oryzae RIB40	XP_001818300.1	2 E-70	545/557
Eukaryota	Fungi	Nectria haematococca mpVI 77-13-4	EEU37829.1	3 E-70	564/557
Eukaryota	Fungi	Chaetomium globosum CBS 148.51	XP_001222939.1	8 E-70	573/557
Eukaryota	Fungi	Podospora anserina DSM 980	XP_001903272.1	2 E-69	567/557
Eukaryota	Fungi	Chaetomium globosum CBS 148.51	XP_001224432.1	3 E-69	539/557
Eukaryota	Fungi	Neurospora crassa OR74A	XP_962853.1	5 E-69	579/557
Eukaryota	Fungi	Gibberella zeae PH-1	XP_387984.1	1 E-68	546/557
Eukaryota	Fungi	Chaetomium globosum CBS 148.51	XP_001222229.1	2 E-67	470/557
Eukaryota	Fungi	Botryotinia fuckeliana B05.10	XP_001549723.1	2 E-67	562/557
Eukaryota	Fungi	Phaeosphaeria nodorum SN15	XP_001797142.1	2 E-66	549/557
Eukaryota	Fungi	Coprinopsis cinerea okayama7#130	XP_001841108.1	3 E-66	536/557
Eukaryota	Fungi	Sclerotinia sclerotiorum 1980 UF-70	XP_001591096.1	3 E-66	469/557
Eukaryota	Fungi	Nectria haematococca mpVI 77-13-4	EEU34972.1	6 E-66	594/557
Eukaryota	Fungi	Nectria haematococca mpVI 77-13-4	EEU44863.1	9 E-66	539/557
Eukaryota	Fungi	Pyrenophora tritici-repentis Pt-1C-BFP	XP_001931063.1	5 E-65	549/557
Eukaryota	Fungi	Aspergillus clavatus NRRL 1	XP_001270732.1	7 E-65	545/557
Eukaryota	Fungi	Phaeosphaeria nodorum SN15	XP_001798846.1	4 E-64	549/557
Eukaryota	Fungi	Coprinopsis cinerea okayama7#130	XP_001841048.1	4 E-64	564/557
Eukaryota	Fungi	Sclerotinia sclerotiorum 1980 UF-70	XP_001585158.1	7 E-64	562/557
Eukaryota	Fungi	Talaromyces stipitatus ATCC 10500	XP_002486732.1	1 E-63	545/557
Eukaryota	Fungi	Podospora anserina DSM 980	XP_001904279.1	1 E-63	566/557
Eukaryota	Fungi	Aspergillus flavus NRRL3357	XP_002383067.1	1 E-63	565/557
Eukaryota	Fungi	Pyrenophora tritici-repentis Pt-1C-BFP	XP_001939318.1	3 E-63	573/557
Eukaryota	Fungi	Podospora anserina DSM 980	XP_001904287.1	2 E-62	572/557
Eukaryota	Fungi	Podospora anserina DSM 980	XP_001903560.1	2 E-62	578/557
Eukaryota	Fungi	Magnaporthe grisea 70-15	XP_001405975.1	6 E-62	557/557
Eukaryota	Fungi	Magnaporthe grisea 70-15	XP_001522269.1	6 E-62	543/557
Eukaryota	Fungi	Nectria haematococca mpVI 77-13-4	EEU44366.1	8 E-62	544/557
Eukaryota	Fungi	Gibberella zeae PH-1	XP_384148.1	1 E-61	562/557
Eukaryota	Fungi	Chaetomium globosum CBS 148.51	XP_001227930.1	2 E-61	556/557
Eukaryota	Fungi	Neosartorya fischeri NRRL 181	XP_001263995.1	7 E-61	554/557
Eukaryota	Fungi	Coprinopsis cinerea okayama7#130	XP_001836380.1	9 E-61	539/557
Eukaryota	Fungi	Chaetomium globosum CBS 148.51	XP_001228624.1	2 E-60	562/557
Eukaryota	Fungi	Chaetomium globosum CBS 148.51	XP_001222477.1	3 E-60	590/557
Eukaryota	Fungi	Magnaporthe grisea 70-15	XP_366574.2	4 E-60	520/557
Eukaryota	Fungi	Coprinopsis cinerea okayama7#130	XP_001838957.1	6 E-60	548/557
Eukaryota	Fungi	Aspergillus oryzae RIB40	XP_001816994.1	6 E-60	558/557
Eukaryota	Fungi	Coccidioides posadasii C735 delta	EER27468.1	1 E-59	560/557
Eukaryota	Fungi	Ajellomyces capsulatus G186AR	EEH07998.1	2 E-59	572/557
Eukaryota	Fungi	Botryotinia fuckeliana B05.10	XP_001547442.1	4 E-59	496/557
Eukaryota	Fungi	Microsporum canis CBS 113480	EEQ33996.1	5 E-59	562/557
Eukaryota	Fungi	Coprinopsis cinerea okayama7#130	XP_001838967.1	9 E-59	568/557
Eukaryota	Fungi	Ajellomyces capsulatus H143	EER39924.1	1 E-58	572/557
Eukaryota	Fungi	Neosartorya fischeri NRRL 181	XP_001261742.1	3 E-58	599/557
Eukaryota	Fungi	Penicillium chrysogenum Wisconsin 54-1255	XP_002569125.1	7 E-58	587/557
Eukaryota	Fungi	Coccidioides posadasii C735 delta	EER23163.1	7 E-58	551/557
Eukaryota	Fungi	Coccidioides immitis RS;	XP_001248265.1	8 E-58	535/557
Eukaryota	Fungi	Phaeosphaeria nodorum SN15	XP_001794565.1	9 E-58	451/557
Eukaryota	Fungi	Aspergillus terreus NIH2624	XP_001215566.1	1 E-57	576/557
Eukaryota	Fungi	Aspergillus fumigatus Af293	XP_753129.2	1 E-57	553/557
Eukaryota	Fungi	Aspergillus terreus NIH2624	XP_001216835.1	2 E-57	577/557
Eukaryota	Fungi	Uncinocarpus reesii 1704	XP_002544591.1	3 E-57	561/557
Eukaryota	Fungi	Aspergillus oryzae RIB40	XP_001821740.1	3 E-57	565/557
Eukaryota	Fungi	Ajellomyces capsulatus NAm1	XP_001541250.1	6 E-57	567/557
Eukaryota	Fungi	Neosartorya fischeri NRRL 181	XP_001261609.1	7 E-57	566/557
Eukaryota	Fungi	Podospora anserina	CAP65560.1	1 E-56	504/557
Eukaryota	Fungi	Aspergillus niger CBS 513.88	XP_001389559.1	2 E-56	587/557

Eukaryota	Fungi	Penicillium chrysogenum Wisconsin 54-1255	XP_002567989.1	2 E-56	585/557
Eukaryota	Fungi	Podospora anserina DSM 980	XP_001911553.1	5 E-56	558/557
Eukaryota	Fungi	Neosartorya fischeri NRRL 181	XP_001267599.1	8 E-56	573/557
Eukaryota	Fungi	Aspergillus flavus NRRL3357	XP_002379680.1	1 E-54	576/557
Eukaryota	Fungi	Aspergillus fumigatus Af293	XP_748853.1	2 E-54	575/557
Eukaryota	Fungi	Aspergillus fumigatus A1163	EDP49092.1	7 E-54	602/557
Eukaryota	Fungi	Aspergillus fumigatus Af293	XP_754868.1	1 E-53	566/557
Eukaryota	Fungi	Coccidioides immitis RS;	XP_001242377.1	1 E-53	551/557
Eukaryota	Fungi	Podospora anserina DSM 980	XP_001906789.1	1 E-53	537/557
Eukaryota	Fungi	Aspergillus fumigatus Af293	XP_747087.1	2 E-53	602/557
Eukaryota	Fungi	Microsporum canis CBS 113480	EEQ35458.1	2 E-53	537/557
Eukaryota	Fungi	Aspergillus terreus NIH2624	XP_001209092.1	3 E-53	570/557
Eukaryota	Fungi	Aspergillus nidulans FGSC A4	XP_663021.1	3 E-53	566/557
Eukaryota	Fungi	Aspergillus fumigatus A1163	EDP52996.1	4 E-53	566/557
Eukaryota	Fungi	Aspergillus fumigatus Af293	XP_746413.1	7 E-53	565/557
Eukaryota	Fungi	Neosartorya fischeri NRRL 181	XP_001262117.1	1 E-52	571/557
Eukaryota	Fungi	Aspergillus nidulans FGSC A4	XP_682500.1	1 E-52	551/557
Eukaryota	Fungi	Gibberella zeae PH-1	XP_386614.1	2 E-52	594/557
Eukaryota	Fungi	Aspergillus fumigatus A1163	EDP47102.1	2 E-52	565/557
Eukaryota	Fungi	Neosartorya fischeri NRRL 181	XP_001263701.1	2 E-52	567/557
Eukaryota	Fungi	Neurospora crassa OR74A	XP_961706.1	5 E-52	552/557
Eukaryota	Fungi	Chaetomium globosum CBS 148.51	XP_001225193.1	7 E-52	566/557
Eukaryota	Fungi	Sclerotinia sclerotiorum 1980 UF-70	XP_001590955.1	1 E-51	582/557
Eukaryota	Fungi	Aspergillus oryzae RIB40	XP_001825017.1	1 E-51	549/557
Eukaryota	Fungi	Aspergillus niger CBS 513.88	XP_001400920.1	5 E-51	552/557
Eukaryota	Fungi	Verticillium albo-atrum VaMs.102	EEY23393.1	6 E-51	535/557
Eukaryota	Fungi	Ajellomyces capsulatus NAm1	XP_001536168.1	9 E-51	565/557
Eukaryota	Fungi	Ajellomyces capsulatus H143	EER37323.1	3 E-50	545/557
Eukaryota	Fungi	Gibberella zeae PH-1	XP_388205.1	4 E-50	564/557
Eukaryota	Fungi	Aspergillus niger CBS 513.88	XP_001393450.1	1 E-49	558/557
Eukaryota	Fungi	Aspergillus oryzae RIB40	XP_001824522.1	3 E-49	581/557
Eukaryota	Fungi	Botryotinia fuckeliana B05.10	XP_001546643.1	3 E-49	555/557
Eukaryota	Fungi	Ajellomyces capsulatus G186AR	EEH03743.1	7 E-49	453/557
Eukaryota	Fungi	Aspergillus fumigatus A1163	EDP48498.1	2 E-48	558/557
Eukaryota	Fungi	Aspergillus flavus NRRL3357	XP_002384119.1	3 E-48	581/557
Eukaryota	Fungi	Magnaporthe grisea 70-15	XP_366742.1	3 E-48	567/557
Eukaryota	Fungi	Ajellomyces capsulatus G186AR	EEH03190.1	5 E-48	563/557
Eukaryota	Fungi	Aspergillus nidulans FGSC A4	XP_664063.1	2 E-47	557/557
Eukaryota	Fungi	Ajellomyces dermatitidis SLH14081	XP_002622940.1	3 E-47	549/557
Eukaryota	Fungi	Aspergillus flavus NRRL3357	XP_002379056.1	3 E-47	545/557
Eukaryota	Fungi	Botryotinia fuckeliana B05.10	XP_001548413.1	5 E-47	466/557
Eukaryota	Fungi	Aspergillus oryzae RIB40	XP_001823632.1	7 E-47	533/557
Eukaryota	Fungi	Aspergillus oryzae RIB40	XP_001727459.1	8 E-47	570/557
Eukaryota	Fungi	Aspergillus clavatus NRRL 1	XP_001270775.1	1 E-46	567/557
Eukaryota	Fungi	Aspergillus fumigatus Af293	XP_756142.1	1 E-46	474/557
Eukaryota	Fungi	Aspergillus nidulans FGSC A4	XP_681421.1	2 E-46	550/557
Eukaryota	Fungi	Neotyphodium lolii	ABM91450.1	3 E-46	550/557
Eukaryota	Fungi	Aspergillus niger CBS 513.88	XP_001399184.1	3 E-46	524/557
Eukaryota	Fungi	Pyrenophora tritici-repentis Pt-1C-BFP	XP_001942342.1	4 E-46	572/557
Eukaryota	Fungi	Aspergillus flavus NRRL3357	XP_002375728.1	5 E-46	570/557
Eukaryota	Fungi	Ajellomyces capsulatus H143	EER37791.1	8 E-46	494/557
Eukaryota	Fungi	Ajellomyces capsulatus NAm1	XP_001544312.1	2 E-45	546/557
Eukaryota	Fungi	Aspergillus oryzae RIB40	XP_001817392.1	3 E-45	568/557
Eukaryota	Fungi	Aspergillus flavus NRRL3357	XP_002372465.1	4 E-45	568/557
Eukaryota	Fungi	Claviceps fusiformis	ABV57823.1	6 E-45	542/557
Eukaryota	Fungi	Sclerotinia sclerotiorum 1980 UF-70	XP_001596924.1	1 E-44	492/557
Eukaryota	Fungi	Penicillium chrysogenum Wisconsin 54-1255	XP_002567314.1	2 E-44	524/557
Eukaryota	Fungi	Gibberella zeae PH-1	XP_381981.1	8 E-44	581/557

Eukaryota	Fungi	Aspergillus fumigatus A1163	EDP48041.1	2 E-43	526/557
Eukaryota	Fungi	Aspergillus fumigatus Af293	XP_746836.1	2 E-43	526/557
Eukaryota	Fungi	Neosartorya fischeri NRRL 181	XP_001262731.1	2 E-42	526/557
Eukaryota	Fungi	Coprinopsis cinerea okayama7#130	XP_001835521.1	4 E-42	521/557
Eukaryota	Fungi	Laccaria bicolor S238N-H82	XP_001882568.1	5 E-42	564/557
Eukaryota	Fungi	Aspergillus terreus NIH2624	XP_001209485.1	7 E-42	480/557
Eukaryota	Fungi	Aspergillus oryzae RIB40	XP_001822172.1	1 E-41	541/557
Eukaryota	Fungi	Nectria haematococca mpVI 77-13-4	EEU33852.1	4 E-41	590/557
Eukaryota	Fungi	Coprinopsis cinerea okayama7#130	XP_001835527.1	3 E-40	527/557
Eukaryota	Fungi	Talaromyces stipitatus ATCC 10500	XP_002486008.1	3 E-37	511/557
Eukaryota	Fungi	Hypomyces subiculosus	ACD39759.1	8 E-37	584/557
Eukaryota	Fungi	Chaetomium globosum CBS 148.51	XP_001226057.1	9 E-36	452/557
Eukaryota	Fungi	Magnaporthe grisea 70-15	XP_362664.2	1 E-34	459/557
Eukaryota	Fungi	Pyrenophora tritici-repentis Pt-1C-BFP	XP_001936188.1	2 E-34	461/557

AFUA_6G11910

Eukaryota	Fungi	Aspergillus fumigatus Af293	XP_751071.1	0.0	856/856
Eukaryota	Fungi	Neosartorya fischeri NRRL 181	XP_001258306.1	0.0	864/856
Eukaryota	Fungi	Aspergillus nidulans FGSC A4	XP_681134.1	0.0	799/856
Eukaryota	Fungi	Aspergillus fumigatus A1163	EDP51467.1	0.0	838/856
Eukaryota	Fungi	Aspergillus fumigatus Af293	XP_753915.1	0.0	838/856
Eukaryota	Fungi	Aspergillus oryzae RIB40	XP_001823113.1	0.0	839/856
Eukaryota	Fungi	Neosartorya fischeri NRRL 181	XP_001259962.1	0.0	838/856
Eukaryota	Fungi	Aspergillus clavatus NRRL 1	XP_001274045.1	0.0	838/856
Eukaryota	Fungi	Aspergillus flavus NRRL3357	XP_002378447.1	0.0	856/856
Eukaryota	Fungi	Emericella nidulans	ABF50853.1	0.0	838/856
Eukaryota	Fungi	Penicillium chrysogenum Wisconsin 54-1255	XP_002568817.1	0.0	840/856
Eukaryota	Fungi	Aspergillus nidulans FGSC A4	XP_659831.1	0.0	833/856
Eukaryota	Fungi	Aspergillus niger CBS 513.88	XP_001398281.1	0.0	818/856
Eukaryota	Fungi	Aspergillus terreus NIH2624	XP_001217951.1	0.0	839/856
Eukaryota	Fungi	Talaromyces stipitatus ATCC 10500	XP_002481927.1	0.0	842/856
Eukaryota	Fungi	Penicillium marneffei ATCC 18224	XP_002147752.1	0.0	842/856
Eukaryota	Fungi	Aspergillus niger CBS 513.88	XP_001390414.1	0.0	832/856
Eukaryota	Fungi	Aspergillus oryzae RIB40	XP_001827086.1	0.0	825/856
Eukaryota	Fungi	Aspergillus flavus NRRL3357	XP_002384315.1	0.0	825/856
Eukaryota	Fungi	Nectria haematococca mpVI 77-13-4	EEU35055.1	0.0	846/856
Eukaryota	Fungi	Nectria haematococca mpVI 77-13-4	EEU37677.1	0.0	840/856
Eukaryota	Fungi	Gibberella zeae PH-1	XP_380264.1	0.0	783/856
Eukaryota	Fungi	Botryotinia fuckeliana B05.10	XP_001547918.1	0.0	826/856
Eukaryota	Fungi	Clavispora lusitaniae ATCC 42720	XP_002618661.1	0.0	837/856
Eukaryota	Fungi	Sclerotinia sclerotiorum 1980 UF-70	XP_001598932.1	0.0	828/856
Eukaryota	Fungi	Nectria haematococca mpVI 77-13-4	EEU39618.1	0.0	804/856
Eukaryota	Fungi	Gibberella zeae PH-1	XP_382808.1	0.0	804/856
Eukaryota	Fungi	Pichia stipitis CBS 6054	XP_001385159.1	0.0	831/856
Eukaryota	Fungi	Debaryomyces hansenii CBS767	XP_457283.1	0.0	837/856
Eukaryota	Fungi	Debaryomyces hansenii	CAG85284.2	0.0	837/856
Eukaryota	Fungi	Magnaporthe grisea 70-15	XP_362540.1	0.0	835/856
Eukaryota	Fungi	Candida tropicalis MYA-3404	XP_002547835.1	0.0	837/856
Eukaryota	Fungi	Pichia guilliermondii ATCC 6260	EDK39818.2	0.0	821/856
Eukaryota	Fungi	Verticillium albo-atrum VaMs.102	EEY15838.1	0.0	805/856
Eukaryota	Fungi	Neosartorya fischeri NRRL 181	XP_001258578.1	0.0	823/856
Eukaryota	Fungi	Pichia guilliermondii ATCC 6260	XP_001484535.1	0.0	821/856
Eukaryota	Fungi	Aspergillus fumigatus Af293	XP_751323.1	0.0	823/856
Eukaryota	Fungi	Yarrowia lipolytica CLIB122	XP_504871.1	0.0	826/856
Eukaryota	Fungi	Hypocrea jecorina	AAP57756.1	0.0	833/856
Eukaryota	Fungi	Aspergillus nidulans FGSC A4	XP_660216.1	0.0	838/856
Eukaryota	Fungi	Aspergillus fumigatus A1163	EDP55338.1	0.0	823/856

Eukaryota	Fungi	Talaromyces stipitatus ATCC 10500	XP_002483357.1	0.0	826/856
Eukaryota	Fungi	Aspergillus clavatus NRRL 1	XP_001268481.1	0.0	823/856
Eukaryota	Fungi	Nectria haematococca mpVI 77-13-4	EEU39827.1	0.0	825/856
Eukaryota	Fungi	Aspergillus flavus NRRL3357	XP_002382282.1	0.0	823/856
Eukaryota	Fungi	Penicillium chrysogenum Wisconsin 54-1255	XP_002560677.1	0.0	822/856
Eukaryota	Fungi	Penicillium marneffei ATCC 18224	XP_002149460.1	0.0	830/856
Eukaryota	Fungi	Nectria haematococca mpVI 77-13-4	EEU42596.1	0.0	831/856
Eukaryota	Fungi	Debaryomyces hansenii	CAG90292.2	0.0	839/856
Eukaryota	Fungi	Debaryomyces hansenii CBS767	XP_461831.1	0.0	839/856
Eukaryota	Fungi	Candida albicans WO-1	EEQ44248.1	0.0	858/856
Eukaryota	Fungi	Aspergillus oryzae RIB40	XP_001822871.1	0.0	805/856
Eukaryota	Fungi	Talaromyces stipitatus ATCC 10500	XP_002481944.1	0.0	830/856
Eukaryota	Fungi	Aspergillus terreus NIH2624	XP_001213247.1	0.0	810/856
Eukaryota	Fungi	Clavispora lusitaniae ATCC 42720	XP_002620022.1	0.0	816/856
Eukaryota	Fungi	Pichia stipitis CBS 6054	XP_001383273.1	0.0	837/856
Eukaryota	Fungi	Pichia stipitis CBS 6054	XP_001387766.1	0.0	843/856
Eukaryota	Fungi	Candida albicans SC5314	XP_721741.1	0.0	860/856
Eukaryota	Fungi	Magnaporthe grisea 70-15	XP_365947.2	0.0	804/856
Eukaryota	Fungi	Pichia guilliermondii ATCC 6260	EDK38776.2	0.0	835/856
Eukaryota	Fungi	Pichia stipitis CBS 6054	XP_001387350.1	0.0	843/856
Eukaryota	Fungi	Pichia pastoris GS115	XP_002493421.1	0.0	839/856
Eukaryota	Fungi	Pichia guilliermondii ATCC 6260	XP_001485145.1	0.0	835/856
Eukaryota	Fungi	Kluyveromyces lactis NRRL Y-1140	XP_454609.1	0.0	845/856
Eukaryota	Fungi	Pichia etchellsii	ACF93471.1	0.0	845/856
Eukaryota	Fungi	Schizosaccharomyces pombe	NP_595060.1	0.0	832/856
Eukaryota	Fungi	Kluyveromyces marxianus	P07337.1	0.0	845/856
Eukaryota	Fungi	Pichia stipitis CBS 6054	XP_001385685.2	0.0	732/856
Eukaryota	Fungi	Pichia stipitis CBS 6054	XP_001384652.2	0.0	732/856
Eukaryota	Fungi	Nectria haematococca mpVI 77-13-4	EEU35060.1	0.0	834/856
Eukaryota	Fungi	Kluyveromyces lactis NRRL Y-1140	XP_453086.1	0.0	746/856
Eukaryota	Fungi	Postia placenta Mad-698-R	XP_002475642.1	0.0	845/856
Eukaryota	Fungi	Cryptococcus neoformans var. neoformans	XP_772555.1	0.0	847/856
Bacteria	Chloroflexi	Roseiflexus castenholzii DSM 13941	YP_001431052.1	0.0	798/856
Eukaryota	Fungi	Volvariella volvacea	AAG59831.1	1 E-179	852/856
Eukaryota	Fungi	Rhizoctonia solani	ABL67526.1	1 E-178	834/856
Eukaryota	Fungi	Coprinopsis cinerea okayama7#130	XP_001833567.1	1 E-177	844/856
Eukaryota	Fungi	Coprinopsis cinerea okayama7#130	XP_001841056.1	1 E-177	866/856
Eukaryota	Fungi	Agaricus bisporus	CAC03462.1	1 E-175	851/856
Eukaryota	Fungi	Cryptococcus neoformans var. neoformans	XP_572229.1	1 E-175	836/856
Bacteria	Chloroflexi	Roseiflexus sp. RS-1	YP_001277788.1	1 E-174	801/856
Eukaryota	Fungi	Postia placenta Mad-698-R	XP_002472846.1	1 E-173	804/856
Eukaryota	Fungi	Postia placenta Mad-698-R	XP_002475984.1	1 E-173	835/856
Eukaryota	Fungi	Postia placenta Mad-698-R	XP_002474718.1	1 E-173	835/856
Eukaryota	Fungi	Nectria haematococca mpVI 77-13-4	EEU47162.1	1 E-171	842/856
Bacteria	Chloroflexi	Chloroflexus aggregans DSM 9485	YP_002463028.1	1 E-171	789/856
Bacteria	Chloroflexi	Chloroflexus aurantiacus J-10-fl	YP_001634694.1	1 E-170	788/856
Eukaryota	Fungi	Gibberella zeae PH-1	XP_385089.1	1 E-170	804/856
Eukaryota	Fungi	Hypocrea jecorina	AAP57759.1	1 E-168	697/856
Eukaryota	Fungi	Neosartorya fischeri NRRL 181	XP_001258628.1	1 E-165	845/856
Eukaryota	Fungi	Nectria haematococca mpVI 77-13-4	EEU33850.1	1 E-164	829/856
Eukaryota	Fungi	Phaeosphaeria nodorum SN15	XP_001795098.1	1 E-163	812/856
Eukaryota	Fungi	Cryptococcus neoformans var. neoformans	XP_775244.1	1 E-163	825/856
Eukaryota	Fungi	Aspergillus fumigatus Af293	XP_748345.1	1 E-162	820/856
Eukaryota	Fungi	Aspergillus fumigatus A1163	EDP53635.1	1 E-162	820/856
Eukaryota	Fungi	Nectria haematococca mpVI 77-13-4	EEU36649.1	1 E-161	833/856
Eukaryota	Fungi	Pyrenophora tritici-repentis Pt-1C-BFP	XP_001937975.1	1 E-160	803/856
Eukaryota	Fungi	Nectria haematococca mpVI 77-13-4	EEU48246.1	1 E-160	778/856
Eukaryota	Fungi	Botryotinia fuckeliana B05.10	XP_001545802.1	1 E-158	848/856

Bacteria	Proteobacteria	Agrobacterium vitis S4	YP_002547944.1	1 E-158	790/856
Eukaryota	Fungi	Nectria haematococca mpVI 77-13-4	EEU33401.1	1 E-158	824/856
Eukaryota	Fungi	Chaetomium globosum CBS 148.51	XP_001220280.1	1 E-158	822/856
Eukaryota	Fungi	Nectria haematococca mpVI 77-13-4	EEU46774.1	1 E-157	796/856
Bacteria	Proteobacteria	Agrobacterium radiobacter K84	YP_002542320.1	1 E-156	790/856
Bacteria	Proteobacteria	Agrobacterium tumefaciens	P27034.1	1 E-155	790/856
Bacteria	Proteobacteria	Rhizobium sp. NGR234	YP_002825217.1	1 E-153	806/856
Eukaryota	Fungi	Nectria haematococca mpVI 77-13-4	EEU37960.1	1 E-153	812/856
Bacteria	Proteobacteria	Agrobacterium radiobacter K84	YP_002540308.1	1 E-152	789/856
Eukaryota	Fungi	Podospora anserina DSM 980	XP_001908543.1	1 E-152	801/856
Bacteria	Proteobacteria	Rhizobium etli CFN 42	YP_473090.1	1 E-152	790/856
Eukaryota	Fungi	Penicillium chrysogenum Wisconsin 54-1255	XP_002561135.1	1 E-151	840/856
Bacteria	Proteobacteria	Rhizobium etli CIAT 652	YP_001984328.1	1 E-151	786/856
Bacteria	Proteobacteria	Rhizobium etli CFN 42	NP_659994.1	1 E-151	786/856
Eukaryota	Fungi	Gibberella zeae PH-1	XP_383586.1	1 E-150	795/856
Eukaryota	Fungi	Neurospora crassa OR74A	XP_965034.2	1 E-150	808/856
Eukaryota	Fungi	Verticillium albo-atrum VaMs.102	EEY17726.1	1 E-150	808/856
Bacteria	Proteobacteria	Rhizobium leguminosarum bv. viciae	YP_764854.1	1 E-149	790/856
Bacteria	Proteobacteria	Rhizobium etli CIAT 652	YP_001985325.1	1 E-148	790/856
Bacteria	Proteobacteria	Rhizobium leguminosarum bv. trifolii	YP_002278768.1	1 E-148	790/856
Eukaryota	Fungi	Coprinopsis cinerea okayama7#130	XP_001837410.1	1 E-147	731/856
Eukaryota	Fungi	Aspergillus nidulans FGSC A4	XP_658316.1	1 E-146	809/856
Eukaryota	Fungi	Aspergillus terreus NIH2624	XP_001210243.1	1 E-144	851/856
Eukaryota	Fungi	Nectria haematococca mpVI 77-13-4	EEU48841.1	1 E-143	808/856
Bacteria	Proteobacteria	Oceanicola granulosus HTCC2516	ZP_01157344.1	1 E-142	772/856
Eukaryota	Fungi	Gibberella zeae PH-1	XP_380943.1	1 E-139	808/856
Eukaryota	Fungi	Nectria haematococca mpVI 77-13-4	EEU36342.1	1 E-137	835/856
Eukaryota	Fungi	Nectria haematococca mpVI 77-13-4	EEU38431.1	1 E-135	783/856
Eukaryota	Fungi	Nectria haematococca mpVI 77-13-4	EEU34937.1	1 E-134	803/856
Eukaryota	Fungi	Gibberella zeae PH-1	XP_383315.1	1 E-134	795/856
Bacteria	Proteobacteria	Leptothrix cholodnii SP-6	YP_001793346.1	1 E-133	811/856
Eukaryota	Fungi	Pyrenophora tritici-repentis Pt-1C-BFP	XP_001933240.1	1 E-131	774/856
Eukaryota	Fungi	Nectria haematococca mpVI 77-13-4	EEU35586.1	1 E-131	851/856
Eukaryota	Fungi	Nectria haematococca mpVI 77-13-4	EEU48977.1	1 E-131	827/856
Eukaryota	Fungi	Nectria haematococca mpVI 77-13-4	EEU35660.1	1 E-130	841/856
Eukaryota	Fungi	Nectria haematococca mpVI 77-13-4	EEU36336.1	1 E-130	808/856
Eukaryota	Fungi	Nectria haematococca mpVI 77-13-4	EEU37082.1	1 E-127	768/856
Eukaryota	Fungi	Gibberella zeae PH-1	XP_383783.1	1 E-127	800/856
Eukaryota	Fungi	Verticillium albo-atrum VaMs.102	EEY21449.1	1 E-127	813/856
Eukaryota	Fungi	Neosartorya fischeri NRRL 181	XP_001262639.1	1 E-126	825/856
Eukaryota	Fungi	Aspergillus flavus NRRL3357	XP_002382325.1	1 E-124	840/856
Eukaryota	Fungi	Neosartorya fischeri NRRL 181	XP_001258338.1	1 E-124	834/856
Eukaryota	Fungi	Aspergillus oryzae RIB40	XP_001822833.1	1 E-123	828/856
Eukaryota	Fungi	Phaeosphaeria nodorum SN15	XP_001794177.1	1 E-121	771/856
Eukaryota	Fungi	Gibberella zeae PH-1	XP_391357.1	1 E-120	825/856
Eukaryota	Fungi	Verticillium albo-atrum VaMs.102	EEY22936.1	1 E-120	795/856
Bacteria	Proteobacteria	Hyphomonas neptunium ATCC 15444	YP_761141.1	1 E-116	793/856
Bacteria	Actinobacteria	Streptomyces sp. C	ZP_05506390.1	1 E-116	789/856
Eukaryota	Fungi	Penicillium chrysogenum Wisconsin 54-1255	XP_002566312.1	1 E-115	817/856
Bacteria	Bacteroidetes	Bacteroides fragilis NCTC 9343	YP_210555.1	1 E-115	829/856
Bacteria	Bacteroidetes	Bacteroides sp. 3_2_5	ZP_04841187.1	1 E-115	829/856
Bacteria	Bacteroidetes	Bacteroides fragilis YCH46	YP_098224.1	1 E-114	829/856
Bacteria	Actinobacteria	Streptomyces hygroscopicus ATCC 53653	ZP_05518125.1	1 E-114	823/856
Bacteria	Actinobacteria	Streptomyces scabiei 87.22	CBG73416.1	1 E-113	789/856
Eukaryota	Fungi	Aspergillus niger CBS 513.88	XP_001391025.1	1 E-113	824/856
Bacteria	Actinobacteria	Streptomyces sp. SPB78	ZP_05486605.1	1 E-113	791/856
Bacteria	Bacteroidetes	Bacteroides sp. D2	ZP_05759495.1	1 E-113	773/856
Bacteria	Actinobacteria	Stackebrandtia nassauensis DSM 44728	ZP_04484563.1	1 E-112	791/856

Bacteria	Actinobacteria	Streptomyces viridochromogenes DSM 40736	ZP_05531194.1	1 E-112	788/856
Bacteria	Bacteroidetes	Bacteroides fragilis 3_1_12	ZP_05279876.1	1 E-112	829/856
Bacteria	Proteobacteria	Saccharophagus degradans 2-40	YP_528146.1	1 E-111	785/856
Bacteria	Bacteroidetes	Bacteroides finegoldii DSM 17565	ZP_05413641.1	1 E-111	789/856
Bacteria	Actinobacteria	Streptomyces albus J1074	ZP_04701787.1	1 E-111	796/856
Bacteria	Actinobacteria	Streptomyces sviceus ATCC 29083	ZP_05020748.1	1 E-111	761/856
Bacteria	Bacteroidetes	Bacteroides sp. D2	ZP_05759671.1	1 E-111	754/856
Bacteria	Acidobacteria	Candidatus Koribacter versatilis Ellin345	YP_593226.1	1 E-110	775/856
Bacteria	Proteobacteria	Shewanella piezotolerans WP3	YP_002310990.1	1 E-109	791/856
Bacteria	Bacteroidetes	Bacteroides fragilis NCTC 9343	YP_210015.1	1 E-108	772/856
Bacteria	Actinobacteria	Clavibacter michiganensis subsp. sepedonicus	YP_001709426.1	1 E-108	804/856
Bacteria	Proteobacteria	Sorangium cellulosum 'So ce	YP_001617546.1	1 E-107	794/856
Bacteria	Actinobacteria	Streptomyces sp. SPB74	ZP_04991290.1	1 E-107	792/856
Bacteria	Bacteroidetes	Bacteroides sp. 3_2_5	ZP_04841779.1	1 E-107	772/856
Bacteria	Bacteroidetes	Bacteroides fragilis YCH46	YP_097613.1	1 E-107	772/856
Bacteria	Bacteroidetes	Bacteroides cellulosilyticus DSM 14838	ZP_03677511.1	1 E-107	753/856
Bacteria	Proteobacteria	Colwellia psychrerythraea 34H	YP_269097.1	1 E-107	800/856
Bacteria	Proteobacteria	Shewanella frigidimarina NCIMB 400	YP_749998.1	1 E-106	803/856
Bacteria	Actinobacteria	Clavibacter michiganensis subsp. michiganensis	YP_001221782.1	1 E-105	804/856
Bacteria	Actinobacteria	Streptomyces ambofaciens ATCC 23877	CAJ88068.1	1 E-105	788/856
Bacteria	Proteobacteria	Shewanella woodyi ATCC 51908	YP_001761424.1	1 E-103	790/856
Eukaryota	Fungi	Gibberella zeae PH-1	XP_384887.1	1 E-102	802/856
Bacteria	Proteobacteria	Colwellia psychrerythraea 34H	YP_270360.1	1 E-102	806/856
Bacteria	Actinobacteria	Saccharopolyspora erythraea NRRL 2338	YP_001103532.1	1 E-102	777/856
Bacteria	Bacteroidetes	Bacteroides cellulosilyticus DSM 14838	ZP_03677510.1	1 E-101	777/856
Bacteria	Actinobacteria	Arthrobacter chlorophenolicus A6	YP_002486350.1	4 E-98	792/856
Bacteria	Bacteroidetes	Bacteroides uniformis ATCC 8492	ZP_02073072.1	1 E-96	687/856
Bacteria	Bacteroidetes	Bacteroides thetaiotaomicron VPI-5482	NP_812212.1	1 E-96	774/856
Bacteria	Bacteroidetes	Bacteroides sp. 1_1_6	ZP_04846359.1	3 E-96	774/856
Bacteria	Actinobacteria	Streptosporangium roseum DSM 43021	ZP_04472758.1	6 E-94	788/856
Bacteria	Actinobacteria	Catenulispora acidiphila DSM 44928	YP_003116825.1	8 E-94	786/856
Bacteria	Actinobacteria	Stackebrandtia nassauensis DSM 44728	ZP_04484749.1	2 E-93	788/856
Bacteria	Bacteroidetes	Rhodothermus marinus DSM 4252	ZP_04424147.1	1 E-91	793/856
Eukaryota	Fungi	Verticillium albo-atrum VaMs.102	EEY15975.1	1 E-91	818/856
Bacteria	Actinobacteria	Acidothermus cellulolyticus 11B	YP_873417.1	3 E-90	783/856
Bacteria	Actinobacteria	Arthrobacter chlorophenolicus A6	YP_002489585.1	7 E-89	778/856
Bacteria	Proteobacteria	Pseudomonas fluorescens SBW25	YP_002873633.1	1 E-88	793/856
Bacteria	Proteobacteria	Pseudomonas syringae pv. syringae	YP_236223.1	1 E-88	818/856
Bacteria	Actinobacteria	Streptomyces narbonensis	AAM88355.1	1 E-88	757/856
Bacteria	Actinobacteria	Aeromicrobium erythreum	AAU93797.1	3 E-87	759/856
Bacteria	Proteobacteria	Pseudomonas syringae pv. oryzae	ZP_04589639.1	8 E-87	810/856
Bacteria	Proteobacteria	Pseudomonas syringae pv. tomato	NP_793101.1	2 E-86	833/856
Bacteria	Proteobacteria	Pseudomonas syringae pv. tabaci	ZP_05639421.1	5 E-86	833/856
Bacteria	Actinobacteria	Streptomyces hygroscopicus ATCC 53653	ZP_05519377.1	6 E-86	734/856
Bacteria	Proteobacteria	Pseudomonas syringae pv. syringae	YP_236107.1	6 E-85	823/856
Bacteria	Actinobacteria	Streptomyces rochei	NP_851452.1	3 E-84	755/856
Bacteria	Actinobacteria	Streptomyces venezuelae	ACR54627.1	3 E-84	752/856
Bacteria	Proteobacteria	Pseudomonas syringae pv. tabaci	ZP_05639217.1	2 E-83	823/856
Bacteria	Proteobacteria	Pseudomonas syringae pv. tomato	NP_792960.1	5 E-83	823/856
Bacteria	Actinobacteria	Saccharopolyspora erythraea NRRL 2338	YP_001102999.1	7 E-83	756/856
Bacteria	Proteobacteria	Pseudomonas syringae pv. phaseolicola	YP_274417.1	2 E-82	816/856
Bacteria	Proteobacteria	Pseudomonas syringae pv. tomato	ZP_03397949.1	3 E-82	823/856
Bacteria	Actinobacteria	Streptomyces venezuelae	AAC68679.1	1 E-81	732/856
Bacteria	Actinobacteria	Cellulomonas biazotea	AAC38196.1	4 E-81	809/856
Bacteria	Proteobacteria	Pseudomonas putida W619	YP_001749054.1	9 E-80	812/856
Bacteria	Proteobacteria	Pseudomonas syringae pv. syringae	YP_234867.1	3 E-79	801/856
Bacteria	Proteobacteria	Pseudomonas syringae pv. tabaci	ZP_05637467.1	3 E-78	766/856
Bacteria	Proteobacteria	Pseudomonas syringae pv. phaseolicola	YP_273043.1	6 E-78	785/856

Archaea	Crenarchaeota	Caldivirga maquilingensis IC-167	YP_001541034.1	7 E-77	728/856
Bacteria	Proteobacteria	Pseudomonas syringae pv. tomato	NP_793468.1	2 E-74	725/856
Bacteria	Proteobacteria	Pseudomonas syringae pv. tomato	ZP_03397007.1	2 E-73	725/856
Bacteria	Bacteroidetes	Chryseobacterium gleum ATCC 35910	ZP_03852245.1	4 E-71	759/856
Bacteria	Actinobacteria	Streptomyces ambofaciens ATCC 23877	CAJ89978.1	6 E-71	737/856

AFUA_6G11920

Eukaryota	Fungi	Aspergillus fumigatus Af293	XP_751072.1	0.0	538/538
Eukaryota	Fungi	Neosartorya fischeri NRRL 181	XP_001258307.1	0.0	522/538
Eukaryota	Fungi	Aspergillus nidulans FGSC A4	XP_681135.1	0.0	523/538
Eukaryota	Fungi	Neosartorya fischeri NRRL 181	XP_001258151.1	1 E-172	504/538
Eukaryota	Fungi	Gibberella zeae PH-1	XP_384096.1	1 E-167	516/538
Eukaryota	Fungi	Nectria haematococca mpVI 77-13-4	EEU38011.1	1 E-166	499/538
Eukaryota	Fungi	Verticillium albo-atrum VaMs.102	EEY23479.1	1 E-165	512/538
Eukaryota	Fungi	Aspergillus terreus NIH2624	XP_001216993.1	1 E-162	459/538
Eukaryota	Fungi	Phaeosphaeria nodorum SN15	XP_001800910.1	1 E-159	489/538
Eukaryota	Fungi	Nectria haematococca mpVI 77-13-4	EEU41986.1	1 E-158	511/538
Eukaryota	Fungi	Magnaporthe grisea 70-15	XP_367940.1	1 E-153	504/538
Eukaryota	Fungi	Penicillium chrysogenum Wisconsin 54-1255	XP_002565374.1	1 E-108	509/538
Eukaryota	Fungi	Sclerotinia sclerotiorum 1980 UF-70	XP_001590658.1	1 E-106	505/538
Eukaryota	Fungi	Neurospora crassa OR74A	XP_965499.1	1 E-103	517/538
Eukaryota	Fungi	Sclerotinia sclerotiorum 1980 UF-70	XP_001597606.1	1 E-102	487/538
Eukaryota	Fungi	Talaromyces stipitatus ATCC 10500	XP_002480263.1	1 E-96	483/538
Eukaryota	Fungi	Penicillium marneffei ATCC 18224	XP_002143899.1	3 E-96	502/538
Eukaryota	Fungi	Ajellomyces capsulatus G186AR	EEH07735.1	7 E-96	518/538
Eukaryota	Fungi	Ajellomyces capsulatus NAm1	XP_001537973.1	3 E-89	515/538
Eukaryota	Fungi	Pichia guilliermondii ATCC 6260	EDK40923.2	4 E-88	492/538
Eukaryota	Fungi	Botryotinia fuckeliana B05.10	XP_001558956.1	5 E-88	443/538
Eukaryota	Fungi	Pichia guilliermondii ATCC 6260	XP_001483066.1	8 E-88	492/538
Eukaryota	Fungi	Gibberella zeae PH-1	XP_384720.1	1 E-87	520/538
Eukaryota	Fungi	Microsporum canis CBS 113480	EEQ32036.1	3 E-87	477/538
Eukaryota	Fungi	Paracoccidioides brasiliensis Pb03;	EEH17194.1	2 E-84	482/538
Eukaryota	Fungi	Paracoccidioides brasiliensis Pb18;	EEH49168.1	5 E-84	453/538
Eukaryota	Fungi	Botryotinia fuckeliana B05.10	XP_001552224.1	5 E-84	450/538
Eukaryota	Fungi	Talaromyces stipitatus ATCC 10500	XP_002340589.1	2 E-82	454/538
Eukaryota	Fungi	Penicillium marneffei ATCC 18224	XP_002152585.1	4 E-80	521/538
Eukaryota	Fungi	Aspergillus terreus NIH2624	XP_001208475.1	3 E-79	519/538
Eukaryota	Fungi	Nectria haematococca mpVI 77-13-4	EEU35698.1	1 E-78	514/538
Eukaryota	Fungi	Ajellomyces dermatitidis SLH14081	XP_002621523.1	2 E-77	508/538
Eukaryota	Fungi	Aspergillus terreus NIH2624	XP_001211059.1	3 E-77	519/538
Eukaryota	Fungi	Talaromyces stipitatus ATCC 10500	XP_002481415.1	9 E-77	481/538
Eukaryota	Fungi	Penicillium chrysogenum Wisconsin 54-1255	XP_002561168.1	1 E-76	486/538
Eukaryota	Fungi	Coccidioides posadasii C735 delta	EER24069.1	1 E-76	481/538
Eukaryota	Fungi	Talaromyces stipitatus ATCC 10500	XP_002486568.1	2 E-76	501/538
Eukaryota	Fungi	Aspergillus oryzae RIB40	XP_001825183.1	6 E-76	517/538
Eukaryota	Fungi	Penicillium chrysogenum Wisconsin 54-1255	XP_002564803.1	6 E-76	522/538
Eukaryota	Fungi	Nectria haematococca mpVI 77-13-4	EEU35935.1	8 E-76	487/538
Eukaryota	Fungi	Aspergillus niger CBS 513.88	XP_001392915.1	2 E-75	484/538
Eukaryota	Fungi	Aspergillus fumigatus A1163	EDP47448.1	2 E-75	513/538
Eukaryota	Fungi	Penicillium marneffei ATCC 18224	XP_002149441.1	3 E-75	509/538
Eukaryota	Fungi	Penicillium marneffei ATCC 18224	XP_002152905.1	5 E-75	523/538
Eukaryota	Fungi	Aspergillus clavatus NRRL 1	XP_001274989.1	6 E-75	488/538
Eukaryota	Fungi	Aspergillus fumigatus Af293	XP_746451.1	7 E-75	513/538
Eukaryota	Fungi	Aspergillus oryzae RIB40	XP_001825755.1	7 E-75	507/538
Eukaryota	Fungi	Talaromyces stipitatus ATCC 10500	XP_002484741.1	7 E-75	508/538
Eukaryota	Fungi	Nectria haematococca mpVI 77-13-4	EEU45941.1	7 E-75	484/538
Eukaryota	Fungi	Neosartorya fischeri NRRL 181	XP_001262582.1	2 E-74	505/538

Eukaryota	Fungi	Neosartorya fischeri NRRL 181	XP_001261590.1	2 E-74	513/538
Eukaryota	Fungi	Clavispora lusitaniae ATCC 42720	XP_002619013.1	2 E-74	533/538
Eukaryota	Fungi	Aspergillus terreus NIH2624	XP_001213875.1	1 E-73	487/538
Eukaryota	Fungi	Penicillium marneffei ATCC 18224	XP_002149657.1	2 E-73	486/538
Eukaryota	Fungi	Aspergillus oryzae RIB40	XP_001818705.1	3 E-73	516/538
Eukaryota	Fungi	Penicillium chrysogenum Wisconsin 54-1255	XP_002556980.1	4 E-73	517/538
Eukaryota	Fungi	Aspergillus flavus NRRL3357	XP_002380123.1	5 E-73	516/538
Eukaryota	Fungi	Aspergillus fumigatus Af293	XP_748871.1	1 E-72	524/538
Eukaryota	Fungi	Aspergillus niger CBS 513.88	XP_001400913.1	1 E-72	516/538
Eukaryota	Fungi	Gibberella zeae PH-1	XP_383009.1	3 E-71	517/538
Eukaryota	Fungi	Clavispora lusitaniae ATCC 42720	XP_002614832.1	5 E-71	508/538
Eukaryota	Fungi	Pichia guilliermondii ATCC 6260	EDK40776.2	1 E-70	528/538
Eukaryota	Fungi	Metarhizium anisopliae	ACS83541.1	1 E-70	474/538
Eukaryota	Fungi	Pichia guilliermondii ATCC 6260	XP_001482919.1	3 E-70	528/538
Eukaryota	Fungi	Clavispora lusitaniae ATCC 42720	XP_002619145.1	6 E-70	515/538
Eukaryota	Fungi	Aspergillus oryzae RIB40	XP_001817794.1	1 E-69	521/538
Eukaryota	Fungi	Aspergillus terreus NIH2624	XP_001216557.1	2 E-69	532/538
Eukaryota	Fungi	Nectria haematococca mpVI 77-13-4	EEU41684.1	2 E-69	492/538
Eukaryota	Fungi	Debaryomyces hansenii CBS767	XP_457651.1	3 E-69	499/538
Eukaryota	Fungi	Nectria haematococca mpVI 77-13-4	EEU39712.1	4 E-69	488/538
Eukaryota	Fungi	Aspergillus flavus NRRL3357	XP_002372938.1	4 E-69	528/538
Eukaryota	Fungi	Debaryomyces hansenii	CAG84640.2	1 E-68	492/538
Eukaryota	Fungi	Pichia pastoris GS115	XP_002493419.1	1 E-68	517/538
Eukaryota	Fungi	Talaromyces stipitatus ATCC 10500	XP_002484658.1	1 E-68	490/538
Eukaryota	Fungi	Cryptococcus neoformans var. neoformans	XP_571538.1	2 E-68	495/538
Eukaryota	Fungi	Debaryomyces hansenii CBS767	XP_456684.1	3 E-68	492/538
Eukaryota	Fungi	Verticillium albo-atrum VaMs.102	EEY19969.1	4 E-68	509/538
Eukaryota	Fungi	Gibberella zeae PH-1	XP_389257.1	4 E-68	473/538
Eukaryota	Fungi	Aspergillus oryzae RIB40	XP_001826733.1	4 E-68	510/538
Eukaryota	Fungi	Cryptococcus neoformans var. neoformans	XP_571470.1	6 E-68	518/538
Eukaryota	Fungi	Nectria haematococca mpVI 77-13-4	EEU34774.1	6 E-68	510/538
Eukaryota	Fungi	Ustilago maydis 521	XP_762105.1	7 E-68	491/538
Eukaryota	Fungi	Cryptococcus neoformans var. neoformans	XP_774729.1	8 E-68	495/538
Eukaryota	Fungi	Aspergillus flavus NRRL3357	XP_002385342.1	8 E-68	510/538
Eukaryota	Fungi	Nectria haematococca mpVI 77-13-4	EEU42879.1	1 E-67	489/538
Eukaryota	Fungi	Debaryomyces hansenii	CAG87563.2	1 E-67	488/538
Eukaryota	Fungi	Debaryomyces hansenii CBS767	XP_459368.1	2 E-67	488/538
Eukaryota	Fungi	Aspergillus clavatus NRRL 1	XP_001273479.1	2 E-67	518/538
Eukaryota	Fungi	Penicillium chrysogenum Wisconsin 54-1255	XP_002557076.1	2 E-67	481/538
Eukaryota	Fungi	Pichia guilliermondii ATCC 6260	EDK39847.2	4 E-67	536/538
Eukaryota	Fungi	Cryptococcus neoformans var. neoformans	XP_568523.1	4 E-67	524/538
Eukaryota	Fungi	Pichia guilliermondii ATCC 6260	XP_001484564.1	7 E-67	536/538
Eukaryota	Fungi	Ustilago maydis 521	XP_762119.1	9 E-67	514/538
Eukaryota	Fungi	Pichia guilliermondii ATCC 6260	EDK39853.2	1 E-66	489/538
Eukaryota	Fungi	Talaromyces stipitatus ATCC 10500	XP_002486635.1	1 E-66	524/538
Eukaryota	Fungi	Aspergillus fumigatus A1163	EDP48478.1	2 E-66	451/538
Eukaryota	Fungi	Pyrenophora tritici-repentis Pt-1C-BFP	XP_001934063.1	2 E-66	513/538
Eukaryota	Fungi	Pichia guilliermondii ATCC 6260	XP_001483222.1	2 E-66	489/538
Eukaryota	Fungi	Nectria haematococca mpVI 77-13-4	EEU36007.1	3 E-66	507/538
Eukaryota	Fungi	Talaromyces stipitatus ATCC 10500	XP_002486121.1	4 E-66	499/538
Eukaryota	Fungi	Yarrowia lipolytica CLIB122	XP_500051.2	4 E-66	498/538
Eukaryota	Fungi	Sclerotinia sclerotiorum 1980 UF-70	XP_001588449.1	4 E-66	485/538
Eukaryota	Fungi	Aspergillus nidulans FGSC A4	CBF83195.1	7 E-66	508/538
Eukaryota	Fungi	Pichia guilliermondii ATCC 6260	XP_001485969.1	7 E-66	529/538
Eukaryota	Fungi	Aspergillus terreus NIH2624	XP_001208453.1	7 E-66	517/538
Eukaryota	Fungi	Aspergillus clavatus NRRL 1	XP_001276758.1	8 E-66	489/538
Eukaryota	Fungi	Verticillium albo-atrum VaMs.102	EEY17428.1	8 E-66	458/538
Eukaryota	Fungi	Candida albicans SC5314	XP_722051.1	1 E-65	522/538

Eukaryota	Fungi	Pichia guilliermondii ATCC 6260	EDK37542.2	2 E-65	529/538
Eukaryota	Fungi	Lodderomyces elongisporus NRRL YB-4239	XP_001526533.1	2 E-65	490/538
Eukaryota	Fungi	Aspergillus nidulans FGSC A4	XP_658317.1	2 E-65	474/538
Eukaryota	Fungi	Pichia stipitis CBS 6054	XP_001385023.1	2 E-65	524/538
Eukaryota	Fungi	Botryotinia fuckeliana B05.10	XP_001555589.1	3 E-65	519/538
Eukaryota	Fungi	Talaromyces stipitatus ATCC 10500	XP_002486653.1	4 E-65	489/538
Eukaryota	Fungi	Talaromyces stipitatus ATCC 10500	XP_002487473.1	4 E-65	511/538
Eukaryota	Fungi	Pyrenophora tritici-repentis Pt-1C-BFP	XP_001932666.1	6 E-65	550/538
Eukaryota	Fungi	Neosartorya fischeri NRRL 181	XP_001262269.1	6 E-65	489/538
Eukaryota	Fungi	Gibberella zeae PH-1	XP_388975.1	6 E-65	512/538
Eukaryota	Fungi	Aspergillus fumigatus Af293	XP_747524.1	7 E-65	489/538
Eukaryota	Fungi	Cryptococcus neoformans var. neoformans	XP_571460.1	9 E-65	515/538
Eukaryota	Fungi	Pyrenophora tritici-repentis Pt-1C-BFP	XP_001936011.1	1 E-64	517/538
Eukaryota	Fungi	Nectria haematococca mpVI 77-13-4	EEU41157.1	1 E-64	473/538
Eukaryota	Fungi	Kluyveromyces lactis	CAB46745.1	2 E-64	487/538
Eukaryota	Fungi	Phaeosphaeria nodorum SN15	XP_001800136.1	4 E-64	470/538
Eukaryota	Fungi	Kluyveromyces lactis NRRL Y-1140	XP_453082.1	4 E-64	529/538
Eukaryota	Fungi	Talaromyces stipitatus ATCC 10500	XP_002483387.1	4 E-64	527/538
Eukaryota	Fungi	Pyrenophora tritici-repentis Pt-1C-BFP	XP_001939030.1	4 E-64	490/538
Eukaryota	Fungi	Kluyveromyces lactis NRRL Y-1140	XP_451541.1	5 E-64	529/538
Eukaryota	Fungi	Pichia stipitis CBS 6054	XP_001382383.1	8 E-64	524/538
Eukaryota	Fungi	Gibberella zeae PH-1	XP_391326.1	1 E-63	527/538
Eukaryota	Fungi	Nectria haematococca mpVI 77-13-4	EEU45744.1	1 E-63	481/538
Eukaryota	Fungi	Nectria haematococca mpVI 77-13-4	EEU42327.1	2 E-63	517/538
Eukaryota	Fungi	Coccidioides immitis RS;	XP_001247112.1	2 E-63	486/538
Eukaryota	Fungi	Penicillium marneffei ATCC 18224	XP_002149573.1	3 E-63	490/538
Eukaryota	Fungi	Gibberella zeae PH-1	XP_383465.1	3 E-63	517/538
Eukaryota	Fungi	Penicillium chrysogenum Wisconsin 54-1255	XP_002560868.1	3 E-63	514/538
Eukaryota	Fungi	Yarrowia lipolytica CLIB122	XP_500343.1	3 E-63	460/538
Eukaryota	Fungi	Coccidioides immitis RS;	XP_001246350.1	4 E-63	492/538
Eukaryota	Fungi	Chaetomium globosum CBS 148.51	XP_001227350.1	4 E-63	460/538
Eukaryota	Fungi	Gibberella zeae PH-1	XP_383545.1	4 E-63	513/538
Eukaryota	Fungi	Pichia guilliermondii ATCC 6260	EDK39837.2	5 E-63	496/538
Eukaryota	Fungi	Nectria haematococca mpVI 77-13-4	EEU43592.1	6 E-63	522/538
Eukaryota	Fungi	Nectria haematococca mpVI 77-13-4	EEU35439.1	7 E-63	519/538
Eukaryota	Fungi	Pichia stipitis CBS 6054	XP_001385456.1	8 E-63	502/538
Eukaryota	Fungi	Penicillium marneffei ATCC 18224	XP_002147060.1	1 E-62	491/538
Eukaryota	Fungi	Penicillium chrysogenum Wisconsin 54-1255	XP_002557389.1	1 E-62	512/538
Eukaryota	Fungi	Aspergillus nidulans FGSC A4	XP_661119.1	2 E-62	496/538
Eukaryota	Fungi	Cryptococcus neoformans var. neoformans	XP_571466.1	2 E-62	516/538
Eukaryota	Fungi	Penicillium chrysogenum Wisconsin 54-1255	XP_002563749.1	2 E-62	489/538
Eukaryota	Fungi	Talaromyces stipitatus ATCC 10500	XP_002342016.1	2 E-62	514/538
Eukaryota	Fungi	Pichia angusta	AAX92669.1	2 E-62	495/538
Eukaryota	Fungi	Magnaporthe grisea 70-15	XP_364762.2	2 E-62	521/538
Eukaryota	Fungi	Magnaporthe grisea 70-15	XP_001521916.1	2 E-62	521/538
Eukaryota	Fungi	Coccidioides posadasii C735 delta	EER24646.1	2 E-62	492/538
Eukaryota	Fungi	Magnaporthe grisea 70-15	XP_366300.1	2 E-62	490/538
Eukaryota	Fungi	Gibberella zeae PH-1	XP_382468.1	3 E-62	518/538
Eukaryota	Fungi	Phaeosphaeria nodorum SN15	XP_001791785.1	4 E-62	525/538
Eukaryota	Fungi	Pichia guilliermondii ATCC 6260	XP_001484554.1	5 E-62	482/538
Eukaryota	Fungi	Magnaporthe grisea 70-15	XP_364046.1	6 E-62	459/538
Eukaryota	Fungi	Penicillium marneffei ATCC 18224	XP_002150756.1	7 E-62	520/538
Eukaryota	Fungi	Clavispora lusitaniae ATCC 42720	XP_002617485.1	7 E-62	502/538
Eukaryota	Fungi	Aspergillus terreus NIH2624	XP_001208393.1	1 E-61	482/538
Eukaryota	Fungi	Verticillium albo-atrum VaMs.102	EEY23970.1	1 E-61	504/538
Eukaryota	Fungi	Nectria haematococca mpVI 77-13-4	EEU36803.1	1 E-61	536/538
Eukaryota	Fungi	Neosartorya fischeri NRRL 181	XP_001260565.1	1 E-61	522/538
Eukaryota	Fungi	Chaetomium globosum CBS 148.51	XP_001221366.1	1 E-61	488/538

Eukaryota	Fungi	Candida dubliniensis CD36	XP_002420747.1	2 E-61	522/538
Eukaryota	Fungi	Aspergillus oryzae RIB40	XP_001826157.1	2 E-61	521/538
Eukaryota	Fungi	Microsporum canis CBS 113480	EEQ29633.1	3 E-61	527/538
Eukaryota	Fungi	Nectria haematococca mpVI 77-13-4	EEU46664.1	3 E-61	497/538
Eukaryota	Fungi	Nectria haematococca mpVI 77-13-4	EEU38771.1	3 E-61	478/538
Eukaryota	Fungi	Gibberella zeae PH-1	XP_390923.1	4 E-61	485/538
Eukaryota	Fungi	Talaromyces stipitatus ATCC 10500	XP_002485169.1	6 E-61	488/538
Eukaryota	Fungi	Pichia guilliermondii ATCC 6260	XP_001485971.1	7 E-61	517/538
Eukaryota	Fungi	Neosartorya fischeri NRRL 181	XP_001261420.1	8 E-61	526/538
Eukaryota	Fungi	Epichloe festucae	ACO52876.1	1 E-60	488/538
Eukaryota	Fungi	Neurospora crassa OR74A	XP_961779.1	1 E-60	481/538
Eukaryota	Fungi	Nectria haematococca mpVI 77-13-4	EEU41691.1	1 E-60	458/538
Eukaryota	Fungi	Aspergillus terreus NIH2624	XP_001208851.1	1 E-60	485/538
Eukaryota	Fungi	Verticillium albo-atrum VaMs.102	EEY22393.1	1 E-60	524/538
Eukaryota	Fungi	Aspergillus nidulans FGSC A4	XP_664723.1	1 E-60	503/538
Eukaryota	Fungi	Uncinocarpus reesii 1704	XP_002540714.1	2 E-60	512/538
Eukaryota	Fungi	Aspergillus niger CBS 513.88	XP_001399421.1	2 E-60	520/538
Eukaryota	Fungi	Gibberella zeae PH-1	XP_389543.1	2 E-60	512/538
Eukaryota	Fungi	Nectria haematococca mpVI 77-13-4	EEU45643.1	3 E-60	513/538
Eukaryota	Fungi	Aspergillus clavatus NRRL 1	XP_001275385.1	4 E-60	485/538
Eukaryota	Fungi	Candida tropicalis MYA-3404	XP_002546603.1	5 E-60	525/538
Eukaryota	Fungi	Aspergillus fumigatus Af293	XP_746441.1	5 E-60	487/538
Eukaryota	Fungi	Aspergillus fumigatus Af293	XP_755424.1	6 E-60	522/538
Eukaryota	Fungi	Verticillium albo-atrum VaMs.102	EEY23489.1	1 E-59	521/538
Eukaryota	Fungi	Candida tropicalis MYA-3404	XP_002546606.1	1 E-59	528/538
Eukaryota	Fungi	Nectria haematococca mpVI 77-13-4	EEU35467.1	1 E-59	521/538
Eukaryota	Fungi	Penicillium marneffei ATCC 18224	XP_002145428.1	2 E-59	527/538
Eukaryota	Fungi	Nectria haematococca mpVI 77-13-4	EEU39532.1	2 E-59	532/538
Eukaryota	Fungi	Candida tropicalis MYA-3404	XP_002546622.1	2 E-59	526/538
Eukaryota	Fungi	Chaetomium globosum CBS 148.51	XP_001226051.1	3 E-59	517/538
Eukaryota	Fungi	Paracoccidioides brasiliensis Pb01;	EEH41847.1	4 E-59	518/538
Eukaryota	Fungi	Magnaporthe grisea 70-15	XP_369523.2	4 E-59	457/538
Eukaryota	Fungi	Nectria haematococca mpVI 77-13-4	EEU35933.1	5 E-59	481/538
Eukaryota	Fungi	Pichia stipitis CBS 6054	XP_001385693.1	7 E-59	524/538
Eukaryota	Fungi	Gibberella zeae PH-1	XP_382534.1	9 E-59	513/538
Eukaryota	Fungi	Lachancea thermotolerans CBS 6340	XP_002556109.1	1 E-58	522/538
Eukaryota	Fungi	Pyrenophora tritici-repentis Pt-1C-BFP	XP_001941424.1	2 E-58	505/538
Eukaryota	Fungi	Debaryomyces hansenii	CAG87534.2	2 E-58	527/538
Eukaryota	Fungi	Verticillium albo-atrum VaMs.102	EEY23935.1	2 E-58	483/538
Eukaryota	Fungi	Gibberella zeae PH-1	XP_389250.1	2 E-58	482/538
Eukaryota	Fungi	Verticillium albo-atrum VaMs.102	EEY15762.1	2 E-58	463/538
Eukaryota	Fungi	Magnaporthe grisea 70-15	XP_001403854.1	3 E-58	501/538
Eukaryota	Fungi	Aspergillus nidulans FGSC A4	XP_680613.1	3 E-58	515/538
Eukaryota	Fungi	Penicillium chrysogenum Wisconsin 54-1255	XP_002557622.1	3 E-58	521/538
Eukaryota	Fungi	Nectria haematococca mpVI 77-13-4	EEU36162.1	3 E-58	524/538
Eukaryota	Fungi	Penicillium chrysogenum Wisconsin 54-1255	XP_002566023.1	3 E-58	533/538
Eukaryota	Fungi	Gibberella zeae PH-1	XP_380210.1	3 E-58	478/538
Eukaryota	Fungi	Gibberella moniliformis	ABV60278.1	4 E-58	519/538
Eukaryota	Fungi	Debaryomyces hansenii CBS767	XP_459351.1	4 E-58	527/538
Eukaryota	Fungi	Candida tropicalis MYA-3404	XP_002546620.1	4 E-58	485/538
Eukaryota	Fungi	Nectria haematococca mpVI 77-13-4	EEU41652.1	4 E-58	484/538
Eukaryota	Fungi	Verticillium albo-atrum VaMs.102	EEY21539.1	4 E-58	482/538
Eukaryota	Fungi	Neurospora crassa	CAD21259.1	4 E-58	510/538
Eukaryota	Fungi	Neosartorya fischeri NRRL 181	XP_001267629.1	5 E-58	497/538
Eukaryota	Fungi	Pichia stipitis CBS 6054	XP_001385340.2	5 E-58	493/538
Eukaryota	Fungi	Aspergillus niger CBS 513.88	XP_001390889.1	8 E-58	514/538
Eukaryota	Fungi	Paracoccidioides brasiliensis Pb03;	EEH18253.1	8 E-58	530/538
Eukaryota	Fungi	Gibberella zeae PH-1	XP_380246.1	9 E-58	518/538

Eukaryota	Fungi	Aspergillus fumigatus Af293	XP_748990.1	1 E-57	518/538
Eukaryota	Fungi	Gibberella zeae PH-1	XP_390837.1	1 E-57	516/538
Eukaryota	Fungi	Pichia guilliermondii ATCC 6260	EDK37543.2	1 E-57	511/538
Eukaryota	Fungi	Penicillium chrysogenum Wisconsin 54-1255	XP_002556753.1	1 E-57	527/538
Eukaryota	Fungi	Nectria haematococca mpVI 77-13-4	EEU35919.1	2 E-57	483/538
Eukaryota	Fungi	Aspergillus terreus NIH2624	XP_001209581.1	2 E-57	522/538
Eukaryota	Fungi	Talaromyces stipitatus ATCC 10500	XP_002483600.1	2 E-57	497/538
Eukaryota	Fungi	Podospora anserina DSM 980	XP_001903151.1	3 E-57	486/538
Eukaryota	Fungi	Pichia guilliermondii ATCC 6260	XP_001485970.1	3 E-57	511/538
Eukaryota	Fungi	Aspergillus terreus NIH2624	XP_001208368.1	3 E-57	527/538
Eukaryota	Fungi	Pyrenophora tritici-repentis Pt-1C-BFP	XP_001933982.1	4 E-57	484/538
Eukaryota	Fungi	Aspergillus nidulans FGSC A4	XP_660205.1	4 E-57	524/538
Eukaryota	Fungi	Gibberella zeae PH-1	XP_384067.1	1 E-56	515/538
Eukaryota	Fungi	Aspergillus nidulans FGSC A4	XP_663521.1	1 E-56	526/538
Eukaryota	Fungi	Nectria haematococca mpVI 77-13-4	EEU43591.1	1 E-56	499/538
Eukaryota	Fungi	Neosartorya fischeri NRRL 181	XP_001266520.1	1 E-56	477/538
Eukaryota	Fungi	Phaeosphaeria nodorum SN15	XP_001791294.1	2 E-56	522/538
Eukaryota	Fungi	Botryotinia fuckeliana B05.10	XP_001555814.1	2 E-56	526/538
Eukaryota	Fungi	Magnaporthe grisea 70-15	XP_362212.1	2 E-56	522/538
Eukaryota	Fungi	Paracoccidioides brasiliensis Pb18;	EEH47173.1	2 E-56	530/538
Eukaryota	Fungi	Ajellomyces dermatitidis ER-3	EEQ91194.1	2 E-56	525/538
Eukaryota	Fungi	Pichia guilliermondii ATCC 6260	EDK40410.2	2 E-56	496/538
Eukaryota	Fungi	Ajellomyces dermatitidis SLH14081	XP_002629466.1	3 E-56	525/538
Eukaryota	Fungi	Debaryomyces hansenii CBS767	XP_456990.1	3 E-56	452/538

AFUA_6G11930

Eukaryota	Fungi	Aspergillus fumigatus Af293	XP_751073.1	0.0	745/745
Eukaryota	Fungi	Aspergillus nidulans FGSC A4	XP_681136.1	0.0	621/745

AFUA_6G13120

Eukaryota	Fungi	Aspergillus fumigatus Af293	XP_751189.2	0.0	2009/2009
Eukaryota	Fungi	Neosartorya fischeri NRRL 181	XP_001258450.1	0.0	2023/2009
Eukaryota	Fungi	Aspergillus clavatus NRRL 1	XP_001268364.1	0.0	2014/2009
Eukaryota	Fungi	Aspergillus oryzae RIB40	XP_001819618.1	0.0	2032/2009
Eukaryota	Fungi	Aspergillus flavus NRRL3357	XP_002374891.1	0.0	2032/2009
Eukaryota	Fungi	Aspergillus niger CBS 513.88	XP_001393140.1	0.0	1755/2009
Eukaryota	Fungi	Aspergillus terreus NIH2624	XP_001212522.1	0.0	1867/2009
Eukaryota	Fungi	Aspergillus nidulans FGSC A4	XP_663103.1	0.0	1786/2009
Eukaryota	Fungi	Paracoccidioides brasiliensis Pb03;	EEH22230.1	0.0	2021/2009
Eukaryota	Fungi	Paracoccidioides brasiliensis Pb18;	EEH48756.1	0.0	2023/2009
Eukaryota	Fungi	Talaromyces stipitatus ATCC 10500	XP_002481402.1	0.0	1997/2009
Eukaryota	Fungi	Penicillium chrysogenum Wisconsin 54-1255	XP_002564273.1	0.0	1922/2009
Eukaryota	Fungi	Ajellomyces dermatitidis SLH14081	XP_002626443.1	0.0	2032/2009
Eukaryota	Fungi	Paracoccidioides brasiliensis Pb01;	EEH33934.1	0.0	1988/2009
Eukaryota	Fungi	Ajellomyces dermatitidis ER-3	EEQ85028.1	0.0	2032/2009
Eukaryota	Fungi	Ajellomyces capsulatus G186AR	EEH09202.1	0.0	2029/2009
Eukaryota	Fungi	Penicillium marneffei ATCC 18224	XP_002147283.1	0.0	1976/2009
Eukaryota	Fungi	Coccidioides posadasii C735 delta	EER23438.1	0.0	1960/2009
Eukaryota	Fungi	Uncinocarpus reesii 1704	XP_002584704.1	0.0	1964/2009
Eukaryota	Fungi	Microsporum canis CBS 113480	EEQ30467.1	0.0	1999/2009
Eukaryota	Fungi	Ajellomyces capsulatus NAm1	XP_001539741.1	0.0	1756/2009
Eukaryota	Fungi	Coccidioides immitis RS;	XP_001247901.1	0.0	1934/2009
Eukaryota	Fungi	Botryotinia fuckeliana B05.10	XP_001549684.1	0.0	1958/2009
Eukaryota	Fungi	Podospora anserina DSM 980	XP_001906109.1	0.0	1901/2009

AFUA_6G13450

Eukaryota	Fungi	Aspergillus fumigatus Af293	XP_751221.1	0.0	419/419
Eukaryota	Fungi	Neosartorya fischeri NRRL 181	XP_001258485.1	0.0	431/419
Eukaryota	Fungi	Aspergillus clavatus NRRL 1	XP_001268400.1	1 E-168	373/419
Eukaryota	Fungi	Talaromyces stipitatus ATCC 10500	XP_002478076.1	1 E-154	394/419
Eukaryota	Fungi	Penicillium chrysogenum Wisconsin 54-1255	XP_002564727.1	1 E-149	350/419
Eukaryota	Fungi	Aspergillus oryzae RIB40	XP_001819649.1	1 E-149	351/419
Eukaryota	Fungi	Aspergillus oryzae	BAC55941.1	1 E-149	351/419
Eukaryota	Fungi	Aspergillus flavus NRRL3357	XP_002374859.1	1 E-148	351/419
Eukaryota	Fungi	Penicillium marneffei ATCC 18224	XP_002145809.1	1 E-148	430/419
Eukaryota	Fungi	Aspergillus niger CBS 513.88	XP_001391017.1	1 E-144	355/419
Eukaryota	Fungi	Penicillium chrysogenum Wisconsin 54-1255	XP_002564145.1	1 E-131	361/419
Eukaryota	Fungi	Phaeosphaeria nodorum SN15	XP_001792987.1	1 E-130	421/419
Eukaryota	Fungi	Pyrenophora tritici-repentis Pt-1C-BFP	XP_001940892.1	1 E-129	424/419
Eukaryota	Fungi	Sclerotinia sclerotiorum 1980 UF-70	XP_001588504.1	1 E-118	381/419
Eukaryota	Fungi	Botryotinia fuckeliana B05.10	XP_001549270.1	1 E-106	349/419
Eukaryota	Fungi	Nectria haematococca mpVI 77-13-4	EEU44066.1	1 E-106	397/419
Eukaryota	Fungi	Neurospora crassa OR74A	XP_963200.1	1 E-103	473/419
Eukaryota	Fungi	Gibberella zeae PH-1	XP_381874.1	1 E-101	408/419
Eukaryota	Fungi	Chaetomium globosum CBS 148.51	XP_001228438.1	2 E-91	433/419
Eukaryota	Fungi	Verticillium albo-atrum VaMs.102	EEY19645.1	2 E-57	429/419
Eukaryota	Fungi	Aspergillus niger CBS 513.88	XP_001393005.1	2 E-39	403/419

AFUA_6G13460

Eukaryota	Fungi	Aspergillus fumigatus Af293	XP_751222.1	0.0	814/814
Eukaryota	Fungi	Neosartorya fischeri NRRL 181	XP_001258486.1	0.0	807/814
Eukaryota	Fungi	Aspergillus clavatus NRRL 1	XP_001268401.1	0.0	827/814
Eukaryota	Fungi	Aspergillus flavus NRRL3357	XP_002385326.1	1 E-168	673/814
Eukaryota	Fungi	Aspergillus oryzae RIB40	XP_001826747.1	1 E-167	659/814
Eukaryota	Fungi	Coccidioides immitis RS;	XP_001249042.1	1 E-128	686/814
Eukaryota	Fungi	Ajellomyces capsulatus G186AR	EEH03914.1	1 E-117	730/814
Eukaryota	Fungi	Ajellomyces capsulatus NAm1	XP_001536347.1	1 E-115	730/814
Eukaryota	Fungi	Paracoccidioides brasiliensis Pb01;	EEH41228.1	1 E-105	652/814

AFUA_6G14550

Eukaryota	Fungi	Aspergillus fumigatus Af293	XP_751329.1	0.0	513/513
Eukaryota	Fungi	Neosartorya fischeri NRRL 181	XP_001258590.1	0.0	513/513
Eukaryota	Fungi	Aspergillus clavatus NRRL 1	XP_001268488.1	0.0	508/513
Eukaryota	Fungi	Aspergillus terreus NIH2624	XP_001214261.1	0.0	510/513
Eukaryota	Fungi	Penicillium chrysogenum Wisconsin 54-1255	XP_002568055.1	0.0	507/513
Eukaryota	Fungi	Aspergillus flavus NRRL3357	XP_002385200.1	0.0	510/513
Eukaryota	Fungi	Nectria haematococca mpVI 77-13-4	EEU37713.1	1 E-175	510/513
Eukaryota	Fungi	Gibberella zeae PH-1	XP_391670.1	1 E-168	505/513
Eukaryota	Fungi	Talaromyces stipitatus ATCC 10500	XP_002487578.1	1 E-153	524/513
Eukaryota	Fungi	Penicillium marneffei ATCC 18224	XP_002145011.1	1 E-152	518/513
Eukaryota	Fungi	Pyrenophora tritici-repentis Pt-1C-BFP	XP_001941096.1	1 E-135	474/513
Eukaryota	Fungi	Phaeosphaeria nodorum SN15	XP_001801241.1	1 E-120	472/513
Eukaryota	Fungi	Nectria haematococca mpVI 77-13-4	EEU40306.1	1 E-117	519/513
Eukaryota	Fungi	Nectria haematococca mpVI 77-13-4	EEU48945.1	1 E-110	502/513
Eukaryota	Fungi	Nectria haematococca mpVI 77-13-4	EEU37970.1	1 E-105	505/513
Eukaryota	Fungi	Nectria haematococca mpVI 77-13-4	EEU33410.1	2 E-96	488/513
Eukaryota	Fungi	Aspergillus clavatus NRRL 1	XP_001268538.1	3 E-92	513/513
Eukaryota	Fungi	Verticillium albo-atrum VaMs.102	EEY16600.1	2 E-80	484/513
Bacteria	Bacteroidetes	Bacteroides uniformis ATCC 8492	ZP_02068918.1	3 E-79	485/513
Bacteria	Bacteroidetes	Bacteroides cellulosilyticus DSM 14838	ZP_03680593.1	5 E-77	486/513
Bacteria	Firmicutes	Geobacillus sp. Y412MC10	YP_003243945.1	2 E-75	489/513

Bacteria	Lentisphaerae	Lentisphaera araneosa HTCC2155	ZP_01873738.1	1 E-74	495/513
Bacteria	Bacteroidetes	Bacteroides coprocola DSM 17136	ZP_03009203.1	2 E-74	484/513
Bacteria	Bacteroidetes	Bacteroides sp. D2	ZP_05759392.1	3 E-73	485/513
Bacteria	Firmicutes	Enterococcus casseliflavus EC20	ZP_05657009.1	4 E-73	480/513
Bacteria	Firmicutes	Geobacillus thermoleovorans	ABC75004.1	4 E-72	460/513
Bacteria	Bacteroidetes	Bacteroides ovatus ATCC 8483	ZP_02065668.1	2 E-71	485/513
Eukaryota	Fungi	Nectria haematococca mpVI 77-13-4	EEU36825.1	4 E-71	548/513
Bacteria	Firmicutes	Clostridium leptum DSM 753	ZP_02079531.1	6 E-71	511/513
Bacteria	Firmicutes	Geobacillus sp. Y412MC10	YP_003244866.1	9 E-71	484/513
Bacteria	Firmicutes	Blautia hansenii DSM 20583	ZP_05853403.1	5 E-70	508/513
Eukaryota	Fungi	Gibberella zeae PH-1	XP_387815.1	6 E-69	495/513
Eukaryota	Fungi	Gibberella zeae	AAV98256.1	6 E-69	495/513
Bacteria	Firmicutes	Paenibacillus sp. JDR-2	YP_003012387.1	2 E-68	487/513
Bacteria	Firmicutes	Paenibacillus sp. JDR-2	YP_003010087.1	3 E-68	488/513
Bacteria	Verrucomicrobia	bacterium Ellin514	ZP_03628838.1	1 E-67	483/513
Bacteria	Actinobacteria	Streptomyces ghanaensis ATCC 14672	ZP_04690491.1	2 E-67	427/513
Bacteria	Firmicutes	Clostridium cellulovorans 743B	ZP_04805150.1	2 E-67	493/513
Archaea	Euryarchaeota	Halorhabdus utahensis DSM 12940	YP_003130309.1	3 E-66	489/513
Bacteria	Actinobacteria	Streptomyces coelicolor A3(2)	NP_624458.1	3 E-65	486/513
Bacteria	Actinobacteria	Streptomyces lividans TK24	ZP_05528774.1	5 E-65	486/513
Eukaryota	Fungi	Magnaporthe grisea 70-15	XP_360104.1	5 E-65	511/513
Bacteria	Proteobacteria	Saccharophagus degradans 2-40	YP_527127.1	6 E-65	505/513
Bacteria	Firmicutes	Eubacterium eligens ATCC 27750	YP_002931095.1	9 E-65	422/513
Bacteria	Firmicutes	Bacillus clausii KSM-K16	YP_174647.1	2 E-64	507/513
Bacteria	Actinobacteria	Micromonospora sp. ATCC 39149	ZP_04607118.1	2 E-64	482/513
Eukaryota	Fungi	Aspergillus oryzae RIB40	XP_001822526.1	2 E-63	435/513
Bacteria	Chloroflexi	Herpetosiphon aurantiacus ATCC 23779	YP_001545284.1	3 E-63	495/513
Bacteria	Firmicutes	Clostridium papyrosolvens DSM 2782	ZP_05497548.1	1 E-62	496/513
Bacteria	uncultured	gamma proteobacterium;	CAI78695.1	1 E-61	481/513
Bacteria	Actinobacteria	Streptomyces flavogriseus ATCC 33331	ZP_05802872.1	4 E-61	492/513
Eukaryota	Fungi	Aspergillus fumigatus Af293	XP_755651.1	1 E-60	510/513
Eukaryota	Fungi	Aspergillus fumigatus A1163	EDP54823.1	2 E-60	510/513
Eukaryota	Fungi	Podospora anserina DSM 980	XP_001906093.1	2 E-60	461/513
Bacteria	Actinobacteria	Catenulispora acidiphila DSM 44928	YP_003114345.1	5 E-60	481/513
Bacteria	Firmicutes	Clostridium phytofermentans ISDg	YP_001559135.1	5 E-60	491/513
Bacteria	Firmicutes	Eubacterium siraeum DSM 15702	ZP_02421518.1	2 E-59	474/513
Bacteria	Proteobacteria	Teredinibacter turnerae T7901	YP_003075897.1	3 E-59	470/513
Bacteria	Verrucomicrobia	Opitutus terrae PB90-1	YP_001820108.1	6 E-59	453/513
Bacteria	Firmicutes	Enterococcus casseliflavus EC30	ZP_05645679.1	5 E-58	478/513
Bacteria	Firmicutes	Roseburia intestinalis L1-82	ZP_04744873.2	7 E-58	499/513
Bacteria	Firmicutes	Oceanobacillus iheyensis HTE831	NP_693008.1	1 E-57	500/513
Eukaryota	Fungi	Penicillium chrysogenum Wisconsin 54-1255	XP_002562741.1	2 E-57	505/513
Bacteria	Actinobacteria	Clavibacter michiganensis subsp. sepedonicus	YP_001710412.1	4 E-57	420/513
Bacteria	Bacteroidetes	Bacteroides sp. D2	ZP_05759391.1	9 E-56	479/513
Bacteria	Firmicutes	Clostridium phytofermentans ISDg	YP_001560107.1	2 E-55	508/513
Bacteria	Bacteroidetes	Spirosoma linguale DSM 74	ZP_04487090.1	2 E-55	520/513
Bacteria	Actinobacteria	Clavibacter michiganensis subsp. michiganensis	YP_001220849.1	2 E-55	422/513
Bacteria	Proteobacteria	Xanthomonas axonopodis pv. citri	NP_644529.2	3 E-55	501/513
Bacteria	Actinobacteria	Bifidobacterium dentium ATCC 27678	ZP_02917643.1	3 E-55	470/513
Bacteria	Proteobacteria	Asticcacaulis excentricus CB 48	ZP_04768707.1	3 E-55	412/513
Bacteria	Proteobacteria	Xanthomonas axonopodis pv. citri	AAM39065.1	3 E-55	501/513
Bacteria	Firmicutes	Bacillus licheniformis ATCC 14580	YP_078087.1	1 E-54	486/513
Bacteria	Proteobacteria	Xanthomonas campestris pv. vesicatoria	YP_366067.1	2 E-54	501/513
Bacteria	Bacteroidetes	Bacteroides cellulosilyticus DSM 14838	ZP_03679080.1	3 E-54	497/513
Bacteria	Proteobacteria	Saccharophagus degradans 2-40	YP_526074.1	3 E-54	419/513
Eukaryota	Fungi	Aspergillus terreus NIH2624	XP_001208657.1	6 E-54	499/513
Bacteria	Proteobacteria	Xanthomonas campestris pv. campestris	NP_636552.2	9 E-54	474/513
Bacteria	Proteobacteria	Xanthomonas campestris pv. campestris	AAM40476.1	1 E-53	474/513

Bacteria	Proteobacteria	Xanthomonas campestris pv. campestris	YP_001904566.1	1 E-53	474/513
Bacteria	Actinobacteria	Arthrobacter chlorophenolicus A6	YP_002486518.1	1 E-53	418/513
Bacteria	Bacteroidetes	Bacteroides plebeius DSM 17135	ZP_03207375.1	1 E-53	503/513
Bacteria	Proteobacteria	Xanthomonas campestris pv. vesicatoria	YP_363056.1	3 E-53	509/513
Bacteria	Proteobacteria	Xanthomonas campestris pv. campestris	NP_639444.2	6 E-53	510/513
Bacteria	Proteobacteria	Xanthomonas campestris pv. campestris	AAM43326.1	8 E-53	510/513
Bacteria	Proteobacteria	Xanthomonas axonopodis pv. citri	AAM36146.1	1 E-52	513/513
Bacteria	Proteobacteria	Xanthomonas axonopodis pv. citri	NP_641610.2	1 E-52	513/513
Bacteria	Proteobacteria	Hirschia baltica ATCC 49814	YP_003061161.1	1 E-52	428/513
Bacteria	Bacteroidetes	Bacteroides pectinophilus ATCC 43243	ZP_03462637.1	3 E-52	500/513
Bacteria	Bacteroidetes	Bacteroides intestinalis DSM 17393	ZP_03016595.1	3 E-52	500/513
Bacteria	Proteobacteria	Sphingomonas sp. SKA58	ZP_01301602.1	6 E-52	418/513
Eukaryota	Viridiplantae	Ricinus communis	XP_002536919.1	1 E-51	418/513
Bacteria	Firmicutes	Bacillus halodurans C-125	NP_244550.1	4 E-51	491/513
Bacteria	Bacteroidetes	Flavobacteriales bacterium ALC-1	ZP_02181785.1	2 E-50	514/513
Bacteria	Bacteroidetes	Bacteroides ovatus ATCC 8483	ZP_02065670.1	2 E-50	464/513
Bacteria	Firmicutes	Eubacterium eligens ATCC 27750	YP_002930428.1	5 E-50	431/513
Bacteria	Verrucomicrobia	Verrucomicrobiae bacterium DG1235	ZP_05058335.1	5 E-50	513/513
Bacteria	Actinobacteria	Streptomyces sp. C	ZP_05504678.1	2 E-49	453/513
Bacteria	Proteobacteria	Cellvibrio japonicus Ueda107	YP_001983521.1	2 E-49	460/513
Bacteria	Proteobacteria	Phenylobacterium zucineum HLK1	YP_002131301.1	3 E-48	516/513
Bacteria	Bacteroidetes	Chryseobacterium gleum ATCC 35910	ZP_03852818.1	3 E-48	509/513
Bacteria	Bacteroidetes	Bacteroides coprocola DSM 17136	ZP_03009666.1	5 E-48	507/513
Bacteria	Bacteroidetes	Flavobacteriaceae bacterium 3519-10	YP_003096243.1	7 E-48	506/513
Bacteria	Proteobacteria	Xanthomonas oryzae pv. oryzicola	ZP_02245254.1	8 E-48	503/513
Bacteria	Bacteroidetes	Bacteroides eggerthii DSM 20697	ZP_03458517.1	1 E-47	508/513
Bacteria	Bacteroidetes	Bacteroides plebeius DSM 17135	ZP_03208324.1	1 E-47	512/513
Bacteria	Bacteroidetes	Sphingobacterium spiritivorum ATCC 33861	ZP_04781266.1	1 E-47	502/513
Eukaryota	Fungi	Nectria haematococca mpVI 77-13-4	EEU42198.1	2 E-47	460/513
Bacteria	Proteobacteria	Xanthomonas oryzae pv. oryzae	YP_453194.1	2 E-47	503/513
Bacteria	Proteobacteria	Xanthomonas oryzae pv. oryzae	AAW77676.1	2 E-47	503/513
Bacteria	Proteobacteria	Xanthomonas oryzae pv. oryzae	YP_001911551.1	2 E-47	503/513
Eukaryota	Fungi	Verticillium albo-atrum VaMs.102	EEY17833.1	3 E-47	529/513
Bacteria	Proteobacteria	Pseudoalteromonas atlantica T6c	YP_660424.1	4 E-47	417/513
Bacteria	Bacteroidetes	Bacteroides ovatus ATCC 8483	ZP_02066424.1	6 E-47	508/513
Bacteria	Bacteroidetes	Bacteroides sp. D1	ZP_04545893.1	7 E-47	508/513
Bacteria	Verrucomicrobia	Opitutus terrae PB90-1	YP_001820422.1	1 E-46	511/513
Bacteria	Firmicutes	Clostridium phytofermentans ISDg	YP_001558000.1	3 E-46	498/513
Bacteria	Proteobacteria	Labrenzia aggregata IAM 12614	ZP_01550320.1	5 E-46	448/513
Bacteria	Firmicutes	Clostridium beijerinckii NCIMB 8052	YP_001312034.1	7 E-46	507/513
Bacteria	Bacteroidetes	Bacteroides sp. 2_2_4	ZP_04550252.1	5 E-45	417/513
Bacteria	Bacteroidetes	Bacteroides sp. D1	ZP_04545889.1	6 E-45	417/513
Eukaryota	Fungi	Neurospora crassa OR74A	XP_958718.1	2 E-44	506/513
Bacteria	Bacteroidetes	Bacteroides ovatus ATCC 8483	ZP_02066428.1	2 E-44	417/513
Bacteria	Firmicutes	Roseburia intestinalis L1-82	ZP_04742695.1	4 E-44	490/513
Bacteria	Bacteroidetes	Bacteroides intestinalis DSM 17393	ZP_03013017.1	4 E-44	518/513
Eukaryota	Fungi	Verticillium albo-atrum VaMs.102	EEY14997.1	4 E-44	465/513
Bacteria	Firmicutes	Enterococcus casseliflavus EC30	ZP_05646924.1	5 E-44	511/513
Bacteria	Proteobacteria	Hoeflea phototrophica DFL-43	ZP_02164691.1	7 E-44	411/513
Bacteria	Firmicutes	Bacillus clausii KSM-K16	YP_174055.1	9 E-44	417/513
Bacteria	Proteobacteria	Sinorhizobium meliloti 1021	NP_386023.1	2 E-43	513/513
Bacteria	Proteobacteria	Hirschia baltica ATCC 49814	YP_003060972.1	3 E-43	509/513
Bacteria	Bacteroidetes	Bacteroides plebeius DSM 17135	ZP_03207387.1	3 E-43	474/513
Bacteria	Proteobacteria	Reinekea blandensis MED297	ZP_01113816.1	1 E-42	413/513
Bacteria	Firmicutes	Enterococcus faecium DO	ZP_00602675.1	1 E-42	489/513
Bacteria	Bacteroidetes	Flavobacterium johnsoniae UW101	YP_001196210.1	2 E-42	509/513
Bacteria	Proteobacteria	Rhizobium sp. NGR234	YP_002826284.1	2 E-42	517/513
Bacteria	Proteobacteria	Sinorhizobium medicae WSM419	YP_001327380.1	3 E-42	513/513

Bacteria	Proteobacteria	Rhizobium leguminosarum bv. viciae	YP_768373.1	5 E-42	518/513
Bacteria	Proteobacteria	Rhizobium leguminosarum bv. trifolii	YP_002976142.1	8 E-42	518/513

AFUA_7G00160

Eukaryota	Fungi	Aspergillus fumigatus Af293	XP_746913.1	0.0	1794/1794
Eukaryota	Fungi	Neosartorya fischeri NRRL 181	XP_001262597.1	0.0	1794/1794
Eukaryota	Fungi	Microsporum canis CBS 113480	EEQ30779.1	0.0	1790/1794
Eukaryota	Fungi	Aspergillus niger CBS 513.88	XP_001394705.1	0.0	1788/1794
Eukaryota	Fungi	Aspergillus nidulans FGSC A4	XP_663604.1	0.0	1782/1794
Eukaryota	Fungi	Microsporum canis CBS 113480	EEQ31623.1	0.0	1786/1794
Eukaryota	Fungi	Aspergillus nidulans FGSC A4	XP_657754.1	0.0	1787/1794
Eukaryota	Fungi	Talaromyces stipitatus ATCC 10500	XP_002482902.1	0.0	1768/1794
Eukaryota	Fungi	Aspergillus terreus NIH2624	XP_001217072.1	0.0	1755/1794
Eukaryota	Fungi	Talaromyces stipitatus ATCC 10500	XP_002482968.1	0.0	1788/1794
Eukaryota	Fungi	Penicillium marneffei ATCC 18224	XP_002144865.1	0.0	1777/1794
Eukaryota	Fungi	Aspergillus fumigatus Af293	XP_751377.1	0.0	1761/1794
Eukaryota	Fungi	Aspergillus oryzae RIB40	XP_001823362.1	0.0	1859/1794
Eukaryota	Fungi	Neosartorya fischeri NRRL 181	XP_001266594.1	0.0	1628/1794
Eukaryota	Fungi	Pyrenophora tritici-repentis Pt-1C-BFP	XP_001933041.1	0.0	1784/1794
Eukaryota	Fungi	Neosartorya fischeri NRRL 181	XP_001266579.1	0.0	1786/1794
Eukaryota	Fungi	Aspergillus fumigatus A1163	EDP50840.1	0.0	1720/1794
Eukaryota	Fungi	Aspergillus fumigatus Af293	XP_746435.1	0.0	1765/1794
Eukaryota	Fungi	Neosartorya fischeri NRRL 181	XP_001267621.1	0.0	1767/1794
Eukaryota	Fungi	Cochliobolus heterostrophus	AAR90273.1	0.0	1794/1794
Eukaryota	Fungi	Aspergillus fumigatus A1163	EDP47078.1	0.0	1766/1794
Eukaryota	Fungi	Aspergillus terreus NIH2624	XP_001211612.1	0.0	1761/1794
Eukaryota	Fungi	Aspergillus terreus	BAB88752.1	0.0	1761/1794
Eukaryota	Fungi	Aspergillus nidulans FGSC A4	XP_664675.1	0.0	1842/1794
Eukaryota	Fungi	Penicillium marneffei ATCC 18224	XP_002149615.1	0.0	1611/1794
Eukaryota	Fungi	Cochliobolus heterostrophus	AAR90274.1	0.0	1445/1794
Eukaryota	Fungi	Botryotinia fuckeliana	AAR90250.1	0.0	1748/1794
Eukaryota	Fungi	Aspergillus clavatus NRRL 1	XP_001275038.1	0.0	1741/1794
Eukaryota	Fungi	Aspergillus flavus NRRL3357	XP_002376725.1	0.0	1739/1794
Eukaryota	Fungi	Bipolaris oryzae	BAD22832.1	0.0	1724/1794
Eukaryota	Fungi	Aspergillus oryzae RIB40	XP_001820992.1	0.0	1723/1794
Eukaryota	Fungi	Botryotinia fuckeliana B05.10	XP_001553397.1	0.0	1637/1794
Eukaryota	Fungi	Cochliobolus heterostrophus	AAR90272.1	0.0	1689/1794
Eukaryota	Fungi	Aspergillus niger CBS 513.88	XP_001402309.1	0.0	1645/1794
Eukaryota	Fungi	Botryotinia fuckeliana B05.10	XP_001554288.1	0.0	1693/1794
Eukaryota	Fungi	Sclerotinia sclerotiorum 1980 UF-70	XP_001586760.1	0.0	1719/1794
Eukaryota	Fungi	Glarea lozoyensis	AAN59953.1	0.0	1684/1794
Eukaryota	Fungi	Elsinoe fawcettii	ABU63483.1	0.0	1734/1794
Eukaryota	Fungi	Sclerotinia sclerotiorum 1980 UF-70	XP_001585805.1	0.0	1682/1794
Eukaryota	Fungi	Ceratocystis resinifera;	AAO60166.1	0.0	1698/1794
Eukaryota	Fungi	Phaeosphaeria nodorum SN15	XP_001802212.1	0.0	1707/1794
Eukaryota	Fungi	Ophiostoma piceae	ABD47522.2	0.0	1705/1794
Eukaryota	Fungi	Aspergillus terreus	BAB88688.1	0.0	1710/1794
Eukaryota	Fungi	Monascus purpureus	CAC94008.1	0.0	1596/1794
Eukaryota	Fungi	Botryotinia fuckeliana	AAR90249.1	0.0	1702/1794
Eukaryota	Fungi	Botryotinia fuckeliana B05.10	XP_001547095.1	0.0	1702/1794
Eukaryota	Fungi	Phaeosphaeria nodorum SN15	XP_001805964.1	0.0	1497/1794
Eukaryota	Fungi	Magnaporthe grisea 70-15	XP_367294.2	0.0	1590/1794
Eukaryota	Fungi	Nodulisporium sp. ATCC74245	AAD38786.1	0.0	1584/1794
Eukaryota	Fungi	Glomerella graminicola;	ACN32207.1	0.0	1546/1794
Eukaryota	Fungi	Aspergillus terreus NIH2624	XP_001210231.1	0.0	1566/1794
Eukaryota	Fungi	Colletotrichum lagenarium;	BAA18956.1	0.0	1582/1794
Eukaryota	Fungi	Penicillium chrysogenum Wisconsin 54-1255	XP_002568608.1	0.0	1708/1794

Eukaryota	Fungi	Pyrenophora tritici-repentis Pt-1C-BFP	XP_001933656.1	0.0	1541/1794
Eukaryota	Fungi	Aspergillus flavus NRRL3357	XP_002382817.1	0.0	1714/1794
Eukaryota	Fungi	Aspergillus oryzae RIB40	XP_001822700.1	0.0	1714/1794
Eukaryota	Fungi	Penicillium marneffei ATCC 18224	XP_002147717.1	0.0	1718/1794
Eukaryota	Fungi	Penicillium marneffei ATCC 18224	XP_002149119.1	0.0	1714/1794
Eukaryota	Fungi	Exophiala dermatitidis	AAD31436.3	0.0	1704/1794
Eukaryota	Fungi	Aspergillus clavatus NRRL 1	XP_001276035.1	0.0	1690/1794
Eukaryota	Fungi	Talaromyces stipitatus ATCC 10500	XP_002481882.1	0.0	1719/1794
Eukaryota	Fungi	Podospora anserina DSM 980	XP_001910795.1	0.0	1591/1794
Eukaryota	Fungi	Aspergillus niger CBS 513.88	XP_001393884.1	0.0	1598/1794
Eukaryota	Fungi	Emericella nidulans	prf1905375A	0.0	1711/1794
Eukaryota	Fungi	Emericella nidulans	Q03149.2	0.0	1711/1794
Eukaryota	Fungi	Chaetomium globosum CBS 148.51	XP_001219763.1	0.0	1710/1794
Eukaryota	Fungi	Nectria haematococca mpVI 77-13-4	EEU40203.1	0.0	1677/1794
Eukaryota	Fungi	Gibberella zeae	AAU10633.1	0.0	1707/1794
Eukaryota	Fungi	Aspergillus fumigatus	ACJ13038.1	0.0	1696/1794
Eukaryota	Fungi	Aspergillus fumigatus	ACJ13039.1	0.0	1696/1794
Eukaryota	Fungi	Aspergillus fumigatus	ACJ13034.1	0.0	1696/1794
Eukaryota	Fungi	Aspergillus fumigatus Af293	XP_756095.1	0.0	1696/1794
Eukaryota	Fungi	Aspergillus fumigatus	ACJ13035.1	0.0	1696/1794
Eukaryota	Fungi	Aspergillus fumigatus A1163	EDP55264.1	0.0	1696/1794
Eukaryota	Fungi	Xanthoria elegans	ABG91136.3	0.0	1499/1794
Eukaryota	Fungi	Nectria haematococca	AAS48892.1	0.0	1677/1794
Eukaryota	Fungi	Aspergillus fumigatus	AAC39471.1	0.0	1696/1794
Eukaryota	Fungi	Neosartorya fischeri NRRL 181	XP_001261235.1	0.0	1694/1794
Eukaryota	Fungi	Aspergillus fumigatus	CAA76740.1	0.0	1696/1794
Eukaryota	Fungi	Microsporum canis CBS 113480	EEQ32235.1	0.0	1719/1794
Eukaryota	Fungi	Aspergillus fumigatus	ACJ13036.1	0.0	1696/1794
Eukaryota	Fungi	Aspergillus flavus NRRL3357	XP_002384329.1	0.0	1674/1794
Eukaryota	Fungi	Aspergillus oryzae RIB40	XP_001827098.1	0.0	1691/1794
Eukaryota	Fungi	Nectria haematococca mpVI 77-13-4	EEU34216.1	0.0	1692/1794
Eukaryota	Fungi	Verticillium albo-atrum VaMs.102	EEY14472.1	0.0	1645/1794
Eukaryota	Fungi	Exophiala lecanii-corni	AAN75188.1	0.0	1727/1794
Eukaryota	Fungi	Sordaria macrospora	CAM35471.1	0.0	1586/1794
Eukaryota	Fungi	Aspergillus fumigatus	ACJ13037.1	0.0	1651/1794
Eukaryota	Fungi	Talaromyces stipitatus ATCC 10500	XP_002483594.1	0.0	1707/1794
Eukaryota	Fungi	Exophiala lecanii-corni	AAN74983.1	0.0	1707/1794
Eukaryota	Fungi	Talaromyces stipitatus ATCC 10500	XP_002478062.1	0.0	1702/1794
Eukaryota	Fungi	Aspergillus flavus NRRL3357	XP_002373130.1	0.0	1705/1794
Eukaryota	Fungi	Neurospora crassa OR74A	XP_960586.2	0.0	1473/1794
Eukaryota	Fungi	Aspergillus niger CBS 513.88	XP_001390425.1	0.0	1706/1794
Eukaryota	Fungi	Aspergillus oryzae RIB40	XP_001817959.1	0.0	1681/1794
Eukaryota	Fungi	Penicillium marneffei ATCC 18224	XP_002145792.1	0.0	1707/1794
Eukaryota	Fungi	Penicillium marneffei ATCC 18224	XP_002152334.1	0.0	1701/1794
Eukaryota	Fungi	Gibberella moniliformis	AAR92210.1	0.0	1686/1794
Eukaryota	Fungi	Podospora anserina DSM 980	XP_001911464.1	0.0	1665/1794
Eukaryota	Fungi	Gibberella fujikuroi	CAB92399.1	0.0	1668/1794
Eukaryota	Fungi	Gibberella moniliformis	AAR92211.1	0.0	1530/1794
Eukaryota	Fungi	Aspergillus flavus	AAS89999.1	0.0	1753/1794
Eukaryota	Fungi	Talaromyces stipitatus ATCC 10500	XP_002483004.1	0.0	1702/1794
Eukaryota	Fungi	Aspergillus terreus	BAB88689.1	0.0	1728/1794
Eukaryota	Fungi	Aspergillus nomius	AAS90047.1	0.0	1745/1794
Eukaryota	Fungi	Aspergillus oryzae	BAE71314.1	0.0	1753/1794
Eukaryota	Fungi	Cercospora nicotianae	AAT69682.1	0.0	1731/1794
Eukaryota	Fungi	Aspergillus oryzae RIB40	XP_001821511.1	0.0	1753/1794
Eukaryota	Fungi	Aspergillus sojae	AAU08792.1	0.0	1753/1794
Eukaryota	Fungi	Aspergillus sp. L	AAR32704.2	0.0	1753/1794
Eukaryota	Fungi	Aspergillus flavus NRRL3357	XP_002379951.1	0.0	1746/1794

Eukaryota	Fungi	Aspergillus parasiticus	Q12053.1	0.0	1753/1794
Eukaryota	Fungi	Podospora anserina DSM 980	XP_001911528.1	0.0	1742/1794
Eukaryota	Fungi	Aspergillus flavus	AAS90022.1	0.0	1753/1794
Eukaryota	Fungi	Microsporum canis CBS 113480	EEQ35716.1	0.0	1639/1794
Eukaryota	Fungi	Aspergillus nidulans FGSC A4	XP_681094.1	0.0	1765/1794
Eukaryota	Fungi	Coccidioides immitis RS;	XP_001241406.1	0.0	1804/1794
Eukaryota	Fungi	Emericella nidulans	Q12397.2	0.0	1765/1794
Eukaryota	Fungi	Phaeosphaeria nodorum SN15	XP_001798923.1	0.0	1480/1794
Eukaryota	Fungi	Coccidioides posadasii C735 delta	EER28351.1	0.0	1788/1794
Eukaryota	Fungi	Aspergillus ochraceoroseus	ACH72912.1	0.0	1753/1794
Eukaryota	Fungi	Mycosphaerella pini	AAZ95017.1	0.0	1632/1794
Eukaryota	Fungi	Emericella nidulans	AAA81586.1	0.0	1754/1794
Eukaryota	Fungi	Talaromyces stipitatus ATCC 10500	XP_002488697.1	0.0	1710/1794
Eukaryota	Fungi	Penicillium marneffei ATCC 18224	XP_002146110.1	0.0	1592/1794
Eukaryota	Fungi	Leptosphaeria maculans	AAS92537.1	0.0	1719/1794
Eukaryota	Fungi	Botryotinia fuckeliana	AAR90251.1	0.0	1558/1794
Eukaryota	Fungi	Hypomyces subiculosus	ACD39753.1	0.0	1696/1794
Eukaryota	Fungi	Hypomyces subiculosus	ACD39762.1	0.0	1696/1794
Eukaryota	Fungi	Penicillium marneffei ATCC 18224	XP_002151003.1	0.0	1732/1794
Eukaryota	Fungi	Chaetomium globosum CBS 148.51	XP_001225797.1	0.0	1585/1794
Eukaryota	Fungi	Gibberella zeae	ABB90282.1	0.0	1683/1794
Eukaryota	Fungi	Chaetomium chiversii	ACM42403.1	0.0	1696/1794
Eukaryota	Fungi	Aspergillus nidulans FGSC A4	XP_681178.1	0.0	1704/1794
Eukaryota	Fungi	Pochonia chlamydosporia	ACD39770.1	0.0	1602/1794
Eukaryota	Fungi	Magnaporthe grisea 70-15	XP_369003.2	0.0	1664/1794
Eukaryota	Fungi	Penicillium chrysogenum Wisconsin 54-1255	XP_002568275.1	0.0	1681/1794
Eukaryota	Fungi	Aspergillus flavus NRRL3357	XP_002377153.1	1 E-164	1590/1794
Eukaryota	Fungi	Aspergillus fumigatus A1163	EDP49937.1	1 E-162	1589/1794
Eukaryota	Fungi	Laccaria bicolor S238N-H82	XP_001876029.1	1 E-161	1619/1794
Eukaryota	Fungi	Aspergillus terreus NIH2624	XP_001210065.1	1 E-152	1661/1794
Eukaryota	Fungi	Podospora anserina DSM 980	XP_001903585.1	1 E-146	1503/1794
Eukaryota	Fungi	Talaromyces stipitatus ATCC 10500	XP_002340065.1	1 E-139	1634/1794
Eukaryota	Fungi	Aspergillus terreus NIH2624	XP_001212807.1	1 E-133	1549/1794

AFUA_7G00805

Eukaryota	Fungi	Aspergillus fumigatus Af293	XP_001481418.1	3 E-38	87/87
Eukaryota	Fungi	Neosartorya fischeri NRRL 181	XP_001262714.1	5 E-28	87/87

AFUA_7G01810

Eukaryota	Fungi	Aspergillus fumigatus Af293	XP_746751.1	0.0	864/864
Eukaryota	Fungi	Neosartorya fischeri NRRL 181	XP_001262818.1	0.0	892/864
Eukaryota	Fungi	Aspergillus clavatus NRRL 1	XP_001272366.1	0.0	846/864
Eukaryota	Fungi	Aspergillus niger CBS 513.88	XP_001395173.1	0.0	764/864
Eukaryota	Fungi	Aspergillus nidulans FGSC A4	CBF82446.1	0.0	710/864
Eukaryota	Fungi	Aspergillus nidulans FGSC A4	XP_682419.1	0.0	771/864
Eukaryota	Fungi	Penicillium chrysogenum Wisconsin 54-1255	XP_002568308.1	0.0	767/864
Eukaryota	Fungi	Talaromyces stipitatus ATCC 10500	XP_002479965.1	1 E-146	702/864
Eukaryota	Fungi	Ajellomyces dermatitidis ER-3	EEQ84300.1	1 E-138	777/864
Eukaryota	Fungi	Penicillium chrysogenum Wisconsin 54-1255	XP_002559480.1	1 E-137	703/864
Eukaryota	Fungi	Ajellomyces dermatitidis SLH14081	XP_002626743.1	1 E-137	772/864
Eukaryota	Fungi	Aspergillus terreus NIH2624	XP_001213724.1	1 E-135	700/864
Eukaryota	Fungi	Aspergillus niger CBS 513.88	XP_001397711.1	1 E-134	737/864
Eukaryota	Fungi	Aspergillus clavatus NRRL 1	XP_001271018.1	1 E-134	712/864
Eukaryota	Fungi	Neosartorya fischeri NRRL 181	XP_001263443.1	1 E-133	704/864
Eukaryota	Fungi	Aspergillus fumigatus Af293	XP_754604.1	1 E-132	704/864
Eukaryota	Fungi	Aspergillus nidulans FGSC A4	CBF76366.1	1 E-132	817/864

Eukaryota	Fungi	Aspergillus nidulans FGSC A4	XP_662576.1	1 E-126	805/864
Eukaryota	Fungi	Penicillium chrysogenum Wisconsin 54-1255	XP_002558856.1	1 E-121	715/864
Eukaryota	Fungi	Penicillium chrysogenum Wisconsin 54-1255	XP_002561750.1	1 E-118	724/864
Eukaryota	Fungi	Aspergillus clavatus NRRL 1	XP_001268672.1	1 E-115	739/864
Eukaryota	Fungi	Neosartorya fischeri NRRL 181	XP_001262225.1	1 E-106	699/864
Eukaryota	Fungi	Talaromyces stipitatus ATCC 10500	XP_002478333.1	1 E-106	720/864
Eukaryota	Fungi	Penicillium marneffei ATCC 18224	XP_002146042.1	1 E-104	712/864
Eukaryota	Fungi	Aspergillus fumigatus A1163	EDP54189.1	1 E-104	713/864
Eukaryota	Fungi	Aspergillus fumigatus Af293	XP_749668.1	1 E-104	713/864
Eukaryota	Fungi	Aspergillus clavatus NRRL 1	XP_001272666.1	1 E-103	694/864
Eukaryota	Fungi	Aspergillus nidulans FGSC A4	XP_682199.1	1 E-103	718/864
Eukaryota	Fungi	Aspergillus terreus NIH2624	XP_001215906.1	1 E-102	737/864
Eukaryota	Fungi	Neosartorya fischeri NRRL 181	XP_001260162.1	1 E-102	719/864
Eukaryota	Fungi	Aspergillus niger CBS 513.88	XP_001393575.1	1 E-101	713/864
Eukaryota	Fungi	Aspergillus nidulans FGSC A4	CBF79522.1	1 E-98	699/864
Eukaryota	Fungi	Penicillium chrysogenum Wisconsin 54-1255	XP_002564675.1	2 E-93	693/864
Eukaryota	Fungi	Aspergillus nidulans FGSC A4	CBF88873.1	8 E-84	697/864

AFUA_7G01820

Eukaryota	Fungi	Aspergillus fumigatus Af293	XP_746750.1	0.0	738/738
Eukaryota	Fungi	Neosartorya fischeri NRRL 181	XP_001262819.1	0.0	705/738
Eukaryota	Fungi	Aspergillus clavatus NRRL 1	XP_001272365.1	0.0	726/738
Eukaryota	Fungi	Aspergillus flavus NRRL3357	XP_002380657.1	0.0	691/738
Eukaryota	Fungi	Aspergillus oryzae RIB40	XP_001825460.1	0.0	668/738
Eukaryota	Fungi	Penicillium chrysogenum Wisconsin 54-1255	XP_002568307.1	0.0	709/738
Eukaryota	Fungi	Aspergillus niger CBS 513.88	XP_001395172.1	0.0	678/738
Eukaryota	Fungi	Talaromyces stipitatus ATCC 10500	XP_002479522.1	1 E-178	708/738
Eukaryota	Fungi	Talaromyces stipitatus ATCC 10500	XP_002479523.1	1 E-175	704/738
Eukaryota	Fungi	Aspergillus nidulans FGSC A4	CBF82447.1	1 E-175	680/738
Eukaryota	Fungi	Neosartorya fischeri NRRL 181	XP_001266363.1	1 E-168	646/738
Eukaryota	Fungi	Penicillium marneffei ATCC 18224	XP_002143227.1	1 E-166	712/738
Eukaryota	Fungi	Aspergillus niger CBS 513.88	XP_001402332.1	1 E-164	610/738
Eukaryota	Fungi	Aspergillus fumigatus Af293	XP_748175.2	1 E-163	609/738
Eukaryota	Fungi	Aspergillus fumigatus A1163	EDP51018.1	1 E-162	609/738
Eukaryota	Fungi	Aspergillus flavus NRRL3357	XP_002373837.1	1 E-160	594/738
Eukaryota	Fungi	Aspergillus nidulans FGSC A4	XP_682419.1	1 E-156	637/738
Eukaryota	Fungi	Aspergillus nidulans FGSC A4	CBF78857.1	1 E-154	632/738
Eukaryota	Fungi	Aspergillus clavatus NRRL 1	XP_001276375.1	1 E-153	655/738
Eukaryota	Fungi	Aspergillus nidulans FGSC A4	XP_680478.1	1 E-151	617/738
Eukaryota	Fungi	Nectria haematococca mpVI 77-13-4	EEU40043.1	5 E-21	591/738

AFUA_7G02290

Eukaryota	Fungi	Aspergillus fumigatus Af293	XP_746703.1	0.0	1080/1080
Eukaryota	Fungi	Neosartorya fischeri NRRL 181	XP_001262866.1	0.0	1078/1080
Eukaryota	Fungi	Aspergillus clavatus NRRL 1	XP_001272315.1	0.0	1048/1080
Eukaryota	Fungi	Aspergillus niger	ABF69939.1	0.0	1083/1080
Eukaryota	Fungi	Aspergillus terreus NIH2624	XP_001209227.1	0.0	1047/1080
Eukaryota	Fungi	Aspergillus flavus NRRL3357	XP_002380704.1	0.0	1047/1080
Eukaryota	Fungi	Aspergillus nidulans FGSC A4	CBF82584.1	0.0	1013/1080
Eukaryota	Fungi	Aspergillus nidulans FGSC A4	XP_682353.1	0.0	1013/1080
Eukaryota	Fungi	Aspergillus niger CBS 513.88	XP_001395122.1	0.0	939/1080
Eukaryota	Fungi	Penicillium marneffei ATCC 18224	XP_002153184.1	0.0	1103/1080
Eukaryota	Fungi	Talaromyces stipitatus ATCC 10500	XP_002488270.1	0.0	1099/1080
Eukaryota	Fungi	Ajellomyces capsulatus H143	EER44499.1	0.0	1072/1080
Eukaryota	Fungi	Ajellomyces capsulatus G186AR	EEH10853.1	0.0	1067/1080
Eukaryota	Fungi	Ajellomyces dermatitidis SLH14081	XP_002625940.1	0.0	1071/1080

Eukaryota	Fungi	Ajellomyces capsulatus NAm1	XP_001540560.1	0.0	1088/1080
Eukaryota	Fungi	Ajellomyces dermatitidis ER-3	EEQ86785.1	0.0	1071/1080
Eukaryota	Fungi	Coccidioides immitis RS;	XP_001240583.1	0.0	1057/1080
Eukaryota	Fungi	Paracoccidioides brasiliensis Pb18;	EEH48215.1	0.0	1053/1080
Eukaryota	Fungi	Paracoccidioides brasiliensis Pb01;	EEH36481.1	0.0	1098/1080
Eukaryota	Fungi	Paracoccidioides brasiliensis Pb03;	EEH18851.1	0.0	1054/1080
Eukaryota	Fungi	Coccidioides posadasii C735 delta	EER25783.1	0.0	1083/1080
Eukaryota	Fungi	Microsporum canis CBS 113480	EEQ33242.1	0.0	1042/1080
Eukaryota	Fungi	Pyrenophora tritici-repentis Pt-1C-BFP	XP_001940969.1	0.0	1000/1080
Eukaryota	Fungi	Botryotinia fuckeliana B05.10	XP_001550380.1	0.0	1019/1080
Eukaryota	Fungi	Sclerotinia sclerotiorum 1980 UF-70	XP_001592720.1	0.0	1017/1080
Eukaryota	Fungi	Chaetomium globosum CBS 148.51	XP_001220953.1	0.0	1025/1080
Eukaryota	Fungi	Magnaporthe grisea 70-15	XP_368577.1	0.0	1058/1080
Eukaryota	Fungi	Nectria haematococca mpVI 77-13-4	EEU48112.1	0.0	1012/1080
Eukaryota	Fungi	Podospora anserina DSM 980	XP_001912538.1	0.0	1030/1080
Eukaryota	Fungi	Gibberella zeae PH-1	XP_380897.1	0.0	1009/1080
Eukaryota	Fungi	Phaeosphaeria nodorum SN15	XP_001791131.1	0.0	928/1080

AFUA_7G02300

Eukaryota	Fungi	Aspergillus fumigatus Af293	XP_746702.1	1 E-144	250/250
Eukaryota	Fungi	Neosartorya fischeri NRRL 181	XP_001262867.1	1 E-138	250/250
Eukaryota	Fungi	Aspergillus clavatus NRRL 1	XP_001272314.1	4 E-90	243/250
Eukaryota	Fungi	Aspergillus oryzae RIB40	XP_001825505.1	5 E-90	246/250
Eukaryota	Fungi	Aspergillus terreus NIH2624	XP_001209227.1	1 E-77	217/250
Eukaryota	Fungi	Aspergillus nidulans FGSC A4	XP_682353.1	1 E-68	220/250
Eukaryota	Fungi	Aspergillus nidulans FGSC A4	CBF82584.1	1 E-68	220/250
Eukaryota	Fungi	Penicillium marneffei ATCC 18224	XP_002153187.1	7 E-65	206/250
Eukaryota	Fungi	Penicillium chrysogenum Wisconsin 54-1255	XP_002568207.1	5 E-58	212/250
Eukaryota	Fungi	Paracoccidioides brasiliensis Pb03;	EEH18857.1	3 E-54	236/250
Eukaryota	Fungi	Paracoccidioides brasiliensis Pb18;	EEH48208.1	5 E-54	217/250

AFUA_7G02310

Eukaryota	Fungi	Aspergillus fumigatus Af293	XP_746701.1	1 E-122	217/217
Eukaryota	Fungi	Neosartorya fischeri NRRL 181	XP_001262868.1	1 E-120	217/217
Eukaryota	Fungi	Aspergillus clavatus NRRL 1	XP_001272313.1	1 E-111	214/217
Eukaryota	Fungi	Aspergillus oryzae RIB40	XP_001825506.1	2 E-97	215/217
Eukaryota	Fungi	Aspergillus niger CBS 513.88	XP_001395120.1	6 E-97	216/217
Eukaryota	Fungi	Aspergillus nidulans FGSC A4	XP_682352.1	2 E-95	213/217
Eukaryota	Fungi	Aspergillus terreus NIH2624	XP_001209228.1	3 E-93	212/217
Eukaryota	Fungi	Penicillium chrysogenum Wisconsin 54-1255	XP_002568206.1	7 E-90	200/217
Eukaryota	Fungi	Ajellomyces dermatitidis SLH14081	XP_002622695.1	6 E-86	212/217
Eukaryota	Fungi	Ajellomyces dermatitidis ER-3	EEQ87277.1	7 E-86	212/217
Eukaryota	Fungi	Ajellomyces capsulatus NAm1	XP_001541941.1	3 E-84	214/217
Eukaryota	Fungi	Ajellomyces capsulatus G186AR	EEH09569.1	5 E-84	214/217
Eukaryota	Fungi	Talaromyces stipitatus ATCC 10500	XP_002488263.1	7 E-83	210/217
Eukaryota	Fungi	Ajellomyces capsulatus H143	EER37899.1	1 E-82	214/217
Eukaryota	Fungi	Penicillium marneffei ATCC 18224	XP_002153189.1	4 E-82	210/217
Eukaryota	Fungi	Coccidioides posadasii C735 delta	EER25785.1	4 E-81	210/217
Eukaryota	Fungi	Coccidioides immitis RS;	XP_001240581.1	9 E-81	210/217
Eukaryota	Fungi	Microsporum canis CBS 113480	EEQ33241.1	2 E-80	202/217
Eukaryota	Fungi	Paracoccidioides brasiliensis Pb01;	EEH36488.1	3 E-79	205/217
Eukaryota	Fungi	Paracoccidioides brasiliensis Pb03;	EEH18856.1	3 E-79	209/217
Eukaryota	Fungi	Paracoccidioides brasiliensis Pb18;	EEH48209.1	4 E-79	209/217
Eukaryota	Fungi	Phaeosphaeria nodorum SN15	XP_001800173.1	1 E-73	195/217
Eukaryota	Fungi	Botryotinia fuckeliana B05.10	XP_001550336.1	2 E-70	215/217
Eukaryota	Fungi	Pyrenophora tritici-repentis Pt-1C-BFP	XP_001941000.1	4 E-69	202/217

Eukaryota	Fungi	Sclerotinia sclerotiorum 1980 UF-70	XP_001592766.1	1 E-67	211/217
Eukaryota	Fungi	Chaetomium globosum CBS 148.51	XP_001220952.1	1 E-62	197/217
Eukaryota	Fungi	Gibberella zeae PH-1	XP_380898.1	3 E-62	205/217
Eukaryota	Fungi	Nectria haematococca mpVI 77-13-4	EEU48825.1	6 E-62	197/217
Eukaryota	Fungi	Yarrowia lipolytica CLIB122	XP_502459.1	2 E-61	180/217
Eukaryota	Fungi	Debaryomyces hansenii CBS767	XP_456410.1	3 E-59	182/217
Eukaryota	Fungi	Podospora anserina DSM 980	XP_001912537.1	2 E-58	177/217
Eukaryota	Fungi	Magnaporthe grisea 70-15	XP_368587.1	3 E-57	214/217
Eukaryota	Fungi	Magnaporthe grisea	AAW69317.1	9 E-57	214/217
Eukaryota	Fungi	Candida albicans SC5314	XP_722498.1	6 E-56	184/217
Eukaryota	Fungi	Pichia stipitis CBS 6054	XP_001382293.1	6 E-56	181/217
Eukaryota	Fungi	Lodderomyces elongisporus NRRL YB-4239	XP_001528616.1	1 E-55	184/217
Eukaryota	Fungi	Candida tropicalis MYA-3404	XP_002546930.1	2 E-55	184/217
Eukaryota	Fungi	Candida glabrata CBS 138	XP_446670.1	3 E-55	177/217
Eukaryota	Fungi	Pichia pastoris GS115	XP_002493386.1	2 E-54	179/217
Eukaryota	Fungi	Lachancea thermotolerans CBS 6340	XP_002555230.1	6 E-53	183/217
Eukaryota	Fungi	Clavispora lusitaniae ATCC 42720	XP_002616039.1	2 E-51	181/217
Eukaryota	Fungi	Kluyveromyces lactis NRRL Y-1140	XP_451589.1	4 E-50	174/217
Eukaryota	Fungi	Coprinopsis cinerea okayama7#130	XP_001837101.1	3 E-49	177/217
Eukaryota	Fungi	Ashbya gossypii ATCC 10895	NP_985181.1	5 E-47	175/217
Eukaryota	Fungi	Laccaria bicolor S238N-H82	XP_001877090.1	5 E-47	177/217
Eukaryota	Fungi	Saccharomyces cerevisiae	NP_013690.1	1 E-46	174/217
Eukaryota	Fungi	Saccharomyces cerevisiae RM11-1a	EDV11483.1	6 E-46	174/217
Eukaryota	Fungi	Saccharomyces cerevisiae	AAA89075.1	2 E-45	174/217
Eukaryota	Fungi	Candida glabrata CBS 138	XP_448168.1	3 E-42	174/217
Eukaryota	Metazoa	Nematostella vectensis	XP_001625135.1	5 E-41	184/217
Eukaryota	Fungi	Ustilago maydis 521	XP_760712.1	8 E-41	186/217
Eukaryota	Metazoa	Branchiostoma floridae	XP_002596474.1	6 E-38	176/217
Eukaryota	Metazoa	Caenorhabditis briggsae AF16	XP_001667822.1	4 E-36	176/217
Eukaryota	Metazoa	Caenorhabditis elegans	NP_491663.1	2 E-34	176/217
Bacteria	Actinobacteria	Streptomyces coelicolor	P52561.1	2 E-34	180/217
Bacteria	Cyanobacteria	Synechococcus sp. RS9917	ZP_01081537.1	2 E-34	174/217
Eukaryota	Metazoa	Schistosoma mansoni	XP_002576728.1	4 E-34	185/217
Bacteria	Proteobacteria	Pseudovibrio sp. JE062	ZP_05083949.1	7 E-34	180/217
Eukaryota	Metazoa	Schistosoma mansoni	XP_002576733.1	9 E-34	188/217
Bacteria	Actinobacteria	Bifidobacterium animalis subsp. lactis	ZP_02963019.1	4 E-33	174/217
Bacteria	Actinobacteria	Streptomyces coelicolor A3(2)	CAA59956.1	7 E-33	180/217
Bacteria	Proteobacteria	Ochrobactrum intermedium LMG 3301	ZP_04680753.1	7 E-33	175/217

AFUA_7G02390

Eukaryota	Fungi	Aspergillus fumigatus Af293	XP_746693.1	0.0	385/385
Eukaryota	Fungi	Neosartorya fischeri NRRL 181	XP_001262875.1	0.0	386/385
Eukaryota	Fungi	Aspergillus niger CBS 513.88	XP_001395107.1	8 E-73	363/385
Eukaryota	Fungi	Aspergillus nidulans FGSC A4	XP_682345.1	2 E-53	347/385
Eukaryota	Fungi	Penicillium chrysogenum Wisconsin 54-1255	XP_002568199.1	4 E-39	361/385

AFUA_7G02630

Eukaryota	Fungi	Aspergillus fumigatus Af293	XP_746669.1	0.0	611/611
Eukaryota	Fungi	Neosartorya fischeri NRRL 181	XP_001262894.1	0.0	645/611
Eukaryota	Fungi	Aspergillus clavatus NRRL 1	XP_001272287.1	1 E-176	606/611
Eukaryota	Fungi	Penicillium chrysogenum Wisconsin 54-1255	XP_002568178.1	1 E-152	533/611
Eukaryota	Fungi	Ajellomyces capsulatus NAm1	XP_001541449.1	1 E-148	586/611
Eukaryota	Fungi	Ajellomyces capsulatus G186AR	EEH03160.1	1 E-148	586/611
Eukaryota	Fungi	Ajellomyces capsulatus H143	EER37760.1	1 E-148	586/611
Eukaryota	Fungi	Aspergillus terreus NIH2624	XP_001209253.1	1 E-148	520/611
Eukaryota	Fungi	Ajellomyces dermatitidis SLH14081	XP_002624330.1	1 E-146	591/611

Eukaryota	Fungi	Microsporum canis CBS 113480	EEQ35522.1	1 E-143	554/611
Eukaryota	Fungi	Talaromyces stipitatus ATCC 10500	XP_002488228.1	1 E-137	621/611
Eukaryota	Fungi	Uncinocarpus reesii 1704	XP_002583215.1	1 E-134	534/611
Eukaryota	Fungi	Coccidioides posadasii C735 delta	EER25579.1	1 E-130	551/611
Eukaryota	Fungi	Penicillium marneffei ATCC 18224	XP_002153221.1	1 E-130	573/611
Eukaryota	Fungi	Pyrenophora tritici-repentis Pt-1C-BFP	XP_001934979.1	1 E-113	630/611
Eukaryota	Fungi	Magnaporthe grisea 70-15	XP_001413338.1	1 E-90	493/611

AFUA_7G05080

Eukaryota	Fungi	Aspergillus fumigatus Af293	XP_749002.1	0.0	664/664
Eukaryota	Fungi	Neosartorya fischeri NRRL 181	XP_001261442.1	0.0	550/664
Eukaryota	Fungi	Nectria haematococca mpVI 77-13-4	EEU36748.1	1 E-165	581/664
Eukaryota	Fungi	Gibberella zeae PH-1	XP_389004.1	1 E-153	574/664
Eukaryota	Fungi	Nectria haematococca mpVI 77-13-4	EEU41326.1	1 E-150	672/664
Eukaryota	Fungi	Nectria haematococca mpVI 77-13-4	EEU43806.1	1 E-137	582/664
Eukaryota	Fungi	Aspergillus nidulans FGSC A4	XP_681695.1	1 E-129	738/664
Eukaryota	Fungi	Verticillium albo-atrum VaMs.102	EEY23949.1	1 E-128	691/664
Eukaryota	Fungi	Aspergillus oryzae RIB40	XP_001823684.1	1 E-122	668/664
Eukaryota	Fungi	Verticillium albo-atrum VaMs.102	EEY17118.1	1 E-120	577/664
Eukaryota	Fungi	Aspergillus flavus NRRL3357	XP_002379116.1	1 E-118	668/664
Eukaryota	Fungi	Verticillium albo-atrum VaMs.102	EEY23416.1	1 E-105	580/664
Eukaryota	Fungi	Nectria haematococca mpVI 77-13-4	EEU34764.1	3 E-30	548/664

AFUA_7G05085

Eukaryota	Fungi	Aspergillus fumigatus Af293	XP_749001.1	0.0	700/700
Eukaryota	Fungi	Neosartorya fischeri NRRL 181	XP_001261443.1	0.0	700/700
Eukaryota	Fungi	Aspergillus nidulans FGSC A4	XP_661596.1	0.0	716/700
Eukaryota	Fungi	Aspergillus nidulans FGSC A4	XP_664693.1	0.0	680/700
Eukaryota	Fungi	Nectria haematococca mpVI 77-13-4	EEU36659.1	0.0	692/700
Eukaryota	Fungi	Magnaporthe grisea 70-15	XP_369724.2	0.0	709/700
Eukaryota	Fungi	Verticillium albo-atrum VaMs.102	EEY22853.1	0.0	680/700
Eukaryota	Fungi	Gibberella zeae PH-1	XP_389003.1	0.0	675/700
Eukaryota	Fungi	Nectria haematococca mpVI 77-13-4	EEU41151.1	0.0	686/700
Eukaryota	Fungi	Aspergillus oryzae RIB40	XP_001817401.1	0.0	688/700
Eukaryota	Fungi	Aspergillus flavus NRRL3357	XP_002372472.1	0.0	688/700
Eukaryota	Fungi	Phaeosphaeria nodorum SN15	XP_001796878.1	1 E-177	676/700
Eukaryota	Fungi	Aspergillus nidulans FGSC A4	XP_681693.1	1 E-152	674/700
Eukaryota	Fungi	Aspergillus flavus NRRL3357	XP_002379120.1	1 E-144	685/700
Eukaryota	Fungi	Aspergillus terreus NIH2624	XP_001212099.1	1 E-124	624/700
Eukaryota	Fungi	Aspergillus clavatus NRRL 1	XP_001272612.1	1 E-122	625/700
Eukaryota	Fungi	Pyrenophora tritici-repentis Pt-1C-BFP	XP_001937311.1	1 E-120	631/700
Eukaryota	Fungi	Neosartorya fischeri NRRL 181	XP_001257814.1	1 E-119	635/700
Eukaryota	Fungi	Phaeosphaeria nodorum SN15	XP_001791476.1	1 E-118	625/700
Eukaryota	Fungi	Aspergillus fumigatus Af293	XP_750590.1	1 E-118	635/700
Eukaryota	Fungi	Coprinopsis cinerea okayama7#130	XP_001839661.1	1 E-118	607/700
Eukaryota	Fungi	Aspergillus fumigatus A1163	EDP49264.1	1 E-117	635/700
Eukaryota	Fungi	Penicillium marneffei ATCC 18224	XP_002152838.1	1 E-115	644/700
Eukaryota	Fungi	Penicillium chrysogenum Wisconsin 54-1255	XP_002560193.1	1 E-115	602/700
Eukaryota	Fungi	Botryotinia fuckeliana B05.10	XP_001550831.1	1 E-111	634/700
Eukaryota	Fungi	Aspergillus oryzae RIB40	XP_001819314.1	1 E-111	614/700
Eukaryota	Fungi	Sclerotinia sclerotiorum 1980 UF-70	XP_001590219.1	1 E-110	625/700
Eukaryota	Fungi	Gibberella zeae PH-1	XP_386410.1	1 E-107	634/700
Eukaryota	Fungi	Magnaporthe grisea 70-15	XP_361589.2	1 E-106	655/700
Eukaryota	Fungi	Nectria haematococca mpVI 77-13-4	EEU44854.1	1 E-103	636/700
Eukaryota	Fungi	Cryptococcus neoformans var. neoformans	XP_569937.1	1 E-103	626/700
Eukaryota	Fungi	Cryptococcus neoformans var. neoformans	XP_776406.1	1 E-103	626/700

Eukaryota	Fungi	Neurospora crassa	CAD70965.1	1 E-101	637/700
Eukaryota	Fungi	Verticillium albo-atrum VaMs.102	EEY13984.1	7 E-98	615/700
Eukaryota	Fungi	Neurospora crassa OR74A	XP_955990.1	2 E-90	616/700
Bacteria	Firmicutes	Paenibacillus sp. JDR-2	YP_003012752.1	1 E-80	574/700
Bacteria	Proteobacteria	Sinorhizobium meliloti 1021	NP_437055.1	2 E-78	564/700
Bacteria	Firmicutes	Paenibacillus sp. oral taxon	ZP_04854297.1	7 E-78	586/700
Bacteria	Proteobacteria	Rhizobium sp. NGR234	YP_002826032.1	3 E-76	562/700
Bacteria	Proteobacteria	Agrobacterium tumefaciens str. C58	NP_357490.1	1 E-75	572/700
Bacteria	Proteobacteria	Rhizobium leguminosarum bv. viciae	YP_765688.1	9 E-75	568/700
Bacteria	Proteobacteria	Rhizobium leguminosarum bv. viciae	YP_765162.1	9 E-75	571/700
Bacteria	Proteobacteria	Rhizobium leguminosarum bv. trifolii	YP_002279250.1	1 E-74	568/700
Bacteria	Proteobacteria	Rhizobium leguminosarum bv. trifolii	YP_002978116.1	2 E-74	568/700
Bacteria	Proteobacteria	Agrobacterium radiobacter K84	YP_002546223.1	2 E-74	566/700
Bacteria	Proteobacteria	Rhizobium leguminosarum bv. trifolii	YP_002283496.1	2 E-73	565/700
Archaea	Euryarchaeota	Halomicrobium mukohataei DSM 12286	YP_003175852.1	1 E-72	572/700
Bacteria	Proteobacteria	Rhizobium etli 8C-3	ZP_03513940.1	1 E-72	565/700
Bacteria	Proteobacteria	Sinorhizobium medicae WSM419	YP_001312328.1	5 E-72	561/700
Bacteria	Proteobacteria	Rhizobium leguminosarum bv. trifolii	YP_002973222.1	3 E-71	571/700
Bacteria	Firmicutes	Enterococcus casseliflavus EC20	ZP_05655750.1	4 E-71	566/700
Bacteria	Proteobacteria	Rhizobium etli CFN 42	YP_467628.1	7 E-71	568/700
Bacteria	Proteobacteria	Agrobacterium vitis S4	YP_002539712.1	4 E-70	563/700
Bacteria	Proteobacteria	Rhizobium etli CFN 42	YP_472939.1	1 E-69	566/700
Bacteria	Proteobacteria	Rhizobium etli CIAT 652	YP_001976254.1	2 E-69	568/700
Bacteria	Firmicutes	Enterococcus casseliflavus EC30	ZP_05646134.1	6 E-69	566/700
Eukaryota	Fungi	Debaryomyces hansenii	CAG85663.2	7 E-68	592/700
Eukaryota	Fungi	Debaryomyces hansenii CBS767	XP_457649.1	1 E-67	592/700
Eukaryota	Fungi	Debaryomyces hansenii CBS767	XP_458458.1	4 E-67	564/700
Eukaryota	Fungi	Debaryomyces hansenii CBS767	XP_458511.1	7 E-67	607/700
Eukaryota	Fungi	Debaryomyces hansenii	CAG86636.2	1 E-66	600/700
Eukaryota	Fungi	Pichia stipitis CBS 6054	XP_001383581.2	2 E-66	582/700
Bacteria	Firmicutes	Enterococcus faecium 1,141,733	ZP_05667848.1	5 E-66	576/700
Bacteria	Firmicutes	Enterococcus faecium TX1330	ZP_03982626.1	1 E-65	576/700
Bacteria	Firmicutes	Enterococcus faecium Com15	ZP_05679279.1	6 E-65	576/700
Bacteria	Firmicutes	Enterococcus faecalis Fly1	ZP_05578090.1	1 E-63	570/700
Bacteria	Firmicutes	Enterococcus faecalis ATCC 4200	ZP_05474035.1	4 E-63	563/700
Bacteria	Firmicutes	Enterococcus faecalis HH22	ZP_03984972.1	6 E-63	570/700
Bacteria	Firmicutes	Enterococcus faecalis ATCC 29200	ZP_04438208.1	7 E-63	563/700
Bacteria	Firmicutes	Enterococcus faecalis DS5	ZP_05561497.1	7 E-63	570/700
Bacteria	Firmicutes	Enterococcus faecalis HIP11704	ZP_05567684.1	8 E-63	563/700
Bacteria	Firmicutes	Enterococcus faecalis V583	NP_815897.1	8 E-63	563/700
Bacteria	Firmicutes	Enterococcus faecalis V583	NP_814553.1	8 E-63	570/700
Bacteria	Firmicutes	Enterococcus faecalis T8	ZP_05559051.1	9 E-63	563/700
Bacteria	Firmicutes	Enterococcus faecalis AR01/DG	ZP_05593544.1	1 E-62	563/700
Bacteria	Firmicutes	Enterococcus faecalis T11	ZP_05595405.1	1 E-62	570/700
Bacteria	Firmicutes	Enterococcus faecalis Merz96	ZP_05564109.1	1 E-62	570/700
Bacteria	Firmicutes	Enterococcus faecalis T11	ZP_05596783.1	1 E-62	563/700
Bacteria	Firmicutes	Enterococcus faecalis T3	ZP_05502151.1	1 E-62	570/700
Bacteria	Firmicutes	Enterococcus faecalis T2	ZP_05424472.1	2 E-62	570/700
Bacteria	Firmicutes	Enterococcus faecalis TX0104	ZP_03947829.1	2 E-62	570/700
Eukaryota	Fungi	Debaryomyces hansenii	CAR65598.1	2 E-62	580/700
Bacteria	Firmicutes	Enterococcus faecalis T3	ZP_05503618.1	2 E-62	563/700
Bacteria	Firmicutes	Enterococcus faecalis CH188	ZP_05584864.1	3 E-62	563/700
Bacteria	Firmicutes	Enterococcus faecalis AR01/DG	ZP_05592371.1	4 E-62	570/700
Bacteria	Firmicutes	Enterococcus faecalis T1	ZP_05421852.1	7 E-62	570/700
Bacteria	Firmicutes	Enterococcus faecalis Merz96	ZP_05565517.1	3 E-61	563/700
Bacteria	Firmicutes	Lactobacillus brevis subsp. gravesensis	ZP_03938322.1	2 E-59	565/700

AFUA_7G05090

Eukaryota	Fungi	Aspergillus fumigatus Af293	XP_749000.1	0.0	541/541
Eukaryota	Fungi	Neosartorya fischeri NRRL 181	XP_001261444.1	0.0	518/541
Eukaryota	Fungi	Aspergillus nidulans FGSC A4	XP_661595.1	1 E-159	487/541
Eukaryota	Fungi	Gibberella zeae PH-1	XP_389005.1	1 E-143	452/541
Eukaryota	Fungi	Nectria haematococca mpVI 77-13-4	EEU35062.1	1 E-137	451/541
Eukaryota	Fungi	Magnaporthe grisea 70-15	XP_360240.1	1 E-133	478/541
Eukaryota	Fungi	Verticillium albo-atrum VaMs.102	EEY17119.1	1 E-128	467/541
Eukaryota	Fungi	Aspergillus oryzae RIB40	XP_001817403.1	1 E-124	484/541
Eukaryota	Fungi	Nectria haematococca mpVI 77-13-4	EEU39292.1	1 E-122	456/541
Eukaryota	Fungi	Nectria haematococca mpVI 77-13-4	EEU41325.1	1 E-122	460/541
Eukaryota	Fungi	Verticillium albo-atrum VaMs.102	EEY23948.1	1 E-122	484/541
Eukaryota	Fungi	Aspergillus flavus NRRL3357	XP_002379117.1	1 E-118	476/541
Eukaryota	Fungi	Aspergillus flavus NRRL3357	XP_002372474.1	1 E-117	447/541
Eukaryota	Fungi	Phaeosphaeria nodorum SN15	XP_001796880.1	1 E-103	443/541
Eukaryota	Fungi	Pyrenophora tritici-repentis Pt-1C-BFP	XP_001931748.1	3 E-98	454/541
Eukaryota	Fungi	Aspergillus niger CBS 513.88	XP_001388574.1	9 E-83	442/541
Eukaryota	Fungi	Aspergillus flavus NRRL3357	XP_002381595.1	3 E-82	451/541
Eukaryota	Fungi	Aspergillus oryzae RIB40	XP_001825598.1	5 E-80	441/541
Eukaryota	Fungi	Neosartorya fischeri NRRL 181	XP_001265520.1	3 E-75	453/541
Eukaryota	Fungi	Aspergillus fumigatus Af293	XP_749875.1	7 E-74	453/541
Eukaryota	Fungi	Nectria haematococca mpVI 77-13-4	EEU34986.1	3 E-73	434/541

AFUA_7G05100

Eukaryota	Fungi	Aspergillus fumigatus Af293	XP_748999.1	0.0	534/534
Eukaryota	Fungi	Neosartorya fischeri NRRL 181	XP_001261445.1	0.0	530/534
Eukaryota	Fungi	Aspergillus nidulans FGSC A4	XP_661594.1	0.0	518/534
Eukaryota	Fungi	Aspergillus nidulans FGSC A4	XP_664692.1	0.0	438/534
Eukaryota	Fungi	Nectria haematococca mpVI 77-13-4	EEU35096.1	0.0	501/534
Eukaryota	Fungi	Gibberella zeae PH-1	XP_380243.1	0.0	503/534
Eukaryota	Fungi	Debaryomyces hansenii	CAG87550.2	1 E-128	511/534
Eukaryota	Fungi	Debaryomyces hansenii CBS767	XP_459360.1	1 E-128	511/534
Eukaryota	Fungi	Pichia guilliermondii ATCC 6260	EDK35912.2	1 E-127	487/534
Eukaryota	Fungi	Pichia guilliermondii ATCC 6260	XP_001486633.1	1 E-126	487/534
Eukaryota	Fungi	Pichia guilliermondii ATCC 6260	EDK39028.2	1 E-117	480/534
Eukaryota	Fungi	Pichia guilliermondii ATCC 6260	XP_001485397.1	1 E-116	480/534
Eukaryota	Fungi	Pichia guilliermondii ATCC 6260	XP_001487010.1	1 E-116	492/534
Eukaryota	Fungi	Debaryomyces hansenii	CAR65725.1	1 E-116	490/534
Eukaryota	Fungi	Debaryomyces hansenii CBS767	XP_459329.1	1 E-116	492/534
Eukaryota	Fungi	Pichia stipitis CBS 6054	XP_001383911.2	1 E-115	478/534
Eukaryota	Fungi	Debaryomyces hansenii	CAG89971.2	1 E-115	483/534
Eukaryota	Fungi	Debaryomyces hansenii CBS767	XP_461825.1	1 E-115	494/534
Eukaryota	Fungi	Debaryomyces hansenii CBS767	XP_461534.1	1 E-115	483/534
Eukaryota	Fungi	Pichia guilliermondii ATCC 6260	EDK38126.2	1 E-113	483/534
Eukaryota	Fungi	Pichia guilliermondii ATCC 6260	XP_001486553.1	1 E-113	483/534
Eukaryota	Fungi	Debaryomyces hansenii	CAG90290.2	1 E-112	503/534
Eukaryota	Fungi	Penicillium chrysogenum Wisconsin 54-1255	XP_002561406.1	1 E-112	511/534
Eukaryota	Fungi	Debaryomyces hansenii CBS767	XP_461829.1	1 E-112	503/534
Eukaryota	Fungi	Nectria haematococca mpVI 77-13-4	EEU46706.1	1 E-112	508/534
Eukaryota	Fungi	Debaryomyces hansenii	CAR65604.1	1 E-110	478/534
Eukaryota	Fungi	Pichia stipitis CBS 6054	XP_001382754.1	1 E-109	486/534
Eukaryota	Fungi	Pichia stipitis CBS 6054	XP_001384653.2	1 E-108	474/534
Eukaryota	Fungi	Coprinopsis cinerea okayama7#130	XP_001832384.1	1 E-107	492/534
Eukaryota	Fungi	Verticillium albo-atrum VaMs.102	EEY20323.1	1 E-106	520/534
Eukaryota	Fungi	Clavispora lusitaniae ATCC 42720	XP_002620020.1	1 E-106	514/534
Eukaryota	Fungi	Paxillus involutus	AAT91306.1	1 E-106	478/534
Eukaryota	Fungi	Pichia stipitis CBS 6054	EAZ62850.2	1 E-106	463/534

Eukaryota	Fungi	Paxillus involutus	AAT91307.1	1 E-106	477/534
Eukaryota	Fungi	Debaryomyces hansenii CBS767	XP_462628.1	1 E-105	478/534
Eukaryota	Fungi	Pichia stipitis CBS 6054	XP_001386873.1	1 E-105	463/534
Eukaryota	Fungi	Laccaria bicolor S238N-H82	XP_001881032.1	1 E-105	469/534
Eukaryota	Fungi	Pichia stipitis CBS 6054	XP_001385684.1	1 E-105	458/534
Eukaryota	Fungi	Pichia stipitis CBS 6054	XP_001387362.1	1 E-105	493/534
Eukaryota	Fungi	Nectria haematococca mpVI 77-13-4	EEU33934.1	1 E-105	516/534
Eukaryota	Fungi	Pichia stipitis CBS 6054	XP_001387757.1	1 E-105	494/534
Eukaryota	Fungi	Candida tropicalis MYA-3404	XP_002550006.1	1 E-104	490/534
Eukaryota	Fungi	Paxillus involutus	AAT91253.1	1 E-104	477/534
Eukaryota	Fungi	Nectria haematococca mpVI 77-13-4	EEU36335.1	1 E-103	522/534
Eukaryota	Fungi	Gibberella zeae PH-1	XP_387792.1	1 E-103	512/534
Eukaryota	Fungi	Aspergillus flavus NRRL3357	XP_002378613.1	1 E-103	447/534
Eukaryota	Fungi	Clavispora lusitaniae ATCC 42720	XP_002620019.1	1 E-103	514/534
Eukaryota	Fungi	Paxillus involutus	AAT91304.1	1 E-102	482/534
Eukaryota	Fungi	Aspergillus oryzae RIB40	XP_001823255.1	1 E-102	489/534
Eukaryota	Fungi	Aspergillus terreus NIH2624	XP_001210859.1	1 E-102	484/534
Eukaryota	Fungi	Candida tropicalis MYA-3404	XP_002548331.1	1 E-102	503/534
Eukaryota	Fungi	Paxillus involutus	AAT91305.1	1 E-102	477/534
Eukaryota	Fungi	Kluyveromyces lactis NRRL Y-1140	XP_453088.1	1 E-101	533/534
Eukaryota	Fungi	Paxillus involutus	AAT91303.1	1 E-101	482/534
Eukaryota	Fungi	Nectria haematococca mpVI 77-13-4	EEU42511.1	1 E-101	508/534
Eukaryota	Fungi	Neosartorya fischeri NRRL 181	XP_001261997.1	1 E-100	446/534
Eukaryota	Fungi	Aspergillus flavus NRRL3357	XP_002374068.1	1 E-100	504/534
Eukaryota	Fungi	Coprinopsis cinerea okayama7#130	XP_001840091.1	1 E-99	480/534
Eukaryota	Fungi	Aspergillus oryzae RIB40	XP_001820343.1	2 E-99	504/534
Eukaryota	Fungi	Nectria haematococca mpVI 77-13-4	EEU33403.1	2 E-99	521/534
Eukaryota	Fungi	Aspergillus nidulans FGSC A4	XP_681381.1	5 E-99	467/534
Eukaryota	Fungi	Aspergillus niger CBS 513.88	XP_001393046.1	6 E-99	493/534
Eukaryota	Fungi	Kluyveromyces lactis NRRL Y-1140	XP_455078.1	8 E-99	531/534
Eukaryota	Fungi	Aspergillus fumigatus Af293	XP_747255.1	1 E-98	514/534
Eukaryota	Fungi	Aspergillus fumigatus A1163	EDP48858.1	2 E-98	514/534
Eukaryota	Fungi	Aspergillus oryzae RIB40	XP_001827087.1	5 E-98	493/534
Eukaryota	Fungi	Debaryomyces hansenii CBS767	XP_461828.1	5 E-98	466/534
Eukaryota	Fungi	Nectria haematococca mpVI 77-13-4	EEU42797.1	1 E-97	482/534
Eukaryota	Fungi	Gibberella zeae PH-1	XP_387866.1	4 E-97	479/534
Eukaryota	Fungi	Gibberella zeae PH-1	XP_383784.1	9 E-97	443/534
Eukaryota	Fungi	Aspergillus flavus NRRL3357	XP_002384316.1	1 E-96	493/534
Eukaryota	Fungi	Nectria haematococca mpVI 77-13-4	EEU36892.1	1 E-96	442/534
Eukaryota	Fungi	Gibberella zeae PH-1	XP_391358.1	1 E-95	467/534
Eukaryota	Fungi	Pichia guilliermondii ATCC 6260	XP_001482369.1	3 E-95	456/534
Eukaryota	Fungi	Pichia guilliermondii ATCC 6260	EDK41291.2	3 E-95	456/534
Eukaryota	Fungi	Aspergillus niger CBS 513.88	XP_001390413.1	4 E-95	511/534
Eukaryota	Fungi	Aspergillus nidulans FGSC A4	XP_660218.1	5 E-95	440/534
Eukaryota	Fungi	Laccaria bicolor S238N-H82	XP_001880629.1	4 E-94	513/534
Eukaryota	Fungi	Sclerotinia sclerotiorum 1980 UF-70	XP_001594466.1	4 E-94	471/534
Eukaryota	Fungi	Coprinopsis cinerea okayama7#130	XP_001839222.1	8 E-94	493/534
Eukaryota	Fungi	Phaeosphaeria nodorum SN15	XP_001800292.1	8 E-94	495/534
Eukaryota	Fungi	Penicillium marneffei ATCC 18224	XP_002145010.1	1 E-93	476/534
Eukaryota	Fungi	Aspergillus oryzae RIB40	XP_001821535.1	1 E-93	496/534
Eukaryota	Fungi	Talaromyces stipitatus ATCC 10500	XP_002481129.1	2 E-93	496/534
Eukaryota	Fungi	Penicillium marneffei ATCC 18224	XP_002147172.1	4 E-93	494/534
Eukaryota	Fungi	Pyrenophora tritici-repentis Pt-1C-BFP	XP_001935811.1	7 E-93	501/534
Eukaryota	Fungi	Aspergillus flavus NRRL3357	XP_002379924.1	8 E-93	496/534
Eukaryota	Fungi	Nectria haematococca mpVI 77-13-4	EEU36861.1	1 E-92	517/534
Eukaryota	Fungi	Gibberella zeae PH-1	XP_391233.1	2 E-92	509/534
Eukaryota	Fungi	Magnaporthe grisea 70-15	XP_366707.1	4 E-92	494/534
Eukaryota	Fungi	Kluyveromyces lactis NRRL Y-1140	XP_454610.1	1 E-91	474/534

Eukaryota	Fungi	Aspergillus terreus NIH2624	XP_001214260.1	1 E-91	521/534
Eukaryota	Fungi	Nectria haematococca mpVI 77-13-4	EEU33784.1	2 E-91	519/534
Eukaryota	Fungi	Talaromyces stipitatus ATCC 10500	XP_002487579.1	4 E-91	506/534
Eukaryota	Fungi	Nectria haematococca mpVI 77-13-4	EEU37335.1	8 E-91	484/534
Eukaryota	Fungi	Aspergillus niger CBS 513.88	XP_001396460.1	9 E-91	443/534
Eukaryota	Fungi	Aspergillus oryzae RIB40	XP_001820663.1	1 E-90	465/534
Eukaryota	Fungi	Aspergillus flavus NRRL3357	XP_002376381.1	3 E-90	465/534
Eukaryota	Fungi	Aspergillus flavus NRRL3357	XP_002385201.1	2 E-89	500/534
Eukaryota	Fungi	Aspergillus oryzae RIB40	XP_001826848.1	2 E-89	500/534
Eukaryota	Fungi	Aspergillus clavatus NRRL 1	XP_001268489.1	6 E-89	501/534
Eukaryota	Fungi	Cryptococcus neoformans var. neoformans	XP_772778.1	9 E-89	490/534
Eukaryota	Fungi	Gibberella zeae PH-1	XP_390861.1	1 E-88	471/534
Eukaryota	Fungi	Cryptococcus neoformans var. neoformans	XP_567678.1	1 E-88	490/534
Eukaryota	Fungi	Nectria haematococca mpVI 77-13-4	EEU36514.1	1 E-88	455/534
Eukaryota	Fungi	Nectria haematococca mpVI 77-13-4	EEU34432.1	1 E-88	485/534
Eukaryota	Fungi	Aspergillus terreus NIH2624	XP_001210244.1	1 E-88	488/534
Eukaryota	Fungi	Nectria haematococca mpVI 77-13-4	EEU45208.1	3 E-88	476/534
Eukaryota	Fungi	Aspergillus fumigatus A1163	EDP55331.1	7 E-88	501/534
Eukaryota	Fungi	Aspergillus fumigatus Af293	XP_751330.1	7 E-88	501/534
Eukaryota	Fungi	Aspergillus parasiticus	AAS66028.1	1 E-87	484/534
Eukaryota	Fungi	Neosartorya fischeri NRRL 181	XP_001258591.1	1 E-87	501/534
Eukaryota	Fungi	Aspergillus nidulans FGSC A4	XP_657617.1	2 E-87	517/534
Eukaryota	Fungi	Podospora anserina DSM 980	XP_001910685.1	2 E-87	497/534
Eukaryota	Fungi	Cryptococcus neoformans var. neoformans	XP_771814.1	4 E-87	475/534
Eukaryota	Fungi	Nectria haematococca mpVI 77-13-4	EEU39954.1	6 E-87	456/534
Eukaryota	Fungi	Aspergillus niger CBS 513.88	XP_001397926.1	7 E-87	477/534
Eukaryota	Fungi	Neosartorya fischeri NRRL 181	XP_001258602.1	9 E-87	441/534
Eukaryota	Fungi	Gibberella zeae PH-1	XP_391671.1	2 E-86	498/534
Eukaryota	Fungi	Penicillium chrysogenum Wisconsin 54-1255	XP_002561986.1	2 E-86	492/534
Eukaryota	Fungi	Aspergillus oryzae	BAC20337.1	2 E-86	503/534
Eukaryota	Fungi	Magnaporthe grisea 70-15	XP_364788.2	3 E-86	474/534
Eukaryota	Fungi	Magnaporthe grisea 70-15	XP_001521940.1	3 E-86	474/534
Eukaryota	Fungi	Gibberella zeae PH-1	XP_383482.1	1 E-85	468/534
Eukaryota	Fungi	Nectria haematococca mpVI 77-13-4	EEU35085.1	1 E-85	457/534
Eukaryota	Fungi	Gibberella zeae PH-1	XP_380263.1	2 E-85	481/534
Eukaryota	Fungi	Magnaporthe grisea 70-15	XP_001415350.1	5 E-85	530/534
Eukaryota	Fungi	Neosartorya fischeri NRRL 181	XP_001264124.1	2 E-84	491/534
Eukaryota	Fungi	Neurospora crassa OR74A	XP_963873.1	2 E-84	515/534
Eukaryota	Fungi	Penicillium chrysogenum Wisconsin 54-1255	XP_002568054.1	2 E-84	509/534
Eukaryota	Fungi	Nectria haematococca mpVI 77-13-4	EEU42194.1	3 E-84	456/534
Eukaryota	Fungi	Neosartorya fischeri NRRL 181	XP_001264000.1	4 E-84	497/534
Eukaryota	Fungi	Podospora anserina DSM 980	XP_001908539.1	8 E-84	466/534
Eukaryota	Fungi	Verticillium albo-atrum VaMs.102	EEY17151.1	1 E-83	477/534
Eukaryota	Fungi	Gibberella zeae PH-1	XP_383316.1	2 E-83	433/534
Eukaryota	Fungi	Penicillium marneffei ATCC 18224	XP_002149459.1	4 E-83	506/534
Eukaryota	Fungi	Penicillium chrysogenum Wisconsin 54-1255	XP_002557208.1	9 E-83	482/534
Eukaryota	Fungi	Aspergillus niger CBS 513.88	XP_001400787.1	3 E-82	497/534
Eukaryota	Fungi	Penicillium chrysogenum Wisconsin 54-1255	XP_002561138.1	3 E-82	503/534
Eukaryota	Fungi	Podospora anserina DSM 980	XP_001903608.1	7 E-82	505/534
Eukaryota	Fungi	Neosartorya fischeri NRRL 181	XP_001258579.1	1 E-81	529/534
Eukaryota	Fungi	Chaetomium globosum CBS 148.51	XP_001226269.1	1 E-81	501/534
Eukaryota	Fungi	Coprinopsis cinerea okayama7#130	XP_001836437.1	2 E-81	436/534
Eukaryota	Fungi	Talaromyces stipitatus ATCC 10500	XP_002477997.1	2 E-81	469/534
Eukaryota	Fungi	Penicillium marneffei ATCC 18224	XP_002150980.1	3 E-81	462/534
Eukaryota	Fungi	Neurospora crassa OR74A	XP_957424.1	3 E-81	506/534
Eukaryota	Fungi	Chaetomium globosum CBS 148.51	XP_001220290.1	4 E-81	516/534
Eukaryota	Fungi	Aspergillus nidulans FGSC A4	XP_682158.1	1 E-80	454/534
Eukaryota	Fungi	Gibberella zeae PH-1	XP_387526.1	2 E-80	464/534

Eukaryota	Fungi	Magnaporthe grisea 70-15	XP_366289.1	4 E-80	519/534
Eukaryota	Fungi	Cryptococcus neoformans var. neoformans	XP_568685.1	1 E-79	495/534
Eukaryota	Fungi	Penicillium chrysogenum Wisconsin 54-1255	XP_002568019.1	1 E-79	474/534
Eukaryota	Fungi	Aspergillus fumigatus Af293	XP_751324.1	3 E-79	534/534
Eukaryota	Fungi	Aspergillus fumigatus Af293	XP_753005.1	3 E-79	493/534
Eukaryota	Fungi	Aspergillus fumigatus A1163	EDP55337.1	5 E-79	534/534
Eukaryota	Fungi	Aspergillus oryzae RIB40	XP_001822870.1	1 E-78	519/534
Eukaryota	Fungi	Verticillium albo-atrum VaMs.102	EEY22433.1	1 E-78	505/534
Eukaryota	Fungi	Pyrenophora tritici-repentis Pt-1C-BFP	XP_001933299.1	2 E-78	493/534
Eukaryota	Fungi	Aspergillus clavatus NRRL 1	XP_001268640.1	2 E-78	491/534
Eukaryota	Fungi	Nectria haematococca mpVI 77-13-4	EEU35618.1	4 E-78	456/534
Eukaryota	Fungi	Aspergillus niger CBS 513.88	XP_001401647.1	4 E-78	486/534
Eukaryota	Fungi	Penicillium chrysogenum Wisconsin 54-1255	XP_002564502.1	7 E-78	460/534
Eukaryota	Fungi	Aspergillus clavatus NRRL 1	XP_001268482.1	7 E-78	544/534
Eukaryota	Fungi	Phaeosphaeria nodorum SN15	XP_001790837.1	8 E-78	454/534
Eukaryota	Fungi	Magnaporthe grisea 70-15	XP_364462.2	1 E-77	472/534
Eukaryota	Fungi	Nectria haematococca mpVI 77-13-4	EEU35678.1	2 E-77	473/534
Eukaryota	Fungi	Talaromyces stipitatus ATCC 10500	XP_002483356.1	2 E-77	474/534
Eukaryota	Fungi	Verticillium albo-atrum VaMs.102	EEY17463.1	6 E-77	456/534
Eukaryota	Fungi	Talaromyces stipitatus ATCC 10500	XP_002481943.1	9 E-77	530/534
Eukaryota	Fungi	Neurospora crassa OR74A	XP_959844.1	1 E-76	480/534
Eukaryota	Fungi	Botryotinia fuckeliana B05.10	XP_001548958.1	2 E-76	447/534
Eukaryota	Fungi	Botryotinia fuckeliana B05.10	XP_001556149.1	2 E-76	475/534
Eukaryota	Fungi	Pyrenophora tritici-repentis Pt-1C-BFP	XP_001932382.1	4 E-76	516/534
Eukaryota	Fungi	Pyrenophora tritici-repentis Pt-1C-BFP	XP_001941095.1	8 E-76	504/534
Eukaryota	Fungi	Gibberella zeae PH-1	XP_387807.1	2 E-75	457/534
Eukaryota	Fungi	Chaetomium globosum CBS 148.51	XP_001223536.1	2 E-75	457/534
Eukaryota	Fungi	Aspergillus fumigatus A1163	EDP53638.1	2 E-75	464/534
Eukaryota	Fungi	Nectria haematococca mpVI 77-13-4	EEU41606.1	3 E-75	481/534
Eukaryota	Fungi	Aspergillus fumigatus Af293	XP_748341.2	6 E-75	464/534
Eukaryota	Fungi	Nectria haematococca mpVI 77-13-4	EEU35801.1	7 E-75	457/534
Eukaryota	Fungi	Gibberella moniliformis	ABV60280.1	1 E-74	457/534
Eukaryota	Fungi	Nectria haematococca mpVI 77-13-4	EEU37575.1	1 E-74	493/534
Eukaryota	Fungi	Cryptococcus neoformans var. neoformans	XP_571045.1	3 E-74	477/534
Eukaryota	Fungi	Nectria haematococca mpVI 77-13-4	EEU37845.1	5 E-74	468/534
Eukaryota	Fungi	Pyrenophora tritici-repentis Pt-1C-BFP	XP_001933447.1	6 E-74	465/534
Eukaryota	Fungi	Phaeosphaeria nodorum SN15	XP_001793796.1	9 E-74	508/534
Eukaryota	Fungi	Phaeosphaeria nodorum SN15	XP_001794107.1	1 E-73	470/534
Eukaryota	Fungi	Penicillium chrysogenum Wisconsin 54-1255	XP_002560679.1	3 E-73	494/534
Eukaryota	Fungi	Penicillium marneffei ATCC 18224	XP_002151471.1	8 E-73	465/534
Eukaryota	Fungi	Aspergillus terreus NIH2624	XP_001213248.1	8 E-73	532/534
Eukaryota	Fungi	Botryotinia fuckeliana B05.10	XP_001548337.1	1 E-72	428/534
Eukaryota	Fungi	Magnaporthe grisea 70-15	XP_362991.2	1 E-72	487/534
Eukaryota	Fungi	Aspergillus fumigatus A1163	EDP53636.1	1 E-72	475/534
Eukaryota	Fungi	Pyrenophora tritici-repentis Pt-1C-BFP	XP_001931768.1	2 E-72	467/534
Eukaryota	Fungi	Magnaporthe grisea 70-15	XP_369159.1	2 E-72	502/534
Eukaryota	Fungi	Phaeosphaeria nodorum SN15	XP_001801375.1	2 E-72	487/534
Eukaryota	Fungi	Aspergillus nidulans FGSC A4	XP_657837.1	2 E-72	498/534
Eukaryota	Fungi	Podospora anserina DSM 980	XP_001903247.1	3 E-72	433/534
Eukaryota	Fungi	Gibberella zeae PH-1	XP_383320.1	4 E-72	511/534
Eukaryota	Fungi	Gibberella zeae PH-1	XP_384852.1	5 E-72	475/534
Eukaryota	Fungi	Verticillium albo-atrum VaMs.102	EEY21493.1	9 E-72	485/534
Eukaryota	Fungi	Aspergillus fumigatus Af293	XP_748344.2	1 E-71	465/534
Eukaryota	Fungi	Neosartorya fischeri NRRL 181	XP_001258627.1	2 E-71	436/534
Eukaryota	Fungi	Nectria haematococca mpVI 77-13-4	EEU43515.1	5 E-71	474/534
Eukaryota	Fungi	Gibberella zeae PH-1	XP_385896.1	5 E-70	479/534
Eukaryota	Fungi	Talaromyces stipitatus ATCC 10500	XP_002341594.1	5 E-70	465/534
Eukaryota	Fungi	Chaetomium globosum CBS 148.51	XP_001224547.1	5 E-70	481/534

Eukaryota	Fungi	Nectria haematococca mpVI 77-13-4	EEU33855.1	3 E-69	472/534
Eukaryota	Fungi	Nectria haematococca mpVI 77-13-4	EEU35196.1	7 E-69	480/534
Eukaryota	Fungi	Verticillium albo-atrum VaMs.102	EEY23963.1	4 E-68	499/534
Eukaryota	Fungi	Neosartorya fischeri NRRL 181	XP_001258822.1	9 E-68	507/534
Eukaryota	Fungi	Talaromyces stipitatus ATCC 10500	XP_002485938.1	5 E-67	440/534
Eukaryota	Fungi	Verticillium albo-atrum VaMs.102	EEY16110.1	8 E-67	447/534
Eukaryota	Fungi	Verticillium albo-atrum VaMs.102	EEY23835.1	2 E-66	524/534
Eukaryota	Fungi	Aspergillus clavatus NRRL 1	XP_001273720.1	3 E-66	498/534
Eukaryota	Fungi	Gibberella zeae PH-1	XP_385528.1	3 E-66	495/534
Eukaryota	Fungi	Magnaporthe grisea 70-15	XP_369535.1	3 E-66	482/534
Eukaryota	Fungi	Cryptococcus neoformans var. neoformans	XP_572092.1	4 E-66	480/534
Eukaryota	Fungi	Coccidioides posadasii C735 delta	EER29768.1	6 E-66	496/534
Eukaryota	Fungi	Cryptococcus neoformans var. neoformans	XP_774549.1	8 E-66	480/534
Eukaryota	Fungi	Sclerotinia sclerotiorum 1980 UF-70	XP_001585622.1	9 E-66	503/534
Eukaryota	Fungi	Aspergillus oryzae RIB40	XP_001824503.1	2 E-64	482/534
Eukaryota	Fungi	Magnaporthe grisea 70-15	XP_361760.2	6 E-63	475/534
Eukaryota	Fungi	Aspergillus terreus NIH2624	XP_001209810.1	8 E-63	507/534
Eukaryota	Fungi	Nectria haematococca mpVI 77-13-4	EEU41662.1	1 E-62	471/534
Eukaryota	Fungi	Aspergillus flavus NRRL3357	XP_002384138.1	1 E-62	487/534
Eukaryota	Fungi	Phaeosphaeria nodorum SN15	XP_001799162.1	2 E-62	500/534
Eukaryota	Fungi	Nectria haematococca mpVI 77-13-4	EEU38316.1	2 E-62	492/534
Eukaryota	Fungi	Verticillium albo-atrum VaMs.102	EEY17963.1	3 E-62	457/534
Eukaryota	Fungi	Coccidioides immitis RS;	XP_001243560.1	3 E-62	516/534
Eukaryota	Fungi	Aspergillus nidulans FGSC A4	XP_664435.1	4 E-62	491/534
Eukaryota	Fungi	Podospora anserina DSM 980	XP_001906434.1	5 E-62	505/534
Eukaryota	Fungi	Aspergillus fumigatus Af293	XP_753203.1	2 E-61	482/534
Eukaryota	Fungi	Verticillium albo-atrum VaMs.102	EEY21884.1	2 E-61	464/534
Eukaryota	Fungi	Aspergillus flavus NRRL3357	XP_002383101.1	2 E-61	527/534
Eukaryota	Fungi	Penicillium marneffei ATCC 18224	XP_002144908.1	2 E-61	520/534
Eukaryota	Fungi	Nectria haematococca mpVI 77-13-4	EEU35547.1	3 E-61	444/534
Eukaryota	Fungi	Neosartorya fischeri NRRL 181	XP_001259225.1	3 E-61	512/534
Eukaryota	Fungi	Nectria haematococca mpVI 77-13-4	EEU33794.1	4 E-61	533/534
Eukaryota	Fungi	Nectria haematococca mpVI 77-13-4	EEU34649.1	5 E-61	477/534
Eukaryota	Fungi	Chaetomium globosum CBS 148.51	XP_001220480.1	5 E-61	468/534
Eukaryota	Fungi	Verticillium albo-atrum VaMs.102	EEY13991.1	6 E-61	490/534

AFUA_7G06140

Eukaryota	Fungi	Aspergillus fumigatus Af293	XP_748896.1	0.0	739/739
Eukaryota	Fungi	Neosartorya fischeri NRRL 181	XP_001261562.1	0.0	739/739
Eukaryota	Fungi	Penicillium chrysogenum Wisconsin 54-1255	XP_002563511.1	0.0	706/739
Eukaryota	Fungi	Aspergillus terreus NIH2624	XP_001216040.1	0.0	729/739
Eukaryota	Fungi	Aspergillus terreus NIH2624	XP_001216552.1	0.0	714/739
Eukaryota	Fungi	Aspergillus nidulans FGSC A4	XP_660432.1	0.0	713/739
Eukaryota	Fungi	Talaromyces stipitatus ATCC 10500	XP_002485128.1	0.0	711/739
Eukaryota	Fungi	Penicillium marneffei ATCC 18224	XP_002149046.1	0.0	695/739
Eukaryota	Fungi	Botryotinia fuckeliana B05.10	XP_001547429.1	0.0	722/739
Eukaryota	Fungi	Sclerotinia sclerotiorum 1980 UF-70	XP_001591221.1	0.0	694/739
Eukaryota	Fungi	Neurospora crassa OR74A	XP_959400.1	0.0	707/739
Eukaryota	Fungi	Pyrenophora tritici-repentis Pt-1C-BFP	XP_001939746.1	0.0	707/739
Eukaryota	Fungi	Hypocrea jecorina	AAA18473.1	0.0	707/739
Eukaryota	Fungi	Trichoderma sp. SSL	ACH92574.1	0.0	707/739
Eukaryota	Fungi	Hypocrea jecorina	prf1713235A	0.0	707/739
Eukaryota	Fungi	Trichoderma viride mitosporic Hypocreaceae;	AAQ76093.1	0.0	710/739
Eukaryota	Fungi	Chaetomium globosum CBS 148.51	XP_001224406.1	0.0	688/739
Eukaryota	Fungi	Podospora anserina DSM 980	XP_001903317.1	0.0	686/739
Eukaryota	Fungi	Magnaporthe grisea 70-15	XP_364573.1	0.0	717/739
Eukaryota	Fungi	Phaeosphaeria nodorum SN15	XP_001803202.1	0.0	676/739

Eukaryota	Fungi	Coprinopsis cinerea okayama7#130	XP_001828909.1	0.0	676/739
Eukaryota	Fungi	Cryptococcus neoformans var. neoformans	XP_776417.1	0.0	742/739
Eukaryota	Fungi	Cryptococcus neoformans var. neoformans	XP_569544.1	0.0	742/739
Eukaryota	Fungi	Phanerochaete chrysosporium	BAB85988.1	0.0	714/739
Eukaryota	Fungi	Phanerochaete chrysosporium	AAC26490.1	0.0	714/739
Eukaryota	Fungi	Phanerochaete chrysosporium	AAC26489.1	0.0	714/739
Eukaryota	Fungi	Verticillium albo-atrum VaMs.102	EEY22483.1	0.0	695/739
Eukaryota	Fungi	Penicillium marneffei ATCC 18224	XP_002144089.1	0.0	815/739
Eukaryota	Fungi	Postia placenta Mad-698-R	XP_002470190.1	0.0	737/739
Eukaryota	Fungi	Laccaria bicolor S238N-H82	XP_001879679.1	0.0	736/739
Eukaryota	Fungi	Aspergillus flavus NRRL3357	XP_002383240.1	0.0	824/739
Eukaryota	Fungi	Aspergillus oryzae RIB40	XP_001816831.1	0.0	824/739
Eukaryota	Fungi	Coccidioides posadasii C735 delta	EER26285.1	0.0	815/739
Eukaryota	Fungi	Uncinocarpus reesii 1704	XP_002542559.1	0.0	816/739
Eukaryota	Fungi	Coccidioides posadasii;	AAF21242.1	0.0	815/739
Eukaryota	Fungi	Coccidioides immitis RS;	XP_001244447.1	0.0	815/739
Eukaryota	Amoebozoa	Physarum polycephalum	BAF02537.1	0.0	722/739
Eukaryota	Amoebozoa	Physarum polycephalum	BAE43955.1	0.0	722/739
Eukaryota	Amoebozoa	Physarum polycephalum	BAF02538.1	0.0	722/739
Eukaryota	Fungi	Aspergillus niger	ABH01182.1	0.0	818/739
Eukaryota	Fungi	Aspergillus kawachii	BAA19913.1	0.0	816/739
Eukaryota	Fungi	Aspergillus niger	CAB75696.1	0.0	818/739
Eukaryota	Fungi	Thermoascus aurantiacus	AAZ95587.1	0.0	629/739
Eukaryota	Fungi	Thermoascus aurantiacus var. levisporus	ABX79553.1	0.0	630/739
Eukaryota	Fungi	Thermoascus aurantiacus var. levisporus	ABX79552.1	0.0	630/739
Eukaryota	Fungi	Penicillium brasilianum	ABP88968.1	0.0	626/739
Eukaryota	Fungi	Phaeosphaeria avenaria f. sp.	AAT95378.1	0.0	829/739
Eukaryota	Fungi	Aspergillus niger	CBA02054.1	1 E-180	818/739
Eukaryota	Fungi	Aspergillus niger	ABB29285.1	1 E-180	818/739
Eukaryota	Fungi	Aspergillus niger CBS 513.88	XP_001398816.1	1 E-180	818/739
Eukaryota	Fungi	Aspergillus niger	ACV91073.1	1 E-180	818/739
Eukaryota	Fungi	Aspergillus niger	ABN73102.1	1 E-180	818/739
Eukaryota	Fungi	Aspergillus niger	ABW87793.1	1 E-180	818/739
Eukaryota	Fungi	Ustilago maydis 521	XP_756593.1	1 E-179	721/739
Eukaryota	Fungi	Chaetomium globosum CBS 148.51	XP_001229937.1	1 E-179	624/739
Eukaryota	Fungi	Sclerotinia sclerotiorum 1980 UF-70	XP_001585739.1	1 E-179	622/739
Eukaryota	Fungi	Penicillium purpurogenum	ACV87737.1	1 E-179	630/739
Eukaryota	Fungi	Aspergillus niger	ACN87968.1	1 E-179	818/739
Eukaryota	Fungi	Periconia sp. BCC 2871	ABX84365.1	1 E-178	622/739
Eukaryota	Fungi	Ajellomyces dermatitidis SLH14081	XP_002625154.1	1 E-178	819/739
Eukaryota	Fungi	Ajellomyces capsulatus	AAA86880.1	1 E-177	819/739
Eukaryota	Fungi	Aspergillus nidulans FGSC A4	XP_663580.1	1 E-177	749/739
Eukaryota	Fungi	Aspergillus niger	ACJ02084.1	1 E-176	818/739
Eukaryota	Fungi	Aspergillus aculeatus	P48825.1	1 E-176	818/739
Eukaryota	Fungi	Botryotinia fuckeliana B05.10	XP_001551395.1	1 E-176	632/739
Eukaryota	Fungi	Phaeosphaeria avenaria f. sp.	AAT95379.1	1 E-176	633/739
Eukaryota	Fungi	Aspergillus niger	ACJ64497.1	1 E-175	818/739
Eukaryota	Fungi	Phaeosphaeria avenaria	CAB82861.1	1 E-175	633/739
Eukaryota	Fungi	Aspergillus oryzae RIB40	XP_001825173.1	1 E-175	750/739
Eukaryota	Fungi	Penicillium chrysogenum Wisconsin 54-1255	XP_002565451.1	1 E-175	747/739
Eukaryota	Fungi	Phaeosphaeria avenaria f. sp.	AAU00986.1	1 E-175	633/739
Eukaryota	Fungi	Chaetomium thermophilum	ABR57325.2	1 E-175	621/739
Eukaryota	Fungi	Phaeosphaeria nodorum SN15	XP_001802118.1	1 E-175	633/739
Eukaryota	Fungi	Phaeosphaeria avenaria f. sp.	AAT95377.1	1 E-175	633/739
Eukaryota	Fungi	Phaeosphaeria avenaria f. sp.	AAU00981.1	1 E-175	633/739
Eukaryota	Fungi	Phaeosphaeria avenaria f. sp.	AAT95376.1	1 E-175	633/739
Eukaryota	Fungi	Phaeosphaeria nodorum	AAT95381.1	1 E-175	633/739
Eukaryota	Fungi	Phaeosphaeria sp. S-93-48	AAT95380.1	1 E-175	633/739

Eukaryota	Fungi	Aspergillus terreus NIH2624	XP_001212225.1	1 E-175	634/739
Eukaryota	Fungi	Phaeosphaeria nodorum	AAT95384.1	1 E-174	633/739
Eukaryota	Fungi	Phaeosphaeria nodorum SN15	XP_001798056.1	1 E-174	835/739
Eukaryota	Fungi	Malassezia globosa CBS 7966	XP_001730658.1	1 E-173	731/739
Eukaryota	Fungi	Sclerotinia sclerotiorum 1980 UF-70	XP_001591700.1	1 E-173	632/739
Eukaryota	Fungi	Penicillium marneffei ATCC 18224	XP_002145351.1	1 E-173	635/739
Eukaryota	Fungi	Ajellomyces dermatitidis ER-3	EEQ83767.1	1 E-173	819/739
Eukaryota	Fungi	Penicillium chrysogenum Wisconsin 54-1255	XP_002562038.1	1 E-173	639/739
Eukaryota	Fungi	Talaromyces stipitatus ATCC 10500	XP_002480480.1	1 E-173	630/739
Eukaryota	Fungi	Aspergillus clavatus NRRL 1	XP_001269582.1	1 E-172	819/739
Eukaryota	Fungi	Aspergillus nidulans FGSC A4	CBF74704.1	1 E-172	632/739
Eukaryota	Fungi	Podospora anserina DSM 980	XP_001907699.1	1 E-172	624/739
Eukaryota	Fungi	Nectria haematococca mpVI 77-13-4	EEU39730.1	1 E-172	633/739
Eukaryota	Fungi	Magnaporthe grisea	AAX07690.1	1 E-171	642/739
Eukaryota	Fungi	Uromyces viciae-fabae	CAE01320.1	1 E-171	738/739
Eukaryota	Fungi	Ajellomyces capsulatus H143	EER45067.1	1 E-171	801/739
Eukaryota	Fungi	Gibberella zeae PH-1	XP_386781.1	1 E-170	653/739
Eukaryota	Fungi	Pyrenophora tritici-repentis Pt-1C-BFP	XP_001941555.1	1 E-169	755/739
Eukaryota	Fungi	Microsporum canis CBS 113480	EEQ28040.1	1 E-169	630/739
Eukaryota	Fungi	Ajellomyces capsulatus NAm1	XP_001543147.1	1 E-168	634/739
Eukaryota	Fungi	Neurospora crassa OR74A	XP_956104.1	1 E-168	625/739
Eukaryota	Fungi	Nectria haematococca mpVI 77-13-4	EEU44862.1	1 E-167	762/739
Eukaryota	Fungi	Phaeosphaeria nodorum SN15	XP_001805849.1	1 E-167	721/739
Eukaryota	Fungi	Hypocrea jecorina	AAP57755.1	1 E-167	635/739
Eukaryota	Fungi	Penicillium decumbens	ACD86466.1	1 E-167	633/739
Eukaryota	Fungi	Phaeosphaeria nodorum SN15	XP_001803882.1	1 E-167	758/739
Eukaryota	Fungi	Aspergillus avenaceus	AAX39011.1	1 E-166	633/739
Eukaryota	Fungi	Talaromyces emersonii	AAL69548.3	1 E-166	625/739
Eukaryota	Fungi	Gibberella zeae PH-1	XP_385129.1	1 E-166	747/739
Eukaryota	Fungi	Podospora anserina DSM 980	XP_001904749.1	1 E-166	625/739
Eukaryota	Fungi	Aspergillus terreus NIH2624	XP_001211891.1	1 E-166	746/739
Eukaryota	Fungi	Neosartorya fischeri NRRL 181	XP_001265091.1	1 E-165	642/739
Eukaryota	Fungi	Thermoascus aurantiacus var. levisporus	ABX56927.1	1 E-165	612/739
Eukaryota	Fungi	Botryotinia fuckeliana B05.10	XP_001554878.1	1 E-165	593/739
Eukaryota	Fungi	Coprinopsis cinerea okayama7#130	XP_001834093.1	1 E-165	769/739
Eukaryota	Fungi	Penicillium marneffei ATCC 18224	XP_002145911.1	1 E-165	611/739
Eukaryota	Fungi	Rhizomucor miehei	CAP58431.2	1 E-165	708/739
Eukaryota	Fungi	Aspergillus fumigatus A1163	EDP55914.1	1 E-164	642/739
Eukaryota	Fungi	Aspergillus fumigatus Af293	XP_750327.1	1 E-164	642/739
Eukaryota	Fungi	Magnaporthe grisea 70-15	XP_364315.2	1 E-164	652/739
Eukaryota	Fungi	Ajellomyces capsulatus G186AR	EEH04992.1	1 E-163	801/739
Eukaryota	Fungi	Aspergillus oryzae RIB40	XP_001823627.1	1 E-163	719/739
Eukaryota	Fungi	Paracoccidioides brasiliensis Pb18;	EEH44194.1	1 E-163	794/739
Eukaryota	Fungi	Talaromyces stipitatus ATCC 10500	XP_002478189.1	1 E-163	629/739
Eukaryota	Fungi	Coccidioides immitis RS;	XP_001247592.1	1 E-163	610/739
Eukaryota	Fungi	Paracoccidioides brasiliensis Pb03;	EEH19824.1	1 E-163	794/739
Eukaryota	Fungi	Penicillium marneffei ATCC 18224	XP_002153599.1	1 E-162	760/739
Eukaryota	Fungi	Coccidioides posadasii C735 delta	EER23687.1	1 E-162	610/739
Eukaryota	Fungi	Aspergillus flavus NRRL3357	XP_002379048.1	1 E-162	719/739
Eukaryota	Fungi	Magnaporthe grisea 70-15	XP_364427.2	1 E-162	615/739
Eukaryota	Fungi	Thermoascus aurantiacus	AAY33982.1	1 E-161	612/739
Eukaryota	Fungi	Thermoascus aurantiacus var. levisporus	ABX56926.1	1 E-161	612/739
Eukaryota	Fungi	Talaromyces emersonii	AAM94393.3	1 E-161	755/739
Eukaryota	Fungi	Coprinopsis cinerea okayama7#130	XP_001834090.1	1 E-161	685/739
Eukaryota	Fungi	Septoria lycopersici	AAB08445.1	1 E-161	758/739
Eukaryota	Fungi	Neosartorya fischeri NRRL 181	XP_001262303.1	1 E-160	747/739
Eukaryota	Fungi	Verticillium albo-atrum VaMs.102	EEY22364.1	1 E-160	754/739
Eukaryota	Fungi	Podospora anserina DSM 980	XP_001905784.1	1 E-160	720/739

Eukaryota	Fungi	Aspergillus nidulans FGSC A4	CBF75129.1	1 E-160	612/739
Eukaryota	Fungi	Chaetomium globosum CBS 148.51	XP_001222902.1	1 E-159	665/739
Eukaryota	Fungi	Uncinocarpus reesii 1704	XP_002541775.1	1 E-159	610/739
Eukaryota	Fungi	Penicillium chrysogenum Wisconsin 54-1255	XP_002557931.1	1 E-159	611/739
Eukaryota	Fungi	Neosartorya fischeri NRRL 181	XP_001257986.1	1 E-159	612/739
Eukaryota	Fungi	Neurospora crassa OR74A	XP_960539.1	1 E-159	757/739
Eukaryota	Fungi	Aspergillus fumigatus Af293	XP_747720.1	1 E-159	656/739
Eukaryota	Fungi	Aspergillus terreus NIH2624	XP_001215795.1	1 E-159	625/739
Eukaryota	Fungi	Verticillium albo-atrum VaMs.102	EEY16958.1	1 E-158	759/739
Eukaryota	Fungi	Sclerotinia sclerotiorum 1980 UF-70	XP_001593940.1	1 E-158	762/739
Eukaryota	Fungi	Nectria haematococca mpVI 77-13-4	EEU45349.1	1 E-158	648/739
Eukaryota	Fungi	Aspergillus nidulans FGSC A4	XP_661706.1	1 E-157	601/739
Eukaryota	Fungi	Sclerotinia sclerotiorum 1980 UF-70	XP_001591716.1	1 E-157	633/739
Eukaryota	Fungi	Gaeumannomyces graminis	AAB09777.1	1 E-157	754/739
Eukaryota	Fungi	Botryotinia fuckeliana B05.10	XP_001551405.1	1 E-157	612/739
Eukaryota	Fungi	Neurospora crassa OR74A	XP_965185.1	1 E-157	641/739
Eukaryota	Fungi	Aspergillus fumigatus A1163	EDP49439.1	1 E-157	631/739
Eukaryota	Fungi	Penicillium chrysogenum Wisconsin 54-1255	XP_002557246.1	1 E-156	752/739
Eukaryota	Fungi	Magnaporthe grisea 70-15	XP_360965.1	1 E-156	725/739
Eukaryota	Fungi	Aspergillus niger CBS 513.88	XP_001397014.1	1 E-156	755/739
Eukaryota	Fungi	Talaromyces stipitatus ATCC 10500	XP_002479651.1	1 E-156	637/739
Eukaryota	Fungi	Aspergillus clavatus NRRL 1	XP_001270189.1	1 E-156	639/739
Eukaryota	Fungi	Pyrenophora tritici-repentis Pt-1C-BFP	XP_001935676.1	1 E-155	647/739
Eukaryota	Fungi	Aspergillus fumigatus Af293	XP_750759.1	1 E-155	631/739
Eukaryota	Fungi	Neurospora crassa	CAC28685.1	1 E-155	623/739
Eukaryota	Fungi	Aspergillus niger CBS 513.88	XP_001394024.1	1 E-155	722/739
Eukaryota	Fungi	Ajellomyces dermatitidis SLH14081	XP_002627412.1	1 E-155	616/739
Eukaryota	Fungi	Aspergillus oryzae RIB40	XP_001727197.1	1 E-155	724/739
Eukaryota	Fungi	Aspergillus flavus NRRL3357	XP_002375439.1	1 E-155	724/739
Eukaryota	Fungi	Ajellomyces dermatitidis ER-3	EEQ88361.1	1 E-154	616/739
Eukaryota	Fungi	Aspergillus nidulans FGSC A4	XP_661508.1	1 E-154	597/739
Eukaryota	Fungi	Botryotinia fuckeliana B05.10	XP_001554522.1	1 E-154	761/739
Eukaryota	Fungi	Hypocrea jecorina	AAP57760.1	1 E-154	732/739
Eukaryota	Fungi	Gibberella zeae PH-1	XP_384034.1	1 E-154	722/739
Eukaryota	Fungi	Podospora anserina	CAP65606.1	1 E-153	656/739
Eukaryota	Fungi	Uncinocarpus reesii 1704	XP_002544076.1	1 E-153	646/739
Eukaryota	Fungi	Nectria haematococca mpVI 77-13-4	EEU37850.1	1 E-153	616/739
Eukaryota	Fungi	Aspergillus flavus NRRL3357	XP_002382041.1	1 E-153	612/739
Eukaryota	Fungi	Botryotinia fuckeliana B05.10	XP_001551665.1	1 E-153	648/739
Eukaryota	Fungi	Gibberella zeae PH-1	XP_383746.1	1 E-152	632/739
Eukaryota	Fungi	Aspergillus oryzae RIB40	XP_001825347.1	1 E-152	748/739
Eukaryota	Fungi	Gibberella zeae PH-1	XP_391735.1	1 E-152	755/739
Eukaryota	Fungi	Gibberella zeae PH-1	XP_380342.1	1 E-152	645/739
Eukaryota	Fungi	Sclerotinia sclerotiorum 1980 UF-70	XP_001593603.1	1 E-152	648/739
Eukaryota	Fungi	Aspergillus flavus NRRL3357	XP_002380526.1	1 E-152	748/739
Eukaryota	Fungi	Penicillium marneffei ATCC 18224	XP_002143344.1	1 E-152	642/739
Eukaryota	Fungi	Aspergillus nidulans FGSC A4	XP_680665.1	1 E-152	727/739
Eukaryota	Fungi	Aspergillus oryzae RIB40	XP_001819055.1	1 E-151	612/739
Eukaryota	Fungi	Ajellomyces capsulatus NAm1	XP_001538164.1	1 E-151	616/739
Eukaryota	Fungi	Neosartorya fischeri NRRL 181	XP_001262119.1	1 E-151	744/739
Eukaryota	Fungi	Aspergillus terreus NIH2624	XP_001209807.1	1 E-150	649/739
Eukaryota	Fungi	Ajellomyces capsulatus G186AR	EEH05641.1	1 E-150	616/739
Eukaryota	Fungi	Microsporum canis CBS 113480	EEQ30264.1	1 E-150	642/739
Eukaryota	Fungi	Neosartorya fischeri NRRL 181	XP_001264012.1	1 E-150	722/739
Eukaryota	Fungi	Ustilago maydis 521	XP_762222.1	1 E-150	775/739
Eukaryota	Fungi	Ajellomyces capsulatus H143	EER43891.1	1 E-150	768/739
Eukaryota	Fungi	Laccaria bicolor S238N-H82	XP_001878554.1	1 E-150	688/739
Eukaryota	Fungi	Aspergillus terreus NIH2624	XP_001218668.1	1 E-150	738/739

Eukaryota	Fungi	Aspergillus fumigatus Af293	XP_753108.1	1 E-149	722/739
Eukaryota	Fungi	Aspergillus fumigatus A1163	EDP56956.1	1 E-149	722/739
Eukaryota	Fungi	Gibberella zeae PH-1	XP_390791.1	1 E-149	732/739
Eukaryota	Fungi	Nectria haematococca mpVI 77-13-4	EEU46688.1	1 E-149	745/739
Eukaryota	Fungi	Podospora anserina DSM 980	XP_001911741.1	1 E-149	624/739
Eukaryota	Fungi	Neosartorya fischeri NRRL 181	XP_001257563.1	1 E-149	620/739
Eukaryota	Fungi	Verticillium albo-atrum VaMs.102	EEY19538.1	1 E-149	790/739
Eukaryota	Fungi	Aspergillus niger CBS 513.88	XP_001400803.1	1 E-149	725/739
Eukaryota	Fungi	Paracoccidioides brasiliensis Pb03;	EEH19553.1	1 E-149	616/739
Eukaryota	Fungi	Nectria haematococca mpVI 77-13-4	EEU37407.1	1 E-149	720/739
Eukaryota	Fungi	Aspergillus oryzae RIB40	XP_001816992.1	1 E-149	640/739
Eukaryota	Fungi	Cochliobolus heterostrophus	AAB84005.1	1 E-149	614/739
Eukaryota	Fungi	Cochliobolus heterostrophus	AAB82946.1	1 E-149	614/739
Eukaryota	Fungi	Ajellomyces dermatitidis ER-3	EEQ91648.1	1 E-148	659/739
Eukaryota	Fungi	Pyrenophora tritici-repentis Pt-1C-BFP	XP_001937375.1	1 E-148	619/739
Eukaryota	Fungi	Aspergillus flavus NRRL3357	XP_002383069.1	1 E-148	640/739
Eukaryota	Fungi	Ajellomyces dermatitidis SLH14081	XP_002624568.1	1 E-148	659/739
Eukaryota	Fungi	Ajellomyces capsulatus NAm1	XP_001539618.1	1 E-148	641/739
Eukaryota	Fungi	Paracoccidioides brasiliensis Pb01;	EEH38380.1	1 E-148	616/739
Eukaryota	Fungi	Penicillium chrysogenum Wisconsin 54-1255	XP_002563832.1	1 E-148	625/739
Eukaryota	Fungi	Neosartorya fischeri NRRL 181	XP_001261853.1	1 E-148	757/739
Eukaryota	Fungi	Coccidioides immitis RS;	XP_001239075.1	1 E-148	647/739
Eukaryota	Fungi	Paracoccidioides brasiliensis Pb18;	EEH47439.1	1 E-147	616/739
Eukaryota	Fungi	Aspergillus niger CBS 513.88	XP_001396728.1	1 E-147	644/739
Eukaryota	Fungi	Coccidioides posadasii C735 delta	EER29906.1	1 E-147	647/739
Eukaryota	Fungi	Verticillium albo-atrum VaMs.102	EEY16137.1	1 E-147	640/739
Eukaryota	Fungi	Kuraishia capsulata	AAA91297.1	1 E-147	708/739
Eukaryota	Fungi	Podospora anserina DSM 980	XP_001911838.1	1 E-147	749/739
Eukaryota	Fungi	Paracoccidioides brasiliensis Pb18;	EEH48625.1	1 E-146	650/739
Eukaryota	Fungi	Aspergillus fumigatus Af293	XP_746996.1	1 E-146	757/739
Eukaryota	Fungi	Magnaporthe grisea 70-15	XP_367723.2	1 E-146	723/739
Eukaryota	Fungi	Paracoccidioides brasiliensis Pb01;	EEH33495.1	1 E-145	660/739
Eukaryota	Fungi	Magnaporthe grisea 70-15	XP_362854.2	1 E-145	707/739
Eukaryota	Fungi	Aspergillus niger CBS 513.88	XP_001391838.1	1 E-144	769/739
Eukaryota	Fungi	Aspergillus nidulans FGSC A4	XP_664256.1	1 E-144	638/739
Eukaryota	Fungi	Botryotinia fuckeliana B05.10	XP_001553209.1	1 E-144	732/739
Eukaryota	Fungi	Aspergillus oryzae RIB40	XP_001826730.1	1 E-143	754/739
Eukaryota	Fungi	Aspergillus oryzae RIB40	XP_001820553.1	1 E-142	757/739
Eukaryota	Fungi	Saccharomycopsis fibuligera	ACH90244.1	1 E-142	628/739
Eukaryota	Fungi	Gibberella zeae PH-1	XP_383563.1	1 E-141	749/739
Eukaryota	Fungi	Saccharomycopsis fibuligera	P22506.1	1 E-141	628/739
Eukaryota	Fungi	Ajellomyces capsulatus G186AR	EEH09336.1	1 E-141	623/739
Eukaryota	Fungi	Botryotinia fuckeliana	CAB61489.1	1 E-140	766/739
Eukaryota	Fungi	Penicillium chrysogenum Wisconsin 54-1255	XP_002568350.1	1 E-140	752/739
Eukaryota	Fungi	Ustilago maydis 521	XP_760179.1	1 E-139	752/739
Eukaryota	Fungi	Candida dubliniensis CD36	XP_002422413.1	1 E-139	616/739
Eukaryota	Fungi	Magnaporthe grisea 70-15	XP_360225.2	1 E-139	730/739
Eukaryota	Fungi	Candida albicans SC5314	XP_716473.1	1 E-139	616/739
Eukaryota	Fungi	Aspergillus flavus NRRL3357	XP_002373846.1	1 E-138	757/739
Eukaryota	Fungi	Coprinopsis cinerea okayama7#130	XP_001839812.1	1 E-138	695/739
Eukaryota	Fungi	Saccharomycopsis fibuligera	P22507.1	1 E-137	630/739

AFUA_7G06900

Eukaryota	Fungi	Aspergillus fumigatus Af293	XP_748821.1	0.0	323/323
Eukaryota	Fungi	Neosartorya fischeri NRRL 181	XP_001261640.1	0.0	323/323
Eukaryota	Fungi	Aspergillus oryzae RIB40	XP_001818566.1	1 E-142	320/323
Eukaryota	Fungi	Aspergillus terreus NIH2624	XP_001209325.1	1 E-138	321/323

Eukaryota	Fungi	Penicillium chrysogenum Wisconsin 54-1255	XP_002564064.1	1 E-135	319/323
Eukaryota	Fungi	Nectria haematococca mpVI 77-13-4	EEU44019.1	1 E-133	320/323
Eukaryota	Fungi	Aspergillus niger CBS 513.88	XP_001402221.1	1 E-128	322/323
Eukaryota	Fungi	Gibberella zeae PH-1	XP_381942.1	1 E-125	320/323
Eukaryota	Fungi	Nectria haematococca mpVI 77-13-4	EEU48681.1	1 E-106	321/323
Bacteria	Proteobacteria	Hyphomonas neptunium ATCC 15444	YP_761201.1	1 E-102	320/323
Bacteria	Proteobacteria	Burkholderia cenocepacia MC0-3	YP_001778575.1	7 E-68	298/323
Bacteria	Proteobacteria	Burkholderia cenocepacia AU 1054	YP_622922.1	1 E-67	298/323
Bacteria	Proteobacteria	Burkholderia cenocepacia HI2424	YP_838940.1	1 E-67	298/323
Bacteria	Proteobacteria	Rhodoferax ferrireducens T118	YP_521744.1	2 E-66	294/323
Bacteria	Actinobacteria	Mycobacterium vanbaalenii PYR-1	YP_955297.1	5 E-66	290/323
Bacteria	Proteobacteria	Roseobacter sp. MED193	ZP_01056119.1	1 E-61	309/323
Bacteria	Proteobacteria	Rhizobium etli CIAT 652	YP_001985405.1	2 E-61	306/323
Bacteria	Proteobacteria	Rhizobium etli 8C-3	ZP_03512482.1	2 E-61	300/323
Bacteria	Proteobacteria	Mesorhizobium opportunistum WSM2075	ZP_05812514.1	1 E-60	296/323
Bacteria	Proteobacteria	Mesorhizobium loti	CAD31279.1	2 E-60	295/323
Bacteria	Proteobacteria	Mesorhizobium loti MAFF303099	NP_085750.1	7 E-60	291/323
Bacteria	Proteobacteria	Labrenzia alexandrii DFL-11	ZP_05112474.1	4 E-58	296/323
Bacteria	Proteobacteria	Mesorhizobium loti MAFF303099	NP_103146.1	6 E-58	296/323
Bacteria	Proteobacteria	Marinomonas sp. MED121	ZP_01078529.1	1 E-56	294/323
Bacteria	Proteobacteria	Rhodobacterales bacterium HTCC2255	ZP_01448442.1	1 E-56	287/323
Bacteria	Proteobacteria	Burkholderia sp. 383	YP_366475.1	3 E-54	299/323
Bacteria	Proteobacteria	Jannaschia sp. CCS1	YP_509064.1	6 E-51	276/323
Archaea	Euryarchaeota	Methanopyrus kandleri AV19	NP_614910.1	5 E-39	276/323
Bacteria	Proteobacteria	gamma proteobacterium NOR51-B	ZP_04956509.1	1 E-38	292/323
Archaea	Euryarchaeota	Methanococcus maripaludis C7	YP_001330348.1	2 E-37	275/323
Archaea	Euryarchaeota	Methanocaldococcus vulcanius M7	YP_003246946.1	4 E-36	275/323
Archaea	Euryarchaeota	Methanococcus maripaludis C6	YP_001548869.1	7 E-36	275/323
Archaea	Euryarchaeota	Methanococcus maripaludis S2	NP_987252.1	8 E-36	275/323
Archaea	Euryarchaeota	Methanocaldococcus fervens AG86	YP_003128228.1	8 E-36	275/323
Archaea	Euryarchaeota	Archaeoglobus fulgidus DSM 4304	NP_069766.1	1 E-35	276/323
Archaea	Euryarchaeota	Methanococcoides burtonii DSM 6242	YP_566199.1	2 E-35	282/323
Archaea	Euryarchaeota	Methanocaldococcus jannaschii DSM 2661	NP_248002.1	3 E-35	275/323
Archaea	Euryarchaeota	Methanococcus maripaludis C5	YP_001098057.1	4 E-35	275/323
Bacteria	Planctomycetes	Blastopirellula marina DSM 3645	ZP_01091695.1	4 E-35	277/323
Bacteria	Firmicutes	Thermoanaerobacter tengcongensis MB4	NP_622585.1	4 E-35	280/323
Bacteria	Firmicutes	Thermoanaerobacter pseudethanolicus ATCC 3	YP_001665400.1	1 E-34	280/323
Bacteria	Firmicutes	Thermoanaerobacter sp. X514	YP_001663707.1	1 E-34	280/323
Bacteria	Planctomycetes	Planctomyces limnophilus DSM 3776	ZP_04426649.1	2 E-34	277/323
Bacteria	Planctomycetes	Rhodopirellula baltica SH 1	NP_868203.1	4 E-34	274/323
Bacteria	Firmicutes	Thermoanaerobacter italicus Ab9	ZP_05332722.1	1 E-33	280/323
Bacteria	Firmicutes	Pelotomaculum thermopropionicum SI	YP_001211075.1	1 E-33	279/323
Archaea	Euryarchaeota	Methanocaldococcus infernus ME	ZP_04790176.1	1 E-33	275/323
Bacteria	Firmicutes	Thermoanaerobacter mathranii subsp. mathranii	ZP_05378490.1	2 E-33	280/323
Bacteria	Firmicutes	Moorella thermoacetica ATCC 39073	YP_431090.1	2 E-33	279/323
Bacteria	Verrucomicrobia	Verrucomicrobiae bacterium DG1235	ZP_05058180.1	2 E-33	276/323
Bacteria	Verrucomicrobia	Akkermansia muciniphila ATCC BAA-835	YP_001877341.1	3 E-33	277/323
Bacteria	Firmicutes	Thermoanaerobacterium thermosaccharolyticum	ZP_05335417.1	4 E-33	280/323
Archaea	Euryarchaeota	Methanococcus vannielii SB	YP_001323649.1	5 E-33	274/323
Bacteria	Firmicutes	Paenibacillus larvae subsp. larvae	ZP_02329846.1	5 E-33	277/323
group	Anaerocellum	Anaerocellum thermophilum DSM 6725	YP_002572561.1	6 E-33	279/323
Bacteria	Firmicutes	Caldicellulosiruptor saccharolyticus DSM 8903	YP_001179644.1	8 E-33	279/323
Archaea	Euryarchaeota	Methanosaeta thermophila PT	YP_843724.1	1 E-32	288/323
Archaea	Euryarchaeota	Methanoculleus marisnigri JR1	YP_001047644.1	1 E-32	275/323
Archaea	Euryarchaeota	Methanospirillum hungatei JF-1	YP_502147.1	1 E-32	273/323
Bacteria	Firmicutes	Geobacillus sp. Y412MC10	YP_003241645.1	2 E-32	277/323
Bacteria	Firmicutes	Bacillus licheniformis ATCC 14580	YP_080106.1	2 E-32	276/323
Archaea	Euryarchaeota	uncultured methanogenic archaeon RC-I	YP_685294.1	2 E-32	276/323

Bacteria	Firmicutes	Alicyclobacillus acidocaldarius subsp. acidocalc	YP_003185630.1	2 E-32	277/323
Bacteria	Firmicutes	Geobacillus thermodenitrificans NG80-2	YP_001126681.1	3 E-32	274/323
Bacteria	Verrucomicrobia	Opitutus terrae PB90-1	YP_001819556.1	3 E-32	274/323
Archaea	Euryarchaeota	Methanocorpusculum labreanum Z	YP_001029917.1	4 E-32	286/323
Bacteria	Elusimicrobia	uncultured Termite group 1	YP_001956590.1	5 E-32	279/323
Bacteria	Firmicutes	Ammonifex degensii KC4	YP_003238241.1	6 E-32	279/323
Bacteria	Firmicutes	Paenibacillus sp. oral taxon	ZP_04853048.1	6 E-32	274/323
Bacteria	Firmicutes	Alicyclobacillus acidocaldarius LAA1	ZP_03494383.1	8 E-32	277/323
Bacteria	Firmicutes	Anoxybacillus flavithermus WK1	YP_002314956.1	8 E-32	274/323
Bacteria	Firmicutes	Bacillus cereus G9241;	ZP_00237313.1	8 E-32	277/323
Archaea	Euryarchaeota	Methanococcus voltae A3	ZP_02193516.1	9 E-32	275/323
Bacteria	Firmicutes	Bacillus thuringiensis serovar andalousiensis	ZP_04095650.1	1 E-31	277/323
Bacteria	Firmicutes	Bacillus thuringiensis serovar huazhongensis	ZP_04083554.1	1 E-31	277/323
Bacteria	Firmicutes	Heliobacterium modesticaldum Ice1	YP_001680093.1	1 E-31	282/323
Bacteria	Firmicutes	Bacillus cereus ATCC 10987	NP_977837.1	1 E-31	277/323
Bacteria	Firmicutes	Syntrophomonas wolfei subsp. wolfei	YP_754810.1	1 E-31	281/323
Bacteria	Firmicutes	Bacillus cereus ATCC 14579	NP_831177.1	1 E-31	277/323
Bacteria	Firmicutes	Bacillus cereus ATCC 4342	ZP_04283176.1	1 E-31	277/323
Bacteria	Firmicutes	Bacillus cereus G9842;	YP_002444838.1	1 E-31	277/323
Bacteria	Firmicutes	Bacillus anthracis str. Ames	NP_843873.1	2 E-31	277/323
Bacteria	Firmicutes	Bacillus cereus AH1134;	ZP_03228943.1	2 E-31	277/323
Archaea	Euryarchaeota	Methanococcus aeolicus Nankai-3	YP_001325384.1	2 E-31	275/323
Bacteria	Firmicutes	Bacillus thuringiensis serovar kurstaki	ZP_04113972.1	3 E-31	277/323
Bacteria	Firmicutes	Epulopiscium sp. 'N.t. morphotype	ZP_02693157.1	3 E-31	277/323
Bacteria	Firmicutes	Dethiobacter alkaliphilus AHT 1	ZP_03734583.1	4 E-31	279/323
Bacteria	Firmicutes	Bacillus thuringiensis serovar konkukian	YP_035614.1	5 E-31	277/323
Bacteria	Firmicutes	Carboxydothermus hydrogenoformans Z-2901	YP_359373.1	5 E-31	278/323
Bacteria	Synergistetes	Anaerobaculum hydrogeniformans ATCC BAA	ZP_05800146.1	6 E-31	285/323
Bacteria	Firmicutes	Geobacillus kaustophilus HTA426	YP_148515.1	7 E-31	274/323
Bacteria	Synergistetes	Thermanaerovibrio acidaminovorans DSM 6589	ZP_04469136.1	1 E-30	279/323
Archaea	Euryarchaeota	Methanosarcina mazei Go1	NP_633069.1	1 E-30	279/323
Archaea	Methanosphaerula	Methanosphaerula palustris E1-9c	YP_002465767.1	2 E-30	275/323
Bacteria	Firmicutes	Bacillus cereus R309803;	ZP_04288442.1	2 E-30	277/323
Bacteria	Firmicutes	Bacillus cereus Rock3-28;	ZP_04232810.1	2 E-30	277/323
Bacteria	Dictyoglomi	Dictyoglomus turgidum DSM 6724	YP_002353252.1	3 E-30	294/323
Bacteria	Firmicutes	Desulfotomaculum acetoxidans DSM 771	YP_003190131.1	3 E-30	279/323
Bacteria	Planctomycetes	Planctomyces maris DSM 8797	ZP_01855039.1	4 E-30	277/323
Bacteria	Firmicutes	Bacillus cereus Rock3-44;	ZP_04216767.1	4 E-30	277/323
Bacteria	Firmicutes	Bacillus cereus Rock4-2;	ZP_04211234.1	4 E-30	277/323
Archaea	Euryarchaeota	Ferroglobus placidus DSM 10642	ZP_05841496.1	4 E-30	277/323
Bacteria	Verrucomicrobia	Chthoniobacter flavus Ellin428	ZP_03130277.1	4 E-30	277/323
Bacteria	Firmicutes	Geobacillus sp. Y412MC52	ZP_04391000.1	5 E-30	274/323
Bacteria	Firmicutes	Bacillus cereus ATCC 14579	AAO91867.1	5 E-30	277/323
Bacteria	Firmicutes	Bacillus mycoides Rock3-17;	ZP_04156245.1	5 E-30	277/323
Bacteria	Firmicutes	Bacillus cereus Rock3-29;	ZP_04226962.1	6 E-30	277/323
Bacteria	Proteobacteria	marine gamma proteobacterium HTCC2080	ZP_01626000.1	6 E-30	285/323
Bacteria	Firmicutes	Bacillus mycoides DSM 2048	ZP_04167997.1	6 E-30	277/323
Bacteria	Firmicutes	Bacillus pumilus SAFR-032	YP_001487696.1	7 E-30	274/323
Bacteria	Firmicutes	Bacillus weihenstephanensis KBAB4;	YP_001644193.1	8 E-30	277/323
Bacteria	Firmicutes	Bacillus cereus Rock4-18;	ZP_04207706.1	8 E-30	277/323
Bacteria	Firmicutes	Bacillus pumilus ATCC 7061	ZP_03055758.1	9 E-30	274/323
Bacteria	Firmicutes	Bacillus sp. NRRL B-14911	ZP_01171798.1	1 E-29	281/323
Bacteria	Firmicutes	Bacillus pseudomycoides DSM 12442	ZP_04150471.1	1 E-29	277/323
Bacteria	Firmicutes	Bacillus cytotoxicus NVH 391-98	YP_001374758.1	2 E-29	283/323
Bacteria	Firmicutes	Bacillus cereus AH1273;	ZP_04173681.1	2 E-29	277/323
Bacteria	Candidatus	Candidatus Methanoregula boonei 6A8	YP_001404582.1	2 E-29	275/323
Bacteria	Firmicutes	Candidatus Desulforudis audaxviator MP104C	YP_001716541.1	2 E-29	279/323
Bacteria	Firmicutes	Desulfitobacterium hafniense Y51	YP_517597.1	3 E-29	279/323

Bacteria	Verrucomicrobia	Verrucomicrobium spinosum DSM 4136	ZP_02926065.1	5 E-29	270/323
Archaea	Euryarchaeota	Methanosarcina barkeri str. Fusaro	YP_304136.1	5 E-29	282/323
Bacteria	Firmicutes	Bacillus thuringiensis IBL 200	ZP_04071437.1	6 E-29	277/323
Bacteria	Firmicutes	Geobacillus sp. Y4.1MC1	ZP_05370937.1	6 E-29	274/323
Bacteria	Symbiobacterium	Symbiobacterium thermophilum IAM 14863	YP_076515.1	1 E-28	270/323
Bacteria	Firmicutes	Bacillus cereus 95/8201;	ZP_04250665.1	1 E-28	277/323
Bacteria	Firmicutes	Bacillus anthracis str. A0442	ZP_02391506.1	1 E-28	277/323
Bacteria	Dictyoglomi	Dictyoglomus thermophilum H-6-12	YP_002251078.1	2 E-28	274/323
Bacteria	Firmicutes	Bacillus anthracis str. Ames	NP_844267.1	2 E-28	277/323
Bacteria	Firmicutes	Bacillus weihenstephanensis KBAB4;	YP_001644573.1	2 E-28	277/323
Bacteria	Firmicutes	Bacillus thuringiensis serovar israelensis	ZP_00740943.1	2 E-28	277/323
Bacteria	Firmicutes	Bacillus cereus G9241;	ZP_00236614.1	2 E-28	277/323
Bacteria	Firmicutes	Geobacillus sp. WCH70	YP_002950558.1	3 E-28	274/323
Bacteria	Firmicutes	Halothermothrix orenii H 168	YP_002508830.1	3 E-28	277/323
Bacteria	Firmicutes	Bacillus cereus AH1271;	ZP_04185666.1	3 E-28	283/323
Bacteria	Firmicutes	Bacillus cereus AH1134;	ZP_03231872.1	3 E-28	277/323
Archaea	Euryarchaeota	Methanosarcina acetivorans C2A	NP_619212.1	4 E-28	278/323
Bacteria	Firmicutes	Bacillus cereus ATCC 10987	NP_978249.1	4 E-28	277/323
Bacteria	Firmicutes	Bacillus cereus Rock3-42;	ZP_04222086.1	4 E-28	283/323
Bacteria	Firmicutes	Bacillus cereus H3081.97;	ZP_03235686.1	5 E-28	277/323
Bacteria	Firmicutes	Bacillus cereus ATCC 4342	ZP_04283572.1	6 E-28	277/323
Bacteria	Firmicutes	Bacillus cereus AH1272;	ZP_04179858.1	6 E-28	277/323
Bacteria	Firmicutes	Bacillus cereus B4264;	YP_002366568.1	7 E-28	277/323
Bacteria	Verrucomicrobia	Opitutaceae bacterium TAV2	ZP_03725016.1	9 E-28	274/323
Bacteria	Firmicutes	Bacillus thuringiensis serovar pondicheriensis	ZP_04089955.1	9 E-28	283/323
Bacteria	Firmicutes	Bacillus mycoides DSM 2048	ZP_04168379.1	9 E-28	283/323
Bacteria	Firmicutes	Bacillus thuringiensis IBL 4222	ZP_04064693.1	1 E-27	283/323
Bacteria	Firmicutes	Bacillus cereus AH621;	ZP_04294507.1	1 E-27	283/323
Bacteria	Firmicutes	Bacillus sp. SG-1	ZP_01861079.1	1 E-27	266/323
Bacteria	Firmicutes	Bacillus cereus MM3;	ZP_04300099.1	2 E-27	283/323
Bacteria	Firmicutes	Bacillus cereus Rock4-18;	ZP_04207832.1	2 E-27	283/323
Bacteria	Firmicutes	Bacillus thuringiensis serovar kurstaki	ZP_04114356.1	2 E-27	274/323
Bacteria	Firmicutes	Bacillus selenitireducens MLS10	ZP_02169903.1	2 E-27	280/323
Bacteria	Firmicutes	Bacillus thuringiensis serovar monterrey	ZP_04107847.1	2 E-27	283/323
Bacteria	Firmicutes	Bacillus cereus Rock1-15;	ZP_04238938.1	2 E-27	274/323
Bacteria	Firmicutes	Bacillus cereus ATCC 14579	NP_831550.1	2 E-27	283/323
Bacteria	Firmicutes	Bacillus thuringiensis serovar berliner	ZP_04101599.1	3 E-27	274/323
Bacteria	Firmicutes	Bacillus cereus F65185;	ZP_04202724.1	3 E-27	274/323
Bacteria	Firmicutes	Bacillus thuringiensis serovar tochigiensis	ZP_04145149.1	3 E-27	274/323
Bacteria	Firmicutes	Bacillus cereus Rock1-3;	ZP_04244731.1	3 E-27	274/323
Bacteria	Firmicutes	Bacillus thuringiensis serovar pulsiensis	ZP_04078087.1	4 E-27	283/323
Bacteria	Firmicutes	Bacillus thuringiensis serovar pakistani	ZP_04119900.1	4 E-27	274/323
Bacteria	Firmicutes	Bacillus cereus AH1273;	ZP_04174087.1	4 E-27	283/323
Bacteria	Planctomycetes	Candidatus Kuenenia stuttgartiensis	CAJ75101.1	4 E-27	274/323
Bacteria	Proteobacteria	Saccharophagus degradans 2-40	YP_528526.1	6 E-27	278/323
Bacteria	Firmicutes	Bacillus mycoides Rock1-4;	ZP_04165435.1	8 E-27	283/323
Bacteria	Firmicutes	Bacillus cereus m1550;	ZP_04278313.1	1 E-26	274/323
Bacteria	Firmicutes	Bacillus thuringiensis serovar huazhongensis	ZP_04083938.1	1 E-26	274/323
Bacteria	Firmicutes	Bacillus pseudomycoides DSM 12442	ZP_04150825.1	1 E-26	283/323
Bacteria	Verrucomicrobia	bacterium Ellin514	ZP_03632275.1	1 E-26	277/323
Bacteria	Firmicutes	Bacillus mycoides Rock3-17;	ZP_04156590.1	2 E-26	283/323
Bacteria	Proteobacteria	Nitrosomonas sp. AL212	ZP_05315101.1	2 E-26	271/323
Bacteria	Proteobacteria	Sideroxydans lithotrophicus ES-1	ZP_05340251.1	3 E-25	271/323
Bacteria	Proteobacteria	Thioalkalivibrio sp. HL-EbGR7	YP_002514768.1	1 E-24	269/323
Bacteria	Firmicutes	Listeria innocua Clip11262	NP_470996.1	1 E-24	272/323
Bacteria	Thermus	Thermus aquaticus Y51MC23	ZP_03497889.1	1 E-24	308/323
Bacteria	Proteobacteria	Rhodospirillum centenum SW	YP_002296395.1	1 E-24	272/323
Bacteria	Proteobacteria	Gallionella ferruginea ES-2	ZP_04830049.1	2 E-24	280/323

Bacteria	Thermus	Thermus thermophilus HB27	YP_005839.1	3 E-24	290/323
Bacteria	Thermus	Thermus thermophilus HB8	YP_143390.1	3 E-24	284/323
Bacteria	Firmicutes	Listeria welshimeri serovar 6b	YP_849832.1	3 E-24	272/323
Bacteria	Proteobacteria	Sorangium cellulosum 'So ce	YP_001610813.1	6 E-24	285/323
Bacteria	Proteobacteria	Comamonas testosteroni KF-1	ZP_03545752.1	7 E-24	294/323
Eukaryota	Viridiplantae	Oryza sativa Japonica Group	EEE59086.1	9 E-24	282/323
Eukaryota	Viridiplantae	Oryza sativa Indica Group	EEC75297.1	9 E-24	282/323
Eukaryota	Viridiplantae	Oryza sativa Japonica Group	ABF96063.1	1 E-23	282/323
Archaea	Euryarchaeota	Methanosphaera stadtmanae DSM 3091	YP_447078.1	3 E-23	288/323
Bacteria	Proteobacteria	Cellvibrio japonicus Ueda107	YP_001981024.1	3 E-23	284/323
Bacteria	Firmicutes	Bacillus sp. NRRL B-14911	ZP_01172509.1	3 E-23	272/323
Bacteria	Proteobacteria	Polaromonas naphthalenivorans CJ2	YP_980492.1	4 E-23	280/323
Bacteria	Firmicutes	Anoxybacillus flavithermus WK1	YP_002314437.1	4 E-23	267/323
Bacteria	Firmicutes	Listeria monocytogenes str. 4b	YP_014238.1	4 E-23	272/323
Bacteria	Proteobacteria	Teredinibacter turnerae T7901	YP_003075250.1	4 E-23	283/323
Bacteria	Proteobacteria	Dickeya dadantii Ech586	ZP_05725818.1	4 E-23	263/323
Bacteria	Candidatus	Candidatus Vesicomyosocius okutanii HA	YP_001219275.1	5 E-23	274/323
Eukaryota	Viridiplantae	Zea mays;	NP_001150217.1	5 E-23	282/323
Bacteria	Firmicutes	Bacillus pumilus SAFR-032	YP_001486160.1	6 E-23	265/323
Bacteria	Proteobacteria	gamma proteobacterium NOR51-B	ZP_04957483.1	7 E-23	285/323
Bacteria	Proteobacteria	Pectobacterium carotovorum subsp. carotovorur	YP_003018265.1	7 E-23	270/323
Bacteria	Proteobacteria	Pectobacterium carotovorum subsp. carotovorur	ZP_03831494.1	7 E-23	270/323
Bacteria	Proteobacteria	Nitrosococcus oceani ATCC 19707	YP_342199.1	8 E-23	293/323
Bacteria	Proteobacteria	Phaeobacter gallaeciensis BS107	ZP_02144680.1	9 E-23	280/323
Bacteria	Proteobacteria	Pectobacterium atrosepticum SCRI1043	YP_051062.1	9 E-23	279/323
Bacteria	Proteobacteria	Salmonella enterica subsp. enterica	YP_002228326.1	1 E-22	281/323
Bacteria	Proteobacteria	Nitrosococcus oceani AFC27	ZP_05049464.1	1 E-22	293/323
Bacteria	Proteobacteria	Shigella dysenteriae	ABD20270.1	1 E-22	281/323
Bacteria	Proteobacteria	Methylophaga thiooxidans DMS010	ZP_05105036.1	1 E-22	265/323
Bacteria	Proteobacteria	Haliangium ochraceum DSM 14365	ZP_03880572.1	1 E-22	273/323
Bacteria	Proteobacteria	Escherichia coli B7A	ZP_03027911.1	1 E-22	281/323
Bacteria	Proteobacteria	Shigella flexneri	ABD20280.1	1 E-22	281/323
Bacteria	Proteobacteria	Pectobacterium carotovorum subsp. brasiliensis	ZP_03825916.1	1 E-22	270/323
Bacteria	Proteobacteria	Kangiella koreensis DSM 16069	YP_003145523.1	1 E-22	279/323
Bacteria	Proteobacteria	Salmonella enterica subsp. enterica	ZP_02659280.1	2 E-22	281/323
Bacteria	Proteobacteria	Shigella boydii	ABD20255.1	2 E-22	281/323
Bacteria	Proteobacteria	Anaeromyxobacter dehalogenans 2CP-C	YP_465861.1	2 E-22	295/323
Bacteria	Proteobacteria	Edwardsiella ictaluri 93-146	YP_002931574.1	2 E-22	281/323
Bacteria	Chloroflexi	Chloroflexus aurantiacus J-10-fl	YP_001634125.1	2 E-22	280/323
Bacteria	Proteobacteria	Alkalilimnicola ehrlichii MLHE-1	YP_741022.1	2 E-22	269/323
Bacteria	Thermus	Meiothermus ruber DSM 1279	ZP_04037930.1	2 E-22	305/323
Bacteria	Firmicutes	Bacillus amyloliquefaciens FZB42	YP_001420585.1	2 E-22	267/323
Bacteria	Proteobacteria	Rhodobacterales bacterium HTCC2654	ZP_01012010.1	2 E-22	282/323
Bacteria	Firmicutes	Geobacillus thermodenitrificans NG80-2	YP_001124200.1	3 E-22	269/323
Bacteria	Proteobacteria	Methylobacillus flagellatus KT	YP_546604.1	3 E-22	271/323
Bacteria	Proteobacteria	Shigella flexneri	ABD20281.1	3 E-22	281/323
Bacteria	Proteobacteria	Dechloromonas aromatica RCB	YP_283519.1	3 E-22	279/323
Bacteria	Proteobacteria	Chelativorans sp. BNC1	YP_674910.1	3 E-22	276/323
Bacteria	Proteobacteria	Escherichia coli O157:H7 EDL933	NP_290401.1	3 E-22	281/323
Bacteria	Proteobacteria	Citrobacter youngae ATCC 29220	ZP_03837885.1	3 E-22	281/323
Bacteria	Proteobacteria	Pantoea sp. At-9b	ZP_05732516.1	3 E-22	281/323
Bacteria	Proteobacteria	Salmonella enterica subsp. enterica	ZP_03344552.1	3 E-22	281/323
Bacteria	Proteobacteria	Citrobacter sp. 30_2	ZP_04558376.1	4 E-22	281/323
Bacteria	Firmicutes	Listeria monocytogenes Finland 1988	ZP_03667548.1	4 E-22	272/323
Bacteria	Proteobacteria	Phaeobacter gallaeciensis 2.10	ZP_02147723.1	4 E-22	280/323
Bacteria	Firmicutes	Listeria monocytogenes EGD-e	NP_465144.1	4 E-22	272/323
Bacteria	Proteobacteria	Hahella chejuensis KCTC 2396	YP_437882.1	4 E-22	277/323
Bacteria	Proteobacteria	Chelativorans sp. BNC1	YP_674697.1	4 E-22	297/323

Bacteria	Proteobacteria	Escherichia coli	prf1104250A	4 E-22	281/323
Bacteria	Proteobacteria	Shigella boydii	ABD20248.1	4 E-22	281/323
Bacteria	Proteobacteria	Shigella boydii	ABD20247.1	4 E-22	281/323
Bacteria	Proteobacteria	Escherichia albertii TW07627	ZP_02904314.1	4 E-22	281/323
Bacteria	Bacteroidetes	Salinibacter ruber DSM 13855	YP_446418.1	4 E-22	279/323
Bacteria	Firmicutes	Bacillus weihenstephanensis KBAB4;	YP_001642963.1	4 E-22	271/323
Eukaryota	Viridiplantae	Physcomitrella patens subsp. patens	XP_001766014.1	5 E-22	287/323
Bacteria	Firmicutes	Staphylococcus carnosus subsp. carnosus	YP_002634446.1	5 E-22	266/323
Bacteria	Proteobacteria	Escherichia coli str. K-12	AAA67573.1	5 E-22	281/323
Bacteria	Proteobacteria	Salmonella enterica subsp. enterica	NP_457845.1	5 E-22	281/323
Bacteria	Proteobacteria	Shigella sp. D9	ZP_05435352.1	5 E-22	281/323

AFUA_7G07010

Eukaryota	Fungi	Aspergillus fumigatus Af293	XP_748810.1	1 E-88	157/157

AFUA_7G08340

Eukaryota	Fungi	Aspergillus fumigatus Af293	XP_748781.1	0.0	597/597
Eukaryota	Fungi	Neosartorya fischeri NRRL 181	XP_001262564.1	0.0	595/597
Eukaryota	Fungi	Neosartorya fischeri NRRL 181	XP_001258706.1	0.0	595/597
Eukaryota	Fungi	Ajellomyces capsulatus G186AR	EEH05591.1	1 E-35	545/597
Eukaryota	Fungi	Ajellomyces capsulatus H143	EER40925.1	9 E-34	585/597
Eukaryota	Fungi	Paracoccidioides brasiliensis Pb03;	EEH16503.1	2 E-28	538/597
Eukaryota	Fungi	Paracoccidioides brasiliensis Pb18;	EEH43233.1	4 E-28	538/597
Eukaryota	Fungi	Podospora anserina DSM 980	XP_001905631.1	1 E-13	534/597

AFUA_7G08350

Eukaryota	Fungi	Aspergillus fumigatus Af293	XP_748780.1	0.0	885/885
Eukaryota	Fungi	Neosartorya fischeri NRRL 181	XP_001262564.1	0.0	864/885
Eukaryota	Fungi	Sclerotinia sclerotiorum 1980 UF-70	XP_001590360.1	0.0	737/885
Eukaryota	Fungi	Aspergillus oryzae RIB40	XP_001824280.1	0.0	725/885
Eukaryota	Fungi	Botryotinia fuckeliana B05.10	XP_001547372.1	1 E-173	728/885
Eukaryota	Fungi	Chaetomium globosum CBS 148.51	XP_001226229.1	1 E-172	763/885
Eukaryota	Fungi	Neurospora crassa	CAB92025.1	1 E-166	754/885
Eukaryota	Fungi	Neurospora crassa OR74A	XP_963151.2	1 E-161	722/885
Eukaryota	Fungi	Botryotinia fuckeliana B05.10	XP_001547968.1	1 E-151	711/885

AFUA_8G00342

Eukaryota	Fungi	Aspergillus fumigatus Af293	XP_001481391.1	0.0	310/310
Eukaryota	Fungi	Neosartorya fischeri NRRL 181	XP_001266435.1	6 E-30	286/310
Eukaryota	Fungi	Aspergillus fumigatus A1163	EDP50946.1	1 E-28	294/310
Eukaryota	Fungi	Aspergillus fumigatus Af293	XP_748240.1	2 E-28	294/310
Eukaryota	Fungi	Aspergillus niger CBS 513.88	XP_001394785.1	3 E-25	292/310
Eukaryota	Fungi	Talaromyces stipitatus ATCC 10500	XP_002483112.1	4 E-24	249/310
Eukaryota	Fungi	Aspergillus clavatus NRRL 1	XP_001276429.1	5 E-22	292/310
Eukaryota	Fungi	Neosartorya fischeri NRRL 181	XP_001258320.1	1 E-19	292/310

AFUA_8G02050

Eukaryota	Fungi	Aspergillus fumigatus Af293	XP_747001.1	0.0	606/606
Eukaryota	Fungi	Ajellomyces capsulatus G186AR	EEH06769.1	1 E-134	496/606
Eukaryota	Fungi	Ajellomyces capsulatus H143	EER38435.1	1 E-134	493/606
Eukaryota	Fungi	Paracoccidioides brasiliensis Pb01;	EEH40181.1	1 E-126	500/606
Eukaryota	Fungi	Phaeosphaeria nodorum SN15	XP_001804063.1	7 E-95	487/606

AFUA_8G02060

Eukaryota	Fungi	Aspergillus fumigatus Af293	XP_747000.1	0.0	351/351
Eukaryota	Fungi	Neosartorya fischeri NRRL 181	XP_001261850.1	1 E-134	304/351
Eukaryota	Fungi	Aspergillus terreus NIH2624	XP_001211088.1	1 E-118	301/351
Eukaryota	Fungi	Aspergillus clavatus NRRL 1	XP_001275078.1	1 E-106	302/351
Eukaryota	Fungi	Aspergillus flavus NRRL3357	XP_002384801.1	4 E-88	302/351
Eukaryota	Fungi	Aspergillus flavus NRRL3357	XP_002373466.1	3 E-75	288/351
Eukaryota	Fungi	Penicillium marneffei ATCC 18224	XP_002152241.1	2 E-70	305/351
Eukaryota	Fungi	Talaromyces stipitatus ATCC 10500	XP_002485804.1	1 E-68	305/351

AFUA_8G02200

Eukaryota	Fungi	Aspergillus fumigatus Af293	XP_746986.1	0.0	548/548
Eukaryota	Fungi	Aspergillus nidulans FGSC A4	XP_659805.1	0.0	544/548
Eukaryota	Fungi	Aspergillus terreus NIH2624	XP_001209322.1	0.0	544/548
Eukaryota	Fungi	Aspergillus flavus NRRL3357	XP_002382423.1	0.0	548/548
Eukaryota	Fungi	Aspergillus oryzae RIB40	XP_001822335.1	0.0	548/548
Eukaryota	Fungi	Penicillium chrysogenum Wisconsin 54-1255	XP_002557699.1	0.0	546/548
Eukaryota	Fungi	Aspergillus niger CBS 513.88	XP_001402294.1	0.0	537/548
Eukaryota	Fungi	Aspergillus nidulans FGSC A4	XP_680511.1	0.0	546/548
Eukaryota	Fungi	Gibberella zeae PH-1	XP_389473.1	0.0	551/548
Eukaryota	Fungi	Verticillium albo-atrum VaMs.102	EEY19350.1	0.0	546/548
Eukaryota	Fungi	Nectria haematococca mpVI 77-13-4	EEU40499.1	0.0	524/548
Eukaryota	Fungi	Nectria haematococca mpVI 77-13-4	EEU33812.1	1 E-174	518/548
Eukaryota	Fungi	Nectria haematococca mpVI 77-13-4	EEU37934.1	1 E-170	525/548
Eukaryota	Fungi	Nectria haematococca mpVI 77-13-4	EEU38449.1	1 E-154	491/548
Eukaryota	Fungi	Sclerotinia sclerotiorum 1980 UF-70	XP_001598775.1	1 E-130	513/548
Eukaryota	Fungi	Botryotinia fuckeliana B05.10	XP_001552845.1	1 E-129	513/548
Eukaryota	Fungi	Microsporum canis CBS 113480	EEQ34229.1	1 E-128	529/548
Eukaryota	Fungi	Nectria haematococca mpVI 77-13-4	EEU47150.1	1 E-125	531/548
Eukaryota	Fungi	Uncinocarpus reesii 1704	XP_002544926.1	1 E-124	498/548
Eukaryota	Fungi	Paracoccidioides brasiliensis Pb01;	EEH36229.1	1 E-124	500/548
Eukaryota	Fungi	Paracoccidioides brasiliensis Pb18;	EEH45987.1	1 E-123	500/548
Eukaryota	Fungi	Paracoccidioides brasiliensis Pb03;	EEH21332.1	1 E-123	500/548
Eukaryota	Fungi	Ajellomyces capsulatus NAm1	XP_001536658.1	1 E-123	501/548
Eukaryota	Fungi	Ajellomyces capsulatus H143	EER36512.1	1 E-122	501/548
Eukaryota	Fungi	Ajellomyces capsulatus G186AR	EEH06253.1	1 E-122	501/548
Eukaryota	Fungi	Coccidioides posadasii C735 delta	EER27172.1	1 E-121	487/548
Eukaryota	Fungi	Pichia stipitis CBS 6054	XP_001383382.2	1 E-120	501/548
Eukaryota	Fungi	Debaryomyces hansenii	CAG86086.2	1 E-118	506/548
Eukaryota	Fungi	Debaryomyces hansenii CBS767	XP_458023.1	1 E-117	506/548
Eukaryota	Fungi	Pyrenophora tritici-repentis Pt-1C-BFP	XP_001940254.1	1 E-117	506/548
Eukaryota	Fungi	Neurospora crassa OR74A	XP_960312.1	1 E-116	546/548
Eukaryota	Fungi	Ajellomyces dermatitidis SLH14081	XP_002625749.1	1 E-115	501/548
Eukaryota	Fungi	Coccidioides immitis RS;	XP_001242005.1	1 E-115	491/548
Eukaryota	Fungi	Aspergillus niger CBS 513.88	XP_001401417.1	1 E-113	521/548
Eukaryota	Fungi	Talaromyces stipitatus ATCC 10500	XP_002481345.1	1 E-113	539/548
Eukaryota	Fungi	Neosartorya fischeri NRRL 181	XP_001267394.1	1 E-112	517/548
Eukaryota	Fungi	Phaeosphaeria nodorum SN15	XP_001797416.1	1 E-111	541/548
Eukaryota	Fungi	Aspergillus terreus NIH2624	XP_001214777.1	1 E-111	526/548
Eukaryota	Fungi	Penicillium marneffei ATCC 18224	XP_002147215.1	1 E-111	506/548
Eukaryota	Fungi	Phaeosphaeria nodorum SN15	XP_001801023.1	1 E-110	516/548
Eukaryota	Fungi	Nectria haematococca mpVI 77-13-4	EEU39754.1	1 E-110	492/548
Eukaryota	Fungi	Microsporum canis CBS 113480	EEQ35766.1	1 E-109	521/548
Eukaryota	Fungi	Lodderomyces elongisporus NRRL YB-4239	XP_001523779.1	1 E-109	495/548
Eukaryota	Fungi	Aspergillus nidulans FGSC A4	XP_661932.1	1 E-109	531/548
Eukaryota	Fungi	Sclerotinia sclerotiorum 1980 UF-70	XP_001584612.1	1 E-109	484/548

Eukaryota	Fungi	Aspergillus fumigatus Af293	XP_752201.1	1 E-109	499/548
Eukaryota	Fungi	Aspergillus fumigatus A1163	EDP50003.1	1 E-109	499/548
Eukaryota	Fungi	Penicillium chrysogenum Wisconsin 54-1255	XP_002565599.1	1 E-109	512/548
Eukaryota	Fungi	Aspergillus clavatus NRRL 1	XP_001271516.1	1 E-109	503/548
Eukaryota	Fungi	Aspergillus oryzae RIB40	XP_001821458.1	1 E-108	517/548
Eukaryota	Fungi	Aspergillus flavus NRRL3357	XP_002377243.1	1 E-108	517/548
Eukaryota	Fungi	Aspergillus flavus NRRL3357	XP_002377314.1	1 E-107	512/548
Eukaryota	Fungi	Neurospora crassa OR74A	XP_962197.2	1 E-107	520/548
Eukaryota	Fungi	Candida dubliniensis CD36	XP_002416912.1	1 E-107	497/548
Eukaryota	Fungi	Gibberella zeae PH-1	XP_384161.1	1 E-106	491/548
Eukaryota	Fungi	Aspergillus oryzae RIB40	XP_001825729.1	1 E-106	523/548
Eukaryota	Fungi	Pyrenophora tritici-repentis Pt-1C-BFP	XP_001931109.1	1 E-106	509/548
Eukaryota	Fungi	Chaetomium globosum CBS 148.51	XP_001223065.1	1 E-106	501/548
Eukaryota	Fungi	Nectria haematococca mpVI 77-13-4	EEU43811.1	1 E-105	504/548
Eukaryota	Fungi	Magnaporthe grisea 70-15	XP_362889.1	1 E-105	505/548
Eukaryota	Fungi	Clavispora lusitaniae ATCC 42720	XP_002616741.1	1 E-105	516/548
Eukaryota	Fungi	Pichia guilliermondii ATCC 6260	XP_001483406.1	1 E-105	503/548
Eukaryota	Fungi	Yarrowia lipolytica CLIB122	XP_501566.1	1 E-104	490/548
Eukaryota	Fungi	Aspergillus terreus NIH2624	XP_001216145.1	1 E-104	489/548
Eukaryota	Fungi	Candida albicans SC5314	XP_721444.1	1 E-103	497/548
Eukaryota	Fungi	Schizosaccharomyces japonicus yFS275	XP_002175801.1	1 E-103	494/548
Eukaryota	Fungi	Candida albicans WO-1	EEQ42902.1	1 E-103	497/548
Eukaryota	Fungi	Nectria haematococca mpVI 77-13-4	EEU33834.1	1 E-103	507/548
Eukaryota	Fungi	Lachancea thermotolerans CBS 6340	XP_002551806.1	1 E-102	496/548
Eukaryota	Fungi	Aspergillus niger CBS 513.88	XP_001396060.1	1 E-102	527/548
Eukaryota	Fungi	Aspergillus clavatus NRRL 1	XP_001272504.1	1 E-102	515/548
Eukaryota	Fungi	Schizosaccharomyces japonicus yFS275	XP_002174130.1	1 E-102	526/548
Eukaryota	Fungi	Debaryomyces hansenii	CAG88384.2	1 E-102	509/548
Eukaryota	Fungi	Debaryomyces hansenii CBS767	XP_460114.1	1 E-102	509/548
Eukaryota	Fungi	Ashbya gossypii ATCC 10895	NP_985703.1	1 E-101	515/548
Eukaryota	Fungi	Clavispora lusitaniae ATCC 42720	XP_002615908.1	1 E-100	517/548
Eukaryota	Fungi	Microsporum canis CBS 113480	EEQ28442.1	1 E-100	497/548
Eukaryota	Fungi	Coprinopsis cinerea okayama7#130	XP_001832972.1	1 E-100	526/548
Eukaryota	Fungi	Debaryomyces hansenii CBS767	XP_459958.1	1 E-100	551/548
Eukaryota	Fungi	Neurospora crassa OR74A	XP_964555.1	1 E-100	524/548
Eukaryota	Fungi	Debaryomyces hansenii CBS767	XP_457714.1	1 E-100	521/548
Eukaryota	Fungi	Debaryomyces hansenii	CAG84997.2	2 E-99	508/548
Eukaryota	Fungi	Nectria haematococca mpVI 77-13-4	EEU33610.1	2 E-99	529/548
Eukaryota	Fungi	Debaryomyces hansenii	CAG85740.2	4 E-99	521/548
Eukaryota	Fungi	Aspergillus oryzae RIB40	XP_001818914.1	6 E-99	518/548
Eukaryota	Fungi	Penicillium marneffei ATCC 18224	XP_002152185.1	8 E-99	526/548
Eukaryota	Fungi	Debaryomyces hansenii CBS767	XP_457012.1	2 E-98	508/548
Eukaryota	Fungi	Sclerotinia sclerotiorum 1980 UF-70	XP_001584822.1	2 E-98	494/548
Eukaryota	Fungi	Aspergillus flavus NRRL3357	XP_002381890.1	2 E-98	518/548
Eukaryota	Fungi	Penicillium marneffei ATCC 18224	XP_002146290.1	2 E-98	515/548
Eukaryota	Fungi	Emericella nidulans	P18696.2	3 E-98	522/548
Eukaryota	Fungi	Pyrenophora tritici-repentis Pt-1C-BFP	XP_001935450.1	5 E-98	477/548
Eukaryota	Fungi	Pichia guilliermondii ATCC 6260	XP_001485560.1	6 E-98	486/548
Eukaryota	Fungi	Phaeosphaeria nodorum SN15	XP_001796395.1	6 E-98	478/548
Eukaryota	Fungi	Mycosphaerella pini	ABS57488.1	7 E-98	537/548
Eukaryota	Fungi	Pichia pastoris GS115	XP_002493078.1	1 E-97	493/548
Eukaryota	Fungi	Aspergillus terreus NIH2624	XP_001217040.1	1 E-97	548/548
Eukaryota	Fungi	Aspergillus nidulans FGSC A4	XP_660891.1	2 E-97	506/548
Eukaryota	Fungi	Penicillium chrysogenum Wisconsin 54-1255	XP_002561023.1	3 E-97	491/548
Eukaryota	Fungi	Ajellomyces capsulatus G186AR	EEH08699.1	3 E-97	478/548
Eukaryota	Fungi	Nectria haematococca mpVI 77-13-4	EEU48917.1	4 E-97	512/548
Eukaryota	Fungi	Aspergillus niger CBS 513.88	XP_001395492.1	9 E-97	508/548
Eukaryota	Fungi	Coccidioides posadasii C735 delta	EER23868.1	1 E-96	499/548

Eukaryota	Fungi	Aspergillus oryzae RIB40	XP_001826395.1	1 E-96	495/548
Eukaryota	Fungi	Coccidioides immitis RS;	XP_001247371.1	1 E-96	499/548
Eukaryota	Fungi	Gibberella zeae PH-1	XP_384358.1	1 E-96	514/548
Eukaryota	Fungi	Aspergillus flavus NRRL3357	XP_002377589.1	1 E-96	516/548
Eukaryota	Fungi	Laccaria bicolor S238N-H82	XP_001873273.1	1 E-96	529/548
Eukaryota	Fungi	Aspergillus flavus NRRL3357	XP_002381545.1	2 E-96	507/548
Eukaryota	Fungi	Paracoccidioides brasiliensis Pb18;	EEH48482.1	3 E-96	450/548
Eukaryota	Fungi	Verticillium albo-atrum VaMs.102	EEY22799.1	4 E-96	489/548
Eukaryota	Fungi	Nectria haematococca mpVI 77-13-4	EEU49037.1	6 E-96	538/548
Eukaryota	Fungi	Penicillium chrysogenum Wisconsin 54-1255	XP_002558813.1	8 E-96	518/548
Eukaryota	Fungi	Aspergillus oryzae RIB40	XP_001825946.1	1 E-95	516/548
Eukaryota	Fungi	Ajellomyces dermatitidis SLH14081	XP_002625710.1	2 E-95	530/548
Eukaryota	Fungi	Nectria haematococca mpVI 77-13-4	EEU40630.1	2 E-95	544/548
Eukaryota	Fungi	Nectria haematococca mpVI 77-13-4	EEU38969.1	2 E-95	547/548
Eukaryota	Fungi	Lachancea thermotolerans CBS 6340	XP_002552788.1	2 E-95	499/548
Eukaryota	Fungi	Ajellomyces dermatitidis ER-3	EEQ87002.1	2 E-95	530/548
Eukaryota	Fungi	Schizosaccharomyces pombe	NP_595009.1	3 E-95	548/548
Eukaryota	Fungi	Talaromyces stipitatus ATCC 10500	XP_002478606.1	3 E-95	499/548
Eukaryota	Fungi	Aspergillus fumigatus Af293	XP_746823.1	3 E-95	501/548
Eukaryota	Fungi	Amanita muscaria	CAB38005.1	5 E-95	530/548
Eukaryota	Fungi	Candida glabrata CBS 138	XP_444823.1	8 E-95	521/548
Eukaryota	Fungi	Aspergillus terreus NIH2624	XP_001218343.1	1 E-94	503/548
Eukaryota	Fungi	Aspergillus nidulans FGSC A4	XP_682549.1	2 E-94	490/548
Eukaryota	Fungi	Lachancea kluyveri	AAO32573.1	2 E-94	532/548
Eukaryota	Fungi	Cryptococcus neoformans var. neoformans	XP_568394.1	3 E-94	512/548
Eukaryota	Fungi	Paracoccidioides brasiliensis Pb18;	EEH47577.1	4 E-94	503/548
Eukaryota	Fungi	Paracoccidioides brasiliensis Pb03;	EEH19421.1	4 E-94	503/548
Eukaryota	Fungi	Pichia pastoris GS115	XP_002490929.1	6 E-94	519/548
Eukaryota	Fungi	Nectria haematococca mpVI 77-13-4	EEU40442.1	7 E-94	491/548
Eukaryota	Fungi	Cryptococcus neoformans var. neoformans	XP_775300.1	9 E-94	512/548
Eukaryota	Fungi	Podospora anserina DSM 980	XP_001906700.1	1 E-93	488/548
Eukaryota	Fungi	Vanderwaltozyma polyspora DSM 70294	XP_001644615.1	1 E-93	514/548
Eukaryota	Fungi	Podospora anserina DSM 980	XP_001912745.1	2 E-93	496/548
Eukaryota	Fungi	Chaetomium globosum CBS 148.51	XP_001220498.1	2 E-93	489/548
Eukaryota	Fungi	Paracoccidioides brasiliensis Pb01;	EEH38246.1	3 E-93	503/548
Eukaryota	Fungi	Botryotinia fuckeliana B05.10	XP_001547953.1	4 E-93	512/548
Eukaryota	Fungi	Cryptococcus neoformans var. neoformans	XP_571087.1	4 E-93	512/548
Eukaryota	Fungi	Aspergillus niger CBS 513.88	XP_001402318.1	4 E-93	494/548
Eukaryota	Fungi	Neosartorya fischeri NRRL 181	XP_001262745.1	5 E-93	501/548
Eukaryota	Fungi	Pichia stipitis CBS 6054	XP_001387910.2	6 E-93	493/548
Eukaryota	Fungi	Nectria haematococca mpVI 77-13-4	EEU38873.1	9 E-93	491/548
Eukaryota	Fungi	Candida tropicalis MYA-3404	XP_002545259.1	1 E-92	524/548
Eukaryota	Fungi	Yarrowia lipolytica CLIB122	XP_502860.1	1 E-92	510/548
Eukaryota	Fungi	Candida tropicalis MYA-3404	XP_002545270.1	1 E-92	530/548
Eukaryota	Fungi	Candida glabrata CBS 138	XP_445933.1	2 E-92	504/548
Eukaryota	Fungi	Pichia stipitis CBS 6054	XP_001387605.2	2 E-92	542/548
Eukaryota	Fungi	Paracoccidioides brasiliensis Pb18;	EEH42620.1	3 E-92	512/548
Eukaryota	Fungi	Nectria haematococca mpVI 77-13-4	EEU35582.1	3 E-92	520/548
Eukaryota	Fungi	Aspergillus nidulans FGSC A4	XP_661480.1	4 E-92	537/548
Eukaryota	Fungi	Vanderwaltozyma polyspora DSM 70294	XP_001642644.1	5 E-92	510/548
Eukaryota	Fungi	Aspergillus niger CBS 513.88	XP_001394599.1	6 E-92	529/548
Eukaryota	Fungi	Magnaporthe grisea 70-15	XP_366823.1	1 E-91	542/548
Eukaryota	Fungi	Uromyces viciae-fabae	AAB39866.1	1 E-91	486/548
Eukaryota	Fungi	Gibberella zeae PH-1	XP_382405.1	2 E-91	512/548
Eukaryota	Fungi	Yarrowia lipolytica CLIB122	XP_500683.1	3 E-91	518/548
Eukaryota	Fungi	Microsporum canis CBS 113480	EEQ29232.1	3 E-91	520/548
Eukaryota	Fungi	Saccharomyces cerevisiae EC1118	CAY86628.1	4 E-91	506/548
Eukaryota	Fungi	Aspergillus oryzae RIB40	XP_001827162.1	4 E-91	518/548

Eukaryota	Fungi	Saccharomyces cerevisiae	NP_014993.1	5 E-91	506/548
Eukaryota	Fungi	Saccharomyces cerevisiae YJM789	EDN63668.1	5 E-91	506/548
Eukaryota	Fungi	Saccharomyces cerevisiae	AAA34925.1	5 E-91	506/548
Eukaryota	Fungi	Vanderwaltozyma polyspora DSM 70294	XP_001642910.1	7 E-91	545/548
Eukaryota	Fungi	Nectria haematococca mpVI 77-13-4	EEU44056.1	9 E-91	507/548
Eukaryota	Fungi	Botryotinia fuckeliana B05.10	XP_001557558.1	1 E-90	517/548
Eukaryota	Fungi	Penicillium chrysogenum Wisconsin 54-1255	XP_002562600.1	1 E-90	510/548
Eukaryota	Fungi	Magnaporthe grisea 70-15	XP_359649.1	1 E-90	500/548
Eukaryota	Fungi	Saccharomyces cerevisiae	NP_009690.1	1 E-90	517/548
Eukaryota	Fungi	Aspergillus clavatus NRRL 1	XP_001272431.1	1 E-90	502/548
Eukaryota	Fungi	Pyrenophora tritici-repentis Pt-1C-BFP	XP_001933097.1	1 E-90	516/548
Eukaryota	Fungi	Penicillium chrysogenum;;	AAS10167.1	2 E-90	510/548
Eukaryota	Fungi	Nectria haematococca mpVI 77-13-4	EEU38383.1	2 E-90	540/548
Eukaryota	Fungi	Saccharomyces cerevisiae RM11-1a	EDV11977.1	4 E-90	499/548
Eukaryota	Fungi	Penicillium chrysogenum Wisconsin 54-1255	XP_002568418.1	4 E-90	496/548
Eukaryota	Fungi	Saccharomyces cerevisiae AWRI1631	EDZ73751.1	5 E-90	499/548
Eukaryota	Fungi	Kluyveromyces lactis NRRL Y-1140	XP_452909.1	5 E-90	500/548
Eukaryota	Fungi	Yarrowia lipolytica CLIB122	XP_501286.1	5 E-90	524/548
Eukaryota	Fungi	Penicillium marneffei ATCC 18224	XP_002151887.1	6 E-90	507/548
Eukaryota	Fungi	Saccharomyces cerevisiae YJM789	EDN64745.1	7 E-90	497/548
Eukaryota	Fungi	Neurospora crassa OR74A	XP_001728277.1	9 E-90	488/548
Eukaryota	Fungi	Aspergillus fumigatus A1163	EDP54415.1	1 E-89	519/548
Eukaryota	Fungi	Coccidioides immitis RS;	XP_001244767.1	2 E-89	462/548
Eukaryota	Fungi	Coccidioides posadasii C735 delta	EER26053.1	2 E-89	439/548
Eukaryota	Fungi	Aspergillus fumigatus Af293	XP_755214.1	2 E-89	519/548
Eukaryota	Fungi	Uromyces viciae-fabae	CAC67419.1	3 E-89	500/548
Eukaryota	Fungi	Paracoccidioides brasiliensis Pb01;	EEH33134.1	3 E-89	511/548
Eukaryota	Fungi	Uncinocarpus reesii 1704	XP_002541563.1	3 E-89	523/548
Eukaryota	Fungi	Candida albicans WO-1	EEQ45350.1	4 E-89	511/548
Eukaryota	Fungi	Pyrenophora tritici-repentis Pt-1C-BFP	XP_001937845.1	4 E-89	550/548
Eukaryota	Fungi	Phaeosphaeria nodorum SN15	XP_001795103.1	7 E-89	492/548
Eukaryota	Fungi	Candida albicans SC5314	XP_722893.1	7 E-89	511/548
Eukaryota	Fungi	Candida dubliniensis CD36	XP_002419779.1	7 E-89	529/548
Eukaryota	Fungi	Pichia guilliermondii ATCC 6260	EDK38451.2	8 E-89	518/548
Eukaryota	Fungi	Saccharomyces cerevisiae JAY291	EEU04317.1	1 E-88	499/548
Eukaryota	Fungi	Pichia pastoris GS115	XP_002491584.1	1 E-88	480/548
Eukaryota	Fungi	Paracoccidioides brasiliensis Pb03;	EEH21760.1	1 E-88	518/548
Eukaryota	Fungi	Pyrenophora tritici-repentis Pt-1C-BFP	XP_001941691.1	1 E-88	535/548
Eukaryota	Fungi	Microsporum canis CBS 113480	EEQ35813.1	2 E-88	501/548
Eukaryota	Fungi	Ashbya gossypii ATCC 10895	NP_983269.1	2 E-88	501/548
Eukaryota	Fungi	Candida glabrata	CAG58109.2	2 E-88	516/548
Eukaryota	Fungi	Penicillium marneffei ATCC 18224	XP_002149494.1	3 E-88	507/548
Eukaryota	Fungi	Candida glabrata CBS 138	XP_445205.1	4 E-88	516/548
Eukaryota	Fungi	Nectria haematococca mpVI 77-13-4	EEU39791.1	5 E-88	502/548
Eukaryota	Fungi	Pichia guilliermondii ATCC 6260	XP_001484820.1	6 E-88	518/548
Eukaryota	Fungi	Cryptococcus neoformans var. neoformans	XP_566899.1	7 E-88	514/548
Eukaryota	Fungi	Talaromyces stipitatus ATCC 10500	XP_002478205.1	1 E-87	498/548
Eukaryota	Fungi	Aspergillus nidulans FGSC A4	XP_659336.1	2 E-87	524/548
Eukaryota	Fungi	Ustilago maydis 521	XP_759472.1	2 E-87	510/548
Eukaryota	Fungi	Ustilago maydis 521	XP_760333.1	3 E-87	514/548
Eukaryota	Fungi	Kluyveromyces lactis NRRL Y-1140	XP_456123.1	3 E-87	537/548
Eukaryota	Fungi	Ajellomyces capsulatus NAm1	XP_001543575.1	5 E-87	496/548
Eukaryota	Fungi	Neosartorya fischeri NRRL 181	XP_001266381.1	7 E-87	532/548
Eukaryota	Fungi	Hebeloma cylindrosporum	AAN52080.1	9 E-87	491/548
Eukaryota	Fungi	Pichia stipitis CBS 6054	XP_001383383.1	1 E-86	514/548
Eukaryota	Fungi	Podospora anserina DSM 980	XP_001910027.1	1 E-86	507/548
Eukaryota	Fungi	Paracoccidioides brasiliensis Pb03;	EEH22035.1	2 E-86	507/548
Eukaryota	Fungi	Saccharomyces cerevisiae YJM789	EDN60883.1	2 E-86	518/548

Eukaryota	Fungi	Penicillium marneffei ATCC 18224	XP_002149857.1	2 E-86	526/548
Eukaryota	Fungi	Saccharomyces cerevisiae	NP_015058.1	2 E-86	518/548
Eukaryota	Fungi	Saccharomyces cerevisiae JAY291	EEU06255.1	3 E-86	518/548
Eukaryota	Fungi	Aspergillus clavatus NRRL 1	XP_001267807.1	3 E-86	539/548
Eukaryota	Fungi	Paracoccidioides brasiliensis Pb01;	EEH33409.1	3 E-86	509/548
Eukaryota	Fungi	Saccharomyces cerevisiae EC1118	CAY86695.1	3 E-86	518/548
Eukaryota	Fungi	Paracoccidioides brasiliensis Pb18;	EEH48536.1	4 E-86	507/548
Eukaryota	Fungi	Talaromyces stipitatus ATCC 10500	XP_002485074.1	7 E-86	517/548
Eukaryota	Fungi	Aspergillus nidulans FGSC A4	XP_663722.1	8 E-86	514/548
Eukaryota	Fungi	Aspergillus nidulans FGSC A4	CBF70149.1	9 E-86	514/548
Eukaryota	Fungi	Aspergillus nidulans FGSC A4	ACC77607.1	9 E-86	514/548
Eukaryota	Fungi	Aspergillus oryzae RIB40	XP_001823622.1	1 E-85	547/548
Eukaryota	Fungi	Gibberella zeae PH-1	XP_384231.1	1 E-85	511/548
Eukaryota	Fungi	Lachancea thermotolerans CBS 6340	XP_002551842.1	1 E-85	492/548
Eukaryota	Fungi	Magnaporthe grisea 70-15	XP_369428.2	1 E-85	498/548
Eukaryota	Fungi	Aspergillus terreus NIH2624	XP_001217244.1	2 E-85	502/548
Eukaryota	Fungi	Talaromyces stipitatus ATCC 10500	XP_002480868.1	2 E-85	509/548
Eukaryota	Fungi	Ustilago maydis 521	XP_761416.1	2 E-85	538/548
Eukaryota	Fungi	Pichia guilliermondii ATCC 6260	EDK39820.2	3 E-85	536/548
Eukaryota	Fungi	Aspergillus flavus NRRL3357	XP_002378085.1	3 E-85	461/548
Eukaryota	Fungi	Clavispora lusitaniae ATCC 42720	XP_002618560.1	3 E-85	493/548
Eukaryota	Fungi	Kluyveromyces lactis NRRL Y-1140	XP_454683.1	5 E-85	510/548
Eukaryota	Fungi	Pichia guilliermondii ATCC 6260	XP_001484537.1	7 E-85	536/548

AFUA_8G02230

Eukaryota	Fungi	Aspergillus fumigatus Af293	XP_746982.1	1 E-118	217/217

AFUA_8G02250

Eukaryota	Fungi	Aspergillus fumigatus Af293	XP_746981.1	2 E-60	115/115
Eukaryota	Fungi	Neosartorya fischeri NRRL 181	XP_001261859.1	3 E-54	115/115
Eukaryota	Fungi	Aspergillus clavatus NRRL 1	XP_001275044.1	2 E-41	119/115

AFUA_8G02260

Eukaryota	Fungi	Aspergillus fumigatus Af293	XP_746980.1	0.0	463/463
Eukaryota	Fungi	Aspergillus flavus NRRL3357	XP_002372802.1	1 E-160	463/463
Eukaryota	Fungi	Penicillium chrysogenum Wisconsin 54-1255	XP_002564799.1	1 E-159	433/463
Eukaryota	Fungi	Aspergillus flavus NRRL3357	XP_002380070.1	1 E-158	417/463
Eukaryota	Fungi	Penicillium chrysogenum Wisconsin 54-1255	XP_002563523.1	1 E-153	446/463
Eukaryota	Fungi	Aspergillus nidulans FGSC A4	XP_659109.1	1 E-153	439/463
Eukaryota	Fungi	Aspergillus terreus NIH2624	XP_001216922.1	1 E-151	428/463
Eukaryota	Fungi	Aspergillus clavatus NRRL 1	XP_001276614.1	1 E-150	453/463
Eukaryota	Fungi	Aspergillus niger CBS 513.88	XP_001398067.1	1 E-149	440/463
Eukaryota	Fungi	Aspergillus fumigatus Af293	XP_747312.1	1 E-149	432/463
Eukaryota	Fungi	Aspergillus nidulans FGSC A4	XP_682302.1	1 E-149	420/463
Eukaryota	Fungi	Aspergillus terreus NIH2624	XP_001210212.1	1 E-149	441/463
Eukaryota	Fungi	Uncinocarpus reesii 1704	XP_002583122.1	1 E-144	444/463
Eukaryota	Fungi	Microsporum canis CBS 113480	EEQ34212.1	1 E-140	455/463
Eukaryota	Fungi	Aspergillus nidulans FGSC A4	XP_660811.1	1 E-139	426/463
Eukaryota	Fungi	Ajellomyces dermatitidis ER-3	EEQ87125.1	1 E-138	438/463
Eukaryota	Fungi	Ajellomyces dermatitidis SLH14081	XP_002625589.1	1 E-137	438/463
Eukaryota	Fungi	Aspergillus flavus NRRL3357	XP_002381601.1	1 E-136	445/463
Eukaryota	Fungi	Penicillium chrysogenum Wisconsin 54-1255	XP_002556987.1	1 E-136	459/463
Eukaryota	Fungi	Neosartorya fischeri NRRL 181	XP_001261225.1	1 E-135	442/463
Eukaryota	Fungi	Aspergillus terreus NIH2624	XP_001209309.1	1 E-135	446/463
Eukaryota	Fungi	Aspergillus niger CBS 513.88	XP_001402298.1	1 E-133	439/463

Eukaryota	Fungi	Aspergillus niger CBS 513.88	XP_001390417.1	1 E-133	435/463
Eukaryota	Fungi	Aspergillus clavatus NRRL 1	XP_001275190.1	1 E-133	431/463
Eukaryota	Fungi	Aspergillus fumigatus Af293	XP_756083.1	1 E-132	457/463
Eukaryota	Fungi	Penicillium chrysogenum Wisconsin 54-1255	XP_002563823.1	1 E-132	452/463
Eukaryota	Fungi	Aspergillus clavatus NRRL 1	XP_001270430.1	1 E-132	438/463
Eukaryota	Fungi	Aspergillus niger CBS 513.88	XP_001400898.1	1 E-132	445/463
Eukaryota	Fungi	Aspergillus fumigatus Af293	XP_749154.1	1 E-131	441/463
Eukaryota	Fungi	Neosartorya fischeri NRRL 181	XP_001265570.1	1 E-130	441/463
Eukaryota	Fungi	Aspergillus clavatus NRRL 1	XP_001276025.1	1 E-129	442/463
Eukaryota	Fungi	Neosartorya fischeri NRRL 181	XP_001261692.1	1 E-129	443/463
Eukaryota	Fungi	Coccidioides posadasii C735 delta	EER24800.1	1 E-128	446/463
Eukaryota	Fungi	Coccidioides immitis RS;	XP_001243460.1	1 E-128	457/463
Eukaryota	Fungi	Aspergillus clavatus NRRL 1	XP_001275060.1	1 E-128	438/463
Eukaryota	Fungi	Aspergillus fumigatus Af293	XP_755881.2	1 E-127	429/463
Eukaryota	Fungi	Aspergillus niger CBS 513.88	XP_001391581.1	1 E-127	436/463
Eukaryota	Fungi	Penicillium chrysogenum Wisconsin 54-1255	XP_002564678.1	1 E-127	434/463
Eukaryota	Fungi	Aspergillus flavus NRRL3357	XP_002383574.1	1 E-126	436/463
Eukaryota	Fungi	Aspergillus niger CBS 513.88	XP_001401336.1	1 E-126	436/463
Eukaryota	Fungi	Aspergillus niger CBS 513.88	XP_001397083.1	1 E-126	445/463
Eukaryota	Fungi	Aspergillus terreus NIH2624	XP_001216539.1	1 E-126	441/463
Eukaryota	Fungi	Penicillium chrysogenum Wisconsin 54-1255	XP_002564689.1	1 E-125	456/463
Eukaryota	Fungi	Aspergillus nidulans FGSC A4	CBF83940.1	1 E-122	451/463
Eukaryota	Fungi	Sclerotinia sclerotiorum 1980 UF-70	XP_001597608.1	1 E-122	459/463
Eukaryota	Fungi	Aspergillus flavus NRRL3357	XP_002375378.1	1 E-122	443/463
Eukaryota	Fungi	Botryotinia fuckeliana B05.10	XP_001558954.1	1 E-120	430/463
Eukaryota	Fungi	Aspergillus terreus NIH2624	XP_001211099.1	1 E-119	447/463
Eukaryota	Fungi	Aspergillus fumigatus A1163	EDP49136.1	1 E-118	392/463
Eukaryota	Fungi	Ajellomyces capsulatus G186AR	EEH06112.1	1 E-118	448/463
Eukaryota	Fungi	Aspergillus niger CBS 513.88	XP_001397349.1	1 E-117	400/463
Eukaryota	Fungi	Neosartorya fischeri NRRL 181	XP_001267441.1	1 E-117	420/463
Eukaryota	Fungi	Aspergillus terreus NIH2624	XP_001211587.1	1 E-117	412/463
Eukaryota	Fungi	Ajellomyces capsulatus H143	EER37576.1	1 E-117	448/463
Eukaryota	Fungi	Aspergillus fumigatus Af293	XP_747133.2	1 E-116	392/463
Eukaryota	Fungi	Penicillium chrysogenum Wisconsin 54-1255	XP_002560554.1	1 E-115	423/463
Eukaryota	Fungi	Penicillium chrysogenum Wisconsin 54-1255	XP_002564177.1	1 E-114	456/463
Eukaryota	Fungi	Aspergillus nidulans FGSC A4	XP_660425.1	1 E-114	422/463
Eukaryota	Fungi	Ajellomyces capsulatus NAm1	XP_001536747.1	1 E-108	395/463
Eukaryota	Fungi	Aspergillus flavus NRRL3357	XP_002375403.1	1 E-107	408/463
Eukaryota	Fungi	Aspergillus oryzae RIB40	XP_001727164.1	1 E-107	439/463
Eukaryota	Fungi	Pyrenophora tritici-repentis Pt-1C-BFP	XP_001934474.1	1 E-106	422/463
Eukaryota	Fungi	Chaetomium globosum CBS 148.51	XP_001224570.1	3 E-97	449/463
Eukaryota	Fungi	Phaeosphaeria nodorum SN15	XP_001795452.1	9 E-97	373/463
Eukaryota	Fungi	Fusarium proliferatum	AAY68036.1	2 E-96	460/463
Eukaryota	Fungi	Verticillium albo-atrum VaMs.102	EEY23387.1	2 E-94	434/463
Eukaryota	Fungi	Gibberella zeae PH-1	XP_388231.1	3 E-94	460/463
Eukaryota	Fungi	Magnaporthe grisea 70-15	XP_001405206.1	9 E-94	462/463
Eukaryota	Fungi	Nectria haematococca mpVI 77-13-4	EEU35905.1	3 E-89	458/463
Eukaryota	Fungi	Verticillium albo-atrum VaMs.102	EEY19358.1	2 E-74	424/463
Eukaryota	Fungi	Pyrenophora tritici-repentis Pt-1C-BFP	XP_001932043.1	1 E-71	463/463
Eukaryota	Fungi	Ustilago maydis 521	XP_756203.1	2 E-68	448/463
Eukaryota	Fungi	Aspergillus oryzae RIB40	XP_001827500.1	3 E-66	463/463
Eukaryota	Fungi	Chaetomium globosum CBS 148.51	XP_001223400.1	2 E-65	422/463
Eukaryota	Fungi	Talaromyces stipitatus ATCC 10500	XP_002477945.1	3 E-65	446/463
Eukaryota	Fungi	Gibberella zeae PH-1	XP_388185.1	5 E-65	457/463
Eukaryota	Fungi	Penicillium marneffei ATCC 18224	XP_002145685.1	1 E-64	445/463
Eukaryota	Fungi	Neurospora crassa OR74A	XP_962361.1	1 E-64	442/463
Eukaryota	Fungi	Gibberella zeae PH-1	XP_389215.1	2 E-64	436/463
Eukaryota	Fungi	Nectria haematococca mpVI 77-13-4	EEU41358.1	7 E-64	442/463

Eukaryota	Fungi	Penicillium chrysogenum Wisconsin 54-1255	XP_002564066.1	1 E-62	449/463
Eukaryota	Fungi	Penicillium chrysogenum;;	AAT45727.1	2 E-62	455/463
Eukaryota	Fungi	Aspergillus flavus NRRL3357	XP_002372191.1	2 E-61	426/463
Eukaryota	Fungi	Aspergillus nidulans FGSC A4	XP_681295.1	2 E-61	392/463
Eukaryota	Fungi	Gibberella zeae PH-1	XP_382052.1	4 E-61	406/463
Eukaryota	Fungi	Aspergillus niger CBS 513.88	XP_001397896.1	5 E-61	419/463
Eukaryota	Fungi	Aspergillus flavus NRRL3357	XP_002384609.1	6 E-61	459/463
Eukaryota	Fungi	Pyrenophora tritici-repentis Pt-1C-BFP	XP_001938799.1	8 E-61	379/463
Eukaryota	Fungi	Nectria haematococca mpVI 77-13-4	EEU34949.1	9 E-61	411/463
Eukaryota	Fungi	Nectria haematococca mpVI 77-13-4	EEU42590.1	9 E-61	431/463
Eukaryota	Fungi	Nectria haematococca mpVI 77-13-4	EEU38048.1	1 E-60	434/463
Eukaryota	Fungi	Aspergillus nidulans FGSC A4	XP_664046.1	2 E-60	422/463
Eukaryota	Fungi	Aspergillus terreus NIH2624	XP_001208586.1	2 E-60	463/463
Eukaryota	Fungi	Gibberella zeae PH-1	XP_383126.1	2 E-60	446/463
Eukaryota	Fungi	Phaeosphaeria nodorum SN15	XP_001804605.1	4 E-60	445/463
Eukaryota	Fungi	Magnaporthe grisea 70-15	XP_363011.1	5 E-60	417/463
Eukaryota	Fungi	Nectria haematococca mpVI 77-13-4	EEU38442.1	5 E-60	422/463
Eukaryota	Fungi	Cryptococcus neoformans var. neoformans	XP_571700.1	6 E-60	412/463
Eukaryota	Fungi	Aspergillus oryzae RIB40	XP_001823549.1	7 E-60	439/463
Eukaryota	Fungi	Cryptococcus neoformans var. neoformans	XP_775019.1	1 E-59	412/463
Eukaryota	Fungi	Nectria haematococca mpVI 77-13-4	EEU35640.1	1 E-59	443/463
Eukaryota	Fungi	Neosartorya fischeri NRRL 181	XP_001262261.1	1 E-59	424/463
Eukaryota	Fungi	Penicillium chrysogenum Wisconsin 54-1255	XP_002563563.1	2 E-59	392/463
Eukaryota	Fungi	Gibberella zeae PH-1	XP_380312.1	2 E-59	470/463
Eukaryota	Fungi	Aspergillus flavus NRRL3357	XP_002378956.1	3 E-59	437/463
Eukaryota	Fungi	Penicillium chrysogenum Wisconsin 54-1255	XP_002568082.1	6 E-59	446/463
Eukaryota	Fungi	Nectria haematococca mpVI 77-13-4	EEU44002.1	6 E-59	404/463
Eukaryota	Fungi	Nectria haematococca mpVI 77-13-4	EEU43545.1	6 E-59	413/463
Eukaryota	Fungi	Gibberella zeae PH-1	XP_386833.1	7 E-59	384/463
Eukaryota	Fungi	Nectria haematococca mpVI 77-13-4	EEU39775.1	2 E-58	453/463
Eukaryota	Fungi	Aspergillus niger CBS 513.88	XP_001395627.1	3 E-58	471/463
Eukaryota	Fungi	Aspergillus fumigatus A1163	EDP48621.1	5 E-58	424/463
Eukaryota	Fungi	Penicillium chrysogenum Wisconsin 54-1255	XP_002568681.1	7 E-58	455/463
Eukaryota	Fungi	Aspergillus fumigatus Af293	XP_747518.2	8 E-58	424/463
Eukaryota	Fungi	Penicillium chrysogenum Wisconsin 54-1255	XP_002564751.1	8 E-58	393/463
Eukaryota	Fungi	Nectria haematococca mpVI 77-13-4	EEU41331.1	9 E-58	463/463
Eukaryota	Fungi	Penicillium chrysogenum Wisconsin 54-1255	XP_002564208.1	2 E-57	452/463
Eukaryota	Fungi	Sclerotinia sclerotiorum 1980 UF-70	XP_001592521.1	2 E-57	421/463
Eukaryota	Fungi	Ustilago maydis 521	XP_758606.1	2 E-57	455/463
Eukaryota	Fungi	Aspergillus oryzae RIB40	XP_001822886.1	3 E-57	410/463
Eukaryota	Fungi	Verticillium albo-atrum VaMs.102	EEY16576.1	6 E-57	425/463
Eukaryota	Fungi	Aspergillus niger CBS 513.88	XP_001397276.1	7 E-57	423/463
Eukaryota	Fungi	Aspergillus oryzae RIB40	XP_001817172.1	1 E-56	450/463
Eukaryota	Fungi	Gibberella zeae PH-1	XP_384644.1	1 E-56	466/463
Eukaryota	Fungi	Aspergillus flavus NRRL3357	XP_002372210.1	2 E-56	450/463
Eukaryota	Fungi	Gibberella zeae PH-1	XP_380294.1	2 E-56	459/463
Eukaryota	Fungi	Aspergillus terreus NIH2624	XP_001208557.1	3 E-56	410/463
Eukaryota	Fungi	Magnaporthe grisea 70-15	XP_370027.1	3 E-56	446/463
Eukaryota	Fungi	Aspergillus flavus NRRL3357	XP_002383608.1	4 E-56	404/463
Eukaryota	Fungi	Phaeosphaeria nodorum SN15	XP_001793107.1	1 E-55	392/463
Eukaryota	Fungi	Nectria haematococca mpVI 77-13-4	EEU45344.1	1 E-55	384/463
Eukaryota	Fungi	Penicillium chrysogenum Wisconsin 54-1255	XP_002560361.1	2 E-55	425/463
Eukaryota	Fungi	Nectria haematococca mpVI 77-13-4	EEU35241.1	2 E-55	433/463
Eukaryota	Fungi	Gibberella zeae PH-1	XP_389254.1	5 E-55	473/463
Eukaryota	Fungi	Aspergillus flavus NRRL3357	XP_002384015.1	5 E-55	437/463
Eukaryota	Fungi	Aspergillus clavatus NRRL 1	XP_001276769.1	1 E-54	435/463
Eukaryota	Fungi	Gibberella zeae PH-1	XP_386713.1	2 E-54	455/463
Eukaryota	Fungi	Nectria haematococca mpVI 77-13-4	EEU46734.1	2 E-54	415/463

Eukaryota	Fungi	Nectria haematococca mpVI 77-13-4	EEU37819.1	3 E-54	452/463
Eukaryota	Fungi	Gibberella zeae PH-1	XP_387986.1	1 E-53	478/463
Eukaryota	Fungi	Kluyveromyces lactis NRRL Y-1140	XP_455272.1	4 E-53	474/463
Eukaryota	Fungi	Aspergillus terreus NIH2624	XP_001212662.1	5 E-53	422/463
Eukaryota	Fungi	Aspergillus flavus NRRL3357	XP_002382262.1	5 E-53	439/463
Eukaryota	Fungi	Botryotinia fuckeliana B05.10	XP_001558429.1	1 E-52	377/463
Eukaryota	Fungi	Aspergillus niger CBS 513.88	XP_001393683.1	2 E-52	372/463
Eukaryota	Fungi	Penicillium chrysogenum Wisconsin 54-1255	XP_002569238.1	4 E-52	406/463
Eukaryota	Fungi	Aspergillus flavus NRRL3357	XP_002384713.1	7 E-52	402/463
Eukaryota	Fungi	Aspergillus oryzae RIB40	XP_001827503.1	1 E-51	402/463
Eukaryota	Fungi	Nectria haematococca mpVI 77-13-4	EEU45336.1	2 E-51	463/463
Eukaryota	Fungi	Nectria haematococca mpVI 77-13-4	EEU41062.1	6 E-51	392/463
Eukaryota	Fungi	Debaryomyces hansenii	CAG90021.2	8 E-51	477/463
Eukaryota	Fungi	Debaryomyces hansenii CBS767	XP_461575.1	2 E-50	476/463
Eukaryota	Fungi	Nectria haematococca mpVI 77-13-4	EEU42715.1	6 E-50	398/463
Eukaryota	Fungi	Aspergillus clavatus NRRL 1	XP_001269185.1	9 E-50	475/463
Eukaryota	Fungi	Aspergillus flavus NRRL3357	XP_002383497.1	2 E-49	465/463
Eukaryota	Fungi	Nectria haematococca mpVI 77-13-4	EEU43540.1	2 E-49	395/463
Eukaryota	Fungi	Kluyveromyces lactis NRRL Y-1140	XP_455267.1	2 E-49	466/463
Eukaryota	Fungi	Cryptococcus neoformans var. neoformans	XP_571440.1	3 E-49	462/463
Eukaryota	Fungi	Gibberella zeae PH-1	XP_389530.1	4 E-49	403/463
Eukaryota	Fungi	Nectria haematococca mpVI 77-13-4	EEU33828.1	5 E-49	377/463
Eukaryota	Fungi	Pyrenophora tritici-repentis Pt-1C-BFP	XP_001932783.1	8 E-49	430/463
Eukaryota	Fungi	Yarrowia lipolytica CLIB122	XP_504271.1	8 E-49	465/463
Eukaryota	Fungi	Verticillium albo-atrum VaMs.102	EEY20248.1	2 E-48	395/463
Eukaryota	Fungi	Gibberella zeae PH-1	XP_383692.1	2 E-48	410/463
Eukaryota	Fungi	Gibberella zeae PH-1	XP_391450.1	2 E-48	410/463
Eukaryota	Fungi	Aspergillus terreus NIH2624	XP_001210355.1	6 E-48	458/463
Eukaryota	Fungi	Nectria haematococca mpVI 77-13-4	EEU45345.1	7 E-48	376/463
Eukaryota	Fungi	Malassezia globosa CBS 7966	XP_001731671.1	9 E-48	449/463
Eukaryota	Fungi	Aspergillus nidulans FGSC A4	XP_657614.1	1 E-47	456/463
Eukaryota	Fungi	Talaromyces stipitatus ATCC 10500	XP_002341507.1	2 E-47	403/463
Eukaryota	Fungi	Aspergillus oryzae RIB40	XP_001825274.1	3 E-47	474/463
Eukaryota	Fungi	Gibberella zeae PH-1	XP_380252.1	8 E-47	396/463
Eukaryota	Fungi	Aspergillus fumigatus A1163	EDP53646.1	1 E-46	391/463
Eukaryota	Fungi	Aspergillus fumigatus Af293	XP_748333.1	2 E-46	391/463
Eukaryota	Fungi	Nectria haematococca mpVI 77-13-4	EEU45615.1	2 E-46	429/463
Eukaryota	Fungi	Candida tropicalis MYA-3404	XP_002548262.1	2 E-46	386/463
Eukaryota	Fungi	Neosartorya fischeri NRRL 181	XP_001259777.1	3 E-46	438/463
Eukaryota	Fungi	Nectria haematococca mpVI 77-13-4	EEU46185.1	7 E-46	416/463
Eukaryota	Fungi	Aspergillus niger CBS 513.88	XP_001391479.1	1 E-45	405/463
Eukaryota	Fungi	Aspergillus fumigatus Af293	XP_753726.1	2 E-45	438/463
Eukaryota	Fungi	Gibberella zeae PH-1	XP_383954.1	2 E-45	395/463
Eukaryota	Fungi	Penicillium marneffei ATCC 18224	XP_002151400.1	2 E-45	458/463
Eukaryota	Fungi	Aspergillus fumigatus Af293	XP_752442.1	5 E-45	470/463
Eukaryota	Fungi	Phaeosphaeria nodorum SN15	XP_001792042.1	5 E-45	434/463
Eukaryota	Fungi	Aspergillus fumigatus A1163	EDP56310.1	6 E-45	470/463
Eukaryota	Fungi	Nectria haematococca mpVI 77-13-4	EEU42678.1	9 E-45	416/463
Eukaryota	Fungi	Nectria haematococca mpVI 77-13-4	EEU46700.1	1 E-44	462/463
Eukaryota	Fungi	Nectria haematococca mpVI 77-13-4	EEU35758.1	1 E-44	450/463
Eukaryota	Fungi	Aspergillus niger CBS 513.88	XP_001392407.1	2 E-44	413/463
Eukaryota	Fungi	Aspergillus terreus NIH2624	XP_001209280.1	2 E-44	459/463
Eukaryota	Fungi	Gibberella zeae PH-1	XP_390551.1	2 E-44	440/463
Eukaryota	Fungi	Neosartorya fischeri NRRL 181	XP_001264692.1	3 E-44	470/463
Eukaryota	Fungi	Penicillium chrysogenum Wisconsin 54-1255	XP_002561032.1	3 E-44	457/463
Eukaryota	Fungi	Aspergillus nidulans FGSC A4	XP_658803.1	5 E-44	448/463
Eukaryota	Fungi	Pichia guilliermondii ATCC 6260	XP_001486466.1	5 E-44	429/463
Eukaryota	Fungi	Pichia guilliermondii ATCC 6260	EDK38039.2	2 E-43	429/463

Eukaryota	Fungi	Ajellomyces capsulatus G186AR	EEH05186.1	4 E-43	450/463
Eukaryota	Fungi	Aspergillus oryzae RIB40	XP_001823379.1	4 E-43	397/463
Eukaryota	Fungi	Microsporum canis CBS 113480	EEQ28743.1	8 E-43	380/463
Eukaryota	Fungi	Cryptococcus neoformans var. neoformans	XP_571577.1	1 E-42	380/463
Eukaryota	Fungi	Nectria haematococca mpVI 77-13-4	EEU34668.1	2 E-42	386/463
Eukaryota	Fungi	Ajellomyces capsulatus G186AR	EEH08289.1	2 E-41	479/463
Eukaryota	Fungi	Aspergillus terreus NIH2624	XP_001215734.1	2 E-41	378/463
Eukaryota	Fungi	Lachancea thermotolerans CBS 6340	XP_002551828.1	3 E-41	463/463
Eukaryota	Fungi	Aspergillus nidulans FGSC A4	CBF88138.1	4 E-41	456/463
Eukaryota	Fungi	Verticillium albo-atrum VaMs.102	EEY15986.1	4 E-41	446/463
Eukaryota	Fungi	Ajellomyces capsulatus H143	EER40943.1	4 E-41	387/463
Eukaryota	Fungi	Ajellomyces capsulatus NAm1	XP_001543262.1	6 E-41	387/463
Eukaryota	Fungi	Aspergillus nidulans FGSC A4	XP_658705.1	7 E-41	396/463
Eukaryota	Fungi	Ajellomyces capsulatus H143	EER39635.1	1 E-40	414/463
Eukaryota	Fungi	Candida tropicalis MYA-3404	XP_002548261.1	1 E-40	371/463
Eukaryota	Fungi	Aspergillus flavus NRRL3357	XP_002378764.1	1 E-40	402/463
Eukaryota	Fungi	Pichia stipitis CBS 6054	XP_001385206.1	5 E-40	379/463
Eukaryota	Fungi	Pichia guilliermondii ATCC 6260	XP_001482680.1	1 E-39	474/463
Eukaryota	Fungi	Lodderomyces elongisporus NRRL YB-4239	XP_001527272.1	2 E-39	372/463
Eukaryota	Fungi	Penicillium chrysogenum Wisconsin 54-1255	XP_002561723.1	3 E-39	433/463
Eukaryota	Fungi	Zygosaccharomyces rouxii CBS 732	XP_002498965.1	4 E-39	468/463
Eukaryota	Fungi	Pyrenophora tritici-repentis Pt-1C-BFP	XP_001932773.1	5 E-39	408/463
Eukaryota	Fungi	Phaeosphaeria nodorum SN15	XP_001797147.1	9 E-39	388/463
Eukaryota	Fungi	Phaeosphaeria nodorum SN15	XP_001802226.1	1 E-38	445/463
Eukaryota	Fungi	Debaryomyces hansenii CBS767	XP_457753.1	3 E-38	433/463

AFUA_8G02810

Eukaryota	Fungi	Aspergillus fumigatus Af293	XP_746926.1	0.0	696/696
Eukaryota	Fungi	Neosartorya fischeri NRRL 181	XP_001261926.1	0.0	696/696
Eukaryota	Fungi	Aspergillus clavatus NRRL 1	XP_001274998.1	0.0	697/696
Eukaryota	Fungi	Aspergillus terreus NIH2624	XP_001218743.1	0.0	670/696
Eukaryota	Fungi	Aspergillus niger CBS 513.88	XP_001390568.1	0.0	697/696
Eukaryota	Fungi	Aspergillus oryzae RIB40	XP_001827723.1	0.0	698/696
Eukaryota	Fungi	Penicillium chrysogenum Wisconsin 54-1255	XP_002560533.1	0.0	693/696
Eukaryota	Fungi	Ajellomyces dermatitidis SLH14081	XP_002628889.1	0.0	718/696
Eukaryota	Fungi	Ajellomyces capsulatus H143	EER42399.1	0.0	720/696
Eukaryota	Fungi	Ajellomyces capsulatus G186AR	EEH08427.1	0.0	720/696
Eukaryota	Fungi	Ajellomyces capsulatus NAm1	XP_001544839.1	0.0	720/696
Eukaryota	Fungi	Penicillium marneffei ATCC 18224	XP_002150896.1	0.0	744/696
Eukaryota	Fungi	Paracoccidioides brasiliensis Pb03;	EEH21583.1	0.0	736/696
Eukaryota	Fungi	Talaromyces stipitatus ATCC 10500	XP_002483182.1	0.0	747/696
Eukaryota	Fungi	Paracoccidioides brasiliensis Pb01;	EEH39296.1	0.0	709/696
Eukaryota	Fungi	Microsporum canis CBS 113480	EEQ34709.1	0.0	724/696
Eukaryota	Fungi	Uncinocarpus reesii 1704	XP_002541923.1	0.0	711/696
Eukaryota	Fungi	Coccidioides posadasii C735 delta	EER28690.1	0.0	712/696
Eukaryota	Fungi	Coccidioides immitis RS;	XP_001248525.1	0.0	705/696
Eukaryota	Fungi	Paracoccidioides brasiliensis Pb18;	EEH43730.1	0.0	709/696
Eukaryota	Fungi	Ajellomyces dermatitidis ER-3	EEQ85277.1	0.0	673/696
Eukaryota	Fungi	Aspergillus flavus NRRL3357	XP_002384974.1	0.0	593/696
Eukaryota	Fungi	Phaeosphaeria nodorum SN15	XP_001806648.1	0.0	705/696
Eukaryota	Fungi	Sclerotinia sclerotiorum 1980 UF-70	XP_001591128.1	0.0	729/696
Eukaryota	Fungi	Pyrenophora tritici-repentis Pt-1C-BFP	XP_001938452.1	0.0	689/696
Eukaryota	Fungi	Magnaporthe grisea 70-15	XP_365099.1	0.0	742/696
Eukaryota	Fungi	Nectria haematococca mpVI 77-13-4	EEU46460.1	0.0	715/696
Eukaryota	Fungi	Gibberella zeae PH-1	XP_387035.1	0.0	714/696
Eukaryota	Fungi	Neurospora crassa OR74A	XP_964417.1	0.0	769/696
Eukaryota	Fungi	Botryotinia fuckeliana B05.10	XP_001547960.1	0.0	675/696

Eukaryota	Fungi	Podospora anserina DSM 980	XP_001912486.1	0.0	739/696
Eukaryota	Fungi	Verticillium albo-atrum VaMs.102	EEY18554.1	1 E-152	626/696
Eukaryota	Fungi	Ustilago maydis 521	XP_760641.1	2 E-75	685/696
Eukaryota	Fungi	Laccaria bicolor S238N-H82	XP_001874576.1	3 E-71	679/696
Eukaryota	Metazoa	Pediculus humanus corporis	XP_002427823.1	6 E-26	606/696
Eukaryota	Metazoa	Macaca mulatta	XP_001101873.1	1 E-24	628/696
Eukaryota	Metazoa	Pan troglodytes	XP_001170150.1	1 E-24	624/696
Eukaryota	Metazoa	Tribolium castaneum	XP_969248.1	2 E-24	596/696
Eukaryota	Metazoa	Pan troglodytes	XP_001170171.1	3 E-24	628/696
Eukaryota	Metazoa	Macaca mulatta	XP_001102332.1	1 E-23	636/696
Eukaryota	Metazoa	Canis lupus familiaris	XP_865674.1	1 E-23	628/696
Eukaryota	Metazoa	Homo sapiens	EAW94079.1	2 E-23	584/696
Eukaryota	Metazoa	Homo sapiens	CAB66721.1	2 E-23	636/696
Eukaryota	Metazoa	Xenopus laevis	NP_001089652.1	3 E-23	636/696
Eukaryota	Metazoa	Mus musculus	BAD90178.1	3 E-23	636/696
Eukaryota	Metazoa	Gallus gallus	XP_417479.2	3 E-23	644/696
Eukaryota	Metazoa	Gallus gallus	NP_001026165.1	4 E-23	640/696
Eukaryota	Metazoa	Mus musculus	NP_525027.1	4 E-23	636/696
Eukaryota	Metazoa	Homo sapiens	NP_055615.8	4 E-23	636/696
Eukaryota	Metazoa	Mus musculus	EDL32679.1	4 E-23	636/696
Eukaryota	Metazoa	Mus musculus	EDL32678.1	4 E-23	636/696
Eukaryota	Metazoa	Homo sapiens	BAG58968.1	5 E-23	613/696
Eukaryota	Fungi	Yarrowia lipolytica CLIB122	XP_500993.1	5 E-23	681/696
Eukaryota	Metazoa	Bos taurus	NP_001106698.1	8 E-23	636/696
Eukaryota	Metazoa	Equus caballus	XP_001501313.1	1 E-22	636/696
Eukaryota	Metazoa	Canis lupus familiaris	XP_852411.1	1 E-22	636/696
Eukaryota	Metazoa	Taeniopygia guttata	XP_002199530.1	2 E-22	636/696
Eukaryota	Metazoa	Salmo salar	NP_001133480.1	4 E-22	641/696
Eukaryota	Metazoa	Homo sapiens	BAB47463.1	9 E-22	632/696
Eukaryota	Metazoa	Homo sapiens	CAI40562.1	9 E-22	630/696
Eukaryota	Metazoa	Homo sapiens	NP_573403.1	1 E-21	632/696
Eukaryota	Metazoa	Monodelphis domestica	XP_001366582.1	1 E-21	632/696
Eukaryota	Metazoa	Equus caballus	XP_001503481.1	1 E-21	632/696
Eukaryota	Metazoa	Danio rerio	AAI25968.1	1 E-21	611/696
Eukaryota	Metazoa	Macaca mulatta	XP_001103324.1	2 E-21	632/696
Eukaryota	Metazoa	Pan troglodytes	XP_001162179.1	2 E-21	632/696
Eukaryota	Metazoa	Pongo abelii	NP_001128780.1	2 E-21	632/696
Eukaryota	Metazoa	Pan troglodytes	XP_001162135.1	3 E-21	631/696
Eukaryota	Metazoa	Pan troglodytes	XP_001162365.1	3 E-21	640/696
Eukaryota	Metazoa	Rattus norvegicus	EDL96460.1	4 E-21	632/696
Eukaryota	Metazoa	Macaca mulatta	XP_001103257.1	4 E-21	631/696
Eukaryota	Metazoa	Mus musculus	NP_525026.2	6 E-21	632/696
Eukaryota	Metazoa	Mus musculus	AAL14465.1	6 E-21	630/696
Eukaryota	Metazoa	Mus musculus	BAC34424.1	8 E-21	632/696
Eukaryota	Metazoa	Bos taurus	NP_001076860.1	9 E-21	632/696
Eukaryota	Metazoa	Monodelphis domestica	XP_001366531.1	1 E-20	644/696
Eukaryota	Metazoa	Canis lupus familiaris	XP_853040.1	1 E-20	644/696
Eukaryota	Metazoa	Canis lupus familiaris	XP_866792.1	2 E-20	640/696
Eukaryota	Metazoa	Homo sapiens	BAG61327.1	2 E-20	644/696
Eukaryota	Metazoa	Macaca mulatta	XP_001103175.1	3 E-20	642/696
Eukaryota	Metazoa	Canis lupus familiaris	XP_866723.1	5 E-20	628/696
Eukaryota	Metazoa	Mus musculus	NP_997589.1	9 E-20	644/696
Eukaryota	Metazoa	Rattus norvegicus	NP_001128427.1	1 E-19	644/696
Eukaryota	Fungi	Cryptococcus neoformans var. neoformans	XP_571309.1	1 E-19	703/696
Eukaryota	Metazoa	Homo sapiens	EAW75744.1	2 E-19	611/696
Eukaryota	Metazoa	Ixodes scapularis	XP_002404223.1	5 E-19	609/696
Eukaryota	Metazoa	Taeniopygia guttata	XP_002195992.1	5 E-19	598/696
Eukaryota	Metazoa	Drosophila melanogaster	AAM51013.1	7 E-17	580/696

Eukaryota	Metazoa	Drosophila melanogaster	NP_609548.1	1 E-16	580/696
Eukaryota	Metazoa	Drosophila pseudoobscura pseudoobscura	XP_001356404.1	2 E-16	609/696
Eukaryota	Metazoa	Drosophila yakuba	XP_002088446.1	5 E-16	580/696
Eukaryota	Metazoa	Drosophila sechellia	XP_002042025.1	9 E-16	580/696
Eukaryota	Metazoa	Drosophila willistoni	XP_002069229.1	9 E-16	626/696
Eukaryota	Metazoa	Drosophila simulans	XP_002079193.1	1 E-15	580/696
Eukaryota	Metazoa	Drosophila erecta	XP_001969764.1	1 E-15	606/696

AFUA_8G02820

Eukaryota	Fungi	Aspergillus fumigatus Af293	XP_746925.1	0.0	722/722
Eukaryota	Fungi	Neosartorya fischeri NRRL 181	XP_001261927.1	0.0	718/722
Eukaryota	Fungi	Aspergillus clavatus NRRL 1	XP_001274997.1	0.0	718/722
Eukaryota	Fungi	Aspergillus flavus NRRL3357	XP_002384975.1	1 E-165	669/722
Eukaryota	Fungi	Aspergillus oryzae RIB40	XP_001827724.1	1 E-164	669/722
Eukaryota	Fungi	Penicillium chrysogenum Wisconsin 54-1255	XP_002560534.1	1 E-158	718/722
Eukaryota	Fungi	Aspergillus niger CBS 513.88	XP_001390569.1	1 E-150	705/722
Eukaryota	Fungi	Aspergillus terreus NIH2624	XP_001218744.1	1 E-148	628/722
Eukaryota	Fungi	Ajellomyces capsulatus NAm1	XP_001544838.1	1 E-134	661/722
Eukaryota	Fungi	Aspergillus nidulans FGSC A4	XP_682145.1	1 E-132	675/722
Eukaryota	Fungi	Aspergillus nidulans FGSC A4	CBF84756.1	1 E-132	675/722
Eukaryota	Fungi	Talaromyces stipitatus ATCC 10500	XP_002483186.1	1 E-131	660/722
Eukaryota	Fungi	Paracoccidioides brasiliensis Pb01;	EEH39297.1	1 E-128	666/722
Eukaryota	Fungi	Coccidioides posadasii C735 delta	EER28689.1	1 E-127	662/722
Eukaryota	Fungi	Penicillium marneffei ATCC 18224	XP_002150895.1	1 E-126	667/722
Eukaryota	Fungi	Ajellomyces capsulatus G186AR	EEH08426.1	1 E-125	756/722
Eukaryota	Fungi	Ajellomyces capsulatus H143	EER42398.1	1 E-125	756/722
Eukaryota	Fungi	Paracoccidioides brasiliensis Pb03;	EEH21584.1	1 E-124	650/722
Eukaryota	Fungi	Paracoccidioides brasiliensis Pb18;	EEH43729.1	1 E-124	650/722
Eukaryota	Fungi	Microsporum canis CBS 113480	EEQ34708.1	1 E-122	646/722
Eukaryota	Fungi	Coccidioides immitis RS;	XP_001248524.1	1 E-121	647/722
Eukaryota	Fungi	Uncinocarpus reesii 1704	XP_002541926.1	1 E-120	692/722
Eukaryota	Fungi	Nectria haematococca mpVI 77-13-4	EEU47822.1	1 E-119	665/722
Eukaryota	Fungi	Ajellomyces dermatitidis SLH14081	XP_002628890.1	1 E-118	643/722
Eukaryota	Fungi	Ajellomyces dermatitidis ER-3	EEQ85276.1	1 E-117	643/722
Eukaryota	Fungi	Gibberella zeae PH-1	XP_381146.1	1 E-115	667/722
Eukaryota	Fungi	Neurospora crassa OR74A	XP_964321.1	1 E-111	627/722
Eukaryota	Fungi	Phaeosphaeria nodorum SN15	XP_001794718.1	1 E-110	582/722
Eukaryota	Fungi	Pyrenophora tritici-repentis Pt-1C-BFP	XP_001938194.1	1 E-109	687/722
Eukaryota	Fungi	Podospora anserina DSM 980	XP_001907322.1	1 E-109	706/722
Eukaryota	Fungi	Sclerotinia sclerotiorum 1980 UF-70	XP_001597566.1	1 E-109	705/722
Eukaryota	Fungi	Magnaporthe grisea 70-15	XP_001413753.1	1 E-101	684/722
Eukaryota	Fungi	Chaetomium globosum CBS 148.51	XP_001222033.1	1 E-101	682/722

AFUA_8G02850

Eukaryota	Fungi	Aspergillus fumigatus Af293	XP_746922.1	0.0	780/780
Eukaryota	Fungi	Neosartorya fischeri NRRL 181	XP_001261929.1	0.0	781/780
Eukaryota	Fungi	Aspergillus clavatus NRRL 1	XP_001274995.1	0.0	789/780
Eukaryota	Fungi	Aspergillus niger CBS 513.88	XP_001390572.1	0.0	780/780
Eukaryota	Fungi	Aspergillus oryzae RIB40	XP_001827727.1	0.0	788/780
Eukaryota	Fungi	Aspergillus terreus NIH2624	XP_001218749.1	0.0	791/780
Eukaryota	Fungi	Penicillium marneffei ATCC 18224	XP_002150891.1	0.0	775/780
Eukaryota	Fungi	Talaromyces stipitatus ATCC 10500	XP_002483189.1	0.0	781/780
Eukaryota	Fungi	Coccidioides immitis RS;	XP_001248520.1	0.0	806/780
Eukaryota	Fungi	Coccidioides posadasii C735 delta	EER28685.1	0.0	806/780
Eukaryota	Fungi	Emericella nidulans	ABO33305.1	0.0	774/780
Eukaryota	Fungi	Paracoccidioides brasiliensis Pb18;	EEH43722.1	0.0	809/780

Eukaryota	Fungi	Paracoccidioides brasiliensis Pb03;	EEH21591.1	0.0	815/780
Eukaryota	Fungi	Uncinocarpus reesii 1704	XP_002541930.1	0.0	808/780
Eukaryota	Fungi	Ajellomyces dermatitidis SLH14081	XP_002628895.1	0.0	817/780
Eukaryota	Fungi	Ajellomyces capsulatus G186AR	EEH08421.1	0.0	825/780
Eukaryota	Fungi	Ajellomyces capsulatus H143	EER42392.1	0.0	825/780
Eukaryota	Fungi	Paracoccidioides brasiliensis Pb01;	EEH39302.1	0.0	788/780
Eukaryota	Fungi	Microsporum canis CBS 113480	EEQ34751.1	0.0	792/780
Eukaryota	Fungi	Phaeosphaeria nodorum SN15	XP_001794527.1	0.0	819/780
Eukaryota	Fungi	Pyrenophora tritici-repentis Pt-1C-BFP	XP_001938411.1	1 E-177	810/780
Eukaryota	Fungi	Gibberella zeae PH-1	XP_381492.1	1 E-175	830/780
Eukaryota	Fungi	Nectria haematococca mpVI 77-13-4	EEU47664.1	1 E-174	819/780
Eukaryota	Fungi	Sclerotinia sclerotiorum 1980 UF-70	XP_001591018.1	1 E-174	822/780
Eukaryota	Fungi	Neurospora crassa OR74A	XP_957869.1	1 E-173	803/780
Eukaryota	Fungi	Chaetomium globosum CBS 148.51	XP_001219959.1	1 E-172	799/780
Eukaryota	Fungi	Magnaporthe grisea 70-15	XP_369843.1	1 E-169	799/780
Eukaryota	Fungi	Aspergillus nidulans FGSC A4	XP_682142.1	1 E-165	728/780
Eukaryota	Fungi	Podospora anserina DSM 980	XP_001908944.1	1 E-163	789/780
Eukaryota	Fungi	Ajellomyces capsulatus NAm1	XP_001544833.1	1 E-162	732/780

AFUA_8G02860

Eukaryota	Fungi	Aspergillus fumigatus Af293	XP_746921.1	0.0	950/950
Eukaryota	Fungi	Aspergillus clavatus NRRL 1	XP_001274993.1	0.0	821/950
Eukaryota	Fungi	Aspergillus niger CBS 513.88	XP_001390573.1	0.0	797/950
Eukaryota	Fungi	Aspergillus oryzae RIB40	XP_001827728.1	0.0	819/950
Eukaryota	Fungi	Aspergillus terreus NIH2624	XP_001218750.1	0.0	809/950
Eukaryota	Fungi	Penicillium chrysogenum Wisconsin 54-1255	XP_002560538.1	0.0	809/950
Eukaryota	Fungi	Coccidioides immitis RS;	XP_001248517.1	0.0	830/950
Eukaryota	Fungi	Coccidioides posadasii C735 delta	EER28683.1	0.0	830/950
Eukaryota	Fungi	Ajellomyces dermatitidis ER-3	EEQ85269.1	0.0	861/950
Eukaryota	Fungi	Ajellomyces dermatitidis SLH14081	XP_002628898.1	0.0	861/950
Eukaryota	Fungi	Uncinocarpus reesii 1704	XP_002541932.1	0.0	904/950
Eukaryota	Fungi	Penicillium marneffei ATCC 18224	XP_002150888.1	0.0	883/950
Eukaryota	Fungi	Microsporum canis CBS 113480	EEQ34927.1	0.0	807/950
Eukaryota	Fungi	Ajellomyces capsulatus G186AR	EEH08419.1	0.0	838/950
Eukaryota	Fungi	Ajellomyces capsulatus NAm1	XP_001544831.1	0.0	838/950
Eukaryota	Fungi	Talaromyces stipitatus ATCC 10500	XP_002483192.1	0.0	860/950
Eukaryota	Fungi	Ajellomyces capsulatus H143	EER42390.1	0.0	838/950
Eukaryota	Fungi	Pyrenophora tritici-repentis Pt-1C-BFP	XP_001938448.1	0.0	840/950
Eukaryota	Fungi	Botryotinia fuckeliana B05.10	XP_001547438.1	0.0	886/950
Eukaryota	Fungi	Gibberella zeae PH-1	XP_381547.1	0.0	825/950
Eukaryota	Fungi	Chaetomium globosum CBS 148.51	XP_001222008.1	0.0	816/950
Eukaryota	Fungi	Magnaporthe grisea 70-15	XP_360256.1	0.0	797/950
Eukaryota	Fungi	Verticillium albo-atrum VaMs.102	EEY18987.1	0.0	809/950
Eukaryota	Fungi	Neurospora crassa OR74A	XP_965366.2	0.0	802/950
Eukaryota	Fungi	Podospora anserina DSM 980	XP_001907339.1	0.0	824/950
Eukaryota	Fungi	Paracoccidioides brasiliensis Pb01;	EEH39306.1	0.0	774/950
Eukaryota	Fungi	Paracoccidioides brasiliensis Pb03;	EEH21593.1	0.0	784/950
Eukaryota	Fungi	Paracoccidioides brasiliensis Pb18;	EEH43720.1	0.0	784/950
Eukaryota	Fungi	Phaeosphaeria nodorum SN15	XP_001806646.1	0.0	782/950

AFUA_8G02870

Eukaryota	Fungi	Aspergillus fumigatus Af293	XP_746920.1	0.0	637/637
Eukaryota	Fungi	Neosartorya fischeri NRRL 181	XP_001261931.1	0.0	636/637
Eukaryota	Fungi	Aspergillus terreus NIH2624	XP_001218751.1	4 E-94	512/637
Eukaryota	Fungi	Talaromyces stipitatus ATCC 10500	XP_002483193.1	5 E-93	652/637
Eukaryota	Fungi	Aspergillus nidulans FGSC A4	CBF84767.1	1 E-88	605/637

Eukaryota	Fungi	Penicillium marneffei ATCC 18224	XP_002150887.1	4 E-85	535/637
Eukaryota	Fungi	Aspergillus nidulans FGSC A4	XP_682140.1	1 E-84	554/637
Eukaryota	Fungi	Ajellomyces capsulatus NAm1	XP_001544830.1	1 E-75	536/637
Eukaryota	Fungi	Ajellomyces capsulatus G186AR	EEH08417.1	3 E-71	620/637
Eukaryota	Fungi	Microsporum canis CBS 113480	EEQ34928.1	5 E-70	536/637
Eukaryota	Fungi	Uncinocarpus reesii 1704	XP_002541933.1	9 E-69	565/637
Eukaryota	Fungi	Paracoccidioides brasiliensis Pb03;	EEH21594.1	2 E-67	631/637
Eukaryota	Fungi	Ajellomyces dermatitidis SLH14081	XP_002628899.1	1 E-65	541/637
Eukaryota	Fungi	Ajellomyces dermatitidis ER-3	EEQ85268.1	3 E-65	541/637
Eukaryota	Fungi	Coccidioides immitis RS;	XP_001248516.1	7 E-64	521/637
Eukaryota	Fungi	Paracoccidioides brasiliensis Pb01;	EEH39308.1	4 E-63	537/637
Eukaryota	Fungi	Coccidioides posadasii C735 delta	EER28682.1	5 E-63	521/637
Eukaryota	Fungi	Phaeosphaeria nodorum SN15	XP_001794956.1	4 E-48	601/637
Eukaryota	Fungi	Pyrenophora tritici-repentis Pt-1C-BFP	XP_001937957.1	4 E-42	544/637
Eukaryota	Fungi	Sclerotinia sclerotiorum 1980 UF-70	XP_001593042.1	3 E-35	542/637

AFUA_8G06470

Eukaryota	Fungi	Aspergillus fumigatus Af293	XP_747448.1	0.0	847/847
Eukaryota	Fungi	Neosartorya fischeri NRRL 181	XP_001262173.1	0.0	847/847
Eukaryota	Fungi	Aspergillus oryzae RIB40	XP_001820722.1	0.0	843/847
Eukaryota	Fungi	Aspergillus flavus NRRL3357	XP_002376448.1	0.0	843/847
Eukaryota	Fungi	Aspergillus terreus NIH2624	XP_001216124.1	0.0	834/847
Eukaryota	Fungi	Pyrenophora tritici-repentis Pt-1C-BFP	XP_001936243.1	0.0	843/847
Eukaryota	Fungi	Aspergillus niger CBS 513.88	XP_001396797.1	0.0	852/847
Eukaryota	Fungi	Phaeosphaeria nodorum SN15	XP_001800564.1	0.0	846/847
Eukaryota	Fungi	Penicillium chrysogenum Wisconsin 54-1255	XP_002558863.1	0.0	827/847
Eukaryota	Fungi	Aspergillus nidulans FGSC A4	XP_681923.1	0.0	820/847
Bacteria	Actinobacteria	Arthrobacter aurescens TC1	YP_946292.1	0.0	830/847
Eukaryota	Fungi	Nectria haematococca mpVI 77-13-4	EEU36643.1	0.0	817/847
Bacteria	Actinobacteria	Arthrobacter chlorophenolicus A6	YP_002489508.1	0.0	830/847
Bacteria	Actinobacteria	Arthrobacter sp. FB24	YP_833193.1	0.0	835/847
Eukaryota	Fungi	Gibberella zeae PH-1	XP_383590.1	0.0	820/847
Bacteria	Actinobacteria	Arthrobacter globiformis	ABB73054.1	0.0	835/847
Bacteria	Actinobacteria	Arthrobacter chlorophenolicus A6	YP_002489514.1	0.0	835/847
Bacteria	Actinobacteria	Saccharomonospora viridis DSM 43017	YP_003133734.1	0.0	813/847
Bacteria	Actinobacteria	Saccharopolyspora erythraea NRRL 2338	YP_001106690.1	0.0	816/847
Bacteria	Actinobacteria	Geodermatophilus obscurus DSM 43160	ZP_03887969.1	0.0	815/847
Bacteria	Actinobacteria	Rhodococcus opacus B4	YP_002778684.1	0.0	813/847
Bacteria	Actinobacteria	Rhodococcus jostii RHA1	YP_701784.1	0.0	812/847
Bacteria	Actinobacteria	Streptomyces sp. AA4	ZP_05480014.1	0.0	797/847
Bacteria	Actinobacteria	Brevibacterium linens BL2	ZP_05912755.1	0.0	842/847
Bacteria	Actinobacteria	Streptomyces hygroscopicus ATCC 53653	ZP_05511904.1	0.0	808/847
Bacteria	Actinobacteria	Streptomyces viridochromogenes DSM 40736	ZP_05535972.1	0.0	809/847
Bacteria	Actinobacteria	Streptomyces scabiei 87.22	CBG67887.1	0.0	807/847
Bacteria	Actinobacteria	Streptomyces avermitilis MA-4680	NP_828127.1	0.0	800/847
Bacteria	Actinobacteria	Streptomyces hygroscopicus ATCC 53653	ZP_05512943.1	0.0	801/847
Bacteria	Actinobacteria	Saccharopolyspora erythraea NRRL 2338	YP_001105352.1	0.0	804/847
Bacteria	Actinobacteria	Mycobacterium sp. MCS	YP_639623.1	0.0	803/847
Bacteria	Actinobacteria	Mycobacterium sp. JLS	YP_001070771.1	0.0	803/847
Bacteria	Actinobacteria	Mycobacterium marinum M	YP_001852682.1	0.0	806/847
Bacteria	Actinobacteria	Mycobacterium gilvum PYR-GCK	YP_001134345.1	0.0	810/847
Bacteria	Actinobacteria	Streptosporangium roseum DSM 43021	ZP_04477888.1	0.0	791/847
Bacteria	Actinobacteria	Rubrobacter xylanophilus DSM 9941	YP_643542.1	0.0	812/847
Bacteria	Actinobacteria	Streptomyces clavuligerus ATCC 27064	ZP_05005957.1	0.0	803/847
Bacteria	Actinobacteria	Saccharopolyspora erythraea NRRL 2338	YP_001107727.1	0.0	809/847
Archaea	Euryarchaeota	Haloarcula marismortui ATCC 43049	YP_134764.1	1 E-145	842/847
Bacteria	Actinobacteria	Streptomyces sp. AA4	ZP_05482949.1	1 E-143	741/847

Archaea	Euryarchaeota	Haloquadratum walsbyi DSM 16790	YP_657525.1	1 E-143	864/847
Archaea	Euryarchaeota	Haloarcula marismortui ATCC 43049	YP_134767.1	1 E-143	831/847
Archaea	Euryarchaeota	Haloquadratum walsbyi DSM 16790	YP_657526.1	1 E-139	836/847
Bacteria	Actinobacteria	Stackebrandtia nassauensis DSM 44728	ZP_04483830.1	1 E-128	827/847
Bacteria	Actinobacteria	Brevibacterium linens BL2	ZP_05913931.1	1 E-124	815/847
Bacteria	Actinobacteria	Brachybacterium faecium DSM 4810	YP_003155370.1	1 E-124	815/847
Bacteria	Actinobacteria	Streptosporangium roseum DSM 43021	ZP_04476092.1	1 E-119	802/847
Bacteria	Proteobacteria	Rhizobium leguminosarum bv. trifolii	YP_002283509.1	1 E-111	798/847
Bacteria	Proteobacteria	Rhizobium leguminosarum bv. viciae	YP_765701.1	1 E-110	799/847
Bacteria	Proteobacteria	Rhizobium etli CIAT 652	YP_001976267.1	1 E-109	799/847
Bacteria	Proteobacteria	Rhizobium leguminosarum bv. trifolii	YP_002978129.1	1 E-109	799/847
Bacteria	Actinobacteria	Nocardioides sp. JS614	YP_921855.1	1 E-109	787/847
Bacteria	Proteobacteria	Agrobacterium radiobacter K84	YP_002545403.1	1 E-107	798/847
Bacteria	Proteobacteria	Mesorhizobium loti MAFF303099	NP_106044.1	1 E-107	780/847
Bacteria	Proteobacteria	Rhizobium etli CFN 42	YP_467641.1	1 E-107	798/847
Bacteria	Proteobacteria	Roseovarius sp. 217	ZP_01035130.1	1 E-107	788/847
Bacteria	Proteobacteria	alpha proteobacterium BAL199	ZP_02188210.1	1 E-107	780/847
Bacteria	Proteobacteria	Ruegeria sp. R11	ZP_05089725.1	1 E-107	793/847
Bacteria	Proteobacteria	alpha proteobacterium BAL199	ZP_02190914.1	1 E-106	796/847
Bacteria	Proteobacteria	Methylobacterium sp. 4-46	YP_001770673.1	1 E-106	791/847
Bacteria	Proteobacteria	Roseobacter sp. SK209-2-6	ZP_01756347.1	1 E-106	786/847
Bacteria	Proteobacteria	Roseovarius sp. TM1035	ZP_01881791.1	1 E-106	791/847
Bacteria	Proteobacteria	Roseobacter sp. SK209-2-6	ZP_01757008.1	1 E-105	793/847
Bacteria	Proteobacteria	Mesorhizobium opportunistum WSM2075	ZP_05812738.1	1 E-105	780/847
Bacteria	Proteobacteria	Rhodobacterales bacterium HTCC2255	ZP_01447227.1	1 E-105	791/847
Bacteria	Proteobacteria	Roseovarius sp. HTCC2601	ZP_01446140.1	1 E-104	799/847
Bacteria	Proteobacteria	Rhodobacteraceae bacterium KLH11	ZP_05122593.1	1 E-104	793/847
Bacteria	Proteobacteria	Rhodobacterales bacterium Y4I	ZP_05078631.1	1 E-104	793/847
Bacteria	Proteobacteria	Phaeobacter gallaeciensis 2.10	ZP_02151486.1	1 E-104	793/847
Bacteria	Proteobacteria	Phaeobacter gallaeciensis BS107	ZP_02145036.1	1 E-103	786/847
Bacteria	Proteobacteria	Phaeobacter gallaeciensis 2.10	ZP_02148200.1	1 E-103	786/847
Bacteria	Proteobacteria	Roseovarius nubinhibens ISM	ZP_00961321.1	1 E-103	786/847
Bacteria	Proteobacteria	Mesorhizobium opportunistum WSM2075	ZP_05807877.1	1 E-103	797/847
Bacteria	Proteobacteria	Silicibacter lacuscaerulensis ITI-1157	ZP_05786320.1	1 E-103	794/847
Bacteria	Proteobacteria	Roseovarius nubinhibens ISM	ZP_00959991.1	1 E-102	789/847
Bacteria	Proteobacteria	Citreicella sp. SE45	ZP_05780319.1	1 E-102	794/847
Bacteria	Proteobacteria	Rhizobium sp. NGR234	YP_002823856.1	1 E-102	799/847
Bacteria	Proteobacteria	marine gamma proteobacterium HTCC2080	ZP_01626142.1	1 E-102	790/847
Bacteria	Proteobacteria	Pseudovibrio sp. JE062	ZP_05082613.1	1 E-102	797/847
Bacteria	Proteobacteria	Rhodobacterales bacterium HTCC2654	ZP_01013622.1	1 E-102	799/847
Bacteria	Proteobacteria	Ruegeria pomeroyi DSS-3	YP_168592.1	1 E-102	793/847
Bacteria	Proteobacteria	Roseobacter sp. AzwK-3b	ZP_01904012.1	1 E-102	803/847
Bacteria	Proteobacteria	uncultured marine proteobacterium	AAL76414.1	1 E-101	790/847
Bacteria	Actinobacteria	Mycobacterium vanbaalenii PYR-1	YP_953110.1	1 E-101	804/847
Bacteria	Candidatus	Candidatus Pelagibacter ubique HTCC1062	YP_266631.1	1 E-101	788/847
Bacteria	Proteobacteria	Roseobacter denitrificans OCh 114	YP_680443.1	1 E-101	791/847
Bacteria	Proteobacteria	Roseobacter sp. MED193	ZP_01055530.1	1 E-101	793/847
Bacteria	Proteobacteria	Phaeobacter gallaeciensis BS107	ZP_02147237.1	1 E-101	793/847
Bacteria	Proteobacteria	Roseobacter sp. CCS2	ZP_01749585.1	1 E-101	797/847
Bacteria	Proteobacteria	Ruegeria pomeroyi DSS-3	YP_167653.1	1 E-101	744/847
Bacteria	Proteobacteria	Mesorhizobium loti MAFF303099	NP_102909.1	1 E-101	797/847
Bacteria	Proteobacteria	marine gamma proteobacterium HTCC2148	ZP_05093783.1	1 E-100	846/847
Bacteria	Proteobacteria	Cupriavidus taiwanensis	YP_002008305.1	1 E-100	801/847
Bacteria	Proteobacteria	Mesorhizobium opportunistum WSM2075	ZP_05808279.1	1 E-100	798/847
Bacteria	Proteobacteria	Burkholderia xenovorans LB400	YP_553650.1	1 E-100	803/847
Bacteria	Candidatus	Candidatus Pelagibacter ubique HTCC1002	ZP_01264985.1	1 E-100	788/847
Bacteria	Proteobacteria	Burkholderia phytofirmans PsJN	YP_001888719.1	1 E-99	804/847
Bacteria	Proteobacteria	Loktanella vestfoldensis SKA53	ZP_01001940.1	1 E-99	787/847

Bacteria	Proteobacteria	Thalassiobium sp. R2A62	ZP_05340764.1	1 E-99	791/847
Eukaryota	Metazoa	Nematostella vectensis	XP_001632395.1	2 E-99	799/847
Bacteria	Proteobacteria	Oceanibulbus indolifex HEL-45	ZP_02153682.1	2 E-99	798/847
Bacteria	Proteobacteria	Rhizobium sp. NGR234	YP_002826695.1	4 E-99	797/847
Bacteria	Proteobacteria	Rhodobacterales bacterium HTCC2083	ZP_05073481.1	4 E-99	791/847
Bacteria	Proteobacteria	Sinorhizobium meliloti 1021	NP_437514.1	6 E-99	791/847
Bacteria	Proteobacteria	Rhodobacterales bacterium HTCC2150	ZP_01741911.1	6 E-99	791/847
Bacteria	Proteobacteria	Roseobacter sp. SK209-2-6	ZP_01753669.1	1 E-98	813/847
Bacteria	Proteobacteria	Thalassiobium sp. R2A62	ZP_05344176.1	2 E-98	805/847
Bacteria	marine	marine bacterium 01-004080	ACA21521.1	2 E-98	802/847
Bacteria	Proteobacteria	Phaeobacter gallaeciensis 2.10	ZP_02148190.1	4 E-98	787/847
Bacteria	Proteobacteria	Sagittula stellata E-37	ZP_01746222.1	5 E-98	788/847
Bacteria	Proteobacteria	Sinorhizobium meliloti 1021	NP_386380.1	8 E-98	798/847
Bacteria	Proteobacteria	Paracoccus denitrificans PD1222	YP_918672.1	1 E-97	806/847
Bacteria	Proteobacteria	Jannaschia sp. CCS1	YP_510791.1	1 E-97	792/847
Bacteria	Proteobacteria	Phaeobacter gallaeciensis BS107	ZP_02145045.1	1 E-97	787/847
Bacteria	Proteobacteria	Pseudovibrio sp. JE062	ZP_05084305.1	2 E-97	807/847
Bacteria	Proteobacteria	Ruegeria sp. TM1040	YP_613249.1	3 E-97	781/847
Bacteria	Proteobacteria	Octadecabacter antarcticus 307	ZP_05051000.1	3 E-97	789/847
Bacteria	Proteobacteria	Hoeflea phototrophica DFL-43	ZP_02165726.1	4 E-97	797/847
Bacteria	Proteobacteria	Marinobacter algicola DG893	ZP_01892358.1	7 E-97	843/847
Bacteria	Proteobacteria	Octadecabacter antarcticus 238	ZP_05063036.1	9 E-97	789/847
Bacteria	Proteobacteria	Sinorhizobium medicae WSM419	YP_001313588.1	9 E-97	763/847
Eukaryota	Metazoa	Branchiostoma floridae	XP_002609761.1	1 E-96	787/847
Bacteria	Candidatus	Candidatus Pelagibacter ubique HTCC1062	YP_266661.1	2 E-96	786/847
Bacteria	Proteobacteria	Rhodobacter sp. SW2	ZP_05843686.1	2 E-96	748/847
Bacteria	Candidatus	Candidatus Pelagibacter ubique HTCC1002	ZP_01264956.1	3 E-96	786/847
Eukaryota	Metazoa	Ixodes scapularis	XP_002403006.1	4 E-96	741/847
Bacteria	Proteobacteria	Sinorhizobium medicae WSM419	YP_001327865.1	8 E-96	798/847
Bacteria	environmental	uncultured marine bacterium 578	AAR38102.1	8 E-96	817/847
Eukaryota	Metazoa	Branchiostoma floridae	XP_002609759.1	1 E-95	725/847
Bacteria	Proteobacteria	Ralstonia eutropha H16	YP_841467.1	2 E-95	765/847
Bacteria	Proteobacteria	Labrenzia alexandrii DFL-11	ZP_05115904.1	2 E-95	788/847
Eukaryota	Metazoa	Branchiostoma floridae	XP_002609760.1	8 E-95	730/847
Eukaryota	Metazoa	Hydra magnipapillata	XP_002156170.1	8 E-95	776/847
Eukaryota	Metazoa	Homo sapiens	BAG37277.1	1 E-94	795/847
Eukaryota	Metazoa	Homo sapiens	EAW95824.1	1 E-94	795/847
Bacteria	Candidatus	Candidatus Pelagibacter sp. HTCC7211	ZP_05069615.1	1 E-94	786/847
Eukaryota	Metazoa	Homo sapiens	BAG51946.1	1 E-94	795/847
Eukaryota	Metazoa	Mus musculus	NP_083048.1	2 E-94	795/847
Bacteria	Proteobacteria	marine gamma proteobacterium HTCC2080	ZP_01626815.1	2 E-94	842/847
Eukaryota	Metazoa	Taeniopygia guttata	XP_002188408.1	2 E-94	795/847
Eukaryota	Metazoa	Mus musculus	AAH89599.1	5 E-94	795/847
Eukaryota	Metazoa	Equus caballus	XP_001503961.1	5 E-94	795/847
Eukaryota	Metazoa	Homo sapiens	NP_037523.2	5 E-94	795/847
Eukaryota	Metazoa	Rattus norvegicus	Q63342.1	7 E-94	795/847
Bacteria	Proteobacteria	Roseobacter sp. GAI101	ZP_05098724.1	7 E-94	788/847
Eukaryota	Metazoa	Pan troglodytes	XP_526883.2	1 E-93	795/847
Bacteria	Proteobacteria	Silicibacter sp. TrichCH4B	ZP_05739704.1	1 E-93	780/847
Bacteria	Proteobacteria	Ochrobactrum intermedium LMG 3301	ZP_04683071.1	2 E-93	807/847
Bacteria	Proteobacteria	Citreicella sp. SE45	ZP_05784103.1	2 E-93	800/847
Bacteria	Proteobacteria	Burkholderia graminis C4D1M	ZP_02884938.1	3 E-93	799/847
Bacteria	Proteobacteria	Mesorhizobium opportunistum WSM2075	ZP_05807839.1	4 E-93	784/847
Bacteria	Proteobacteria	Jannaschia sp. CCS1	YP_510430.1	5 E-93	806/847
Eukaryota	Metazoa	Homo sapiens	Q9UI17.1	5 E-93	795/847
Eukaryota	Metazoa	Xenopus laevis	NP_001087085.1	6 E-93	795/847
Bacteria	Proteobacteria	Mesorhizobium loti MAFF303099	NP_102887.1	6 E-93	839/847
Eukaryota	Metazoa	Rattus norvegicus	NP_620802.2	1 E-92	795/847

Bacteria	Proteobacteria	Burkholderia phymatum STM815	YP_001859375.1	1 E-92	800/847
Bacteria	Proteobacteria	marine gamma proteobacterium HTCC2148	ZP_05093713.1	2 E-92	784/847
Bacteria	Candidatus	Candidatus Pelagibacter sp. HTCC7211	ZP_05069445.1	3 E-92	786/847
Bacteria	Actinobacteria	Nocardioides sp. JS614	YP_923782.1	4 E-92	748/847
Bacteria	Proteobacteria	marine gamma proteobacterium HTCC2080	ZP_01625483.1	6 E-92	793/847
Bacteria	Proteobacteria	Rhizobium sp. NGR234	YP_002827076.1	7 E-92	839/847
Bacteria	Proteobacteria	Agrobacterium radiobacter K84	YP_002540765.1	7 E-92	848/847
Bacteria	Proteobacteria	Sinorhizobium meliloti 1021	NP_386627.1	1 E-91	839/847
Bacteria	Proteobacteria	Roseobacter litoralis Och 149	ZP_02141722.1	1 E-91	695/847
Bacteria	Proteobacteria	Agrobacterium radiobacter K84	YP_002540243.1	1 E-91	807/847
Eukaryota	Metazoa	Monodelphis domestica	XP_001381588.1	2 E-91	795/847
Bacteria	Proteobacteria	Sinorhizobium medicae WSM419	YP_001328097.1	2 E-91	839/847
Bacteria	Proteobacteria	Brucella neotomae 5K33	ZP_05451536.1	3 E-91	839/847
Eukaryota	Metazoa	Bos taurus	XP_580581.3	3 E-91	795/847
Bacteria	Proteobacteria	Roseovarius sp. HTCC2601	ZP_01445119.1	4 E-91	840/847
Bacteria	Proteobacteria	Brucella sp. 83/13	ZP_05181175.1	4 E-91	839/847
Bacteria	Proteobacteria	Brucella abortus bv. 3	ZP_05155842.1	4 E-91	839/847
Bacteria	Proteobacteria	Brucella pinnipedialis B2/94	ZP_05172190.1	4 E-91	839/847
Bacteria	Proteobacteria	Brucella ceti str. Cudo	ZP_03785822.1	4 E-91	839/847
Bacteria	Proteobacteria	Mesorhizobium loti MAFF303099	NP_103793.1	5 E-91	783/847
Bacteria	Proteobacteria	Brucella suis ATCC 23445	YP_001627983.1	6 E-91	839/847
Bacteria	Proteobacteria	Brucella pinnipedialis M163/99/10	ZP_05169837.1	9 E-91	839/847
Bacteria	Proteobacteria	Citreicella sp. SE45	ZP_05783614.1	1 E-90	840/847
Bacteria	Proteobacteria	Labrenzia alexandrii DFL-11	ZP_05113775.1	1 E-90	840/847
Bacteria	Proteobacteria	Labrenzia aggregata IAM 12614	ZP_01550934.1	2 E-90	840/847
Bacteria	Proteobacteria	Ochrobactrum anthropi ATCC 49188	YP_001370409.1	2 E-90	839/847
Bacteria	Proteobacteria	Rhizobium leguminosarum bv. trifolii	YP_002282804.1	3 E-90	800/847
Bacteria	Proteobacteria	Brucella suis bv. 5	ZP_05163880.1	3 E-90	839/847
Bacteria	Proteobacteria	Octadecabacter antarcticus 307	ZP_05053643.1	4 E-90	801/847
Bacteria	Proteobacteria	marine gamma proteobacterium HTCC2143	ZP_01616406.1	5 E-90	813/847
Bacteria	Proteobacteria	Roseovarius nubinhibens ISM	ZP_00958893.1	7 E-90	780/847
Bacteria	Proteobacteria	Ochrobactrum intermedium LMG 3301	ZP_04680539.1	9 E-90	839/847
Bacteria	Proteobacteria	Mesorhizobium opportunistum WSM2075	ZP_05807897.1	1 E-89	839/847
unclassified	environmental	uncultured marine microorganism HF4000_141[illegible]	ABZ06757.1	1 E-89	786/847
Bacteria	Proteobacteria	Mesorhizobium loti MAFF303099	NP_102906.1	2 E-89	790/847
Bacteria	Proteobacteria	Roseobacter sp. SK209-2-6	ZP_01753315.1	2 E-89	780/847
Eukaryota	Metazoa	Canis lupus familiaris	XP_546052.2	3 E-89	785/847
Eukaryota	Metazoa	Monodelphis domestica	XP_001371908.1	4 E-89	840/847
Bacteria	Proteobacteria	Roseovarius nubinhibens ISM	ZP_00958745.1	7 E-89	779/847
Bacteria	Proteobacteria	marine gamma proteobacterium HTCC2148	ZP_05094735.1	9 E-89	790/847
Bacteria	Proteobacteria	Rhizobium leguminosarum bv. trifolii	YP_002278185.1	1 E-88	839/847
Bacteria	Proteobacteria	Rhizobium leguminosarum bv. trifolii	YP_002984880.1	1 E-88	839/847
Eukaryota	Metazoa	Strongylocentrotus purpuratus	XP_786380.2	1 E-88	821/847
Bacteria	Proteobacteria	Oceanicola batsensis HTCC2597	ZP_00998424.1	2 E-88	822/847
Bacteria	Proteobacteria	Ruegeria sp. TM1040	YP_612429.1	2 E-88	803/847
Bacteria	Proteobacteria	Rhizobium leguminosarum bv. viciae	YP_771270.1	2 E-88	839/847
Bacteria	Proteobacteria	Rhizobium etli CFN 42	YP_472250.1	2 E-88	839/847
Bacteria	Proteobacteria	marine gamma proteobacterium HTCC2148	ZP_05093353.1	4 E-88	791/847
Bacteria	Proteobacteria	Aurantimonas manganoxydans SI85-9A1	ZP_01226037.1	7 E-88	815/847
Bacteria	Proteobacteria	Ruegeria pomeroyi DSS-3	YP_165890.1	7 E-88	824/847
Bacteria	Proteobacteria	Pseudovibrio sp. JE062	ZP_05082586.1	1 E-87	839/847
Bacteria	Proteobacteria	Silicibacter lacuscaerulensis ITI-1157	ZP_05786562.1	1 E-87	824/847
Bacteria	Proteobacteria	Roseovarius nubinhibens ISM	ZP_00961260.1	2 E-87	787/847
Bacteria	Proteobacteria	Loktanella vestfoldensis SKA53	ZP_01003550.1	3 E-87	802/847
Bacteria	Proteobacteria	Ruegeria sp. TM1040	YP_613421.1	4 E-87	805/847
Bacteria	Proteobacteria	alpha proteobacterium BAL199	ZP_02189867.1	4 E-87	789/847
Bacteria	Proteobacteria	Rhizobium etli CIAT 652	YP_001985700.1	7 E-87	839/847
Eukaryota	Metazoa	Danio rerio	AAI55631.1	1 E-86	802/847

Bacteria	Proteobacteria	Roseovarius nubinhibens ISM	ZP_00960506.1	1 E-86	831/847
Eukaryota	Metazoa	Hydra magnipapillata	XP_002158472.1	1 E-86	848/847
Bacteria	Proteobacteria	Silicibacter sp. TrichCH4B	ZP_05739931.1	1 E-86	805/847
Bacteria	Proteobacteria	Phaeobacter gallaeciensis BS107	ZP_02143696.1	4 E-86	783/847
Bacteria	Proteobacteria	Rhizobium leguminosarum bv. trifolii	YP_002282803.1	5 E-86	805/847
Bacteria	Proteobacteria	Roseobacter sp. CCS2	ZP_01752205.1	6 E-86	802/847
Bacteria	Proteobacteria	Rhodobacterales bacterium HTCC2083	ZP_05076132.1	1 E-85	822/847
Bacteria	Proteobacteria	gamma proteobacterium NOR51-B	ZP_04958072.1	1 E-85	803/847
Bacteria	Proteobacteria	Rhodobacterales bacterium Y4I	ZP_05080682.1	2 E-85	775/847
Bacteria	Proteobacteria	Dinoroseobacter shibae DFL 12	YP_001532828.1	2 E-85	783/847
Bacteria	Proteobacteria	Mesorhizobium opportunistum WSM2075	ZP_05807880.1	2 E-85	790/847
Bacteria	Proteobacteria	Rhodobacterales bacterium HTCC2654	ZP_01014159.1	2 E-85	824/847
Bacteria	Proteobacteria	Labrenzia aggregata IAM 12614	ZP_01545674.1	3 E-85	782/847
Bacteria	Proteobacteria	Rhodobacterales bacterium HTCC2083	ZP_05074798.1	3 E-85	782/847
Bacteria	Proteobacteria	Roseovarius sp. 217	ZP_01034275.1	3 E-85	806/847
Bacteria	Proteobacteria	Roseobacter sp. MED193	ZP_01058535.1	3 E-85	783/847
Bacteria	Proteobacteria	Silicibacter lacuscaerulensis ITI-1157	ZP_05787949.1	4 E-85	783/847
Bacteria	Proteobacteria	Roseovarius sp. 217	ZP_01034276.1	4 E-85	780/847
Eukaryota	Metazoa	Aedes aegypti	XP_001660213.1	4 E-85	782/847
Eukaryota	Metazoa	Rattus norvegicus	EDL93439.1	5 E-85	851/847
Bacteria	Proteobacteria	Roseobacter sp. SK209-2-6	ZP_01753314.1	7 E-85	783/847
Eukaryota	Metazoa	Rattus norvegicus	NP_446116.1	7 E-85	851/847
Bacteria	Proteobacteria	Roseobacter sp. SK209-2-6	ZP_01755266.1	8 E-85	782/847
Bacteria	Proteobacteria	Roseobacter sp. MED193	ZP_01054849.1	8 E-85	824/847
Bacteria	Proteobacteria	Phaeobacter gallaeciensis 2.10	ZP_02148586.1	1 E-84	783/847
Bacteria	Proteobacteria	Jannaschia sp. CCS1	YP_508861.1	1 E-84	830/847
Eukaryota	Metazoa	Bos taurus	XP_871256.2	1 E-84	848/847
Bacteria	Proteobacteria	Sinorhizobium meliloti 1021	NP_386381.1	1 E-84	804/847
Bacteria	Proteobacteria	Roseobacter sp. GAI101	ZP_05099539.1	1 E-84	802/847

AFUA_8G06820

Eukaryota	Fungi	Aspergillus fumigatus Af293	XP_747481.1	1 E-110	194/194
Eukaryota	Fungi	Aspergillus terreus NIH2624	XP_001213087.1	6 E-58	193/194
Eukaryota	Fungi	Aspergillus nidulans FGSC A4	XP_660801.1	4 E-48	203/194
Bacteria	Actinobacteria	Kytococcus sedentarius DSM 20547	YP_003148363.1	3 E-30	178/194
Bacteria	Actinobacteria	Sanguibacter keddieii DSM 10542	ZP_05817864.1	2 E-27	177/194
Bacteria	Proteobacteria	Vibrio fischeri MJ11	YP_002156494.1	4 E-26	171/194
Bacteria	Proteobacteria	Vibrio fischeri ES114	YP_205058.1	2 E-25	171/194
Bacteria	Planctomycetes	Planctomyces limnophilus DSM 3776	ZP_04426453.1	3 E-25	174/194
Bacteria	Proteobacteria	Stenotrophomonas sp. SKA14	ZP_05136865.1	4 E-25	182/194
Bacteria	Proteobacteria	Pseudomonas entomophila L48	YP_610129.1	4 E-24	176/194
Bacteria	Proteobacteria	Klebsiella pneumoniae subsp. pneumoniae	YP_001336598.1	4 E-24	171/194
Bacteria	Actinobacteria	Kineococcus radiotolerans SRS30216	YP_001362421.1	4 E-24	168/194
Bacteria	Proteobacteria	Aliivibrio salmonicida LFI1238	YP_002262582.1	6 E-24	172/194
Bacteria	Proteobacteria	Vibrio vulnificus YJ016	NP_937586.1	8 E-24	172/194
Bacteria	Bacteroidetes	Algoriphagus sp. PR1	ZP_01721347.1	8 E-24	172/194
Bacteria	Proteobacteria	Escherichia coli UMN026	YP_002413662.1	2 E-23	171/194
Bacteria	Proteobacteria	Vibrio parahaemolyticus RIMD 2210633	NP_800810.1	2 E-23	169/194
Bacteria	Proteobacteria	Desulfotalea psychrophila LSv54	YP_064441.1	2 E-23	173/194
Bacteria	Proteobacteria	Vibrio vulnificus CMCP6	NP_762952.1	5 E-23	172/194
Bacteria	Proteobacteria	Shewanella loihica PV-4	YP_001092907.1	5 E-23	169/194
Bacteria	Cyanobacteria	Acaryochloris marina MBIC11017	YP_001519137.1	8 E-23	175/194
Bacteria	Proteobacteria	Photobacterium profundum SS9	YP_132121.1	1 E-22	172/194
Bacteria	Proteobacteria	Desulfotalea psychrophila LSv54	YP_066952.1	2 E-22	173/194
Bacteria	Proteobacteria	Azoarcus sp. BH72	YP_934490.1	2 E-22	171/194
Bacteria	Proteobacteria	Proteus penneri ATCC 35198	ZP_03805842.1	2 E-22	169/194
Bacteria	Proteobacteria	Photobacterium profundum 3TCK	ZP_01221409.1	2 E-22	172/194

Bacteria	Proteobacteria	Vibrio parahaemolyticus AQ4037	ZP_05908928.1	2 E-22	169/194
Bacteria	Proteobacteria	Vibrio harveyi HY01	ZP_01986316.1	3 E-22	169/194
Bacteria	Proteobacteria	Vibrio parahaemolyticus AQ3810	ZP_01991531.1	5 E-22	169/194
Bacteria	Proteobacteria	Vibrio sp. MED222	ZP_01063031.1	6 E-22	169/194
Bacteria	Proteobacteria	Polaromonas sp. JS666	YP_547149.1	6 E-22	174/194
Bacteria	Planctomycetes	Planctomyces maris DSM 8797	ZP_01855200.1	1 E-21	179/194
Bacteria	Proteobacteria	Vibrio coralliilyticus ATCC BAA-450	ZP_05885949.1	1 E-21	169/194
Bacteria	Actinobacteria	Stackebrandtia nassauensis DSM 44728	ZP_04486037.1	1 E-21	172/194
Bacteria	Proteobacteria	Marinomonas sp. MWYL1	YP_001342193.1	1 E-21	169/194
Bacteria	Proteobacteria	Vibrio splendidus 12B01	ZP_00989532.1	2 E-21	169/194
Bacteria	Actinobacteria	Kribbella flavida DSM 17836	ZP_03860270.1	2 E-21	170/194
Bacteria	Proteobacteria	Ralstonia metallidurans CH34	YP_587231.1	2 E-21	169/194
Bacteria	Proteobacteria	Vibrio splendidus LGP32	YP_002394707.1	3 E-21	169/194
Bacteria	Proteobacteria	Shewanella piezotolerans WP3	YP_002311738.1	3 E-21	182/194
Bacteria	Proteobacteria	Vibrio shilonii AK1	ZP_01865631.1	7 E-21	169/194
Bacteria	Proteobacteria	Proteus mirabilis ATCC 29906	ZP_03840074.1	8 E-21	169/194
Bacteria	Cyanobacteria	Synechocystis sp. PCC 6803	NP_942232.1	1 E-20	176/194
Bacteria	Proteobacteria	Shewanella woodyi ATCC 51908	YP_001762114.1	2 E-20	169/194
Bacteria	Proteobacteria	Acidovorax citrulli AAC00-1	YP_970497.1	2 E-20	174/194
Bacteria	Proteobacteria	Cupriavidus taiwanensis	YP_001796727.1	2 E-20	169/194
Bacteria	Proteobacteria	Yersinia kristensenii ATCC 33638	ZP_04622238.1	2 E-20	170/194
Bacteria	Proteobacteria	Plesiocystis pacifica SIR-1	ZP_01906785.1	2 E-20	180/194
Bacteria	Proteobacteria	Shewanella halifaxensis HAW-EB4	YP_001675612.1	4 E-20	157/194
Bacteria	Cyanobacteria	Cyanothece sp. PCC 8802	YP_003136435.1	5 E-20	168/194
Bacteria	Proteobacteria	Methylophaga thiooxidans DMS010	ZP_05103176.1	2 E-19	164/194
Bacteria	Cyanobacteria	Cyanothece sp. PCC 8801	YP_002370880.1	2 E-19	168/194
Bacteria	Proteobacteria	Pseudoalteromonas tunicata D2	ZP_01131783.1	3 E-19	182/194
Bacteria	Thermus	Deinococcus radiodurans R1	NP_294517.1	4 E-19	169/194
Bacteria	Proteobacteria	Ralstonia eutropha H16	YP_726311.1	4 E-19	169/194
Bacteria	Proteobacteria	Rhodobacterales bacterium HTCC2150	ZP_01740870.1	5 E-19	173/194
Bacteria	Proteobacteria	Legionella pneumophila str. Paris	YP_122236.1	5 E-19	173/194
Bacteria	Proteobacteria	Psychromonas sp. CNPT3	ZP_01217468.1	8 E-19	180/194
Bacteria	Proteobacteria	Providencia alcalifaciens DSM 30120	ZP_03318003.1	9 E-19	170/194
Bacteria	Proteobacteria	Legionella pneumophila subsp. pneumophila	YP_096004.1	9 E-19	174/194
Bacteria	Proteobacteria	Legionella pneumophila str. Paris	YP_124283.1	9 E-19	174/194
Bacteria	Proteobacteria	Providencia stuartii ATCC 25827	ZP_02960706.1	1 E-18	156/194
Bacteria	Proteobacteria	gamma proteobacterium HTCC2207	ZP_01223390.1	1 E-18	182/194
Bacteria	Proteobacteria	Reinekea blandensis MED297	ZP_01116435.1	1 E-18	178/194
Bacteria	Acidobacteria	Candidatus Koribacter versatilis Ellin345	YP_591556.1	1 E-18	170/194
Bacteria	Actinobacteria	Brevibacterium linens BL2	ZP_05912833.1	1 E-18	175/194
Bacteria	Proteobacteria	Shewanella baltica OS155	YP_001052205.1	2 E-18	178/194
Bacteria	Proteobacteria	Shewanella baltica OS195	YP_001552905.1	2 E-18	178/194
Bacteria	Proteobacteria	Labrenzia aggregata IAM 12614	ZP_01546418.1	2 E-18	169/194
Bacteria	Proteobacteria	Thioalkalivibrio sp. HL-EbGR7	YP_002515236.1	3 E-18	169/194
Bacteria	Proteobacteria	Shewanella baltica OS185	YP_001364680.1	3 E-18	178/194
Bacteria	Bacteroidetes	Flavobacteriaceae bacterium 3519-10	YP_003095713.1	3 E-18	171/194
Bacteria	Proteobacteria	Ralstonia eutropha JMP134	YP_296119.1	3 E-18	169/194
Bacteria	Proteobacteria	Legionella pneumophila str. Lens	YP_127299.1	4 E-18	174/194
Bacteria	Proteobacteria	Delftia acidovorans SPH-1	YP_001564131.1	4 E-18	170/194
Bacteria	Proteobacteria	Shewanella baltica OS223	YP_002356431.1	4 E-18	178/194
Bacteria	Proteobacteria	Pseudomonas fluorescens Pf-5	YP_260733.1	5 E-18	169/194
Bacteria	Proteobacteria	Agrobacterium radiobacter K84	YP_002542442.1	1 E-17	169/194
Bacteria	Proteobacteria	Shewanella putrefaciens CN-32	YP_001182062.1	1 E-17	178/194
Bacteria	Proteobacteria	Limnobacter sp. MED105	ZP_01913752.1	2 E-17	175/194
Bacteria	Actinobacteria	Geodermatophilus obscurus DSM 43160	ZP_03889941.1	2 E-17	179/194
Bacteria	Proteobacteria	Shewanella sp. W3-18-1	YP_965003.1	3 E-17	178/194
Bacteria	Proteobacteria	Rhodobacterales bacterium HTCC2654	ZP_01015753.1	3 E-17	170/194
Bacteria	Bacteroidetes	Candidatus Amoebophilus asiaticus 5a2	YP_001958308.1	4 E-17	170/194

Bacteria	Proteobacteria	Burkholderia cenocepacia J2315	YP_002230986.1	5 E-17	174/194
Bacteria	Proteobacteria	Shewanella putrefaciens 200	ZP_01704653.1	6 E-17	178/194
Bacteria	Bacteroidetes	Microscilla marina ATCC 23134	ZP_01688704.1	9 E-17	172/194
Bacteria	Proteobacteria	Burkholderia cenocepacia AU 1054	YP_626130.1	1 E-16	171/194
Archaea	Euryarchaeota	Methanococcoides burtonii DSM 6242	YP_566016.1	2 E-16	170/194
Bacteria	Proteobacteria	Mesorhizobium opportunistum WSM2075	ZP_05811414.1	2 E-16	172/194
Bacteria	Cyanobacteria	Cyanothece sp. PCC 7822	ZP_03155610.1	3 E-16	165/194
Bacteria	Bacteroidetes	Cytophaga hutchinsonii ATCC 33406	YP_677909.1	4 E-16	169/194
Archaea	Euryarchaeota	Methanosarcina acetivorans C2A	NP_619399.1	4 E-16	177/194
Bacteria	Cyanobacteria	Cyanothece sp. PCC 7425	YP_002483643.1	7 E-16	165/194
Bacteria	Proteobacteria	Labrenzia aggregata IAM 12614	ZP_01546081.1	2 E-15	163/194
Bacteria	Proteobacteria	Aeromonas hydrophila subsp. hydrophila	YP_855385.1	2 E-15	170/194
Bacteria	Bacteroidetes	Spirosoma linguale DSM 74	ZP_04490714.1	2 E-15	174/194
Bacteria	Cyanobacteria	Synechococcus elongatus PCC 6301	YP_172985.1	3 E-15	162/194
Bacteria	Proteobacteria	Moritella sp. PE36	ZP_01899748.1	8 E-15	168/194
Bacteria	Bacteroidetes	Dyadobacter fermentans DSM 18053	YP_003087352.1	1 E-14	171/194
Bacteria	Firmicutes	Bacillus sp. NRRL B-14911	ZP_01170048.1	2 E-14	166/194
Bacteria	Cyanobacteria	Anabaena variabilis ATCC 29413	YP_324814.1	3 E-14	167/194
Bacteria	Firmicutes	Clostridium acetobutylicum ATCC 824	NP_348123.1	9 E-14	168/194
Eukaryota	Viridiplantae	Micromonas sp. RCC299	XP_002503354.1	1 E-13	187/194
Bacteria	Spirochaetes	Leptospira biflexa serovar Patoc	YP_001965047.1	1 E-13	170/194
Bacteria	Cyanobacteria	Nostoc punctiforme PCC 73102	YP_001868631.1	1 E-13	169/194
Bacteria	Firmicutes	Lactococcus lactis subsp. lactis	NP_268117.1	2 E-13	166/194
Bacteria	Firmicutes	Oceanobacillus iheyensis HTE831	NP_691648.1	2 E-13	168/194
Bacteria	Proteobacteria	Haliangium ochraceum DSM 14365	ZP_03881780.1	4 E-13	180/194
Bacteria	Firmicutes	Pediococcus pentosaceus ATCC 25745	YP_805003.1	4 E-13	171/194
Bacteria	Bacteroidetes	Chryseobacterium gleum ATCC 35910	ZP_03849651.1	1 E-12	171/194
Bacteria	Proteobacteria	Anaeromyxobacter sp. Fw109-5	YP_001380916.1	1 E-12	173/194
Bacteria	Firmicutes	Bacillus subtilis subsp. subtilis	NP_391603.1	1 E-12	168/194
Bacteria	Proteobacteria	Hahella chejuensis KCTC 2396	YP_436514.1	1 E-12	165/194
Bacteria	Verrucomicrobia	Opitutus terrae PB90-1	YP_001818252.1	2 E-12	184/194
Bacteria	Firmicutes	Epulopiscium sp. 'N.t. morphotype	ZP_02691608.1	5 E-12	158/194
Bacteria	Actinobacteria	Micromonospora sp. ATCC 39149	ZP_04605114.1	8 E-12	184/194
Bacteria	Actinobacteria	Nocardia farcinica IFM 10152	YP_120395.1	3 E-11	191/194
Archaea	Methanosphaerula	Methanosphaerula palustris E1-9c	YP_002465959.1	5 E-11	176/194
Bacteria	Proteobacteria	Magnetospirillum magnetotacticum MS-1	ZP_00056282.1	6 E-11	165/194

AFUA_8G07380

Eukaryota	Fungi	Aspergillus fumigatus Af293	XP_747539.1	0.0	336/336
Eukaryota	Fungi	Neosartorya fischeri NRRL 181	XP_001262299.1	0.0	331/336
Eukaryota	Fungi	Aspergillus niger CBS 513.88	XP_001395159.1	1 E-108	333/336
Eukaryota	Fungi	Aspergillus terreus NIH2624	XP_001209187.1	1 E-106	337/336
Eukaryota	Fungi	Aspergillus oryzae RIB40	XP_001825471.1	1 E-102	299/336
Eukaryota	Fungi	Gibberella zeae PH-1	XP_390813.1	2 E-98	317/336
Eukaryota	Fungi	Aspergillus nidulans FGSC A4	XP_682391.1	8 E-95	327/336
Eukaryota	Fungi	Penicillium chrysogenum Wisconsin 54-1255	XP_002559891.1	7 E-89	314/336
Eukaryota	Fungi	Nectria haematococca mpVI 77-13-4	EEU42921.1	2 E-86	316/336
Eukaryota	Fungi	Nectria haematococca mpVI 77-13-4	EEU36515.1	1 E-79	306/336
Eukaryota	Fungi	Nectria haematococca mpVI 77-13-4	EEU35506.1	5 E-33	304/336
Eukaryota	Fungi	Nectria haematococca mpVI 77-13-4	EEU38492.1	2 E-31	298/336
Eukaryota	Fungi	Nectria haematococca mpVI 77-13-4	EEU33572.1	2 E-30	298/336
Eukaryota	Fungi	Aspergillus oryzae RIB40	XP_001727697.1	2 E-29	270/336
Eukaryota	Fungi	Neosartorya fischeri NRRL 181	XP_001261568.1	6 E-29	323/336
Eukaryota	Fungi	Uncinocarpus reesii 1704	XP_002542778.1	1 E-27	309/336
Eukaryota	Fungi	Phaeosphaeria nodorum SN15	XP_001797375.1	2 E-27	326/336
Eukaryota	Fungi	Botryotinia fuckeliana B05.10	XP_001550802.1	1 E-26	327/336
Eukaryota	Fungi	Microsporum canis CBS 113480	EEQ34647.1	4 E-26	309/336

Eukaryota	Fungi	Penicillium marneffei ATCC 18224	XP_002145720.1	1 E-25	310/336
Eukaryota	Fungi	Aspergillus clavatus NRRL 1	XP_001273603.1	2 E-25	336/336
Eukaryota	Fungi	Aspergillus nidulans FGSC A4	XP_681652.1	8 E-25	342/336
Eukaryota	Fungi	Aspergillus nidulans FGSC A4	XP_664052.1	8 E-25	348/336
Eukaryota	Fungi	Podospora anserina DSM 980	XP_001905653.1	3 E-23	322/336
Eukaryota	Fungi	Aspergillus terreus NIH2624	XP_001209382.1	2 E-22	313/336
Eukaryota	Fungi	Talaromyces stipitatus ATCC 10500	XP_002339967.1	2 E-22	313/336
Eukaryota	Fungi	Podospora anserina DSM 980	XP_001906796.1	3 E-22	327/336
Eukaryota	Fungi	Coccidioides immitis RS;	XP_001245248.1	8 E-22	329/336
Eukaryota	Fungi	Coccidioides immitis RS;	XP_001243721.1	3 E-21	341/336
Eukaryota	Fungi	Coccidioides posadasii C735 delta	EER26821.1	3 E-21	339/336
Eukaryota	Fungi	Coccidioides posadasii C735 delta	EER29448.1	5 E-21	329/336
Eukaryota	Fungi	Aspergillus terreus NIH2624	XP_001212807.1	2 E-20	337/336
Eukaryota	Fungi	Chaetomium globosum CBS 148.51	XP_001228055.1	6 E-20	336/336
Bacteria	Planctomycetes	Planctomyces maris DSM 8797	ZP_01856522.1	9 E-19	269/336
Eukaryota	Fungi	Aspergillus oryzae RIB40	XP_001823191.1	5 E-14	306/336
Eukaryota	Fungi	Aspergillus flavus NRRL3357	XP_002378531.1	2 E-13	306/336
Eukaryota	Fungi	Nectria haematococca mpVI 77-13-4	EEU34992.1	5 E-13	324/336
Eukaryota	Fungi	Aspergillus terreus NIH2624	XP_001214922.1	2 E-12	306/336

AFUA_8G07390

Eukaryota	Fungi	Aspergillus fumigatus Af293	XP_747540.1	8 E-21	50/50
