# Supplementary material for: Whole genome evaluation of horizontal transfers in the pathogenic fungus Aspergillus fumigatus
Source: BMC Genomics. 2010 Mar 12;11:171. doi: 10.1186/1471-2164-11-171 (PMC2848249; doi:10.1186/1471-2164-11-171)
Supplement: Additional file 4 — Phylogenetic trees. Phylogenetic trees for genes/proteins AFUA.1G11310, AFUA.2G07440 and AFUA.2G17620 and their respective SSU rRNA trees. A. fumigatus and N. fischieri were highlighted in blue, main incongruencies between SSU rRNA tree and protein tree are indicated with red arrows or bars. Numbers at nodes correspond to the number of bootstrap trees out of 1000 supporting that node when this number is inferior to 500. [file 1471-2164-11-171-S4.DOC]

**Additional file table S3**:

Annotated and putative function of the genes included in the atypical regions

| Gene AC # | Function |
| --- | --- |
|  |  |
| AFUA_1G01020 | Pfs, NACHT and Ankyrin domains protein |
| AFUA_1G01490 | NACHT domain protein |
| AFUA_1G01660 | alpha-L-rhamnosidase A, putative |
| AFUA_1G01670 | formaldehyde dehydrogenase, putative |
| AFUA_1G01680 | branched-chain amino acid aminotransferase |
| AFUA_1G01690 | cytochrome P450 alkane hydroxylase, putative |
| AFUA_1G04400 | 37S ribosomal protein Mrp10, mitochondrial |
| AFUA_1G05200 | eukaryotic translation initiation factor 3 subunit EifCa, putative |
| AFUA_1G05850 | erythromycin esterase, putative |
| AFUA_1G05960 | Peptidase M28 family |
| AFUA_1G06800 | Fe-containing alcohol dehydrogenase, putative |
| AFUA_1G06810 | aconitate hydratase, mitochondrial |
| AFUA_1G11280 | oxidoreductase, putative |
| AFUA_1G11300 | methyltransferase, putative |
| AFUA_1G11320 | class II DAHP synthetase family protein |
| AFUA_1G11330 | lactonohydrolase, putative |
| AFUA_1G11340 | glycosyl hydrolase family 88, putative |
| AFUA_1G11350 | MFS transporter of unkown specificity |
| AFUA_1G11360 | aldehyde reductase II |
| AFUA_1G11370 | versicolorin B synthase, putative |
| AFUA_1G14520 | pyridine nucleotide-disulphide oxidoreductase, putative |
| AFUA_1G14530 | general amidase, putative |
| AFUA_1G14540 | oxidoreductase, short-chain dehydrogenase/reductase family |
| AFUA_2G00720 | aldehyde dehydrogenase, putative |
| AFUA_2G00750 | oxidoreductase, 2OG-Fe(II) oxygenase family, putative |
| AFUA_2G04480 | xylosidase/arabinosidase, putative |
| AFUA_2G04490 | D-3-phosphoglycerate dehydrogenase |
| AFUA_2G04520 | Fe-containing alcohol dehydrogenase, putative |
| AFUA_2G04533 | Bys1 family protein |
| AFUA_2G05150 | cell wall galactomannoprotein Mp2/allergen F17-like |
| AFUA_2G06205 | yippee family protein |
| AFUA_2G06330 | ubiquitin C-terminal hydrolase, putative |
| AFUA_2G07440 | thioesterase family protein |
| AFUA_2G07710 | mRNA splicing factor RNA helicase (Cdc28), putative |
| AFUA_2G08670 | acetyl-CoA carboxylase |
| AFUA_2G09490 | eukaryotic translation initiation factor subunit eIF-4F, putative |
| AFUA_2G10360 | kynureninase |
| AFUA_2G10370 | iron-sulfur cluster assembly accessory protein Isa2, putative |
| AFUA_2G12710 | MFS monocarboxylate transporter, putative |
| AFUA_2G13295 | aminotransferase family protein (LolT), putative |
| AFUA_2G17610 | sulfatase domain protein |
| AFUA_2G17620 | cellobiose dehydrogenase |
| AFUA_2G18070 | metalloproteinase, putative |
| AFUA_3G01280 | alpha/beta hydrolase, putative |
| AFUA_3G01290 | AMID-like mitochondrial oxidoreductase, putative |
| AFUA_3G03540 | PKS-like enzyme, putative |
| AFUA_3G04300 | actin cytoskeleton organization and biogenesis protein, putative |
| AFUA_3G07850 | pheromone maturation dipeptidyl aminopeptidase DapB |
| AFUA_3G07860 | glycosyl transferase, putative |
| AFUA_3G08120 | PWI domain mRNA processing protein, putative |
| AFUA_3G09450 | alpha/beta fold family hydrolase, putative |
| AFUA_3G11840 | NAD binding Rossmann fold oxidoreductase, putative |
| AFUA_3G14680 | lysophospholipase Plb3 |
| AFUA_3G14690 | aminotransferase, putative |
| AFUA_3G15300 | amine transporter, putative |
| AFUA_3G15350 | short chain dehydrogenase family protein, putative |
| AFUA_3G15390 | MFS transporter, putative |
| AFUA_4G00610 | aryl-alcohol dehydrogenase, putative |
| AFUA_4G00620 | cell wall glycosyl hydrolase Dfg5, putative |
| AFUA_4G03510 | amino acid permease family protein, putative |
| AFUA_4G04820 | C-4 methyl sterol oxidase Erg25, putative |
| AFUA_4G06420 | fungal specific transcription factor, putative |
| AFUA_4G07710 | pyruvate carboxylase, putative |
| AFUA_4G09420 | carbonic anhydrase, putative |
| AFUA_4G09440 | sodium P-type ATPase, putative |
| AFUA_4G09560 | ZIP Zinc transporter, putative |
| AFUA_4G13770 | glycosyl hydrolase, putative |
| AFUA_4G13780 | polyphenol monooxygenase, putative |
| AFUA_4G13800 | extracellular sialidase/neuraminidase, putative |
| AFUA_4G14130 | ABC multidrug transporter, putative |
| AFUA_4G14560 | polyketide synthase, putative |
| AFUA_5G01680 | MFS transporter, putative |
| AFUA_5G01700 | C6 transcription factor, putative |
| AFUA_5G01710 | cytochrome P450 phenylacetate 2-hydroxylase, putative |
| AFUA_5G03960 | class V chitinase Chi100 |
| AFUA_5G04440 | nucleolar RNAse III, putative |
| AFUA_5G06800 | C6 transcription factor, putative |
| AFUA_5G06840 | class V chitinase, putative |
| AFUA_5G07510 | C6 transcription factor AlcR |
| AFUA_5G07570 | pyruvate carboxylase, putative |
| AFUA_5G07580 | methylmalonyl-CoA decarboxylase, alpha subunit, putative |
| AFUA_5G09980 | acyl-CoA dehydrogenase, putative |
| AFUA_5G09990 | C6 transcription factor, putative |
| AFUA_5G10120 | NRPS-like enzyme, putative |
| AFUA_5G13190 | kinesin family protein |
| AFUA_6G00440 | cation diffusion facilitator, putative |
| AFUA_6G00450 | feruloyl esterase, putative |
| AFUA_6G01860 | MFS lactose permease, putative |
| AFUA_6G01900 | flavin-binding monooxygenase-like protein |
| AFUA_6G03480 | nonribosomal peptide synthase, putative |
| AFUA_6G03630 | very-long-chain acyl-CoA synthetase family protein (CefD1), putative |
| AFUA_6G05350 | aspartic-type endopeptidase (OpsB), putative |
| AFUA_6G06350 | proteasome subunit alpha type 3, putative |
| AFUA_6G08640 | metallopeptidase Mip1 |
| AFUA_6G09590 | zinc alcohol dehydrogenase, putative |
| AFUA_6G09600 | zinc metallopeptidase, putative |
| AFUA_6G09610 | nonribosomal peptide synthase, putative |
| AFUA_6G09650 | membrane dipeptidase GliJ |
| AFUA_6G09660 | nonribosomal peptide synthase GliP |
| AFUA_6G09720 | methyltransferase GliN |
| AFUA_6G09730 | cytochrome P450 oxidoreductase GliF |
| AFUA_6G09740 | thioredoxin reductase GliT |
| AFUA_6G10100 | MFS monocarboxylate transporter, putative |
| AFUA_6G10120 | zinc-binding oxidoreductase ToxD, putative |
| AFUA_6G11580 | carboxy-cis,cis-muconate cyclase, putative |
| AFUA_6G11630 | FAD-dependent isoamyl alcohol oxidase, putative |
| AFUA_6G11910 | glycosyl hydrolase family 3, putative |
| AFUA_6G11920 | MFS alpha-glucoside transporter, putative |
| AFUA_6G13120 | filament-forming protein (Tpr/p270), putative |
| AFUA_6G13450 | nitrilase |
| AFUA_6G14550 | xylosidase/arabinosidase, putative |
| AFUA_7G00160 | polyketide synthase, putative |
| AFUA_7G01810 | C6 transcription factor, putative |
| AFUA_7G01820 | C6 transcription factor, putative |
| AFUA_7G02290 | ER glycosyl hydrolase (Edem), putative |
| AFUA_7G02310 | adenine phosphoribosyltransferase 1 |
| AFUA_7G05080 | C6 transcription factor, putative |
| AFUA_7G05090 | glucuronyl hydrolase, putative |
| AFUA_7G05100 | hexose transporter protein |
| AFUA_7G06140 | beta-D-glucoside glucohydrolase |
| AFUA_7G06900 | branched-chain amino acid aminotransferase, putative |
| AFUA_7G08340 | subtilisin-like alkaline protease, putative |
| AFUA_7G08350 | alpha-1,3-glucanase, putative |
| AFUA_8G02060 | glycan biosynthesis protein (PigL), putative |
| AFUA_8G02200 | proline permease, putative |
| AFUA_8G02260 | neutral amino acid permease |
| AFUA_8G02810 | ELMO/CED-12 family protein |
| AFUA_8G02820 | cell cycle checkpoint protein Rad17, putative |
| AFUA_8G02860 | AMP deaminase Amd1, putative |
| AFUA_8G06470 | N,N-dimethylglycine oxidase, putative |
| AFUA_8G06820 | dihydrofolate reductase family protein |
